# A Regional Modeling Framework of Phosphorus Sources and Transport in Streams of the Southeastern United States^1^

**DOI:** 10.1111/j.1752-1688.2010.00517.x

**Published:** 2011-10

**Authors:** Ana María García, Anne B Hoos, Silvia Terziotti

**Keywords:** nutrients, nonpoint source pollution, phosphorus, transport and fate, simulation, watersheds, SPARROW

## Abstract

**Abstract:**

We applied the SPARROW model to estimate phosphorus transport from catchments to stream reaches and subsequent delivery to major receiving water bodies in the Southeastern United States (U.S.). We show that six source variables and five land-to-water transport variables are significant (*p*<0.05) in explaining 67% of the variability in long-term log-transformed mean annual phosphorus yields. Three land-to-water variables are a subset of landscape characteristics that have been used as transport factors in phosphorus indices developed by state agencies and are identified through experimental research as influencing land-to-water phosphorus transport at field and plot scales. Two land-to-water variables – soil organic matter and soil pH – are associated with phosphorus sorption, a significant finding given that most state-developed phosphorus indices do not explicitly contain variables for sorption processes. Our findings for Southeastern U.S. streams emphasize the importance of accounting for phosphorus present in the soil profile to predict attainable instream water quality. Regional estimates of phosphorus associated with soil-parent rock were highly significant in explaining instream phosphorus yield variability. Model predictions associate 31% of phosphorus delivered to receiving water bodies to geology and the highest total phosphorus yields in the Southeast were catchments with already high background levels that have been impacted by human activity.

## Introduction

**FIGURE 1:**
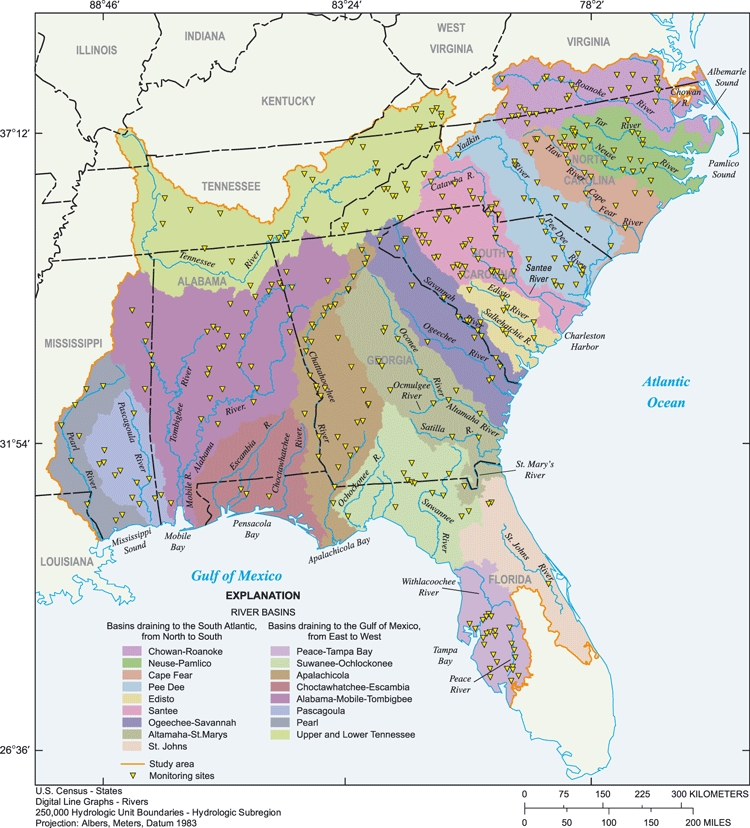
The Southeastern U.S. Study Area, Including Major River Basins Draining to the South Atlantic Coast, Eastern and Central Gulf Coast, and the Tennessee River.

The quality of coastal and freshwater resources in the Southeastern United States (U.S.) is threatened by eutrophication (Greening and Janicki, 2006; Lehrter, 2008; Pearl, 2009). Phosphorus (P) is recognized as the limiting nutrient in lakes and reservoirs and may have a greater role than previously thought in the eutrophication of estuaries, such as the Albemarle-Pamlico Sound (Figure 1), the second largest estuary in the U.S. (Carpenter, 2008; Schindler *et al.*, 2008; Pearl, 2009).

Reductions in phosphate concentrations in permitted discharges have decreased phosphorus concentrations and loads in some river basins in the Southeastern U.S. over the last three decades (Adler *et al.*, 1993; Harned *et al.*, 2009). Attention has now shifted to nonpoint sources of phosphorus pollution and, in particular, to increased phosphorus loading to water bodies from recently intensified animal production. Given the import of large quantities of animal feed and the low net export of grain, phosphorus accumulation in the Southeastern U.S. has become a significant concern (Cahoon *et al.*, 1999). Increased animal production upstream from many phosphorus-sensitive water bodies along with the voluntary nature of nutrient-management programs are the impetus for an improved assessment of the regional phosphorus budget to better target load-reduction strategies.

Nutrient-management policy in the U.S. has relied on risk indicators, such as the site-assessment index referred to as the P-index (Lemunyon and Gilbert, 1993). The P-index was developed to predict a potential for the transport of phosphorus to streams, or phosphorus loss, specifically from areas where phosphorus is applied to the land in association with agricultural activities. The index identifies site characteristics that control phosphorus availability and transport and ranks the vulnerability of agricultural fields according to potential for phosphorus loss. A federal water-quality initiative (U.S. Department of Agriculture and U.S. Environmental Protection Agency, 1999) prompted 47 states to develop individual P-indices to guide phosphorus-based nutrient management of confined animal operations. These P-indices have proven useful in site assessments, and most are based on field-scale (1 ha) and plot-scale (1 m^2^) process-based experimental studies (Heathwaite, 2003; Sharpley *et al.*, 2003). The indices vary considerably from state to state, limiting their use for regional applications, and although P-indices for individual states have been generalized elsewhere (Birr and Mulla, 2001; Heathwaite, 2003), no such effort has been made for the Southeastern U.S.

Another limitation in current nonpoint phosphorus-pollution management is the absence of regional-scale information on baseline loadings to account for the background sources of phosphorus. All P-indices include a source variable for labile soil phosphorus (referred to as soil test P), which is a site-specific measurement that accounts for contributions of phosphorus attributed to geology as well as accumulated phosphorus from historic and recent fertilizer applications (Hooda *et al.*, 2001). Natural sources of soil phosphorus are of particular concern in the Southeastern U.S., where local deposits of phosphate-rich parent rock are used to produce and export more than 85% of the phosphate fertilizer used in the U.S. (Jasinski *et al.*, 1999). Thus, management strategies for phosphorus would benefit from improved estimates of background sources of phosphorus at the watershed or regional scale.

In this paper we use the SPARROW (SPAtially Referenced Regression On Watershed attributes) framework to develop a regional-scale annual phosphorus transport model that is based in part on research on phosphorus loss from agriculture. More specifically, the model is based on P-indices developed from field research in the Southeastern U.S. We build on a previous SPARROW model that was developed to estimate nitrogen transport and delivery (Hoos and McMahon, 2009) and apply the new model to river basins draining to the South Atlantic coast, the Eastern and Central Gulf coast, and the Tennessee River (Figure 1). The study is one of seven SPARROW models developed for major river basins in the conterminous U.S. by the U.S. Geological Survey National Water Quality Assessment (NAWQA) Program and described in the Featured Collection. We incorporate source information, including background sources and information about transport processes, to link these together to predict incremental catchment yield delivery to receiving waters. We use the SPARROW model to (1) assess which source and transport parameters explain most of the variability in observed long-term mean annual phosphorus loads in the region, (2) identify variables that are represented in the nutrient-management P-indices that scale up to predict regional transport, and (3) predict phosphorus loads delivered annually to local streams and to targeted downstream receiving water bodies based on conditions in 2002.

## Methods

The SPARROW model performs a nonlinear least-squares multiple regression on elements of a hydrologic framework to solve a mathematical expression of constituent load. The expression for load at element *i*, *L*_*i*_, includes terms quantifying effects from sources, land-to-water delivery, and instream processes. Details on the theoretical development of the SPARROW model are provided by Alexander *et al.* (2008) and Schwarz *et al.* (2006). A simplified explanation follows.

Load at the outlet of catchment, *i*, is expressed by 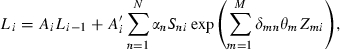
(1)where *L*_*i−*1_ is the load upstream to catchment, *i*, and *A*_*i*_ is the instream and reservoir processing term. The source term, *α*_*n*_*S*_*ni*_, is composed of an array of source variables, *S*_*ni*_, where *n* ranges from 1 to the total number of source variables, *N*, and a vector of coefficients, *α*_*n*_, which are estimated by nonlinear least-squares regression. The land-to-water delivery term, 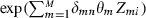, is similarly composed of an array of attributes, 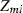, where *m* ranges from 1 to the total number of land-to-water variables, *M*, the delivery matrix, *δ*_*mn*_, and a vector of estimated coefficients, *θ*_*m*_.

The processing term, *A*_*i*_, is specified for either instream processing or reservoir processing, depending on the reach type. For instream processing, *A*_*i*_ is a first-order decay function, where the fraction of phosphorus mass transported through a stream reach is a continuous function of the mean reach water travel time, *T*_*i*_, mean water depth, *D*_*i*_, and an estimated coefficient, *β*. To obtain the incremental yield for a catchment, *i*, the processing term *A*_*i*_ is computed for half of the travel time. For reservoir processing, *A*_*i*_ is the first-order mass transfer rate dependent on the inverse of areal hydraulic loading, in units of year per meter and a model-estimated coefficient, *γ*. Further details on the functional forms of *A*_*i*_ are provided by Schwarz *et al.* (2006), and in the Supporting Information.

Although Equation (1) is an empirical relationship, it can be constrained by a process-based understanding of phosphorus transport. Estimated coefficients (*α*_*n*_, *θ*_*m*_, *β*, and *γ*) can be physically interpreted. For example, the source coefficients, *α*_*n*_, for land-use surrogates were compared with land-use export coefficients reported in the literature. Estimated source coefficients were standardized for the mean of the land-to-water delivery conditions to facilitate comparisons between sources and models. A mechanistic understanding of phosphorus transport can also be incorporated into the assemblage of source and delivery variables by defining the delivery matrix, *δ*_*mn*_, which is an array of 1 or 0 values that describe whether a land-to-water variable has an effect on transport to the stream from the source, *S*_*ni*_.

Configuration of the SPARROW model involved assembling input datasets describing watershed and reach characteristics, developing estimates of the dependent variable, mean annual instream load, 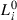, and numerically estimating 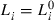 in logarithm space by nonlinear weighted least squares, for drainage areas with water-quality monitoring stations. The objective of the model estimation was to obtain a converged model composed of statistically significant (*p*<0.05) and physically interpretable coefficient values (*α*_*n*_, *θ*_*m*_, *β*, and *γ)*, the lowest unexplained error, and relatively independent predictor variables. Independence was assessed by calculating the variance inflation factor and the eigenspread. If a variable was not statistically significant, the variance inflation factor was evaluated to investigate whether multicollinearity was masking the significance of the coefficient. Multicollinearity was also evaluated by calculating the eigenspread of the predictor variables: a predictor variable that caused an eigenspread >100 was not retained (Schwarz *et al.*, 2006). Model accuracy was assessed by the root mean-square error (RMSE) of predicted load. The estimated coefficients were then applied to Equation (1) to predict stream loads for all elements in the spatial framework of the study area.

The spatial framework is a hydrologic network based on the 1:500,000-scale Enhanced River Reach File 2.0 (Hoos *et al.*, 2008; Brakebill *et al.*, 2010; this volume). Connected surface-water flow paths, referred to in this paper as reaches, have associated discrete or incremental drainage areas, which are referred to here as catchments. These spatial elements, reaches and catchments, were used to spatially reference input datasets in a manner similar to other spatially explicit watershed models, such as the Soil and Water Assessment Tool (Arnold *et al.*, 1998). The resulting data layers contained 8,321 catchments that ranged in area from 7 to 208 km^2^ (10 and 90 percentiles of the distribution, respectively) with a median of 62 km^2^. Catchments in southern Florida, where hydrologic boundaries do not correspond with delineated catchments from surface topography, were excluded from the analysis. The temporal framework was established by the dependent regression variable, long-term mean annual phosphorus loads computed at 370 water-quality monitoring sites (Figure 1). Sites had upstream drainage areas that ranged from 143 to 13,606 km^2^ (10 and 90 percentiles, respectively) with a median of 1,172.9 km^2^. Load estimation methods, including the adjusted likelihood method (Cohn *et al.*, 1992), were applied to water-quality and streamflow data collected between 1975 and 2004 by state and federal agencies. To make the estimated loads compatible with source data, loads were detrended and centered producing estimates of the mean phosphorus load that would have occurred in 2002 – the baseline year – if mean annual flow conditions from 1975 to 2004 had prevailed. Estimates ranged from 0.057 to 13,700 metric tons per year, with a mean of 195 metric tons per year. Further details on the development of these input data sets are provided by Hoos *et al.* (2008).

The use of long-term flow and water-quality records implies that model inputs and outputs are representative of long-term hydrologic variability. For example, instream loss rate coefficients estimated by SPARROW reflect the *mean annual* rates of contaminant removal in the stream reach. Although annual statistics do not predict seasonal loads or short-term intra-annual cycling, they are generally indicative of conditions during the high-flow periods of the year that often occur during the winter and spring.

To facilitate assembling a physically interpretable model, experimental field research summarized in the individual P-indices of the seven states that have major river basins in the study area was used to inform the selection of variables for evaluation in SPARROW. The P-indices define two categories – source and transport – of site characteristics or variables identified in plot- or field-scale research; the source and transport variables are combined to rank the risk of a site to phosphorus loss (Sharpley *et al.*, 1993; DeLaune *et al.*, 2004). A total of 17 independent source and transport variables are contained in the phosphorus indices of Alabama, Florida, Georgia, Mississippi, North Carolina, South Carolina, and Tennessee; the North Carolina phosphorus index contains the greatest number (12) of variables (Osmond *et al.*, 2006). The source and transport categories are analogous to the source and land-to-water components in the SPARROW model. Therefore, we assembled and tested variables in the SPARROW model that are equivalent to those used in the P-indices in the Southeastern states.

Previous SPARROW models (Hoos and McMahon, 2009) have prioritized direct measures (defined in terms of phosphorus mass) over indirect measures (defined in terms other than mass; e.g., area of a specific land cover or an index of potential contribution) of phosphorus mass supplied to the land surface and surface waters. We followed this approach and retained indirect measures of sources for which we did not have direct measures. A stepwise approach was followed; we first assembled a model with source terms only, retained significant source variables, and then included land-to-water variables as the second step. Collinearity among variables was evaluated by examining variance inflation factors and eigenspread; variable selection was changed if the eigenspread exceeded 100.

Seven source variables were tested for significance in explaining the observed instream phosphorus load variability (Table 1). All these sources were converted to mass units; in the case of fertilizer, for example, the original information was reported as a rate. Surrogate variables were scaled by catchment area so that the mass-balance estimation (Equation 1) could be performed. We tested phosphorus load in wastewater discharge as a direct measure of contribution from point sources (permitted facilities), and area in urban land as an indirect measure of the diffuse urban sources that contribute to phosphorus runoff.

**TABLE 1:** Phosphorus-Source Variables Tested for Use in Developing the SPARROW Model for the Southeastern U.S

Phosphorus-Source Variable	Mass Unit^1^	Spatial Dataset Tested in SPARROW	P-Index Source Factor That Is Equivalent to the Dataset Tested in SPARROW^2^
Point source	kg/year	P in NPDES-permitted discharge of municipal, domestic, and industrial wastewater (McMahon *et al.*, 2007)	P in wastewater discharge (included only in Florida P-index)
Urban land^3^	km^2^	Area in urban land as classified by the 2001 National Land Cover Dataset (Homer *et al.*, 2004)	NA
Manure	kg/year	P in animal waste from both confined and unconfined sources, as estimated by Ruddy *et al.* (2006)	Manure application rate
Commercial fertilizer	kg/year	Inorganic P fertilizer applied to cropland, estimated from county-level fertilizer sales (Ruddy *et al.*, 2006)	Inorganic P fertilizer application rate
Fertilized land	km^2^	Area classified as agricultural land in the National Land Cover Dataset (Homer *et al.*, 2004)	Inorganic P fertilizer application rate and soil-test P (the portion of soil-test P associated with P accumulation in agricultural soils)
Soil-parent rock	ppm km^2^	P content of bed sediment in headwater streams based on regionalizing National Geochemical Survey data (Terziotti *et al.*, 2009), multiplied by catchment area	The portion of soil-test P associated with soil-parent rock
Phosphate mines^3^	ppm km^2^	P content of bed sediment in headwater streams affected by mined land, inferred from National Geochemical Survey data (Terziotti *et al.*, 2009), multiplied by catchment area	NA

Notes: NA, not applicable; P, phosphorus; NPDES, National Pollutant Discharge Elimination System; kg/year, kilogram per year; km^2^, kilometer squared; ppm, parts per million.

1 Mass unit (as opposed to rate or concentration) used to perform the mass-balance estimation in the SPARROW model.

2 Unless noted otherwise, factor is included in P-indices for all states in the study area.

3 P load from this source is not accounted for in point-source data.

We used streambed-sediment phosphorus concentrations derived by Terziotti *et al.* (2009) to provide indirect measures of phosphorus in soil-parent rock. Bed-sediment samples collected at headwater streams in relatively undisturbed areas were aggregated by using geochemical map units and ecoregion classifications. The mapped value, concentration in parts per million (ppm), was then scaled by catchment area to serve as a surrogate in the SPARROW model for the mass of phosphorus contributed by geology.

Similarly, an indirect measure was used to represent phosphorus loads from phosphate-mine runoff and not accounted for in wastewater-discharge estimates. The variable for phosphate mines in Table 1 was calculated by using data on locations of active and inactive mines and streambed-sediment concentrations from geochemical surveys (Terziotti *et al.*, 2009). More specifically, phosphorus concentrations for mines were calculated by using bed-sediment concentration maps and subtracting the background bed-sediment concentration value from the value adjusted for the presence of active and inactive mines. This approach provided a basis for separating the effects of mining activities from the phosphate contribution from areas naturally high in phosphate.

The selection of variables to represent phosphorus contributions from agriculture was guided by the P-indices, which contain source terms for commercial fertilizer, manure from livestock operations, and labile soil phosphorus as measured by a soil test (soil-test P). We tested two variables to represent contributions from commercial fertilizer – estimates of annual application of phosphorus mass from commercial fertilizer and agricultural land. We reasoned that agricultural land could represent both contribution components from fertilized lands; that is, current application of commercial fertilizer and accumulated soil phosphorus from historic applications of commercial fertilizer. Soil-test P is used in the P-indices to account for not only the accumulated soil phosphorus from historic fertilization (legacy effects) but also phosphorus that occurs as a result of the weathering of geologic materials. Soil-test P data were not widely available for the study area and therefore could not be tested as a source variable.

Primary pathways for the transport of phosphorus to the stream channel are represented in the SPARROW model by using land-to-water delivery variables (Table 2). In general, phosphorus is transported to streams as particulate phosphorus (bound to soil particles) in surface runoff and as soluble phosphorus in subsurface flow. It is generally recognized that particulate phosphorus is the largest component of total phosphorus contribution from the landscape and is influenced by factors affecting erosion. P-indices for the Southeastern U.S. include a transport variable for erosion estimated by the Universal Soil Loss Equation (Williams and Berndt, 1977), which predicts erosion rates based on rainfall pattern, soil type, topography, crop system, and management practices. Our datasets did not include estimates of soil erosion for use in model estimation, and an argument could be made for including eroded material as a source variable. However, because we evaluated source variables that account for phosphorus in the soil, we tested erosion variables to represent land-to-water delivery. The three variables evaluated are components of the Universal Soil Loss Equation – precipitation, instead of regional rainfall erosivity, soil-erodibility factor (also known as K-factor), and slope.

**TABLE 2:** Land-to-Water Variables Tested for Use in Developing the SPARROW Model for the Southeastern U.S

Land-to-Water Variable	Unit of Measure	Spatial Dataset Tested in SPARROW	P-Index Source Factor That Is Equivalent to the Dataset Tested in SPARROW^1^
Soil erodibility factor (K)	Dimensionless	Susceptibility of soil to erosion obtained from the STATSGO database (U.S. Department of Agriculture, 1994)	Soil erosion
Slope	%	Land-surface slope from 100-m surface-elevation data (Falcone, 2003)	
Precipitation	mm	Annual mean precipitation, 1971-2000, PRISM (Oregon State University, 2007)	Implicit in the soil erosion and curve number factors
Soil hydrologic group	Dimensionless	Rating of hydrologic soil group	Soil hydrologic group and runoff class (included in P-index for five states)
Soil permeability	m^2^	Soil permeability, high value reported in STATSGO (U.S. Department of Agriculture, 1994)	Associated with the following p-index factors: runoff potential, runoff class, and SCS curve number
Depth to the water table	m	Water table depth, high value reported in STATSGO (U.S. Department of Agriculture, 1994)	Depth to the water table (included only in P-index for Georgia)
Artificial drainage	km^2^	Area of land with artificial drainage from the 1992 NRI	Subsurface drainage potential, and related drainage parameters
Riparian buffer	Ratio	Fraction of catchment area classified as riparian forest or wetland based on NLCD (Homer *et al.*, 2004) classifications	Buffer/filter strip (included in P-index for four states)
Soil pH	Dimensionless	Soil PH, high value, STATSGO (U.S. Department of Agriculture, 1994)	Fe-content (the only factor associated with sorption; included only in P-index for North Carolina)
Organic matter	%	Organic matter in soil, STATSGO (U.S. Department of Agriculture, 1994)	
Clay content	%	Clay in soil, STATSGO (U.S. Department of Agriculture, 1994)	

Notes: NRI, National Resources Inventory; PRISM, Parameter-elevation Regressions on Independent Slopes Model; STATSGO, State Soil Geographic Database; SCS, Soil Conservation Service; P, phosphorus.

1 Unless noted otherwise, factor is included in P-index for all states in the study area.

Characteristics that are related to soluble phosphorus loss were also tested, but large differences were noted among P-indices with respect to runoff and subsurface-flow variables. For example, variables related to distance to stream and hydrologic soil group are present in P-indices for five states, whereas riparian area is present in P-indices for four states and depth to the water table in one. An effort was made to compile as many equivalent land-to-water variables as practical for the SPARROW model estimation. These variables included soil hydrologic group, soil permeability, seasonably high depth to the water table, percentage of land artificially drained, soil pH, soil organic matter (content), and percentage of clay in the soil (Table 2). A measure of riparian buffer was calculated as the ratio of the catchment area classified as riparian forest or wetland from data in the 2001 National Land Cover Dataset (Homer *et al.*, 2004). The tested variable reflects natural conditions and is not equivalent to the buffer variable in the phosphorus index, which represents conservation-management strategies. Although the P-indices include other variables documented as modifying phosphorus delivery rates – time of application, method of application, grazing practices, and manure type – these were not accounted for in the variables assembled for testing in the SPARROW model. Our efforts were limited by data availability, especially in terms of management practices.

The primary pathway of delivery from a phosphorus source was simulated in the SPARROW model by specifying the delta function *δ*_*mn*_ (Equation 1) to be 1 or 0 to indicate interaction between land-to-water transport variables and the phosphorus sources. Point sources are discharged directly to the stream and, therefore, are not influenced by land-to-water variables (*δ*_*mn*_ = 0). On the other hand, phosphorus contributions from commercial fertilizer and agricultural land source variables are closely associated with both soil erosion and surface runoff (*δ*_*mn*_ = 1). Phosphorus associated with urban land was modeled to represent primarily soluble loads in urban runoff. To do this, *δ*_*mn*_ was set to 0 for the soil-erodibility factor, which is associated uniquely with erosion from nonurban areas. Current plot-scale experimental research on manure phosphorus losses indicates that manure phosphorus losses are primarily soluble (Kleinman *et al.*, 2007, 2009; García *et al.*, 2008). P-indices for several states include a manure-solubility factor to account for this. To test whether the SPARROW model could separate soluble from nonsoluble loads, the manure-source variable was tested with both *δ*_*mn*_ specifications.

Using the estimated model, it was possible to make predictions of phosphorus yield and delivered load to receiving water bodies. Predictions of catchment yield were computed by using estimated coefficients and evaluating the source and land-to-water delivery components, including the incremental contribution of instream or reservoir processing, 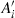. Catchment-level predictions of phosphorus yield were computed using the second term in Equation (1) such that 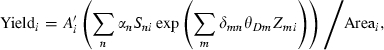
(2)where the range *i* is from 1 to 8,321, the total number incremental drainage areas, or catchments in the study area as defined by the stream network. Predictions of delivered load to major river basins were computed by evaluating both terms of Equation (1) at the most downstream locations.

## Results and Discussion

### Model Estimation

Model accuracy and predictive power were evaluated by diagnostic statistics from the estimation procedure, model goodness of fit, and physical interpretation of the estimated values of the coefficients. Confidence intervals for the estimated coefficients were obtained by re-sampling using bootstrap analysis.

The source and land-to-water variables retained in the final model estimate and their associated statistics are presented in Table 3. Of the seven source variables tested (Table 1), six were significant (*p*<0.05) in explaining variations in phosphorus loads across the Southeast (Table 3). The least significant of these variables, manure (*p*=0.003) and phosphate mines (*p*=0.003), presented asymmetrical confidence intervals from bootstrap re-sampling, with large lower bounds. Bootstrap estimates of model coefficients demonstrated robustness with estimates between 0 and 5% of the least-squares estimate. The bootstrap coefficient estimated for the phosphate mines variable deviated from the nonlinear least-squares estimate by 30%. These diagnostics suggest possible limitations in the source variable for phosphate mines and subsequent model predictions associated with this variable. There are several potential reasons for these limitations. The variable is distributed regionally but localized, given that all phosphate mines are in three states: Florida, Tennessee, and North Carolina. Although the input dataset accounted for areas of mining activity using local information, estimates of phosphate contribution may be inaccurate in areas of high mining activity, which are already naturally high in phosphate.

**TABLE 3:** Estimated SPARROW Model Coefficients *α*_*n*_ and *θ*_*n*_ for the Total Phosphorus Model Developed for the Southeastern U.S

			90% Confidence Interval for Model Coefficient				
						
Variable	Model Coefficient Units	Model Coefficient	Lower	Upper	Standard Error of Coefficient	*p*-Value^1^	Nonparametric Bootstrap Estimate of Coefficient (mean)^2^
Sources^3^							
Point sources (kg/year)	Dimensionless	0.67	0.47	0.88	0.12	<0.001	0.66
Urban land (km^2^)	kg/km^2^/year	88.0	59.4	116.6	17.4	<0.001	84.6
Manure (kg/year)	Dimensionless	0.013	0.005	0.020	0.005	0.0033	0.012
Agricultural land (km^2^)	kg/km^2^/year	48.4	25.7	71.1	13.81	<0.001	47.3
Soil-parent rock (ppm km^2^)	kg/ppm/km^2^	0.037	0.025	0.050	0.01	<0.001	0.038
Phosphate mines (ppm km^2^)	kg/ppm/km^2^	0.33	0.137	0.529	0.12	0.003	0.234
Land-to-water							
Soil erodibility factor(dimensionless)	Dimensionless	4.1	2.6	5.5	0.88	<0.001	4.0
Precipitation [log(mm)]	log (mm)^−1^	2.0	1.4	2.7	0.39	<0.001	2.05
Organic matter (%)	%	−0.17	−0.23	−0.11	0.035	<0.001	−0.18
Depth to the water table (m)	m	−0.35	−0.45	−0.26	0.058	<0.001	−0.35
Soil pH (dimensionless)	Dimensionless	0.46	0.10	0.82	0.22	0.038	0.48
Instream loss							
Product of travel time andinverse of mean waterdepth (day/m)	m/day	0.048	0.002	0.094	0.028	0.085	0.046
Reservoir loss							
Inverse of areal hydraulicloading (year/m)	m/year	29.8	15.9	43.7	8.4	<0.001	29.8
Model diagnostics							
MSE		0.29				*R*^2^ load^4^	0.91
RMSE^5^		0.54				*R*^2^ yield^6^	0.67
No. observations		370					

Notes: MSE, mean-squared error; RMSE, root mean-squared error.

1 Reported *p*-values are for a single-tailed *t*-test for source, instream loss, and reservoir-loss coefficients and a two-tailed test for land-to-water coefficients.

2 Estimate from performing a nonparametric bootstrap.

3 Estimated source coefficients were standardized for the mean of the land-to-water delivery conditions.

4 Coefficient of determination of log-transformed load estimate.

5 As calculated by Equation (3).

6 Coefficient of determination of log-transformed yield estimate.

The source-only model highlighted limitations in another of the tested source variables; the estimates of phosphorus in commercial fertilizer applied to agricultural land did not meet statistical significance criteria (*p* = 0.45). Assumptions made in the development of these estimates (Ruddy *et al.*, 2006) may be problematic in the Southeastern U.S.; specifically, nutrient management in agricultural areas underlain by phosphate-rich limestone may not follow generalized assumptions about phosphorus fertilizer application. Agricultural land was tested and found to be highly significant (*p* < 0.0001). It is possible that the land-based variable may act as a surrogate for the combined effect of current phosphorus application rates and accumulated soil phosphorus (storage) and, thus, be a better predictor for that reason.

Of the eleven land-to-water variables tested, five were found to be significant (Table 3). In general, we do not interpret lack of significance to necessarily mean a variable does not have an effect on phosphorus transport. Lack of statistical significance can also indicate limitations in the input data, including lack of spatial resolution. Variables that were not retained in the final model failed to meet either the significance or independence criteria (i.e., either *p*>0.05 or the variable inflated the eigenspread to above 100). These include fraction of catchment with artificial drainage (*p*=0.11), average catchment slope (*p*=*0.22*), tile drainage (*p* = 0.82), and the surrogate variable tested for riparian buffer (*p*=0.65). The variables soil hydrologic group and permeability variables were found to be marginally significant (*p*=0.05 and 0.045, respectively). They were not retained because of collinearity with other variables. In the final model, the variables retained led to an eigenspread of 33, which was deemed an acceptable level of interaction among the variables. The variable with the largest inflation factor (4.9 for agricultural land) is expected to interact most with the other variables. Information on the extent of agricultural land was used to develop other variables, such as the manure variable. Four transport variables that compose the final model are a subset of the transport variables in P-indices of states in the Southeast. The organic-matter content variable is not directly contained in any of the state indices.

Goodness of model fit was evaluated by computing the RMSE as 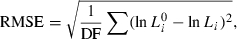
(3)where DF are the degrees of freedom of the log-transformed error. The coefficients of determination (*R*^2^) for both total load and yield (Table 3) were also calculated on log-transformed variables and model evaluation emphasized *R*^2^ of the logarithm of phosphorus yield, yield *R*^*2*^ in Table 3, which adjusts for the effects of drainage area. The value of 0.67 for *R*^2^ in the final model indicates that 67% of the variance of observed phosphorus yield is explained by the estimated model. The RMSE can be interpreted as a measure of the average error of the model-estimated load or yield compared with the observed load or yield, and the value of 0.54 is approximately equal to a mean percentage error of 54%. The RMSE can be used to compare accuracy among SPARROW models; for example, the national phosphorus SPARROW model that was applied to the Mississippi River basin is 0.60 (Alexander *et al.*, 2008).

**FIGURE 2:**
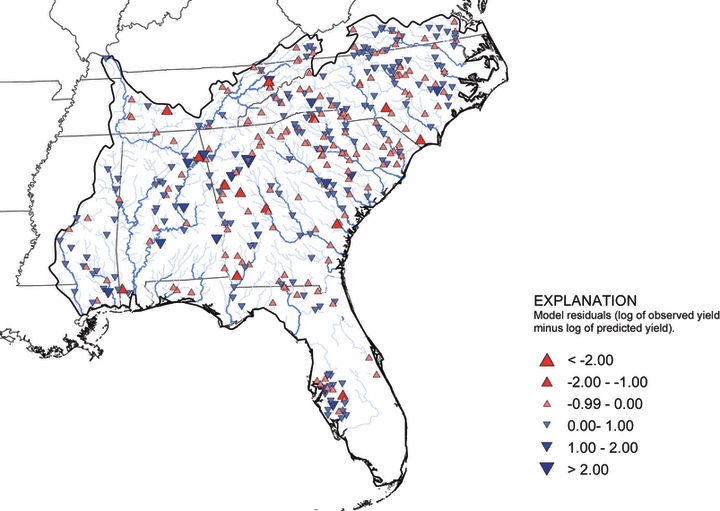
Map of Residuals for the SPARROW Model Phosphorus Predictions for the Southeast. Negative residuals indicate model overpredictions and positive residuals indicate model underpredictions.

Model estimation was performed in logarithm space, so log-transformed residuals are the appropriate residuals to evaluate and compare SPARROW models. The spatial distribution of residuals (log of observed yield minus log of predicted yield) (Figure 2) is a disperse pattern of over- and underpredictions with no pronounced tendency to over- or underpredict in specific areas. Spatial structure would indicate potential shortcomings in the model specification (missing source or transport terms) or in the input datasets. Most residuals lie between −0.65 and 0.59 (10 and 90 percentiles of the distribution, respectively) with a median of 0.034. Percentage errors are a measure of accuracy in real space and are provided as Supporting Information. Percentage errors range from −93 to 88% (10 and 90 percentiles of the distribution) with a median of 3%.

The estimated source coefficients (*α*_*n*_) associate the mass of phosphorus delivered to a stream with the unit measure of source input and modulate the input variable to reflect phosphorus availability. An *α* of 1 is expected for the point-source variable because discharge is directly to streams and, thus, there is no potential for landscape attenuation; the lower-than-expected model estimate of 0.67 may be compensating for potential overestimation of phosphorus mass inputs from permitted discharges or could be indicative of shared variance with the urban land source (Table 3).

The model estimates of *α* for urban and agricultural land associate the mass of phosphorus delivered to a stream with unit area of urban or agricultural land and are interpreted as land-use export coefficients. The estimate of 88.0 kg/km^2^ for urban land compared closely with values reported by Rast and Lee (1983) but was half of the national average reported by Reckhow *et al.* (1980). The export coefficient for agricultural land can be compared with studies of field-scale agricultural phosphorus loss. A study of annual loads from citrus and vegetable crop production systems in Florida reported export values between 42 and 2,169 kg/km^2^ (He *et al.*, 2006). Langdale *et al.* (1985) reported total P export values between 8 and 408 kg/km^2^ for small agricultural watersheds in the Southern Piedmont of Georgia. The value estimated by the SPARROW model (48.4 kg/km^2^/year, Table 3), which represents an average across all catchments in the study area, falls within reported ranges. The Southeast fertilized-land export factor would, in general, be expected to be less than measured export coefficients given that the model separates background phosphorus contributions from fertilizer contributions.

Because the input variable for manure was in kilograms of phosphorus, the estimated coefficient is a dimensionless ratio that estimates the true manure phosphorus availability as it pertains to phosphorus loss. Factors that may be accounted for in this coefficient include manure source type, application method, and loss through plant uptake, because these processes were not explicitly represented. Current research indicates that only water-extractable phosphorus, as opposed to total phosphorus, is related to phosphorus delivery from manure-amended areas and the ratio of water-extractable phosphorus to total phosphorus can vary from 0.002 to 0.1 depending on the manure type (Kleinman *et al.*, 2005). The SPARROW model estimate for the manure coefficient of 0.013 falls within this range (Table 3), which indicates some consistency of our results with findings in recent manure research.

The model estimate of 0.037 for *α* associated with the soil-parent rock variable (Table 3) is mass units of phosphorus (kg) delivered to the stream per ppm km^2^, which represents this background phosphorus source. Similarly, the model estimate of 0.33 kg/ppm/km^2^ (Table 3) for *α* for mined land associates the mass of phosphorus delivered to the stream. These estimates cannot be compared with literature values as these index variables have been uniquely defined for this model study.

Land-to-water variables that were associated with erosion, soluble phosphorus transport, and phosphorus sorption in the soil profile were found to be significant predictors of variability of instream phosphorus load. Significant variables associated with erosion processes were precipitation and the soil erodibility factor (*K*). The model-fitted values of the coefficients were positive, indicating that the variables are associated with areas of enhanced phosphorus delivery by erosion and runoff.

The finding that variables associated with the sorption capacities of the soil – percentage of organic matter, water-table depth, and soil pH – are significant predictors of variability of instream phosphorus load at the regional scale is unexpected and notable given the omission of these factors from the P-indices for most states in the Southeast. The percentage of organic matter in soil was found to be highly significant, and a negative coefficient was estimated by the model, indicating that an increase in organic matter was associated with a decrease in the observed loads.

**FIGURE 3:**
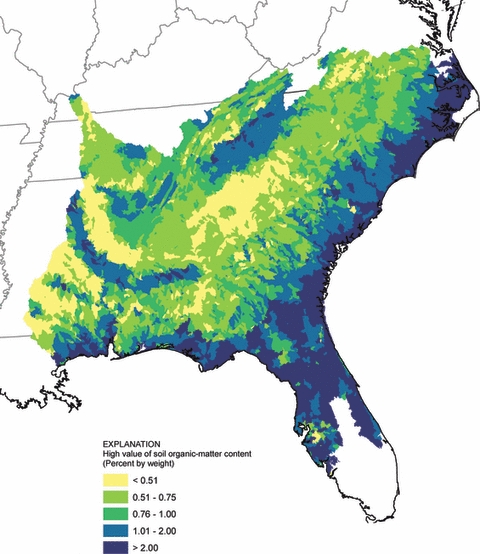
Spatial Distribution of Soil Organic Matter Content in Soils in the Southeastern U.S. [data from State Soil Geographic (STATSGO) database].

The relation between organic matter and observed phosphorus loads varies among regions of the U.S.; for example, P-indices for the Midwest express the opposite relation (increased organic content of the soil is associated with higher losses of sediment-bound organic phosphorus). The relation observed in the Southeastern U.S. (i.e., increased organic content is associated with lower losses to the stream) may be due to binding and immobilization of phosphorus occurring in saturated, hydric, aluminum-rich soils (Hogan *et al.*, 2004). The soils with the highest organic matter content in the Southeastern U.S. are associated with saturated hydric soils and some form part of vast palustrine wetlands and buffer a substantial portion of the South Atlantic and Gulf coasts (Figure 3). Many of these soils are shallow and aluminum-rich. Recent research has shown that aluminum-rich organic soils retain phosphorus by forming aluminum-bound forms of phosphorus, which are highly insoluble (Dell'Olio *et al.*, 2008). The sign of estimated coefficients for the water-table depth and soil pH variables are consistent with this conclusion. The soil pH variable also accounts for the process of soil sorption. Both acidic and alkaline soils produce conditions that can lead to insoluble forms, but acidic soils (pH < 5.5) form insoluble aluminum-bound forms of phosphorus. The positive value of the model-fitted coefficient for soil pH indicates that as pH increases, instream phosphorus load increases; as pH decreases, conditions become favorable for phosphorus sorption and instream phosphorus load decreases.

### Predictions of Phosphorus Yield for the Southeastern U.S

**FIGURE 4:**
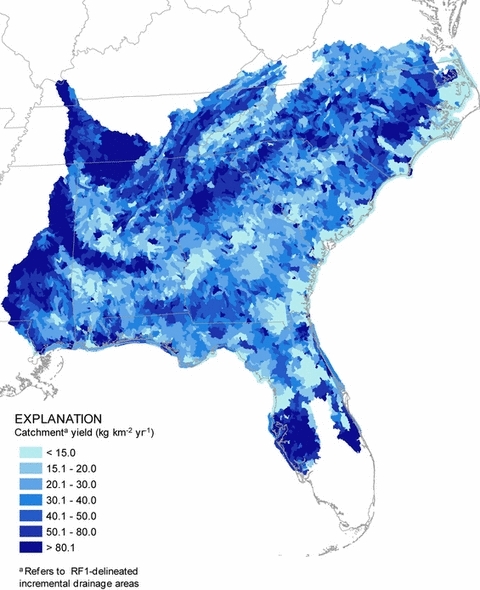
Predicted Annual Phosphorus Yield (Equation 2) for Catchments in the Southeastern U.S.

Catchment-level predictions of phosphorus yield for 8,321 catchments in the study area are presented in Figure 4 and provided as data files in Table S1 as part of the Supporting Information of this volume. Model predictions of annual catchment yield for the Southeast ranged from 15.2 to 106.6 kg/km^2^/year (10th and 90th percentiles of the distribution, respectively), with a median value of 34 kg/km^2^/year. Summary statistics on catchment yield and associated source shares are presented in Table 4.

**TABLE 4:** Summary Statistics for Yield and Source Shares for 8,321 Catchments in the SPARROW Model for the Southeastern U.S

			Percentiles				
			
	Mean	SD	10th	25th	50th	75th	90th
Catchment yield, in (kg/km^2^/year)	87.1	1,450.4	15.2	22.0	33.9	56.2	106.6
Contribution from individual sources (%)							
Point sources	4.4	15.5	0.0	0.0	0.0	0.0	7.2
Urban land	20.9	16.5	3.6	9.8	17.6	27.1	41.6
Manure	9.4	9.9	0.8	2.8	6.3	12.5	22.2
Agricultural land	23.9	15.8	2.2	10.6	23.3	35.6	45.8
Soil-parent rock	41.1	21.8	16.9	25.4	36.9	53.3	73.0
Phosphate mines	0.39	4.9	0.0	0.0	0.0	0.0	0.0

Notes: Catchment yield is the predicted load at each incremental drainage area, per unit area. SD, standard deviation.

**FIGURE 5:**
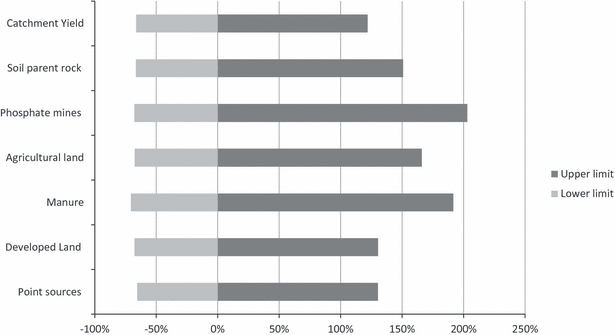
Average Percentage Difference (Equation 4) of Upper and Lower Confidence Interval Limits and Predicted Catchment Yield From All and Individual Sources.

Confidence intervals for predicted yield, which are also provided in Table S1, are a measure of accuracy in model predictions. The stochastic intervals have a 90% probability of including predicted yields and account for the sampling error of estimated coefficients and limitations in accounting for all factors that determine annual phosphorus loads. To compare these confidence intervals, the average percent difference (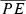) between predicted yield and the upper and lower bounds of the confidence interval (*L*_*i*,CI_) was calculated (Figure 5) such that, for the total number of catchments, *N*
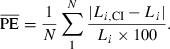
(4)

For catchment-level yields from all sources, the upper limit deviated from the mean by 122% and the lower limit by −66% on average. Predicted yields from specific sources presented a similar level of uncertainty. The phosphate mines variable had the largest confidence interval ranges, with the upper limit 203% greater and the lower limit 68% less than predicted yield on average.

**FIGURE 6:**
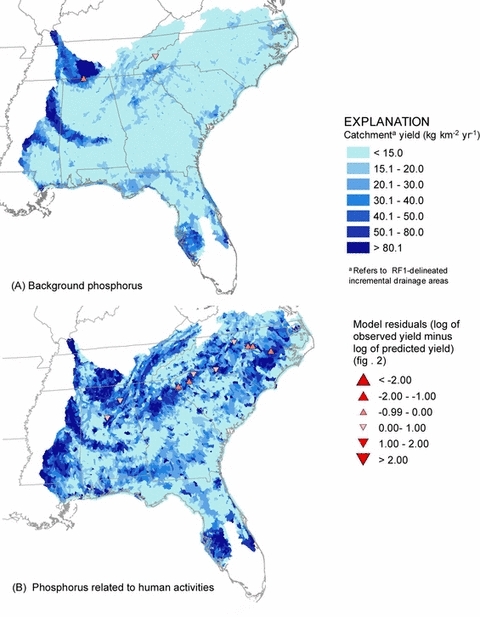
Predicted Catchment-Level Phosphorus Yields Associated With the (a) Background Phosphorus Source (soil-parent rock variable) and (b) Human-Related Phosphorus Source Variables (point sources, urban land, manure, agricultural land, and phosphate mines). Residuals are displayed for monitored catchments where background or human-related sources are dominant.

Separating background phosphorus yields from those associated with human activities can allow water resource managers to develop attainable standards in terms of water quality within different subregions of the Southeast. The soil-parent rock source variable is spatially correlated with natural geology and yields associated with this variable represent a background phosphorus level (Figure 6a). Catchments with the highest estimated yields from naturally occurring phosphorus (>15 kg/km^2^/year) are highly localized, present in Florida, middle Tennessee, eastern Mississippi, and central Alabama. These are areas (especially Florida and Tennessee) long known to be underlain by soils naturally rich in phosphate, but a systematic estimation of instream yields associated with geology has not been reported previously.

An assessment of the accuracy predictions of background phosphorus yields was limited by the observed dataset, which contained few undisturbed catchments in areas with high phosphate soils. Two such sites are identified in Figure 6a. For a site on the Elk River in Tennessee, the model overpredicted phosphorus yield and the model residual was −0.79 (equivalent to a percentage error of −121). For a monitored site on Chattahoochee Creek in western North Carolina, the model underpredicted phosphorus yield and the residual was 0.44 (equivalent to a percentage error of 36). Confidence intervals of background level predictions (Table S1, Figure 5) account for uncertainties in separating legacy effects from natural conditions in areas where human activities such as mining and historic farming have disturbed phosphate-rich soils.

Phosphorus yields associated with the point source, urban land, manure, agricultural land, and phosphate mines variables are indicative of human impact (Figure 6b). Several monitored sites were located in catchments that are highly impacted by human activities. The model residuals for these catchments ranged from −0.75 to 0.66 (equivalent to a percentage error range from −112 to 48). The spatial distribution of catchments highly impacted (>80 kg/km^2^/year) by human activities is widespread, yet there are similarities to the spatial distribution of background phosphorus. In fact, the highest yield predictions correspond to catchments with high background levels that have been disturbed by human activity such as those in the lower Tennessee River basin, the Tampa Bay drainage, and the Black Belt region in Mississippi and Alabama.

The North Carolina phosphorus index uses threshold values of edge-of-field phosphorus loss to classify agricultural fields into low-, medium-, and high-risk indicators (Johnson *et al.*, 2005). To compare SPARROW predictions with phosphorus indices a catchment-scale equivalent was computed. The SPARROW model predictions of the combined catchment phosphorus yield from three sources – agricultural land, manure, and soil-parent rock – were calculated as: 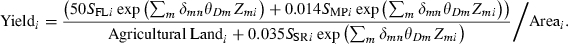
(5)

**FIGURE 7:**
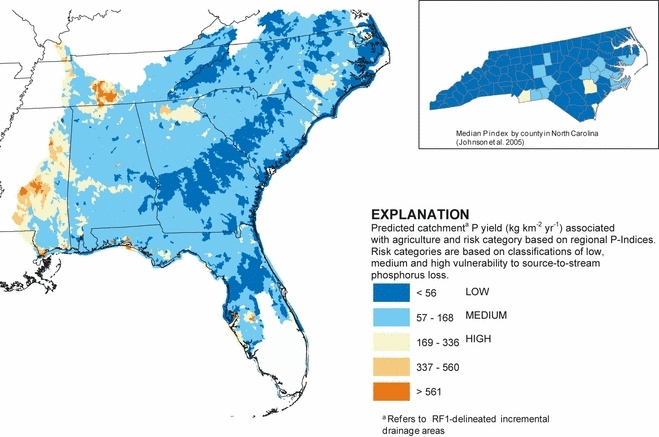
Predicted Phosphorus Yield From Fertilized Land, Phosphorus in Manure, and Soil-Parent Material, and Comparison for North Carolina in Relation to the P-Index, Estimated by Johnson *et al.* (2005).

Instream and reservoir processing were excluded. The predictions from Equation (5) are equivalent to phosphorus loss estimated for nutrient management. Relative ranking, by sorting high to low phosphorus-loss predictions, can be compared with P-index rankings of phosphorus-loss potential; we show this comparison for North Carolina (Figure 7). P-index rankings from other states in the Southeastern U.S. were not readily available. Although the county-level phosphorus indices show most of North Carolina in a low-risk category, the SPARROW model yield predictions lead to a medium-risk classification for most of the state, with the exception of mountain region and the coastal wetlands. There are several potential reasons for this discrepancy, including the manner in which conservation practices are credited with reducing phosphorus loss. The model implicitly accounts for these practices in the estimated coefficients, whereas they are explicitly represented in the phosphorus index methodology. It is possible that an explicit representation which includes assumed efficiencies of conservation practices may overestimate the impact on instream water quality. Both methodologies coincide in location of areas that are at high risk for phosphorus loss, which include areas with animal production facilities. At a regional scale, the model results show that catchments in the Pearl and Pascagoula basins are at the highest risk levels for agriculture-related phosphorus loss.

### Predictions of Phosphorus Delivery for the Southeastern U.S

SPARROW model predictions of phosphorus loads delivered to coastal water bodies, such as estuaries and bays, in the Southeastern U.S. are presented in Table 5. These estimates were made by computing the phosphorus loads delivered to the outlet of the basin. The estimates include the predicted mean net effects of sedimentation in reservoirs and biological processing during instream transport but do not include coastal processes such as water intrusion or dilution. It is important to note that coastal sites were not included in the observed data set.

**TABLE 5:** Phosphorus Load Delivered to Coastal Areas and Source Shares in Hydrologic Subregions (HUC4) of the Southeastern U.S

				Source Share for Delivered Load					
				
Major Hydrologic Subregion and Receiving Water Body	Basin Area (km^2^)	Delivered P Load (m tonne)	P yield (kg/km^2^/year)	P in Point Sources (%)	Urban Land (%)	P in Manure (%)	Agricultural Land (%)	Phosphate Mines (%)	Soil-Parent Rock (%)
*Basins draining to the South Atlantic, from North to South*									
Albemarle Sound tributaries: Chowan and Roanoke Rivers	46,824	1,053 (424-1,687)	22.5 (9-36)	18 (15-23)	7 (5-10)	8 (5-13)	39 (31-53)	*	28 (22-41)
Pamlico Sound tributaries: Pungo, Tar, Neuse, and Trent Rivers (also Bogue Sound)	32,594	1,035 (438-1,619)	31.8 (13-50)	14 (12-19)	16 (12-21)	17 (12-26)	30 (24-44)	2 (2-4)	20 (16-30)
Cape Fear Estuary, Long Bay, and New River Estuary	28,034	1,257 (484-1,842)	44.8 (17-66)	33 (28-41)	12 (9-17)	27 (20-39)	14 (10-22)	*	14 (11-21)
Winyah Bay tributaries: Pee Dee, Waccamaw, and Black Rivers	47,262	1,729 (839-2,623)	36.6 (18-55)	26 (22-32)	14 (11-19)	12 (8-19)	26 (20-38)	*	21 (17-32)
St. Helena Sound and Stono River Estuary tributaries: Edisto and Stono Rivers	16,263	316 (132-546)	19.4 (8-34)	5 (4-7)	16 (12-21)	5 (3-9)	33 (25-46)	*	41 (33-57)
Santee River Estuary and Charleston Harbor tributaries: Santee, Ashley, Cooper, Wando Rivers	43,948	705 (332-1,280)	16.1 (8-29)	44 (39-53)	19 (14-25)	3 (2-5)	12 (9-18)	*	22 (18-32)
Savannah River Estuary, Port Royal, Ossabaw, St. Catherines, and Sapelo Sound tributaries	43,873	1,051 (482-1,616)	23.9 (11-37)	30 (26-38)	16 (12-22)	6 (4-10)	17 (13-25)	*	30 (24-42)
Altamaha and St. Marys Rivers: St. Andrew, St. Simons, Cumberland and Nassau Sounds	52,877	1,238 (624-1,917)	23.4 (12-36)	21 (18-27)	21 (17-28)	8 (5-12)	20 (15-30)	*	30 (25-43)
St. Johns and Indian Rivers, and Daytona-St. Augustine	31,518	1,102 (503-1,815)	35.0 (16-58)	11 (9-15)	26 (19-34)	5 (3-8)	20 (15-31)	*	38 (31-53)
*Basins draining to the Gulf of Mexico, from East to West*									
Charlotte Harbor-Peace River, Sarasota, Tampa, and Crystal Bays, and Withlacoochee Estuary	27,041	3,521 (1,536-5,794)	130.2 (57-214)	13 (10-18)	10 (7-14)	3 (2-5)	8 (6-12)	49 (42-68)	17 (12-25)
Suwanee River, Waccasassa, and Apalachee Bays	46,909	2,006 (925-3,765)	42.8 (20-80)	43 (38-51)	12 (9-16)	4 (2-6)	13 (9-19)	1 (0-1)	28 (23-39)
Apalachicola Bay tributaries: Apalachicola, Chattahoochee, and Flint Rivers	52,414	928 (395-1,546)	17.7 (8-30)	13 (11-17)	21 (16-27)	5 (3-8)	27 (20-40)	*	34 (27-48)
Pensacola, Perdido, Choctawhatchee, and St. Andrew Bays	41,174	1,420 (654-2,177)	34.5 (16-53)	11 (9-14)	29 (23-36)	8 (5-13)	22 (17-34)	*	30 (25-43)
Mobile Bay tributaries: Coosa, Tallapoosa, Alabama, Tombigbee, and Mobile Rivers	115,532	4,461 (2,220-6,785)	38.6 (19-59)	14 (12-18)	14 (11-19)	8 (5-12)	29 (22-42)	*	35 (28-49)
East Mississippi Sound tributaries: Pascagoula River	25,618	1,205 (456-1,789)	47.0 (18-70)	14 (11-18)	16 (12-22)	20 (14-29)	22 (16-34)	*	28 (23-38)
West Mississippi Sound/Lake Borgne tributaries: Pearl River	27,716	1,894 (729-3,042)	68.3 (26-110)	11 (9-16)	13 (9-18)	17 (12-25)	26 (19-39)	*	33 (27-48)
Tennessee River	106,296	4,488 (2,069-7,314)	42.2 (19-69)	9 (7-13)	8 (6-11)	7 (5-11)	23 (16-34)	1 (1-2)	52 (44-67)
Entire study area	785,894	29,408 (14,781-43,109)	37.4 (19-55)	18 (15-23)	14 (11-19)	9 (6-14)	22 (16-32)	6 (5-10)	31 (25-44)

Notes: Values in parentheses represent 90% CI. Because of rounding, percentages may not add to 100. Confidence intervals for model predictions reflect both parameter variability and model error as estimated from percentiles of the bootstrap distributions. P, phosphorus; m ton, metric ton; kg/km^2^/year, kilogram per kilometer squared per year.

* Source share <0.01%.

Model predictions of delivered phosphorus vary from 16.1 kg/km^2^/year (for the Santee River estuary and Charleston Harbor) to 130.2 kg/km^2^/year (for Tampa Bay and Peace River), with a mean delivered yield of 37.4 kg/km^2^/year. Because predictions of delivered yield account for instream and reservoir attenuation, these are smaller than predictions of catchment yield, for which the mean is 87.1 kg/km^2^/year (Table 4). Thus, the model results can be interpreted to mean that instream and reservoir attenuation remove an average of 49.7 kg/km^2^/year of phosphorus mass during transport between the catchment and receiving water body. The sources contributing the largest amounts of phosphorus to receiving water bodies for the study area are background phosphorus in soil-parent rock (31%) and agricultural land (22%). The next largest contributors are wastewater discharge (18%) and urban land (14%). Manure (9%) and mined lands (6%) contribute less, although these two sources contribute substantially in specific regions.

Predicted source shares for receiving water bodies can be compared with other basin nutrient budgets to highlight consistencies with documented studies. Nutrient-source inputs have been documented in many studies; however, few studies have tracked the fate and delivery of nutrients from specific sources. Source share comparisons are presented in Table 6 for three river basins in the Southeastern U.S. For the Tar and Pamlico River basins, share allocations for agricultural-related activities as predicted by SPARROW are in agreement with a 1999 study (North Carolina Department of Environment and Natural Resources, 1999). Differences in phosphorus associated with point and urban sources can be attributed to changes between 1999 and 2002, which is the SPARROW benchmark year. For example, the phosphate ban in the 1990s may have further reduced point-source loads from the 1999 values. Also, rapid urbanization that occurred in the 1990s in North Carolina may have contributed to the higher 2002 urban phosphorus loads.

**TABLE 6:** SPARROW Model-Predicted Source Shares and Source Shares Reported in Other Studies for Selected River Basins (%)

	Tar and Pamlico River Basin		Peace River Basin		Upper St. Johns River Basin	
			
Source Variable	Literature^1^	SPARROW	Literature^2^	SPARROW	Literature^3^	SPARROW
Point sources	34	17	19	3	-	3
Nonpermitted urban sources	4	17	74^4^	2	11	13
Animal manure	15	21		5	78^5^	10
Cropland and fertilizer	22	27		14		34
Nonpermitted phosphate mines	-	-		62	-	-
Background source	21^6^	18	7	13	11	40

1 From the North Carolina Department of Environment and Natural Resources (1999).

2 From Siquires *et al.* (1998).

3 From the U.S. Environmental Protection Agency Region 4 (2004).

4 Reported as share of phosphorus load associated with all nonpoint sources.

5 Reported as share of contributions from inorganic fertilizer and animal manure.

6 Reported as share of phosphorus load associated with forest, shrubland, and barrenland.

A 1997 study of the Peace River in Florida (Siquires *et al.*, 1998) attributed 74% of total phosphorus delivered to Charlotte Harbor to nonpoint-source pollution, and most of this amount was delivered from catchments in the Upper Peace River where more than 50% of the land had been converted to agriculture or phosphate mining (Southwest Florida Water Management District, 2001). The nonpoint-source categories in the SPARROW model – background, agriculture and nonpermitted urban and mining sources – add up to 84% of the total load delivered and 75% is attributed to nonpoint-source pollution associated with mining activities and background sources. For the Upper St. Johns River basin, the SPARROW-predicted shares are a factor of 4 higher for the background source and a factor of 4 lower for the combined fertilizer and manure source than study results published by the U.S. Environmental Protection Agency Region 4 (2004). The discrepancies for the Upper St. Johns River may be due to many factors, including in part to inaccurate representation of the hydrologic network by the digital segmented network used in the SPARROW model. Predictions for drainage areas where the natural hydrology has been modified may be inaccurate (Hoos and McMahon, 2009). However, site-specific limitations in the input data in the Upper St. Johns do not greatly affect the overall model calibration; these represent only a few of the 370 used in model estimation.

## Conclusions

The SPARROW model was successfully applied to describe mean annual phosphorus transport in streams throughout the Southeastern U.S. and to evaluate the explanatory power of process-based knowledge contained in field-scale risk indicators (phosphorus indices) in estimating regional-scale transport. Six source variables and five land-to-water transport variables explained 67% of observed phosphorus yield variability measured at 370 sites. A regional phosphorus index for the Southeast U.S. was developed using the estimated SPARROW model.

The six source variables were a subset of seven tested. The estimation procedure revealed how limitations in the input data sets could restrict model accuracy. Fertilizer applied to agricultural land did not meet statistical significance criteria. The manner in which agriculture contributes to phosphorus levels in streams is relatively complex and is especially so in certain parts of the U.S. because of the phosphorus content of the soil profile, a function of natural geology and legacy effects, which affects the regional phosphorus budget. Based on model simulations, agricultural lands account for 24%, on average, of catchment-level phosphorus yield; we suggest that this includes the effects of both the current annual application of phosphorus in commercial fertilizer and accumulated phosphorus in the soil profile from legacy effects. Phosphorus associated with soil-parent rock source represents a background phosphorus level and account for 41%, on average, of catchment-level yields. Model estimation also highlighted limits in the source variable used to account for nonpoint source pollution from phosphate mines. While the variable was retained as a component of the Southeast SPARROW model, the spatial distribution of the variable coupled with uncertainties in the input data set led to larger confidence intervals in model predictions.

Five land-to-water variables that represent both particulate and soluble phosphorus losses to streams were found to be significant in model estimation. The transport variables that scaled-up from the nutrient-management phosphorus indices were soil erodibility, precipitation, and depth of the water table. Two land-to-water variables were uniquely associated with phosphorus sorption – organic matter content and soil pH – an important finding given that most state-developed phosphorus indices do not explicitly contain variables for sorption processes. Model-fitted coefficients for organic matter and water-table depth are negative, which indicates a mitigating role of areas with high organic matter or high water tables and suggests an important role of the coastal wetlands that line much of the Southeastern U.S. shoreline. The model simulations may be useful for investigating the buffering capacity of established coastal wetlands.

The model was used to simulate total phosphorus yield delivered from each catchment to local streams and to coastal water bodies: the estimated average yield to local streams in the Southeast region is 37.4 kg/km^2^/yr, and the estimated average yield delivered to coastal areas is 87.1 kg/km^2^/yr. Model predictions indicate that the highest total yields in the Southeast are catchments with high background levels that have been impacted by human activity. These areas are in Florida, middle Tennessee, the Black Belt region in Mississippi and Alabama. Although it has long been known that stream phosphorus loads in these areas, especially in Florida and Tennessee, are affected by soils naturally rich in phosphate, a region-wide and systematic estimation of instream yields associated with geology had not been previously reported. The model predictions of phosphorus yield from soil parent material represent background yield and are useful as benchmarks for comparison with water quality standards developed for different subregions of the Southeast.

Limitations of the Southeast SPARROW model are noted. The model is based on a single year of input data; more current and accurate results could be obtained with updated information. Conservation practices were not explicitly represented in this framework – their impact is implicit in estimated model coefficients – due to a lack of regional data sets. However, the use of the field-scale P-indices to guide the formulation of the empirical SPARROW model integrates the strengths of two different approaches for phosphorus risk assessment. The research basis afforded by the P-index facilitated the development of a physically interpretable and probably more accurate SPARROW model, and the regional scale afforded by the SPARROW estimates produces a tool that is useful to water-quality managers who need to quantify phosphorus loads, sources, and transport at the watershed and regional scales.

## SUPPORTING INFORMATION

**Figure S1.** Map of percentage error for the SPARROW model phosphorus predictions for the Southeast. Negative error indicates model overpredictions and positive residuals indicate model underpredictions.

**Table S1.** SPARROW model-predicted phosphorus yield from catchments in Southeastern U.S.

Please note: Neither AWRA nor Wiley-Blackwell is responsible for the content or functionality of any supporting materials supplied by the authors. Any queries (other than missing material) should be directed to the corresponding author for the article.
