# Supplementary material for: A Regional Modeling Framework of Phosphorus Sources and Transport in Streams of the Southeastern United States
Source: J Am Water Resour Assoc. 2011 Oct;47(5):991–1010. doi: 10.1111/j.1752-1688.2010.00517.x (PMC3307618; doi:10.1111/j.1752-1688.2010.00517.x)
Supplement: Supplementary file 1 [file jawr0047-0991-SD1.pdf]

SUPPORTING INFORMATION FOR THE ARTICLE:

A Regional Framework of Phosphorus Sources and Transport in Streams of the Southeastern United States

By Ana María García, Anne B. Hoos, and Silvia Terziotti

Percentage error for model predictions

Model estimation was performed in logarithm space, so log-transformed residuals are the appropriate measures to evaluate the estimation procedure in the SPARROW model. However, percentage error (PE) can provide measures of model accuracy in real-space. Percentage errors presented in Figure S1 were calculated by

$$PE\_i = \frac{L_i^O - L_i^P}{L_i^P} \qquad (S1)$$

where L_i^O is the observed annual mean load and L_i^P is the predicted annual mean load. Most percentage errors lie between -93 and 88 percent (10 and 90 percentiles of the distribution, respectively) with a median of 3 percent. The distribution has a pronounced skew, as expected when the dependent variable –observed mean annual load– takes only positive values.

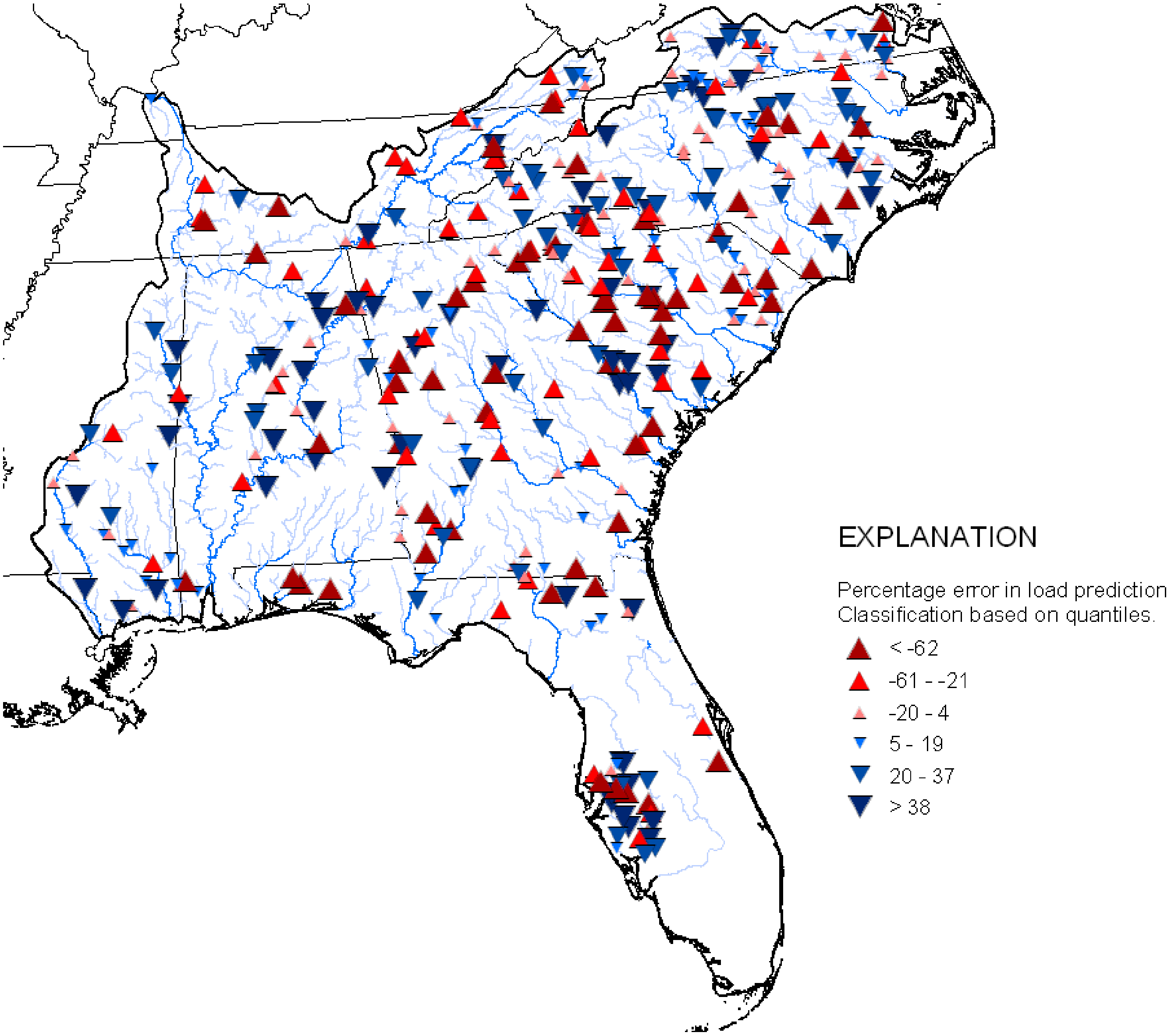

Figure S1. Map of percentage error for the SPARROW model phosphorus predictions for the Southeast. Negative error indicates model overpredictions and positive residuals indicate model under-predictions.

Calculations of Phosphorus Catchment-level Yield

Catchment-level predictions of phosphorus yield for 8,321 catchments in the study area are presented in figure 4 of the main body of this article and provided as data files in table S1 as part of the supporting information. Mean annual load, L_i was computed by substituting estimated coefficients (table 4) in equation 2 such that for catchment, *i*

$$L_i = 0.67S_{PS_i} + 88S_{UL_i}e^{2Z_{K_i}-0.17Z_{P_i}} + \left(0.013S_{M_i} + 48.4S_{AL_i} + 0.037S_{SPR_i} + 0.33S_{PM_i}\right)e^{4.1Z_{K_i}+2Z_{P_i}-0.17Z_{OM_i}-0.35Z_{WT_i}+0.46Z_{PH_i}} \tag{S2}$$

where S_{PS_i} denotes point sources, S_{UL_i}, urban land, S_{M_i}, manure, S_{AL_i}, agricultural land, S_{SPR_i}, soil parent rock and S_{PM_i}, phosphate mines. The land to water variables are represented by Z_{K_i}, which denotes the soil erodibility factor, Z_{P_i}, precipitation, Z_{OM_i}, organic matter, Z_{WT_i}, depth to the water table, Z_{PH_i}, soil pH. Yields were obtained by dividing L_i by catchment area and including the incremental contribution of instream or reservoir processing. For instream nutrient loss the functional form is given by

$$A_i = exp\left(\beta \frac{T_i}{D_i}\right) \tag{S3}$$

where A_i is the fraction of phosphorus mass transported through a stream reach mean T_i , reach water travel time, D_i, mean water depth, and β, an estimated coefficient. For catchment-level yield A_i is evaluated for half the mean travel time for the incremental catchment. For reservoir processing, A_i is first-order mass transfer rate dependent on the inverse of areal hydraulic loading, H_i in units of year meter^{-1}and a model-estimated coefficient, γ.

$$A_i = \frac{1}{1 + \gamma H_i^{-1}} \tag{S4}$$

Applying the A_i term to equation S2 for catchments within a target basin provides an estimate of mass delivered downstream. In table S1,we report the fraction (of 1.0) of phosphorus mass that is transported through the stream network and ultimately delivered to coastal locations or, for reaches in the Tennessee River Basin, to the confluence with the Ohio River.

Table S1. Model-predicted phosphorus yield and confidence intervals from catchments in Southeastern U. S.

[Confidence intervals for model predictions reflect both parameter variability and model error as estimated from percentiles of the bootstrap distributions. Model predictions are less reliable for reaches representing small watersheds partly because the ancillary data used to estimate source inputs is county-level information therefore, predictions for very small catchments (< 1 km2) have been removed.

HUC, Hydrologic Cataloguing Unit; ID, catchment identification; kg, kilogram, km^2, square kilometer, CI, confidence interval; Frac, fraction of mass that is delivered to the basin outlet (the coast or, for reaches in the Tennessee River Basin, the confluence with the Ohio River)]

8-digit HUC	ID	Name	Area	Catchment Yield		Point sources		Developed Land		Manure		Agricultural Land		Phosphate Mines		Soil parent rock		Frac
				kg/km^2	90% CI	kg/km^2	90% CI	kg/km^2	90% CI	kg/km^2	90% CI	kg/km^2	90% CI	kg/km^2	90% CI	kg/km^2	90% CI	
3010101	4557	BIG OTTER R	71.1	36.4	14 - 97	0.0	0.0 - 0.0	5.7	1.8 - 14.2	3.5	1.0 - 11.0	12.4	4.5 - 37.2	0.0	0.0 - 0.0	14.8	5.5 - 40.2	0.33
3010101	4558	FLAT CR	105.3	31.8	11 - 82	0.0	0.0 - 0.0	7.1	2.4 - 16.4	2.6	0.8 - 7.9	9.1	3.0 - 27.0	0.0	0.0 - 0.0	13.0	4.6 - 36.3	0.32
3010101	4559	BIG OTTER R	222.1	35.0	12 - 77	0.0	0.0 - 0.0	8.2	2.5 - 17.8	3.9	1.1 - 11.5	12.9	3.9 - 34.1	0.0	0.0 - 0.0	10.0	3.1 - 24.1	0.32
3010101	4560	BIG OTTER R	16.7	37.5	11 - 79	0.0	0.0 - 0.0	10.8	3.4 - 24.8	4.3	1.4 - 11.1	13.7	4.1 - 32.4	0.0	0.0 - 0.0	8.7	2.7 - 22.8	0.31
3010101	4561	ELK CR	111.4	36.6	14 - 75	0.0	0.0 - 0.0	9.1	2.9 - 21.1	3.7	1.2 - 10.9	11.7	4.3 - 29.7	0.0	0.0 - 0.0	12.1	4.3 - 28.4	0.30
3010101	4562	BIG OTTER R	45.4	42.2	14 - 71	0.0	0.0 - 0.0	9.9	3.4 - 17.7	4.1	1.2 - 12.4	13.2	4.4 - 26.7	0.0	0.0 - 0.0	15.0	4.8 - 29.2	0.30
3010101	4563	*A	130.2	21.7	9 - 35	0.0	0.0 - 0.0	3.2	1.1 - 5.5	1.5	0.5 - 3.3	4.9	1.7 - 12.5	0.0	0.0 - 0.0	12.2	5.2 - 23.5	0.28
3010101	4564	BIG OTTER R	141.9	24.1	8 - 46	0.0	0.0 - 0.0	5.1	1.6 - 10.9	1.7	0.5 - 4.4	5.5	1.7 - 12.3	0.0	0.0 - 0.0	11.9	4.0 - 27.0	0.28
3010101	4565	LITTLE OTTER R	181.0	48.0	16 - 158	8.3	2.9 - 25.8	11.2	3.5 - 41.0	4.1	1.3 - 16.9	13.6	4.3 - 45.5	0.0	0.0 - 0.0	10.8	3.6 - 33.7	0.31
3010101	4566	ROANOKE R	174.6	51.0	18 - 129	14.8	4.9 - 40.6	11.1	3.9 - 31.8	2.6	0.7 - 10.3	11.1	3.8 - 31.8	0.0	0.0 - 0.0	11.4	3.9 - 31.1	0.33
3010101	4567	GOOSE CR	550.3	26.0	9 - 67	0.0	0.0 - 0.0	4.4	1.4 - 9.9	2.8	0.9 - 8.0	9.0	2.9 - 32.4	0.0	0.0 - 0.0	9.8	3.4 - 26.3	0.33
3010101	4568	GOOSE CR, N FK	82.9	20.5	7 - 43	0.0	0.0 - 0.0	5.2	1.9 - 11.1	1.7	0.5 - 5.2	5.4	1.7 - 15.7	0.0	0.0 - 0.0	8.2	2.8 - 22.3	0.27
3010101	4569	GOOSE CR, S FK	36.5	20.0	7 - 45	0.0	0.0 - 0.0	8.7	2.8 - 22.6	1.0	0.4 - 2.9	3.1	1.0 - 6.8	0.0	0.0 - 0.0	7.3	2.4 - 17.3	0.27
3010101	4570	ROANOKE R	9.4	24.0	8 - 50	0.0	0.0 - 0.0	2.2	0.7 - 4.8	2.4	0.7 - 6.5	8.8	3.1 - 19.1	0.0	0.0 - 0.0	10.6	3.2 - 23.4	0.33
3010101	4571	ROANOKE R	23.5	46.5	16 - 90	0.0	0.0 - 0.0	30.9	10.5 - 64.6	0.9	0.3 - 2.6	3.3	1.3 - 7.7	0.0	0.0 - 0.0	11.4	3.4 - 25.8	0.05
3010101	4572	*B	87.1	39.3	11 - 71	0.0	0.0 - 0.0	24.1	6.7 - 41.3	1.6	0.4 - 4.2	5.5	1.6 - 14.5	0.0	0.0 - 0.0	8.1	2.4 - 18.4	0.05
3010101	4574	*C	201.1	39.0	15 - 84	0.0	0.0 - 0.0	26.3	9.4 - 57.8	1.4	0.5 - 3.7	4.7	1.7 - 13.4	0.0	0.0 - 0.0	6.6	2.3 - 14.5	0.05
3010101	4575	ROANOKE R	111.2	63.3	22 - 138	0.0	0.0 - 0.0	53.4	18.0 - 121.5	0.2	0.1 - 0.5	1.0	0.4 - 2.9	0.0	0.0 - 0.0	8.7	3.1 - 18.4	0.05
3010101	4576	MASON CR	76.7	17.9	6 - 37	0.0	0.0 - 0.0	12.9	4.5 - 27.7	0.2	0.1 - 0.5	0.7	0.2 - 2.0	0.0	0.0 - 0.0	4.2	1.5 - 10.3	0.05
3010101	4577	ROANOKE R	212.2	21.6	6 - 44	0.0	0.0 - 0.0	14.4	4.2 - 29.8	0.4	0.1 - 1.2	1.6	0.5 - 3.4	0.0	0.0 - 0.0	5.2	1.5 - 11.0	0.05
3010101	4578	ROANOKE R, N FK	35.6	24.5	8 - 65	0.0	0.0 - 0.0	9.1	2.9 - 23.2	1.5	0.4 - 5.2	4.0	1.3 - 12.1	0.0	0.0 - 0.0	9.9	3.1 - 26.1	0.04
3010101	4579	BRADSHAW CR	104.8	10.3	4 - 21	0.0	0.0 - 0.0	3.5	1.1 - 7.5	0.6	0.2 - 1.7	1.8	0.6 - 4.2	0.0	0.0 - 0.0	4.4	1.6 - 10.7	0.04
3010101	4580	ROANOKE R, N FK	168.5	25.8	9 - 51	0.0	0.0 - 0.0	7.2	2.5 - 14.6	2.7	0.8 - 7.5	8.1	2.7 - 19.1	0.0	0.0 - 0.0	7.9	3.0 - 17.1	0.04
3010101	4581	ROANOKE R, S FK	35.6	13.5	5 - 33	0.0	0.0 - 0.0	4.2	1.5 - 9.9	0.7	0.2 - 2.0	2.0	0.7 - 5.9	0.0	0.0 - 0.0	6.6	2.5 - 16.4	0.04
3010101	4582	ELLIOT CR	92.6	17.8	6 - 43	0.0	0.0 - 0.0	4.1	1.5 - 10.2	1.7	0.5 - 4.7	4.8	1.8 - 12.5	0.0	0.0 - 0.0	7.1	2.2 - 18.1	0.04
3010101	4583	ROANOKE R, S FK	16.9	12.5	4 - 27	0.0	0.0 - 0.0	3.3	1.0 - 7.8	0.7	0.2 - 2.0	2.0	0.6 - 4.9	0.0	0.0 - 0.0	6.5	1.9 - 15.8	0.04
3010101	4584	PURGATORY CR	14.8	11.4	4 - 29	0.0	0.0 - 0.0	1.2	0.4 - 3.0	0.7	0.2 - 2.1	2.0	0.6 - 5.5	0.0	0.0 - 0.0	7.5	2.5 - 20.2	0.04
3010101	4585	ROANOKE R, S FK	11.0	14.0	5 - 26	0.0	0.0 - 0.0	3.3	1.0 - 6.2	0.6	0.2 - 1.3	1.6	0.5 - 4.0	0.0	0.0 - 0.0	8.5	2.7 - 18.1	0.04
3010101	4586	ROANOKE R, LICK	61.4	13.3	5 - 29	0.0	0.0 - 0.0	4.6	1.5 - 12.0	0.5	0.2 - 1.7	1.4	0.4 - 4.8	0.0	0.0 - 0.0	6.8	2.4 - 17.0	0.04
3010101	4587	ROANOKE R, S FK	72.8	14.9	5 - 32	0.0	0.0 - 0.0	4.9	1.5 - 10.1	0.5	0.1 - 1.4	2.0	0.6 - 5.5	0.0	0.0 - 0.0	7.4	2.3 - 18.3	0.04
3010101	4588	BACK CR	149.4	22.0	7 - 38	0.0	0.0 - 0.0	12.5	4.1 - 27.3	0.4	0.1 - 1.1	1.5	0.5 - 3.1	0.0	0.0 - 0.0	7.6	2.5 - 15.1	0.05
3010101	4589	MAGGOTTY CR	117.0	21.4	9 - 37	0.0	0.0 - 0.0	3.6	1.2 - 7.6	2.4	0.8 - 6.4	7.6	3.0 - 17.1	0.0	0.0 - 0.0	7.8	2.8 - 15.3	0.05

8-digit HUC	ID	Name	Area	Catchment Yield		Point sources		Developed Land		Manure		Agricultural Land		Phosphate Mines		Soil parent rock		Frac
				kg/km^2	90% CI	kg/km^2	90% CI	kg/km^2	90% CI	kg/km^2	90% CI	kg/km^2	90% CI	kg/km^2	90% CI	kg/km^2	90% CI	
3010101	4590	BLACKWATER R	149.7	28.8	10 - 69	0.0	0.0 - 0.0	4.5	1.5 - 10.8	4.1	1.3 - 12.6	13.0	4.7 - 35.5	0.0	0.0 - 0.0	7.2	2.4 - 19.7	0.05
3010101	4591	BLACKWATER R, I	82.5	12.4	4 - 24	0.0	0.0 - 0.0	2.1	0.7 - 3.9	1.1	0.3 - 3.3	3.5	1.3 - 7.9	0.0	0.0 - 0.0	5.6	1.9 - 11.8	0.04
3010101	4592	BLACKWATER R, S	72.6	12.0	4 - 26	0.0	0.0 - 0.0	2.2	0.7 - 5.1	1.1	0.3 - 3.3	3.5	1.2 - 8.6	0.0	0.0 - 0.0	5.1	1.6 - 12.9	0.04
3010101	4593	PIGG R	31.2	24.4	7 - 59	0.0	0.0 - 0.0	3.5	1.0 - 8.2	2.1	0.5 - 6.3	8.8	2.6 - 27.1	0.0	0.0 - 0.0	10.0	3.0 - 27.1	0.22
3010101	4594	PIGG R	95.3	28.3	11 - 61	0.0	0.0 - 0.0	2.4	0.8 - 5.4	3.5	1.1 - 9.5	11.1	3.9 - 28.7	0.0	0.0 - 0.0	11.3	4.1 - 27.2	0.22
3010101	4595	PIGG R	128.6	40.3	14 - 93	6.2	2.1 - 15.8	8.6	2.8 - 21.7	4.2	1.2 - 14.0	13.6	4.6 - 36.8	0.0	0.0 - 0.0	7.7	2.5 - 17.9	0.19
3010101	4596	HATCHET CR	107.4	11.5	3 - 22	0.0	0.0 - 0.0	2.5	0.6 - 4.8	1.3	0.3 - 3.1	4.0	1.1 - 9.3	0.0	0.0 - 0.0	3.7	1.0 - 7.4	0.14
3010101	4597	PIGG R	45.6	18.8	6 - 40	0.0	0.0 - 0.0	4.3	1.2 - 9.5	2.0	0.5 - 5.6	6.2	2.0 - 15.2	0.0	0.0 - 0.0	6.4	2.2 - 15.1	0.14
3010101	4598	BIG CHESTNUT CR	34.4	19.6	6 - 42	0.0	0.0 - 0.0	2.0	0.6 - 4.5	2.1	0.6 - 5.6	6.8	2.1 - 16.9	0.0	0.0 - 0.0	8.7	2.6 - 21.7	0.19
3010101	4599	LITTLE CHESTNUT	45.7	24.9	8 - 47	0.0	0.0 - 0.0	4.5	1.3 - 9.2	3.2	1.0 - 7.8	10.0	3.3 - 22.6	0.0	0.0 - 0.0	7.2	2.4 - 15.3	0.16
3010101	4600	BIG CHESTNUT CR	79.0	20.2	7 - 43	0.0	0.0 - 0.0	3.4	1.3 - 8.0	2.2	0.6 - 5.5	6.9	2.1 - 16.9	0.0	0.0 - 0.0	7.7	2.5 - 20.3	0.16
3010101	4601	SNOW CR	4.2	27.0	8 - 53	0.0	0.0 - 0.0	3.3	1.0 - 6.4	2.9	0.8 - 9.3	12.9	4.0 - 27.6	0.0	0.0 - 0.0	7.8	2.5 - 17.8	0.22
3010101	4602	SNOW CR	165.0	22.4	8 - 56	0.0	0.0 - 0.0	2.1	0.7 - 5.2	3.2	1.0 - 10.3	10.0	3.3 - 30.1	0.0	0.0 - 0.0	7.1	2.7 - 18.3	0.21
3010101	4603	TURKEYCOCK CR	99.2	19.7	6 - 40	0.0	0.0 - 0.0	2.8	0.8 - 6.0	1.8	0.5 - 5.4	8.2	2.5 - 19.7	0.0	0.0 - 0.0	7.0	2.0 - 16.8	0.21
3010101	4604	TOMAHAWK CR	47.6	25.2	7 - 42	0.0	0.0 - 0.0	4.0	1.2 - 7.4	2.6	0.8 - 7.1	11.6	3.3 - 24.7	0.0	0.0 - 0.0	7.0	1.9 - 16.4	0.22
3010102	4605	ROANOKE CR	5.6	50.1	18 - 129	0.0	0.0 - 0.0	1.8	0.6 - 4.8	5.3	1.6 - 18.8	21.6	7.3 - 64.9	0.0	0.0 - 0.0	21.4	7.1 - 67.1	0.36
3010102	4606	HORSEPEN CR	111.4	28.3	8 - 67	0.0	0.0 - 0.0	2.1	0.6 - 4.9	3.0	0.7 - 8.5	11.0	3.3 - 28.0	0.0	0.0 - 0.0	12.1	3.5 - 32.3	0.36
3010102	4607	ROANOKE CR	12.8	45.1	14 - 99	0.0	0.0 - 0.0	1.2	0.3 - 2.5	4.6	1.2 - 13.7	17.7	6.0 - 55.3	0.0	0.0 - 0.0	21.7	6.8 - 52.0	0.36
3010102	4608	TWITTYS CR	85.2	24.8	11 - 67	0.0	0.0 - 0.0	2.3	0.9 - 5.6	2.3	0.8 - 6.5	7.5	2.9 - 26.2	0.0	0.0 - 0.0	12.7	5.1 - 33.1	0.35
3010102	4609	ROANOKE CR	29.9	29.5	11 - 67	0.0	0.0 - 0.0	2.3	0.8 - 4.7	2.9	0.9 - 8.8	10.4	4.0 - 26.1	0.0	0.0 - 0.0	14.0	4.8 - 35.3	0.35
3010102	4610	ROANOKE CR	183.5	21.4	9 - 51	0.0	0.0 - 0.0	2.5	1.0 - 5.4	2.3	0.7 - 6.6	8.3	2.8 - 22.1	0.0	0.0 - 0.0	8.2	3.2 - 21.9	0.34
3010102	4611	*A	138.5	26.4	8 - 58	0.0	0.0 - 0.0	2.4	0.7 - 4.1	3.0	0.7 - 8.3	11.0	3.2 - 29.4	0.0	0.0 - 0.0	10.1	3.4 - 22.9	0.34
3010102	4612	ROANOKE R	157.3	27.6	8 - 62	0.0	0.0 - 0.0	2.6	0.8 - 5.5	2.4	0.7 - 7.0	10.0	2.6 - 25.2	0.0	0.0 - 0.0	12.6	3.8 - 29.5	0.36
3010102	4613	CLUB CR	10.3	26.9	10 - 63	0.0	0.0 - 0.0	1.5	0.5 - 3.4	2.8	0.8 - 8.2	10.0	3.5 - 25.9	0.0	0.0 - 0.0	12.6	4.2 - 29.9	0.36
3010102	4614	CLUB CR	77.8	24.3	9 - 58	0.0	0.0 - 0.0	2.7	0.9 - 6.8	2.3	0.8 - 7.2	9.0	3.1 - 25.2	0.0	0.0 - 0.0	10.3	3.5 - 27.0	0.35
3010102	4615	*B	20.8	28.0	9 - 55	0.0	0.0 - 0.0	2.9	0.9 - 5.6	2.9	0.8 - 10.3	11.5	3.9 - 28.3	0.0	0.0 - 0.0	10.7	3.1 - 23.6	0.32
3010102	4616	CLUB CR	225.3	28.6	11 - 75	0.0	0.0 - 0.0	2.3	0.8 - 6.2	3.3	1.1 - 12.1	11.0	3.4 - 37.6	0.0	0.0 - 0.0	12.1	4.5 - 33.1	0.32
3010102	4617	*C	51.8	24.7	8 - 47	0.0	0.0 - 0.0	2.7	0.8 - 5.7	2.8	0.8 - 6.6	10.6	3.3 - 26.4	0.0	0.0 - 0.0	8.6	2.7 - 23.6	0.35
3010102	4618	ROANOKE R	16.5	35.9	13 - 76	0.0	0.0 - 0.0	1.5	0.6 - 3.0	3.6	1.3 - 11.2	14.5	5.6 - 36.0	0.0	0.0 - 0.0	16.1	5.3 - 37.9	0.36
3010102	4619	ROANOKE R	28.9	39.3	13 - 92	0.0	0.0 - 0.0	3.4	1.1 - 7.5	3.3	1.0 - 9.2	15.0	4.8 - 41.2	0.0	0.0 - 0.0	17.6	6.1 - 46.3	0.35
3010102	4620	TURNIP CR	89.6	22.5	7 - 53	0.0	0.0 - 0.0	3.1	1.1 - 6.8	2.4	0.7 - 6.4	8.7	2.6 - 24.2	0.0	0.0 - 0.0	8.3	2.6 - 21.6	0.35
3010102	4621	ROANOKE R	13.6	20.8	8 - 37	0.0	0.0 - 0.0	2.3	0.8 - 4.3	0.8	0.2 - 2.3	3.8	1.4 - 8.9	0.0	0.0 - 0.0	14.0	4.7 - 31.3	0.35
3010102	4622	ROANOKE R	90.6	25.5	8 - 51	0.0	0.0 - 0.0	5.0	1.4 - 10.8	1.6	0.4 - 4.3	8.2	2.4 - 18.4	0.0	0.0 - 0.0	10.7	3.3 - 24.7	0.35
3010102	4623	FALLING R	64.1	27.6	9 - 58	0.0	0.0 - 0.0	6.0	1.7 - 13.6	2.8	0.7 - 7.4	9.7	3.1 - 25.5	0.0	0.0 - 0.0	9.0	3.0 - 20.2	0.35
3010102	4624	LITTLE FALLING R	115.2	36.6	14 - 101	0.0	0.0 - 0.0	2.4	0.9 - 5.5	4.8	1.3 - 17.1	15.8	5.9 - 45.4	0.0	0.0 - 0.0	13.6	4.9 - 42.7	0.34
3010102	4625	FALLING R	10.4	51.5	21 - 133	0.0	0.0 - 0.0	6.1	2.3 - 15.9	6.6	2.3 - 24.8	23.2	9.2 - 65.2	0.0	0.0 - 0.0	15.5	6.1 - 43.4	0.34
3010102	4626	FALLING R	14.1	19.3	5 - 41	0.0	0.0 - 0.0	1.2	0.3 - 2.2	1.7	0.5 - 4.3	3.8	1.2 - 8.5	0.0	0.0 - 0.0	12.6	3.7 - 30.9	0.33
3010102	4627	FALLING R	16.5	27.2	9 - 62	0.0	0.0 - 0.0	1.9	0.6 - 4.0	3.1	0.9 - 8.0	10.7	3.3 - 27.8	0.0	0.0 - 0.0	11.5	3.8 - 33.7	0.32
3010102	4628	FALLING R	167.7	37.0	13 - 81	0.0	0.0 - 0.0	3.6	1.2 - 7.7	4.4	1.3 - 13.9	14.7	4.7 - 33.4	0.0	0.0 - 0.0	14.3	4.7 - 34.0	0.32

8-digit HUC	ID	Name	Area	Catchment Yield		Point sources		Developed Land		Manure		Agricultural Land		Phosphate Mines		Soil parent rock		Frac
				kg/km^2	90% CI	kg/km^2	90% CI	kg/km^2	90% CI	kg/km^2	90% CI	kg/km^2	90% CI	kg/km^2	90% CI	kg/km^2	90% CI	
3010102	4629	FALLING R, S FK	124.8	29.7	10 - 61	0.0	0.0 - 0.0	3.0	1.1 - 6.0	3.6	1.1 - 9.7	12.9	4.7 - 31.4	0.0	0.0 - 0.0	10.2	3.7 - 23.0	0.32
3010102	4630	*D	73.1	35.5	11 - 91	0.0	0.0 - 0.0	4.4	1.3 - 11.0	4.7	1.1 - 14.5	16.7	5.9 - 46.7	0.0	0.0 - 0.0	9.7	3.1 - 25.0	0.32
3010102	4631	*E	36.1	34.8	13 - 72	0.0	0.0 - 0.0	3.8	1.3 - 7.5	4.8	1.5 - 11.4	16.4	5.8 - 39.8	0.0	0.0 - 0.0	9.8	3.9 - 21.9	0.33
3010102	4632	ROANOKE R	33.2	23.4	8 - 62	0.0	0.0 - 0.0	6.5	2.5 - 16.4	1.4	0.5 - 4.3	5.6	2.0 - 17.9	0.0	0.0 - 0.0	9.8	3.4 - 28.3	0.35
3010102	4633	*F	59.8	28.4	9 - 56	0.0	0.0 - 0.0	4.4	1.4 - 9.5	3.4	1.1 - 10.3	11.4	3.6 - 27.1	0.0	0.0 - 0.0	9.2	3.2 - 23.4	0.35
3010102	4634	ROANOKE R	61.9	24.5	8 - 45	0.0	0.0 - 0.0	4.4	1.4 - 9.9	2.0	0.6 - 5.5	8.9	3.0 - 20.1	0.0	0.0 - 0.0	9.2	2.9 - 20.0	0.35
3010102	4635	ROANOKE R	35.5	28.5	11 - 64	0.0	0.0 - 0.0	3.8	1.5 - 8.3	3.9	1.5 - 11.9	13.1	5.2 - 35.5	0.0	0.0 - 0.0	7.7	3.1 - 21.5	0.34
3010102	4636	SENECA R	143.7	29.7	9 - 56	0.0	0.0 - 0.0	3.6	1.1 - 6.6	3.3	0.9 - 8.9	11.2	4.1 - 25.8	0.0	0.0 - 0.0	11.6	3.3 - 25.8	0.34
3010102	4637	ROANOKE R	127.3	29.2	10 - 71	0.0	0.0 - 0.0	4.2	1.4 - 11.0	2.6	0.8 - 8.1	10.6	3.7 - 30.8	0.0	0.0 - 0.0	11.7	3.9 - 30.7	0.34
3010102	4638	*G	93.3	28.8	9 - 66	0.0	0.0 - 0.0	4.5	1.4 - 10.2	3.0	0.8 - 7.9	13.4	4.4 - 31.5	0.0	0.0 - 0.0	7.9	2.4 - 18.8	0.34
3010102	4639	CATAWBA CR	71.5	23.0	7 - 54	0.0	0.0 - 0.0	3.8	1.2 - 8.7	1.5	0.4 - 4.3	7.3	2.3 - 18.7	0.0	0.0 - 0.0	10.4	2.9 - 27.5	0.35
3010102	4640	BUCKSKIN CR	30.9	26.4	9 - 52	0.0	0.0 - 0.0	3.5	1.1 - 7.6	2.0	0.6 - 5.6	9.4	3.1 - 22.8	0.0	0.0 - 0.0	11.6	3.8 - 28.5	0.35
3010102	4641	GRASSY CR	95.6	24.5	7 - 43	0.0	0.0 - 0.0	2.7	0.7 - 5.1	1.2	0.3 - 3.2	8.2	2.8 - 18.9	0.0	0.0 - 0.0	12.5	3.2 - 29.1	0.36
3010102	4642	LITTLE GRASSY C	62.5	25.3	9 - 60	0.0	0.0 - 0.0	2.7	1.0 - 6.6	1.3	0.4 - 3.6	8.8	3.5 - 25.6	0.0	0.0 - 0.0	12.5	4.9 - 30.3	0.36
3010102	4643	ISLAND CR	102.4	24.4	9 - 52	0.0	0.0 - 0.0	3.1	1.1 - 7.4	1.3	0.4 - 4.0	8.3	2.7 - 20.9	0.0	0.0 - 0.0	11.7	3.9 - 28.9	0.36
3010102	4644	LITTLE ISLAND CR	55.9	13.8	5 - 34	0.0	0.0 - 0.0	3.0	1.0 - 7.2	0.3	0.1 - 1.1	4.4	1.5 - 10.6	0.0	0.0 - 0.0	6.1	2.2 - 19.6	0.36
3010103	4645	SANDY CR	84.2	33.1	12 - 79	0.0	0.0 - 0.0	10.6	3.6 - 23.6	2.5	0.8 - 6.1	10.8	3.6 - 31.1	0.0	0.0 - 0.0	9.2	3.2 - 23.3	0.35
3010103	4646	DAN R	1.2	97.9	37 - 173	0.0	0.0 - 0.0	70.0	26.3 - 131.9	0.0	0.0 - 0.0	4.8	1.8 - 9.8	0.0	0.0 - 0.0	23.1	9.3 - 54.9	0.35
3010103	4647	SANDY R	139.7	32.3	9 - 67	0.0	0.0 - 0.0	8.0	2.3 - 15.4	2.5	0.6 - 6.3	11.3	3.1 - 29.8	0.0	0.0 - 0.0	10.6	3.0 - 25.0	0.35
3010103	4648	SANDY R	38.0	26.4	9 - 60	0.0	0.0 - 0.0	4.0	1.4 - 9.5	2.6	0.7 - 7.7	11.5	4.1 - 30.8	0.0	0.0 - 0.0	8.4	2.9 - 20.9	0.31
3010103	4649	SANDY R	28.3	26.3	9 - 59	0.0	0.0 - 0.0	4.6	1.6 - 10.9	2.4	0.8 - 8.7	11.3	4.0 - 28.5	0.0	0.0 - 0.0	7.9	2.7 - 20.1	0.29
3010103	4650	FLIPPINS CR	34.0	20.4	7 - 35	0.0	0.0 - 0.0	2.5	0.7 - 4.7	1.6	0.4 - 4.4	8.0	2.6 - 17.7	0.0	0.0 - 0.0	8.3	2.7 - 17.6	0.29
3010103	4651	SANDY R, GLADY	71.1	27.3	9 - 74	0.0	0.0 - 0.0	4.0	1.4 - 9.4	2.3	0.6 - 7.4	12.0	4.2 - 34.3	0.0	0.0 - 0.0	9.0	3.1 - 23.5	0.31
3010103	4652	DAN R	49.3	44.9	15 - 86	0.0	0.0 - 0.0	20.7	6.5 - 42.5	1.5	0.5 - 4.3	7.6	2.3 - 19.6	0.0	0.0 - 0.0	15.1	5.5 - 36.9	0.35
3010103	4653	DAN R	112.0	25.5	9 - 51	0.0	0.0 - 0.0	3.4	1.1 - 7.5	2.0	0.7 - 5.4	10.0	3.6 - 24.9	0.0	0.0 - 0.0	10.0	3.6 - 24.5	0.34
3010103	4654	MCGUFF CR	1.3	272.2	104 - 668	229.2	87.1 - 553.6	0.7	0.3 - 1.4	3.6	1.4 - 10.4	28.0	9.6 - 73.7	0.0	0.0 - 0.0	10.7	4.1 - 25.6	0.34
3010103	4655	MCGUFF CR	23.7	26.1	9 - 52	0.0	0.0 - 0.0	2.9	0.8 - 6.4	2.5	0.7 - 9.1	11.9	4.1 - 25.3	0.0	0.0 - 0.0	8.9	2.7 - 21.9	0.33
3010103	4656	TROTTERS CR	85.1	26.0	10 - 48	0.0	0.0 - 0.0	6.2	2.1 - 13.1	1.7	0.5 - 4.3	7.9	2.8 - 18.3	0.0	0.0 - 0.0	10.2	3.9 - 21.1	0.33
3010103	4657	DAN R	28.4	30.1	8 - 63	0.0	0.0 - 0.0	10.3	2.8 - 23.2	1.2	0.3 - 3.6	8.7	2.2 - 22.3	0.0	0.0 - 0.0	9.9	2.8 - 20.9	0.34
3010103	4658	DAN R	24.0	1171.1	412 - 2309	1133.0	401.2 - 2259.4	20.8	5.9 - 43.5	0.9	0.2 - 2.5	6.7	2.3 - 15.7	0.0	0.0 - 0.0	9.6	3.4 - 22.4	0.33
3010103	4659	SMITH R	125.8	31.8	11 - 78	0.0	0.0 - 0.0	11.5	4.0 - 26.8	1.1	0.3 - 2.9	6.7	2.3 - 18.9	0.0	0.0 - 0.0	12.5	4.4 - 33.2	0.33
3010103	4660	LEATHERWOOD C	47.3	27.1	7 - 61	0.0	0.0 - 0.0	11.5	3.2 - 25.7	1.0	0.2 - 2.9	5.8	1.5 - 16.7	0.0	0.0 - 0.0	8.7	2.6 - 22.0	0.32
3010103	4661	LEATHERWOOD C	67.8	19.1	6 - 37	0.0	0.0 - 0.0	3.6	1.0 - 7.0	1.1	0.3 - 3.2	6.6	2.0 - 16.0	0.0	0.0 - 0.0	7.8	2.1 - 16.2	0.29
3010103	4662	LEATHERWOOD C	76.3	16.4	5 - 34	0.0	0.0 - 0.0	2.7	0.8 - 5.9	0.8	0.2 - 2.3	5.1	1.6 - 13.0	0.0	0.0 - 0.0	7.8	2.8 - 19.1	0.29
3010103	4663	SMITH R	8.7	17.1	7 - 38	0.0	0.0 - 0.0	1.4	0.5 - 3.4	0.9	0.3 - 2.7	4.8	1.9 - 14.2	0.0	0.0 - 0.0	9.9	3.8 - 26.2	0.32
3010103	4664	SMITH R	72.2	212.1	72 - 457	168.5	55.1 - 386.1	27.6	8.8 - 65.6	0.8	0.2 - 2.4	4.8	1.6 - 13.1	0.0	0.0 - 0.0	10.3	3.6 - 24.9	0.32
3010103	4665	BEAVER CR	67.4	40.8	13 - 74	0.0	0.0 - 0.0	25.5	7.9 - 51.5	0.9	0.3 - 2.9	5.0	1.5 - 11.4	0.0	0.0 - 0.0	9.5	3.2 - 22.6	0.30
3010103	4666	SMITH R	58.0	95.7	34 - 222	58.6	20.6 - 132.0	21.5	6.6 - 52.1	1.0	0.3 - 3.1	5.3	1.6 - 15.7	0.0	0.0 - 0.0	9.2	3.0 - 24.2	0.30
3010103	4667	REED CR	75.5	26.6	10 - 66	0.0	0.0 - 0.0	10.8	4.1 - 28.4	1.3	0.4 - 3.9	6.3	2.2 - 17.3	0.0	0.0 - 0.0	8.2	2.9 - 20.8	0.30

8-digit HUC	ID	Name	Area	Catchment Yield		Point sources		Developed Land		Manure		Agricultural Land		Phosphate Mines		Soil parent rock		Frac
				kg/km^2	90% CI	kg/km^2	90% CI	kg/km^2	90% CI	kg/km^2	90% CI	kg/km^2	90% CI	kg/km^2	90% CI	kg/km^2	90% CI	
3010103	4668	SMITH R	88.0	27.8	9 - 63	0.0	0.0 - 0.0	13.6	4.5 - 30.2	0.7	0.2 - 2.2	3.6	1.3 - 11.3	0.0	0.0 - 0.0	9.8	2.9 - 26.0	0.30
3010103	4669	*A	99.2	15.1	5 - 26	0.0	0.0 - 0.0	2.8	1.0 - 5.7	1.2	0.3 - 2.9	3.8	1.2 - 8.5	0.0	0.0 - 0.0	7.3	2.5 - 16.2	0.29
3010103	4670	BEARDS CR	54.4	16.6	5 - 28	0.0	0.0 - 0.0	3.2	0.9 - 6.7	1.9	0.5 - 4.3	5.9	1.8 - 12.9	0.0	0.0 - 0.0	5.7	1.8 - 12.2	0.08
3010103	4671	SMITH R	10.1	18.3	7 - 48	0.0	0.0 - 0.0	3.2	1.2 - 8.0	0.9	0.3 - 2.4	2.7	0.9 - 7.5	0.0	0.0 - 0.0	11.5	4.0 - 29.9	0.08
3010103	4672	OTTER CR	3.3	8.6	3 - 16	0.0	0.0 - 0.0	1.2	0.4 - 2.3	0.2	0.1 - 0.5	0.5	0.2 - 1.2	0.0	0.0 - 0.0	6.8	2.1 - 14.5	0.07
3010103	4673	OTTER CR	38.3	9.0	3 - 17	0.0	0.0 - 0.0	1.9	0.6 - 3.9	0.7	0.2 - 1.6	2.0	0.6 - 5.2	0.0	0.0 - 0.0	4.4	1.4 - 10.1	0.07
3010103	4674	RUNNETT BAG CR	43.3	9.1	3 - 24	0.0	0.0 - 0.0	2.3	0.8 - 5.3	0.5	0.2 - 1.3	1.5	0.4 - 4.7	0.0	0.0 - 0.0	4.9	1.8 - 12.7	0.07
3010103	4675	SMITH R	127.6	15.2	5 - 33	0.0	0.0 - 0.0	3.0	0.9 - 6.8	0.7	0.2 - 2.1	2.1	0.7 - 5.8	0.0	0.0 - 0.0	9.3	2.8 - 20.8	0.07
3010103	4676	SMITH R	1.6	29.4	9 - 57	0.0	0.0 - 0.0	0.0	0.0 - 0.0	3.3	1.1 - 8.4	11.4	3.6 - 26.4	0.0	0.0 - 0.0	14.7	4.9 - 32.7	0.07
3010103	4677	ROCK CASTLE CR	42.1	19.8	7 - 39	0.0	0.0 - 0.0	5.2	1.8 - 9.8	1.0	0.3 - 2.4	3.1	1.0 - 8.4	0.0	0.0 - 0.0	10.6	3.3 - 23.1	0.06
3010103	4678	SMITH R	79.6	23.7	9 - 56	0.0	0.0 - 0.0	4.8	1.7 - 11.9	1.9	0.6 - 5.8	5.6	1.7 - 17.3	0.0	0.0 - 0.0	11.4	4.3 - 29.0	0.06
3010103	4679	SYCAMORE CR	47.7	21.9	7 - 48	0.0	0.0 - 0.0	5.0	1.6 - 12.0	1.3	0.4 - 3.5	3.7	1.2 - 10.1	0.0	0.0 - 0.0	11.9	3.9 - 32.5	0.07
3010103	4680	MARROWBONE CI	77.9	21.7	7 - 41	0.0	0.0 - 0.0	7.3	2.4 - 14.1	0.9	0.2 - 3.0	4.4	1.3 - 10.2	0.0	0.0 - 0.0	9.1	2.6 - 22.9	0.32
3010103	4681	DAN R	7.7	56.7	21 - 119	0.0	0.0 - 0.0	42.6	14.1 - 91.8	0.7	0.2 - 1.7	4.4	1.6 - 9.6	0.0	0.0 - 0.0	9.0	3.6 - 20.2	0.33
3010103	4682	MATRIMONY CR	67.0	30.2	11 - 72	0.0	0.0 - 0.0	10.8	3.7 - 26.1	1.0	0.3 - 2.8	6.7	2.4 - 19.8	0.0	0.0 - 0.0	11.7	4.2 - 28.6	0.33
3010103	4683	DAN R	1.5	35.0	12 - 92	0.0	0.0 - 0.0	7.2	2.5 - 18.3	2.1	0.6 - 7.7	15.1	5.0 - 41.2	0.0	0.0 - 0.0	10.7	3.7 - 37.5	0.33
3010103	4684	BUFFALO CR	58.5	26.0	8 - 64	0.3	0.1 - 0.7	7.6	2.3 - 19.0	1.2	0.3 - 3.4	9.3	3.3 - 25.5	0.0	0.0 - 0.0	7.6	2.6 - 20.2	0.33
3010103	4685	DAN R	175.4	25.9	9 - 48	0.0	0.0 - 0.0	6.2	2.2 - 12.1	1.2	0.3 - 3.0	8.9	2.9 - 22.4	0.0	0.0 - 0.0	9.5	3.1 - 23.0	0.33
3010103	4686	DAN R	4.7	26.6	8 - 64	0.0	0.0 - 0.0	2.8	0.9 - 6.1	1.8	0.5 - 5.8	12.0	3.9 - 31.2	0.0	0.0 - 0.0	10.0	2.8 - 28.2	0.32
3010103	4687	DAN R	9.1	27.3	8 - 61	0.0	0.0 - 0.0	6.4	1.8 - 13.9	1.3	0.3 - 3.1	8.8	2.1 - 25.3	0.0	0.0 - 0.0	10.7	3.0 - 26.8	0.32
3010103	4688	MAYO R	157.5	34.5	13 - 70	11.2	4.0 - 24.1	7.6	2.6 - 15.6	0.7	0.2 - 2.0	4.2	1.4 - 10.1	0.0	0.0 - 0.0	10.8	4.2 - 27.8	0.32
3010103	4689	MAYO R	14.6	16.8	5 - 29	0.0	0.0 - 0.0	1.5	0.5 - 3.1	1.0	0.3 - 2.3	5.2	1.8 - 11.7	0.0	0.0 - 0.0	9.2	2.9 - 20.3	0.30
3010103	4690	HORSE PASTURE (	70.0	23.3	9 - 53	0.0	0.0 - 0.0	6.1	2.4 - 13.3	1.3	0.5 - 3.5	7.6	2.8 - 18.8	0.0	0.0 - 0.0	8.4	3.1 - 19.5	0.29
3010103	4691	N MAYO R	18.9	21.1	8 - 54	0.0	0.0 - 0.0	4.4	1.6 - 12.1	1.1	0.3 - 3.5	6.4	2.2 - 19.6	0.0	0.0 - 0.0	9.2	3.2 - 22.2	0.29
3010103	4693	KOGER CR	37.1	19.0	7 - 37	0.0	0.0 - 0.0	3.8	1.4 - 8.7	1.1	0.3 - 2.9	5.5	1.9 - 13.4	0.0	0.0 - 0.0	8.6	3.1 - 18.2	0.27
3010103	4694	N MAYO R	98.8	20.9	7 - 39	0.0	0.0 - 0.0	3.7	1.2 - 7.0	1.8	0.5 - 5.3	4.9	1.7 - 12.0	0.0	0.0 - 0.0	10.4	3.6 - 24.0	0.27
3010103	4695	MILL CR	43.2	21.0	7 - 39	0.0	0.0 - 0.0	4.6	1.6 - 9.2	2.1	0.6 - 5.3	5.5	1.7 - 14.7	0.0	0.0 - 0.0	8.9	3.1 - 17.6	0.27
3010103	4696	S MAYO R	4.3	15.3	6 - 33	0.0	0.0 - 0.0	2.1	0.7 - 4.4	0.7	0.2 - 1.8	3.7	1.3 - 8.1	0.0	0.0 - 0.0	8.8	3.0 - 22.3	0.30
3010103	4697	S MAYO R	35.7	18.5	7 - 52	0.0	0.0 - 0.0	2.5	1.0 - 6.9	1.7	0.6 - 6.6	4.9	1.7 - 16.0	0.0	0.0 - 0.0	9.4	3.0 - 31.0	0.29
3010103	4698	SPOON CR	52.6	25.8	10 - 48	0.0	0.0 - 0.0	6.1	2.1 - 11.8	2.7	0.8 - 6.9	7.4	2.5 - 16.9	0.0	0.0 - 0.0	9.6	3.3 - 22.2	0.28
3010103	4699	S MAYO R	10.9	23.9	8 - 50	0.0	0.0 - 0.0	5.0	1.6 - 11.0	2.4	0.7 - 6.6	6.7	2.1 - 17.6	0.0	0.0 - 0.0	9.8	3.2 - 24.6	0.28
3010103	4700	S MAYO R	96.8	33.2	11 - 76	3.6	1.3 - 8.1	8.4	2.6 - 19.0	2.5	0.7 - 7.5	7.3	2.3 - 17.1	0.0	0.0 - 0.0	11.5	3.8 - 26.7	0.27
3010103	4701	POORHOUSE CR	20.4	19.1	7 - 45	0.0	0.0 - 0.0	2.6	0.8 - 6.0	1.1	0.3 - 2.9	3.4	1.0 - 7.9	0.0	0.0 - 0.0	12.0	4.4 - 27.9	0.22
3010103	4702	S MAYO R	44.6	28.3	9 - 77	0.0	0.0 - 0.0	4.5	1.5 - 10.7	2.6	0.7 - 9.0	7.9	2.4 - 26.1	0.0	0.0 - 0.0	13.3	4.0 - 38.9	0.22
3010103	4703	RUSSELL CR	65.8	32.2	13 - 67	0.0	0.0 - 0.0	5.8	2.1 - 13.2	4.2	1.2 - 12.8	13.1	4.6 - 36.9	0.0	0.0 - 0.0	9.0	3.7 - 20.6	0.27
3010103	4704	CROOKED CR	52.0	23.6	7 - 45	0.0	0.0 - 0.0	4.4	1.2 - 10.0	2.2	0.5 - 5.9	8.9	2.6 - 20.2	0.0	0.0 - 0.0	8.2	2.5 - 18.2	0.29
3010103	4705	DAN R	9.6	126.6	42 - 235	88.0	29.0 - 181.7	17.7	5.2 - 37.5	1.1	0.3 - 3.0	9.3	2.9 - 22.4	0.0	0.0 - 0.0	10.5	3.1 - 23.6	0.32
3010103	4707	ISLAND CR	62.6	24.5	8 - 46	0.0	0.0 - 0.0	7.5	2.3 - 16.7	1.0	0.3 - 2.9	5.8	1.7 - 13.9	0.0	0.0 - 0.0	10.2	3.5 - 23.5	0.31
3010103	4708	REED CR	36.9	22.9	7 - 55	0.0	0.0 - 0.0	6.2	1.8 - 15.0	1.3	0.4 - 4.1	6.4	2.1 - 17.3	0.0	0.0 - 0.0	9.0	3.1 - 22.7	0.31

8-digit HUC	ID	Name	Area	Catchment Yield kg/km^2	Catchment Yield 90% CI	Point sources kg/km^2	Point sources 90% CI	Developed Land kg/km^2	Developed Land 90% CI	Manure kg/km^2	Manure 90% CI	Agricultural Land kg/km^2	Agricultural Land 90% CI	Phosphate Mines kg/km^2	Phosphate Mines 90% CI	Soil parent rock kg/km^2	Soil parent rock 90% CI	Frac
3010103	4709	DAN R	64.6	26.4	10 - 66	0.0	0.0 - 0.0	5.4	1.8 - 13.5	1.9	0.7 - 6.1	10.4	4.2 - 29.9	0.0	0.0 - 0.0	8.7	3.1 - 21.7	0.32
3010103	4710	DAN R	75.1	23.6	8 - 44	0.0	0.0 - 0.0	5.4	1.8 - 9.9	1.5	0.4 - 3.5	6.0	2.0 - 14.2	0.0	0.0 - 0.0	10.6	3.1 - 25.3	0.31
3010103	4711	SNOW CR	108.1	23.2	8 - 45	0.0	0.0 - 0.0	4.8	1.5 - 9.9	1.6	0.4 - 3.7	7.3	2.5 - 17.7	0.0	0.0 - 0.0	9.6	3.4 - 22.0	0.30
3010103	4712	DAN R	112.2	21.1	6 - 55	1.2	0.4 - 3.1	4.3	1.4 - 9.1	0.8	0.2 - 2.5	3.5	1.0 - 11.8	0.0	0.0 - 0.0	11.3	3.4 - 34.3	0.30
3010103	4713	DAN R	3.7	26.4	10 - 48	0.0	0.0 - 0.0	5.9	1.8 - 11.0	1.4	0.5 - 4.4	6.6	2.3 - 15.7	0.0	0.0 - 0.0	12.4	4.5 - 25.2	0.29
3010103	4714	DAN R	8.3	22.1	8 - 52	3.1	1.2 - 7.0	4.5	1.6 - 10.6	1.3	0.4 - 3.4	5.6	1.8 - 14.4	0.0	0.0 - 0.0	7.6	2.7 - 18.8	0.29
3010103	4715	PETERS CR	83.9	25.4	8 - 53	0.0	0.0 - 0.0	5.1	1.5 - 11.6	2.1	0.6 - 4.9	7.6	2.7 - 20.4	0.0	0.0 - 0.0	10.6	3.5 - 23.8	0.29
3010103	4716	DAN R	12.3	21.5	7 - 37	0.0	0.0 - 0.0	4.5	1.2 - 8.3	1.4	0.4 - 3.9	6.2	1.8 - 12.7	0.0	0.0 - 0.0	9.4	2.7 - 22.5	0.29
3010103	4717	ELK CR	24.8	26.5	9 - 52	0.0	0.0 - 0.0	4.2	1.4 - 8.3	2.4	0.7 - 7.0	8.2	3.1 - 20.5	0.0	0.0 - 0.0	11.6	4.4 - 26.9	0.28
3010103	4718	DAN R	26.4	27.0	10 - 68	0.0	0.0 - 0.0	5.7	1.9 - 13.9	2.0	0.7 - 6.4	8.9	3.0 - 22.7	0.0	0.0 - 0.0	10.5	3.7 - 25.8	0.28
3010103	4719	LITTLE DAN R	29.1	28.7	9 - 67	0.0	0.0 - 0.0	5.9	2.1 - 13.3	3.0	0.9 - 8.2	9.0	3.1 - 27.4	0.0	0.0 - 0.0	10.8	3.3 - 27.2	0.27
3010103	4720	LITTLE DAN R	39.1	15.1	5 - 29	0.0	0.0 - 0.0	2.6	0.8 - 5.9	1.1	0.3 - 3.2	3.1	1.0 - 7.6	0.0	0.0 - 0.0	8.3	2.5 - 17.4	0.25
3010103	4721	HOOKERS CR	14.5	18.9	6 - 47	0.0	0.0 - 0.0	4.4	1.3 - 10.4	1.7	0.5 - 4.7	4.9	1.6 - 13.3	0.0	0.0 - 0.0	7.8	2.5 - 19.5	0.25
3010103	4722	DAN R	172.7	29.1	10 - 60	0.0	0.0 - 0.0	5.2	1.9 - 10.6	3.4	1.0 - 9.2	9.6	2.9 - 22.2	0.0	0.0 - 0.0	10.8	4.0 - 24.2	0.27
3010103	4723	BIG CR	116.0	24.0	9 - 54	0.0	0.0 - 0.0	5.2	2.0 - 12.0	2.8	0.8 - 7.4	8.3	2.5 - 22.6	0.0	0.0 - 0.0	7.7	2.8 - 16.6	0.29
3010103	4724	N DOUBLE CR	80.1	20.0	6 - 52	0.0	0.0 - 0.0	4.8	1.5 - 11.6	0.9	0.3 - 3.0	4.0	1.6 - 11.6	0.0	0.0 - 0.0	10.2	3.4 - 28.5	0.29
3010103	4725	NEATMANS CR	96.4	30.2	9 - 75	3.8	1.2 - 10.3	7.4	2.3 - 19.0	1.7	0.5 - 5.3	8.4	2.7 - 24.3	0.0	0.0 - 0.0	9.0	2.9 - 25.0	0.31
3010103	4726	NEATMANS CR	83.3	26.7	8 - 73	0.0	0.0 - 0.0	8.8	2.5 - 22.9	1.4	0.4 - 4.2	7.7	2.5 - 22.6	0.0	0.0 - 0.0	8.8	2.7 - 27.7	0.29
3010103	4727	NEATMANS CR	51.2	23.6	8 - 47	0.6	0.2 - 1.3	4.3	1.5 - 10.5	1.4	0.4 - 3.5	6.3	2.3 - 15.7	0.0	0.0 - 0.0	11.0	3.7 - 24.2	0.28
3010103	4728	NEATMANS CR, T(	72.1	24.5	7 - 65	1.0	0.3 - 2.6	5.7	1.6 - 16.4	1.4	0.4 - 4.8	6.4	2.2 - 19.1	0.0	0.0 - 0.0	10.1	3.0 - 25.3	0.28
3010103	4729	OLD FIELD CR	44.1	21.4	7 - 49	0.0	0.0 - 0.0	6.7	2.1 - 15.4	0.9	0.2 - 2.7	5.6	1.8 - 15.7	0.0	0.0 - 0.0	8.2	2.5 - 21.8	0.29
3010103	4730	BELEWS CR	7.8	20.5	7 - 39	0.0	0.0 - 0.0	2.9	0.8 - 6.0	0.9	0.3 - 2.3	6.7	2.3 - 15.7	0.0	0.0 - 0.0	10.1	3.6 - 25.2	0.31
3010103	4731	HOGAN CR	63.3	30.9	9 - 58	0.5	0.1 - 0.9	6.3	1.5 - 13.6	1.5	0.4 - 4.0	12.5	3.9 - 29.3	0.0	0.0 - 0.0	10.1	2.6 - 23.7	0.32
3010103	4732	JACOBS CR	96.6	22.2	7 - 35	0.4	0.1 - 0.7	3.9	1.2 - 7.2	1.0	0.2 - 2.6	8.2	2.6 - 17.7	0.0	0.0 - 0.0	8.7	2.5 - 17.1	0.32
3010103	4733	*B	39.6	20.3	6 - 38	0.0	0.0 - 0.0	4.8	1.4 - 9.7	0.8	0.2 - 2.0	6.2	1.8 - 13.9	0.0	0.0 - 0.0	8.5	2.6 - 17.2	0.33
3010103	4734	WOLF ISLAND CR	178.2	30.2	10 - 77	0.0	0.0 - 0.1	8.5	2.6 - 23.2	1.3	0.4 - 4.2	10.5	3.4 - 32.3	0.0	0.0 - 0.0	9.9	3.3 - 26.6	0.34
3010103	4735	DAN R	30.8	26.1	12 - 61	0.0	0.0 - 0.0	6.8	2.8 - 13.7	2.0	0.8 - 5.7	8.0	3.0 - 21.6	0.0	0.0 - 0.0	9.3	4.1 - 23.8	0.31
3010104	4736	DAN R	12.4	40.8	15 - 79	0.0	0.0 - 0.0	0.9	0.3 - 2.0	3.3	1.0 - 9.6	13.3	4.2 - 31.5	0.0	0.0 - 0.0	23.2	7.8 - 48.9	0.37
3010104	4737	DAN R	43.2	35.0	11 - 74	0.0	0.0 - 0.0	2.4	0.7 - 5.5	2.2	0.6 - 5.7	8.8	2.6 - 22.3	0.0	0.0 - 0.0	21.5	6.9 - 56.4	0.37
3010104	4738	DAN R	75.0	86.8	26 - 221	36.5	11.5 - 93.4	18.7	5.2 - 45.1	2.4	0.7 - 6.8	10.1	2.9 - 29.3	0.0	0.0 - 0.0	19.2	5.9 - 57.9	0.36
3010104	4739	DAN R	132.5	37.5	12 - 77	0.0	0.0 - 0.0	4.6	1.5 - 10.1	3.0	0.9 - 9.5	13.5	4.4 - 33.1	0.0	0.0 - 0.0	16.4	5.4 - 39.0	0.36
3010104	4740	BIRCH CR	163.7	22.7	8 - 50	0.0	0.0 - 0.0	3.6	1.2 - 8.5	1.8	0.5 - 5.6	7.5	2.4 - 19.1	0.0	0.0 - 0.0	9.9	3.0 - 21.7	0.36
3010104	4741	DAN R	43.3	33.3	12 - 59	0.0	0.0 - 0.0	3.4	1.1 - 6.7	3.0	1.0 - 8.3	14.1	4.6 - 28.2	0.0	0.0 - 0.0	12.9	4.9 - 25.5	0.36
3010104	4742	DOUBLE CR	62.7	23.2	7 - 59	0.0	0.0 - 0.0	3.3	1.0 - 8.8	2.0	0.6 - 6.8	7.7	2.2 - 20.6	0.0	0.0 - 0.0	10.3	3.4 - 26.7	0.36
3010104	4743	DAN R	52.9	32.5	11 - 64	0.0	0.0 - 0.0	3.9	1.3 - 8.7	2.7	0.8 - 7.1	12.4	3.8 - 30.0	0.0	0.0 - 0.0	13.5	3.9 - 30.5	0.36
3010104	4744	SANDY CR	49.1	28.7	10 - 64	0.0	0.0 - 0.0	5.0	1.7 - 11.1	2.6	0.8 - 8.2	10.4	3.4 - 27.8	0.0	0.0 - 0.0	10.6	3.5 - 26.6	0.35
3010104	4745	DAN R	11.8	51.1	18 - 139	0.0	0.0 - 0.0	5.3	1.6 - 12.4	5.8	1.9 - 16.7	25.1	8.2 - 72.3	0.0	0.0 - 0.0	14.9	5.4 - 41.2	0.35
3010104	4746	DAN R	20.9	25.0	8 - 49	0.0	0.0 - 0.0	2.4	0.8 - 4.6	1.8	0.5 - 5.2	9.6	2.7 - 22.8	0.0	0.0 - 0.0	11.2	3.4 - 25.2	0.35
3010104	4747	CANE CR	57.9	34.2	11 - 66	0.0	0.0 - 0.0	8.9	2.9 - 17.5	2.7	0.8 - 7.9	11.8	3.8 - 31.3	0.0	0.0 - 0.0	10.8	3.2 - 26.0	0.35

8-digit HUC	ID	Name	Area	Catchment Yield		Point sources		Developed Land		Manure		Agricultural Land		Phosphate Mines		Soil parent rock		Frac
				kg/km^2	90% CI	kg/km^2	90% CI	kg/km^2	90% CI	kg/km^2	90% CI	kg/km^2	90% CI	kg/km^2	90% CI	kg/km^2	90% CI	
3010104	4748	DAN R	84.0	28.3	9 - 55	0.0	0.0 - 0.0	4.4	1.4 - 9.5	1.9	0.6 - 7.8	10.4	3.4 - 24.5	0.0	0.0 - 0.0	11.5	3.8 - 28.8	0.35
3010104	4749	DAN R	2.1	29.4	11 - 62	0.0	0.0 - 0.0	1.7	0.6 - 3.5	2.6	0.9 - 7.0	13.8	4.9 - 35.1	0.0	0.0 - 0.0	11.3	3.5 - 27.8	0.35
3010104	4750	DAN R	67.1	47.3	16 - 91	0.0	0.0 - 0.0	25.7	7.5 - 52.9	0.9	0.2 - 2.5	7.6	2.9 - 17.2	0.0	0.0 - 0.0	13.1	4.4 - 33.3	0.35
3010104	4751	LAWLESS CR	97.5	164.5	58 - 440	129.6	44.9 - 358.8	15.3	5.3 - 42.8	1.9	0.6 - 5.5	8.1	2.5 - 23.4	0.0	0.0 - 0.0	9.6	3.5 - 32.6	0.35
3010104	4752	DAN R	12.4	93.5	28 - 197	0.0	0.0 - 0.0	78.6	24.2 - 172.3	0.0	0.0 - 0.0	0.6	0.2 - 1.6	0.0	0.0 - 0.0	14.3	4.4 - 39.3	0.35
3010104	4753	HOGANS CR	266.1	22.5	8 - 45	0.0	0.0 - 0.0	5.7	2.1 - 10.9	1.0	0.3 - 2.7	6.5	2.2 - 16.3	0.0	0.0 - 0.0	9.4	3.4 - 20.1	0.35
3010104	4754	MOON CR	45.1	23.9	9 - 51	0.0	0.0 - 0.0	4.6	1.6 - 10.9	1.4	0.5 - 4.7	7.5	2.6 - 17.5	0.0	0.0 - 0.0	10.2	3.8 - 23.5	0.35
3010104	4755	MOON CR	35.8	22.6	7 - 45	0.0	0.0 - 0.0	2.9	0.9 - 6.3	1.5	0.4 - 4.2	8.8	2.9 - 22.3	0.0	0.0 - 0.0	9.4	3.0 - 22.8	0.30
3010104	4756	MOON CR, E PRON	45.4	22.2	8 - 37	0.0	0.0 - 0.0	2.3	0.8 - 4.7	1.6	0.5 - 5.0	8.7	2.7 - 18.6	0.0	0.0 - 0.0	9.5	3.0 - 22.5	0.30
3010104	4757	COUNTRY LINE C	99.6	23.5	9 - 68	2.5	1.0 - 7.0	2.8	1.0 - 6.8	1.3	0.4 - 3.8	6.5	2.4 - 16.6	0.0	0.0 - 0.0	10.5	3.9 - 34.0	0.35
3010104	4758	COUNTRY LINE C	145.1	26.7	11 - 54	5.4	2.4 - 12.4	3.3	1.1 - 6.7	1.2	0.4 - 2.9	6.5	2.3 - 16.2	0.0	0.0 - 0.0	10.3	4.2 - 24.4	0.30
3010104	4759	PENSON CR	114.4	18.8	6 - 45	0.0	0.0 - 0.0	2.2	0.6 - 4.8	1.2	0.3 - 3.1	5.8	1.9 - 15.5	0.0	0.0 - 0.0	9.6	3.1 - 23.3	0.30
3010104	4760	LAWSONS CR	101.9	40.7	16 - 93	0.0	0.0 - 0.0	6.5	2.5 - 14.3	3.4	1.1 - 9.4	15.2	6.3 - 39.7	0.0	0.0 - 0.0	15.6	5.7 - 36.2	0.36
3010104	4761	HYCO R	65.3	40.8	14 - 86	0.0	0.0 - 0.0	4.0	1.3 - 9.0	3.1	1.0 - 7.9	13.3	4.7 - 30.7	0.0	0.0 - 0.0	20.4	6.9 - 52.4	0.37
3010104	4762	HYCO R	38.1	33.3	12 - 71	0.0	0.0 - 0.0	3.3	1.0 - 7.5	2.1	0.6 - 5.8	7.8	2.4 - 18.8	0.0	0.0 - 0.0	20.2	6.7 - 46.4	0.36
3010104	4763	HYCO R	101.7	39.1	13 - 97	0.0	0.0 - 0.0	3.4	1.0 - 7.6	2.9	0.8 - 8.1	13.0	4.3 - 34.2	0.0	0.0 - 0.0	19.9	6.8 - 60.3	0.35
3010104	4764	HYCO R	25.7	29.4	9 - 76	0.0	0.0 - 0.0	2.6	0.8 - 7.1	2.5	0.7 - 8.1	8.8	3.1 - 22.4	0.0	0.0 - 0.0	15.5	4.0 - 39.0	0.33
3010104	4765	STORYS CR	5.6	14.7	5 - 30	0.0	0.0 - 0.0	0.2	0.0 - 0.3	0.9	0.3 - 2.4	2.1	0.7 - 5.0	0.0	0.0 - 0.0	11.5	4.1 - 25.5	0.33
3010104	4766	STORYS CR	48.4	25.2	9 - 46	0.0	0.0 - 0.0	5.1	1.8 - 10.3	1.5	0.5 - 4.3	9.7	3.6 - 23.4	0.0	0.0 - 0.0	8.8	2.9 - 20.8	0.32
3010104	4767	MARLOWE CR	57.0	86.6	31 - 157	54.4	20.7 - 117.3	13.7	4.4 - 26.4	1.3	0.4 - 3.1	7.4	2.6 - 18.6	0.0	0.0 - 0.0	9.8	3.7 - 23.4	0.32
3010104	4768	MAYO CR	158.2	4.3	1 - 8	0.0	0.0 - 0.0	0.5	0.2 - 1.0	0.3	0.1 - 0.6	1.5	0.4 - 2.9	0.0	0.0 - 0.0	2.0	0.6 - 4.0	0.35
3010104	4769	BIG BLUEWING C	67.7	33.4	13 - 80	0.0	0.0 - 0.0	2.5	0.8 - 5.7	2.4	0.7 - 7.0	11.7	3.6 - 27.4	0.0	0.0 - 0.0	16.7	5.7 - 40.1	0.36
3010104	4770	AARONS CR	171.1	38.3	13 - 81	0.0	0.0 - 0.0	3.2	1.0 - 8.0	3.1	0.9 - 9.1	15.6	5.2 - 37.1	0.0	0.0 - 0.0	16.3	5.3 - 44.0	0.37
3010105	4771	BANISTER R	127.6	38.5	12 - 83	0.0	0.0 - 0.0	6.2	1.8 - 13.2	2.9	0.7 - 8.7	12.7	4.2 - 33.4	0.0	0.0 - 0.0	16.6	5.3 - 41.6	0.36
3010105	4772	TERRIBLE CR	18.3	20.1	6 - 49	0.0	0.0 - 0.0	5.2	1.4 - 12.4	1.2	0.3 - 3.3	4.4	1.4 - 11.0	0.0	0.0 - 0.0	9.3	2.8 - 22.3	0.35
3010105	4773	LITTLE TERRIBLE	43.5	24.3	8 - 45	0.0	0.0 - 0.0	3.6	1.3 - 7.1	1.7	0.4 - 5.2	7.7	2.6 - 17.5	0.0	0.0 - 0.0	11.2	3.6 - 25.4	0.32
3010105	4774	TERRIBLE CR	39.8	28.7	9 - 56	0.0	0.0 - 0.0	4.5	1.4 - 8.8	2.2	0.8 - 6.2	11.0	3.3 - 25.2	0.0	0.0 - 0.0	10.9	3.1 - 29.4	0.32
3010105	4775	BANISTER R	25.0	22.0	9 - 41	0.0	0.0 - 0.0	5.9	2.1 - 10.3	1.2	0.4 - 2.7	4.3	1.5 - 11.6	0.0	0.0 - 0.0	10.7	3.9 - 24.6	0.35
3010105	4776	BANISTER R	22.8	25.5	9 - 50	0.0	0.0 - 0.0	5.1	1.7 - 11.0	1.9	0.5 - 4.6	8.4	3.0 - 19.3	0.0	0.0 - 0.0	10.1	3.1 - 23.1	0.35
3010105	4777	BANISTER R	46.1	30.9	12 - 55	0.0	0.0 - 0.0	3.8	1.5 - 7.3	2.4	0.8 - 6.2	10.6	4.4 - 23.9	0.0	0.0 - 0.0	14.1	5.1 - 31.9	0.34
3010105	4778	BRADLEY CR	29.1	24.0	10 - 60	0.0	0.0 - 0.0	4.5	1.8 - 10.1	1.6	0.6 - 4.1	7.9	3.2 - 22.4	0.0	0.0 - 0.0	10.0	3.7 - 25.2	0.33
3010105	4779	BANISTER R	40.3	33.2	11 - 81	0.0	0.0 - 0.0	4.2	1.4 - 10.0	2.3	0.8 - 6.5	12.1	4.0 - 35.7	0.0	0.0 - 0.0	14.5	4.7 - 43.9	0.33
3010105	4780	BANISTER R	96.0	23.5	8 - 53	0.0	0.0 - 0.0	4.2	1.5 - 8.4	1.8	0.6 - 5.1	8.2	2.4 - 20.3	0.0	0.0 - 0.0	9.3	2.8 - 24.4	0.33
3010105	4781	STINKING CR	90.9	27.5	11 - 63	0.0	0.0 - 0.0	5.2	1.8 - 12.0	2.6	1.0 - 7.5	11.6	4.4 - 33.2	0.0	0.0 - 0.0	8.2	3.1 - 19.7	0.31
3010105	4782	BANISTER R	34.7	24.6	8 - 58	0.0	0.0 - 0.0	3.8	1.2 - 8.3	2.6	0.7 - 6.9	9.9	3.0 - 25.2	0.0	0.0 - 0.0	8.3	2.8 - 20.5	0.31
3010105	4783	WHITEHORN CR	2.0	22.5	8 - 45	0.0	0.0 - 0.0	3.3	0.9 - 6.6	3.3	1.0 - 8.5	10.0	3.5 - 23.7	0.0	0.0 - 0.0	6.0	2.2 - 13.6	0.31
3010105	4784	GEORGES CR	61.3	28.6	10 - 60	0.0	0.0 - 0.0	7.1	2.4 - 15.8	2.5	0.8 - 6.5	11.0	3.8 - 29.5	0.0	0.0 - 0.0	8.0	2.8 - 19.7	0.30
3010105	4785	WHITEHORN CR	2.1	25.3	8 - 52	0.0	0.0 - 0.0	3.2	1.0 - 6.5	3.5	0.9 - 9.7	12.7	4.0 - 28.8	0.0	0.0 - 0.0	5.9	1.8 - 15.5	0.30
3010105	4786	WHITEHORN CR	70.9	34.1	12 - 78	0.0	0.0 - 0.0	7.9	2.6 - 17.7	3.2	0.9 - 9.9	15.0	4.7 - 34.8	0.0	0.0 - 0.0	8.0	2.9 - 21.8	0.30

8-digit HUC	ID	Name	Area	Catchment Yield		Point sources		Developed Land		Manure		Agricultural Land		Phosphate Mines		Soil parent rock		Frac
				kg/km^2	90% CI	kg/km^2	90% CI	kg/km^2	90% CI	kg/km^2	90% CI	kg/km^2	90% CI	kg/km^2	90% CI	kg/km^2	90% CI	
3010105	4787	MILL CR	35.0	27.2	9 - 70	0.0	0.0 - 0.0	5.9	1.7 - 14.7	2.7	0.7 - 9.1	11.7	4.0 - 33.4	0.0	0.0 - 0.0	6.9	2.4 - 18.3	0.30
3010105	4788	BANISTER R	41.0	19.8	8 - 45	0.0	0.0 - 0.0	3.0	1.2 - 6.4	2.8	1.1 - 8.0	8.0	2.9 - 18.5	0.0	0.0 - 0.0	6.1	2.4 - 14.3	0.31
3010105	4789	CHERRYSTONE CI	118.4	29.2	12 - 70	0.0	0.0 - 0.0	7.3	2.9 - 20.0	2.7	0.9 - 7.5	12.1	4.5 - 34.9	0.0	0.0 - 0.0	7.1	2.7 - 16.7	0.28
3010105	4790	BANISTER R	71.8	30.9	12 - 72	0.0	0.0 - 0.0	7.2	2.6 - 15.7	3.0	1.1 - 9.0	13.4	5.4 - 34.7	0.0	0.0 - 0.0	7.3	3.1 - 19.0	0.28
3010105	4791	BEARSKIN CR	57.2	29.7	10 - 70	0.0	0.0 - 0.0	6.2	1.8 - 13.5	3.0	1.0 - 8.3	13.3	4.2 - 40.5	0.0	0.0 - 0.0	7.1	2.6 - 18.2	0.26
3010105	4792	BANISTER R	26.3	33.4	10 - 62	0.0	0.0 - 0.0	6.4	1.9 - 13.6	3.4	0.9 - 9.2	15.7	4.9 - 35.9	0.0	0.0 - 0.0	7.8	2.3 - 18.3	0.26
3010105	4793	WET SLEEVE CR	53.1	25.9	10 - 50	0.0	0.0 - 0.0	4.9	1.6 - 10.4	2.5	0.8 - 6.8	11.2	4.0 - 25.5	0.0	0.0 - 0.0	7.4	2.6 - 17.8	0.24
3010105	4794	STRAWBERRY CR	29.3	25.0	8 - 60	0.0	0.0 - 0.0	4.6	1.3 - 9.8	2.3	0.7 - 6.4	10.8	3.2 - 29.0	0.0	0.0 - 0.0	7.3	2.4 - 18.9	0.24
3010105	4795	ELKHORN CR	58.2	20.6	9 - 44	0.0	0.0 - 0.0	3.8	1.5 - 7.7	1.5	0.5 - 3.7	5.8	2.1 - 14.7	0.0	0.0 - 0.0	9.5	3.9 - 21.4	0.33
3010105	4796	SANDY CR	254.3	23.8	8 - 66	0.0	0.0 - 0.0	4.0	1.4 - 9.2	2.1	0.8 - 5.9	8.3	3.3 - 28.1	0.0	0.0 - 0.0	9.5	3.2 - 32.9	0.34
3010105	4797	POLECAT CR	49.9	28.2	10 - 56	0.0	0.0 - 0.0	3.6	1.2 - 7.7	2.3	0.7 - 6.3	10.4	3.4 - 26.5	0.0	0.0 - 0.0	11.8	4.2 - 27.2	0.35
3010106	4798	FLAT CR	78.1	35.3	13 - 90	13.0	4.5 - 34.1	6.3	2.2 - 14.1	1.9	0.6 - 5.7	6.6	2.0 - 16.8	0.0	0.0 - 0.0	7.6	2.6 - 20.7	0.70
3010106	4799	ROANOKE R	2.9	17.9	5 - 31	0.0	0.0 - 0.0	1.9	0.5 - 3.6	1.9	0.5 - 4.5	6.1	1.7 - 12.0	0.0	0.0 - 0.0	7.9	2.3 - 15.7	0.70
3010106	4800	MILES CR	120.8	23.9	9 - 51	0.0	0.0 - 0.0	4.7	1.7 - 9.8	2.6	0.9 - 7.2	10.5	3.9 - 25.7	0.0	0.0 - 0.0	6.1	2.4 - 15.4	0.70
3010106	4801	ROANOKE R	38.3	21.0	6 - 40	0.0	0.0 - 0.0	2.0	0.5 - 4.1	2.6	0.7 - 6.1	9.3	2.8 - 20.3	0.0	0.0 - 0.0	7.1	2.2 - 17.0	0.70
3010106	4802	ALLENS CR	14.4	25.8	10 - 52	0.0	0.0 - 0.0	1.9	0.7 - 4.5	3.2	1.1 - 9.5	10.0	4.0 - 20.7	0.0	0.0 - 0.0	10.7	3.8 - 29.0	0.69
3010106	4803	COX CR	45.1	20.6	8 - 44	0.0	0.0 - 0.0	2.5	0.8 - 5.4	2.7	0.8 - 7.8	10.5	3.8 - 28.1	0.0	0.0 - 0.0	4.8	1.7 - 11.2	0.69
3010106	4804	ALLENS CR	29.8	24.2	7 - 48	0.0	0.0 - 0.0	1.5	0.5 - 3.1	2.8	0.8 - 7.4	10.4	3.0 - 27.5	0.0	0.0 - 0.0	9.5	3.1 - 23.1	0.69
3010106	4805	ALLENS CR	21.7	31.9	12 - 66	0.0	0.0 - 0.0	3.4	1.3 - 7.2	3.2	1.1 - 7.4	11.6	4.1 - 27.1	0.0	0.0 - 0.0	13.6	4.2 - 30.8	0.66
3010106	4806	LAYTON CR	57.6	32.6	10 - 81	0.0	0.0 - 0.0	1.5	0.5 - 3.9	3.2	0.9 - 11.7	12.4	4.1 - 37.9	0.0	0.0 - 0.0	15.5	4.7 - 45.6	0.63
3010106	4807	ALLENS CR	76.7	35.3	11 - 84	0.0	0.0 - 0.0	1.8	0.6 - 4.7	3.2	0.9 - 11.8	12.8	3.8 - 33.8	0.0	0.0 - 0.0	17.4	5.2 - 41.9	0.63
3010106	4808	*A	36.8	32.0	11 - 64	0.0	0.0 - 0.0	4.9	1.6 - 10.6	2.4	0.7 - 7.6	7.6	2.6 - 18.0	0.0	0.0 - 0.0	17.1	5.3 - 38.2	0.66
3010106	4809	ROANOKE R	18.2	23.7	6 - 44	0.0	0.0 - 0.0	3.8	1.0 - 8.0	2.6	0.6 - 7.0	8.8	2.4 - 17.9	0.0	0.0 - 0.0	8.5	2.6 - 21.1	0.69
3010106	4810	LITTLE DEEP CR	92.6	26.8	9 - 54	0.0	0.0 - 0.0	4.8	1.4 - 9.9	4.9	1.5 - 14.5	9.2	3.2 - 21.9	0.0	0.0 - 0.0	7.9	2.5 - 19.8	0.68
3010106	4811	BLUE MUD CR	40.0	33.7	10 - 68	0.0	0.0 - 0.0	9.8	3.2 - 20.2	5.8	1.7 - 16.2	11.1	3.4 - 26.8	0.0	0.0 - 0.0	7.1	2.2 - 16.4	0.68
3010107	4812	ROANOKE R	27.0	62.6	21 - 141	0.0	0.0 - 0.0	0.7	0.2 - 1.6	10.9	3.3 - 34.9	28.6	8.7 - 82.0	0.0	0.0 - 0.0	22.4	7.5 - 74.4	1.00
3010107	4813	CASHIE R	141.3	53.1	19 - 126	0.0	0.0 - 0.0	0.6	0.2 - 1.2	9.4	2.8 - 32.8	24.5	9.2 - 60.1	0.0	0.0 - 0.0	18.6	6.6 - 51.2	1.00
3010107	4814	CASHIE R	52.7	69.7	20 - 165	5.5	1.7 - 14.2	4.9	1.4 - 12.1	12.4	3.7 - 33.3	31.9	8.9 - 93.6	0.0	0.0 - 0.0	15.1	4.5 - 39.2	0.96
3010107	4815	HOGGARD MILL C	147.5	55.1	19 - 145	0.0	0.0 - 0.0	1.2	0.4 - 2.7	9.8	2.7 - 26.9	24.9	7.8 - 69.3	0.0	0.0 - 0.0	19.1	5.8 - 57.6	0.94
3010107	4816	CASHIE R	296.0	49.3	16 - 116	0.4	0.2 - 1.0	1.4	0.4 - 3.2	9.6	2.6 - 21.7	24.7	7.8 - 65.2	0.0	0.0 - 0.0	13.3	4.7 - 31.9	0.94
3010107	4817	ROQUIST CR	214.2	41.7	15 - 88	0.0	0.0 - 0.0	0.4	0.1 - 0.8	8.5	2.5 - 20.9	21.9	7.5 - 47.4	0.0	0.0 - 0.0	10.9	3.7 - 28.3	0.96
3010107	4818	ROANOKE R	470.4	148.0	54 - 327	102.4	36.9 - 263.3	3.8	1.4 - 8.2	4.4	1.2 - 12.6	20.8	7.3 - 54.8	0.0	0.0 - 0.0	16.6	6.1 - 40.0	1.00
3010107	4819	SWEETWATER CR	306.0	43.1	14 - 102	0.0	0.0 - 0.0	5.3	1.6 - 12.1	2.8	0.8 - 8.5	19.9	6.8 - 57.6	0.0	0.0 - 0.0	15.0	5.0 - 42.3	0.98
3010201	4820	NOTTOWAY R	381.9	38.3	12 - 65	0.0	0.0 - 0.0	1.2	0.4 - 2.3	3.2	0.8 - 7.2	22.7	7.3 - 48.1	0.0	0.0 - 0.0	11.1	3.3 - 19.8	0.98
3010201	4821	ASSAMOOSIAK SV	267.4	26.7	9 - 54	0.0	0.0 - 0.0	0.4	0.1 - 0.8	1.1	0.3 - 2.7	14.0	4.8 - 36.0	0.0	0.0 - 0.0	11.3	4.1 - 27.5	0.94
3010201	4822	NOTTOWAY R	57.5	18.4	7 - 40	0.0	0.0 - 0.0	0.7	0.3 - 1.3	1.5	0.5 - 4.3	11.1	4.2 - 31.9	0.0	0.0 - 0.0	5.1	1.8 - 11.8	0.94
3010201	4823	NOTTOWAY R	12.3	24.8	8 - 71	0.0	0.0 - 0.0	0.4	0.1 - 1.0	2.0	0.5 - 5.6	14.3	4.6 - 54.9	0.0	0.0 - 0.0	8.2	2.9 - 23.8	0.94
3010201	4824	NOTTOWAY R	42.2	16.7	6 - 39	0.0	0.0 - 0.0	0.2	0.1 - 0.5	0.6	0.2 - 1.7	8.8	2.7 - 23.9	0.0	0.0 - 0.0	7.0	2.4 - 22.8	0.94
3010201	4825	NOTTOWAY R	72.0	23.0	7 - 50	0.0	0.0 - 0.0	0.2	0.1 - 0.4	0.3	0.1 - 0.9	13.3	4.2 - 34.2	0.0	0.0 - 0.0	9.2	3.0 - 24.8	0.93

8-digit HUC	ID	Name	Area	Catchment Yield		Point sources		Developed Land		Manure		Agricultural Land		Phosphate Mines		Soil parent rock		Frac
				kg/km^2	90% CI	kg/km^2	90% CI	kg/km^2	90% CI	kg/km^2	90% CI	kg/km^2	90% CI	kg/km^2	90% CI	kg/km^2	90% CI	
3010201	4826	JOSEPH SWAMP	90.1	31.1	12 - 64	0.0	0.0 - 0.0	0.4	0.1 - 0.8	0.5	0.2 - 1.3	15.9	5.7 - 38.6	0.0	0.0 - 0.0	14.3	5.7 - 38.3	0.92
3010201	4827	NOTTOWAY R	27.1	17.9	7 - 38	0.0	0.0 - 0.0	0.4	0.1 - 0.7	0.2	0.1 - 0.6	8.9	2.8 - 21.6	0.0	0.0 - 0.0	8.5	3.1 - 22.2	0.92
3010201	4828	JONES HOLE SWAI	92.6	23.1	7 - 54	0.0	0.0 - 0.0	1.2	0.3 - 2.6	0.6	0.1 - 1.6	12.5	3.8 - 33.5	0.0	0.0 - 0.0	9.0	2.6 - 23.0	0.91
3010201	4829	NOTTOWAY R	48.4	16.1	5 - 36	0.0	0.0 - 0.0	0.6	0.2 - 1.5	0.3	0.1 - 0.7	9.6	3.1 - 24.9	0.0	0.0 - 0.0	5.7	1.8 - 14.6	0.91
3010201	4830	ROWANTY CR	329.9	15.8	5 - 34	0.0	0.0 - 0.0	4.0	1.1 - 9.5	0.5	0.1 - 1.3	6.6	2.0 - 16.1	0.0	0.0 - 0.0	4.6	1.5 - 10.3	0.90
3010201	4831	NOTTOWAY R	38.7	14.0	5 - 36	0.0	0.0 - 0.0	1.0	0.4 - 2.3	0.2	0.1 - 0.5	8.4	3.0 - 27.5	0.0	0.0 - 0.0	4.4	1.4 - 12.4	0.90
3010201	4832	STONY CR	14.2	31.2	11 - 67	0.0	0.0 - 0.0	5.5	1.7 - 12.5	0.5	0.1 - 1.5	21.2	6.7 - 48.5	0.0	0.0 - 0.0	4.0	1.4 - 10.0	0.89
3010201	4833	STONY CR	231.9	17.8	5 - 41	0.0	0.0 - 0.0	3.0	1.0 - 7.6	0.7	0.2 - 1.9	7.5	2.0 - 19.2	0.0	0.0 - 0.0	6.6	1.9 - 17.2	0.88
3010201	4834	WHITE OAK CR	82.2	19.2	7 - 53	0.0	0.0 - 0.0	3.2	1.1 - 7.8	0.6	0.2 - 1.6	5.8	2.2 - 17.2	0.0	0.0 - 0.0	9.5	3.5 - 29.4	0.76
3010201	4835	BUTTERWOOD CR	97.7	17.6	6 - 45	0.0	0.0 - 0.0	2.2	0.7 - 5.5	0.7	0.2 - 2.8	6.9	2.1 - 19.3	0.0	0.0 - 0.0	7.7	2.5 - 21.7	0.76
3010201	4836	SAPONY CR	174.5	18.8	6 - 47	0.0	0.0 - 0.0	2.3	0.7 - 5.3	0.8	0.2 - 2.0	8.8	2.7 - 26.5	0.0	0.0 - 0.0	6.9	2.3 - 17.8	0.88
3010201	4837	NOTTOWAY R	322.4	20.8	8 - 43	0.0	0.0 - 0.0	2.2	0.8 - 4.0	0.5	0.2 - 1.3	10.1	3.5 - 25.4	0.0	0.0 - 0.0	8.1	2.8 - 17.3	0.89
3010201	4838	NOTTOWAY R	14.2	27.4	8 - 60	0.0	0.0 - 0.0	1.9	0.5 - 3.5	1.0	0.3 - 2.9	8.8	2.6 - 23.1	0.0	0.0 - 0.0	15.7	4.2 - 42.9	0.83
3010201	4839	NOTTOWAY R	133.3	20.6	6 - 37	0.0	0.0 - 0.0	3.9	1.3 - 8.4	0.9	0.2 - 2.2	8.3	2.3 - 18.1	0.0	0.0 - 0.0	7.5	2.5 - 17.5	0.82
3010201	4840	TOMMEHETON CR	1.9	23.8	8 - 46	0.0	0.0 - 0.0	10.6	3.8 - 20.9	0.5	0.2 - 1.3	5.0	1.6 - 10.3	0.0	0.0 - 0.0	7.7	2.4 - 16.8	0.79
3010201	4841	TOMMEHETON CR	65.4	19.8	6 - 49	0.0	0.0 - 0.0	6.8	2.1 - 14.5	1.5	0.5 - 4.4	4.4	1.4 - 12.0	0.0	0.0 - 0.0	7.1	2.4 - 16.6	0.77
3010201	4842	BIRCHIN CR	40.4	20.0	8 - 39	0.0	0.0 - 0.0	9.8	3.8 - 19.2	1.4	0.5 - 3.6	1.6	0.5 - 3.9	0.0	0.0 - 0.0	7.2	2.8 - 16.1	0.77
3010201	4843	NOTTOWAY R	112.4	35.6	15 - 79	12.9	5.4 - 28.1	10.4	3.6 - 23.2	1.2	0.4 - 3.3	3.5	1.1 - 9.6	0.0	0.0 - 0.0	7.6	2.9 - 18.8	0.79
3010201	4844	NOTTOWAY R	70.5	22.3	8 - 50	0.0	0.0 - 0.0	4.1	1.4 - 9.1	2.5	0.7 - 7.9	8.3	3.0 - 22.1	0.0	0.0 - 0.0	7.2	2.7 - 19.7	0.76
3010201	4845	LITTLE NOTTOWA	195.7	23.9	8 - 64	0.0	0.0 - 0.0	3.9	1.3 - 10.4	4.8	1.5 - 16.6	8.2	2.7 - 23.2	0.0	0.0 - 0.0	7.0	2.6 - 21.2	0.74
3010201	4846	NOTTOWAY R	23.8	24.6	8 - 57	0.0	0.0 - 0.0	2.1	0.6 - 4.8	5.0	1.3 - 15.1	10.0	3.7 - 26.1	0.0	0.0 - 0.0	7.6	2.4 - 19.6	0.74
3010201	4847	NOTTOWAY R	204.1	15.0	5 - 35	0.0	0.0 - 0.0	1.9	0.7 - 4.6	2.0	0.6 - 6.6	4.6	1.5 - 12.4	0.0	0.0 - 0.0	6.6	2.3 - 15.8	0.71
3010201	4848	*A	61.7	20.8	7 - 52	0.0	0.0 - 0.0	5.0	1.6 - 12.4	1.8	0.5 - 4.6	6.5	2.1 - 20.2	0.0	0.0 - 0.0	7.4	2.4 - 21.9	0.71
3010201	4849	CROOKED CR	41.5	19.0	6 - 53	0.0	0.0 - 0.0	2.8	0.8 - 6.7	1.7	0.4 - 5.4	7.8	2.6 - 28.6	0.0	0.0 - 0.0	6.7	2.1 - 18.3	0.76
3010201	4850	WAQUA CR	121.6	17.7	6 - 32	0.0	0.0 - 0.0	3.5	1.0 - 6.4	0.8	0.2 - 2.2	6.3	2.0 - 16.2	0.0	0.0 - 0.0	7.1	2.4 - 16.7	0.82
3010201	4851	STUREGEON CR	137.1	20.6	9 - 38	0.0	0.0 - 0.0	4.4	1.8 - 10.1	1.0	0.3 - 3.5	7.4	2.7 - 16.3	0.0	0.0 - 0.0	7.8	3.1 - 18.9	0.83
3010201	4852	HUNTING QUARTE	90.0	13.0	4 - 28	0.0	0.0 - 0.0	0.4	0.1 - 0.9	0.2	0.1 - 0.4	7.4	2.4 - 18.8	0.0	0.0 - 0.0	5.1	1.8 - 12.5	0.93
3010201	4853	SPRING CR	173.9	14.1	5 - 32	0.0	0.0 - 0.0	0.5	0.2 - 1.1	0.2	0.1 - 0.6	7.5	2.5 - 22.3	0.0	0.0 - 0.0	5.8	2.0 - 16.1	0.94
3010201	4854	THREE CR	133.4	17.7	6 - 36	0.0	0.0 - 0.0	1.0	0.3 - 2.3	1.3	0.4 - 4.1	10.0	3.6 - 24.4	0.0	0.0 - 0.0	5.4	2.1 - 13.7	0.94
3010201	4855	POPLAR SWAMP	69.9	31.3	11 - 79	0.0	0.0 - 0.0	2.1	0.6 - 5.0	0.6	0.2 - 2.1	15.9	4.9 - 37.1	0.0	0.0 - 0.0	12.6	4.8 - 35.7	0.88
3010201	4856	THREE CR	188.8	36.0	15 - 70	3.4	1.2 - 7.0	1.8	0.7 - 3.5	1.3	0.4 - 3.5	17.6	6.5 - 38.8	0.0	0.0 - 0.0	11.9	4.2 - 28.5	0.88
3010201	4857	THREE CR	116.6	17.2	5 - 34	0.0	0.0 - 0.0	2.0	0.6 - 4.1	0.6	0.1 - 1.3	8.6	2.7 - 19.1	0.0	0.0 - 0.0	6.0	1.6 - 13.5	0.76
3010201	4858	MACLINS CR	53.7	34.8	12 - 79	0.0	0.0 - 0.0	4.1	1.5 - 9.8	0.5	0.1 - 1.4	19.2	5.9 - 50.5	0.0	0.0 - 0.0	11.0	3.8 - 23.9	0.76
3010202	4859	BLACKWATER R	109.0	390.4	121 - 899	335.7	103.8 - 795.8	4.4	1.4 - 9.3	3.8	1.1 - 11.3	32.3	9.4 - 84.1	0.0	0.0 - 0.0	14.3	4.7 - 38.0	0.98
3010202	4860	KINGSALE SWAMI	93.5	46.4	17 - 95	0.0	0.0 - 0.0	3.9	1.4 - 9.5	6.2	2.0 - 19.7	25.8	11.0 - 63.3	0.0	0.0 - 0.0	10.5	3.5 - 24.1	0.95
3010202	4861	BLACKWATER R	84.3	91.2	28 - 187	26.6	8.8 - 65.6	6.3	1.9 - 14.3	6.3	1.5 - 16.4	34.9	11.8 - 102.1	0.0	0.0 - 0.0	17.0	4.4 - 41.0	0.95
3010202	4862	BLACKWATER R	137.9	52.0	17 - 90	0.0	0.0 - 0.0	1.3	0.4 - 2.5	9.1	2.6 - 24.3	28.0	8.9 - 60.5	0.0	0.0 - 0.0	13.6	4.4 - 29.6	0.92
3010202	4863	MILL SWAMP	213.9	39.3	13 - 76	0.0	0.0 - 0.0	0.2	0.1 - 0.4	5.3	1.6 - 14.1	21.9	7.1 - 47.8	0.0	0.0 - 0.0	11.9	3.9 - 30.0	0.89
3010202	4864	BLACKWATER R	137.8	40.2	15 - 113	0.0	0.0 - 0.0	0.5	0.1 - 1.1	3.3	1.1 - 9.2	25.1	9.5 - 75.3	0.0	0.0 - 0.0	11.4	4.0 - 32.6	0.89

8-digit HUC	ID	Name	Area	Catchment Yield		Point sources		Developed Land		Manure		Agricultural Land		Phosphate Mines		Soil parent rock		Frac
				kg/km^2	90% CI	kg/km^2	90% CI	kg/km^2	90% CI	kg/km^2	90% CI	kg/km^2	90% CI	kg/km^2	90% CI	kg/km^2	90% CI	
3010202	4865	CYPRESS SWAMP	145.0	12.0	4 - 31	0.0	0.0 - 0.0	0.3	0.1 - 0.7	0.1	0.0 - 0.3	6.2	2.0 - 18.1	0.0	0.0 - 0.0	5.4	1.7 - 16.9	0.86
3010202	4866	BLACKWATER R	154.6	46.3	17 - 101	0.0	0.0 - 0.0	1.5	0.6 - 3.3	0.6	0.2 - 1.5	29.4	11.2 - 71.6	0.0	0.0 - 0.0	14.8	5.2 - 34.5	0.86
3010202	4867	OTTERDAM SWAM	59.2	18.3	5 - 39	0.0	0.0 - 0.0	0.1	0.0 - 0.2	0.2	0.0 - 0.5	8.8	2.3 - 20.6	0.0	0.0 - 0.0	9.1	2.6 - 24.5	0.81
3010202	4868	BLACKWATER R	61.3	42.1	15 - 105	0.0	0.0 - 0.0	1.4	0.5 - 3.6	0.5	0.2 - 1.8	23.4	8.3 - 67.4	0.0	0.0 - 0.0	16.8	5.6 - 48.4	0.81
3010202	4869	BLACKWATER R	214.5	31.1	12 - 66	0.0	0.0 - 0.0	5.1	1.9 - 11.8	0.7	0.2 - 1.7	15.5	5.7 - 39.2	0.0	0.0 - 0.0	9.9	3.6 - 27.6	0.77
3010202	4870	WARWICK SWAMI	99.3	25.1	8 - 57	2.8	1.0 - 7.1	0.9	0.3 - 2.0	0.6	0.2 - 1.7	12.4	4.5 - 31.1	0.0	0.0 - 0.0	8.4	2.6 - 20.9	0.77
3010202	4871	SEACOCK SWAMP	111.1	33.5	13 - 87	0.0	0.0 - 0.0	0.6	0.2 - 1.4	2.9	0.9 - 7.2	20.5	7.3 - 60.9	0.0	0.0 - 0.0	9.6	3.4 - 26.3	0.92
3010202	4872	SEACOCK SWAMP	101.3	31.6	11 - 76	0.0	0.0 - 0.0	2.4	0.9 - 5.5	1.3	0.4 - 3.5	17.1	5.9 - 43.1	0.0	0.0 - 0.0	10.7	3.8 - 29.4	0.86
3010202	4873	*A	57.3	24.1	7 - 56	0.0	0.0 - 0.0	0.4	0.1 - 0.8	1.7	0.5 - 4.5	14.7	4.6 - 36.2	0.0	0.0 - 0.0	7.3	2.3 - 17.9	0.86
3010203	4874	BENNETT CR	435.6	49.5	15 - 82	0.2	0.0 - 0.3	1.3	0.4 - 2.2	7.3	2.1 - 22.4	25.8	8.3 - 55.5	0.0	0.0 - 0.0	15.0	4.2 - 35.8	1.00
3010203	4875	CHOWAN R	16.9	20.0	7 - 52	0.0	0.0 - 0.0	0.1	0.0 - 0.2	2.8	0.8 - 8.4	8.0	2.5 - 23.2	0.0	0.0 - 0.0	9.1	3.3 - 25.9	1.00
3010203	4876	CHOWAN R	129.4	37.2	13 - 74	0.0	0.0 - 0.0	1.3	0.4 - 2.7	5.2	1.8 - 14.6	14.9	5.7 - 30.5	0.0	0.0 - 0.0	15.8	5.3 - 37.3	1.00
3010203	4877	CHOWAN R	60.0	21.1	6 - 50	0.0	0.0 - 0.0	0.0	0.0 - 0.0	2.2	0.7 - 6.0	6.7	1.9 - 19.7	0.0	0.0 - 0.0	12.2	3.5 - 34.8	0.99
3010203	4878	SOMERTON CR	120.1	40.2	14 - 83	0.0	0.0 - 0.0	0.1	0.1 - 0.3	3.7	1.1 - 9.0	21.5	8.1 - 54.8	0.0	0.0 - 0.0	14.9	5.6 - 38.1	0.98
3010203	4879	*A	30.9	35.7	12 - 64	0.0	0.0 - 0.0	0.6	0.2 - 1.3	4.5	1.4 - 9.9	19.9	6.5 - 41.3	0.0	0.0 - 0.0	10.6	3.7 - 23.8	0.91
3010203	4880	SOMERTON CR	193.7	41.9	17 - 68	0.0	0.0 - 0.0	1.1	0.4 - 2.0	1.9	0.7 - 4.4	26.9	10.4 - 55.4	0.0	0.0 - 0.0	12.0	4.8 - 25.2	0.91
3010203	4881	WICCACON R	807.0	59.1	20 - 116	0.0	0.0 - 0.0	2.0	0.6 - 4.4	11.3	3.2 - 29.2	29.4	10.2 - 67.7	0.0	0.0 - 0.0	16.4	5.6 - 40.2	1.00
3010203	4882	CHOWAN R	41.6	30.3	9 - 58	0.0	0.0 - 0.0	0.8	0.3 - 1.8	5.6	1.6 - 14.0	15.8	4.3 - 40.0	0.0	0.0 - 0.0	8.1	2.7 - 19.0	0.98
3010204	4883	MEHERIN R	1.0	32.0	8 - 73	0.0	0.0 - 0.0	0.0	0.0 - 0.0	4.6	1.2 - 14.5	12.9	3.3 - 40.3	0.0	0.0 - 0.0	14.6	3.4 - 35.5	0.99
3010204	4884	MEHERIN R	88.3	42.6	12 - 70	0.0	0.0 - 0.0	3.0	0.9 - 5.4	8.1	2.3 - 19.2	21.6	6.0 - 49.1	0.0	0.0 - 0.0	9.8	2.8 - 23.3	0.99
3010204	4885	MEHERIN R	495.0	37.6	14 - 69	1.0	0.3 - 2.2	0.5	0.1 - 1.0	5.0	1.4 - 14.1	21.3	8.0 - 43.9	0.0	0.0 - 0.0	9.7	3.6 - 20.0	0.97
3010204	4886	MEHERIN R	172.9	50.9	18 - 118	8.4	3.0 - 19.3	3.6	1.4 - 8.9	1.1	0.4 - 3.3	22.3	8.4 - 65.0	0.0	0.0 - 0.0	15.5	5.2 - 45.7	0.93
3010204	4887	GREAT CR	218.7	28.2	10 - 56	6.0	2.2 - 16.8	7.2	2.6 - 15.2	0.9	0.3 - 2.9	6.4	2.2 - 18.9	0.0	0.0 - 0.0	7.6	2.6 - 21.2	0.86
3010204	4888	MEHERIN R	142.7	17.8	7 - 36	0.0	0.0 - 0.0	4.0	1.4 - 8.4	0.7	0.2 - 1.9	5.0	1.9 - 10.4	0.0	0.0 - 0.0	8.0	3.1 - 18.8	0.86
3010204	4889	MEHERIN R	170.6	20.0	6 - 42	0.0	0.0 - 0.0	5.9	1.9 - 12.1	1.0	0.3 - 3.8	5.4	1.9 - 14.9	0.0	0.0 - 0.0	7.7	2.4 - 17.3	0.84
3010204	4890	STONY CR	60.2	20.0	7 - 42	0.0	0.0 - 0.0	3.8	1.3 - 7.5	2.0	0.6 - 5.9	7.1	2.4 - 15.7	0.0	0.0 - 0.0	7.1	2.2 - 16.3	0.80
3010204	4891	MEHERIN R	41.0	24.0	7 - 57	0.0	0.0 - 0.0	6.6	1.8 - 14.6	1.9	0.5 - 5.3	7.6	1.8 - 20.1	0.0	0.0 - 0.0	7.9	2.4 - 20.6	0.80
3010204	4892	FLAT ROCK CR	133.5	19.6	7 - 40	0.0	0.0 - 0.0	3.0	1.2 - 6.2	2.1	0.7 - 6.7	7.7	2.6 - 19.5	0.0	0.0 - 0.0	6.8	2.7 - 15.7	0.79
3010204	4893	MEHERIN R	104.7	23.3	8 - 47	0.0	0.0 - 0.0	3.8	1.1 - 7.9	2.5	0.8 - 7.8	10.2	3.1 - 25.6	0.0	0.0 - 0.0	6.8	2.1 - 16.8	0.79
3010204	4894	MASON CR	53.4	19.6	6 - 44	0.0	0.0 - 0.0	1.5	0.4 - 4.0	1.3	0.4 - 3.8	3.7	1.1 - 10.9	0.0	0.0 - 0.0	13.1	4.6 - 33.5	0.77
3010204	4895	MEHERIN R	37.3	26.9	9 - 52	0.0	0.0 - 0.0	2.5	0.8 - 5.9	2.6	0.8 - 7.0	10.7	3.1 - 23.3	0.0	0.0 - 0.0	11.2	3.6 - 23.6	0.77
3010204	4896	N MEHERIN R	21.2	20.3	6 - 47	0.0	0.0 - 0.0	1.0	0.3 - 2.0	1.5	0.4 - 4.0	4.2	1.4 - 10.2	0.0	0.0 - 0.0	13.6	4.0 - 36.6	0.75
3010204	4897	REEDY CR	54.2	23.3	8 - 44	0.0	0.0 - 0.0	2.8	0.9 - 5.8	2.0	0.5 - 5.3	6.9	2.4 - 16.2	0.0	0.0 - 0.0	11.6	3.7 - 27.1	0.73
3010204	4898	N MEHERIN R	50.1	23.7	7 - 57	0.0	0.0 - 0.0	1.6	0.5 - 3.9	1.9	0.5 - 6.3	6.9	2.5 - 17.6	0.0	0.0 - 0.0	13.3	4.4 - 35.9	0.73
3010204	4899	COUCHES CR	43.1	34.6	13 - 80	0.0	0.0 - 0.0	3.6	1.3 - 8.2	3.5	1.1 - 9.2	12.3	4.0 - 32.4	0.0	0.0 - 0.0	15.1	5.8 - 37.6	0.69
3010204	4900	N MEHERIN R	6.0	22.9	7 - 58	0.0	0.0 - 0.0	1.9	0.6 - 4.2	0.9	0.3 - 2.6	1.5	0.5 - 3.7	0.0	0.0 - 0.0	18.6	5.5 - 50.3	0.69
3010204	4901	N MEHERIN R	47.6	20.0	7 - 41	0.0	0.0 - 0.0	2.8	0.9 - 6.4	1.8	0.5 - 5.4	6.5	2.3 - 17.1	0.0	0.0 - 0.0	8.9	3.2 - 21.2	0.69
3010204	4902	*A	45.2	20.3	7 - 40	0.0	0.0 - 0.0	1.9	0.6 - 4.1	1.5	0.5 - 4.3	4.9	1.8 - 12.4	0.0	0.0 - 0.0	12.0	4.2 - 28.2	0.64
3010204	4903	*B	74.3	33.0	11 - 68	0.0	0.0 - 0.0	3.7	1.3 - 7.5	2.9	0.8 - 7.5	10.2	3.3 - 25.5	0.0	0.0 - 0.0	16.2	5.3 - 39.8	0.64

8-digit HUC	ID	Name	Area	Catchment Yield		Point sources		Developed Land		Manure		Agricultural Land		Phosphate Mines		Soil parent rock		Frac
				kg/km^2	90% CI	kg/km^2	90% CI	kg/km^2	90% CI	kg/km^2	90% CI	kg/km^2	90% CI	kg/km^2	90% CI	kg/km^2	90% CI	
3010204	4904	JUNIPER CR	56.6	28.5	11 - 60	0.0	0.0 - 0.0	3.5	1.3 - 8.0	2.8	0.9 - 7.5	10.4	4.0 - 26.4	0.0	0.0 - 0.0	11.8	4.4 - 27.2	0.69
3010204	4905	MEHERIN R	28.4	35.3	12 - 79	0.0	0.0 - 0.0	1.4	0.4 - 3.0	3.3	1.1 - 8.6	13.9	4.3 - 32.2	0.0	0.0 - 0.0	16.7	5.3 - 37.9	0.75
3010204	4906	M MEHERIN R	105.5	36.4	10 - 64	0.0	0.0 - 0.0	2.2	0.6 - 4.0	4.0	1.0 - 11.0	14.7	4.4 - 31.6	0.0	0.0 - 0.0	15.6	4.8 - 34.5	0.72
3010204	4907	S MEHERIN R	133.2	37.3	12 - 80	0.0	0.0 - 0.0	1.8	0.7 - 3.6	3.7	1.1 - 9.9	13.7	4.6 - 32.9	0.0	0.0 - 0.0	18.1	6.0 - 43.9	0.72
3010204	4908	GENITO CR	62.4	20.3	7 - 48	0.0	0.0 - 0.0	5.7	2.1 - 12.8	1.0	0.3 - 2.7	6.3	2.2 - 17.9	0.0	0.0 - 0.0	7.4	2.6 - 17.2	0.84
3010204	4909	FONTAINE CR	619.7	28.1	9 - 52	0.0	0.0 - 0.0	2.7	0.9 - 6.0	1.8	0.5 - 5.3	13.7	5.0 - 32.7	0.0	0.0 - 0.0	9.9	3.0 - 25.0	0.93
3010204	4910	KIRBYS CR	207.3	22.6	8 - 49	0.0	0.0 - 0.0	1.7	0.6 - 3.6	4.6	1.3 - 11.2	11.9	4.0 - 28.9	0.0	0.0 - 0.0	4.6	1.4 - 11.0	0.97
3010204	4911	POTECASI CR	105.7	49.1	16 - 107	0.0	0.0 - 0.0	1.0	0.4 - 2.3	9.4	2.8 - 28.6	25.1	7.5 - 60.1	0.0	0.0 - 0.0	13.6	4.8 - 32.5	0.99
3010204	4912	POTECASI CR	93.4	30.5	11 - 71	0.0	0.0 - 0.0	1.2	0.4 - 2.6	6.4	1.9 - 14.9	16.4	5.3 - 44.9	0.0	0.0 - 0.0	6.6	2.5 - 13.7	0.93
3010204	4913	POTECASI CR	185.3	27.9	10 - 64	0.0	0.0 - 0.0	2.5	0.9 - 6.0	5.5	1.7 - 14.7	14.1	4.5 - 39.6	0.0	0.0 - 0.0	5.9	2.4 - 17.0	0.87
3010204	4914	URAHAW SWAMP	150.1	70.2	24 - 137	0.0	0.0 - 0.0	1.3	0.4 - 2.4	14.0	3.6 - 40.6	36.5	13.3 - 78.6	0.0	0.0 - 0.0	18.3	5.3 - 41.0	0.87
3010204	4915	CUTAWHISKIE CR	110.5	67.3	22 - 134	0.0	0.0 - 0.0	1.3	0.3 - 2.7	12.9	3.7 - 33.1	34.1	10.8 - 77.3	0.0	0.0 - 0.0	18.9	5.5 - 48.7	0.93
3010205	4916	NORTHWEST R	516.6	1.3	0 - 3	1.2	0.5 - 2.6	0.0	0.0 - 0.0	0.0	0.0 - 0.0	0.1	0.0 - 0.6	0.0	0.0 - 0.0	0.0	0.0 - 0.3	1.00
3010205	4917	ALLIGATOR R, NW	167.5	0.0	0 - 0	0.0	0.0 - 0.0	0.0	0.0 - 0.0	0.0	0.0 - 0.0	0.0	0.0 - 0.2	0.0	0.0 - 0.0	0.0	0.0 - 0.1	1.00
3010205	4918	ALLIGATOR R, SW	116.9	0.0	0 - 0	0.0	0.0 - 0.0	0.0	0.0 - 0.0	0.0	0.0 - 0.0	0.0	0.0 - 0.1	0.0	0.0 - 0.0	0.0	0.0 - 0.1	1.00
3020101	4920	SWIFT CR	324.8	34.1	11 - 87	0.1	0.0 - 0.2	5.6	1.8 - 12.7	6.5	1.8 - 19.9	15.1	4.6 - 44.9	0.0	0.0 - 0.0	7.0	2.3 - 18.2	0.93
3020101	4921	SANDY CR	131.4	22.6	7 - 41	0.0	0.0 - 0.0	4.0	1.3 - 9.3	2.4	0.8 - 6.6	9.2	3.3 - 20.7	0.0	0.0 - 0.0	7.0	2.4 - 15.7	0.78
3020101	4922	SANDY CR	60.4	23.7	9 - 60	0.0	0.0 - 0.0	3.5	1.2 - 10.1	3.0	1.0 - 9.0	8.9	3.4 - 22.9	0.0	0.0 - 0.0	8.3	2.7 - 22.5	0.68
3020101	4923	WEAVER CR	25.3	27.5	11 - 54	0.0	0.0 - 0.0	6.5	2.5 - 13.5	0.8	0.3 - 2.7	12.7	4.7 - 27.6	0.0	0.0 - 0.0	7.5	3.3 - 16.2	0.56
3020101	4924	SANDY CR	60.4	38.0	13 - 82	0.0	0.0 - 0.0	20.6	6.7 - 45.3	0.6	0.2 - 1.7	9.2	3.6 - 25.1	0.0	0.0 - 0.0	7.6	2.9 - 19.4	0.56
3020101	4925	DEVILS CRADLE C	4.4	18.3	6 - 48	0.0	0.0 - 0.0	0.6	0.2 - 1.5	1.8	0.5 - 5.4	7.0	2.3 - 19.4	0.0	0.0 - 0.0	8.9	2.7 - 27.2	0.68
3020101	4926	RED BUD CR	48.6	19.8	9 - 48	0.0	0.0 - 0.0	3.4	1.3 - 8.8	2.0	0.8 - 5.6	7.7	3.0 - 22.5	0.0	0.0 - 0.0	6.7	2.8 - 16.1	0.78
3020101	4927	TAR R	21.8	58.3	20 - 106	0.0	0.0 - 0.0	4.7	1.5 - 8.6	12.1	3.3 - 26.7	29.0	10.4 - 67.8	0.0	0.0 - 0.0	12.5	4.0 - 27.1	0.93
3020101	4928	STONY CR	188.2	36.6	10 - 90	0.0	0.0 - 0.0	11.9	3.6 - 28.6	5.9	1.6 - 16.9	13.1	3.5 - 36.3	0.0	0.0 - 0.0	5.7	1.8 - 13.0	0.89
3020101	4929	STONY CR	81.3	30.9	10 - 72	0.0	0.0 - 0.0	4.1	1.3 - 8.7	4.7	1.3 - 11.9	13.6	3.8 - 33.6	0.0	0.0 - 0.0	8.4	2.7 - 18.6	0.77
3020101	4930	LITTLE PEACH CR	34.7	28.0	10 - 57	0.0	0.0 - 0.0	4.1	1.3 - 8.5	4.3	1.1 - 11.3	11.8	4.1 - 28.1	0.0	0.0 - 0.0	7.8	2.5 - 15.3	0.77
3020101	4931	TAR R	77.8	40.3	14 - 86	0.3	0.1 - 0.8	17.1	6.0 - 39.0	5.2	1.6 - 13.3	11.6	3.7 - 30.4	0.0	0.0 - 0.0	6.1	2.5 - 13.5	0.89
3020101	4932	SAPONY CR	170.7	36.1	11 - 63	0.0	0.0 - 0.0	8.2	2.4 - 17.1	6.9	2.1 - 19.0	15.2	4.6 - 34.8	0.0	0.0 - 0.0	5.9	1.9 - 13.0	0.79
3020101	4933	TAR R	193.3	33.4	11 - 76	2.3	0.8 - 5.1	6.4	2.0 - 13.0	5.5	1.5 - 17.3	12.8	4.5 - 32.2	0.0	0.0 - 0.0	6.6	2.2 - 15.6	0.79
3020101	4934	CYPRESS CR	80.4	28.9	9 - 59	0.1	0.0 - 0.1	8.2	2.5 - 18.1	2.6	0.7 - 8.6	10.9	3.3 - 27.3	0.0	0.0 - 0.0	7.2	2.1 - 18.2	0.74
3020101	4935	TAR R	4.4	21.0	6 - 45	0.0	0.0 - 0.0	5.9	1.9 - 12.5	1.8	0.5 - 4.3	7.4	1.9 - 19.3	0.0	0.0 - 0.0	6.0	1.7 - 14.6	0.74
3020101	4936	TAR R	40.7	27.8	9 - 53	0.0	0.0 - 0.0	5.5	1.8 - 10.4	2.8	0.8 - 7.6	11.6	3.8 - 26.0	0.0	0.0 - 0.0	7.9	2.8 - 18.3	0.74
3020101	4937	TAR R	76.5	33.6	14 - 61	1.4	0.5 - 2.9	6.2	2.5 - 13.0	3.4	1.1 - 8.4	14.0	5.4 - 33.0	0.0	0.0 - 0.0	8.6	3.3 - 18.9	0.72
3020101	4938	*A	27.2	29.9	11 - 56	0.0	0.0 - 0.0	10.1	3.3 - 19.9	2.6	0.8 - 6.2	9.8	3.1 - 20.9	0.0	0.0 - 0.0	7.4	2.6 - 17.2	0.70
3020101	4939	TAR R	42.9	28.5	9 - 57	0.0	0.0 - 0.0	8.0	2.5 - 15.6	2.5	0.7 - 6.2	9.4	3.4 - 22.0	0.0	0.0 - 0.0	8.5	2.7 - 21.2	0.70
3020101	4940	TAR R	1.7	15.7	5 - 28	0.0	0.0 - 0.0	0.6	0.2 - 1.0	1.7	0.5 - 4.5	6.0	2.1 - 14.6	0.0	0.0 - 0.0	7.4	2.6 - 15.6	0.69
3020101	4941	LYNCH CR	92.4	22.7	8 - 36	0.0	0.0 - 0.0	4.5	1.5 - 9.0	1.6	0.5 - 4.0	8.8	3.1 - 18.7	0.0	0.0 - 0.0	7.9	2.7 - 16.7	0.69
3020101	4942	TAR R	56.4	19.8	6 - 38	0.0	0.0 - 0.0	3.5	1.0 - 7.5	1.6	0.4 - 3.9	6.3	2.0 - 15.8	0.0	0.0 - 0.0	8.5	2.5 - 18.9	0.69
3020101	4943	BUFFALO CR	20.6	17.9	8 - 45	0.0	0.0 - 0.0	3.1	1.3 - 8.1	0.5	0.2 - 1.4	7.1	3.0 - 20.3	0.0	0.0 - 0.0	7.2	3.1 - 17.6	0.67

8-digit HUC	ID	Name	Area	Catchment Yield		Point sources		Developed Land		Manure		Agricultural Land		Phosphate Mines		Soil parent rock		Frac
				kg/km^2	90% CI	kg/km^2	90% CI	kg/km^2	90% CI	kg/km^2	90% CI	kg/km^2	90% CI	kg/km^2	90% CI	kg/km^2	90% CI	
3020101	4944	TAR R	9.1	22.7	7 - 46	0.0	0.0 - 0.0	5.3	1.7 - 12.4	1.5	0.4 - 3.7	7.4	2.5 - 17.1	0.0	0.0 - 0.0	8.5	2.8 - 20.6	0.67
3020101	4945	TABBS CR	46.3	22.6	8 - 59	2.4	0.9 - 5.7	6.9	2.3 - 16.0	0.4	0.1 - 1.3	4.8	1.6 - 14.0	0.0	0.0 - 0.0	8.0	3.4 - 20.3	0.67
3020101	4946	RUIN CR	78.7	28.4	10 - 53	0.0	0.0 - 0.0	14.6	4.7 - 27.3	0.4	0.1 - 1.0	6.3	2.2 - 13.1	0.0	0.0 - 0.0	7.1	2.3 - 15.4	0.63
3020101	4947	TABBS CR	61.7	21.4	6 - 42	0.0	0.0 - 0.0	6.0	1.9 - 12.5	0.9	0.2 - 2.3	7.7	2.3 - 16.9	0.0	0.0 - 0.0	6.8	2.4 - 15.2	0.63
3020101	4948	TAR R	39.0	19.5	6 - 37	0.0	0.0 - 0.0	5.9	1.9 - 12.0	1.0	0.3 - 2.5	3.9	1.3 - 10.0	0.0	0.0 - 0.0	8.7	2.6 - 19.3	0.67
3020101	4949	TAR R	48.5	20.0	6 - 35	0.0	0.0 - 0.0	3.3	1.0 - 6.8	1.0	0.3 - 2.6	6.7	2.1 - 17.8	0.0	0.0 - 0.0	9.0	3.0 - 21.5	0.65
3020101	4950	COON CR	16.0	25.2	9 - 54	0.0	0.0 - 0.0	3.8	1.3 - 10.1	1.4	0.4 - 4.1	10.8	3.7 - 26.4	0.0	0.0 - 0.0	9.2	3.3 - 21.7	0.63
3020101	4951	COON CR	70.0	29.3	10 - 71	0.0	0.0 - 0.0	10.8	3.7 - 24.9	1.4	0.4 - 3.7	9.6	3.3 - 27.5	0.0	0.0 - 0.0	7.5	2.8 - 17.6	0.55
3020101	4952	HATCHERS RUN	35.0	71.3	25 - 160	31.1	10.4 - 69.6	23.6	7.7 - 52.8	1.2	0.4 - 3.2	9.1	3.4 - 27.1	0.0	0.0 - 0.0	6.3	2.2 - 15.5	0.55
3020101	4953	TAR R	2.8	32.0	10 - 65	0.0	0.0 - 0.0	5.6	1.8 - 13.0	1.8	0.5 - 4.4	13.7	4.4 - 31.4	0.0	0.0 - 0.0	10.9	3.5 - 32.5	0.63
3020101	4954	TAR R	15.1	47.7	17 - 105	0.0	0.0 - 0.0	3.6	1.3 - 7.7	3.2	1.1 - 8.4	25.2	9.2 - 63.8	0.0	0.0 - 0.0	15.6	5.4 - 34.2	0.62
3020101	4955	TAR R	2.4	38.3	12 - 74	0.0	0.0 - 0.0	2.3	0.7 - 4.4	2.7	0.7 - 6.9	18.3	5.3 - 43.6	0.0	0.0 - 0.0	15.0	4.6 - 34.4	0.61
3020101	4956	BOULDING CR	15.4	27.9	10 - 74	0.0	0.0 - 0.0	6.7	2.3 - 15.5	1.7	0.6 - 5.3	11.7	4.0 - 31.5	0.0	0.0 - 0.0	7.8	2.7 - 21.1	0.60
3020101	4957	TAR R	47.6	31.6	12 - 67	0.0	0.0 - 0.0	6.4	2.2 - 14.8	1.9	0.6 - 5.4	12.7	5.1 - 30.1	0.0	0.0 - 0.0	10.5	3.9 - 23.2	0.60
3020101	4958	TAR R	26.0	33.5	10 - 68	0.0	0.0 - 0.0	3.7	1.0 - 7.3	2.7	0.7 - 6.8	19.4	5.6 - 47.7	0.0	0.0 - 0.0	7.7	2.1 - 16.0	0.56
3020101	4959	TAR R, N FK	57.9	26.9	10 - 61	0.0	0.0 - 0.0	3.9	1.3 - 8.4	1.8	0.5 - 6.7	13.1	4.4 - 30.5	0.0	0.0 - 0.0	8.1	3.1 - 21.5	0.53
3020101	4961	SHELTON CR	11.5	33.8	14 - 67	0.0	0.0 - 0.0	5.1	1.9 - 10.6	2.0	0.7 - 5.3	15.6	6.4 - 40.8	0.0	0.0 - 0.0	11.1	4.4 - 25.1	0.53
3020101	4962	FOX CR	25.3	20.2	8 - 50	0.0	0.0 - 0.0	2.1	0.7 - 5.2	1.2	0.3 - 3.3	7.4	2.5 - 21.1	0.0	0.0 - 0.0	9.5	3.5 - 23.4	0.45
3020101	4963	SHELTON CR	28.4	22.5	8 - 53	0.0	0.0 - 0.0	1.9	0.6 - 4.9	1.4	0.4 - 4.3	8.3	3.1 - 19.2	0.0	0.0 - 0.0	10.9	3.6 - 28.0	0.45
3020101	4964	TAR R	29.5	30.8	11 - 66	0.0	0.0 - 0.0	3.6	1.1 - 9.2	1.7	0.5 - 5.6	12.9	4.7 - 35.0	0.0	0.0 - 0.0	12.6	4.1 - 29.5	0.53
3020101	4965	TAR R	69.8	26.7	8 - 60	0.0	0.0 - 0.0	2.7	0.8 - 6.6	1.8	0.5 - 4.4	10.7	3.6 - 28.3	0.0	0.0 - 0.0	11.4	3.7 - 30.2	0.46
3020101	4966	CUB CR	45.7	26.0	10 - 50	0.0	0.0 - 0.0	2.8	0.9 - 5.4	1.6	0.5 - 3.8	9.8	3.5 - 22.7	0.0	0.0 - 0.0	11.8	3.7 - 26.2	0.46
3020101	4967	JACKSON CR	19.5	25.6	8 - 48	0.0	0.0 - 0.0	2.9	0.9 - 6.8	1.8	0.4 - 6.0	12.8	3.8 - 26.5	0.0	0.0 - 0.0	8.0	2.7 - 17.6	0.56
3020101	4968	JOHNSON CR	21.1	31.4	10 - 74	0.0	0.0 - 0.0	6.4	1.8 - 14.7	1.9	0.5 - 4.8	12.4	3.8 - 32.2	0.0	0.0 - 0.0	10.6	3.5 - 26.0	0.61
3020101	4969	*B	14.9	42.1	15 - 91	0.0	0.0 - 0.0	4.4	1.5 - 10.0	2.8	0.9 - 7.4	20.4	6.9 - 50.6	0.0	0.0 - 0.0	14.5	5.1 - 38.4	0.62
3020101	4970	MIDDLE CR	43.9	20.3	6 - 40	0.0	0.0 - 0.0	4.5	1.5 - 9.1	1.1	0.4 - 2.4	6.5	1.7 - 17.1	0.0	0.0 - 0.0	8.2	2.5 - 18.4	0.65
3020101	4971	BUFFALO CR	27.8	28.2	9 - 64	0.0	0.0 - 0.0	3.9	1.2 - 9.5	3.2	0.9 - 9.2	12.4	3.8 - 31.8	0.0	0.0 - 0.0	8.7	3.1 - 24.7	0.69
3020101	4972	CEDAR CR	168.3	28.9	9 - 63	0.8	0.3 - 1.7	6.8	1.9 - 15.4	2.6	0.7 - 6.8	10.4	3.0 - 28.3	0.0	0.0 - 0.0	8.3	2.7 - 19.3	0.72
3020101	4973	CROOKED CR	15.2	24.8	11 - 58	0.0	0.0 - 0.0	4.1	1.5 - 10.4	2.5	0.8 - 7.9	10.6	4.4 - 26.1	0.0	0.0 - 0.0	7.6	3.1 - 19.3	0.74
3020101	4974	CROOKED CR	83.7	30.6	9 - 53	4.3	1.4 - 8.3	5.6	1.5 - 10.7	2.6	0.8 - 6.0	10.4	3.3 - 22.2	0.0	0.0 - 0.0	7.7	2.3 - 16.1	0.69
3020101	4975	NORMS CR	38.0	26.9	7 - 64	0.0	0.0 - 0.0	7.2	2.0 - 21.6	2.6	0.6 - 8.6	10.8	3.1 - 27.9	0.0	0.0 - 0.0	6.3	2.0 - 16.7	0.69
3020101	4976	TAR R	52.4	40.1	13 - 83	0.0	0.0 - 0.0	8.3	2.8 - 16.7	7.1	2.3 - 24.7	17.1	5.4 - 43.1	0.0	0.0 - 0.0	7.6	2.5 - 19.4	0.94
3020102	4978	FISHING CR	113.3	40.9	14 - 112	0.0	0.0 - 0.0	4.2	1.7 - 11.3	7.6	2.5 - 28.5	19.4	6.5 - 58.0	0.0	0.0 - 0.0	9.7	3.4 - 29.7	0.94
3020102	4979	BEECH SWAMP	120.4	35.8	13 - 76	0.0	0.0 - 0.0	3.8	1.1 - 8.1	4.2	1.1 - 14.1	18.7	6.3 - 44.7	0.0	0.0 - 0.0	9.2	3.0 - 25.3	0.91
3020102	4980	MARSH SWAMP	244.7	23.2	8 - 54	0.0	0.0 - 0.0	4.3	1.4 - 10.8	2.5	0.8 - 7.7	11.2	3.7 - 33.2	0.0	0.0 - 0.0	5.2	2.0 - 14.1	0.84
3020102	4981	BEECH SWAMP	100.4	28.1	11 - 73	0.1	0.1 - 0.3	8.1	2.8 - 17.3	2.6	0.9 - 7.0	11.7	4.1 - 30.2	0.0	0.0 - 0.0	5.6	2.2 - 14.3	0.84
3020102	4982	FISHING CR	102.6	48.1	13 - 84	7.5	2.1 - 17.3	4.3	1.2 - 8.0	6.7	1.8 - 16.0	19.5	5.3 - 45.0	0.0	0.0 - 0.0	10.2	2.8 - 23.8	0.91
3020102	4983	LITTLE FISHING C	262.8	16.7	7 - 31	0.7	0.3 - 1.6	3.7	1.5 - 8.1	1.2	0.4 - 3.1	4.5	1.8 - 10.5	0.0	0.0 - 0.0	6.5	2.4 - 16.9	0.84
3020102	4984	LITTLE FISHING C	132.4	20.7	7 - 39	0.0	0.0 - 0.0	4.6	1.6 - 9.0	2.9	0.7 - 8.2	5.9	1.9 - 13.1	0.0	0.0 - 0.0	7.4	2.5 - 20.6	0.73

8-digit HUC	ID	Name	Area	Catchment Yield		Point sources		Developed Land		Manure		Agricultural Land		Phosphate Mines		Soil parent rock		Frac
				kg/km^2	90% CI	kg/km^2	90% CI	kg/km^2	90% CI	kg/km^2	90% CI	kg/km^2	90% CI	kg/km^2	90% CI	kg/km^2	90% CI	
3020102	4985	REEDY CR	91.3	20.6	8 - 40	0.0	0.0 - 0.0	3.8	1.3 - 8.1	3.0	1.1 - 8.1	5.4	2.1 - 13.2	0.0	0.0 - 0.0	8.4	3.3 - 19.7	0.73
3020102	4986	FISHING CR	102.9	15.6	6 - 34	0.0	0.0 - 0.0	3.6	1.3 - 8.3	1.8	0.6 - 4.9	4.7	1.4 - 11.8	0.0	0.0 - 0.0	5.5	1.9 - 14.3	0.84
3020102	4987	FISHING CR	350.0	21.7	8 - 57	1.2	0.5 - 3.5	5.3	2.0 - 14.0	2.5	0.8 - 7.4	5.1	1.8 - 16.3	0.0	0.0 - 0.0	7.6	2.4 - 23.6	0.78
3020102	4988	SHOCCO CR	217.4	18.5	7 - 36	0.0	0.0 - 0.0	2.9	0.8 - 6.1	2.5	0.8 - 6.7	5.7	2.0 - 13.6	0.0	0.0 - 0.0	7.6	2.8 - 17.1	0.78
3020102	4989	DEEP CR	233.8	38.1	11 - 79	3.9	1.1 - 9.0	4.9	1.6 - 12.0	4.5	1.4 - 12.5	17.3	5.2 - 42.5	0.0	0.0 - 0.0	7.4	2.3 - 18.5	0.94
3020103	4990	TRANTERS CR	623.0	53.4	18 - 105	3.9	1.3 - 7.2	5.5	1.8 - 11.6	5.4	1.6 - 13.7	25.8	8.0 - 59.7	0.0	0.0 - 0.0	12.8	4.1 - 29.2	1.00
3020103	4991	TAR R	42.7	82.7	25 - 215	0.0	0.0 - 0.0	4.5	1.1 - 11.0	12.2	3.3 - 42.6	47.3	16.1 - 128.9	0.0	0.0 - 0.0	18.6	5.3 - 57.7	1.00
3020103	4992	GRINDLE CR	258.8	71.3	24 - 145	0.0	0.0 - 0.0	4.8	1.6 - 10.7	13.8	4.3 - 33.9	34.6	11.1 - 77.4	0.0	0.0 - 0.0	18.1	6.2 - 37.1	0.99
3020103	4993	TAR R	187.2	118.1	47 - 204	41.2	15.2 - 78.6	5.5	1.8 - 10.7	15.2	4.0 - 39.3	40.4	15.0 - 89.0	0.0	0.0 - 0.0	15.8	5.9 - 32.1	0.99
3020103	4994	TAR R	160.9	65.4	21 - 141	0.8	0.2 - 1.8	24.3	6.9 - 55.2	8.4	2.3 - 19.5	20.5	6.3 - 50.8	0.0	0.0 - 0.0	11.4	3.3 - 28.2	0.98
3020103	4996	CONETOE CR	14.8	92.9	30 - 184	0.0	0.0 - 0.0	3.9	1.4 - 8.2	20.4	5.7 - 56.3	49.7	15.9 - 118.1	0.0	0.0 - 0.0	18.9	6.4 - 50.9	0.94
3020103	4997	CRISP CR	59.3	41.5	13 - 83	0.0	0.0 - 0.0	4.2	1.3 - 9.4	6.2	1.7 - 15.0	20.2	6.5 - 48.2	0.0	0.0 - 0.0	10.8	3.8 - 26.0	0.86
3020103	4998	CONETOE CR	131.5	38.5	12 - 98	0.0	0.0 - 0.0	3.6	1.1 - 9.8	7.7	2.4 - 21.9	19.0	5.8 - 50.6	0.0	0.0 - 0.0	8.3	2.7 - 23.3	0.86
3020103	4999	*A	2.3	65.9	23 - 122	0.0	0.0 - 0.0	4.1	1.4 - 7.6	13.6	3.9 - 38.8	33.1	11.4 - 72.7	0.0	0.0 - 0.0	15.1	5.1 - 38.0	0.94
3020103	5001	TAR R	68.3	74.4	24 - 120	40.1	13.4 - 72.4	14.6	4.2 - 25.4	4.4	1.2 - 9.1	10.7	3.6 - 21.6	0.0	0.0 - 0.0	4.6	1.4 - 10.5	0.96
3020103	5002	TOWN CR	512.6	31.8	12 - 65	1.3	0.5 - 3.0	6.8	2.4 - 13.3	4.2	1.4 - 9.9	13.6	5.2 - 38.5	0.0	0.0 - 0.0	5.9	2.3 - 13.4	0.96
3020103	5003	HARDEE CR	29.2	76.2	22 - 150	0.0	0.0 - 0.0	25.4	8.0 - 54.6	10.9	2.8 - 27.7	27.3	8.9 - 65.5	0.0	0.0 - 0.0	12.5	3.8 - 28.8	0.98
3020104	5004	PANTEGO CR	524.6	31.9	9 - 77	0.0	0.0 - 0.0	1.4	0.4 - 3.6	2.8	0.7 - 9.6	18.2	4.9 - 48.3	0.0	0.0 - 0.0	9.5	2.5 - 26.4	0.99
3020104	5005	BROAD CR	104.5	51.9	20 - 98	0.0	0.0 - 0.0	2.1	0.7 - 4.2	4.8	1.5 - 11.1	28.9	9.6 - 68.4	0.0	0.0 - 0.0	16.1	5.7 - 31.8	0.99
3020104	5006	INTRACOASTAL W	363.6	0.8	0 - 3	0.0	0.0 - 0.0	0.1	0.0 - 0.3	0.0	0.0 - 0.0	0.5	0.1 - 2.3	0.0	0.0 - 0.0	0.3	0.1 - 1.2	1.00
3020105	5007	OUTFALL CANAL	120.7	33.6	14 - 71	0.0	0.0 - 0.0	1.7	0.6 - 3.9	0.1	0.0 - 0.3	6.6	2.6 - 14.1	0.0	0.0 - 0.0	25.3	9.9 - 65.2	1.00
3020105	5008	INTRACOASTAL W	65.8	14.0	4 - 38	0.0	0.0 - 0.0	0.6	0.2 - 1.4	0.1	0.0 - 0.2	5.7	1.8 - 17.6	0.0	0.0 - 0.1	7.6	2.4 - 21.7	1.00
3020106	5009	HUNTERS CR	168.5	2.1	1 - 5	0.0	0.0 - 0.0	0.3	0.1 - 0.6	0.1	0.0 - 0.3	0.3	0.1 - 1.0	0.0	0.0 - 0.0	1.4	0.4 - 3.8	0.99
3020106	5010	WHITE OAK R	779.0	11.6	3 - 23	1.3	0.4 - 3.2	1.5	0.4 - 3.3	2.6	0.8 - 7.0	2.5	0.6 - 6.9	0.0	0.0 - 0.0	3.6	1.0 - 8.4	0.99
3020106	5011	QUEEN CR	79.2	19.8	7 - 42	0.2	0.1 - 0.5	5.3	1.7 - 12.3	5.2	1.4 - 13.5	5.5	1.8 - 14.0	0.0	0.0 - 0.0	3.5	1.4 - 9.1	1.00
3020201	5012	NEUSE R	24.8	101.6	33 - 232	0.0	0.0 - 0.0	56.5	19.4 - 120.7	16.9	5.4 - 38.5	12.0	4.1 - 33.9	0.0	0.0 - 0.0	16.2	5.6 - 38.4	0.93
3020201	5013	LITTLE R	349.0	60.5	17 - 99	6.2	1.7 - 11.4	9.5	2.5 - 16.6	16.8	5.0 - 35.5	20.4	5.1 - 45.2	0.0	0.0 - 0.0	7.6	2.2 - 15.1	0.93
3020201	5014	*A	69.1	31.2	11 - 83	0.0	0.0 - 0.0	4.6	1.5 - 12.3	6.7	1.9 - 20.7	14.6	5.0 - 39.0	0.0	0.0 - 0.0	5.3	1.8 - 14.2	0.87
3020201	5015	*A	241.1	20.5	7 - 49	0.1	0.0 - 0.3	5.2	2.0 - 11.9	1.5	0.4 - 4.2	8.7	3.1 - 24.5	0.0	0.0 - 0.0	5.1	1.7 - 14.8	0.65
3020201	5016	BUFFALO CR	28.3	23.4	7 - 61	0.0	0.0 - 0.0	3.1	0.9 - 8.3	4.6	1.4 - 16.8	10.4	3.0 - 28.1	0.0	0.0 - 0.0	5.4	1.8 - 15.4	0.65
3020201	5017	BUFFALO CR	152.4	35.1	13 - 71	0.1	0.0 - 0.2	7.3	2.7 - 16.4	5.0	1.4 - 14.5	15.0	5.3 - 34.0	0.0	0.0 - 0.0	7.8	2.9 - 17.1	0.87
3020201	5018	NEUSE R	90.6	62.2	22 - 114	0.0	0.0 - 0.0	6.6	2.2 - 11.7	25.3	7.9 - 61.1	19.4	7.1 - 41.5	0.0	0.0 - 0.0	10.9	3.7 - 25.9	0.93
3020201	5019	NEUSE R	6.1	50.3	17 - 103	0.0	0.0 - 0.0	1.3	0.5 - 2.8	16.5	5.3 - 44.7	15.9	5.2 - 41.5	0.0	0.0 - 0.0	16.6	5.4 - 44.5	0.92
3020201	5021	MOCCASIN CR	93.3	39.0	15 - 83	0.0	0.0 - 0.0	4.8	1.8 - 10.7	10.1	3.3 - 26.2	17.6	6.5 - 37.6	0.0	0.0 - 0.0	6.4	2.4 - 15.6	0.92
3020201	5022	NEUSE R	10.1	43.1	15 - 89	0.0	0.0 - 0.0	1.1	0.3 - 2.5	7.5	2.1 - 23.4	17.0	5.1 - 40.0	0.0	0.0 - 0.0	17.5	6.3 - 39.1	0.92
3020201	5023	BAWDY CR	65.0	32.9	12 - 74	0.0	0.0 - 0.0	5.3	1.8 - 12.3	6.8	2.2 - 20.5	14.5	5.0 - 39.3	0.0	0.0 - 0.0	6.3	2.2 - 15.6	0.92
3020201	5024	NEUSE R	132.9	40.5	13 - 97	0.0	0.0 - 0.0	2.9	0.8 - 6.3	7.4	2.0 - 22.0	15.8	5.2 - 39.1	0.0	0.0 - 0.0	14.4	4.6 - 34.2	0.92
3020201	5025	NEUSE R	12.0	121.4	34 - 303	85.6	23.1 - 222.2	9.5	2.7 - 23.6	6.7	1.9 - 16.9	15.0	4.5 - 41.8	0.0	0.0 - 0.0	4.6	1.6 - 13.6	0.89
3020201	5026	NEUSE R	9.3	50.6	18 - 92	0.0	0.0 - 0.0	37.9	13.4 - 70.1	2.3	0.7 - 5.0	4.4	1.6 - 9.8	0.0	0.0 - 0.0	6.0	2.5 - 12.5	0.89

8-digit HUC	ID	Name	Area	Catchment Yield		Point sources		Developed Land		Manure		Agricultural Land		Phosphate Mines		Soil parent rock		Frac
				kg/km^2	90% CI	kg/km^2	90% CI	kg/km^2	90% CI	kg/km^2	90% CI	kg/km^2	90% CI	kg/km^2	90% CI	kg/km^2	90% CI	
3020201	5027	NEUSE R	4.4	50.0	16 - 125	18.2	5.9 - 45.7	21.2	6.2 - 51.5	0.2	0.1 - 0.7	2.8	0.8 - 7.9	0.0	0.0 - 0.0	7.6	2.5 - 21.0	0.85
3020201	5028	NEUSE R	128.2	40.4	15 - 71	3.7	1.1 - 7.7	21.9	7.9 - 44.4	0.5	0.2 - 1.3	6.6	2.1 - 17.6	0.0	0.0 - 0.0	7.6	2.5 - 16.3	0.85
3020201	5029	NEUSE R	132.3	22.0	7 - 47	6.1	1.9 - 16.0	10.2	3.1 - 21.2	0.2	0.1 - 0.7	2.2	0.7 - 6.5	0.0	0.0 - 0.0	3.2	1.1 - 8.1	0.83
3020201	5031	NEUSE R	9.2	14.1	6 - 33	0.0	0.0 - 0.0	3.3	1.0 - 7.8	0.2	0.1 - 0.5	1.7	0.6 - 3.9	0.0	0.0 - 0.0	9.0	3.7 - 25.4	0.31
3020201	5032	NEW LIGHT CR	61.0	18.2	7 - 34	0.0	0.0 - 0.0	3.6	1.4 - 7.2	0.7	0.2 - 2.0	5.6	2.0 - 12.6	0.0	0.0 - 0.0	8.3	3.2 - 20.1	0.31
3020201	5033	NEUSE R	13.9	13.3	5 - 29	0.0	0.0 - 0.0	2.7	0.9 - 5.9	0.1	0.0 - 0.3	1.5	0.6 - 3.5	0.0	0.0 - 0.0	9.0	3.3 - 22.9	0.31
3020201	5034	BEAVERDAM CR	29.8	24.5	8 - 52	0.0	0.0 - 0.0	3.7	1.2 - 7.7	0.7	0.3 - 1.9	5.4	1.9 - 13.1	0.0	0.0 - 0.0	14.7	5.3 - 38.5	0.30
3020201	5035	SMITH CR	28.4	21.9	8 - 41	0.0	0.0 - 0.0	3.0	1.0 - 5.4	1.1	0.4 - 3.0	5.5	2.0 - 12.8	0.0	0.0 - 0.0	12.3	4.3 - 29.1	0.29
3020201	5037	BEAVERDAM CR	36.6	46.8	15 - 72	0.0	0.0 - 0.0	3.7	1.1 - 6.3	2.9	0.8 - 6.0	21.6	6.7 - 42.1	0.0	0.0 - 0.0	18.6	5.6 - 36.0	0.28
3020201	5038	ROBERTSON CR	42.6	42.7	16 - 100	0.0	0.0 - 0.0	8.1	2.9 - 17.6	2.9	0.8 - 7.5	18.1	6.0 - 48.6	0.0	0.0 - 0.0	13.6	4.8 - 38.3	0.28
3020201	5039	NEUSE R	2.7	19.9	7 - 37	0.0	0.0 - 0.0	2.7	0.9 - 5.3	0.2	0.1 - 0.6	2.3	0.8 - 5.3	0.0	0.0 - 0.0	14.6	4.9 - 32.2	0.30
3020201	5040	NEUSE R	5.7	30.4	9 - 73	0.0	0.0 - 0.0	0.9	0.3 - 2.0	0.4	0.1 - 1.2	2.7	0.9 - 6.3	0.0	0.0 - 0.0	26.3	7.9 - 70.2	0.30
3020201	5041	LODGE CR	8.3	32.7	11 - 77	0.0	0.0 - 0.0	1.7	0.5 - 4.4	1.1	0.3 - 4.4	13.5	4.4 - 33.1	0.0	0.0 - 0.0	16.5	4.8 - 46.1	0.30
3020201	5042	LODGE CR	20.8	40.4	13 - 77	0.1	0.0 - 0.2	12.5	4.0 - 24.9	1.8	0.6 - 4.4	12.5	4.0 - 25.5	0.0	0.0 - 0.0	13.5	4.4 - 30.8	0.29
3020201	5043	*B	25.8	37.9	12 - 76	0.0	0.0 - 0.0	7.3	2.2 - 16.6	2.3	0.6 - 8.1	15.9	5.3 - 46.0	0.0	0.0 - 0.0	12.4	4.0 - 26.3	0.27
3020201	5044	LODGE CR	19.6	36.6	11 - 66	0.0	0.0 - 0.0	10.3	3.0 - 21.2	1.8	0.5 - 5.2	12.5	3.5 - 30.0	0.0	0.0 - 0.0	12.0	3.6 - 33.0	0.27
3020201	5045	*C	12.4	49.2	18 - 116	0.0	0.0 - 0.0	9.4	3.4 - 22.4	3.1	0.9 - 10.0	23.9	8.9 - 65.5	0.0	0.0 - 0.0	12.9	4.4 - 34.0	0.29
3020201	5046	NEUSE R	5.2	22.3	8 - 56	0.0	0.0 - 0.0	0.5	0.2 - 0.9	0.4	0.1 - 0.9	2.9	0.9 - 7.0	0.0	0.0 - 0.0	18.6	6.2 - 49.5	0.30
3020201	5047	NEUSE R	12.4	39.2	12 - 75	0.0	0.0 - 0.0	2.7	0.7 - 5.9	2.2	0.5 - 5.5	17.2	5.0 - 38.1	0.0	0.0 - 0.0	17.1	4.7 - 44.7	0.30
3020201	5048	*D	29.5	35.5	13 - 80	0.0	0.0 - 0.0	14.2	5.0 - 30.4	1.6	0.5 - 4.0	8.7	3.1 - 21.5	0.0	0.0 - 0.0	11.1	4.4 - 30.0	0.30
3020201	5050	NEUSE R	72.1	142.1	50 - 376	58.9	21.1 - 153.0	57.7	19.0 - 152.8	1.3	0.4 - 4.7	5.8	2.0 - 14.9	0.0	0.0 - 0.0	18.4	6.7 - 44.4	0.29
3020201	5051	NEUSE R	4.3	27.1	10 - 54	0.0	0.0 - 0.0	2.6	0.9 - 5.1	1.7	0.5 - 4.2	5.4	1.8 - 12.4	0.0	0.0 - 0.0	17.4	6.4 - 42.1	0.29
3020201	5052	KNAP OF REEDS C	122.3	25.0	8 - 53	12.2	4.3 - 29.0	3.2	1.1 - 7.4	0.6	0.2 - 1.7	3.6	1.3 - 8.3	0.0	0.0 - 0.0	5.4	1.6 - 13.4	0.29
3020201	5053	NEUSE R	4.8	41.8	12 - 96	0.0	0.0 - 0.0	0.9	0.3 - 2.3	3.2	0.8 - 9.8	20.0	5.7 - 49.8	0.0	0.0 - 0.0	17.6	5.0 - 45.4	0.29
3020201	5055	DEEP CR	100.6	28.8	11 - 68	0.0	0.0 - 0.0	3.2	1.3 - 7.6	1.9	0.6 - 5.7	11.8	4.7 - 32.5	0.0	0.0 - 0.0	12.0	4.8 - 30.2	0.20
3020201	5056	N FLAT R	103.7	36.3	13 - 72	0.0	0.0 - 0.0	8.1	2.6 - 16.7	2.3	0.8 - 6.1	14.6	5.2 - 37.5	0.0	0.0 - 0.0	11.2	4.0 - 26.4	0.19
3020201	5057	S FLAT R	41.8	38.1	14 - 93	0.0	0.0 - 0.0	6.1	2.1 - 14.7	2.9	0.9 - 7.4	16.8	6.5 - 49.9	0.0	0.0 - 0.0	12.4	4.2 - 28.6	0.19
3020201	5058	*E	24.0	31.2	8 - 87	0.0	0.0 - 0.0	3.5	1.0 - 9.4	2.4	0.6 - 8.4	14.9	4.4 - 37.7	0.0	0.0 - 0.0	10.3	2.9 - 33.0	0.17
3020201	5059	S FLAT R	85.4	33.0	12 - 59	0.0	0.0 - 0.0	4.4	1.4 - 8.3	2.5	0.8 - 6.6	16.0	5.3 - 38.2	0.0	0.0 - 0.0	10.0	3.3 - 24.8	0.17
3020201	5060	NEUSE R	10.3	31.6	11 - 65	0.0	0.0 - 0.0	4.0	1.4 - 7.7	2.2	0.6 - 5.8	12.9	4.3 - 32.8	0.0	0.0 - 0.0	12.4	3.7 - 29.0	0.29
3020201	5061	LITTLE R	18.1	30.1	10 - 56	0.0	0.0 - 0.0	12.4	4.2 - 25.4	1.2	0.3 - 3.5	6.4	2.0 - 15.7	0.0	0.0 - 0.0	10.1	3.3 - 23.3	0.28
3020201	5062	MOUNTAIN CR	2.1	40.8	15 - 86	0.0	0.0 - 0.0	3.1	1.0 - 6.8	2.9	0.9 - 7.8	21.6	7.6 - 51.1	0.0	0.0 - 0.0	13.2	4.6 - 31.2	0.26
3020201	5063	LITTLE R	28.3	36.3	13 - 71	0.0	0.0 - 0.0	10.6	3.4 - 22.2	1.6	0.4 - 4.2	10.0	3.5 - 23.3	0.0	0.0 - 0.0	14.1	4.5 - 33.2	0.26
3020201	5064	N FK LITTLE R	85.8	30.5	8 - 55	0.0	0.0 - 0.0	4.7	1.1 - 10.6	2.7	0.7 - 6.3	12.7	3.8 - 28.5	0.0	0.0 - 0.0	10.5	2.8 - 24.2	0.24
3020201	5065	S FK LITTLE R	20.6	31.9	9 - 72	0.0	0.0 - 0.0	4.9	1.4 - 12.7	2.6	0.8 - 7.5	11.0	3.4 - 34.2	0.0	0.0 - 0.0	13.4	4.5 - 35.1	0.24
3020201	5066	S FK LITTLE R	58.9	35.3	11 - 84	0.0	0.0 - 0.0	4.7	1.5 - 11.3	3.5	0.9 - 10.9	16.1	4.9 - 44.1	0.0	0.0 - 0.0	11.0	3.4 - 23.9	0.21
3020201	5067	*F	21.5	32.6	13 - 70	0.0	0.0 - 0.0	3.4	1.2 - 8.1	3.1	1.1 - 7.4	13.7	4.7 - 37.1	0.0	0.0 - 0.0	12.4	4.6 - 27.6	0.21
3020201	5068	ENO R	3.9	30.5	9 - 58	0.0	0.0 - 0.0	11.4	3.4 - 23.4	1.8	0.5 - 5.0	8.2	2.6 - 19.4	0.0	0.0 - 0.0	9.1	2.5 - 19.6	0.28
3020201	5069	ENO R	51.5	36.5	12 - 94	0.1	0.0 - 0.2	6.1	2.2 - 13.8	3.3	1.1 - 8.9	14.5	4.2 - 44.1	0.0	0.0 - 0.0	12.7	4.8 - 34.1	0.24

8-digit HUC	ID	Name	Area	Catchment Yield		Point sources		Developed Land		Manure		Agricultural Land		Phosphate Mines		Soil parent rock		Frac
				kg/km^2	90% CI	kg/km^2	90% CI	kg/km^2	90% CI	kg/km^2	90% CI	kg/km^2	90% CI	kg/km^2	90% CI	kg/km^2	90% CI	
3020201	5070	*G	30.0	39.2	14 - 68	0.0	0.0 - 0.0	6.2	2.1 - 11.4	3.9	1.3 - 9.3	18.0	5.6 - 38.4	0.0	0.0 - 0.0	11.1	3.8 - 25.3	0.21
3020201	5071	*H	29.9	34.6	11 - 61	0.0	0.0 - 0.0	4.9	1.5 - 8.5	3.4	0.8 - 8.3	16.0	5.4 - 32.7	0.0	0.0 - 0.0	10.3	3.1 - 23.4	0.21
3020201	5072	SEVENMILE CR	43.8	31.1	11 - 60	0.0	0.0 - 0.0	8.0	2.8 - 16.7	2.0	0.7 - 4.6	8.4	2.8 - 21.4	0.0	0.0 - 0.0	12.7	4.2 - 27.4	0.24
3020201	5073	PANTHER CR	17.2	39.6	13 - 78	0.5	0.2 - 1.1	10.9	3.4 - 23.1	2.3	0.7 - 6.3	12.1	4.3 - 25.7	0.0	0.0 - 0.0	13.9	4.6 - 33.1	0.29
3020201	5074	CHUNKY PIPE CR	58.3	51.8	15 - 93	0.0	0.0 - 0.0	26.4	7.5 - 53.8	1.9	0.5 - 4.7	9.2	3.0 - 21.0	0.0	0.0 - 0.0	14.3	4.0 - 31.2	0.30
3020201	5075	LICK CR	57.7	28.2	11 - 58	0.0	0.0 - 0.0	6.7	2.7 - 14.0	1.4	0.4 - 3.8	5.7	2.1 - 13.3	0.0	0.0 - 0.0	14.4	5.0 - 34.1	0.30
3020201	5076	UPPER BARTON Cl	34.5	28.3	9 - 77	3.4	1.2 - 9.8	13.9	4.5 - 36.6	0.2	0.1 - 0.7	2.7	0.8 - 8.7	0.0	0.0 - 0.0	8.1	2.8 - 22.6	0.31
3020201	5077	LOWER BARTON (	34.0	39.9	12 - 83	2.2	0.7 - 4.7	26.5	7.7 - 59.1	0.2	0.1 - 0.6	2.9	0.9 - 7.4	0.0	0.0 - 0.0	8.1	2.7 - 19.2	0.31
3020201	5078	PERRY CR	29.6	72.1	25 - 166	0.0	0.0 - 0.0	62.4	20.7 - 151.1	0.1	0.0 - 0.4	1.4	0.5 - 3.7	0.0	0.0 - 0.0	8.2	3.0 - 24.1	0.83
3020201	5079	CRABTREE CR	157.5	86.4	31 - 185	0.0	0.0 - 0.0	74.6	25.8 - 162.2	0.1	0.0 - 0.2	0.9	0.3 - 2.4	0.0	0.0 - 0.0	10.8	4.1 - 24.3	0.85
3020201	5080	WALNUT CR	116.7	67.0	19 - 137	0.0	0.0 - 0.0	55.0	15.5 - 114.5	0.2	0.0 - 0.6	2.2	0.6 - 5.3	0.0	0.0 - 0.0	9.6	2.5 - 22.8	0.85
3020201	5082	SWIFT CR	410.2	15.9	6 - 32	2.0	0.7 - 4.3	8.2	2.8 - 18.3	0.8	0.3 - 2.0	2.5	0.9 - 5.7	0.0	0.0 - 0.0	2.4	0.8 - 5.4	0.89
3020201	5083	MIDDLE CR	317.5	37.7	14 - 68	10.4	4.0 - 20.4	11.3	4.0 - 23.4	2.3	0.8 - 6.3	8.3	2.7 - 19.6	0.0	0.0 - 0.0	5.5	2.0 - 11.7	0.89
3020201	5084	BLACK CR	266.5	16.1	5 - 40	0.0	0.0 - 0.0	4.2	1.3 - 10.3	2.5	0.7 - 9.0	6.8	2.2 - 17.0	0.0	0.0 - 0.0	2.5	0.8 - 7.3	0.89
3020201	5085	MILL CR	64.2	46.8	15 - 107	0.0	0.0 - 0.0	2.0	0.6 - 4.6	14.7	3.9 - 38.1	16.5	5.2 - 43.4	0.0	0.0 - 0.0	13.5	4.5 - 35.1	0.92
3020201	5086	HANNAH CR	169.7	29.1	10 - 63	4.7	1.6 - 11.5	6.3	1.9 - 14.4	4.4	1.4 - 12.5	9.7	3.4 - 23.2	0.0	0.0 - 0.0	4.1	1.3 - 10.5	0.74
3020201	5087	MILL CR	26.4	39.2	12 - 79	0.0	0.0 - 0.0	2.0	0.6 - 4.5	7.9	2.2 - 17.6	16.5	5.3 - 37.4	0.0	0.0 - 0.0	12.9	4.0 - 32.8	0.74
3020201	5088	STONE CR	72.8	33.8	10 - 62	0.0	0.0 - 0.0	5.1	1.5 - 10.1	6.4	1.7 - 16.3	13.9	4.3 - 32.1	0.0	0.0 - 0.0	8.3	2.3 - 18.6	0.64
3020201	5089	MILL CR	85.1	40.4	15 - 94	0.0	0.0 - 0.0	3.5	1.2 - 9.3	11.3	3.7 - 38.9	15.6	5.4 - 38.9	0.0	0.0 - 0.0	10.1	3.4 - 27.6	0.64
3020201	5090	*I	18.8	50.2	16 - 102	0.0	0.0 - 0.0	5.1	1.3 - 10.5	10.6	2.6 - 26.8	23.6	7.5 - 59.1	0.0	0.0 - 0.0	10.8	3.7 - 25.9	0.31
3020201	5091	*J	27.2	51.0	17 - 129	0.0	0.0 - 0.0	4.7	1.5 - 12.2	16.3	4.5 - 46.9	19.3	7.2 - 52.2	0.0	0.0 - 0.0	10.7	3.7 - 28.1	0.31
3020201	5092	*K	27.7	47.6	16 - 89	0.0	0.0 - 0.0	2.8	0.9 - 5.9	21.1	5.7 - 51.6	14.8	5.4 - 33.6	0.0	0.0 - 0.0	8.9	2.6 - 18.5	0.92
3020201	5093	BEAVERDAM CR	110.4	40.6	13 - 88	0.0	0.0 - 0.0	3.5	1.2 - 8.0	17.7	5.4 - 49.5	12.0	3.8 - 30.0	0.0	0.0 - 0.0	7.4	2.3 - 17.8	0.73
3020201	5094	*K	35.8	39.6	15 - 106	0.0	0.0 - 0.0	7.9	2.8 - 22.9	14.5	4.8 - 43.9	10.2	4.1 - 28.7	0.0	0.0 - 0.0	7.0	2.6 - 20.5	0.73
3020201	5095	THOROUGHFARE !	86.3	65.3	19 - 137	0.0	0.0 - 0.0	5.4	1.6 - 12.7	29.6	8.3 - 75.5	19.9	6.0 - 52.2	0.0	0.0 - 0.0	10.4	3.7 - 24.4	0.61
3020201	5096	BUCK SWAMP	47.1	53.2	18 - 114	0.0	0.0 - 0.0	8.0	2.8 - 18.0	21.7	6.3 - 59.8	15.4	4.9 - 35.6	0.0	0.0 - 0.0	8.1	2.6 - 17.5	0.61
3020202	5097	NEUSE R	6.8	31.4	10 - 67	0.0	0.0 - 0.0	4.1	1.3 - 10.4	3.6	0.9 - 10.7	9.3	3.1 - 27.9	0.0	0.0 - 0.0	14.5	4.3 - 37.0	1.00
3020202	5098	NEUSE R	9.5	17.8	6 - 40	0.0	0.0 - 0.0	1.6	0.5 - 3.4	0.5	0.1 - 1.9	1.4	0.4 - 3.1	0.0	0.0 - 0.0	14.3	4.6 - 34.1	1.00
3020202	5099	SWIFT CR	1.2	26.8	9 - 68	0.0	0.0 - 0.0	5.6	1.9 - 13.7	0.1	0.0 - 0.2	0.0	0.0 - 0.0	0.0	0.0 - 0.0	21.2	6.7 - 57.0	1.00
3020202	5100	LITTLE SWIFT CR	361.9	25.8	8 - 47	0.0	0.0 - 0.0	2.7	0.8 - 5.3	2.2	0.6 - 5.3	8.1	2.6 - 17.2	0.0	0.0 - 0.0	12.8	3.9 - 25.8	0.99
3020202	5101	SWIFT CR	204.5	53.2	17 - 116	0.9	0.3 - 2.2	5.3	1.7 - 12.9	9.1	2.4 - 27.1	26.5	8.8 - 64.8	0.0	0.0 - 0.0	11.3	3.6 - 27.4	0.99
3020202	5102	CLAYROOT SWAM	219.8	62.6	24 - 139	0.0	0.0 - 0.0	4.2	1.3 - 9.6	10.8	3.4 - 31.8	28.7	12.0 - 70.5	0.0	0.0 - 0.0	19.0	6.5 - 42.7	0.91
3020202	5103	SWIFT CR	89.6	61.7	19 - 119	0.0	0.0 - 0.0	2.8	0.9 - 5.8	12.5	3.8 - 32.4	32.6	10.0 - 89.5	0.0	0.0 - 0.0	13.7	4.0 - 35.4	0.91
3020202	5104	FORK SWAMP	73.7	84.0	30 - 158	0.0	0.0 - 0.0	18.8	6.3 - 34.6	14.3	4.1 - 30.9	35.9	12.4 - 84.5	0.0	0.0 - 0.0	15.1	5.1 - 35.8	0.83
3020202	5105	SWIFT CR	105.0	72.3	24 - 149	0.0	0.0 - 0.0	11.1	3.6 - 26.8	13.2	4.2 - 36.3	32.9	12.2 - 79.4	0.0	0.0 - 0.0	15.1	4.8 - 44.1	0.83
3020202	5106	NEUSE R	102.6	170.2	63 - 417	125.8	47.1 - 325.5	3.4	1.3 - 9.0	7.7	2.7 - 22.9	21.4	8.5 - 63.8	0.0	0.0 - 0.0	11.9	4.3 - 30.1	1.00
3020202	5107	NEUSE R	13.7	57.6	19 - 126	0.0	0.0 - 0.0	3.9	1.3 - 8.6	11.2	3.8 - 31.4	32.5	10.2 - 78.7	0.0	0.0 - 0.0	9.9	3.6 - 21.6	0.99
3020202	5108	NEUSE R	288.3	58.0	23 - 120	5.5	2.2 - 11.0	5.5	2.0 - 11.8	15.9	5.3 - 40.3	19.1	7.4 - 45.3	0.0	0.0 - 0.0	11.9	4.2 - 28.3	0.98
3020202	5109	NEUSE R	38.8	129.6	43 - 235	34.4	11.8 - 69.2	43.1	15.1 - 76.7	13.5	3.8 - 37.4	14.0	4.6 - 31.9	0.0	0.0 - 0.0	24.5	8.6 - 50.1	0.97

8-digit HUC	ID	Name	Area	Catchment Yield		Point sources		Developed Land		Manure		Agricultural Land		Phosphate Mines		Soil parent rock		Frac
				kg/km^2	90% CI	kg/km^2	90% CI	kg/km^2	90% CI	kg/km^2	90% CI	kg/km^2	90% CI	kg/km^2	90% CI	kg/km^2	90% CI	
3020202	5110	FALLING CR	171.9	67.6	23 - 122	1.6	0.5 - 3.2	9.3	3.2 - 18.5	21.9	6.5 - 52.8	22.3	7.4 - 58.4	0.0	0.0 - 0.0	12.5	3.8 - 29.3	0.96
3020202	5111	NEUSE R	75.2	101.7	30 - 245	0.2	0.1 - 0.4	5.3	1.8 - 11.9	33.7	9.6 - 96.8	35.3	11.0 - 95.4	0.0	0.0 - 0.0	27.1	8.5 - 74.0	0.96
3020202	5112	BEAR CR	27.0	117.2	39 - 242	0.0	0.0 - 0.0	5.3	1.9 - 10.9	49.3	15.4 - 126.4	45.0	14.0 - 107.8	0.0	0.0 - 0.0	17.6	6.2 - 42.5	0.96
3020202	5113	NEUSE R	322.5	70.5	22 - 130	0.3	0.1 - 0.8	13.9	4.7 - 30.1	24.3	7.8 - 73.2	18.4	6.3 - 43.6	0.0	0.0 - 0.0	13.6	5.2 - 32.4	0.96
3020202	5114	SOUTHWEST CR	166.7	59.1	21 - 106	0.1	0.0 - 0.1	5.7	1.9 - 11.7	19.1	5.6 - 49.8	19.5	7.0 - 44.7	0.0	0.0 - 0.0	14.7	5.8 - 33.7	0.97
3020202	5115	CORE CR	201.1	12.9	4 - 35	0.0	0.0 - 0.0	1.0	0.3 - 2.9	2.7	0.7 - 8.4	6.6	1.8 - 19.6	0.0	0.0 - 0.0	2.5	0.7 - 7.1	0.99
3020202	5116	BACHELOR CR	172.8	29.4	9 - 61	0.0	0.0 - 0.0	3.7	1.1 - 8.8	5.7	1.5 - 15.8	11.1	3.5 - 25.8	0.0	0.0 - 0.0	8.9	2.6 - 22.6	1.00
3020203	5117	CONTENTNEA CR	71.2	97.3	33 - 195	26.2	8.9 - 56.8	6.5	2.2 - 14.8	18.7	5.1 - 46.8	26.1	8.4 - 67.9	0.0	0.0 - 0.0	19.8	6.3 - 50.0	0.98
3020203	5118	LITTLE CONTENTI	76.2	84.8	28 - 189	0.0	0.0 - 0.0	4.8	1.7 - 12.4	28.9	8.9 - 94.4	32.2	10.1 - 79.6	0.0	0.0 - 0.0	18.9	6.3 - 51.9	0.97
3020203	5119	CONTENTNEA CR	133.4	102.7	33 - 245	0.1	0.0 - 0.3	4.2	1.5 - 10.1	46.3	12.7 - 128.8	33.3	11.3 - 93.1	0.0	0.0 - 0.0	18.8	6.5 - 40.2	0.97
3020203	5120	CONTENTNEA CR	91.4	88.5	31 - 190	3.6	1.3 - 8.2	3.9	1.2 - 8.3	44.1	13.7 - 116.5	26.8	9.6 - 64.4	0.0	0.0 - 0.0	10.0	3.4 - 28.3	0.93
3020203	5121	TOISNOT CR	316.6	34.9	13 - 63	0.1	0.0 - 0.1	9.3	2.9 - 20.9	3.9	1.3 - 11.1	15.5	6.0 - 39.6	0.0	0.0 - 0.0	6.0	1.7 - 14.5	0.91
3020203	5122	CONTENTNEA CR	589.4	44.0	19 - 105	4.9	2.1 - 11.5	10.9	4.2 - 25.3	6.6	2.5 - 18.4	15.6	6.8 - 40.5	0.0	0.0 - 0.0	6.1	2.4 - 15.0	0.91
3020203	5123	TURKEY CR	194.7	28.8	11 - 67	0.0	0.0 - 0.0	7.7	2.7 - 19.2	4.4	1.5 - 13.0	10.9	3.4 - 29.6	0.0	0.0 - 0.0	5.8	2.2 - 13.7	0.80
3020203	5124	MOCCASIN CR	163.6	28.3	9 - 68	2.4	0.7 - 6.0	8.9	2.5 - 19.8	2.5	0.7 - 6.7	9.1	2.8 - 22.1	0.0	0.0 - 0.0	5.3	1.7 - 14.2	0.80
3020203	5125	NAHUNTA SWAMI	53.2	78.3	25 - 153	0.0	0.0 - 0.0	4.5	1.3 - 9.2	40.3	12.7 - 95.7	24.4	7.7 - 55.8	0.0	0.0 - 0.0	9.1	3.2 - 21.2	0.93
3020204	5126	TRENT R	400.7	52.2	18 - 142	0.6	0.2 - 1.4	5.4	1.7 - 14.6	19.7	6.0 - 60.8	15.0	5.2 - 40.6	0.0	0.0 - 0.0	11.4	4.1 - 35.1	1.00
3020204	5127	BEAVER CR	142.3	53.8	16 - 111	0.0	0.0 - 0.0	2.8	0.8 - 5.7	23.1	6.9 - 65.3	18.5	5.7 - 59.9	0.0	0.0 - 0.0	9.4	2.8 - 20.2	0.95
3020204	5128	TRENT R	86.5	92.2	32 - 198	0.0	0.0 - 0.0	3.5	1.2 - 7.5	44.5	14.6 - 122.3	32.3	12.5 - 74.6	0.0	0.0 - 0.0	12.0	3.9 - 27.8	0.95
3020204	5129	TRENT R, TRENT F	113.9	54.0	15 - 126	0.0	0.0 - 0.0	4.3	1.2 - 10.8	19.0	5.4 - 48.4	18.3	6.0 - 50.0	0.0	0.0 - 0.0	12.4	3.4 - 33.9	0.90
3020204	5130	TRENT R	202.6	45.2	14 - 113	0.0	0.0 - 0.0	3.2	0.9 - 9.7	22.9	6.5 - 74.3	10.0	3.3 - 28.2	0.0	0.0 - 0.0	9.1	2.5 - 23.5	0.90
3020204	5131	BRICE CR	116.4	7.5	3 - 14	0.0	0.0 - 0.0	2.0	0.6 - 4.0	0.5	0.1 - 1.3	1.3	0.4 - 3.5	0.0	0.0 - 0.0	3.7	1.1 - 8.0	1.00
3020204	5132	INTRACOASTAL W	85.4	22.3	7 - 44	0.0	0.0 - 0.0	1.8	0.5 - 3.4	0.4	0.1 - 1.1	9.0	2.3 - 23.3	0.0	0.0 - 0.0	11.1	3.3 - 24.9	0.00
3030002	5133	HAW R	63.8	20.6	7 - 45	2.0	0.7 - 4.9	2.1	0.7 - 5.2	5.1	1.4 - 15.9	4.2	1.4 - 11.9	0.0	0.0 - 0.0	7.1	2.2 - 16.1	0.88
3030002	5134	NEW HOPE R	12.4	20.6	6 - 45	0.0	0.0 - 0.0	1.1	0.3 - 2.3	3.2	0.8 - 8.4	1.7	0.5 - 3.7	0.0	0.0 - 0.0	14.7	4.5 - 35.6	0.40
3030002	5135	BEAVER CR	101.8	40.3	14 - 85	0.0	0.0 - 0.0	14.6	4.9 - 30.5	3.9	1.3 - 10.7	10.1	3.1 - 27.0	0.0	0.0 - 0.0	11.7	3.9 - 30.5	0.40
3030002	5136	NEW HOPE R	174.1	34.7	13 - 65	1.2	0.4 - 2.1	9.4	3.5 - 17.0	4.5	1.5 - 14.7	6.3	2.5 - 13.5	0.0	0.0 - 0.0	13.4	4.8 - 33.5	0.40
3030002	5137	NEW HOPE R	22.0	44.1	14 - 89	0.0	0.0 - 0.0	2.4	0.8 - 5.4	13.1	3.7 - 42.5	13.0	4.4 - 31.5	0.0	0.0 - 0.0	15.6	4.9 - 37.8	0.39
3030002	5138	NORTHEAST CR	65.8	36.4	14 - 88	0.0	0.0 - 0.0	10.0	3.8 - 23.2	3.2	1.0 - 9.2	8.6	3.3 - 18.7	0.0	0.0 - 0.0	14.6	5.1 - 38.9	0.38
3030002	5139	NEW HOPE CR	98.9	56.7	22 - 149	0.0	0.0 - 0.0	36.5	13.3 - 100.1	1.6	0.5 - 4.9	3.9	1.4 - 11.4	0.0	0.0 - 0.0	14.7	6.3 - 43.5	0.38
3030002	5140	MORGAN CR	42.4	38.7	12 - 82	4.1	1.3 - 9.1	12.2	3.9 - 25.2	4.8	1.3 - 14.7	5.5	1.7 - 14.5	0.0	0.0 - 0.0	12.1	3.6 - 30.7	0.39
3030002	5141	HAW R	171.8	23.8	7 - 50	4.1	1.3 - 9.4	6.0	1.6 - 13.3	3.3	1.1 - 8.7	3.0	0.8 - 7.7	0.0	0.0 - 0.0	7.5	2.4 - 17.4	0.40
3030002	5142	HAW R	77.3	31.8	10 - 77	0.6	0.2 - 1.3	4.0	1.5 - 10.3	4.0	1.1 - 12.1	11.6	4.1 - 30.1	0.0	0.0 - 0.0	11.6	3.6 - 27.8	0.39
3030002	5143	HAW R	8.0	39.2	13 - 80	0.0	0.0 - 0.0	5.2	1.8 - 10.5	3.7	1.0 - 8.8	15.9	5.1 - 37.7	0.0	0.0 - 0.0	14.3	4.2 - 39.9	0.39
3030002	5144	LANE CR	85.9	37.1	13 - 73	0.0	0.0 - 0.0	4.4	1.5 - 7.7	3.7	1.1 - 9.6	15.6	5.2 - 34.8	0.0	0.0 - 0.0	13.3	4.5 - 31.8	0.38
3030002	5145	HAW R	132.9	31.4	11 - 60	0.1	0.1 - 0.3	5.7	2.0 - 12.1	3.4	1.0 - 9.3	12.7	4.7 - 30.3	0.0	0.0 - 0.0	9.6	3.7 - 21.7	0.38
3030002	5146	HAW CR	74.3	41.2	16 - 86	0.0	0.0 - 0.0	11.9	4.2 - 25.6	3.5	1.2 - 9.7	13.5	4.9 - 35.7	0.0	0.0 - 0.1	12.3	4.8 - 31.7	0.38
3030002	5147	HAW R	5.1	45.1	14 - 112	0.0	0.0 - 0.0	12.8	3.8 - 27.8	4.1	1.2 - 10.7	14.5	4.4 - 40.5	0.0	0.0 - 0.0	13.7	4.6 - 34.7	0.38
3030002	5148	HAW R	7.6	69.6	23 - 167	0.0	0.0 - 0.0	44.2	14.4 - 108.6	2.6	0.8 - 7.0	10.0	3.4 - 30.2	0.0	0.0 - 0.0	12.8	4.3 - 33.0	0.37

8-digit HUC	ID	Name	Area	Catchment Yield		Point sources		Developed Land		Manure		Agricultural Land		Phosphate Mines		Soil parent rock		Frac
				kg/km^2	90% CI	kg/km^2	90% CI	kg/km^2	90% CI	kg/km^2	90% CI	kg/km^2	90% CI	kg/km^2	90% CI	kg/km^2	90% CI	
3030002	5149	BACK QUAKER CR	211.0	43.9	15 - 78	5.8	1.9 - 11.7	11.5	3.7 - 21.9	3.3	1.0 - 9.3	13.5	4.1 - 32.3	0.0	0.0 - 0.0	9.7	3.3 - 22.1	0.37
3030002	5150	HAW R	20.2	239.6	88 - 552	169.7	61.5 - 403.9	55.4	17.4 - 130.8	1.1	0.3 - 3.5	3.5	1.1 - 8.8	0.0	0.0 - 0.0	9.9	3.5 - 26.8	0.37
3030002	5151	JORDAN CR	31.9	33.1	11 - 71	0.0	0.0 - 0.0	7.0	2.3 - 15.6	3.5	1.0 - 11.0	13.5	4.1 - 32.1	0.0	0.0 - 0.0	9.2	3.3 - 22.2	0.36
3030002	5152	JORDAN CR	67.4	30.9	11 - 57	0.0	0.0 - 0.0	4.7	1.8 - 9.2	3.4	1.1 - 8.7	14.4	4.7 - 36.4	0.0	0.0 - 0.0	8.4	3.0 - 20.8	0.35
3030002	5153	STONY CR	170.7	10.0	3 - 22	0.0	0.0 - 0.0	1.3	0.4 - 3.2	1.0	0.3 - 2.6	4.6	1.5 - 12.5	0.0	0.0 - 0.0	3.2	1.2 - 7.0	0.35
3030002	5154	HAW R	86.0	46.6	19 - 92	0.8	0.3 - 1.7	17.4	7.5 - 36.2	3.6	1.3 - 10.7	15.5	6.6 - 45.4	0.0	0.0 - 0.0	9.4	3.9 - 24.4	0.36
3030002	5155	HAW R	129.9	64.8	23 - 127	22.5	7.7 - 48.7	12.7	4.5 - 26.7	2.4	0.7 - 7.4	15.7	5.5 - 41.4	0.0	0.0 - 0.0	11.4	3.9 - 28.0	0.35
3030002	5156	TROUBLESOME CI	144.0	33.4	10 - 76	0.1	0.0 - 0.2	6.9	2.1 - 15.5	1.7	0.5 - 5.1	14.4	4.1 - 31.5	0.0	0.0 - 0.0	10.3	3.4 - 26.2	0.32
3030002	5157	HAW R	85.4	34.6	11 - 73	0.0	0.0 - 0.1	5.5	1.7 - 12.3	2.3	0.7 - 6.3	16.0	5.0 - 39.1	0.0	0.0 - 0.0	10.7	3.2 - 21.9	0.32
3030002	5158	HAW R	95.3	31.7	9 - 59	0.8	0.3 - 1.5	6.5	1.8 - 13.6	2.1	0.6 - 7.0	12.7	4.2 - 28.6	0.0	0.0 - 0.0	9.7	3.1 - 25.1	0.28
3030002	5159	MEARS CR	32.7	28.3	9 - 66	0.0	0.0 - 0.0	5.3	1.6 - 11.8	2.1	0.6 - 6.1	11.9	3.8 - 33.0	0.0	0.0 - 0.0	9.0	2.8 - 20.7	0.28
3030002	5160	HAW R, REEDY FK	60.1	43.7	18 - 97	0.2	0.1 - 0.3	5.6	2.0 - 12.0	4.0	1.2 - 9.9	22.5	8.4 - 57.8	0.0	0.0 - 0.0	11.5	4.3 - 26.6	0.35
3030002	5161	HAW R, REEDY FK	66.3	39.9	17 - 94	2.3	1.0 - 5.2	9.8	3.6 - 24.5	2.4	0.9 - 6.8	13.7	5.6 - 38.2	0.0	0.0 - 0.0	11.7	4.8 - 30.9	0.34
3030002	5162	HAW R, REEDY FK	1.8	14.7	4 - 37	0.0	0.0 - 0.0	0.5	0.2 - 1.1	0.8	0.2 - 2.6	3.8	1.2 - 10.1	0.0	0.0 - 0.0	9.6	2.7 - 27.4	0.03
3030002	5163	HAW R, REEDY FK	94.2	35.1	10 - 76	0.0	0.0 - 0.0	11.7	3.5 - 26.2	2.0	0.6 - 5.8	11.8	3.5 - 26.0	0.0	0.0 - 0.0	9.7	3.0 - 22.8	0.03
3030002	5164	BRUSH CR	32.1	48.5	15 - 107	0.0	0.0 - 0.0	34.6	11.5 - 79.9	0.8	0.2 - 2.0	4.4	1.2 - 10.9	0.0	0.0 - 0.0	8.7	2.5 - 21.0	0.03
3030002	5165	BUFFALO CR	30.8	44.8	17 - 83	2.8	0.9 - 6.3	7.5	2.4 - 14.8	3.6	1.1 - 8.9	20.5	6.8 - 52.8	0.0	0.0 - 0.0	10.4	3.8 - 23.8	0.34
3030002	5166	BUFFALO CR	17.2	39.3	15 - 100	1.4	0.6 - 3.4	11.1	4.2 - 29.4	2.4	0.8 - 7.3	13.6	5.4 - 40.1	0.0	0.0 - 0.0	10.9	3.7 - 30.0	0.32
3030002	5167	*A	4.7	38.9	14 - 83	0.0	0.0 - 0.0	10.2	3.4 - 23.3	2.7	0.8 - 7.1	15.6	5.0 - 42.7	0.0	0.0 - 0.0	10.4	3.5 - 23.4	0.32
3030002	5168	BIG ALAMANCE C	4.5	2562.8	856 - 4903	2516.6	839.0 - 4894.7	12.3	4.0 - 24.5	4.4	1.5 - 10.6	16.7	5.2 - 34.2	0.0	0.0 - 0.0	12.7	4.3 - 28.2	0.37
3030002	5169	GUM CR	42.5	76.0	28 - 180	0.0	0.0 - 0.0	64.4	24.6 - 165.3	0.6	0.2 - 1.9	2.2	0.7 - 6.0	0.0	0.0 - 0.0	8.8	3.9 - 23.4	0.37
3030002	5170	BIG ALAMANCE C	6.6	44.9	14 - 97	0.0	0.0 - 0.0	9.6	2.9 - 18.9	4.8	1.4 - 13.9	17.4	5.8 - 47.2	0.0	0.0 - 0.0	13.1	4.7 - 31.2	0.37
3030002	5171	BIG ALAMANCE C	39.2	59.8	20 - 119	0.0	0.0 - 0.0	40.1	12.4 - 88.7	2.2	0.6 - 5.7	7.4	2.4 - 17.8	0.0	0.0 - 0.0	10.0	3.4 - 21.2	0.37
3030002	5172	*B	36.0	47.7	15 - 106	0.0	0.0 - 0.0	26.1	8.2 - 58.9	2.2	0.6 - 7.4	10.1	3.5 - 24.0	0.0	0.0 - 0.0	9.2	2.6 - 22.9	0.35
3030002	5173	BIG ALAMANCE C	43.8	36.1	11 - 86	0.0	0.0 - 0.0	4.7	1.5 - 10.9	3.4	1.0 - 9.0	17.3	5.5 - 48.5	0.0	0.0 - 0.0	10.7	3.1 - 29.9	0.35
3030002	5174	L ALAMANCE CR	159.5	40.5	12 - 83	0.4	0.1 - 0.8	15.8	4.9 - 32.0	2.2	0.7 - 6.5	12.8	4.0 - 32.2	0.0	0.0 - 0.0	9.3	3.4 - 20.9	0.34
3030002	5175	BIG ALAMANCE C	45.6	32.3	14 - 79	0.0	0.0 - 0.0	5.1	1.9 - 12.0	2.5	0.8 - 9.1	14.9	5.4 - 43.3	0.0	0.0 - 0.0	9.8	3.8 - 26.2	0.34
3030002	5176	BIG ALAMANCE C	52.4	35.9	11 - 78	0.0	0.0 - 0.0	9.2	2.6 - 19.9	2.5	0.7 - 8.0	14.5	4.3 - 36.0	0.0	0.0 - 0.0	9.7	3.0 - 23.1	0.30
3030002	5177	*C	33.4	32.2	12 - 57	0.0	0.0 - 0.0	6.0	2.0 - 12.1	2.7	1.0 - 6.5	15.0	5.7 - 35.3	0.0	0.0 - 0.0	8.5	3.3 - 18.2	0.30
3030002	5178	STINKING QUARTI	56.4	36.8	14 - 76	0.0	0.0 - 0.0	6.1	2.2 - 15.3	4.1	1.2 - 11.1	15.9	5.9 - 41.5	0.0	0.0 - 0.0	10.7	4.0 - 26.9	0.37
3030002	5179	STINKING QUARTI	66.5	36.0	12 - 73	0.1	0.0 - 0.2	5.8	1.8 - 12.1	3.3	1.0 - 11.2	17.3	5.7 - 47.5	0.0	0.0 - 0.0	9.5	2.7 - 26.1	0.35
3030002	5180	*D	93.3	34.2	11 - 91	0.0	0.0 - 0.0	4.7	1.3 - 12.8	5.1	1.4 - 16.0	15.7	5.0 - 43.7	0.0	0.0 - 0.0	8.7	2.7 - 26.1	0.35
3030002	5181	CANE CR	183.1	42.1	14 - 77	0.1	0.0 - 0.2	4.5	1.2 - 8.6	7.0	1.9 - 18.5	19.5	6.3 - 41.3	0.0	0.0 - 0.0	11.0	3.2 - 24.7	0.39
3030002	5182	TERRELLS CR	74.0	50.4	18 - 120	0.0	0.0 - 0.0	2.9	1.0 - 6.5	17.2	5.3 - 55.0	17.1	5.8 - 46.4	0.0	0.0 - 0.0	13.1	4.6 - 40.3	0.39
3030002	5183	HORSEPEN CR	51.7	69.9	22 - 146	0.0	0.0 - 0.0	53.9	16.0 - 120.4	0.6	0.1 - 1.7	3.2	1.0 - 7.3	0.0	0.0 - 0.0	12.2	3.7 - 27.9	0.03
3030003	5184	DEEP R	28.4	37.9	13 - 83	0.0	0.0 - 0.1	8.2	2.9 - 20.4	6.5	2.1 - 19.0	10.8	3.6 - 35.4	0.0	0.0 - 0.0	12.4	4.3 - 31.5	0.88
3030003	5185	ROCKY R	23.2	18.0	7 - 43	0.0	0.0 - 0.0	4.8	1.6 - 13.0	2.9	0.8 - 9.0	2.5	1.0 - 8.6	0.0	0.0 - 0.0	7.9	3.0 - 20.8	0.87
3030003	5186	ROCKY R	149.8	18.5	6 - 35	0.0	0.0 - 0.0	2.7	0.9 - 5.4	4.8	1.3 - 10.7	4.4	1.5 - 9.6	0.0	0.0 - 0.0	6.6	2.6 - 14.1	0.85
3030003	5187	ROCKY R	257.9	65.6	25 - 129	15.8	6.4 - 33.8	9.3	3.3 - 18.2	14.6	4.9 - 40.9	15.4	6.0 - 35.6	0.0	0.0 - 0.0	10.5	4.0 - 21.8	0.79

8-digit HUC	ID	Name	Area	Catchment Yield		Point sources		Developed Land		Manure		Agricultural Land		Phosphate Mines		Soil parent rock		Frac
				kg/km^2	90% CI	kg/km^2	90% CI	kg/km^2	90% CI	kg/km^2	90% CI	kg/km^2	90% CI	kg/km^2	90% CI	kg/km^2	90% CI	
3030003	5188	TICK CR	57.6	31.8	10 - 73	0.0	0.0 - 0.0	4.3	1.4 - 9.4	10.0	3.0 - 26.8	9.8	3.3 - 28.3	0.0	0.0 - 0.0	7.7	2.2 - 19.7	0.79
3030003	5189	BEAR CR	132.1	20.9	8 - 58	0.2	0.1 - 0.6	3.9	1.4 - 10.7	6.1	2.2 - 20.6	5.9	2.1 - 20.1	0.0	0.0 - 0.0	4.9	1.8 - 16.3	0.85
3030003	5190	DEEP R	548.0	80.7	28 - 184	52.6	18.2 - 125.0	8.2	3.1 - 18.2	5.6	1.5 - 14.9	6.4	1.9 - 17.3	0.0	0.0 - 0.0	7.9	2.8 - 19.2	0.87
3030003	5191	DEEP R	236.1	41.6	16 - 90	0.6	0.3 - 1.4	3.7	1.3 - 8.7	13.2	4.2 - 35.2	11.5	4.0 - 26.9	0.0	0.0 - 0.0	12.6	5.0 - 27.7	0.84
3030003	5192	DEEP R	43.6	59.2	20 - 144	0.0	0.0 - 0.0	4.1	1.4 - 10.9	22.6	6.4 - 58.6	17.8	6.0 - 46.8	0.0	0.0 - 0.0	14.7	4.2 - 45.2	0.80
3030003	5193	DEEP R	77.3	44.1	18 - 88	0.1	0.1 - 0.3	3.3	1.1 - 7.4	14.3	4.2 - 37.9	14.2	6.2 - 35.1	0.0	0.0 - 0.0	12.3	4.7 - 28.8	0.80
3030003	5194	BRUSH CR	183.9	43.1	15 - 97	0.0	0.0 - 0.1	4.3	1.5 - 10.7	14.0	4.1 - 42.6	14.0	4.3 - 37.5	0.0	0.0 - 0.0	10.7	3.3 - 30.3	0.78
3030003	5195	DEEP R	2.8	44.4	18 - 100	0.0	0.0 - 0.0	3.0	1.1 - 6.9	13.3	4.7 - 35.5	13.8	5.4 - 40.0	0.0	0.0 - 0.0	14.3	5.6 - 34.3	0.78
3030003	5196	DEEP R	168.5	64.2	25 - 115	3.6	1.3 - 7.2	6.7	2.4 - 14.7	20.1	6.3 - 52.4	20.6	8.2 - 42.7	0.0	0.0 - 0.0	13.3	5.0 - 29.2	0.78
3030003	5197	SANDY CR	153.0	45.3	16 - 103	0.0	0.0 - 0.0	5.1	1.5 - 12.0	14.8	5.0 - 39.4	15.6	5.6 - 48.9	0.0	0.0 - 0.0	9.8	3.5 - 25.3	0.75
3030003	5198	DEEP R	132.2	129.5	42 - 334	74.0	23.2 - 200.3	18.2	5.4 - 51.3	11.3	3.0 - 37.8	11.9	3.9 - 34.3	0.0	0.0 - 0.0	14.1	4.6 - 37.7	0.75
3030003	5199	POLECAT CR	146.5	47.5	16 - 104	0.8	0.2 - 1.9	8.6	2.5 - 19.4	11.0	3.1 - 36.9	16.8	5.3 - 46.8	0.0	0.0 - 0.0	10.3	3.2 - 29.6	0.72
3030003	5200	DEEP R	37.6	107.7	36 - 250	46.2	15.3 - 110.0	21.1	6.9 - 45.5	12.9	4.0 - 43.5	13.4	4.8 - 35.7	0.0	0.0 - 0.0	14.1	4.3 - 34.4	0.72
3030003	5201	DEEP R	34.0	59.4	22 - 143	0.9	0.4 - 2.2	11.2	4.4 - 26.4	16.4	5.8 - 48.1	20.7	7.6 - 59.4	0.0	0.0 - 0.0	10.1	4.0 - 26.8	0.70
3030003	5203	DEEP R	102.4	20.2	6 - 40	0.0	0.0 - 0.0	14.1	4.3 - 29.0	0.4	0.1 - 1.0	2.4	0.7 - 5.5	0.0	0.0 - 0.0	3.4	1.2 - 8.0	0.61
3030003	5204	MUDDY CR	64.9	70.0	20 - 124	0.3	0.1 - 0.7	26.9	7.3 - 49.4	15.7	4.0 - 39.0	16.9	5.2 - 37.5	0.0	0.0 - 0.0	10.2	2.8 - 24.0	0.70
3030003	5205	RICHLAND CR	169.0	47.6	16 - 81	0.0	0.0 - 0.0	8.8	2.8 - 17.4	12.4	3.4 - 39.3	12.7	4.3 - 32.5	0.0	0.0 - 0.0	13.8	4.7 - 29.2	0.78
3030003	5206	FORK CR	125.3	49.3	15 - 89	0.1	0.0 - 0.3	3.0	0.9 - 6.0	16.2	4.3 - 38.0	16.1	5.2 - 35.3	0.0	0.0 - 0.0	13.9	4.1 - 31.7	0.80
3030003	5207	CABIN CR	22.0	45.8	15 - 105	0.0	0.0 - 0.0	11.0	3.6 - 25.5	11.2	3.3 - 33.0	8.9	3.0 - 24.4	0.0	0.0 - 0.0	14.7	5.1 - 40.0	0.80
3030003	5208	BEAR CR	133.6	45.3	17 - 83	0.0	0.0 - 0.0	4.8	1.5 - 10.1	15.5	5.0 - 43.5	11.6	3.9 - 30.5	0.0	0.0 - 0.0	13.4	4.8 - 32.5	0.78
3030003	5209	CABIN CR	47.4	28.3	8 - 57	0.0	0.0 - 0.0	3.7	1.1 - 7.6	6.4	1.6 - 15.7	4.7	1.3 - 11.1	0.0	0.0 - 0.0	13.5	4.1 - 28.7	0.78
3030003	5210	CABIN CR	9.7	30.7	9 - 75	0.0	0.0 - 0.0	3.8	1.1 - 8.9	7.0	1.8 - 22.4	5.5	1.5 - 13.6	0.0	0.0 - 0.0	14.5	4.2 - 35.0	0.76
3030003	5211	CABIN CR	73.7	45.9	14 - 116	5.1	1.6 - 12.3	9.9	3.1 - 24.4	11.0	3.0 - 26.2	7.0	2.2 - 19.2	0.0	0.0 - 0.0	13.0	4.0 - 34.6	0.72
3030003	5212	MILL CR	44.4	27.9	11 - 48	0.0	0.0 - 0.0	3.2	1.2 - 6.1	8.0	2.8 - 19.1	5.8	2.2 - 13.0	0.0	0.0 - 0.0	10.9	4.2 - 23.0	0.72
3030003	5213	WET CR	44.2	23.9	9 - 58	0.0	0.0 - 0.0	3.1	1.1 - 7.8	5.8	2.1 - 23.0	4.4	1.5 - 12.7	0.0	0.0 - 0.0	10.5	3.9 - 28.4	0.76
3030003	5214	MCLENDON'S CR	15.5	22.3	7 - 45	0.0	0.0 - 0.0	3.3	1.0 - 6.4	6.0	1.5 - 15.9	4.7	1.5 - 11.4	0.0	0.0 - 0.0	8.4	2.8 - 17.7	0.84
3030003	5215	RICHLAND CR	67.0	19.5	7 - 39	0.0	0.0 - 0.0	1.9	0.6 - 4.1	5.1	1.5 - 15.6	3.8	1.4 - 8.6	0.0	0.0 - 0.0	8.6	3.1 - 20.7	0.82
3030003	5216	MCLENDON'S CR	179.5	23.4	10 - 55	0.0	0.0 - 0.0	5.4	2.1 - 13.4	4.4	1.6 - 14.6	3.2	1.3 - 8.7	0.0	0.0 - 0.0	10.4	4.3 - 25.9	0.82
3030004	5217	CAPE FEAR R	53.5	595.8	211 - 1412	584.1	206.0 - 1381.5	2.7	0.8 - 5.5	2.1	0.6 - 6.4	3.5	1.3 - 9.3	0.0	0.0 - 0.0	3.4	1.2 - 8.5	0.94
3030004	5218	CAPE FEAR R	42.5	47.4	15 - 114	0.0	0.0 - 0.0	26.5	8.2 - 63.3	3.6	1.1 - 10.5	6.2	2.2 - 14.7	0.0	0.0 - 0.0	11.1	3.6 - 30.1	0.94
3030004	5219	CAPE FEAR R	407.1	111.3	41 - 215	68.7	24.9 - 150.2	9.8	3.6 - 18.5	7.5	2.4 - 18.4	13.2	4.8 - 30.7	0.0	0.0 - 0.0	12.1	4.5 - 29.6	0.93
3030004	5220	CAPE FEAR R	34.0	165.8	61 - 372	99.0	37.2 - 235.2	9.9	3.5 - 21.3	18.5	6.1 - 51.6	24.0	8.6 - 55.7	0.0	0.0 - 0.0	14.4	5.4 - 37.9	0.92
3030004	5221	CAPE FEAR R	59.1	47.9	16 - 94	12.1	3.5 - 27.9	6.7	2.2 - 16.7	10.3	2.9 - 33.5	13.0	4.5 - 31.1	0.0	0.0 - 0.0	5.8	1.7 - 15.7	0.91
3030004	5222	BLUES CR	78.7	38.7	14 - 92	10.0	3.0 - 27.2	8.8	2.9 - 21.9	7.6	2.2 - 20.0	10.1	3.5 - 27.4	0.0	0.0 - 0.0	2.2	0.7 - 5.5	0.91
3030004	5223	CAPE FEAR R	22.3	66.2	25 - 140	26.1	10.3 - 70.4	14.1	5.3 - 32.9	8.5	2.9 - 27.1	11.4	4.3 - 30.0	0.0	0.0 - 0.0	6.1	2.4 - 17.0	0.91
3030004	5224	NEAL CR	103.7	53.1	20 - 146	24.3	9.1 - 65.5	12.3	4.5 - 32.8	4.3	1.3 - 13.1	8.1	3.0 - 21.9	0.0	0.0 - 0.0	4.1	1.5 - 11.5	0.90
3030004	5225	CAPE FEAR R	285.1	25.1	9 - 49	0.4	0.1 - 0.8	4.6	1.4 - 9.2	5.1	1.6 - 12.3	7.4	2.4 - 16.6	0.0	0.0 - 0.0	7.6	2.5 - 15.8	0.90
3030004	5226	WHITEOAK CR	217.6	38.2	14 - 81	11.8	4.4 - 27.4	8.8	3.2 - 21.1	1.3	0.4 - 4.0	4.9	2.0 - 12.7	0.0	0.0 - 0.0	11.3	4.4 - 28.6	0.89
3030004	5227	CAPE FEAR R	52.8	35.7	11 - 82	0.0	0.0 - 0.0	2.7	0.8 - 5.8	7.5	2.3 - 22.9	7.6	2.6 - 18.4	0.0	0.0 - 0.0	17.9	5.2 - 51.4	0.89

8-digit HUC	ID	Name	Area	Catchment Yield kg/km2	Catchment Yield 90% CI	Point sources kg/km2	Point sources 90% CI	Developed Land kg/km2	Developed Land 90% CI	Manure kg/km2	Manure 90% CI	Agricultural Land kg/km2	Agricultural Land 90% CI	Phosphate Mines kg/km2	Phosphate Mines 90% CI	Soil parent rock kg/km2	Soil parent rock 90% CI	Frac
3030004	5228	CAPE FEAR R	19.2	50.4	14 - 125	0.0	0.0 - 0.0	7.1	1.9 - 17.3	12.0	2.7 - 33.7	15.6	4.8 - 42.6	0.0	0.0 - 0.0	15.7	4.3 - 42.9	0.89
3030004	5229	LICK CR	120.4	27.2	10 - 55	0.0	0.0 - 0.0	5.6	1.9 - 12.0	3.9	1.2 - 11.7	8.6	3.4 - 19.6	0.0	0.0 - 0.0	9.0	3.2 - 21.6	0.89
3030004	5230	UPPER LITTLE R	6.7	48.5	12 - 128	0.0	0.0 - 0.0	5.5	1.4 - 15.9	11.1	2.4 - 36.4	11.8	3.7 - 33.0	0.0	0.0 - 0.0	20.1	4.7 - 57.0	0.91
3030004	5231	UPPER LITTLE R	178.8	34.7	10 - 85	2.7	0.8 - 7.0	12.5	3.5 - 30.1	4.7	1.2 - 16.8	8.5	2.6 - 21.9	0.0	0.0 - 0.0	6.4	1.9 - 15.9	0.77
3030004	5232	BARBECUE CR	175.6	23.6	8 - 50	0.0	0.0 - 0.0	7.4	2.7 - 15.5	3.5	1.1 - 8.4	4.8	1.6 - 13.1	0.0	0.0 - 0.0	7.8	2.5 - 16.9	0.77
3030004	5233	LOWER LITTLE R	64.7	41.5	12 - 92	0.0	0.0 - 0.0	6.0	1.6 - 13.1	10.7	2.8 - 29.6	14.4	4.0 - 31.5	0.0	0.0 - 0.0	10.5	3.0 - 22.6	0.92
3030004	5234	ANDERSON CR	96.7	22.7	9 - 44	0.0	0.0 - 0.0	6.8	2.5 - 13.5	3.7	1.1 - 10.0	4.5	1.6 - 9.8	0.0	0.0 - 0.0	7.9	3.0 - 17.4	0.90
3030004	5235	LOWER LITTLE R	61.9	28.1	10 - 59	0.0	0.0 - 0.0	8.3	2.7 - 17.7	4.1	1.3 - 11.3	5.6	1.7 - 12.1	0.0	0.0 - 0.0	10.1	3.3 - 25.0	0.90
3030004	5236	JUMPING RUN CR	74.1	25.4	10 - 66	3.0	1.1 - 7.2	11.0	3.7 - 29.0	2.0	0.7 - 6.1	2.1	0.7 - 6.3	0.0	0.0 - 0.0	7.3	2.8 - 19.3	0.88
3030004	5237	LOWER LITTLE R	282.5	49.7	15 - 123	26.2	9.0 - 70.9	13.3	4.5 - 31.3	1.1	0.3 - 3.6	1.1	0.4 - 3.1	0.0	0.0 - 0.0	8.0	2.6 - 20.0	0.88
3030004	5238	CRANE CR	263.9	25.3	10 - 70	0.2	0.1 - 0.5	7.0	2.3 - 20.0	5.0	1.8 - 16.0	4.4	1.5 - 13.7	0.0	0.0 - 0.0	8.7	3.2 - 23.6	0.83
3030004	5239	LOWER LITTLE R	18.4	16.0	5 - 36	0.0	0.0 - 0.0	4.4	1.3 - 10.3	1.9	0.5 - 5.5	1.5	0.5 - 4.3	0.0	0.0 - 0.0	8.2	2.6 - 21.0	0.83
3030004	5240	LOWER LITTLE R	29.3	24.0	8 - 44	1.9	0.6 - 4.0	4.0	1.4 - 7.2	5.7	1.7 - 13.6	4.1	1.5 - 10.1	0.0	0.0 - 0.0	8.3	3.0 - 16.0	0.82
3030004	5241	LOWER LITTLE R	207.8	31.7	11 - 60	0.0	0.0 - 0.0	14.3	4.9 - 27.0	4.1	1.3 - 9.1	3.0	1.0 - 8.3	0.0	0.0 - 0.0	10.4	3.6 - 23.8	0.78
3030004	5242	MILL CR	54.6	41.4	12 - 89	0.1	0.0 - 0.2	26.8	7.3 - 60.0	3.6	1.2 - 10.6	2.7	0.9 - 7.0	0.0	0.0 - 0.0	8.2	2.7 - 19.2	0.78
3030004	5243	JAMES CR	87.4	20.4	7 - 39	0.0	0.0 - 0.0	7.5	2.4 - 13.6	2.8	0.8 - 7.4	2.1	0.8 - 4.7	0.0	0.0 - 0.0	8.0	2.6 - 18.2	0.82
3030004	5244	BRANSON CR	3.0	83.6	29 - 217	0.0	0.0 - 0.0	68.3	23.1 - 185.2	2.5	0.6 - 7.7	4.1	1.3 - 11.3	0.0	0.0 - 0.0	8.6	3.1 - 24.0	0.93
3030004	5245	*A	3.6	96.1	35 - 198	0.0	0.0 - 0.0	85.1	29.3 - 172.0	0.1	0.0 - 0.2	0.1	0.0 - 0.3	0.0	0.0 - 0.0	10.8	4.0 - 22.6	0.91
3030004	5246	*A	41.4	48.4	15 - 106	0.0	0.0 - 0.0	40.0	12.9 - 87.1	0.5	0.1 - 1.8	0.9	0.3 - 2.3	0.0	0.0 - 0.0	7.1	2.1 - 18.4	0.87
3030004	5247	*B	24.7	57.4	18 - 123	0.0	0.0 - 0.0	49.3	15.8 - 105.9	0.3	0.1 - 0.6	0.4	0.1 - 1.1	0.0	0.0 - 0.0	7.4	2.5 - 16.0	0.87
3030004	5248	BRANSON CR	28.4	72.1	24 - 155	0.0	0.0 - 0.0	64.7	21.1 - 137.0	0.2	0.1 - 0.7	0.3	0.1 - 0.8	0.0	0.0 - 0.0	6.9	2.6 - 19.1	0.91
3030004	5249	ROCKFISH CR	47.1	39.5	12 - 113	0.0	0.0 - 0.0	22.6	6.5 - 65.3	3.3	0.9 - 10.2	5.9	1.8 - 15.8	0.0	0.0 - 0.0	7.7	2.2 - 22.1	0.94
3030004	5250	LITTLE ROCKFISH	37.5	68.3	23 - 112	0.0	0.0 - 0.0	60.8	20.8 - 108.4	0.6	0.2 - 1.7	0.9	0.3 - 2.2	0.0	0.0 - 0.0	5.9	1.9 - 12.9	0.92
3030004	5251	BEAVER CR	30.3	65.1	22 - 195	0.0	0.0 - 0.0	58.2	19.3 - 167.2	0.7	0.2 - 2.3	1.2	0.4 - 3.2	0.0	0.0 - 0.0	5.0	1.8 - 12.7	0.88
3030004	5252	BEAVER CR	42.3	63.8	21 - 147	0.0	0.0 - 0.0	55.8	17.9 - 127.1	0.6	0.2 - 2.0	1.1	0.3 - 3.3	0.0	0.0 - 0.0	6.2	2.1 - 15.4	0.80
3030004	5253	STEWART CR	20.0	39.6	13 - 108	0.0	0.0 - 0.0	32.9	10.2 - 86.2	0.4	0.1 - 1.0	0.6	0.2 - 1.7	0.0	0.0 - 0.0	5.8	1.8 - 18.7	0.80
3030004	5254	LITTLE ROCKFISH	22.9	46.4	17 - 94	0.0	0.0 - 0.0	37.1	13.6 - 78.2	1.4	0.4 - 3.2	2.4	0.8 - 5.6	0.0	0.0 - 0.0	5.5	2.2 - 11.5	0.88
3030004	5255	BONES CR	53.1	27.0	8 - 56	0.0	0.0 - 0.0	18.4	5.0 - 41.3	0.8	0.2 - 2.1	1.2	0.4 - 3.8	0.0	0.0 - 0.0	6.6	2.0 - 17.5	0.83
3030004	5256	LITTLE ROCKFISH	51.8	20.2	8 - 44	0.0	0.0 - 0.0	8.9	3.6 - 20.2	1.7	0.5 - 6.0	2.8	1.1 - 6.2	0.0	0.0 - 0.0	6.8	2.7 - 17.4	0.83
3030004	5257	ROCKFISH CR	267.0	49.3	18 - 90	21.4	7.7 - 41.7	13.1	4.5 - 28.5	3.0	1.0 - 7.0	5.2	1.6 - 12.7	0.0	0.0 - 0.0	6.7	2.5 - 14.3	0.92
3030004	5258	NICHOLSON CR	52.9	18.8	7 - 37	0.0	0.0 - 0.0	9.9	3.7 - 21.7	0.6	0.2 - 1.4	0.9	0.3 - 2.5	0.0	0.0 - 0.0	7.5	2.7 - 16.1	0.81
3030004	5259	ROCKFISH CR	25.8	15.9	7 - 38	0.0	0.0 - 0.0	5.2	2.0 - 12.2	0.9	0.3 - 2.7	1.4	0.5 - 3.8	0.0	0.0 - 0.0	8.4	3.6 - 20.5	0.81
3030004	5260	JUNIPER CR	36.7	16.1	5 - 31	0.0	0.0 - 0.0	8.3	2.3 - 15.8	0.1	0.0 - 0.2	0.0	0.0 - 0.0	0.0	0.0 - 0.0	7.7	2.2 - 16.8	0.78
3030004	5261	ROCKFISH CR	116.3	15.9	5 - 32	0.0	0.0 - 0.0	7.9	2.6 - 16.0	0.1	0.0 - 0.3	0.2	0.0 - 0.4	0.0	0.0 - 0.0	7.7	2.8 - 19.8	0.78
3030005	5262	NE CAPE FEAR R	24.9	481.7	173 - 1071	458.2	167.9 - 1081.4	21.5	6.5 - 49.9	0.0	0.0 - 0.1	0.2	0.1 - 0.7	0.0	0.0 - 0.0	1.7	0.6 - 3.6	1.00
3030005	5263	CAPE FEAR R	266.7	61.7	24 - 141	36.9	15.4 - 87.8	10.7	3.2 - 23.1	1.9	0.6 - 7.2	3.7	1.5 - 11.5	0.0	0.0 - 0.0	8.5	3.1 - 23.0	1.00
3030005	5264	CAPE FEAR R	54.4	12.4	4 - 25	0.0	0.0 - 0.0	2.3	0.7 - 4.7	3.7	1.0 - 8.1	1.5	0.5 - 4.1	0.0	0.0 - 0.0	4.9	1.5 - 10.7	0.97
3030005	5265	TURNBULL CR	235.0	5.8	2 - 12	0.0	0.0 - 0.0	0.7	0.2 - 1.5	2.2	0.6 - 5.6	1.1	0.3 - 2.6	0.0	0.0 - 0.0	1.8	0.6 - 4.2	0.96
3030005	5266	CAPE FEAR R	64.5	68.7	28 - 134	25.6	10.5 - 56.6	11.5	4.6 - 23.5	17.1	6.2 - 48.3	7.2	3.1 - 21.8	0.0	0.0 - 0.0	7.2	3.0 - 20.4	0.96

8-digit HUC	ID	Name	Area	Catchment Yield		Point sources		Developed Land		Manure		Agricultural Land		Phosphate Mines		Soil parent rock		Frac
				kg/km^2	90% CI	kg/km^2	90% CI	kg/km^2	90% CI	kg/km^2	90% CI	kg/km^2	90% CI	kg/km^2	90% CI	kg/km^2	90% CI	
3030005	5267	ELLIS CR	69.4	17.9	6 - 40	0.0	0.0 - 0.0	1.0	0.3 - 2.3	8.4	2.7 - 23.2	3.5	1.1 - 10.2	0.0	0.0 - 0.0	5.0	1.7 - 11.3	0.96
3030005	5268	*A	39.6	1.9	1 - 5	0.0	0.0 - 0.0	0.1	0.0 - 0.2	0.6	0.2 - 2.0	0.2	0.1 - 0.9	0.0	0.0 - 0.0	0.9	0.3 - 2.5	0.88
3030005	5269	ELLIS CR	21.8	4.0	1 - 9	0.0	0.0 - 0.0	0.3	0.1 - 0.8	1.3	0.4 - 4.2	0.5	0.1 - 1.5	0.0	0.0 - 0.0	1.8	0.6 - 4.6	0.88
3030005	5270	CAPE FEAR R	40.6	54.3	19 - 119	0.0	0.0 - 0.0	3.5	1.2 - 8.5	28.6	9.8 - 69.0	12.1	4.1 - 33.6	0.0	0.0 - 0.0	10.2	3.1 - 27.0	0.96
3030005	5271	HARRISON CR	163.7	4.0	1 - 10	0.0	0.0 - 0.0	0.4	0.1 - 1.1	1.5	0.4 - 4.6	1.0	0.3 - 4.0	0.0	0.0 - 0.0	1.0	0.3 - 2.7	0.96
3030005	5272	CAPE FEAR R	235.0	490.4	175 - 1136	452.4	156.7 - 1084.2	4.3	1.5 - 9.0	13.9	4.2 - 37.0	10.9	3.7 - 27.2	0.0	0.0 - 0.0	8.9	3.3 - 19.7	0.96
3030005	5273	HAMMOND CR	92.9	47.3	17 - 106	0.0	0.0 - 0.0	2.9	1.0 - 7.3	25.9	8.1 - 84.3	10.6	3.9 - 31.1	0.0	0.0 - 0.0	7.9	2.7 - 20.2	0.97
3030005	5274	CAPE FEAR R	43.5	22.1	7 - 61	0.0	0.0 - 0.0	1.0	0.3 - 2.3	2.7	0.7 - 9.6	5.4	1.8 - 14.5	0.0	0.0 - 0.0	12.9	3.9 - 40.7	0.99
3030006	5275	BLACK R	15.2	21.9	9 - 56	0.0	0.0 - 0.0	1.2	0.4 - 3.3	2.0	0.6 - 5.9	1.4	0.6 - 4.8	0.0	0.0 - 0.0	17.3	6.9 - 45.0	0.99
3030006	5276	BLACK R	36.0	10.5	3 - 25	0.0	0.0 - 0.0	0.6	0.1 - 1.4	2.1	0.6 - 5.8	1.4	0.3 - 3.7	0.0	0.0 - 0.0	6.4	2.0 - 16.8	0.99
3030006	5277	MOORES CR	242.4	19.4	6 - 41	0.0	0.0 - 0.0	1.4	0.5 - 2.9	8.0	2.2 - 20.8	5.6	1.9 - 16.4	0.0	0.0 - 0.0	4.4	1.6 - 10.9	0.98
3030006	5278	BLACK R	278.7	5.4	2 - 10	0.0	0.0 - 0.0	0.5	0.1 - 1.0	2.1	0.5 - 5.2	0.9	0.3 - 2.5	0.0	0.0 - 0.0	1.9	0.6 - 4.2	0.98
3030006	5279	BLACK R	143.1	19.5	7 - 44	0.0	0.0 - 0.0	1.1	0.4 - 2.9	8.5	2.4 - 25.2	4.5	1.7 - 11.1	0.0	0.0 - 0.0	5.4	2.0 - 12.4	0.98
3030006	5280	BLACK R	160.3	44.8	18 - 124	0.0	0.0 - 0.0	3.3	1.3 - 8.7	22.8	7.6 - 71.0	7.6	3.3 - 22.0	0.0	0.0 - 0.0	11.1	4.1 - 34.9	0.96
3030006	5281	SIX RUNS CR	50.2	72.3	23 - 169	0.0	0.0 - 0.0	3.6	1.1 - 8.3	41.5	11.6 - 124.1	13.9	4.3 - 31.1	0.0	0.0 - 0.0	13.4	4.2 - 32.3	0.93
3030006	5282	STEWART CR	199.7	83.4	24 - 146	4.5	1.4 - 8.1	5.7	1.7 - 10.3	46.2	12.9 - 102.0	12.9	4.1 - 26.6	0.0	0.0 - 0.0	14.1	5.0 - 28.6	0.90
3030006	5283	SIX RUNS CR	456.8	117.0	38 - 285	0.0	0.0 - 0.0	5.4	1.8 - 11.7	72.3	22.7 - 202.1	23.8	7.4 - 62.5	0.0	0.0 - 0.0	15.5	6.0 - 38.3	0.90
3030006	5284	BLACK R	51.9	74.3	28 - 175	3.3	1.2 - 8.4	5.6	2.1 - 12.1	39.0	15.4 - 114.7	13.1	5.0 - 35.9	0.0	0.0 - 0.0	13.4	4.7 - 32.4	0.93
3030006	5285	GREAT COHARIE C	385.0	157.6	54 - 392	61.7	22.8 - 160.5	8.7	2.6 - 20.7	55.3	16.0 - 165.1	18.6	6.8 - 49.2	0.0	0.0 - 0.0	13.3	4.8 - 36.9	0.91
3030006	5286	GREAT COHARIE C	8.6	160.6	54 - 343	0.0	0.0 - 0.0	5.0	1.7 - 11.1	102.1	30.1 - 299.1	34.3	13.0 - 87.0	0.0	0.0 - 0.0	19.2	6.3 - 48.3	0.83
3030006	5287	KILL SWAMP	26.4	114.0	36 - 350	0.0	0.0 - 0.0	6.6	2.1 - 16.4	70.4	20.1 - 268.9	23.7	7.4 - 62.5	0.0	0.0 - 0.0	13.3	4.5 - 37.2	0.81
3030006	5288	GREAT COHARIE C	63.5	120.9	40 - 251	2.1	0.7 - 4.9	8.8	2.5 - 19.9	71.5	21.3 - 216.2	24.9	7.9 - 67.0	0.0	0.0 - 0.0	13.6	4.7 - 41.9	0.81
3030006	5289	SEVEN MILE SWAI	51.0	110.0	39 - 280	0.0	0.0 - 0.0	4.8	1.6 - 13.5	68.0	22.7 - 203.1	23.1	7.8 - 66.8	0.0	0.0 - 0.0	14.0	4.4 - 36.2	0.83
3030006	5290	SITTLE COHARIE C	82.5	101.9	28 - 201	0.0	0.0 - 0.0	3.2	0.9 - 6.3	61.0	16.2 - 143.2	20.8	6.1 - 47.7	0.0	0.0 - 0.0	16.9	5.1 - 39.2	0.91
3030006	5291	BEARSKIN SWAMI	71.2	89.7	29 - 183	0.0	0.0 - 0.0	5.8	1.8 - 12.9	54.3	15.7 - 137.6	18.1	6.2 - 39.7	0.0	0.0 - 0.0	11.5	3.8 - 24.4	0.87
3030006	5292	SITTLE COHARIE C	253.6	101.1	34 - 201	1.5	0.5 - 3.2	7.0	2.0 - 14.1	59.6	18.4 - 166.0	20.0	6.9 - 53.2	0.0	0.0 - 0.0	12.9	4.5 - 27.5	0.87
3030006	5293	SOUTH R	5.4	159.2	54 - 312	0.0	0.0 - 0.0	2.1	0.6 - 3.9	99.2	29.6 - 222.5	41.2	12.8 - 94.2	0.0	0.0 - 0.0	16.7	5.5 - 34.7	0.96
3030006	5294	SOUTH R	292.3	43.9	16 - 89	2.4	0.9 - 5.3	2.0	0.6 - 4.6	22.0	7.2 - 72.7	8.6	2.8 - 24.9	0.0	0.0 - 0.0	8.8	3.1 - 22.3	0.96
3030006	5295	SOUTH R	10.4	42.6	15 - 86	0.0	0.0 - 0.0	2.0	0.7 - 4.2	13.2	4.3 - 37.2	8.7	2.9 - 20.7	0.0	0.0 - 0.0	18.7	6.6 - 50.0	0.88
3030006	5296	BIG SWAMP	119.6	131.2	39 - 279	0.0	0.0 - 0.0	4.8	1.5 - 9.9	79.8	22.2 - 230.9	26.7	8.1 - 60.2	0.0	0.0 - 0.0	20.0	6.1 - 45.6	0.87
3030006	5297	SOUTH R	364.7	70.5	24 - 181	0.0	0.0 - 0.0	4.4	1.5 - 10.7	32.2	9.1 - 91.3	18.8	5.9 - 50.6	0.0	0.0 - 0.0	15.2	5.3 - 43.6	0.87
3030006	5298	MINGO SWAMP	203.9	71.1	20 - 154	0.0	0.0 - 0.0	8.9	2.7 - 20.5	32.2	8.6 - 82.5	19.6	6.1 - 48.7	0.0	0.0 - 0.0	10.3	3.4 - 23.8	0.73
3030006	5299	BLACK R	149.8	35.9	10 - 79	0.0	0.0 - 0.0	10.5	3.0 - 24.2	8.4	2.2 - 26.8	11.5	3.8 - 28.3	0.0	0.0 - 0.0	5.5	1.6 - 12.5	0.73
3030006	5300	BEAVERDAM CR	72.8	17.7	5 - 41	0.0	0.0 - 0.0	2.0	0.7 - 4.8	3.6	1.0 - 11.4	6.1	1.8 - 17.1	0.0	0.0 - 0.0	6.1	1.9 - 15.2	0.88
3030006	5301	LAKE CR	76.3	5.9	2 - 13	0.2	0.1 - 0.4	0.3	0.1 - 0.7	3.1	0.7 - 7.9	1.3	0.3 - 3.6	0.0	0.0 - 0.0	1.1	0.3 - 2.6	0.96
3030006	5302	LYON SWAMP CAI	123.5	19.2	6 - 38	0.0	0.0 - 0.0	0.7	0.2 - 1.4	10.1	2.8 - 29.1	4.4	1.3 - 9.8	0.0	0.0 - 0.0	3.9	1.3 - 8.2	0.99
3030007	5303	NE CAPE FEAR R	154.3	87.0	31 - 185	37.4	13.5 - 80.8	35.4	12.5 - 77.2	0.2	0.1 - 0.5	4.6	1.7 - 12.4	0.0	0.0 - 0.0	9.4	3.2 - 21.7	1.00
3030007	5304	NE CAPE FEAR R	577.4	19.2	7 - 43	2.7	1.0 - 5.9	2.3	0.8 - 5.0	5.1	1.6 - 13.9	4.1	1.4 - 11.5	0.0	0.0 - 0.0	5.1	1.6 - 12.6	0.98
3030007	5305	HOLLY CR	45.4	7.5	2 - 15	0.0	0.0 - 0.0	0.6	0.2 - 1.3	2.2	0.6 - 6.0	1.6	0.6 - 3.6	0.0	0.0 - 0.0	3.1	1.0 - 6.8	0.95

8-digit HUC	ID	Name	Area	Catchment Yield kg/km^2	Catchment Yield 90% CI	Point sources kg/km^2	Point sources 90% CI	Developed Land kg/km^2	Developed Land 90% CI	Manure kg/km^2	Manure 90% CI	Agricultural Land kg/km^2	Agricultural Land 90% CI	Phosphate Mines kg/km^2	Phosphate Mines 90% CI	Soil parent rock kg/km^2	Soil parent rock 90% CI	Frac
3030007	5306	SHAKEN CR	573.0	3.8	1 - 10	0.0	0.0 - 0.0	0.2	0.1 - 0.6	0.5	0.1 - 1.5	0.5	0.1 - 1.5	0.0	0.0 - 0.0	2.5	0.7 - 7.6	0.93
3030007	5307	ANGOLA CR	427.8	17.7	5 - 38	0.0	0.0 - 0.0	0.6	0.2 - 1.5	9.4	2.6 - 28.3	3.9	1.1 - 11.0	0.0	0.0 - 0.0	3.8	1.2 - 9.4	0.93
3030007	5308	NE CAPE FEAR R	104.2	17.9	7 - 41	0.0	0.0 - 0.0	2.2	0.8 - 5.0	6.6	2.2 - 20.3	4.7	1.9 - 13.1	0.0	0.0 - 0.0	4.5	1.8 - 11.4	0.95
3030007	5309	NE CAPE FEAR R	348.6	98.5	31 - 213	2.6	0.9 - 6.6	4.2	1.4 - 9.6	67.7	21.5 - 172.8	16.9	4.7 - 45.1	0.0	0.0 - 0.0	7.2	2.2 - 17.5	0.92
3030007	5310	NE CAPE FEAR R	26.6	113.1	31 - 209	0.0	0.0 - 0.0	1.5	0.5 - 2.9	76.8	21.7 - 184.9	19.3	6.1 - 49.6	0.0	0.0 - 0.0	15.5	5.2 - 40.1	0.90
3030007	5311	NE CAPE FEAR R	48.6	138.3	51 - 306	0.0	0.0 - 0.0	3.1	1.1 - 6.7	88.2	28.7 - 245.1	21.9	7.9 - 54.5	0.0	0.0 - 0.0	25.1	9.6 - 58.6	0.87
3030007	5312	NE CAPE FEAR R	369.5	95.5	28 - 179	2.4	0.7 - 4.7	4.8	1.5 - 9.5	57.5	15.8 - 151.2	18.2	5.0 - 40.0	0.0	0.0 - 0.0	12.5	3.6 - 27.6	0.86
3030007	5313	GOSHEN SWAMP	222.7	112.5	38 - 262	1.8	0.7 - 3.6	2.6	0.9 - 6.0	74.7	23.6 - 190.6	18.7	7.4 - 52.4	0.0	0.0 - 0.0	14.7	5.3 - 34.5	0.86
3030007	5314	GOSHEN SWAMP	205.4	152.6	57 - 444	0.0	0.0 - 0.0	5.9	2.1 - 16.7	101.9	35.2 - 378.4	30.0	11.2 - 92.1	0.0	0.0 - 0.0	14.8	5.4 - 42.1	0.80
3030007	5315	NAHUNGA CR	53.0	176.5	64 - 391	0.0	0.0 - 0.0	4.8	1.9 - 10.0	123.9	41.6 - 308.9	30.6	11.3 - 70.3	0.0	0.0 - 0.0	17.2	5.6 - 41.4	0.80
3030007	5316	GROVE CR	109.3	95.9	32 - 240	4.0	1.4 - 11.5	5.0	1.8 - 11.7	57.1	17.6 - 139.0	14.3	5.1 - 48.8	0.0	0.0 - 0.0	15.5	5.2 - 41.7	0.87
3030007	5317	STOCKINGHEAD C	174.1	62.6	19 - 121	0.0	0.0 - 0.0	4.4	1.5 - 8.8	41.0	12.1 - 105.5	10.2	3.7 - 25.0	0.0	0.0 - 0.0	7.0	2.5 - 15.1	0.90
3030007	5318	ROCKFISH CR	122.9	65.0	18 - 133	7.2	2.2 - 15.0	5.7	1.5 - 12.2	31.0	7.8 - 78.2	14.3	4.5 - 34.2	0.0	0.0 - 0.0	6.9	1.9 - 15.7	0.92
3030007	5319	ROCKFISH CR	180.0	125.5	50 - 285	31.6	12.1 - 85.7	3.8	1.4 - 9.7	61.9	23.8 - 174.5	15.6	6.5 - 45.7	0.0	0.0 - 0.0	12.6	4.5 - 33.0	0.89
3030007	5320	DOCTORS CR	146.7	59.0	18 - 134	0.0	0.0 - 0.0	3.0	0.9 - 6.5	37.2	10.5 - 109.8	11.4	3.7 - 26.3	0.0	0.0 - 0.0	7.3	2.3 - 17.4	0.89
3030007	5321	LONG CR	379.1	17.3	5 - 34	0.0	0.0 - 0.1	1.1	0.3 - 2.8	7.3	2.0 - 21.4	5.2	1.7 - 12.6	0.0	0.0 - 0.0	3.7	1.4 - 7.8	0.98
3040101	5322	YADKIN R	73.9	32.3	9 - 78	0.0	0.0 - 0.0	4.4	1.3 - 10.5	3.4	1.0 - 9.7	12.3	3.6 - 34.7	0.0	0.0 - 0.0	12.2	3.7 - 31.5	0.47
3040101	5323	YADKIN R	49.3	29.6	10 - 56	0.0	0.0 - 0.0	4.6	1.5 - 8.8	2.8	0.7 - 6.9	10.5	3.2 - 24.6	0.0	0.0 - 0.0	11.8	3.9 - 24.1	0.47
3040101	5324	MUDDY CR	29.7	35.8	10 - 63	1.0	0.3 - 2.1	4.9	1.2 - 10.0	3.8	0.9 - 9.7	15.0	4.7 - 35.4	0.0	0.0 - 0.0	11.0	3.1 - 25.1	0.46
3040101	5325	*A	42.6	36.3	12 - 71	0.5	0.2 - 1.2	13.1	4.4 - 28.6	3.0	0.8 - 9.5	12.1	4.3 - 27.3	0.0	0.0 - 0.0	7.6	2.4 - 17.6	0.45
3040101	5326	MUDDY CR	2.1	23.6	8 - 57	0.0	0.0 - 0.0	0.8	0.3 - 1.9	2.3	0.7 - 6.8	11.3	4.0 - 31.2	0.0	0.0 - 0.0	9.1	3.1 - 25.0	0.45
3040101	5327	MUDDY CR, S FK	114.8	46.5	21 - 110	0.1	0.1 - 0.3	27.1	10.9 - 62.8	1.5	0.5 - 5.1	9.1	4.0 - 21.9	0.0	0.0 - 0.0	8.7	3.6 - 19.5	0.45
3040101	5328	MUDDY CR	2.1	33.1	11 - 60	0.0	0.0 - 0.0	3.8	1.3 - 6.7	2.2	0.6 - 5.5	17.6	6.3 - 37.7	0.0	0.0 - 0.0	9.5	3.3 - 19.5	0.45
3040101	5329	SALEM CR	179.8	235.2	82 - 552	186.9	63.3 - 456.6	36.1	12.5 - 83.8	0.4	0.1 - 1.1	2.9	1.0 - 7.3	0.0	0.0 - 0.0	8.8	2.9 - 22.7	0.45
3040101	5330	MUDDY CR	29.6	73.8	27 - 156	0.1	0.0 - 0.2	54.4	19.0 - 117.9	0.8	0.3 - 2.0	5.5	2.1 - 12.5	0.0	0.0 - 0.0	13.1	4.0 - 34.9	0.45
3040101	5331	SILAS CR	32.9	89.9	28 - 211	0.0	0.0 - 0.0	71.7	23.5 - 153.9	0.2	0.0 - 0.5	1.3	0.4 - 3.2	0.0	0.0 - 0.0	16.8	5.0 - 42.0	0.44
3040101	5332	MUDDY CR	49.9	63.0	19 - 118	0.0	0.0 - 0.0	44.0	13.6 - 85.7	0.8	0.2 - 2.1	6.3	1.9 - 13.6	0.0	0.0 - 0.0	11.9	3.3 - 26.5	0.44
3040101	5333	MILL CR	85.5	71.7	24 - 163	0.1	0.0 - 0.3	55.1	18.6 - 125.0	0.4	0.1 - 1.3	3.2	1.0 - 9.4	0.0	0.0 - 0.0	13.0	4.9 - 28.4	0.41
3040101	5334	MUDDY CR	92.2	43.0	14 - 101	0.0	0.0 - 0.0	23.0	6.8 - 50.3	1.2	0.3 - 3.0	8.1	2.3 - 21.1	0.0	0.0 - 0.0	10.7	3.6 - 26.9	0.41
3040101	5335	YADKIN R	213.0	267.6	112 - 571	224.6	95.0 - 515.5	13.9	5.0 - 30.3	3.6	1.2 - 10.7	13.2	5.2 - 33.3	0.0	0.0 - 0.0	12.2	4.6 - 31.8	0.46
3040101	5336	YADKIN R	3.9	79.4	28 - 180	0.0	0.0 - 0.0	11.6	3.5 - 28.8	13.4	3.9 - 40.2	29.6	10.7 - 80.3	0.0	0.0 - 0.0	24.8	8.2 - 72.1	0.45
3040101	5337	YADKIN R	156.4	36.0	12 - 81	0.5	0.2 - 1.4	8.6	2.6 - 19.7	3.7	1.1 - 9.2	10.4	3.5 - 29.9	0.0	0.0 - 0.0	12.8	4.7 - 29.9	0.45
3040101	5338	LITTLE YADKIN R	78.1	38.8	14 - 106	0.0	0.0 - 0.0	15.6	4.8 - 41.9	1.8	0.5 - 6.9	7.8	2.8 - 26.0	0.0	0.0 - 0.0	13.6	4.8 - 39.8	0.45
3040101	5339	LITTLE YADKIN R	38.3	27.2	10 - 63	0.0	0.0 - 0.0	6.1	2.0 - 13.8	2.1	0.6 - 6.0	10.0	3.5 - 26.4	0.0	0.0 - 0.0	9.0	3.5 - 22.5	0.39
3040101	5340	LITTLE YADKIN R	44.0	29.6	12 - 54	0.0	0.0 - 0.0	6.0	2.4 - 11.8	2.1	0.7 - 5.2	9.4	3.5 - 25.6	0.0	0.0 - 0.0	12.1	5.1 - 26.2	0.39
3040101	5341	YADKIN R	97.2	36.2	11 - 73	0.0	0.0 - 0.1	5.8	1.8 - 12.8	6.2	1.8 - 16.6	9.9	2.9 - 22.4	0.0	0.0 - 0.0	14.2	4.4 - 32.0	0.45
3040101	5342	ARARAT R	106.8	38.7	14 - 75	0.0	0.0 - 0.0	6.9	2.3 - 16.9	8.2	2.7 - 25.3	11.4	3.9 - 28.7	0.0	0.0 - 0.0	12.1	4.4 - 25.5	0.44
3040101	5343	TOMS CR	99.0	40.1	11 - 84	8.4	2.6 - 17.7	9.0	2.7 - 19.8	5.5	1.3 - 15.7	8.9	2.4 - 22.1	0.0	0.0 - 0.0	8.4	2.6 - 18.5	0.42
3040101	5344	ARARAT R	110.5	129.9	48 - 264	94.3	35.6 - 199.7	10.3	4.0 - 17.8	7.1	2.4 - 20.8	9.9	3.7 - 27.9	0.0	0.0 - 0.0	8.3	3.0 - 18.8	0.42

8-digit HUC	ID	Name	Area	Catchment Yield		Point sources		Developed Land		Manure		Agricultural Land		Phosphate Mines		Soil parent rock		Frac
				kg/km^2	90% CI	kg/km^2	90% CI	kg/km^2	90% CI	kg/km^2	90% CI	kg/km^2	90% CI	kg/km^2	90% CI	kg/km^2	90% CI	
3040101	5345	ARARAT R	8.8	63.5	17 - 139	0.0	0.0 - 0.0	45.4	12.5 - 104.2	3.5	1.0 - 9.5	4.8	1.3 - 12.1	0.0	0.0 - 0.0	9.9	2.8 - 23.8	0.40
3040101	5346	ARARAT R	188.3	31.3	10 - 73	0.1	0.0 - 0.3	10.7	3.3 - 24.1	3.3	0.8 - 9.2	7.3	2.3 - 18.9	0.0	0.0 - 0.0	9.8	3.3 - 25.4	0.40
3040101	5347	LOVILLS CR	92.7	42.1	15 - 103	0.0	0.0 - 0.0	23.9	7.5 - 59.9	2.4	0.9 - 7.5	5.5	1.8 - 13.1	0.0	0.0 - 0.0	10.2	3.7 - 24.5	0.40
3040101	5348	STEWARTS CR	38.3	55.2	20 - 125	0.4	0.2 - 0.9	22.8	8.3 - 53.4	9.4	2.9 - 26.6	13.4	5.0 - 36.9	0.0	0.0 - 0.0	9.3	3.5 - 21.2	0.40
3040101	5349	PAULS CR	76.1	34.5	12 - 84	0.0	0.0 - 0.0	12.9	4.3 - 31.9	3.7	1.2 - 10.4	7.4	2.7 - 21.2	0.0	0.0 - 0.0	10.5	4.1 - 27.3	0.38
3040101	5350	STEWARTS CR	93.3	33.2	15 - 63	1.8	0.8 - 3.7	9.0	3.8 - 18.8	4.9	2.0 - 13.8	7.8	2.8 - 20.0	0.0	0.0 - 0.0	9.8	3.9 - 21.7	0.38
3040101	5351	YADKIN R	143.1	43.6	11 - 74	2.3	0.6 - 4.4	6.3	1.7 - 13.3	9.1	2.3 - 21.1	14.3	4.0 - 33.7	0.0	0.0 - 0.0	11.6	3.1 - 24.7	0.44
3040101	5352	FISHER R	31.6	37.6	14 - 98	0.0	0.0 - 0.0	6.0	2.3 - 16.6	9.3	2.9 - 26.5	13.0	4.9 - 36.0	0.0	0.0 - 0.0	9.4	3.9 - 25.1	0.44
3040101	5353	FISHER R	90.6	107.4	38 - 268	63.1	22.3 - 156.7	8.3	2.8 - 22.4	10.9	3.5 - 34.6	15.4	5.1 - 43.2	0.0	0.0 - 0.0	9.7	3.4 - 25.2	0.43
3040101	5354	LITTLE FISHER R	99.9	38.0	13 - 73	2.2	0.7 - 4.5	8.1	2.6 - 17.5	7.4	2.3 - 20.5	10.6	3.2 - 25.5	0.0	0.0 - 0.0	9.7	2.9 - 23.0	0.40
3040101	5355	FISHER R	159.5	25.7	8 - 47	0.0	0.0 - 0.0	5.5	1.9 - 10.6	4.6	1.4 - 11.7	6.6	2.2 - 15.8	0.0	0.0 - 0.0	9.0	3.0 - 20.6	0.40
3040101	5356	CODY CR	44.2	66.3	20 - 174	5.4	1.6 - 14.3	9.3	2.8 - 21.9	16.8	4.5 - 51.4	24.2	7.3 - 65.4	0.0	0.0 - 0.0	10.6	3.6 - 28.6	0.43
3040101	5357	YADKIN R	26.1	32.4	11 - 66	0.0	0.0 - 0.0	7.4	2.4 - 15.3	6.4	2.0 - 19.8	10.2	3.6 - 27.1	0.0	0.0 - 0.0	8.4	3.0 - 20.5	0.44
3040101	5358	MITCHELL R	4.1	31.5	10 - 60	0.0	0.0 - 0.0	9.4	2.9 - 19.5	5.9	1.7 - 14.9	7.9	2.5 - 19.2	0.0	0.0 - 0.0	8.3	2.5 - 17.9	0.43
3040101	5359	SNOW CR	46.8	53.1	19 - 99	0.0	0.0 - 0.0	8.0	2.8 - 15.3	14.2	4.8 - 32.5	20.0	6.5 - 43.9	0.0	0.0 - 0.0	11.0	3.9 - 23.9	0.43
3040101	5360	MITCHELL R	38.2	42.3	17 - 92	0.0	0.0 - 0.0	14.6	5.2 - 34.1	7.9	2.6 - 22.6	11.0	3.9 - 33.4	0.0	0.0 - 0.0	8.8	3.6 - 25.0	0.43
3040101	5361	MITCHELL R	129.5	19.2	7 - 38	0.0	0.0 - 0.0	3.3	1.2 - 7.1	2.5	0.8 - 7.0	3.5	1.1 - 8.6	0.0	0.0 - 0.0	9.9	3.2 - 25.6	0.41
3040101	5362	MITCHELL R, S FK	64.3	37.2	12 - 79	0.2	0.1 - 0.5	6.7	1.9 - 14.6	8.1	2.5 - 19.6	11.2	3.6 - 26.0	0.0	0.0 - 0.0	11.0	3.7 - 25.5	0.41
3040101	5363	ELKIN R	93.0	55.7	20 - 108	0.0	0.0 - 0.0	11.2	4.1 - 20.1	21.9	7.1 - 65.9	12.6	4.5 - 30.4	0.0	0.0 - 0.0	9.9	3.4 - 23.8	0.43
3040101	5364	YADKIN R	14.3	63.9	21 - 127	0.0	0.0 - 0.0	11.7	3.8 - 22.5	21.5	6.5 - 57.4	18.9	6.3 - 50.1	0.0	0.0 - 0.0	11.8	4.1 - 28.1	0.43
3040101	5365	LITTLE ELKIN R	34.1	57.5	24 - 134	0.0	0.0 - 0.0	6.0	2.1 - 13.7	27.4	9.6 - 79.9	15.2	5.6 - 39.1	0.0	0.0 - 0.0	8.8	3.5 - 20.5	0.43
3040101	5366	YADKIN R	75.2	76.9	27 - 172	0.4	0.2 - 1.0	5.1	1.9 - 11.7	36.8	11.4 - 99.1	21.4	8.6 - 53.1	0.0	0.0 - 0.0	13.2	5.3 - 36.6	0.43
3040101	5367	BIG BUGABOO CR	48.0	62.6	19 - 128	0.0	0.0 - 0.0	4.3	1.4 - 9.0	31.3	8.8 - 73.9	17.5	5.8 - 37.8	0.0	0.0 - 0.0	9.5	3.5 - 24.6	0.42
3040101	5368	YADKIN R	37.0	61.3	21 - 118	0.4	0.1 - 0.8	4.8	1.5 - 10.7	28.8	8.7 - 80.3	15.7	4.5 - 44.1	0.0	0.0 - 0.0	11.6	3.7 - 27.1	0.42
3040101	5369	ROARING R	47.2	47.5	16 - 123	0.0	0.0 - 0.0	5.1	1.6 - 12.8	20.7	6.1 - 59.8	11.7	3.5 - 36.2	0.0	0.0 - 0.0	10.0	3.4 - 28.3	0.42
3040101	5370	ROARING R, E PRC	146.6	28.8	10 - 55	0.1	0.0 - 0.2	4.1	1.4 - 7.7	9.9	3.1 - 28.1	5.8	2.1 - 14.3	0.0	0.0 - 0.0	8.9	3.3 - 18.5	0.41
3040101	5371	ROARING R	22.3	49.9	18 - 115	1.3	0.4 - 3.3	6.2	2.0 - 15.4	20.3	5.8 - 67.7	11.2	3.4 - 27.3	0.0	0.0 - 0.0	10.8	3.7 - 31.1	0.41
3040101	5372	ROARING R, M PRO	87.2	20.4	8 - 50	0.0	0.0 - 0.0	2.4	0.8 - 5.7	5.5	2.0 - 18.7	3.1	1.2 - 9.6	0.0	0.0 - 0.0	9.4	3.8 - 24.4	0.39
3040101	5373	ROARING R, W PRO	59.0	20.3	6 - 39	0.0	0.0 - 0.0	2.1	0.7 - 4.1	5.0	1.4 - 11.8	2.8	0.8 - 6.8	0.0	0.0 - 0.0	10.5	2.8 - 25.7	0.39
3040101	5374	YADKIN R	7.8	80.1	24 - 174	0.0	0.0 - 0.0	8.6	2.8 - 19.2	36.8	10.1 - 104.8	20.2	6.6 - 48.4	0.0	0.0 - 0.0	14.5	4.7 - 35.9	0.42
3040101	5375	YADKIN R	30.4	72.0	23 - 164	0.0	0.0 - 0.0	7.3	2.4 - 16.9	33.7	9.1 - 99.6	19.1	6.2 - 51.2	0.0	0.0 - 0.0	12.0	3.8 - 30.5	0.42
3040101	5376	MULBERRY CR	127.8	37.4	13 - 89	0.3	0.1 - 0.8	7.2	2.4 - 16.0	12.4	4.1 - 35.2	6.9	2.6 - 18.9	0.0	0.0 - 0.0	10.5	3.8 - 30.6	0.42
3040101	5377	YADKIN R	27.5	1722.2	619 - 4957	1654.4	594.7 - 4866.3	26.4	9.6 - 77.0	18.1	6.1 - 62.0	9.9	3.3 - 31.0	0.0	0.0 - 0.0	13.4	5.0 - 39.5	0.42
3040101	5378	REDDIES R	87.6	48.8	15 - 124	0.0	0.0 - 0.0	12.2	4.1 - 32.4	16.2	4.7 - 51.9	9.1	3.2 - 27.4	0.0	0.0 - 0.0	11.3	4.0 - 31.9	0.41
3040101	5379	REDDIES R, N FK	76.0	16.2	5 - 32	0.0	0.0 - 0.0	2.1	0.6 - 4.2	2.7	0.7 - 7.7	1.6	0.5 - 4.4	0.0	0.0 - 0.0	9.8	3.3 - 23.7	0.38
3040101	5380	REDDIES R	3.8	27.2	10 - 51	0.0	0.0 - 0.0	2.2	0.8 - 4.2	8.5	2.8 - 21.0	4.8	1.7 - 10.7	0.0	0.0 - 0.0	11.7	4.4 - 26.9	0.38
3040101	5381	REDDIES R, M FK	45.7	20.7	8 - 39	0.0	0.0 - 0.0	3.7	1.4 - 7.8	4.6	1.5 - 10.9	2.9	1.0 - 8.0	0.0	0.0 - 0.0	9.4	3.8 - 20.5	0.37
3040101	5382	REDDIES R	30.7	19.4	6 - 47	0.0	0.0 - 0.0	3.3	0.8 - 8.4	3.8	1.1 - 11.0	2.1	0.6 - 5.7	0.0	0.0 - 0.0	10.2	3.6 - 27.7	0.37
3040101	5383	YADKIN R	39.0	58.2	19 - 122	0.0	0.0 - 0.0	25.6	8.6 - 55.4	13.1	4.0 - 36.6	7.0	2.0 - 16.2	0.0	0.0 - 0.0	12.5	4.3 - 26.9	0.41

8-digit HUC	ID	Name	Area	Catchment Yield		Point sources		Developed Land		Manure		Agricultural Land		Phosphate Mines		Soil parent rock		Frac
				kg/km^2	90% CI	kg/km^2	90% CI	kg/km^2	90% CI	kg/km^2	90% CI	kg/km^2	90% CI	kg/km^2	90% CI	kg/km^2	90% CI	
3040101	5384	YADKIN R	65.3	33.1	11 - 64	0.0	0.0 - 0.0	12.3	4.0 - 26.7	9.2	2.7 - 25.7	5.1	1.3 - 12.7	0.0	0.0 - 0.0	6.5	2.1 - 14.4	0.41
3040101	5385	YADKIN R	1.0	22.9	8 - 45	0.0	0.0 - 0.0	10.2	3.6 - 23.3	2.5	0.8 - 6.7	1.3	0.5 - 3.6	0.0	0.0 - 0.0	8.8	3.4 - 21.7	0.22
3040101	5386	LEWIS FK CR, N PF	22.1	51.4	18 - 93	0.6	0.2 - 1.3	6.7	2.2 - 13.0	20.8	6.4 - 42.1	11.6	3.8 - 25.2	0.0	0.0 - 0.0	11.6	4.1 - 27.9	0.22
3040101	5387	LEWIS FK CR, N PF	89.7	29.4	11 - 51	0.0	0.0 - 0.0	3.6	1.1 - 6.5	9.6	3.3 - 28.3	5.4	1.9 - 11.1	0.0	0.0 - 0.0	10.7	3.9 - 24.4	0.22
3040101	5388	LEWIS FK CR, S PR	94.9	21.8	6 - 48	0.0	0.0 - 0.0	3.6	1.0 - 6.8	5.5	1.3 - 14.1	3.1	0.9 - 6.4	0.0	0.0 - 0.0	9.6	2.6 - 24.4	0.22
3040101	5389	YADKIN R	13.6	19.5	7 - 33	0.0	0.0 - 0.0	4.2	1.3 - 7.5	3.9	1.2 - 8.7	2.1	0.7 - 4.8	0.0	0.0 - 0.0	9.3	3.2 - 18.9	0.22
3040101	5390	YADKIN R, STONY	98.8	19.1	7 - 38	0.0	0.0 - 0.0	3.6	1.3 - 8.4	3.6	1.1 - 8.5	3.1	1.2 - 7.7	0.0	0.0 - 0.0	8.8	3.4 - 17.4	0.22
3040101	5391	YADKIN R	70.8	31.0	12 - 70	0.0	0.0 - 0.0	3.0	1.0 - 6.6	9.2	3.2 - 23.9	6.1	2.1 - 15.6	0.0	0.0 - 0.0	12.7	4.4 - 30.8	0.22
3040101	5393	ELK CR	39.1	19.6	6 - 38	0.0	0.0 - 0.0	6.0	1.6 - 12.5	0.7	0.2 - 2.0	2.1	0.6 - 5.0	0.0	0.0 - 0.0	10.8	3.5 - 24.5	0.19
3040101	5394	*B	20.9	17.4	6 - 34	0.0	0.0 - 0.0	2.3	0.8 - 4.6	0.3	0.1 - 0.7	0.9	0.3 - 2.1	0.0	0.0 - 0.0	14.0	4.8 - 29.0	0.19
3040101	5395	YADKIN R	128.6	23.6	8 - 48	0.1	0.0 - 0.3	2.9	0.9 - 6.8	1.1	0.3 - 3.3	7.0	2.4 - 16.7	0.0	0.0 - 0.0	12.3	4.3 - 26.6	0.21
3040101	5396	BUFFALO CR	29.2	13.0	5 - 25	0.0	0.0 - 0.1	1.4	0.5 - 2.9	0.3	0.1 - 0.6	1.6	0.6 - 4.1	0.0	0.0 - 0.0	9.7	3.7 - 19.7	0.20
3040101	5397	*C	22.8	17.9	5 - 39	0.0	0.0 - 0.0	3.9	1.1 - 8.0	0.3	0.1 - 0.7	1.1	0.3 - 3.5	0.0	0.0 - 0.0	12.6	3.6 - 32.1	0.19
3040101	5398	BUFFALO CR	33.6	17.7	5 - 40	0.0	0.0 - 0.0	2.4	0.7 - 5.1	0.3	0.1 - 0.7	0.9	0.3 - 2.1	0.0	0.0 - 0.0	14.2	4.4 - 34.0	0.19
3040101	5399	YADKIN R	109.7	21.6	7 - 56	0.3	0.1 - 0.8	4.4	1.5 - 10.9	0.6	0.2 - 1.7	3.7	1.2 - 8.6	0.0	0.0 - 0.0	12.6	4.2 - 35.1	0.20
3040101	5400	WARRIOR CR	76.4	30.3	11 - 58	0.1	0.0 - 0.2	3.7	1.3 - 7.2	10.7	3.3 - 31.2	5.8	1.9 - 15.8	0.0	0.0 - 0.0	9.9	3.9 - 20.8	0.22
3040101	5401	MORAVIAN CR	65.0	36.6	12 - 93	0.0	0.0 - 0.0	8.3	2.5 - 20.1	11.4	3.4 - 35.4	6.2	1.9 - 15.0	0.0	0.0 - 0.0	10.7	3.5 - 28.3	0.41
3040101	5402	*D	49.1	46.6	14 - 93	0.0	0.0 - 0.0	9.2	2.7 - 20.3	17.5	5.2 - 47.1	9.5	2.6 - 23.3	0.0	0.0 - 0.0	10.4	3.3 - 22.3	0.42
3040101	5403	LITTLE ELKIN R	139.2	37.8	12 - 79	0.5	0.2 - 1.1	5.5	1.9 - 11.9	8.1	2.4 - 24.3	14.0	4.4 - 31.3	0.0	0.0 - 0.0	9.7	3.0 - 24.9	0.45
3040101	5404	DEEP CR	14.0	49.0	14 - 103	0.0	0.0 - 0.0	9.6	2.7 - 23.1	9.3	2.3 - 25.5	15.5	4.7 - 38.3	0.0	0.0 - 0.0	14.6	4.0 - 34.3	0.45
3040101	5405	N DEEP CR	113.8	60.6	18 - 157	17.1	5.4 - 41.3	8.9	2.5 - 22.7	9.3	2.4 - 26.5	16.1	5.0 - 38.2	0.0	0.0 - 0.0	9.4	3.2 - 24.0	0.43
3040101	5406	S DEEP CR	194.1	41.6	14 - 85	0.3	0.1 - 0.6	7.9	2.8 - 16.1	8.9	2.6 - 24.4	15.6	5.5 - 44.5	0.0	0.0 - 0.0	8.9	2.8 - 22.8	0.43
3040101	5407	DUTCHMANS CR	118.2	55.5	22 - 111	19.6	7.8 - 45.8	8.9	3.2 - 17.4	3.5	1.3 - 8.9	12.1	4.5 - 28.1	0.0	0.0 - 0.0	11.3	4.6 - 25.2	0.47
3040101	5408	CEDAR CR	60.2	42.8	13 - 90	0.0	0.0 - 0.0	7.0	2.1 - 14.6	5.5	1.5 - 14.4	18.0	5.6 - 45.0	0.0	0.0 - 0.0	12.3	4.0 - 37.0	0.44
3040101	5409	DUTCHMANS CR	155.9	39.1	15 - 103	0.1	0.0 - 0.2	5.6	1.9 - 14.2	6.0	1.9 - 18.1	17.8	6.4 - 49.8	0.0	0.0 - 0.0	9.7	3.4 - 27.0	0.44
3040101	5410	YADKIN R	77.0	61.8	24 - 142	11.3	4.2 - 27.0	19.7	7.5 - 47.3	7.8	3.0 - 22.3	12.7	4.4 - 36.3	0.0	0.0 - 0.0	10.3	4.2 - 23.8	0.43
3040102	5411	S YADKIN R	6.5	23.4	8 - 55	0.0	0.0 - 0.0	2.0	0.6 - 4.3	1.3	0.4 - 4.4	6.7	2.0 - 16.8	0.0	0.0 - 0.0	13.4	4.6 - 35.5	0.47
3040102	5412	S YADKIN R	11.5	29.8	14 - 59	0.0	0.0 - 0.0	5.8	2.4 - 13.6	2.4	0.8 - 6.5	9.4	4.0 - 22.8	0.0	0.0 - 0.0	12.2	5.5 - 26.9	0.47
3040102	5413	S YADKIN R	25.4	117.5	35 - 283	78.6	24.9 - 206.0	9.7	2.8 - 25.7	3.9	1.0 - 10.7	13.9	3.9 - 40.4	0.0	0.0 - 0.0	11.4	3.5 - 31.4	0.47
3040102	5414	BEAR CR	76.2	40.5	13 - 78	9.7	3.4 - 21.9	7.4	2.5 - 17.3	3.3	0.9 - 10.4	11.8	4.0 - 27.2	0.0	0.0 - 0.0	8.3	2.7 - 16.7	0.46
3040102	5415	S YADKIN R	5.2	37.2	12 - 83	0.0	0.0 - 0.0	2.2	0.7 - 5.2	4.4	1.3 - 11.7	19.7	6.0 - 49.0	0.0	0.0 - 0.0	10.8	3.4 - 25.4	0.46
3040102	5416	HUNTING CR	154.9	36.5	11 - 79	0.0	0.0 - 0.0	5.3	1.6 - 10.6	5.7	1.5 - 13.4	15.2	4.4 - 40.0	0.0	0.0 - 0.0	10.3	3.1 - 21.0	0.46
3040102	5417	S YADKIN R	19.0	37.5	13 - 71	0.0	0.0 - 0.0	2.8	1.1 - 5.9	3.6	1.1 - 10.1	19.0	6.3 - 43.4	0.0	0.0 - 0.0	12.1	4.7 - 29.0	0.46
3040102	5418	LITTLE CR	18.6	38.1	15 - 93	0.0	0.0 - 0.0	8.3	3.1 - 19.5	5.0	1.7 - 14.6	16.3	6.9 - 42.2	0.0	0.0 - 0.0	8.5	3.3 - 23.0	0.45
3040102	5419	S YADKIN R	47.3	31.0	13 - 53	0.0	0.0 - 0.0	4.6	1.8 - 9.0	4.1	1.5 - 11.1	13.0	5.0 - 30.9	0.0	0.0 - 0.0	9.2	3.5 - 20.2	0.45
3040102	5420	S YADKIN R	8.1	40.4	15 - 88	0.0	0.0 - 0.0	6.2	2.3 - 13.4	6.8	2.2 - 17.1	15.7	5.3 - 41.7	0.0	0.0 - 0.0	11.7	4.2 - 27.7	0.44
3040102	5421	S YADKIN R	69.0	47.9	19 - 110	0.0	0.0 - 0.0	6.1	2.5 - 13.0	9.5	3.2 - 29.2	21.6	9.0 - 55.9	0.0	0.0 - 0.0	10.7	4.2 - 27.8	0.44
3040102	5422	ROCKY R	11.7	42.4	15 - 93	0.0	0.0 - 0.0	8.4	2.5 - 20.0	7.5	2.2 - 22.5	17.1	5.7 - 41.3	0.0	0.0 - 0.0	9.5	3.5 - 24.8	0.42
3040102	5423	ROCKY R	159.8	31.1	11 - 83	0.0	0.0 - 0.0	4.5	1.5 - 11.8	5.6	1.7 - 15.7	10.8	3.5 - 31.5	0.0	0.0 - 0.0	10.1	3.2 - 24.9	0.41

8-digit HUC	ID	Name	Area	Catchment Yield		Point sources		Developed Land		Manure		Agricultural Land		Phosphate Mines		Soil parent rock		Frac
				kg/km^2	90% CI	kg/km^2	90% CI	kg/km^2	90% CI	kg/km^2	90% CI	kg/km^2	90% CI	kg/km^2	90% CI	kg/km^2	90% CI	
3040102	5424	OLIN CR	3.5	52.8	19 - 121	0.0	0.0 - 0.0	4.4	1.4 - 11.1	11.4	3.4 - 33.8	27.5	10.0 - 73.0	0.0	0.0 - 0.0	9.5	3.5 - 25.2	0.41
3040102	5425	OLIN CR	33.6	42.9	13 - 110	0.0	0.0 - 0.0	5.3	1.7 - 13.3	8.3	2.2 - 22.8	18.5	5.8 - 48.5	0.0	0.0 - 0.0	10.7	3.3 - 26.2	0.39
3040102	5426	PATTERSON CR	54.8	45.4	16 - 98	0.3	0.1 - 0.8	5.9	2.0 - 12.2	8.6	2.5 - 22.5	19.6	6.3 - 45.7	0.0	0.0 - 0.0	10.9	3.8 - 28.2	0.39
3040102	5427	S YADKIN R	52.0	37.9	12 - 88	0.0	0.0 - 0.0	7.7	2.4 - 15.8	6.5	1.8 - 17.1	14.5	4.3 - 43.5	0.0	0.0 - 0.0	9.2	3.1 - 21.9	0.42
3040102	5428	SNOW CR	76.6	38.9	13 - 70	0.0	0.0 - 0.0	4.8	1.5 - 9.8	9.3	2.6 - 25.1	14.7	5.0 - 38.8	0.0	0.0 - 0.0	10.2	3.5 - 21.1	0.40
3040102	5429	S YADKIN R	64.9	36.5	12 - 85	0.0	0.0 - 0.0	6.0	1.8 - 14.4	8.7	2.4 - 34.2	12.6	4.3 - 36.3	0.0	0.0 - 0.0	9.2	3.3 - 24.8	0.40
3040102	5430	S YADKIN R	93.5	37.9	12 - 71	0.0	0.0 - 0.0	4.4	1.4 - 9.0	12.1	3.5 - 34.0	11.3	3.7 - 26.5	0.0	0.0 - 0.0	10.0	3.3 - 25.2	0.35
3040102	5431	WALLACE CR	43.8	52.9	19 - 117	0.0	0.0 - 0.0	9.4	3.2 - 18.9	17.4	4.8 - 50.7	15.7	5.4 - 35.7	0.0	0.0 - 0.0	10.3	3.4 - 24.2	0.35
3040102	5432	THIRD CR	14.9	36.0	11 - 89	0.0	0.0 - 0.0	4.0	1.3 - 11.9	3.1	0.9 - 10.4	16.5	4.9 - 54.9	0.0	0.0 - 0.0	12.5	4.0 - 35.8	0.47
3040102	5433	FOURTH CR	148.9	93.6	28 - 186	42.7	13.0 - 89.4	19.0	5.3 - 40.7	4.8	1.2 - 14.4	15.5	4.7 - 41.1	0.0	0.0 - 0.0	11.5	3.6 - 29.9	0.46
3040102	5434	*B	39.0	48.0	16 - 88	0.0	0.0 - 0.0	9.1	3.1 - 20.5	8.7	2.4 - 24.1	20.0	6.9 - 52.1	0.0	0.0 - 0.0	10.3	3.5 - 21.1	0.41
3040102	5435	*C	28.8	60.6	21 - 132	0.0	0.0 - 0.0	30.1	10.2 - 62.5	6.4	2.0 - 17.7	14.2	5.2 - 34.7	0.0	0.0 - 0.0	9.8	3.6 - 24.6	0.41
3040102	5436	THIRD CR	262.2	74.1	26 - 181	29.7	11.3 - 76.4	12.8	4.5 - 28.3	6.5	2.0 - 15.8	14.7	4.6 - 38.8	0.0	0.0 - 0.0	10.4	3.5 - 25.8	0.46
3040102	5437	SECOND CR	67.3	49.5	16 - 110	16.8	5.3 - 35.4	5.1	1.7 - 11.3	2.7	0.7 - 8.1	14.2	5.2 - 34.8	0.0	0.0 - 0.0	10.8	3.4 - 25.9	0.47
3040102	5438	SECOND CR	18.3	47.0	16 - 88	1.4	0.5 - 2.9	7.1	2.1 - 16.6	4.0	1.2 - 12.1	22.7	7.8 - 54.6	0.0	0.0 - 0.0	11.9	3.9 - 27.9	0.45
3040102	5439	BEAVERDAM CR	26.1	38.9	10 - 80	0.0	0.0 - 0.0	6.3	1.7 - 14.7	3.6	0.9 - 10.8	19.0	5.6 - 51.5	0.0	0.0 - 0.0	10.0	2.7 - 22.4	0.43
3040102	5440	WITHROW CR	84.1	39.0	11 - 80	0.0	0.0 - 0.0	6.1	1.6 - 13.9	6.1	1.5 - 18.6	17.2	5.5 - 44.9	0.0	0.0 - 0.0	9.5	2.8 - 20.7	0.43
3040102	5441	CARNELL CR	14.4	39.2	15 - 74	0.0	0.0 - 0.0	3.4	1.2 - 6.3	3.8	1.1 - 10.6	20.3	7.1 - 50.2	0.0	0.0 - 0.0	11.7	4.0 - 28.7	0.45
3040102	5442	SILLS CR	3.4	41.3	14 - 89	0.0	0.0 - 0.0	4.6	1.4 - 9.8	3.8	1.1 - 11.8	21.9	7.3 - 53.1	0.0	0.0 - 0.0	10.9	3.4 - 28.5	0.44
3040102	5443	BACK CR	48.8	44.9	13 - 107	0.0	0.0 - 0.0	11.8	3.6 - 27.2	5.0	1.4 - 18.1	18.6	5.5 - 45.7	0.0	0.0 - 0.0	9.5	3.2 - 24.8	0.44
3040102	5444	SILLS CR	46.4	42.1	13 - 89	0.0	0.0 - 0.0	4.1	1.3 - 9.4	4.1	1.3 - 9.3	22.5	7.8 - 50.7	0.0	0.0 - 0.0	11.3	3.2 - 21.5	0.44
3040102	5445	CARNELL CR	54.3	41.1	15 - 86	0.0	0.0 - 0.0	3.9	1.2 - 8.1	3.7	1.2 - 11.8	20.9	7.0 - 51.1	0.0	0.0 - 0.0	12.6	4.6 - 27.0	0.44
3040102	5446	N HUNTING CR	140.0	50.6	20 - 119	0.3	0.1 - 0.5	7.5	3.0 - 14.6	14.2	4.6 - 38.7	18.3	6.6 - 45.8	0.0	0.0 - 0.0	10.3	3.8 - 23.1	0.42
3040102	5447	HUNTING CR	87.4	47.0	18 - 113	0.0	0.0 - 0.0	6.7	2.4 - 16.9	11.1	3.7 - 30.7	17.0	6.0 - 45.3	0.0	0.0 - 0.0	12.3	4.5 - 29.0	0.42
3040102	5448	LITTLE HUNTING (	49.2	39.2	13 - 89	0.0	0.0 - 0.0	3.0	1.0 - 6.9	15.8	4.6 - 51.6	8.8	3.2 - 22.9	0.0	0.0 - 0.0	11.6	4.5 - 27.7	0.38
3040102	5449	HUNTING CR	98.5	31.2	8 - 68	0.0	0.0 - 0.0	3.0	0.7 - 6.0	11.1	2.9 - 27.3	6.3	2.0 - 16.8	0.0	0.0 - 0.0	10.9	3.4 - 25.2	0.38
3040102	5450	SAWMILL BR	75.4	45.3	17 - 95	0.9	0.3 - 2.3	13.8	4.9 - 29.7	6.6	2.2 - 20.1	15.0	5.1 - 45.4	0.0	0.0 - 0.0	8.9	3.1 - 20.9	0.44
3040103	5451	PEE DEE R	1.8	20.4	7 - 43	0.0	0.0 - 0.0	2.9	1.0 - 6.1	0.2	0.1 - 0.4	0.0	0.0 - 0.0	0.0	0.0 - 0.0	17.3	5.7 - 37.4	0.71
3040103	5452	UWHARRIE R	429.3	33.4	10 - 91	0.0	0.0 - 0.1	2.3	0.7 - 5.7	8.8	2.4 - 30.5	9.1	3.0 - 25.9	0.0	0.0 - 0.0	13.3	3.8 - 37.2	0.71
3040103	5453	BACK CR	35.6	44.4	19 - 120	0.0	0.0 - 0.0	7.5	3.0 - 19.4	11.2	4.1 - 37.0	11.7	4.3 - 30.3	0.0	0.0 - 0.0	14.0	5.3 - 40.0	0.62
3040103	5454	BACK CR	102.2	52.3	17 - 97	0.0	0.0 - 0.0	16.8	5.6 - 32.6	10.4	3.6 - 20.9	10.9	3.7 - 24.8	0.0	0.0 - 0.0	14.3	4.8 - 33.9	0.60
3040103	5455	CARAWAY CR	120.0	42.5	14 - 80	0.1	0.0 - 0.2	7.6	2.4 - 16.3	11.8	3.6 - 36.3	11.8	3.8 - 31.4	0.0	0.0 - 0.0	11.2	3.9 - 29.3	0.60
3040103	5456	UWHARRIE R	104.0	38.1	12 - 80	0.2	0.1 - 0.5	3.4	1.0 - 7.8	10.0	2.7 - 29.2	10.8	3.6 - 26.3	0.0	0.0 - 0.0	13.8	4.8 - 33.3	0.62
3040103	5457	UWHARRIE R	108.2	48.3	19 - 119	0.0	0.0 - 0.0	16.2	6.1 - 40.3	10.5	3.8 - 34.9	10.8	4.3 - 28.5	0.0	0.0 - 0.0	10.8	4.0 - 31.2	0.57
3040103	5458	LITTLE UWHARRII	117.4	36.5	11 - 91	0.0	0.0 - 0.0	10.1	2.9 - 27.2	5.7	1.5 - 15.6	10.7	3.1 - 31.3	0.0	0.0 - 0.0	9.9	3.0 - 26.7	0.57
3040103	5459	PEE DEE R	24.4	8.8	3 - 22	0.0	0.0 - 0.0	0.6	0.2 - 1.3	0.0	0.0 - 0.1	0.0	0.0 - 0.0	0.0	0.0 - 0.0	8.2	2.3 - 19.8	0.71
3040103	5460	FLAT SWAMP CR	44.2	19.8	7 - 47	0.0	0.0 - 0.0	3.8	1.1 - 8.5	1.8	0.6 - 5.3	6.1	1.9 - 14.9	0.0	0.0 - 0.0	8.1	2.7 - 22.0	0.63
3040103	5461	FOURMILE CR	40.8	25.5	10 - 67	0.0	0.0 - 0.0	3.8	1.2 - 8.3	2.7	1.0 - 6.5	10.3	3.5 - 30.1	0.0	0.0 - 0.0	8.7	2.8 - 25.2	0.63
3040103	5462	ABBOTTS CR	2.8	30.1	11 - 57	0.0	0.0 - 0.0	8.1	2.9 - 18.2	2.1	0.6 - 5.6	8.9	2.8 - 21.3	0.0	0.0 - 0.0	10.9	3.6 - 26.0	0.46

8-digit HUC	ID	Name	Area	Catchment Yield		Point sources		Developed Land		Manure		Agricultural Land		Phosphate Mines		Soil parent rock		Frac
				kg/km^2	90% CI	kg/km^2	90% CI	kg/km^2	90% CI	kg/km^2	90% CI	kg/km^2	90% CI	kg/km^2	90% CI	kg/km^2	90% CI	
3040103	5463	ABBOTTS CR, RICI	1.6	24.1	8 - 46	0.0	0.0 - 0.0	4.3	1.4 - 9.3	1.7	0.5 - 4.7	7.0	2.3 - 17.5	0.0	0.0 - 0.0	11.0	3.8 - 23.6	0.45
3040103	5464	HAMBY CR	76.5	138.7	51 - 349	93.6	34.8 - 246.7	29.6	10.0 - 74.8	1.7	0.5 - 5.8	5.7	2.0 - 14.9	0.0	0.0 - 0.0	8.1	2.8 - 20.5	0.45
3040103	5465	ABBOTTS CR, RICI	127.5	86.0	31 - 171	39.4	13.5 - 93.0	31.7	11.7 - 58.4	1.5	0.5 - 4.6	5.6	1.8 - 13.7	0.0	0.0 - 0.0	7.8	2.5 - 19.3	0.45
3040103	5466	ABBOTTS CR	184.3	6.4	2 - 16	0.1	0.0 - 0.3	2.2	0.6 - 5.7	0.5	0.1 - 1.3	2.0	0.5 - 5.6	0.0	0.0 - 0.0	1.6	0.4 - 3.6	0.45
3040103	5467	YADKIN R	36.6	29.8	9 - 54	0.0	0.0 - 0.0	5.2	1.5 - 10.1	2.5	0.7 - 6.1	12.1	3.8 - 25.4	0.0	0.0 - 0.0	10.0	3.3 - 23.4	0.48
3040103	5468	GRANTS CR	176.3	133.2	41 - 286	85.9	27.2 - 192.1	25.3	8.4 - 55.1	1.9	0.6 - 6.1	10.2	3.1 - 25.5	0.0	0.0 - 0.0	9.9	3.1 - 24.6	0.48
3040103	5469	*A	47.7	37.8	15 - 74	0.4	0.1 - 0.9	14.2	5.5 - 28.9	2.8	0.9 - 8.0	11.1	4.1 - 26.7	0.0	0.0 - 0.0	9.3	3.6 - 23.6	0.46
3040104	5470	PEE DEE R	76.3	16.9	7 - 37	0.0	0.0 - 0.0	2.2	0.8 - 5.0	2.5	0.9 - 6.3	1.6	0.5 - 4.1	0.0	0.0 - 0.0	10.6	4.3 - 24.8	0.83
3040104	5471	BIG MOUNTAIN CI	26.6	9.5	3 - 16	0.0	0.0 - 0.0	2.2	0.7 - 4.1	1.2	0.3 - 3.1	0.7	0.2 - 1.7	0.0	0.0 - 0.0	5.5	1.8 - 11.4	0.80
3040104	5472	LITTLE MOUNTAII	63.3	34.3	11 - 75	7.3	2.4 - 17.5	7.8	2.5 - 18.4	7.6	2.2 - 20.6	4.6	1.5 - 11.9	0.0	0.0 - 0.0	6.9	2.1 - 16.0	0.77
3040104	5473	BIG MOUNTAIN CI	103.4	20.2	6 - 45	0.0	0.0 - 0.0	3.5	1.1 - 8.1	5.2	1.7 - 15.7	3.1	0.9 - 7.8	0.0	0.0 - 0.0	8.3	2.8 - 20.9	0.77
3040104	5474	PEE DEE R	60.9	20.7	7 - 43	0.0	0.0 - 0.0	1.8	0.6 - 4.2	3.2	1.0 - 8.4	2.2	0.8 - 5.7	0.0	0.0 - 0.0	13.4	4.7 - 33.3	0.80
3040104	5475	LITTLE R	27.4	21.7	8 - 49	0.0	0.0 - 0.0	1.9	0.6 - 4.4	6.2	1.9 - 15.5	3.9	1.2 - 11.2	0.0	0.0 - 0.0	9.6	3.2 - 25.6	0.80
3040104	5476	LITTLE R	34.8	33.5	14 - 69	0.0	0.0 - 0.0	3.2	1.3 - 7.4	9.5	3.2 - 25.7	6.2	2.6 - 15.2	0.0	0.0 - 0.0	14.5	6.7 - 34.3	0.79
3040104	5477	CHEEK CR	87.8	37.3	12 - 84	0.0	0.0 - 0.0	2.5	0.8 - 5.5	11.7	3.5 - 31.2	6.7	2.4 - 17.0	0.0	0.0 - 0.0	16.3	5.6 - 43.6	0.78
3040104	5479	LITTLE R	178.0	41.5	14 - 82	0.0	0.0 - 0.0	7.5	2.5 - 14.9	11.3	3.3 - 36.6	9.8	3.0 - 21.6	0.0	0.0 - 0.0	12.9	4.2 - 31.3	0.69
3040104	5480	LITTLE R, W FK	94.9	38.4	14 - 78	0.0	0.0 - 0.0	2.9	1.1 - 5.9	13.1	4.2 - 37.0	9.2	3.6 - 19.6	0.0	0.0 - 0.0	13.1	4.0 - 32.5	0.69
3040104	5481	HAMER CR	58.4	55.5	17 - 111	0.0	0.0 - 0.0	4.6	1.6 - 9.7	21.7	6.1 - 57.8	13.4	5.2 - 33.3	0.0	0.0 - 0.0	15.7	5.5 - 39.7	0.79
3040104	5482	PEE DEE R	97.0	68.9	20 - 153	0.0	0.0 - 0.0	3.3	1.1 - 7.2	28.9	8.1 - 92.5	19.6	6.1 - 49.0	0.0	0.0 - 0.0	17.0	4.9 - 45.9	0.80
3040104	5483	PEE DEE R	57.4	94.5	29 - 207	1.3	0.5 - 3.3	3.3	1.1 - 6.9	40.6	11.8 - 119.4	31.3	9.3 - 75.8	0.0	0.0 - 0.0	18.1	5.8 - 47.0	0.79
3040104	5484	PEE DEE R	22.7	35.9	12 - 70	0.0	0.0 - 0.0	1.2	0.4 - 2.2	5.9	1.7 - 15.8	11.2	3.4 - 29.9	0.0	0.0 - 0.0	17.7	5.6 - 43.2	0.79
3040104	5485	BROWN CR	469.7	50.4	20 - 127	0.0	0.0 - 0.0	5.1	2.0 - 14.5	16.5	5.4 - 54.5	14.4	5.6 - 37.7	0.0	0.0 - 0.0	14.4	5.5 - 43.8	0.79
3040105	5486	ROCKY R	94.6	73.5	27 - 156	5.3	2.0 - 11.2	5.5	2.0 - 12.9	17.5	5.5 - 49.1	28.4	9.8 - 68.9	0.0	0.0 - 0.0	16.7	5.6 - 40.4	0.79
3040105	5487	ROCKY R	77.3	52.1	16 - 122	0.0	0.0 - 0.0	4.9	1.4 - 10.9	17.4	4.6 - 43.6	18.2	5.6 - 46.0	0.0	0.0 - 0.0	11.6	3.5 - 28.8	0.77
3040105	5488	ROCKY R	22.7	22.3	7 - 39	0.0	0.0 - 0.0	5.1	1.5 - 9.7	4.7	1.5 - 12.2	6.5	2.3 - 15.3	0.0	0.0 - 0.0	6.1	2.0 - 13.4	0.77
3040105	5489	LONG CR	16.5	37.3	13 - 68	0.7	0.2 - 1.3	6.2	2.1 - 13.8	6.6	2.0 - 19.0	15.1	5.5 - 34.4	0.0	0.0 - 0.0	8.8	2.8 - 18.3	0.76
3040105	5490	LONG CR	32.8	63.4	18 - 127	11.9	3.5 - 28.7	5.9	1.6 - 13.5	10.4	2.9 - 31.3	23.1	6.2 - 58.1	0.0	0.0 - 0.0	12.1	3.9 - 29.0	0.75
3040105	5491	LONG CR	193.0	173.7	53 - 383	119.9	35.4 - 250.3	15.3	4.0 - 33.1	8.0	2.3 - 19.2	18.3	4.9 - 49.2	0.0	0.0 - 0.0	12.2	3.8 - 27.2	0.73
3040105	5492	LITTLE BEAR CR	36.0	63.5	22 - 143	0.0	0.0 - 0.0	6.4	2.3 - 15.8	13.4	3.7 - 39.0	30.4	11.1 - 75.7	0.0	0.0 - 0.0	13.4	4.7 - 33.6	0.73
3040105	5493	BEAR CR	5.7	35.5	10 - 77	0.0	0.0 - 0.0	4.1	1.3 - 9.0	6.9	1.9 - 19.9	15.2	4.3 - 40.2	0.0	0.0 - 0.0	9.2	2.4 - 23.5	0.75
3040105	5494	BEAR CR	186.7	51.6	18 - 113	0.0	0.0 - 0.0	5.0	1.8 - 11.2	9.6	3.1 - 30.7	23.8	9.0 - 56.5	0.0	0.0 - 0.0	13.0	4.3 - 33.6	0.73
3040105	5495	STONY RUN	47.8	48.8	14 - 90	0.0	0.0 - 0.0	8.6	2.4 - 17.0	9.0	2.3 - 24.3	20.2	6.4 - 41.5	0.0	0.0 - 0.0	11.0	3.3 - 25.8	0.73
3040105	5496	ROCKY R	220.5	34.7	12 - 60	0.0	0.0 - 0.0	6.7	2.4 - 12.9	8.3	2.6 - 21.0	12.5	4.6 - 26.5	0.0	0.0 - 0.0	7.3	2.5 - 17.7	0.76
3040105	5497	ROCKY R	15.7	32.4	12 - 69	0.0	0.0 - 0.0	3.1	1.3 - 7.1	8.7	3.2 - 23.6	13.3	5.5 - 36.9	0.0	0.0 - 0.0	7.2	2.4 - 16.7	0.73
3040105	5499	ROCKY R	139.1	35.8	12 - 65	0.5	0.2 - 0.9	6.7	2.1 - 12.6	4.4	1.3 - 11.3	13.7	4.3 - 34.5	0.0	0.0 - 0.0	10.5	3.5 - 24.4	0.72
3040105	5500	DUTCH BUFFALO (	255.5	38.5	13 - 75	0.0	0.0 - 0.1	5.8	1.7 - 11.4	4.5	1.2 - 11.5	16.5	5.7 - 37.2	0.0	0.0 - 0.0	11.7	3.6 - 23.2	0.70
3040105	5501	ROCKY R	38.4	1531.6	564 - 3701	1498.7	547.4 - 3635.4	6.0	2.0 - 13.5	3.2	1.0 - 9.2	10.7	4.0 - 29.7	0.0	0.0 - 0.0	13.1	5.0 - 31.9	0.70
3040105	5502	IRISH BUFFALO CI	1.2	40.4	13 - 99	0.0	0.0 - 0.0	8.9	2.9 - 22.2	4.3	1.2 - 15.9	13.2	4.3 - 42.8	0.0	0.0 - 0.0	14.0	5.4 - 38.4	0.69
3040105	5503	COLDWATER CR	166.9	47.0	13 - 105	0.0	0.0 - 0.0	28.4	8.6 - 65.2	1.9	0.5 - 5.5	6.7	2.1 - 17.1	0.0	0.0 - 0.0	9.9	3.3 - 23.8	0.68

8-digit HUC	ID	Name	Area	Catchment Yield		Point sources		Developed Land		Manure		Agricultural Land		Phosphate Mines		Soil parent rock		Frac
				kg/km^2	90% CI	kg/km^2	90% CI	kg/km^2	90% CI	kg/km^2	90% CI	kg/km^2	90% CI	kg/km^2	90% CI	kg/km^2	90% CI	
3040105	5504	IRISH BUFFALO CI	118.4	66.6	25 - 129	0.0	0.0 - 0.0	48.3	18.0 - 93.6	1.4	0.4 - 3.3	5.6	1.8 - 11.5	0.0	0.0 - 0.0	11.3	3.7 - 24.7	0.68
3040105	5505	ROCKY R	8.1	37.4	12 - 67	0.0	0.0 - 0.0	14.5	5.0 - 28.7	2.4	0.8 - 6.7	7.0	2.3 - 19.5	0.0	0.0 - 0.0	13.6	4.5 - 30.8	0.69
3040105	5506	ROCKY R	5.7	31.4	11 - 61	0.0	0.0 - 0.0	7.1	2.2 - 14.3	2.6	0.8 - 8.2	7.4	2.7 - 20.1	0.0	0.0 - 0.0	14.4	4.6 - 32.2	0.68
3040105	5508	CODDLE CR	204.0	44.1	13 - 74	0.0	0.0 - 0.0	16.6	5.6 - 30.6	3.9	1.1 - 10.1	13.2	4.1 - 31.8	0.0	0.0 - 0.0	10.5	3.3 - 23.3	0.67
3040105	5509	ROCKY R	12.2	70.5	28 - 120	0.0	0.0 - 0.0	41.8	16.1 - 78.8	3.4	1.2 - 7.5	10.6	3.7 - 26.0	0.0	0.0 - 0.0	14.7	6.3 - 32.0	0.67
3040105	5510	ROCKY R	32.7	67.7	22 - 162	4.8	1.6 - 12.0	39.6	13.3 - 96.1	2.6	0.8 - 7.7	8.6	3.1 - 22.1	0.0	0.0 - 0.0	12.1	4.2 - 30.0	0.65
3040105	5511	ROCKY R	32.1	34.1	12 - 70	0.3	0.1 - 0.6	9.0	3.1 - 19.0	3.4	1.1 - 9.2	10.7	3.7 - 26.1	0.0	0.0 - 0.0	10.8	4.0 - 26.3	0.61
3040105	5512	ROCKY R	37.4	276.5	108 - 734	237.1	93.6 - 636.9	16.2	5.1 - 40.6	4.3	1.5 - 13.7	10.4	3.4 - 30.7	0.0	0.0 - 0.0	8.6	3.1 - 26.4	0.55
3040105	5513	ROCKY R, W BR	56.7	38.1	16 - 104	3.0	1.2 - 8.0	14.0	5.5 - 37.2	2.8	0.9 - 8.8	9.7	3.5 - 29.3	0.0	0.0 - 0.0	8.6	3.3 - 24.6	0.55
3040105	5514	CLARKE CR	71.6	39.0	11 - 71	0.6	0.2 - 1.4	19.5	5.8 - 33.6	1.2	0.3 - 3.1	8.3	2.3 - 19.4	0.0	0.0 - 0.0	9.3	2.6 - 18.1	0.61
3040105	5515	MALLARD CR	108.1	238.6	77 - 566	176.0	59.0 - 413.2	48.8	13.4 - 101.8	0.5	0.1 - 1.5	3.3	1.1 - 9.0	0.0	0.0 - 0.0	10.0	3.4 - 19.6	0.65
3040105	5516	BACK CR	40.1	58.1	17 - 120	0.6	0.2 - 1.5	33.7	10.4 - 74.4	2.2	0.6 - 6.6	9.7	2.8 - 23.2	0.0	0.0 - 0.0	11.8	3.9 - 27.5	0.67
3040105	5517	REEDY CR	112.4	51.2	18 - 138	10.5	3.3 - 28.0	21.6	7.9 - 58.1	1.7	0.5 - 5.0	6.9	2.1 - 19.3	0.0	0.0 - 0.0	10.5	3.5 - 29.5	0.68
3040105	5518	CLEAR CR	62.9	36.9	11 - 91	0.4	0.1 - 0.9	10.3	2.7 - 24.4	3.8	1.0 - 11.5	12.1	3.9 - 28.6	0.0	0.0 - 0.0	10.3	3.0 - 26.8	0.72
3040105	5519	GOOSE CR	148.0	73.9	23 - 140	10.3	3.5 - 20.3	19.1	5.7 - 40.0	14.7	3.8 - 35.1	17.0	5.7 - 41.2	0.0	0.0 - 0.0	12.9	4.2 - 27.2	0.73
3040105	5520	CROOKED CR	90.2	145.1	45 - 337	63.5	22.2 - 158.8	16.7	5.0 - 39.9	25.9	7.1 - 67.7	25.6	7.9 - 63.4	0.0	0.0 - 0.0	13.3	4.1 - 37.0	0.73
3040105	5521	RICHARDSON CR	613.1	141.3	52 - 249	74.5	26.5 - 168.7	11.1	4.0 - 22.0	22.6	7.6 - 72.7	22.0	8.0 - 46.9	0.0	0.0 - 0.0	11.1	3.7 - 27.9	0.77
3040105	5522	LANES CR	136.1	72.9	25 - 147	0.0	0.0 - 0.0	6.9	2.1 - 14.6	27.6	8.7 - 64.6	24.0	7.1 - 54.0	0.0	0.0 - 0.0	14.3	5.0 - 37.1	0.77
3040105	5523	BEAVERDAM CR	48.9	89.6	31 - 186	0.0	0.0 - 0.0	4.4	1.5 - 10.8	35.7	10.2 - 101.3	34.9	12.0 - 91.5	0.0	0.0 - 0.0	14.6	5.1 - 35.8	0.66
3040105	5524	LANES CR	169.9	68.6	21 - 165	0.0	0.0 - 0.0	4.2	1.5 - 9.8	26.6	8.0 - 74.3	26.4	9.3 - 65.3	0.0	0.0 - 0.0	11.4	3.5 - 28.0	0.66
3040201	5525	PEE DEE R	21.4	51.9	21 - 104	0.0	0.0 - 0.0	12.8	4.4 - 25.7	0.4	0.1 - 0.8	2.1	0.8 - 4.6	0.0	0.0 - 0.0	36.7	14.4 - 74.3	1.00
3040201	5526	CATFISH CR	573.7	31.3	8 - 55	0.0	0.0 - 0.0	4.5	1.3 - 9.0	2.7	0.8 - 6.8	12.9	3.3 - 32.7	0.0	0.0 - 0.0	11.2	3.1 - 25.5	0.88
3040201	5527	PEE DEE R	66.2	24.9	9 - 46	3.2	1.1 - 7.1	1.9	0.6 - 4.2	0.9	0.3 - 2.4	10.1	3.4 - 24.8	0.0	0.0 - 0.0	8.7	2.7 - 20.1	0.88
3040201	5528	PEE DEE R	165.5	218.7	75 - 535	187.0	65.6 - 464.6	2.0	0.7 - 4.6	3.0	0.8 - 8.1	8.9	2.5 - 22.9	0.0	0.0 - 0.0	17.8	5.8 - 46.1	0.87
3040201	5529	PEE DEE R	217.1	27.0	10 - 53	0.0	0.0 - 0.0	2.3	0.8 - 5.3	1.4	0.4 - 3.5	11.2	4.2 - 25.2	0.0	0.0 - 0.0	12.2	4.4 - 28.4	0.87
3040201	5530	THREE CR	263.9	40.0	14 - 116	0.9	0.3 - 2.7	4.6	1.6 - 11.7	2.8	0.9 - 8.5	20.2	7.1 - 59.6	0.0	0.0 - 0.0	11.6	4.1 - 34.7	0.86
3040201	5531	PEE DEE R	287.8	22.8	9 - 45	0.0	0.0 - 0.0	2.1	0.8 - 4.1	0.8	0.2 - 1.9	8.8	3.3 - 20.8	0.0	0.0 - 0.0	11.1	4.1 - 26.8	0.86
3040201	5532	PEE DEE R	35.2	20.1	7 - 41	0.0	0.0 - 0.0	2.8	1.0 - 6.2	1.2	0.4 - 3.4	5.4	1.8 - 13.3	0.0	0.0 - 0.0	10.8	3.9 - 25.6	0.85
3040201	5533	CROOKED CR	216.6	41.8	15 - 72	12.7	4.9 - 24.3	8.1	2.5 - 17.3	2.2	0.7 - 5.3	9.8	3.4 - 26.6	0.0	0.0 - 0.0	9.0	3.1 - 18.5	0.85
3040201	5534	PEE DEE R	14.9	1085.7	320 - 2647	1053.2	309.2 - 2619.2	3.3	1.0 - 9.1	2.1	0.6 - 5.9	10.9	3.3 - 31.6	0.0	0.0 - 0.0	16.2	5.9 - 43.5	0.85
3040201	5535	NAKED CR	83.0	25.5	10 - 45	0.0	0.0 - 0.0	4.3	1.6 - 8.7	1.6	0.6 - 3.7	10.8	4.0 - 23.5	0.0	0.0 - 0.0	8.8	3.6 - 19.0	0.85
3040201	5536	PEE DEE R	88.7	23.4	8 - 41	0.0	0.0 - 0.0	3.2	1.1 - 6.3	1.6	0.5 - 3.9	6.7	2.4 - 15.7	0.0	0.0 - 0.0	12.0	3.9 - 26.5	0.85
3040201	5537	WOLF CR	87.8	22.1	9 - 43	0.0	0.0 - 0.0	4.3	1.6 - 8.3	0.9	0.3 - 2.5	5.4	1.9 - 13.1	0.0	0.0 - 0.0	11.5	4.1 - 26.0	0.85
3040201	5539	PEE DEE R	11.6	301.3	107 - 620	229.4	79.2 - 490.3	26.3	8.9 - 55.9	3.8	1.1 - 10.2	18.6	6.3 - 44.8	0.0	0.0 - 0.0	23.2	7.7 - 52.8	0.85
3040201	5540	MARKS CR	119.1	28.6	10 - 62	1.1	0.3 - 2.5	13.1	4.6 - 27.6	3.8	1.1 - 10.5	2.5	0.7 - 5.9	0.0	0.0 - 0.0	8.1	2.6 - 19.1	0.84
3040201	5541	PEE DEE R	93.0	26.1	10 - 44	0.0	0.0 - 0.0	5.2	1.9 - 9.5	6.1	2.0 - 13.1	5.0	2.0 - 11.1	0.0	0.0 - 0.0	9.8	3.4 - 21.1	0.84
3040201	5542	PEE DEE R	97.3	48.5	19 - 122	24.2	9.2 - 66.2	6.4	2.3 - 15.0	5.5	1.8 - 14.5	3.4	1.2 - 9.2	0.0	0.0 - 0.0	9.1	3.3 - 25.9	0.84
3040201	5543	HITCHCOCK CR	153.3	37.4	14 - 76	0.0	0.0 - 0.0	23.7	8.6 - 47.8	3.4	1.1 - 9.7	2.0	0.7 - 5.7	0.0	0.0 - 0.0	8.3	3.0 - 20.8	0.84
3040201	5544	CHOCK CR	114.6	22.8	7 - 53	0.0	0.0 - 0.0	9.2	3.0 - 22.0	3.6	1.1 - 9.5	2.2	0.6 - 5.7	0.0	0.0 - 0.0	7.8	2.4 - 19.0	0.73

8-digit HUC	ID	Name	Area	Catchment Yield		Point sources		Developed Land		Manure		Agricultural Land		Phosphate Mines		Soil parent rock		Frac
				kg/km^2	90% CI	kg/km^2	90% CI	kg/km^2	90% CI	kg/km^2	90% CI	kg/km^2	90% CI	kg/km^2	90% CI	kg/km^2	90% CI	
3040201	5545	HITCHCOCK CR	101.2	7.9	3 - 17	0.0	0.0 - 0.0	2.7	0.9 - 6.4	1.5	0.4 - 4.5	0.9	0.3 - 2.2	0.0	0.0 - 0.0	2.9	0.9 - 5.9	0.73
3040201	5546	PEE DEE R	43.1	16.8	6 - 34	0.0	0.0 - 0.0	7.5	2.5 - 14.8	1.4	0.4 - 3.2	0.7	0.2 - 1.9	0.0	0.0 - 0.0	7.2	2.4 - 17.7	0.84
3040201	5547	JONES CR	51.8	18.3	5 - 32	0.0	0.0 - 0.0	5.0	1.4 - 9.4	2.6	0.7 - 7.0	2.0	0.5 - 4.2	0.0	0.0 - 0.0	8.7	2.1 - 18.4	0.84
3040201	5548	JONES CR, N FK	96.6	43.0	14 - 76	0.0	0.0 - 0.0	12.2	4.2 - 21.4	9.4	2.9 - 22.4	7.6	3.0 - 19.4	0.0	0.0 - 0.0	13.8	5.1 - 29.5	0.76
3040201	5549	JONES CR, S FK	106.7	65.6	22 - 156	42.0	14.0 - 101.4	4.1	1.1 - 9.5	7.4	2.1 - 22.6	5.8	1.7 - 15.9	0.0	0.0 - 0.0	6.3	2.2 - 15.8	0.76
3040201	5550	THOMPSON CR	11.2	38.3	13 - 80	0.0	0.0 - 0.0	1.0	0.3 - 1.8	3.1	0.8 - 7.7	12.6	4.0 - 29.4	0.0	0.0 - 0.0	21.5	7.3 - 47.1	0.85
3040201	5551	THOMPSON CR	57.0	28.6	9 - 52	0.0	0.0 - 0.0	6.6	2.1 - 12.9	2.0	0.6 - 4.0	7.4	2.2 - 17.1	0.0	0.0 - 0.0	12.6	3.8 - 26.5	0.84
3040201	5552	THOMPSON CR	140.6	29.2	8 - 49	2.3	0.7 - 4.2	7.7	2.1 - 16.4	1.9	0.5 - 5.5	5.6	1.8 - 12.5	0.0	0.0 - 0.0	11.7	3.2 - 26.0	0.81
3040201	5553	THOMPSON CR	54.5	37.2	13 - 71	0.0	0.0 - 0.0	4.7	1.7 - 9.7	8.6	2.7 - 20.6	12.8	4.6 - 29.9	0.0	0.0 - 0.0	11.1	3.6 - 25.8	0.75
3040201	5554	DEADFALL CR	78.7	18.9	7 - 39	0.0	0.0 - 0.0	2.5	0.9 - 5.5	4.3	1.4 - 12.8	3.3	1.3 - 8.5	0.0	0.0 - 0.0	8.9	3.3 - 22.4	0.69
3040201	5555	THOMPSON CR	108.6	39.1	15 - 110	0.0	0.0 - 0.0	5.2	1.9 - 12.2	7.1	2.4 - 26.6	13.0	5.2 - 41.8	0.0	0.0 - 0.0	13.9	5.5 - 42.2	0.69
3040201	5556	DEEP CR	89.1	26.5	9 - 65	0.0	0.0 - 0.0	7.6	2.5 - 17.6	1.8	0.5 - 4.8	6.5	2.1 - 15.2	0.0	0.0 - 0.0	10.6	3.3 - 29.9	0.75
3040201	5557	BEAR CR	186.8	15.4	5 - 35	0.0	0.0 - 0.0	5.3	1.7 - 11.7	0.8	0.2 - 2.0	2.7	0.8 - 7.4	0.0	0.0 - 0.0	6.7	2.3 - 16.7	0.81
3040201	5558	JUNIPER CR	166.0	15.0	5 - 34	0.0	0.0 - 0.0	5.5	1.7 - 13.7	0.7	0.2 - 1.8	2.4	0.7 - 7.9	0.0	0.0 - 0.0	6.4	2.0 - 16.7	0.84
3040201	5559	CEDAR CR	179.3	18.2	5 - 36	0.0	0.0 - 0.0	4.9	1.3 - 9.9	1.1	0.3 - 3.3	5.4	1.2 - 13.3	0.0	0.0 - 0.0	6.8	1.8 - 15.8	0.85
3040201	5560	BLACK CR	76.2	31.3	12 - 80	0.0	0.0 - 0.0	5.2	1.8 - 12.5	1.0	0.3 - 2.7	12.4	4.5 - 34.8	0.0	0.0 - 0.0	12.7	4.7 - 35.7	0.87
3040201	5561	BLACK CR	33.3	43.0	15 - 103	12.6	4.5 - 31.5	6.9	2.3 - 15.4	0.7	0.2 - 2.0	13.3	4.5 - 35.5	0.0	0.0 - 0.0	9.4	3.1 - 26.4	0.84
3040201	5562	BLACK CR	268.8	108.4	40 - 245	75.0	27.2 - 161.5	7.7	2.7 - 16.7	0.8	0.3 - 1.9	15.4	4.9 - 41.5	0.0	0.0 - 0.0	9.4	3.4 - 25.7	0.82
3040201	5563	LITTLE BLACK CR	42.1	19.0	7 - 38	0.0	0.0 - 0.0	6.4	2.2 - 13.1	1.1	0.4 - 3.3	4.0	1.4 - 9.9	0.0	0.0 - 0.0	7.5	2.7 - 18.7	0.30
3040201	5564	BIG BLACK CR	81.8	30.5	11 - 78	5.4	1.9 - 14.0	9.9	3.3 - 23.4	1.5	0.5 - 4.3	5.3	1.8 - 18.6	0.0	0.0 - 0.0	8.5	2.5 - 23.8	0.30
3040201	5565	SWIFT CR	151.2	41.0	16 - 97	0.4	0.1 - 0.9	10.2	3.5 - 23.3	1.0	0.3 - 3.0	19.7	7.6 - 54.3	0.0	0.0 - 0.0	9.6	3.9 - 24.3	0.82
3040201	5566	HIGH HILL CR	130.6	40.7	14 - 76	0.0	0.0 - 0.0	10.7	3.9 - 23.8	1.1	0.3 - 2.6	19.1	6.4 - 41.5	0.0	0.0 - 0.0	9.8	3.6 - 28.5	0.84
3040201	5567	JEFFRIES CR	18.6	13.8	5 - 35	0.0	0.0 - 0.0	1.6	0.6 - 4.1	0.5	0.2 - 1.5	5.7	1.9 - 15.2	0.0	0.0 - 0.0	5.9	2.2 - 15.9	0.87
3040201	5568	JEFFRIES CR	157.4	25.3	8 - 56	0.0	0.0 - 0.0	6.7	2.2 - 14.9	0.8	0.3 - 2.5	10.1	3.3 - 24.6	0.0	0.0 - 0.0	7.7	2.9 - 20.1	0.86
3040201	5569	JEFFRIES CR	187.5	48.3	17 - 93	0.0	0.0 - 0.0	23.3	8.2 - 46.8	0.8	0.3 - 2.1	14.5	5.3 - 32.6	0.0	0.0 - 0.0	9.7	3.2 - 22.5	0.83
3040201	5570	MIDDLE SWAMP	113.7	42.5	14 - 89	0.0	0.0 - 0.0	14.2	3.9 - 28.7	1.2	0.3 - 3.3	16.4	5.2 - 40.1	0.0	0.0 - 0.0	10.7	3.3 - 29.0	0.83
3040201	5571	WILLOW CR	127.8	23.4	9 - 62	0.0	0.0 - 0.0	4.4	1.4 - 9.7	1.0	0.3 - 2.9	11.8	4.3 - 34.5	0.0	0.0 - 0.0	6.3	2.4 - 19.0	0.86
3040201	5572	MUDDY CR	82.6	23.0	9 - 49	0.0	0.0 - 0.0	2.1	0.7 - 4.8	1.2	0.4 - 3.1	6.7	2.4 - 16.2	0.0	0.0 - 0.0	13.0	5.5 - 28.5	0.99
3040201	5573	PEE DEE R	290.7	21.0	8 - 53	0.0	0.0 - 0.0	1.2	0.4 - 2.9	0.5	0.2 - 1.2	2.4	0.9 - 7.4	0.0	0.0 - 0.0	17.0	6.4 - 47.4	1.00
3040201	5574	PEE DEE R	30.8	23.2	9 - 58	0.0	0.0 - 0.0	1.3	0.5 - 3.4	1.1	0.4 - 3.9	6.2	2.1 - 20.1	0.0	0.0 - 0.0	14.6	5.6 - 37.3	0.99
3040201	5575	PEE DEE R	37.0	19.9	6 - 52	0.0	0.0 - 0.0	2.5	0.6 - 6.6	0.8	0.2 - 3.0	6.1	1.9 - 19.8	0.0	0.0 - 0.0	10.5	3.2 - 30.8	0.88
3040202	5576	LYNCHES R	179.3	56.6	17 - 128	19.1	5.6 - 45.2	6.0	1.5 - 13.7	1.4	0.4 - 4.6	18.8	5.1 - 47.2	0.0	0.0 - 0.0	11.3	3.7 - 29.1	0.88
3040202	5577	LYNCHES R	39.5	30.0	11 - 69	0.0	0.0 - 0.0	2.9	1.1 - 6.2	1.3	0.4 - 3.0	16.3	5.5 - 39.0	0.0	0.0 - 0.0	9.6	3.4 - 25.2	0.87
3040202	5578	BIG SWAMP	155.0	27.8	10 - 61	0.0	0.0 - 0.0	4.0	1.3 - 9.2	1.2	0.3 - 3.8	15.2	5.2 - 37.0	0.0	0.0 - 0.0	7.4	2.4 - 22.0	0.87
3040202	5579	LYNCHES R	6.7	51.0	16 - 110	0.0	0.0 - 0.0	4.4	1.4 - 9.7	2.0	0.5 - 6.1	26.9	8.6 - 66.9	0.0	0.0 - 0.0	17.6	4.9 - 45.8	0.87
3040202	5580	SPARROW SWAMP	45.5	31.9	11 - 70	0.0	0.0 - 0.0	4.1	1.4 - 9.5	1.4	0.4 - 4.3	17.5	5.6 - 41.0	0.0	0.0 - 0.0	8.8	3.1 - 22.3	0.83
3040202	5581	LAKE SWAMP	112.2	35.6	13 - 71	0.0	0.0 - 0.0	5.9	2.1 - 12.9	1.3	0.4 - 3.1	19.3	6.8 - 45.1	0.0	0.0 - 0.0	9.1	3.3 - 21.6	0.81
3040202	5582	SPARROW SWAMP	155.6	44.0	14 - 94	5.0	1.8 - 10.5	4.0	1.3 - 8.2	1.6	0.5 - 3.9	23.0	7.3 - 50.2	0.0	0.0 - 0.0	10.5	3.8 - 26.5	0.81
3040202	5583	SPARROW SWAMP	17.3	40.0	14 - 88	0.0	0.0 - 0.0	8.4	2.9 - 18.4	1.1	0.3 - 3.1	20.1	6.4 - 59.2	0.0	0.0 - 0.0	10.5	3.3 - 28.3	0.67

8-digit HUC	ID	Name	Area	Catchment Yield		Point sources		Developed Land		Manure		Agricultural Land		Phosphate Mines		Soil parent rock		Frac
				kg/km^2	90% CI	kg/km^2	90% CI	kg/km^2	90% CI	kg/km^2	90% CI	kg/km^2	90% CI	kg/km^2	90% CI	kg/km^2	90% CI	
3040202	5584	*A	65.3	34.1	12 - 84	0.0	0.0 - 0.0	4.7	1.5 - 11.6	1.0	0.3 - 3.2	18.9	6.6 - 56.3	0.0	0.0 - 0.0	9.5	3.9 - 24.6	0.62
3040202	5585	SPARROW SWAM	92.2	32.5	12 - 81	0.0	0.0 - 0.0	4.0	1.4 - 9.2	1.5	0.5 - 4.2	18.8	7.5 - 59.3	0.0	0.0 - 0.0	8.2	3.1 - 20.1	0.62
3040202	5586	*B	67.7	35.9	12 - 68	0.0	0.0 - 0.0	6.0	1.9 - 13.7	1.3	0.4 - 3.5	18.6	5.9 - 45.1	0.0	0.0 - 0.0	10.1	3.5 - 21.6	0.67
3040202	5587	LYNCHES R	565.1	35.1	13 - 63	3.4	1.3 - 6.5	3.9	1.3 - 8.2	1.5	0.5 - 3.2	16.3	5.2 - 38.3	0.0	0.0 - 0.0	10.0	3.7 - 22.2	0.83
3040202	5588	LYNCHES R	294.1	18.4	6 - 35	0.0	0.0 - 0.0	3.9	1.4 - 7.2	1.6	0.4 - 3.5	4.3	1.6 - 8.8	0.0	0.0 - 0.0	8.7	3.0 - 17.9	0.75
3040202	5589	LYNCHES R	106.1	23.4	8 - 44	0.0	0.0 - 0.0	5.1	1.8 - 11.1	2.0	0.5 - 5.2	6.4	2.0 - 14.9	0.0	0.0 - 0.0	9.9	3.2 - 23.1	0.71
3040202	5590	LITTLE FORK CR	69.2	21.1	8 - 41	0.0	0.0 - 0.0	6.8	2.4 - 14.9	1.4	0.4 - 3.8	4.9	1.6 - 13.1	0.0	0.0 - 0.0	8.0	2.4 - 19.0	0.69
3040202	5591	LYNCHES R	4.8	17.8	7 - 40	0.0	0.0 - 0.0	5.3	1.9 - 12.1	1.1	0.3 - 2.7	1.5	0.5 - 4.0	0.0	0.0 - 0.0	10.0	3.5 - 24.4	0.69
3040202	5592	LYNCHES R	128.8	24.1	9 - 64	0.0	0.0 - 0.0	4.1	1.4 - 10.6	3.0	0.9 - 10.2	6.9	2.2 - 19.0	0.0	0.0 - 0.0	10.1	3.7 - 28.4	0.69
3040202	5593	*C	61.6	56.6	19 - 158	4.0	1.4 - 11.1	8.5	2.9 - 22.3	6.0	1.6 - 18.7	20.5	6.8 - 61.8	0.0	0.0 - 0.0	17.6	4.8 - 47.6	0.63
3040202	5594	LYNCHES R	131.6	40.5	16 - 79	0.0	0.0 - 0.0	4.3	1.5 - 9.2	12.2	3.8 - 31.8	15.1	5.4 - 36.9	0.0	0.0 - 0.0	8.9	3.1 - 20.0	0.63
3040202	5595	FLAT CR	124.6	15.8	5 - 33	0.0	0.0 - 0.0	3.2	0.9 - 7.4	1.5	0.4 - 4.1	3.1	0.9 - 7.3	0.0	0.0 - 0.0	8.0	2.1 - 18.5	0.69
3040202	5596	BUFFALO CR	93.5	14.7	5 - 29	0.0	0.0 - 0.0	4.8	1.5 - 9.5	1.3	0.4 - 3.6	2.4	0.9 - 6.3	0.0	0.0 - 0.0	6.2	2.3 - 14.2	0.71
3040202	5597	LITTLE LYNCHES	268.0	17.2	5 - 43	0.0	0.0 - 0.0	3.2	1.0 - 7.3	1.8	0.4 - 5.5	3.6	1.1 - 9.0	0.0	0.0 - 0.0	8.5	2.4 - 21.0	0.75
3040202	5598	LITTLE LYNCHES	162.5	18.5	6 - 33	0.4	0.1 - 0.8	4.9	1.5 - 9.1	1.8	0.5 - 3.9	3.9	1.1 - 9.4	0.0	0.0 - 0.0	7.5	2.6 - 14.4	0.68
3040202	5599	HANGING ROCK C	80.8	35.8	13 - 66	9.0	3.3 - 17.1	8.0	2.4 - 16.7	2.4	0.8 - 6.6	5.2	1.9 - 12.7	0.0	0.0 - 0.0	11.1	3.8 - 23.0	0.68
3040202	5600	LAKE SWAMP	76.2	38.1	12 - 86	0.0	0.0 - 0.0	3.7	1.3 - 8.5	1.7	0.5 - 4.6	23.0	8.1 - 55.3	0.0	0.0 - 0.0	9.7	2.9 - 24.7	0.87
3040202	5601	*D	196.3	38.0	13 - 75	0.0	0.0 - 0.0	8.6	2.9 - 17.6	1.6	0.5 - 4.4	19.6	7.0 - 49.1	0.0	0.0 - 0.0	8.3	2.9 - 22.0	0.81
3040202	5602	LAKE SWAMP	116.2	26.8	10 - 57	0.0	0.0 - 0.0	3.8	1.4 - 8.2	1.0	0.3 - 2.7	13.7	5.0 - 34.4	0.0	0.0 - 0.0	8.2	3.1 - 19.9	0.81
3040203	5603	LUMBER R	47.6	33.8	11 - 69	2.1	0.7 - 4.6	5.2	1.7 - 10.8	2.1	0.5 - 5.0	11.2	3.2 - 25.4	0.0	0.0 - 0.0	13.3	4.7 - 30.7	0.95
3040203	5604	LUMBER R	187.4	47.9	17 - 116	1.1	0.4 - 3.0	4.0	1.5 - 9.9	9.5	2.6 - 28.8	23.5	8.7 - 70.1	0.0	0.0 - 0.0	9.8	3.4 - 29.5	0.94
3040203	5605	PORTER SWAMP	234.3	44.6	16 - 106	0.0	0.0 - 0.0	4.4	1.6 - 11.8	11.9	3.7 - 42.3	20.3	7.0 - 50.4	0.0	0.0 - 0.0	8.0	3.0 - 19.9	0.93
3040203	5606	LUMBER R	102.7	37.0	15 - 82	0.0	0.0 - 0.0	2.1	0.7 - 4.6	7.7	2.4 - 26.0	11.8	4.3 - 30.5	0.0	0.0 - 0.0	15.4	5.4 - 35.0	0.93
3040203	5607	BIG SWAMP	95.4	41.6	15 - 77	0.0	0.0 - 0.0	2.5	0.9 - 4.8	14.9	4.5 - 37.9	12.8	4.6 - 29.8	0.0	0.0 - 0.0	11.4	4.2 - 25.4	0.91
3040203	5608	BRYANT SWAMP	66.5	71.4	28 - 151	3.6	1.3 - 8.2	6.0	2.2 - 13.1	37.1	12.1 - 86.5	15.1	4.7 - 32.3	0.0	0.0 - 0.0	9.6	3.6 - 21.0	0.89
3040203	5609	BIG SWAMP	67.9	70.1	23 - 135	0.0	0.0 - 0.0	2.3	0.8 - 4.9	29.0	8.3 - 66.8	22.3	7.1 - 53.6	0.0	0.0 - 0.0	16.6	5.3 - 36.5	0.89
3040203	5610	BEAR FORD SWAM	115.0	71.1	23 - 176	0.0	0.0 - 0.0	3.1	1.1 - 6.4	40.5	11.2 - 120.7	16.6	6.1 - 43.8	0.0	0.0 - 0.0	10.9	3.7 - 28.2	0.87
3040203	5611	BIG SWAMP	187.2	59.8	18 - 127	0.0	0.0 - 0.0	2.9	0.8 - 6.9	27.0	7.7 - 78.6	18.2	5.4 - 42.7	0.0	0.0 - 0.0	11.6	3.7 - 28.9	0.87
3040203	5612	BIG SWAMP	18.2	68.8	24 - 191	0.0	0.0 - 0.0	3.2	1.0 - 7.6	31.4	10.5 - 104.4	18.9	6.2 - 53.7	0.0	0.0 - 0.0	15.3	5.3 - 41.8	0.83
3040203	5613	BIG SWAMP	10.7	76.3	28 - 208	0.0	0.0 - 0.0	5.0	1.7 - 13.4	34.1	12.3 - 117.7	20.1	6.1 - 52.6	0.0	0.0 - 0.0	17.0	5.8 - 47.2	0.82
3040203	5614	MARSH SWAMP	154.1	42.9	15 - 88	2.9	0.9 - 6.6	5.5	1.8 - 11.7	10.2	3.6 - 28.7	16.0	5.3 - 41.7	0.0	0.0 - 0.0	8.3	2.9 - 20.8	0.82
3040203	5615	TENMILE SWAMP	177.3	48.7	16 - 87	0.0	0.0 - 0.0	3.6	1.1 - 7.2	14.2	4.2 - 33.0	20.9	7.2 - 48.6	0.0	0.0 - 0.0	10.0	3.1 - 20.9	0.83
3040203	5616	LUMBER R	261.7	118.0	44 - 291	62.6	22.6 - 167.3	11.3	3.9 - 25.9	12.8	4.0 - 43.7	18.8	6.7 - 60.5	0.0	0.0 - 0.0	12.6	4.3 - 33.0	0.91
3040203	5617	RAFT SWAMP	41.6	58.7	22 - 110	0.0	0.0 - 0.0	7.9	2.7 - 16.9	15.2	4.8 - 46.8	22.6	8.2 - 55.1	0.0	0.0 - 0.0	13.1	5.8 - 29.9	0.87
3040203	5618	RAFT SWAMP	41.9	46.5	14 - 117	0.0	0.0 - 0.0	3.2	0.9 - 7.6	12.8	3.4 - 33.6	18.5	5.6 - 52.8	0.0	0.0 - 0.0	12.0	3.5 - 31.5	0.85
3040203	5619	RAFT SWAMP	140.7	39.9	12 - 98	0.0	0.0 - 0.0	6.2	1.8 - 14.9	9.3	2.3 - 27.1	15.9	5.3 - 44.6	0.0	0.0 - 0.0	8.5	2.8 - 22.5	0.81
3040203	5620	LITTLE RAFT SWA	121.3	49.1	16 - 121	9.3	3.2 - 24.7	7.7	2.5 - 16.9	9.3	2.9 - 25.2	15.5	4.8 - 40.0	0.0	0.0 - 0.0	7.3	2.2 - 20.7	0.81
3040203	5621	RICHLAND SWAM	116.5	48.0	17 - 114	0.0	0.0 - 0.0	4.4	1.3 - 10.5	13.9	4.8 - 40.8	21.2	6.4 - 60.2	0.0	0.0 - 0.0	8.5	3.0 - 21.2	0.85
3040203	5622	LUMBER R	25.7	70.3	23 - 130	0.0	0.0 - 0.0	21.2	6.9 - 40.5	13.8	3.5 - 34.6	20.4	6.7 - 43.6	0.0	0.0 - 0.0	15.0	4.9 - 32.2	0.87

8-digit HUC	ID	Name	Area	Catchment Yield		Point sources		Developed Land		Manure		Agricultural Land		Phosphate Mines		Soil parent rock		Frac
				kg/km^2	90% CI	kg/km^2	90% CI	kg/km^2	90% CI	kg/km^2	90% CI	kg/km^2	90% CI	kg/km^2	90% CI	kg/km^2	90% CI	
3040203	5623	BEAR SWAMP	165.8	52.1	18 - 105	0.0	0.0 - 0.0	6.0	2.1 - 11.4	15.0	4.3 - 42.3	22.2	8.2 - 60.6	0.0	0.0 - 0.0	8.9	2.9 - 23.5	0.87
3040203	5624	LUMBER R	247.5	58.1	20 - 110	4.4	1.4 - 8.8	6.6	2.2 - 12.2	13.5	4.0 - 31.9	21.4	7.7 - 48.3	0.0	0.0 - 0.0	12.2	4.2 - 24.8	0.87
3040203	5625	DROWNING CR	108.7	159.8	59 - 293	111.8	43.6 - 215.2	32.6	11.7 - 67.1	3.0	1.0 - 7.4	2.2	0.8 - 4.8	0.0	0.0 - 0.0	10.1	3.8 - 21.1	0.74
3040203	5626	HORSE CR	11.2	16.2	6 - 34	0.0	0.0 - 0.0	1.6	0.5 - 3.0	2.8	0.9 - 6.6	2.2	0.8 - 5.5	0.0	0.0 - 0.0	9.7	3.8 - 23.5	0.73
3040203	5627	HORSE CR	44.1	29.6	10 - 73	0.0	0.0 - 0.0	16.3	5.3 - 38.5	3.2	0.9 - 8.8	2.0	0.7 - 5.3	0.0	0.0 - 0.0	8.1	3.1 - 23.1	0.70
3040203	5628	DEEP CR	61.3	20.0	7 - 48	0.0	0.0 - 0.0	6.2	2.1 - 16.8	3.5	1.1 - 10.0	2.4	0.9 - 7.1	0.0	0.0 - 0.0	7.9	2.7 - 19.7	0.70
3040203	5629	DROWNING CR	34.2	28.8	9 - 56	0.0	0.0 - 0.0	8.3	2.6 - 17.8	4.6	1.2 - 12.7	3.0	1.0 - 7.4	0.0	0.0 - 0.0	13.0	4.1 - 28.0	0.73
3040203	5630	DROWNING CR	71.4	25.1	8 - 60	0.0	0.0 - 0.0	4.5	1.4 - 10.9	5.4	1.6 - 14.2	3.5	1.2 - 8.6	0.0	0.0 - 0.0	11.6	3.9 - 30.0	0.70
3040203	5632	DROWNING CR	145.0	23.6	8 - 64	0.0	0.0 - 0.0	6.0	2.2 - 15.8	4.5	1.5 - 14.8	3.0	0.9 - 9.8	0.0	0.0 - 0.0	10.2	3.6 - 28.4	0.65
3040203	5633	NAKED CR	101.6	27.7	9 - 64	0.0	0.0 - 0.0	6.6	2.0 - 18.5	7.9	2.3 - 22.5	4.8	1.6 - 12.6	0.0	0.0 - 0.0	8.4	2.6 - 19.3	0.70
3040203	5634	*A	49.9	25.3	9 - 70	0.0	0.0 - 0.0	12.5	4.2 - 33.7	2.7	0.8 - 9.5	2.2	0.8 - 5.8	0.0	0.0 - 0.0	7.9	2.7 - 22.8	0.74
3040203	5635	ASHPOLE CR	38.4	32.1	10 - 85	0.0	0.0 - 0.0	2.7	0.8 - 6.5	4.5	1.2 - 13.5	11.6	3.8 - 36.2	0.0	0.0 - 0.0	13.3	3.9 - 41.4	0.94
3040203	5636	ASHPOLE CR	24.2	57.5	22 - 114	0.0	0.0 - 0.0	2.3	1.0 - 4.3	15.0	4.6 - 37.5	22.2	8.5 - 49.0	0.0	0.0 - 0.0	18.0	7.0 - 38.1	0.92
3040203	5637	INDIAN SWAMP	80.4	50.5	18 - 118	0.0	0.0 - 0.0	3.7	1.3 - 8.8	15.1	4.8 - 34.8	22.3	7.5 - 61.1	0.0	0.0 - 0.0	9.4	3.4 - 24.6	0.91
3040203	5638	ASHPOLE CR	18.2	55.6	17 - 113	0.0	0.0 - 0.0	2.2	0.7 - 4.6	15.5	4.5 - 42.3	23.9	8.7 - 60.3	0.0	0.0 - 0.0	14.0	4.7 - 35.3	0.91
3040203	5639	HOG SWAMP	167.3	62.5	23 - 174	0.0	0.0 - 0.0	6.7	2.4 - 19.3	17.9	5.5 - 51.3	26.6	10.6 - 95.7	0.0	0.0 - 0.0	11.2	4.1 - 33.6	0.89
3040203	5640	ASHPOLE CR	242.3	62.8	22 - 119	0.0	0.0 - 0.0	4.9	1.6 - 10.5	18.9	6.2 - 57.0	28.0	8.9 - 72.0	0.0	0.0 - 0.0	11.0	3.7 - 28.0	0.89
3040203	5641	BEAR SWAMP	200.1	40.6	12 - 88	1.2	0.4 - 2.9	5.5	1.8 - 11.5	8.6	2.8 - 20.2	17.0	4.9 - 43.0	0.0	0.0 - 0.0	8.3	2.5 - 19.8	0.92
3040204	5642	*A	143.9	36.6	11 - 76	0.0	0.0 - 0.0	3.4	1.1 - 7.0	3.2	0.8 - 9.4	16.6	5.3 - 36.7	0.0	0.0 - 0.0	13.4	4.0 - 34.5	0.99
3040204	5644	BROWN SWAMP	85.4	42.1	15 - 107	0.0	0.0 - 0.0	6.4	2.2 - 14.9	3.8	1.2 - 11.0	21.5	6.9 - 56.5	0.0	0.0 - 0.0	10.4	3.7 - 28.6	0.98
3040204	5645	LITTLE PEE DEE R	18.8	33.9	9 - 88	0.0	0.0 - 0.0	1.5	0.4 - 3.7	2.0	0.5 - 7.0	10.3	2.6 - 32.7	0.0	0.0 - 0.0	20.1	4.9 - 55.2	0.98
3040204	5646	CHINNERS SWAMI	4.0	24.0	8 - 55	0.0	0.0 - 0.0	0.0	0.0 - 0.0	2.0	0.6 - 6.1	4.4	1.7 - 11.7	0.0	0.0 - 0.0	17.7	6.1 - 47.8	0.98
3040204	5647	BRUNSON SWAMP	82.4	45.6	18 - 100	0.0	0.0 - 0.0	6.9	2.4 - 15.9	4.2	1.2 - 11.1	24.5	9.0 - 58.6	0.0	0.0 - 0.0	10.1	3.4 - 25.2	0.96
3040204	5648	CHINNERS SWAMI	125.9	42.2	14 - 90	0.0	0.0 - 0.0	4.3	1.3 - 9.2	4.0	1.2 - 11.2	21.7	7.2 - 57.5	0.0	0.0 - 0.0	12.2	3.8 - 30.1	0.96
3040204	5649	LITTLE PEE DEE R	100.9	38.4	12 - 75	0.0	0.0 - 0.0	2.4	0.9 - 4.6	2.5	0.8 - 7.3	14.1	5.1 - 32.6	0.0	0.0 - 0.0	19.4	6.7 - 49.1	0.98
3040204	5650	LITTLE PEE DEE R	84.9	38.3	14 - 89	0.0	0.0 - 0.0	3.4	1.2 - 8.5	3.1	1.0 - 9.5	17.8	6.8 - 49.9	0.0	0.0 - 0.0	13.9	4.6 - 37.2	0.97
3040204	5651	LAKE SWAMP	456.0	46.6	16 - 93	1.6	0.5 - 3.7	5.9	1.7 - 12.0	4.7	1.5 - 11.2	25.0	8.6 - 62.5	0.0	0.0 - 0.0	9.4	3.1 - 21.2	0.96
3040204	5652	LITTLE PEE DEE R	67.2	38.0	12 - 79	0.0	0.0 - 0.0	1.9	0.6 - 4.2	2.2	0.6 - 5.9	14.2	4.5 - 32.8	0.0	0.0 - 0.0	19.7	7.0 - 45.7	0.96
3040204	5653	CEDAR CR	118.3	32.6	11 - 73	0.0	0.0 - 0.0	3.8	1.2 - 8.3	3.1	0.9 - 9.4	16.7	6.1 - 40.7	0.0	0.0 - 0.0	8.9	2.6 - 23.8	0.95
3040204	5654	LITTLE PEE DEE R	35.9	99.7	38 - 230	53.0	19.5 - 121.2	12.8	4.4 - 28.2	2.3	0.8 - 5.9	19.2	6.8 - 46.4	0.0	0.0 - 0.0	12.4	5.0 - 30.0	0.95
3040204	5655	LITTLE PEE DEE R	48.7	34.5	11 - 72	0.0	0.0 - 0.0	6.8	2.0 - 13.9	2.1	0.6 - 5.0	16.1	5.4 - 41.6	0.0	0.0 - 0.0	9.4	3.2 - 27.5	0.95
3040204	5656	LITTLE PEE DEE R	416.1	76.8	28 - 159	13.7	4.9 - 29.2	7.7	2.9 - 17.1	13.2	4.1 - 36.1	27.9	9.1 - 75.9	0.0	0.0 - 0.0	14.2	5.6 - 30.9	0.93
3040204	5657	BIG SHOE HEEL CI	423.4	36.0	12 - 91	2.0	0.8 - 5.1	4.7	1.8 - 10.4	6.9	2.4 - 23.7	13.6	4.2 - 38.9	0.0	0.0 - 0.0	8.8	3.1 - 25.1	0.87
3040204	5658	LITTLE PEE DEE R	29.8	49.6	16 - 96	0.0	0.0 - 0.0	3.8	1.2 - 7.8	9.1	2.5 - 23.6	20.2	6.4 - 41.7	0.0	0.0 - 0.0	16.6	5.5 - 33.9	0.87
3040204	5659	BRIDGES CR	56.8	49.7	20 - 121	0.0	0.0 - 0.0	3.5	1.3 - 8.7	11.7	3.7 - 38.6	24.8	9.9 - 55.2	0.0	0.0 - 0.0	9.8	3.7 - 24.4	0.85
3040204	5660	LEITH CR	100.0	108.8	41 - 214	62.9	22.2 - 130.4	14.8	5.8 - 30.3	5.1	1.8 - 10.2	17.8	6.3 - 41.5	0.0	0.0 - 0.0	8.2	2.9 - 18.3	0.81
3040204	5661	BRIDGES CR	33.8	39.2	13 - 94	0.0	0.0 - 0.0	14.1	4.7 - 31.4	3.7	1.1 - 12.0	13.9	4.5 - 39.8	0.0	0.0 - 0.0	7.5	2.2 - 21.8	0.81
3040204	5662	LITTLE PEE DEE R	31.3	35.0	13 - 86	0.0	0.0 - 0.0	4.7	1.7 - 11.1	3.1	1.0 - 7.8	17.4	5.8 - 44.8	0.0	0.0 - 0.0	9.9	3.6 - 24.4	0.85
3040204	5663	GUM SEAMP CR	374.9	23.5	7 - 52	0.9	0.3 - 2.1	6.7	2.0 - 14.3	2.4	0.6 - 7.0	5.8	1.8 - 15.1	0.0	0.0 - 0.0	7.7	2.4 - 17.4	0.82

8-digit HUC	ID	Name	Area	Catchment Yield		Point sources		Developed Land		Manure		Agricultural Land		Phosphate Mines		Soil parent rock		Frac
				kg/km^2	90% CI	kg/km^2	90% CI	kg/km^2	90% CI	kg/km^2	90% CI	kg/km^2	90% CI	kg/km^2	90% CI	kg/km^2	90% CI	
3040204	5664	LITTLE PEE DEE R	78.0	34.7	10 - 65	0.0	0.0 - 0.0	6.3	1.8 - 14.3	2.6	0.6 - 6.2	17.8	5.0 - 39.1	0.0	0.0 - 0.0	8.0	2.1 - 16.9	0.82
3040204	5665	BUCK SWAMP	403.3	57.1	16 - 124	1.4	0.4 - 3.3	5.6	1.6 - 12.1	9.2	2.6 - 24.2	26.7	8.2 - 69.2	0.0	0.0 - 0.0	14.2	4.4 - 37.6	0.93
3040204	5666	REEDY CR	127.6	39.5	13 - 70	0.0	0.0 - 0.0	5.1	1.5 - 9.8	2.4	0.6 - 6.1	20.1	6.4 - 46.0	0.0	0.0 - 0.0	12.0	3.8 - 28.3	0.97
3040205	5667	BLACK R	324.4	20.6	8 - 41	0.5	0.2 - 1.2	2.3	0.9 - 4.8	0.7	0.2 - 2.0	3.2	1.2 - 7.8	0.0	0.0 - 0.0	13.8	5.5 - 31.5	1.00
3040205	5668	BLACK MINGO CR	675.8	30.6	9 - 56	0.0	0.0 - 0.0	3.6	1.0 - 6.6	1.2	0.3 - 3.2	15.3	4.6 - 32.6	0.0	0.0 - 0.0	10.6	3.2 - 23.9	0.97
3040205	5670	*A	262.8	32.4	10 - 70	0.0	0.0 - 0.0	5.7	1.8 - 11.9	1.6	0.5 - 4.4	18.0	5.6 - 45.4	0.0	0.0 - 0.0	7.2	2.3 - 18.3	0.90
3040205	5671	BLACK R	103.2	29.8	13 - 64	0.0	0.0 - 0.0	3.4	1.4 - 7.6	1.1	0.3 - 3.3	16.7	6.9 - 37.2	0.0	0.0 - 0.0	8.6	3.4 - 21.4	0.90
3040205	5672	*B	396.9	27.9	8 - 66	0.9	0.3 - 2.5	3.5	1.0 - 9.7	2.5	0.7 - 6.8	15.1	4.6 - 43.5	0.0	0.0 - 0.0	6.0	1.8 - 14.0	0.88
3040205	5673	BLACK R	124.8	25.2	6 - 59	0.0	0.0 - 0.0	2.7	0.8 - 5.9	2.4	0.6 - 6.5	12.6	3.3 - 31.2	0.0	0.0 - 0.0	7.5	1.8 - 19.9	0.88
3040205	5674	BLACK R	289.6	35.1	13 - 92	0.0	0.0 - 0.0	4.0	1.4 - 10.5	3.4	1.1 - 8.8	17.9	6.1 - 54.5	0.0	0.0 - 0.0	9.8	3.7 - 26.5	0.85
3040205	5675	BLACK R	237.5	39.8	14 - 86	0.0	0.0 - 0.0	3.7	1.1 - 8.1	2.5	0.8 - 7.0	24.5	8.4 - 58.4	0.0	0.0 - 0.0	9.1	3.1 - 23.2	0.81
3040205	5676	TIMBER CR	36.5	49.6	18 - 87	0.0	0.0 - 0.0	3.8	1.3 - 7.7	3.1	1.0 - 10.0	24.5	8.0 - 52.9	0.0	0.0 - 0.0	18.2	6.4 - 46.9	0.81
3040205	5677	TIMBER CR	155.6	31.6	12 - 65	0.0	0.0 - 0.0	3.2	1.1 - 7.1	1.9	0.5 - 5.0	17.7	5.8 - 47.1	0.0	0.0 - 0.0	8.8	2.9 - 22.3	0.80
3040205	5678	ROCKY BLUFF SW	50.0	46.6	13 - 88	0.0	0.0 - 0.0	5.7	1.7 - 11.3	3.0	0.8 - 8.9	23.5	6.3 - 55.6	0.0	0.0 - 0.0	14.5	4.7 - 30.4	0.80
3040205	5679	COWPEN SWAMP	70.2	34.6	11 - 79	0.0	0.0 - 0.0	4.3	1.4 - 9.8	2.4	0.7 - 7.1	21.3	6.8 - 54.6	0.0	0.0 - 0.0	6.7	2.4 - 16.5	0.76
3040205	5680	ROCKY BLUFF SW	150.1	28.7	9 - 51	0.0	0.0 - 0.0	9.6	2.7 - 18.3	1.5	0.5 - 3.7	11.9	3.7 - 30.0	0.0	0.0 - 0.0	5.7	2.0 - 13.8	0.76
3040205	5681	POCOTALIGO R	322.7	41.6	14 - 109	5.5	1.7 - 16.1	6.8	2.0 - 18.5	3.9	1.1 - 9.7	17.2	5.5 - 43.6	0.0	0.0 - 0.0	8.3	3.1 - 21.4	0.85
3040205	5682	POCOTALIGO R	151.5	156.3	54 - 355	112.4	38.8 - 261.1	9.1	3.0 - 20.1	3.0	0.9 - 8.3	22.2	7.2 - 55.8	0.0	0.0 - 0.0	9.6	3.2 - 26.9	0.79
3040205	5683	POCOTALIGO R	157.2	41.9	14 - 101	0.2	0.1 - 0.5	30.5	10.3 - 71.3	0.8	0.2 - 1.9	5.7	2.0 - 18.3	0.0	0.0 - 0.0	4.8	1.7 - 13.4	0.73
3040205	5684	NASTY BR	202.4	18.7	6 - 52	0.4	0.1 - 1.0	5.3	1.5 - 12.8	0.9	0.2 - 2.7	6.9	2.2 - 19.8	0.0	0.0 - 0.0	5.3	1.7 - 15.5	0.73
3040205	5685	SAMMY SWAMP	162.8	34.0	14 - 76	0.0	0.0 - 0.0	3.9	1.5 - 7.6	3.8	1.3 - 11.1	19.1	7.4 - 49.1	0.0	0.0 - 0.0	7.2	3.0 - 16.0	0.79
3040206	5686	WACCAMAW R	43.9	36.6	11 - 90	0.0	0.0 - 0.0	2.4	0.7 - 6.7	2.4	0.6 - 8.5	11.2	3.2 - 28.2	0.0	0.0 - 0.0	20.6	5.8 - 56.6	0.97
3040206	5687	WACCAMAW R	239.0	36.4	13 - 64	0.0	0.0 - 0.0	8.5	2.7 - 16.3	2.2	0.8 - 5.7	9.3	3.4 - 20.3	0.0	0.0 - 0.0	16.4	6.0 - 36.7	0.95
3040206	5688	WACCAMAW R	117.0	53.5	21 - 108	0.0	0.0 - 0.0	6.9	2.7 - 13.0	4.5	1.4 - 14.2	21.3	8.1 - 53.0	0.0	0.0 - 0.0	20.8	7.8 - 55.6	0.92
3040206	5689	WACCAMAW R	31.2	54.5	18 - 102	7.4	2.5 - 15.3	8.4	2.7 - 17.0	2.6	0.6 - 7.1	13.2	4.0 - 38.0	0.0	0.0 - 0.0	22.9	7.0 - 57.1	0.91
3040206	5690	*A	101.1	47.0	14 - 99	2.9	0.9 - 5.7	14.4	4.1 - 32.8	6.4	2.0 - 18.4	13.7	4.7 - 35.2	0.0	0.0 - 0.0	9.6	3.3 - 23.7	0.90
3040206	5691	WACCAMAW R	31.1	35.3	12 - 78	0.0	0.0 - 0.0	1.5	0.5 - 3.3	4.2	1.2 - 12.1	9.6	3.2 - 29.6	0.0	0.0 - 0.0	20.0	6.5 - 58.0	0.90
3040206	5692	*B	46.7	50.0	16 - 134	0.0	0.0 - 0.0	3.4	1.0 - 9.5	10.2	3.0 - 27.3	22.1	6.1 - 66.3	0.0	0.0 - 0.0	14.3	4.7 - 38.4	0.90
3040206	5693	WACCAMAW R	28.2	41.1	13 - 89	0.0	0.0 - 0.0	4.6	1.4 - 10.7	7.1	2.1 - 21.0	11.5	3.7 - 30.3	0.0	0.0 - 0.0	18.0	5.5 - 48.0	0.90
3040206	5694	WACCAMAW R	95.6	33.7	12 - 81	0.1	0.0 - 0.3	2.1	0.7 - 5.3	6.7	2.2 - 18.8	13.8	5.3 - 33.6	0.0	0.0 - 0.0	10.9	3.8 - 34.1	0.89
3040206	5695	WACCAMAW R	30.4	65.7	22 - 130	0.0	0.0 - 0.0	4.1	1.4 - 8.1	14.1	4.6 - 35.9	30.4	9.5 - 68.8	0.0	0.0 - 0.0	17.1	6.4 - 43.7	0.88
3040206	5696	JUNIPER CR	486.4	8.9	3 - 21	0.0	0.0 - 0.0	0.6	0.1 - 1.3	1.3	0.3 - 4.0	2.3	0.6 - 6.0	0.0	0.0 - 0.0	4.7	1.3 - 11.2	0.86
3040206	5697	WACCAMAW R	28.2	37.2	13 - 82	0.0	0.0 - 0.0	2.1	0.8 - 4.7	6.5	2.0 - 17.1	10.1	3.6 - 26.0	0.0	0.0 - 0.0	18.5	6.5 - 51.2	0.86
3040206	5698	WACCAMAW R	29.7	24.8	7 - 57	0.0	0.0 - 0.0	1.2	0.3 - 3.1	3.2	0.9 - 10.9	4.6	1.4 - 11.3	0.0	0.0 - 0.0	15.8	5.2 - 42.0	0.85
3040206	5699	WACCAMAW R	7.9	21.8	8 - 56	0.0	0.0 - 0.0	1.3	0.4 - 3.3	3.3	0.9 - 10.4	4.2	1.5 - 11.3	0.0	0.0 - 0.0	13.0	4.4 - 39.4	0.82
3040206	5700	WACCAMAW R	144.3	39.0	13 - 73	0.0	0.0 - 0.0	2.2	0.7 - 5.5	16.3	5.5 - 45.6	10.6	3.6 - 25.5	0.0	0.0 - 0.0	9.9	3.5 - 24.2	0.76
3040206	5701	SLAP SWAMP	73.8	37.4	12 - 77	0.0	0.0 - 0.0	3.5	1.3 - 6.8	10.9	3.3 - 27.3	11.5	4.3 - 27.0	0.0	0.0 - 0.0	11.5	3.9 - 30.1	0.76
3040206	5702	BOGUE SWAMP	112.2	48.3	15 - 100	2.5	0.9 - 6.0	3.3	1.0 - 7.7	9.8	2.8 - 32.4	16.6	4.9 - 42.8	0.0	0.0 - 0.0	16.0	4.8 - 42.4	0.82
3040206	5703	WHITE MARSH	140.0	42.6	16 - 86	0.1	0.0 - 0.3	2.5	0.9 - 5.6	9.2	3.0 - 23.4	15.6	5.7 - 35.6	0.0	0.0 - 0.0	15.2	5.5 - 38.7	0.85

8-digit HUC	ID	Name	Area	Catchment Yield		Point sources		Developed Land		Manure		Agricultural Land		Phosphate Mines		Soil parent rock		Frac
				kg/km^2	90% CI	kg/km^2	90% CI	kg/km^2	90% CI	kg/km^2	90% CI	kg/km^2	90% CI	kg/km^2	90% CI	kg/km^2	90% CI	
3040206	5704	WHITE MARSH	322.4	68.2	23 - 126	5.8	2.0 - 11.8	4.9	1.6 - 9.0	21.9	7.3 - 50.1	24.4	7.6 - 56.2	0.0	0.0 - 0.0	11.2	3.6 - 24.0	0.81
3040206	5705	ELKTON SWAMP	103.6	64.6	20 - 154	0.0	0.0 - 0.0	2.6	0.9 - 6.4	37.8	10.5 - 108.8	15.7	6.1 - 46.0	0.0	0.0 - 0.0	8.5	2.8 - 22.5	0.73
3040206	5706	BROWN MARSH SV	152.3	84.7	29 - 209	0.0	0.0 - 0.0	2.9	0.9 - 7.0	49.2	15.2 - 130.2	21.0	6.2 - 53.5	0.0	0.0 - 0.0	11.5	4.1 - 31.4	0.73
3040206	5707	WHITE MARSH, WI	128.4	57.2	19 - 140	3.2	1.1 - 7.8	10.9	4.0 - 25.9	13.5	4.6 - 43.4	23.2	7.4 - 61.8	0.0	0.0 - 0.0	6.3	2.4 - 14.8	0.81
3040206	5708	*H	68.7	37.4	13 - 77	0.0	0.0 - 0.0	2.0	0.6 - 4.0	10.0	3.1 - 27.5	16.9	4.8 - 38.3	0.0	0.0 - 0.0	8.6	3.0 - 23.3	0.88
3040206	5709	SEVEN CREEKS	77.0	62.6	20 - 122	0.0	0.0 - 0.0	4.5	1.5 - 9.7	15.5	3.9 - 44.6	25.2	8.2 - 62.4	0.0	0.0 - 0.0	17.4	5.4 - 41.7	0.89
3040206	5710	SEVEN CREEKS	29.6	78.4	28 - 155	0.0	0.0 - 0.0	4.4	1.4 - 8.9	21.0	7.3 - 58.5	36.4	12.0 - 90.3	0.0	0.0 - 0.0	16.6	5.7 - 36.7	0.86
3040206	5711	SEVEN CREEKS	111.2	53.1	18 - 103	0.0	0.0 - 0.0	4.9	1.6 - 9.6	15.4	4.9 - 39.0	26.2	8.3 - 57.1	0.0	0.0 - 0.0	6.6	2.2 - 14.7	0.82
3040206	5712	GUM SWAMP	117.5	44.2	17 - 98	0.0	0.0 - 0.0	4.5	1.6 - 10.5	12.4	4.4 - 31.5	21.1	7.4 - 55.8	0.0	0.0 - 0.0	6.3	2.3 - 17.2	0.82
3040206	5713	GRISSET SWAMP	189.3	45.1	16 - 85	4.0	1.3 - 7.6	7.3	2.2 - 14.1	9.3	3.0 - 25.5	18.1	5.9 - 41.3	0.0	0.0 - 0.0	6.5	2.1 - 14.2	0.86
3040206	5714	BUCK CR	146.7	46.0	13 - 92	0.0	0.0 - 0.0	7.5	2.0 - 14.4	5.0	1.5 - 14.0	18.1	5.1 - 49.7	0.0	0.0 - 0.0	15.4	4.6 - 42.5	0.91
3040206	5715	SIMPSON CR	105.5	68.2	19 - 146	0.0	0.0 - 0.0	5.4	1.5 - 12.0	6.9	1.6 - 17.2	38.7	11.5 - 97.6	0.0	0.0 - 0.0	17.1	4.8 - 37.8	0.92
3040206	5716	*K	312.4	48.1	19 - 92	0.0	0.0 - 0.0	8.7	3.3 - 17.8	4.1	1.3 - 11.8	22.6	8.4 - 51.2	0.0	0.0 - 0.0	12.7	4.8 - 31.5	0.95
3040206	5717	INTRACOASTAL W	160.3	47.9	14 - 103	0.9	0.3 - 1.9	34.3	10.1 - 73.6	1.0	0.3 - 2.4	2.0	0.7 - 4.8	0.0	0.0 - 0.0	9.7	3.1 - 22.7	0.97
3040207	5718	*A	127.9	8.8	2 - 20	0.0	0.0 - 0.0	4.0	1.0 - 9.5	1.0	0.3 - 3.4	2.0	0.6 - 5.1	0.0	0.0 - 0.0	1.8	0.5 - 4.5	1.00
3040207	5719	*A	156.0	23.2	8 - 56	0.1	0.0 - 0.2	4.0	1.4 - 9.6	4.0	1.4 - 12.0	8.2	2.7 - 25.3	0.0	0.0 - 0.0	6.9	2.8 - 16.4	0.95
3040207	5720	*B	81.1	12.5	4 - 29	0.0	0.0 - 0.0	2.3	0.7 - 4.8	1.9	0.5 - 6.2	4.1	1.2 - 11.5	0.0	0.0 - 0.0	4.2	1.2 - 9.4	0.95
3040207	5721	*C	109.5	19.4	6 - 37	0.0	0.0 - 0.0	8.9	2.5 - 19.1	2.4	0.7 - 6.3	5.0	1.5 - 10.6	0.0	0.0 - 0.0	3.2	1.1 - 6.9	1.96
3040207	5722	SAMPIT R	513.4	76.3	24 - 192	58.6	19.5 - 162.6	4.4	1.4 - 10.4	0.6	0.2 - 1.6	2.6	0.9 - 7.7	0.0	0.0 - 0.0	10.1	3.2 - 27.1	1.00
3040207	5723	INTRACOASTAL W	246.9	48.5	16 - 86	35.9	12.2 - 79.3	8.7	2.7 - 18.5	0.5	0.1 - 1.2	1.2	0.3 - 3.1	0.0	0.0 - 0.0	2.3	0.8 - 4.7	0.97
3050101	5724	GUNPOWDER CR	95.2	53.2	15 - 103	0.0	0.0 - 0.0	38.9	11.4 - 93.5	0.6	0.2 - 1.7	4.4	1.3 - 10.2	0.0	0.0 - 0.0	9.3	3.1 - 20.2	0.20
3050101	5726	CATAWBA R	186.6	38.2	16 - 104	1.7	0.7 - 4.1	20.3	7.5 - 55.1	1.0	0.4 - 3.7	6.1	2.4 - 17.8	0.0	0.0 - 0.0	9.0	3.9 - 26.0	0.20
3050101	5727	CATAWBA R	45.6	34.4	10 - 65	0.0	0.0 - 0.0	7.1	2.1 - 15.8	4.7	1.2 - 14.4	12.5	3.8 - 31.2	0.0	0.0 - 0.0	10.1	3.3 - 21.6	0.06
3050101	5728	ELK SHOAL CR	42.1	59.2	22 - 140	0.0	0.0 - 0.0	4.3	1.4 - 9.2	23.7	8.4 - 59.5	21.8	7.0 - 54.1	0.0	0.0 - 0.0	9.4	3.7 - 21.9	0.06
3050101	5729	CATAWBA R	33.1	37.6	11 - 80	0.0	0.0 - 0.0	4.3	1.3 - 9.6	8.5	2.4 - 24.1	16.4	5.0 - 36.2	0.0	0.0 - 0.0	8.3	2.6 - 20.9	0.06
3050101	5730	LOWER LITTLE R	102.3	71.0	27 - 139	13.3	5.1 - 28.8	12.2	4.8 - 26.9	18.0	6.0 - 56.1	16.4	5.9 - 40.2	0.0	0.0 - 0.0	11.1	3.7 - 26.5	0.06
3050101	5731	L. LITTLE R, MUDI	32.4	64.0	19 - 141	0.0	0.0 - 0.0	12.3	3.7 - 31.8	21.2	6.3 - 60.0	19.6	6.2 - 45.7	0.0	0.0 - 0.0	10.9	3.5 - 26.9	0.05
3050101	5732	LOWER LITTLE R	115.6	36.5	13 - 83	0.0	0.0 - 0.0	4.7	1.4 - 10.5	10.3	3.3 - 27.5	9.4	3.2 - 21.8	0.0	0.0 - 0.0	12.0	3.5 - 31.6	0.05
3050101	5733	CATAWBA R	43.1	41.8	16 - 108	0.0	0.0 - 0.0	15.3	5.8 - 38.6	7.2	2.1 - 23.9	10.6	3.6 - 30.4	0.0	0.0 - 0.0	8.7	3.0 - 22.4	0.06
3050101	5734	M LITTLE R	156.1	31.1	11 - 72	0.0	0.0 - 0.0	6.8	2.0 - 13.5	7.0	2.1 - 18.2	7.4	2.5 - 20.9	0.0	0.0 - 0.0	9.9	3.5 - 22.1	0.04
3050101	5735	CATAWBA R	51.4	278.3	81 - 548	199.4	59.4 - 424.2	60.6	20.3 - 134.3	2.2	0.6 - 5.3	4.5	1.3 - 11.4	0.0	0.0 - 0.0	11.6	3.4 - 28.2	0.04
3050101	5736	UPPER LITTLE R	122.4	31.1	11 - 79	0.1	0.0 - 0.3	8.5	3.0 - 19.8	2.8	0.9 - 9.7	9.7	3.1 - 27.9	0.0	0.0 - 0.0	10.0	3.4 - 31.1	0.04
3050101	5737	CATAWBA R	5.1	52.7	18 - 119	0.0	0.0 - 0.0	28.3	8.9 - 63.4	1.5	0.4 - 4.1	9.0	3.2 - 23.8	0.0	0.0 - 0.0	13.8	4.6 - 36.2	0.04
3050101	5738	GUNPOWDER CR	116.3	78.1	24 - 136	27.0	7.8 - 53.5	32.6	11.0 - 60.6	1.2	0.3 - 3.3	7.5	2.4 - 17.8	0.0	0.0 - 0.0	9.8	2.9 - 19.2	0.04
3050101	5739	CATAWBA R	30.2	85.7	27 - 177	0.0	0.0 - 0.0	73.0	21.9 - 146.5	0.4	0.1 - 0.9	1.7	0.5 - 4.8	0.0	0.0 - 0.0	10.7	3.4 - 26.7	0.04
3050101	5740	CATAWBA R	202.1	112.0	38 - 198	77.4	25.4 - 150.1	19.6	5.8 - 33.9	1.1	0.3 - 2.6	5.3	1.6 - 12.3	0.0	0.0 - 0.0	8.5	2.7 - 17.5	0.04
3050101	5741	LOWER CR	254.8	61.7	26 - 150	22.2	7.8 - 49.3	18.1	7.3 - 42.7	1.3	0.4 - 3.7	6.9	2.9 - 19.0	0.0	0.0 - 0.0	13.3	5.3 - 33.4	0.03
3050101	5742	CATAWBA R	4.5	19.9	8 - 44	0.0	0.0 - 0.0	3.5	1.3 - 7.0	1.5	0.5 - 4.2	4.5	1.7 - 11.4	0.0	0.0 - 0.0	10.4	4.0 - 24.3	0.03
3050101	5743	JOHNS R	64.4	354.3	122 - 657	328.5	112.9 - 621.3	3.4	1.2 - 7.0	1.5	0.5 - 4.1	5.2	1.7 - 13.2	0.0	0.0 - 0.0	15.6	5.5 - 30.5	0.03

8-digit HUC	ID	Name	Area	Catchment Yield		Point sources		Developed Land		Manure		Agricultural Land		Phosphate Mines		Soil parent rock		Frac
				kg/km^2	90% CI	kg/km^2	90% CI	kg/km^2	90% CI	kg/km^2	90% CI	kg/km^2	90% CI	kg/km^2	90% CI	kg/km^2	90% CI	
3050101	5744	JOHNS R	14.7	24.6	9 - 44	0.0	0.0 - 0.0	2.4	0.8 - 4.6	0.5	0.1 - 1.1	3.3	1.1 - 8.0	0.0	0.0 - 0.0	18.4	6.1 - 35.5	0.03
3050101	5745	MULBERRY CR	106.2	18.1	5 - 45	0.0	0.0 - 0.0	2.6	0.7 - 6.2	0.4	0.1 - 1.2	2.6	0.7 - 6.4	0.0	0.0 - 0.0	12.6	4.3 - 29.5	0.03
3050101	5746	JOHNS R	89.2	17.6	6 - 38	0.2	0.1 - 0.5	2.0	0.7 - 3.8	0.2	0.1 - 0.7	1.4	0.5 - 3.6	0.0	0.0 - 0.0	13.7	4.5 - 31.8	0.03
3050101	5747	JOHNS R	49.4	17.1	6 - 39	0.0	0.0 - 0.0	4.2	1.4 - 9.1	0.1	0.0 - 0.3	0.8	0.3 - 1.9	0.0	0.0 - 0.0	12.0	4.5 - 27.2	0.03
3050101	5748	*A	38.9	15.9	5 - 35	0.0	0.0 - 0.0	4.6	1.6 - 9.8	0.1	0.0 - 0.2	0.7	0.3 - 1.7	0.0	0.0 - 0.0	10.6	3.8 - 25.1	0.03
3050101	5749	WILSON CR	178.5	15.9	5 - 44	0.0	0.0 - 0.0	2.5	0.8 - 5.9	0.1	0.0 - 0.2	0.5	0.2 - 1.4	0.0	0.0 - 0.0	12.8	3.8 - 36.2	0.03
3050101	5751	CATAWBA R	4.4	53.8	18 - 130	0.0	0.0 - 0.0	39.9	12.7 - 99.4	0.7	0.2 - 2.1	2.4	0.8 - 6.0	0.0	0.0 - 0.0	10.8	3.7 - 26.5	0.03
3050101	5752	CATAWBA R, WAF	25.5	56.7	20 - 122	0.0	0.0 - 0.0	16.8	5.7 - 39.1	3.5	1.0 - 10.6	12.4	4.5 - 30.2	0.0	0.0 - 0.0	24.0	8.9 - 63.4	0.03
3050101	5753	CATAWBA R, WAF	15.3	32.2	13 - 69	0.0	0.0 - 0.0	5.5	2.1 - 12.0	2.9	0.9 - 8.3	10.4	4.3 - 27.4	0.0	0.0 - 0.0	13.4	5.1 - 35.2	0.03
3050101	5754	UPPER CR	52.2	16.0	5 - 44	0.0	0.0 - 0.0	3.3	1.1 - 7.6	0.3	0.1 - 0.9	1.2	0.4 - 3.5	0.0	0.0 - 0.0	11.2	3.4 - 36.6	0.03
3050101	5755	CATAWBA R, WAF	44.3	14.6	5 - 32	0.0	0.0 - 0.0	3.1	1.0 - 7.0	0.1	0.0 - 0.4	0.5	0.2 - 1.3	0.0	0.0 - 0.0	10.8	3.8 - 24.1	0.03
3050101	5756	IRISH CR	89.1	19.8	6 - 44	0.0	0.0 - 0.0	3.8	1.0 - 9.8	0.8	0.3 - 2.6	2.8	0.8 - 7.2	0.0	0.0 - 0.0	12.4	4.2 - 33.1	0.03
3050101	5757	CATAWBA R	10.0	100.9	29 - 189	0.0	0.0 - 0.0	70.2	18.9 - 139.8	2.1	0.6 - 4.7	7.6	2.3 - 16.0	0.0	0.0 - 0.0	21.0	6.3 - 50.7	0.03
3050101	5758	CATAWBA R	91.8	42.0	14 - 76	0.0	0.0 - 0.0	14.6	4.9 - 27.5	2.2	0.6 - 5.2	7.3	2.4 - 19.6	0.0	0.0 - 0.0	18.0	6.5 - 38.9	0.03
3050101	5759	CATAWBA R	18.2	38.5	13 - 74	0.0	0.0 - 0.0	14.3	4.9 - 26.0	2.3	0.6 - 6.2	7.5	2.4 - 19.9	0.0	0.0 - 0.0	14.5	4.1 - 31.3	0.03
3050101	5760	BUCK CR	68.3	15.3	5 - 34	0.0	0.0 - 0.0	2.7	0.7 - 5.8	0.3	0.1 - 0.8	1.0	0.3 - 2.5	0.0	0.0 - 0.0	11.4	3.5 - 25.0	0.01
3050101	5761	CATAWBA R	3.6	48.9	14 - 119	0.0	0.0 - 0.0	25.5	7.6 - 59.8	3.0	0.9 - 9.7	10.1	3.2 - 27.6	0.0	0.0 - 0.0	10.2	3.3 - 26.1	0.01
3050101	5762	CATAWBA R	13.5	26.4	9 - 64	0.0	0.0 - 0.0	8.8	2.7 - 20.6	1.9	0.6 - 5.6	6.4	1.8 - 17.6	0.0	0.0 - 0.0	9.3	3.0 - 26.0	0.01
3050101	5763	CURTIS CR	45.0	13.8	6 - 34	0.0	0.0 - 0.0	2.4	0.9 - 5.8	0.1	0.0 - 0.3	0.4	0.2 - 1.2	0.0	0.0 - 0.0	10.8	4.5 - 27.4	0.01
3050101	5764	CATAWBA R	4.3	259.5	87 - 562	231.7	78.4 - 520.7	13.4	4.1 - 31.1	1.9	0.5 - 5.6	6.4	2.0 - 17.5	0.0	0.0 - 0.0	6.1	1.9 - 14.1	0.01
3050101	5765	MILL CR	56.3	13.5	4 - 29	0.0	0.0 - 0.0	2.8	0.8 - 5.4	0.1	0.0 - 0.3	0.3	0.1 - 0.7	0.0	0.0 - 0.0	10.3	2.9 - 24.2	0.01
3050101	5766	CATAWBA R	38.4	14.7	5 - 25	0.0	0.0 - 0.0	5.9	1.9 - 11.7	0.4	0.1 - 1.0	1.2	0.4 - 2.9	0.0	0.0 - 0.0	7.2	2.7 - 14.1	0.01
3050101	5767	CROOKED CR	92.6	20.0	7 - 44	0.0	0.0 - 0.0	4.5	1.7 - 9.3	1.2	0.4 - 3.5	4.0	1.5 - 11.4	0.0	0.0 - 0.0	10.3	3.7 - 25.3	0.01
3050101	5768	MUDDY CR	4.9	49.6	16 - 113	0.0	0.0 - 0.0	16.9	5.5 - 33.9	2.1	0.6 - 5.2	7.0	1.9 - 18.5	0.0	0.0 - 0.0	23.5	7.1 - 57.5	0.03
3050101	5769	N MUDDY CR	146.1	43.2	13 - 75	8.5	2.5 - 15.9	12.7	4.0 - 23.3	2.2	0.6 - 4.9	7.4	2.2 - 16.2	0.0	0.0 - 0.0	12.3	3.8 - 26.7	0.03
3050101	5770	S MUDDY CR	102.8	29.2	11 - 61	0.0	0.0 - 0.0	5.6	2.0 - 12.0	1.9	0.6 - 5.6	6.5	2.4 - 15.9	0.0	0.0 - 0.0	15.1	5.7 - 39.0	0.03
3050101	5771	SILVER CR	158.3	41.6	11 - 76	0.0	0.0 - 0.0	18.7	5.0 - 42.8	2.1	0.5 - 6.0	7.4	2.1 - 18.3	0.0	0.0 - 0.0	13.4	3.5 - 32.0	0.03
3050101	5772	HUNTING CR	65.7	53.0	20 - 110	0.0	0.0 - 0.0	35.9	12.0 - 77.4	1.6	0.5 - 4.8	5.5	2.2 - 14.7	0.0	0.0 - 0.0	10.0	3.8 - 27.5	0.03
3050101	5773	DROWNING CR	46.4	56.6	23 - 118	0.0	0.0 - 0.0	36.8	12.6 - 72.7	2.3	0.8 - 6.9	7.7	2.7 - 20.0	0.0	0.0 - 0.0	9.8	4.0 - 22.6	0.04
3050101	5774	LYLE CR	6.6	73.0	25 - 135	25.6	8.1 - 53.1	15.0	5.7 - 30.9	3.7	1.1 - 9.8	16.4	5.9 - 40.5	0.0	0.0 - 0.0	12.4	4.4 - 28.4	0.06
3050101	5775	LYLE CR	117.0	64.7	20 - 112	11.7	3.9 - 23.7	26.5	7.6 - 53.2	3.1	0.9 - 7.5	14.1	4.3 - 31.7	0.0	0.0 - 0.0	9.4	3.0 - 20.8	0.06
3050101	5776	*B	68.0	64.4	19 - 146	20.6	6.2 - 53.4	16.7	4.7 - 38.8	3.0	0.8 - 9.0	13.4	3.9 - 37.5	0.0	0.0 - 0.0	10.7	3.4 - 27.5	0.06
3050101	5777	DUTCHMANS CR	79.1	36.6	12 - 67	0.0	0.0 - 0.0	19.2	5.8 - 36.3	1.5	0.5 - 4.6	5.9	1.8 - 13.3	0.0	0.0 - 0.0	10.0	3.4 - 22.0	0.20
3050101	5778	DUTCHMANS CR	41.3	69.7	28 - 149	42.6	15.7 - 98.8	11.7	4.1 - 23.5	1.6	0.5 - 4.3	6.1	2.1 - 15.5	0.0	0.0 - 0.0	7.8	3.0 - 19.2	0.19
3050101	5779	KILLIAN CR	36.1	26.1	7 - 65	0.0	0.0 - 0.0	9.9	2.8 - 24.7	1.5	0.4 - 3.6	6.5	1.7 - 16.5	0.0	0.0 - 0.0	8.2	2.3 - 21.8	0.19
3050101	5780	ANDERSON CR	57.1	26.8	10 - 47	0.0	0.0 - 0.0	4.4	1.7 - 8.1	2.4	0.9 - 6.5	10.0	3.6 - 24.8	0.0	0.0 - 0.0	10.0	3.7 - 22.4	0.19
3050101	5781	LEEPERS CR	152.3	33.4	12 - 62	0.0	0.0 - 0.1	8.0	2.7 - 18.3	2.8	0.9 - 7.6	11.9	4.2 - 34.4	0.0	0.0 - 0.0	10.7	3.6 - 25.4	0.19
3050101	5782	CROWDERS CR	37.3	67.3	26 - 172	34.1	12.9 - 95.6	9.1	3.1 - 20.0	2.4	0.6 - 6.0	10.7	3.8 - 30.8	0.0	0.0 - 0.0	10.9	3.6 - 27.3	0.20
3050101	5783	*C	18.6	54.9	20 - 98	0.5	0.2 - 1.0	33.1	11.7 - 62.3	1.9	0.6 - 5.2	8.0	2.9 - 17.3	0.0	0.0 - 0.0	11.5	4.1 - 26.0	0.19

8-digit HUC	ID	Name	Area	Catchment Yield		Point sources		Developed Land		Manure		Agricultural Land		Phosphate Mines		Soil parent rock		Frac
				kg/km^2	90% CI	kg/km^2	90% CI	kg/km^2	90% CI	kg/km^2	90% CI	kg/km^2	90% CI	kg/km^2	90% CI	kg/km^2	90% CI	
3050101	5784	CROWDERS CR	180.2	39.5	12 - 96	0.2	0.0 - 0.4	20.8	6.8 - 50.9	1.5	0.4 - 4.0	6.4	1.8 - 14.8	0.0	0.0 - 0.0	10.7	3.1 - 26.5	0.19
3050101	5785	BEAVERDAM CR	47.7	36.1	13 - 70	0.0	0.0 - 0.1	11.0	3.4 - 22.1	2.6	0.8 - 7.1	11.3	3.7 - 28.0	0.0	0.0 - 0.0	11.1	3.8 - 25.5	0.20
3050102	5786	CATAWBA R, S FK	9.8	34.5	13 - 78	0.0	0.0 - 0.0	9.5	3.4 - 21.1	2.7	0.8 - 7.1	11.1	4.5 - 24.3	0.0	0.0 - 0.0	11.2	3.5 - 28.3	0.20
3050102	5787	LITTLE HOYLE CR	75.6	39.9	12 - 82	4.1	1.3 - 8.6	12.7	3.6 - 28.2	2.5	0.7 - 7.7	10.9	3.1 - 34.3	0.0	0.0 - 0.0	9.6	2.8 - 22.3	0.20
3050102	5788	CATAWBA R, S FK	78.1	42.1	14 - 89	0.6	0.2 - 1.3	13.3	4.3 - 30.5	2.8	0.8 - 8.2	11.9	3.7 - 31.1	0.0	0.0 - 0.0	13.6	4.4 - 31.4	0.20
3050102	5789	CATAWBA R, S FK	5.8	43.6	15 - 86	0.0	0.0 - 0.0	15.8	5.4 - 33.1	2.7	0.8 - 8.0	12.5	4.1 - 29.8	0.0	0.0 - 0.0	12.5	4.0 - 30.9	0.19
3050102	5790	CATAWBA R, S FK	31.2	325.5	118 - 827	251.9	91.2 - 622.3	49.4	17.0 - 124.4	1.9	0.5 - 6.1	8.7	2.9 - 23.8	0.0	0.0 - 0.0	13.7	5.0 - 37.6	0.19
3050102	5791	CLARK CR	66.4	57.4	20 - 133	0.0	0.0 - 0.0	27.4	8.8 - 66.0	3.3	1.0 - 8.9	14.8	4.8 - 37.8	0.0	0.0 - 0.0	11.9	3.8 - 28.6	0.19
3050102	5792	MAIDEN CR	68.4	56.5	19 - 136	14.7	5.2 - 39.2	13.6	4.3 - 31.0	3.3	1.1 - 8.9	14.8	4.7 - 41.6	0.0	0.0 - 0.0	10.0	3.6 - 29.5	0.17
3050102	5793	CLARK CR	102.3	109.1	33 - 210	49.4	15.2 - 98.6	36.8	10.6 - 71.7	2.3	0.6 - 5.8	10.7	3.3 - 26.5	0.0	0.0 - 0.0	9.9	2.9 - 22.0	0.17
3050102	5794	CATAWBA R, S FK	10.8	67.9	21 - 168	0.0	0.0 - 0.0	15.2	5.0 - 31.4	7.0	2.0 - 19.9	31.6	9.1 - 87.0	0.0	0.0 - 0.0	14.2	4.6 - 35.0	0.19
3050102	5795	CATAWBA R, S FK	54.7	51.2	19 - 101	0.0	0.0 - 0.0	5.7	2.0 - 11.1	5.9	1.8 - 16.9	28.0	9.7 - 70.1	0.0	0.0 - 0.0	11.5	3.9 - 27.9	0.18
3050102	5796	CATAWBA R, S FK	297.1	43.1	17 - 82	10.5	4.4 - 23.7	15.6	5.9 - 31.5	1.4	0.5 - 3.7	5.4	2.0 - 12.9	0.0	0.0 - 0.0	10.3	3.9 - 21.6	0.18
3050102	5797	CATAWBA R, S FK	47.8	34.6	10 - 84	0.0	0.0 - 0.0	11.5	3.2 - 26.8	2.4	0.6 - 7.7	10.6	3.4 - 30.4	0.0	0.0 - 0.0	10.0	3.5 - 27.1	0.18
3050102	5798	CAMP CR	26.7	28.7	9 - 69	0.1	0.0 - 0.3	6.1	2.0 - 15.4	2.6	0.8 - 8.2	10.2	3.2 - 25.5	0.0	0.0 - 0.0	9.6	3.0 - 24.8	0.16
3050102	5799	CATAWBA R, S FK	63.7	31.0	9 - 72	0.0	0.0 - 0.0	5.3	1.6 - 13.6	2.9	0.9 - 8.3	12.6	4.1 - 33.4	0.0	0.0 - 0.0	10.2	3.5 - 26.3	0.16
3050102	5800	WHITENER CR	17.0	24.8	9 - 62	0.0	0.0 - 0.0	5.2	1.8 - 14.0	2.0	0.5 - 6.6	7.2	2.3 - 19.9	0.0	0.0 - 0.0	10.4	3.4 - 28.6	0.15
3050102	5801	CATAWBA R, S FK	28.4	28.7	10 - 61	0.0	0.0 - 0.0	6.0	2.1 - 14.1	2.6	0.8 - 7.9	9.4	3.4 - 22.1	0.0	0.0 - 0.0	10.8	4.1 - 24.1	0.15
3050102	5802	*A	69.4	36.5	12 - 67	0.7	0.2 - 1.5	5.5	1.7 - 10.8	3.8	1.1 - 10.6	16.7	5.5 - 43.0	0.0	0.0 - 0.0	9.8	3.2 - 24.7	0.18
3050102	5803	HOWARDS CR	19.8	59.5	17 - 138	0.0	0.0 - 0.0	9.6	2.7 - 22.4	6.5	1.7 - 17.5	29.9	9.0 - 89.6	0.0	0.0 - 0.0	13.6	3.8 - 29.6	0.19
3050102	5804	*B	18.9	44.1	18 - 106	0.0	0.0 - 0.0	4.5	1.6 - 12.2	5.0	1.7 - 15.9	22.3	8.1 - 63.7	0.0	0.0 - 0.0	12.2	5.2 - 29.7	0.18
3050102	5805	HOWARDS CR	49.0	41.6	16 - 82	0.0	0.0 - 0.0	5.4	2.0 - 10.0	4.5	1.4 - 13.4	20.1	6.9 - 50.6	0.0	0.0 - 0.0	11.6	4.4 - 24.3	0.18
3050102	5807	INDIAN CR, LEON	21.7	45.6	16 - 77	0.0	0.0 - 0.0	5.3	1.9 - 9.5	5.0	1.6 - 13.6	22.5	6.9 - 50.4	0.0	0.0 - 0.0	12.8	4.7 - 24.3	0.18
3050102	5808	INDIAN CR	32.4	97.6	32 - 216	44.9	14.8 - 88.5	15.2	4.8 - 37.5	4.2	1.1 - 12.2	18.8	6.2 - 46.9	0.0	0.0 - 0.0	14.3	4.4 - 33.3	0.18
3050102	5809	INDIAN CR	85.6	43.0	16 - 104	0.3	0.1 - 0.7	6.2	2.2 - 15.3	4.5	1.4 - 11.5	19.8	7.9 - 60.0	0.0	0.0 - 0.0	12.2	4.7 - 31.7	0.17
3050102	5810	BEAVERDAM CR	20.8	36.6	12 - 94	0.0	0.0 - 0.0	4.7	1.6 - 13.2	3.6	1.0 - 12.6	15.8	5.2 - 44.0	0.0	0.0 - 0.0	12.6	4.3 - 33.7	0.19
3050102	5811	BEAVERDAM CR	23.0	44.8	16 - 120	0.0	0.0 - 0.0	13.6	4.6 - 34.7	3.5	1.0 - 9.9	16.2	5.7 - 45.5	0.0	0.0 - 0.0	11.4	4.1 - 28.5	0.17
3050102	5812	LITTLE BEAVERD	17.8	40.2	14 - 97	0.0	0.0 - 0.0	6.6	2.1 - 16.6	3.8	1.1 - 12.0	17.4	5.4 - 49.4	0.0	0.0 - 0.0	12.4	4.5 - 35.3	0.17
3050102	5813	LITTLE LONG CR	1.5	14.9	5 - 35	0.0	0.0 - 0.0	3.6	1.0 - 8.5	0.3	0.1 - 0.9	0.3	0.1 - 0.9	0.0	0.0 - 0.0	10.8	3.3 - 31.0	0.20
3050102	5814	LONG CR	23.8	49.9	15 - 130	0.8	0.2 - 2.0	23.2	7.5 - 55.7	2.4	0.7 - 5.9	10.1	3.1 - 29.5	0.0	0.0 - 0.0	13.4	4.2 - 33.9	0.20
3050102	5815	LITTLE LONG CR	51.4	135.5	46 - 244	56.6	20.5 - 120.8	54.3	17.9 - 108.3	1.5	0.5 - 3.6	6.4	2.1 - 15.6	0.0	0.0 - 0.0	16.7	5.9 - 30.7	0.20
3050102	5816	CATAWBA R, S FK	2.2	101.2	35 - 162	0.0	0.0 - 0.0	73.7	25.4 - 128.8	2.2	0.7 - 5.6	9.8	3.3 - 20.6	0.0	0.0 - 0.0	15.5	5.3 - 32.6	0.19
3050102	5817	MILL CR	18.3	45.8	16 - 79	0.0	0.0 - 0.0	7.1	2.5 - 11.9	4.6	1.3 - 10.3	21.1	7.6 - 43.0	0.0	0.0 - 0.0	13.0	4.9 - 26.3	0.17
3050103	5819	CEDAR CR	85.7	13.9	5 - 29	0.0	0.0 - 0.0	1.7	0.6 - 3.9	0.8	0.2 - 2.1	1.3	0.4 - 3.3	0.0	0.0 - 0.0	10.2	3.2 - 22.8	0.32
3050103	5822	CAMP CR	114.9	25.0	8 - 51	0.0	0.0 - 0.0	3.1	0.9 - 6.0	2.7	0.7 - 6.7	5.7	1.7 - 13.5	0.0	0.0 - 0.0	13.7	4.0 - 31.1	0.31
3050103	5823	CATAWBA R	2.8	45.1	14 - 111	0.0	0.0 - 0.0	30.4	9.4 - 80.9	0.6	0.2 - 1.7	1.7	0.6 - 5.3	0.0	0.0 - 0.0	12.4	4.4 - 32.6	0.31
3050103	5824	CANE CR	44.2	97.2	27 - 249	57.0	16.7 - 155.2	18.0	4.3 - 40.9	2.6	0.6 - 9.1	5.4	1.9 - 13.3	0.0	0.0 - 0.0	14.2	4.5 - 37.2	0.29
3050103	5825	BEAR CR	173.1	49.2	16 - 112	0.0	0.0 - 0.0	16.0	5.0 - 37.7	6.5	1.9 - 20.2	14.4	4.7 - 32.3	0.0	0.0 - 0.0	12.4	4.2 - 28.8	0.27
3050103	5826	CANE CR	2.6	48.4	16 - 85	0.0	0.0 - 0.0	31.0	10.4 - 54.1	0.8	0.2 - 2.0	2.6	0.8 - 5.5	0.0	0.0 - 0.0	14.0	4.6 - 30.0	0.27

8-digit HUC	ID	Name	Area	Catchment Yield kg/km^2	Catchment Yield 90% CI	Point sources kg/km^2	Point sources 90% CI	Developed Land kg/km^2	Developed Land 90% CI	Manure kg/km^2	Manure 90% CI	Agricultural Land kg/km^2	Agricultural Land 90% CI	Phosphate Mines kg/km^2	Phosphate Mines 90% CI	Soil parent rock kg/km^2	Soil parent rock 90% CI	Frac
3050103	5827	CAMP CR	73.2	37.6	13 - 61	0.7	0.2 - 1.3	7.9	2.4 - 16.0	5.3	1.7 - 12.6	11.7	3.9 - 24.4	0.0	0.0 - 0.0	12.0	4.2 - 22.4	0.26
3050103	5828	CANE CR	130.8	15.4	5 - 31	0.0	0.0 - 0.0	1.9	0.6 - 4.3	3.9	1.1 - 11.9	4.6	1.4 - 11.5	0.0	0.0 - 0.0	5.0	1.5 - 12.5	0.26
3050103	5829	CATAWBA R	2.7	41.8	17 - 71	0.0	0.0 - 0.0	23.2	9.1 - 49.2	1.2	0.4 - 3.3	4.2	1.7 - 9.7	0.0	0.0 - 0.0	13.1	4.7 - 30.2	0.29
3050103	5830	WAXHAW CR	135.4	31.1	11 - 70	0.0	0.0 - 0.0	4.5	1.4 - 11.3	7.5	2.4 - 23.0	8.7	3.0 - 25.7	0.0	0.0 - 0.0	10.5	3.5 - 25.1	0.29
3050103	5831	CATAWBA R	35.7	544.2	180 - 1190	520.5	170.2 - 1149.3	5.2	1.6 - 11.2	1.4	0.4 - 3.9	4.5	1.6 - 11.0	0.0	0.0 - 0.0	12.6	4.1 - 30.4	0.29
3050103	5832	TWELVEMILE CR	125.2	56.7	18 - 103	21.7	6.5 - 41.8	7.4	2.1 - 15.8	6.7	1.7 - 15.6	9.2	2.8 - 23.2	0.0	0.0 - 0.0	11.6	4.0 - 30.0	0.29
3050103	5833	CATAWBA R	76.0	34.8	12 - 85	1.3	0.4 - 3.1	8.1	2.6 - 18.8	2.9	0.9 - 10.0	9.2	2.9 - 28.1	0.0	0.0 - 0.0	13.3	4.3 - 36.8	0.29
3050103	5834	SUGAR CR	2.1	20.3	8 - 47	0.0	0.0 - 0.0	3.3	1.1 - 7.8	1.9	0.6 - 5.8	5.6	1.9 - 16.0	0.0	0.0 - 0.0	9.5	3.7 - 27.0	0.29
3050103	5835	SIXMILE CR	70.4	64.9	21 - 144	0.0	0.0 - 0.0	40.6	13.1 - 90.7	4.6	1.3 - 12.6	8.1	2.7 - 21.0	0.0	0.0 - 0.0	11.6	3.2 - 32.2	0.29
3050103	5836	SUGAR CR	39.8	42.5	13 - 70	0.0	0.0 - 0.0	19.2	5.9 - 32.1	2.1	0.5 - 5.3	6.1	2.0 - 14.0	0.0	0.0 - 0.0	15.1	4.6 - 32.7	0.29
3050103	5837	SUGAR CR	3.3	53.2	18 - 96	0.0	0.0 - 0.0	23.7	8.1 - 44.0	3.4	1.1 - 9.3	7.5	2.2 - 15.4	0.0	0.0 - 0.0	18.5	6.4 - 40.2	0.28
3050103	5838	MCALPINE CR	7.9	55.9	15 - 126	0.0	0.0 - 0.0	32.6	9.2 - 71.8	2.3	0.5 - 6.3	5.4	1.7 - 13.7	0.0	0.0 - 0.0	15.5	4.3 - 39.9	0.28
3050103	5839	FOURMILE CR	48.8	77.0	25 - 193	0.0	0.0 - 0.0	65.2	20.2 - 165.6	0.2	0.1 - 0.5	1.5	0.5 - 3.7	0.0	0.0 - 0.0	10.1	3.4 - 22.2	0.27
3050103	5840	MCALPINE CR	35.0	94.9	24 - 214	0.0	0.0 - 0.0	81.9	20.9 - 201.9	0.0	0.0 - 0.1	0.3	0.1 - 0.6	0.0	0.0 - 0.0	12.7	3.7 - 32.2	0.27
3050103	5841	SUGAR CR	1.6	60.4	20 - 148	0.0	0.0 - 0.0	39.6	13.2 - 99.1	0.5	0.1 - 1.6	2.1	0.8 - 4.9	0.0	0.0 - 0.0	18.2	6.0 - 45.2	0.28
3050103	5842	LITTLE SUGAR CR	6.2	56.7	22 - 122	0.0	0.0 - 0.0	40.3	14.8 - 91.2	0.5	0.2 - 1.4	1.1	0.4 - 2.9	0.0	0.0 - 0.0	14.7	5.3 - 37.2	0.28
3050103	5843	SUGAR CR	14.2	76.7	24 - 139	7.1	2.2 - 14.3	42.8	14.1 - 80.6	1.9	0.5 - 5.2	9.8	2.7 - 19.3	0.0	0.0 - 0.0	15.1	4.8 - 28.6	0.28
3050103	5844	SUGAR CR, KING'S	114.8	259.0	88 - 606	175.4	58.0 - 428.6	70.3	22.7 - 172.2	0.2	0.0 - 0.4	1.2	0.4 - 3.1	0.0	0.0 - 0.0	11.9	4.1 - 28.0	0.26
3050103	5845	SUGAR CR	27.4	60.9	19 - 151	0.0	0.0 - 0.0	47.7	14.4 - 120.4	0.3	0.1 - 0.9	2.2	0.6 - 6.3	0.0	0.0 - 0.0	10.6	3.2 - 30.3	0.26
3050103	5846	STEELE CR	87.8	57.9	20 - 147	0.0	0.0 - 0.0	38.1	12.5 - 91.1	1.2	0.3 - 3.5	6.3	2.0 - 15.7	0.0	0.0 - 0.0	12.3	4.3 - 30.1	0.28
3050103	5847	CATAWBA R	5.5	20.8	7 - 46	0.0	0.0 - 0.0	1.2	0.4 - 3.0	1.4	0.4 - 4.3	5.7	2.0 - 13.3	0.0	0.0 - 0.0	12.5	4.2 - 33.5	0.29
3050103	5848	CATAWBA R	71.9	344.4	115 - 778	280.3	94.6 - 638.4	46.9	15.2 - 113.9	0.7	0.2 - 2.1	2.6	0.8 - 7.5	0.0	0.0 - 0.0	13.8	3.8 - 29.1	0.29
3050103	5849	BURGIS CR	17.9	35.0	12 - 84	0.3	0.1 - 0.7	13.1	3.4 - 33.8	2.0	0.6 - 5.8	8.6	3.1 - 22.7	0.0	0.0 - 0.0	11.0	3.9 - 28.4	0.29
3050103	5850	TINKER'S CR	69.7	23.4	7 - 53	0.0	0.0 - 0.1	5.3	1.5 - 11.8	1.8	0.5 - 6.2	7.3	2.3 - 21.6	0.0	0.0 - 0.0	9.1	2.8 - 25.5	0.26
3050103	5851	FISHING CR	71.3	33.2	10 - 68	5.3	1.8 - 11.3	6.0	1.7 - 12.1	1.9	0.5 - 5.7	7.8	2.4 - 16.8	0.0	0.0 - 0.0	12.2	4.2 - 26.4	0.26
3050103	5852	FISHING CR	59.8	32.2	11 - 60	0.0	0.0 - 0.0	7.2	2.3 - 14.6	2.5	0.7 - 6.7	10.2	2.9 - 24.1	0.0	0.0 - 0.0	12.3	3.8 - 28.7	0.24
3050103	5853	*A	49.6	41.2	15 - 107	0.0	0.0 - 0.0	22.4	8.5 - 61.5	1.4	0.4 - 3.7	5.6	1.9 - 15.6	0.0	0.0 - 0.0	11.8	4.4 - 28.5	0.21
3050103	5854	FISHING CR	43.8	47.6	16 - 103	0.0	0.0 - 0.0	6.0	1.9 - 15.4	5.6	1.6 - 18.6	23.8	8.1 - 70.0	0.0	0.0 - 0.0	12.3	3.6 - 31.9	0.21
3050103	5855	FISHING CR	13.8	63.1	23 - 128	0.0	0.0 - 0.0	40.8	13.7 - 88.1	1.6	0.5 - 4.8	5.7	2.1 - 15.1	0.0	0.0 - 0.0	15.1	5.8 - 31.4	0.20
3050103	5856	FISHING CR, TOOL	26.6	69.6	25 - 125	0.0	0.0 - 0.0	55.5	18.4 - 109.7	0.7	0.2 - 1.9	2.9	1.0 - 7.5	0.0	0.0 - 0.0	10.5	4.3 - 23.0	0.18
3050103	5857	FISHING CR	39.6	42.9	14 - 82	0.5	0.2 - 1.1	19.4	5.4 - 40.5	2.5	0.6 - 6.3	10.9	4.0 - 22.8	0.0	0.0 - 0.0	9.5	2.9 - 19.6	0.18
3050103	5858	FISHING CR, STON	128.3	44.3	15 - 95	9.5	3.4 - 20.6	10.8	3.5 - 27.5	2.8	0.8 - 8.1	12.3	4.2 - 31.5	0.0	0.0 - 0.0	8.8	3.0 - 23.8	0.20
3050103	5859	FISHING CR, S FK	171.2	33.9	13 - 90	0.0	0.0 - 0.0	4.5	1.6 - 11.5	3.6	1.1 - 11.2	15.4	6.0 - 44.0	0.0	0.0 - 0.0	10.3	3.8 - 24.7	0.24
3050103	5860	ROCKY CR	1.2	15.1	4 - 32	0.0	0.0 - 0.0	3.0	0.8 - 6.1	0.4	0.1 - 1.1	1.8	0.5 - 4.2	0.0	0.0 - 0.0	9.9	3.1 - 23.5	0.32
3050103	5861	ROCKY CR	3.0	30.0	11 - 64	0.0	0.0 - 0.0	8.8	2.5 - 21.9	2.0	0.6 - 6.7	6.3	2.0 - 16.2	0.0	0.0 - 0.0	12.9	4.5 - 27.6	0.31
3050103	5862	BEAVERDAM CR	50.4	24.8	7 - 57	0.0	0.0 - 0.0	3.3	0.9 - 7.6	1.8	0.4 - 5.1	7.0	2.1 - 18.2	0.0	0.0 - 0.0	12.6	3.2 - 33.5	0.30
3050103	5863	ROCKY CR	275.8	37.2	13 - 76	9.5	3.3 - 21.8	6.7	2.2 - 15.5	1.8	0.5 - 6.0	6.5	2.1 - 16.1	0.0	0.0 - 0.0	12.8	4.1 - 31.5	0.30
3050103	5864	LITTLE ROCKY CR	20.2	25.1	9 - 48	0.0	0.0 - 0.0	5.8	2.0 - 11.0	1.3	0.4 - 3.8	5.0	1.8 - 13.1	0.0	0.0 - 0.0	13.0	5.0 - 29.3	0.31
3050103	5865	LITTLE ROCKY CR	84.3	19.8	7 - 52	0.0	0.0 - 0.0	2.9	0.9 - 6.8	1.2	0.3 - 3.8	3.7	1.1 - 10.2	0.0	0.0 - 0.0	12.1	4.3 - 36.3	0.29

8-digit HUC	ID	Name	Area	Catchment Yield kg/km^2	Catchment Yield 90% CI	Point sources kg/km^2	Point sources 90% CI	Developed Land kg/km^2	Developed Land 90% CI	Manure kg/km^2	Manure 90% CI	Agricultural Land kg/km^2	Agricultural Land 90% CI	Phosphate Mines kg/km^2	Phosphate Mines 90% CI	Soil parent rock kg/km^2	Soil parent rock 90% CI	Frac
3050103	5866	LITTLE ROCKY CR	38.2	17.1	5 - 40	0.0	0.0 - 0.0	2.0	0.6 - 4.5	0.9	0.2 - 2.7	2.1	0.6 - 5.3	0.0	0.0 - 0.0	12.2	3.7 - 30.5	0.29
3050104	5867	WATEREE R	252.8	142.8	46 - 283	129.0	40.4 - 271.8	1.0	0.3 - 2.0	0.3	0.1 - 0.7	2.4	0.7 - 6.4	0.0	0.0 - 0.0	10.0	3.0 - 21.3	0.44
3050104	5868	WATEREE R, ROBI	92.9	46.7	19 - 101	31.9	12.7 - 75.4	3.7	1.2 - 8.4	0.5	0.1 - 1.6	3.0	1.1 - 9.6	0.0	0.0 - 0.0	7.6	2.7 - 18.2	0.43
3050104	5869	WATEREE R	9.1	12.2	4 - 27	0.0	0.0 - 0.0	3.6	0.9 - 7.6	0.1	0.0 - 0.4	1.4	0.4 - 3.9	0.0	0.0 - 0.0	7.1	2.3 - 15.4	0.43
3050104	5871	WATEREE R	39.1	21.6	6 - 47	0.0	0.0 - 0.0	1.8	0.6 - 4.6	1.0	0.3 - 3.0	6.6	1.7 - 18.2	0.0	0.0 - 0.0	12.2	3.3 - 29.4	0.43
3050104	5872	RAFTING CR	13.4	23.9	7 - 48	0.0	0.0 - 0.0	3.8	1.2 - 7.5	1.4	0.5 - 3.6	9.0	3.3 - 22.1	0.0	0.0 - 0.0	9.7	3.3 - 22.3	0.43
3050104	5873	RAFTING CR	56.4	11.6	4 - 25	0.0	0.0 - 0.1	3.7	1.3 - 8.2	0.4	0.1 - 1.2	2.4	0.7 - 5.3	0.0	0.0 - 0.0	5.1	1.6 - 11.7	0.41
3050104	5874	*A	77.1	13.9	5 - 25	0.0	0.0 - 0.0	2.8	0.9 - 5.7	0.5	0.2 - 1.2	3.8	1.3 - 7.8	0.0	0.0 - 0.0	6.8	2.6 - 14.7	0.41
3050104	5875	WATEREE R	1.1	16.2	6 - 43	0.0	0.0 - 0.0	0.0	0.0 - 0.0	0.0	0.0 - 0.0	0.0	0.0 - 0.0	0.0	0.0 - 0.0	16.2	5.6 - 42.7	0.43
3050104	5876	SWIFT CR	170.6	19.3	6 - 44	0.0	0.0 - 0.0	3.7	1.1 - 8.5	2.1	0.6 - 6.2	6.7	2.0 - 16.8	0.0	0.0 - 0.0	6.8	2.1 - 16.4	0.43
3050104	5877	WATEREE R	172.2	28.6	9 - 56	2.5	0.9 - 4.7	4.3	1.2 - 9.4	3.1	1.0 - 6.9	6.3	2.0 - 16.5	0.0	0.0 - 0.0	12.3	3.6 - 30.0	0.43
3050104	5878	BIG PINE TREE CR	178.2	19.2	7 - 36	0.0	0.0 - 0.0	9.6	3.4 - 20.4	1.2	0.4 - 2.9	2.3	0.8 - 5.0	0.0	0.0 - 0.0	6.2	2.2 - 13.8	0.42
3050104	5879	WATEREE R	34.6	147.5	50 - 332	99.7	34.3 - 231.1	27.9	9.3 - 71.2	2.5	0.9 - 6.6	5.1	1.6 - 13.1	0.0	0.0 - 0.0	12.2	4.3 - 31.2	0.42
3050104	5881	GRANNYS QUART	69.0	11.4	5 - 28	0.0	0.0 - 0.0	3.6	1.4 - 7.9	0.9	0.3 - 2.8	1.7	0.6 - 4.5	0.0	0.0 - 0.0	5.1	2.1 - 13.6	0.24
3050104	5882	FLAT ROCK CR	77.2	13.8	5 - 33	0.0	0.0 - 0.0	3.0	1.0 - 7.5	0.9	0.3 - 2.3	1.7	0.6 - 4.5	0.0	0.0 - 0.0	8.1	3.0 - 20.3	0.24
3050104	5883	LITTLE WATEREE	10.2	15.7	6 - 35	0.0	0.0 - 0.0	3.9	1.3 - 7.8	0.2	0.1 - 0.7	0.3	0.1 - 0.8	0.0	0.0 - 0.0	11.3	3.9 - 26.4	0.29
3050104	5884	LITTLE WATEREE	46.0	15.7	6 - 29	0.0	0.0 - 0.0	1.9	0.6 - 3.6	0.8	0.2 - 1.8	2.0	0.7 - 4.4	0.0	0.0 - 0.0	11.1	4.2 - 23.0	0.26
3050104	5885	*A	92.1	18.3	6 - 41	0.0	0.0 - 0.0	5.0	1.6 - 11.1	0.6	0.1 - 1.9	1.3	0.4 - 3.5	0.0	0.0 - 0.0	11.5	3.8 - 29.4	0.26
3050104	5886	*B	21.6	15.3	5 - 29	0.0	0.0 - 0.0	4.4	1.3 - 9.1	0.2	0.1 - 0.7	0.4	0.1 - 1.1	0.0	0.0 - 0.0	10.3	3.1 - 22.0	0.29
3050104	5887	DUTCHMAN'S CR	49.7	17.4	6 - 40	0.0	0.0 - 0.0	3.9	1.3 - 9.3	0.5	0.1 - 1.9	1.2	0.5 - 3.0	0.0	0.0 - 0.0	11.8	4.3 - 33.0	0.27
3050104	5888	*G	20.7	15.1	6 - 33	0.0	0.0 - 0.0	1.3	0.5 - 3.2	0.7	0.2 - 2.4	1.8	0.7 - 4.9	0.0	0.0 - 0.0	11.2	4.4 - 25.5	0.27
3050104	5889	THORNTREE CR	32.7	18.2	7 - 36	0.0	0.0 - 0.0	2.4	0.9 - 4.8	1.3	0.4 - 3.7	3.7	1.3 - 9.6	0.0	0.0 - 0.0	10.8	4.2 - 23.6	0.24
3050104	5890	SAWNEYS CR	58.7	20.9	6 - 49	0.0	0.0 - 0.0	2.5	0.7 - 6.5	1.9	0.5 - 5.4	5.8	1.8 - 17.0	0.0	0.0 - 0.0	10.8	3.2 - 25.2	0.24
3050104	5891	FIVE AND TWENT	13.8	30.0	12 - 56	0.0	0.0 - 0.0	18.7	7.2 - 38.5	1.1	0.3 - 2.9	2.1	0.7 - 5.4	0.0	0.0 - 0.0	8.2	2.9 - 16.6	0.42
3050104	5892	*C	14.1	17.9	6 - 48	0.0	0.0 - 0.0	5.9	2.0 - 14.8	1.9	0.6 - 7.4	3.6	1.3 - 11.2	0.0	0.0 - 0.0	6.5	2.2 - 18.9	0.41
3050104	5893	FIVE AND TWENT	42.2	20.4	6 - 35	0.0	0.0 - 0.0	11.0	3.2 - 21.4	0.8	0.2 - 2.0	1.5	0.5 - 3.3	0.0	0.0 - 0.0	7.3	2.1 - 15.5	0.41
3050104	5895	*D	19.7	13.2	5 - 22	0.0	0.0 - 0.0	3.7	1.2 - 7.5	0.8	0.3 - 1.9	1.6	0.5 - 3.8	0.0	0.0 - 0.0	7.1	2.3 - 14.2	0.40
3050104	5896	FIVE AND TWENT	19.2	20.9	5 - 50	0.0	0.0 - 0.0	13.6	3.6 - 33.4	0.6	0.1 - 1.7	1.0	0.3 - 2.9	0.0	0.0 - 0.0	5.6	1.7 - 14.2	0.40
3050104	5897	BEAR CR	38.4	18.3	5 - 35	0.0	0.0 - 0.0	3.8	1.1 - 7.8	0.9	0.2 - 2.6	2.8	0.9 - 6.4	0.0	0.0 - 0.0	10.8	3.2 - 23.7	0.39
3050104	5898	FIVE AND TWENT	20.1	21.0	8 - 40	0.0	0.0 - 0.0	7.7	2.8 - 16.5	0.9	0.3 - 2.2	3.3	1.1 - 8.5	0.0	0.0 - 0.0	9.2	3.8 - 20.8	0.39
3050104	5899	FIVE AND TWENT	4.9	22.4	8 - 56	0.0	0.0 - 0.0	6.8	2.3 - 17.5	2.9	0.9 - 7.9	6.0	2.0 - 17.6	0.0	0.0 - 0.0	6.7	2.4 - 17.8	0.38
3050104	5900	FIVE AND TWENT	63.8	24.1	8 - 55	0.0	0.0 - 0.0	7.5	2.5 - 16.5	0.5	0.2 - 1.5	4.7	1.5 - 12.8	0.0	0.0 - 0.0	11.3	3.8 - 27.0	0.36
3050104	5901	RICE CR	36.4	40.2	15 - 83	0.0	0.0 - 0.0	32.6	11.6 - 69.0	0.1	0.0 - 0.3	1.4	0.5 - 3.3	0.0	0.0 - 0.0	6.0	2.2 - 14.0	0.36
3050104	5902	*E	34.4	34.8	12 - 91	0.0	0.0 - 0.0	25.8	8.8 - 68.0	0.6	0.2 - 2.0	2.2	0.7 - 6.7	0.0	0.0 - 0.0	6.2	2.4 - 16.7	0.38
3050104	5903	*F	14.8	32.3	12 - 72	0.0	0.0 - 0.0	23.9	8.9 - 60.5	0.8	0.3 - 2.7	1.6	0.6 - 5.0	0.0	0.0 - 0.0	5.9	2.3 - 16.0	0.40
3050104	5904	SPEARS CR	208.7	21.1	7 - 41	1.5	0.5 - 2.9	7.3	2.1 - 17.2	1.1	0.3 - 3.1	2.8	0.9 - 5.9	0.0	0.0 - 0.0	8.4	2.9 - 18.0	0.43
3050104	5905	COLONELS CR	180.7	12.3	4 - 26	0.0	0.0 - 0.0	5.0	1.4 - 11.1	0.1	0.0 - 0.3	1.2	0.4 - 2.9	0.0	0.0 - 0.0	5.9	1.8 - 12.9	0.43
3050105	5906	BROAD R	8.1	29.4	11 - 50	0.0	0.0 - 0.0	3.8	1.3 - 8.0	2.4	0.6 - 6.7	8.6	3.2 - 19.3	0.0	0.0 - 0.0	14.6	5.0 - 32.5	0.34
3050105	5907	BULLOCK CR	110.4	26.4	7 - 52	0.0	0.0 - 0.0	4.5	1.3 - 9.9	2.1	0.5 - 5.1	8.9	2.4 - 22.9	0.0	0.0 - 0.0	10.9	2.9 - 23.9	0.34

8-digit HUC	ID	Name	Area	Catchment Yield		Point sources		Developed Land		Manure		Agricultural Land		Phosphate Mines		Soil parent rock		Frac
				kg/km^2	90% CI	kg/km^2	90% CI	kg/km^2	90% CI	kg/km^2	90% CI	kg/km^2	90% CI	kg/km^2	90% CI	kg/km^2	90% CI	
3050105	5908	BULLOCK CR	95.5	26.1	8 - 53	0.0	0.0 - 0.0	4.9	1.6 - 9.1	1.9	0.6 - 5.3	7.9	2.6 - 21.0	0.0	0.0 - 0.0	11.5	4.0 - 24.5	0.31
3050105	5909	BROAD R, CLARK	111.8	12.3	4 - 20	0.0	0.0 - 0.0	2.9	0.9 - 5.7	0.7	0.2 - 2.0	2.9	0.9 - 6.5	0.0	0.0 - 0.0	5.7	1.9 - 11.1	0.31
3050105	5910	BROAD R	25.5	29.2	12 - 62	0.0	0.0 - 0.0	3.4	1.2 - 8.5	2.9	0.9 - 8.1	12.1	4.2 - 33.9	0.0	0.0 - 0.0	10.9	4.1 - 27.2	0.34
3050105	5911	BROAD R	8.8	39.6	13 - 75	0.0	0.0 - 0.0	3.4	1.1 - 7.9	4.2	1.4 - 11.5	20.4	6.9 - 46.9	0.0	0.0 - 0.0	11.6	3.4 - 27.5	0.33
3050105	5912	BROAD R	58.3	18.5	8 - 31	0.0	0.0 - 0.0	2.9	1.1 - 5.1	1.1	0.4 - 3.4	4.1	1.6 - 9.7	0.0	0.0 - 0.0	10.3	3.8 - 19.3	0.33
3050105	5913	KINGS CR	178.7	23.5	9 - 48	0.0	0.0 - 0.0	8.4	3.2 - 18.5	1.3	0.5 - 3.6	6.0	2.3 - 16.5	0.0	0.0 - 0.0	7.8	3.1 - 17.9	0.33
3050105	5914	BROAD R	39.6	19.8	8 - 45	0.0	0.0 - 0.0	5.4	1.9 - 14.1	1.0	0.4 - 3.1	5.1	1.8 - 14.0	0.0	0.0 - 0.0	8.2	3.0 - 21.0	0.33
3050105	5915	BROAD R	25.5	170.2	60 - 311	132.6	47.6 - 265.4	17.2	5.8 - 31.4	1.9	0.5 - 3.9	9.8	3.1 - 22.3	0.0	0.0 - 0.0	8.8	3.0 - 18.7	0.32
3050105	5916	BROAD R	14.0	66.4	25 - 142	43.2	16.2 - 91.4	13.8	4.6 - 31.3	0.6	0.2 - 1.6	3.1	1.1 - 8.8	0.0	0.0 - 0.0	5.6	2.0 - 13.0	0.32
3050105	5917	BUFFALO CR	83.5	46.4	20 - 90	2.0	0.8 - 4.2	13.5	5.1 - 27.2	3.7	1.3 - 10.3	14.6	5.3 - 34.6	0.0	0.0 - 0.0	12.6	5.3 - 28.4	0.32
3050105	5918	BEASON CR	47.2	52.1	18 - 121	0.0	0.0 - 0.0	17.4	5.7 - 44.4	5.3	1.7 - 16.5	17.2	5.9 - 44.3	0.0	0.0 - 0.0	12.2	4.2 - 32.5	0.31
3050105	5919	BUFFALO CR	27.3	55.7	19 - 98	0.0	0.0 - 0.0	24.9	7.9 - 59.6	4.2	1.2 - 12.1	14.3	4.9 - 32.2	0.0	0.0 - 0.0	12.3	3.8 - 29.6	0.31
3050105	5920	POTTS CR	116.1	124.9	45 - 256	74.7	26.7 - 165.5	14.4	4.8 - 31.2	5.0	1.4 - 15.7	17.5	5.5 - 41.4	0.0	0.0 - 0.0	13.3	4.7 - 30.6	0.30
3050105	5921	BUFFALO CR	93.7	48.3	16 - 113	0.0	0.0 - 0.0	14.4	4.6 - 33.0	5.1	1.6 - 14.8	16.6	5.9 - 45.5	0.0	0.0 - 0.0	12.2	4.0 - 29.8	0.30
3050105	5922	*A	30.2	49.0	16 - 83	0.0	0.0 - 0.0	5.1	1.7 - 10.5	5.8	1.8 - 14.5	25.0	8.5 - 68.7	0.0	0.0 - 0.0	13.0	3.8 - 27.6	0.28
3050105	5923	BUFFALO CR	60.4	45.6	17 - 86	0.0	0.0 - 0.0	7.8	2.7 - 15.4	5.5	1.9 - 14.3	19.9	6.5 - 52.9	0.0	0.0 - 0.0	12.4	4.7 - 29.1	0.28
3050105	5924	BROAD R	4.9	30.4	10 - 57	0.0	0.0 - 0.0	13.1	4.0 - 24.7	1.2	0.4 - 3.4	6.2	2.0 - 14.8	0.0	0.0 - 0.0	9.9	3.1 - 21.8	0.32
3050105	5925	BROAD R	12.9	31.3	12 - 56	0.0	0.0 - 0.0	4.7	1.8 - 9.5	3.5	1.1 - 8.8	11.8	3.6 - 25.2	0.0	0.0 - 0.0	11.4	4.4 - 26.7	0.32
3050105	5926	FIRST BROAD R	100.0	53.9	18 - 154	0.0	0.0 - 0.0	13.3	4.5 - 36.1	6.4	2.0 - 20.5	21.5	6.9 - 72.5	0.0	0.0 - 0.0	12.7	4.4 - 34.3	0.32
3050105	5927	FIRST BROAD R	107.1	51.3	22 - 109	0.4	0.2 - 1.0	13.1	5.3 - 27.7	5.8	2.2 - 15.0	19.3	7.3 - 51.7	0.0	0.0 - 0.0	12.7	5.0 - 27.9	0.31
3050105	5928	MAPLE CR	22.7	49.8	18 - 130	0.0	0.0 - 0.0	6.7	2.2 - 16.6	7.1	2.2 - 23.3	23.9	8.7 - 67.5	0.0	0.0 - 0.0	12.0	4.3 - 35.5	0.30
3050105	5930	*B	109.1	32.9	10 - 65	0.1	0.0 - 0.1	6.0	1.9 - 12.8	4.0	1.2 - 10.0	13.0	3.7 - 32.1	0.0	0.0 - 0.0	9.9	3.4 - 25.5	0.30
3050105	5931	FIRST BROAD R	92.9	33.6	11 - 87	0.0	0.0 - 0.0	6.3	2.2 - 13.7	3.6	1.1 - 11.2	12.6	4.1 - 36.0	0.0	0.0 - 0.0	11.1	3.7 - 34.7	0.30
3050105	5932	WARDS CR	45.8	24.0	7 - 42	0.0	0.0 - 0.0	3.8	1.1 - 8.1	2.3	0.7 - 4.8	7.4	1.8 - 18.7	0.0	0.0 - 0.0	10.5	3.2 - 19.8	0.28
3050105	5933	FIRST BROAD R	219.3	20.0	7 - 47	0.0	0.0 - 0.0	2.8	1.0 - 7.5	1.1	0.3 - 3.3	4.8	1.6 - 11.8	0.0	0.0 - 0.0	11.2	3.5 - 32.1	0.28
3050105	5934	BRUSHY CR	75.0	46.9	16 - 114	0.0	0.0 - 0.0	8.2	2.6 - 18.9	6.2	1.8 - 17.3	20.7	6.8 - 59.5	0.0	0.0 - 0.0	11.8	4.2 - 28.3	0.31
3050105	5935	SANDY RUN CR	173.2	47.6	18 - 109	4.0	1.7 - 10.0	9.4	3.3 - 19.8	4.6	1.7 - 13.6	17.4	6.5 - 45.9	0.0	0.0 - 0.0	12.2	5.0 - 29.3	0.32
3050105	5936	BROAD R	67.7	42.4	14 - 100	0.0	0.0 - 0.0	7.1	2.1 - 14.1	4.0	1.1 - 12.2	18.0	5.7 - 47.1	0.0	0.0 - 0.0	13.3	4.3 - 34.4	0.32
3050105	5937	SECOND BROAD R	155.3	128.3	41 - 263	81.9	25.6 - 170.9	18.1	5.8 - 38.3	2.8	0.8 - 7.0	12.3	3.8 - 29.0	0.0	0.0 - 0.0	13.3	4.2 - 27.6	0.32
3050105	5938	SECOND BROAD R	70.4	33.7	12 - 69	0.0	0.0 - 0.0	5.6	2.1 - 13.2	2.8	0.9 - 7.4	12.3	4.3 - 28.5	0.0	0.0 - 0.0	13.0	4.5 - 30.8	0.30
3050105	5939	SECOND BROAD R	121.9	63.9	24 - 113	26.9	10.1 - 50.7	11.4	4.0 - 23.6	2.2	0.7 - 5.8	8.4	2.9 - 18.3	0.0	0.0 - 0.0	15.0	5.5 - 30.3	0.30
3050105	5940	SECOND BROAD R	149.9	23.6	7 - 49	0.0	0.0 - 0.0	4.8	1.6 - 10.1	1.4	0.4 - 3.5	4.3	1.4 - 9.6	0.0	0.0 - 0.0	13.1	4.0 - 27.5	0.29
3050105	5941	BROAD R	65.3	41.8	17 - 89	0.0	0.0 - 0.0	12.1	4.6 - 24.6	3.0	1.0 - 8.3	14.7	5.3 - 35.2	0.0	0.0 - 0.0	11.8	4.8 - 26.9	0.32
3050105	5942	*C	72.6	52.8	22 - 143	0.0	0.0 - 0.0	28.1	12.6 - 79.2	2.1	0.7 - 5.8	9.8	3.8 - 27.5	0.0	0.0 - 0.0	12.8	5.3 - 35.3	0.31
3050105	5943	BROAD R	9.2	19.5	6 - 38	0.0	0.0 - 0.0	1.5	0.4 - 3.3	1.2	0.4 - 3.0	5.5	1.7 - 13.5	0.0	0.0 - 0.0	11.4	3.2 - 28.5	0.31
3050105	5944	RICHARDSON CR	25.4	37.6	11 - 84	0.0	0.0 - 0.0	8.4	2.3 - 18.1	3.1	0.9 - 7.8	13.7	4.7 - 34.5	0.0	0.0 - 0.0	12.4	3.6 - 29.9	0.31
3050105	5945	BROAD R	85.2	29.9	12 - 70	0.0	0.0 - 0.0	5.7	1.9 - 12.3	2.1	0.7 - 5.6	10.0	3.7 - 28.6	0.0	0.0 - 0.0	12.2	4.5 - 27.3	0.31
3050105	5946	BROAD R	62.6	62.8	27 - 147	17.9	7.8 - 39.1	25.3	9.8 - 64.0	1.4	0.5 - 4.3	5.7	2.3 - 16.3	0.0	0.0 - 0.0	12.4	5.6 - 29.0	0.31
3050105	5947	MOUNTAIN CR	121.8	32.0	13 - 77	0.0	0.0 - 0.0	7.0	2.7 - 17.6	1.7	0.5 - 5.4	5.7	2.3 - 18.0	0.0	0.0 - 0.0	17.6	6.3 - 45.0	0.30

8-digit HUC	ID	Name	Area	Catchment Yield		Point sources		Developed Land		Manure		Agricultural Land		Phosphate Mines		Soil parent rock		Frac
				kg/km^2	90% CI	kg/km^2	90% CI	kg/km^2	90% CI	kg/km^2	90% CI	kg/km^2	90% CI	kg/km^2	90% CI	kg/km^2	90% CI	
3050105	5948	BROAD R	66.3	30.7	11 - 69	0.0	0.0 - 0.0	5.4	1.9 - 10.4	1.4	0.5 - 3.8	4.7	1.6 - 11.4	0.0	0.0 - 0.0	19.2	7.4 - 48.7	0.30
3050105	5949	COVE CR	207.5	21.0	7 - 58	0.0	0.0 - 0.0	4.4	1.6 - 11.9	0.9	0.3 - 3.1	2.6	0.8 - 8.7	0.0	0.0 - 0.0	13.1	4.5 - 35.4	0.29
3050105	5950	BROAD R	23.6	72.5	25 - 152	45.5	15.3 - 103.9	9.4	2.8 - 19.4	0.7	0.2 - 1.9	1.5	0.5 - 3.9	0.0	0.0 - 0.0	15.3	5.1 - 33.1	0.29
3050105	5951	GREEN R	11.1	21.7	8 - 43	0.0	0.0 - 0.0	4.5	1.6 - 8.7	0.8	0.3 - 2.0	4.0	1.3 - 9.3	0.0	0.0 - 0.0	12.3	4.7 - 30.2	0.31
3050105	5952	GREEN R	42.3	29.7	9 - 56	0.0	0.0 - 0.0	7.5	2.4 - 13.6	0.7	0.2 - 2.1	3.2	0.9 - 7.2	0.0	0.0 - 0.0	18.3	5.8 - 44.3	0.31
3050105	5953	WALNUT CR	56.0	32.3	11 - 59	0.0	0.0 - 0.0	8.3	2.9 - 15.5	0.9	0.2 - 2.3	4.7	1.6 - 10.6	0.0	0.0 - 0.0	18.5	5.6 - 39.4	0.30
3050105	5954	GREEN R	200.3	24.8	8 - 45	0.0	0.0 - 0.1	7.0	2.0 - 14.1	0.4	0.1 - 1.0	2.3	0.5 - 5.4	0.0	0.0 - 0.0	15.0	4.6 - 28.9	0.30
3050105	5955	HUNGRY R	56.1	33.8	10 - 58	0.0	0.0 - 0.0	11.3	3.4 - 20.4	1.4	0.4 - 3.6	8.3	2.7 - 17.6	0.0	0.0 - 0.0	12.8	4.5 - 25.0	0.24
3050105	5956	GREEN R	135.5	31.0	11 - 60	0.0	0.0 - 0.0	14.2	4.8 - 28.1	0.6	0.2 - 1.6	3.2	1.2 - 10.2	0.0	0.0 - 0.0	12.9	5.2 - 30.4	0.24
3050105	5957	ROSS CR	45.8	44.9	15 - 102	0.0	0.0 - 0.0	6.8	2.2 - 14.7	3.9	1.2 - 10.1	20.5	6.8 - 57.9	0.0	0.0 - 0.0	13.8	4.7 - 35.6	0.32
3050105	5958	CHEROKEE CR	62.6	43.7	13 - 101	0.0	0.0 - 0.0	19.3	5.7 - 46.4	2.2	0.6 - 7.2	12.7	3.5 - 34.8	0.0	0.0 - 0.0	9.6	2.8 - 26.4	0.32
3050105	5959	PEOPLES CR	24.2	47.7	18 - 115	0.0	0.0 - 0.0	31.2	11.5 - 76.9	1.2	0.4 - 3.9	7.2	2.5 - 17.3	0.0	0.0 - 0.0	8.1	2.7 - 20.7	0.32
3050105	5960	ABINGDON CR	36.8	26.5	9 - 46	0.0	0.0 - 0.0	4.1	1.5 - 7.8	1.6	0.5 - 5.0	6.0	2.0 - 14.8	0.0	0.0 - 0.0	14.8	5.4 - 31.1	0.33
3050105	5961	GILKEY CR	9.8	24.7	8 - 49	0.0	0.0 - 0.0	4.4	1.4 - 8.8	1.4	0.4 - 4.6	7.7	2.5 - 18.8	0.0	0.0 - 0.0	11.1	3.4 - 24.2	0.33
3050105	5962	GILKEY CR	71.1	29.4	9 - 64	0.0	0.0 - 0.0	5.5	1.8 - 11.4	1.9	0.5 - 5.4	8.9	2.4 - 21.9	0.0	0.0 - 0.0	13.2	3.8 - 30.9	0.33
3050105	5963	THICKETTY CR	140.4	27.1	8 - 48	0.0	0.0 - 0.1	3.6	1.1 - 7.0	1.7	0.5 - 3.8	8.4	2.4 - 20.7	0.0	0.0 - 0.0	13.3	3.9 - 28.3	0.33
3050105	5964	LIMESTONE CR	27.9	49.3	18 - 128	0.0	0.0 - 0.0	23.2	7.8 - 60.0	2.0	0.6 - 6.6	11.3	3.9 - 32.5	0.0	0.0 - 0.0	12.9	4.9 - 36.0	0.29
3050105	5965	THICKETTY CR	157.8	60.5	22 - 105	22.2	8.6 - 40.5	14.5	4.8 - 28.1	1.8	0.6 - 4.8	10.3	3.1 - 23.7	0.0	0.0 - 0.0	11.7	4.1 - 24.2	0.29
3050105	5966	PACOLET R	265.2	25.7	9 - 55	0.7	0.2 - 1.6	5.8	1.9 - 12.7	1.1	0.3 - 2.8	6.1	1.9 - 16.0	0.0	0.0 - 0.0	12.0	4.3 - 27.8	0.34
3050105	5967	PACOLET R	25.3	45.9	20 - 91	17.8	7.2 - 34.8	7.6	2.9 - 15.8	1.5	0.5 - 4.2	8.1	3.2 - 19.8	0.0	0.0 - 0.0	11.0	4.7 - 24.8	0.32
3050105	5968	BUCK CR	96.2	54.4	16 - 111	2.6	0.9 - 5.1	13.7	4.0 - 27.7	3.0	1.0 - 8.7	20.8	6.9 - 50.5	0.0	0.0 - 0.0	14.2	4.6 - 38.7	0.30
3050105	5969	PACOLET R	38.2	48.2	16 - 103	0.0	0.0 - 0.0	12.6	4.5 - 27.6	2.6	0.8 - 6.4	17.5	5.8 - 53.2	0.0	0.0 - 0.0	15.5	5.8 - 30.1	0.30
3050105	5970	N PACOLET R	5.6	35.9	12 - 66	0.0	0.0 - 0.0	5.6	1.7 - 10.4	2.1	0.5 - 5.0	12.3	4.5 - 30.7	0.0	0.0 - 0.0	16.0	5.5 - 34.0	0.29
3050105	5971	N PACOLET R	73.6	51.1	18 - 119	2.7	1.0 - 7.3	24.6	8.0 - 58.1	0.4	0.1 - 1.2	2.8	0.9 - 10.1	0.0	0.0 - 0.0	20.6	8.6 - 57.9	0.27
3050105	5972	*D	25.7	126.0	43 - 224	70.9	24.7 - 149.7	30.1	9.2 - 62.5	0.2	0.0 - 0.5	0.6	0.2 - 1.5	0.0	0.0 - 0.0	24.1	8.6 - 50.1	0.27
3050105	5973	S PACOLET R	2.8	28.9	10 - 52	0.0	0.0 - 0.0	7.8	2.7 - 15.9	0.7	0.2 - 1.7	5.0	1.4 - 12.0	0.0	0.0 - 0.0	15.4	5.9 - 30.3	0.29
3050105	5974	S PACOLET R	94.4	49.8	17 - 97	0.0	0.0 - 0.0	15.1	4.7 - 28.8	1.6	0.4 - 4.3	9.2	2.7 - 23.8	0.0	0.0 - 0.0	24.0	7.6 - 55.3	0.07
3050105	5975	MOLTOW CR	31.7	59.1	20 - 120	0.5	0.2 - 1.0	16.4	5.2 - 35.3	3.2	0.8 - 9.6	21.0	7.0 - 56.8	0.0	0.0 - 0.0	18.1	6.0 - 40.8	0.07
3050105	5976	LAWSONS FORK C	191.7	65.9	26 - 176	0.0	0.0 - 0.0	42.5	16.2 - 110.2	1.3	0.4 - 5.4	8.6	3.2 - 27.4	0.0	0.0 - 0.0	13.5	5.4 - 44.0	0.32
3050105	5977	LAWSONS FORK C	16.7	122.6	39 - 295	58.7	18.4 - 151.0	36.2	12.1 - 87.1	1.9	0.6 - 5.6	12.9	4.0 - 31.3	0.0	0.0 - 0.0	12.9	4.1 - 31.9	0.27
3050105	5978	WHITEOAK CR	132.3	43.8	16 - 99	3.5	1.3 - 8.2	12.0	4.3 - 25.2	1.4	0.4 - 4.3	8.7	3.2 - 24.4	0.0	0.0 - 0.0	18.2	6.6 - 42.0	0.31
3050105	5979	HICKORY CR	50.1	70.9	26 - 167	0.0	0.0 - 0.0	44.1	16.0 - 95.2	3.2	1.1 - 9.7	10.5	3.5 - 28.6	0.0	0.0 - 0.0	13.1	4.9 - 36.2	0.32
3050105	5980	FIRST BROAD R	16.3	573.5	204 - 1256	494.8	177.9 - 1105.9	54.4	17.6 - 110.6	2.8	0.8 - 8.6	9.3	3.0 - 26.2	0.0	0.0 - 0.0	12.3	3.8 - 26.8	0.32
3050105	5981	BROAD R	10.9	28.9	9 - 50	0.0	0.0 - 0.0	4.0	1.2 - 7.1	3.1	0.8 - 9.1	9.9	3.0 - 23.2	0.0	0.0 - 0.0	11.9	3.6 - 25.5	0.32
3050106	5982	BROAD R	6.2	72.5	26 - 146	0.0	0.0 - 0.0	64.8	22.5 - 135.2	0.1	0.0 - 0.1	0.6	0.2 - 1.5	0.0	0.0 - 0.0	7.0	2.7 - 14.6	0.43
3050106	5983	CRANE R	174.8	41.5	13 - 96	0.0	0.0 - 0.0	30.2	9.1 - 75.5	0.1	0.0 - 0.3	1.5	0.4 - 3.9	0.0	0.0 - 0.0	9.7	2.7 - 24.0	0.43
3050106	5984	BROAD R	28.1	51.8	15 - 128	0.0	0.0 - 0.0	24.8	7.3 - 65.2	0.7	0.2 - 1.9	11.9	3.7 - 28.8	0.0	0.0 - 0.0	14.3	4.2 - 40.7	0.43
3050106	5985	STATESMEN CR	14.0	23.5	8 - 50	0.0	0.0 - 0.0	6.0	1.9 - 13.0	0.3	0.1 - 0.8	3.7	1.3 - 8.3	0.0	0.0 - 0.0	13.4	4.3 - 30.5	0.43
3050106	5986	BROAD R	57.5	79.5	27 - 154	36.8	13.3 - 77.5	24.3	8.4 - 54.5	0.2	0.1 - 0.8	3.5	1.2 - 8.8	0.0	0.0 - 0.0	14.6	4.8 - 36.8	0.43

8-digit HUC	ID	Name	Area	Catchment Yield		Point sources		Developed Land		Manure		Agricultural Land		Phosphate Mines		Soil parent rock		Frac
				kg/km^2	90% CI	kg/km^2	90% CI	kg/km^2	90% CI	kg/km^2	90% CI	kg/km^2	90% CI	kg/km^2	90% CI	kg/km^2	90% CI	
3050106	5987	CEDAR CR	35.2	24.2	7 - 53	0.0	0.0 - 0.0	6.7	2.0 - 15.3	0.2	0.1 - 0.5	3.3	0.9 - 8.6	0.0	0.0 - 0.0	14.0	4.1 - 32.5	0.42
3050106	5988	CEDAR CR	137.8	26.3	9 - 61	0.9	0.3 - 2.2	7.2	2.5 - 15.4	0.9	0.2 - 2.3	4.1	1.7 - 9.3	0.0	0.0 - 0.0	13.2	4.0 - 34.3	0.40
3050106	5989	CROOKED RUN CR	88.7	21.8	9 - 51	0.0	0.0 - 0.0	4.0	1.6 - 9.9	1.2	0.4 - 3.0	3.8	1.4 - 10.2	0.0	0.0 - 0.0	12.8	4.9 - 29.8	0.40
3050106	5990	BROAD R	1.7	28.5	10 - 67	0.0	0.0 - 0.0	11.5	4.0 - 27.1	0.2	0.1 - 0.6	2.3	0.9 - 6.9	0.0	0.0 - 0.0	14.5	4.6 - 36.7	0.42
3050106	5991	BROAD R	12.7	24.3	7 - 66	0.0	0.0 - 0.0	6.0	2.0 - 15.7	0.2	0.0 - 0.7	2.5	0.8 - 7.7	0.0	0.0 - 0.0	15.5	4.7 - 42.3	0.42
3050106	5992	LITTLE R	144.8	18.3	6 - 37	0.0	0.0 - 0.0	3.0	0.9 - 6.2	0.7	0.2 - 2.0	1.8	0.6 - 4.7	0.0	0.0 - 0.0	12.8	4.5 - 27.3	0.42
3050106	5993	MILL CR	54.3	22.6	6 - 52	0.0	0.0 - 0.0	4.2	1.3 - 8.4	1.6	0.4 - 4.6	5.0	1.8 - 13.2	0.0	0.0 - 0.0	11.9	3.5 - 27.2	0.40
3050106	5994	LITTLE R	32.8	17.9	6 - 38	0.0	0.0 - 0.0	2.8	0.9 - 6.0	0.9	0.3 - 2.9	2.2	0.7 - 5.4	0.0	0.0 - 0.0	11.9	4.1 - 29.0	0.40
3050106	5995	JACKSON CR	100.1	33.0	11 - 65	9.5	3.3 - 21.2	7.3	2.3 - 16.3	1.1	0.4 - 2.8	3.3	1.0 - 8.2	0.0	0.0 - 0.0	11.8	4.4 - 26.6	0.39
3050106	5996	LITTLE R	30.4	18.2	5 - 36	0.0	0.0 - 0.0	1.8	0.5 - 3.5	0.9	0.2 - 2.7	2.0	0.5 - 4.8	0.0	0.0 - 0.0	13.4	3.8 - 31.3	0.39
3050106	5997	LITTLE R	14.9	24.1	8 - 56	0.0	0.0 - 0.0	1.8	0.7 - 4.0	2.1	0.7 - 5.6	7.0	2.4 - 20.4	0.0	0.0 - 0.0	13.1	4.8 - 32.0	0.37
3050106	5998	LITTLE R, N FK	52.7	16.9	6 - 37	0.0	0.0 - 0.0	2.2	0.8 - 4.8	0.6	0.2 - 1.5	1.2	0.4 - 2.7	0.0	0.0 - 0.0	12.8	4.8 - 29.4	0.36
3050106	5999	LITTLE R, E FK	111.7	18.2	6 - 49	0.0	0.0 - 0.0	2.5	0.9 - 6.9	0.9	0.3 - 3.4	2.3	0.7 - 7.0	0.0	0.0 - 0.0	12.4	4.2 - 36.9	0.36
3050106	6000	LITTLE R, W FK	87.6	19.0	6 - 44	0.0	0.0 - 0.0	1.8	0.6 - 4.3	1.3	0.4 - 4.5	3.8	1.3 - 10.3	0.0	0.0 - 0.0	12.1	4.1 - 30.0	0.37
3050106	6001	BROAD R	32.0	21.8	7 - 55	0.0	0.0 - 0.0	4.1	1.3 - 10.0	0.4	0.1 - 1.2	2.6	0.8 - 7.3	0.0	0.0 - 0.0	14.7	5.0 - 45.2	0.42
3050106	6002	BROAD R	40.0	28.4	8 - 51	12.3	3.6 - 24.6	3.1	0.9 - 6.1	0.6	0.2 - 1.7	1.7	0.5 - 4.0	0.0	0.0 - 0.0	10.6	3.1 - 21.2	0.42
3050106	6003	BROAD R	6.4	19.7	7 - 46	0.0	0.0 - 0.0	6.6	2.4 - 15.4	1.5	0.4 - 3.8	1.9	0.6 - 5.2	0.0	0.0 - 0.0	9.8	3.3 - 27.1	0.42
3050106	6004	BROAD R	176.6	31.2	11 - 79	0.3	0.1 - 0.8	8.1	2.8 - 21.0	4.9	1.5 - 16.3	6.5	2.2 - 18.0	0.0	0.0 - 0.0	11.3	4.0 - 28.5	0.40
3050106	6005	BROAD R	67.5	18.2	6 - 47	0.0	0.0 - 0.0	2.7	0.9 - 6.4	0.9	0.3 - 3.3	1.5	0.5 - 4.3	0.0	0.0 - 0.0	13.1	4.2 - 35.7	0.35
3050106	6007	BEAVER CR	113.1	20.1	7 - 39	0.0	0.0 - 0.0	2.0	0.7 - 3.4	1.1	0.3 - 3.3	2.9	1.0 - 6.2	0.0	0.0 - 0.0	14.2	4.8 - 31.4	0.35
3050106	6008	BROAD R	20.7	19.4	8 - 44	0.0	0.0 - 0.0	1.9	0.6 - 4.2	0.9	0.3 - 2.4	1.0	0.3 - 2.3	0.0	0.0 - 0.0	15.6	6.3 - 37.4	0.35
3050106	6009	BROAD R	30.8	19.2	6 - 44	0.0	0.0 - 0.0	1.7	0.6 - 3.2	0.3	0.1 - 0.9	0.7	0.2 - 1.8	0.0	0.0 - 0.0	16.4	5.3 - 42.1	0.34
3050106	6010	SANDY CR	39.9	19.2	7 - 37	0.0	0.0 - 0.0	2.3	0.8 - 4.4	1.0	0.3 - 2.5	2.2	0.7 - 5.3	0.0	0.0 - 0.0	13.7	4.9 - 31.0	0.34
3050106	6011	*A	111.4	21.7	8 - 69	0.0	0.0 - 0.0	2.8	1.0 - 7.2	1.5	0.5 - 5.5	4.7	1.6 - 14.5	0.0	0.0 - 0.0	12.7	4.6 - 45.3	0.33
3050106	6012	SANDY CR	8.0	19.9	8 - 48	0.0	0.0 - 0.0	2.1	0.7 - 5.2	0.9	0.3 - 2.3	2.5	0.8 - 7.4	0.0	0.0 - 0.0	14.3	5.1 - 36.4	0.33
3050106	6013	SANDY CR	19.6	24.0	7 - 53	0.0	0.0 - 0.0	1.6	0.4 - 3.1	1.9	0.5 - 5.5	6.4	1.8 - 16.0	0.0	0.0 - 0.0	14.1	4.4 - 36.8	0.32
3050106	6014	SANDY CR	119.1	53.0	20 - 155	21.3	8.2 - 63.5	11.8	3.4 - 35.2	1.5	0.4 - 4.6	5.6	2.0 - 16.5	0.0	0.0 - 0.0	12.7	4.9 - 34.1	0.31
3050106	6015	SEELEY CR	71.0	26.5	8 - 58	0.0	0.0 - 0.0	3.8	0.9 - 9.3	2.3	0.6 - 6.0	9.3	3.1 - 24.9	0.0	0.0 - 0.0	11.2	3.4 - 27.5	0.31
3050106	6016	BRUSHY CR	55.0	18.8	7 - 41	0.0	0.0 - 0.0	2.1	0.8 - 4.6	1.3	0.4 - 3.4	4.3	1.3 - 10.0	0.0	0.0 - 0.0	11.1	4.3 - 26.1	0.32
3050106	6017	BROAD R	21.0	30.9	9 - 76	0.0	0.0 - 0.0	8.1	2.4 - 18.1	0.9	0.2 - 2.6	4.1	1.4 - 10.7	0.0	0.0 - 0.0	17.8	5.1 - 51.2	0.34
3050106	6018	BROAD R	41.2	28.4	11 - 66	0.0	0.0 - 0.0	4.8	1.7 - 11.0	1.1	0.3 - 3.9	9.8	3.6 - 26.6	0.0	0.0 - 0.0	12.7	4.7 - 29.7	0.34
3050106	6019	TURKEY CR	78.7	21.8	7 - 39	0.0	0.0 - 0.0	3.8	1.1 - 6.7	1.4	0.4 - 3.3	4.4	1.3 - 8.6	0.0	0.0 - 0.0	12.2	4.3 - 28.8	0.34
3050106	6020	SUSYBOLE CR	62.5	23.3	6 - 55	0.0	0.0 - 0.0	3.4	0.9 - 7.5	2.0	0.6 - 6.4	7.6	2.0 - 23.6	0.0	0.0 - 0.0	10.4	2.9 - 25.0	0.32
3050106	6021	TURKEY CR	78.1	20.2	5 - 42	0.0	0.0 - 0.0	3.1	0.9 - 6.1	1.1	0.3 - 3.1	3.7	1.1 - 9.1	0.0	0.0 - 0.0	12.2	3.7 - 29.4	0.32
3050106	6022	TURKEY CR, WRIC	76.8	20.4	8 - 41	0.0	0.0 - 0.0	3.8	1.4 - 8.2	1.4	0.5 - 4.1	5.5	2.1 - 12.8	0.0	0.0 - 0.0	9.7	3.7 - 23.9	0.28
3050106	6023	TURKEY CR	84.1	28.3	11 - 72	0.0	0.0 - 0.0	8.7	3.2 - 20.5	1.8	0.5 - 5.7	7.3	2.6 - 25.1	0.0	0.0 - 0.0	10.5	4.0 - 27.3	0.28
3050106	6024	BROAD R	74.8	31.7	9 - 54	2.1	0.5 - 3.9	5.5	1.5 - 9.8	1.5	0.4 - 3.9	10.0	2.7 - 21.8	0.0	0.0 - 0.0	12.6	3.7 - 24.5	0.34
3050106	6025	BROWNS CR	137.5	29.9	11 - 61	4.0	1.4 - 8.6	6.7	2.2 - 14.3	0.9	0.3 - 2.5	7.2	2.8 - 16.6	0.0	0.0 - 0.0	11.1	3.7 - 26.2	0.34
3050106	6026	HELLERS CR	104.8	24.9	8 - 61	0.0	0.0 - 0.0	4.7	1.5 - 10.1	4.3	1.2 - 11.7	6.1	2.0 - 15.6	0.0	0.0 - 0.0	9.8	2.9 - 24.3	0.35

8-digit HUC	ID	Name	Area	Catchment Yield		Point sources		Developed Land		Manure		Agricultural Land		Phosphate Mines		Soil parent rock		Frac
				kg/km^2	90% CI	kg/km^2	90% CI	kg/km^2	90% CI	kg/km^2	90% CI	kg/km^2	90% CI	kg/km^2	90% CI	kg/km^2	90% CI	
3050106	6027	ROCKY CR	85.8	25.3	7 - 54	0.0	0.0 - 0.0	7.8	2.0 - 17.1	3.2	0.8 - 8.1	4.1	1.1 - 10.9	0.0	0.0 - 0.0	10.2	3.1 - 23.0	0.42
3050106	6028	*B	66.4	30.3	8 - 60	0.0	0.0 - 0.0	10.5	2.4 - 20.7	1.6	0.4 - 4.2	4.6	1.3 - 11.9	0.0	0.0 - 0.0	13.5	3.9 - 33.7	0.42
3050106	6029	HOPE CR	45.2	37.3	13 - 75	0.0	0.0 - 0.0	19.0	6.5 - 40.6	0.3	0.1 - 0.7	4.4	1.4 - 11.8	0.0	0.0 - 0.0	13.6	4.5 - 27.9	0.42
3050106	6030	FREES CR	50.1	25.0	7 - 64	0.0	0.0 - 0.0	3.5	1.0 - 8.4	1.1	0.3 - 2.9	3.2	1.0 - 8.7	0.0	0.0 - 0.0	17.2	5.0 - 46.8	0.40
3050106	6031	BROAD R	8.0	16.4	5 - 36	0.0	0.0 - 0.0	2.3	0.8 - 4.6	1.2	0.4 - 3.4	1.4	0.5 - 3.6	0.0	0.0 - 0.0	11.6	3.9 - 29.1	0.40
3050107	6032	TYGER R	1.1	21.4	8 - 45	0.0	0.0 - 0.0	5.3	1.8 - 12.0	0.0	0.0 - 0.0	0.0	0.0 - 0.0	0.0	0.0 - 0.0	16.0	5.7 - 35.1	0.34
3050107	6033	CANE CR	64.9	19.0	6 - 44	0.0	0.0 - 0.0	3.3	1.3 - 7.0	0.5	0.2 - 1.7	1.9	0.7 - 4.9	0.0	0.0 - 0.0	13.3	4.6 - 33.6	0.34
3050107	6034	TYGER R	68.1	20.5	7 - 46	0.0	0.0 - 0.0	2.5	0.9 - 6.2	0.6	0.2 - 2.0	1.5	0.5 - 4.0	0.0	0.0 - 0.0	15.9	5.6 - 38.1	0.34
3050107	6036	TINKER CR	74.7	30.6	9 - 51	2.6	0.8 - 4.9	7.8	2.3 - 14.4	0.9	0.2 - 2.3	7.4	2.1 - 15.3	0.0	0.0 - 0.0	11.8	3.9 - 22.8	0.34
3050107	6037	TYGER R	15.2	22.1	6 - 50	0.0	0.0 - 0.0	5.3	1.5 - 12.7	0.2	0.1 - 0.7	1.7	0.5 - 4.1	0.0	0.0 - 0.0	14.9	3.9 - 37.3	0.34
3050107	6038	FAIR FOREST CR	116.2	72.3	28 - 137	36.0	13.3 - 69.5	13.4	5.3 - 27.8	0.9	0.3 - 2.7	6.5	2.4 - 15.6	0.0	0.0 - 0.0	15.5	5.9 - 35.6	0.34
3050107	6039	FAIR FOREST CR	90.7	28.9	11 - 59	1.4	0.5 - 3.4	5.5	1.9 - 12.8	1.2	0.3 - 3.0	7.1	2.3 - 17.0	0.0	0.0 - 0.0	13.7	4.9 - 35.4	0.32
3050107	6040	SPEAR CR	26.6	32.1	10 - 58	0.0	0.0 - 0.0	9.2	2.7 - 19.6	1.1	0.3 - 2.7	9.8	2.7 - 22.7	0.0	0.0 - 0.0	12.0	4.1 - 28.5	0.30
3050107	6041	SUGAR CR	48.3	29.8	10 - 71	0.0	0.0 - 0.0	3.8	1.2 - 9.2	1.2	0.4 - 3.7	10.0	3.4 - 28.6	0.0	0.0 - 0.0	14.8	4.8 - 34.5	0.32
3050107	6042	TYGER R	106.0	18.5	7 - 40	0.0	0.0 - 0.0	1.9	0.6 - 4.2	0.4	0.1 - 1.3	2.8	0.8 - 6.9	0.0	0.0 - 0.0	13.4	4.4 - 32.6	0.34
3050107	6043	DUTCHMANS CR	86.4	36.9	11 - 66	0.0	0.0 - 0.0	4.7	1.6 - 8.9	2.2	0.6 - 4.8	13.8	4.4 - 27.5	0.0	0.0 - 0.0	16.3	5.4 - 35.8	0.32
3050107	6044	TYGER R	68.1	38.5	15 - 81	9.4	3.6 - 22.7	4.1	1.6 - 9.1	1.7	0.5 - 4.9	8.0	3.1 - 18.9	0.0	0.0 - 0.0	15.3	5.2 - 34.9	0.32
3050107	6045	TYGER R	65.7	45.0	16 - 117	0.9	0.3 - 2.5	12.1	4.1 - 26.4	2.3	0.7 - 7.3	14.6	5.1 - 42.5	0.0	0.0 - 0.0	15.0	5.5 - 40.6	0.31
3050107	6046	N TYGER R	163.1	52.7	19 - 155	0.1	0.0 - 0.3	25.3	9.5 - 68.4	1.8	0.5 - 6.9	11.1	3.4 - 34.2	0.0	0.0 - 0.0	14.4	5.2 - 48.3	0.30
3050107	6047	M TYGER R	9.3	30.2	10 - 62	0.0	0.0 - 0.0	8.0	2.6 - 18.5	1.8	0.5 - 5.1	11.9	3.7 - 29.9	0.0	0.0 - 0.0	8.6	2.9 - 19.1	0.30
3050107	6048	S TYGER R	123.7	44.6	14 - 115	0.6	0.2 - 1.6	7.4	2.4 - 20.5	2.8	0.8 - 8.8	18.3	6.1 - 55.9	0.0	0.0 - 0.0	15.4	5.3 - 44.8	0.31
3050107	6049	S TYGER R	54.8	38.1	12 - 85	0.9	0.3 - 2.0	10.5	3.5 - 21.8	1.3	0.3 - 4.2	8.2	2.4 - 21.3	0.0	0.0 - 0.0	17.1	6.2 - 44.2	0.25
3050107	6050	MUSH CR	34.0	39.5	12 - 70	0.0	0.0 - 0.0	10.9	4.0 - 21.7	1.6	0.4 - 4.1	8.1	2.8 - 18.0	0.0	0.0 - 0.0	18.9	5.4 - 41.0	0.25
3050107	6051	PADGETTS CR	36.4	23.2	8 - 55	0.0	0.0 - 0.0	4.3	1.5 - 9.2	0.7	0.2 - 2.1	4.7	1.9 - 11.8	0.0	0.0 - 0.0	13.4	4.8 - 31.5	0.34
3050107	6052	FAIR FOREST CR	283.2	47.0	18 - 95	0.7	0.3 - 1.6	25.8	10.4 - 54.9	0.9	0.3 - 2.9	5.6	1.6 - 13.5	0.0	0.0 - 0.0	13.9	4.7 - 39.4	0.30
3050107	6053	S TYGER R	43.0	59.6	19 - 125	0.0	0.0 - 0.0	30.4	8.6 - 70.0	1.9	0.6 - 5.6	12.0	4.2 - 29.9	0.0	0.0 - 0.0	15.3	5.3 - 37.6	0.30
3050107	6054	BENS CR	26.5	42.7	16 - 80	0.0	0.0 - 0.0	6.3	2.3 - 15.4	3.0	1.0 - 8.0	21.1	7.7 - 50.8	0.0	0.0 - 0.0	12.2	4.0 - 27.2	0.30
3050107	6055	TYGER R	23.0	34.7	13 - 63	0.0	0.0 - 0.0	4.1	1.3 - 8.6	1.7	0.5 - 4.8	9.5	3.2 - 23.4	0.0	0.0 - 0.0	19.4	6.9 - 41.2	0.32
3050107	6056	JIMMIES CR	53.8	38.0	13 - 97	0.0	0.0 - 0.0	10.8	3.8 - 26.8	1.7	0.5 - 5.4	9.8	3.2 - 27.7	0.0	0.0 - 0.0	15.6	5.1 - 44.6	0.32
3050108	6057	ENOREE R	68.3	17.2	6 - 33	0.0	0.0 - 0.0	3.2	1.1 - 6.5	0.6	0.2 - 1.7	0.5	0.2 - 1.3	0.0	0.0 - 0.0	12.8	4.4 - 26.0	0.35
3050108	6058	ENOREE R	4.7	17.9	7 - 41	0.0	0.0 - 0.0	3.2	1.1 - 6.9	0.0	0.0 - 0.1	0.0	0.0 - 0.0	0.0	0.0 - 0.0	14.7	5.9 - 37.7	0.34
3050108	6059	ENOREE R	35.1	20.9	7 - 43	0.0	0.0 - 0.0	2.2	0.7 - 4.8	1.1	0.3 - 3.3	2.0	0.7 - 5.1	0.0	0.0 - 0.0	15.7	4.9 - 33.9	0.34
3050108	6060	ENOREE R	11.5	27.3	8 - 53	0.0	0.0 - 0.0	6.5	1.9 - 13.3	1.6	0.4 - 4.9	2.6	0.8 - 6.4	0.0	0.0 - 0.0	16.6	5.1 - 37.3	0.33
3050108	6061	ENOREE CR	42.5	31.3	12 - 67	0.0	0.0 - 0.0	5.3	1.8 - 12.2	1.9	0.6 - 5.4	10.6	3.7 - 25.3	0.0	0.0 - 0.0	13.4	5.3 - 32.1	0.30
3050108	6062	ENOREE R	34.7	27.4	9 - 70	0.0	0.0 - 0.0	8.6	3.0 - 20.9	1.3	0.4 - 3.6	3.4	1.1 - 9.0	0.0	0.0 - 0.0	14.2	4.7 - 37.8	0.30
3050108	6063	ENOREE R	69.4	37.4	14 - 86	1.0	0.4 - 2.5	10.4	3.9 - 27.2	2.1	0.6 - 7.5	9.7	3.3 - 26.9	0.0	0.0 - 0.0	14.2	5.1 - 36.9	0.29
3050108	6064	ENOREE R	44.4	39.7	15 - 85	0.0	0.0 - 0.0	10.0	3.0 - 21.4	2.5	0.8 - 6.2	13.1	4.6 - 32.9	0.0	0.0 - 0.0	14.1	5.4 - 32.0	0.28
3050108	6065	ENOREE R	71.0	41.6	15 - 105	7.1	2.8 - 18.0	6.8	2.1 - 16.6	2.6	0.8 - 7.7	14.2	4.5 - 42.2	0.0	0.0 - 0.0	10.8	3.8 - 29.0	0.26
3050108	6066	ENOREE R	25.5	512.8	183 - 1168	466.6	165.0 - 1041.3	14.9	4.7 - 32.5	2.4	0.8 - 6.2	16.0	4.6 - 42.6	0.0	0.0 - 0.0	12.9	4.6 - 29.3	0.23

8-digit HUC	ID	Name	Area	Catchment Yield		Point sources		Developed Land		Manure		Agricultural Land		Phosphate Mines		Soil parent rock		Frac
				kg/km^2	90% CI	kg/km^2	90% CI	kg/km^2	90% CI	kg/km^2	90% CI	kg/km^2	90% CI	kg/km^2	90% CI	kg/km^2	90% CI	
3050108	6067	ABNER CR	30.0	54.2	22 - 94	0.0	0.0 - 0.0	21.2	8.2 - 45.9	2.3	0.7 - 6.8	16.2	5.9 - 36.3	0.0	0.0 - 0.0	14.5	5.9 - 34.8	0.21
3050108	6068	ENOREE R	60.5	359.5	118 - 828	280.3	87.0 - 655.1	54.0	18.4 - 141.5	1.1	0.3 - 3.9	7.5	2.2 - 18.7	0.0	0.0 - 0.0	16.6	5.7 - 43.7	0.21
3050108	6069	ENOREE R	179.0	162.6	49 - 287	104.8	33.8 - 206.7	35.6	11.3 - 70.0	1.2	0.4 - 2.8	7.2	2.2 - 15.6	0.0	0.0 - 0.0	13.9	4.3 - 31.6	0.19
3050108	6070	BRUSHY CR	38.4	99.2	36 - 232	0.0	0.0 - 0.0	82.5	27.7 - 193.4	0.2	0.1 - 0.6	1.4	0.5 - 4.0	0.0	0.0 - 0.0	15.1	5.5 - 41.5	0.19
3050108	6071	HORSEPEN CR	8.2	34.1	14 - 81	0.0	0.0 - 0.0	8.1	3.2 - 18.9	1.4	0.4 - 4.0	11.2	4.3 - 32.5	0.0	0.0 - 0.0	13.4	5.0 - 31.9	0.23
3050108	6072	GILDER CR	13.0	56.1	19 - 118	0.0	0.0 - 0.0	26.8	9.5 - 54.0	1.8	0.6 - 5.1	14.2	5.0 - 38.3	0.0	0.0 - 0.0	13.2	4.2 - 33.3	0.21
3050108	6073	HORSEPEN CR	73.7	70.8	25 - 167	0.0	0.0 - 0.0	49.3	17.8 - 127.8	1.0	0.3 - 3.3	7.5	2.5 - 20.1	0.0	0.0 - 0.0	13.0	4.7 - 31.9	0.21
3050108	6074	DURBIN CR	4.2	35.5	12 - 76	0.0	0.0 - 0.0	5.0	1.7 - 10.2	3.5	1.1 - 9.2	12.6	4.4 - 33.8	0.0	0.0 - 0.0	14.4	5.2 - 35.7	0.26
3050108	6075	DURBIN CR	80.2	77.8	25 - 220	36.9	12.5 - 104.7	16.5	4.9 - 47.7	2.2	0.6 - 7.2	11.5	3.5 - 30.2	0.0	0.0 - 0.0	10.8	3.9 - 30.4	0.25
3050108	6076	S DURBIN CR	49.9	38.6	13 - 100	0.0	0.0 - 0.0	9.5	2.9 - 29.0	3.6	1.2 - 11.4	14.6	5.0 - 41.9	0.0	0.0 - 0.0	10.8	4.1 - 30.8	0.25
3050108	6077	BEAVER DAM CR	47.8	40.2	14 - 87	0.0	0.0 - 0.0	7.7	2.7 - 15.6	4.2	1.3 - 11.2	16.4	5.8 - 39.2	0.0	0.0 - 0.0	11.9	4.2 - 27.2	0.28
3050108	6078	WARRIOR CR	94.1	31.8	10 - 81	0.0	0.0 - 0.0	5.1	1.6 - 13.5	3.0	0.9 - 8.5	11.3	3.7 - 33.7	0.0	0.0 - 0.0	12.4	4.1 - 33.0	0.29
3050108	6079	DUNCAN CR	34.5	51.8	19 - 95	22.3	7.5 - 41.9	10.8	3.1 - 18.9	1.8	0.6 - 6.3	2.4	0.8 - 5.1	0.0	0.0 - 0.0	14.5	4.8 - 33.1	0.33
3050108	6080	DUNCAN CR	109.3	28.2	9 - 50	0.0	0.0 - 0.0	8.1	2.6 - 15.7	1.5	0.4 - 4.0	4.2	1.3 - 8.9	0.0	0.0 - 0.0	14.3	4.1 - 29.8	0.32
3050108	6081	DUNCAN CR	75.2	33.4	11 - 55	0.0	0.0 - 0.0	9.0	2.9 - 16.1	2.5	0.7 - 6.5	9.2	3.2 - 19.1	0.0	0.0 - 0.0	12.6	3.7 - 27.5	0.28
3050108	6082	BEARDS CR	29.8	34.4	15 - 70	0.0	0.0 - 0.0	17.2	6.8 - 34.9	1.4	0.5 - 4.7	4.0	1.5 - 9.5	0.0	0.0 - 0.0	11.8	4.6 - 27.3	0.28
3050108	6083	INDIAN CR	30.9	22.8	7 - 48	0.0	0.0 - 0.0	5.3	1.7 - 11.8	2.3	0.7 - 6.4	2.7	0.9 - 7.6	0.0	0.0 - 0.0	12.5	4.0 - 27.8	0.34
3050108	6084	INDIAN CR	52.7	18.5	5 - 44	0.0	0.0 - 0.0	4.1	1.2 - 10.6	1.2	0.3 - 3.2	1.6	0.4 - 3.8	0.0	0.0 - 0.0	11.7	3.5 - 28.6	0.32
3050108	6085	HEADLEYS CR	28.5	22.5	8 - 43	0.0	0.0 - 0.0	6.9	2.4 - 13.1	1.2	0.4 - 4.3	3.0	1.0 - 7.3	0.0	0.0 - 0.0	11.4	4.0 - 26.6	0.30
3050108	6086	INDIAN CR	88.8	24.2	9 - 51	0.0	0.0 - 0.0	5.4	1.8 - 11.9	2.0	0.6 - 5.8	4.2	1.4 - 9.8	0.0	0.0 - 0.0	12.5	4.2 - 30.8	0.30
3050108	6087	GILDERS CR	50.4	25.2	7 - 44	0.0	0.0 - 0.0	5.3	1.4 - 9.6	3.6	0.9 - 8.5	5.1	1.3 - 11.2	0.0	0.0 - 0.0	11.2	3.3 - 23.4	0.32
3050108	6088	KINGS CR	67.4	22.7	8 - 52	0.0	0.0 - 0.0	7.4	2.7 - 17.7	2.1	0.7 - 6.3	3.0	1.1 - 7.2	0.0	0.0 - 0.0	10.2	3.3 - 25.3	0.34
3050108	6089	DUNCAN CR, S FK	62.1	24.4	9 - 62	0.0	0.0 - 0.0	4.7	1.9 - 12.0	1.3	0.4 - 5.5	4.5	1.6 - 13.0	0.0	0.0 - 0.0	13.8	4.8 - 38.4	0.32
3050109	6090	SALADA R	12.7	86.1	29 - 177	0.0	0.0 - 0.0	78.9	27.2 - 170.6	0.2	0.1 - 0.7	0.3	0.1 - 0.9	0.0	0.0 - 0.0	6.6	2.0 - 16.4	0.43
3050109	6091	SALADA R	42.4	80.7	27 - 158	16.1	6.0 - 36.5	45.0	15.0 - 103.6	1.1	0.3 - 2.8	2.8	1.0 - 6.6	0.0	0.0 - 0.0	15.6	6.2 - 34.0	0.42
3050109	6092	LITTLE R	16.2	35.2	13 - 70	0.0	0.0 - 0.0	3.8	1.1 - 8.0	7.7	2.1 - 23.0	10.9	3.8 - 26.5	0.0	0.0 - 0.0	12.8	4.4 - 31.1	0.12
3050109	6093	LITTLE R	45.9	31.3	9 - 72	0.0	0.0 - 0.0	4.3	1.3 - 9.7	6.3	1.6 - 15.3	8.8	3.0 - 22.8	0.0	0.0 - 0.0	12.0	3.0 - 25.6	0.11
3050109	6094	GARRISON CR	48.7	27.9	8 - 62	0.0	0.0 - 0.0	5.0	1.4 - 11.7	3.5	1.0 - 10.2	8.5	2.8 - 22.2	0.0	0.0 - 0.0	10.8	3.4 - 30.1	0.11
3050109	6095	LITTLE R	21.3	25.0	9 - 48	0.0	0.0 - 0.0	5.6	2.2 - 12.4	2.4	0.8 - 7.5	3.6	1.3 - 8.6	0.0	0.0 - 0.0	13.5	5.4 - 29.1	0.11
3050109	6096	*A	33.9	26.5	11 - 55	0.0	0.0 - 0.0	4.5	1.6 - 9.8	2.1	0.7 - 5.7	6.6	2.4 - 17.2	0.0	0.0 - 0.0	13.3	4.9 - 30.9	0.10
3050109	6097	LITTLE R	1.8	28.0	11 - 53	0.0	0.0 - 0.0	3.8	1.5 - 8.2	4.7	1.6 - 11.6	4.6	1.8 - 10.0	0.0	0.0 - 0.0	14.9	5.8 - 35.1	0.10
3050109	6098	NORTH CR	45.1	28.9	9 - 53	0.0	0.0 - 0.0	8.9	2.6 - 18.5	2.0	0.6 - 5.8	6.9	2.4 - 16.0	0.0	0.0 - 0.0	11.2	4.0 - 24.1	0.10
3050109	6099	LITTLE R	173.1	47.1	16 - 132	11.8	4.5 - 38.7	13.3	4.3 - 34.6	2.3	0.7 - 7.6	8.1	2.7 - 25.1	0.0	0.0 - 0.0	11.6	4.1 - 35.2	0.10
3050109	6100	BEAVER DAM CR	51.3	27.0	8 - 61	0.0	0.0 - 0.0	3.7	1.1 - 8.9	2.9	0.8 - 8.5	9.4	2.8 - 26.3	0.0	0.0 - 0.0	11.1	3.6 - 27.9	0.10
3050109	6101	MUDLICK CR	132.5	24.9	8 - 56	0.0	0.0 - 0.0	4.4	1.6 - 9.4	2.7	0.9 - 7.3	6.3	2.3 - 15.4	0.0	0.0 - 0.0	11.5	4.1 - 31.5	0.11
3050109	6102	SALADA R	96.8	25.8	10 - 64	0.0	0.0 - 0.0	4.0	1.4 - 9.4	4.1	1.3 - 13.7	4.6	1.8 - 13.1	0.0	0.0 - 0.0	13.2	5.3 - 36.5	0.12
3050109	6103	SALADA R	9.0	24.2	8 - 47	0.0	0.0 - 0.0	5.4	1.7 - 10.8	2.1	0.6 - 4.8	2.5	0.8 - 6.1	0.0	0.0 - 0.0	14.3	4.8 - 33.4	0.12
3050109	6105	N RABON CR	133.8	36.8	15 - 66	0.0	0.0 - 0.0	9.2	3.2 - 18.7	3.3	1.0 - 8.2	12.8	4.9 - 31.5	0.0	0.0 - 0.0	11.5	4.3 - 23.1	0.06
3050109	6106	S RABON CR	22.0	33.2	11 - 86	0.0	0.0 - 0.0	6.4	1.9 - 16.1	3.5	1.1 - 11.1	13.0	3.8 - 41.5	0.0	0.0 - 0.0	10.3	3.9 - 26.5	0.06

8-digit HUC	ID	Name	Area	Catchment Yield		Point sources		Developed Land		Manure		Agricultural Land		Phosphate Mines		Soil parent rock		Frac
				kg/km^2	90% CI	kg/km^2	90% CI	kg/km^2	90% CI	kg/km^2	90% CI	kg/km^2	90% CI	kg/km^2	90% CI	kg/km^2	90% CI	
3050109	6107	REEDY R	84.7	31.6	12 - 64	0.0	0.0 - 0.0	5.8	1.8 - 13.0	3.0	0.9 - 10.4	10.5	3.7 - 24.4	0.0	0.0 - 0.0	12.2	4.1 - 27.5	0.06
3050109	6108	REEDY R	81.6	26.5	11 - 57	0.0	0.0 - 0.0	3.8	1.4 - 8.2	1.7	0.5 - 4.9	8.2	3.0 - 21.2	0.0	0.0 - 0.0	12.8	4.7 - 29.5	0.06
3050109	6109	REEDY R	13.9	32.9	13 - 61	0.0	0.0 - 0.0	6.1	2.0 - 12.3	1.8	0.5 - 4.8	11.2	3.7 - 25.3	0.0	0.0 - 0.0	13.9	4.5 - 32.3	0.06
3050109	6110	HUFF CR	92.8	52.2	20 - 100	0.5	0.2 - 1.1	19.3	7.3 - 36.8	2.2	0.6 - 5.8	15.5	6.2 - 34.5	0.0	0.0 - 0.0	14.7	5.2 - 33.1	0.06
3050109	6111	HORSE CR	60.9	27.7	10 - 49	0.0	0.0 - 0.0	5.2	1.8 - 10.2	1.5	0.4 - 3.9	8.2	2.7 - 17.6	0.0	0.0 - 0.0	12.8	4.5 - 26.1	0.06
3050109	6112	WALNUT CR	31.5	43.5	17 - 107	0.0	0.0 - 0.0	5.8	2.1 - 15.0	4.7	1.6 - 17.2	16.9	5.5 - 45.7	0.0	0.0 - 0.0	16.2	6.8 - 41.8	0.06
3050109	6113	SALADA R	26.4	24.8	8 - 50	0.0	0.0 - 0.0	3.6	1.1 - 8.3	1.7	0.6 - 4.7	4.9	1.6 - 11.2	0.0	0.0 - 0.0	14.6	4.7 - 35.9	0.06
3050109	6114	SALADA R	1.4	22.7	8 - 40	0.0	0.0 - 0.0	5.0	1.7 - 10.5	1.9	0.7 - 4.5	3.3	1.1 - 8.0	0.0	0.0 - 0.0	12.4	4.5 - 23.9	0.06
3050109	6115	SALADA R	13.6	26.4	8 - 50	0.0	0.0 - 0.0	5.4	1.8 - 11.0	2.2	0.7 - 5.3	6.2	1.9 - 14.0	0.0	0.0 - 0.0	12.5	3.6 - 29.0	0.06
3050109	6116	MOUNTAIN CR	54.1	27.5	9 - 51	0.0	0.0 - 0.0	6.0	1.8 - 12.7	1.4	0.4 - 4.3	7.7	2.7 - 20.5	0.0	0.0 - 0.0	12.4	4.1 - 28.1	0.06
3050109	6117	SALADA R	55.9	93.1	30 - 238	59.6	19.5 - 159.7	5.9	1.8 - 16.6	3.4	0.9 - 9.8	11.5	3.5 - 34.8	0.0	0.0 - 0.0	12.7	3.8 - 32.2	0.06
3050109	6118	SALADA R	8.0	205.1	58 - 388	175.4	49.3 - 334.0	5.4	1.6 - 10.7	2.0	0.5 - 5.6	6.6	1.7 - 16.9	0.0	0.0 - 0.0	15.8	5.0 - 28.8	0.05
3050109	6119	GROVE CR	37.1	35.4	13 - 75	0.0	0.0 - 0.0	6.4	2.2 - 13.4	1.8	0.5 - 4.8	11.7	3.9 - 29.2	0.0	0.0 - 0.0	15.6	5.1 - 34.0	0.05
3050109	6120	SALADA R	80.0	74.0	28 - 149	14.3	5.9 - 31.1	25.5	9.2 - 53.5	4.1	1.3 - 11.3	12.8	4.9 - 30.3	0.0	0.0 - 0.0	17.3	6.4 - 35.5	0.05
3050109	6121	SALADA R	41.6	68.2	22 - 179	0.0	0.0 - 0.0	43.9	14.6 - 116.2	1.6	0.4 - 4.9	4.4	1.5 - 14.3	0.0	0.0 - 0.0	18.3	6.0 - 50.1	0.05
3050109	6122	SALADA R	32.5	49.6	16 - 105	0.0	0.0 - 0.0	30.7	10.6 - 68.3	0.7	0.2 - 1.9	3.9	1.2 - 12.3	0.0	0.0 - 0.0	14.2	4.4 - 32.2	0.05
3050109	6123	N SALUDA R	198.4	16.2	6 - 29	0.9	0.3 - 1.7	5.2	2.0 - 10.4	0.4	0.1 - 1.0	2.0	0.6 - 4.8	0.0	0.0 - 0.0	7.8	2.8 - 15.4	0.04
3050109	6124	S SALUDA R	46.5	28.0	11 - 56	0.0	0.0 - 0.0	9.4	3.3 - 19.1	1.9	0.6 - 4.1	6.0	2.1 - 14.1	0.0	0.0 - 0.0	10.8	4.7 - 22.6	0.04
3050109	6125	MID SALUDA R	74.7	23.8	8 - 50	0.0	0.0 - 0.0	8.4	3.0 - 18.9	0.5	0.1 - 1.4	2.8	1.0 - 6.8	0.0	0.0 - 0.0	12.1	4.3 - 28.2	0.03
3050109	6126	S SALUDA R	17.3	26.6	9 - 66	0.0	0.0 - 0.0	8.0	2.3 - 17.1	1.2	0.3 - 3.3	4.6	1.4 - 13.0	0.0	0.0 - 0.0	12.7	4.3 - 31.7	0.03
3050109	6127	S SALUDA R	249.2	13.2	4 - 30	0.0	0.0 - 0.1	4.2	1.0 - 8.9	0.3	0.1 - 0.9	1.1	0.3 - 2.7	0.0	0.0 - 0.0	7.6	2.4 - 17.4	0.03
3050109	6128	GEORGES CR	84.7	62.3	22 - 116	7.3	2.3 - 15.1	28.3	9.1 - 60.4	2.2	0.6 - 6.9	10.8	3.5 - 30.4	0.0	0.0 - 0.0	13.6	4.3 - 30.7	0.05
3050109	6129	BIG BRUSHY CR	95.6	100.3	35 - 198	33.6	11.0 - 78.4	33.0	10.1 - 71.8	3.6	1.0 - 10.2	12.0	4.2 - 30.7	0.0	0.0 - 0.0	18.1	6.9 - 42.5	0.05
3050109	6130	*B	53.3	55.2	15 - 99	0.0	0.0 - 0.0	18.2	4.4 - 35.8	5.3	1.4 - 15.2	16.0	4.1 - 35.6	0.0	0.0 - 0.0	15.7	4.6 - 29.8	0.05
3050109	6131	LITTLE CR	11.9	35.6	13 - 66	0.0	0.0 - 0.0	7.2	2.4 - 14.7	4.4	1.3 - 11.6	13.2	4.8 - 30.4	0.0	0.0 - 0.0	10.8	3.8 - 25.0	0.06
3050109	6132	BROAD MOUTH CI	84.9	38.6	13 - 90	0.0	0.0 - 0.0	10.8	3.3 - 26.9	4.1	1.3 - 12.9	11.7	3.7 - 32.5	0.0	0.0 - 0.0	12.0	4.4 - 29.0	0.06
3050109	6133	TURKEY CR	118.9	27.8	10 - 57	0.0	0.0 - 0.0	6.8	2.2 - 14.7	2.4	0.7 - 6.1	7.4	2.8 - 16.1	0.0	0.0 - 0.0	11.2	4.3 - 27.6	0.06
3050109	6134	NINETY-SIX CR	9.8	22.4	6 - 47	2.1	0.6 - 4.3	6.8	1.7 - 13.5	0.5	0.1 - 1.8	0.6	0.2 - 1.4	0.0	0.0 - 0.0	12.4	3.6 - 29.0	0.12
3050109	6135	CORONACA CR	201.6	146.9	53 - 273	109.9	39.5 - 220.3	19.6	6.9 - 37.7	1.6	0.5 - 4.8	5.5	2.0 - 13.3	0.0	0.0 - 0.0	10.3	3.5 - 24.8	0.11
3050109	6136	NINETY-SIX CR	161.1	30.8	9 - 77	2.9	0.9 - 7.9	7.1	2.1 - 18.3	2.2	0.6 - 7.7	7.4	2.2 - 19.3	0.0	0.0 - 0.0	11.2	3.7 - 31.0	0.11
3050109	6137	HALFWAY SWAMI	90.5	31.4	11 - 65	0.0	0.0 - 0.0	3.9	1.2 - 9.3	5.4	1.7 - 16.5	9.1	2.9 - 22.6	0.0	0.0 - 0.0	13.0	4.4 - 31.2	0.12
3050109	6138	LITTLE SALUDA R	49.4	62.3	24 - 119	0.0	0.0 - 0.0	10.2	3.5 - 19.7	15.9	4.7 - 45.0	21.6	8.5 - 50.8	0.0	0.0 - 0.0	14.5	5.1 - 39.4	0.12
3050109	6139	RED BANK CR	112.1	51.4	21 - 132	0.0	0.0 - 0.0	6.5	2.4 - 17.8	13.7	4.5 - 40.5	17.8	6.5 - 54.7	0.0	0.0 - 0.0	13.3	5.3 - 40.2	0.11
3050109	6140	MINE CR	116.3	36.1	9 - 71	0.0	0.0 - 0.0	5.0	1.3 - 11.0	8.8	1.9 - 24.5	10.9	2.9 - 24.6	0.0	0.0 - 0.0	11.5	2.8 - 26.6	0.11
3050109	6141	RICHLAND CR	50.4	54.6	17 - 99	0.0	0.0 - 0.0	5.3	1.7 - 10.5	15.4	4.6 - 40.8	20.6	6.9 - 46.6	0.0	0.0 - 0.0	13.4	4.5 - 30.0	0.12
3050109	6142	TWELVEMILE CR	153.9	61.2	21 - 161	19.7	6.8 - 52.7	24.9	8.7 - 67.6	3.0	0.8 - 8.8	5.1	1.7 - 15.6	0.0	0.0 - 0.0	8.5	3.2 - 22.5	0.42
3050110	6143	CONGAREE R	7.1	24.3	9 - 54	0.0	0.0 - 0.0	2.3	0.7 - 5.1	0.5	0.1 - 1.2	10.8	3.2 - 25.3	0.0	0.0 - 0.0	10.7	4.0 - 23.9	0.44
3050110	6144	CONGAREE R	2.0	15.9	6 - 41	0.0	0.0 - 0.0	2.0	0.7 - 5.2	0.1	0.0 - 0.2	0.0	0.0 - 0.0	0.0	0.0 - 0.0	13.8	4.9 - 34.6	0.44
3050110	6145	*A	44.5	27.2	10 - 58	0.0	0.0 - 0.0	6.0	1.9 - 13.3	0.7	0.2 - 1.8	9.8	3.8 - 23.5	0.0	0.0 - 0.0	10.8	3.8 - 27.0	0.44

8-digit HUC	ID	Name	Area	Catchment Yield		Point sources		Developed Land		Manure		Agricultural Land		Phosphate Mines		Soil parent rock		Frac
				kg/km^2	90% CI	kg/km^2	90% CI	kg/km^2	90% CI	kg/km^2	90% CI	kg/km^2	90% CI	kg/km^2	90% CI	kg/km^2	90% CI	
3050110	6146	CONGAREE R	1.3	18.0	6 - 46	0.0	0.0 - 0.0	1.6	0.5 - 3.6	0.0	0.0 - 0.1	0.6	0.2 - 1.6	0.0	0.0 - 0.0	15.7	5.2 - 41.9	0.44
3050110	6147	GRIFFIN CR	49.8	21.4	6 - 50	0.0	0.0 - 0.0	5.7	1.7 - 12.6	0.4	0.1 - 1.2	5.9	1.7 - 14.9	0.0	0.0 - 0.0	9.4	2.8 - 24.7	0.44
3050110	6148	CONGAREE R	19.8	13.8	5 - 36	0.0	0.0 - 0.0	0.7	0.2 - 1.4	0.0	0.0 - 0.1	0.4	0.1 - 1.0	0.0	0.0 - 0.0	12.7	4.2 - 34.8	0.44
3050110	6149	TOMS CR	131.5	19.5	7 - 55	0.0	0.0 - 0.0	4.6	1.3 - 14.5	0.4	0.1 - 1.5	6.3	2.1 - 19.3	0.0	0.0 - 0.0	8.2	2.6 - 20.1	0.43
3050110	6150	CONGAREE R	1.5	15.4	5 - 38	0.0	0.0 - 0.0	0.4	0.1 - 1.1	0.1	0.0 - 0.3	2.0	0.7 - 5.2	0.0	0.0 - 0.0	12.9	4.2 - 34.6	0.43
3050110	6151	CONGAREE R	69.6	14.4	5 - 27	0.0	0.0 - 0.0	3.6	1.1 - 7.6	0.2	0.1 - 0.7	3.7	1.1 - 8.9	0.0	0.0 - 0.0	6.9	2.2 - 17.4	0.43
3050110	6152	CEDAR CR	33.0	22.1	9 - 54	0.2	0.1 - 0.4	2.1	0.8 - 5.5	0.4	0.1 - 0.9	4.6	1.7 - 11.7	0.0	0.0 - 0.0	14.9	5.4 - 37.6	0.43
3050110	6153	CONGAREE R	8.0	16.6	5 - 44	0.0	0.0 - 0.0	1.4	0.4 - 3.5	0.1	0.0 - 0.3	2.2	0.6 - 6.5	0.0	0.0 - 0.0	12.9	4.1 - 35.2	0.43
3050110	6154	CONGAREE R, DRY	64.7	22.4	7 - 42	0.0	0.0 - 0.0	5.9	1.7 - 12.5	0.5	0.1 - 1.2	6.9	2.6 - 17.8	0.0	0.0 - 0.0	9.2	2.7 - 19.3	0.43
3050110	6156	CONGAREE R	7.8	20.1	7 - 53	0.0	0.0 - 0.0	0.4	0.1 - 1.0	0.2	0.1 - 0.7	2.5	0.8 - 7.1	0.0	0.0 - 0.0	17.0	5.5 - 45.6	0.43
3050110	6157	MEYERS CR	17.8	16.3	5 - 39	0.0	0.0 - 0.0	0.1	0.0 - 0.1	0.0	0.0 - 0.1	0.2	0.1 - 0.4	0.0	0.0 - 0.0	16.0	5.2 - 38.0	0.43
3050110	6158	CEDAR CR	98.1	20.4	6 - 45	0.2	0.1 - 0.5	8.4	2.4 - 18.8	0.3	0.1 - 1.0	4.6	1.4 - 15.7	0.0	0.0 - 0.0	6.9	2.2 - 18.5	0.42
3050110	6159	MEYERS CR	6.0	38.4	13 - 73	0.0	0.0 - 0.0	6.9	2.3 - 13.2	0.9	0.3 - 2.6	12.4	4.9 - 28.0	0.0	0.0 - 0.0	18.1	5.8 - 41.2	0.42
3050110	6160	MEYERS CR, CABI	41.1	26.9	8 - 54	0.5	0.2 - 1.2	9.7	2.9 - 20.4	0.6	0.2 - 1.7	8.9	2.9 - 21.3	0.0	0.0 - 0.0	7.2	2.3 - 15.8	0.41
3050110	6161	MEYERS CR	31.5	26.4	10 - 52	0.0	0.0 - 0.0	8.5	2.9 - 16.2	0.5	0.1 - 1.1	5.9	2.0 - 12.8	0.0	0.0 - 0.0	11.5	4.0 - 25.8	0.41
3050110	6162	CONGAREE R	5.6	20.2	7 - 49	0.0	0.0 - 0.0	0.7	0.2 - 1.3	0.1	0.0 - 0.2	0.2	0.1 - 0.4	0.0	0.0 - 0.0	19.3	7.0 - 47.9	0.43
3050110	6163	MILL CR	127.8	30.3	10 - 55	0.0	0.0 - 0.0	13.3	4.2 - 24.9	0.3	0.1 - 0.8	4.2	1.4 - 9.9	0.0	0.0 - 0.0	12.4	3.8 - 23.6	0.43
3050110	6164	CONGAREE R	19.5	18.8	7 - 35	0.0	0.0 - 0.0	2.7	1.0 - 5.5	0.2	0.1 - 0.5	2.5	0.9 - 6.5	0.0	0.0 - 0.0	13.4	4.9 - 28.8	0.43
3050110	6165	CONGAREE R	67.3	23.2	6 - 42	1.5	0.5 - 3.1	7.4	2.3 - 15.1	0.7	0.2 - 1.8	3.0	0.9 - 7.0	0.0	0.0 - 0.0	10.5	3.1 - 23.8	0.43
3050110	6166	GILLS CR	45.1	64.6	20 - 140	0.0	0.0 - 0.0	35.1	11.2 - 77.6	0.9	0.3 - 2.4	14.2	4.8 - 39.3	0.0	0.0 - 0.0	14.5	4.5 - 35.5	0.43
3050110	6167	*A	62.2	32.7	13 - 64	0.0	0.0 - 0.0	23.8	8.9 - 47.2	0.1	0.0 - 0.4	1.2	0.4 - 3.5	0.0	0.0 - 0.0	7.5	3.0 - 15.5	0.39
3050110	6168	GILLS CR	17.6	85.4	31 - 192	0.0	0.0 - 0.0	76.0	27.2 - 173.6	0.0	0.0 - 0.0	0.2	0.1 - 0.5	0.0	0.0 - 0.0	9.3	3.2 - 24.2	0.39
3050110	6169	*B	19.4	58.5	16 - 135	0.2	0.1 - 0.5	50.7	13.1 - 119.0	0.0	0.0 - 0.1	0.4	0.1 - 1.2	0.0	0.0 - 0.0	7.1	2.2 - 18.7	0.37
3050110	6170	GILLS CR	26.6	70.6	29 - 135	0.0	0.0 - 0.0	62.3	24.3 - 120.0	0.0	0.0 - 0.1	0.2	0.1 - 0.6	0.0	0.0 - 0.0	8.1	3.3 - 19.1	0.37
3050110	6171	CONGAREE R	34.8	37.5	12 - 76	5.3	1.6 - 11.6	18.8	5.4 - 41.9	1.9	0.6 - 5.5	3.0	0.8 - 7.8	0.0	0.0 - 0.0	8.5	2.9 - 19.1	0.43
3050110	6172	CONGAREE R	43.4	1986.0	817 - 5190	1908.4	770.6 - 5091.3	65.8	25.9 - 151.7	0.2	0.1 - 0.7	1.9	0.7 - 5.5	0.0	0.0 - 0.0	9.7	4.1 - 32.0	0.43
3050110	6173	CONGAREE CR	66.2	55.3	19 - 116	0.5	0.2 - 1.0	45.6	16.1 - 98.6	0.5	0.2 - 1.3	0.8	0.3 - 1.7	0.0	0.0 - 0.0	7.9	2.8 - 18.0	0.43
3050110	6174	*C	18.7	59.8	18 - 132	0.0	0.0 - 0.0	50.5	14.9 - 118.3	0.6	0.2 - 2.1	0.9	0.3 - 2.2	0.0	0.0 - 0.0	7.8	2.3 - 19.3	0.41
3050110	6175	CONGAREE CR	2.1	34.4	11 - 96	0.0	0.0 - 0.0	22.7	7.1 - 54.8	1.4	0.4 - 5.0	1.8	0.6 - 6.6	0.0	0.0 - 0.0	8.4	2.9 - 25.7	0.41
3050110	6176	CONGAREE CR	9.8	45.9	13 - 116	0.0	0.0 - 0.0	33.6	8.8 - 86.7	1.6	0.4 - 4.3	2.3	0.7 - 7.4	0.0	0.0 - 0.0	8.4	2.6 - 24.3	0.40
3050110	6177	RED BANK CR	84.3	48.3	19 - 120	8.5	3.6 - 20.3	27.8	9.8 - 73.2	1.6	0.6 - 4.7	2.6	1.1 - 7.4	0.0	0.0 - 0.0	7.7	2.9 - 19.3	0.40
3050110	6178	CONGAREE CR	35.0	31.7	12 - 69	0.0	0.0 - 0.0	19.3	7.0 - 42.8	1.8	0.6 - 4.8	2.7	1.1 - 7.4	0.0	0.0 - 0.0	7.9	2.9 - 18.2	0.40
3050110	6179	CONGAREE CR	34.1	20.6	6 - 33	0.0	0.0 - 0.0	7.0	2.1 - 13.0	2.2	0.5 - 5.1	3.5	0.9 - 7.6	0.0	0.0 - 0.0	7.8	2.3 - 15.3	0.35
3050110	6180	*D	26.8	18.0	7 - 45	0.0	0.0 - 0.0	7.5	2.7 - 19.1	1.8	0.6 - 6.5	2.5	0.9 - 7.2	0.0	0.0 - 0.0	6.1	2.4 - 15.5	0.35
3050110	6181	FIRST CR	13.5	48.5	16 - 110	0.0	0.0 - 0.0	37.2	11.7 - 85.3	1.3	0.4 - 3.3	1.7	0.5 - 5.3	0.0	0.0 - 0.0	8.3	2.7 - 22.1	0.40
3050110	6182	SECOND CR	39.4	41.6	16 - 120	21.4	8.9 - 62.9	7.8	3.0 - 20.1	2.4	0.8 - 7.9	3.9	1.4 - 12.9	0.0	0.0 - 0.0	6.2	2.2 - 18.7	0.38
3050110	6183	FIRST CR	37.5	26.5	10 - 44	0.0	0.0 - 0.0	14.3	5.3 - 26.0	2.4	0.7 - 6.5	3.6	1.3 - 8.1	0.0	0.0 - 0.0	6.1	2.2 - 13.0	0.38
3050110	6184	SANDY RUN	97.7	17.6	7 - 42	0.0	0.0 - 0.0	7.1	2.7 - 16.1	0.9	0.3 - 2.6	3.0	1.1 - 8.7	0.0	0.0 - 0.0	6.6	2.3 - 16.4	0.43
3050110	6185	BIG BEAVER CR	134.2	16.3	5 - 32	0.0	0.0 - 0.0	5.2	1.4 - 11.8	0.5	0.1 - 1.3	2.3	0.6 - 6.0	0.0	0.0 - 0.0	8.4	2.6 - 17.5	0.43

8-digit HUC	ID	Name	Area	Catchment Yield		Point sources		Developed Land		Manure		Agricultural Land		Phosphate Mines		Soil parent rock		Frac
				kg/km^2	90% CI	kg/km^2	90% CI	kg/km^2	90% CI	kg/km^2	90% CI	kg/km^2	90% CI	kg/km^2	90% CI	kg/km^2	90% CI	
3050110	6186	BUCKHEAD CR	49.4	15.9	5 - 30	0.0	0.0 - 0.0	3.3	1.0 - 6.6	0.2	0.1 - 0.5	4.3	1.5 - 9.9	0.0	0.0 - 0.0	8.1	2.7 - 17.5	0.44
3050112	6187	N SANTEE R	115.0	2.2	1 - 5	0.5	0.2 - 0.9	0.0	0.0 - 0.1	0.0	0.0 - 0.0	0.0	0.0 - 0.2	0.0	0.0 - 0.0	1.6	0.3 - 5.0	1.00
3050112	6188	SANTEE R	41.9	13.8	5 - 31	0.0	0.0 - 0.0	0.1	0.1 - 0.3	0.1	0.0 - 0.1	0.2	0.1 - 0.6	0.0	0.0 - 0.0	13.3	4.8 - 30.7	0.99
3050112	6189	SANTEE R	610.5	19.1	5 - 37	0.8	0.2 - 1.6	1.8	0.4 - 3.7	0.4	0.1 - 1.0	6.8	1.8 - 15.4	0.0	0.0 - 0.0	9.2	2.6 - 18.6	0.99
3050112	6190	*A	168.0	19.9	8 - 39	0.0	0.0 - 0.0	4.5	1.6 - 9.0	0.9	0.3 - 2.8	8.6	3.3 - 24.5	0.0	0.0 - 0.0	5.9	2.1 - 14.9	0.98
3050112	6191	SANTEE R	262.9	16.3	6 - 33	0.0	0.0 - 0.0	1.3	0.4 - 2.8	0.9	0.3 - 2.5	5.8	2.3 - 13.8	0.0	0.0 - 0.0	8.3	2.9 - 23.5	0.98
3050112	6192	ECHAW CR	133.2	10.0	3 - 27	0.0	0.0 - 0.0	0.6	0.2 - 1.5	0.1	0.0 - 0.2	1.1	0.4 - 3.1	0.0	0.0 - 0.0	8.3	2.5 - 20.7	0.99
3050112	6193	WAMBAW CR	263.6	9.4	3 - 21	0.0	0.0 - 0.0	1.1	0.4 - 2.4	0.1	0.0 - 0.2	0.2	0.1 - 0.5	0.0	0.0 - 0.0	8.0	2.9 - 19.4	1.00
3050112	6194	S SANTEE R	84.7	1.0	0 - 5	0.0	0.0 - 0.0	0.0	0.0 - 0.1	0.0	0.0 - 0.0	0.0	0.0 - 0.1	0.0	0.0 - 0.0	0.9	0.2 - 4.4	1.00
3050112	6195	S SANTEE R	6.2	33.3	11 - 89	0.0	0.0 - 0.0	0.0	0.0 - 0.0	0.0	0.0 - 0.0	0.0	0.0 - 0.0	0.0	0.0 - 0.0	33.3	10.9 - 89.3	1.00
3050201	6196	COOPER R	27.6	708.9	244 - 1843	708.8	244.3 - 1842.1	0.0	0.0 - 0.1	0.0	0.0 - 0.0	0.0	0.0 - 0.1	0.0	0.0 - 0.0	0.1	0.0 - 0.7	1.00
3050201	6197	COOPER R	60.6	54.9	16 - 106	54.1	15.5 - 109.3	0.1	0.0 - 0.6	0.0	0.0 - 0.0	0.0	0.0 - 0.2	0.0	0.0 - 0.0	0.5	0.1 - 2.0	0.99
3050201	6198	COOPER R, E BR	16.7	5.6	2 - 13	0.0	0.0 - 0.0	0.1	0.0 - 0.1	0.0	0.0 - 0.0	0.1	0.0 - 0.1	0.0	0.0 - 0.0	5.4	1.6 - 12.9	0.97
3050201	6199	*A	61.3	17.9	6 - 33	0.0	0.0 - 0.0	1.5	0.5 - 2.8	0.1	0.0 - 0.3	1.7	0.6 - 3.3	0.0	0.0 - 0.0	14.6	5.0 - 27.8	0.96
3050201	6200	COOPER R, E BR	31.0	2.7	1 - 7	0.0	0.0 - 0.0	0.0	0.0 - 0.1	0.0	0.0 - 0.1	0.3	0.1 - 1.1	0.0	0.0 - 0.0	2.3	0.6 - 6.1	0.96
3050201	6201	QUINBY CR	100.9	13.9	4 - 28	0.0	0.0 - 0.0	1.3	0.4 - 2.6	0.1	0.0 - 0.3	1.5	0.4 - 2.9	0.0	0.0 - 0.0	11.0	3.1 - 25.0	0.91
3050201	6202	COOPER R, E BR	66.7	16.8	5 - 41	0.0	0.0 - 0.0	1.4	0.4 - 3.3	0.2	0.1 - 0.6	2.6	1.0 - 6.2	0.0	0.0 - 0.0	12.6	3.5 - 30.1	0.91
3050201	6203	TURKEY CR	55.9	14.7	5 - 41	0.0	0.0 - 0.0	0.9	0.3 - 2.3	0.1	0.0 - 0.3	0.7	0.2 - 2.0	0.0	0.0 - 0.0	13.0	4.6 - 36.6	0.87
3050201	6204	COOPER R, E BR	109.0	10.7	3 - 24	0.0	0.0 - 0.0	0.4	0.1 - 0.9	0.0	0.0 - 0.1	0.4	0.1 - 1.2	0.0	0.0 - 0.0	9.9	3.0 - 21.8	0.87
3050201	6205	COOPER RIVER W	20.6	0.8	0 - 4	0.0	0.0 - 0.0	0.0	0.0 - 0.3	0.0	0.0 - 0.0	0.0	0.0 - 0.3	0.0	0.0 - 0.0	0.7	0.1 - 3.1	0.97
3050201	6206	COOPER RIVER W	87.9	39.0	15 - 73	23.2	8.9 - 50.7	3.7	1.2 - 7.5	0.2	0.1 - 0.7	4.3	1.5 - 9.7	0.0	0.0 - 0.0	7.6	2.9 - 17.8	0.96
3050201	6207	WADBOO SWAMP	127.5	21.5	7 - 40	0.0	0.0 - 0.0	2.4	0.7 - 4.4	0.2	0.1 - 0.6	4.3	1.4 - 9.4	0.0	0.0 - 0.0	14.6	4.7 - 34.4	0.95
3050201	6208	*B	58.8	11.8	4 - 22	0.0	0.0 - 0.0	0.7	0.2 - 1.4	0.1	0.0 - 0.3	2.4	1.0 - 4.8	0.0	0.0 - 0.0	8.5	3.0 - 18.6	0.88
3050201	6209	WADBOO SWAMP	173.5	25.3	9 - 61	0.0	0.0 - 0.1	3.9	1.1 - 9.6	0.6	0.2 - 1.7	11.7	3.9 - 29.5	0.0	0.0 - 0.0	9.1	2.9 - 25.9	0.88
3050201	6210	COOPER RIVER W	37.3	51.2	17 - 107	0.0	0.0 - 0.0	15.2	4.5 - 29.8	0.9	0.2 - 2.5	17.7	5.5 - 45.6	0.0	0.0 - 0.0	17.4	5.4 - 47.6	0.95
3050201	6211	COOPER R, MOLLY	47.5	34.5	12 - 82	4.8	1.4 - 11.6	5.6	1.6 - 14.0	0.5	0.1 - 1.8	9.0	2.7 - 23.7	0.0	0.0 - 0.0	14.6	5.1 - 42.1	0.96
3050201	6212	BACK R	7.2	13.6	4 - 30	0.0	0.0 - 0.0	6.3	1.7 - 13.8	0.1	0.0 - 0.2	0.6	0.2 - 1.6	0.0	0.0 - 0.0	6.6	2.0 - 14.9	0.99
3050201	6213	BACK R	140.5	20.0	7 - 39	0.1	0.0 - 0.2	4.9	1.4 - 10.6	0.1	0.0 - 0.3	2.0	0.6 - 4.7	0.0	0.0 - 0.0	12.9	4.4 - 28.3	0.96
3050201	6214	FOSTER CR	39.5	53.4	17 - 111	0.0	0.0 - 0.0	22.8	7.7 - 51.1	0.2	0.1 - 0.5	2.6	0.9 - 6.9	0.0	0.0 - 0.0	27.8	8.9 - 69.0	0.96
3050201	6215	GOOSE CR	135.6	23.1	7 - 53	0.0	0.0 - 0.0	12.4	3.1 - 32.0	0.1	0.0 - 0.3	1.4	0.4 - 5.1	0.0	0.0 - 0.0	9.2	2.7 - 22.9	1.00
3050202	6218	*A	104.7	138.3	41 - 269	75.5	24.8 - 148.9	39.6	11.9 - 76.7	0.3	0.1 - 0.8	2.1	0.7 - 4.8	0.0	0.0 - 0.0	20.8	5.8 - 43.6	0.93
3050202	6219	STONO R	102.0	2.1	0 - 7	0.0	0.0 - 0.0	0.2	0.0 - 1.0	0.0	0.0 - 0.1	0.4	0.1 - 1.6	0.0	0.0 - 0.0	1.4	0.3 - 5.0	1.00
3050202	6220	STONO R	42.4	2.1	0 - 6	0.0	0.0 - 0.0	0.2	0.0 - 0.7	0.0	0.0 - 0.1	0.4	0.1 - 1.4	0.0	0.0 - 0.0	1.5	0.3 - 5.1	0.96
3050202	6221	RANTOWLES CR	93.7	14.1	5 - 34	0.0	0.0 - 0.0	3.5	1.0 - 7.5	0.1	0.0 - 0.4	1.2	0.4 - 3.5	0.0	0.0 - 0.0	9.3	3.9 - 22.6	0.93
3050202	6222	*C	47.8	37.4	12 - 85	0.0	0.0 - 0.0	0.5	0.1 - 0.9	0.4	0.1 - 0.9	1.1	0.3 - 2.2	0.0	0.0 - 0.0	35.4	11.2 - 81.9	0.88
3050202	6223	RANTOWLES CR	131.9	48.2	18 - 113	0.0	0.0 - 0.0	1.1	0.4 - 2.4	1.0	0.3 - 3.0	3.6	1.3 - 8.6	0.0	0.0 - 0.0	42.5	15.3 - 100.1	0.88
3050202	6224	WALLACE R	104.2	32.6	11 - 68	0.0	0.0 - 0.0	2.6	0.8 - 5.7	0.4	0.1 - 1.1	5.6	1.5 - 17.3	0.0	0.0 - 0.0	23.9	7.5 - 55.4	0.93
3050202	6225	KIAWAH R	79.1	0.6	0 - 3	0.0	0.0 - 0.0	0.0	0.0 - 0.2	0.0	0.0 - 0.1	0.2	0.0 - 0.9	0.0	0.0 - 0.0	0.4	0.0 - 1.6	1.00
3050202	6226	ASHLEY R	652.8	38.4	12 - 71	0.0	0.0 - 0.0	5.4	1.6 - 10.6	1.4	0.4 - 3.7	10.2	3.2 - 21.2	0.0	0.0 - 0.0	21.4	6.4 - 45.0	0.93

8-digit HUC	ID	Name	Area	Catchment Yield		Point sources		Developed Land		Manure		Agricultural Land		Phosphate Mines		Soil parent rock		Frac
				kg/km^2	90% CI	kg/km^2	90% CI	kg/km^2	90% CI	kg/km^2	90% CI	kg/km^2	90% CI	kg/km^2	90% CI	kg/km^2	90% CI	
3050202	6227	INTRACOASTAL W	344.9	2.0	0 - 5	0.0	0.0 - 0.0	0.1	0.0 - 0.4	0.0	0.0 - 0.1	0.2	0.0 - 0.8	0.0	0.0 - 0.0	1.7	0.3 - 4.4	1.00
3050203	6228	EDISTO R, N FK	31.0	29.0	9 - 68	0.0	0.0 - 0.0	1.9	0.5 - 5.0	2.2	0.6 - 8.5	9.8	3.0 - 22.5	0.0	0.0 - 0.0	15.2	4.8 - 43.0	0.88
3050203	6229	EDISTO R, N FK	16.6	31.1	11 - 68	0.0	0.0 - 0.0	2.7	0.9 - 5.2	2.3	0.7 - 6.1	11.8	3.7 - 29.3	0.0	0.0 - 0.0	14.3	5.3 - 36.6	0.86
3050203	6230	EDISTO R, N FK	64.9	136.7	50 - 299	98.1	36.5 - 212.3	24.1	9.2 - 50.6	1.3	0.4 - 3.6	6.4	2.3 - 17.7	0.0	0.0 - 0.0	6.8	2.8 - 16.4	0.85
3050203	6231	CAW CAW SWAMI	207.9	19.6	6 - 46	0.0	0.0 - 0.0	8.2	2.1 - 18.3	0.5	0.1 - 1.6	5.6	1.5 - 15.5	0.0	0.0 - 0.0	5.4	1.8 - 13.6	0.83
3050203	6232	EDISTO R, N FK	50.5	27.1	9 - 58	0.0	0.0 - 0.0	13.8	4.2 - 31.5	1.3	0.4 - 3.4	5.6	1.7 - 13.8	0.0	0.0 - 0.0	6.4	2.3 - 16.9	0.83
3050203	6234	LIMESTONE CR	56.2	14.9	5 - 24	0.0	0.0 - 0.0	4.1	1.3 - 7.4	0.9	0.2 - 2.1	4.8	1.8 - 11.4	0.0	0.0 - 0.0	5.3	1.7 - 10.8	0.82
3050203	6236	BULL SWAMP CR	255.8	19.0	6 - 33	0.0	0.0 - 0.0	6.2	1.9 - 11.9	2.6	0.7 - 6.8	6.0	2.0 - 14.5	0.0	0.0 - 0.0	4.2	1.2 - 8.4	0.80
3050203	6238	EDISTO R, N FK	31.6	29.2	10 - 61	0.0	0.0 - 0.0	13.0	3.9 - 30.0	1.5	0.4 - 5.1	8.0	3.0 - 21.4	0.0	0.0 - 0.0	6.7	2.6 - 16.1	0.80
3050203	6239	EDISTO R, N FK	7.2	68.9	21 - 175	25.9	7.3 - 60.9	22.5	7.1 - 52.9	1.9	0.7 - 4.9	10.5	2.9 - 27.8	0.0	0.0 - 0.0	8.0	2.5 - 21.4	0.79
3050203	6240	POND BR	18.3	26.4	9 - 64	0.0	0.0 - 0.0	8.6	2.8 - 19.3	1.9	0.5 - 4.8	10.5	3.5 - 35.0	0.0	0.0 - 0.0	5.4	1.7 - 12.0	0.78
3050203	6241	EDISTO R, N FK	195.1	19.1	6 - 42	0.0	0.0 - 0.0	4.5	1.5 - 9.6	3.0	0.9 - 9.0	6.8	2.1 - 16.2	0.0	0.0 - 0.0	4.8	1.8 - 12.8	0.78
3050203	6242	CEDAR CR	113.6	24.3	9 - 51	0.0	0.0 - 0.0	7.2	2.4 - 15.1	4.4	1.3 - 13.9	7.5	2.5 - 17.9	0.0	0.0 - 0.0	5.1	1.6 - 11.2	0.74
3050203	6243	EDISTO R, N FK	7.9	21.4	7 - 38	0.0	0.0 - 0.0	6.8	2.1 - 13.1	2.8	0.7 - 8.2	5.1	1.5 - 12.6	0.0	0.0 - 0.0	6.8	2.0 - 14.2	0.74
3050203	6244	BLACK CR	177.6	21.9	7 - 58	0.0	0.0 - 0.0	5.6	1.8 - 12.4	3.4	1.0 - 11.0	5.8	2.0 - 17.1	0.0	0.0 - 0.0	7.1	2.1 - 21.6	0.73
3050203	6245	EDISTO R, N FK	238.6	16.5	5 - 34	0.0	0.0 - 0.0	4.7	1.4 - 10.7	1.8	0.5 - 4.5	3.6	1.1 - 9.0	0.0	0.0 - 0.0	6.4	2.2 - 14.2	0.73
3050203	6246	LIGHTWOOD KNO	96.5	24.0	8 - 47	0.0	0.0 - 0.0	8.2	2.7 - 18.3	3.0	0.9 - 8.8	5.1	1.4 - 13.5	0.0	0.0 - 0.0	7.7	2.6 - 18.5	0.64
3050203	6247	EDISTO R, N FK	111.6	35.8	13 - 69	11.5	4.1 - 23.3	8.4	2.9 - 17.2	2.8	0.9 - 7.2	5.6	2.0 - 13.9	0.0	0.0 - 0.0	7.5	2.6 - 17.7	0.64
3050203	6248	BIG BEAVER CR	51.9	16.0	6 - 36	0.0	0.0 - 0.0	4.8	1.6 - 10.3	1.1	0.4 - 3.3	6.0	2.1 - 15.5	0.0	0.0 - 0.0	4.1	1.5 - 9.9	0.79
3050203	6249	TURKEY BR	36.5	14.5	4 - 35	0.0	0.0 - 0.0	4.1	1.3 - 10.4	0.9	0.3 - 2.7	4.8	1.4 - 13.2	0.0	0.0 - 0.0	4.7	1.6 - 12.7	0.80
3050203	6250	GREAT BR	31.7	15.8	5 - 38	0.0	0.0 - 0.0	4.6	1.4 - 10.3	1.2	0.3 - 3.8	5.2	1.4 - 13.7	0.0	0.0 - 0.0	4.8	1.3 - 12.8	0.82
3050203	6251	WHIRLWIND 24	36.8	26.1	10 - 66	0.5	0.2 - 1.3	5.4	1.8 - 13.4	2.4	0.8 - 7.7	12.2	4.3 - 35.1	0.0	0.0 - 0.0	5.6	1.8 - 14.8	0.85
3050203	6252	COOPER SWAMP	62.1	27.3	11 - 68	0.0	0.0 - 0.0	5.0	1.8 - 11.3	2.7	1.0 - 7.6	14.5	5.5 - 43.3	0.0	0.0 - 0.0	5.1	1.9 - 13.3	0.86
3050204	6253	EDISTO R, S FK	68.5	25.7	10 - 50	0.0	0.0 - 0.0	3.2	1.3 - 7.1	1.8	0.6 - 4.6	8.3	3.4 - 21.0	0.0	0.0 - 0.0	12.4	4.6 - 29.4	0.88
3050204	6254	SCRATCHNOSE SV	24.0	24.7	8 - 45	0.0	0.0 - 0.0	4.0	1.3 - 7.7	2.3	0.7 - 6.1	12.5	4.3 - 31.0	0.0	0.0 - 0.0	5.9	2.0 - 12.0	0.84
3050204	6255	EDISTO R, S FK	1.2	14.9	5 - 28	0.0	0.0 - 0.0	2.1	0.7 - 4.3	0.4	0.1 - 1.1	1.0	0.3 - 2.5	0.0	0.0 - 0.0	11.3	3.3 - 24.1	0.84
3050204	6256	EDISTO R, S FK	10.6	29.6	11 - 80	0.0	0.0 - 0.0	2.4	0.8 - 6.3	2.6	0.9 - 8.0	15.8	5.4 - 41.1	0.0	0.0 - 0.0	8.8	3.1 - 22.3	0.84
3050204	6257	EDISTO R, S FK	20.7	42.5	14 - 95	0.0	0.0 - 0.0	4.0	1.2 - 10.3	3.3	1.0 - 9.9	22.8	7.6 - 59.3	0.0	0.0 - 0.0	12.4	4.4 - 33.0	0.83
3050204	6258	WILLOW SWAMP	54.7	24.0	8 - 45	0.9	0.3 - 1.9	7.5	2.4 - 18.0	1.7	0.6 - 4.9	9.3	3.1 - 24.8	0.0	0.0 - 0.0	4.6	1.6 - 10.6	0.83
3050204	6259	EDISTO R, S FK	55.5	27.2	8 - 81	0.0	0.0 - 0.0	1.8	0.5 - 5.3	2.2	0.5 - 6.4	14.5	4.0 - 50.3	0.0	0.0 - 0.0	8.7	2.5 - 22.3	0.83
3050204	6260	ROCKY SWAMP CI	75.0	17.4	6 - 40	0.0	0.0 - 0.0	2.8	0.9 - 6.5	1.6	0.5 - 5.0	8.5	3.0 - 26.6	0.0	0.0 - 0.0	4.5	1.6 - 10.4	0.81
3050204	6261	EDISTO R, S FK	1.0	20.1	5 - 41	0.0	0.0 - 0.0	0.0	0.0 - 0.0	1.1	0.3 - 3.2	0.0	0.0 - 0.0	0.0	0.0 - 0.0	19.0	5.3 - 40.5	0.81
3050204	6262	EDISTO R, S FK	4.8	29.3	9 - 67	0.0	0.0 - 0.0	1.3	0.4 - 2.7	1.2	0.3 - 3.8	16.3	4.6 - 44.3	0.0	0.0 - 0.0	10.6	3.3 - 29.3	0.81
3050204	6263	GOODLAND CR	12.4	24.6	9 - 44	0.0	0.0 - 0.0	2.7	1.0 - 5.5	2.1	0.7 - 5.5	12.0	4.1 - 25.4	0.0	0.0 - 0.0	7.8	3.0 - 15.1	0.81
3050204	6264	TAMPA CR	42.5	19.9	6 - 47	0.0	0.0 - 0.0	4.5	1.6 - 10.8	1.8	0.5 - 5.7	9.6	2.9 - 28.0	0.0	0.0 - 0.0	4.1	1.5 - 10.8	0.73
3050204	6265	GOODLAND CR	58.5	16.3	5 - 29	1.1	0.4 - 2.1	4.4	1.4 - 8.6	1.2	0.4 - 4.0	5.1	1.5 - 12.9	0.0	0.0 - 0.0	4.5	1.4 - 9.9	0.73
3050204	6266	EDISTO R, S FK	4.3	23.1	7 - 50	0.0	0.0 - 0.0	2.1	0.6 - 5.4	1.1	0.3 - 3.2	12.3	3.4 - 30.2	0.0	0.0 - 0.0	7.6	2.6 - 18.3	0.81
3050204	6267	EDISTO R, S FK	8.1	30.2	9 - 63	0.0	0.0 - 0.0	2.4	0.7 - 5.3	1.6	0.5 - 4.7	10.9	3.1 - 26.9	0.0	0.0 - 0.0	15.3	4.3 - 38.2	0.80
3050204	6268	EDISTO R, S FK	11.7	34.9	9 - 72	0.0	0.0 - 0.0	7.2	1.8 - 14.3	2.7	0.7 - 6.7	14.9	4.0 - 37.8	0.0	0.0 - 0.0	10.2	3.3 - 20.9	0.80

8-digit HUC	ID	Name	Area	Catchment Yield		Point sources		Developed Land		Manure		Agricultural Land		Phosphate Mines		Soil parent rock		Frac
				kg/km^2	90% CI	kg/km^2	90% CI	kg/km^2	90% CI	kg/km^2	90% CI	kg/km^2	90% CI	kg/km^2	90% CI	kg/km^2	90% CI	
3050204	6269	DEAN SWAMP	181.3	16.6	5 - 28	0.4	0.1 - 0.8	4.2	1.2 - 8.3	1.9	0.6 - 5.3	4.9	1.5 - 11.3	0.0	0.0 - 0.0	5.3	1.8 - 12.3	0.79
3050204	6270	EDISTO R, S FK	10.1	21.2	7 - 57	0.0	0.0 - 0.0	0.4	0.1 - 1.0	1.2	0.3 - 4.0	5.5	1.8 - 15.3	0.0	0.0 - 0.0	14.1	4.5 - 38.0	0.79
3050204	6271	EDISTO R, S FK	5.1	16.5	6 - 45	0.0	0.0 - 0.0	0.0	0.0 - 0.0	1.1	0.3 - 4.0	2.4	0.8 - 6.2	0.0	0.0 - 0.0	13.0	4.7 - 41.2	0.78
3050204	6272	EDISTO R, S FK	22.8	13.5	4 - 25	0.0	0.0 - 0.0	2.2	0.6 - 4.5	1.8	0.5 - 4.9	3.1	0.8 - 9.1	0.0	0.0 - 0.0	6.5	1.9 - 15.1	0.77
3050204	6273	HUNTER BRANCH	59.0	15.5	6 - 32	0.0	0.0 - 0.0	2.8	1.1 - 5.8	2.1	0.7 - 6.7	5.3	1.9 - 16.6	0.0	0.0 - 0.0	5.3	1.8 - 13.8	0.77
3050204	6274	EDISTO R, S FK	80.8	16.3	6 - 35	0.0	0.0 - 0.0	2.6	1.0 - 5.5	2.2	0.7 - 5.5	5.0	1.6 - 11.9	0.0	0.0 - 0.0	6.6	2.3 - 15.8	0.77
3050204	6275	EDISTO R, S FK	1.6	39.1	13 - 86	0.0	0.0 - 0.0	0.0	0.0 - 0.0	6.1	1.9 - 20.0	11.9	4.0 - 28.6	0.0	0.0 - 0.0	21.1	7.0 - 53.8	0.74
3050204	6276	CEDAR CR	44.6	17.1	5 - 40	0.0	0.0 - 0.0	3.4	1.0 - 7.3	2.1	0.7 - 6.8	5.1	1.4 - 13.8	0.0	0.0 - 0.0	6.5	1.9 - 16.6	0.73
3050204	6277	EDISTO R, S FK	45.1	15.4	6 - 29	0.0	0.0 - 0.0	3.7	1.2 - 7.1	1.5	0.5 - 3.8	3.3	1.2 - 7.8	0.0	0.0 - 0.0	7.0	2.6 - 15.5	0.73
3050204	6278	ROCKY SPRING CR	72.2	15.8	6 - 29	0.0	0.0 - 0.0	5.0	1.6 - 8.5	1.6	0.5 - 4.0	3.6	1.2 - 7.8	0.0	0.0 - 0.0	5.6	2.0 - 11.7	0.70
3050204	6279	MCTIER CR	65.6	15.2	5 - 38	0.0	0.0 - 0.0	4.7	1.7 - 14.1	1.4	0.5 - 4.4	3.0	0.9 - 8.0	0.0	0.0 - 0.0	6.1	1.9 - 17.4	0.67
3050204	6280	EDISTO R, S FK	148.7	19.3	7 - 47	0.0	0.0 - 0.0	4.9	1.7 - 11.4	2.5	0.8 - 6.3	5.7	1.9 - 15.4	0.0	0.0 - 0.0	6.1	2.5 - 16.6	0.67
3050204	6281	BEECH CR	41.4	23.7	8 - 39	0.0	0.0 - 0.0	6.4	2.2 - 12.0	4.0	1.1 - 9.7	7.1	2.6 - 14.9	0.0	0.0 - 0.0	6.1	2.0 - 12.5	0.58
3050204	6282	EDISTO R, S FK	53.8	35.4	11 - 70	12.0	3.7 - 25.2	8.5	2.7 - 17.4	1.9	0.6 - 5.8	8.7	2.7 - 22.4	0.0	0.0 - 0.0	4.4	1.4 - 10.4	0.58
3050204	6283	SHAW CR	113.1	17.0	6 - 38	0.0	0.0 - 0.0	3.1	1.0 - 7.0	2.3	0.7 - 6.9	5.6	1.9 - 14.4	0.0	0.0 - 0.0	6.1	2.1 - 14.1	0.74
3050204	6284	SHAW CR	203.5	18.5	7 - 50	0.0	0.0 - 0.0	6.9	2.2 - 16.9	1.5	0.5 - 4.6	5.3	2.0 - 16.5	0.0	0.0 - 0.0	4.8	1.6 - 12.6	0.66
3050204	6285	*A	28.6	30.6	10 - 57	0.0	0.0 - 0.0	14.5	4.6 - 27.8	2.4	0.7 - 6.0	5.9	2.0 - 16.3	0.0	0.0 - 0.0	7.7	2.6 - 16.8	0.66
3050204	6286	POND BR	76.3	15.0	6 - 28	0.0	0.0 - 0.0	4.7	1.7 - 8.6	1.8	0.5 - 5.3	4.0	1.4 - 10.9	0.0	0.0 - 0.0	4.5	1.6 - 12.3	0.77
3050204	6287	YARROW BRANCH	47.6	15.0	5 - 29	0.0	0.0 - 0.0	3.7	1.2 - 7.8	0.7	0.2 - 1.9	5.9	2.1 - 13.5	0.0	0.0 - 0.0	4.7	1.7 - 9.9	0.78
3050204	6288	SPUR BRANCH	53.9	20.4	7 - 36	0.0	0.0 - 0.0	3.9	1.3 - 7.0	0.8	0.2 - 1.7	10.5	3.6 - 23.6	0.0	0.0 - 0.0	5.2	2.0 - 10.6	0.80
3050204	6289	WHALEY CR	46.0	23.1	9 - 51	0.0	0.0 - 0.0	3.3	1.1 - 7.7	0.9	0.3 - 2.3	13.7	5.0 - 36.0	0.0	0.0 - 0.0	5.2	1.9 - 11.3	0.80
3050204	6290	WINDY HILL CR	49.7	29.6	9 - 71	6.8	2.1 - 15.5	4.7	1.3 - 12.5	0.9	0.2 - 2.5	12.1	4.1 - 35.1	0.0	0.0 - 0.0	5.1	1.5 - 12.8	0.81
3050204	6291	*B	23.5	19.6	7 - 45	0.0	0.0 - 0.0	4.0	1.2 - 8.2	1.1	0.3 - 3.3	10.4	3.4 - 28.4	0.0	0.0 - 0.0	4.0	1.4 - 9.9	0.83
3050204	6292	HAYS MILL CR	45.3	21.0	8 - 54	0.0	0.0 - 0.0	7.8	2.8 - 18.0	0.9	0.3 - 2.7	8.3	3.0 - 23.2	0.0	0.0 - 0.0	3.9	1.4 - 11.3	0.84
3050204	6293	EDISTO R, S FK	40.1	13.2	5 - 28	0.0	0.0 - 0.0	3.7	1.3 - 8.4	1.1	0.4 - 4.3	2.3	0.8 - 6.1	0.0	0.0 - 0.0	6.1	2.4 - 13.5	0.70
3050205	6294	N EDISTO R	6.6	0.0	0 - 0	0.0	0.0 - 0.0	0.0	0.0 - 0.0	0.0	0.0 - 0.0	0.0	0.0 - 0.0	0.0	0.0 - 0.0	0.0	0.0 - 0.2	1.00
3050205	6295	BOHICKET CR	107.7	1.5	0 - 5	0.2	0.1 - 0.4	0.1	0.0 - 0.3	0.0	0.0 - 0.1	0.5	0.1 - 2.7	0.0	0.0 - 0.0	0.8	0.1 - 3.1	1.00
3050205	6296	N EDISTO R	37.9	0.5	0 - 2	0.0	0.0 - 0.0	0.0	0.0 - 0.0	0.0	0.0 - 0.0	0.1	0.0 - 0.7	0.0	0.0 - 0.0	0.4	0.0 - 1.7	1.00
3050205	6297	WADMALAW R	293.0	2.9	1 - 9	0.0	0.0 - 0.0	0.2	0.0 - 0.5	0.1	0.0 - 0.2	0.9	0.2 - 3.4	0.0	0.0 - 0.0	1.8	0.3 - 5.7	0.99
3050205	6298	N EDISTO R	64.3	0.6	0 - 3	0.0	0.0 - 0.0	0.0	0.0 - 0.0	0.0	0.0 - 0.0	0.1	0.0 - 0.7	0.0	0.0 - 0.0	0.5	0.1 - 3.0	0.99
3050205	6299	EDISTO R	39.1	0.1	0 - 0	0.0	0.0 - 0.0	0.0	0.0 - 0.0	0.0	0.0 - 0.0	0.0	0.0 - 0.0	0.0	0.0 - 0.0	0.0	0.0 - 0.4	0.97
3050205	6300	ADAMS RUN	89.7	23.3	9 - 52	0.0	0.0 - 0.0	1.6	0.6 - 3.8	0.3	0.1 - 0.8	2.5	0.9 - 6.5	0.0	0.0 - 0.0	18.8	7.2 - 43.2	0.96
3050205	6301	EDISTO R	23.4	0.6	0 - 2	0.0	0.0 - 0.0	0.0	0.0 - 0.1	0.0	0.0 - 0.0	0.1	0.0 - 0.4	0.0	0.0 - 0.0	0.4	0.1 - 1.7	0.96
3050205	6302	EDISTO R	88.6	25.1	6 - 49	0.0	0.0 - 0.0	3.7	1.0 - 7.3	1.2	0.2 - 2.8	6.8	1.6 - 15.7	0.0	0.0 - 0.0	13.4	3.4 - 30.4	0.93
3050205	6303	INDIAN FIELD SW	15.3	22.4	7 - 46	0.0	0.0 - 0.0	1.7	0.5 - 3.8	1.4	0.4 - 4.0	6.7	2.0 - 17.6	0.0	0.0 - 0.0	12.6	4.0 - 28.1	0.92
3050205	6304	INDIAN FIELD SW	21.6	20.7	7 - 43	0.0	0.0 - 0.0	1.3	0.5 - 2.7	1.3	0.4 - 3.6	6.1	1.9 - 16.4	0.0	0.0 - 0.0	12.0	4.5 - 32.4	0.90
3050205	6305	INDIAN FIELD SW	192.2	31.2	11 - 63	0.8	0.3 - 1.8	4.7	1.6 - 10.5	2.7	0.8 - 7.6	14.5	5.1 - 33.5	0.0	0.0 - 0.0	8.4	3.1 - 21.2	0.86
3050205	6306	INDIAN FIELD SW	42.7	33.1	12 - 69	0.0	0.0 - 0.0	8.1	2.7 - 16.6	2.6	0.8 - 7.3	13.2	4.5 - 36.9	0.0	0.0 - 0.0	9.1	3.2 - 19.9	0.86
3050205	6307	POLK SWAMP	177.6	39.2	14 - 95	4.4	1.6 - 12.0	4.8	1.7 - 11.6	3.4	1.0 - 12.2	18.0	6.3 - 52.3	0.0	0.0 - 0.0	8.5	2.7 - 27.4	0.90

8-digit HUC	ID	Name	Area	Catchment Yield		Point sources		Developed Land		Manure		Agricultural Land		Phosphate Mines		Soil parent rock		Frac
				kg/km^2	90% CI	kg/km^2	90% CI	kg/km^2	90% CI	kg/km^2	90% CI	kg/km^2	90% CI	kg/km^2	90% CI	kg/km^2	90% CI	
3050205	6308	EDISTO R	185.8	28.8	9 - 62	0.0	0.0 - 0.0	4.5	1.4 - 10.1	0.8	0.2 - 2.6	7.4	2.5 - 19.4	0.0	0.0 - 0.0	16.1	5.1 - 42.5	0.92
3050205	6309	CATTLE CR	121.1	28.8	11 - 63	0.0	0.0 - 0.0	3.5	1.3 - 8.0	2.8	0.9 - 6.0	15.2	5.6 - 39.8	0.0	0.0 - 0.0	7.2	2.5 - 16.3	0.90
3050205	6310	EDISTO R	121.0	25.9	9 - 48	0.0	0.0 - 0.0	3.1	0.9 - 5.8	1.6	0.6 - 4.0	10.4	3.6 - 25.9	0.0	0.0 - 0.0	10.8	3.8 - 24.0	0.90
3050205	6311	EDISTO R	3.2	30.1	9 - 68	0.0	0.0 - 0.0	1.3	0.4 - 2.7	1.4	0.4 - 4.1	5.5	1.6 - 13.1	0.0	0.0 - 0.0	21.8	6.1 - 57.6	0.88
3050205	6312	EDISTO R, PEN BR	61.1	22.7	9 - 50	0.0	0.0 - 0.0	4.7	1.6 - 10.5	1.9	0.6 - 5.4	10.3	3.5 - 25.1	0.0	0.0 - 0.0	5.8	1.9 - 15.5	0.88
3050205	6313	EDISTO R	13.4	46.0	16 - 92	11.3	3.8 - 22.7	8.1	2.7 - 18.9	2.5	0.7 - 6.7	11.0	3.5 - 25.6	0.0	0.0 - 0.0	13.1	4.5 - 32.6	0.88
3050205	6314	EDISTO R, BETTY	71.0	25.5	8 - 45	0.0	0.0 - 0.0	2.9	0.8 - 5.6	2.4	0.6 - 7.7	12.0	4.2 - 25.0	0.0	0.0 - 0.0	8.3	2.6 - 17.4	0.88
3050205	6315	EDISTO R	13.6	37.6	12 - 99	0.0	0.0 - 0.0	5.1	1.6 - 13.2	2.4	0.8 - 8.8	13.6	4.7 - 37.1	0.0	0.0 - 0.0	16.5	5.5 - 50.4	0.88
3050205	6316	*A	28.4	16.1	5 - 43	0.0	0.0 - 0.0	2.3	0.8 - 6.2	0.9	0.3 - 3.5	5.9	2.1 - 15.2	0.0	0.0 - 0.0	7.0	2.2 - 19.4	0.88
3050205	6317	S EDISTO R	196.9	0.5	0 - 2	0.0	0.0 - 0.0	0.0	0.0 - 0.0	0.0	0.0 - 0.0	0.1	0.0 - 0.3	0.0	0.0 - 0.0	0.5	0.1 - 2.2	1.00
3050206	6318	FOURHOLE SWAM	954.3	34.1	11 - 68	0.0	0.0 - 0.0	4.3	1.3 - 10.1	2.9	0.8 - 10.0	16.5	5.1 - 40.2	0.0	0.0 - 0.0	10.4	3.6 - 27.0	0.93
3050206	6319	FOURHOLE SWAM	257.1	48.5	15 - 109	0.0	0.0 - 0.0	7.8	2.5 - 16.1	3.9	1.1 - 14.5	27.9	8.9 - 76.8	0.0	0.0 - 0.0	8.9	3.0 - 25.6	0.82
3050206	6320	FOURHOLE SWAM	122.7	27.5	8 - 57	0.0	0.0 - 0.0	4.7	1.3 - 11.4	1.0	0.3 - 3.3	16.2	5.1 - 36.1	0.0	0.0 - 0.0	5.6	1.7 - 13.0	0.76
3050206	6321	*A	7.5	47.3	19 - 123	0.0	0.0 - 0.0	2.3	0.8 - 6.3	5.4	1.8 - 19.2	29.9	11.5 - 89.6	0.0	0.0 - 0.0	9.7	3.6 - 25.9	0.76
3050206	6322	BULL SWAMP	49.2	28.3	9 - 57	0.0	0.0 - 0.0	4.8	1.5 - 9.7	2.2	0.7 - 6.7	14.0	5.0 - 32.4	0.0	0.0 - 0.0	7.2	2.6 - 18.4	0.75
3050206	6323	*A	57.8	44.7	15 - 91	1.0	0.4 - 2.2	18.9	6.3 - 44.4	2.5	0.8 - 7.8	14.5	5.2 - 32.0	0.0	0.0 - 0.0	7.6	2.6 - 19.0	0.75
3050206	6324	COW CASTLE CR	82.4	40.4	14 - 82	2.0	0.7 - 4.8	6.4	2.1 - 14.6	3.9	1.3 - 11.7	22.2	6.9 - 57.6	0.0	0.0 - 0.0	5.8	1.8 - 14.5	0.82
3050206	6325	COW CASTLE CR	7.1	37.2	12 - 76	0.0	0.0 - 0.0	5.7	1.5 - 11.7	3.8	1.1 - 9.6	22.1	6.8 - 47.8	0.0	0.0 - 0.0	5.6	1.6 - 13.0	0.75
3050206	6326	COW CASTLE CR, I	68.3	31.9	12 - 76	0.0	0.0 - 0.0	2.8	0.8 - 6.9	3.6	1.0 - 11.8	19.7	7.2 - 49.3	0.0	0.0 - 0.0	5.9	2.1 - 15.9	0.75
3050207	6328	LITTLE SALKEHAT	2.3	7.1	3 - 16	0.0	0.0 - 0.0	0.3	0.1 - 0.7	0.2	0.1 - 0.5	0.0	0.0 - 0.0	0.0	0.0 - 0.0	6.6	2.5 - 16.4	0.91
3050207	6329	LITTLE SALKEHAT	17.0	16.7	6 - 37	0.0	0.0 - 0.0	1.6	0.5 - 3.7	0.3	0.1 - 0.9	3.6	1.3 - 9.3	0.0	0.0 - 0.0	11.1	3.9 - 25.5	0.91
3050207	6330	INDIAN CR	97.5	31.8	11 - 74	0.0	0.0 - 0.0	3.2	1.1 - 9.3	0.7	0.2 - 2.5	9.6	3.3 - 25.9	0.0	0.0 - 0.0	18.2	6.9 - 48.2	0.89
3050207	6331	LITTLE SALKEHAT	48.1	30.3	10 - 70	0.0	0.0 - 0.0	2.8	0.8 - 6.4	0.9	0.3 - 2.4	11.1	3.4 - 28.6	0.0	0.0 - 0.0	15.5	5.1 - 39.1	0.89
3050207	6332	LITTLE SALKEHAT	25.0	29.7	9 - 64	0.0	0.0 - 0.0	4.0	1.1 - 8.3	0.7	0.2 - 2.0	8.5	2.4 - 22.9	0.0	0.0 - 0.0	16.6	5.0 - 39.0	0.85
3050207	6333	BUCKHEAD CR	13.0	35.3	12 - 100	0.0	0.0 - 0.0	3.9	1.2 - 9.9	0.8	0.2 - 2.2	11.5	3.8 - 34.7	0.0	0.0 - 0.0	19.1	6.7 - 55.2	0.84
3050207	6334	BUCKHEAD CR	42.0	31.7	11 - 69	0.4	0.1 - 0.8	3.2	1.1 - 6.8	0.7	0.2 - 2.4	4.7	1.6 - 13.2	0.0	0.0 - 0.0	22.7	7.7 - 57.4	0.79
3050207	6335	BUCKHEAD CR, BI	65.2	32.0	10 - 64	0.0	0.0 - 0.0	3.3	1.1 - 6.1	0.7	0.2 - 1.8	6.8	2.1 - 15.8	0.0	0.0 - 0.0	21.3	7.5 - 49.7	0.71
3050207	6336	BUCKHEAD CR	90.1	26.2	10 - 52	0.0	0.0 - 0.0	3.3	1.3 - 6.6	0.7	0.2 - 1.6	7.2	2.8 - 17.3	0.0	0.0 - 0.0	15.0	5.6 - 33.9	0.71
3050207	6337	BUCKHEAD CR, HO	28.8	33.9	12 - 72	0.0	0.0 - 0.0	4.2	1.3 - 9.1	0.8	0.2 - 2.1	9.8	3.2 - 26.5	0.0	0.0 - 0.0	19.1	6.5 - 50.0	0.79
3050207	6338	LITTLE SALKEHAT	22.6	24.0	6 - 58	0.0	0.0 - 0.0	2.0	0.5 - 4.9	0.5	0.1 - 1.4	6.5	1.8 - 18.4	0.0	0.0 - 0.0	14.9	4.1 - 41.9	0.84
3050207	6339	*A	34.7	34.4	11 - 75	0.0	0.0 - 0.0	3.2	1.0 - 6.8	1.1	0.3 - 3.3	13.0	4.6 - 29.8	0.0	0.0 - 0.0	17.0	5.2 - 49.8	0.81
3050207	6340	LITTLE SALKEHAT	97.8	36.8	12 - 76	0.0	0.0 - 0.0	3.6	1.2 - 7.1	1.7	0.5 - 5.1	16.8	5.2 - 41.2	0.0	0.0 - 0.0	14.7	4.6 - 35.3	0.81
3050207	6341	LEMON CR	206.8	17.3	6 - 29	0.0	0.0 - 0.0	5.5	1.7 - 9.8	0.9	0.3 - 2.2	6.2	2.0 - 12.3	0.0	0.0 - 0.0	4.6	1.6 - 9.6	0.75
3050207	6342	LITTLE SALKEHAT	9.6	57.0	16 - 141	0.0	0.0 - 0.0	4.9	1.3 - 12.8	3.1	0.7 - 11.0	30.7	7.8 - 79.6	0.0	0.0 - 0.0	18.3	5.3 - 47.6	0.75
3050207	6343	LITTLE SALKEHAT	194.6	21.0	6 - 41	2.7	0.9 - 5.5	4.4	1.2 - 9.6	1.0	0.3 - 2.5	8.9	2.9 - 21.1	0.0	0.0 - 0.0	4.1	1.3 - 9.7	0.71
3050207	6344	LITTLE SALKEHAT	70.7	22.7	9 - 46	0.0	0.0 - 0.0	3.2	1.1 - 6.2	1.3	0.4 - 3.4	8.4	3.5 - 22.0	0.0	0.0 - 0.0	9.8	3.5 - 26.1	0.71
3050207	6345	WILLOW SWAMP	138.7	35.5	11 - 94	0.0	0.0 - 0.0	2.8	0.8 - 6.1	1.1	0.3 - 3.2	14.5	4.2 - 40.4	0.0	0.0 - 0.0	17.2	6.2 - 43.7	0.85
3050207	6346	RICEPATH CR	69.3	21.8	8 - 43	0.0	0.0 - 0.0	2.2	0.6 - 4.4	0.4	0.1 - 1.2	5.2	1.4 - 12.9	0.0	0.0 - 0.0	14.0	4.8 - 31.0	0.91
3050207	6347	SALKEHATCHIE R	44.2	17.7	6 - 33	0.0	0.0 - 0.0	1.5	0.5 - 3.0	0.2	0.1 - 0.5	4.4	1.4 - 11.0	0.0	0.0 - 0.0	11.7	4.3 - 26.0	0.91

8-digit HUC	ID	Name	Area	Catchment Yield		Point sources		Developed Land		Manure		Agricultural Land		Phosphate Mines		Soil parent rock		Frac
				kg/km^2	90% CI	kg/km^2	90% CI	kg/km^2	90% CI	kg/km^2	90% CI	kg/km^2	90% CI	kg/km^2	90% CI	kg/km^2	90% CI	
3050207	6348	SALKEHATCHIE R	3.4	18.6	7 - 44	0.0	0.0 - 0.0	2.2	0.7 - 5.2	0.4	0.1 - 1.2	6.0	2.0 - 16.2	0.0	0.0 - 0.0	10.0	3.5 - 27.2	0.87
3050207	6349	SAVANNAH CR	49.1	41.1	16 - 80	0.8	0.3 - 1.8	5.4	1.9 - 10.4	1.9	0.6 - 4.4	17.3	6.4 - 43.1	0.0	0.0 - 0.0	15.7	6.1 - 36.2	0.83
3050207	6350	SALKEHATCHIE R	6.3	24.2	9 - 47	0.0	0.0 - 0.0	1.8	0.6 - 3.4	1.0	0.3 - 2.4	10.8	4.0 - 25.9	0.0	0.0 - 0.0	10.7	3.9 - 24.8	0.83
3050207	6351	SALKEHATCHIE R	49.9	28.2	11 - 64	0.0	0.0 - 0.0	3.1	1.2 - 7.2	1.5	0.5 - 4.1	10.6	3.8 - 23.1	0.0	0.0 - 0.0	13.0	4.6 - 35.8	0.83
3050207	6352	SALKEHATCHIE R	87.3	26.1	8 - 44	0.0	0.0 - 0.0	3.2	1.0 - 7.6	1.2	0.3 - 3.3	11.2	3.3 - 22.6	0.0	0.0 - 0.0	10.5	3.2 - 21.0	0.83
3050207	6353	SALKEHATCHIE R	17.9	19.9	7 - 46	0.0	0.0 - 0.0	2.1	0.8 - 4.4	0.9	0.3 - 2.9	11.5	3.8 - 32.5	0.0	0.0 - 0.0	5.5	2.0 - 14.6	0.79
3050207	6354	SALKEHATCHIE R	34.4	20.5	7 - 50	0.0	0.0 - 0.0	6.9	1.8 - 18.4	0.9	0.2 - 2.3	7.5	2.1 - 21.9	0.0	0.0 - 0.0	5.3	1.7 - 13.6	0.78
3050207	6355	SALKEHATCHIE R	8.1	13.7	4 - 26	0.0	0.0 - 0.0	0.4	0.1 - 0.8	0.7	0.2 - 2.0	6.1	2.0 - 14.8	0.0	0.0 - 0.0	6.5	2.0 - 13.7	0.78
3050207	6356	GEORGES CR	50.0	12.8	4 - 35	0.0	0.0 - 0.0	3.4	1.1 - 8.6	0.5	0.2 - 1.9	5.3	1.7 - 15.7	0.0	0.0 - 0.0	3.5	1.0 - 10.1	0.77
3050207	6357	SALKEHATCHIE R	1.7	11.4	4 - 30	0.0	0.0 - 0.0	0.0	0.0 - 0.0	0.3	0.1 - 0.9	4.0	1.2 - 10.8	0.0	0.0 - 0.0	7.2	2.2 - 17.9	0.77
3050207	6358	HERCULES CR	54.0	15.7	5 - 29	0.0	0.0 - 0.0	4.1	1.5 - 7.9	0.6	0.2 - 1.8	7.8	2.5 - 19.9	0.0	0.0 - 0.0	3.1	0.9 - 6.1	0.76
3050207	6359	SALKEHATCHIE R	36.0	15.3	5 - 33	0.0	0.0 - 0.0	2.4	0.7 - 5.3	0.5	0.2 - 1.8	6.0	2.0 - 14.6	0.0	0.0 - 0.0	6.4	2.6 - 14.7	0.76
3050207	6360	TOBY CR	94.1	16.5	5 - 30	0.0	0.0 - 0.0	3.7	1.2 - 7.2	0.6	0.2 - 1.4	7.8	2.5 - 16.1	0.0	0.0 - 0.0	4.4	1.7 - 10.5	0.73
3050207	6362	SALKEHATCHIE R	22.3	80.2	26 - 201	59.1	18.9 - 154.5	4.0	1.4 - 9.9	0.7	0.2 - 1.9	9.7	3.1 - 28.8	0.0	0.0 - 0.0	6.8	2.2 - 17.1	0.73
3050207	6363	TURKEY CR	79.6	21.8	7 - 41	0.0	0.0 - 0.0	9.3	3.1 - 17.9	0.6	0.2 - 1.4	7.1	2.3 - 15.2	0.0	0.0 - 0.0	4.9	1.7 - 10.1	0.70
3050207	6364	SALKEHATCHIE R	60.0	15.9	6 - 37	0.0	0.0 - 0.0	4.7	1.6 - 11.8	0.5	0.2 - 1.6	5.5	2.2 - 14.2	0.0	0.0 - 0.0	5.2	1.8 - 12.6	0.70
3050207	6365	BUCK CR	45.8	16.7	7 - 44	0.0	0.0 - 0.0	3.6	1.6 - 9.5	0.6	0.2 - 1.9	8.0	3.1 - 22.2	0.0	0.0 - 0.0	4.5	1.8 - 11.8	0.60
3050207	6366	ROSEMARY CR	79.3	12.9	4 - 28	0.0	0.0 - 0.0	4.6	1.3 - 9.9	0.3	0.1 - 1.0	3.6	1.1 - 9.8	0.0	0.0 - 0.0	4.4	1.2 - 11.5	0.60
3050207	6367	HURRICANE CR	28.2	16.1	5 - 29	0.0	0.0 - 0.0	3.3	0.9 - 6.7	0.5	0.1 - 1.3	6.0	1.7 - 12.1	0.0	0.0 - 0.0	6.3	1.8 - 14.5	0.73
3050207	6368	WELLS BR	50.5	17.5	5 - 43	0.0	0.0 - 0.0	2.0	0.6 - 4.2	0.7	0.2 - 1.9	7.2	2.1 - 21.2	0.0	0.0 - 0.0	7.7	2.4 - 17.9	0.79
3050207	6369	WHIPPY SWAMP	64.2	27.4	8 - 53	0.0	0.0 - 0.0	3.9	1.1 - 7.6	0.3	0.1 - 0.7	8.3	2.4 - 17.9	0.0	0.0 - 0.0	14.9	4.2 - 31.5	0.87
3050207	6370	CALICO CR	58.8	20.2	7 - 36	0.0	0.0 - 0.0	2.7	0.8 - 5.0	0.5	0.1 - 1.4	7.5	2.1 - 16.7	0.0	0.0 - 0.0	9.6	3.2 - 21.0	0.80
3050207	6371	WHIPPY SWAMP	3.9	41.3	16 - 82	0.0	0.0 - 0.0	3.6	1.3 - 6.8	0.5	0.1 - 1.1	23.2	8.1 - 55.4	0.0	0.0 - 0.0	14.0	4.7 - 29.2	0.80
3050207	6372	WHIPPY SWAMP, J	25.4	30.1	8 - 87	0.0	0.0 - 0.0	3.4	0.9 - 8.9	1.0	0.2 - 3.0	14.9	4.0 - 41.4	0.0	0.0 - 0.0	10.8	2.8 - 31.4	0.78
3050207	6373	MILLER CR	78.9	40.4	16 - 96	0.0	0.0 - 0.0	4.9	1.9 - 10.3	1.9	0.6 - 5.4	22.2	8.6 - 56.0	0.0	0.0 - 0.0	11.5	4.5 - 29.9	0.67
3050207	6374	WHIPPY SWAMP, J	57.2	36.0	13 - 69	0.0	0.0 - 0.0	5.7	1.7 - 10.9	1.6	0.5 - 3.9	18.7	7.0 - 47.5	0.0	0.0 - 0.0	10.1	3.4 - 23.5	0.67
3050207	6375	CAW CAW SWAMI	69.0	33.4	12 - 70	0.0	0.0 - 0.0	6.5	2.5 - 13.3	0.5	0.2 - 1.3	13.4	4.8 - 33.6	0.0	0.0 - 0.0	12.9	4.7 - 33.1	0.78
3050207	6376	SALKEHATCHIE R	65.3	29.1	8 - 55	0.0	0.0 - 0.0	3.3	0.9 - 7.0	0.2	0.1 - 0.6	6.4	1.9 - 14.5	0.0	0.0 - 0.0	19.2	5.4 - 41.8	0.91
3050208	6377	ASHEPOO R	298.4	0.4	0 - 2	0.0	0.0 - 0.0	0.0	0.0 - 0.1	0.0	0.0 - 0.0	0.0	0.0 - 0.2	0.0	0.0 - 0.0	0.4	0.1 - 1.9	1.00
3050208	6378	HORSESHOE CR	406.6	36.2	12 - 92	0.0	0.0 - 0.0	3.7	1.1 - 8.1	0.7	0.2 - 2.2	6.8	2.0 - 18.7	0.0	0.0 - 0.0	25.0	8.0 - 69.7	0.93
3050208	6379	ASHEPOO R	375.5	40.1	12 - 76	4.9	1.7 - 10.9	7.1	1.9 - 14.7	0.7	0.2 - 1.9	8.1	2.4 - 19.6	0.0	0.0 - 0.0	19.4	5.7 - 48.7	0.93
3050208	6380	COMBAHEE R	636.7	4.9	1 - 13	0.3	0.1 - 0.9	0.4	0.1 - 1.1	0.1	0.0 - 0.3	0.7	0.2 - 2.6	0.0	0.0 - 0.0	3.5	1.0 - 9.6	1.00
3050208	6381	BLACK CR	78.5	25.5	10 - 62	0.0	0.0 - 0.0	5.4	2.0 - 11.9	0.5	0.2 - 1.3	5.2	1.8 - 16.9	0.0	0.0 - 0.0	14.5	5.6 - 34.3	0.92
3050208	6382	COMBAHEE R	22.2	29.1	11 - 68	0.0	0.0 - 0.0	2.8	0.9 - 6.5	0.4	0.1 - 1.0	11.2	3.9 - 29.8	0.0	0.0 - 0.0	14.7	5.1 - 41.8	0.92
3050208	6383	SANDY RUN	32.0	19.8	7 - 36	0.0	0.0 - 0.0	3.9	1.2 - 6.5	0.3	0.1 - 0.6	2.3	0.7 - 4.7	0.0	0.0 - 0.0	13.3	4.4 - 27.2	0.91
3050208	6385	TULFINY R	86.1	20.3	7 - 42	0.0	0.0 - 0.0	2.3	0.7 - 5.0	0.2	0.0 - 0.5	3.2	1.0 - 7.0	0.0	0.0 - 0.0	14.6	4.9 - 37.7	1.00
3050208	6386	COOSAWHATCHIE	153.7	26.0	10 - 54	0.0	0.0 - 0.0	2.2	0.8 - 4.7	0.2	0.1 - 0.5	4.1	1.5 - 10.6	0.0	0.0 - 0.0	19.5	7.2 - 43.5	1.00
3050208	6387	COOSAWHATCHIE	168.0	38.0	14 - 81	0.0	0.0 - 0.0	3.3	1.2 - 7.2	0.4	0.1 - 0.9	10.9	3.6 - 22.9	0.0	0.0 - 0.0	23.5	8.6 - 55.5	0.97
3050208	6388	COOSAWHATCHIE	40.6	27.7	8 - 60	0.0	0.0 - 0.0	3.8	1.1 - 7.8	0.2	0.1 - 0.5	5.2	1.5 - 12.5	0.0	0.0 - 0.0	18.4	5.5 - 46.0	0.91

8-digit HUC	ID	Name	Area	Catchment Yield		Point sources		Developed Land		Manure		Agricultural Land		Phosphate Mines		Soil parent rock		Frac
				kg/km^2	90% CI	kg/km^2	90% CI	kg/km^2	90% CI	kg/km^2	90% CI	kg/km^2	90% CI	kg/km^2	90% CI	kg/km^2	90% CI	
3050208	6389	COOSAWHATCHIE	33.8	36.5	15 - 68	4.5	1.7 - 9.0	5.1	1.9 - 10.4	0.3	0.1 - 0.8	9.8	3.6 - 24.3	0.0	0.0 - 0.0	16.8	6.4 - 36.5	0.88
3050208	6390	COOSAWHATCHIE	16.9	33.5	12 - 84	0.0	0.0 - 0.0	5.7	1.7 - 14.0	1.0	0.3 - 3.2	12.4	4.0 - 38.9	0.0	0.0 - 0.0	14.4	5.1 - 44.5	0.84
3050208	6391	DUCK CR	37.6	49.8	14 - 108	0.0	0.0 - 0.0	9.7	2.8 - 19.0	2.0	0.6 - 5.2	24.4	6.9 - 65.5	0.0	0.0 - 0.0	13.6	4.4 - 30.9	0.81
3050208	6392	COOSAWHATCHIE	108.2	32.6	12 - 73	0.0	0.0 - 0.0	5.5	1.8 - 11.1	1.2	0.4 - 4.0	14.2	4.8 - 36.7	0.0	0.0 - 0.0	11.7	4.2 - 26.4	0.81
3050208	6393	BEECH CR	45.8	20.9	8 - 44	0.0	0.0 - 0.0	2.2	0.8 - 4.2	0.4	0.1 - 1.0	4.3	1.5 - 9.3	0.0	0.0 - 0.0	13.9	5.1 - 32.8	0.84
3050208	6394	BLOOD HILL CR	34.6	22.0	8 - 42	0.0	0.0 - 0.0	3.9	1.2 - 7.5	0.3	0.1 - 0.7	6.3	2.0 - 16.0	0.0	0.0 - 0.0	11.5	4.1 - 24.0	0.88
3050208	6395	BLACK CR	174.1	31.0	9 - 72	0.0	0.0 - 0.0	6.4	1.9 - 14.1	0.3	0.1 - 1.0	11.3	3.6 - 31.5	0.0	0.0 - 0.0	13.1	3.8 - 32.8	0.91
3050208	6396	CYPRESS CR	235.4	30.1	9 - 55	0.0	0.0 - 0.0	3.1	0.9 - 6.1	0.3	0.1 - 1.0	9.1	3.2 - 20.0	0.0	0.0 - 0.0	17.5	4.8 - 34.7	0.97
3050208	6397	*A	109.5	23.3	7 - 41	0.0	0.0 - 0.0	6.1	1.9 - 11.3	0.3	0.1 - 0.8	7.3	2.2 - 18.6	0.0	0.0 - 0.0	9.6	2.6 - 21.9	1.00
3050208	6398	EUHAW CR	145.2	10.3	3 - 25	0.0	0.0 - 0.0	0.7	0.2 - 1.7	0.1	0.0 - 0.3	0.9	0.2 - 2.8	0.0	0.0 - 0.0	8.5	2.5 - 22.3	1.00
3050208	6399	NEW R	691.4	7.5	3 - 18	0.0	0.0 - 0.0	0.7	0.2 - 2.0	0.1	0.0 - 0.2	0.9	0.3 - 2.4	0.0	0.0 - 0.0	5.9	1.9 - 15.2	1.00
3050208	6400	SANDERS BR	60.1	50.4	15 - 92	16.0	5.1 - 32.1	12.2	3.7 - 25.1	0.3	0.1 - 0.8	7.0	2.2 - 18.2	0.0	0.0 - 0.0	15.0	4.3 - 31.9	0.93
3060101	6402	GOLDEN CR	41.3	69.3	25 - 147	20.8	7.8 - 48.6	25.5	9.0 - 64.4	2.7	0.9 - 6.9	11.1	3.5 - 27.8	0.0	0.0 - 0.0	9.2	3.2 - 21.1	0.13
3060101	6404	RICES CR	41.3	54.6	18 - 98	0.0	0.0 - 0.0	24.7	7.6 - 46.7	3.0	0.8 - 7.2	16.5	5.0 - 35.0	0.0	0.0 - 0.0	10.4	3.1 - 19.1	0.12
3060101	6405	TWELVEMILE CR	28.6	54.2	19 - 108	0.0	0.0 - 0.0	10.7	3.7 - 21.4	4.1	1.2 - 11.5	23.4	7.3 - 60.9	0.0	0.0 - 0.0	16.0	4.8 - 38.3	0.12
3060101	6406	WOLF CR	40.8	44.6	16 - 83	0.0	0.0 - 0.0	21.2	6.2 - 43.8	2.4	0.7 - 6.8	9.9	3.2 - 23.4	0.0	0.0 - 0.0	11.1	3.8 - 22.1	0.12
3060101	6407	TWELVEMILE CR	66.5	59.3	23 - 135	8.4	3.1 - 20.0	20.2	7.5 - 43.7	3.0	0.9 - 8.4	15.1	5.1 - 44.5	0.0	0.0 - 0.0	12.6	4.5 - 34.7	0.12
3060101	6408	TWELVEMILE CR,	41.7	32.4	11 - 73	0.0	0.0 - 0.0	10.6	4.2 - 23.1	1.6	0.5 - 4.2	6.1	1.7 - 15.4	0.0	0.0 - 0.0	14.2	4.9 - 32.8	0.12
3060101	6409	TWELVEMILE CR	48.4	28.5	10 - 50	0.0	0.0 - 0.0	7.5	2.4 - 13.5	1.5	0.4 - 4.2	4.7	1.3 - 11.1	0.0	0.0 - 0.0	14.9	5.3 - 32.6	0.12
3060101	6410	LITTLE R	1.7	36.4	13 - 86	0.0	0.0 - 0.0	7.3	2.5 - 18.2	11.7	3.6 - 36.8	6.2	1.9 - 18.9	0.0	0.0 - 0.0	11.2	3.8 - 31.7	0.04
3060101	6411	LITTLE R	54.7	32.2	11 - 76	0.0	0.0 - 0.0	10.9	3.4 - 27.5	4.2	1.4 - 11.9	3.6	1.3 - 10.9	0.0	0.0 - 0.0	13.5	4.6 - 31.6	0.04
3060101	6412	FLAT SHOALS R	88.3	30.4	10 - 77	0.8	0.3 - 1.9	6.0	2.0 - 14.4	4.2	1.2 - 14.2	4.1	1.4 - 11.5	0.0	0.0 - 0.0	15.2	5.4 - 36.1	0.04
3060101	6413	*A	42.6	27.7	8 - 47	0.0	0.0 - 0.0	5.8	1.6 - 11.5	5.2	1.4 - 17.4	4.9	1.5 - 11.8	0.0	0.0 - 0.0	11.8	3.8 - 23.4	0.04
3060102	6414	CHAUGA R	215.0	25.4	11 - 59	0.7	0.3 - 1.6	5.6	2.3 - 13.0	3.1	0.9 - 8.0	3.2	1.2 - 8.7	0.0	0.0 - 0.0	12.9	6.0 - 32.3	0.12
3060102	6415	TAXAWAY CR	56.4	23.2	8 - 53	0.0	0.0 - 0.0	5.0	1.6 - 12.2	3.6	1.1 - 9.6	3.4	1.0 - 10.4	0.0	0.0 - 0.0	11.2	3.6 - 28.8	0.12
3060102	6416	TUGALOO R	3.7	24.1	9 - 57	0.0	0.0 - 0.0	2.6	0.9 - 6.6	4.3	1.2 - 13.7	4.3	1.6 - 11.4	0.0	0.0 - 0.0	13.0	4.7 - 34.4	0.13
3060102	6417	TUGALOO R	63.7	22.4	7 - 61	0.0	0.0 - 0.0	4.9	1.5 - 12.5	4.3	1.2 - 16.3	3.5	0.9 - 9.7	0.0	0.0 - 0.0	9.8	3.2 - 27.5	0.13
3060102	6418	BRASSWATER CR	38.1	23.0	8 - 42	0.0	0.0 - 0.0	3.3	1.0 - 6.7	1.6	0.5 - 4.2	1.7	0.5 - 4.1	0.0	0.0 - 0.0	16.4	5.2 - 34.8	0.12
3060102	6420	TUGALOO R	16.0	17.8	6 - 36	0.0	0.0 - 0.0	3.0	1.0 - 6.7	1.8	0.6 - 5.3	0.7	0.3 - 1.9	0.0	0.0 - 0.0	12.2	4.2 - 26.8	0.12
3060102	6421	TUGALOO R	42.1	25.2	10 - 52	0.0	0.0 - 0.0	4.5	1.6 - 10.1	1.4	0.5 - 3.7	1.1	0.4 - 3.2	0.0	0.0 - 0.0	18.1	7.4 - 41.1	0.12
3060102	6422	CHATOOGA R	30.4	40.6	14 - 89	0.0	0.0 - 0.0	7.5	2.5 - 16.8	7.7	2.3 - 18.7	8.7	2.6 - 23.5	0.0	0.0 - 0.0	16.7	5.5 - 38.3	0.11
3060102	6423	CHATOOGA R	20.1	27.8	9 - 56	0.0	0.0 - 0.0	3.3	1.0 - 7.7	0.2	0.1 - 0.5	0.1	0.0 - 0.4	0.0	0.0 - 0.0	24.2	7.5 - 53.2	0.11
3060102	6424	CHATOOGA R	136.9	39.9	16 - 101	0.3	0.1 - 0.8	5.7	1.8 - 13.4	0.1	0.0 - 0.2	0.4	0.1 - 1.2	0.0	0.0 - 0.0	33.3	13.0 - 85.3	0.11
3060102	6425	FOWLER CR	11.5	99.8	40 - 287	0.0	0.0 - 0.0	61.8	23.1 - 159.0	0.1	0.0 - 0.2	0.2	0.1 - 0.5	0.0	0.0 - 0.0	37.8	13.9 - 128.7	0.10
3060102	6426	CHATOOGA R	23.6	68.6	20 - 161	4.3	1.1 - 11.4	24.1	6.8 - 59.2	0.2	0.1 - 0.7	2.6	0.7 - 7.1	0.0	0.0 - 0.0	37.4	10.2 - 91.5	0.10
3060102	6427	*A	149.7	43.4	15 - 75	0.1	0.0 - 0.2	9.5	2.7 - 17.5	0.7	0.2 - 1.9	1.5	0.5 - 3.7	0.0	0.0 - 0.0	31.7	11.0 - 63.9	0.11
3060102	6428	WARWOMAN CR	100.7	39.7	15 - 84	0.0	0.0 - 0.0	6.8	2.3 - 17.4	1.4	0.4 - 4.5	1.7	0.5 - 4.4	0.0	0.0 - 0.0	29.8	11.2 - 65.9	0.11
3060102	6429	STEKOA CR	125.6	64.6	24 - 142	4.9	1.9 - 10.7	25.8	10.1 - 60.2	3.6	1.1 - 11.9	4.1	1.4 - 10.3	0.0	0.0 - 0.0	26.2	9.8 - 62.8	0.11
3060102	6430	TALLULAH R	29.5	37.6	14 - 93	0.0	0.0 - 0.0	18.6	6.4 - 48.6	3.0	0.9 - 10.4	1.8	0.6 - 5.1	0.0	0.0 - 0.0	14.2	4.7 - 36.1	0.12

8-digit HUC	ID	Name	Area	Catchment Yield		Point sources		Developed Land		Manure		Agricultural Land		Phosphate Mines		Soil parent rock		Frac
				kg/km^2	90% CI	kg/km^2	90% CI	kg/km^2	90% CI	kg/km^2	90% CI	kg/km^2	90% CI	kg/km^2	90% CI	kg/km^2	90% CI	
3060102	6431	TIGER CR	63.8	45.6	17 - 87	0.0	0.0 - 0.0	16.4	5.3 - 31.4	3.5	1.1 - 9.4	3.9	1.3 - 10.0	0.0	0.0 - 0.0	21.7	8.2 - 44.7	0.09
3060102	6432	TALLULAH R	92.6	36.2	12 - 100	0.0	0.0 - 0.0	13.1	4.1 - 33.9	1.4	0.4 - 3.9	1.5	0.5 - 4.7	0.0	0.0 - 0.0	20.2	7.9 - 55.6	0.09
3060102	6433	TALLULAH R	18.0	34.4	11 - 89	0.0	0.0 - 0.0	1.3	0.4 - 3.3	0.2	0.1 - 0.6	0.1	0.0 - 0.4	0.0	0.0 - 0.0	32.7	10.5 - 85.9	0.03
3060102	6434	*B	59.5	31.4	9 - 63	0.0	0.0 - 0.0	2.3	0.8 - 5.3	0.1	0.0 - 0.4	0.3	0.1 - 0.7	0.0	0.0 - 0.0	28.6	8.1 - 57.2	0.03
3060102	6435	PANTHER CR	85.9	39.4	14 - 81	0.0	0.0 - 0.0	10.3	3.3 - 21.4	10.4	2.9 - 31.1	3.3	1.1 - 9.7	0.0	0.0 - 0.0	15.3	5.3 - 32.9	0.12
3060102	6436	*C	78.0	44.6	18 - 139	5.7	2.4 - 18.1	15.3	5.4 - 50.6	6.5	2.5 - 19.8	3.4	1.3 - 11.8	0.0	0.0 - 0.0	13.7	5.6 - 41.1	0.13
3060102	6437	*D	1.6	52.9	16 - 125	0.0	0.0 - 0.0	10.2	3.1 - 23.5	6.8	1.5 - 18.5	5.0	1.6 - 14.0	0.0	0.0 - 0.0	30.9	9.3 - 73.3	0.13
3060102	6438	*E	12.7	48.9	19 - 108	0.0	0.0 - 0.0	25.2	9.1 - 52.3	5.0	1.5 - 16.3	2.6	0.9 - 6.8	0.0	0.0 - 0.0	16.2	6.2 - 42.5	0.12
3060102	6439	*F	21.3	54.6	16 - 149	0.0	0.0 - 0.0	29.0	8.4 - 80.6	7.2	2.0 - 20.3	3.9	1.2 - 10.4	0.0	0.0 - 0.0	14.5	4.4 - 38.6	0.12
3060103	6440	BIG CURLTAIL CR	88.0	31.5	11 - 66	0.0	0.0 - 0.0	12.6	4.3 - 27.8	1.6	0.4 - 3.9	5.5	1.9 - 13.0	0.0	0.0 - 0.0	11.8	4.1 - 28.6	0.40
3060103	6441	LONG CANE CR	14.2	91.3	33 - 211	73.8	26.0 - 175.7	2.6	0.9 - 6.1	0.8	0.2 - 2.4	1.4	0.5 - 2.9	0.0	0.0 - 0.0	12.7	3.8 - 31.4	0.40
3060103	6442	LONG CANE CR	5.3	20.9	6 - 60	0.0	0.0 - 0.0	2.7	1.0 - 7.6	1.3	0.4 - 5.4	4.2	1.4 - 13.4	0.0	0.0 - 0.0	12.7	3.9 - 38.8	0.39
3060103	6443	LONG CANE CR	14.3	21.1	7 - 60	0.0	0.0 - 0.0	5.6	1.7 - 13.4	0.8	0.3 - 2.6	2.2	0.7 - 6.5	0.0	0.0 - 0.0	12.5	4.3 - 40.0	0.39
3060103	6444	JOHNS CR	39.1	26.4	8 - 54	0.0	0.0 - 0.0	5.3	1.6 - 11.2	2.2	0.7 - 5.9	7.3	2.2 - 18.3	0.0	0.0 - 0.0	11.5	4.1 - 25.7	0.37
3060103	6445	LONG CANE CR	153.4	31.1	10 - 56	0.0	0.0 - 0.0	5.4	1.8 - 10.9	3.4	1.1 - 9.6	11.5	3.9 - 25.8	0.0	0.0 - 0.0	10.7	3.4 - 21.8	0.37
3060103	6446	MCCORD CR	26.6	27.3	8 - 73	0.0	0.0 - 0.0	6.7	2.0 - 17.4	2.4	0.7 - 7.8	6.2	1.8 - 15.3	0.0	0.0 - 0.0	12.0	3.7 - 31.4	0.39
3060103	6447	*A	47.2	41.1	14 - 89	0.0	0.0 - 0.0	22.7	7.3 - 51.9	1.8	0.4 - 4.8	4.5	1.5 - 13.2	0.0	0.0 - 0.0	12.2	3.3 - 27.9	0.39
3060103	6448	HILLBERN CR	2.7	37.1	13 - 102	0.0	0.0 - 0.0	5.3	1.8 - 14.1	3.0	0.9 - 10.4	18.7	5.8 - 60.6	0.0	0.0 - 0.0	10.1	3.9 - 30.1	0.41
3060103	6449	HILLBERN CR	64.2	25.2	8 - 51	0.0	0.0 - 0.0	5.7	1.7 - 13.0	1.7	0.5 - 4.4	5.7	1.7 - 14.4	0.0	0.0 - 0.0	12.1	4.2 - 25.9	0.41
3060103	6450	CALHOUN CR	99.9	28.0	9 - 50	0.0	0.0 - 0.0	5.6	1.8 - 11.3	2.5	0.7 - 6.1	8.3	3.0 - 20.0	0.0	0.0 - 0.0	11.6	4.0 - 23.5	0.41
3060103	6451	LITTLE R	3.0	12.4	5 - 25	0.0	0.0 - 0.0	1.7	0.6 - 3.4	0.3	0.1 - 0.9	1.0	0.4 - 2.4	0.0	0.0 - 0.0	9.3	3.9 - 19.9	0.41
3060103	6452	LITTLE R	16.5	17.3	6 - 34	0.0	0.0 - 0.0	2.9	1.0 - 5.9	0.9	0.3 - 2.4	1.5	0.6 - 3.6	0.0	0.0 - 0.0	12.1	4.0 - 25.6	0.41
3060103	6453	LITTLE R	19.1	22.6	7 - 46	0.0	0.0 - 0.0	5.3	1.7 - 10.2	1.7	0.5 - 4.8	3.0	1.0 - 8.0	0.0	0.0 - 0.0	12.5	4.0 - 29.3	0.40
3060103	6454	LITTLE R	14.9	23.7	8 - 54	0.0	0.0 - 0.0	4.4	1.3 - 9.5	1.9	0.5 - 5.0	5.2	1.7 - 12.5	0.0	0.0 - 0.0	12.2	3.6 - 27.6	0.39
3060103	6455	LITTLE R	19.2	19.2	6 - 31	0.0	0.0 - 0.0	3.0	0.9 - 5.6	1.5	0.5 - 3.8	4.5	1.4 - 9.9	0.0	0.0 - 0.0	10.3	3.0 - 19.0	0.38
3060103	6456	PARK CR	54.9	35.4	15 - 73	3.7	1.4 - 7.6	6.4	2.3 - 15.2	2.8	0.9 - 8.2	9.9	3.9 - 27.2	0.0	0.0 - 0.0	12.6	4.9 - 33.3	0.37
3060103	6457	LITTLE R	4.7	22.8	8 - 46	0.0	0.0 - 0.0	5.5	2.0 - 10.6	1.6	0.5 - 4.7	1.8	0.7 - 4.5	0.0	0.0 - 0.0	13.9	4.9 - 33.0	0.37
3060103	6458	LITTLE R	7.3	23.6	7 - 48	0.0	0.0 - 0.0	3.7	1.1 - 8.7	1.9	0.5 - 4.8	2.8	0.9 - 6.7	0.0	0.0 - 0.0	15.2	4.3 - 36.0	0.36
3060103	6459	CHICKASAW CR	26.3	31.9	12 - 82	0.0	0.0 - 0.0	5.9	2.3 - 13.8	3.3	1.0 - 9.5	11.4	4.4 - 35.1	0.0	0.0 - 0.0	11.2	4.4 - 32.6	0.35
3060103	6460	LITTLE R	1.4	30.3	11 - 61	0.0	0.0 - 0.0	9.1	3.2 - 18.7	1.3	0.4 - 4.1	3.9	1.4 - 8.9	0.0	0.0 - 0.0	15.9	5.0 - 37.4	0.35
3060103	6461	HOGSKIN CR	49.5	32.8	10 - 86	0.0	0.0 - 0.0	5.3	1.6 - 12.1	3.7	1.0 - 11.2	12.6	4.0 - 34.2	0.0	0.0 - 0.0	11.2	3.5 - 27.9	0.35
3060103	6462	LITTLE R	82.0	37.9	12 - 69	0.0	0.0 - 0.0	9.3	3.1 - 17.9	4.1	1.1 - 9.7	12.8	4.4 - 30.2	0.0	0.0 - 0.0	11.7	3.8 - 30.0	0.35
3060103	6463	LITTLE R	16.6	45.9	15 - 82	0.0	0.0 - 0.0	13.5	4.0 - 24.4	4.9	1.4 - 13.7	15.6	4.9 - 36.1	0.0	0.0 - 0.0	11.9	4.1 - 24.8	0.28
3060103	6464	BARKERS CR	17.8	48.6	15 - 98	0.0	0.0 - 0.0	7.9	2.6 - 15.4	6.4	2.0 - 17.3	21.5	6.1 - 52.9	0.0	0.0 - 0.0	12.8	4.0 - 29.1	0.28
3060103	6466	JOHNSON CR	39.0	30.5	11 - 71	0.0	0.0 - 0.0	5.5	1.8 - 12.5	3.3	1.0 - 10.6	10.9	4.1 - 27.8	0.0	0.0 - 0.0	10.8	3.8 - 27.6	0.36
3060103	6467	SPUR CR	19.4	30.2	9 - 51	0.0	0.0 - 0.0	5.9	1.9 - 11.5	2.9	0.8 - 7.4	9.9	3.1 - 23.6	0.0	0.0 - 0.0	11.4	3.9 - 24.1	0.36
3060103	6468	PENNY CR	41.9	27.9	10 - 51	0.0	0.0 - 0.0	3.9	1.4 - 7.8	3.1	1.1 - 8.5	9.6	3.4 - 24.9	0.0	0.0 - 0.0	11.2	3.9 - 26.8	0.38
3060103	6469	SHANKLIN CR	38.1	28.9	10 - 62	0.0	0.0 - 0.0	4.2	1.4 - 9.2	3.1	1.0 - 8.8	10.5	4.0 - 25.3	0.0	0.0 - 0.0	11.2	3.8 - 26.6	0.39
3060103	6470	GILL CR	24.0	45.0	17 - 102	0.0	0.0 - 0.0	7.8	2.7 - 17.7	6.0	2.1 - 18.4	20.4	6.8 - 50.6	0.0	0.0 - 0.0	10.8	3.9 - 25.8	0.40

8-digit HUC	ID	Name	Area	Catchment Yield		Point sources		Developed Land		Manure		Agricultural Land		Phosphate Mines		Soil parent rock		Frac
				kg/km^2	90% CI	kg/km^2	90% CI	kg/km^2	90% CI	kg/km^2	90% CI	kg/km^2	90% CI	kg/km^2	90% CI	kg/km^2	90% CI	
3060103	6471	MORROW CR	28.5	23.8	7 - 57	0.0	0.0 - 0.0	3.5	1.0 - 8.7	2.4	0.6 - 6.4	7.5	2.4 - 19.4	0.0	0.0 - 0.0	10.4	2.8 - 26.9	0.37
3060103	6472	GILL CR	42.5	30.6	11 - 54	0.0	0.0 - 0.0	5.3	1.7 - 10.0	3.2	0.9 - 7.6	10.5	3.4 - 22.8	0.0	0.0 - 0.0	11.7	3.8 - 25.7	0.37
3060103	6473	SAWNEY CR	34.8	51.7	16 - 128	17.3	5.4 - 39.1	12.0	3.8 - 31.1	2.3	0.7 - 8.3	9.5	3.5 - 24.3	0.0	0.0 - 0.0	10.6	3.6 - 28.5	0.41
3060103	6474	SAVANNAH R	1.6	20.3	6 - 46	0.0	0.0 - 0.0	0.7	0.2 - 1.4	0.8	0.2 - 2.2	0.8	0.3 - 2.2	0.0	0.0 - 0.0	18.1	5.5 - 42.2	0.41
3060103	6475	ROCKY R	109.0	29.5	9 - 58	0.0	0.0 - 0.0	5.6	1.7 - 12.2	1.8	0.5 - 4.8	4.7	1.6 - 11.6	0.0	0.0 - 0.0	17.4	5.3 - 36.4	0.41
3060103	6476	ROCKY R	2.5	24.1	8 - 53	0.0	0.0 - 0.0	2.5	0.8 - 5.3	1.6	0.5 - 4.0	0.7	0.2 - 1.9	0.0	0.0 - 0.0	19.3	7.1 - 51.8	0.39
3060103	6477	BEAR CR	23.7	34.5	11 - 77	0.0	0.0 - 0.0	7.3	2.1 - 18.0	4.1	1.1 - 10.0	10.8	3.5 - 27.8	0.0	0.0 - 0.0	12.3	4.1 - 28.6	0.15
3060103	6478	HENCOOP CR	39.6	39.8	15 - 82	0.0	0.0 - 0.0	7.5	2.4 - 16.0	5.0	1.6 - 12.8	14.6	4.9 - 31.5	0.0	0.0 - 0.0	12.6	4.2 - 30.7	0.15
3060103	6479	HENCOOP CR	15.7	37.7	13 - 87	0.0	0.0 - 0.0	10.6	3.1 - 27.9	3.6	1.0 - 9.2	10.6	3.2 - 30.5	0.0	0.0 - 0.0	12.9	4.1 - 33.5	0.13
3060103	6480	*B	21.2	42.4	14 - 108	0.0	0.0 - 0.0	14.7	4.9 - 39.3	3.8	1.3 - 10.8	10.8	3.3 - 29.7	0.0	0.0 - 0.0	13.1	4.6 - 35.6	0.13
3060103	6481	ROCKY R	3.2	34.0	9 - 70	0.0	0.0 - 0.0	6.0	1.4 - 13.5	3.1	0.7 - 8.5	9.6	2.8 - 25.9	0.0	0.0 - 0.0	15.3	3.6 - 36.5	0.15
3060103	6482	ROCKY R	1.2	35.0	13 - 75	0.0	0.0 - 0.0	2.7	0.9 - 5.8	3.8	1.1 - 8.7	12.2	4.4 - 30.9	0.0	0.0 - 0.0	16.3	6.1 - 37.2	0.15
3060103	6483	BROADWAY CR	51.4	46.1	17 - 113	0.0	0.0 - 0.0	19.4	6.9 - 48.8	3.1	1.0 - 8.1	8.1	3.0 - 25.9	0.0	0.0 - 0.0	15.4	5.8 - 40.0	0.15
3060103	6484	PEA CR	15.4	57.9	21 - 147	0.0	0.0 - 0.0	22.7	7.8 - 54.7	4.8	1.6 - 17.0	14.6	5.7 - 40.6	0.0	0.0 - 0.0	15.8	6.0 - 46.8	0.14
3060103	6485	BROADWAY CR	52.8	55.6	20 - 126	0.0	0.0 - 0.0	14.2	4.7 - 33.0	6.1	1.8 - 18.9	19.1	6.2 - 57.9	0.0	0.0 - 0.0	16.1	5.3 - 38.2	0.14
3060103	6486	ROCKY R	146.4	112.8	38 - 261	53.6	17.6 - 127.8	26.3	7.1 - 57.0	4.3	1.2 - 11.4	12.8	4.4 - 33.2	0.0	0.0 - 0.0	15.8	5.7 - 40.6	0.15
3060103	6487	BEAVER CR	44.2	47.7	14 - 92	0.0	0.0 - 0.0	16.8	5.4 - 37.0	4.2	1.1 - 11.9	11.5	3.7 - 32.0	0.0	0.0 - 0.0	15.2	4.7 - 35.7	0.15
3060103	6488	WILSON CR	106.1	40.4	16 - 93	0.9	0.4 - 2.1	8.7	3.1 - 19.2	4.1	1.3 - 13.1	12.7	5.0 - 31.3	0.0	0.0 - 0.0	13.9	5.7 - 34.9	0.39
3060103	6489	SAVANNAH R	40.0	22.9	7 - 57	0.0	0.0 - 0.0	1.2	0.4 - 2.7	0.7	0.2 - 1.8	0.3	0.1 - 0.7	0.0	0.0 - 0.0	20.8	5.9 - 52.5	0.41
3060103	6490	SAVANNAH R	5.1	29.5	10 - 70	0.0	0.0 - 0.0	2.0	0.7 - 3.9	0.5	0.1 - 1.3	0.5	0.2 - 1.1	0.0	0.0 - 0.0	26.5	8.7 - 62.9	0.41
3060103	6491	ALLEN CR	30.6	23.9	9 - 45	0.0	0.0 - 0.0	4.2	1.5 - 8.8	1.6	0.5 - 3.6	3.3	1.2 - 8.0	0.0	0.0 - 0.0	14.9	5.2 - 32.3	0.41
3060103	6492	SAVANNAH R	2.3	29.6	10 - 77	0.0	0.0 - 0.0	2.9	1.0 - 7.0	0.2	0.0 - 0.5	0.0	0.0 - 0.0	0.0	0.0 - 0.0	26.6	9.4 - 73.7	0.41
3060103	6493	SAVANNAH R	50.5	33.5	12 - 86	0.0	0.0 - 0.0	4.3	1.4 - 11.2	4.2	1.2 - 12.1	7.7	2.4 - 19.2	0.0	0.0 - 0.0	17.2	6.3 - 46.9	0.41
3060103	6494	LITTLE GENEROST	88.6	43.2	16 - 77	1.7	0.6 - 3.2	7.2	2.6 - 13.5	5.1	1.5 - 12.7	16.2	5.3 - 35.5	0.0	0.0 - 0.0	13.0	4.8 - 27.1	0.41
3060103	6495	SAVANNAH R	23.1	26.9	10 - 64	0.0	0.0 - 0.0	2.8	1.0 - 7.2	2.8	0.8 - 8.4	3.8	1.6 - 9.2	0.0	0.0 - 0.0	17.5	6.6 - 41.8	0.41
3060103	6496	SAVANNAH R	15.3	26.9	9 - 51	0.0	0.0 - 0.0	5.2	1.6 - 10.8	2.5	0.7 - 7.4	4.7	1.6 - 11.3	0.0	0.0 - 0.0	14.5	4.6 - 32.8	0.41
3060103	6497	BIG GENROSTEE C	60.1	43.7	14 - 93	0.0	0.0 - 0.0	5.7	1.8 - 13.4	5.7	1.9 - 15.2	16.6	5.4 - 45.1	0.0	0.0 - 0.0	15.7	5.2 - 36.0	0.41
3060103	6499	MOUNTAIN CR	33.5	33.2	11 - 71	0.0	0.0 - 0.0	6.7	2.1 - 15.5	3.7	1.0 - 10.9	9.7	2.7 - 25.6	0.0	0.0 - 0.0	13.1	4.7 - 34.4	0.37
3060103	6500	BIG GENROSTEE C	93.2	191.3	68 - 332	130.3	44.7 - 268.3	33.9	11.9 - 66.2	3.7	1.0 - 11.6	11.2	3.2 - 27.3	0.0	0.0 - 0.0	12.2	4.3 - 28.6	0.37
3060103	6501	DEVIL FK CR	28.0	52.4	17 - 104	0.0	0.0 - 0.0	9.0	2.8 - 19.7	7.1	2.1 - 17.2	23.6	8.1 - 55.0	0.0	0.0 - 0.0	12.6	3.8 - 26.7	0.38
3060103	6502	SAVANNAH R	2.8	30.1	8 - 60	0.0	0.0 - 0.0	0.4	0.1 - 0.9	3.3	0.9 - 8.7	2.8	0.9 - 7.7	0.0	0.0 - 0.0	23.6	6.2 - 51.6	0.41
3060103	6503	CEDAR CR	93.7	55.1	17 - 99	0.0	0.0 - 0.0	13.0	4.1 - 28.7	17.2	5.0 - 51.3	12.5	3.8 - 34.9	0.0	0.0 - 0.0	12.4	3.8 - 32.0	0.41
3060103	6504	COLDWATER CR	198.8	46.5	13 - 94	0.0	0.0 - 0.0	6.2	1.7 - 11.8	13.9	3.6 - 38.9	13.8	4.0 - 33.4	0.0	0.0 - 0.0	12.6	3.6 - 28.0	0.41
3060103	6505	VAN CR	62.2	21.7	7 - 46	0.0	0.0 - 0.0	3.2	1.0 - 7.2	2.2	0.6 - 5.7	3.0	0.9 - 7.1	0.0	0.0 - 0.0	13.4	4.6 - 28.7	0.41
3060103	6506	BEAVERDAM CR	135.9	37.1	12 - 66	4.8	1.6 - 10.4	9.9	2.8 - 21.8	3.4	1.1 - 8.0	5.3	1.7 - 15.4	0.0	0.0 - 0.0	13.7	4.0 - 28.4	0.41
3060103	6507	LITTLE BEAVERDA	76.2	54.2	18 - 121	0.0	0.0 - 0.0	6.9	2.0 - 15.9	19.1	5.4 - 50.9	16.0	5.5 - 41.5	0.0	0.0 - 0.0	12.1	3.8 - 30.1	0.36
3060103	6508	BEAVERDAM CR	110.7	63.1	19 - 150	0.0	0.0 - 0.0	8.4	2.6 - 18.8	22.9	6.3 - 67.6	19.0	5.6 - 54.2	0.0	0.0 - 0.0	12.8	3.8 - 31.3	0.36
3060104	6510	COODY R	41.0	53.9	19 - 123	0.0	0.0 - 0.0	4.9	1.7 - 12.9	11.9	3.3 - 36.0	21.4	7.0 - 54.6	0.0	0.0 - 0.0	15.7	5.3 - 43.8	0.42
3060104	6511	BROAD R	83.8	47.0	14 - 98	0.0	0.0 - 0.0	4.6	1.4 - 9.7	8.1	2.2 - 27.3	14.1	4.2 - 39.3	0.0	0.0 - 0.0	20.2	6.0 - 56.1	0.42

8-digit HUC	ID	Name	Area	Catchment Yield kg/km2	Catchment Yield 90% CI	Point sources kg/km2	Point sources 90% CI	Developed Land kg/km2	Developed Land 90% CI	Manure kg/km2	Manure 90% CI	Agricultural Land kg/km2	Agricultural Land 90% CI	Phosphate Mines kg/km2	Phosphate Mines 90% CI	Soil parent rock kg/km2	Soil parent rock 90% CI	Frac
3060104	6512	WAHATCHEE R	54.7	64.4	20 - 145	0.0	0.0 - 0.0	5.2	1.5 - 11.3	13.7	3.2 - 38.6	25.8	7.9 - 69.0	0.0	0.0 - 0.0	19.6	6.1 - 52.1	0.41
3060104	6513	BROAD R	34.8	65.3	19 - 115	0.0	0.0 - 0.0	4.8	1.5 - 8.2	15.2	4.5 - 35.1	27.2	8.1 - 62.9	0.0	0.0 - 0.0	18.2	5.2 - 40.7	0.41
3060104	6514	BROAD R	2.9	32.3	12 - 92	0.0	0.0 - 0.0	2.1	0.7 - 6.1	3.7	1.0 - 15.3	0.3	0.1 - 1.0	0.0	0.0 - 0.0	26.1	9.8 - 75.9	0.41
3060104	6515	FALLING CR	121.1	47.5	14 - 86	6.4	2.1 - 12.9	10.9	3.5 - 22.5	5.7	1.5 - 15.7	9.7	3.2 - 24.7	0.0	0.0 - 0.0	14.7	5.1 - 32.1	0.41
3060104	6516	BROAD R	154.5	31.6	9 - 82	0.0	0.0 - 0.0	5.6	1.6 - 14.2	6.7	1.9 - 22.1	4.1	1.3 - 11.5	0.0	0.0 - 0.0	15.1	4.9 - 39.3	0.41
3060104	6517	DOVE CR	88.1	28.0	8 - 51	0.0	0.0 - 0.0	7.0	2.1 - 13.7	3.6	1.1 - 9.1	5.8	1.7 - 15.3	0.0	0.0 - 0.0	11.6	3.4 - 26.1	0.40
3060104	6518	BROAD R	13.5	21.5	7 - 49	0.0	0.0 - 0.0	4.2	1.3 - 10.4	3.5	1.0 - 10.5	2.2	0.7 - 6.4	0.0	0.0 - 0.0	11.6	3.9 - 29.0	0.40
3060104	6519	BROAD R	135.7	36.6	12 - 72	0.0	0.0 - 0.0	6.3	2.1 - 12.6	7.8	2.1 - 23.7	7.9	2.4 - 18.9	0.0	0.0 - 0.0	14.5	5.0 - 33.5	0.40
3060104	6520	MILL SHOAL CR	70.7	50.9	17 - 119	0.0	0.0 - 0.0	8.0	2.3 - 18.9	16.5	5.0 - 51.5	14.4	4.7 - 43.4	0.0	0.0 - 0.0	12.0	3.6 - 27.8	0.39
3060104	6521	BROAD R	8.6	37.2	13 - 72	0.0	0.0 - 0.0	7.2	2.6 - 16.0	10.1	3.2 - 32.4	5.0	1.5 - 13.0	0.0	0.0 - 0.0	14.9	4.7 - 31.6	0.39
3060104	6522	BROAD R	122.9	62.6	22 - 126	3.0	1.1 - 5.9	8.4	2.8 - 17.9	23.4	7.3 - 77.1	12.3	4.3 - 31.4	0.0	0.0 - 0.0	15.5	6.1 - 35.7	0.38
3060104	6523	BROAD R	26.8	87.4	32 - 186	0.0	0.0 - 0.0	13.5	5.0 - 30.1	40.8	13.1 - 133.9	16.4	6.0 - 39.7	0.0	0.0 - 0.0	16.8	6.0 - 43.9	0.38
3060104	6524	BROAD R, N FK	39.3	80.8	32 - 166	0.0	0.0 - 0.0	15.8	6.0 - 34.7	34.7	12.2 - 114.5	13.3	4.6 - 33.1	0.0	0.0 - 0.0	17.0	6.5 - 42.5	0.37
3060104	6525	DOUBLE CR	2.3	72.6	25 - 168	0.0	0.0 - 0.0	7.8	3.1 - 17.6	34.6	11.2 - 115.2	12.7	4.6 - 35.5	0.0	0.0 - 0.0	17.6	7.2 - 44.4	0.36
3060104	6526	DOUBLE CR	34.0	95.5	31 - 186	0.0	0.0 - 0.0	12.5	4.2 - 28.0	46.1	13.4 - 111.5	20.5	6.9 - 54.3	0.0	0.0 - 0.0	16.4	5.1 - 36.6	0.36
3060104	6527	UNA WATTI CR	50.1	118.9	37 - 297	18.2	6.8 - 50.8	15.0	4.3 - 38.3	50.8	15.7 - 146.7	20.7	6.3 - 64.6	0.0	0.0 - 0.0	14.3	4.9 - 39.5	0.36
3060104	6528	BROAD R, N FK	220.8	71.0	26 - 149	0.0	0.0 - 0.0	12.7	4.4 - 27.7	29.9	9.5 - 77.3	13.9	4.6 - 39.4	0.0	0.0 - 0.0	14.5	5.2 - 37.1	0.36
3060104	6529	BROAD R, MIDDLE	193.1	78.3	30 - 188	0.0	0.0 - 0.0	10.1	3.4 - 23.5	39.0	13.2 - 107.9	15.4	5.1 - 39.3	0.0	0.0 - 0.0	13.8	5.0 - 36.4	0.37
3060104	6530	LEATHERWOOD C	62.2	44.7	15 - 92	0.0	0.0 - 0.0	6.4	2.1 - 14.7	16.0	4.7 - 52.8	7.4	2.3 - 19.2	0.0	0.0 - 0.0	15.0	5.9 - 33.8	0.33
3060104	6531	BROAD R, MIDDLE	158.9	53.7	20 - 109	0.0	0.0 - 0.0	9.2	3.4 - 21.0	21.1	7.2 - 58.4	7.9	3.2 - 20.0	0.0	0.0 - 0.0	15.5	6.0 - 38.3	0.33
3060104	6532	HUDSON R	11.8	66.2	20 - 157	0.0	0.0 - 0.0	9.6	3.0 - 24.7	26.4	6.3 - 71.0	13.5	4.0 - 38.3	0.0	0.0 - 0.0	16.7	5.4 - 41.2	0.38
3060104	6533	NAILS CR	31.5	81.8	29 - 201	0.0	0.0 - 0.0	7.5	2.5 - 16.8	41.4	13.2 - 108.9	16.5	5.1 - 46.3	0.0	0.0 - 0.0	16.4	5.8 - 39.4	0.37
3060104	6534	NAILS CR	68.5	79.2	26 - 168	0.0	0.0 - 0.0	9.4	3.3 - 21.8	41.0	12.3 - 108.7	15.6	5.4 - 45.4	0.0	0.0 - 0.0	13.1	4.6 - 33.9	0.35
3060104	6535	CURLAN CR	49.9	75.8	21 - 162	0.0	0.0 - 0.0	8.8	2.8 - 15.6	39.2	9.8 - 104.0	15.3	4.5 - 34.7	0.0	0.0 - 0.0	12.5	3.7 - 29.6	0.35
3060104	6536	HUDSON R	62.5	68.7	24 - 152	0.0	0.0 - 0.0	7.3	2.3 - 15.6	29.5	8.9 - 88.6	14.9	4.5 - 38.3	0.0	0.0 - 0.0	16.9	6.0 - 43.9	0.37
3060104	6537	HUDSON R	11.0	77.3	21 - 145	0.0	0.0 - 0.0	8.4	2.4 - 16.3	41.6	11.2 - 96.9	15.8	5.1 - 38.6	0.0	0.0 - 0.0	11.5	2.9 - 24.3	0.36
3060104	6538	HUDSON R	260.7	62.6	23 - 127	0.0	0.0 - 0.0	9.8	3.3 - 20.8	27.9	10.0 - 72.7	10.4	3.2 - 31.6	0.0	0.0 - 0.0	14.5	5.8 - 33.1	0.36
3060104	6539	GROVE CR	103.0	76.8	25 - 175	11.2	3.4 - 30.0	17.2	6.5 - 43.7	25.9	7.4 - 71.3	10.5	3.5 - 31.6	0.0	0.0 - 0.0	12.0	3.8 - 30.4	0.36
3060104	6540	HICKORY LEVEL C	47.9	58.8	18 - 144	0.0	0.0 - 0.0	8.2	2.5 - 17.4	26.6	7.7 - 81.5	9.9	2.8 - 26.0	0.0	0.0 - 0.0	14.1	4.6 - 39.1	0.33
3060104	6541	GROVE CR	103.9	70.3	26 - 134	0.0	0.0 - 0.0	8.9	3.0 - 17.8	34.5	12.4 - 90.7	13.1	5.4 - 30.8	0.0	0.0 - 0.0	13.8	4.8 - 32.1	0.33
3060104	6542	BLACK CR	42.2	66.7	25 - 128	0.0	0.0 - 0.0	7.9	2.7 - 20.0	29.6	10.9 - 64.2	13.2	4.4 - 37.0	0.0	0.0 - 0.0	16.0	6.4 - 32.0	0.36
3060104	6543	SCULL SHOAL CR	51.0	59.7	22 - 157	0.0	0.0 - 0.0	7.4	2.7 - 17.6	24.9	8.0 - 75.7	13.3	5.0 - 37.7	0.0	0.0 - 0.0	14.0	5.1 - 39.3	0.38
3060104	6544	BROAD R, S FK	73.1	54.9	19 - 106	0.0	0.0 - 0.0	8.0	2.6 - 17.1	21.6	7.1 - 49.1	11.6	3.8 - 28.9	0.0	0.0 - 0.0	13.6	5.0 - 36.2	0.40
3060104	6545	BROAD R, S FK	3.0	19.8	8 - 42	0.0	0.0 - 0.0	2.6	0.9 - 5.1	3.1	1.0 - 9.0	1.6	0.6 - 3.5	0.0	0.0 - 0.0	12.5	4.9 - 32.5	0.39
3060104	6546	BROAD R, S FK	3.0	42.8	16 - 90	0.0	0.0 - 0.0	8.8	3.0 - 17.2	12.6	3.8 - 37.5	6.2	2.2 - 15.7	0.0	0.0 - 0.0	15.3	4.9 - 38.3	0.39
3060104	6547	BROAD R, S FK	136.0	59.7	20 - 125	0.0	0.0 - 0.0	11.1	3.5 - 26.4	20.9	5.7 - 62.9	13.1	4.3 - 32.4	0.0	0.0 - 0.0	14.7	5.1 - 39.5	0.38
3060104	6548	BROAD R, S FK	129.1	73.2	24 - 177	0.0	0.0 - 0.0	9.8	3.3 - 27.6	31.1	9.4 - 109.4	17.2	5.1 - 52.4	0.0	0.0 - 0.0	15.0	4.7 - 36.7	0.36
3060104	6549	*A	93.9	59.5	20 - 118	0.0	0.0 - 0.0	12.7	4.6 - 26.1	21.6	6.0 - 63.0	11.4	3.3 - 27.7	0.0	0.0 - 0.0	13.7	4.9 - 30.2	0.36
3060104	6550	BIG CLOUD CR	42.1	51.8	14 - 105	0.0	0.0 - 0.0	6.7	1.8 - 12.7	18.0	4.3 - 44.7	12.4	3.5 - 30.5	0.0	0.0 - 0.0	14.8	4.2 - 35.8	0.38

8-digit HUC	ID	Name	Area	Catchment Yield		Point sources		Developed Land		Manure		Agricultural Land		Phosphate Mines		Soil parent rock		Frac
				kg/km^2	90% CI	kg/km^2	90% CI	kg/km^2	90% CI	kg/km^2	90% CI	kg/km^2	90% CI	kg/km^2	90% CI	kg/km^2	90% CI	
3060104	6551	BIG COULD CR, LC	28.6	54.2	19 - 111	0.0	0.0 - 0.0	5.1	1.9 - 11.8	20.4	6.1 - 55.8	14.5	5.0 - 34.8	0.0	0.0 - 0.0	14.2	5.3 - 31.8	0.36
3060104	6552	BIG CLOUD CR	13.8	60.2	18 - 103	0.0	0.0 - 0.0	4.1	1.2 - 8.2	24.2	6.2 - 55.3	17.1	4.9 - 46.4	0.0	0.0 - 0.0	14.9	4.5 - 32.9	0.36
3060104	6553	MILL CR	10.9	70.3	23 - 135	0.0	0.0 - 0.0	5.9	2.1 - 11.0	29.0	8.8 - 69.7	21.1	6.7 - 51.1	0.0	0.0 - 0.0	14.2	4.5 - 32.8	0.33
3060104	6554	BIG CLOUD CR	27.0	51.5	19 - 117	0.0	0.0 - 0.0	5.9	2.2 - 14.3	19.1	6.3 - 70.5	13.0	5.0 - 34.2	0.0	0.0 - 0.0	13.4	4.9 - 35.2	0.33
3060104	6555	GROVE CR	55.1	36.5	14 - 73	0.0	0.0 - 0.0	7.4	2.5 - 16.6	10.9	3.5 - 24.9	6.9	2.5 - 16.9	0.0	0.0 - 0.0	11.3	4.2 - 25.0	0.39
3060104	6556	BEAVER CR	19.2	25.1	8 - 51	0.0	0.0 - 0.0	4.3	1.5 - 9.0	6.2	1.9 - 19.5	3.8	1.2 - 9.6	0.0	0.0 - 0.0	10.8	3.4 - 26.2	0.39
3060104	6557	LONG CR	13.6	33.0	12 - 63	0.0	0.0 - 0.0	3.5	1.3 - 7.4	3.9	1.1 - 11.8	1.8	0.6 - 4.8	0.0	0.0 - 0.0	23.8	8.5 - 51.9	0.41
3060104	6558	LONG CR	29.4	30.0	10 - 69	0.0	0.0 - 0.0	3.3	1.2 - 8.1	4.1	1.1 - 13.7	2.3	0.8 - 6.1	0.0	0.0 - 0.0	20.2	7.4 - 51.6	0.40
3060104	6559	MACK'S CR	59.2	30.9	9 - 66	0.0	0.0 - 0.0	6.1	2.0 - 11.6	6.5	1.7 - 20.9	3.9	1.3 - 9.2	0.0	0.0 - 0.0	14.4	4.1 - 35.4	0.40
3060104	6560	LONG CR	15.0	27.5	10 - 58	0.0	0.0 - 0.0	6.6	2.2 - 13.3	4.4	1.3 - 12.8	1.4	0.5 - 3.7	0.0	0.0 - 0.0	15.2	5.7 - 36.5	0.40
3060104	6561	LONG CR	21.8	30.8	12 - 61	0.0	0.0 - 0.0	6.2	2.3 - 11.8	4.4	1.4 - 10.8	2.3	0.9 - 5.3	0.0	0.0 - 0.0	18.0	6.8 - 39.5	0.39
3060104	6562	INDIAN CR	62.5	34.1	10 - 77	0.0	0.0 - 0.0	5.5	1.8 - 12.3	10.0	2.9 - 29.7	6.4	2.1 - 18.2	0.0	0.0 - 0.0	12.2	4.0 - 30.3	0.38
3060104	6563	LONG CR	5.5	45.2	14 - 109	0.0	0.0 - 0.0	8.6	2.6 - 20.5	8.8	2.4 - 28.5	4.0	1.2 - 9.3	0.0	0.0 - 0.0	23.7	7.2 - 60.8	0.38
3060104	6564	LONG CR	121.6	29.3	11 - 70	0.0	0.0 - 0.0	5.8	2.0 - 14.6	7.2	1.9 - 23.1	4.5	1.6 - 13.9	0.0	0.0 - 0.0	11.7	4.4 - 28.1	0.37
3060104	6565	BUFFALO CR	62.2	56.5	21 - 136	0.0	0.0 - 0.0	5.5	1.7 - 11.8	20.7	6.7 - 63.1	13.1	4.6 - 35.8	0.0	0.0 - 0.0	17.2	6.0 - 42.9	0.37
3060104	6566	LONG CR, DRY FK	2.9	25.2	9 - 56	0.0	0.0 - 0.0	5.4	2.0 - 13.2	0.9	0.3 - 2.4	0.0	0.0 - 0.0	0.0	0.0 - 0.0	18.9	6.5 - 44.6	0.39
3060104	6567	LONG CR, DRY FK	122.0	42.6	14 - 88	0.0	0.0 - 0.0	4.7	1.7 - 9.9	11.0	3.2 - 36.5	9.1	3.0 - 20.6	0.0	0.0 - 0.0	17.7	5.6 - 39.8	0.38
3060104	6568	HUTTON CR	37.5	34.5	9 - 69	0.0	0.0 - 0.0	4.6	1.4 - 10.2	6.0	1.3 - 18.1	8.5	2.6 - 24.1	0.0	0.0 - 0.0	15.4	4.1 - 36.5	0.38
3060104	6569	CLARK CR	134.9	22.8	7 - 56	0.0	0.0 - 0.0	3.1	1.0 - 8.4	4.8	1.5 - 14.1	7.1	2.3 - 19.0	0.0	0.0 - 0.0	7.8	2.4 - 21.8	0.40
3060105	6570	LLOYD CR	61.9	23.2	8 - 44	0.0	0.0 - 0.0	3.7	1.2 - 7.1	3.0	0.9 - 8.5	7.8	2.2 - 19.9	0.0	0.0 - 0.0	8.7	2.5 - 20.0	0.41
3060105	6571	LITTLE R	30.9	31.4	11 - 69	0.0	0.0 - 0.0	3.1	1.0 - 7.1	1.4	0.5 - 4.6	5.6	1.9 - 14.9	0.0	0.0 - 0.0	21.3	7.8 - 55.0	0.41
3060105	6572	LITTLE R	8.5	35.3	12 - 76	0.0	0.0 - 0.0	5.6	1.8 - 12.2	2.2	0.7 - 6.7	7.5	2.6 - 19.3	0.0	0.0 - 0.0	20.0	6.6 - 51.2	0.41
3060105	6573	LITTLE R	166.7	31.6	10 - 83	0.0	0.0 - 0.0	4.4	1.4 - 11.1	5.7	1.5 - 20.1	9.4	3.1 - 26.7	0.0	0.0 - 0.0	12.1	3.6 - 37.9	0.41
3060105	6574	ROCKY CR	85.2	55.4	17 - 99	18.3	5.6 - 34.2	6.9	2.1 - 13.6	6.9	1.9 - 17.5	11.2	3.6 - 27.4	0.0	0.0 - 0.0	12.1	3.2 - 28.8	0.40
3060105	6575	LITTLE R	56.2	15.8	6 - 33	0.0	0.0 - 0.0	3.4	1.1 - 7.5	1.7	0.5 - 5.6	1.3	0.4 - 3.2	0.0	0.0 - 0.0	9.4	3.3 - 24.1	0.40
3060105	6576	LITTLE R	63.3	17.2	6 - 33	0.0	0.0 - 0.0	3.7	1.3 - 7.5	1.8	0.5 - 5.4	2.6	0.8 - 8.1	0.0	0.0 - 0.0	9.0	3.1 - 19.1	0.38
3060105	6577	LITTLE R	19.7	43.4	15 - 90	0.0	0.0 - 0.0	3.2	1.2 - 6.2	9.8	3.1 - 23.4	16.8	5.4 - 39.2	0.0	0.0 - 0.0	13.7	4.9 - 32.5	0.37
3060105	6578	*A	90.3	30.6	12 - 61	0.0	0.0 - 0.0	5.2	2.0 - 9.5	5.2	1.7 - 13.6	7.7	2.7 - 16.4	0.0	0.0 - 0.0	12.5	4.5 - 27.0	0.36
3060105	6579	LITTLE R	16.2	27.6	10 - 50	0.0	0.0 - 0.0	2.8	0.9 - 5.9	4.8	1.5 - 17.4	7.7	2.8 - 17.9	0.0	0.0 - 0.0	12.3	4.5 - 28.3	0.36
3060105	6580	KETTLE CR	133.0	26.9	9 - 87	0.0	0.0 - 0.0	3.0	0.9 - 7.6	4.6	1.5 - 15.0	7.1	2.2 - 22.6	0.0	0.0 - 0.0	12.2	4.4 - 42.1	0.35
3060105	6581	LITTLE R	69.7	24.2	9 - 50	0.0	0.0 - 0.0	4.5	1.5 - 11.3	2.1	0.6 - 5.2	4.2	1.5 - 10.9	0.0	0.0 - 0.0	13.4	5.3 - 30.7	0.35
3060105	6582	LITTLE R, N FK	54.7	31.3	12 - 59	0.0	0.0 - 0.0	3.0	1.1 - 5.1	3.2	1.0 - 8.4	6.7	2.5 - 14.5	0.0	0.0 - 0.0	18.5	6.8 - 43.7	0.34
3060105	6583	LITTLE R, N FK, SY	57.2	28.7	9 - 51	0.0	0.0 - 0.0	4.6	1.4 - 8.5	5.7	1.7 - 18.5	3.0	1.0 - 6.7	0.0	0.0 - 0.0	15.4	5.3 - 34.4	0.30
3060105	6584	LITTLE R, N FK	73.7	25.2	10 - 48	0.0	0.0 - 0.0	3.3	1.2 - 7.2	5.2	1.9 - 14.0	3.8	1.3 - 9.7	0.0	0.0 - 0.0	12.9	4.9 - 29.4	0.30
3060105	6585	LITTLE R, S FK	31.6	18.2	5 - 38	0.0	0.0 - 0.0	4.2	1.2 - 10.4	1.1	0.3 - 3.0	2.8	0.9 - 7.6	0.0	0.0 - 0.0	10.1	3.0 - 23.6	0.34
3060105	6586	LITTLE R, S FK	71.6	23.9	7 - 48	0.0	0.0 - 0.0	3.7	1.1 - 8.8	3.1	0.7 - 10.3	4.5	1.5 - 10.4	0.0	0.0 - 0.0	12.6	3.7 - 30.1	0.32
3060105	6587	SHERRILLS CR	45.0	19.1	6 - 47	0.0	0.0 - 0.0	3.8	1.1 - 9.1	1.4	0.4 - 3.5	1.9	0.6 - 5.6	0.0	0.0 - 0.0	12.0	4.1 - 33.9	0.32
3060105	6588	KINDRICK CR	66.0	21.4	8 - 44	0.0	0.0 - 0.0	3.1	1.0 - 6.8	1.7	0.5 - 4.7	3.9	1.3 - 10.6	0.0	0.0 - 0.0	12.7	4.5 - 29.6	0.37
3060105	6589	WILLIAMS CR	104.6	19.9	6 - 43	0.0	0.0 - 0.0	4.0	1.2 - 8.6	1.1	0.3 - 2.9	2.8	0.9 - 7.7	0.0	0.0 - 0.0	12.0	3.7 - 28.6	0.38

8-digit HUC	ID	Name	Area	Catchment Yield		Point sources		Developed Land		Manure		Agricultural Land		Phosphate Mines		Soil parent rock		Frac
				kg/km^2	90% CI	kg/km^2	90% CI	kg/km^2	90% CI	kg/km^2	90% CI	kg/km^2	90% CI	kg/km^2	90% CI	kg/km^2	90% CI	
3060105	6590	MIDDLE CR	8.8	39.0	12 - 75	0.0	0.0 - 0.0	7.7	2.3 - 14.8	1.2	0.3 - 3.0	4.6	1.4 - 11.1	0.0	0.0 - 0.0	25.5	7.0 - 57.7	0.41
3060105	6591	HART CR	108.5	21.8	7 - 40	0.0	0.0 - 0.0	3.7	1.3 - 9.0	1.4	0.5 - 3.8	3.1	0.9 - 8.6	0.0	0.0 - 0.0	13.5	4.4 - 30.8	0.40
3060105	6592	MIDDLE CR	24.1	106.9	40 - 248	76.8	27.3 - 185.6	5.0	1.8 - 10.6	1.4	0.4 - 4.5	3.7	1.3 - 9.9	0.0	0.0 - 0.0	20.0	7.4 - 56.7	0.40
3060105	6593	MIDDLE CR	93.9	23.6	9 - 46	0.0	0.0 - 0.0	5.5	1.8 - 10.9	1.4	0.5 - 3.1	3.9	1.4 - 9.0	0.0	0.0 - 0.0	12.8	4.9 - 26.8	0.36
3060105	6594	MATLOX CR	60.1	31.1	10 - 67	0.0	0.0 - 0.0	13.0	4.3 - 26.8	1.5	0.5 - 4.1	5.4	1.6 - 14.0	0.0	0.0 - 0.0	11.1	3.5 - 28.1	0.36
3060105	6595	GERMANY CR	102.9	28.1	9 - 50	0.0	0.0 - 0.0	7.2	2.6 - 14.7	2.0	0.6 - 5.7	6.8	2.2 - 15.2	0.0	0.0 - 0.0	12.1	4.1 - 25.6	0.41
3060106	6596	SAVANNAH R	108.0	24.3	8 - 49	0.0	0.0 - 0.0	1.8	0.6 - 3.5	0.8	0.3 - 2.2	6.2	2.2 - 13.1	0.0	0.0 - 0.0	15.6	5.5 - 44.5	0.97
3060106	6597	SAVANNAH R, WA	98.3	17.6	6 - 50	0.0	0.0 - 0.0	3.2	1.0 - 7.6	0.6	0.2 - 1.9	4.9	1.8 - 13.7	0.0	0.0 - 0.0	8.9	2.8 - 25.2	0.96
3060106	6598	SAVANNAH R	70.7	20.4	8 - 42	0.0	0.0 - 0.0	2.0	0.7 - 4.0	0.4	0.1 - 1.2	4.6	1.6 - 12.3	0.0	0.0 - 0.0	13.5	5.0 - 32.6	0.96
3060106	6599	BRIER CR	77.2	15.3	5 - 28	0.0	0.0 - 0.0	2.8	0.9 - 5.8	0.4	0.1 - 1.1	2.2	0.7 - 4.8	0.0	0.0 - 0.0	10.0	3.1 - 21.4	0.96
3060106	6600	SAVANNAH R	8.5	23.3	6 - 38	0.0	0.0 - 0.0	2.8	0.8 - 5.3	0.8	0.2 - 2.1	7.8	2.1 - 18.8	0.0	0.0 - 0.0	11.9	3.4 - 23.4	0.96
3060106	6601	LOWER THREE RU	40.8	14.5	7 - 36	0.0	0.0 - 0.0	1.7	0.7 - 3.5	0.4	0.2 - 1.2	4.6	2.0 - 12.6	0.0	0.0 - 0.0	7.7	3.3 - 19.8	0.95
3060106	6602	LOWER THREE RU	17.3	14.1	6 - 29	0.0	0.0 - 0.0	2.8	1.1 - 6.0	0.4	0.1 - 1.0	3.9	1.5 - 10.2	0.0	0.0 - 0.0	7.0	2.8 - 16.9	0.91
3060106	6603	MILLER CR	105.7	13.4	6 - 31	0.0	0.0 - 0.0	2.6	0.8 - 6.8	0.5	0.2 - 1.2	4.2	1.5 - 11.1	0.0	0.0 - 0.0	6.2	2.5 - 13.6	0.87
3060106	6604	LOWER THREE RU	103.3	0.9	0 - 2	0.0	0.0 - 0.0	0.1	0.0 - 0.3	0.0	0.0 - 0.1	0.3	0.1 - 0.8	0.0	0.0 - 0.0	0.4	0.1 - 0.9	0.87
3060106	6605	MILL CR	52.8	16.5	5 - 29	0.0	0.0 - 0.0	2.9	1.0 - 5.3	0.7	0.2 - 1.7	8.1	2.6 - 17.3	0.0	0.0 - 0.0	4.7	1.6 - 9.4	0.91
3060106	6606	SAVANNAH R	19.8	15.3	5 - 41	0.0	0.0 - 0.0	1.4	0.4 - 3.8	0.5	0.1 - 1.5	5.0	1.5 - 14.6	0.0	0.0 - 0.0	8.4	2.5 - 25.1	0.95
3060106	6607	SAVANNAH R	37.6	19.0	7 - 55	0.0	0.0 - 0.0	1.6	0.5 - 4.5	0.9	0.3 - 2.6	9.9	3.6 - 28.3	0.0	0.0 - 0.0	6.7	2.5 - 17.4	0.95
3060106	6608	STEEL CR	18.6	9.6	3 - 23	0.0	0.0 - 0.0	1.1	0.4 - 2.6	0.2	0.1 - 0.7	2.8	0.9 - 7.3	0.0	0.0 - 0.0	5.4	1.9 - 14.1	0.95
3060106	6609	STEEL CR	19.2	6.4	3 - 14	0.0	0.0 - 0.0	1.1	0.4 - 2.4	0.1	0.0 - 0.2	0.2	0.1 - 0.6	0.0	0.0 - 0.0	5.1	2.0 - 12.3	0.94
3060106	6610	MEYERS CR	50.0	5.5	2 - 12	0.0	0.0 - 0.0	0.2	0.1 - 0.4	0.1	0.0 - 0.2	0.3	0.1 - 0.7	0.0	0.0 - 0.0	5.0	1.6 - 11.5	0.84
3060106	6611	STEEL CR	24.7	9.6	3 - 23	0.0	0.0 - 0.0	1.9	0.6 - 4.4	0.2	0.0 - 0.5	0.6	0.2 - 1.8	0.0	0.0 - 0.0	6.9	2.3 - 17.7	0.84
3060106	6612	PEN CR	99.1	7.4	2 - 15	0.0	0.0 - 0.0	1.6	0.6 - 3.1	0.1	0.0 - 0.2	0.2	0.1 - 0.5	0.0	0.0 - 0.0	5.4	1.8 - 12.8	0.94
3060106	6613	SAVANNAH R	29.5	14.0	5 - 32	0.0	0.0 - 0.0	2.3	0.8 - 5.2	0.8	0.2 - 2.1	4.8	1.7 - 12.1	0.0	0.0 - 0.0	6.1	2.2 - 15.2	0.95
3060106	6614	SAVANNAH R	38.5	18.0	6 - 30	0.0	0.0 - 0.0	8.5	2.7 - 14.1	0.6	0.2 - 1.3	2.0	0.6 - 4.6	0.0	0.0 - 0.0	6.8	2.1 - 12.7	0.95
3060106	6615	FOURMILE CR	35.0	7.8	2 - 15	0.0	0.0 - 0.0	0.9	0.3 - 1.9	0.1	0.0 - 0.3	0.1	0.0 - 0.3	0.0	0.0 - 0.0	6.6	1.9 - 13.2	0.95
3060106	6616	SAVANNAH R	33.5	17.8	7 - 35	0.0	0.0 - 0.0	4.0	1.4 - 8.0	0.7	0.2 - 1.9	3.4	1.2 - 8.3	0.0	0.0 - 0.0	9.7	3.5 - 24.9	0.95
3060106	6617	UPPER THREE RUN	28.6	15.0	5 - 37	0.0	0.0 - 0.0	1.2	0.4 - 2.5	0.4	0.1 - 1.1	0.2	0.1 - 0.6	0.0	0.0 - 0.0	13.2	4.4 - 33.4	0.94
3060106	6618	UPPER THREE RUN	29.1	15.0	5 - 34	0.0	0.0 - 0.0	7.5	2.3 - 18.3	0.2	0.0 - 0.6	0.0	0.0 - 0.0	0.0	0.0 - 0.0	7.3	2.3 - 18.2	0.86
3060106	6619	TINKER CR	14.1	11.4	4 - 23	0.0	0.0 - 0.0	4.9	1.5 - 8.8	0.2	0.1 - 0.5	0.0	0.0 - 0.0	0.0	0.0 - 0.0	6.3	1.9 - 15.9	0.81
3060106	6620	MILL CR	22.9	6.8	3 - 13	0.0	0.0 - 0.0	0.4	0.1 - 0.6	0.1	0.0 - 0.4	0.0	0.0 - 0.0	0.0	0.0 - 0.0	6.3	2.4 - 12.6	0.68
3060106	6621	UPPER THREE RUN	129.8	8.0	2 - 19	0.0	0.0 - 0.0	0.8	0.2 - 2.1	0.4	0.1 - 1.2	1.0	0.3 - 2.7	0.0	0.0 - 0.0	5.8	1.5 - 14.7	0.81
3060106	6622	UPPER THREE RUN	4.2	5.3	1 - 12	0.0	0.0 - 0.0	0.0	0.0 - 0.0	0.0	0.0 - 0.0	0.0	0.0 - 0.0	0.0	0.0 - 0.0	5.3	1.5 - 11.8	0.73
3060106	6623	UPPER THREE RUN	41.1	11.2	4 - 25	0.0	0.0 - 0.0	1.5	0.5 - 3.1	1.5	0.5 - 4.2	3.3	1.2 - 7.7	0.0	0.0 - 0.0	5.0	1.5 - 13.6	0.73
3060106	6624	UPPER THREE RUN	93.2	13.6	5 - 27	0.0	0.0 - 0.0	3.2	1.0 - 6.9	1.7	0.6 - 4.9	3.9	1.2 - 9.6	0.0	0.0 - 0.0	4.8	1.8 - 11.3	0.71
3060106	6625	CEDAR CR	74.5	20.7	8 - 55	0.0	0.0 - 0.0	7.6	2.7 - 19.6	2.3	0.8 - 7.0	5.6	2.0 - 16.3	0.0	0.0 - 0.0	5.3	1.9 - 17.0	0.71
3060106	6626	UPPER THREE RUN	48.3	222.2	76 - 437	212.5	71.5 - 426.4	3.0	0.9 - 5.6	0.4	0.1 - 1.2	0.7	0.2 - 1.8	0.0	0.0 - 0.0	5.6	1.6 - 11.9	0.86
3060106	6627	SAVANNAH R	19.8	21.8	8 - 42	0.0	0.0 - 0.0	3.1	1.0 - 6.5	1.2	0.4 - 3.3	7.0	2.5 - 15.8	0.0	0.0 - 0.0	10.5	3.7 - 23.6	0.94
3060106	6628	SAVANNAH R	14.7	18.0	5 - 39	0.0	0.0 - 0.0	1.2	0.3 - 2.4	0.5	0.1 - 1.5	5.2	1.3 - 11.5	0.0	0.0 - 0.0	11.1	3.0 - 26.8	0.94

8-digit HUC	ID	Name	Area	Catchment Yield		Point sources		Developed Land		Manure		Agricultural Land		Phosphate Mines		Soil parent rock		Frac
				kg/km^2	90% CI	kg/km^2	90% CI	kg/km^2	90% CI	kg/km^2	90% CI	kg/km^2	90% CI	kg/km^2	90% CI	kg/km^2	90% CI	
3060106	6629	SAVANNAH R	5.7	21.2	7 - 37	0.0	0.0 - 0.0	4.1	1.5 - 9.0	0.6	0.2 - 1.7	5.6	2.0 - 11.9	0.0	0.0 - 0.0	11.0	3.7 - 23.3	0.94
3060106	6630	SAVANNAH R	129.0	24.5	8 - 59	0.0	0.0 - 0.0	3.3	1.0 - 8.1	2.4	0.7 - 6.6	5.9	2.0 - 16.4	0.0	0.0 - 0.0	12.9	4.4 - 34.3	0.94
3060106	6631	HOLLOW CR	30.4	20.8	9 - 37	0.0	0.0 - 0.0	2.7	1.1 - 5.3	2.8	0.9 - 7.6	6.8	2.6 - 17.6	0.0	0.0 - 0.0	8.5	3.3 - 18.8	0.94
3060106	6632	HOLLOW CR	120.3	19.5	6 - 42	0.0	0.0 - 0.0	9.4	2.9 - 19.0	1.5	0.5 - 4.4	3.7	1.3 - 9.6	0.0	0.0 - 0.0	4.9	1.7 - 12.0	0.84
3060106	6633	TOWN CR	99.5	20.4	8 - 40	0.0	0.0 - 0.0	8.3	2.9 - 16.1	1.9	0.6 - 5.2	4.5	1.7 - 11.5	0.0	0.0 - 0.0	5.6	1.9 - 12.7	0.84
3060106	6634	SAVANNAH R	19.2	26.3	8 - 58	0.0	0.0 - 0.0	3.6	1.0 - 7.6	0.8	0.2 - 2.3	4.0	1.1 - 9.4	0.0	0.0 - 0.0	18.0	5.7 - 44.2	0.94
3060106	6635	BEAR ISLAND R	51.7	28.2	11 - 53	0.0	0.0 - 0.0	1.8	0.7 - 3.4	3.9	1.3 - 11.0	9.6	3.7 - 23.2	0.0	0.0 - 0.0	12.9	5.0 - 30.8	0.94
3060106	6636	SAVANNAH R	3.9	10305.3	3521 - 20630	########	3511.3 - 2056[illegible]	1.8	0.6 - 4.1	1.4	0.4 - 3.6	3.2	1.1 - 7.0	0.0	0.0 - 0.0	21.0	7.3 - 53.6	0.94
3060106	6637	SAVANNAH R	15.7	53.1	17 - 103	0.0	0.0 - 0.0	22.9	7.3 - 49.1	3.6	1.1 - 10.6	9.3	2.7 - 24.1	0.0	0.0 - 0.0	17.2	6.0 - 40.0	0.94
3060106	6639	HORSE CR	31.9	35.9	12 - 91	0.0	0.0 - 0.0	22.3	7.1 - 58.7	1.6	0.5 - 5.5	3.4	1.2 - 10.5	0.0	0.0 - 0.0	8.6	3.2 - 23.6	0.93
3060106	6640	HORSE CR	45.0	30.5	12 - 67	0.1	0.0 - 0.3	21.9	8.1 - 48.7	0.8	0.3 - 1.9	1.5	0.5 - 3.9	0.0	0.0 - 0.0	6.2	2.1 - 14.0	0.90
3060106	6641	*A	35.3	50.0	17 - 102	0.0	0.0 - 0.0	41.9	14.6 - 93.7	0.8	0.2 - 1.9	1.8	0.7 - 4.2	0.0	0.0 - 0.0	5.6	2.5 - 10.5	0.83
3060106	6642	HORSE CR	34.4	52.7	16 - 106	0.0	0.0 - 0.0	45.3	13.1 - 93.2	0.3	0.1 - 0.8	0.7	0.2 - 1.8	0.0	0.0 - 0.0	6.4	1.7 - 13.8	0.83
3060106	6643	BRIDGE CR	28.0	22.4	7 - 44	0.0	0.0 - 0.0	14.1	4.5 - 29.2	1.2	0.3 - 3.3	2.6	0.7 - 6.0	0.0	0.0 - 0.0	4.5	1.5 - 10.2	0.80
3060106	6644	HORSE CR	132.4	14.5	5 - 32	0.0	0.0 - 0.0	5.8	2.0 - 14.1	1.2	0.3 - 3.9	2.8	1.0 - 8.4	0.0	0.0 - 0.0	4.7	1.7 - 12.3	0.80
3060106	6645	LITTLE HORSE CR	52.0	21.9	8 - 37	0.0	0.0 - 0.0	14.2	4.7 - 27.9	0.6	0.2 - 1.6	0.9	0.3 - 2.1	0.0	0.0 - 0.0	6.2	2.4 - 13.0	0.90
3060106	6646	LITTLE HORSE CR	42.6	16.6	6 - 33	0.0	0.0 - 0.0	8.1	2.8 - 18.3	1.1	0.3 - 2.8	2.4	0.8 - 5.7	0.0	0.0 - 0.0	5.0	1.8 - 13.2	0.76
3060106	6647	*B	23.5	17.3	6 - 29	0.0	0.0 - 0.0	6.7	2.1 - 12.6	1.3	0.4 - 3.0	3.3	0.9 - 8.5	0.0	0.0 - 0.0	6.0	2.0 - 13.4	0.76
3060106	6648	SAVANNAH R	207.3	93.0	34 - 197	25.4	9.5 - 56.7	52.1	18.4 - 125.5	0.6	0.2 - 1.9	2.6	0.8 - 7.3	0.0	0.0 - 0.0	12.3	4.6 - 25.4	0.93
3060106	6649	SAVANNAH R	49.0	34.1	12 - 65	0.0	0.0 - 0.0	16.2	5.4 - 34.8	0.3	0.1 - 0.8	2.6	0.8 - 5.5	0.0	0.0 - 0.0	15.0	5.3 - 35.1	0.92
3060106	6650	SAVANNAH R	15.0	25.3	9 - 48	0.0	0.0 - 0.0	3.9	1.2 - 8.6	0.8	0.2 - 2.5	1.6	0.5 - 3.9	0.0	0.0 - 0.0	19.0	6.7 - 41.5	0.92
3060106	6651	SAVANNAH R	1.4	39.1	15 - 90	0.0	0.0 - 0.0	4.6	1.8 - 10.6	1.7	0.5 - 5.8	9.7	3.7 - 23.1	0.0	0.0 - 0.0	23.1	8.8 - 56.4	0.92
3060106	6652	SAVANNAH R	38.7	22.5	8 - 62	0.0	0.0 - 0.0	5.9	2.0 - 13.1	0.6	0.2 - 1.9	1.1	0.4 - 2.8	0.0	0.0 - 0.0	15.1	5.3 - 47.6	0.92
3060106	6653	KIOKEE CR	79.2	18.9	6 - 43	0.0	0.0 - 0.0	3.9	1.1 - 8.7	0.5	0.1 - 1.4	2.6	0.8 - 7.8	0.0	0.0 - 0.0	11.9	3.7 - 31.6	0.92
3060106	6654	GREENBRIER CR	89.7	17.2	6 - 42	0.0	0.0 - 0.0	3.5	1.2 - 8.7	0.7	0.2 - 2.1	4.2	1.4 - 11.4	0.0	0.0 - 0.0	8.9	3.4 - 21.6	0.84
3060106	6655	KIOKEE CR	42.1	22.6	8 - 46	0.0	0.0 - 0.0	7.1	2.5 - 15.7	0.3	0.1 - 0.9	3.4	1.1 - 8.3	0.0	0.0 - 0.0	11.8	4.3 - 27.1	0.84
3060106	6656	KIOKEE CR	60.0	18.0	5 - 36	0.0	0.0 - 0.0	3.7	1.0 - 8.2	1.0	0.3 - 2.6	4.3	1.4 - 9.8	0.0	0.0 - 0.0	8.9	2.7 - 21.0	0.73
3060106	6657	KIOKEE CR, CAT [illegible]	17.6	23.6	10 - 50	0.0	0.0 - 0.0	7.3	2.8 - 16.2	0.5	0.1 - 1.4	5.6	1.8 - 15.9	0.0	0.0 - 0.0	10.1	4.2 - 24.0	0.73
3060106	6658	LITTLE KIOKEE C[illegible]	194.2	24.3	8 - 60	0.0	0.0 - 0.0	9.7	3.2 - 26.3	0.4	0.1 - 1.1	4.0	1.3 - 10.3	0.0	0.0 - 0.0	10.2	3.4 - 28.1	0.92
3060106	6659	UTCHEE CR	5.3	34.3	10 - 65	0.0	0.0 - 0.0	16.6	5.0 - 34.9	0.3	0.1 - 0.9	3.4	1.0 - 7.8	0.0	0.0 - 0.0	14.1	4.2 - 31.5	0.92
3060106	6660	UTCHEE CR	43.1	70.6	25 - 127	32.5	11.8 - 65.0	26.1	8.4 - 52.8	0.3	0.1 - 0.8	2.8	1.0 - 6.7	0.0	0.0 - 0.0	8.9	3.5 - 19.1	0.82
3060106	6661	*C	3.1	46.7	16 - 92	0.0	0.0 - 0.0	31.7	10.2 - 61.8	0.3	0.1 - 0.7	3.6	1.1 - 8.0	0.0	0.0 - 0.0	11.0	3.2 - 22.9	0.82
3060106	6662	BUTLER CR	24.8	1896.8	671 - 5658	1845.3	649.9 - 5514.[illegible]	42.6	14.0 - 118.3	0.1	0.0 - 0.5	1.0	0.4 - 2.9	0.0	0.0 - 0.0	7.7	2.8 - 21.5	0.93
3060106	6663	SPIRIT CR	15.5	473.3	157 - 1149	437.5	144.7 - 1069.[illegible]	21.7	6.3 - 53.9	0.2	0.1 - 0.8	2.6	0.8 - 7.1	0.0	0.0 - 0.0	11.3	3.9 - 30.5	0.94
3060106	6664	SPIRIT CR	184.8	52.8	14 - 131	21.7	6.0 - 55.2	23.2	6.3 - 54.5	0.2	0.0 - 0.5	1.6	0.4 - 4.4	0.0	0.0 - 0.0	6.2	2.1 - 16.2	0.88
3060106	6665	LITTLE SPIRIT CR	67.6	21.6	8 - 40	0.0	0.0 - 0.0	10.7	3.7 - 21.7	0.3	0.1 - 0.8	3.8	1.4 - 10.3	0.0	0.0 - 0.0	6.9	2.3 - 13.6	0.88
3060106	6666	MCBEAN CR	225.8	18.4	6 - 33	0.0	0.0 - 0.0	6.3	1.8 - 12.4	0.5	0.2 - 1.2	4.5	1.4 - 9.8	0.0	0.0 - 0.0	7.1	2.4 - 15.8	0.94
3060106	6667	BOGGY GUT CR	77.3	17.8	5 - 40	0.0	0.0 - 0.0	5.1	1.6 - 12.7	0.7	0.2 - 1.8	5.8	1.7 - 15.5	0.0	0.0 - 0.0	6.2	2.0 - 15.0	0.94
3060106	6668	NEWBERRY CR	52.4	15.0	5 - 32	0.0	0.0 - 0.0	5.7	1.8 - 13.8	0.6	0.2 - 2.0	3.8	1.3 - 9.9	0.0	0.0 - 0.0	4.8	1.7 - 11.8	0.94

8-digit HUC	ID	Name	Area	Catchment Yield kg/km^2	Catchment Yield 90% CI	Point sources kg/km^2	Point sources 90% CI	Developed Land kg/km^2	Developed Land 90% CI	Manure kg/km^2	Manure 90% CI	Agricultural Land kg/km^2	Agricultural Land 90% CI	Phosphate Mines kg/km^2	Phosphate Mines 90% CI	Soil parent rock kg/km^2	Soil parent rock 90% CI	Frac
3060106	6669	BEAVERDAM CR	54.2	12.6	4 - 25	0.0	0.0 - 0.0	3.5	1.2 - 7.5	0.7	0.2 - 2.4	3.6	1.2 - 9.3	0.0	0.0 - 0.0	4.8	1.7 - 11.5	0.95
3060106	6670	SWEETWATER CR	78.1	17.8	5 - 44	0.0	0.0 - 0.0	4.2	1.3 - 10.1	1.0	0.3 - 2.9	7.8	2.1 - 21.2	0.0	0.0 - 0.0	4.8	1.6 - 12.6	0.95
3060107	6671	STEVENS CR	55.1	22.3	7 - 51	0.0	0.0 - 0.0	6.9	2.1 - 15.6	1.0	0.3 - 3.0	3.7	1.2 - 11.0	0.0	0.0 - 0.0	10.6	3.6 - 25.8	0.92
3060107	6672	CHEVES CR	99.1	17.1	5 - 45	0.1	0.0 - 0.2	4.3	1.3 - 10.8	0.9	0.3 - 2.5	3.4	1.1 - 11.4	0.0	0.0 - 0.0	8.4	2.7 - 22.7	0.91
3060107	6673	STEVENS CR	11.9	16.4	5 - 32	0.0	0.0 - 0.0	1.9	0.5 - 4.2	0.4	0.1 - 1.0	1.3	0.4 - 3.2	0.0	0.0 - 0.0	12.9	3.8 - 29.5	0.91
3060107	6674	HORN CR	206.7	16.5	6 - 32	0.0	0.0 - 0.0	2.7	0.9 - 5.9	0.8	0.3 - 1.7	2.6	0.9 - 6.3	0.0	0.0 - 0.0	10.4	3.8 - 25.5	0.91
3060107	6675	STEVENS CR	72.5	20.6	8 - 44	0.0	0.0 - 0.0	2.3	0.8 - 4.5	0.9	0.3 - 2.6	3.1	1.2 - 7.1	0.0	0.0 - 0.0	14.3	5.4 - 32.6	0.91
3060107	6676	GUNDY CR	41.5	21.0	8 - 46	0.0	0.0 - 0.0	2.9	0.9 - 7.4	1.2	0.4 - 3.2	5.4	1.8 - 14.8	0.0	0.0 - 0.0	11.4	4.3 - 29.6	0.89
3060107	6677	STEVENS CR	41.7	15.9	6 - 38	0.0	0.0 - 0.0	2.6	0.9 - 5.7	0.4	0.1 - 1.3	1.1	0.4 - 2.8	0.0	0.0 - 0.0	11.8	4.3 - 29.4	0.89
3060107	6678	TURKEY CR	27.6	19.2	6 - 40	0.0	0.0 - 0.0	1.7	0.5 - 3.2	0.8	0.2 - 2.1	1.5	0.5 - 3.0	0.0	0.0 - 0.0	15.1	4.9 - 38.0	0.86
3060107	6679	BEAVERDAM CR	114.4	23.8	9 - 57	3.0	1.2 - 7.2	5.1	1.9 - 13.0	0.9	0.3 - 2.9	3.1	1.1 - 9.3	0.0	0.0 - 0.0	11.8	4.2 - 31.4	0.85
3060107	6680	TURKEY CR	25.7	20.2	6 - 51	0.0	0.0 - 0.0	4.1	1.1 - 9.3	0.4	0.1 - 1.1	0.9	0.3 - 2.2	0.0	0.0 - 0.0	14.9	4.2 - 36.2	0.85
3060107	6681	TURKEY CR	114.5	23.6	8 - 52	0.0	0.0 - 0.0	3.5	1.1 - 7.8	1.2	0.4 - 3.5	4.4	1.5 - 10.4	0.0	0.0 - 0.0	14.6	4.7 - 37.0	0.83
3060107	6682	LOG CR	63.0	25.7	9 - 50	0.0	0.0 - 0.0	4.8	1.6 - 9.8	1.6	0.5 - 3.9	7.4	2.5 - 17.8	0.0	0.0 - 0.0	12.0	3.9 - 24.5	0.80
3060107	6683	TURKEY CR	82.7	27.9	10 - 73	0.0	0.0 - 0.0	4.5	1.4 - 11.3	1.8	0.5 - 5.6	7.0	2.2 - 18.8	0.0	0.0 - 0.0	14.7	4.7 - 46.6	0.80
3060107	6684	TURKEY CR	99.7	22.2	9 - 54	0.0	0.0 - 0.0	4.2	1.6 - 12.2	1.5	0.6 - 4.5	7.1	2.8 - 23.1	0.0	0.0 - 0.0	9.4	3.9 - 25.3	0.77
3060107	6685	LITTLE STEVENS (	1.8	16.1	5 - 34	0.0	0.0 - 0.0	0.0	0.0 - 0.0	0.2	0.1 - 0.6	0.0	0.0 - 0.0	0.0	0.0 - 0.0	15.9	4.8 - 33.1	0.77
3060107	6686	LITTLE STEVENS (	6.5	19.2	7 - 50	0.0	0.0 - 0.0	2.1	0.6 - 5.5	0.8	0.2 - 2.3	1.2	0.4 - 3.7	0.0	0.0 - 0.0	15.1	5.6 - 40.5	0.76
3060107	6687	ROCKY CR	35.4	30.1	10 - 65	0.0	0.0 - 0.0	3.6	1.1 - 8.4	4.9	1.4 - 13.4	8.2	2.6 - 22.5	0.0	0.0 - 0.0	13.4	4.4 - 32.1	0.73
3060107	6688	LITTLE STEVENS (	72.7	29.7	9 - 55	0.0	0.0 - 0.0	3.5	1.0 - 6.3	4.6	1.3 - 10.0	8.0	2.1 - 19.0	0.0	0.0 - 0.0	13.5	4.2 - 29.3	0.73
3060107	6689	SLEEPY CR	71.8	24.2	8 - 48	0.0	0.0 - 0.0	3.4	1.0 - 7.9	2.7	0.7 - 7.6	4.8	1.6 - 12.6	0.0	0.0 - 0.0	13.3	4.5 - 30.7	0.76
3060107	6690	ROCKY CR	25.7	19.4	7 - 40	0.0	0.0 - 0.0	2.9	1.0 - 5.5	0.8	0.3 - 2.5	2.1	0.7 - 4.9	0.0	0.0 - 0.0	13.6	4.5 - 33.1	0.83
3060107	6691	STEVENS CR	96.4	20.2	7 - 44	0.0	0.0 - 0.0	5.0	1.6 - 12.1	0.8	0.3 - 2.1	3.2	1.1 - 8.9	0.0	0.0 - 0.0	11.2	4.1 - 27.5	0.86
3060107	6692	HARD LABOR CR	9.5	21.0	8 - 52	0.0	0.0 - 0.0	3.2	1.2 - 7.4	0.5	0.2 - 1.6	2.2	0.7 - 5.7	0.0	0.0 - 0.0	15.1	5.3 - 42.5	0.82
3060107	6693	CUFFYTOWN CR	240.5	25.7	10 - 71	0.0	0.0 - 0.0	5.2	1.8 - 13.7	1.8	0.6 - 5.8	5.9	2.2 - 17.2	0.0	0.0 - 0.0	12.9	4.7 - 39.3	0.81
3060107	6694	HARD LABOR CR	115.5	25.3	9 - 50	0.0	0.0 - 0.0	4.4	1.4 - 7.8	1.7	0.5 - 3.5	4.8	1.4 - 10.2	0.0	0.0 - 0.0	14.4	4.7 - 30.7	0.81
3060107	6695	HARD LABOR CR	5.4	22.4	8 - 46	0.0	0.0 - 0.0	4.2	1.4 - 8.3	1.6	0.5 - 4.1	3.4	1.2 - 7.5	0.0	0.0 - 0.0	13.1	5.0 - 29.5	0.71
3060107	6696	BIG COWHEAD CR	4.1	18.3	6 - 42	0.0	0.0 - 0.0	3.7	1.3 - 8.6	0.3	0.1 - 0.8	1.2	0.4 - 3.2	0.0	0.0 - 0.0	13.1	5.0 - 32.2	0.68
3060107	6697	BIG COWHEAD CR	24.1	26.2	9 - 53	0.0	0.0 - 0.0	7.0	1.9 - 15.6	1.6	0.5 - 3.8	6.3	1.6 - 14.5	0.0	0.0 - 0.0	11.2	3.7 - 23.1	0.67
3060107	6698	LITTLE COWHEAD	9.6	36.1	12 - 63	0.0	0.0 - 0.0	7.1	2.1 - 14.1	3.5	1.0 - 8.4	14.4	4.7 - 30.3	0.0	0.0 - 0.0	11.2	4.1 - 23.1	0.67
3060107	6699	HARD LABOR CR	57.7	74.6	28 - 175	34.2	12.4 - 82.1	18.1	6.7 - 44.5	2.4	0.7 - 6.9	8.9	3.0 - 23.9	0.0	0.0 - 0.0	10.9	4.1 - 28.8	0.68
3060107	6700	HARD LABOR CR,	20.1	18.8	6 - 36	0.0	0.0 - 0.0	4.6	1.4 - 8.1	1.0	0.3 - 2.4	2.2	0.8 - 4.7	0.0	0.0 - 0.0	11.0	3.4 - 25.5	0.71
3060107	6701	ROCKY CR	62.3	43.2	15 - 109	15.0	4.8 - 38.2	7.9	2.6 - 18.8	1.1	0.3 - 3.4	5.0	1.6 - 12.2	0.0	0.0 - 0.0	14.2	4.9 - 36.6	0.82
3060108	6702	BRIER CR	194.4	26.4	9 - 48	5.5	1.7 - 12.1	4.5	1.5 - 10.3	0.6	0.2 - 1.5	7.0	2.1 - 15.3	0.0	0.0 - 0.0	8.8	2.8 - 20.1	0.97
3060108	6703	BRIER CR	115.9	23.9	7 - 51	0.0	0.0 - 0.0	4.7	1.4 - 10.9	0.7	0.2 - 1.7	9.3	2.6 - 23.6	0.0	0.0 - 0.0	9.2	2.9 - 24.5	0.95
3060108	6704	BRIER CR	70.2	24.2	8 - 60	0.0	0.0 - 0.0	7.2	2.1 - 21.2	1.0	0.3 - 3.9	6.7	2.3 - 18.6	0.0	0.0 - 0.0	9.3	3.3 - 23.2	0.85
3060108	6705	BRIER CR	10.5	27.2	9 - 58	0.0	0.0 - 0.0	6.3	1.9 - 14.2	1.2	0.4 - 3.3	9.3	3.2 - 20.6	0.0	0.0 - 0.0	10.3	3.2 - 23.3	0.84
3060108	6706	SANDY RUN CR	93.2	17.4	5 - 40	0.0	0.0 - 0.0	7.6	2.3 - 17.5	0.2	0.1 - 0.7	2.3	0.6 - 6.1	0.0	0.0 - 0.0	7.2	2.3 - 17.3	0.83
3060108	6707	BRIER CR	4.4	18.2	6 - 50	0.0	0.0 - 0.0	2.4	0.8 - 7.4	0.6	0.2 - 1.9	5.6	1.8 - 14.9	0.0	0.0 - 0.0	9.5	3.3 - 28.9	0.83

8-digit HUC	ID	Name	Area	Catchment Yield		Point sources		Developed Land		Manure		Agricultural Land		Phosphate Mines		Soil parent rock		Frac
				kg/km^2	90% CI	kg/km^2	90% CI	kg/km^2	90% CI	kg/km^2	90% CI	kg/km^2	90% CI	kg/km^2	90% CI	kg/km^2	90% CI	
3060108	6708	BOGGY CUT CR	60.3	13.1	5 - 25	0.0	0.0 - 0.0	4.2	1.3 - 9.0	0.4	0.1 - 1.2	1.4	0.5 - 3.4	0.0	0.0 - 0.0	7.1	2.5 - 15.7	0.82
3060108	6709	BRIER CR	237.4	17.2	7 - 38	0.0	0.0 - 0.0	4.7	1.7 - 10.8	1.1	0.4 - 3.1	4.2	1.5 - 10.3	0.0	0.0 - 0.0	7.2	2.6 - 18.8	0.82
3060108	6710	SWEETWATER CR	65.0	18.5	6 - 33	0.0	0.0 - 0.0	5.0	1.5 - 9.9	1.4	0.4 - 3.8	5.9	1.7 - 14.1	0.0	0.0 - 0.0	6.2	2.1 - 13.6	0.72
3060108	6711	BRIER CR	145.3	23.7	8 - 45	0.0	0.0 - 0.0	7.2	2.1 - 15.6	1.4	0.4 - 5.0	5.8	1.6 - 14.4	0.0	0.0 - 0.0	9.3	3.0 - 22.6	0.72
3060108	6712	REEDY CR	147.4	18.4	6 - 35	0.0	0.0 - 0.0	5.0	1.5 - 12.8	0.8	0.2 - 2.2	4.9	1.4 - 12.9	0.0	0.0 - 0.0	7.7	2.3 - 16.5	0.84
3060108	6713	BRUSHY CR	161.1	26.4	8 - 49	0.0	0.0 - 0.0	7.9	2.5 - 16.1	1.1	0.3 - 3.0	8.9	2.7 - 22.1	0.0	0.0 - 0.0	8.5	2.7 - 17.4	0.85
3060108	6714	BEAVERDAM CR	335.4	16.7	7 - 30	0.0	0.0 - 0.0	3.5	1.2 - 6.1	0.6	0.2 - 1.3	6.1	2.4 - 14.4	0.0	0.0 - 0.0	6.5	2.5 - 13.2	0.95
3060109	6715	SAVANNAH R	64.4	0.2	0 - 2	0.0	0.0 - 0.0	0.0	0.0 - 0.0	0.0	0.0 - 0.0	0.0	0.0 - 0.1	0.0	0.0 - 0.0	0.2	0.0 - 1.5	1.00
3060109	6716	SAVANNAH R	4.3	6.9	1 - 22	0.0	0.0 - 0.0	1.1	0.2 - 4.1	0.0	0.0 - 0.1	0.2	0.0 - 0.6	0.0	0.0 - 0.0	5.7	1.2 - 18.4	1.00
3060109	6717	SAVANNAH R	112.5	0.9	0 - 3	0.1	0.0 - 0.3	0.0	0.0 - 0.1	0.0	0.0 - 0.0	0.0	0.0 - 0.2	0.0	0.0 - 0.0	0.7	0.1 - 3.0	0.99
3060109	6718	SAVANNAH R	207.8	9.5	3 - 22	3.4	1.2 - 8.2	0.9	0.2 - 2.1	0.1	0.0 - 0.3	0.6	0.2 - 1.7	0.0	0.0 - 0.0	4.5	1.2 - 10.8	0.99
3060109	6719	SAVANNAH R	33.6	1852.7	562 - 4641	1795.6	549.4 - 4460.1	34.3	9.7 - 67.5	0.1	0.0 - 0.4	0.6	0.2 - 1.5	0.0	0.0 - 0.0	22.2	6.8 - 59.8	0.99
3060109	6720	SAVANNAH R	12.6	2127.0	659 - 5058	2059.0	652.7 - 4897.[illegible]	39.8	10.1 - 97.4	0.4	0.1 - 1.5	2.9	0.8 - 7.9	0.0	0.0 - 0.0	24.9	7.0 - 71.0	0.99
3060109	6721	PIPEMAKERS CAN	175.9	63.0	23 - 137	19.4	7.2 - 55.5	25.9	9.1 - 59.0	0.5	0.2 - 1.4	3.9	1.6 - 11.1	0.0	0.0 - 0.0	13.4	4.5 - 33.5	0.99
3060109	6722	SAVANNAH R	62.8	338.0	124 - 708	308.0	112.4 - 662.1	3.2	1.0 - 6.3	0.4	0.1 - 1.0	3.0	1.0 - 7.1	0.0	0.0 - 0.0	23.4	7.8 - 60.6	0.99
3060109	6723	SAVANNAH R	8.2	27.1	7 - 71	0.0	0.0 - 0.0	2.3	0.6 - 5.0	0.2	0.1 - 0.8	0.9	0.2 - 2.6	0.0	0.0 - 0.0	23.7	6.3 - 65.7	0.98
3060109	6724	SAVANNAH R	1.5	24.2	8 - 61	0.0	0.0 - 0.0	0.0	0.0 - 0.0	0.1	0.0 - 0.4	0.5	0.2 - 1.5	0.0	0.0 - 0.0	23.6	7.9 - 59.6	0.98
3060109	6725	*A	299.5	30.8	10 - 60	0.0	0.0 - 0.0	2.8	0.8 - 5.5	0.3	0.1 - 0.8	4.0	1.3 - 8.3	0.0	0.0 - 0.0	23.7	7.7 - 50.5	0.98
3060109	6726	SAVANNAH R	83.1	26.4	10 - 60	0.0	0.0 - 0.0	1.6	0.6 - 3.6	0.6	0.2 - 2.3	7.4	2.5 - 19.3	0.0	0.0 - 0.0	16.8	5.5 - 43.8	0.98
3060109	6727	*B	209.6	34.8	12 - 64	0.0	0.0 - 0.0	3.5	1.1 - 6.9	0.3	0.1 - 0.8	9.3	3.1 - 24.4	0.0	0.0 - 0.0	21.6	8.1 - 49.5	0.98
3060109	6728	SAVANNAH R	90.1	24.3	8 - 55	0.0	0.0 - 0.0	1.1	0.4 - 2.5	0.4	0.1 - 1.2	4.0	1.4 - 9.8	0.0	0.0 - 0.0	18.7	6.4 - 47.8	0.98
3060109	6729	SAVANNAH R, DR	123.0	25.9	8 - 59	0.0	0.0 - 0.0	2.0	0.6 - 4.6	0.3	0.1 - 0.8	9.3	3.0 - 27.9	0.0	0.0 - 0.0	14.4	5.0 - 34.6	0.97
3060109	6730	SAVANNAH R	172.6	22.4	7 - 56	0.0	0.0 - 0.0	1.7	0.5 - 3.9	0.6	0.2 - 2.0	8.1	2.4 - 22.8	0.0	0.0 - 0.0	12.0	3.9 - 31.3	0.97
3060109	6731	EBENEZER CR	23.6	16.3	4 - 32	0.0	0.0 - 0.0	3.9	1.0 - 9.0	0.4	0.1 - 1.1	1.9	0.5 - 5.0	0.0	0.0 - 0.0	10.1	2.9 - 24.2	0.98
3060109	6732	EBENEZER CR, RU	250.4	29.1	8 - 64	0.0	0.0 - 0.0	3.4	0.9 - 7.6	1.0	0.3 - 3.1	11.2	2.9 - 26.0	0.0	0.0 - 0.0	13.6	3.8 - 37.3	0.90
3060109	6733	TURKEY CR	120.0	33.7	11 - 59	0.0	0.0 - 0.0	3.4	1.1 - 6.1	1.1	0.3 - 3.0	11.2	3.5 - 23.7	0.0	0.0 - 0.0	17.9	5.6 - 42.5	0.90
3060109	6734	LOCKNERS CR	65.8	15.5	6 - 31	0.0	0.0 - 0.0	5.2	2.0 - 10.7	0.4	0.1 - 1.0	2.2	0.9 - 5.9	0.0	0.0 - 0.0	7.7	3.1 - 17.4	0.98
3060109	6735	ABERCORN CR	23.7	5.9	2 - 13	0.0	0.0 - 0.0	0.6	0.2 - 1.2	0.1	0.0 - 0.2	0.6	0.2 - 1.7	0.0	0.0 - 0.0	4.6	1.4 - 10.9	0.99
3060109	6736	DASHERS CR	40.6	17.6	6 - 48	0.0	0.0 - 0.0	7.7	2.6 - 19.7	0.3	0.1 - 1.0	2.1	0.7 - 6.0	0.0	0.0 - 0.0	7.6	2.7 - 22.4	0.89
3060109	6737	ABERCORN CR	21.4	53.7	16 - 110	23.5	7.4 - 54.3	18.7	5.4 - 38.8	0.4	0.1 - 1.0	2.9	0.9 - 7.8	0.0	0.0 - 0.0	8.3	2.5 - 20.7	0.89
3060109	6738	SAVANNAH R, S C	17.7	5.3	1 - 17	0.0	0.0 - 0.0	0.2	0.0 - 0.8	0.0	0.0 - 0.0	0.1	0.0 - 0.3	0.0	0.0 - 0.0	5.0	1.1 - 17.4	1.00
3060201	6739	OGEECHEE R	2.8	52.9	19 - 113	0.0	0.0 - 0.0	3.2	1.0 - 6.4	2.6	0.7 - 7.7	16.0	5.2 - 41.0	0.0	0.0 - 0.0	31.0	10.9 - 77.8	0.90
3060201	6740	BUCKHEAD CR	374.2	22.6	8 - 44	0.0	0.0 - 0.0	5.3	1.9 - 11.3	1.0	0.3 - 2.6	9.4	3.3 - 22.6	0.0	0.0 - 0.0	7.0	2.5 - 15.7	0.90
3060201	6741	ROCKY CR	183.2	22.7	7 - 49	0.0	0.0 - 0.0	4.6	1.3 - 8.7	1.1	0.3 - 3.0	11.5	3.4 - 28.1	0.0	0.0 - 0.0	5.5	2.0 - 14.2	0.74
3060201	6742	BUCKHEAD CR	206.6	22.0	8 - 54	0.0	0.0 - 0.0	4.4	1.5 - 10.2	1.1	0.3 - 4.0	10.8	3.8 - 30.2	0.0	0.0 - 0.0	5.7	2.0 - 13.6	0.74
3060201	6743	OGEECHEE R	130.4	38.0	11 - 80	0.0	0.0 - 0.0	4.3	1.4 - 10.0	1.9	0.5 - 4.7	19.5	5.7 - 43.3	0.0	0.0 - 0.0	12.3	3.8 - 31.2	0.90
3060201	6744	CHEW MILL CR	60.1	25.9	10 - 61	0.0	0.0 - 0.0	2.9	1.0 - 6.4	1.4	0.4 - 4.3	14.7	5.7 - 39.1	0.0	0.0 - 0.0	6.9	2.4 - 17.8	0.89
3060201	6745	OGEECHEE R	11.5	65.3	19 - 165	0.0	0.0 - 0.0	2.9	0.8 - 6.8	3.4	0.9 - 10.6	36.7	11.7 - 91.7	0.0	0.0 - 0.0	22.4	5.9 - 66.3	0.89
3060201	6746	OGEECHEE R	8.1	65.4	23 - 132	0.0	0.0 - 0.0	5.1	1.7 - 11.1	4.2	1.0 - 11.4	28.4	9.5 - 61.7	0.0	0.0 - 0.0	27.7	8.6 - 62.7	0.88

8-digit HUC	ID	Name	Area	Catchment Yield		Point sources		Developed Land		Manure		Agricultural Land		Phosphate Mines		Soil parent rock		Frac
				kg/km^2	90% CI	kg/km^2	90% CI	kg/km^2	90% CI	kg/km^2	90% CI	kg/km^2	90% CI	kg/km^2	90% CI	kg/km^2	90% CI	
3060201	6747	MILL CR	96.9	23.1	8 - 53	0.0	0.0 - 0.0	3.4	1.1 - 7.6	1.2	0.4 - 3.1	12.0	4.1 - 30.5	0.0	0.0 - 0.0	6.5	2.2 - 15.4	0.88
3060201	6748	OGEECHEE R	13.1	29.3	9 - 69	0.0	0.0 - 0.0	4.2	1.3 - 9.9	1.3	0.4 - 3.4	10.0	3.2 - 25.6	0.0	0.0 - 0.0	13.7	4.1 - 36.1	0.88
3060201	6749	OGEECHEE R	45.2	34.0	10 - 65	0.0	0.0 - 0.0	8.8	2.5 - 17.8	1.4	0.4 - 3.4	13.1	4.5 - 29.9	0.0	0.0 - 0.0	10.6	3.4 - 21.5	0.88
3060201	6750	OGEECHEE R	12.4	29.7	10 - 55	0.0	0.0 - 0.0	5.2	1.7 - 10.5	1.6	0.6 - 4.7	11.9	4.1 - 25.8	0.0	0.0 - 0.0	11.1	3.9 - 27.9	0.88
3060201	6751	OGEECHEE R, BUL	51.1	20.3	7 - 52	0.0	0.0 - 0.0	4.3	1.4 - 10.5	0.9	0.3 - 2.4	9.6	3.3 - 26.2	0.0	0.0 - 0.0	5.4	1.8 - 13.3	0.87
3060201	6752	OGEECHEE R	12.4	16.7	5 - 38	0.0	0.0 - 0.0	1.9	0.6 - 4.4	0.8	0.2 - 2.6	5.8	2.2 - 14.9	0.0	0.0 - 0.0	8.3	2.6 - 20.4	0.87
3060201	6753	OGEECHEE R	179.1	25.3	7 - 53	0.0	0.0 - 0.0	4.6	1.2 - 8.9	1.3	0.3 - 4.6	12.1	3.8 - 33.7	0.0	0.0 - 0.0	7.3	2.2 - 17.4	0.87
3060201	6754	BIG CR	259.6	25.5	7 - 54	0.0	0.0 - 0.0	4.5	1.2 - 11.6	1.1	0.3 - 3.3	9.4	2.8 - 21.5	0.0	0.0 - 0.0	10.5	2.7 - 26.4	0.86
3060201	6755	OGEECHEE R	34.6	30.2	10 - 71	0.0	0.0 - 0.0	10.7	3.2 - 26.4	1.0	0.3 - 2.5	8.2	2.5 - 21.3	0.0	0.0 - 0.0	10.3	3.0 - 25.8	0.86
3060201	6756	DUHART CR	191.2	24.3	8 - 47	0.0	0.0 - 0.0	5.1	1.5 - 9.9	1.1	0.3 - 2.5	9.6	2.8 - 23.3	0.0	0.0 - 0.0	8.5	3.1 - 18.4	0.85
3060201	6757	OGEECHEE R	60.4	28.9	11 - 69	0.0	0.0 - 0.0	5.2	1.7 - 14.4	1.3	0.4 - 3.3	11.2	4.2 - 29.7	0.0	0.0 - 0.0	11.1	3.7 - 31.5	0.85
3060201	6758	ROCKY COMFORT	124.1	19.1	7 - 38	0.0	0.0 - 0.0	3.0	1.0 - 6.7	0.8	0.3 - 2.0	6.7	2.3 - 16.4	0.0	0.0 - 0.0	8.5	3.1 - 19.7	0.84
3060201	6759	ROCKY COMFORT	6.6	17.7	5 - 33	0.0	0.0 - 0.0	2.2	0.7 - 4.4	1.0	0.3 - 2.5	3.2	0.9 - 7.7	0.0	0.0 - 0.0	11.4	3.4 - 24.5	0.77
3060201	6760	DEEP CR	70.4	16.4	6 - 32	0.0	0.0 - 0.0	3.9	1.5 - 8.5	0.8	0.3 - 2.2	5.0	2.1 - 11.8	0.0	0.0 - 0.0	6.6	2.4 - 15.6	0.76
3060201	6761	ROCKY COMFORT	219.1	20.6	8 - 51	0.0	0.0 - 0.0	4.8	1.7 - 12.9	1.2	0.4 - 4.3	6.3	2.6 - 16.9	0.0	0.0 - 0.0	8.3	3.4 - 22.9	0.76
3060201	6762	GOLDEN'S CR	21.9	25.7	9 - 59	0.0	0.0 - 0.0	7.3	2.1 - 17.2	1.3	0.4 - 3.8	5.7	1.9 - 16.3	0.0	0.0 - 0.0	11.4	3.9 - 28.3	0.63
3060201	6763	ROCKY COMFORT	47.4	16.8	5 - 29	0.0	0.0 - 0.0	5.0	1.7 - 8.6	0.8	0.2 - 1.7	2.2	0.6 - 5.3	0.0	0.0 - 0.0	8.9	2.9 - 18.2	0.63
3060201	6764	JOE'S CR	71.4	13.1	4 - 33	0.0	0.0 - 0.0	3.2	1.0 - 6.9	0.7	0.2 - 1.8	3.8	1.0 - 11.0	0.0	0.0 - 0.0	5.5	1.6 - 14.1	0.77
3060201	6765	OGEECHEE R	180.9	19.1	7 - 48	0.0	0.0 - 0.0	1.7	0.5 - 4.7	0.7	0.2 - 2.3	5.5	1.6 - 14.1	0.0	0.0 - 0.0	11.1	3.5 - 28.5	0.84
3060201	6766	BIG CR	37.1	15.3	4 - 36	0.0	0.0 - 0.0	2.9	0.8 - 6.6	0.8	0.2 - 2.5	4.8	1.5 - 11.7	0.0	0.0 - 0.0	6.8	2.1 - 17.1	0.80
3060201	6768	OGEECHEE R	8.5	24.2	8 - 52	0.0	0.0 - 0.0	0.3	0.1 - 0.6	0.4	0.1 - 0.8	0.4	0.1 - 1.1	0.0	0.0 - 0.0	23.1	7.9 - 50.2	0.80
3060201	6769	OGEECHEE R	23.9	21.3	8 - 51	0.0	0.0 - 0.0	2.6	0.9 - 5.7	0.8	0.3 - 2.2	3.9	1.5 - 10.9	0.0	0.0 - 0.0	14.0	4.9 - 34.0	0.79
3060201	6770	OGEECHEE R	129.7	16.7	4 - 39	0.0	0.0 - 0.0	3.5	1.0 - 8.0	0.6	0.2 - 1.8	4.1	0.9 - 11.1	0.0	0.0 - 0.0	8.4	2.2 - 19.8	0.78
3060201	6771	LONG CR	98.1	19.6	8 - 50	0.0	0.0 - 0.0	3.8	1.5 - 9.2	1.3	0.4 - 3.7	4.7	1.8 - 13.0	0.0	0.0 - 0.0	9.8	3.7 - 27.6	0.75
3060201	6772	OGEECHEE R	13.2	20.9	6 - 39	0.0	0.0 - 0.0	3.9	1.2 - 8.4	1.2	0.3 - 3.3	5.4	1.6 - 14.3	0.0	0.0 - 0.0	10.3	3.2 - 24.6	0.75
3060201	6773	OGEECHEE R	129.2	20.9	6 - 62	0.0	0.0 - 0.0	3.8	1.1 - 9.9	0.7	0.2 - 2.3	1.7	0.5 - 5.3	0.0	0.0 - 0.0	14.6	4.3 - 45.1	0.74
3060201	6774	OGEECHEE R	91.4	25.9	8 - 51	0.0	0.0 - 0.0	6.0	1.8 - 11.5	1.2	0.3 - 3.1	3.2	1.0 - 7.7	0.0	0.0 - 0.0	15.5	5.1 - 35.7	0.70
3060201	6775	OGEECHEE R, N FI	77.9	23.3	6 - 53	0.0	0.0 - 0.0	8.6	2.1 - 18.5	1.8	0.4 - 4.7	3.3	1.0 - 7.4	0.0	0.0 - 0.0	9.7	2.7 - 25.1	0.66
3060201	6776	OGEECHEE R, S FK	81.5	23.6	7 - 64	0.0	0.0 - 0.0	4.9	1.2 - 14.8	3.2	0.9 - 12.5	5.4	1.6 - 16.9	0.0	0.0 - 0.0	10.2	2.9 - 29.8	0.66
3060201	6777	POWELL CR	60.2	22.0	9 - 49	0.0	0.0 - 0.0	3.8	1.4 - 8.4	1.2	0.5 - 3.8	3.3	1.4 - 8.1	0.0	0.0 - 0.0	13.6	5.8 - 35.1	0.70
3060201	6778	FULSOME CR	64.3	17.9	6 - 41	0.0	0.0 - 0.0	3.5	1.2 - 8.8	0.4	0.1 - 1.1	2.3	0.9 - 6.5	0.0	0.0 - 0.0	11.7	3.8 - 27.1	0.74
3060201	6780	LITTLE OGEECHEI	151.5	14.0	4 - 25	0.0	0.0 - 0.0	3.0	0.9 - 7.0	0.4	0.1 - 1.2	3.3	0.9 - 8.8	0.0	0.0 - 0.0	7.3	2.3 - 19.0	0.78
3060201	6781	COWPEN CR	48.6	10.4	5 - 21	0.0	0.0 - 0.0	1.4	0.5 - 2.8	0.4	0.1 - 1.1	2.5	0.9 - 6.1	0.0	0.0 - 0.0	6.1	2.5 - 13.6	0.78
3060201	6782	FLOYD CR	19.5	12.9	5 - 27	0.0	0.0 - 0.0	2.1	0.7 - 4.6	0.6	0.2 - 1.5	2.4	0.9 - 6.6	0.0	0.0 - 0.0	7.8	2.7 - 18.7	0.79
3060201	6783	STEPHENS CR	41.0	10.9	4 - 26	0.0	0.0 - 0.0	1.2	0.4 - 2.7	0.4	0.1 - 1.3	1.7	0.5 - 4.8	0.0	0.0 - 0.0	7.6	2.5 - 21.6	0.80
3060201	6785	WILLIAMSON SWA	382.1	22.4	8 - 59	0.0	0.0 - 0.0	3.9	1.2 - 8.7	1.1	0.3 - 3.4	9.3	2.8 - 26.5	0.0	0.0 - 0.0	8.1	2.5 - 21.5	0.86
3060201	6786	ROCKY CR	68.1	13.9	5 - 33	0.0	0.0 - 0.0	3.3	1.1 - 7.6	0.6	0.2 - 2.0	4.9	1.5 - 13.0	0.0	0.0 - 0.0	5.1	1.7 - 12.4	0.86
3060201	6787	DANIEL'S CR	109.6	14.5	5 - 36	0.0	0.0 - 0.0	3.3	1.2 - 7.8	0.5	0.2 - 1.6	3.2	1.2 - 8.9	0.0	0.0 - 0.0	7.4	2.6 - 17.4	0.88
3060201	6788	BIG LONG CR	102.1	17.7	7 - 49	0.0	0.0 - 0.0	3.7	1.4 - 8.5	0.7	0.2 - 2.3	6.5	2.3 - 20.8	0.0	0.0 - 0.0	6.9	2.5 - 18.5	0.88

8-digit HUC	ID	Name	Area	Catchment Yield		Point sources		Developed Land		Manure		Agricultural Land		Phosphate Mines		Soil parent rock		Frac
				kg/km2	90% CI	kg/km2	90% CI	kg/km2	90% CI	kg/km2	90% CI	kg/km2	90% CI	kg/km2	90% CI	kg/km2	90% CI	
3060201	6789	DEEP CR	93.2	19.7	6 - 34	0.0	0.0 - 0.0	3.1	0.8 - 6.0	0.8	0.2 - 2.3	8.0	2.3 - 19.6	0.0	0.0 - 0.0	7.8	2.1 - 15.3	0.88
3060202	6790	OGEECHEE R	224.1	29.0	9 - 63	11.4	3.6 - 23.1	2.2	0.6 - 5.1	0.0	0.0 - 0.1	0.3	0.1 - 0.7	0.0	0.0 - 0.0	15.1	4.5 - 37.7	1.00
3060202	6791	OGEECHEE R	137.1	17.0	6 - 33	0.0	0.0 - 0.0	4.5	1.3 - 10.0	0.1	0.0 - 0.3	0.4	0.1 - 1.0	0.0	0.0 - 0.0	11.9	4.5 - 27.5	0.98
3060202	6793	OGEECHEE R	49.2	21.7	8 - 52	0.0	0.0 - 0.0	2.6	1.0 - 5.8	0.9	0.3 - 2.3	8.1	2.9 - 22.7	0.0	0.0 - 0.0	10.1	3.6 - 27.7	0.94
3060202	6794	OGEECHEE CR	213.2	28.2	10 - 69	0.0	0.0 - 0.1	4.0	1.3 - 11.4	0.8	0.3 - 2.6	12.1	4.3 - 34.0	0.0	0.0 - 0.0	11.3	3.6 - 28.6	0.93
3060202	6795	OGEECHEE CR	109.1	18.0	6 - 40	0.0	0.0 - 0.0	5.0	1.8 - 9.7	0.4	0.1 - 1.1	5.8	1.6 - 14.7	0.0	0.0 - 0.0	6.7	2.2 - 14.9	0.71
3060202	6796	OGEECHEE CR, S F	79.7	21.6	7 - 43	0.0	0.0 - 0.0	3.6	1.1 - 7.5	0.6	0.2 - 1.9	10.4	3.0 - 29.4	0.0	0.0 - 0.0	7.0	2.4 - 16.6	0.71
3060202	6797	OGEECHEE R	135.2	34.4	11 - 83	0.0	0.0 - 0.0	3.5	1.0 - 8.0	1.2	0.3 - 4.3	15.9	5.1 - 46.2	0.0	0.0 - 0.0	13.8	3.9 - 33.2	0.93
3060202	6798	OGEECHEE R, HUN	32.6	23.1	8 - 46	0.0	0.0 - 0.0	3.5	1.2 - 7.7	0.7	0.2 - 1.6	11.3	4.1 - 30.0	0.0	0.0 - 0.0	7.6	2.5 - 16.7	0.92
3060202	6799	OGEECHEE R	68.1	38.0	14 - 68	0.0	0.0 - 0.0	6.7	2.3 - 12.7	1.6	0.5 - 5.0	16.6	5.6 - 45.0	0.0	0.0 - 0.0	13.2	4.4 - 32.1	0.92
3060202	6800	OGEECHEE R, JAC	33.9	23.3	8 - 55	0.0	0.0 - 0.0	3.5	1.2 - 7.5	0.7	0.2 - 1.9	11.9	3.9 - 28.6	0.0	0.0 - 0.0	7.2	2.3 - 17.7	0.92
3060202	6801	OGEECHEE R	20.2	32.7	11 - 57	0.0	0.0 - 0.0	3.2	1.2 - 5.8	1.1	0.3 - 2.9	11.6	4.2 - 24.3	0.0	0.0 - 0.0	16.8	5.2 - 35.8	0.92
3060202	6802	OGEECHEE R	55.6	31.4	10 - 76	0.0	0.0 - 0.0	3.6	1.1 - 8.9	1.0	0.2 - 3.1	14.8	4.9 - 47.1	0.0	0.0 - 0.0	12.0	3.8 - 30.5	0.92
3060202	6803	HORSE CR	228.0	15.8	6 - 32	0.0	0.0 - 0.0	2.9	1.0 - 6.1	0.6	0.2 - 1.3	6.1	2.4 - 14.5	0.0	0.0 - 0.0	6.3	2.1 - 13.0	0.91
3060202	6804	OGEECHEE R	69.2	28.6	9 - 65	0.0	0.0 - 0.0	3.1	1.0 - 6.9	1.1	0.3 - 3.3	9.2	2.9 - 26.9	0.0	0.0 - 0.0	15.3	4.2 - 35.1	0.91
3060202	6805	OGEECHEE R	8.6	27.3	10 - 53	0.0	0.0 - 0.0	4.2	1.6 - 7.8	1.2	0.4 - 3.1	7.3	2.5 - 16.7	0.0	0.0 - 0.0	14.6	4.6 - 31.4	0.90
3060202	6806	SCULLS CR	188.2	23.1	6 - 51	0.0	0.0 - 0.0	4.1	1.0 - 9.1	1.0	0.2 - 2.9	10.1	2.4 - 25.6	0.0	0.0 - 0.0	7.9	1.9 - 20.3	0.90
3060202	6807	BAY GALL CR	6.4	29.0	10 - 73	0.0	0.0 - 0.0	4.2	1.3 - 9.8	1.5	0.4 - 4.7	15.0	4.3 - 42.6	0.0	0.0 - 0.0	8.3	2.9 - 20.3	0.90
3060202	6808	BAY GALL CR	62.3	19.5	7 - 35	0.0	0.0 - 0.0	3.3	1.1 - 6.3	0.9	0.3 - 2.4	8.9	2.9 - 21.0	0.0	0.0 - 0.0	6.4	2.4 - 13.6	0.82
3060202	6809	WYATTS CR	67.2	20.7	7 - 49	0.0	0.0 - 0.0	5.2	1.7 - 12.7	0.9	0.3 - 3.1	8.0	2.5 - 20.2	0.0	0.0 - 0.0	6.5	2.3 - 17.4	0.82
3060202	6810	COLEMAN CR	19.2	27.4	10 - 55	0.0	0.0 - 0.0	5.4	1.9 - 11.4	1.4	0.4 - 4.1	13.1	4.8 - 31.6	0.0	0.0 - 0.0	7.5	2.4 - 16.1	0.90
3060202	6811	MILL CR	18.2	25.0	10 - 43	0.0	0.0 - 0.0	4.7	1.8 - 8.3	1.4	0.4 - 3.2	12.6	4.7 - 25.1	0.0	0.0 - 0.0	6.3	2.3 - 13.3	0.94
3060202	6812	SPRING CR	44.1	61.1	20 - 116	0.0	0.0 - 0.0	5.0	1.5 - 10.1	3.6	1.0 - 9.4	33.8	10.2 - 76.0	0.0	0.0 - 0.0	18.8	6.0 - 38.1	0.90
3060202	6813	MILL CR	238.9	37.8	13 - 96	0.0	0.0 - 0.1	9.9	3.1 - 22.5	1.7	0.5 - 5.3	16.1	5.0 - 43.4	0.0	0.0 - 0.0	10.1	3.2 - 27.7	0.90
3060202	6814	BLACK CR	18.5	20.0	7 - 45	0.0	0.0 - 0.0	5.4	1.8 - 11.6	0.2	0.1 - 0.5	3.6	1.2 - 10.5	0.0	0.0 - 0.0	10.8	3.5 - 27.0	0.96
3060202	6815	BLACK CR	22.0	30.5	9 - 56	0.0	0.0 - 0.0	11.6	3.3 - 23.1	0.2	0.1 - 0.6	6.1	2.0 - 15.2	0.0	0.0 - 0.0	12.6	3.7 - 32.0	0.95
3060202	6816	UPPER BLACK CR	13.1	23.8	9 - 47	0.0	0.0 - 0.0	2.4	0.8 - 5.5	1.2	0.4 - 3.0	8.6	3.1 - 23.4	0.0	0.0 - 0.0	11.6	4.6 - 27.5	0.91
3060202	6817	UPPER BLACK CR,	42.6	39.5	12 - 96	0.0	0.0 - 0.0	6.0	1.7 - 13.0	1.8	0.5 - 5.1	15.9	5.8 - 42.1	0.0	0.0 - 0.0	15.8	5.3 - 46.0	0.89
3060202	6819	UPPER BLACK CR,	64.0	36.2	12 - 67	0.0	0.0 - 0.0	2.8	0.9 - 5.5	1.9	0.6 - 5.3	16.2	5.4 - 35.1	0.0	0.0 - 0.0	15.4	5.2 - 35.7	0.88
3060202	6820	UPPER BLACK CR	102.3	35.8	12 - 81	0.0	0.0 - 0.0	5.3	1.7 - 11.7	2.1	0.6 - 4.6	18.5	6.4 - 49.2	0.0	0.0 - 0.0	10.0	3.0 - 25.5	0.88
3060202	6821	LOWER BLACK CR	2.4	60.4	23 - 118	0.0	0.0 - 0.0	13.4	4.6 - 28.7	3.1	0.9 - 8.6	28.4	11.0 - 62.6	0.0	0.0 - 0.0	15.5	5.6 - 37.1	0.91
3060202	6822	LOWER BLACK CR	25.3	42.3	15 - 106	0.0	0.0 - 0.0	9.7	3.2 - 24.3	1.9	0.6 - 5.3	15.4	5.0 - 38.9	0.0	0.0 - 0.0	15.4	5.5 - 38.8	0.90
3060202	6823	LOWER BLACK CR	134.9	36.4	11 - 85	0.0	0.0 - 0.0	6.6	2.1 - 13.6	2.0	0.5 - 5.9	19.3	6.0 - 53.9	0.0	0.0 - 0.0	8.6	2.5 - 20.6	0.84
3060202	6824	LOWER BLACK CR	64.8	14.7	5 - 39	0.0	0.0 - 0.0	2.4	0.8 - 6.1	0.6	0.2 - 1.8	4.5	1.5 - 13.6	0.0	0.0 - 0.0	7.1	2.6 - 19.3	0.84
3060202	6825	LOWER BLACK CR	219.4	28.0	9 - 68	1.2	0.4 - 3.3	5.5	1.8 - 12.2	0.4	0.1 - 1.3	5.1	1.7 - 15.6	0.0	0.0 - 0.0	15.8	4.9 - 44.9	0.90
3060202	6826	MILL CR	64.4	32.9	13 - 62	0.0	0.0 - 0.0	8.0	2.8 - 16.7	0.3	0.1 - 0.9	10.0	4.1 - 22.1	0.0	0.0 - 0.0	14.6	5.1 - 31.8	0.95
3060202	6827	BAY GALL CR	1.4	42.1	14 - 101	0.0	0.0 - 0.0	1.4	0.4 - 3.0	1.8	0.5 - 5.4	14.1	4.5 - 38.0	0.0	0.0 - 0.0	24.8	7.6 - 67.3	0.92
3060203	6828	CANOOCHEE R	321.9	21.7	7 - 44	0.0	0.0 - 0.0	3.5	1.1 - 6.7	0.1	0.0 - 0.3	0.4	0.1 - 0.8	0.0	0.0 - 0.0	17.7	5.5 - 41.4	0.98
3060203	6829	CANOOCHEE R	219.3	21.7	7 - 55	0.0	0.0 - 0.0	4.3	1.1 - 9.3	0.4	0.1 - 1.3	1.4	0.4 - 3.3	0.0	0.0 - 0.0	15.6	4.7 - 44.2	0.95

8-digit HUC	ID	Name	Area	Catchment Yield		Point sources		Developed Land		Manure		Agricultural Land		Phosphate Mines		Soil parent rock		Frac
				kg/km^2	90% CI	kg/km^2	90% CI	kg/km^2	90% CI	kg/km^2	90% CI	kg/km^2	90% CI	kg/km^2	90% CI	kg/km^2	90% CI	
3060203	6830	CANOOCHEE R	4.9	31.3	12 - 70	0.0	0.0 - 0.0	12.3	4.3 - 25.9	1.0	0.3 - 2.4	7.3	2.6 - 19.4	0.0	0.0 - 0.0	10.7	4.1 - 26.2	0.92
3060203	6831	LOTTS R	17.5	18.7	7 - 39	0.0	0.0 - 0.0	2.1	0.7 - 4.5	1.0	0.3 - 2.6	4.0	1.4 - 10.0	0.0	0.0 - 0.0	11.5	4.1 - 27.0	0.91
3060203	6832	LITTLE LOTTS R	22.1	24.2	8 - 54	0.0	0.0 - 0.0	3.5	1.1 - 9.0	1.4	0.4 - 4.3	11.3	3.1 - 34.0	0.0	0.0 - 0.0	8.0	2.5 - 21.8	0.90
3060203	6833	LOTTS R	624.9	41.5	14 - 78	8.5	2.9 - 17.6	9.4	3.0 - 15.8	1.7	0.5 - 4.2	13.4	4.3 - 30.3	0.0	0.0 - 0.0	8.4	2.9 - 15.8	0.90
3060203	6834	CANOOCHEE R	116.3	35.8	12 - 95	0.0	0.0 - 0.0	11.2	3.8 - 29.0	4.6	1.5 - 13.9	9.6	3.6 - 30.4	0.0	0.0 - 0.0	10.4	3.4 - 28.3	0.91
3060203	6835	CANOOCHEE R	249.8	32.0	12 - 65	0.0	0.0 - 0.0	5.2	1.7 - 11.2	3.8	1.2 - 11.5	13.9	5.0 - 32.3	0.0	0.0 - 0.0	9.1	3.1 - 22.0	0.89
3060203	6836	CANOOCHEE R	5.1	23.1	8 - 46	0.0	0.0 - 0.0	0.2	0.0 - 0.3	1.2	0.4 - 3.3	10.0	3.5 - 25.1	0.0	0.0 - 0.0	11.7	3.4 - 25.7	0.85
3060203	6837	FIFTEENMILE CR	402.9	26.3	10 - 50	0.0	0.0 - 0.0	6.5	2.2 - 14.9	1.1	0.4 - 3.0	11.1	4.3 - 24.1	0.0	0.0 - 0.0	7.6	2.4 - 17.3	0.84
3060203	6838	CANOOCHEE R	545.6	22.0	8 - 57	0.0	0.0 - 0.0	5.9	2.2 - 17.1	0.9	0.3 - 2.9	7.6	2.7 - 21.2	0.0	0.0 - 0.0	7.6	2.7 - 21.3	0.84
3060203	6839	WOLFE CR	62.3	25.3	9 - 65	0.0	0.0 - 0.0	4.9	1.6 - 11.3	1.4	0.4 - 4.2	10.3	3.5 - 30.1	0.0	0.0 - 0.0	8.6	2.6 - 23.0	0.85
3060203	6840	CEDAR CR	154.4	37.1	12 - 71	0.6	0.2 - 1.2	6.8	2.5 - 15.9	9.3	2.6 - 23.3	12.3	4.3 - 30.1	0.0	0.0 - 0.0	8.1	3.0 - 18.4	0.89
3060203	6841	BULL CR	151.6	37.7	12 - 91	0.0	0.0 - 0.0	7.9	2.2 - 17.6	7.4	2.3 - 24.2	13.4	4.3 - 37.3	0.0	0.0 - 0.0	9.0	3.0 - 24.2	0.92
3060203	6842	CANOOCHEE CR	28.4	21.2	7 - 46	0.0	0.0 - 0.0	5.5	2.0 - 11.5	0.3	0.1 - 0.9	0.0	0.0 - 0.0	0.0	0.0 - 0.0	15.4	5.2 - 34.3	0.95
3060203	6843	CANOOCHEE CR	300.9	21.8	7 - 43	0.0	0.0 - 0.0	4.7	1.6 - 8.9	1.6	0.5 - 4.0	2.7	0.9 - 5.5	0.0	0.0 - 0.0	12.9	3.8 - 29.4	0.92
3060203	6844	TAYLOR'S CR	307.1	59.1	21 - 110	30.1	10.9 - 63.6	9.9	3.1 - 20.8	0.9	0.3 - 2.6	3.7	1.2 - 9.3	0.0	0.0 - 0.0	14.6	5.2 - 36.5	0.92
3060204	6845	JERICO R	194.1	18.1	4 - 41	0.0	0.0 - 0.0	1.9	0.6 - 4.2	0.2	0.0 - 0.6	0.9	0.2 - 2.2	0.0	0.0 - 0.0	15.2	3.6 - 36.2	0.99
3060204	6846	JONES CR	37.4	11.3	3 - 31	0.0	0.0 - 0.0	0.3	0.1 - 0.9	0.0	0.0 - 0.1	0.0	0.0 - 0.1	0.0	0.0 - 0.0	10.9	2.7 - 30.5	0.99
3060204	6847	N NEWPORT R	578.0	24.3	9 - 55	0.1	0.1 - 0.3	4.2	1.3 - 8.6	0.5	0.1 - 1.6	1.1	0.4 - 3.6	0.0	0.0 - 0.0	18.3	7.3 - 42.9	1.00
3060204	6848	CATHEAD CR	333.5	13.7	4 - 28	0.9	0.3 - 1.7	1.4	0.4 - 3.1	0.1	0.0 - 0.2	0.4	0.1 - 0.9	0.0	0.0 - 0.0	11.0	3.7 - 24.3	1.00
3070101	6849	FORD CR	89.1	18.3	6 - 33	0.0	0.0 - 0.0	3.5	1.1 - 6.9	0.4	0.1 - 1.1	2.1	0.7 - 5.5	0.0	0.0 - 0.0	12.3	4.2 - 27.8	0.54
3070101	6850	NEEL CR	3.8	19.6	6 - 48	0.0	0.0 - 0.0	6.8	1.8 - 15.9	0.1	0.0 - 0.3	0.5	0.2 - 1.6	0.0	0.0 - 0.0	12.2	3.5 - 32.5	0.54
3070101	6851	NEEL CR	5.2	20.6	7 - 41	0.0	0.0 - 0.0	7.2	2.3 - 15.0	0.2	0.1 - 0.5	1.3	0.4 - 3.1	0.0	0.0 - 0.0	11.9	4.0 - 29.1	0.53
3070101	6852	NEEL CR	5.4	15.3	5 - 34	0.0	0.0 - 0.0	2.8	0.9 - 6.4	0.2	0.0 - 0.5	2.3	0.8 - 5.6	0.0	0.0 - 0.0	10.0	3.4 - 24.0	0.52
3070101	6853	NEEL CR	85.8	19.2	6 - 40	0.0	0.0 - 0.0	4.0	1.1 - 9.1	0.5	0.1 - 1.2	2.8	0.8 - 6.9	0.0	0.0 - 0.0	11.9	3.7 - 29.9	0.51
3070101	6854	WHITTEN CR	64.3	16.7	6 - 43	0.0	0.0 - 0.0	2.8	0.9 - 7.0	0.4	0.2 - 1.3	2.1	0.7 - 7.0	0.0	0.0 - 0.0	11.3	4.0 - 31.5	0.51
3070101	6855	LUNDY CR	111.8	23.1	7 - 40	0.0	0.0 - 0.0	4.0	1.3 - 8.3	2.6	0.6 - 6.1	4.8	1.3 - 12.5	0.0	0.0 - 0.0	11.7	3.4 - 26.3	0.52
3070101	6856	KIMBRO CR	47.3	19.9	6 - 38	0.0	0.0 - 0.0	3.8	1.2 - 8.5	1.9	0.6 - 4.9	2.3	0.7 - 6.2	0.0	0.0 - 0.0	11.9	3.7 - 24.6	0.53
3070101	6857	RICHLAND CR	61.7	34.9	12 - 92	0.0	0.0 - 0.0	8.7	3.0 - 23.0	3.3	1.0 - 9.1	3.7	1.1 - 10.2	0.0	0.0 - 0.0	19.1	6.3 - 50.1	0.26
3070101	6858	OLIVER CR	18.3	32.5	10 - 73	0.0	0.0 - 0.0	8.8	2.9 - 20.0	3.8	1.0 - 11.3	3.8	1.1 - 11.5	0.0	0.0 - 0.0	16.0	4.8 - 44.5	0.25
3070101	6859	STEWART CR	27.1	25.7	10 - 60	0.0	0.0 - 0.0	4.1	1.5 - 8.9	3.9	1.3 - 10.1	5.3	2.1 - 15.8	0.0	0.0 - 0.0	12.4	4.6 - 31.7	0.24
3070101	6860	OLIVER CR	77.3	30.2	9 - 76	0.0	0.0 - 0.0	7.1	2.3 - 17.1	4.4	1.1 - 15.3	6.1	1.9 - 15.2	0.0	0.0 - 0.0	12.6	4.2 - 31.7	0.24
3070101	6861	RICHLAND CR	123.3	31.6	11 - 67	2.2	0.8 - 5.2	7.4	2.6 - 17.1	3.9	1.1 - 10.9	5.8	2.0 - 15.5	0.0	0.0 - 0.0	12.2	4.5 - 28.4	0.25
3070101	6862	OCONEE R	41.1	33.6	12 - 68	0.0	0.0 - 0.0	12.2	4.1 - 25.0	1.6	0.4 - 4.8	2.3	0.8 - 4.8	0.0	0.0 - 0.0	17.6	6.0 - 38.3	0.26
3070101	6863	OCONEE R	27.0	40.5	14 - 69	0.0	0.0 - 0.0	14.4	5.0 - 27.6	2.4	0.7 - 5.6	4.1	1.3 - 9.5	0.0	0.0 - 0.0	19.6	6.4 - 40.4	0.26
3070101	6864	OCONEE R	36.9	31.6	10 - 65	0.0	0.0 - 0.0	6.0	2.0 - 12.8	3.7	1.0 - 10.5	4.5	1.4 - 11.1	0.0	0.0 - 0.0	17.3	5.4 - 40.0	0.26
3070101	6865	OCONEE R	28.1	32.8	10 - 67	0.0	0.0 - 0.0	6.1	1.7 - 12.3	4.7	1.2 - 12.7	6.8	2.4 - 15.3	0.0	0.0 - 0.0	15.2	4.8 - 39.6	0.26
3070101	6866	TOWN CR	76.8	19.9	7 - 39	0.0	0.0 - 0.0	3.4	1.3 - 7.1	2.4	0.8 - 7.3	3.1	1.2 - 8.3	0.0	0.0 - 0.0	11.0	4.1 - 23.6	0.25
3070101	6867	OCONEE R	16.5	31.2	10 - 61	0.0	0.0 - 0.0	2.9	0.9 - 5.9	4.1	1.2 - 11.6	6.3	2.1 - 15.1	0.0	0.0 - 0.0	17.9	6.2 - 37.1	0.25
3070101	6868	OCONEE R	11.0	23.0	7 - 45	0.0	0.0 - 0.0	2.2	0.7 - 5.0	2.3	0.6 - 5.9	3.7	1.1 - 8.6	0.0	0.0 - 0.0	14.8	4.5 - 33.3	0.25

8-digit HUC	ID	Name	Area	Catchment Yield		Point sources		Developed Land		Manure		Agricultural Land		Phosphate Mines		Soil parent rock		Frac
				kg/km^2	90% CI	kg/km^2	90% CI	kg/km^2	90% CI	kg/km^2	90% CI	kg/km^2	90% CI	kg/km^2	90% CI	kg/km^2	90% CI	
3070101	6869	FISHING CR	101.8	18.5	6 - 45	0.0	0.0 - 0.0	2.9	0.9 - 7.2	2.7	0.8 - 8.0	1.8	0.6 - 4.7	0.0	0.0 - 0.0	11.1	3.5 - 25.5	0.25
3070101	6870	OCONEE R	3.2	15.5	5 - 38	0.0	0.0 - 0.0	0.0	0.0 - 0.0	1.1	0.4 - 3.1	1.5	0.5 - 3.9	0.0	0.0 - 0.0	12.9	3.9 - 32.0	0.25
3070101	6871	HARRIS CR	20.4	22.6	8 - 40	0.0	0.0 - 0.0	3.8	1.2 - 7.9	3.5	0.9 - 9.1	4.9	1.3 - 11.8	0.0	0.0 - 0.0	10.4	3.5 - 21.1	0.25
3070101	6872	OCONEE R	28.6	30.6	12 - 57	0.0	0.0 - 0.0	4.9	1.6 - 11.1	5.7	2.0 - 15.3	7.0	2.6 - 18.1	0.0	0.0 - 0.0	13.1	4.5 - 29.6	0.25
3070101	6873	SANDY CR	34.9	19.7	7 - 38	0.0	0.0 - 0.0	3.3	1.2 - 7.9	3.1	1.1 - 8.6	2.4	0.9 - 5.6	0.0	0.0 - 0.0	10.8	3.7 - 21.7	0.25
3070101	6875	OCONEE R	10.4	22.7	8 - 52	0.0	0.0 - 0.0	3.7	1.3 - 9.2	3.3	1.1 - 9.0	2.0	0.7 - 5.5	0.0	0.0 - 0.0	13.7	4.9 - 31.9	0.25
3070101	6876	FALLING CR	35.8	24.9	9 - 48	0.0	0.0 - 0.0	4.0	1.4 - 8.9	6.4	2.1 - 18.2	3.8	1.5 - 8.3	0.0	0.0 - 0.0	10.8	4.0 - 24.2	0.25
3070101	6877	OCONEE R	6.7	20.1	8 - 41	0.0	0.0 - 0.0	3.0	1.1 - 6.2	2.8	0.8 - 8.0	1.0	0.4 - 2.2	0.0	0.0 - 0.0	13.3	5.2 - 30.4	0.25
3070101	6878	BIG CR	1.7	28.7	9 - 60	0.0	0.0 - 0.0	5.0	1.5 - 11.1	7.7	2.3 - 20.0	3.1	0.9 - 7.9	0.0	0.0 - 0.0	12.8	3.7 - 29.7	0.25
3070101	6879	BARROW CR	73.3	25.7	7 - 49	0.0	0.0 - 0.0	3.8	1.0 - 7.9	6.9	1.8 - 19.2	4.3	1.3 - 11.4	0.0	0.0 - 0.0	10.6	2.8 - 28.1	0.24
3070101	6880	BIG CR	78.3	43.3	12 - 93	0.0	0.0 - 0.0	6.4	1.7 - 13.3	13.2	3.4 - 37.6	10.5	3.2 - 26.3	0.0	0.0 - 0.0	13.2	4.3 - 33.7	0.24
3070101	6881	OCONEE R	33.2	44.1	15 - 97	0.0	0.0 - 0.0	5.4	1.7 - 11.2	12.3	3.5 - 39.6	10.8	3.3 - 31.0	0.0	0.0 - 0.0	15.6	4.6 - 36.3	0.25
3070101	6882	SHOAL CR	47.7	46.3	14 - 116	0.0	0.0 - 0.0	17.0	5.1 - 41.1	8.0	1.9 - 26.9	8.9	2.8 - 21.7	0.0	0.0 - 0.0	12.4	3.9 - 33.6	0.25
3070101	6883	OCONEE R	51.3	156.1	51 - 369	102.7	33.2 - 240.9	22.1	7.2 - 53.0	8.8	2.5 - 26.2	8.6	2.6 - 25.5	0.0	0.0 - 0.0	13.8	4.4 - 35.7	0.25
3070101	6884	N OCONEE R	68.9	1117.2	348 - 2891	1046.8	333.1 - 2699.[illegible]	50.1	14.5 - 138.1	3.3	1.0 - 10.4	3.7	1.2 - 10.5	0.0	0.0 - 0.0	13.4	4.1 - 37.1	0.24
3070101	6885	SANDY CR	167.9	68.4	20 - 122	0.0	0.0 - 0.0	12.1	3.7 - 23.4	26.6	7.1 - 67.9	14.6	4.9 - 32.3	0.0	0.0 - 0.0	15.2	4.8 - 33.0	0.24
3070101	6886	N OCONEE R	83.1	58.0	20 - 99	0.0	0.0 - 0.0	17.4	5.8 - 32.3	16.6	4.6 - 40.3	9.2	3.3 - 20.6	0.0	0.0 - 0.0	14.7	5.7 - 34.6	0.24
3070101	6887	N OCONEE R	187.0	64.3	25 - 110	0.0	0.0 - 0.0	12.6	4.2 - 23.6	24.4	8.2 - 75.5	12.5	4.7 - 29.5	0.0	0.0 - 0.0	14.8	5.4 - 31.5	0.23
3070101	6888	PARKS CR	47.4	86.3	30 - 180	0.0	0.0 - 0.0	9.6	3.5 - 20.2	42.5	14.4 - 102.7	20.2	7.3 - 55.3	0.0	0.0 - 0.0	14.1	5.1 - 32.9	0.21
3070101	6889	N OCONEE R	153.7	59.9	19 - 109	0.0	0.0 - 0.0	13.3	4.2 - 30.3	24.4	7.2 - 59.8	10.7	3.1 - 31.3	0.0	0.0 - 0.0	11.7	3.9 - 27.4	0.21
3070101	6890	CURRY CR	72.1	69.2	19 - 143	2.7	0.8 - 6.4	12.2	3.5 - 27.0	25.8	6.7 - 65.6	13.4	3.5 - 37.2	0.0	0.0 - 0.0	15.0	4.2 - 33.8	0.23
3070101	6891	M OCONEE R	40.8	56.6	21 - 133	0.0	0.0 - 0.0	23.5	7.9 - 58.4	10.0	2.8 - 30.0	9.2	3.4 - 21.9	0.0	0.0 - 0.0	13.9	5.3 - 35.5	0.24
3070101	6892	M OCONEE R	244.7	88.1	30 - 188	23.4	8.2 - 55.5	23.8	7.5 - 54.2	15.0	4.5 - 41.8	11.0	3.8 - 30.2	0.0	0.0 - 0.0	14.9	5.0 - 40.7	0.24
3070101	6893	M OCONEE R	73.3	65.4	19 - 101	0.3	0.1 - 0.4	12.2	3.6 - 21.1	25.1	5.8 - 60.6	13.2	3.6 - 28.1	0.0	0.0 - 0.0	14.7	4.1 - 31.1	0.23
3070101	6894	M OCONEE R, PON	9.9	60.8	22 - 133	0.0	0.0 - 0.0	24.5	7.6 - 59.3	14.1	4.6 - 41.0	7.1	2.2 - 20.1	0.0	0.0 - 0.0	15.1	5.7 - 36.3	0.22
3070101	6895	M OCONEE R, PON	90.2	75.1	27 - 121	0.0	0.0 - 0.0	12.1	3.9 - 20.4	33.5	11.4 - 74.9	16.4	5.2 - 37.2	0.0	0.0 - 0.0	13.0	4.4 - 25.8	0.21
3070101	6896	ALLEN CR	65.7	64.5	24 - 159	0.0	0.0 - 0.0	16.7	6.2 - 40.3	25.1	8.1 - 79.5	11.9	3.6 - 32.7	0.0	0.0 - 0.0	10.8	3.8 - 27.5	0.21
3070101	6897	M OCONEE R	143.3	65.6	25 - 136	0.0	0.0 - 0.0	19.0	7.4 - 42.4	23.8	8.3 - 69.0	11.5	4.6 - 32.8	0.0	0.0 - 0.0	11.3	4.0 - 25.2	0.22
3070101	6898	MULBERRY R	59.5	73.5	23 - 158	0.0	0.0 - 0.0	7.3	2.5 - 14.2	31.4	9.1 - 84.5	19.8	5.6 - 43.7	0.0	0.0 - 0.0	15.0	5.1 - 37.2	0.23
3070101	6899	MULBERRY R	58.8	69.3	25 - 120	0.0	0.0 - 0.0	11.4	3.9 - 21.5	27.0	9.3 - 66.5	16.1	5.5 - 38.4	0.0	0.0 - 0.0	14.8	6.0 - 33.9	0.22
3070101	6900	MULBERRY R	173.3	64.5	24 - 136	0.0	0.0 - 0.0	24.6	8.8 - 46.8	16.9	5.6 - 39.9	10.0	3.9 - 26.9	0.0	0.0 - 0.0	13.1	4.5 - 31.7	0.21
3070101	6901	LITTLE MULBERR	78.9	61.4	21 - 117	0.0	0.0 - 0.0	24.8	8.2 - 52.3	9.8	3.3 - 28.8	12.4	4.4 - 30.3	0.0	0.0 - 0.0	14.3	4.9 - 31.7	0.21
3070101	6902	ROCKY CR	36.0	75.7	29 - 147	0.0	0.0 - 0.0	35.6	13.6 - 72.7	13.3	4.1 - 29.8	11.2	3.7 - 25.9	0.0	0.0 - 0.0	15.6	5.5 - 34.0	0.22
3070101	6903	BARBER CR	1.6	69.4	20 - 140	0.0	0.0 - 0.0	53.8	15.5 - 121.0	2.1	0.6 - 5.8	1.6	0.6 - 4.0	0.0	0.0 - 0.0	11.8	3.6 - 26.8	0.24
3070101	6904	MCNUTT CR	39.3	61.3	19 - 105	0.0	0.0 - 0.0	36.3	11.4 - 63.0	6.1	1.9 - 14.4	6.0	2.1 - 12.5	0.0	0.0 - 0.0	12.9	4.2 - 21.8	0.24
3070101	6905	BARBER CR	109.1	58.5	19 - 134	0.1	0.0 - 0.2	17.3	5.9 - 40.1	14.1	4.2 - 38.4	13.2	4.1 - 35.6	0.0	0.0 - 0.0	13.7	4.2 - 32.6	0.24
3070101	6906	ROSE CR	61.4	41.4	16 - 95	0.0	0.0 - 0.0	3.8	1.3 - 7.4	12.5	4.3 - 36.3	12.0	4.3 - 35.5	0.0	0.0 - 0.0	13.1	4.6 - 33.3	0.25
3070101	6907	GREENBRIER CR	85.6	32.2	11 - 70	0.0	0.0 - 0.0	3.9	1.2 - 7.6	7.9	2.4 - 20.6	8.4	2.7 - 21.7	0.0	0.0 - 0.0	12.1	4.3 - 28.8	0.25
3070101	6908	APALACHEE R	59.9	33.6	10 - 61	0.0	0.0 - 0.0	5.7	1.8 - 11.8	5.5	1.4 - 14.3	7.0	2.3 - 15.7	0.0	0.0 - 0.0	15.3	4.6 - 31.5	0.26

8-digit HUC	ID	Name	Area	Catchment Yield		Point sources		Developed Land		Manure		Agricultural Land		Phosphate Mines		Soil parent rock		Frac
				kg/km^2	90% CI	kg/km^2	90% CI	kg/km^2	90% CI	kg/km^2	90% CI	kg/km^2	90% CI	kg/km^2	90% CI	kg/km^2	90% CI	
3070101	6909	APALACHEE R	83.2	29.0	9 - 82	0.0	0.0 - 0.0	3.8	1.3 - 9.0	5.9	1.8 - 18.5	6.0	2.1 - 17.0	0.0	0.0 - 0.0	13.2	4.2 - 37.4	0.25
3070101	6910	APALACHEE R	166.8	42.6	13 - 79	0.0	0.0 - 0.0	6.9	2.2 - 13.1	10.7	3.1 - 27.7	11.5	4.1 - 28.1	0.0	0.0 - 0.0	13.6	4.2 - 28.4	0.24
3070101	6911	APALACHEE R	19.3	55.1	18 - 122	0.0	0.0 - 0.0	6.4	2.1 - 16.1	16.8	4.8 - 41.3	16.9	5.0 - 42.4	0.0	0.0 - 0.0	15.1	5.3 - 40.9	0.22
3070101	6912	MARBURG CR	71.2	71.0	22 - 179	8.9	2.7 - 25.1	19.4	5.4 - 50.0	14.7	3.6 - 38.1	12.6	4.2 - 36.9	0.0	0.0 - 0.0	15.3	5.4 - 34.7	0.22
3070101	6913	APALACHEE R	179.9	54.0	16 - 99	0.0	0.0 - 0.0	18.1	5.6 - 35.6	8.6	2.4 - 21.7	12.2	4.0 - 28.4	0.0	0.0 - 0.0	15.2	5.0 - 36.9	0.22
3070101	6914	SHOAL CR	48.1	41.3	12 - 87	0.0	0.0 - 0.0	7.9	2.5 - 18.1	6.4	1.9 - 17.4	13.8	4.3 - 42.2	0.0	0.0 - 0.0	13.2	4.4 - 36.2	0.22
3070101	6915	JACK'S CR	150.8	62.1	23 - 139	19.0	6.3 - 43.7	11.6	4.0 - 22.9	6.6	2.2 - 18.8	12.2	4.3 - 32.9	0.0	0.0 - 0.0	12.6	4.2 - 30.0	0.24
3070101	6916	BIG SANDY CR	25.2	29.1	10 - 70	0.0	0.0 - 0.0	6.8	2.2 - 16.2	4.3	1.2 - 10.6	5.3	1.8 - 15.2	0.0	0.0 - 0.0	12.7	4.2 - 32.4	0.25
3070101	6917	BIG SANDY CR	175.7	43.2	17 - 103	0.0	0.0 - 0.0	5.2	2.1 - 10.9	9.9	3.3 - 30.5	16.1	6.0 - 38.8	0.0	0.0 - 0.0	12.1	4.9 - 29.8	0.24
3070101	6918	HARD LABOR CR	223.9	34.0	13 - 86	0.0	0.0 - 0.0	6.8	2.5 - 15.6	5.6	1.9 - 14.8	9.4	3.4 - 26.0	0.0	0.0 - 0.0	12.1	3.9 - 30.8	0.24
3070101	6919	SUGAR CR	19.8	32.4	11 - 65	0.0	0.0 - 0.0	6.3	2.3 - 12.4	3.0	0.9 - 7.5	4.8	1.6 - 11.3	0.0	0.0 - 0.0	18.3	6.3 - 43.9	0.26
3070101	6920	SUGAR CR	37.7	36.0	10 - 72	0.0	0.0 - 0.0	6.1	2.0 - 13.9	7.6	2.5 - 20.1	9.9	3.0 - 24.3	0.0	0.0 - 0.0	12.5	3.9 - 28.6	0.25
3070101	6921	SUGAR CR	40.3	39.9	11 - 76	0.0	0.0 - 0.0	11.6	3.0 - 24.8	7.3	1.7 - 21.9	9.8	2.8 - 24.2	0.0	0.0 - 0.0	11.2	3.3 - 25.9	0.22
3070101	6922	S SUGAR CR	29.3	37.7	13 - 88	0.0	0.0 - 0.0	7.8	2.5 - 18.1	7.7	2.2 - 25.6	11.1	3.8 - 27.2	0.0	0.0 - 0.0	11.1	3.7 - 27.1	0.22
3070101	6923	LITTLE SUGAR CR	85.6	30.5	10 - 67	0.0	0.0 - 0.0	3.9	1.3 - 9.0	6.6	1.9 - 16.7	9.0	2.9 - 23.0	0.0	0.0 - 0.0	11.0	3.7 - 26.4	0.25
3070101	6924	GLADE BK	72.9	30.2	12 - 66	0.0	0.0 - 0.0	4.4	1.6 - 10.4	4.2	1.5 - 13.9	8.4	2.8 - 21.0	0.0	0.0 - 0.0	13.1	4.6 - 34.3	0.26
3070101	6925	GLADLY CR	86.2	23.3	8 - 45	0.0	0.0 - 0.0	4.8	1.7 - 10.5	2.2	0.7 - 5.1	3.6	1.3 - 9.0	0.0	0.0 - 0.0	12.8	4.4 - 30.1	0.52
3070101	6926	LITTLE R	32.2	21.7	8 - 54	0.0	0.0 - 0.0	3.9	1.3 - 9.6	1.4	0.5 - 4.4	2.5	0.8 - 6.4	0.0	0.0 - 0.0	13.8	5.2 - 34.4	0.52
3070101	6927	INDIAN CR	211.7	32.9	9 - 59	0.0	0.0 - 0.0	6.2	1.5 - 13.7	6.5	1.5 - 16.5	9.0	2.5 - 20.8	0.0	0.0 - 0.0	11.1	3.1 - 24.6	0.50
3070101	6928	LITTLE R	261.3	31.2	11 - 59	0.0	0.0 - 0.0	4.0	1.4 - 7.8	6.5	2.1 - 15.2	9.2	3.1 - 18.7	0.0	0.0 - 0.0	11.5	4.1 - 25.1	0.50
3070101	6929	HUNNICUT CR	23.0	42.9	16 - 78	0.0	0.0 - 0.0	8.9	3.2 - 18.4	9.2	2.8 - 23.8	13.0	4.8 - 27.5	0.0	0.0 - 0.0	11.8	4.6 - 25.9	0.41
3070101	6930	LITTLE R	69.6	42.6	15 - 86	8.6	3.3 - 19.5	9.5	3.0 - 21.1	3.5	1.1 - 8.9	9.0	3.0 - 22.5	0.0	0.0 - 0.0	12.0	4.2 - 28.9	0.41
3070101	6931	MURDER CR	86.6	26.5	11 - 51	0.0	0.0 - 0.0	5.4	1.9 - 12.0	5.1	1.6 - 14.1	3.8	1.3 - 9.2	0.0	0.0 - 0.0	12.1	4.5 - 25.9	0.51
3070101	6932	MURDER CR	44.2	28.7	11 - 59	0.0	0.0 - 0.0	4.3	1.5 - 8.9	6.3	2.3 - 15.8	6.0	2.2 - 14.7	0.0	0.0 - 0.0	12.1	4.2 - 28.1	0.48
3070101	6933	PITTMAN CR	78.6	34.0	13 - 91	1.2	0.5 - 3.3	5.7	2.1 - 14.4	6.1	1.9 - 21.0	9.0	3.2 - 27.6	0.0	0.0 - 0.0	11.9	4.3 - 32.9	0.45
3070101	6934	MURDER CR	63.2	26.2	10 - 50	0.0	0.0 - 0.0	4.0	1.5 - 8.5	5.2	1.8 - 14.2	5.2	2.1 - 13.5	0.0	0.0 - 0.0	11.7	4.5 - 25.2	0.45
3070101	6935	SHOAL CR	80.6	37.7	11 - 67	0.0	0.0 - 0.0	7.8	2.7 - 14.5	8.6	2.3 - 22.2	9.8	3.0 - 22.5	0.0	0.0 - 0.0	11.5	3.4 - 25.1	0.48
3070101	6936	WOLF CR, N FK	63.1	28.7	8 - 56	0.0	0.0 - 0.0	3.6	1.2 - 7.0	6.8	1.5 - 18.9	6.9	2.3 - 16.3	0.0	0.0 - 0.0	11.4	3.3 - 28.1	0.51
3070101	6938	CEDAR CR	170.3	19.4	7 - 39	0.0	0.0 - 0.0	3.6	1.3 - 7.1	2.4	0.7 - 6.8	2.3	0.8 - 5.4	0.0	0.0 - 0.0	11.1	3.9 - 24.2	0.49
3070101	6939	BIG CEDAR CR	43.7	18.5	6 - 41	0.0	0.0 - 0.0	3.7	1.1 - 9.5	0.9	0.3 - 2.8	2.8	0.9 - 8.3	0.0	0.0 - 0.0	11.0	3.6 - 25.7	0.49
3070101	6940	HOG CR	54.6	21.6	7 - 43	0.0	0.0 - 0.0	3.9	1.1 - 8.1	1.6	0.5 - 4.5	5.1	1.5 - 13.3	0.0	0.0 - 0.0	11.0	3.4 - 25.9	0.50
3070102	6941	OCONEE R	201.9	21.5	6 - 43	0.0	0.0 - 0.0	2.7	0.7 - 5.5	0.8	0.2 - 2.1	7.3	2.3 - 17.6	0.0	0.0 - 0.0	10.8	2.9 - 25.5	0.96
3070102	6942	*A	41.2	18.7	6 - 39	0.0	0.0 - 0.0	3.8	1.2 - 9.2	0.8	0.2 - 2.1	5.8	2.2 - 14.1	0.0	0.0 - 0.0	8.4	2.7 - 21.7	0.95
3070102	6943	OCONEE R	99.3	20.2	6 - 36	0.0	0.0 - 0.0	6.2	1.9 - 13.7	0.6	0.2 - 1.6	4.0	1.4 - 9.5	0.0	0.0 - 0.0	9.5	3.0 - 22.0	0.95
3070102	6944	OCONEE R	58.4	18.5	6 - 32	0.0	0.0 - 0.0	4.4	1.4 - 8.5	0.8	0.2 - 1.9	4.0	1.4 - 9.6	0.0	0.0 - 0.0	9.2	2.9 - 18.6	0.95
3070102	6945	BEAR CR	47.1	19.6	8 - 51	0.0	0.0 - 0.0	3.8	1.5 - 9.5	0.9	0.3 - 2.8	6.9	2.9 - 18.6	0.0	0.0 - 0.0	8.1	3.1 - 22.8	0.94
3070102	6946	OCONEE R	93.4	18.9	6 - 35	0.0	0.0 - 0.0	3.4	1.1 - 6.6	0.7	0.2 - 1.8	5.1	1.7 - 11.2	0.0	0.0 - 0.0	9.8	3.5 - 23.6	0.94
3070102	6947	BULL CR	115.9	18.8	6 - 38	0.0	0.0 - 0.0	6.7	2.0 - 14.1	0.4	0.1 - 1.1	4.6	1.7 - 12.4	0.0	0.0 - 0.0	7.1	2.5 - 16.9	0.94
3070102	6948	OCONEE R	65.7	18.0	5 - 47	0.0	0.0 - 0.0	3.0	1.0 - 7.1	0.6	0.2 - 1.7	5.2	1.7 - 14.5	0.0	0.0 - 0.0	9.2	3.0 - 25.2	0.94

8-digit HUC	ID	Name	Area	Catchment Yield		Point sources		Developed Land		Manure		Agricultural Land		Phosphate Mines		Soil parent rock		Frac
				kg/km^2	90% CI	kg/km^2	90% CI	kg/km^2	90% CI	kg/km^2	90% CI	kg/km^2	90% CI	kg/km^2	90% CI	kg/km^2	90% CI	
3070102	6949	OCONEE R	17.9	20.0	8 - 53	0.0	0.0 - 0.0	1.1	0.4 - 3.1	0.5	0.2 - 2.0	3.6	1.4 - 9.5	0.0	0.0 - 0.0	14.7	5.9 - 43.4	0.94
3070102	6950	MERCER CR	109.2	15.1	5 - 33	0.0	0.0 - 0.0	4.2	1.5 - 9.8	0.5	0.2 - 1.6	3.7	1.2 - 9.0	0.0	0.0 - 0.0	6.6	2.0 - 16.2	0.93
3070102	6951	OCONEE R	96.4	14.6	7 - 32	0.0	0.0 - 0.0	2.0	0.8 - 4.8	0.6	0.2 - 1.8	3.0	1.1 - 8.3	0.0	0.0 - 0.0	9.0	3.9 - 22.4	0.93
3070102	6952	OCONEE R	19.1	17.7	6 - 39	0.0	0.0 - 0.0	3.1	1.0 - 6.3	0.9	0.3 - 2.1	5.5	2.0 - 12.3	0.0	0.0 - 0.0	8.2	2.7 - 19.3	0.93
3070102	6953	PUGHES CR	170.8	16.3	6 - 27	0.0	0.0 - 0.0	4.2	1.3 - 7.7	0.7	0.2 - 1.7	5.0	1.6 - 11.5	0.0	0.0 - 0.0	6.4	2.2 - 14.6	0.92
3070102	6954	OCONEE R	8.2	15.5	6 - 38	0.0	0.0 - 0.0	2.4	0.9 - 5.1	0.0	0.0 - 0.1	0.1	0.0 - 0.2	0.0	0.0 - 0.0	13.0	4.5 - 32.7	0.92
3070102	6955	*B	35.7	424.9	148 - 1019	401.0	141.0 - 978.5	6.9	2.2 - 15.8	1.2	0.4 - 3.5	9.3	2.9 - 24.7	0.0	0.0 - 0.0	6.5	2.3 - 15.6	0.92
3070102	6956	OCONEE R	50.8	145.8	49 - 294	100.5	37.1 - 215.9	28.6	9.5 - 59.0	0.9	0.3 - 2.6	7.6	2.6 - 18.2	0.0	0.0 - 0.0	8.1	2.8 - 17.8	0.92
3070102	6957	OCONEE R	6.2	23.1	7 - 60	0.0	0.0 - 0.0	8.0	2.3 - 20.6	1.1	0.3 - 3.2	3.7	1.1 - 8.9	0.0	0.0 - 0.0	10.3	3.3 - 30.6	0.92
3070102	6958	BIG CR	25.9	29.7	11 - 57	0.0	0.0 - 0.0	11.5	3.9 - 21.8	1.2	0.4 - 3.3	9.4	3.6 - 23.5	0.0	0.0 - 0.0	7.6	3.0 - 15.4	0.92
3070102	6959	HIGHTOWER CR	70.7	17.3	5 - 49	0.0	0.0 - 0.0	4.0	1.0 - 10.8	0.8	0.2 - 2.4	5.8	1.3 - 19.9	0.0	0.0 - 0.0	6.7	2.0 - 20.1	0.87
3070102	6960	BIG CR	15.7	18.6	7 - 39	0.0	0.0 - 0.0	2.9	1.0 - 6.8	0.8	0.2 - 2.4	6.1	2.4 - 15.7	0.0	0.0 - 0.0	8.8	3.2 - 21.0	0.87
3070102	6961	BIG CR, BIG BR	37.4	23.3	6 - 42	0.0	0.0 - 0.0	3.6	1.1 - 7.7	1.4	0.4 - 4.2	10.3	2.8 - 24.3	0.0	0.0 - 0.0	8.0	2.3 - 19.9	0.80
3070102	6962	BIG CR	39.7	21.2	7 - 49	0.0	0.0 - 0.0	2.2	0.6 - 5.0	1.4	0.3 - 3.9	9.9	3.0 - 27.2	0.0	0.0 - 0.0	7.7	2.3 - 21.0	0.80
3070102	6963	OCONEE R	53.4	26.1	10 - 62	0.0	0.0 - 0.0	4.1	1.6 - 12.0	1.6	0.5 - 4.6	11.7	4.3 - 31.3	0.0	0.0 - 0.0	8.7	3.2 - 27.0	0.92
3070102	6964	OCONEE R	5.7	27.5	9 - 49	0.0	0.0 - 0.0	2.2	0.7 - 4.5	1.7	0.5 - 4.3	12.6	4.2 - 28.6	0.0	0.0 - 0.0	11.0	4.0 - 22.8	0.91
3070102	6965	BUCKEYE CR	38.0	16.8	5 - 35	0.0	0.0 - 0.0	1.1	0.4 - 2.3	0.9	0.2 - 2.6	5.9	1.9 - 14.3	0.0	0.0 - 0.0	8.8	2.8 - 22.4	0.91
3070102	6966	FORDS CR	23.4	19.1	7 - 49	0.0	0.0 - 0.0	2.8	1.1 - 6.7	1.3	0.4 - 3.8	6.9	2.5 - 20.0	0.0	0.0 - 0.0	8.1	2.9 - 21.2	0.81
3070102	6967	BUCKEYE CR	65.6	18.3	5 - 33	0.0	0.0 - 0.0	2.5	0.8 - 4.8	1.2	0.3 - 2.9	6.5	2.2 - 15.3	0.0	0.0 - 0.0	8.1	2.5 - 17.5	0.81
3070102	6968	OCONEE R	4.4	16.0	6 - 41	0.0	0.0 - 0.0	0.2	0.1 - 0.4	0.0	0.0 - 0.0	0.0	0.0 - 0.0	0.0	0.0 - 0.0	15.8	5.7 - 41.0	0.91
3070102	6969	OCONEE R	5.8	16.0	5 - 38	0.0	0.0 - 0.0	0.0	0.0 - 0.0	0.2	0.1 - 0.7	0.2	0.0 - 0.5	0.0	0.0 - 0.0	15.6	5.1 - 37.7	0.91
3070102	6970	DEEP CR	45.8	14.2	5 - 34	0.0	0.0 - 0.0	2.6	0.9 - 5.4	0.7	0.2 - 2.0	3.1	1.0 - 7.9	0.0	0.0 - 0.0	7.7	2.7 - 19.7	0.91
3070102	6971	OCONEE R	5.2	16.8	6 - 35	0.0	0.0 - 0.0	0.3	0.1 - 0.6	0.7	0.2 - 1.9	0.7	0.3 - 1.7	0.0	0.0 - 0.0	15.0	5.6 - 32.2	0.91
3070102	6972	OCONEE R	76.6	13.8	5 - 41	0.0	0.0 - 0.0	1.6	0.6 - 5.4	0.3	0.1 - 1.2	1.8	0.7 - 6.0	0.0	0.0 - 0.0	10.1	3.6 - 31.2	0.91
3070102	6973	OCONEE R	8.9	14.7	4 - 42	0.0	0.0 - 0.0	0.7	0.2 - 1.8	0.1	0.0 - 0.5	0.1	0.0 - 0.4	0.0	0.0 - 0.0	13.6	4.1 - 41.6	0.90
3070102	6975	SANDY HILL CR	93.0	14.7	5 - 36	0.0	0.0 - 0.0	2.8	1.0 - 6.5	0.4	0.1 - 1.3	2.7	1.0 - 7.2	0.0	0.0 - 0.0	8.9	3.0 - 25.6	0.90
3070102	6976	BUFFALO CR	37.9	12.3	5 - 28	0.0	0.0 - 0.0	1.7	0.7 - 4.2	0.4	0.1 - 1.1	0.5	0.2 - 1.4	0.0	0.0 - 0.0	9.7	3.4 - 24.2	0.90
3070102	6977	LAMARS CR	57.4	14.4	5 - 28	0.0	0.0 - 0.0	1.7	0.6 - 4.1	0.4	0.1 - 1.3	3.0	1.1 - 8.3	0.0	0.0 - 0.0	9.2	3.2 - 20.5	0.87
3070102	6978	BUFFALO CR	37.7	12.2	4 - 26	0.0	0.0 - 0.0	2.3	0.9 - 4.6	0.5	0.2 - 1.5	0.6	0.2 - 1.5	0.0	0.0 - 0.0	8.8	2.9 - 21.3	0.87
3070102	6979	KEG CR	15.5	14.3	5 - 32	0.0	0.0 - 0.0	2.2	0.7 - 4.8	0.6	0.2 - 2.0	2.7	1.0 - 6.5	0.0	0.0 - 0.0	8.8	2.7 - 19.6	0.85
3070102	6980	ROBINSON CR	48.7	45.7	17 - 92	21.3	7.4 - 42.7	12.7	4.2 - 26.8	0.4	0.1 - 1.1	1.7	0.7 - 4.3	0.0	0.0 - 0.0	9.7	3.3 - 22.4	0.83
3070102	6981	KEG CR	79.1	16.1	6 - 35	0.0	0.0 - 0.0	4.1	1.5 - 10.3	0.6	0.2 - 1.4	2.9	1.0 - 8.7	0.0	0.0 - 0.0	8.6	3.2 - 19.3	0.83
3070102	6982	LITTLE KEG CR	37.6	15.5	5 - 33	0.0	0.0 - 0.0	1.2	0.4 - 2.5	0.8	0.2 - 2.1	5.2	1.7 - 11.4	0.0	0.0 - 0.0	8.3	2.5 - 22.0	0.76
3070102	6983	KEG CR	67.7	9.5	3 - 18	0.0	0.0 - 0.0	1.1	0.3 - 2.0	0.4	0.1 - 1.1	2.3	0.6 - 5.7	0.0	0.0 - 0.0	5.7	1.4 - 12.3	0.76
3070102	6984	BUFFALO CR	9.9	9.6	3 - 20	0.0	0.0 - 0.0	0.7	0.2 - 1.4	0.6	0.2 - 1.6	0.1	0.0 - 0.2	0.0	0.0 - 0.0	8.2	2.5 - 18.5	0.85
3070102	6985	BUFFALO CR	93.8	11.2	4 - 22	0.0	0.0 - 0.0	2.3	0.8 - 5.2	0.3	0.1 - 1.0	1.2	0.4 - 2.6	0.0	0.0 - 0.0	7.4	2.8 - 15.5	0.85
3070102	6986	BUFFALO CR	8.6	14.3	5 - 35	0.0	0.0 - 0.0	2.3	0.7 - 5.2	0.3	0.1 - 0.8	1.5	0.4 - 3.8	0.0	0.0 - 0.0	10.3	3.6 - 26.8	0.79
3070102	6987	BUFFALO CR	17.7	18.1	5 - 40	0.0	0.0 - 0.0	2.4	0.7 - 5.6	0.4	0.1 - 1.0	1.5	0.5 - 3.6	0.0	0.0 - 0.0	13.8	4.0 - 37.4	0.78
3070102	6988	SANDY RUN CR	36.0	13.3	5 - 31	0.0	0.0 - 0.0	2.9	1.1 - 6.6	0.3	0.1 - 0.9	2.4	1.0 - 6.1	0.0	0.0 - 0.0	7.8	2.7 - 21.9	0.75

8-digit HUC	ID	Name	Area	Catchment Yield		Point sources		Developed Land		Manure		Agricultural Land		Phosphate Mines		Soil parent rock		Frac
				kg/km^2	90% CI	kg/km^2	90% CI	kg/km^2	90% CI	kg/km^2	90% CI	kg/km^2	90% CI	kg/km^2	90% CI	kg/km^2	90% CI	
3070102	6989	BUFFALO CR	4.1	29.9	10 - 57	0.0	0.0 - 0.0	4.1	1.4 - 8.3	0.8	0.2 - 2.1	0.6	0.2 - 1.2	0.0	0.0 - 0.0	24.5	8.6 - 50.1	0.75
3070102	6990	BUFFALO CR	5.1	27.7	10 - 51	0.0	0.0 - 0.0	2.8	1.0 - 5.0	0.3	0.1 - 0.8	0.4	0.2 - 0.9	0.0	0.0 - 0.0	24.1	9.1 - 47.9	0.72
3070102	6991	*C	31.2	22.7	8 - 41	0.0	0.0 - 0.0	4.6	1.5 - 9.0	0.7	0.2 - 1.9	6.0	2.0 - 14.3	0.0	0.0 - 0.0	11.4	4.0 - 24.0	0.69
3070102	6992	BUFFALO CR	48.7	26.1	8 - 48	0.0	0.0 - 0.0	8.1	2.3 - 16.7	0.4	0.1 - 1.1	2.2	0.8 - 5.1	0.0	0.0 - 0.0	15.4	4.4 - 32.5	0.69
3070102	6993	ROCKY CR	10.8	33.6	11 - 79	0.0	0.0 - 0.0	5.0	1.7 - 11.2	0.7	0.2 - 1.8	5.6	1.8 - 12.8	0.0	0.0 - 0.0	22.4	7.4 - 58.2	0.72
3070102	6994	LITTLE BUFFALO (	58.2	17.4	6 - 45	0.0	0.0 - 0.0	3.0	1.0 - 7.9	0.4	0.1 - 1.2	2.4	0.8 - 7.1	0.0	0.0 - 0.0	11.7	4.2 - 33.6	0.78
3070102	6995	SWIFT CR	22.5	13.5	5 - 31	0.0	0.0 - 0.0	4.1	1.5 - 9.6	0.2	0.1 - 0.5	1.4	0.5 - 4.0	0.0	0.0 - 0.0	7.8	3.0 - 18.8	0.79
3070102	6996	DEEPSTEP CR	30.5	9.6	3 - 25	0.0	0.0 - 0.0	2.0	0.6 - 4.9	0.3	0.1 - 0.8	1.4	0.5 - 3.4	0.0	0.0 - 0.0	6.0	1.9 - 17.4	0.85
3070102	6997	OCONEE R	34.5	16.1	5 - 35	0.0	0.0 - 0.0	0.7	0.2 - 1.5	0.4	0.1 - 1.0	0.4	0.1 - 0.8	0.0	0.0 - 0.0	14.6	4.8 - 35.7	0.90
3070102	6998	OCONEE R	37.9	18.5	6 - 41	0.0	0.0 - 0.0	0.3	0.1 - 0.7	1.0	0.3 - 3.2	2.7	0.9 - 6.3	0.0	0.0 - 0.0	14.5	4.6 - 35.6	0.90
3070102	6999	BLUFF CR	43.9	12.3	4 - 24	0.0	0.0 - 0.0	2.4	0.8 - 5.3	0.6	0.2 - 1.3	1.8	0.6 - 4.5	0.0	0.0 - 0.0	7.5	2.7 - 17.0	0.89
3070102	7000	OCONEE R	4.2	21.5	6 - 58	0.0	0.0 - 0.0	0.0	0.0 - 0.0	0.8	0.2 - 2.7	0.3	0.1 - 0.7	0.0	0.0 - 0.0	20.5	5.4 - 54.9	0.89
3070102	7001	*D	41.5	9.8	4 - 21	0.0	0.0 - 0.0	1.5	0.6 - 3.4	0.5	0.2 - 1.2	1.0	0.4 - 2.3	0.0	0.0 - 0.0	6.7	2.9 - 14.4	0.89
3070102	7002	OCONEE R	2.7	24.2	8 - 57	0.0	0.0 - 0.0	0.0	0.0 - 0.0	1.6	0.5 - 4.7	2.1	0.7 - 5.6	0.0	0.0 - 0.0	20.5	6.9 - 50.6	0.89
3070102	7003	TOWN CR	33.8	18.0	6 - 41	0.0	0.0 - 0.0	2.6	0.8 - 5.5	1.3	0.4 - 3.7	3.7	1.1 - 10.7	0.0	0.0 - 0.0	10.5	3.7 - 24.6	0.89
3070102	7004	SPRING CR	40.7	11.6	4 - 23	0.0	0.0 - 0.0	3.2	1.0 - 6.7	0.4	0.1 - 1.0	2.0	0.6 - 5.2	0.0	0.0 - 0.0	6.0	2.0 - 12.7	0.79
3070102	7005	TOWN CR	83.2	15.0	5 - 28	0.0	0.0 - 0.0	4.1	1.1 - 7.7	0.7	0.2 - 1.7	2.8	1.0 - 6.7	0.0	0.0 - 0.0	7.5	2.6 - 16.2	0.79
3070102	7006	OCONEE R	67.7	28.6	10 - 62	0.0	0.0 - 0.0	2.6	0.8 - 5.4	2.7	0.8 - 7.2	9.1	2.7 - 22.8	0.0	0.0 - 0.0	14.2	5.2 - 37.6	0.89
3070102	7007	*E	22.3	21.4	6 - 46	0.0	0.0 - 0.0	5.0	1.6 - 12.1	1.4	0.4 - 4.3	3.6	1.0 - 9.9	0.0	0.0 - 0.0	11.5	3.5 - 26.2	0.88
3070102	7008	OCONEE R	5.5	40.5	14 - 86	0.0	0.0 - 0.0	5.6	1.6 - 11.9	3.6	0.9 - 10.4	10.6	3.1 - 26.3	0.0	0.0 - 0.0	20.7	6.4 - 47.9	0.88
3070102	7009	OCONEE R	17.9	472.4	130 - 1091	437.3	117.9 - 1003.1	10.5	3.2 - 21.3	1.8	0.5 - 4.2	4.7	1.4 - 12.9	0.0	0.0 - 0.0	18.0	5.4 - 40.6	0.88
3070102	7010	OCONEE R	3.3	32.6	11 - 74	0.0	0.0 - 0.0	13.3	4.1 - 30.7	1.5	0.4 - 4.6	2.6	0.9 - 6.8	0.0	0.0 - 0.0	15.2	5.6 - 37.8	0.88
3070102	7011	FISHING CR	27.2	62.5	19 - 140	0.0	0.0 - 0.0	38.2	12.3 - 96.6	1.7	0.5 - 6.2	5.7	2.1 - 15.5	0.0	0.0 - 0.0	16.9	4.9 - 39.1	0.88
3070102	7012	*F	22.8	20.7	7 - 49	0.0	0.0 - 0.0	4.8	1.5 - 11.3	1.5	0.4 - 4.5	2.3	0.8 - 6.3	0.0	0.0 - 0.0	12.0	4.4 - 30.5	0.82
3070102	7013	FISHING CR	19.7	21.9	8 - 47	0.0	0.0 - 0.0	4.0	1.3 - 7.7	1.6	0.5 - 4.1	3.7	1.2 - 10.2	0.0	0.0 - 0.0	12.5	4.0 - 31.7	0.82
3070102	7014	FISHING CR	26.0	18.1	7 - 36	0.0	0.0 - 0.0	3.0	1.0 - 5.2	1.4	0.4 - 3.5	2.4	0.7 - 5.5	0.0	0.0 - 0.0	11.4	3.9 - 27.6	0.74
3070102	7015	MOORE CR	78.7	21.0	7 - 49	0.0	0.0 - 0.0	3.7	1.2 - 8.5	1.7	0.5 - 5.5	5.0	1.6 - 12.7	0.0	0.0 - 0.0	10.6	3.6 - 25.3	0.74
3070102	7016	CAMP CR	86.0	24.7	8 - 51	0.0	0.0 - 0.0	10.0	3.5 - 22.9	1.3	0.4 - 3.6	3.8	1.3 - 9.8	0.0	0.0 - 0.0	9.6	3.2 - 20.2	0.88
3070102	7017	BLACK CR	77.0	12.7	4 - 29	0.0	0.0 - 0.0	1.1	0.4 - 2.5	0.2	0.1 - 0.7	1.2	0.4 - 2.9	0.0	0.0 - 0.0	10.3	3.5 - 23.0	0.90
3070102	7018	BLACK CR	35.3	18.3	7 - 45	0.0	0.0 - 0.0	3.0	1.0 - 6.9	1.3	0.5 - 3.3	5.5	2.0 - 17.0	0.0	0.0 - 0.0	8.5	3.3 - 21.8	0.78
3070102	7019	*G	71.4	12.8	5 - 28	0.0	0.0 - 0.0	3.2	1.1 - 7.3	0.4	0.1 - 1.3	3.3	1.2 - 9.0	0.0	0.0 - 0.0	5.8	2.0 - 13.6	0.78
3070102	7020	COMMISSIONER C	153.1	12.5	4 - 32	0.0	0.0 - 0.0	3.3	1.0 - 7.6	0.1	0.0 - 0.3	1.2	0.4 - 2.9	0.0	0.0 - 0.0	7.9	2.6 - 19.5	0.90
3070102	7021	COMMISSIONER C	193.5	14.2	5 - 32	0.0	0.0 - 0.0	3.3	1.0 - 7.3	0.7	0.2 - 1.8	2.9	0.9 - 7.3	0.0	0.0 - 0.0	7.2	2.5 - 17.8	0.84
3070102	7022	COMMISSIONER C	31.4	21.0	8 - 42	0.0	0.0 - 0.0	4.0	1.4 - 8.7	1.5	0.5 - 3.9	5.3	1.9 - 11.3	0.0	0.0 - 0.0	10.2	3.8 - 21.8	0.69
3070102	7023	WOLF CR	45.8	28.5	10 - 60	0.0	0.0 - 0.0	7.8	2.7 - 15.8	2.0	0.6 - 5.3	7.3	2.5 - 21.8	0.0	0.0 - 0.0	11.5	3.9 - 26.6	0.69
3070102	7024	LITTLE COMMISSI	126.0	13.6	4 - 26	0.0	0.0 - 0.0	5.0	1.5 - 9.7	0.3	0.1 - 0.8	1.4	0.4 - 3.3	0.0	0.0 - 0.0	6.9	2.2 - 13.9	0.84
3070102	7026	LITTLE SANDY CR	26.9	12.1	4 - 24	0.0	0.0 - 0.0	2.0	0.6 - 4.3	0.2	0.1 - 0.6	1.8	0.5 - 4.6	0.0	0.0 - 0.0	8.1	2.2 - 17.4	0.91
3070102	7027	BIG SANDY CR	22.5	12.7	4 - 26	0.0	0.0 - 0.0	1.6	0.5 - 3.0	0.2	0.0 - 0.5	2.2	0.6 - 5.6	0.0	0.0 - 0.0	8.6	2.5 - 20.3	0.91
3070102	7029	*H	19.7	10.6	3 - 22	0.0	0.0 - 0.0	2.0	0.5 - 4.2	0.2	0.0 - 0.4	2.1	0.7 - 5.6	0.0	0.0 - 0.0	6.3	1.9 - 14.6	0.88

8-digit HUC	ID	Name	Area	Catchment Yield		Point sources		Developed Land		Manure		Agricultural Land		Phosphate Mines		Soil parent rock		Frac
				kg/km^2	90% CI	kg/km^2	90% CI	kg/km^2	90% CI	kg/km^2	90% CI	kg/km^2	90% CI	kg/km^2	90% CI	kg/km^2	90% CI	
3070102	7030	BIG SANDY CR	21.1	10.9	3 - 24	0.0	0.0 - 0.0	1.9	0.6 - 4.1	0.1	0.0 - 0.3	1.2	0.4 - 3.3	0.0	0.0 - 0.0	7.7	2.1 - 20.2	0.88
3070102	7031	BIG SANDY CR	64.9	11.7	4 - 27	0.0	0.0 - 0.0	3.5	1.2 - 8.7	0.3	0.1 - 0.7	0.7	0.2 - 2.1	0.0	0.0 - 0.0	7.3	2.7 - 16.3	0.86
3070102	7032	BIG SANDY CR	56.0	13.4	5 - 28	0.0	0.0 - 0.0	4.8	1.5 - 10.6	0.1	0.0 - 0.4	1.3	0.5 - 3.3	0.0	0.0 - 0.0	7.2	2.4 - 16.9	0.82
3070102	7033	CLEAR CR	60.9	8.7	3 - 19	0.0	0.0 - 0.0	2.8	1.0 - 5.6	0.1	0.0 - 0.4	0.5	0.2 - 1.3	0.0	0.0 - 0.0	5.3	1.9 - 12.6	0.75
3070102	7034	BIG SANDY CR	254.5	10.3	3 - 20	0.0	0.0 - 0.0	2.8	0.8 - 5.7	0.2	0.1 - 0.6	1.0	0.3 - 2.3	0.0	0.0 - 0.0	6.3	1.9 - 13.6	0.75
3070102	7035	PORTER CR	105.0	8.0	3 - 14	0.0	0.0 - 0.0	1.2	0.4 - 2.1	0.1	0.0 - 0.2	0.5	0.2 - 1.2	0.0	0.0 - 0.0	6.2	2.2 - 12.3	0.82
3070102	7036	CEDAR CR	8.1	10.6	3 - 26	0.0	0.0 - 0.0	0.4	0.1 - 0.9	0.2	0.1 - 0.7	0.6	0.2 - 1.7	0.0	0.0 - 0.0	9.3	2.7 - 23.4	0.86
3070102	7037	MAIDEN CR	38.8	11.1	3 - 23	0.0	0.0 - 0.0	2.6	0.8 - 5.4	0.2	0.1 - 0.5	1.1	0.3 - 2.9	0.0	0.0 - 0.0	7.1	2.2 - 16.1	0.83
3070102	7038	CEDAR CR	74.6	8.8	3 - 22	0.0	0.0 - 0.0	1.8	0.6 - 4.7	0.2	0.1 - 0.6	1.9	0.6 - 5.0	0.0	0.0 - 0.0	5.0	1.8 - 13.7	0.83
3070102	7039	*I	70.3	15.5	5 - 28	0.0	0.0 - 0.0	2.9	1.0 - 5.6	0.4	0.1 - 1.3	5.3	1.9 - 11.7	0.0	0.0 - 0.0	6.9	2.6 - 14.2	0.89
3070102	7040	DRY CR	36.6	14.5	5 - 30	0.0	0.0 - 0.0	2.2	0.8 - 4.7	0.6	0.2 - 1.7	4.2	1.3 - 10.5	0.0	0.0 - 0.0	7.5	2.4 - 19.6	0.91
3070102	7041	*J	44.2	14.4	5 - 28	0.0	0.0 - 0.0	3.2	1.1 - 6.6	0.7	0.2 - 2.0	4.7	1.6 - 12.0	0.0	0.0 - 0.0	5.8	2.1 - 13.4	0.91
3070102	7042	STRAWBERRY BK	7.7	40.6	13 - 87	0.0	0.0 - 0.0	32.4	10.5 - 67.8	0.4	0.1 - 1.1	2.6	0.7 - 6.9	0.0	0.0 - 0.0	5.2	1.7 - 11.8	0.92
3070102	7043	STRAWBERRY BK	16.6	26.8	9 - 46	0.0	0.0 - 0.0	13.4	3.8 - 24.8	0.9	0.3 - 2.6	6.4	2.3 - 13.5	0.0	0.0 - 0.0	6.1	1.9 - 13.8	0.87
3070102	7044	*K	45.6	36.6	15 - 81	0.0	0.0 - 0.0	21.5	8.1 - 54.9	0.9	0.4 - 2.8	7.9	3.0 - 19.4	0.0	0.0 - 0.0	6.3	2.2 - 15.9	0.87
3070102	7045	TURKEY CR	11.2	18.9	5 - 36	0.0	0.0 - 0.0	0.9	0.3 - 2.0	0.8	0.2 - 2.4	5.0	1.5 - 12.5	0.0	0.0 - 0.0	12.1	3.6 - 30.1	0.93
3070102	7046	TURKEY CR	113.5	20.5	8 - 36	0.0	0.0 - 0.0	5.1	1.9 - 10.2	1.0	0.3 - 2.7	7.4	2.7 - 16.6	0.0	0.0 - 0.0	7.0	2.6 - 14.3	0.83
3070102	7047	TURKEY CR	214.6	20.1	8 - 40	0.0	0.0 - 0.0	4.7	1.7 - 10.1	0.8	0.3 - 2.3	7.2	2.4 - 18.7	0.0	0.0 - 0.0	7.4	2.5 - 17.1	0.69
3070102	7048	TURKEY CR	104.9	16.4	6 - 30	3.1	1.1 - 6.5	3.7	1.2 - 7.8	0.3	0.1 - 0.8	4.0	1.3 - 9.9	0.0	0.0 - 0.0	5.3	1.6 - 11.2	0.57
3070102	7049	ROCKY CR	87.8	20.4	7 - 37	0.0	0.0 - 0.0	5.4	1.4 - 12.1	0.9	0.3 - 2.4	7.4	2.4 - 15.8	0.0	0.0 - 0.0	6.6	2.1 - 17.4	0.69
3070102	7050	ROCKY CR	61.6	23.5	6 - 59	0.0	0.0 - 0.0	6.6	1.9 - 16.9	1.0	0.3 - 3.0	9.6	2.7 - 27.4	0.0	0.0 - 0.0	6.3	1.6 - 18.8	0.57
3070102	7051	ROCKY CR	27.2	25.9	11 - 53	0.0	0.0 - 0.0	3.9	1.5 - 8.7	1.6	0.6 - 5.2	14.1	6.1 - 36.3	0.0	0.0 - 0.0	6.2	2.7 - 15.0	0.48
3070102	7052	*L	23.7	30.3	11 - 59	0.0	0.0 - 0.0	9.1	3.2 - 19.1	1.4	0.4 - 3.6	13.5	4.3 - 35.6	0.0	0.0 - 0.0	6.3	2.3 - 16.5	0.40
3070102	7053	ROCKY CR	5.2	26.5	9 - 56	0.0	0.0 - 0.0	1.8	0.6 - 3.6	1.7	0.4 - 5.5	16.1	5.0 - 36.9	0.0	0.0 - 0.0	7.0	2.2 - 17.3	0.40
3070102	7054	ROCKY CR	72.9	22.9	9 - 55	0.0	0.0 - 0.0	5.8	2.5 - 15.6	1.0	0.4 - 3.1	10.0	3.5 - 31.3	0.0	0.0 - 0.0	6.2	2.6 - 17.2	0.38
3070102	7055	CROOKED CR	45.4	19.3	7 - 38	0.0	0.0 - 0.0	3.3	1.2 - 6.6	1.3	0.4 - 3.8	8.8	3.0 - 22.8	0.0	0.0 - 0.0	5.9	2.2 - 12.3	0.38
3070102	7056	BUCKHORN CR	82.7	20.8	6 - 44	0.0	0.0 - 0.0	3.7	1.0 - 7.7	1.2	0.4 - 4.4	9.6	2.9 - 26.4	0.0	0.0 - 0.0	6.3	2.0 - 17.0	0.48
3070102	7057	BOGGY CR	27.9	21.1	6 - 52	0.0	0.0 - 0.0	4.5	1.4 - 10.0	1.0	0.3 - 3.1	9.2	2.6 - 26.9	0.0	0.0 - 0.0	6.4	2.3 - 18.0	0.57
3070102	7058	*M	52.4	16.8	5 - 28	0.0	0.0 - 0.0	4.7	1.8 - 8.8	0.7	0.3 - 1.8	4.8	1.7 - 10.4	0.0	0.0 - 0.0	6.5	2.3 - 13.4	0.83
3070102	7059	WHITEWATER CR	45.7	10.0	4 - 26	0.0	0.0 - 0.0	1.9	0.6 - 4.2	0.4	0.1 - 1.0	1.4	0.5 - 3.6	0.0	0.0 - 0.0	6.5	2.3 - 14.8	0.94
3070102	7060	OCHWALKEE CR	196.1	13.4	4 - 28	0.0	0.0 - 0.0	3.0	1.0 - 6.3	0.5	0.1 - 1.3	2.7	0.9 - 6.7	0.0	0.0 - 0.0	7.2	2.2 - 17.7	0.95
3070103	7061	OCMULGEE R	2.9	19.5	7 - 56	0.0	0.0 - 0.0	0.0	0.0 - 0.0	0.0	0.0 - 0.0	0.0	0.0 - 0.0	0.0	0.0 - 0.0	19.5	6.9 - 56.0	0.87
3070103	7062	OCMULGEE R	5.8	18.0	6 - 48	0.0	0.0 - 0.0	0.4	0.1 - 0.9	0.0	0.0 - 0.1	0.3	0.1 - 0.8	0.0	0.0 - 0.0	17.3	5.8 - 45.3	0.87
3070103	7063	STONE CR	129.9	13.0	4 - 30	0.0	0.0 - 0.0	5.8	1.7 - 12.3	0.2	0.0 - 0.4	1.1	0.4 - 2.9	0.0	0.0 - 0.0	5.9	1.9 - 15.3	0.86
3070103	7064	OCMULGEE R	2.2	21.5	8 - 57	0.0	0.0 - 0.0	0.0	0.0 - 0.0	0.1	0.0 - 0.3	1.9	0.7 - 4.3	0.0	0.0 - 0.0	19.4	6.9 - 53.5	0.86
3070103	7065	OCMULGEE R	74.0	377.7	137 - 790	346.1	124.1 - 757.2	17.3	6.5 - 36.7	0.5	0.2 - 1.2	1.5	0.5 - 3.6	0.0	0.0 - 0.0	12.3	4.3 - 30.5	0.86
3070103	7066	WALNUT CR	255.7	25.9	8 - 70	0.0	0.0 - 0.0	10.5	3.2 - 28.6	1.1	0.3 - 3.5	3.5	1.0 - 11.2	0.0	0.0 - 0.0	10.7	3.9 - 28.6	0.85
3070103	7067	OCMULGEE R	60.4	60.0	19 - 152	0.0	0.0 - 0.0	43.2	13.7 - 109.7	0.5	0.1 - 1.5	1.7	0.6 - 4.4	0.0	0.0 - 0.0	14.5	4.7 - 36.1	0.85
3070103	7068	OCMULGEE R	9.1	25.7	8 - 55	0.0	0.0 - 0.0	5.2	1.8 - 10.4	1.5	0.4 - 4.1	1.6	0.5 - 3.7	0.0	0.0 - 0.0	17.4	5.2 - 43.1	0.84

8-digit HUC	ID	Name	Area	Catchment Yield		Point sources		Developed Land		Manure		Agricultural Land		Phosphate Mines		Soil parent rock		Frac
				kg/km^2	90% CI	kg/km^2	90% CI	kg/km^2	90% CI	kg/km^2	90% CI	kg/km^2	90% CI	kg/km^2	90% CI	kg/km^2	90% CI	
3070103	7069	FALLING CR	90.9	16.6	6 - 36	0.0	0.0 - 0.0	3.7	1.3 - 7.8	0.4	0.1 - 1.0	0.6	0.2 - 1.5	0.0	0.0 - 0.0	12.0	4.3 - 28.2	0.84
3070103	7070	FALLING CR	127.9	16.5	5 - 40	0.0	0.0 - 0.0	3.4	1.0 - 8.2	1.1	0.3 - 3.2	0.8	0.3 - 2.1	0.0	0.0 - 0.0	11.3	3.7 - 28.3	0.78
3070103	7071	LITTLE FALLING C	59.2	17.5	6 - 40	0.0	0.0 - 0.0	3.5	1.2 - 8.4	1.2	0.3 - 3.5	1.0	0.3 - 2.9	0.0	0.0 - 0.0	11.8	4.2 - 27.2	0.78
3070103	7072	OCMULGEE R	76.8	25.4	8 - 45	0.0	0.0 - 0.0	5.2	1.6 - 9.3	2.1	0.5 - 5.8	2.0	0.6 - 3.3	0.0	0.0 - 0.0	16.2	5.0 - 33.9	0.84
3070103	7073	OCMULGEE R	42.7	22.9	8 - 53	0.0	0.0 - 0.0	3.8	1.4 - 8.3	2.2	0.8 - 5.4	1.7	0.6 - 4.3	0.0	0.0 - 0.0	15.1	5.5 - 40.5	0.83
3070103	7074	OCMULGEE R	65.3	24.0	7 - 43	0.0	0.0 - 0.0	3.7	1.1 - 6.8	1.3	0.4 - 3.7	4.8	1.3 - 12.3	0.0	0.0 - 0.0	14.2	5.1 - 32.5	0.82
3070103	7075	KINNARD CR	72.8	23.5	8 - 40	0.0	0.0 - 0.0	3.9	1.2 - 7.4	3.9	1.2 - 8.2	3.8	1.2 - 8.5	0.0	0.0 - 0.0	11.9	4.2 - 24.0	0.81
3070103	7076	OCMULGEE R	29.7	17.1	6 - 38	0.0	0.0 - 0.0	1.8	0.5 - 3.8	0.9	0.3 - 2.7	1.8	0.6 - 5.2	0.0	0.0 - 0.0	12.5	4.1 - 27.6	0.81
3070103	7077	OCMULGEE R	1.2	27.9	10 - 61	0.0	0.0 - 0.0	12.7	4.4 - 31.6	1.3	0.4 - 3.9	1.1	0.3 - 3.1	0.0	0.0 - 0.0	12.9	4.1 - 31.1	0.81
3070103	7078	HERDS CR	62.3	24.8	8 - 46	0.0	0.0 - 0.0	3.6	1.0 - 7.6	5.3	1.5 - 15.1	4.6	1.4 - 10.2	0.0	0.0 - 0.0	11.3	3.7 - 21.5	0.81
3070103	7079	OCMULGEE R	1.6	24.3	8 - 58	0.0	0.0 - 0.0	8.7	2.9 - 22.7	1.4	0.3 - 4.2	0.9	0.3 - 2.4	0.0	0.0 - 0.0	13.4	4.4 - 32.2	0.81
3070103	7080	ALCOVY R	29.0	38.1	10 - 88	0.0	0.0 - 0.0	12.1	3.4 - 27.1	2.8	0.6 - 9.2	9.7	2.7 - 24.3	0.0	0.0 - 0.0	13.4	3.9 - 35.0	0.55
3070103	7081	ALCOVY R	108.2	41.5	15 - 99	0.0	0.0 - 0.0	16.3	6.0 - 34.6	3.8	1.2 - 11.2	7.8	2.6 - 21.1	0.0	0.0 - 0.0	13.6	4.4 - 34.6	0.54
3070103	7082	MOUNTAIN CR	35.8	40.1	13 - 77	0.0	0.0 - 0.0	8.6	2.5 - 16.6	5.4	1.7 - 13.9	11.6	3.9 - 28.2	0.0	0.0 - 0.0	14.4	4.8 - 30.3	0.50
3070103	7083	ALCOVY R	24.8	35.1	12 - 76	0.0	0.0 - 0.0	10.4	3.4 - 21.3	3.3	1.0 - 9.3	6.3	2.2 - 16.2	0.0	0.0 - 0.0	15.1	5.2 - 35.3	0.50
3070103	7084	ALCOVY R	159.1	57.3	20 - 112	0.0	0.0 - 0.0	33.6	11.0 - 70.1	1.1	0.4 - 2.8	6.9	2.6 - 17.9	0.0	0.0 - 0.0	15.7	5.9 - 33.7	0.48
3070103	7085	BAY CR	39.7	44.2	14 - 102	0.0	0.0 - 0.0	15.9	5.4 - 38.9	2.8	0.9 - 6.8	9.6	3.4 - 26.7	0.0	0.0 - 0.0	15.9	5.3 - 37.2	0.48
3070103	7086	CORNISH CR	77.0	37.1	12 - 64	0.0	0.0 - 0.0	7.3	2.3 - 13.5	4.7	1.3 - 10.7	12.5	3.8 - 27.5	0.0	0.0 - 0.0	12.7	4.1 - 23.7	0.55
3070103	7087	GUM CR	81.4	35.2	13 - 58	0.0	0.0 - 0.0	9.2	2.9 - 16.2	3.8	1.2 - 10.2	11.8	3.8 - 26.6	0.0	0.0 - 0.0	10.5	4.2 - 21.9	0.54
3070103	7088	YELLOW R	9.3	38.5	11 - 84	0.0	0.0 - 0.0	14.3	3.8 - 36.0	2.5	0.7 - 7.3	10.9	3.5 - 29.0	0.0	0.0 - 0.0	10.8	3.6 - 26.4	0.54
3070103	7089	BIG HAYNES CR	12.4	32.2	10 - 83	0.0	0.0 - 0.0	13.3	3.7 - 32.3	1.3	0.3 - 3.8	6.6	2.2 - 20.5	0.0	0.0 - 0.0	11.0	3.6 - 30.9	0.54
3070103	7090	LITTLE HAYNES C	68.9	42.3	15 - 84	0.0	0.0 - 0.0	15.1	5.2 - 31.2	4.3	1.3 - 10.1	9.9	3.2 - 20.6	0.0	0.0 - 0.0	12.9	4.3 - 30.0	0.51
3070103	7091	BIG HAYNES CR	48.8	34.4	13 - 71	0.0	0.0 - 0.0	11.5	4.5 - 22.1	1.7	0.6 - 4.9	8.6	3.0 - 22.3	0.0	0.0 - 0.0	12.6	4.6 - 28.6	0.51
3070103	7092	BRUSHY FK CR	10.4	51.1	19 - 87	0.0	0.0 - 0.0	16.4	5.6 - 31.3	3.7	1.1 - 9.5	15.3	6.0 - 39.5	0.0	0.0 - 0.0	15.7	6.3 - 31.8	0.46
3070103	7093	BIG HAYNES CR	6.9	52.8	16 - 119	0.0	0.0 - 0.0	18.5	5.8 - 46.9	1.9	0.5 - 4.5	16.3	4.7 - 41.3	0.0	0.0 - 0.0	16.1	5.5 - 40.1	0.46
3070103	7094	YELLOW R	8.6	38.1	12 - 96	0.0	0.0 - 0.0	22.6	6.1 - 55.3	0.3	0.1 - 1.1	2.1	0.6 - 6.0	0.0	0.0 - 0.0	13.0	4.1 - 35.8	0.54
3070103	7095	YELLOW R	1.2	63.1	22 - 151	0.0	0.0 - 0.0	43.4	13.2 - 112.5	0.3	0.1 - 0.7	0.9	0.3 - 2.5	0.0	0.0 - 0.0	18.5	6.9 - 45.2	0.50
3070103	7096	YELLOW R	142.2	95.9	33 - 183	0.0	0.0 - 0.0	78.3	25.8 - 147.8	0.2	0.1 - 0.7	1.9	0.6 - 4.3	0.0	0.0 - 0.0	15.5	5.4 - 33.2	0.47
3070103	7098	BEAVER RUIN CR	4.4	96.2	29 - 181	0.0	0.0 - 0.0	71.2	21.1 - 136.9	0.2	0.0 - 0.5	0.8	0.3 - 2.1	0.0	0.0 - 0.0	24.1	6.5 - 59.5	0.47
3070103	7099	FOX CR	62.6	107.2	34 - 220	8.0	2.6 - 21.3	83.4	27.4 - 180.2	0.3	0.1 - 0.7	2.3	0.8 - 5.6	0.0	0.0 - 0.0	13.3	4.4 - 35.2	0.45
3070103	7100	BEAVER RUIN CR	59.6	116.6	41 - 259	0.0	0.0 - 0.0	93.6	32.0 - 204.7	0.2	0.1 - 0.4	1.3	0.5 - 2.9	0.0	0.0 - 0.0	21.4	7.6 - 51.9	0.45
3070103	7101	JACKSON CR	56.3	123.0	48 - 315	9.7	3.7 - 26.1	82.5	29.8 - 208.8	0.2	0.1 - 0.7	1.8	0.6 - 4.5	0.0	0.0 - 0.0	28.8	10.6 - 78.3	0.47
3070103	7102	STONE MOUNTAIN	74.1	74.1	24 - 185	0.0	0.0 - 0.0	54.9	18.0 - 129.6	0.1	0.0 - 0.2	1.4	0.4 - 3.8	0.0	0.0 - 0.0	17.8	5.5 - 45.8	0.50
3070103	7103	SNAPPING SHOALS	1.7	26.1	9 - 55	0.0	0.0 - 0.0	12.1	3.9 - 27.4	0.9	0.2 - 2.7	2.1	0.7 - 5.4	0.0	0.0 - 0.0	11.0	3.8 - 30.2	0.57
3070103	7104	SNAPPING SHOALS	89.8	61.1	21 - 159	4.3	1.6 - 11.3	37.2	11.8 - 95.8	1.1	0.3 - 3.2	5.9	1.9 - 17.5	0.0	0.0 - 0.0	12.6	4.6 - 37.8	0.56
3070103	7105	SAMPSON CR	12.1	40.0	14 - 90	0.0	0.0 - 0.0	15.0	5.0 - 32.5	2.1	0.6 - 5.2	8.6	2.9 - 21.7	0.0	0.0 - 0.0	14.3	4.9 - 31.4	0.56
3070103	7106	SOUTH R	40.8	33.3	11 - 66	0.0	0.0 - 0.0	6.0	1.7 - 13.6	2.4	0.7 - 5.6	9.1	2.9 - 21.3	0.0	0.0 - 0.0	15.9	5.2 - 38.9	0.57
3070103	7107	SOUTH R	11.0	33.6	12 - 62	0.0	0.0 - 0.0	4.7	1.4 - 9.4	1.8	0.5 - 5.0	11.0	3.8 - 27.2	0.0	0.0 - 0.0	16.1	5.4 - 32.3	0.56
3070103	7108	SOUTH R	44.5	51.2	22 - 113	5.1	2.2 - 12.8	19.2	7.1 - 41.9	1.4	0.6 - 4.2	9.3	3.6 - 27.2	0.0	0.0 - 0.0	16.1	7.1 - 41.3	0.55

8-digit HUC	ID	Name	Area	Catchment Yield		Point sources		Developed Land		Manure		Agricultural Land		Phosphate Mines		Soil parent rock		Frac
				kg/km^2	90% CI	kg/km^2	90% CI	kg/km^2	90% CI	kg/km^2	90% CI	kg/km^2	90% CI	kg/km^2	90% CI	kg/km^2	90% CI	
3070103	7109	HONEY CR	73.0	53.9	15 - 92	10.7	3.4 - 19.5	25.7	7.2 - 51.0	0.5	0.1 - 1.4	4.9	1.4 - 11.4	0.0	0.0 - 0.0	12.1	3.7 - 23.7	0.54
3070103	7110	SOUTH R	53.9	31.6	10 - 81	0.0	0.0 - 0.0	10.8	3.0 - 29.7	0.6	0.2 - 2.7	5.5	1.6 - 15.6	0.0	0.0 - 0.0	14.7	4.5 - 37.4	0.54
3070103	7111	POLE BRIDGE CR	56.0	63.6	20 - 126	0.0	0.0 - 0.0	50.6	15.9 - 102.1	0.1	0.0 - 0.2	1.5	0.4 - 3.9	0.0	0.0 - 0.0	11.4	3.5 - 27.2	0.51
3070103	7112	SOUTH R	162.0	132.0	46 - 318	44.6	15.8 - 108.5	64.7	21.0 - 138.7	0.3	0.1 - 0.8	2.0	0.7 - 5.8	0.0	0.0 - 0.0	20.5	7.7 - 52.7	0.51
3070103	7113	SOUTH R	2.1	95.3	31 - 242	0.0	0.0 - 0.0	77.5	25.7 - 198.4	0.0	0.0 - 0.1	0.9	0.3 - 2.5	0.0	0.0 - 0.0	16.9	5.2 - 38.3	0.48
3070103	7114	COBB CR	50.5	113.7	36 - 230	0.0	0.0 - 0.0	85.9	28.0 - 188.2	0.0	0.0 - 0.1	0.6	0.2 - 1.4	0.0	0.0 - 0.0	27.2	8.9 - 60.5	0.47
3070103	7115	SOUTH R	163.8	149.0	50 - 299	33.3	11.6 - 79.8	81.7	28.9 - 164.3	0.1	0.0 - 0.3	1.2	0.4 - 3.4	0.0	0.0 - 0.0	32.7	11.0 - 80.3	0.47
3070103	7116	CONLEY CR	39.5	74.3	29 - 147	0.0	0.0 - 0.0	52.0	18.5 - 105.4	0.4	0.1 - 1.2	2.8	1.2 - 6.7	0.0	0.0 - 0.0	19.1	7.6 - 48.0	0.48
3070103	7117	BIG COTTON CR	26.6	47.9	21 - 82	0.0	0.0 - 0.0	15.2	5.6 - 28.5	2.5	0.8 - 5.7	14.2	5.5 - 30.6	0.0	0.0 - 0.0	15.9	6.2 - 29.2	0.55
3070103	7118	BIG COTTON CR	20.1	47.1	15 - 91	0.0	0.0 - 0.0	15.4	5.3 - 30.9	2.3	0.7 - 6.5	13.5	4.6 - 35.5	0.0	0.0 - 0.0	15.9	5.8 - 36.0	0.53
3070103	7119	JAMES CR	35.9	48.6	17 - 106	0.0	0.0 - 0.0	24.6	8.2 - 57.7	1.4	0.5 - 4.3	7.7	2.6 - 19.9	0.0	0.0 - 0.0	14.8	5.4 - 35.4	0.50
3070103	7120	BIG COTTON CR	92.3	91.9	30 - 205	12.5	3.8 - 32.8	55.6	18.7 - 143.5	0.6	0.1 - 1.6	4.0	1.3 - 11.0	0.0	0.0 - 0.0	19.3	5.6 - 45.7	0.50
3070103	7121	INDIAN CR	78.9	73.6	23 - 192	0.0	0.0 - 0.0	51.6	14.4 - 130.5	0.6	0.2 - 2.0	3.4	1.1 - 10.0	0.0	0.0 - 0.0	18.0	5.6 - 42.0	0.50
3070103	7122	*A	46.0	50.9	18 - 95	0.0	0.0 - 0.0	30.5	10.8 - 61.4	0.6	0.2 - 1.4	3.6	1.4 - 9.0	0.0	0.0 - 0.0	16.2	5.8 - 37.8	0.50
3070103	7123	WALNUT CR	182.1	51.4	18 - 116	1.4	0.5 - 2.7	23.8	7.6 - 57.9	1.8	0.6 - 5.1	9.7	2.9 - 30.7	0.0	0.0 - 0.0	14.8	4.6 - 32.0	0.56
3070103	7124	YELLOW WATER (	80.9	43.8	13 - 109	6.2	1.7 - 14.5	10.8	3.0 - 25.3	1.5	0.4 - 4.6	10.2	2.4 - 29.7	0.0	0.0 - 0.0	15.1	4.1 - 41.1	0.81
3070103	7125	BIG SANDY CR	10.5	27.4	10 - 53	0.0	0.0 - 0.0	6.2	1.9 - 14.8	1.0	0.3 - 2.6	2.1	0.8 - 5.3	0.0	0.0 - 0.0	18.0	6.7 - 42.9	0.82
3070103	7126	BIG SANDY CR	99.8	31.2	9 - 85	2.6	0.7 - 7.1	7.4	2.2 - 19.9	1.0	0.2 - 3.3	5.3	1.8 - 15.9	0.0	0.0 - 0.0	14.9	4.2 - 44.2	0.80
3070103	7127	ROCKY CR	38.0	25.7	9 - 46	0.0	0.0 - 0.0	3.0	1.0 - 5.9	2.9	0.9 - 6.8	5.7	1.9 - 12.6	0.0	0.0 - 0.0	14.1	4.8 - 33.3	0.80
3070103	7128	TOWALIGA CR	181.8	31.0	10 - 88	0.0	0.0 - 0.0	3.8	1.3 - 9.6	3.8	1.2 - 12.8	3.9	1.3 - 10.0	0.0	0.0 - 0.0	19.4	6.3 - 51.9	0.83
3070103	7129	TOWALIGA CR	61.0	29.4	10 - 78	0.0	0.0 - 0.0	5.4	1.8 - 14.1	2.6	0.7 - 8.9	4.1	1.4 - 13.0	0.0	0.0 - 0.0	17.3	5.9 - 48.8	0.80
3070103	7130	TOWALIGA CR	15.1	38.8	14 - 68	0.0	0.0 - 0.0	10.1	3.5 - 20.6	2.0	0.6 - 5.6	10.0	3.2 - 25.8	0.0	0.0 - 0.0	16.7	5.5 - 33.4	0.78
3070103	7131	TOWALIGA CR	41.3	35.5	10 - 87	0.0	0.0 - 0.0	6.5	1.8 - 16.8	1.6	0.5 - 5.6	10.8	2.9 - 26.5	0.0	0.0 - 0.0	16.6	4.7 - 42.7	0.76
3070103	7132	INDIAN CR	71.6	45.5	15 - 119	0.0	0.0 - 0.0	20.3	7.4 - 58.3	1.8	0.5 - 6.1	8.5	2.6 - 28.1	0.0	0.0 - 0.0	14.9	5.4 - 40.0	0.73
3070103	7133	TOWALIGA CR	177.8	33.8	11 - 63	0.0	0.0 - 0.0	7.5	2.7 - 17.2	1.8	0.5 - 5.6	10.3	3.2 - 28.1	0.0	0.0 - 0.0	14.2	4.8 - 30.9	0.73
3070103	7134	CABIN CR	87.7	42.6	17 - 93	4.3	1.8 - 9.1	15.1	5.5 - 36.1	1.3	0.4 - 4.3	8.9	3.6 - 22.7	0.0	0.0 - 0.0	13.0	4.9 - 34.0	0.76
3070103	7135	BUCK CR	127.8	35.3	13 - 63	0.0	0.0 - 0.0	7.9	2.6 - 15.7	2.7	0.8 - 8.2	10.0	3.6 - 23.7	0.0	0.0 - 0.0	14.6	5.4 - 32.4	0.78
3070103	7136	LITTLE TOWALIG	67.1	35.3	13 - 68	0.0	0.0 - 0.0	4.2	1.3 - 9.5	3.4	1.0 - 9.4	5.2	1.7 - 12.4	0.0	0.0 - 0.0	22.5	7.4 - 49.0	0.80
3070103	7137	EDIE CR	53.7	38.4	13 - 63	0.0	0.0 - 0.0	7.5	2.5 - 14.3	5.0	1.5 - 13.4	9.8	2.4 - 23.0	0.0	0.0 - 0.0	16.0	5.4 - 32.3	0.71
3070103	7138	LITTLE TOWALIG	42.3	34.9	13 - 90	0.0	0.0 - 0.0	10.8	3.8 - 31.3	3.1	0.9 - 10.1	5.5	2.3 - 15.5	0.0	0.0 - 0.0	15.5	5.4 - 45.1	0.71
3070103	7139	RUM CR	9.5	33.6	11 - 79	0.0	0.0 - 0.0	6.9	2.3 - 15.9	3.2	1.0 - 9.6	2.8	1.0 - 7.2	0.0	0.0 - 0.0	20.6	6.4 - 51.5	0.84
3070103	7140	RUM CR	100.7	29.4	11 - 73	1.1	0.4 - 3.0	6.8	2.3 - 18.8	4.3	1.2 - 13.8	4.5	1.8 - 13.2	0.0	0.0 - 0.0	12.7	4.2 - 31.0	0.81
3070103	7141	DEE R CR	81.1	25.2	9 - 73	0.0	0.0 - 0.0	4.7	1.5 - 11.0	4.3	1.3 - 14.6	4.6	1.5 - 14.6	0.0	0.0 - 0.0	11.6	4.2 - 32.2	0.81
3070103	7142	TOBESOFKEE R	28.3	27.5	10 - 58	0.0	0.0 - 0.0	9.8	3.5 - 24.3	0.9	0.3 - 2.5	5.6	2.1 - 14.1	0.0	0.0 - 0.0	11.2	3.7 - 28.0	0.86
3070103	7143	ROCKY CR	131.4	286.5	109 - 683	237.9	90.0 - 601.3	34.0	12.6 - 72.8	1.2	0.4 - 3.3	2.4	0.8 - 6.6	0.0	0.0 - 0.0	11.0	3.6 - 24.8	0.85
3070103	7144	TOBESOFKEE R	6.6	13.2	5 - 26	0.0	0.0 - 0.0	5.4	1.7 - 12.3	0.4	0.1 - 1.2	1.9	0.7 - 4.5	0.0	0.0 - 0.0	5.5	1.7 - 13.7	0.85
3070103	7145	TOBESOFKEE R	237.9	42.2	18 - 73	11.1	4.8 - 22.6	5.9	2.3 - 12.4	4.7	1.6 - 12.2	6.4	2.4 - 13.5	0.0	0.0 - 0.0	14.0	6.1 - 27.4	0.41
3070103	7146	LITTLE TOBESOFK	115.5	24.2	8 - 60	0.0	0.0 - 0.0	2.3	0.8 - 5.4	4.7	1.3 - 12.2	5.4	1.6 - 12.8	0.0	0.0 - 0.0	11.8	4.2 - 31.2	0.41
3070103	7147	WOLF CR	20.9	49.2	15 - 89	0.0	0.0 - 0.0	3.8	1.1 - 7.3	9.1	2.5 - 18.7	17.4	5.3 - 35.7	0.0	0.0 - 0.0	18.9	6.0 - 40.4	0.33

8-digit HUC	ID	Name	Area	Catchment Yield kg/km^2	Catchment Yield 90% CI	Point sources kg/km^2	Point sources 90% CI	Developed Land kg/km^2	Developed Land 90% CI	Manure kg/km^2	Manure 90% CI	Agricultural Land kg/km^2	Agricultural Land 90% CI	Phosphate Mines kg/km^2	Phosphate Mines 90% CI	Soil parent rock kg/km^2	Soil parent rock 90% CI	Frac
3070103	7148	LITTLE TOBESOFK	22.0	45.7	15 - 79	0.0	0.0 - 0.0	7.1	2.3 - 14.3	6.3	1.8 - 16.6	12.9	3.9 - 28.2	0.0	0.0 - 0.0	19.4	6.0 - 41.7	0.33
3070103	7149	ECHECONNEE CR	190.2	30.0	11 - 75	0.0	0.0 - 0.0	15.3	4.9 - 35.4	0.9	0.3 - 2.8	5.4	2.1 - 15.5	0.0	0.0 - 0.0	8.4	3.0 - 20.7	0.87
3070103	7150	ECHECONNEE CR	39.9	24.7	10 - 55	0.0	0.0 - 0.0	5.8	2.1 - 14.8	1.4	0.5 - 4.4	8.3	3.0 - 20.1	0.0	0.0 - 0.0	9.1	3.5 - 21.1	0.80
3070103	7151	ECHECONNEE CR	31.7	31.4	10 - 62	0.0	0.0 - 0.0	4.8	1.5 - 10.1	2.0	0.6 - 5.0	8.1	2.6 - 18.5	0.0	0.0 - 0.0	16.6	5.5 - 32.9	0.77
3070103	7152	ECHECONNEE CR	221.1	26.5	9 - 54	0.0	0.0 - 0.0	3.3	1.2 - 6.9	3.8	1.1 - 10.1	6.1	2.0 - 15.7	0.0	0.0 - 0.0	13.2	4.7 - 33.8	0.73
3070103	7153	LITTLE ECHECONI	55.1	20.8	7 - 53	0.0	0.0 - 0.0	2.6	0.8 - 6.3	0.6	0.2 - 1.8	2.1	0.7 - 5.8	0.0	0.0 - 0.0	15.6	4.9 - 50.1	0.73
3070103	7154	SWEETWATER CR	51.6	17.3	6 - 42	0.0	0.0 - 0.0	4.5	1.5 - 11.0	0.9	0.3 - 2.9	4.0	1.4 - 10.4	0.0	0.0 - 0.0	7.9	2.9 - 21.1	0.77
3070103	7155	DEEP CR	72.7	12.0	4 - 32	0.0	0.0 - 0.0	3.0	1.0 - 7.9	0.6	0.2 - 1.8	2.5	0.9 - 8.8	0.0	0.0 - 0.0	6.0	2.3 - 16.5	0.80
3070103	7156	INDIAN CR	27.9	58.2	19 - 115	3.6	1.2 - 8.0	30.1	10.1 - 67.3	1.6	0.5 - 4.5	7.1	2.1 - 15.9	0.0	0.0 - 0.0	15.9	4.8 - 35.7	0.53
3070104	7157	OCMULGEE R	166.5	30.8	9 - 58	7.0	2.1 - 17.1	10.1	3.1 - 21.7	0.4	0.1 - 1.3	5.1	1.5 - 14.7	0.0	0.0 - 0.0	8.3	2.4 - 17.5	0.96
3070104	7158	OCMULGEE R	3.4	21.1	7 - 51	0.0	0.0 - 0.0	8.5	2.4 - 20.9	0.3	0.1 - 0.9	0.2	0.1 - 0.6	0.0	0.0 - 0.0	12.1	3.8 - 34.2	0.96
3070104	7159	BIG HORSE CR	130.1	16.8	5 - 37	0.0	0.0 - 0.0	3.1	1.0 - 7.2	0.7	0.2 - 2.0	5.7	1.8 - 16.6	0.0	0.0 - 0.0	7.2	2.7 - 15.6	0.95
3070104	7160	HORSE CR	197.4	18.8	6 - 50	0.0	0.0 - 0.0	4.0	1.0 - 9.9	0.9	0.2 - 2.3	7.0	2.2 - 20.3	0.0	0.0 - 0.0	6.9	2.0 - 19.1	0.89
3070104	7161	ALLIGATOR CR	76.1	18.1	6 - 39	0.0	0.0 - 0.0	3.6	1.1 - 7.3	0.8	0.2 - 2.2	6.9	2.4 - 17.1	0.0	0.0 - 0.0	6.9	2.1 - 15.8	0.89
3070104	7162	OCMULGEE R	359.0	18.0	6 - 38	0.0	0.0 - 0.0	2.7	0.9 - 6.7	1.7	0.5 - 4.9	4.6	1.5 - 10.8	0.0	0.0 - 0.0	9.0	3.0 - 24.0	0.95
3070104	7163	OCMULGEE R	104.0	17.8	7 - 38	0.0	0.0 - 0.0	2.1	0.8 - 5.0	0.9	0.3 - 2.6	5.2	1.8 - 12.7	0.0	0.0 - 0.0	9.7	3.4 - 21.6	0.94
3070104	7165	OCMULGEE R	258.7	21.0	8 - 37	0.0	0.0 - 0.0	3.3	1.2 - 5.9	1.3	0.4 - 4.1	7.6	2.4 - 17.7	0.0	0.0 - 0.0	8.8	3.2 - 16.5	0.93
3070104	7166	CROOKED CR	171.3	19.6	7 - 33	0.0	0.0 - 0.0	4.1	1.3 - 7.2	1.2	0.3 - 2.9	7.2	2.2 - 16.0	0.0	0.0 - 0.0	7.1	2.5 - 14.6	0.92
3070104	7167	OCMULGEE R	247.0	19.8	6 - 42	0.0	0.0 - 0.0	4.0	1.3 - 8.8	1.6	0.5 - 4.4	6.3	2.0 - 18.2	0.0	0.0 - 0.0	7.9	3.0 - 18.9	0.92
3070104	7168	OCMULGEE R	26.2	21.8	9 - 38	0.0	0.0 - 0.0	2.2	0.8 - 4.1	0.8	0.2 - 1.7	8.0	3.1 - 17.2	0.0	0.0 - 0.0	10.7	3.8 - 23.0	0.91
3070104	7169	MOSQUITO CR	186.0	21.6	6 - 42	0.0	0.0 - 0.0	4.4	1.2 - 9.1	1.0	0.3 - 2.9	9.5	2.9 - 23.0	0.0	0.0 - 0.0	6.7	2.0 - 15.5	0.91
3070104	7170	OCMULGEE R	17.0	24.4	8 - 48	0.0	0.0 - 0.0	3.4	1.2 - 7.3	1.9	0.6 - 5.7	9.1	3.1 - 23.0	0.0	0.0 - 0.0	10.1	3.6 - 20.6	0.91
3070104	7171	OCMULGEE R	43.5	33.2	11 - 60	0.0	0.0 - 0.0	5.9	1.8 - 11.9	0.5	0.1 - 1.2	18.5	5.7 - 40.7	0.0	0.0 - 0.0	8.4	2.4 - 16.3	0.90
3070104	7172	OCMULGEE R	13.6	25.7	8 - 64	0.0	0.0 - 0.0	3.6	1.1 - 8.0	0.4	0.1 - 1.2	13.5	4.2 - 39.4	0.0	0.0 - 0.0	8.2	2.7 - 19.0	0.90
3070104	7173	*A	155.5	26.8	11 - 52	0.0	0.0 - 0.0	6.0	2.3 - 12.5	1.3	0.4 - 3.3	12.8	4.3 - 33.6	0.0	0.0 - 0.0	6.7	2.4 - 18.3	0.90
3070104	7174	OCMULGEE R	80.7	32.5	13 - 71	0.0	0.0 - 0.0	14.1	4.9 - 30.4	0.3	0.1 - 1.0	11.1	4.5 - 28.7	0.0	0.0 - 0.0	6.9	2.4 - 19.1	0.90
3070104	7175	JORDAN CR	74.9	28.5	9 - 69	0.0	0.0 - 0.0	10.6	3.2 - 25.0	1.2	0.4 - 3.1	10.5	3.2 - 30.8	0.0	0.0 - 0.0	6.1	2.2 - 16.2	0.89
3070104	7176	OCMULGEE R	77.1	23.4	9 - 44	0.0	0.0 - 0.0	3.6	1.3 - 6.7	0.6	0.2 - 1.5	9.4	3.2 - 21.7	0.0	0.0 - 0.0	9.6	3.5 - 19.6	0.89
3070104	7177	OCMULGEE R	15.4	21.1	8 - 44	0.0	0.0 - 0.0	1.4	0.5 - 2.6	0.9	0.3 - 2.4	5.2	1.9 - 13.3	0.0	0.0 - 0.0	13.6	5.1 - 31.0	0.89
3070104	7178	SHELLSTONE CR	1.4	16.5	5 - 39	0.0	0.0 - 0.0	0.0	0.0 - 0.0	0.0	0.0 - 0.0	0.0	0.0 - 0.0	0.0	0.0 - 0.0	16.5	4.8 - 38.7	0.89
3070104	7179	S SHELLSTONE CR	29.3	17.7	5 - 53	0.0	0.0 - 0.0	2.0	0.7 - 4.9	1.0	0.3 - 3.1	7.4	2.3 - 22.2	0.0	0.0 - 0.0	7.3	2.3 - 20.5	0.87
3070104	7180	S SHELLSTONE CR	28.8	26.6	9 - 70	0.0	0.0 - 0.0	7.3	2.4 - 20.2	1.6	0.5 - 5.3	11.6	4.1 - 32.8	0.0	0.0 - 0.0	6.1	2.2 - 19.7	0.76
3070104	7181	EVERGREEN CR	38.0	22.9	8 - 45	0.0	0.0 - 0.0	4.2	1.4 - 8.7	1.5	0.5 - 4.0	11.3	3.9 - 27.4	0.0	0.0 - 0.0	5.9	1.9 - 14.6	0.76
3070104	7182	SHELLSTONE CR	120.9	11.6	4 - 25	0.0	0.0 - 0.0	2.4	0.8 - 4.9	0.3	0.1 - 0.9	3.9	1.2 - 9.5	0.0	0.0 - 0.0	4.9	1.5 - 11.2	0.87
3070104	7183	OCMULGEE R	140.7	18.6	6 - 39	0.0	0.0 - 0.0	3.4	1.2 - 7.3	0.5	0.1 - 1.4	1.6	0.6 - 4.3	0.0	0.0 - 0.0	13.1	4.0 - 29.1	0.89
3070104	7184	SAVAGE CR	20.5	10.9	4 - 28	0.0	0.0 - 0.0	2.1	0.7 - 4.0	0.1	0.0 - 0.4	0.1	0.1 - 0.4	0.0	0.0 - 0.0	8.5	3.1 - 23.8	0.88
3070104	7185	RICHLAND CR	61.4	12.4	4 - 23	0.0	0.0 - 0.0	3.8	1.2 - 7.9	0.2	0.1 - 0.5	2.2	0.7 - 4.3	0.0	0.0 - 0.0	6.1	2.0 - 12.4	0.78
3070104	7186	SAVAGE CR	88.1	9.9	3 - 26	0.0	0.0 - 0.0	2.3	0.7 - 6.2	0.1	0.0 - 0.4	0.8	0.3 - 2.6	0.0	0.0 - 0.0	6.6	2.2 - 18.3	0.78
3070104	7187	OCMULGEE R	8.8	43.2	17 - 101	0.0	0.0 - 0.0	10.5	4.1 - 23.5	3.3	1.2 - 10.2	14.3	4.9 - 37.4	0.0	0.0 - 0.0	15.1	6.1 - 39.0	0.88

8-digit HUC	ID	Name	Area	Catchment Yield		Point sources		Developed Land		Manure		Agricultural Land		Phosphate Mines		Soil parent rock		Frac
				kg/km^2	90% CI	kg/km^2	90% CI	kg/km^2	90% CI	kg/km^2	90% CI	kg/km^2	90% CI	kg/km^2	90% CI	kg/km^2	90% CI	
3070104	7188	FLAT CR	111.5	11.8	4 - 20	0.0	0.0 - 0.0	2.7	0.9 - 5.1	0.3	0.1 - 0.6	1.9	0.7 - 3.6	0.0	0.0 - 0.0	7.1	2.5 - 15.6	0.88
3070104	7189	OCMULGEE R	5.6	39.4	13 - 97	0.0	0.0 - 0.0	0.5	0.2 - 1.3	3.2	0.9 - 11.8	14.6	4.8 - 39.4	0.0	0.0 - 0.0	21.1	6.4 - 56.5	0.88
3070104	7190	OCMULGEE R	97.7	49.5	18 - 91	13.3	4.3 - 27.8	21.4	7.8 - 39.9	0.3	0.1 - 0.7	1.7	0.6 - 4.0	0.0	0.0 - 0.0	12.8	4.6 - 28.1	0.87
3070104	7191	SANDY RUN CR	33.0	421.1	162 - 1048	381.5	146.6 - 950.9	27.0	9.6 - 55.1	1.0	0.3 - 2.6	3.8	1.4 - 10.1	0.0	0.0 - 0.0	7.8	2.9 - 17.4	0.87
3070104	7192	BAY GALL CR	49.9	51.9	18 - 130	0.0	0.0 - 0.0	39.3	13.4 - 98.6	1.1	0.3 - 3.4	5.5	1.9 - 15.0	0.0	0.0 - 0.0	6.0	2.0 - 16.6	0.79
3070104	7193	SANDY RUN CR	63.2	41.2	15 - 80	0.0	0.0 - 0.0	27.7	9.9 - 56.8	1.1	0.4 - 2.7	6.4	2.4 - 17.3	0.0	0.0 - 0.0	5.9	2.2 - 13.8	0.79
3070104	7195	BIG INDIAN CR	54.0	13.3	5 - 27	0.0	0.0 - 0.0	2.2	0.6 - 4.0	0.4	0.1 - 1.2	0.8	0.3 - 1.7	0.0	0.0 - 0.0	9.8	3.2 - 23.3	0.89
3070104	7196	MOSSY CR	214.7	27.8	8 - 59	0.0	0.0 - 0.0	10.2	2.9 - 22.9	1.6	0.4 - 4.0	8.8	2.6 - 20.6	0.0	0.0 - 0.0	7.1	2.1 - 15.8	0.85
3070104	7197	MULE CR	61.0	25.5	11 - 72	0.0	0.0 - 0.0	5.2	1.9 - 12.6	1.6	0.6 - 5.0	12.6	4.6 - 38.9	0.0	0.0 - 0.0	6.2	2.4 - 16.9	0.66
3070104	7198	INDIAN CR	107.6	21.3	7 - 44	0.0	0.0 - 0.0	4.9	1.5 - 10.8	1.3	0.3 - 3.6	8.7	2.6 - 27.1	0.0	0.0 - 0.0	6.4	1.9 - 16.9	0.66
3070104	7199	BIG INDIAN CR	86.6	19.4	7 - 43	0.0	0.0 - 0.0	3.3	1.1 - 7.5	1.5	0.5 - 3.6	6.0	2.4 - 14.2	0.0	0.0 - 0.0	8.5	3.4 - 19.4	0.85
3070104	7200	BIG INDIAN CR	30.3	179.5	58 - 395	131.3	41.2 - 289.8	35.0	10.4 - 70.6	1.5	0.4 - 4.0	6.1	1.9 - 13.8	0.0	0.0 - 0.0	5.5	1.6 - 12.8	0.80
3070104	7201	BAY CR	80.6	50.1	15 - 113	24.2	7.4 - 60.2	8.9	2.6 - 20.9	1.2	0.4 - 3.2	9.3	2.6 - 25.6	0.0	0.0 - 0.0	6.6	2.3 - 15.5	0.76
3070104	7202	BIG INDIAN CR	33.8	18.2	6 - 39	0.0	0.0 - 0.0	6.4	2.1 - 15.0	1.2	0.3 - 3.5	6.9	2.4 - 17.0	0.0	0.0 - 0.0	3.7	1.0 - 8.7	0.76
3070104	7203	BIG INDIAN CR	98.9	25.4	8 - 53	0.0	0.0 - 0.0	10.0	3.1 - 22.3	1.7	0.5 - 4.3	7.2	2.3 - 17.4	0.0	0.0 - 0.0	6.6	2.2 - 13.5	0.68
3070104	7204	BAPTIST CR	38.7	23.5	7 - 43	0.0	0.0 - 0.0	3.9	1.2 - 7.1	5.0	1.3 - 13.3	9.8	3.2 - 19.7	0.0	0.0 - 0.0	4.8	1.4 - 10.0	0.68
3070104	7205	BIG INDIAN CR	79.0	22.9	10 - 61	0.0	0.0 - 0.0	4.7	1.7 - 12.1	2.5	0.9 - 9.5	9.1	3.9 - 24.5	0.0	0.0 - 0.0	6.6	2.7 - 19.0	0.80
3070104	7206	DRY CR	63.6	20.1	6 - 43	0.0	0.0 - 0.0	4.5	1.2 - 10.1	1.4	0.4 - 4.4	6.4	2.2 - 16.0	0.0	0.0 - 0.0	7.6	2.2 - 16.9	0.89
3070104	7207	BIG CR	30.0	29.8	10 - 67	0.0	0.0 - 0.0	5.8	1.7 - 12.3	0.4	0.1 - 1.2	16.1	5.2 - 40.1	0.0	0.0 - 0.0	7.5	2.3 - 18.8	0.90
3070104	7208	BIG CR	5.0	31.6	10 - 64	0.0	0.0 - 0.0	4.9	1.5 - 11.3	0.5	0.1 - 1.4	18.7	5.8 - 50.4	0.0	0.0 - 0.0	7.5	2.3 - 18.0	0.88
3070104	7209	BIG CR	21.3	21.4	8 - 50	0.0	0.0 - 0.0	2.4	0.8 - 6.5	2.3	0.7 - 6.3	10.7	3.6 - 28.2	0.0	0.0 - 0.0	6.0	2.1 - 16.6	0.85
3070104	7210	BIG CR	176.2	26.3	8 - 63	0.0	0.0 - 0.0	4.0	1.2 - 9.6	2.7	0.7 - 8.4	12.7	3.7 - 31.5	0.0	0.0 - 0.0	6.8	2.1 - 17.8	0.83
3070104	7211	CAMP CR	42.6	33.5	11 - 63	0.0	0.0 - 0.0	6.1	1.8 - 12.0	2.7	0.8 - 6.1	18.5	5.8 - 42.9	0.0	0.0 - 0.0	6.3	2.0 - 12.7	0.83
3070104	7212	CEDAR CR	151.7	34.5	13 - 75	0.0	0.0 - 0.0	4.7	1.6 - 11.6	2.2	0.6 - 6.4	20.8	7.2 - 52.0	0.0	0.0 - 0.0	6.8	2.3 - 15.8	0.88
3070104	7213	BLUFF CR	33.1	33.7	13 - 70	0.0	0.0 - 0.0	6.0	2.1 - 13.2	0.6	0.2 - 1.6	19.6	7.0 - 44.9	0.0	0.0 - 0.0	7.6	2.8 - 18.3	0.90
3070104	7214	TENMILE CR	139.4	22.9	8 - 46	0.0	0.0 - 0.0	3.7	1.1 - 9.2	0.9	0.3 - 2.2	11.6	3.6 - 28.0	0.0	0.0 - 0.0	6.7	2.3 - 12.8	0.87
3070104	7215	BLUFF CR	38.7	29.1	10 - 72	0.0	0.0 - 0.0	5.3	1.5 - 13.7	2.3	0.7 - 5.9	15.3	5.3 - 43.0	0.0	0.0 - 0.0	6.3	2.2 - 17.3	0.87
3070104	7216	CEDAR CR	46.2	27.5	9 - 52	0.0	0.0 - 0.0	3.4	1.0 - 6.4	2.8	0.6 - 8.7	9.3	3.0 - 23.4	0.0	0.0 - 0.0	12.1	3.9 - 29.2	0.91
3070104	7217	CEDAR CR	78.1	28.5	8 - 55	0.0	0.0 - 0.0	3.8	1.2 - 7.8	3.5	0.9 - 9.0	13.9	4.3 - 33.0	0.0	0.0 - 0.0	7.2	2.1 - 16.6	0.86
3070104	7218	BRUSHY CR	93.9	16.2	6 - 46	0.0	0.0 - 0.0	2.7	0.9 - 7.3	1.4	0.4 - 5.4	5.1	1.8 - 15.6	0.0	0.0 - 0.0	7.0	2.6 - 18.7	0.86
3070104	7219	HOUSE CR	20.8	20.4	8 - 40	0.0	0.0 - 0.0	6.2	1.9 - 14.1	1.1	0.4 - 2.7	3.6	1.2 - 9.5	0.0	0.0 - 0.0	9.4	3.8 - 23.9	0.93
3070104	7220	HOUSE CR	214.4	20.4	8 - 41	0.0	0.0 - 0.0	4.8	1.6 - 10.1	2.0	0.7 - 4.3	7.4	2.4 - 17.1	0.0	0.0 - 0.0	6.3	2.3 - 15.5	0.90
3070104	7221	LITTLE HOUSE CR	102.8	18.9	6 - 43	0.0	0.0 - 0.0	3.9	1.3 - 9.3	1.6	0.5 - 4.5	6.8	2.3 - 17.2	0.0	0.0 - 0.0	6.6	2.1 - 17.8	0.90
3070104	7222	OTTER CR	98.3	19.9	7 - 37	0.0	0.0 - 0.0	6.0	1.9 - 13.5	1.2	0.4 - 3.5	5.5	1.8 - 14.5	0.0	0.0 - 0.0	7.3	2.5 - 16.3	0.93
3070104	7223	STURGEON CR	146.2	17.2	5 - 47	0.0	0.0 - 0.0	4.1	1.2 - 10.5	1.1	0.3 - 3.8	4.3	1.2 - 13.3	0.0	0.0 - 0.0	7.7	2.1 - 22.7	0.94
3070104	7224	BIG CR, S PRONG	107.0	33.4	11 - 70	0.0	0.0 - 0.0	7.3	2.6 - 16.8	2.3	0.7 - 5.9	17.7	5.8 - 42.8	0.0	0.0 - 0.0	6.1	1.9 - 14.6	0.85
3070105	7225	LITTLE OCMULGE	26.7	17.6	6 - 41	0.0	0.0 - 0.0	7.2	2.3 - 16.2	0.5	0.2 - 1.7	2.6	0.8 - 8.0	0.0	0.0 - 0.0	7.2	2.7 - 17.1	0.96
3070105	7226	ALLIGATOR CR	93.5	14.4	5 - 34	0.0	0.0 - 0.0	2.1	0.6 - 5.1	0.6	0.2 - 1.7	4.1	1.4 - 10.4	0.0	0.0 - 0.0	7.6	2.6 - 20.6	0.95
3070105	7227	LITTLE CR	72.0	13.9	4 - 27	0.0	0.0 - 0.0	3.0	0.9 - 5.7	0.4	0.1 - 1.1	3.0	0.8 - 7.4	0.0	0.0 - 0.0	7.5	2.2 - 16.6	0.92

8-digit HUC	ID	Name	Area	Catchment Yield		Point sources		Developed Land		Manure		Agricultural Land		Phosphate Mines		Soil parent rock		Frac
				kg/km^2	90% CI	kg/km^2	90% CI	kg/km^2	90% CI	kg/km^2	90% CI	kg/km^2	90% CI	kg/km^2	90% CI	kg/km^2	90% CI	
3070105	7228	ALLIGATOR CR	163.0	17.3	7 - 39	0.0	0.0 - 0.0	4.0	1.4 - 9.4	0.7	0.2 - 1.7	5.2	1.9 - 14.0	0.0	0.0 - 0.0	7.5	2.7 - 19.7	0.92
3070105	7229	LIME SINK CR	148.2	18.7	6 - 33	0.0	0.0 - 0.0	4.5	1.4 - 7.8	0.8	0.3 - 2.0	6.3	2.1 - 13.8	0.0	0.0 - 0.0	7.0	2.2 - 13.7	0.87
3070105	7230	ALLIGATOR CR	172.7	17.3	5 - 34	0.0	0.0 - 0.0	3.9	1.2 - 7.7	0.8	0.2 - 2.3	5.7	1.7 - 13.1	0.0	0.0 - 0.0	6.8	2.1 - 15.1	0.87
3070105	7232	LITTLE OCMULGE	186.3	18.6	7 - 38	0.0	0.0 - 0.0	5.4	1.8 - 11.2	0.6	0.2 - 1.9	4.3	1.4 - 10.3	0.0	0.0 - 0.0	8.2	2.9 - 18.5	0.95
3070105	7233	JOINER CR	73.5	15.3	5 - 35	0.0	0.0 - 0.0	3.6	1.2 - 7.7	0.7	0.2 - 1.7	4.5	1.3 - 11.6	0.0	0.0 - 0.0	6.6	2.1 - 15.1	0.88
3070105	7234	LITTLE OCMULGE	301.7	17.4	5 - 39	0.0	0.0 - 0.0	5.3	1.6 - 11.6	0.9	0.3 - 2.3	4.6	1.4 - 12.2	0.0	0.0 - 0.0	6.5	2.1 - 17.7	0.88
3070105	7235	LITTLE OCMULGE	309.2	20.4	6 - 38	0.0	0.0 - 0.0	4.3	1.3 - 8.0	1.1	0.4 - 3.1	8.8	2.8 - 19.3	0.0	0.0 - 0.0	6.2	2.0 - 12.7	0.74
3070105	7236	*A	65.7	17.6	6 - 44	0.0	0.0 - 0.0	4.1	1.3 - 9.1	1.1	0.3 - 3.4	6.2	2.0 - 16.8	0.0	0.0 - 0.0	6.1	2.0 - 17.4	0.74
3070105	7237	SUGAR CR	15.7	16.5	5 - 34	0.0	0.0 - 0.0	4.3	1.3 - 8.6	0.7	0.2 - 1.8	5.0	1.7 - 12.9	0.0	0.0 - 0.0	6.6	2.1 - 15.9	0.95
3070105	7238	SUGAR CR	251.9	18.7	6 - 42	0.0	0.0 - 0.0	7.5	2.4 - 19.1	0.6	0.2 - 1.7	4.0	1.4 - 10.0	0.0	0.0 - 0.0	6.6	2.1 - 15.5	0.94
3070105	7239	TURNPIKE CR	78.3	15.5	5 - 34	0.0	0.0 - 0.0	3.5	1.1 - 8.0	0.6	0.2 - 1.6	4.3	1.4 - 12.1	0.0	0.0 - 0.0	7.0	2.4 - 17.4	0.94
3070106	7240	ALTAMAHA R	457.7	10.6	3 - 27	0.0	0.0 - 0.0	1.3	0.4 - 3.1	0.2	0.1 - 1.0	0.6	0.2 - 1.5	0.0	0.0 - 0.0	8.4	2.6 - 21.7	1.00
3070106	7241	ALTAMAHA R	22.3	2.1	1 - 5	0.0	0.0 - 0.0	0.4	0.1 - 0.8	0.1	0.0 - 0.2	0.0	0.0 - 0.1	0.0	0.0 - 0.0	1.6	0.5 - 4.0	0.99
3070106	7242	DOCTORS CR	315.2	16.6	6 - 36	0.3	0.1 - 0.8	3.6	1.1 - 8.2	0.9	0.3 - 2.5	2.2	0.7 - 5.5	0.0	0.0 - 0.0	9.5	3.7 - 22.1	0.99
3070106	7243	ALTAMAHA R	40.7	0.9	0 - 2	0.0	0.0 - 0.0	0.1	0.0 - 0.3	0.0	0.0 - 0.1	0.1	0.0 - 0.2	0.0	0.0 - 0.0	0.7	0.2 - 1.8	0.99
3070106	7244	ALTAMAHA R	188.1	11.1	4 - 26	0.0	0.0 - 0.0	1.8	0.5 - 4.2	0.5	0.1 - 1.5	1.7	0.5 - 4.9	0.0	0.0 - 0.0	7.2	2.5 - 17.5	0.98
3070106	7245	MUSHMELON CR	4.7	18.4	6 - 36	0.0	0.0 - 0.0	0.9	0.3 - 1.8	0.9	0.3 - 2.5	1.4	0.5 - 3.3	0.0	0.0 - 0.0	15.2	5.3 - 33.4	0.98
3070106	7246	BEARDS CR	264.2	43.7	14 - 103	0.0	0.0 - 0.0	7.4	2.1 - 16.7	11.1	3.0 - 35.6	15.0	4.8 - 49.6	0.0	0.0 - 0.0	10.1	3.6 - 30.2	0.96
3070106	7247	MUSHMELON CR	201.0	36.8	12 - 68	0.0	0.0 - 0.0	5.5	1.6 - 10.1	8.3	2.1 - 26.3	9.5	3.0 - 21.9	0.0	0.0 - 0.0	13.5	3.4 - 30.3	0.96
3070106	7248	ALTAMAHA R	149.5	21.0	6 - 38	0.0	0.0 - 0.0	3.9	1.1 - 7.0	1.7	0.4 - 4.5	4.7	1.5 - 11.5	0.0	0.0 - 0.0	10.6	3.5 - 23.9	0.98
3070106	7249	ALTAMAHA R	2.2	14.7	5 - 31	0.0	0.0 - 0.0	0.0	0.0 - 0.0	0.4	0.1 - 0.9	0.0	0.0 - 0.0	0.0	0.0 - 0.0	14.4	4.7 - 30.9	0.98
3070106	7250	ALTAMAHA R	235.1	29.7	8 - 72	0.0	0.0 - 0.0	3.4	0.8 - 8.6	4.4	1.1 - 11.9	11.2	3.1 - 26.1	0.0	0.0 - 0.0	10.8	3.2 - 26.9	0.97
3070106	7251	COBB CR	106.4	25.5	10 - 47	0.0	0.0 - 0.0	5.0	1.8 - 10.3	1.9	0.6 - 4.6	10.1	3.8 - 22.3	0.0	0.0 - 0.0	8.5	3.2 - 18.3	0.97
3070106	7252	*A	5.7	28.9	7 - 62	0.0	0.0 - 0.0	6.3	1.5 - 14.6	2.5	0.7 - 6.8	11.2	2.5 - 29.3	0.0	0.0 - 0.0	8.9	2.2 - 22.1	0.85
3070106	7253	*C	25.8	26.3	8 - 67	0.0	0.0 - 0.0	4.3	1.3 - 10.3	1.6	0.5 - 5.1	11.4	3.4 - 30.5	0.0	0.0 - 0.0	9.0	3.3 - 22.9	0.83
3070106	7254	ALTAMAHA R	107.0	29.1	12 - 73	0.0	0.0 - 0.0	5.7	2.1 - 12.4	3.0	0.9 - 11.0	10.3	4.0 - 27.7	0.0	0.0 - 0.0	10.2	4.0 - 27.7	0.97
3070106	7255	*E	40.4	34.1	12 - 78	0.0	0.0 - 0.0	4.3	1.5 - 9.0	3.0	0.8 - 8.7	16.3	5.0 - 44.8	0.0	0.0 - 0.0	10.5	3.5 - 28.6	0.97
3070106	7256	*F	22.7	24.1	8 - 46	0.0	0.0 - 0.0	4.7	1.6 - 10.1	1.7	0.5 - 5.0	9.8	3.0 - 21.3	0.0	0.0 - 0.0	7.9	2.6 - 18.7	0.93
3070106	7257	*G	58.6	25.2	10 - 65	0.0	0.0 - 0.0	6.1	2.2 - 14.5	1.2	0.4 - 3.2	9.7	3.4 - 26.4	0.0	0.0 - 0.0	8.2	2.8 - 20.3	0.93
3070106	7258	ALTAMAHA R	1.1	18.1	6 - 40	0.0	0.0 - 0.0	0.0	0.0 - 0.0	0.3	0.1 - 0.8	0.0	0.0 - 0.0	0.0	0.0 - 0.0	17.8	5.7 - 40.4	0.97
3070106	7259	ALTAMAHA R	87.2	22.4	8 - 48	0.0	0.0 - 0.0	3.8	1.4 - 7.9	1.0	0.4 - 2.7	6.8	2.4 - 17.9	0.0	0.0 - 0.0	10.8	3.9 - 25.5	0.97
3070106	7260	BULLARDS CR	113.1	22.7	8 - 41	0.0	0.0 - 0.0	5.4	1.9 - 9.6	1.2	0.4 - 3.0	8.3	2.8 - 20.9	0.0	0.0 - 0.0	7.7	2.7 - 18.6	0.97
3070106	7261	TENMILE CR	267.3	24.6	9 - 63	0.0	0.0 - 0.0	6.4	2.2 - 15.0	3.0	0.9 - 9.4	7.9	2.6 - 23.1	0.0	0.0 - 0.0	7.3	2.5 - 20.8	0.97
3070106	7262	GOOSE CR	181.0	20.8	6 - 48	0.0	0.0 - 0.0	6.7	1.9 - 14.4	0.8	0.2 - 2.0	8.3	2.4 - 19.0	0.0	0.0 - 0.0	4.9	1.5 - 12.8	0.98
3070106	7263	PENHOLLOWAY C	685.3	11.0	4 - 23	0.0	0.0 - 0.0	3.6	1.1 - 7.2	0.2	0.1 - 0.7	1.0	0.4 - 2.3	0.0	0.0 - 0.0	6.1	2.0 - 15.7	0.99
3070106	7264	*H	28.1	4.2	1 - 10	0.0	0.0 - 0.0	0.3	0.1 - 1.0	0.0	0.0 - 0.0	0.0	0.0 - 0.1	0.0	0.0 - 0.0	3.9	1.1 - 9.5	1.00
3070107	7265	OHOOPEE R	116.8	46.5	14 - 102	0.0	0.0 - 0.0	6.5	1.8 - 15.5	13.3	3.7 - 45.7	15.9	5.0 - 40.0	0.0	0.0 - 0.0	10.7	3.1 - 29.1	0.98
3070107	7266	THOMAS CR	170.1	32.0	11 - 72	0.0	0.0 - 0.0	5.4	1.9 - 13.9	8.2	2.7 - 24.1	9.6	3.0 - 22.3	0.0	0.0 - 0.0	8.8	2.9 - 19.4	0.96
3070107	7267	OHOOPEE R	35.0	60.5	21 - 124	30.5	10.9 - 71.2	6.2	2.2 - 11.2	6.6	2.1 - 17.9	7.6	2.8 - 16.7	0.0	0.0 - 0.0	9.5	3.4 - 20.2	0.96

8-digit HUC	ID	Name	Area	Catchment Yield		Point sources		Developed Land		Manure		Agricultural Land		Phosphate Mines		Soil parent rock		Frac
				kg/km^2	90% CI	kg/km^2	90% CI	kg/km^2	90% CI	kg/km^2	90% CI	kg/km^2	90% CI	kg/km^2	90% CI	kg/km^2	90% CI	
3070107	7268	BRAZELLS CR	85.3	36.4	12 - 66	0.0	0.0 - 0.0	8.7	2.9 - 16.6	9.1	2.7 - 19.9	11.0	4.1 - 23.1	0.0	0.0 - 0.0	7.5	2.6 - 16.2	0.95
3070107	7269	OHOOPEE R	35.1	33.7	13 - 72	0.0	0.0 - 0.0	5.0	1.9 - 9.5	8.1	2.3 - 26.6	12.3	4.3 - 30.9	0.0	0.0 - 0.0	8.2	3.1 - 18.2	0.95
3070107	7270	OHOOPEE R	90.3	29.2	10 - 52	0.0	0.0 - 0.0	3.5	1.1 - 6.9	7.0	2.2 - 20.3	9.2	2.8 - 22.7	0.0	0.0 - 0.0	9.5	3.2 - 21.4	0.94
3070107	7271	BEAVER CR	38.6	29.3	11 - 61	0.0	0.0 - 0.0	5.3	1.8 - 11.2	6.2	1.9 - 17.3	10.7	3.6 - 29.8	0.0	0.0 - 0.0	7.2	2.5 - 16.9	0.92
3070107	7272	OHOOPEE R	81.0	29.2	11 - 47	0.0	0.0 - 0.0	4.1	1.4 - 7.5	3.3	1.0 - 7.9	12.7	4.9 - 25.6	0.0	0.0 - 0.0	9.0	3.1 - 18.9	0.92
3070107	7273	JACKS CR	148.8	19.6	6 - 45	0.0	0.0 - 0.0	5.5	1.4 - 11.5	0.7	0.2 - 1.7	6.3	2.1 - 16.9	0.0	0.0 - 0.0	7.1	2.0 - 18.6	0.90
3070107	7274	OHOOPEE R	44.3	23.9	9 - 63	0.0	0.0 - 0.0	5.8	1.9 - 14.0	1.1	0.3 - 4.0	7.5	2.5 - 22.7	0.0	0.0 - 0.0	9.5	3.6 - 25.9	0.90
3070107	7275	YAM DANDY CR	160.3	29.7	11 - 73	7.2	2.9 - 18.5	11.1	4.2 - 26.0	0.5	0.2 - 1.8	4.1	1.5 - 13.2	0.0	0.0 - 0.0	6.8	2.8 - 19.7	0.88
3070107	7276	OHOOPEE R	49.0	18.5	7 - 44	0.0	0.0 - 0.0	5.0	1.7 - 12.8	0.6	0.2 - 1.9	4.5	1.6 - 13.8	0.0	0.0 - 0.0	8.5	3.2 - 22.2	0.88
3070107	7277	LITTLE OHOOPIE (	65.2	19.1	6 - 42	0.0	0.0 - 0.0	4.5	1.4 - 10.1	0.7	0.2 - 1.9	5.2	2.0 - 13.8	0.0	0.0 - 0.0	8.7	2.9 - 23.4	0.87
3070107	7278	LITTLE OHOOPIE (	110.6	17.9	6 - 39	0.0	0.0 - 0.0	4.6	1.4 - 9.9	0.7	0.2 - 1.8	5.5	2.0 - 13.3	0.0	0.0 - 0.0	7.2	2.5 - 16.4	0.82
3070107	7279	SARDIS CR	52.6	17.5	6 - 29	0.0	0.0 - 0.0	2.8	0.9 - 5.1	0.9	0.3 - 2.3	7.2	2.2 - 15.2	0.0	0.0 - 0.0	6.5	2.1 - 14.0	0.78
3070107	7280	LITTLE OHOOPIE (	14.4	17.5	6 - 42	0.0	0.0 - 0.0	3.8	1.2 - 8.7	0.9	0.3 - 2.7	4.8	1.5 - 12.9	0.0	0.0 - 0.0	8.0	2.7 - 20.1	0.78
3070107	7281	*A	14.2	26.3	10 - 69	0.0	0.0 - 0.0	6.1	2.4 - 16.3	2.0	0.7 - 6.4	11.7	4.1 - 34.9	0.0	0.0 - 0.0	6.5	2.6 - 16.8	0.76
3070107	7282	LITTLE OHOOPIE (	23.0	20.8	6 - 49	0.0	0.0 - 0.0	6.5	2.0 - 16.6	1.2	0.4 - 4.0	5.7	1.7 - 17.0	0.0	0.0 - 0.0	7.4	2.6 - 19.8	0.76
3070107	7283	LITTLE OHOOPIE (	36.1	22.7	7 - 39	0.0	0.0 - 0.0	4.7	1.6 - 8.3	1.6	0.4 - 3.6	9.0	2.7 - 19.0	0.0	0.0 - 0.0	7.4	2.3 - 17.7	0.75
3070107	7284	SMITH CR	31.0	16.6	6 - 31	0.0	0.0 - 0.0	3.8	1.4 - 8.4	0.9	0.3 - 3.5	5.1	1.8 - 12.6	0.0	0.0 - 0.0	6.9	2.4 - 17.4	0.71
3070107	7285	LITTLE OHOOPIE (	33.0	22.5	8 - 54	0.0	0.0 - 0.0	3.1	1.0 - 7.8	1.7	0.5 - 5.4	9.5	3.2 - 22.8	0.0	0.0 - 0.0	8.2	3.1 - 25.1	0.71
3070107	7286	NEALEY CR	34.3	17.8	7 - 40	0.0	0.0 - 0.0	2.2	0.8 - 5.1	0.7	0.2 - 1.8	4.3	1.7 - 11.2	0.0	0.0 - 0.0	10.7	4.0 - 27.0	0.64
3070107	7287	LITTLE OHOOPIE (	119.7	18.9	7 - 38	0.0	0.0 - 0.0	2.5	0.8 - 5.8	0.7	0.2 - 2.0	5.6	2.2 - 16.8	0.0	0.0 - 0.0	10.1	3.7 - 24.6	0.64
3070107	7288	BATTLEGROUND (	60.6	16.1	5 - 28	0.0	0.0 - 0.0	3.2	0.9 - 5.7	1.1	0.3 - 3.1	5.7	1.6 - 14.7	0.0	0.0 - 0.0	6.1	2.1 - 13.0	0.75
3070107	7289	*B	45.4	16.8	7 - 29	0.0	0.0 - 0.0	4.4	1.6 - 8.4	0.7	0.2 - 1.6	5.1	1.6 - 11.1	0.0	0.0 - 0.0	6.7	2.4 - 14.8	0.82
3070107	7290	OHOOPEE R	63.4	19.9	6 - 39	0.0	0.0 - 0.0	4.8	1.4 - 10.5	0.6	0.2 - 1.5	6.1	2.0 - 13.3	0.0	0.0 - 0.0	8.5	2.5 - 18.8	0.87
3070107	7291	MULPEN CR	50.8	15.4	5 - 41	0.0	0.0 - 0.0	2.7	0.9 - 6.3	0.7	0.2 - 2.2	5.0	1.6 - 18.1	0.0	0.0 - 0.0	6.9	2.4 - 19.8	0.83
3070107	7292	OHOOPEE R	143.5	16.2	7 - 27	0.0	0.0 - 0.0	4.1	1.4 - 7.2	0.8	0.3 - 2.0	4.1	1.6 - 9.0	0.0	0.0 - 0.0	7.2	2.9 - 13.7	0.83
3070107	7294	NEELS CR	49.9	14.1	4 - 36	0.0	0.0 - 0.0	2.8	0.8 - 6.5	0.9	0.3 - 3.1	4.3	1.3 - 11.5	0.0	0.0 - 0.0	6.1	1.9 - 16.8	0.77
3070107	7295	OHOOPEE R	34.0	16.8	5 - 35	0.0	0.0 - 0.0	3.0	0.9 - 6.5	1.1	0.3 - 3.5	4.8	1.5 - 13.6	0.0	0.0 - 0.0	7.9	2.7 - 21.3	0.77
3070107	7296	*D	37.0	18.4	7 - 40	0.0	0.0 - 0.0	3.6	1.3 - 7.8	1.3	0.4 - 4.0	7.1	2.4 - 18.3	0.0	0.0 - 0.0	6.3	2.5 - 15.5	0.74
3070107	7297	OHOOPEE R	4.2	15.5	5 - 31	0.0	0.0 - 0.0	4.3	1.5 - 7.9	0.2	0.1 - 0.5	1.3	0.5 - 2.9	0.0	0.0 - 0.0	9.7	3.2 - 22.4	0.74
3070107	7298	BIG CEDAR CR	17.9	29.2	11 - 71	0.0	0.0 - 0.0	9.9	3.6 - 26.7	1.6	0.5 - 5.0	9.4	3.5 - 22.6	0.0	0.0 - 0.0	8.3	2.9 - 21.4	0.73
3070107	7299	LITTLE CEDAR CR	39.3	13.7	6 - 30	0.0	0.0 - 0.0	2.7	1.0 - 6.3	0.8	0.3 - 2.2	4.0	1.4 - 10.3	0.0	0.0 - 0.0	6.2	2.7 - 15.0	0.68
3070107	7300	BIG CEDAR CR	73.6	21.7	9 - 53	0.0	0.0 - 0.0	4.7	1.6 - 11.7	0.9	0.3 - 2.9	6.4	2.5 - 17.7	0.0	0.0 - 0.0	9.6	3.9 - 21.7	0.68
3070107	7301	OHOOPEE R	114.0	22.3	8 - 56	0.0	0.0 - 0.0	3.8	1.1 - 8.9	1.4	0.4 - 4.1	8.5	2.6 - 24.4	0.0	0.0 - 0.0	8.6	2.9 - 21.7	0.73
3070107	7302	OHOOPEE R	62.7	30.0	9 - 64	0.0	0.0 - 0.0	3.9	1.1 - 7.9	1.4	0.4 - 3.6	11.4	3.1 - 28.9	0.0	0.0 - 0.0	13.5	4.1 - 30.8	0.57
3070107	7303	DYERS CR	16.9	21.6	7 - 50	0.0	0.0 - 0.0	3.0	1.1 - 6.0	0.8	0.3 - 2.3	6.7	2.1 - 17.4	0.0	0.0 - 0.0	11.1	3.9 - 28.3	0.57
3070107	7304	*C	61.9	15.4	6 - 41	0.0	0.0 - 0.0	2.8	1.0 - 7.8	1.0	0.3 - 2.5	5.4	1.9 - 15.5	0.0	0.0 - 0.0	6.2	2.5 - 16.8	0.78
3070107	7305	PENDLETON CR	70.7	32.6	12 - 58	7.7	2.7 - 15.1	8.4	2.4 - 18.2	1.6	0.5 - 4.3	6.9	2.4 - 14.9	0.0	0.0 - 0.0	8.0	2.5 - 17.5	0.94
3070107	7306	PENDLETON CR	39.1	29.0	8 - 61	0.0	0.0 - 0.0	5.0	1.4 - 10.6	2.4	0.6 - 7.6	13.9	4.0 - 37.1	0.0	0.0 - 0.0	7.6	2.2 - 18.0	0.92
3070107	7307	*E	35.2	26.4	11 - 52	0.0	0.0 - 0.0	7.8	2.9 - 15.1	1.1	0.4 - 3.1	11.1	4.1 - 27.2	0.0	0.0 - 0.0	6.4	2.3 - 16.4	0.89

8-digit HUC	ID	Name	Area	Catchment Yield		Point sources		Developed Land		Manure		Agricultural Land		Phosphate Mines		Soil parent rock		Frac
				kg/km^2	90% CI	kg/km^2	90% CI	kg/km^2	90% CI	kg/km^2	90% CI	kg/km^2	90% CI	kg/km^2	90% CI	kg/km^2	90% CI	
3070107	7308	PENDLETON CR	37.0	22.5	7 - 45	0.0	0.0 - 0.0	3.9	1.4 - 9.6	1.4	0.4 - 4.3	9.4	3.1 - 20.9	0.0	0.0 - 0.0	7.8	2.7 - 19.7	0.89
3070107	7309	PENDLETON CR	279.3	17.0	6 - 39	0.0	0.0 - 0.0	4.5	1.4 - 10.4	0.6	0.2 - 1.7	5.1	1.6 - 14.1	0.0	0.0 - 0.0	6.8	2.2 - 16.4	0.87
3070107	7310	TIGER CR	173.0	15.5	4 - 31	0.0	0.0 - 0.0	4.0	1.0 - 8.6	0.7	0.2 - 1.7	4.6	1.3 - 13.8	0.0	0.0 - 0.0	6.3	1.6 - 16.7	0.87
3070107	7311	SWIFT CR	146.1	34.7	11 - 76	8.5	2.8 - 18.2	9.5	2.8 - 20.2	1.4	0.4 - 3.7	8.1	2.8 - 22.0	0.0	0.0 - 0.0	7.2	2.4 - 15.1	0.92
3070107	7312	ROCKY CR	81.7	26.5	9 - 45	0.0	0.0 - 0.0	5.7	1.9 - 11.7	2.5	0.7 - 4.9	10.6	3.4 - 23.0	0.0	0.0 - 0.0	7.7	2.4 - 17.3	0.96
3070107	7313	*	45.0	27.4	9 - 47	0.3	0.1 - 0.5	10.0	2.8 - 18.9	1.6	0.4 - 3.9	8.6	2.8 - 19.4	0.0	0.0 - 0.0	7.0	2.1 - 14.6	0.88
3070107	7314	ROCKY CR	96.2	30.1	11 - 66	0.0	0.0 - 0.0	15.5	5.1 - 32.9	1.4	0.4 - 4.3	6.2	2.2 - 14.3	0.0	0.0 - 0.0	7.1	2.4 - 18.1	0.88
3070107	7315	OHOOPEE R	56.4	24.0	8 - 49	0.0	0.0 - 0.0	5.5	1.8 - 12.0	3.0	1.0 - 8.3	7.3	2.4 - 18.1	0.0	0.0 - 0.0	8.3	3.2 - 20.1	0.96
3070201	7316	SATILLA R	156.4	6.1	2 - 14	0.0	0.0 - 0.0	1.2	0.3 - 3.2	0.3	0.1 - 0.8	0.5	0.1 - 1.3	0.0	0.0 - 0.0	4.1	1.3 - 10.0	0.95
3070201	7317	ALABAHA R	270.6	32.5	13 - 67	0.0	0.0 - 0.0	7.5	2.7 - 15.4	1.8	0.5 - 4.4	17.6	7.0 - 44.2	0.0	0.0 - 0.0	5.6	2.2 - 13.1	0.91
3070201	7318	HURRICANE CR	365.4	32.2	10 - 82	2.3	0.7 - 6.5	6.0	1.7 - 15.8	6.2	1.6 - 24.9	13.6	3.8 - 34.0	0.0	0.0 - 0.0	4.2	1.1 - 11.0	0.86
3070201	7319	HURRICANE CR	119.1	28.6	10 - 66	0.0	0.0 - 0.0	4.5	1.8 - 9.3	1.1	0.4 - 3.3	18.7	7.4 - 47.8	0.0	0.0 - 0.0	4.2	1.4 - 10.6	0.70
3070201	7320	WHITEHEAD CR	96.2	21.9	8 - 53	0.0	0.0 - 0.0	4.1	1.5 - 9.8	0.8	0.3 - 3.0	12.8	4.5 - 33.1	0.0	0.0 - 0.0	4.2	1.4 - 11.8	0.70
3070201	7321	LITTLE HURRICAN	434.4	32.0	12 - 65	0.0	0.0 - 0.0	5.0	1.9 - 11.2	6.4	2.3 - 16.0	15.3	6.0 - 40.9	0.0	0.0 - 0.0	5.3	2.0 - 14.6	0.86
3070201	7322	SATILLA R	30.3	27.3	11 - 69	0.0	0.0 - 0.0	4.5	1.6 - 10.2	4.8	1.6 - 12.4	8.2	3.1 - 22.2	0.0	0.0 - 0.0	9.8	3.4 - 24.9	0.91
3070201	7323	SATILLA R	176.6	65.8	23 - 120	25.1	8.9 - 46.7	19.1	6.5 - 43.4	2.2	0.7 - 6.5	12.9	4.2 - 30.9	0.0	0.0 - 0.0	6.4	2.2 - 15.3	0.90
3070201	7324	SATILLA R	64.6	39.6	13 - 77	0.0	0.0 - 0.0	7.7	2.5 - 14.4	3.8	1.1 - 10.1	20.5	6.5 - 49.3	0.0	0.0 - 0.0	7.5	2.7 - 16.7	0.88
3070201	7325	HOG CR	184.4	28.2	10 - 58	0.0	0.0 - 0.0	4.1	1.3 - 9.0	4.3	1.3 - 11.1	13.7	4.8 - 36.2	0.0	0.0 - 0.0	6.1	2.1 - 15.3	0.85
3070201	7326	HOG CR	117.5	39.0	14 - 87	0.0	0.0 - 0.0	4.0	1.1 - 9.6	11.2	3.4 - 32.7	18.8	6.3 - 48.9	0.0	0.0 - 0.0	5.0	1.7 - 13.8	0.77
3070201	7327	HURRICANE CR	135.2	40.7	13 - 73	0.0	0.0 - 0.0	4.8	1.4 - 10.1	11.7	2.5 - 36.5	18.3	6.5 - 42.7	0.0	0.0 - 0.0	5.9	2.0 - 14.0	0.77
3070201	7328	SATILLA R	43.3	31.2	10 - 79	0.0	0.0 - 0.0	6.1	1.8 - 14.8	2.1	0.6 - 6.0	6.2	1.7 - 17.8	0.0	0.0 - 0.0	16.7	4.9 - 54.8	0.85
3070201	7329	SEVENTEENMILE	249.0	70.4	27 - 147	38.8	15.4 - 96.1	5.5	2.0 - 12.4	7.6	2.7 - 21.8	12.2	4.3 - 33.2	0.0	0.0 - 0.0	6.2	2.6 - 13.4	0.85
3070201	7330	OTTER CR	93.6	34.2	13 - 72	0.0	0.0 - 0.0	4.3	1.6 - 8.9	8.3	2.7 - 29.1	13.6	4.6 - 32.2	0.0	0.0 - 0.0	8.0	2.7 - 20.1	0.76
3070201	7331	SEVENTEENMILE	111.3	48.7	16 - 82	0.0	0.0 - 0.0	19.3	5.6 - 37.7	7.3	2.2 - 16.0	12.0	3.7 - 27.3	0.0	0.0 - 0.0	10.2	3.3 - 21.6	0.76
3070201	7332	SEVENTEENMILE	45.0	39.2	13 - 67	0.0	0.0 - 0.0	6.0	1.7 - 10.6	8.7	2.8 - 22.3	14.2	4.5 - 30.6	0.0	0.0 - 0.0	10.4	3.6 - 22.7	0.71
3070201	7333	SEVENTEENMILE	42.2	34.1	13 - 77	0.0	0.0 - 0.0	3.3	1.1 - 6.8	6.4	2.0 - 20.1	16.5	6.2 - 45.2	0.0	0.0 - 0.0	7.9	2.8 - 19.4	0.66
3070201	7334	BROXTON CR	49.7	37.4	11 - 71	0.0	0.0 - 0.0	3.9	1.1 - 6.9	9.3	2.5 - 23.9	15.6	4.4 - 40.6	0.0	0.0 - 0.0	8.7	2.5 - 18.1	0.66
3070201	7335	ROSE CR	12.9	37.8	12 - 75	0.0	0.0 - 0.0	5.8	1.6 - 12.1	7.7	2.1 - 21.5	12.4	4.0 - 30.9	0.0	0.0 - 0.0	11.8	3.5 - 25.0	0.71
3070201	7336	ROSE CR	63.4	40.2	13 - 69	0.0	0.0 - 0.0	4.5	1.4 - 7.7	9.6	2.8 - 24.2	16.7	5.3 - 33.3	0.0	0.0 - 0.0	9.4	3.3 - 18.0	0.69
3070201	7337	HALLS CR	103.8	34.7	12 - 68	0.0	0.0 - 0.0	4.4	1.5 - 8.3	7.4	2.0 - 23.2	13.6	4.3 - 36.1	0.0	0.0 - 0.0	9.3	3.1 - 21.6	0.69
3070201	7338	SATILLA R	152.0	33.7	11 - 72	0.0	0.0 - 0.0	5.8	1.7 - 12.9	4.5	1.3 - 15.6	9.5	3.5 - 22.3	0.0	0.0 - 0.0	14.0	4.4 - 36.0	0.85
3070201	7339	SATILLA R	162.6	37.1	11 - 79	0.0	0.0 - 0.0	5.6	1.6 - 12.7	9.5	3.0 - 22.5	12.5	3.5 - 30.0	0.0	0.0 - 0.0	9.5	2.7 - 21.0	0.83
3070201	7340	SATILLA R	121.8	44.0	14 - 99	0.0	0.0 - 0.0	9.2	2.7 - 22.4	9.7	2.7 - 27.9	15.5	4.7 - 41.0	0.0	0.0 - 0.0	9.6	3.0 - 25.9	0.79
3070201	7341	INDIAN CR	74.2	42.7	16 - 103	0.0	0.0 - 0.0	6.9	2.5 - 15.4	10.1	3.2 - 26.4	16.7	6.2 - 51.6	0.0	0.0 - 0.0	9.1	3.2 - 25.3	0.76
3070201	7342	SATILLA R	187.0	33.2	14 - 85	0.0	0.0 - 0.0	3.3	1.3 - 8.9	7.2	2.5 - 25.2	12.3	4.9 - 35.1	0.0	0.0 - 0.0	10.4	4.2 - 28.9	0.76
3070201	7343	WIGGINS CR	96.4	32.9	10 - 63	0.0	0.0 - 0.0	3.3	1.0 - 7.3	4.5	1.4 - 11.4	15.2	4.5 - 34.2	0.0	0.0 - 0.0	9.8	3.0 - 20.9	0.67
3070201	7344	SATILLA R	159.5	27.8	10 - 61	0.0	0.0 - 0.0	3.6	1.2 - 7.2	1.6	0.5 - 4.1	14.4	4.7 - 40.6	0.0	0.0 - 0.0	8.2	2.6 - 22.5	0.67
3070201	7345	PUDDING CR	220.7	57.0	20 - 103	0.0	0.0 - 0.0	5.4	1.8 - 9.8	15.2	4.0 - 41.0	19.8	6.8 - 47.3	0.0	0.0 - 0.0	16.6	5.3 - 38.5	0.79
3070201	7346	RED BLUFF CR	352.9	28.1	10 - 57	1.3	0.4 - 2.9	3.8	1.0 - 8.1	4.2	1.3 - 10.3	5.7	1.9 - 13.6	0.0	0.0 - 0.0	13.0	4.5 - 32.1	0.83

8-digit HUC	ID	Name	Area	Catchment Yield		Point sources		Developed Land		Manure		Agricultural Land		Phosphate Mines		Soil parent rock		Frac
				kg/km^2	90% CI	kg/km^2	90% CI	kg/km^2	90% CI	kg/km^2	90% CI	kg/km^2	90% CI	kg/km^2	90% CI	kg/km^2	90% CI	
3070201	7347	KETTLE CR	119.4	38.1	12 - 68	0.0	0.0 - 0.0	17.5	5.7 - 33.7	2.0	0.6 - 6.0	6.6	2.1 - 15.8	0.0	0.0 - 0.0	12.1	3.5 - 27.9	0.88
3070201	7348	MILL CR	41.4	32.1	11 - 61	0.0	0.0 - 0.0	9.2	2.9 - 17.2	4.6	1.2 - 11.5	7.0	2.4 - 14.4	0.0	0.0 - 0.0	11.5	3.8 - 26.6	0.90
3070201	7349	MILL CR	51.3	21.9	8 - 51	0.0	0.0 - 0.0	6.7	2.2 - 15.8	3.7	1.0 - 10.5	6.4	2.3 - 16.3	0.0	0.0 - 0.0	5.1	1.6 - 14.1	0.87
3070201	7350	BIG CR	5.7	28.2	9 - 65	0.0	0.0 - 0.0	11.7	3.7 - 29.3	4.9	1.3 - 15.5	7.0	2.2 - 16.4	0.0	0.0 - 0.0	4.5	1.6 - 13.1	0.87
3070201	7351	BIG CR	60.8	27.6	9 - 71	0.0	0.0 - 0.0	7.3	2.2 - 17.2	2.7	0.8 - 7.9	5.0	1.7 - 14.1	0.0	0.0 - 0.0	12.6	3.7 - 30.8	0.84
3070201	7352	BIG CR, S PRONG	90.4	20.4	7 - 38	0.0	0.0 - 0.0	3.0	0.9 - 5.8	3.2	0.9 - 7.7	4.8	1.6 - 10.7	0.0	0.0 - 0.0	9.4	3.3 - 20.7	0.84
3070201	7353	BUFFALO CR	411.6	17.0	6 - 32	0.0	0.0 - 0.0	3.2	1.1 - 7.2	1.5	0.5 - 3.9	2.2	0.7 - 4.8	0.0	0.0 - 0.0	10.0	3.7 - 19.8	0.95
3070201	7354	SATILLA R	215.8	16.8	6 - 34	0.0	0.0 - 0.0	2.9	0.9 - 5.8	1.7	0.6 - 4.4	2.6	1.1 - 6.6	0.0	0.0 - 0.0	9.6	3.5 - 21.7	0.93
3070202	7355	LITTLE SATILLA R	424.9	22.7	8 - 59	0.5	0.1 - 1.1	4.4	1.5 - 10.7	1.2	0.4 - 3.9	6.9	2.5 - 17.3	0.0	0.0 - 0.0	9.7	3.4 - 25.6	0.93
3070202	7356	LITTLE SATILLA C	3.0	23.5	8 - 63	0.0	0.0 - 0.0	7.7	2.7 - 22.3	0.9	0.3 - 2.8	6.6	2.0 - 18.6	0.0	0.0 - 0.0	8.2	3.1 - 23.4	0.88
3070202	7357	LITTLE SATILLA C	25.1	31.1	10 - 77	0.0	0.0 - 0.0	9.7	2.7 - 26.2	1.1	0.3 - 3.2	10.6	3.5 - 26.0	0.0	0.0 - 0.0	9.7	3.4 - 24.3	0.87
3070202	7358	LITTLE SATILLA C	252.4	19.0	7 - 50	0.0	0.0 - 0.0	4.7	1.5 - 11.4	1.1	0.3 - 2.7	7.6	2.6 - 20.6	0.0	0.0 - 0.0	5.6	1.9 - 15.6	0.85
3070202	7359	DRY CR	137.1	18.5	8 - 33	0.1	0.1 - 0.3	4.1	1.5 - 7.9	1.1	0.4 - 2.8	9.2	3.9 - 22.6	0.0	0.0 - 0.0	3.9	1.5 - 8.5	0.85
3070202	7360	REEDY CR	117.7	28.3	8 - 71	0.0	0.0 - 0.0	4.7	1.3 - 11.3	3.5	0.9 - 9.7	16.0	4.7 - 46.1	0.0	0.0 - 0.0	4.1	1.3 - 11.6	0.87
3070202	7362	COLEMAN CR	230.0	25.1	8 - 57	0.0	0.0 - 0.0	5.3	1.6 - 12.9	3.7	1.0 - 11.0	12.1	4.1 - 32.0	0.0	0.0 - 0.0	4.0	1.1 - 10.9	0.87
3070202	7363	BIG SATILLA CR	42.4	33.7	13 - 76	0.0	0.0 - 0.0	4.4	1.6 - 9.0	4.0	1.2 - 14.1	20.4	7.0 - 53.6	0.0	0.0 - 0.0	4.9	1.9 - 13.3	0.87
3070202	7364	BIG SATILLA CR	54.0	37.9	12 - 95	0.0	0.0 - 0.0	5.5	1.7 - 12.4	6.7	1.6 - 20.0	21.0	7.1 - 58.2	0.0	0.0 - 0.0	4.7	1.5 - 12.1	0.85
3070202	7365	SWEETWATER CR	209.6	32.9	11 - 75	0.0	0.0 - 0.0	7.7	2.6 - 19.8	5.4	1.6 - 17.0	15.7	5.3 - 41.9	0.0	0.0 - 0.0	4.2	1.5 - 11.5	0.81
3070202	7366	BIG SATILLA CR	151.8	36.4	15 - 75	0.0	0.0 - 0.0	5.7	2.1 - 13.8	7.7	2.9 - 19.9	18.4	7.1 - 42.2	0.0	0.0 - 0.0	4.6	2.0 - 9.5	0.81
3070202	7367	BISHOP CR	146.8	33.5	10 - 86	0.0	0.0 - 0.0	6.7	1.9 - 17.0	4.5	1.2 - 13.3	17.8	5.9 - 55.3	0.0	0.0 - 0.0	4.4	1.3 - 11.5	0.71
3070202	7368	BIG SATILLA CR	162.1	29.8	12 - 54	0.0	0.0 - 0.0	6.1	2.3 - 11.7	4.2	1.4 - 11.6	15.4	6.3 - 36.4	0.0	0.0 - 0.0	4.0	1.5 - 9.1	0.71
3070202	7369	FISHING CR	134.4	31.1	11 - 70	0.0	0.0 - 0.0	5.2	1.7 - 11.7	4.7	1.5 - 13.3	16.5	5.6 - 39.6	0.0	0.0 - 0.0	4.7	1.6 - 11.7	0.85
3070204	7371	NORTH R	29.2	8.4	2 - 24	0.0	0.0 - 0.0	2.3	0.6 - 7.7	0.0	0.0 - 0.1	0.5	0.1 - 1.5	0.0	0.0 - 0.0	5.6	1.7 - 17.3	1.00
3070204	7372	ST MARYS R	91.6	35.3	13 - 91	32.3	11.8 - 87.2	0.5	0.1 - 1.9	0.0	0.0 - 0.2	0.0	0.0 - 0.1	0.0	0.0 - 0.0	2.4	0.6 - 8.6	0.99
3070204	7373	ST MARYS R	206.7	1.9	1 - 5	0.0	0.0 - 0.0	0.3	0.1 - 0.9	0.2	0.0 - 0.6	0.2	0.0 - 0.6	0.0	0.0 - 0.0	1.2	0.4 - 3.6	0.98
3070204	7374	ST MARYS R	14.4	0.3	0 - 2	0.0	0.0 - 0.0	0.1	0.0 - 0.9	0.0	0.0 - 0.1	0.0	0.0 - 0.1	0.0	0.0 - 0.0	0.1	0.0 - 0.5	0.94
3070204	7375	SPANISH CR	9.3	19.0	7 - 42	0.0	0.0 - 0.0	6.5	2.3 - 13.1	0.3	0.1 - 0.9	2.0	0.7 - 5.2	0.0	0.0 - 0.0	10.2	3.2 - 25.5	0.94
3070204	7376	*A	79.9	43.9	13 - 92	16.1	5.2 - 36.9	12.6	3.7 - 27.6	0.3	0.1 - 1.0	1.5	0.5 - 3.8	0.0	0.0 - 0.0	13.5	4.4 - 33.9	0.92
3070204	7377	SPANISH CR	141.5	20.8	9 - 50	0.0	0.0 - 0.0	4.5	1.8 - 10.3	0.5	0.2 - 1.5	2.6	1.1 - 8.4	0.0	0.0 - 0.0	13.3	5.5 - 35.9	0.92
3070204	7378	ST MARYS R	98.9	21.6	7 - 54	0.0	0.0 - 0.0	4.9	1.5 - 11.3	3.1	1.0 - 8.4	2.3	0.7 - 6.6	0.0	0.0 - 0.0	11.4	3.5 - 30.7	0.94
3070204	7379	SUWANNEE CANA	1.3	10.8	4 - 27	0.0	0.0 - 0.0	5.3	1.7 - 12.0	0.0	0.0 - 0.0	0.0	0.0 - 0.0	0.0	0.0 - 0.0	5.5	1.8 - 16.2	0.93
3070204	7380	SUWANNEE CANA	384.6	0.7	0 - 2	0.0	0.0 - 0.0	0.1	0.0 - 0.3	0.0	0.0 - 0.0	0.0	0.0 - 0.1	0.0	0.0 - 0.0	0.6	0.2 - 1.5	0.92
3070204	7381	CORNHOUSE CR	36.7	21.0	8 - 46	0.0	0.0 - 0.0	5.2	1.8 - 9.7	0.3	0.1 - 0.8	1.3	0.5 - 3.1	0.0	0.0 - 0.0	14.2	5.1 - 35.2	0.92
3070204	7382	ST MARYS R	185.8	22.9	8 - 54	0.0	0.0 - 0.0	6.3	2.2 - 16.9	3.1	1.0 - 11.5	2.0	0.7 - 6.2	0.0	0.0 - 0.0	11.5	4.1 - 34.2	0.93
3070204	7383	*B	54.9	18.7	7 - 37	0.0	0.0 - 0.0	4.8	1.6 - 10.7	0.2	0.0 - 0.4	0.6	0.2 - 1.4	0.0	0.0 - 0.0	13.1	5.2 - 29.6	0.91
3070204	7384	ST MARYS R	433.6	23.6	10 - 50	0.0	0.0 - 0.0	5.7	2.2 - 11.2	2.8	1.0 - 7.0	2.1	0.8 - 5.6	0.0	0.0 - 0.0	13.0	5.1 - 29.9	0.91
3070204	7385	ST MARYS R	17.5	18.3	7 - 43	0.0	0.0 - 0.0	3.8	1.3 - 9.2	0.6	0.2 - 2.0	0.6	0.2 - 1.6	0.0	0.0 - 0.0	13.3	5.2 - 33.9	0.88
3070204	7386	ST MARYS R, N PR	65.9	25.5	8 - 65	0.0	0.0 - 0.0	7.4	2.3 - 18.4	1.3	0.4 - 4.0	2.9	0.9 - 7.2	0.0	0.0 - 0.0	14.0	4.3 - 39.5	0.88
3070204	7387	ST MARYS R, N PR	51.8	34.2	11 - 85	0.0	0.0 - 0.0	5.4	1.7 - 12.0	4.1	1.2 - 10.9	9.1	2.4 - 27.9	0.0	0.0 - 0.0	15.6	4.4 - 38.8	0.87

8-digit HUC	ID	Name	Area	Catchment Yield		Point sources		Developed Land		Manure		Agricultural Land		Phosphate Mines		Soil parent rock		Frac
				kg/km^2	90% CI	kg/km^2	90% CI	kg/km^2	90% CI	kg/km^2	90% CI	kg/km^2	90% CI	kg/km^2	90% CI	kg/km^2	90% CI	
3070204	7388	ST MARYS R, N PR	214.6	9.9	3 - 22	0.0	0.0 - 0.0	1.8	0.5 - 4.4	0.7	0.2 - 1.8	1.1	0.3 - 3.3	0.0	0.0 - 0.0	6.2	2.1 - 15.8	0.85
3070204	7389	ST MARYS R, M PR	160.9	8.6	2 - 16	0.0	0.0 - 0.0	1.9	0.5 - 3.8	1.1	0.3 - 2.4	1.0	0.3 - 2.5	0.1	0.0 - 0.3	4.5	1.4 - 10.4	0.85
3070204	7390	CEDAR CR	207.8	21.0	7 - 45	0.0	0.0 - 0.0	3.7	1.3 - 8.3	2.2	0.7 - 6.1	2.1	0.6 - 5.6	0.0	0.0 - 0.0	13.0	4.3 - 32.7	0.87
3070204	7391	ST MARYS R, S PR	506.4	22.5	6 - 39	0.8	0.2 - 1.6	5.8	1.4 - 10.8	2.7	0.6 - 6.7	2.5	0.7 - 6.1	0.0	0.0 - 0.0	10.7	2.9 - 21.8	0.88
3070204	7392	*C	185.5	29.9	10 - 80	0.8	0.2 - 2.0	8.6	2.4 - 22.1	1.9	0.5 - 5.5	1.3	0.4 - 3.6	0.0	0.0 - 0.0	17.3	5.4 - 48.3	0.88
3070204	7393	*D	34.4	18.8	6 - 40	0.0	0.0 - 0.0	5.0	1.4 - 9.8	2.9	0.9 - 7.1	1.5	0.5 - 4.2	0.0	0.0 - 0.0	9.4	3.4 - 23.5	0.94
3070204	7394	LITTLE ST MARYS	200.5	27.9	8 - 58	0.0	0.0 - 0.0	4.6	1.3 - 9.7	4.0	1.1 - 10.6	2.1	0.7 - 5.1	0.0	0.0 - 0.0	17.3	5.0 - 38.9	0.98
3070204	7395	ST MARYS R	14.4	45.5	14 - 106	44.8	13.7 - 103.6	0.1	0.0 - 0.7	0.0	0.0 - 0.0	0.0	0.0 - 0.0	0.0	0.0 - 0.0	0.5	0.1 - 2.1	1.00
3070204	7396	BELLS R	38.4	1.5	0 - 5	0.0	0.0 - 0.0	0.2	0.0 - 0.7	0.0	0.0 - 0.2	0.0	0.0 - 0.0	0.0	0.0 - 0.0	1.2	0.2 - 4.4	1.00
3070205	7397	NASSAU R	60.0	0.1	0 - 1	0.0	0.0 - 0.0	0.0	0.0 - 0.1	0.0	0.0 - 0.0	0.0	0.0 - 0.0	0.0	0.0 - 0.0	0.1	0.0 - 0.6	1.00
3070205	7398	*A	124.8	14.3	5 - 24	0.0	0.0 - 0.0	5.0	1.5 - 9.5	0.6	0.2 - 1.3	0.3	0.1 - 0.6	0.0	0.0 - 0.0	8.5	3.0 - 15.7	0.98
3070205	7399	NASSAU R	77.0	0.5	0 - 2	0.0	0.0 - 0.0	0.1	0.0 - 0.5	0.0	0.0 - 0.1	0.0	0.0 - 0.1	0.0	0.0 - 0.0	0.4	0.1 - 1.6	0.98
3070205	7400	*B	407.6	19.4	6 - 37	0.5	0.1 - 1.0	2.7	0.9 - 5.6	3.5	1.0 - 9.4	2.0	0.6 - 5.0	0.0	0.0 - 0.0	10.8	3.5 - 22.1	0.93
3070205	7401	THOMAS CR	170.5	13.8	4 - 27	0.0	0.0 - 0.0	2.3	0.7 - 5.0	2.2	0.5 - 6.6	1.8	0.6 - 4.5	0.0	0.0 - 0.0	7.4	2.4 - 16.4	0.93
3080101	7402	WEKIVA R	6.4	23.5	7 - 66	0.0	0.0 - 0.0	0.0	0.0 - 0.0	1.3	0.4 - 4.0	3.4	1.2 - 8.9	0.0	0.0 - 0.0	18.8	5.9 - 50.0	0.94
3080101	7403	BLACK WATER CR	169.4	27.2	7 - 47	0.0	0.0 - 0.0	5.5	1.5 - 11.2	1.5	0.4 - 3.5	8.7	2.4 - 18.4	0.4	0.1 - 1.0	11.1	3.0 - 21.9	0.94
3080101	7404	WEKIVA R	476.0	57.6	20 - 134	9.4	3.2 - 24.1	33.0	10.5 - 79.4	0.7	0.2 - 2.0	2.6	0.8 - 7.4	0.0	0.0 - 0.0	11.9	3.7 - 33.9	0.94
3080101	7405	ST JOHNS R	953.4	67.2	22 - 163	2.8	0.9 - 7.5	44.2	15.1 - 113.1	0.6	0.2 - 1.4	1.5	0.5 - 4.0	0.0	0.0 - 0.0	18.2	6.6 - 47.7	0.94
3080101	7406	ST JOHNS R	3.8	50.9	14 - 147	0.0	0.0 - 0.0	2.0	0.7 - 4.5	1.7	0.4 - 5.9	2.1	0.6 - 7.4	0.0	0.0 - 0.0	45.1	11.8 - 132.0	0.92
3080101	7407	LITTLE ECOHLOC	202.6	115.5	46 - 224	27.5	10.6 - 61.2	63.8	23.1 - 133.0	0.2	0.1 - 0.5	0.7	0.2 - 1.5	0.0	0.0 - 0.0	23.4	7.9 - 54.2	0.87
3080101	7408	ECOHLOCKHATC	480.3	11.1	4 - 25	0.0	0.0 - 0.0	2.8	0.8 - 8.2	0.6	0.2 - 1.7	2.4	0.7 - 6.2	0.0	0.0 - 0.0	5.2	1.7 - 13.3	0.87
3080101	7409	JIM CR	288.2	47.7	19 - 111	0.0	0.0 - 0.0	3.3	1.3 - 8.0	2.1	0.7 - 7.4	14.0	4.9 - 40.8	0.0	0.0 - 0.0	28.2	11.2 - 79.3	0.89
3080101	7410	ST JOHNS R	239.6	18.7	5 - 41	0.5	0.2 - 1.0	5.1	1.6 - 14.5	0.1	0.0 - 0.2	0.3	0.1 - 0.8	0.0	0.0 - 0.0	12.7	3.6 - 33.3	0.89
3080101	7411	TAYLOR CR	426.3	73.5	18 - 141	0.0	0.0 - 0.0	0.5	0.2 - 1.1	12.7	3.0 - 36.3	37.8	12.0 - 82.6	0.0	0.0 - 0.0	22.4	5.5 - 52.1	0.87
3080101	7412	ST JOHNS R	7.5	24.4	6 - 53	0.0	0.0 - 0.0	0.3	0.1 - 0.5	2.2	0.4 - 6.5	6.1	1.3 - 17.9	0.0	0.0 - 0.0	15.8	3.6 - 36.4	0.87
3080101	7413	CRABGRASS CR	222.2	19.4	6 - 39	0.0	0.0 - 0.0	1.1	0.3 - 2.6	2.8	0.7 - 8.3	7.8	2.3 - 18.4	0.0	0.0 - 0.0	7.7	2.3 - 16.5	0.79
3080101	7414	JANE GREEN CR	185.3	33.3	12 - 75	0.0	0.0 - 0.0	0.2	0.0 - 0.4	4.7	1.3 - 15.2	15.5	4.5 - 39.5	0.0	0.0 - 0.0	12.9	4.3 - 32.1	0.83
3080101	7415	BULL CR	434.1	32.5	11 - 68	0.0	0.0 - 0.0	0.6	0.2 - 1.4	4.6	1.4 - 15.9	13.4	4.2 - 36.0	0.0	0.0 - 0.0	13.8	4.6 - 37.2	0.79
3080101	7416	BLUE CYPRESS CR	442.3	70.1	20 - 145	0.0	0.0 - 0.0	1.6	0.5 - 3.3	11.5	3.2 - 27.2	36.1	10.1 - 83.7	0.0	0.0 - 0.0	20.8	5.8 - 48.2	0.80
3080101	7417	PADGETT BR	136.8	54.1	16 - 124	0.0	0.0 - 0.0	1.3	0.4 - 3.3	10.6	2.9 - 30.7	24.5	7.7 - 64.0	0.0	0.0 - 0.0	17.8	5.4 - 45.0	0.79
3080101	7418	FORT DRUM CR	2117.1	88.6	26 - 196	0.0	0.0 - 0.0	4.4	1.3 - 10.1	12.3	3.5 - 47.8	44.8	12.1 - 118.8	0.0	0.0 - 0.0	27.1	7.7 - 85.9	0.79
3080101	7419	*A	222.2	36.9	14 - 75	0.0	0.0 - 0.0	5.5	1.8 - 12.7	1.6	0.4 - 4.6	4.8	1.4 - 13.6	0.0	0.0 - 0.0	25.1	9.4 - 52.9	0.92
3080102	7420	SWEETWATER CR	127.5	41.3	12 - 96	0.0	0.0 - 0.0	14.0	4.9 - 32.3	1.0	0.2 - 2.7	3.2	1.0 - 9.4	0.0	0.0 - 0.0	23.1	6.5 - 59.8	0.64
3080102	7421	OKLAWAHA R	42.5	27.6	9 - 56	0.0	0.0 - 0.0	5.0	1.6 - 9.3	0.6	0.2 - 1.6	1.9	0.7 - 4.5	0.0	0.0 - 0.0	20.1	7.1 - 48.2	0.64
3080102	7422	ORANGE CR	5.8	32.4	11 - 62	0.0	0.0 - 0.0	7.3	2.8 - 15.2	1.2	0.4 - 3.5	4.7	1.4 - 11.0	0.0	0.0 - 0.0	19.1	6.6 - 41.3	0.64
3080102	7423	LITTLE CABBAGE	81.1	22.4	8 - 40	0.0	0.0 - 0.0	13.8	5.0 - 29.1	0.4	0.1 - 0.8	1.1	0.4 - 2.9	0.0	0.0 - 0.0	7.3	2.8 - 14.0	0.63
3080102	7424	ORANGE CR	388.8	20.8	7 - 43	0.0	0.0 - 0.0	3.3	1.2 - 8.4	1.4	0.5 - 4.8	4.6	1.4 - 11.9	0.0	0.0 - 0.1	11.5	3.5 - 26.9	0.63
3080102	7425	CROSS CR	8.6	3.0	1 - 9	0.0	0.0 - 0.0	0.4	0.1 - 1.2	0.1	0.0 - 0.3	0.1	0.0 - 0.3	0.0	0.0 - 0.0	2.5	0.7 - 7.3	0.57
3080102	7426	LOCHLOOSA CR	156.0	38.5	14 - 83	0.0	0.0 - 0.0	4.6	1.4 - 9.4	2.6	0.8 - 7.1	4.2	1.3 - 11.0	0.1	0.0 - 0.2	27.0	9.6 - 69.3	0.55

8-digit HUC	ID	Name	Area	Catchment Yield		Point sources		Developed Land		Manure		Agricultural Land		Phosphate Mines		Soil parent rock		Frac
				kg/km^2	90% CI	kg/km^2	90% CI	kg/km^2	90% CI	kg/km^2	90% CI	kg/km^2	90% CI	kg/km^2	90% CI	kg/km^2	90% CI	
3080102	7427	HATCHET R	189.9	33.1	12 - 72	0.0	0.0 - 0.0	2.9	1.1 - 5.5	3.4	1.1 - 7.6	3.0	1.2 - 7.2	0.0	0.0 - 0.0	23.9	8.4 - 55.3	0.53
3080102	7428	OKLAWAHA R	271.9	23.1	8 - 69	0.0	0.0 - 0.0	3.4	1.1 - 7.9	0.9	0.2 - 2.6	1.7	0.6 - 4.6	0.0	0.0 - 0.0	17.1	5.5 - 53.9	0.64
3080102	7429	OKLAWAHA R	480.6	4.9	2 - 13	0.0	0.0 - 0.0	0.6	0.2 - 1.7	0.2	0.1 - 0.6	0.3	0.1 - 0.9	0.0	0.0 - 0.0	3.8	1.2 - 10.3	0.63
3080102	7430	OKLAWAHA R	7.3	11.4	3 - 25	0.0	0.0 - 0.0	2.7	0.7 - 6.5	0.2	0.1 - 0.6	1.4	0.4 - 3.7	0.0	0.0 - 0.0	7.1	2.2 - 15.0	0.21
3080102	7431	*C	1183.0	16.2	4 - 36	0.0	0.0 - 0.0	4.9	1.3 - 9.9	0.7	0.2 - 1.9	4.1	1.2 - 11.2	0.0	0.0 - 0.0	6.5	1.9 - 13.8	0.00
3080103	7438	*B	103.0	49.5	16 - 103	11.6	3.9 - 27.9	18.2	5.4 - 36.3	1.2	0.4 - 3.0	1.5	0.4 - 3.6	0.0	0.0 - 0.0	16.9	5.0 - 40.6	0.98
3080103	7439	BLACK CR	7.0	42.2	15 - 102	0.0	0.0 - 0.0	16.8	5.8 - 39.0	1.8	0.5 - 4.7	5.6	1.9 - 14.9	0.0	0.0 - 0.0	18.0	6.0 - 44.3	0.98
3080103	7441	YELLOW WATER (	147.5	30.9	11 - 56	0.0	0.0 - 0.0	12.7	5.1 - 26.9	1.4	0.5 - 4.2	1.2	0.4 - 3.3	0.0	0.0 - 0.0	15.5	5.7 - 35.2	0.92
3080103	7442	BLACK CR, N FK	228.6	27.6	10 - 59	0.0	0.0 - 0.0	10.0	3.6 - 22.1	0.8	0.3 - 2.6	1.1	0.4 - 2.9	0.0	0.0 - 0.0	15.7	6.0 - 39.8	0.92
3080103	7443	BLACK CR, S FK	148.7	38.1	12 - 90	0.3	0.1 - 0.8	20.2	6.2 - 48.3	0.8	0.2 - 2.6	1.9	0.6 - 4.7	0.0	0.0 - 0.0	14.9	5.6 - 41.5	0.96
3080103	7444	BLACK CR, S FK	21.9	25.9	10 - 56	0.0	0.0 - 0.0	8.7	3.5 - 17.8	0.7	0.3 - 1.9	1.0	0.4 - 2.5	0.0	0.0 - 0.0	15.4	5.4 - 38.0	0.90
3080103	7445	BLACK CR, S FK	96.1	24.9	10 - 50	0.1	0.0 - 0.2	12.7	4.9 - 27.2	0.5	0.2 - 1.6	0.3	0.1 - 0.7	0.0	0.0 - 0.0	11.3	4.8 - 24.0	0.89
3080103	7446	ATES CR	110.1	19.2	7 - 41	0.0	0.0 - 0.0	5.4	1.7 - 11.0	0.8	0.3 - 2.3	2.0	0.6 - 5.2	0.0	0.0 - 0.0	10.9	4.0 - 27.7	0.89
3080103	7447	GREENS CR	111.5	16.0	5 - 45	0.0	0.0 - 0.0	3.6	1.0 - 8.8	1.0	0.2 - 3.6	1.1	0.4 - 3.3	0.0	0.0 - 0.0	10.3	3.4 - 31.2	0.90
3080103	7452	SIMMS CR	116.0	21.2	6 - 41	0.0	0.0 - 0.0	4.4	1.4 - 8.8	1.0	0.3 - 2.8	1.7	0.5 - 4.1	0.0	0.0 - 0.0	14.0	4.2 - 31.2	0.98
3080103	7453	ETONIA CR	31.7	14.3	5 - 27	0.0	0.0 - 0.0	3.2	1.0 - 6.2	0.7	0.2 - 1.9	2.5	0.8 - 5.9	0.0	0.0 - 0.0	7.8	2.5 - 16.2	0.98
3080103	7454	RICE CR	86.3	18.0	7 - 34	0.0	0.0 - 0.0	4.2	1.5 - 7.6	0.5	0.2 - 1.4	1.1	0.4 - 2.5	0.0	0.0 - 0.0	12.2	4.7 - 25.4	0.91
3080103	7455	ETONIA CR	499.0	10.9	3 - 20	0.0	0.0 - 0.0	5.4	1.4 - 12.5	0.3	0.1 - 0.8	0.9	0.2 - 2.4	0.0	0.0 - 0.0	4.3	1.1 - 8.7	0.91
3080103	7456	ST JOHNS R	73.1	16.7	6 - 40	0.2	0.1 - 0.5	5.5	1.7 - 13.2	0.5	0.1 - 1.2	2.2	0.7 - 5.9	0.0	0.0 - 0.0	8.3	3.0 - 20.0	1.00
3080103	7457	HAW CR	438.7	12.7	4 - 24	0.7	0.2 - 1.4	2.2	0.7 - 4.4	0.4	0.1 - 1.0	1.8	0.6 - 4.7	0.0	0.0 - 0.0	7.5	2.4 - 15.2	0.98
3080103	7458	LITTLE HAW CR	43.0	87.7	24 - 229	0.0	0.0 - 0.0	2.1	0.7 - 4.7	5.4	1.3 - 18.3	22.0	6.9 - 69.4	0.0	0.0 - 0.0	58.3	17.3 - 169.2	0.93
3080103	7459	HAW CR	7.6	67.5	19 - 155	0.0	0.0 - 0.0	1.2	0.3 - 2.7	2.7	0.6 - 8.6	12.3	3.6 - 40.1	0.0	0.0 - 0.0	51.4	14.2 - 134.3	0.93
3080103	7460	MIDDLE HAW CR	230.2	13.3	4 - 30	0.0	0.0 - 0.0	1.1	0.3 - 2.7	0.7	0.2 - 2.2	2.7	0.8 - 7.8	0.0	0.0 - 0.0	8.9	2.6 - 20.6	0.92
3080103	7461	HAW CR	28.0	97.0	25 - 243	0.0	0.0 - 0.0	2.4	0.7 - 5.3	6.7	1.5 - 24.6	23.7	5.4 - 69.5	0.0	0.0 - 0.0	64.3	16.5 - 171.2	0.92
3080103	7462	HAW CR, BLACK E	283.2	34.7	11 - 62	1.9	0.6 - 3.3	7.3	2.2 - 13.2	1.2	0.3 - 2.8	2.3	0.7 - 4.9	0.0	0.0 - 0.0	21.9	6.6 - 42.8	0.89
3080103	7463	HAW CR	18.6	62.5	21 - 194	0.0	0.0 - 0.0	4.3	1.5 - 10.4	2.2	0.6 - 8.9	9.0	2.9 - 27.5	0.0	0.0 - 0.0	47.1	15.3 - 154.8	0.89
3080103	7464	*D	87.4	85.2	30 - 168	6.7	2.5 - 13.8	68.4	23.3 - 141.4	0.0	0.0 - 0.1	0.1	0.0 - 0.1	0.0	0.0 - 0.0	10.0	3.6 - 19.9	1.00
3080201	7465	MOULTRE CR	172.8	40.0	13 - 78	1.8	0.6 - 4.0	17.5	5.4 - 35.5	0.3	0.1 - 0.9	1.4	0.4 - 3.7	0.0	0.0 - 0.0	19.0	5.9 - 43.3	1.00
3080201	7466	PELLICER CR	44.6	42.1	15 - 101	0.0	0.0 - 0.0	3.8	1.3 - 7.5	0.7	0.2 - 1.9	1.1	0.4 - 2.9	0.0	0.0 - 0.0	36.5	12.4 - 93.2	1.00
3080201	7467	*A	70.8	31.5	9 - 73	0.0	0.0 - 0.0	6.6	2.1 - 14.4	0.5	0.1 - 1.7	0.6	0.2 - 1.6	0.0	0.0 - 0.0	23.8	7.0 - 55.2	0.94
3080201	7470	PELLICER CR	35.3	3.7	1 - 9	0.0	0.0 - 0.0	0.4	0.1 - 1.1	0.0	0.0 - 0.1	0.1	0.0 - 0.2	0.0	0.0 - 0.0	3.1	0.8 - 7.9	0.92
3080201	7471	PELLICER CR, PRII	82.9	22.1	7 - 46	0.0	0.0 - 0.0	2.6	0.8 - 5.8	1.4	0.4 - 3.5	1.0	0.4 - 2.7	0.0	0.0 - 0.0	17.0	5.2 - 38.3	0.92
3080201	7472	*C	87.8	55.2	16 - 105	0.0	0.0 - 0.0	32.2	8.8 - 68.0	0.7	0.2 - 1.8	1.1	0.3 - 2.8	0.0	0.0 - 0.0	21.2	6.3 - 47.0	0.94
3080201	7474	*D	98.7	83.0	28 - 189	0.2	0.1 - 0.6	47.2	16.5 - 100.0	1.0	0.3 - 3.0	4.0	1.3 - 10.7	0.0	0.0 - 0.0	30.6	8.8 - 77.6	0.95
3080201	7475	SPRUCE CR	757.8	4.1	1 - 10	0.5	0.2 - 1.0	0.7	0.1 - 2.0	0.1	0.0 - 0.4	0.3	0.1 - 1.1	0.0	0.0 - 0.0	2.5	0.6 - 6.6	0.95
3080201	7476	INTRACOASTAL W	298.1	131.4	40 - 230	56.5	19.0 - 109.5	43.9	11.3 - 82.0	0.3	0.1 - 0.8	0.6	0.2 - 1.4	0.0	0.0 - 0.0	30.1	9.1 - 67.3	0.00
3080201	7477	INTRACOASTAL W	162.5	51.7	19 - 111	9.4	3.9 - 18.5	8.5	3.3 - 17.2	0.2	0.1 - 0.5	1.0	0.4 - 2.2	0.0	0.0 - 0.0	32.7	12.5 - 79.9	1.00
3080203	7486	SEBASTION CR	14.6	84.3	30 - 175	0.0	0.0 - 0.0	52.7	19.9 - 111.7	0.1	0.0 - 0.2	0.0	0.0 - 0.0	0.0	0.0 - 0.0	31.6	12.5 - 77.6	1.00
3080203	7487	SEBASTION CR, N	74.0	82.6	30 - 191	0.7	0.2 - 2.0	7.3	2.3 - 17.1	5.2	1.5 - 15.8	20.9	6.9 - 68.6	0.0	0.0 - 0.0	48.5	16.6 - 124.9	0.99

8-digit HUC	ID	Name	Area	Catchment Yield		Point sources		Developed Land		Manure		Agricultural Land		Phosphate Mines		Soil parent rock		Frac
				kg/km^2	90% CI	kg/km^2	90% CI	kg/km^2	90% CI	kg/km^2	90% CI	kg/km^2	90% CI	kg/km^2	90% CI	kg/km^2	90% CI	
3080203	7488	SEBASTION CR	142.7	127.4	45 - 273	0.1	0.1 - 0.3	30.3	9.3 - 67.1	9.2	2.6 - 27.0	44.5	15.6 - 104.8	0.0	0.0 - 0.0	43.4	14.5 - 120.2	0.99
3080203	7489	MAIN CANAL	14.5	76.4	25 - 172	0.0	0.0 - 0.0	53.1	16.5 - 127.0	1.4	0.5 - 4.9	4.6	1.7 - 14.0	0.0	0.0 - 0.0	17.3	6.1 - 47.7	1.00
3100101	7514	SHELL CR	359.0	16.2	6 - 36	0.0	0.0 - 0.0	0.8	0.2 - 2.0	1.4	0.4 - 4.2	5.5	1.6 - 14.1	0.0	0.0 - 0.0	8.5	3.1 - 19.2	0.98
3100101	7515	PRAIRIE CR	90.6	60.3	18 - 114	0.0	0.0 - 0.0	5.4	1.5 - 10.7	8.4	1.8 - 24.3	32.2	9.1 - 75.6	0.0	0.0 - 0.0	14.3	4.3 - 30.2	0.98
3100101	7516	PEACE R	57.4	49.8	16 - 125	0.0	0.0 - 0.0	3.2	1.1 - 7.9	6.0	1.7 - 17.9	20.3	6.0 - 56.0	0.0	0.0 - 0.0	20.4	6.3 - 53.1	1.00
3100101	7517	JOSHUA CR	3.9	71.3	25 - 177	0.0	0.0 - 0.0	17.5	6.1 - 38.7	3.9	1.2 - 11.6	9.6	3.1 - 25.6	0.0	0.0 - 0.0	40.3	14.4 - 105.5	0.98
3100101	7518	*A	101.0	125.0	40 - 222	0.0	0.0 - 0.0	5.0	1.6 - 9.0	19.7	5.3 - 46.9	69.6	24.8 - 154.6	0.0	0.0 - 0.0	30.6	9.5 - 68.4	0.87
3100101	7519	JOSHUA CR	91.3	111.1	33 - 249	0.0	0.0 - 0.0	3.4	1.1 - 7.5	16.6	4.8 - 44.5	57.7	18.2 - 176.6	0.0	0.0 - 0.0	33.4	10.2 - 88.1	0.87
3100101	7520	PEACE R	34.6	83.6	28 - 187	0.0	0.0 - 0.0	22.4	7.3 - 45.2	6.1	1.7 - 20.3	20.0	7.3 - 45.4	0.0	0.0 - 0.0	35.1	10.7 - 101.8	0.98
3100101	7521	CHARLIE CR	26.9	258.0	95 - 494	0.0	0.0 - 0.0	4.6	1.4 - 9.0	21.0	5.9 - 59.3	50.7	16.6 - 126.9	133.0	45.5 - 343.4	48.7	18.1 - 116.5	0.96
3100101	7522	OAK CR	239.9	81.1	25 - 164	0.0	0.0 - 0.0	1.6	0.5 - 3.6	15.0	4.6 - 41.7	41.4	11.7 - 111.3	0.0	0.0 - 0.0	23.1	7.4 - 59.7	0.92
3100101	7523	CHARLIE CR	82.4	99.1	32 - 202	0.0	0.0 - 0.0	2.9	0.9 - 6.3	18.4	4.6 - 49.6	44.0	14.4 - 127.8	0.0	0.0 - 0.0	33.8	10.3 - 74.4	0.92
3100101	7524	*B	132.8	22.8	7 - 42	0.0	0.0 - 0.0	1.7	0.5 - 3.9	3.2	0.9 - 8.5	8.6	3.0 - 19.6	0.0	0.0 - 0.0	9.2	2.8 - 20.7	0.88
3100101	7525	CHARLIE CR	64.3	133.4	49 - 350	0.0	0.0 - 0.0	3.3	1.1 - 9.4	24.2	7.6 - 73.2	59.5	20.9 - 192.9	0.0	0.0 - 0.0	46.4	15.3 - 127.5	0.88
3100101	7526	*C	114.9	65.7	21 - 145	0.0	0.0 - 0.0	9.7	3.2 - 22.7	8.8	2.6 - 28.0	21.9	7.7 - 60.9	0.0	0.0 - 0.0	25.2	7.7 - 58.7	0.85
3100101	7527	CHARLIE CR	61.8	97.5	28 - 189	0.0	0.0 - 0.0	4.6	1.3 - 9.7	16.1	4.6 - 52.6	38.5	11.3 - 85.6	0.0	0.0 - 0.0	38.2	10.5 - 81.3	0.85
3100101	7528	OLD TOWN CR	56.7	22.6	5 - 45	0.0	0.0 - 0.0	3.8	0.9 - 8.0	2.9	0.7 - 8.4	8.2	2.2 - 20.2	0.0	0.0 - 0.0	7.7	2.1 - 18.7	0.75
3100101	7529	CHARLIE CR	48.1	111.1	37 - 216	0.0	0.0 - 0.0	1.8	0.6 - 3.2	15.5	4.6 - 46.1	44.4	15.5 - 115.4	4.1	1.3 - 10.4	45.2	14.4 - 107.2	0.75
3100101	7530	PEACE R	75.6	81.7	27 - 190	0.0	0.0 - 0.0	1.7	0.5 - 3.9	12.9	3.7 - 42.3	32.8	10.9 - 81.3	0.0	0.0 - 0.0	34.3	12.8 - 91.2	0.96
3100101	7531	PEACE R	60.4	109.8	34 - 305	0.0	0.0 - 0.0	0.8	0.2 - 2.1	16.9	4.3 - 48.6	39.8	11.7 - 129.5	0.0	0.0 - 0.0	52.3	18.4 - 145.5	0.95
3100101	7532	PEACE R	73.1	112.0	34 - 274	0.0	0.0 - 0.0	6.8	2.2 - 14.2	17.7	5.0 - 60.3	42.0	13.2 - 113.0	0.0	0.0 - 0.0	45.4	15.2 - 131.2	0.94
3100101	7533	LITTLE CHARLIE C	107.9	170.5	55 - 449	0.0	0.0 - 0.0	2.1	0.7 - 5.2	24.3	6.7 - 64.7	60.8	20.8 - 186.7	35.6	10.0 - 114.3	47.8	15.7 - 130.6	0.91
3100101	7534	PEACE R	34.1	102.1	32 - 294	0.0	0.0 - 0.0	10.1	3.0 - 24.1	16.7	4.6 - 43.4	40.4	13.5 - 112.5	0.0	0.0 - 0.0	34.9	11.5 - 109.2	0.91
3100101	7535	PEACE R	60.4	792.1	252 - 1884	105.7	32.2 - 255.0	6.3	1.8 - 15.3	8.8	2.4 - 32.1	21.9	7.2 - 65.2	611.4	183.0 - 1502	38.0	12.4 - 91.1	0.90
3100101	7536	PEACE R	5.2	906.2	263 - 2299	0.0	0.0 - 0.0	8.1	2.5 - 17.3	7.3	1.6 - 25.6	16.9	4.8 - 49.7	818.3	230.0 - 2107	55.6	15.5 - 131.1	0.88
3100101	7537	BOWLEGS CR	69.2	906.7	344 - 2523	0.0	0.0 - 0.0	3.1	1.1 - 6.7	11.9	3.6 - 38.0	34.3	12.9 - 93.8	814.5	302.8 - 2375	42.8	17.4 - 117.7	0.87
3100101	7538	*D	32.1	76.0	24 - 145	0.0	0.0 - 0.0	5.6	1.8 - 10.7	9.2	2.7 - 23.5	29.1	9.3 - 66.3	0.0	0.0 - 0.0	32.1	10.2 - 70.2	0.81
3100101	7539	*E	82.3	51.4	17 - 110	0.0	0.0 - 0.0	5.6	1.7 - 11.9	6.4	1.9 - 18.9	21.1	7.4 - 52.5	0.0	0.0 - 0.0	18.3	6.2 - 47.6	0.81
3100101	7540	PEACE R	109.8	878.7	290 - 2546	0.0	0.0 - 0.0	11.4	3.6 - 21.7	10.4	2.7 - 32.9	18.0	5.2 - 41.6	801.7	275.4 - 2410	37.2	11.4 - 82.0	0.87
3100101	7541	PEACE R	753.4	18.9	5 - 44	0.0	0.0 - 0.0	6.2	1.6 - 16.9	1.5	0.4 - 4.3	4.8	1.2 - 13.6	0.8	0.2 - 2.3	5.6	1.7 - 13.9	0.79
3100101	7542	SADDLE CR	397.2	222.1	77 - 545	6.8	2.5 - 13.5	19.6	6.4 - 47.3	2.2	0.7 - 5.1	6.4	2.1 - 15.4	171.6	55.4 - 482.5	15.5	5.7 - 35.1	0.79
3100101	7543	WHIDDEN CR	120.2	3307.6	940 - 9098	74.7	26.1 - 172.0	5.9	1.8 - 12.5	10.0	2.2 - 33.8	2.7	0.6 - 7.7	3147.3	885.5 - 8804	67.0	17.2 - 176.9	0.88
3100101	7544	PAYNE CR	3.5	104.4	31 - 285	0.0	0.0 - 0.0	14.4	4.8 - 31.8	13.8	3.6 - 49.4	33.0	9.9 - 102.2	0.0	0.0 - 0.0	43.2	12.0 - 130.8	0.90
3100101	7545	LITTLE PAYNE CR	86.8	2437.8	724 - 6091	0.0	0.0 - 0.0	6.2	2.0 - 13.7	10.8	2.4 - 37.0	2.8	0.9 - 7.2	2354.5	715.1 - 5923	63.4	18.6 - 180.2	0.89
3100101	7546	PAYNE CR	145.3	1020.5	363 - 2441	0.0	0.0 - 0.0	3.8	1.1 - 7.7	15.0	4.7 - 50.3	24.1	7.6 - 62.1	926.0	320.7 - 2423	51.6	19.9 - 146.0	0.89
3100101	7547	TROUBLESOME CI	65.8	218.8	78 - 482	0.0	0.0 - 0.0	3.5	1.3 - 7.8	20.8	6.0 - 53.1	51.0	18.7 - 129.0	101.3	31.1 - 274.8	42.1	12.9 - 101.0	0.94
3100101	7548	OAK CR	63.0	99.7	40 - 240	0.0	0.0 - 0.0	2.2	0.8 - 5.3	16.6	5.7 - 62.8	40.4	15.4 - 114.2	0.0	0.0 - 0.0	40.5	16.4 - 100.3	0.95
3100101	7549	HORSE CR	127.6	83.0	30 - 170	0.0	0.0 - 0.0	1.7	0.6 - 3.7	10.7	2.8 - 27.8	33.6	10.9 - 84.1	0.0	0.0 - 0.0	37.0	12.5 - 85.8	1.00
3100101	7550	BRUSH CR	117.1	221.7	72 - 625	0.0	0.0 - 0.0	2.0	0.7 - 5.7	13.0	3.6 - 36.4	30.0	9.8 - 83.0	134.3	47.7 - 466.1	42.4	14.2 - 119.3	0.87

8-digit HUC	ID	Name	Area	Catchment Yield		Point sources		Developed Land		Manure		Agricultural Land		Phosphate Mines		Soil parent rock		Frac
				kg/km^2	90% CI	kg/km^2	90% CI	kg/km^2	90% CI	kg/km^2	90% CI	kg/km^2	90% CI	kg/km^2	90% CI	kg/km^2	90% CI	
3100101	7551	HORSE CR	61.0	111.1	38 - 197	0.0	0.0 - 0.0	1.9	0.6 - 3.6	18.9	5.5 - 59.1	45.6	15.4 - 101.6	0.0	0.0 - 0.0	44.8	16.4 - 112.0	0.87
3100102	7552	BIG SLOUGH CAN/	547.9	80.9	28 - 154	0.0	0.0 - 0.0	7.5	2.5 - 15.7	8.2	2.5 - 20.7	23.4	7.9 - 61.2	3.3	1.2 - 9.2	38.5	13.3 - 85.7	1.00
3100102	7553	MYAKKA R	42.0	74.5	21 - 180	0.0	0.0 - 0.0	13.8	4.7 - 25.8	2.1	0.5 - 7.3	4.1	1.3 - 9.8	0.0	0.0 - 0.0	54.4	14.6 - 158.4	1.00
3100102	7554	OWEN CR	112.9	48.2	17 - 122	0.0	0.0 - 0.0	0.5	0.1 - 1.1	7.0	2.0 - 20.2	21.4	7.0 - 75.1	0.0	0.0 - 0.0	19.3	6.1 - 51.6	0.89
3100102	7555	MYAKKA R	8.4	128.2	43 - 224	0.0	0.0 - 0.0	11.0	3.4 - 21.9	14.5	3.6 - 34.9	45.5	13.5 - 100.5	0.0	0.0 - 0.0	57.1	20.7 - 122.1	0.89
3100102	7556	LONG CR	107.7	102.0	34 - 228	0.0	0.0 - 0.0	3.8	1.2 - 7.9	9.1	2.6 - 30.3	28.3	9.0 - 78.9	29.8	9.0 - 104.2	30.9	9.8 - 78.7	0.88
3100102	7557	OGLEBY CR	92.5	74.5	23 - 177	0.0	0.0 - 0.0	5.4	1.5 - 13.0	8.3	2.2 - 25.5	26.3	8.5 - 64.7	0.0	0.0 - 0.0	34.6	10.7 - 108.7	0.88
3100103	7558	*A	150.4	76.0	25 - 144	0.0	0.0 - 0.0	37.6	12.1 - 71.2	0.3	0.1 - 0.9	0.5	0.1 - 1.4	0.0	0.0 - 0.0	37.7	11.0 - 105.9	1.00
3100103	7559	*B	79.6	18.0	5 - 38	0.0	0.0 - 0.0	3.6	0.9 - 8.5	0.4	0.1 - 1.1	1.4	0.4 - 3.2	0.0	0.0 - 0.0	12.6	3.5 - 28.0	1.00
3100103	7560	*C	77.3	34.5	11 - 75	0.0	0.0 - 0.0	8.7	2.7 - 19.4	1.2	0.3 - 3.3	4.5	1.5 - 11.2	0.0	0.0 - 0.0	20.2	6.6 - 49.2	1.00
3100103	7562	*E	30.4	62.2	21 - 165	0.0	0.0 - 0.0	28.8	10.1 - 76.1	2.3	0.6 - 7.9	8.1	2.9 - 22.5	0.0	0.0 - 0.0	23.0	8.5 - 69.8	1.00
3100103	7563	*D	22.7	33.6	11 - 70	0.0	0.0 - 0.0	15.4	4.8 - 37.2	1.7	0.4 - 4.8	6.1	1.8 - 15.6	0.0	0.0 - 0.0	10.4	2.7 - 24.6	1.00
3100201	7564	*A	55.4	121.6	42 - 252	0.0	0.0 - 0.0	73.0	25.8 - 151.1	0.1	0.0 - 0.2	0.0	0.0 - 0.0	0.0	0.0 - 0.0	48.5	17.9 - 127.2	1.00
3100201	7565	*B	272.7	126.5	56 - 229	19.2	8.3 - 37.4	29.5	13.2 - 52.9	11.1	4.1 - 26.7	25.2	10.8 - 55.0	0.0	0.0 - 0.0	41.5	18.1 - 87.0	1.00
3100201	7566	*C	120.6	117.4	41 - 255	0.0	0.0 - 0.0	73.1	22.0 - 150.0	2.0	0.6 - 5.8	3.8	1.3 - 9.3	0.0	0.0 - 0.0	38.5	13.6 - 106.8	1.00
3100202	7567	MANATEE R	45.4	18.2	6 - 34	0.0	0.0 - 0.0	2.9	0.8 - 6.3	1.8	0.5 - 5.6	5.9	1.8 - 14.7	0.0	0.0 - 0.0	7.6	2.6 - 16.5	1.00
3100202	7568	MANATEE R, E FK	66.8	119.5	44 - 256	0.0	0.0 - 0.0	1.4	0.5 - 2.9	7.2	2.2 - 18.4	21.7	7.1 - 50.9	58.2	19.5 - 154.5	31.0	10.5 - 67.2	0.23
3100202	7569	MANATEE R, N FK	72.9	83.2	32 - 166	0.0	0.0 - 0.0	1.0	0.3 - 2.1	6.1	1.9 - 14.6	19.7	6.6 - 42.7	20.9	6.2 - 68.0	35.5	12.4 - 82.9	0.23
3100202	7570	GAMBLE CR	172.0	87.4	25 - 221	0.0	0.0 - 0.0	4.5	1.3 - 9.9	10.8	2.6 - 30.5	34.6	11.2 - 98.2	0.0	0.0 - 0.0	37.4	12.2 - 93.9	1.00
3100203	7571	LITTLE MANATEE	166.9	117.0	38 - 193	0.0	0.0 - 0.0	30.0	9.4 - 56.9	11.8	3.1 - 33.1	29.6	10.8 - 69.2	0.0	0.0 - 0.0	45.5	14.5 - 108.4	1.00
3100203	7572	LITTLE MANATEE	102.9	112.3	35 - 238	0.0	0.0 - 0.0	2.7	0.8 - 5.9	11.1	3.0 - 31.2	34.2	10.9 - 88.7	28.8	8.6 - 75.6	35.5	11.2 - 78.7	0.91
3100203	7573	LITTLE MANATEE	155.5	224.0	66 - 478	0.0	0.0 - 0.0	3.2	1.0 - 6.4	18.0	4.8 - 46.3	42.1	12.1 - 113.0	119.5	33.9 - 410.7	41.2	11.9 - 100.5	0.91
3100204	7574	ALAFIA R	14.9	225.9	85 - 602	0.0	0.0 - 0.0	61.2	21.8 - 128.2	1.9	0.6 - 5.7	3.9	1.4 - 11.0	113.3	41.8 - 345.4	45.7	18.3 - 109.7	1.00
3100204	7575	FISHHAWK CR	60.5	471.5	150 - 988	0.0	0.0 - 0.0	3.8	1.3 - 7.2	13.7	4.2 - 39.8	30.4	10.9 - 67.2	380.8	115.1 - 888.[illegible]	42.7	15.9 - 105.0	0.96
3100204	7576	ALAFIA R	32.9	79.9	24 - 175	0.0	0.0 - 0.0	47.9	14.8 - 101.9	2.3	0.6 - 6.6	4.8	1.5 - 12.8	0.0	0.0 - 0.0	24.9	7.5 - 55.6	0.96
3100204	7577	ALAFIA R	23.3	385.4	132 - 859	0.0	0.0 - 0.0	13.2	4.3 - 28.4	13.0	3.6 - 43.2	27.6	8.3 - 68.4	272.5	92.8 - 789.8	59.0	20.6 - 134.6	0.95
3100204	7578	ALAFIA R, S PRON	78.9	261.2	91 - 610	12.0	3.9 - 27.8	7.3	2.7 - 16.8	14.2	4.5 - 42.9	28.9	9.2 - 71.9	150.4	48.9 - 451.8	48.4	16.0 - 120.4	0.93
3100204	7579	ALAFIA R, N PRON	18.0	1956.8	557 - 4858	0.0	0.0 - 0.0	6.7	2.1 - 12.6	11.1	2.1 - 35.0	21.8	5.9 - 61.1	1848.5	542.7 - 4657	68.8	19.4 - 184.7	0.93
3100204	7580	*A	234.2	1289.9	432 - 3053	209.4	71.4 - 444.8	30.2	8.9 - 60.0	5.5	1.5 - 18.4	3.8	1.3 - 8.7	988.4	332.9 - 2592	52.7	17.6 - 126.5	0.89
3100204	7581	*B	95.7	271.0	110 - 640	0.0	0.0 - 0.0	33.4	12.0 - 96.2	8.3	2.7 - 26.2	19.4	8.2 - 52.6	167.7	65.2 - 589.1	42.1	14.9 - 109.0	0.89
3100204	7582	TURKEY CR	1.9	75.6	25 - 163	0.0	0.0 - 0.0	21.5	7.3 - 46.0	5.3	1.7 - 15.6	11.8	3.7 - 27.4	0.0	0.0 - 0.0	37.1	13.0 - 80.5	0.95
3100204	7583	*C	62.7	744.9	266 - 1820	26.5	10.4 - 57.4	19.1	6.4 - 40.8	12.3	4.4 - 31.9	26.6	9.6 - 72.2	615.9	214.2 - 1706	44.4	18.5 - 103.4	0.93
3100204	7584	TURKEY CR	60.3	559.3	172 - 1388	10.5	3.3 - 23.3	27.1	8.1 - 58.8	5.5	1.3 - 15.3	11.8	3.8 - 30.2	470.6	142.3 - 1411	33.9	12.1 - 74.9	0.93
3100205	7585	HILLSBOROUGH R	7.4	131.1	52 - 263	0.0	0.0 - 0.0	92.3	33.2 - 199.8	0.0	0.0 - 0.1	0.0	0.0 - 0.0	0.0	0.0 - 0.0	38.8	14.0 - 95.8	1.00
3100205	7586	HILLSBOROUGH R	14.2	61.3	20 - 155	0.0	0.0 - 0.0	3.7	1.2 - 8.5	0.3	0.1 - 0.9	0.0	0.0 - 0.0	0.0	0.0 - 0.0	57.3	18.2 - 152.1	0.95
3100205	7587	MILL CR	2.0	54.1	16 - 118	0.0	0.0 - 0.0	17.5	5.4 - 36.0	6.0	1.4 - 17.3	12.1	3.5 - 27.3	0.0	0.0 - 0.0	18.4	5.6 - 45.7	0.92
3100205	7588	HILLSBOROUGH R	74.2	73.1	24 - 179	0.0	0.0 - 0.0	10.5	3.4 - 23.9	8.8	2.2 - 33.0	19.4	6.4 - 56.0	0.0	0.0 - 0.0	34.4	10.9 - 86.4	0.92
3100205	7589	HILLSBOROUGH R	7.0	42.3	13 - 128	0.0	0.0 - 0.0	2.0	0.6 - 6.0	2.9	0.8 - 13.2	6.6	2.4 - 24.0	0.0	0.0 - 0.0	30.8	9.7 - 101.1	0.90
3100205	7590	BLACKWATER CR	45.6	150.5	48 - 285	47.9	16.0 - 101.6	10.5	3.3 - 18.6	17.0	4.5 - 49.8	37.2	12.6 - 87.5	0.2	0.1 - 0.5	37.7	11.8 - 90.1	0.89

8-digit HUC	ID	Name	Area	Catchment Yield		Point sources		Developed Land		Manure		Agricultural Land		Phosphate Mines		Soil parent rock		Frac
				kg/km^2	90% CI	kg/km^2	90% CI	kg/km^2	90% CI	kg/km^2	90% CI	kg/km^2	90% CI	kg/km^2	90% CI	kg/km^2	90% CI	
3100205	7591	*A	14.6	126.2	40 - 277	0.0	0.0 - 0.0	0.6	0.2 - 1.2	21.4	6.0 - 70.4	50.1	15.3 - 113.8	0.0	0.0 - 0.0	54.1	19.4 - 163.6	0.84
3100205	7592	*B	45.3	122.0	48 - 279	4.1	1.5 - 10.0	37.0	13.1 - 92.0	8.9	2.8 - 31.6	19.7	7.4 - 61.6	9.4	3.5 - 31.0	43.0	15.5 - 108.0	0.79
3100205	7593	*C	93.9	69.8	21 - 140	14.4	5.0 - 31.1	14.3	4.2 - 32.4	1.9	0.6 - 4.8	4.8	1.6 - 15.1	22.1	7.2 - 67.3	12.3	3.7 - 28.3	0.79
3100205	7594	BLACKWATER CR	103.2	60.0	22 - 131	0.0	0.0 - 0.0	19.0	6.8 - 39.1	4.8	1.7 - 11.7	12.1	3.9 - 27.0	0.0	0.0 - 0.0	24.1	8.5 - 51.8	0.84
3100205	7595	HILLSBOROUGH R	33.9	66.9	22 - 150	0.0	0.0 - 0.0	21.4	6.9 - 44.2	5.7	1.8 - 15.8	13.0	4.7 - 29.6	0.0	0.0 - 0.0	26.8	9.0 - 63.7	0.89
3100205	7596	*D	115.1	90.0	29 - 204	0.0	0.0 - 0.0	22.3	7.6 - 45.3	11.8	3.7 - 30.2	26.9	7.8 - 61.2	0.0	0.0 - 0.0	28.9	9.6 - 81.7	0.90
3100205	7597	TROUT CR	91.6	8.4	2 - 19	0.0	0.0 - 0.0	3.4	0.9 - 8.4	0.3	0.1 - 0.9	0.5	0.1 - 1.3	0.0	0.0 - 0.0	4.3	1.3 - 10.2	0.95
3100206	7598	BULLFROG CR	33.0	158.6	63 - 286	0.0	0.0 - 0.0	47.8	16.4 - 92.1	6.9	2.3 - 19.4	14.6	5.2 - 34.7	46.3	16.9 - 113.0	43.0	17.1 - 92.4	1.00
3100206	7599	*A	161.7	124.8	40 - 280	13.6	4.9 - 30.5	79.8	26.7 - 180.4	0.7	0.2 - 2.4	1.4	0.4 - 3.6	0.0	0.0 - 0.0	29.3	10.0 - 71.9	1.00
3100206	7600	SWEETWATER CR	61.5	109.7	36 - 220	0.0	0.0 - 0.0	77.8	23.7 - 164.7	0.0	0.0 - 0.1	0.0	0.0 - 0.0	0.0	0.0 - 0.0	31.9	10.6 - 78.4	1.00
3100206	7601	ROCKY CR	188.1	81.3	31 - 165	8.8	3.5 - 19.6	44.1	16.0 - 94.5	0.8	0.2 - 2.2	1.4	0.6 - 3.7	0.0	0.0 - 0.0	26.3	9.6 - 68.3	1.00
3100206	7602	BRUSHY CR	70.2	57.9	18 - 135	0.0	0.0 - 0.0	35.8	10.2 - 80.6	0.3	0.1 - 1.0	0.6	0.2 - 1.6	0.0	0.0 - 0.0	21.1	7.3 - 61.3	0.97
3100206	7603	ROCKY CR	1.1	86.6	31 - 205	0.0	0.0 - 0.0	72.6	25.1 - 165.7	0.0	0.0 - 0.0	0.0	0.0 - 0.0	0.0	0.0 - 0.0	13.9	5.1 - 36.5	0.97
3100207	7604	ANCLOTE R	131.6	80.6	29 - 161	2.7	1.0 - 6.5	51.7	19.0 - 101.5	1.8	0.6 - 5.0	3.8	1.3 - 9.6	0.0	0.0 - 0.0	20.6	7.1 - 49.8	1.00
3100207	7605	PITHLACHASCOTE	166.5	45.0	17 - 77	0.0	0.0 - 0.0	23.8	8.0 - 42.3	1.1	0.4 - 2.9	2.3	0.9 - 5.5	0.0	0.0 - 0.0	17.8	6.8 - 38.1	1.00
3100208	7606	WITHLACOOCHEE	181.0	10.2	4 - 23	0.0	0.0 - 0.0	3.4	1.1 - 8.1	0.5	0.2 - 1.6	1.6	0.5 - 4.1	0.0	0.0 - 0.1	4.7	1.8 - 11.7	0.09
3100208	7607	WITHLACOOCHEE	815.2	10.9	4 - 19	0.0	0.0 - 0.0	2.7	0.8 - 5.8	1.0	0.3 - 2.8	2.8	0.9 - 6.9	0.0	0.0 - 0.0	4.4	1.6 - 8.6	0.09
3100208	7608	WITHLACOOCHEE	359.7	42.4	16 - 91	0.0	0.0 - 0.0	17.5	6.3 - 39.3	3.9	1.3 - 10.4	8.8	3.3 - 22.6	0.0	0.0 - 0.0	12.2	4.9 - 29.2	0.08
3100208	7609	*A	445.8	66.0	20 - 148	0.0	0.0 - 0.0	7.7	2.2 - 18.2	4.1	1.1 - 11.0	13.5	4.3 - 33.5	25.0	7.6 - 84.9	15.7	4.7 - 34.4	0.07
3100208	7610	WITHLACOOCHEE	544.9	3.1	1 - 10	0.0	0.0 - 0.0	0.1	0.0 - 0.5	0.3	0.0 - 0.8	1.0	0.2 - 3.9	0.0	0.0 - 0.0	1.7	0.4 - 5.3	0.07
3100208	7611	LITTLE WITHLACO	560.9	59.1	22 - 115	0.0	0.0 - 0.0	4.0	1.6 - 8.5	7.0	2.2 - 16.8	19.9	7.0 - 50.1	0.0	0.0 - 0.0	28.2	9.6 - 63.1	0.08
3100208	7612	*B	864.8	30.5	11 - 64	0.0	0.0 - 0.0	12.5	4.5 - 26.6	1.9	0.6 - 5.4	6.3	1.9 - 15.5	0.0	0.0 - 0.0	9.8	3.3 - 24.9	0.09
3100208	7613	DEAD R	301.5	44.4	16 - 87	0.0	0.0 - 0.0	18.3	6.0 - 35.3	2.3	0.7 - 6.0	6.4	2.2 - 15.5	0.0	0.0 - 0.1	17.3	6.1 - 41.3	0.09
3110101	7614	SPRING RUN	81.7	6.4	2 - 15	0.0	0.0 - 0.0	0.3	0.1 - 0.8	0.3	0.1 - 1.0	0.0	0.0 - 0.1	0.0	0.0 - 0.0	5.7	1.5 - 13.9	1.00
3110101	7615	COW CR	7.3	3.8	1 - 13	0.0	0.0 - 0.0	0.0	0.0 - 0.0	0.0	0.0 - 0.0	0.0	0.0 - 0.0	0.0	0.0 - 0.0	3.8	0.6 - 13.4	1.00
3110101	7616	TENMILE CR	257.9	32.1	10 - 75	0.0	0.0 - 0.0	7.0	2.3 - 16.5	1.7	0.5 - 5.8	3.6	1.1 - 10.0	0.0	0.0 - 0.0	19.8	6.9 - 50.2	0.99
3110101	7617	COW CR	110.2	24.3	9 - 51	0.0	0.0 - 0.0	2.7	0.9 - 5.6	1.4	0.4 - 3.9	2.4	0.9 - 6.0	0.0	0.0 - 0.0	17.8	6.9 - 42.0	0.99
3110101	7618	WACCASASSA R	7.1	3.7	1 - 12	0.0	0.0 - 0.0	0.0	0.0 - 0.0	0.0	0.0 - 0.0	0.0	0.0 - 0.0	0.0	0.0 - 0.0	3.7	0.6 - 12.5	1.00
3110101	7619	WACCASASSA R	4.8	0.1	0 - 1	0.0	0.0 - 0.0	0.0	0.0 - 0.0	0.0	0.0 - 0.0	0.0	0.0 - 0.0	0.0	0.0 - 0.0	0.1	0.0 - 0.9	1.00
3110101	7620	WEKIVA R	262.0	40.0	15 - 93	0.0	0.0 - 0.0	7.5	2.3 - 19.0	3.8	1.2 - 10.5	10.3	4.3 - 27.7	0.0	0.0 - 0.0	18.4	6.6 - 42.0	0.99
3110101	7621	WACCASASSA R	673.5	25.9	9 - 57	0.0	0.0 - 0.0	4.6	1.7 - 9.9	3.1	1.0 - 8.5	7.3	2.4 - 22.0	0.0	0.0 - 0.0	10.8	4.0 - 24.9	0.99
3110101	7622	OTTER CR	556.1	15.6	5 - 33	0.0	0.0 - 0.0	1.6	0.4 - 3.6	1.7	0.4 - 5.0	2.9	0.8 - 6.8	0.0	0.0 - 0.0	9.4	2.8 - 22.0	1.00
3110102	7623	SANDERS CR	285.0	23.3	9 - 47	0.0	0.0 - 0.0	5.1	1.8 - 10.6	0.5	0.1 - 1.3	0.9	0.3 - 2.3	0.0	0.0 - 0.0	16.9	6.3 - 36.3	1.00
3110102	7624	ROCKY CR	123.1	29.0	9 - 63	0.0	0.0 - 0.0	5.1	1.5 - 10.4	0.5	0.1 - 1.6	1.6	0.5 - 3.8	0.0	0.0 - 0.0	21.7	6.8 - 57.2	1.00
3110102	7625	STEINHATCHEE R	108.9	30.9	11 - 80	0.0	0.0 - 0.0	9.7	3.1 - 23.5	0.7	0.2 - 2.6	1.7	0.5 - 4.5	0.0	0.0 - 0.0	18.8	6.6 - 49.7	1.00
3110102	7626	EIGHTMILE CR	329.1	20.7	7 - 59	0.0	0.0 - 0.0	5.2	1.7 - 11.6	1.0	0.3 - 2.5	1.1	0.3 - 2.8	0.0	0.0 - 0.0	13.4	4.7 - 43.5	0.96
3110102	7627	STEINHATCHEE R	122.8	13.6	5 - 26	0.0	0.0 - 0.0	2.7	1.0 - 5.5	0.3	0.1 - 0.8	0.3	0.1 - 0.7	0.0	0.0 - 0.0	10.3	4.0 - 20.9	0.96
3110102	7628	STEINHATCHEE R	187.2	19.3	6 - 35	0.0	0.0 - 0.0	3.7	1.1 - 6.6	0.9	0.2 - 2.3	0.4	0.1 - 1.0	0.0	0.0 - 0.1	14.2	4.8 - 30.8	0.95
3110102	7629	KETTLE CR	161.6	18.8	6 - 42	0.0	0.0 - 0.0	4.6	1.4 - 10.8	0.5	0.2 - 1.7	0.8	0.2 - 2.2	0.0	0.0 - 0.0	12.9	4.2 - 32.4	0.95

8-digit HUC	ID	Name	Area	Catchment Yield		Point sources		Developed Land		Manure		Agricultural Land		Phosphate Mines		Soil parent rock		Frac
				kg/km^2	90% CI	kg/km^2	90% CI	kg/km^2	90% CI	kg/km^2	90% CI	kg/km^2	90% CI	kg/km^2	90% CI	kg/km^2	90% CI	
3110102	7630	SPRING WARRIOR	299.1	15.3	4 - 36	0.0	0.0 - 0.0	3.8	1.0 - 8.9	0.7	0.2 - 2.1	1.8	0.5 - 4.7	0.0	0.0 - 0.1	9.0	2.4 - 22.7	1.00
3110102	7631	FENHOLLOWAY R	169.3	23.1	7 - 46	0.0	0.0 - 0.0	5.1	1.7 - 10.0	0.6	0.2 - 1.9	2.7	1.0 - 6.0	0.0	0.0 - 0.0	14.7	4.8 - 34.8	1.00
3110102	7632	FENHOLLOWAY R	117.0	24.6	8 - 56	0.0	0.0 - 0.0	11.8	3.7 - 27.1	1.0	0.3 - 3.0	4.1	1.4 - 11.5	0.0	0.0 - 0.0	7.6	2.3 - 19.4	0.97
3110102	7633	SPRING CR	11.1	26.6	7 - 61	0.0	0.0 - 0.0	10.0	2.5 - 20.9	1.0	0.3 - 3.6	5.6	1.4 - 15.7	0.0	0.0 - 0.0	9.9	2.8 - 23.3	0.97
3110102	7634	SPRING CR	91.8	43.3	15 - 89	5.6	2.0 - 12.5	21.7	8.4 - 44.1	1.4	0.4 - 4.3	6.2	2.1 - 16.0	0.0	0.0 - 0.0	8.4	2.9 - 18.6	0.97
3110102	7635	ROCKY CR	190.5	19.0	5 - 43	0.0	0.0 - 0.0	6.6	1.8 - 13.8	0.9	0.2 - 2.7	2.8	0.7 - 7.7	0.0	0.0 - 0.0	8.8	2.6 - 20.8	0.97
3110102	7636	ECONFINA R	383.0	17.3	7 - 37	0.0	0.0 - 0.0	4.3	1.4 - 9.8	0.8	0.3 - 2.6	1.0	0.3 - 2.4	0.0	0.0 - 0.0	11.2	4.3 - 26.1	1.00
3110103	7637	AUCILLA R	79.4	30.8	12 - 78	0.0	0.0 - 0.0	2.5	1.0 - 5.4	0.0	0.0 - 0.1	0.1	0.0 - 0.3	0.0	0.0 - 0.0	28.2	10.9 - 72.3	1.00
3110103	7638	AUCILLA R	43.3	28.8	11 - 64	0.0	0.0 - 0.0	5.3	1.9 - 12.2	0.5	0.2 - 1.6	1.3	0.5 - 3.5	0.0	0.0 - 0.0	21.7	8.3 - 55.7	0.99
3110103	7639	AUCILLA R	1066.6	29.2	10 - 62	0.3	0.1 - 0.6	3.5	1.1 - 9.7	1.9	0.6 - 5.2	5.0	1.6 - 12.9	0.0	0.0 - 0.0	18.4	6.0 - 44.0	0.97
3110103	7640	LITTLE AUCILLA F	472.4	32.6	10 - 69	0.0	0.0 - 0.0	2.6	0.9 - 5.6	2.3	0.7 - 6.8	7.3	2.8 - 17.1	0.0	0.0 - 0.0	20.4	6.8 - 47.5	0.89
3110103	7641	AUCILLA R	81.4	46.5	18 - 119	0.0	0.0 - 0.0	5.7	2.0 - 15.7	3.3	1.0 - 10.1	11.1	4.0 - 30.6	0.0	0.0 - 0.0	26.4	9.7 - 66.9	0.89
3110103	7642	*A	31.2	36.2	14 - 72	0.0	0.0 - 0.0	1.7	0.6 - 3.8	1.9	0.6 - 5.1	10.5	3.6 - 24.4	0.0	0.0 - 0.0	22.1	8.0 - 45.6	0.86
3110103	7643	AUCILLA R	1.5	38.1	13 - 103	0.0	0.0 - 0.0	0.0	0.0 - 0.0	1.6	0.5 - 4.5	5.9	1.9 - 20.2	0.0	0.0 - 0.0	30.6	11.0 - 87.3	0.86
3110103	7644	AUCILLA R	188.8	43.0	15 - 78	0.6	0.2 - 1.0	5.7	2.1 - 11.6	1.9	0.6 - 5.0	13.9	5.0 - 31.1	0.0	0.0 - 0.0	20.9	7.5 - 45.9	0.85
3110103	7645	AUCILLA R	115.9	40.6	12 - 79	0.0	0.0 - 0.0	9.8	2.8 - 21.7	1.5	0.4 - 3.9	12.5	4.2 - 32.0	0.0	0.0 - 0.0	16.8	5.9 - 33.8	0.70
3110103	7646	OLIVE CR	53.9	45.7	17 - 103	0.0	0.0 - 0.0	18.1	5.6 - 43.3	0.8	0.3 - 2.4	6.7	2.2 - 16.9	0.0	0.0 - 0.0	20.1	7.3 - 47.4	0.70
3110103	7647	*B	123.6	37.7	15 - 70	0.0	0.0 - 0.0	4.4	1.6 - 9.3	1.5	0.5 - 3.5	7.5	2.5 - 17.4	0.0	0.0 - 0.0	24.3	8.3 - 55.7	0.85
3110103	7648	*C	105.0	24.4	9 - 59	0.0	0.0 - 0.0	3.9	1.2 - 8.5	1.1	0.3 - 3.2	3.1	1.1 - 7.9	0.0	0.0 - 0.0	16.3	5.9 - 45.2	0.97
3110103	7649	WACISSA R	315.3	25.6	7 - 50	0.0	0.0 - 0.0	4.4	1.2 - 8.9	0.7	0.2 - 2.0	2.0	0.5 - 4.8	0.0	0.0 - 0.0	18.4	5.5 - 36.2	0.99
3110201	7650	SUWANNEE R	143.1	37.2	11 - 71	0.0	0.0 - 0.0	6.7	2.1 - 13.3	3.8	1.1 - 9.4	8.8	3.0 - 20.2	0.0	0.0 - 0.0	17.9	5.5 - 37.1	0.94
3110201	7651	SUWANNEE R	109.9	47.2	16 - 120	0.0	0.0 - 0.0	7.0	2.3 - 15.6	4.0	1.2 - 13.8	7.7	2.8 - 19.6	0.0	0.0 - 0.0	28.5	9.6 - 73.4	0.94
3110201	7652	*A	73.0	41.6	11 - 72	0.0	0.0 - 0.0	6.4	2.0 - 11.3	5.8	1.6 - 13.1	8.3	2.1 - 23.7	0.0	0.0 - 0.0	21.2	6.9 - 45.2	0.91
3110201	7653	SUWANNEE R	254.9	3371.8	1111 - 8344	3300.8	1087.8 - 8201.	8.2	2.9 - 19.0	2.1	0.6 - 6.1	4.6	1.5 - 12.8	36.8	12.8 - 90.7	19.3	6.3 - 45.2	0.91
3110201	7654	ROBINSON CR	210.3	21.4	7 - 54	0.0	0.0 - 0.0	4.2	1.2 - 9.4	0.6	0.2 - 1.6	1.3	0.5 - 3.9	0.2	0.1 - 0.8	15.1	5.4 - 39.8	0.90
3110201	7655	SUWANNEE R	21.6	27.3	9 - 66	0.0	0.0 - 0.0	2.5	0.8 - 6.4	2.1	0.7 - 5.7	7.9	2.7 - 21.8	0.0	0.0 - 0.1	14.8	5.2 - 36.7	0.90
3110201	7656	DEEP CR	159.2	6.7	2 - 15	0.0	0.0 - 0.0	1.2	0.3 - 2.9	0.2	0.1 - 0.6	0.4	0.1 - 1.1	0.0	0.0 - 0.1	4.9	1.4 - 11.6	0.90
3110201	7657	SUWANNEE R	461.5	19.9	6 - 40	0.0	0.0 - 0.0	3.2	1.0 - 7.1	0.9	0.3 - 2.0	1.4	0.4 - 4.0	2.7	0.7 - 7.0	11.8	3.7 - 24.7	0.90
3110201	7658	SUWANNEE R	78.8	8.4	2 - 21	0.0	0.0 - 0.0	1.5	0.4 - 3.5	0.1	0.0 - 0.3	0.3	0.1 - 0.9	0.0	0.0 - 0.0	6.5	1.7 - 15.9	0.87
3110201	7659	*B	339.0	2.9	1 - 6	0.0	0.0 - 0.0	0.7	0.2 - 1.6	0.1	0.0 - 0.2	0.1	0.0 - 0.2	0.0	0.0 - 0.1	2.0	0.6 - 4.5	0.87
3110201	7660	SUWANNEE R	14.0	26.2	9 - 70	0.0	0.0 - 0.0	4.2	1.3 - 10.4	0.1	0.0 - 0.2	0.2	0.0 - 0.4	0.0	0.0 - 0.0	21.8	7.1 - 59.0	0.87
3110201	7661	SUWANNEE R	16.0	21.7	7 - 55	0.0	0.0 - 0.0	6.1	2.0 - 15.7	0.1	0.0 - 0.3	0.4	0.1 - 1.4	0.0	0.0 - 0.0	15.0	5.1 - 44.5	0.86
3110201	7662	SUWANNEE R	41.2	8.6	3 - 21	0.0	0.0 - 0.0	2.0	0.7 - 5.3	0.1	0.0 - 0.2	0.1	0.0 - 0.1	0.0	0.0 - 0.0	6.5	2.2 - 16.1	0.85
3110201	7663	SUWANNEE R	353.8	1.5	1 - 3	0.0	0.0 - 0.0	0.3	0.1 - 0.7	0.0	0.0 - 0.0	0.0	0.0 - 0.0	0.0	0.0 - 0.0	1.2	0.4 - 2.7	0.85
3110201	7664	SUWANNEE R	5.8	1.3	0 - 3	0.0	0.0 - 0.0	0.7	0.2 - 1.7	0.0	0.0 - 0.0	0.0	0.0 - 0.0	0.0	0.0 - 0.0	0.5	0.2 - 1.2	0.84
3110201	7665	SUWANNEE R, M F	786.1	0.6	0 - 1	0.0	0.0 - 0.0	0.1	0.0 - 0.2	0.0	0.0 - 0.0	0.0	0.0 - 0.1	0.0	0.0 - 0.0	0.4	0.1 - 1.1	0.84
3110201	7666	BLACK R	267.4	15.1	5 - 29	0.0	0.0 - 0.0	2.4	0.7 - 4.1	0.6	0.2 - 1.4	1.6	0.5 - 3.5	0.0	0.0 - 0.0	10.5	3.3 - 21.6	0.76
3110201	7667	SUWANNEE R, GRI	284.5	8.6	3 - 17	0.0	0.0 - 0.0	1.5	0.5 - 3.2	0.3	0.1 - 0.8	0.8	0.3 - 2.1	0.0	0.0 - 0.0	6.0	2.2 - 13.2	0.82
3110201	7668	SUWANNEE CR	464.0	18.1	5 - 43	1.2	0.4 - 2.9	4.2	1.3 - 9.8	0.3	0.1 - 0.9	1.5	0.5 - 4.3	0.0	0.0 - 0.0	10.9	3.1 - 27.0	0.82

8-digit HUC	ID	Name	Area	Catchment Yield		Point sources		Developed Land		Manure		Agricultural Land		Phosphate Mines		Soil parent rock		Frac
				kg/km^2	90% CI	kg/km^2	90% CI	kg/km^2	90% CI	kg/km^2	90% CI	kg/km^2	90% CI	kg/km^2	90% CI	kg/km^2	90% CI	
3110201	7669	*A	39.0	1.2	0 - 4	0.0	0.0 - 0.0	0.2	0.0 - 0.5	0.0	0.0 - 0.0	0.0	0.0 - 0.0	0.0	0.0 - 0.0	1.0	0.3 - 3.0	0.84
3110201	7670	*B	22.3	0.3	0 - 1	0.0	0.0 - 0.0	0.0	0.0 - 0.0	0.0	0.0 - 0.0	0.0	0.0 - 0.0	0.0	0.0 - 0.0	0.3	0.1 - 0.7	0.82
3110201	7671	*C	112.7	9.0	3 - 21	0.0	0.0 - 0.0	1.5	0.4 - 3.5	0.2	0.0 - 0.5	0.4	0.1 - 1.0	0.0	0.0 - 0.0	7.0	2.3 - 18.7	0.79
3110201	7672	*D	73.9	14.4	5 - 34	0.0	0.0 - 0.0	2.6	0.8 - 6.8	0.1	0.0 - 0.3	0.7	0.2 - 1.9	0.0	0.0 - 0.0	11.0	3.2 - 26.8	0.79
3110201	7673	*E	104.4	9.2	3 - 19	0.0	0.0 - 0.0	2.0	0.6 - 3.8	0.1	0.0 - 0.1	0.2	0.1 - 0.5	0.0	0.0 - 0.0	7.0	2.2 - 16.3	0.82
3110201	7674	*F	151.5	12.8	4 - 29	0.0	0.0 - 0.0	2.6	0.8 - 5.3	0.1	0.0 - 0.2	0.2	0.1 - 0.4	0.0	0.0 - 0.0	10.0	3.2 - 24.9	0.85
3110201	7675	TATUM CR	249.9	15.2	5 - 33	0.0	0.0 - 0.0	3.2	1.0 - 6.9	0.1	0.0 - 0.2	0.2	0.1 - 0.5	0.0	0.0 - 0.0	11.7	3.9 - 28.8	0.85
3110201	7676	SUWANOOCHEE C	1145.0	18.1	5 - 37	0.0	0.0 - 0.0	3.3	1.0 - 6.9	0.3	0.1 - 0.8	1.1	0.4 - 2.5	0.0	0.0 - 0.0	13.4	4.1 - 31.1	0.86
3110201	7677	TOMS CR	394.6	25.3	7 - 45	0.0	0.0 - 0.0	3.3	0.9 - 6.3	0.5	0.1 - 1.2	0.8	0.2 - 1.6	5.6	1.4 - 16.8	15.1	4.6 - 36.7	0.87
3110202	7679	ALPAHA R	563.1	26.8	8 - 50	0.0	0.0 - 0.0	4.0	1.2 - 7.5	0.4	0.1 - 0.9	4.4	1.3 - 10.1	0.0	0.0 - 0.0	17.9	5.2 - 43.0	0.92
3110202	7680	ALPAHA R	369.5	34.3	13 - 77	0.0	0.0 - 0.0	3.8	1.2 - 9.9	2.9	0.9 - 9.1	10.4	3.5 - 27.1	0.0	0.0 - 0.0	17.1	5.9 - 45.0	0.86
3110202	7681	WILLACOOCHEE F	100.5	40.9	14 - 76	0.0	0.0 - 0.0	3.4	1.0 - 7.4	6.2	1.8 - 15.9	19.8	6.3 - 55.4	0.0	0.0 - 0.0	11.5	4.0 - 28.8	0.81
3110202	7682	WILLACOOCHEE F	237.3	44.4	16 - 122	9.5	3.4 - 25.5	9.3	3.3 - 22.6	1.9	0.6 - 5.8	15.6	4.9 - 48.3	0.0	0.0 - 0.0	8.1	2.7 - 22.1	0.76
3110202	7683	REEDY CR	253.7	34.5	11 - 85	0.0	0.0 - 0.0	6.3	2.0 - 15.4	1.5	0.4 - 4.8	18.4	5.8 - 51.0	0.0	0.0 - 0.0	8.3	2.6 - 23.8	0.76
3110202	7684	ALPAHA R	403.8	26.6	10 - 56	0.0	0.0 - 0.0	3.7	1.3 - 7.8	1.2	0.4 - 3.0	10.5	3.6 - 28.3	0.0	0.0 - 0.0	11.2	3.8 - 24.8	0.81
3110202	7685	ALPAHA R	141.8	33.1	12 - 77	0.0	0.0 - 0.0	4.9	1.5 - 12.6	2.1	0.7 - 7.9	17.1	6.5 - 43.8	0.0	0.0 - 0.0	8.9	3.2 - 22.6	0.69
3110202	7686	BIG CR	80.4	24.9	9 - 53	0.0	0.0 - 0.0	3.7	1.1 - 7.4	1.3	0.4 - 2.9	13.2	4.3 - 35.6	0.0	0.0 - 0.0	6.8	2.5 - 14.4	0.66
3110202	7687	ALPAHA R	25.9	22.8	6 - 65	0.0	0.0 - 0.0	4.4	1.3 - 12.9	0.9	0.2 - 2.7	8.8	2.5 - 29.7	0.0	0.0 - 0.0	8.7	2.4 - 26.5	0.66
3110202	7688	ALPAHA R	92.7	23.7	10 - 44	0.0	0.0 - 0.0	3.1	1.2 - 5.9	1.7	0.6 - 4.4	11.3	4.5 - 25.9	0.0	0.0 - 0.0	7.5	2.9 - 16.2	0.63
3110202	7689	ALPAHA R	87.7	28.9	9 - 70	0.0	0.0 - 0.0	4.8	1.3 - 10.4	3.0	0.8 - 7.5	13.6	4.0 - 38.7	0.0	0.0 - 0.0	7.4	2.5 - 20.2	0.56
3110202	7690	MILL CR	54.4	29.6	9 - 68	0.0	0.0 - 0.0	7.9	2.7 - 18.8	3.1	0.9 - 10.5	12.4	4.4 - 37.5	0.0	0.0 - 0.0	6.1	2.1 - 17.1	0.47
3110202	7691	ALPAHA R	197.8	26.2	9 - 51	0.0	0.0 - 0.0	4.6	1.3 - 10.0	2.3	0.7 - 5.4	11.6	3.4 - 26.6	0.0	0.0 - 0.0	7.7	2.9 - 16.5	0.47
3110202	7692	DOUBLE RUN CR	95.1	28.8	10 - 88	0.0	0.0 - 0.0	4.3	1.1 - 11.8	2.6	0.7 - 9.2	12.5	3.9 - 41.2	0.0	0.0 - 0.0	9.4	3.1 - 29.5	0.56
3110202	7693	DEEP CR	140.4	32.8	13 - 70	0.0	0.0 - 0.0	4.2	1.4 - 10.9	2.7	0.9 - 6.9	15.2	5.4 - 41.3	0.0	0.0 - 0.0	10.7	4.1 - 22.9	0.63
3110202	7694	DEEP CR	154.2	31.1	11 - 80	0.0	0.0 - 0.0	4.2	1.4 - 9.5	1.5	0.5 - 4.1	15.9	5.4 - 45.8	0.0	0.0 - 0.0	9.5	3.4 - 22.5	0.52
3110202	7695	DEEP CR, W FK	181.4	31.8	10 - 72	0.0	0.0 - 0.0	5.9	2.0 - 15.4	1.8	0.5 - 5.8	14.8	5.1 - 38.6	0.0	0.0 - 0.0	9.3	3.0 - 22.2	0.52
3110202	7696	MIDDLE CR	3.0	23.3	9 - 65	0.0	0.0 - 0.0	1.8	0.5 - 4.2	1.0	0.3 - 3.3	11.7	4.3 - 37.6	0.0	0.0 - 0.0	8.8	3.3 - 23.4	0.69
3110202	7697	HAT CR	119.7	42.5	15 - 98	9.4	3.0 - 23.1	8.0	2.6 - 18.2	2.3	0.7 - 6.9	14.6	4.8 - 37.7	0.0	0.0 - 0.0	8.2	3.1 - 17.5	0.68
3110202	7698	MIDDLE CR	64.2	24.6	9 - 50	0.0	0.0 - 0.0	7.3	2.5 - 15.0	1.2	0.4 - 3.2	9.4	3.0 - 23.1	0.0	0.0 - 0.0	6.6	2.4 - 14.0	0.68
3110202	7699	BIG CR	103.8	13.9	4 - 32	0.0	0.0 - 0.0	1.9	0.5 - 4.5	0.4	0.1 - 1.4	4.9	1.6 - 15.7	0.0	0.0 - 0.0	6.6	1.9 - 17.4	0.86
3110202	7700	FIVEMILE CR	85.9	44.6	17 - 120	0.0	0.0 - 0.0	4.0	1.5 - 11.3	2.0	0.6 - 6.1	16.7	6.2 - 47.4	0.0	0.0 - 0.0	21.8	8.4 - 64.4	0.82
3110202	7701	TENMILE CR	97.0	45.1	14 - 84	0.0	0.0 - 0.0	4.3	1.4 - 8.0	2.0	0.7 - 5.1	18.0	5.2 - 40.8	0.0	0.0 - 0.0	20.8	6.7 - 47.0	0.82
3110202	7703	GRAND BAY CR	316.9	15.1	5 - 32	0.0	0.0 - 0.0	2.5	0.8 - 6.1	0.3	0.1 - 1.0	3.1	1.1 - 8.3	0.0	0.0 - 0.0	9.2	3.5 - 21.9	0.90
3110202	7704	MUD CR	250.2	41.1	17 - 97	12.3	5.1 - 32.8	9.8	3.8 - 22.3	0.7	0.3 - 2.2	6.4	2.2 - 15.8	0.0	0.0 - 0.0	11.8	4.8 - 28.0	0.90
3110203	7705	WITHLACOOCHEE	719.3	29.3	11 - 54	0.0	0.0 - 0.0	6.3	2.2 - 13.4	2.5	0.7 - 6.2	7.3	2.4 - 16.2	0.0	0.0 - 0.0	13.2	4.3 - 27.7	0.94
3110203	7706	*A	55.9	39.1	14 - 74	0.0	0.0 - 0.0	7.2	2.2 - 13.9	1.1	0.4 - 3.2	9.8	3.2 - 27.3	0.0	0.0 - 0.0	20.9	7.7 - 46.0	0.90
3110203	7707	WITHLACOOCHEE	34.4	33.4	14 - 70	0.0	0.0 - 0.0	6.3	2.5 - 13.7	1.0	0.4 - 2.5	6.4	2.3 - 16.2	0.0	0.0 - 0.0	19.7	7.7 - 42.7	0.90
3110203	7708	WITHLACOOCHEE	47.0	33.2	13 - 60	0.0	0.0 - 0.0	3.6	1.2 - 7.0	0.8	0.2 - 2.1	5.2	1.9 - 11.2	0.0	0.0 - 0.0	23.6	8.9 - 48.5	0.88
3110203	7709	WITHLACOOCHEE	159.2	49.0	17 - 100	0.0	0.0 - 0.0	27.3	9.9 - 61.5	0.8	0.2 - 2.3	7.5	2.7 - 22.3	0.0	0.0 - 0.0	13.4	4.3 - 37.0	0.86

8-digit HUC	ID	Name	Area	Catchment Yield		Point sources		Developed Land		Manure		Agricultural Land		Phosphate Mines		Soil parent rock		Frac
				kg/km^2	90% CI	kg/km^2	90% CI	kg/km^2	90% CI	kg/km^2	90% CI	kg/km^2	90% CI	kg/km^2	90% CI	kg/km^2	90% CI	
3110203	7710	CAT CR	198.4	34.2	11 - 75	0.0	0.0 - 0.0	6.5	2.0 - 13.2	1.5	0.4 - 4.8	13.3	3.8 - 33.6	0.0	0.0 - 0.0	12.9	4.0 - 32.6	0.83
3110203	7711	WITHLACOOCHEE	39.6	30.6	9 - 66	0.0	0.0 - 0.0	3.8	1.2 - 7.5	1.2	0.3 - 3.0	12.5	3.8 - 30.8	0.0	0.0 - 0.0	13.0	4.1 - 30.7	0.83
3110203	7712	WITHLACOOCHEE	80.0	34.8	10 - 70	0.0	0.0 - 0.0	3.7	1.0 - 7.0	1.6	0.4 - 4.7	15.8	4.2 - 39.0	0.0	0.0 - 0.0	13.8	4.0 - 32.4	0.82
3110203	7713	WITHLACOOCHEE	5.6	44.6	17 - 90	0.0	0.0 - 0.0	4.4	1.6 - 8.8	1.8	0.6 - 4.6	21.0	7.7 - 49.6	0.0	0.0 - 0.0	17.3	6.1 - 44.2	0.79
3110203	7714	WITHLACOOCHEE	8.6	35.3	13 - 76	0.0	0.0 - 0.0	1.9	0.7 - 4.3	1.8	0.5 - 5.9	17.2	6.3 - 46.0	0.0	0.0 - 0.0	14.4	5.0 - 40.7	0.79
3110203	7715	WITHLACOOCHEE	345.0	40.1	14 - 93	0.0	0.0 - 0.0	5.5	1.6 - 14.0	2.0	0.5 - 6.7	17.7	5.9 - 47.9	0.0	0.0 - 0.0	14.8	4.3 - 37.5	0.78
3110203	7716	NEW R	24.6	39.9	13 - 81	0.0	0.0 - 0.0	4.8	1.4 - 9.4	1.7	0.5 - 4.5	19.8	6.1 - 50.4	0.0	0.0 - 0.0	13.6	4.8 - 31.5	0.78
3110203	7717	NEW R	122.4	34.2	12 - 77	0.0	0.0 - 0.0	4.3	1.3 - 9.2	1.6	0.4 - 4.5	15.5	5.1 - 47.2	0.0	0.0 - 0.0	12.7	4.6 - 28.1	0.76
3110203	7718	NEW R	145.3	83.8	32 - 187	44.1	16.2 - 112.2	12.1	4.1 - 24.7	1.9	0.6 - 5.1	14.9	5.2 - 32.7	0.0	0.0 - 0.0	10.8	4.3 - 27.2	0.66
3110203	7719	*B	31.0	38.0	11 - 98	0.0	0.0 - 0.0	6.9	1.9 - 15.6	2.0	0.5 - 5.0	18.8	6.6 - 59.6	0.0	0.0 - 0.0	10.4	3.2 - 26.3	0.66
3110203	7720	BRUSHY CR	74.6	48.4	21 - 96	0.0	0.0 - 0.0	5.7	2.2 - 11.9	1.8	0.6 - 4.8	24.6	9.4 - 60.3	0.0	0.0 - 0.0	16.3	6.7 - 40.3	0.76
3110203	7721	YOUNGS MILL CR	31.8	45.3	16 - 94	0.0	0.0 - 0.0	5.3	1.7 - 12.5	1.7	0.5 - 4.9	23.3	7.4 - 53.6	0.0	0.0 - 0.0	15.0	5.1 - 36.6	0.79
3110203	7722	DAYS CR	105.6	70.2	23 - 139	17.6	5.5 - 38.1	11.7	4.0 - 26.1	1.6	0.4 - 4.7	21.4	6.3 - 49.9	0.2	0.1 - 0.5	17.7	5.6 - 43.7	0.79
3110203	7723	HUTCHINSON MIL	53.8	37.2	13 - 84	0.0	0.0 - 0.0	5.6	2.0 - 12.8	1.4	0.4 - 3.8	17.1	6.3 - 53.7	0.0	0.0 - 0.0	13.1	4.6 - 30.0	0.82
3110203	7724	OKAPILCO CR	1.3	42.2	16 - 106	0.0	0.0 - 0.0	0.0	0.0 - 0.0	0.4	0.1 - 1.0	0.7	0.3 - 1.8	0.0	0.0 - 0.0	41.1	15.7 - 105.5	0.88
3110203	7725	OKAPILCO CR	124.0	37.2	11 - 70	0.0	0.0 - 0.0	7.9	2.4 - 14.9	2.0	0.5 - 5.0	12.3	3.6 - 31.8	0.0	0.0 - 0.0	15.1	5.0 - 31.7	0.88
3110203	7726	OKAPILCO CR	25.1	45.5	18 - 87	0.0	0.0 - 0.0	4.3	1.5 - 8.5	3.5	1.2 - 8.4	22.9	8.2 - 48.8	0.0	0.0 - 0.0	14.8	5.7 - 30.4	0.81
3110203	7727	OKAPILCO CR	414.4	41.5	15 - 85	0.0	0.0 - 0.0	7.2	2.6 - 14.8	3.5	1.3 - 10.4	18.4	5.8 - 42.5	0.0	0.0 - 0.0	12.4	4.6 - 31.9	0.80
3110203	7728	LITTLE CR	50.5	39.8	14 - 77	0.0	0.0 - 0.0	3.4	1.1 - 6.8	2.9	0.9 - 7.3	18.3	5.7 - 43.7	0.0	0.0 - 0.0	15.2	5.0 - 34.3	0.80
3110203	7729	MULE CR	148.6	40.4	13 - 86	0.0	0.0 - 0.0	5.8	1.9 - 11.3	2.7	0.7 - 7.2	18.8	6.2 - 48.7	0.0	0.0 - 0.0	13.2	4.1 - 28.7	0.81
3110203	7730	PISCOLA CR	259.5	41.4	15 - 103	0.0	0.0 - 0.0	7.2	2.4 - 16.3	2.5	0.8 - 7.5	15.9	5.2 - 45.2	0.0	0.0 - 0.0	15.8	4.9 - 34.0	0.88
3110203	7731	*C	56.7	41.1	12 - 96	0.0	0.0 - 0.0	6.1	1.8 - 12.8	2.9	0.9 - 7.6	17.7	6.4 - 46.6	0.0	0.0 - 0.0	14.4	3.9 - 42.0	0.72
3110203	7732	PISCOLA CR	109.3	42.6	17 - 86	0.0	0.0 - 0.0	5.4	2.0 - 11.5	2.4	0.8 - 6.4	21.8	7.4 - 54.5	0.0	0.0 - 0.0	13.1	4.9 - 29.2	0.72
3110204	7733	LITTLE R	10.7	35.6	13 - 62	0.0	0.0 - 0.0	9.1	2.7 - 17.4	1.0	0.2 - 2.3	5.6	1.8 - 11.4	0.0	0.0 - 0.0	20.0	7.2 - 43.5	0.86
3110204	7734	FRANKS CR	121.7	29.9	11 - 68	0.0	0.0 - 0.0	9.4	3.4 - 25.2	0.9	0.3 - 2.4	9.3	2.9 - 26.1	0.0	0.0 - 0.0	10.3	4.3 - 25.5	0.86
3110204	7735	LITTLE R	209.1	40.2	12 - 111	0.0	0.0 - 0.0	5.7	1.5 - 13.1	2.4	0.7 - 8.6	16.1	4.5 - 46.2	0.0	0.0 - 0.0	16.1	5.1 - 43.4	0.86
3110204	7736	MORRISON CR	74.1	44.0	15 - 92	0.0	0.0 - 0.0	8.6	2.9 - 18.0	1.5	0.5 - 4.7	20.3	6.4 - 51.8	0.0	0.0 - 0.0	13.6	5.1 - 32.7	0.83
3110204	7737	LITTLE R	60.4	41.4	16 - 82	0.0	0.0 - 0.0	3.2	1.1 - 6.8	2.3	0.7 - 6.3	18.2	6.0 - 40.6	0.0	0.0 - 0.0	17.7	7.0 - 42.5	0.83
3110204	7738	LITTLE R	155.2	40.9	10 - 91	0.0	0.0 - 0.0	4.8	1.0 - 10.8	2.6	0.6 - 7.2	18.9	4.4 - 50.8	0.0	0.0 - 0.0	14.6	3.6 - 34.4	0.81
3110204	7739	LITTLE R	5.7	47.8	14 - 93	0.0	0.0 - 0.0	2.2	0.7 - 4.4	1.9	0.6 - 6.6	24.4	7.5 - 79.9	0.0	0.0 - 0.0	19.3	6.3 - 48.9	0.77
3110204	7740	LITTLE R	206.7	42.1	16 - 84	0.0	0.0 - 0.0	13.3	4.8 - 30.1	1.9	0.6 - 5.8	15.3	5.6 - 38.0	0.0	0.0 - 0.0	11.6	4.3 - 26.0	0.77
3110204	7741	LITTLE R	128.9	34.0	10 - 64	0.0	0.0 - 0.0	6.2	1.8 - 12.9	2.6	0.7 - 8.2	15.2	4.3 - 35.0	0.0	0.0 - 0.0	10.1	3.1 - 29.4	0.64
3110204	7742	DANIELS CR	85.9	36.7	11 - 82	0.0	0.0 - 0.0	3.8	1.2 - 8.7	2.9	0.8 - 8.7	18.9	4.9 - 47.2	0.0	0.0 - 0.0	11.1	3.0 - 27.2	0.64
3110204	7743	DANIELS CR	48.8	42.8	14 - 75	0.0	0.0 - 0.0	5.8	1.8 - 11.1	3.8	1.1 - 8.7	21.4	6.8 - 49.3	0.0	0.0 - 0.0	11.7	4.0 - 20.2	0.77
3110204	7744	GUM CR	20.2	37.1	12 - 89	0.0	0.0 - 0.0	3.1	1.0 - 7.5	3.6	1.1 - 10.2	15.2	4.8 - 40.5	0.0	0.0 - 0.0	15.1	5.1 - 42.1	0.77
3110204	7745	WARRIOR CR	288.7	36.6	12 - 92	0.0	0.0 - 0.0	5.3	1.6 - 12.9	2.0	0.6 - 5.3	18.6	5.9 - 55.9	0.0	0.0 - 0.0	10.7	3.1 - 23.7	0.76
3110204	7746	GUM CR	123.5	33.9	11 - 69	0.0	0.0 - 0.0	5.0	1.6 - 11.3	2.6	0.8 - 10.1	14.3	4.4 - 39.5	0.0	0.0 - 0.0	12.0	3.8 - 29.5	0.76
3110204	7747	TY TY CR	60.9	40.5	15 - 91	0.0	0.0 - 0.0	5.7	2.1 - 13.9	1.8	0.6 - 5.4	21.6	7.9 - 58.7	0.0	0.0 - 0.0	11.3	4.2 - 26.8	0.69
3110204	7748	GUM CR	38.0	25.7	8 - 55	0.0	0.0 - 0.0	3.5	1.2 - 7.5	1.0	0.3 - 2.7	8.6	2.7 - 22.1	0.0	0.0 - 0.0	12.6	3.9 - 31.7	0.69

8-digit HUC	ID	Name	Area	Catchment Yield		Point sources		Developed Land		Manure		Agricultural Land		Phosphate Mines		Soil parent rock		Frac
				kg/km^2	90% CI	kg/km^2	90% CI	kg/km^2	90% CI	kg/km^2	90% CI	kg/km^2	90% CI	kg/km^2	90% CI	kg/km^2	90% CI	
3110204	7749	GUM CR	58.3	38.7	15 - 77	0.0	0.0 - 0.0	4.1	1.4 - 8.8	1.7	0.5 - 5.4	20.4	7.8 - 47.8	0.0	0.0 - 0.0	12.4	4.5 - 31.0	0.66
3110204	7750	LOLLY CR	77.4	36.6	13 - 83	0.0	0.0 - 0.0	7.9	2.5 - 16.8	1.4	0.4 - 4.7	16.5	5.5 - 41.2	0.0	0.0 - 0.0	10.9	3.9 - 26.8	0.59
3110204	7751	TOWN CR	40.2	43.9	15 - 91	0.0	0.0 - 0.0	21.7	6.9 - 43.7	0.9	0.3 - 2.8	10.7	3.7 - 29.2	0.0	0.0 - 0.0	10.5	3.4 - 25.3	0.59
3110204	7752	HORSE CR	86.2	28.4	9 - 59	0.0	0.0 - 0.0	4.6	1.6 - 9.2	1.0	0.3 - 2.9	11.7	3.9 - 32.7	0.0	0.0 - 0.0	11.1	3.6 - 26.1	0.66
3110204	7753	INDIAN CR	1.4	37.2	13 - 95	0.0	0.0 - 0.0	2.1	0.7 - 5.5	3.5	1.2 - 11.6	14.3	5.1 - 42.7	0.0	0.0 - 0.0	17.3	6.5 - 54.8	0.81
3110204	7754	BULL CR	56.2	36.9	12 - 77	0.0	0.0 - 0.0	5.2	1.8 - 10.4	3.8	1.3 - 9.7	16.5	5.4 - 43.9	0.0	0.0 - 0.0	11.4	4.5 - 25.6	0.80
3110204	7756	BULL CR	97.4	38.6	15 - 73	0.0	0.0 - 0.0	5.2	2.0 - 10.6	4.0	1.3 - 11.2	17.4	7.0 - 43.6	0.0	0.0 - 0.0	12.0	4.5 - 27.9	0.80
3110204	7757	INDIAN CR	127.4	37.5	10 - 68	0.0	0.0 - 0.0	6.3	1.7 - 12.0	3.7	1.1 - 9.0	15.9	3.7 - 43.5	0.0	0.0 - 0.0	11.6	3.5 - 23.1	0.80
3110205	7758	SUWANNEE R	114.5	2.0	1 - 7	0.0	0.0 - 0.0	0.2	0.0 - 0.7	0.0	0.0 - 0.2	0.0	0.0 - 0.1	0.0	0.0 - 0.0	1.8	0.5 - 5.8	1.00
3110205	7759	SUWANNEE R	354.9	29.9	12 - 74	0.0	0.0 - 0.0	8.1	3.0 - 20.0	3.5	1.1 - 9.3	11.1	4.1 - 29.8	0.0	0.0 - 0.0	7.2	2.9 - 20.4	0.99
3110205	7760	SUWANNEE R	216.4	21.1	7 - 47	0.0	0.0 - 0.0	4.6	1.5 - 11.0	3.8	1.1 - 11.2	3.8	1.3 - 9.7	0.0	0.0 - 0.0	8.8	2.9 - 21.3	0.97
3110205	7761	SUWANNEE R	109.8	33.6	10 - 77	0.0	0.0 - 0.0	8.9	2.6 - 18.5	6.8	2.1 - 19.1	10.2	3.4 - 26.3	0.0	0.0 - 0.0	7.7	2.7 - 16.7	0.94
3110205	7762	*A	140.2	49.7	17 - 102	0.0	0.0 - 0.0	7.3	2.2 - 14.4	8.3	2.5 - 20.7	12.4	4.3 - 31.4	0.6	0.2 - 1.3	21.1	7.5 - 53.8	0.00
3110205	7763	*B	533.9	26.6	9 - 48	0.2	0.1 - 0.4	9.9	3.2 - 18.8	1.2	0.4 - 3.0	4.6	1.4 - 11.2	0.0	0.0 - 0.0	10.7	3.6 - 21.7	0.99
3110206	7764	SANTE FE R	82.2	35.1	12 - 75	0.0	0.0 - 0.0	12.2	4.2 - 27.1	5.7	1.8 - 16.8	9.7	3.6 - 26.0	0.0	0.0 - 0.0	7.4	2.9 - 20.0	0.97
3110206	7765	SANTE FE R	69.7	29.2	11 - 59	0.0	0.0 - 0.0	7.8	2.9 - 16.2	2.7	0.9 - 7.2	7.3	2.5 - 14.9	0.0	0.0 - 0.0	11.4	3.3 - 27.4	0.97
3110206	7766	*A	241.9	27.5	12 - 51	0.0	0.0 - 0.0	3.8	1.5 - 8.3	3.0	1.1 - 9.3	6.6	2.5 - 16.5	0.0	0.0 - 0.0	14.1	5.9 - 29.6	0.96
3110206	7767	SANTE FE R	1.4	52.3	18 - 86	0.0	0.0 - 0.0	8.9	2.7 - 15.9	5.8	1.8 - 14.9	23.6	7.8 - 53.7	0.0	0.0 - 0.0	14.1	4.6 - 28.2	0.96
3110206	7768	SANTE FE R	101.0	52.2	18 - 95	0.0	0.0 - 0.0	4.3	1.4 - 7.7	7.1	2.1 - 17.6	21.7	7.1 - 50.7	0.0	0.0 - 0.0	19.1	6.6 - 37.3	0.93
3110206	7769	SANTE FE R	206.4	38.4	13 - 66	0.0	0.0 - 0.0	3.9	1.3 - 7.2	5.6	1.9 - 14.4	10.2	3.5 - 23.3	0.7	0.2 - 1.7	18.0	5.7 - 36.9	0.91
3110206	7770	*B	66.1	25.8	9 - 49	0.0	0.0 - 0.0	1.3	0.4 - 2.4	3.9	1.2 - 8.8	6.8	2.4 - 14.9	0.0	0.0 - 0.0	13.8	4.6 - 30.1	0.85
3110206	7771	SANTE FE R	274.5	32.2	10 - 55	1.2	0.4 - 2.4	6.5	1.8 - 12.5	3.9	1.0 - 9.8	4.0	1.3 - 8.5	1.9	0.5 - 4.5	14.6	4.6 - 30.1	0.85
3110206	7772	SANTE FE R	186.6	18.3	6 - 36	0.3	0.1 - 0.6	3.0	1.0 - 6.3	1.8	0.5 - 4.1	2.6	0.8 - 6.5	0.0	0.0 - 0.0	10.7	3.5 - 21.8	0.78
3110206	7773	*C	23.2	32.1	12 - 82	0.0	0.0 - 0.0	6.9	2.3 - 16.1	3.8	1.4 - 12.3	3.7	1.3 - 11.2	0.0	0.0 - 0.0	17.7	7.0 - 52.2	0.78
3110206	7774	NEW R	229.6	38.3	10 - 69	0.0	0.0 - 0.0	5.7	1.4 - 10.7	6.7	1.7 - 15.9	12.3	3.3 - 30.4	0.1	0.0 - 0.2	13.5	3.5 - 29.0	0.91
3110206	7775	*D	73.9	42.7	15 - 80	0.0	0.0 - 0.0	6.4	2.0 - 13.0	8.9	2.6 - 22.3	11.4	3.7 - 26.0	0.0	0.0 - 0.0	16.0	5.7 - 37.0	0.84
3110206	7776	NEW R	49.9	40.9	12 - 76	0.0	0.0 - 0.0	7.8	2.5 - 17.0	5.8	1.7 - 13.9	11.0	3.3 - 26.0	0.0	0.0 - 0.0	16.3	5.6 - 41.3	0.84
3110206	7777	ALLIGATOR CR	154.3	33.9	10 - 69	0.0	0.0 - 0.0	6.8	1.9 - 14.9	5.0	1.3 - 12.7	5.4	1.8 - 14.3	0.0	0.0 - 0.0	16.6	4.7 - 37.8	0.81
3110206	7778	NEW R	158.3	16.2	5 - 35	0.0	0.0 - 0.0	2.9	1.1 - 6.7	1.1	0.3 - 2.8	0.9	0.3 - 2.5	0.0	0.0 - 0.0	11.3	3.5 - 26.5	0.81
3110206	7779	OLUSTEE R	92.4	54.8	19 - 112	0.0	0.0 - 0.0	8.3	2.8 - 17.1	5.1	1.7 - 14.0	17.7	5.6 - 45.3	0.0	0.0 - 0.0	23.7	7.8 - 60.3	0.93
3110206	7780	SWIFT CR	199.9	21.3	7 - 37	0.0	0.0 - 0.0	3.5	1.1 - 7.0	1.7	0.5 - 3.9	2.6	0.8 - 5.2	0.0	0.0 - 0.0	13.5	4.4 - 25.9	0.91
3110206	7781	OLUSTEE R	187.6	31.9	11 - 65	0.0	0.0 - 0.0	5.8	1.6 - 12.1	2.1	0.6 - 5.1	2.4	0.8 - 5.8	3.3	0.9 - 9.8	18.3	5.9 - 40.6	0.91
3110206	7782	ITCHETUCKNEE R	672.6	38.9	12 - 77	0.3	0.1 - 0.7	13.2	4.0 - 28.4	2.7	0.8 - 8.2	8.4	2.4 - 19.5	0.0	0.0 - 0.0	14.2	4.4 - 32.2	0.97
3120001	7783	ST MARKS R	16.6	33.3	10 - 85	0.0	0.0 - 0.0	1.7	0.6 - 3.8	0.1	0.0 - 0.2	0.0	0.0 - 0.0	0.0	0.0 - 0.0	31.5	9.2 - 86.3	1.00
3120001	7784	ST MARKS R	229.5	29.7	9 - 58	0.0	0.0 - 0.0	9.9	3.1 - 20.7	0.4	0.1 - 1.4	2.0	0.6 - 5.5	0.0	0.0 - 0.0	17.3	5.2 - 38.9	1.00
3120001	7785	ST MARKS R	16.8	58.0	20 - 136	0.0	0.0 - 0.0	9.6	3.7 - 21.5	2.6	0.7 - 8.2	10.4	3.1 - 30.7	0.0	0.0 - 0.0	35.5	11.9 - 92.9	0.94
3120001	7786	*A	81.8	46.2	16 - 98	0.0	0.0 - 0.0	6.4	2.1 - 15.1	2.6	0.8 - 6.7	8.9	2.9 - 27.6	0.0	0.0 - 0.0	28.3	11.0 - 67.8	0.93
3120001	7787	ST MARKS R	32.4	50.9	20 - 117	0.0	0.0 - 0.0	6.4	2.2 - 12.9	3.1	0.9 - 9.2	12.9	4.9 - 34.1	0.0	0.0 - 0.0	28.5	10.6 - 71.9	0.93
3120001	7788	WARD CR	41.0	32.5	12 - 61	0.0	0.0 - 0.0	5.9	2.1 - 12.3	0.4	0.1 - 1.0	1.9	0.7 - 4.7	0.0	0.0 - 0.0	24.3	8.8 - 47.8	0.85

8-digit HUC	ID	Name	Area	Catchment Yield		Point sources		Developed Land		Manure		Agricultural Land		Phosphate Mines		Soil parent rock		Frac
				kg/km^2	90% CI	kg/km^2	90% CI	kg/km^2	90% CI	kg/km^2	90% CI	kg/km^2	90% CI	kg/km^2	90% CI	kg/km^2	90% CI	
3120001	7789	PINE CR	107.3	32.5	13 - 70	0.0	0.0 - 0.0	5.2	1.9 - 12.0	0.6	0.2 - 2.0	3.4	1.3 - 8.4	0.0	0.0 - 0.0	23.3	8.8 - 56.0	0.85
3120001	7790	*B	128.8	44.8	19 - 110	0.0	0.0 - 0.0	10.6	4.5 - 23.5	0.7	0.3 - 2.3	5.5	2.2 - 14.4	0.0	0.0 - 0.0	28.0	11.3 - 68.8	0.94
3120001	7791	WAKULLA CR	274.3	27.1	9 - 65	0.0	0.0 - 0.0	10.6	3.0 - 24.6	0.4	0.1 - 1.1	3.5	1.1 - 8.9	0.0	0.0 - 0.0	12.6	4.2 - 32.0	1.00
3120001	7792	LOST CR	372.8	25.0	7 - 61	0.0	0.0 - 0.0	6.0	1.6 - 14.7	0.2	0.1 - 0.6	2.0	0.6 - 5.8	0.0	0.0 - 0.0	16.7	4.7 - 39.7	0.00
3120002	7793	OCHLOCKNEE R	226.3	39.1	15 - 81	0.0	0.0 - 0.0	5.5	1.9 - 10.3	2.3	0.7 - 6.0	10.3	3.8 - 26.6	0.0	0.0 - 0.0	20.9	7.0 - 42.0	0.58
3120002	7794	OCHLOCKNEE R	147.4	88.0	28 - 169	45.1	13.5 - 92.3	19.9	6.0 - 47.2	0.9	0.3 - 2.2	6.0	1.7 - 15.1	0.0	0.0 - 0.0	16.1	5.0 - 41.8	0.56
3120002	7795	OCHLOCKNEE R	264.4	45.7	16 - 109	0.0	0.0 - 0.0	6.3	2.3 - 14.8	2.4	0.7 - 7.2	20.2	6.9 - 50.2	0.0	0.0 - 0.0	16.8	5.2 - 41.6	0.55
3120002	7796	OCHLOCKNEE R	32.4	44.1	13 - 103	0.0	0.0 - 0.0	3.9	1.1 - 8.1	3.8	1.0 - 11.0	20.8	5.4 - 56.4	0.0	0.0 - 0.0	15.7	4.8 - 35.8	0.52
3120002	7797	OCHLOCKNEE R	341.5	58.1	21 - 148	19.0	6.9 - 53.1	8.5	2.8 - 20.7	2.6	0.8 - 7.7	16.1	5.6 - 49.8	0.0	0.0 - 0.0	11.8	3.8 - 31.3	0.51
3120002	7798	*A	60.6	38.5	12 - 79	0.0	0.0 - 0.0	4.0	1.3 - 8.5	4.1	1.2 - 9.8	18.7	5.8 - 45.4	0.0	0.0 - 0.0	11.7	4.0 - 25.9	0.51
3120002	7799	BRIDGE CR	189.8	41.1	13 - 93	0.0	0.0 - 0.0	5.1	1.5 - 12.9	4.1	1.2 - 11.3	19.9	5.6 - 56.4	0.0	0.0 - 0.0	12.0	3.8 - 28.8	0.52
3120002	7800	LITTLE OCHLOCKI	85.0	41.5	15 - 97	2.0	0.7 - 4.8	8.9	3.4 - 19.3	1.9	0.5 - 5.4	14.3	4.6 - 34.6	0.0	0.0 - 0.0	14.4	4.7 - 35.9	0.55
3120002	7801	LITTLE OCHLOCKI	8.9	24.8	9 - 50	0.0	0.0 - 0.0	3.3	1.1 - 7.7	1.5	0.4 - 3.8	5.3	1.8 - 14.3	0.0	0.0 - 0.0	14.8	5.0 - 35.2	0.52
3120002	7802	LITTLE OCHLOCKI	139.2	47.1	14 - 101	0.0	0.0 - 0.0	5.1	1.4 - 11.2	6.5	1.9 - 18.1	22.3	7.3 - 55.1	0.0	0.0 - 0.0	13.2	4.4 - 33.4	0.51
3120002	7803	LOST CR	93.5	48.2	17 - 124	0.0	0.0 - 0.0	4.2	1.4 - 11.3	8.7	2.6 - 25.4	21.6	7.8 - 67.4	0.0	0.0 - 0.0	13.7	5.0 - 36.9	0.51
3120002	7804	LITTLE CR	108.1	47.4	16 - 107	0.0	0.0 - 0.0	8.2	2.7 - 18.3	7.4	2.2 - 23.8	18.4	5.8 - 48.9	0.0	0.0 - 0.0	13.4	4.8 - 30.2	0.52
3120002	7805	BARNETTS CR	34.3	40.7	14 - 89	0.0	0.0 - 0.0	4.9	1.6 - 11.2	3.1	0.9 - 10.3	15.3	4.9 - 40.2	0.0	0.0 - 0.0	17.4	5.4 - 42.8	0.56
3120002	7806	BARNETTS CR, E E	101.4	40.2	15 - 72	0.0	0.0 - 0.0	6.2	2.3 - 14.8	3.8	1.2 - 8.8	17.7	6.1 - 41.9	0.0	0.0 - 0.0	12.6	4.7 - 25.0	0.53
3120002	7807	BARNETTS CR	8.5	37.8	12 - 86	0.0	0.0 - 0.0	5.0	1.6 - 11.4	2.4	0.7 - 7.0	13.5	4.6 - 39.0	0.0	0.0 - 0.0	17.0	6.1 - 40.8	0.53
3120002	7808	BARNETTS CR	82.3	47.1	17 - 109	0.0	0.0 - 0.0	5.7	1.9 - 12.6	5.2	1.5 - 14.6	23.0	8.1 - 55.6	0.0	0.0 - 0.0	13.2	4.3 - 30.7	0.51
3120002	7809	BARNETTS CR, W	80.3	44.8	13 - 106	0.0	0.0 - 0.0	5.1	1.5 - 13.2	4.5	1.1 - 11.3	21.5	5.8 - 52.3	0.0	0.0 - 0.0	13.7	4.2 - 36.4	0.51
3120002	7810	TIRED CR	30.4	30.5	12 - 65	0.0	0.0 - 0.0	4.1	1.6 - 7.9	1.7	0.6 - 4.1	6.1	2.2 - 16.7	0.0	0.0 - 0.0	18.5	7.2 - 41.2	0.58
3120002	7811	TIRED CR	2.2	27.8	10 - 66	0.0	0.0 - 0.0	2.5	0.9 - 5.8	1.8	0.5 - 4.4	7.2	2.5 - 19.1	0.0	0.0 - 0.0	16.2	5.8 - 40.8	0.56
3120002	7812	LITTLE TIRED CR	75.0	45.7	16 - 88	0.0	0.0 - 0.0	13.4	4.4 - 26.9	3.3	1.1 - 8.6	14.3	5.1 - 34.7	0.0	0.0 - 0.0	14.6	5.1 - 33.6	0.56
3120002	7813	TIRED CR	191.5	42.7	13 - 88	0.0	0.0 - 0.0	8.7	2.8 - 18.7	3.5	0.9 - 8.1	15.9	5.0 - 45.6	0.0	0.0 - 0.0	14.6	4.3 - 34.6	0.56
3120002	7814	TURKEY CR	83.2	43.1	15 - 111	0.0	0.0 - 0.0	5.0	1.7 - 11.8	3.9	1.2 - 11.7	18.6	6.8 - 62.1	0.0	0.0 - 0.0	15.6	4.7 - 42.6	0.56
3120003	7815	SOPCHOPPY CR	180.9	22.8	7 - 56	0.0	0.0 - 0.0	4.6	1.3 - 10.6	0.2	0.0 - 0.4	1.2	0.4 - 3.2	0.0	0.0 - 0.0	16.8	5.0 - 42.9	1.00
3120003	7816	OCHOCKONEE R	462.7	30.2	11 - 82	0.0	0.0 - 0.0	3.5	1.2 - 8.8	0.0	0.0 - 0.1	0.2	0.1 - 0.7	0.0	0.0 - 0.0	26.4	9.8 - 74.2	1.00
3120003	7817	OCHOCKONEE R	139.6	28.4	11 - 56	0.0	0.0 - 0.0	5.5	1.7 - 11.7	0.4	0.1 - 1.0	2.2	0.8 - 5.5	0.0	0.0 - 0.0	20.4	7.7 - 46.9	0.96
3120003	7818	OCHOCKONEE R	359.4	41.0	15 - 96	0.0	0.0 - 0.0	9.0	3.0 - 19.8	0.7	0.2 - 2.4	3.4	1.2 - 9.4	0.0	0.0 - 0.0	27.9	9.7 - 79.1	0.60
3120003	7819	OCHOCKONEE R	75.5	37.6	13 - 72	0.0	0.0 - 0.0	3.7	1.3 - 6.9	1.8	0.6 - 4.9	7.4	2.3 - 22.0	0.0	0.0 - 0.0	24.7	7.8 - 56.2	0.59
3120003	7820	*A	48.6	41.1	13 - 79	0.0	0.0 - 0.0	4.9	1.5 - 9.3	3.2	1.0 - 8.9	14.9	4.7 - 36.8	0.0	0.0 - 0.0	18.0	5.7 - 42.2	0.59
3120003	7821	*B	38.5	38.0	13 - 70	0.0	0.0 - 0.0	12.0	4.3 - 24.3	1.1	0.4 - 3.3	9.2	2.9 - 25.8	0.0	0.0 - 0.0	15.7	5.8 - 35.4	0.60
3120003	7822	LITTLE CR	50.7	35.9	12 - 80	0.0	0.0 - 0.0	9.0	3.1 - 18.4	0.8	0.2 - 1.9	5.2	1.6 - 13.5	0.0	0.0 - 0.0	21.0	7.6 - 48.1	0.59
3120003	7823	SWAMP CR	148.7	42.1	15 - 86	0.0	0.0 - 0.0	5.4	1.9 - 12.3	2.8	1.0 - 7.9	15.6	4.8 - 39.3	0.0	0.0 - 0.0	18.3	5.6 - 42.9	0.58
3120003	7824	ATTAPULGUS CR	255.1	37.9	11 - 99	1.3	0.4 - 3.3	6.3	1.7 - 14.0	1.8	0.4 - 5.4	12.8	3.2 - 34.1	0.0	0.0 - 0.0	15.7	5.0 - 36.3	0.58
3120003	7825	WILLACOOCHEE C	155.5	37.0	15 - 86	0.0	0.0 - 0.0	5.3	1.8 - 12.8	1.8	0.5 - 4.8	14.2	5.0 - 37.5	0.0	0.0 - 0.0	15.7	6.2 - 39.6	0.59
3120003	7826	*C	110.3	37.8	13 - 101	0.0	0.0 - 0.0	12.3	4.1 - 30.7	1.0	0.3 - 2.8	8.8	3.2 - 23.5	0.0	0.0 - 0.0	15.7	5.1 - 41.9	0.61
3120003	7827	BEAR CR	29.4	23.2	7 - 51	0.0	0.0 - 0.0	5.5	1.7 - 12.2	0.6	0.1 - 1.7	2.7	0.9 - 6.9	0.0	0.0 - 0.0	14.5	4.4 - 37.1	0.61

8-digit HUC	ID	Name	Area	Catchment Yield kg/km^2	Catchment Yield 90% CI	Point sources kg/km^2	Point sources 90% CI	Developed Land kg/km^2	Developed Land 90% CI	Manure kg/km^2	Manure 90% CI	Agricultural Land kg/km^2	Agricultural Land 90% CI	Phosphate Mines kg/km^2	Phosphate Mines 90% CI	Soil parent rock kg/km^2	Soil parent rock 90% CI	Frac
3120003	7828	TELOGIA CR	369.2	34.2	10 - 86	0.0	0.0 - 0.0	6.0	1.7 - 14.1	0.3	0.1 - 1.0	2.1	0.6 - 5.0	0.0	0.0 - 0.0	25.8	7.5 - 67.2	0.96
3130001	7829	CHATTAHOOCHEE	4.6	2249.1	894 - 4528	2126.0	837.8 - 4320.3	94.3	29.6 - 191.3	0.3	0.1 - 0.7	0.4	0.2 - 0.9	0.0	0.0 - 0.0	28.0	11.8 - 58.0	0.17
3130001	7831	PEACHTREE CR, S	22.0	130.4	42 - 353	0.0	0.0 - 0.0	88.6	28.8 - 238.5	0.1	0.0 - 0.4	0.5	0.2 - 1.5	0.0	0.0 - 0.0	41.1	13.7 - 103.5	0.17
3130001	7832	PEACHTREE CR, S	77.4	125.5	48 - 231	0.0	0.0 - 0.0	92.0	32.9 - 178.6	0.0	0.0 - 0.1	0.5	0.1 - 1.1	0.0	0.0 - 0.0	33.0	11.6 - 75.4	0.16
3130001	7833	PEACHTREE CR, N	101.7	133.6	48 - 256	0.0	0.0 - 0.0	99.1	36.2 - 189.3	0.0	0.0 - 0.1	0.3	0.1 - 0.7	0.0	0.0 - 0.0	34.2	11.7 - 84.2	0.16
3130001	7834	NANCY CR	98.5	109.1	40 - 250	0.0	0.0 - 0.0	87.8	32.0 - 197.5	0.1	0.0 - 0.2	0.7	0.2 - 1.9	0.0	0.0 - 0.0	20.5	6.6 - 54.3	0.17
3130001	7835	CHATTAHOOCHEE	22.7	101.9	36 - 238	0.0	0.0 - 0.0	81.2	28.6 - 187.5	0.2	0.1 - 0.6	1.5	0.6 - 4.3	0.0	0.0 - 0.0	19.0	7.9 - 49.2	0.17
3130001	7837	*A	20.2	85.7	26 - 186	0.0	0.0 - 0.0	67.7	20.2 - 146.1	0.1	0.0 - 0.3	0.4	0.1 - 1.2	0.0	0.0 - 0.0	17.5	5.2 - 40.0	0.17
3130001	7838	CHATTAHOOCHEE	219.8	158.5	58 - 369	42.5	15.3 - 102.5	83.4	30.4 - 193.9	0.2	0.1 - 0.7	1.4	0.5 - 3.4	0.0	0.0 - 0.0	31.0	11.0 - 86.1	0.17
3130001	7839	CHATTAHOOCHEE	151.1	447.0	136 - 895	337.6	108.0 - 696.7	89.7	22.9 - 183.9	0.6	0.2 - 1.8	2.0	0.5 - 4.5	0.0	0.0 - 0.0	17.0	5.5 - 38.0	0.17
3130001	7840	SWANNEE CR	14.0	75.8	23 - 137	0.0	0.0 - 0.0	65.0	20.1 - 123.3	0.3	0.1 - 0.8	1.7	0.5 - 3.7	0.0	0.0 - 0.0	8.9	2.7 - 20.0	0.17
3130001	7841	IVY CR	57.1	58.8	22 - 136	0.0	0.0 - 0.0	39.7	13.8 - 94.7	0.9	0.3 - 2.3	5.8	2.1 - 15.5	0.0	0.0 - 0.0	12.3	4.4 - 28.2	0.13
3130001	7842	SWANNEE CR	45.0	76.7	21 - 155	13.0	3.8 - 28.9	51.4	13.0 - 114.5	1.5	0.4 - 4.5	2.2	0.6 - 6.0	0.0	0.0 - 0.0	8.6	2.8 - 17.3	0.13
3130001	7843	CHATTAHOOCHEE	5.0	67.9	22 - 144	0.0	0.0 - 0.0	46.5	14.9 - 110.6	3.3	1.0 - 9.8	9.6	3.0 - 21.6	0.0	0.0 - 0.0	8.5	3.1 - 19.7	0.17
3130001	7844	LEVEL CR	23.3	62.9	24 - 132	0.0	0.0 - 0.0	52.2	19.0 - 116.1	0.4	0.1 - 1.2	3.3	1.2 - 9.6	0.0	0.0 - 0.0	7.0	2.6 - 20.5	0.16
3130001	7845	CHATTAHOOCHEE	101.1	72.1	24 - 150	3.3	1.2 - 7.0	45.9	15.8 - 101.6	4.6	1.2 - 14.9	4.1	1.4 - 10.2	0.0	0.0 - 0.0	14.3	5.1 - 34.0	0.16
3130001	7846	CHATTAHOOCHEE	44.7	105.4	38 - 190	0.0	0.0 - 0.0	15.6	5.0 - 26.1	48.3	15.3 - 128.4	21.1	7.3 - 46.5	0.0	0.0 - 0.0	20.3	7.1 - 39.9	0.04
3130001	7847	SOQUE R	40.3	112.8	39 - 241	0.0	0.0 - 0.0	16.8	5.6 - 39.3	54.0	15.7 - 152.7	17.9	6.0 - 47.1	0.0	0.0 - 0.0	24.0	9.1 - 47.9	0.04
3130001	7848	HAZEL CR	3.1	64.2	20 - 124	0.0	0.0 - 0.0	23.6	7.1 - 52.1	21.7	5.9 - 57.5	5.9	1.9 - 14.9	0.0	0.0 - 0.0	13.1	3.9 - 29.6	0.04
3130001	7849	CAMP CR	21.0	102.6	32 - 235	0.0	0.0 - 0.0	48.0	15.7 - 106.1	32.6	9.1 - 97.3	10.9	3.3 - 25.1	0.0	0.0 - 0.0	11.2	3.7 - 24.8	0.04
3130001	7850	HAZEL CR	58.1	115.6	33 - 249	0.0	0.0 - 0.0	24.4	7.5 - 49.2	60.0	17.0 - 174.1	19.9	6.7 - 49.5	0.0	0.0 - 0.0	11.3	3.5 - 28.2	0.04
3130001	7851	SOQUE R	57.6	127.9	41 - 292	6.9	2.5 - 15.7	23.7	7.2 - 51.4	54.2	14.9 - 147.9	17.9	5.8 - 42.5	0.0	0.0 - 0.0	25.2	8.2 - 66.1	0.04
3130001	7853	GLADE CR	27.0	144.6	45 - 304	0.0	0.0 - 0.0	27.6	8.5 - 56.8	78.7	23.6 - 224.6	26.5	9.0 - 72.7	0.0	0.0 - 0.0	11.8	4.3 - 28.2	0.04
3130001	7854	DEEP CR	50.7	92.2	29 - 164	0.0	0.0 - 0.0	21.1	7.4 - 43.2	37.6	10.9 - 98.3	12.5	3.9 - 32.4	0.0	0.0 - 0.0	21.0	7.2 - 49.8	0.04
3130001	7855	SOQUE R	107.1	65.6	18 - 125	0.0	0.0 - 0.0	8.8	2.6 - 18.9	27.8	7.2 - 84.5	9.7	2.5 - 23.3	0.0	0.0 - 0.0	19.2	5.6 - 47.9	0.04
3130001	7856	SOQUE R, RIGHT F	25.2	60.6	20 - 179	0.0	0.0 - 0.0	11.8	3.9 - 31.0	14.1	4.5 - 57.0	4.5	1.5 - 15.4	0.0	0.0 - 0.0	30.1	9.5 - 92.2	0.03
3130001	7857	SOQUE R	22.5	43.8	14 - 77	0.0	0.0 - 0.0	6.4	2.0 - 12.5	8.3	2.5 - 21.4	2.9	0.9 - 7.1	0.0	0.0 - 0.0	26.3	8.5 - 56.1	0.03
3130001	7858	CHATTAHOOCHEE	27.7	85.2	28 - 183	0.0	0.0 - 0.0	14.3	4.8 - 36.0	33.0	9.5 - 90.4	12.6	4.6 - 36.1	0.0	0.0 - 0.0	25.2	9.0 - 71.5	0.04
3130001	7859	CHATTAHOOCHEE	62.8	79.9	27 - 201	0.0	0.0 - 0.0	15.4	5.5 - 34.4	25.4	7.7 - 77.6	9.0	3.1 - 28.0	0.0	0.0 - 0.0	30.0	11.1 - 76.5	0.04
3130001	7860	SAUTEE CR	92.9	61.3	18 - 125	0.0	0.0 - 0.0	14.4	4.9 - 31.5	16.6	5.0 - 42.9	6.6	2.2 - 16.8	0.0	0.0 - 0.0	23.6	7.2 - 53.8	0.04
3130001	7861	CHATTAHOOCHEE	4.0	129.2	44 - 293	0.0	0.0 - 0.0	13.7	4.6 - 31.4	59.4	17.3 - 179.2	26.6	8.0 - 70.8	0.0	0.0 - 0.0	29.5	9.9 - 71.7	0.04
3130001	7862	CHATTAHOOCHEE	4.0	88.0	32 - 188	0.0	0.0 - 0.0	10.2	3.9 - 19.3	38.8	13.2 - 114.7	16.6	5.4 - 43.0	0.0	0.0 - 0.0	22.3	8.6 - 52.4	0.04
3130001	7863	SMITH CR	27.1	35.3	11 - 66	0.0	0.0 - 0.0	13.8	4.2 - 32.3	1.8	0.5 - 4.4	0.8	0.2 - 2.0	0.0	0.0 - 0.0	19.0	6.2 - 39.2	0.03
3130001	7864	CHATTAHOOCHEE	6.4	59.8	16 - 124	0.0	0.0 - 0.0	32.8	9.3 - 71.1	9.3	2.0 - 24.4	4.1	1.1 - 10.0	0.0	0.0 - 0.0	13.6	3.7 - 30.3	0.03
3130001	7865	CENTER CR	24.1	32.0	11 - 68	0.0	0.0 - 0.0	10.6	3.9 - 20.3	0.4	0.1 - 1.0	0.2	0.1 - 0.5	0.0	0.0 - 0.0	20.8	6.5 - 46.2	0.03
3130001	7866	CHATTAHOOCHEE	57.2	24.0	9 - 57	0.0	0.0 - 0.0	2.9	1.0 - 7.3	0.1	0.0 - 0.2	0.1	0.0 - 0.2	0.0	0.0 - 0.0	20.9	7.1 - 50.0	0.03
3130001	7867	DUKE CR	57.8	30.2	11 - 62	0.0	0.0 - 0.0	8.6	2.5 - 18.2	2.0	0.6 - 5.3	0.8	0.3 - 2.0	0.0	0.0 - 0.0	18.8	6.5 - 43.9	0.04
3130001	7868	BLUE CR	31.5	80.3	22 - 156	0.0	0.0 - 0.0	12.9	3.6 - 26.0	28.3	6.6 - 84.3	11.3	3.5 - 26.4	0.0	0.0 - 0.0	27.8	8.5 - 60.4	0.04
3130001	7869	MOSSY CR	77.6	112.1	38 - 227	0.0	0.0 - 0.0	18.9	6.9 - 35.7	47.0	14.2 - 130.2	20.3	6.0 - 56.9	0.0	0.0 - 0.0	25.9	8.1 - 59.8	0.04

8-digit HUC	ID	Name	Area	Catchment Yield kg/km^2	Catchment Yield 90% CI	Point sources kg/km^2	Point sources 90% CI	Developed Land kg/km^2	Developed Land 90% CI	Manure kg/km^2	Manure 90% CI	Agricultural Land kg/km^2	Agricultural Land 90% CI	Phosphate Mines kg/km^2	Phosphate Mines 90% CI	Soil parent rock kg/km^2	Soil parent rock 90% CI	Frac
3130001	7870	CHESTATEE R	6.0	23.2	8 - 55	0.0	0.0 - 0.0	13.1	4.3 - 31.5	0.3	0.1 - 1.0	0.1	0.0 - 0.3	0.0	0.0 - 0.0	9.7	3.0 - 25.4	0.04
3130001	7871	CHESTATEE R	9.6	35.5	13 - 83	0.0	0.0 - 0.0	9.6	3.3 - 20.1	7.7	2.7 - 21.4	3.7	1.3 - 9.7	0.0	0.0 - 0.0	14.5	5.1 - 36.2	0.04
3130001	7872	TESNATEE CR	45.5	92.3	30 - 195	0.0	0.0 - 0.0	15.8	4.7 - 36.1	36.2	11.0 - 90.3	16.1	4.9 - 45.1	0.0	0.0 - 0.0	24.2	7.4 - 56.4	0.04
3130001	7873	TESNATEE CR	72.3	85.9	27 - 196	6.4	2.2 - 16.7	26.8	8.2 - 60.3	22.9	6.2 - 61.6	10.0	2.7 - 26.9	0.0	0.0 - 0.0	19.8	7.0 - 48.7	0.04
3130001	7874	TOWN CR	67.0	49.6	15 - 96	0.0	0.0 - 0.0	13.9	4.7 - 27.4	13.3	4.1 - 32.9	6.0	1.8 - 15.1	0.0	0.0 - 0.0	16.4	4.6 - 34.2	0.04
3130001	7875	CHESTATEE R	162.7	36.6	12 - 66	0.0	0.0 - 0.0	7.8	2.5 - 16.2	6.7	1.8 - 17.6	3.5	1.2 - 8.1	0.0	0.0 - 0.0	18.6	6.1 - 34.5	0.04
3130001	7876	YAHOOLA CR	89.8	47.8	17 - 83	2.1	0.8 - 3.8	16.1	5.5 - 33.1	9.2	3.1 - 20.1	5.0	1.6 - 13.3	0.0	0.0 - 0.0	15.4	5.8 - 30.5	0.04
3130001	7877	WARD CR	69.1	53.5	19 - 104	0.0	0.0 - 0.0	13.8	4.5 - 33.5	12.7	4.6 - 34.0	7.0	2.2 - 16.9	0.0	0.0 - 0.0	19.9	7.1 - 42.0	0.04
3130001	7878	BIG CR	268.6	98.1	36 - 264	0.4	0.2 - 1.1	61.7	22.6 - 173.2	9.3	3.0 - 25.9	6.9	2.6 - 22.2	0.0	0.0 - 0.0	19.9	6.6 - 49.6	0.17
3130001	7879	*B	51.4	139.9	40 - 252	0.0	0.0 - 0.0	109.4	30.8 - 206.3	0.1	0.0 - 0.3	1.0	0.3 - 2.1	0.0	0.0 - 0.0	29.5	9.4 - 60.0	0.17
3130002	7880	CHATTAHOOCHEE	26.3	69.9	19 - 176	0.0	0.0 - 0.0	53.9	14.3 - 129.2	0.3	0.1 - 0.7	2.4	0.6 - 7.7	0.0	0.0 - 0.0	13.4	3.9 - 36.2	0.35
3130002	7881	STANDING BOY C	184.6	31.1	11 - 50	0.0	0.0 - 0.0	10.5	3.2 - 19.7	0.7	0.2 - 1.9	4.4	1.4 - 11.4	0.0	0.0 - 0.0	15.4	5.0 - 27.7	0.34
3130002	7882	CHATTAHOOCHEE	88.5	27.0	8 - 58	0.0	0.0 - 0.0	6.8	2.0 - 14.9	0.7	0.2 - 1.9	4.7	1.4 - 11.3	0.0	0.0 - 0.0	14.8	4.0 - 32.8	0.34
3130002	7883	MULBERRY CR	590.6	28.5	9 - 55	0.1	0.0 - 0.2	6.4	1.8 - 13.9	0.9	0.2 - 2.2	4.9	1.5 - 10.7	0.0	0.0 - 0.0	16.2	4.8 - 38.5	0.34
3130002	7884	CHATTAHOOCHEE	107.2	24.2	7 - 50	0.0	0.0 - 0.0	4.9	1.5 - 10.1	0.9	0.2 - 2.4	5.7	1.7 - 13.6	0.0	0.0 - 0.0	12.7	3.7 - 31.2	0.34
3130002	7885	MOUNTAIN CR	180.0	20.8	8 - 46	0.0	0.0 - 0.0	6.5	2.1 - 13.3	0.5	0.2 - 1.4	2.4	0.9 - 6.6	0.0	0.0 - 0.0	11.5	4.2 - 29.6	0.30
3130002	7886	CHATTAHOOCHEE	139.8	26.5	10 - 46	0.0	0.0 - 0.0	4.8	1.7 - 9.6	1.1	0.4 - 3.0	6.6	2.2 - 17.8	0.0	0.0 - 0.0	14.0	5.1 - 31.1	0.30
3130002	7887	FLAT SHOAL CR	572.9	27.2	9 - 46	0.0	0.0 - 0.0	4.9	1.5 - 8.8	1.6	0.5 - 4.2	8.3	2.4 - 19.3	0.0	0.0 - 0.0	12.4	4.0 - 25.6	0.30
3130002	7888	CHATTAHOOCHEE	16.3	303.8	88 - 828	222.1	63.5 - 646.6	63.3	18.6 - 169.8	0.7	0.2 - 2.3	5.0	1.5 - 14.7	0.0	0.0 - 0.0	12.6	4.1 - 33.8	0.30
3130002	7889	CHATTAHOOCHEE	38.8	27.4	10 - 65	0.0	0.0 - 0.0	6.4	2.0 - 15.1	1.7	0.5 - 4.9	8.1	2.5 - 19.8	0.0	0.0 - 0.0	11.3	4.2 - 31.2	0.30
3130002	7890	CHATTAHOOCHEE	7.5	46.0	16 - 115	4.5	1.4 - 12.1	17.6	5.9 - 43.0	4.5	1.3 - 13.8	5.3	1.7 - 14.6	0.0	0.0 - 0.0	14.1	4.7 - 35.3	0.18
3130002	7891	CHATTAHOOCHEE	87.6	26.0	10 - 55	0.0	0.0 - 0.0	4.9	1.8 - 10.4	3.9	1.2 - 10.2	4.9	1.8 - 12.9	0.0	0.0 - 0.0	12.3	4.8 - 27.2	0.18
3130002	7892	CEDAR CR	134.7	25.7	10 - 45	0.0	0.0 - 0.0	7.6	2.9 - 17.6	0.8	0.3 - 2.2	5.8	2.0 - 13.9	0.0	0.0 - 0.0	11.4	4.3 - 27.9	0.18
3130002	7894	CHATTAHOOCHEE	169.9	33.4	11 - 57	0.0	0.0 - 0.0	6.9	2.2 - 13.2	3.9	1.2 - 9.7	8.7	2.7 - 17.4	0.0	0.0 - 0.0	13.9	4.8 - 30.4	0.18
3130002	7895	CHATTAHOOCHEE	4.3	21.6	9 - 44	0.0	0.0 - 0.0	0.6	0.2 - 1.1	0.8	0.3 - 2.3	7.8	2.7 - 18.6	0.0	0.0 - 0.0	12.4	5.2 - 28.0	0.18
3130002	7896	BEAR CR	80.4	32.7	13 - 71	4.7	1.8 - 10.8	11.2	4.3 - 23.3	0.8	0.3 - 2.2	4.0	1.3 - 11.5	0.0	0.0 - 0.0	12.0	4.8 - 27.4	0.18
3130002	7897	CHATTAHOOCHEE	111.3	43.3	16 - 103	0.1	0.0 - 0.1	18.4	5.7 - 39.1	1.0	0.3 - 3.0	6.7	2.2 - 16.8	0.0	0.0 - 0.0	17.2	6.1 - 42.6	0.18
3130002	7898	CAMP CR	117.1	95.4	35 - 227	33.8	12.2 - 90.2	42.5	14.1 - 99.2	0.5	0.2 - 1.4	2.1	0.8 - 6.9	0.0	0.0 - 0.0	16.4	5.8 - 41.3	0.17
3130002	7899	CHATTAHOOCHEE	23.4	70.1	21 - 165	26.1	7.9 - 62.0	26.7	6.8 - 66.2	0.6	0.2 - 2.1	3.8	1.0 - 10.5	0.0	0.0 - 0.0	12.9	3.8 - 36.3	0.17
3130002	7900	CHATTAHOOCHEE	20.7	62.4	19 - 122	0.0	0.0 - 0.0	47.3	14.2 - 98.1	0.3	0.1 - 1.0	2.0	0.5 - 5.4	0.0	0.0 - 0.0	12.8	4.3 - 29.3	0.17
3130002	7901	*A	90.1	95.6	31 - 194	0.0	0.0 - 0.0	72.5	24.0 - 147.2	0.2	0.0 - 0.4	0.5	0.2 - 1.1	0.0	0.0 - 0.0	22.5	6.8 - 54.4	0.17
3130002	7902	CHATTAHOOCHEE	136.2	196.4	64 - 389	84.5	26.5 - 178.1	83.8	29.0 - 182.2	0.3	0.1 - 0.8	1.8	0.6 - 5.0	0.0	0.0 - 0.0	26.0	8.9 - 68.0	0.17
3130002	7903	SWEETWATER CR	64.2	70.2	21 - 161	0.0	0.0 - 0.0	42.0	11.4 - 94.1	0.5	0.1 - 1.6	4.4	1.4 - 12.3	0.0	0.0 - 0.0	23.2	7.1 - 62.4	0.17
3130002	7904	NOSES CR	161.5	82.2	33 - 175	0.0	0.0 - 0.0	63.4	25.3 - 129.9	0.6	0.2 - 1.6	4.9	1.9 - 13.0	0.0	0.0 - 0.0	13.4	5.5 - 30.2	0.17
3130002	7905	SWEETWATER CR	408.9	52.3	16 - 122	0.9	0.3 - 2.1	29.4	8.7 - 62.5	3.2	0.9 - 10.1	8.7	2.7 - 26.5	0.0	0.0 - 0.0	10.1	3.1 - 25.8	0.17
3130002	7906	DOG CR	203.8	34.3	13 - 81	0.0	0.0 - 0.0	14.5	4.7 - 30.8	2.9	1.0 - 7.9	6.5	2.2 - 14.9	0.0	0.0 - 0.0	10.5	3.8 - 27.1	0.18
3130002	7907	SNAKE CR	35.2	27.4	8 - 57	0.0	0.0 - 0.0	6.4	2.0 - 12.8	5.9	1.6 - 17.6	4.2	1.4 - 9.7	0.0	0.0 - 0.0	10.9	3.6 - 22.8	0.18
3130002	7908	WHOOPING CR	81.7	40.1	14 - 84	0.0	0.0 - 0.0	9.1	3.0 - 18.9	10.9	3.4 - 25.2	10.7	3.6 - 26.5	0.0	0.0 - 0.0	9.3	3.3 - 21.9	0.18
3130002	7909	HILLABATCHEE C	208.6	24.5	10 - 58	0.0	0.0 - 0.0	4.0	1.5 - 9.3	4.8	1.6 - 11.0	6.0	2.1 - 15.5	0.0	0.0 - 0.0	9.7	4.0 - 26.1	0.18

8-digit HUC	ID	Name	Area	Catchment Yield		Point sources		Developed Land		Manure		Agricultural Land		Phosphate Mines		Soil parent rock		Frac
				kg/km^2	90% CI	kg/km^2	90% CI	kg/km^2	90% CI	kg/km^2	90% CI	kg/km^2	90% CI	kg/km^2	90% CI	kg/km^2	90% CI	
3130002	7910	OSELIGEE CR	226.7	23.6	7 - 40	0.0	0.0 - 0.0	5.6	1.3 - 10.7	1.3	0.3 - 3.5	5.7	1.5 - 13.2	0.0	0.0 - 0.0	11.0	3.4 - 22.9	0.30
3130002	7911	WAHOO CR	90.3	55.3	21 - 137	7.2	2.8 - 17.5	28.6	10.6 - 67.6	0.8	0.3 - 2.5	6.5	2.5 - 18.6	0.0	0.0 - 0.0	12.2	4.9 - 31.1	0.18
3130002	7912	MOUNTAIN CR	159.8	37.0	15 - 95	2.8	1.1 - 6.8	12.9	4.7 - 31.7	1.0	0.3 - 2.9	8.1	2.8 - 24.1	0.0	0.0 - 0.0	12.3	4.6 - 32.7	0.18
3130002	7913	CHATTAHOOCHEE	16.0	33.7	11 - 62	0.0	0.0 - 0.0	7.7	2.3 - 15.5	4.2	1.1 - 11.2	7.4	2.3 - 17.3	0.0	0.0 - 0.0	14.4	4.1 - 31.2	0.18
3130002	7914	NEW R	89.6	28.7	9 - 67	0.0	0.0 - 0.0	6.6	2.0 - 16.2	1.3	0.4 - 3.7	9.0	2.9 - 22.2	0.0	0.0 - 0.0	11.7	3.9 - 29.1	0.18
3130002	7915	LONG CANE CR	216.6	52.9	20 - 96	12.6	4.7 - 24.8	20.7	7.0 - 43.9	1.2	0.4 - 3.4	7.1	2.1 - 15.9	0.0	0.0 - 0.0	11.4	4.1 - 23.6	0.30
3130002	7916	CHATTAHOOCHEE	75.0	96.9	36 - 189	49.2	17.9 - 101.1	28.3	10.6 - 62.8	1.0	0.3 - 2.7	5.3	1.8 - 11.3	0.0	0.0 - 0.0	13.1	4.8 - 29.1	0.30
3130002	7917	ANNEEWAKEE CR	77.7	98.7	30 - 177	15.5	5.3 - 30.3	55.9	17.5 - 109.0	0.6	0.2 - 1.6	5.5	1.9 - 12.8	0.0	0.0 - 0.0	21.1	6.8 - 51.4	0.17
3130003	7919	PATAULA CR	422.0	13.0	5 - 30	0.0	0.0 - 0.0	1.8	0.6 - 4.1	0.2	0.1 - 0.6	4.3	1.3 - 11.5	0.0	0.0 - 0.0	6.7	2.6 - 16.9	0.35
3130003	7920	HODCHODKEE CR	345.9	15.1	5 - 26	1.4	0.5 - 2.4	2.3	0.8 - 4.5	0.2	0.1 - 0.6	3.9	1.3 - 8.0	0.0	0.0 - 0.0	7.2	2.3 - 14.6	0.35
3130003	7921	CHATTAHOOCHEE	15.9	16.8	7 - 42	0.0	0.0 - 0.0	5.0	1.8 - 11.4	0.3	0.1 - 0.8	2.8	0.9 - 6.5	0.0	0.0 - 0.0	8.8	3.4 - 22.3	0.36
3130003	7922	HANNAHATCHEE	376.0	12.3	4 - 23	0.0	0.0 - 0.0	2.1	0.7 - 3.9	0.1	0.0 - 0.3	2.5	0.8 - 5.3	0.0	0.0 - 0.0	7.7	2.6 - 17.2	0.36
3130003	7923	CHATTAHOOCHEE	181.4	17.1	6 - 37	0.0	0.0 - 0.0	4.9	1.7 - 10.5	0.5	0.2 - 1.2	4.5	1.6 - 12.6	0.0	0.0 - 0.0	7.3	2.6 - 16.0	0.36
3130003	7924	HICHITEE CR	144.3	11.1	3 - 28	0.0	0.0 - 0.0	3.6	1.1 - 8.3	0.0	0.0 - 0.0	0.8	0.3 - 2.3	0.0	0.0 - 0.0	6.7	2.0 - 16.9	0.36
3130003	7925	CHATTAHOOCHEE	63.1	21.9	7 - 50	0.0	0.0 - 0.0	3.5	0.9 - 7.9	0.7	0.2 - 2.0	4.8	1.4 - 12.7	0.0	0.0 - 0.0	12.8	3.7 - 32.2	0.36
3130003	7926	OSWICHEE CR	86.6	14.5	5 - 32	0.0	0.0 - 0.0	6.4	2.3 - 13.9	0.0	0.0 - 0.0	0.4	0.1 - 0.9	0.0	0.0 - 0.0	7.7	2.6 - 18.4	0.36
3130003	7927	CHATTAHOOCHEE	2.0	17.4	6 - 41	0.0	0.0 - 0.0	0.5	0.2 - 1.1	0.3	0.1 - 0.7	2.9	0.9 - 7.4	0.0	0.0 - 0.0	13.7	4.7 - 33.4	0.36
3130003	7928	CHATTAHOOCHEE	53.5	762.2	257 - 1699	715.6	241.0 - 1608.7	36.4	11.5 - 76.8	0.2	0.1 - 0.6	1.7	0.5 - 4.0	0.0	0.0 - 0.0	8.4	2.8 - 19.7	0.36
3130003	7929	UPATOI CR	73.0	40.4	15 - 80	0.0	0.0 - 0.0	26.3	9.3 - 55.1	0.2	0.1 - 0.5	1.2	0.4 - 2.9	0.0	0.0 - 0.0	12.7	5.0 - 28.7	0.36
3130003	7930	OCHILEE CR	172.2	12.8	4 - 20	0.0	0.0 - 0.0	5.4	1.9 - 9.7	0.0	0.0 - 0.0	1.2	0.4 - 2.5	0.0	0.0 - 0.0	6.2	2.1 - 11.8	0.35
3130003	7931	UPATOI CR	33.9	13.2	5 - 35	0.0	0.0 - 0.0	5.1	1.9 - 13.7	0.2	0.0 - 0.5	0.5	0.2 - 1.6	0.0	0.0 - 0.0	7.4	2.5 - 19.7	0.35
3130003	7932	UPATOI CR	25.8	14.4	5 - 29	0.0	0.0 - 0.0	5.8	1.8 - 12.0	0.1	0.0 - 0.2	0.9	0.3 - 2.3	0.0	0.0 - 0.0	7.6	2.6 - 18.4	0.35
3130003	7933	PINE KNOT CR	201.5	11.0	3 - 27	0.0	0.0 - 0.0	3.3	1.0 - 8.4	0.5	0.1 - 1.7	1.8	0.5 - 6.0	0.0	0.0 - 0.0	5.3	1.4 - 12.6	0.34
3130003	7934	UPATOI CR	36.7	12.8	4 - 30	0.0	0.0 - 0.0	4.8	1.5 - 10.7	0.2	0.0 - 0.5	0.6	0.2 - 1.8	0.0	0.0 - 0.0	7.2	2.2 - 20.4	0.34
3130003	7935	UPATOI CR	416.9	15.8	6 - 38	0.0	0.0 - 0.0	3.9	1.4 - 8.7	0.6	0.2 - 1.8	3.0	1.0 - 9.1	0.0	0.0 - 0.0	8.4	2.9 - 21.4	0.33
3130003	7936	LOX CR	69.4	18.7	7 - 39	0.0	0.0 - 0.0	3.9	1.3 - 8.4	0.9	0.3 - 2.8	3.6	1.3 - 8.8	0.0	0.0 - 0.0	10.4	3.4 - 23.5	0.33
3130003	7937	RANDALL CR	135.3	18.6	5 - 45	0.0	0.0 - 0.0	6.1	1.7 - 13.8	0.4	0.1 - 1.2	2.2	0.7 - 5.1	0.0	0.0 - 0.0	9.9	2.8 - 23.9	0.35
3130003	7938	CHATTAHOOCHEE	64.9	47.4	18 - 104	0.0	0.0 - 0.0	31.6	11.4 - 74.6	0.6	0.2 - 1.6	5.2	1.9 - 11.7	0.0	0.0 - 0.0	9.9	3.4 - 23.1	0.36
3130003	7939	BULL CR	13.1	97.0	36 - 220	0.0	0.0 - 0.0	88.0	32.8 - 206.8	0.1	0.0 - 0.2	0.4	0.2 - 1.3	0.0	0.0 - 0.0	8.5	3.1 - 21.1	0.35
3130003	7940	BULL CR	142.1	49.1	17 - 120	0.0	0.0 - 0.0	37.9	12.5 - 97.7	0.3	0.1 - 1.0	2.1	0.7 - 6.9	0.0	0.0 - 0.0	8.8	2.9 - 23.3	0.34
3130003	7941	*A	26.3	95.6	35 - 193	0.0	0.0 - 0.0	80.7	28.2 - 161.7	0.2	0.0 - 0.4	1.2	0.4 - 3.0	0.0	0.0 - 0.0	13.5	5.0 - 32.7	0.34
3130003	7942	CHATTAHOOCHEE	95.1	73.8	26 - 139	0.0	0.0 - 0.0	65.0	22.6 - 124.6	0.3	0.1 - 0.8	2.8	1.0 - 6.2	0.0	0.0 - 0.0	5.8	2.0 - 14.8	0.35
3130003	7943	UCHEE CR	82.5	24.9	8 - 60	0.0	0.0 - 0.0	9.4	2.8 - 19.9	0.8	0.2 - 2.4	7.2	2.3 - 17.4	0.0	0.0 - 0.0	7.4	2.2 - 17.8	0.36
3130003	7944	LITTLE UCHEE CR	354.4	25.8	10 - 49	0.0	0.0 - 0.0	11.4	3.7 - 24.3	0.6	0.2 - 1.7	5.6	1.8 - 14.4	0.0	0.0 - 0.0	8.2	3.1 - 17.2	0.35
3130003	7946	BRUSH CR	50.8	16.6	6 - 38	0.0	0.0 - 0.0	5.2	1.9 - 11.4	0.8	0.3 - 2.3	8.2	2.7 - 21.0	0.0	0.0 - 0.0	2.5	0.8 - 5.6	0.31
3130003	7947	UCHEE CR	15.6	13.4	5 - 25	0.0	0.0 - 0.0	2.7	1.0 - 5.0	0.4	0.1 - 0.8	3.6	1.4 - 8.8	0.0	0.0 - 0.0	6.8	2.5 - 14.6	0.31
3130003	7948	SNAKE CR	38.5	17.9	6 - 36	0.0	0.0 - 0.0	4.7	1.5 - 9.8	0.9	0.3 - 2.6	9.7	3.5 - 24.7	0.0	0.0 - 0.0	2.6	0.8 - 6.0	0.30
3130003	7949	UCHEE CR	21.4	13.3	4 - 33	0.0	0.0 - 0.0	2.8	0.8 - 6.8	0.4	0.1 - 1.2	3.5	1.0 - 8.8	0.0	0.0 - 0.0	6.6	1.9 - 16.1	0.30
3130003	7950	WATOLEE CR	64.3	14.6	6 - 34	0.0	0.0 - 0.0	4.6	1.8 - 10.2	0.6	0.2 - 1.7	6.9	2.8 - 18.9	0.0	0.0 - 0.0	2.5	1.0 - 6.5	0.29

8-digit HUC	ID	Name	Area	Catchment Yield		Point sources		Developed Land		Manure		Agricultural Land		Phosphate Mines		Soil parent rock		Frac
				kg/km^2	90% CI	kg/km^2	90% CI	kg/km^2	90% CI	kg/km^2	90% CI	kg/km^2	90% CI	kg/km^2	90% CI	kg/km^2	90% CI	
3130003	7951	UCHEE CR	48.4	16.2	6 - 35	0.0	0.0 - 0.0	3.3	1.1 - 6.2	0.7	0.2 - 1.7	7.5	2.6 - 23.8	0.0	0.0 - 0.0	4.8	2.1 - 10.1	0.29
3130003	7952	HATCHECHUBEE C	378.0	19.2	6 - 33	0.0	0.0 - 0.0	4.6	1.3 - 8.9	0.6	0.2 - 1.4	4.9	1.5 - 10.7	0.0	0.0 - 0.0	9.2	2.8 - 19.3	0.36
3130003	7953	COWIKEE CR, N FK	9.7	29.7	10 - 50	0.0	0.0 - 0.0	5.3	1.9 - 9.0	4.4	1.4 - 9.7	11.6	3.4 - 22.3	0.0	0.0 - 0.0	8.4	2.7 - 17.0	0.36
3130003	7954	COWIKEE CR, N FK	123.7	22.9	8 - 47	0.0	0.0 - 0.0	3.4	1.2 - 7.2	1.3	0.4 - 4.0	7.6	2.7 - 17.9	0.0	0.0 - 0.0	10.7	3.5 - 25.9	0.36
3130003	7955	COWIKEE CR, N FK	144.5	24.9	8 - 56	0.0	0.0 - 0.0	4.1	1.5 - 9.6	0.9	0.3 - 2.7	8.3	2.8 - 23.2	0.0	0.0 - 0.0	11.5	4.0 - 29.3	0.33
3130003	7956	HURTS BORO CR	70.7	28.1	12 - 70	0.0	0.0 - 0.0	5.2	2.1 - 12.6	0.8	0.3 - 2.8	7.3	2.8 - 22.8	0.0	0.0 - 0.0	14.7	6.1 - 41.6	0.33
3130003	7957	COWIKEE CR, M F	457.0	19.8	7 - 46	0.0	0.0 - 0.0	2.9	1.0 - 6.2	1.1	0.4 - 3.3	5.4	2.2 - 13.4	0.0	0.0 - 0.0	10.3	3.6 - 28.1	0.36
3130003	7958	COWIKEE CR, S FK	71.3	11.1	4 - 21	0.0	0.0 - 0.0	2.0	0.7 - 3.9	0.8	0.2 - 2.0	2.0	0.7 - 4.5	0.0	0.0 - 0.0	6.4	2.3 - 13.3	0.36
3130004	7959	CHATTAHOOCHEE	134.5	47.1	17 - 124	0.0	0.0 - 0.0	5.4	2.1 - 13.4	2.3	0.7 - 9.1	26.5	8.6 - 78.1	0.0	0.0 - 0.0	12.8	4.3 - 32.2	0.63
3130004	7960	SAWHATCHEE CR	211.1	37.7	15 - 71	0.0	0.0 - 0.0	4.8	2.0 - 9.7	1.6	0.5 - 4.3	18.5	6.5 - 45.0	0.0	0.0 - 0.0	12.9	5.0 - 32.2	0.63
3130004	7961	CHATTAHOOCHEE	72.2	375.4	135 - 941	336.3	119.6 - 838.7	9.4	3.3 - 25.4	2.0	0.6 - 6.5	15.3	5.6 - 44.0	0.0	0.0 - 0.0	12.4	4.9 - 37.1	0.63
3130004	7962	CHATTAHOOCHEE	58.6	30.6	8 - 72	0.0	0.0 - 0.0	3.1	0.8 - 6.4	1.7	0.4 - 4.4	14.1	3.4 - 37.7	0.0	0.0 - 0.0	11.7	3.5 - 28.1	0.62
3130004	7963	CHATTAHOOCHEE	17.9	27.1	9 - 66	0.0	0.0 - 0.0	8.5	3.0 - 19.1	0.7	0.2 - 2.0	6.0	2.3 - 19.6	0.0	0.0 - 0.0	11.9	4.4 - 29.9	0.61
3130004	7964	*A	52.2	30.2	11 - 69	0.0	0.0 - 0.0	3.1	1.0 - 8.0	1.3	0.4 - 4.3	15.9	5.4 - 45.2	0.0	0.0 - 0.0	9.8	3.5 - 24.8	0.61
3130004	7965	CHATTAHOOCHEE	35.7	41.3	13 - 111	0.0	0.0 - 0.0	5.0	1.5 - 11.9	3.8	1.1 - 11.1	22.0	6.7 - 68.1	0.0	0.0 - 0.0	10.6	3.3 - 29.9	0.61
3130004	7966	CHATTAHOOCHEE	34.1	33.8	14 - 85	0.0	0.0 - 0.0	3.3	1.2 - 8.6	2.7	1.0 - 8.6	16.7	6.1 - 53.4	0.0	0.0 - 0.0	11.1	4.5 - 28.9	0.61
3130004	7967	CHATTAHOOCHEE	132.6	26.9	8 - 60	0.0	0.0 - 0.0	3.0	0.9 - 6.6	2.1	0.6 - 5.6	13.3	4.2 - 37.7	0.0	0.0 - 0.0	8.5	3.0 - 22.1	0.61
3130004	7968	KOLOMOKI CR	11.7	16.2	6 - 38	0.0	0.0 - 0.0	3.2	1.2 - 7.8	0.5	0.2 - 1.4	7.5	2.7 - 19.7	0.0	0.0 - 0.0	5.1	2.1 - 13.0	0.61
3130004	7969	KOLOMOKI CR	34.8	22.0	6 - 53	0.0	0.0 - 0.0	2.6	0.9 - 5.9	1.0	0.3 - 2.5	11.4	3.1 - 28.4	0.0	0.0 - 0.0	7.0	2.5 - 18.6	0.59
3130004	7970	KOLOMOKI CR	63.0	35.8	15 - 76	0.0	0.0 - 0.0	4.4	1.8 - 10.6	1.7	0.6 - 4.9	20.5	8.3 - 48.7	0.0	0.0 - 0.0	9.2	3.7 - 20.7	0.55
3130004	7971	KOLOMOKI CR N F	71.6	23.8	7 - 41	0.0	0.0 - 0.0	3.8	1.2 - 7.3	0.9	0.3 - 2.2	11.5	3.4 - 26.0	0.0	0.0 - 0.0	7.6	2.5 - 15.8	0.55
3130004	7972	FLAT CR	77.1	21.1	6 - 43	0.0	0.0 - 0.0	2.5	0.8 - 4.5	0.8	0.2 - 2.0	11.2	3.0 - 29.5	0.0	0.0 - 0.0	6.5	1.9 - 15.0	0.59
3130004	7973	CHATTAHOOCHEE	97.7	23.8	9 - 58	0.0	0.0 - 0.0	4.5	1.6 - 10.4	1.4	0.5 - 3.6	10.5	3.8 - 26.7	0.0	0.0 - 0.0	7.4	2.6 - 18.3	0.61
3130004	7974	CEMOCHECHOBEE	72.3	14.7	6 - 37	0.0	0.0 - 0.0	3.9	1.3 - 10.9	0.4	0.1 - 1.3	3.5	1.2 - 9.4	0.0	0.0 - 0.0	6.8	2.5 - 17.7	0.61
3130004	7975	SANDY CR	43.2	32.1	10 - 66	0.0	0.0 - 0.0	5.8	2.0 - 12.4	2.9	0.8 - 7.3	14.2	4.1 - 35.2	0.0	0.0 - 0.0	9.2	3.3 - 19.8	0.61
3130004	7976	PETERMAN CR	83.7	23.8	7 - 45	0.0	0.0 - 0.0	4.9	1.4 - 9.8	1.8	0.4 - 4.5	8.8	2.5 - 21.8	0.0	0.0 - 0.0	8.3	2.4 - 19.2	0.59
3130004	7977	SANDY CR	43.5	33.5	12 - 68	0.0	0.0 - 0.0	6.8	2.2 - 15.1	3.3	1.0 - 7.8	16.5	5.5 - 43.6	0.0	0.0 - 0.0	6.9	2.6 - 15.7	0.59
3130004	7978	ABBIE CR	229.3	20.1	6 - 34	0.0	0.0 - 0.0	7.6	2.2 - 15.0	1.1	0.3 - 2.8	5.4	1.5 - 11.4	0.0	0.0 - 0.0	6.1	2.0 - 12.5	0.58
3130004	7979	SANDY CR	13.5	33.2	9 - 82	0.0	0.0 - 0.0	4.9	1.3 - 12.0	3.1	0.7 - 9.5	15.8	4.0 - 46.4	0.0	0.0 - 0.0	9.4	2.4 - 23.2	0.58
3130004	7980	*B	35.3	37.5	12 - 75	0.0	0.0 - 0.0	9.5	3.2 - 20.9	3.2	1.0 - 8.6	15.8	5.0 - 46.5	0.0	0.0 - 0.0	9.0	3.0 - 21.5	0.56
3130004	7981	SANDY CR	64.1	47.1	14 - 111	0.0	0.0 - 0.0	9.4	2.7 - 21.3	4.5	1.3 - 12.8	22.9	7.5 - 59.8	0.0	0.0 - 0.0	10.3	2.9 - 24.7	0.56
3130004	7982	*C	45.8	39.4	14 - 86	0.0	0.0 - 0.0	6.3	2.1 - 13.7	4.1	1.3 - 12.2	20.3	7.1 - 49.9	0.0	0.0 - 0.0	8.8	2.9 - 19.7	0.61
3130004	7983	OMUSEE CR	23.7	39.6	12 - 78	0.0	0.0 - 0.0	9.7	2.8 - 20.1	2.8	0.8 - 6.6	16.4	4.9 - 40.1	0.0	0.0 - 0.0	10.7	3.3 - 24.9	0.61
3130004	7984	OMUSEE CR	86.3	37.3	13 - 73	0.0	0.0 - 0.0	6.0	2.1 - 12.4	3.6	1.1 - 9.1	18.0	5.5 - 46.6	0.0	0.0 - 0.0	9.6	3.3 - 21.0	0.61
3130004	7985	*D	48.1	50.3	16 - 101	0.0	0.0 - 0.0	6.9	2.2 - 14.8	5.6	1.6 - 14.1	27.5	9.0 - 72.4	0.0	0.0 - 0.0	10.3	3.6 - 23.5	0.59
3130004	7986	OMUSEE CR	219.9	75.4	24 - 168	17.6	5.9 - 43.6	20.3	6.6 - 40.9	3.6	1.2 - 11.3	23.1	8.8 - 60.7	0.0	0.0 - 0.0	10.8	3.3 - 28.5	0.59
3130004	7987	*E	80.4	45.9	13 - 84	0.0	0.0 - 0.0	7.9	2.3 - 15.3	3.2	0.8 - 7.7	23.9	7.7 - 53.3	0.0	0.0 - 0.0	10.9	2.7 - 24.3	0.61
3130004	7988	CEDAR CR	79.8	45.9	13 - 90	0.0	0.0 - 0.0	7.7	2.3 - 14.6	3.1	0.8 - 7.7	23.8	6.1 - 56.9	0.0	0.0 - 0.0	11.3	3.3 - 25.3	0.62
3130004	7989	*F	51.1	29.5	10 - 73	0.0	0.0 - 0.0	3.9	1.5 - 9.5	1.8	0.5 - 5.8	13.5	4.1 - 39.0	0.0	0.0 - 0.0	10.4	3.8 - 25.2	0.63

8-digit HUC	ID	Name	Area	Catchment Yield		Point sources		Developed Land		Manure		Agricultural Land		Phosphate Mines		Soil parent rock		Frac
				kg/km^2	90% CI	kg/km^2	90% CI	kg/km^2	90% CI	kg/km^2	90% CI	kg/km^2	90% CI	kg/km^2	90% CI	kg/km^2	90% CI	
3130004	7990	CEMOCHECHOBE	171.6	12.9	5 - 37	0.0	0.0 - 0.0	2.3	0.7 - 7.5	0.3	0.1 - 1.0	4.3	1.4 - 16.0	0.0	0.0 - 0.0	6.1	2.5 - 18.3	0.57
3130005	7991	FLINT R	118.0	23.3	7 - 43	0.0	0.0 - 0.0	2.9	0.9 - 5.7	4.8	1.1 - 12.0	9.6	3.1 - 22.0	0.0	0.0 - 0.0	6.0	1.8 - 13.7	0.48
3130005	7992	FLINT R	301.4	19.8	6 - 48	0.0	0.0 - 0.0	4.3	1.3 - 9.7	1.3	0.4 - 4.3	4.9	1.5 - 13.4	0.0	0.0 - 0.0	9.4	2.8 - 24.5	0.47
3130005	7993	ULCOHATCHEE C	2.2	15.6	6 - 40	0.0	0.0 - 0.0	1.8	0.6 - 4.6	0.2	0.1 - 0.7	0.4	0.1 - 1.2	0.0	0.0 - 0.0	13.1	4.6 - 36.2	0.46
3130005	7994	ULCOHATCHEE C	146.3	19.8	7 - 37	0.0	0.0 - 0.0	3.4	1.1 - 5.4	0.8	0.2 - 2.4	2.5	0.9 - 6.5	0.0	0.0 - 0.0	13.2	4.8 - 29.0	0.46
3130005	7995	AUCHUMPKEE CR	140.1	22.0	7 - 46	0.0	0.0 - 0.0	2.9	0.9 - 7.1	2.8	0.7 - 8.6	4.4	1.2 - 12.2	0.0	0.0 - 0.0	11.9	3.6 - 27.2	0.46
3130005	7996	FLINT R	20.7	21.3	7 - 45	0.0	0.0 - 0.0	3.3	1.0 - 7.5	1.3	0.3 - 3.2	3.5	1.0 - 9.0	0.0	0.0 - 0.0	13.3	4.2 - 33.1	0.46
3130005	7997	SWIFT CR	27.4	19.4	7 - 44	0.0	0.0 - 0.0	3.4	1.1 - 7.1	1.5	0.4 - 4.8	1.6	0.6 - 4.2	0.0	0.0 - 0.0	13.0	4.6 - 31.7	0.46
3130005	7998	TOBLER CR	163.6	33.8	13 - 86	0.0	0.0 - 0.0	4.5	1.7 - 10.4	3.7	1.2 - 11.5	7.8	2.8 - 21.4	0.0	0.0 - 0.0	17.9	6.5 - 45.8	0.45
3130005	7999	SWIFT CR	94.8	39.1	15 - 80	0.0	0.0 - 0.0	8.7	3.2 - 20.9	3.8	1.1 - 12.2	7.8	2.7 - 20.4	0.0	0.0 - 0.0	18.7	6.4 - 47.1	0.45
3130005	8000	FLINT R	192.1	19.0	7 - 47	0.0	0.0 - 0.0	2.6	0.8 - 6.2	0.7	0.2 - 2.0	2.3	0.8 - 6.6	0.0	0.0 - 0.0	13.4	4.9 - 33.6	0.46
3130005	8001	POTATOE CR	554.6	43.8	13 - 102	4.3	1.3 - 11.2	10.1	3.1 - 25.1	3.7	1.1 - 8.9	9.8	2.8 - 26.3	0.0	0.0 - 0.0	15.9	5.4 - 40.1	0.45
3130005	8002	FLINT R	37.2	15.6	5 - 36	0.0	0.0 - 0.0	2.2	0.6 - 5.0	0.1	0.0 - 0.3	0.3	0.1 - 0.8	0.0	0.0 - 0.0	13.0	4.0 - 31.9	0.45
3130005	8003	FLINT R	117.5	24.2	8 - 59	0.0	0.0 - 0.0	3.5	1.0 - 9.2	1.1	0.3 - 2.9	3.1	1.0 - 8.8	0.0	0.0 - 0.0	16.5	5.7 - 45.5	0.45
3130005	8004	FLINT R	208.7	28.7	11 - 72	0.0	0.0 - 0.0	5.7	2.1 - 13.7	2.0	0.7 - 7.7	8.6	3.1 - 24.1	0.0	0.0 - 0.0	12.4	4.9 - 32.4	0.44
3130005	8005	ELKINS CR	264.7	33.0	12 - 64	1.3	0.5 - 2.7	6.4	2.5 - 11.9	2.8	0.8 - 6.8	10.3	4.0 - 26.3	0.0	0.0 - 0.0	12.2	4.2 - 26.9	0.44
3130005	8006	FLINT R	6.6	46.3	18 - 123	0.0	0.0 - 0.0	5.4	2.1 - 13.6	4.2	1.5 - 13.0	20.4	7.0 - 64.1	0.0	0.0 - 0.0	16.3	5.9 - 42.0	0.44
3130005	8007	FLINT R	140.2	37.6	15 - 90	0.0	0.0 - 0.1	5.1	1.9 - 13.0	3.7	1.2 - 12.1	15.2	5.6 - 39.5	0.0	0.0 - 0.0	13.7	5.8 - 37.6	0.44
3130005	8008	FLINT R	36.8	32.9	12 - 58	0.0	0.0 - 0.0	4.8	1.6 - 9.0	3.1	1.0 - 7.9	13.0	4.3 - 29.8	0.0	0.0 - 0.0	12.0	4.5 - 28.4	0.43
3130005	8009	FLINT R	99.0	37.1	13 - 70	0.0	0.0 - 0.0	6.8	2.2 - 13.1	2.7	0.8 - 6.8	15.3	4.8 - 32.9	0.0	0.0 - 0.0	12.3	3.9 - 29.6	0.43
3130005	8010	LINE CR	95.9	34.7	11 - 70	0.0	0.0 - 0.0	7.0	2.3 - 13.6	1.6	0.5 - 4.3	12.1	3.8 - 32.0	0.0	0.0 - 0.0	14.0	4.3 - 31.0	0.43
3130005	8011	WHITEWATER CR	230.1	58.7	18 - 103	13.5	4.5 - 27.1	23.3	6.5 - 50.9	1.2	0.3 - 3.4	7.8	2.3 - 17.4	0.0	0.0 - 0.0	13.0	4.1 - 26.6	0.41
3130005	8012	LINE CR	312.0	62.5	22 - 138	14.1	5.5 - 32.4	27.6	9.4 - 58.5	0.9	0.3 - 2.9	6.8	2.3 - 17.5	0.0	0.0 - 0.0	13.2	4.5 - 37.2	0.41
3130005	8013	WHITE OAK CR	396.6	32.4	10 - 56	0.0	0.0 - 0.0	6.8	2.1 - 12.0	1.8	0.6 - 4.2	10.6	3.3 - 22.4	0.0	0.0 - 0.0	13.2	4.0 - 25.8	0.43
3130005	8014	RED OAK CR	375.4	26.8	10 - 55	0.7	0.2 - 1.9	4.7	1.6 - 10.3	1.9	0.6 - 5.4	6.6	2.2 - 16.6	0.0	0.0 - 0.0	12.9	4.4 - 36.9	0.44
3130005	8015	PIGEON CR	83.6	30.5	12 - 58	0.0	0.0 - 0.0	11.7	4.3 - 24.5	1.5	0.4 - 3.5	5.5	1.9 - 12.9	0.0	0.0 - 0.0	11.8	4.1 - 24.4	0.44
3130005	8016	LAZER CR	438.3	21.4	8 - 41	0.3	0.1 - 0.7	4.5	1.6 - 10.0	0.5	0.2 - 1.3	3.6	1.2 - 8.4	0.0	0.0 - 0.0	12.4	4.4 - 28.1	0.45
3130005	8017	PATSILIGA CR	343.7	16.2	5 - 31	0.0	0.0 - 0.0	3.5	1.2 - 6.8	1.3	0.4 - 3.4	3.8	1.3 - 9.3	0.0	0.0 - 0.0	7.6	2.6 - 16.6	0.47
3130005	8018	WHITEWATER CR	106.9	15.8	6 - 32	0.0	0.0 - 0.0	3.2	1.0 - 6.6	2.9	1.0 - 8.0	6.3	2.3 - 17.5	0.0	0.0 - 0.0	3.4	1.4 - 7.5	0.48
3130005	8019	WHITEWATER CR	282.4	13.7	5 - 25	0.0	0.0 - 0.0	3.1	1.1 - 6.3	1.5	0.4 - 3.9	4.4	1.7 - 10.3	0.0	0.0 - 0.0	4.6	1.7 - 9.8	0.46
3130005	8020	CEDAR CR	208.7	10.6	3 - 24	0.0	0.0 - 0.0	3.6	1.1 - 8.1	1.0	0.3 - 2.9	2.9	0.8 - 7.5	0.0	0.0 - 0.0	3.1	1.0 - 7.0	0.46
3130005	8021	TURKEY CR	17.1	51.9	19 - 109	0.0	0.0 - 0.0	31.6	10.9 - 66.0	0.7	0.2 - 1.7	6.6	2.2 - 19.2	0.0	0.0 - 0.0	13.0	4.8 - 28.0	0.39
3130005	8022	WHITE OAK CR	50.4	78.2	28 - 139	19.3	7.1 - 40.3	41.8	14.3 - 80.7	0.5	0.1 - 1.2	3.3	1.2 - 7.2	0.0	0.0 - 0.0	13.5	4.7 - 30.0	0.39
3130005	8023	MORNING CR	106.0	111.1	40 - 201	43.3	14.9 - 96.7	43.9	15.6 - 88.2	0.8	0.2 - 2.2	5.0	1.8 - 11.4	0.0	0.0 - 0.0	18.0	6.2 - 40.8	0.40
3130005	8024	FLINT R	188.0	101.2	36 - 217	0.0	0.0 - 0.0	74.9	26.3 - 165.0	0.3	0.1 - 0.7	2.4	0.9 - 6.3	0.0	0.0 - 0.0	23.7	8.5 - 52.6	0.40
3130005	8025	TURKEY CR	25.3	161.7	62 - 356	101.7	37.4 - 241.4	35.8	13.1 - 80.0	2.3	0.8 - 5.5	4.8	1.6 - 12.3	0.0	0.0 - 0.0	17.2	6.6 - 36.5	0.45
3130005	8026	POTATOE CR	35.7	15.2	5 - 38	0.0	0.0 - 0.0	2.9	1.0 - 6.9	0.9	0.3 - 2.6	0.5	0.2 - 1.4	0.0	0.0 - 0.0	11.0	3.3 - 31.3	0.45
3130005	8027	HORSE CR	90.2	20.3	5 - 51	0.0	0.0 - 0.0	6.6	1.8 - 15.9	2.6	0.7 - 7.7	6.8	1.8 - 21.4	0.0	0.0 - 0.0	4.2	1.2 - 11.5	0.47
3130005	8028	FLINT R	262.7	23.5	7 - 57	0.0	0.0 - 0.0	2.9	0.9 - 7.0	3.2	0.8 - 9.4	8.5	2.6 - 20.2	0.0	0.0 - 0.0	9.0	2.5 - 24.3	0.47

8-digit HUC	ID	Name	Area	Catchment Yield		Point sources		Developed Land		Manure		Agricultural Land		Phosphate Mines		Soil parent rock		Frac
				kg/km^2	90% CI	kg/km^2	90% CI	kg/km^2	90% CI	kg/km^2	90% CI	kg/km^2	90% CI	kg/km^2	90% CI	kg/km^2	90% CI	
3130006	8029	MILL CR	133.1	28.4	10 - 68	0.0	0.0 - 0.0	5.1	1.8 - 13.1	1.1	0.4 - 3.5	12.9	4.3 - 35.4	0.0	0.0 - 0.0	9.3	3.6 - 27.5	0.60
3130006	8030	FLINT R	15.9	18.5	6 - 35	0.0	0.0 - 0.0	3.1	1.1 - 7.1	0.9	0.3 - 2.9	6.6	2.3 - 16.7	0.0	0.0 - 0.0	7.9	2.5 - 18.5	0.60
3130006	8031	ABRAMS CR	217.0	28.2	10 - 52	0.0	0.0 - 0.0	4.7	1.6 - 9.7	1.2	0.4 - 3.1	11.6	4.3 - 28.4	0.0	0.0 - 0.0	10.7	4.3 - 21.6	0.60
3130006	8032	FLINT R	207.6	26.7	7 - 68	0.0	0.0 - 0.0	4.7	1.3 - 10.5	1.0	0.3 - 2.8	11.2	3.0 - 32.7	0.0	0.0 - 0.0	9.8	2.8 - 24.1	0.60
3130006	8033	FLINT R	5.5	36.6	13 - 81	0.0	0.0 - 0.0	2.4	0.8 - 5.4	1.7	0.5 - 4.1	21.3	7.5 - 53.4	0.0	0.0 - 0.0	11.1	3.3 - 25.0	0.59
3130006	8034	TURKEY CR	13.6	21.1	7 - 36	0.0	0.0 - 0.0	4.8	1.4 - 8.3	1.1	0.3 - 2.5	5.4	1.8 - 13.6	0.0	0.0 - 0.0	9.8	3.8 - 21.6	0.49
3130006	8035	PEENAHATCHEE C	16.6	33.6	12 - 78	0.0	0.0 - 0.0	4.0	1.3 - 8.7	2.6	1.0 - 7.0	18.2	6.9 - 44.4	0.0	0.0 - 0.0	8.8	2.8 - 19.8	0.47
3130006	8036	PEENAHATCHEE C	14.4	35.4	12 - 63	0.0	0.0 - 0.0	4.4	1.4 - 8.9	2.7	0.9 - 7.8	19.1	6.3 - 42.5	0.0	0.0 - 0.0	9.2	3.4 - 22.3	0.45
3130006	8037	PEENAHATCHEE C	91.1	39.8	12 - 72	0.0	0.0 - 0.0	8.5	2.6 - 15.8	3.0	1.0 - 8.6	20.6	6.0 - 43.9	0.0	0.0 - 0.0	7.6	2.5 - 19.7	0.42
3130006	8038	SANDY MOUNT CI	54.9	35.6	12 - 86	0.0	0.0 - 0.0	5.6	1.9 - 13.0	3.0	0.9 - 8.5	20.8	7.4 - 53.4	0.0	0.0 - 0.0	6.3	2.2 - 14.0	0.42
3130006	8039	LITTLE PENNAHA'	77.8	33.0	10 - 57	0.0	0.0 - 0.0	4.3	1.3 - 8.0	2.7	0.7 - 7.8	18.9	5.4 - 41.0	0.0	0.0 - 0.0	7.1	2.2 - 13.6	0.45
3130006	8040	TURKEY CR	209.8	32.4	11 - 84	0.0	0.0 - 0.0	3.9	1.3 - 10.2	2.7	0.8 - 8.0	18.6	6.8 - 52.1	0.0	0.0 - 0.0	7.2	2.6 - 18.9	0.47
3130006	8042	HOGCRAWL CR	249.3	33.4	12 - 57	0.0	0.0 - 0.0	2.5	0.9 - 5.3	6.6	2.0 - 16.4	15.9	5.3 - 38.4	0.0	0.0 - 0.0	8.4	2.9 - 16.7	0.48
3130006	8043	FLINT R	3.8	14.9	4 - 33	0.0	0.0 - 0.0	0.4	0.1 - 1.0	0.2	0.1 - 0.7	0.4	0.1 - 0.9	0.0	0.0 - 0.0	13.9	4.1 - 32.9	0.48
3130006	8044	FLINT R	5.3	24.9	7 - 44	0.0	0.0 - 0.0	2.0	0.5 - 3.8	3.7	0.8 - 9.3	7.2	1.8 - 15.7	0.0	0.0 - 0.0	12.0	3.0 - 26.2	0.48
3130006	8045	FLINT R	110.9	41.8	16 - 84	8.8	3.3 - 19.8	6.0	2.1 - 12.8	6.3	2.1 - 15.5	12.5	4.5 - 28.9	0.0	0.0 - 0.0	8.2	2.9 - 20.5	0.48
3130006	8046	BEAVER CR	117.2	28.4	10 - 75	0.0	0.0 - 0.0	4.7	1.7 - 13.5	5.8	1.8 - 19.4	11.2	3.8 - 33.1	0.0	0.0 - 0.0	6.7	2.5 - 20.2	0.48
3130006	8048	FLINT R	13.7	30.8	12 - 64	0.0	0.0 - 0.0	6.7	2.4 - 14.1	2.8	0.9 - 8.7	4.7	1.6 - 10.9	0.0	0.0 - 0.0	16.7	6.3 - 38.9	0.48
3130006	8049	BUCK CR	30.5	20.1	7 - 34	0.0	0.0 - 0.0	4.1	1.2 - 7.7	3.3	0.9 - 7.5	6.5	2.3 - 13.6	0.0	0.0 - 0.0	6.3	2.1 - 12.8	0.48
3130006	8050	CAMP CR	42.4	9.0	2 - 16	0.0	0.0 - 0.0	3.0	0.7 - 6.2	1.1	0.3 - 3.1	2.0	0.5 - 5.2	0.0	0.0 - 0.0	2.8	0.8 - 5.5	0.46
3130006	8051	BUCK CR	217.6	12.6	4 - 28	0.0	0.0 - 0.0	2.9	1.0 - 6.5	1.8	0.5 - 5.1	3.9	1.4 - 11.9	0.0	0.0 - 0.0	4.1	1.4 - 10.9	0.46
3130006	8052	BUCK CR	45.7	12.7	4 - 22	0.0	0.0 - 0.0	3.2	1.0 - 5.6	1.4	0.4 - 4.1	4.4	1.4 - 11.0	0.0	0.0 - 0.0	3.7	1.1 - 7.3	0.38
3130006	8053	SHOAL CR	123.8	16.4	6 - 30	0.0	0.0 - 0.0	3.6	1.4 - 7.2	1.7	0.6 - 4.4	6.4	2.2 - 15.8	0.0	0.0 - 0.0	4.6	1.7 - 9.7	0.34
3130006	8054	HARDAGE FORD C	47.6	14.5	5 - 34	0.0	0.0 - 0.0	2.3	0.8 - 5.5	1.5	0.5 - 3.7	5.6	2.1 - 13.8	0.0	0.0 - 0.0	5.2	1.9 - 12.6	0.34
3130006	8055	OOCHEE CR	93.9	10.0	4 - 20	0.0	0.0 - 0.0	2.4	0.9 - 5.3	0.9	0.4 - 2.5	2.9	1.1 - 6.6	0.0	0.0 - 0.0	3.8	1.5 - 7.8	0.38
3130006	8056	CAMP CR	153.0	13.6	5 - 37	0.0	0.0 - 0.0	2.4	0.8 - 5.7	2.0	0.6 - 5.7	4.2	1.5 - 13.6	0.0	0.0 - 0.0	4.9	1.6 - 14.2	0.48
3130006	8057	SWEETWATER CR	109.4	18.4	7 - 33	0.0	0.0 - 0.0	4.2	1.5 - 8.2	1.5	0.4 - 3.5	6.2	2.4 - 13.4	0.0	0.0 - 0.0	6.5	2.5 - 14.6	0.48
3130006	8058	CHOKEE CR	180.2	28.9	11 - 69	0.0	0.0 - 0.0	4.3	1.6 - 8.7	1.8	0.5 - 5.7	14.4	5.5 - 43.0	0.0	0.0 - 0.0	8.4	2.9 - 20.5	0.59
3130007	8059	MUCKALEE CR	138.8	37.1	13 - 81	0.0	0.0 - 0.0	4.6	1.4 - 9.1	2.0	0.6 - 5.5	18.8	6.6 - 49.4	0.0	0.0 - 0.0	11.7	3.8 - 27.5	0.58
3130007	8060	PHILLEMA CR	89.9	37.9	17 - 106	0.0	0.0 - 0.0	5.6	2.3 - 14.0	3.0	1.1 - 10.3	20.1	8.2 - 58.1	0.0	0.0 - 0.0	9.3	3.8 - 25.9	0.55
3130007	8061	MUCKALEE CR	66.5	36.3	13 - 77	0.0	0.0 - 0.0	4.1	1.4 - 8.9	2.6	0.8 - 7.9	17.8	6.4 - 49.7	0.0	0.0 - 0.0	11.7	3.7 - 29.2	0.55
3130007	8062	MILL CR	11.9	33.0	11 - 65	0.0	0.0 - 0.0	14.3	4.1 - 29.7	1.5	0.4 - 5.0	8.8	2.5 - 21.6	0.0	0.0 - 0.0	8.4	2.5 - 21.3	0.53
3130007	8063	MUCKALEE CR	51.6	141.8	59 - 255	87.8	34.6 - 167.7	38.6	15.8 - 85.9	0.9	0.3 - 2.6	5.0	1.8 - 13.3	0.0	0.0 - 0.0	9.5	3.6 - 22.7	0.53
3130007	8064	MUCKALEE CR	1.3	47.4	16 - 96	0.0	0.0 - 0.0	31.7	9.9 - 59.8	0.5	0.1 - 1.3	2.7	0.9 - 6.4	0.0	0.0 - 0.0	12.5	4.5 - 31.2	0.52
3130007	8065	*A	2.6	56.7	17 - 111	0.0	0.0 - 0.0	46.4	14.0 - 98.7	0.4	0.1 - 1.1	2.2	0.7 - 5.9	0.0	0.0 - 0.0	7.7	2.4 - 19.4	0.52
3130007	8066	*A	23.8	34.8	13 - 80	0.0	0.0 - 0.0	10.2	3.6 - 27.2	2.1	0.6 - 6.5	14.4	5.1 - 32.4	0.0	0.0 - 0.0	8.1	3.0 - 20.6	0.50
3130007	8067	*B	30.1	27.3	9 - 66	0.0	0.0 - 0.0	4.3	1.5 - 10.7	3.2	0.8 - 10.5	12.9	4.2 - 33.2	0.0	0.0 - 0.0	6.9	2.1 - 19.9	0.50
3130007	8068	MUCKALEE CR	8.2	31.3	12 - 60	0.0	0.0 - 0.0	9.3	2.9 - 18.1	1.5	0.4 - 4.1	10.1	3.7 - 22.3	0.0	0.0 - 0.0	10.4	3.3 - 23.8	0.52
3130007	8069	LITTLE MUCKALE	81.0	20.8	7 - 49	0.0	0.0 - 0.0	5.2	1.8 - 11.6	2.5	0.7 - 8.1	6.8	2.2 - 15.5	0.0	0.0 - 0.0	6.4	2.3 - 14.6	0.51

8-digit HUC	ID	Name	Area	Catchment Yield		Point sources		Developed Land		Manure		Agricultural Land		Phosphate Mines		Soil parent rock		Frac
				kg/km2	90% CI	kg/km2	90% CI	kg/km2	90% CI	kg/km2	90% CI	kg/km2	90% CI	kg/km2	90% CI	kg/km2	90% CI	
3130007	8070	MUCKALEE CR	222.7	16.9	6 - 34	0.0	0.0 - 0.0	2.6	0.8 - 5.7	1.8	0.5 - 4.7	6.8	2.3 - 16.6	0.0	0.0 - 0.0	5.7	1.8 - 13.9	0.51
3130007	8071	WOLF CR	44.4	28.0	10 - 54	0.0	0.0 - 0.0	4.4	1.6 - 8.7	2.0	0.6 - 5.4	13.4	4.4 - 34.2	0.0	0.0 - 0.0	8.2	3.1 - 19.1	0.52
3130007	8072	MUCKALOOCHEE	167.4	31.8	11 - 66	0.0	0.0 - 0.0	4.2	1.3 - 8.7	2.0	0.6 - 5.6	16.0	5.6 - 37.8	0.0	0.0 - 0.0	9.7	3.1 - 22.3	0.58
3130007	8073	KINCHAFOONEE C	154.4	38.3	14 - 82	0.0	0.0 - 0.0	7.0	2.5 - 13.7	1.6	0.5 - 3.3	18.7	5.8 - 51.2	0.0	0.0 - 0.0	11.1	4.5 - 29.7	0.60
3130007	8074	KINCHAFOONEE C	130.6	23.7	8 - 45	0.0	0.0 - 0.0	3.2	1.1 - 7.2	1.5	0.5 - 4.1	10.7	3.9 - 26.5	0.0	0.0 - 0.0	8.3	3.0 - 21.2	0.57
3130007	8075	CHOCTAWHATCH	101.4	17.4	6 - 36	0.0	0.0 - 0.0	2.8	0.8 - 6.1	1.1	0.3 - 3.3	7.4	2.5 - 18.9	0.0	0.0 - 0.0	6.1	1.9 - 13.3	0.55
3130007	8076	KINCHAFOONEE C	37.8	15.5	5 - 37	0.0	0.0 - 0.0	2.3	0.7 - 5.7	0.8	0.3 - 2.3	7.0	2.4 - 18.1	0.0	0.0 - 0.0	5.2	1.9 - 12.2	0.55
3130007	8077	LANAHASSEE CR	134.5	12.7	4 - 22	0.0	0.0 - 0.0	2.5	0.8 - 4.4	0.8	0.2 - 2.2	3.2	1.0 - 7.0	0.0	0.0 - 0.0	6.3	2.0 - 12.7	0.55
3130007	8078	KINCHAFOONEE C	125.1	16.5	6 - 37	0.0	0.0 - 0.0	4.0	1.3 - 9.0	0.8	0.2 - 2.3	6.0	2.0 - 15.6	0.0	0.0 - 0.0	5.7	2.0 - 15.7	0.55
3130007	8079	KINCHAFOONEE C	18.1	10.3	4 - 20	0.0	0.0 - 0.0	1.9	0.7 - 4.0	0.5	0.2 - 1.4	3.3	1.1 - 7.7	0.0	0.0 - 0.0	4.5	1.7 - 10.0	0.52
3130007	8080	KINCHAFOONEE C	214.4	12.9	5 - 23	0.0	0.0 - 0.0	2.5	0.8 - 4.9	1.0	0.4 - 2.6	3.6	1.4 - 8.1	0.0	0.0 - 0.0	5.8	2.0 - 11.3	0.51
3130007	8081	DRY CR	54.2	7.5	3 - 15	0.0	0.0 - 0.0	1.6	0.5 - 3.3	0.4	0.1 - 1.1	1.7	0.6 - 4.0	0.0	0.0 - 0.0	3.9	1.3 - 9.3	0.51
3130007	8082	SLAUGHTER CR	121.6	9.9	3 - 24	0.0	0.0 - 0.0	1.6	0.5 - 4.1	0.2	0.1 - 0.8	3.1	1.1 - 9.8	0.0	0.0 - 0.0	4.9	1.6 - 12.8	0.52
3130007	8083	BEAR CR	186.0	20.0	6 - 47	0.0	0.0 - 0.0	2.8	0.9 - 7.0	1.0	0.3 - 3.3	10.6	3.7 - 29.8	0.0	0.0 - 0.0	5.6	1.9 - 14.3	0.57
3130007	8084	FOWLTOWN CR	94.0	34.9	12 - 79	0.0	0.0 - 0.0	7.8	2.3 - 18.3	1.0	0.3 - 3.3	15.8	5.4 - 39.1	0.0	0.0 - 0.0	10.2	3.5 - 27.2	0.60
3130008	8085	BIG SLOUGH	843.2	43.9	14 - 73	0.0	0.0 - 0.0	6.7	2.0 - 12.8	6.6	1.7 - 19.5	19.1	5.8 - 49.8	0.0	0.0 - 0.0	11.4	3.8 - 24.7	0.63
3130008	8086	FLINT R	199.4	45.6	14 - 123	0.0	0.0 - 0.0	5.4	1.7 - 12.2	4.9	1.3 - 13.2	24.7	7.6 - 66.4	0.0	0.0 - 0.0	10.7	3.3 - 28.7	0.63
3130008	8087	FLINT R	141.1	43.2	17 - 114	0.0	0.0 - 0.0	6.4	2.4 - 15.3	6.8	2.4 - 20.5	19.8	6.5 - 63.1	0.0	0.0 - 0.0	10.2	3.7 - 24.8	0.62
3130008	8089	RACOON CR	20.8	46.4	16 - 95	0.0	0.0 - 0.0	8.2	2.6 - 19.2	7.9	2.3 - 20.4	18.5	6.1 - 49.0	0.0	0.0 - 0.0	11.9	4.1 - 25.5	0.61
3130008	8090	WETHINGTON SLO	50.9	34.7	12 - 89	0.0	0.0 - 0.0	4.6	1.6 - 10.9	5.5	1.6 - 17.0	13.1	4.3 - 36.2	0.0	0.0 - 0.0	11.4	3.7 - 29.5	0.60
3130008	8091	RACOON CR	194.8	34.3	13 - 76	0.0	0.0 - 0.0	5.7	1.8 - 14.8	4.7	1.4 - 12.8	13.1	4.9 - 34.8	0.0	0.0 - 0.0	10.8	4.1 - 25.7	0.60
3130008	8092	FLINT R	117.2	28.0	10 - 68	0.9	0.4 - 2.3	7.7	2.7 - 19.4	2.2	0.6 - 5.8	6.1	1.9 - 16.5	0.0	0.0 - 0.0	11.0	3.5 - 25.2	0.61
3130008	8093	DRY CR	155.0	28.5	12 - 58	0.0	0.0 - 0.0	4.8	1.7 - 9.9	2.2	0.7 - 6.1	10.9	4.1 - 27.4	0.0	0.0 - 0.0	10.7	4.5 - 23.7	0.61
3130008	8094	FLINT R	272.9	169.8	46 - 454	117.2	31.7 - 327.3	31.5	7.9 - 74.8	0.7	0.2 - 1.9	9.2	2.6 - 26.3	0.0	0.0 - 0.0	11.1	3.1 - 28.9	0.61
3130008	8095	COOLEEWAHEE C	324.4	40.3	13 - 97	0.0	0.0 - 0.0	15.0	4.1 - 30.7	1.5	0.4 - 4.5	11.5	3.5 - 33.0	0.0	0.0 - 0.0	12.3	4.4 - 36.1	0.62
3130009	8096	ICHAWAYNOCHAW	2.3	38.8	12 - 72	0.0	0.0 - 0.0	9.2	3.1 - 18.5	2.6	0.8 - 7.2	16.3	5.6 - 40.0	0.0	0.0 - 0.0	10.8	3.0 - 24.9	0.62
3130009	8097	ICHAWAYNOCHAW	6.9	25.8	8 - 52	0.0	0.0 - 0.0	6.4	2.0 - 13.2	1.4	0.4 - 3.2	8.6	2.5 - 21.7	0.0	0.0 - 0.0	9.4	3.2 - 21.8	0.62
3130009	8098	CHICKASAWHATC	154.7	29.5	10 - 78	0.0	0.0 - 0.0	3.1	1.0 - 8.3	1.6	0.5 - 5.0	10.9	3.9 - 30.9	0.0	0.0 - 0.0	13.8	4.1 - 37.1	0.61
3130009	8099	KIOKEE CR	241.9	21.3	7 - 55	0.0	0.0 - 0.0	2.2	0.7 - 5.4	0.4	0.1 - 1.3	6.4	2.1 - 18.0	0.0	0.0 - 0.0	12.3	3.3 - 34.1	0.58
3130009	8100	CHICKASAWHATC	279.8	33.7	13 - 70	0.0	0.0 - 0.0	2.8	0.9 - 6.2	0.9	0.3 - 2.3	17.0	6.5 - 40.5	0.0	0.0 - 0.0	13.0	4.2 - 27.8	0.58
3130009	8101	CHICKASAWHATC	82.8	40.9	14 - 89	0.0	0.0 - 0.0	6.3	1.9 - 14.3	0.5	0.1 - 1.4	23.8	8.1 - 65.1	0.0	0.0 - 0.0	10.3	3.1 - 27.7	0.52
3130009	8102	BRANTLEY CR	49.1	184.2	68 - 399	140.7	52.3 - 315.4	16.1	5.6 - 37.9	0.3	0.1 - 0.9	16.2	5.3 - 44.4	0.0	0.0 - 0.0	10.7	3.8 - 27.1	0.52
3130009	8103	ICHAWAYNOCHAW	82.2	50.1	18 - 108	0.0	0.0 - 0.0	4.0	1.4 - 8.6	4.1	1.1 - 11.1	28.2	8.7 - 74.5	0.0	0.0 - 0.0	13.7	4.9 - 28.7	0.61
3130009	8104	ICHAWAYNOCHAW	147.2	38.4	13 - 83	0.0	0.0 - 0.0	4.0	1.2 - 8.4	2.6	0.8 - 8.6	20.0	6.3 - 49.7	0.0	0.0 - 0.0	11.8	4.2 - 28.5	0.60
3130009	8105	ICHAWAYNOCHAW	350.8	42.0	14 - 88	0.0	0.0 - 0.0	4.0	1.2 - 7.6	1.6	0.5 - 3.8	24.4	7.3 - 65.8	0.0	0.0 - 0.0	12.0	4.2 - 27.6	0.59
3130009	8106	ICHAWAYNOCHAW	5.4	41.4	14 - 79	0.0	0.0 - 0.0	6.5	2.0 - 12.8	0.4	0.1 - 1.1	18.6	6.6 - 43.4	0.0	0.0 - 0.0	15.9	5.2 - 41.6	0.55
3130009	8107	WOLF CR	48.4	31.7	13 - 58	0.0	0.0 - 0.0	4.0	1.6 - 7.7	0.4	0.1 - 0.8	18.0	6.8 - 47.4	0.0	0.0 - 0.0	9.3	3.7 - 20.2	0.54
3130009	8108	ICHAWAYNOCHAW	14.7	26.2	10 - 52	0.0	0.0 - 0.0	3.1	1.0 - 6.2	0.3	0.1 - 0.8	13.6	4.7 - 28.0	0.0	0.0 - 0.0	9.2	2.9 - 22.1	0.54
3130009	8109	*A	1.3	49.2	17 - 93	0.0	0.0 - 0.0	3.1	1.0 - 5.8	0.4	0.1 - 1.4	26.6	8.6 - 66.3	0.0	0.0 - 0.0	19.0	6.7 - 41.0	0.53

8-digit HUC	ID	Name	Area	Catchment Yield		Point sources		Developed Land		Manure		Agricultural Land		Phosphate Mines		Soil parent rock		Frac
				kg/km^2	90% CI	kg/km^2	90% CI	kg/km^2	90% CI	kg/km^2	90% CI	kg/km^2	90% CI	kg/km^2	90% CI	kg/km^2	90% CI	
3130009	8110	*A	36.5	27.0	10 - 73	0.0	0.0 - 0.0	4.4	1.5 - 9.4	0.3	0.1 - 0.8	13.7	4.5 - 41.0	0.0	0.0 - 0.0	8.7	2.9 - 26.1	0.52
3130009	8111	*B	59.8	23.8	7 - 45	0.0	0.0 - 0.0	2.8	0.7 - 5.5	0.5	0.1 - 1.5	11.9	3.1 - 29.9	0.0	0.0 - 0.0	8.6	2.6 - 19.7	0.52
3130009	8112	ICHAWAYNOCHAV	139.5	17.1	7 - 40	0.0	0.0 - 0.0	2.2	0.9 - 4.8	0.7	0.2 - 1.5	8.2	3.0 - 21.8	0.0	0.0 - 0.0	6.0	2.1 - 15.0	0.53
3130009	8113	*C	160.3	21.0	7 - 52	0.6	0.2 - 1.5	3.3	1.0 - 8.3	0.5	0.1 - 1.3	10.0	3.7 - 27.1	0.0	0.0 - 0.0	6.6	2.3 - 18.8	0.55
3130009	8114	PACHITLA CR	79.2	36.2	11 - 91	0.0	0.0 - 0.0	3.1	1.0 - 8.8	2.2	0.5 - 7.3	18.7	6.6 - 53.5	0.0	0.0 - 0.0	12.2	4.0 - 31.9	0.59
3130009	8115	PACHITLA CR	6.9	42.8	14 - 85	0.0	0.0 - 0.0	3.0	1.0 - 6.5	2.5	0.7 - 8.4	20.4	6.6 - 61.5	0.0	0.0 - 0.0	16.9	5.6 - 37.3	0.57
3130009	8116	PACHITLA CR	14.7	51.4	21 - 113	0.0	0.0 - 0.0	2.7	0.9 - 5.2	3.7	1.3 - 10.4	31.1	12.4 - 81.1	0.0	0.0 - 0.0	13.9	5.0 - 31.4	0.56
3130009	8117	PACHITLA CR	180.9	23.8	8 - 53	0.0	0.0 - 0.0	2.5	0.8 - 5.0	0.6	0.2 - 1.6	13.1	3.8 - 33.7	0.0	0.0 - 0.0	7.6	2.5 - 17.5	0.56
3130009	8118	CARTER CR	158.8	20.6	8 - 42	0.0	0.0 - 0.0	5.0	1.9 - 10.0	0.4	0.1 - 1.3	7.7	2.9 - 19.4	0.0	0.0 - 0.0	7.5	2.7 - 16.4	0.56
3130009	8119	LITTLE PACHITLA	115.2	33.5	12 - 73	0.0	0.0 - 0.0	4.6	1.5 - 9.1	1.6	0.5 - 4.7	16.7	5.6 - 41.5	0.0	0.0 - 0.0	10.7	3.7 - 23.8	0.56
3130009	8120	NEALS CR	91.6	39.8	14 - 81	0.0	0.0 - 0.0	5.1	1.6 - 11.4	2.5	0.8 - 6.9	20.7	6.4 - 50.6	0.0	0.0 - 0.0	11.5	4.0 - 24.3	0.57
3130009	8121	*D	75.5	39.8	13 - 102	0.0	0.0 - 0.0	5.7	1.8 - 13.5	2.4	0.7 - 6.9	21.3	7.2 - 68.7	0.0	0.0 - 0.0	10.4	3.4 - 26.4	0.60
3130009	8122	BIG CYPRESS CR	157.2	46.5	17 - 119	0.0	0.0 - 0.0	4.6	1.5 - 13.3	3.5	1.0 - 13.0	26.6	8.6 - 74.7	0.0	0.0 - 0.0	11.8	4.6 - 36.8	0.62
3130010	8123	SPRING CR	3.8	75.8	25 - 177	0.0	0.0 - 0.0	2.6	0.9 - 5.8	4.9	1.2 - 16.5	43.5	13.1 - 126.1	0.0	0.0 - 0.0	24.8	8.1 - 66.3	0.62
3130010	8124	SPRING CR	199.2	58.9	21 - 140	0.0	0.0 - 0.0	8.2	3.1 - 16.6	3.5	1.1 - 10.1	32.8	10.3 - 101.7	0.0	0.0 - 0.0	14.4	5.1 - 32.4	0.61
3130010	8125	SPRING CR, LONG	134.2	48.5	17 - 122	0.0	0.0 - 0.0	5.7	2.0 - 12.8	2.5	0.7 - 7.1	26.5	9.5 - 67.9	0.0	0.0 - 0.0	13.8	4.5 - 34.2	0.59
3130010	8126	SPRING CR	15.7	74.8	28 - 138	0.0	0.0 - 0.0	5.2	1.8 - 10.3	4.3	1.4 - 12.1	41.7	14.8 - 89.7	0.0	0.0 - 0.0	23.5	8.2 - 57.0	0.59
3130010	8127	SPRING CR	116.9	47.1	16 - 94	0.0	0.0 - 0.0	3.1	1.0 - 6.1	2.2	0.6 - 7.6	27.3	9.6 - 67.1	0.0	0.0 - 0.0	14.5	5.0 - 37.9	0.57
3130010	8128	PERRY CR	56.1	46.3	17 - 94	0.0	0.0 - 0.0	4.9	1.8 - 9.6	2.7	0.9 - 7.1	25.7	8.7 - 55.0	0.0	0.0 - 0.0	13.1	4.7 - 26.9	0.52
3130010	8129	SPRING CR	141.5	42.7	17 - 115	0.0	0.0 - 0.0	3.0	1.2 - 7.2	2.2	0.8 - 5.6	24.4	8.9 - 78.6	0.0	0.0 - 0.0	13.1	5.2 - 36.4	0.52
3130010	8130	DRY CR	276.3	48.3	18 - 116	5.1	2.0 - 14.3	6.9	2.4 - 19.9	1.9	0.6 - 6.2	21.4	7.7 - 65.5	0.0	0.0 - 0.0	13.0	4.9 - 37.7	0.57
3130010	8131	AYCOCKS CR	5.4	74.0	25 - 153	0.0	0.0 - 0.0	4.2	1.3 - 8.5	5.1	1.4 - 12.7	47.3	16.6 - 104.7	0.0	0.0 - 0.0	17.4	4.8 - 44.5	0.61
3130010	8132	CYPRESS CR	61.9	33.6	12 - 77	0.0	0.0 - 0.0	4.2	1.4 - 8.5	1.6	0.4 - 5.7	14.1	4.6 - 35.1	0.0	0.0 - 0.0	13.8	4.1 - 35.3	0.55
3130010	8133	AYCOCKS CR	123.8	44.8	16 - 87	0.0	0.0 - 0.0	4.4	1.5 - 9.1	2.5	0.7 - 6.5	24.5	9.1 - 53.2	0.0	0.0 - 0.0	13.4	4.2 - 29.9	0.55
3130010	8134	DRY CR	144.7	65.5	18 - 167	0.0	0.0 - 0.0	5.4	1.5 - 10.6	4.6	1.2 - 14.0	40.8	10.8 - 111.8	0.0	0.0 - 0.0	14.8	5.1 - 37.3	0.62
3130011	8135	EAST R	76.8	15.3	5 - 33	0.0	0.0 - 0.0	1.4	0.4 - 2.9	0.0	0.0 - 0.0	0.0	0.0 - 0.1	0.0	0.0 - 0.0	13.9	4.1 - 32.5	1.00
3130011	8136	APALACHICOLA R	70.3	24.9	8 - 56	0.0	0.0 - 0.0	4.2	1.4 - 8.5	0.0	0.0 - 0.0	0.7	0.2 - 1.6	0.0	0.0 - 0.0	19.9	6.5 - 47.9	1.00
3130011	8137	APALACHICOLA R	150.6	18.9	5 - 37	0.0	0.0 - 0.0	0.8	0.2 - 1.9	0.0	0.0 - 0.1	0.2	0.1 - 0.5	0.0	0.0 - 0.0	17.8	5.1 - 36.3	0.99
3130011	8138	FLORIDA R	302.6	29.2	9 - 69	0.0	0.0 - 0.0	3.0	0.8 - 6.4	0.2	0.1 - 0.8	1.8	0.5 - 5.1	0.0	0.0 - 0.0	24.1	7.8 - 58.4	0.99
3130011	8139	APALACHICOLA R	318.7	25.7	9 - 53	0.2	0.1 - 0.5	4.2	1.4 - 8.0	0.5	0.1 - 1.5	3.9	1.2 - 9.5	0.0	0.0 - 0.0	16.9	5.1 - 38.3	0.99
3130011	8140	APALACHICOLA R	354.3	29.4	9 - 73	0.0	0.0 - 0.0	5.1	1.5 - 13.9	1.2	0.3 - 4.6	8.3	2.8 - 23.1	0.0	0.0 - 0.0	14.9	4.7 - 42.1	0.98
3130011	8141	FLAT CR	2.6	9.4	3 - 29	0.0	0.0 - 0.0	1.0	0.3 - 3.1	0.0	0.0 - 0.0	0.0	0.0 - 0.0	0.0	0.0 - 0.0	8.3	2.6 - 26.7	0.98
3130011	8142	*A	50.4	15.8	5 - 36	0.0	0.0 - 0.0	2.5	0.9 - 5.3	0.3	0.1 - 0.8	1.7	0.6 - 4.4	0.0	0.0 - 0.0	11.3	3.6 - 29.1	0.97
3130011	8143	APALACHICOLA R	310.2	33.7	12 - 82	2.9	1.0 - 7.5	7.5	2.5 - 18.5	1.3	0.4 - 3.4	8.0	2.5 - 20.6	0.0	0.0 - 0.0	14.0	4.7 - 39.0	0.98
3130011	8144	STAFFORD CR	99.4	36.7	12 - 63	0.0	0.0 - 0.0	9.7	3.1 - 18.7	0.9	0.3 - 2.4	11.6	4.0 - 24.3	0.0	0.0 - 0.0	14.4	4.4 - 35.1	0.98
3130011	8145	APALACHICOLA R	27.2	6.7	2 - 17	0.0	0.0 - 0.0	1.6	0.4 - 3.7	0.0	0.0 - 0.0	0.1	0.0 - 0.2	0.0	0.0 - 0.0	5.0	1.5 - 14.5	1.00
3130011	8146	APALACHICOLA R	2.8	5.7	2 - 14	0.0	0.0 - 0.0	0.0	0.0 - 0.0	0.0	0.0 - 0.0	0.0	0.0 - 0.0	0.0	0.0 - 0.0	5.7	2.2 - 13.9	1.00
3130011	8147	BROTHERS R	135.0	18.8	6 - 47	0.0	0.0 - 0.0	1.3	0.5 - 3.1	0.0	0.0 - 0.0	0.4	0.1 - 1.0	0.0	0.0 - 0.0	17.1	6.0 - 45.8	1.00
3130011	8148	*B	27.6	2.6	0 - 10	0.0	0.0 - 0.0	0.0	0.0 - 0.2	0.0	0.0 - 0.0	0.0	0.0 - 0.0	0.0	0.0 - 0.0	2.5	0.4 - 9.5	1.00

8-digit HUC	ID	Name	Area	Catchment Yield		Point sources		Developed Land		Manure		Agricultural Land		Phosphate Mines		Soil parent rock		Frac
				kg/km^2	90% CI	kg/km^2	90% CI	kg/km^2	90% CI	kg/km^2	90% CI	kg/km^2	90% CI	kg/km^2	90% CI	kg/km^2	90% CI	
3130011	8149	FLAT CR	84.6	20.0	7 - 36	0.0	0.0 - 0.0	5.3	1.7 - 11.4	0.5	0.1 - 1.2	2.8	0.8 - 7.2	0.0	0.0 - 0.0	11.4	3.5 - 21.3	0.97
3130012	8150	CHIPOLA R	95.4	27.5	9 - 75	2.3	0.8 - 6.5	6.4	1.9 - 18.2	0.0	0.0 - 0.1	3.9	1.2 - 11.5	0.0	0.0 - 0.0	14.9	4.7 - 41.7	0.99
3130012	8151	CHIPOLA R	77.6	33.4	13 - 60	0.0	0.0 - 0.0	7.3	2.5 - 15.5	0.5	0.2 - 1.4	5.4	1.8 - 12.6	0.0	0.0 - 0.0	20.2	7.7 - 47.1	0.70
3130012	8152	CHIPOLA R	231.1	26.9	10 - 65	0.0	0.0 - 0.0	7.7	2.5 - 18.9	0.6	0.2 - 1.8	4.4	1.3 - 11.3	0.0	0.0 - 0.0	14.2	4.9 - 33.8	0.70
3130012	8153	CHIPOLA R	592.1	46.2	16 - 93	3.2	1.1 - 7.1	10.6	3.6 - 22.0	3.1	1.0 - 8.5	15.8	4.8 - 40.8	0.0	0.0 - 0.0	13.6	4.3 - 28.7	0.68
3130012	8154	COWARTS CR	399.1	49.4	17 - 116	0.0	0.0 - 0.0	7.3	2.3 - 16.9	3.7	1.1 - 10.9	26.0	8.5 - 74.2	0.0	0.0 - 0.0	12.4	4.5 - 29.3	0.66
3130012	8155	BIG CR	564.6	46.8	14 - 87	0.4	0.1 - 1.0	10.1	3.2 - 21.1	3.5	1.1 - 9.1	20.6	6.0 - 47.6	0.0	0.0 - 0.0	12.0	3.8 - 30.0	0.66
3130012	8156	DRY CR	386.1	25.8	8 - 66	0.0	0.0 - 0.0	7.3	2.2 - 19.2	1.5	0.4 - 5.9	6.2	2.0 - 18.3	0.0	0.0 - 0.0	10.9	3.6 - 33.4	0.68
3130012	8157	FOURMILE CR	98.7	21.0	8 - 62	0.0	0.0 - 0.0	8.5	2.9 - 22.3	0.4	0.1 - 1.3	2.5	0.9 - 8.3	0.0	0.0 - 0.0	9.6	3.4 - 29.7	0.70
3130012	8158	JUNIPER CR	159.7	23.7	8 - 74	0.0	0.0 - 0.0	7.5	2.5 - 22.2	0.4	0.1 - 1.2	1.5	0.4 - 4.3	0.0	0.0 - 0.0	14.4	4.6 - 36.1	0.70
3130013	8159	NEW R	13.6	21.2	6 - 48	0.0	0.0 - 0.0	8.2	2.3 - 18.3	0.0	0.0 - 0.0	0.2	0.1 - 0.5	0.0	0.0 - 0.0	12.8	3.6 - 31.1	1.00
3130013	8160	CROOKED R	203.7	28.2	10 - 77	0.0	0.0 - 0.0	4.1	1.4 - 10.7	0.0	0.0 - 0.0	0.2	0.1 - 0.4	0.0	0.0 - 0.0	23.9	8.4 - 69.4	0.99
3130013	8161	NEW R	610.0	31.6	11 - 75	0.0	0.0 - 0.0	3.2	1.0 - 7.4	0.0	0.0 - 0.1	0.0	0.0 - 0.1	0.0	0.0 - 0.0	28.4	9.7 - 68.6	0.99
3130013	8162	NEW R, CAT BR	50.3	37.4	14 - 79	0.0	0.0 - 0.0	2.2	0.8 - 4.8	0.1	0.0 - 0.2	0.0	0.0 - 0.0	0.0	0.0 - 0.0	35.2	12.8 - 76.6	0.92
3130013	8163	NEW R	270.0	29.1	10 - 62	0.0	0.0 - 0.0	3.0	1.0 - 6.5	0.0	0.0 - 0.1	0.1	0.0 - 0.2	0.0	0.0 - 0.0	26.0	8.4 - 60.8	0.92
3140101	8164	WETAPPO CR	273.8	26.0	9 - 60	0.0	0.0 - 0.0	1.7	0.5 - 3.7	0.0	0.0 - 0.1	0.2	0.1 - 0.6	0.0	0.0 - 0.0	24.0	8.1 - 62.9	1.00
3140101	8165	SANDY CR	4.2	25.1	8 - 54	0.0	0.0 - 0.0	3.3	1.0 - 7.3	0.1	0.0 - 0.3	0.0	0.0 - 0.0	0.0	0.0 - 0.0	21.7	7.3 - 47.9	1.00
3140101	8166	ALLIGATOR CR	60.6	29.1	8 - 76	0.0	0.0 - 0.0	2.1	0.7 - 4.9	0.0	0.0 - 0.0	0.5	0.1 - 1.4	0.0	0.0 - 0.0	26.5	7.2 - 66.1	0.99
3140101	8167	SANDY CR	88.1	28.2	9 - 58	0.0	0.0 - 0.0	5.7	1.9 - 11.0	0.1	0.0 - 0.4	0.0	0.0 - 0.1	0.0	0.0 - 0.0	22.3	7.6 - 51.6	0.99
3140101	8168	CALAWAY CR	47.1	33.0	9 - 62	0.0	0.0 - 0.0	11.0	3.2 - 21.7	0.1	0.0 - 0.2	0.3	0.1 - 0.7	0.0	0.0 - 0.0	21.6	6.1 - 46.9	1.00
3140101	8169	BAYOU GEORGEJ	154.9	31.4	9 - 59	0.0	0.0 - 0.0	11.4	3.0 - 23.4	0.0	0.0 - 0.1	0.6	0.2 - 1.5	0.0	0.0 - 0.0	19.5	5.9 - 44.0	1.00
3140101	8170	BEAR CR	73.1	32.2	10 - 75	0.0	0.0 - 0.0	9.6	2.8 - 27.1	0.3	0.1 - 0.8	2.7	0.8 - 8.3	0.0	0.0 - 0.0	19.6	6.3 - 49.0	1.00
3140101	8171	BEAR CR	162.5	25.9	8 - 50	0.0	0.0 - 0.0	8.9	2.7 - 16.6	0.2	0.1 - 0.4	1.6	0.5 - 3.7	0.0	0.0 - 0.0	15.1	5.2 - 34.9	0.97
3140101	8172	LITTLE BEAR CR	84.9	21.6	8 - 57	0.0	0.0 - 0.0	9.1	3.0 - 25.0	0.2	0.1 - 0.5	1.1	0.4 - 3.1	0.0	0.0 - 0.0	11.2	3.8 - 31.3	0.97
3140101	8173	ECONFINA CR	1.1	13.8	5 - 37	0.0	0.0 - 0.0	0.7	0.2 - 1.7	0.0	0.0 - 0.1	1.3	0.4 - 4.2	0.0	0.0 - 0.0	11.8	4.4 - 33.8	1.00
3140101	8174	ECONFINA CR	25.1	11.7	4 - 26	0.0	0.0 - 0.0	2.8	0.7 - 6.4	0.1	0.0 - 0.2	0.3	0.1 - 0.8	0.0	0.0 - 0.0	8.5	2.8 - 23.4	1.00
3140101	8175	CEDAR CR	292.5	26.9	8 - 63	0.0	0.0 - 0.0	13.8	3.7 - 33.1	0.5	0.1 - 1.2	1.2	0.3 - 3.4	0.0	0.0 - 0.0	11.5	3.8 - 25.3	1.00
3140101	8176	BURNT MILL CR	127.6	18.1	6 - 32	0.0	0.0 - 0.0	6.4	2.0 - 11.7	0.1	0.0 - 0.1	0.1	0.0 - 0.2	0.0	0.0 - 0.0	11.6	3.8 - 25.8	1.00
3140101	8177	BIG CROOKED CR	56.2	17.6	6 - 38	0.0	0.0 - 0.0	4.7	1.4 - 9.8	0.1	0.0 - 0.4	0.1	0.0 - 0.2	0.0	0.0 - 0.0	12.6	3.8 - 29.9	1.00
3140101	8178	INTRACOASTAL W	160.3	16.9	5 - 32	0.0	0.0 - 0.0	4.4	1.3 - 9.8	0.1	0.0 - 0.4	0.1	0.0 - 0.4	0.0	0.0 - 0.0	12.3	3.4 - 29.4	1.00
3140101	8179	INTRACOASTAL W	343.1	25.5	10 - 54	0.0	0.0 - 0.0	2.9	1.0 - 5.5	0.0	0.0 - 0.0	0.2	0.1 - 0.5	0.0	0.0 - 0.0	22.4	8.2 - 51.3	1.00
3140102	8180	LAFAYETTE CR	1.1	31.8	12 - 73	0.0	0.0 - 0.0	8.4	2.8 - 19.7	1.6	0.5 - 5.1	4.6	1.6 - 14.5	0.0	0.0 - 0.0	17.2	7.0 - 41.1	1.00
3140102	8181	LAFAYETTE CR	223.3	35.4	14 - 72	0.0	0.0 - 0.0	8.1	2.8 - 17.3	4.4	1.4 - 12.8	14.6	5.7 - 38.2	0.0	0.0 - 0.0	8.3	3.4 - 21.1	0.99
3140102	8182	FIVEMILE CR	39.8	27.1	10 - 64	0.0	0.0 - 0.0	14.2	5.2 - 32.8	1.7	0.6 - 4.9	4.6	1.5 - 11.2	0.0	0.0 - 0.0	6.6	2.5 - 17.0	0.99
3140102	8183	ALAQUA CR	31.0	20.9	7 - 40	0.0	0.0 - 0.0	10.0	3.4 - 20.4	1.0	0.3 - 2.8	1.4	0.5 - 3.4	0.0	0.0 - 0.0	8.5	3.3 - 19.9	1.00
3140102	8184	ALAQUA CR	65.8	15.7	5 - 32	0.0	0.0 - 0.0	7.1	2.4 - 15.8	0.3	0.1 - 0.8	0.7	0.2 - 1.7	0.0	0.0 - 0.0	7.6	2.7 - 16.7	0.98
3140102	8185	*A	33.0	32.6	12 - 73	0.0	0.0 - 0.0	21.5	7.8 - 46.3	0.6	0.2 - 1.8	2.1	0.7 - 6.1	0.0	0.0 - 0.0	8.4	3.0 - 18.8	0.95
3140102	8186	ALAQUA CR	19.4	12.8	4 - 32	0.0	0.0 - 0.0	4.5	1.4 - 12.3	1.1	0.3 - 3.3	0.2	0.1 - 0.5	0.0	0.0 - 0.0	6.9	2.2 - 17.8	0.95
3140102	8187	LITTLE ALAQUA C	78.6	13.0	4 - 31	0.0	0.0 - 0.0	6.2	1.8 - 15.9	0.6	0.2 - 2.1	0.0	0.0 - 0.1	0.0	0.0 - 0.0	6.2	2.0 - 17.3	0.98

8-digit HUC	ID	Name	Area	Catchment Yield		Point sources		Developed Land		Manure		Agricultural Land		Phosphate Mines		Soil parent rock		Frac
				kg/km^2	90% CI	kg/km^2	90% CI	kg/km^2	90% CI	kg/km^2	90% CI	kg/km^2	90% CI	kg/km^2	90% CI	kg/km^2	90% CI	
3140102	8188	BASIN CR	102.6	14.9	5 - 35	0.0	0.0 - 0.0	8.3	2.5 - 18.9	0.8	0.2 - 2.5	0.0	0.0 - 0.1	0.0	0.0 - 0.0	5.7	2.0 - 15.2	1.00
3140102	8189	ROCKY CR	48.3	10.1	3 - 19	0.0	0.0 - 0.0	5.6	1.9 - 11.2	0.2	0.1 - 0.7	0.0	0.0 - 0.0	0.0	0.0 - 0.0	4.2	1.3 - 9.8	1.00
3140102	8190	ROCKY CR	122.0	17.3	7 - 40	0.0	0.0 - 0.0	10.0	3.7 - 23.6	0.8	0.3 - 2.2	0.1	0.0 - 0.3	0.0	0.0 - 0.0	6.4	2.5 - 16.6	0.97
3140102	8191	M ROCKY CR	48.9	16.2	6 - 38	0.0	0.0 - 0.0	9.9	3.3 - 23.8	0.1	0.0 - 0.4	0.0	0.0 - 0.1	0.0	0.0 - 0.0	6.1	2.2 - 16.2	0.97
3140102	8192	SWIFT CR	24.9	60.8	17 - 120	0.0	0.0 - 0.0	54.5	14.6 - 109.5	0.2	0.0 - 0.4	1.1	0.3 - 3.1	0.0	0.0 - 0.0	5.0	2.0 - 10.0	1.00
3140102	8193	TURKEY CR	13.4	43.2	12 - 96	0.0	0.0 - 0.0	38.6	10.5 - 84.6	0.1	0.0 - 0.4	0.0	0.0 - 0.0	0.0	0.0 - 0.0	4.4	1.2 - 10.4	1.00
3140102	8194	*B	8.1	17.8	5 - 37	0.0	0.0 - 0.0	13.6	3.9 - 29.8	0.1	0.0 - 0.2	0.0	0.0 - 0.0	0.0	0.0 - 0.0	4.1	1.3 - 10.7	0.98
3140102	8195	POINT LOOKOUT (	44.9	22.4	6 - 43	0.0	0.0 - 0.0	16.0	4.6 - 36.4	0.1	0.0 - 0.3	0.1	0.0 - 0.2	0.0	0.0 - 0.0	6.3	1.7 - 13.8	0.96
3140102	8196	*B	22.5	22.6	8 - 46	0.0	0.0 - 0.0	16.2	5.5 - 35.8	0.1	0.0 - 0.3	0.0	0.0 - 0.0	0.0	0.0 - 0.0	6.3	2.2 - 13.9	0.96
3140102	8197	TURKEY CR	85.9	21.2	6 - 39	0.0	0.0 - 0.0	15.0	4.2 - 33.2	0.1	0.0 - 0.3	0.0	0.0 - 0.1	0.0	0.0 - 0.0	6.2	1.8 - 13.4	0.98
3140103	8198	YELLOW R	83.1	27.6	9 - 50	0.0	0.0 - 0.0	13.0	4.1 - 27.5	0.5	0.1 - 1.5	6.1	1.9 - 15.7	0.0	0.0 - 0.0	8.0	3.0 - 17.1	1.00
3140103	8199	BOILING CR	98.4	23.6	8 - 48	0.0	0.0 - 0.0	16.6	5.2 - 34.2	0.5	0.2 - 1.5	0.0	0.0 - 0.0	0.0	0.0 - 0.0	6.5	2.2 - 14.4	0.99
3140103	8200	YELLOW R	138.9	23.8	7 - 46	0.0	0.0 - 0.0	12.5	3.7 - 25.0	0.3	0.1 - 0.7	2.7	0.9 - 6.9	0.0	0.0 - 0.0	8.3	2.9 - 17.7	0.99
3140103	8201	*A	32.3	20.8	7 - 52	0.0	0.0 - 0.0	14.6	4.9 - 40.4	0.1	0.0 - 0.4	0.0	0.0 - 0.0	0.0	0.0 - 0.0	6.0	2.2 - 15.7	0.98
3140103	8202	YELLOW R	23.3	36.1	12 - 68	0.0	0.0 - 0.0	19.2	5.7 - 39.1	0.6	0.2 - 1.4	4.3	1.2 - 11.0	0.0	0.0 - 0.0	12.0	4.5 - 24.3	0.98
3140103	8203	METTS CR	21.8	24.4	8 - 57	0.0	0.0 - 0.0	17.4	5.2 - 42.2	0.0	0.0 - 0.2	0.1	0.0 - 0.4	0.0	0.0 - 0.0	6.8	2.2 - 17.9	0.98
3140103	8204	YELLOW R	22.8	32.2	10 - 65	0.0	0.0 - 0.0	14.4	4.4 - 31.3	0.6	0.2 - 1.9	5.5	1.9 - 15.8	0.0	0.0 - 0.0	11.7	3.9 - 27.0	0.98
3140103	8205	MALONE CR	26.4	19.2	6 - 37	0.0	0.0 - 0.0	12.9	4.2 - 26.1	0.1	0.0 - 0.2	0.0	0.0 - 0.0	0.0	0.0 - 0.0	6.3	2.1 - 13.7	0.97
3140103	8206	YELLOW R	41.2	20.4	7 - 40	0.0	0.0 - 0.0	10.7	3.4 - 22.3	0.1	0.0 - 0.3	0.8	0.2 - 2.0	0.0	0.0 - 0.0	8.8	3.1 - 20.0	0.97
3140103	8207	TURKEY GOBLER	31.8	15.7	6 - 37	0.0	0.0 - 0.0	9.6	3.3 - 24.5	0.1	0.0 - 0.3	0.0	0.0 - 0.0	0.0	0.0 - 0.0	6.0	2.2 - 13.7	0.97
3140103	8208	YELLOW R	9.8	20.1	8 - 44	0.0	0.0 - 0.0	12.5	4.7 - 27.0	0.1	0.0 - 0.3	0.0	0.0 - 0.0	0.0	0.0 - 0.0	7.5	2.8 - 16.0	0.97
3140103	8209	SHOAL R	93.7	38.0	13 - 71	0.0	0.0 - 0.0	28.6	9.2 - 68.4	0.2	0.1 - 0.5	0.8	0.2 - 2.1	0.0	0.0 - 0.0	8.4	3.0 - 18.5	0.97
3140103	8210	TITI CR	201.6	28.0	10 - 49	0.0	0.0 - 0.0	15.1	5.2 - 27.7	0.7	0.2 - 1.5	4.5	1.4 - 10.2	0.0	0.0 - 0.0	7.7	2.7 - 15.3	0.96
3140103	8211	SHOAL R	180.8	39.8	14 - 93	0.0	0.0 - 0.0	25.1	8.6 - 55.3	0.7	0.2 - 2.4	5.5	2.1 - 13.9	0.0	0.0 - 0.0	8.4	3.1 - 19.4	0.96
3140103	8212	SHOAL R	170.2	35.5	10 - 68	0.0	0.0 - 0.0	18.0	4.6 - 42.1	2.5	0.8 - 6.1	8.6	2.7 - 22.7	0.0	0.0 - 0.0	6.5	2.0 - 13.3	0.93
3140103	8213	GUM CR	114.6	48.1	14 - 120	0.0	0.0 - 0.0	16.7	4.7 - 42.7	3.7	0.9 - 11.8	13.1	4.3 - 43.8	0.0	0.0 - 0.0	14.5	4.5 - 37.0	0.89
3140103	8214	CANEY CR	107.4	42.6	13 - 93	0.0	0.0 - 0.0	5.9	1.8 - 13.4	5.7	1.6 - 17.6	20.3	5.9 - 59.6	0.0	0.0 - 0.0	10.7	3.4 - 27.4	0.89
3140103	8215	POND CR	4.4	14.5	5 - 30	0.0	0.0 - 0.0	2.2	0.8 - 4.4	0.7	0.2 - 2.0	2.6	0.9 - 6.5	0.0	0.0 - 0.0	9.0	3.1 - 20.9	0.93
3140103	8216	LONG CR	75.1	23.0	9 - 50	0.0	0.0 - 0.0	4.9	1.9 - 10.5	2.6	0.9 - 6.8	8.1	3.1 - 19.6	0.0	0.0 - 0.0	7.4	2.9 - 17.9	0.93
3140103	8218	PINE LOG CR	85.6	22.2	8 - 41	0.0	0.0 - 0.0	4.5	1.4 - 9.6	2.4	0.7 - 6.7	8.5	2.8 - 22.3	0.0	0.0 - 0.0	6.8	2.4 - 14.4	0.93
3140103	8219	POND CR	247.4	31.7	12 - 68	0.0	0.0 - 0.0	9.5	3.4 - 21.8	3.3	0.9 - 12.9	12.6	4.0 - 33.1	0.0	0.0 - 0.0	6.4	2.3 - 13.8	0.93
3140103	8220	YELLOW R	76.1	36.3	13 - 77	0.0	0.0 - 0.0	19.4	6.2 - 43.3	0.7	0.2 - 2.1	5.0	1.7 - 13.0	0.0	0.0 - 0.0	11.2	4.1 - 27.5	0.97
3140103	8221	YELLOW R	46.6	43.9	16 - 90	0.0	0.0 - 0.0	7.3	2.6 - 14.0	6.0	2.1 - 15.6	12.5	4.5 - 29.7	0.0	0.0 - 0.0	18.1	7.0 - 38.5	0.92
3140103	8222	LIMESTONE CR	153.1	41.2	12 - 106	0.0	0.0 - 0.0	9.7	2.9 - 22.4	4.6	1.1 - 14.7	10.1	3.0 - 29.4	0.0	0.0 - 0.0	16.7	5.1 - 43.6	0.91
3140103	8223	YELLOW R	96.2	46.4	20 - 113	0.0	0.0 - 0.0	5.9	2.2 - 14.3	6.7	2.2 - 19.2	14.4	5.3 - 40.3	0.0	0.0 - 0.0	19.5	7.9 - 49.2	0.91
3140103	8224	INDIAN CR	58.9	56.3	20 - 147	0.0	0.0 - 0.0	19.4	6.4 - 52.0	7.3	2.5 - 26.1	15.9	5.6 - 43.0	0.0	0.0 - 0.0	13.7	4.8 - 38.4	0.89
3140103	8225	YELLOW R	59.9	47.5	16 - 108	0.0	0.0 - 0.0	7.4	2.4 - 19.8	8.0	2.1 - 22.1	17.3	6.3 - 43.0	0.0	0.0 - 0.0	14.8	5.8 - 43.1	0.89
3140103	8226	POND CR	20.4	45.6	15 - 101	0.0	0.0 - 0.0	10.7	3.5 - 24.9	8.5	2.6 - 26.1	18.3	5.8 - 47.7	0.0	0.0 - 0.0	8.1	2.4 - 21.2	0.88
3140103	8227	POND CR	29.6	77.6	26 - 140	23.5	9.1 - 45.2	23.2	8.2 - 40.2	8.0	2.3 - 20.0	17.6	5.5 - 41.7	0.0	0.0 - 0.0	5.2	1.8 - 10.2	0.87

8-digit HUC	ID	Name	Area	Catchment Yield		Point sources		Developed Land		Manure		Agricultural Land		Phosphate Mines		Soil parent rock		Frac
				kg/km^2	90% CI	kg/km^2	90% CI	kg/km^2	90% CI	kg/km^2	90% CI	kg/km^2	90% CI	kg/km^2	90% CI	kg/km^2	90% CI	
3140103	8228	POND CR	50.5	51.7	18 - 113	0.0	0.0 - 0.0	7.6	2.4 - 17.6	15.1	4.8 - 42.9	23.8	8.1 - 60.6	0.0	0.0 - 0.0	5.1	1.6 - 13.0	0.84
3140103	8229	LIGHTWOOD KNO	119.1	40.6	13 - 101	0.0	0.0 - 0.0	6.7	2.0 - 14.7	12.8	3.4 - 47.5	16.7	4.8 - 43.4	0.0	0.0 - 0.0	4.4	1.4 - 11.7	0.84
3140103	8230	POLEY CR	85.5	40.3	13 - 71	0.0	0.0 - 0.0	6.2	1.8 - 11.4	9.8	2.9 - 25.9	19.9	5.8 - 49.7	0.0	0.0 - 0.0	4.5	1.4 - 8.3	0.87
3140103	8231	YELLOW R	104.9	37.5	10 - 79	0.0	0.0 - 0.0	7.5	1.8 - 16.6	8.1	1.8 - 23.5	17.2	5.1 - 52.6	0.0	0.0 - 0.0	4.6	1.3 - 10.7	0.88
3140103	8232	FIVE RUNS CR	148.0	38.5	13 - 80	0.0	0.0 - 0.0	7.0	2.5 - 14.9	4.9	1.3 - 16.1	10.5	3.9 - 28.2	0.0	0.0 - 0.0	16.2	6.2 - 37.1	0.92
3140103	8233	FIVE RUNS CR	138.5	39.8	15 - 79	0.0	0.0 - 0.0	11.7	4.4 - 22.3	7.3	2.3 - 21.3	15.6	5.8 - 38.4	0.0	0.0 - 0.0	5.4	2.2 - 11.3	0.88
3140103	8234	HOG FOOT CR	39.7	61.6	21 - 128	0.0	0.0 - 0.0	22.7	6.7 - 49.9	11.1	3.4 - 36.4	24.0	7.4 - 60.0	0.0	0.0 - 0.0	3.7	1.3 - 9.0	0.88
3140104	8235	BLACKWATER R	2.2	35.8	11 - 65	0.0	0.0 - 0.0	15.6	4.6 - 30.4	0.3	0.1 - 0.6	3.8	1.1 - 9.1	0.0	0.0 - 0.0	16.1	5.9 - 34.0	1.00
3140104	8236	BLACKWATER R	44.9	98.7	40 - 179	11.5	4.5 - 24.2	72.7	27.7 - 148.2	0.3	0.1 - 0.6	3.8	1.4 - 10.5	0.0	0.0 - 0.0	10.5	4.1 - 20.3	1.00
3140104	8237	BLACKWATER R	32.1	32.9	11 - 63	0.0	0.0 - 0.0	18.5	5.8 - 38.0	0.6	0.1 - 1.8	4.1	1.2 - 10.1	0.0	0.0 - 0.0	9.8	3.2 - 26.3	0.99
3140104	8238	BLACKWATER R	32.8	23.3	8 - 52	0.0	0.0 - 0.0	9.3	2.8 - 20.9	0.3	0.1 - 0.9	2.7	0.9 - 7.5	0.0	0.0 - 0.0	10.9	4.0 - 25.9	0.98
3140104	8239	BLACKWATER R	254.5	30.8	12 - 53	0.0	0.0 - 0.0	11.0	4.1 - 19.9	0.9	0.3 - 2.5	8.5	3.0 - 20.6	0.0	0.0 - 0.0	10.4	4.2 - 20.3	0.97
3140104	8240	PANTHER CR	78.0	28.2	10 - 69	0.0	0.0 - 0.0	6.9	2.0 - 14.7	0.9	0.3 - 2.4	9.6	3.2 - 30.4	0.0	0.0 - 0.0	10.8	3.7 - 23.9	0.92
3140104	8241	BLACKWATER R	222.1	23.7	8 - 52	0.0	0.0 - 0.0	5.9	1.9 - 12.6	0.7	0.2 - 1.8	5.1	1.6 - 12.5	0.0	0.0 - 0.0	12.1	4.2 - 26.1	0.92
3140104	8242	*A	78.8	23.2	9 - 46	0.0	0.0 - 0.0	4.9	1.7 - 10.3	2.8	1.0 - 6.9	6.3	2.0 - 15.6	0.0	0.0 - 0.0	9.2	3.7 - 21.6	0.86
3140104	8244	PANTHER CR	58.8	26.4	9 - 57	0.0	0.0 - 0.0	4.6	1.3 - 9.3	4.4	1.3 - 11.8	10.1	3.5 - 24.4	0.0	0.0 - 0.0	7.3	2.4 - 17.2	0.86
3140104	8245	BLACKWATER R	77.4	16.1	6 - 43	0.0	0.0 - 0.0	2.7	1.0 - 6.6	1.1	0.4 - 2.7	5.2	1.8 - 18.4	0.0	0.0 - 0.0	7.1	2.6 - 20.0	0.86
3140104	8246	BIG JUNIPER CR	108.4	23.2	8 - 46	0.0	0.0 - 0.0	8.2	2.8 - 18.8	0.3	0.1 - 0.9	2.2	0.7 - 5.5	0.0	0.0 - 0.0	12.6	4.4 - 27.0	0.97
3140104	8247	SWEETWATER CR	154.1	21.6	7 - 47	0.0	0.0 - 0.0	8.1	2.8 - 17.0	0.2	0.1 - 0.6	1.8	0.6 - 4.7	0.0	0.0 - 0.0	11.5	4.3 - 29.9	0.92
3140104	8248	BIG JUNIPER CR	115.3	23.9	8 - 62	0.0	0.0 - 0.0	7.1	2.1 - 17.2	0.4	0.1 - 1.3	3.7	1.2 - 10.5	0.0	0.0 - 0.0	12.7	4.3 - 33.1	0.92
3140104	8249	BIG COLDWATER	23.3	69.7	28 - 148	0.0	0.0 - 0.0	50.9	19.8 - 111.1	0.4	0.2 - 1.3	6.0	2.1 - 18.3	0.0	0.0 - 0.0	12.4	5.0 - 30.5	0.98
3140104	8250	BIG COLDWATER	84.6	25.7	9 - 62	0.0	0.0 - 0.0	8.4	2.7 - 19.5	0.5	0.1 - 1.4	4.7	1.4 - 12.9	0.0	0.0 - 0.0	12.2	3.8 - 34.0	0.96
3140104	8251	BIG COLDWATER	79.3	25.9	10 - 61	0.0	0.0 - 0.0	7.5	2.7 - 17.5	0.6	0.2 - 1.8	3.8	1.3 - 9.9	0.0	0.0 - 0.0	13.9	5.4 - 37.2	0.91
3140104	8252	DIXON CR	83.0	25.1	9 - 55	0.0	0.0 - 0.0	7.3	2.3 - 16.9	0.3	0.1 - 1.0	4.1	1.1 - 11.3	0.0	0.0 - 0.0	13.4	4.7 - 31.3	0.91
3140104	8253	BIG COLDWATER	68.7	53.9	19 - 116	0.0	0.0 - 0.0	9.0	3.0 - 18.7	2.1	0.7 - 6.0	31.2	10.9 - 80.1	0.0	0.0 - 0.0	11.6	4.4 - 23.4	0.96
3140104	8254	JUNIPER CR	46.9	42.8	12 - 107	0.0	0.0 - 0.0	10.0	2.9 - 25.4	1.3	0.4 - 4.3	19.7	5.6 - 54.4	0.0	0.0 - 0.0	11.8	4.0 - 31.2	0.93
3140104	8255	BIG COLDWATER	163.7	50.6	19 - 103	0.0	0.0 - 0.0	12.0	4.3 - 24.8	1.8	0.6 - 5.2	26.2	9.9 - 70.1	0.0	0.0 - 0.0	10.6	4.0 - 23.3	0.93
3140104	8256	CLEAR CR	66.5	47.6	17 - 118	0.0	0.0 - 0.0	21.4	7.6 - 47.9	1.1	0.3 - 3.4	15.3	4.9 - 44.1	0.0	0.0 - 0.0	9.7	3.9 - 24.6	0.99
3140104	8257	POND CR	246.0	50.0	20 - 130	0.0	0.0 - 0.0	27.2	9.5 - 70.7	1.0	0.4 - 2.6	12.3	4.1 - 39.2	0.0	0.0 - 0.0	9.5	3.7 - 22.1	1.00
3140105	8258	E BAY R	65.9	18.4	6 - 46	0.0	0.0 - 0.0	9.8	3.0 - 23.1	0.1	0.0 - 0.4	0.1	0.0 - 0.2	0.0	0.0 - 0.0	8.5	2.8 - 21.5	1.00
3140105	8259	TURTLE CR	108.3	29.2	11 - 81	3.6	1.3 - 9.8	18.4	7.0 - 52.2	0.3	0.1 - 0.9	0.4	0.2 - 1.4	0.0	0.0 - 0.0	6.5	2.4 - 20.0	0.98
3140105	8260	LIVEOAT CR	112.8	15.0	5 - 38	0.0	0.0 - 0.0	8.5	2.7 - 24.6	0.3	0.1 - 0.9	0.0	0.0 - 0.0	0.0	0.0 - 0.0	6.2	2.2 - 15.9	0.98
3140105	8261	CARPENTER CR	48.9	153.3	56 - 363	0.0	0.0 - 0.0	148.0	53.7 - 349.7	0.0	0.0 - 0.1	0.1	0.0 - 0.3	0.0	0.0 - 0.0	5.1	2.0 - 13.1	1.00
3140106	8262	PERDIDO R	19.9	21.7	9 - 49	0.0	0.0 - 0.0	2.4	0.8 - 5.0	0.2	0.1 - 0.6	0.0	0.0 - 0.0	0.0	0.0 - 0.0	19.0	7.9 - 42.5	1.00
3140106	8263	PERDIDO R	24.5	32.3	11 - 65	0.0	0.0 - 0.0	6.9	2.2 - 14.6	1.3	0.4 - 3.0	7.2	2.7 - 16.6	0.0	0.0 - 0.0	16.9	6.0 - 45.7	1.00
3140106	8264	PERDIDO R	278.3	43.5	14 - 92	0.0	0.0 - 0.0	13.7	4.5 - 26.6	2.2	0.6 - 6.1	14.0	4.3 - 36.2	0.0	0.0 - 0.0	13.5	4.4 - 29.6	0.99
3140106	8265	*A	69.2	51.9	15 - 96	0.0	0.0 - 0.0	11.7	3.5 - 23.2	3.3	1.0 - 7.7	24.7	6.7 - 58.5	0.0	0.0 - 0.0	12.2	3.7 - 27.3	0.97
3140106	8266	PERDIDO R	41.8	23.6	7 - 47	0.0	0.0 - 0.0	5.8	1.6 - 12.0	1.0	0.3 - 2.8	2.9	0.9 - 8.2	0.0	0.0 - 0.0	14.0	4.4 - 33.2	0.97
3140106	8267	BRUSHY CR	131.8	35.5	14 - 73	0.0	0.0 - 0.0	10.0	3.8 - 23.0	2.0	0.6 - 6.2	10.9	3.7 - 28.0	0.0	0.0 - 0.0	12.6	4.6 - 29.4	0.95

8-digit HUC	ID	Name	Area	Catchment Yield kg/km^2	Catchment Yield 90% CI	Point sources kg/km^2	Point sources 90% CI	Developed Land kg/km^2	Developed Land 90% CI	Manure kg/km^2	Manure 90% CI	Agricultural Land kg/km^2	Agricultural Land 90% CI	Phosphate Mines kg/km^2	Phosphate Mines 90% CI	Soil parent rock kg/km^2	Soil parent rock 90% CI	Frac
3140106	8268	PERDIDO R	84.7	20.0	7 - 46	0.0	0.0 - 0.0	4.0	1.4 - 8.0	0.5	0.2 - 1.4	1.2	0.4 - 3.2	0.0	0.0 - 0.0	14.3	4.9 - 40.1	0.95
3140106	8269	PERDIDO R	184.9	38.2	14 - 94	0.0	0.0 - 0.0	6.5	2.4 - 16.7	2.1	0.7 - 6.9	16.8	5.2 - 49.9	0.0	0.0 - 0.0	12.8	5.6 - 31.4	0.93
3140106	8270	DYAS CR	23.3	25.8	10 - 56	0.0	0.0 - 0.0	3.1	1.0 - 6.9	1.3	0.4 - 3.7	6.1	2.2 - 15.5	0.0	0.0 - 0.0	15.3	6.3 - 38.1	0.93
3140106	8271	DYAS CR	25.3	20.6	7 - 45	0.0	0.0 - 0.0	3.5	1.2 - 7.5	0.4	0.1 - 1.4	1.3	0.4 - 3.5	0.0	0.0 - 0.0	15.4	5.6 - 39.9	0.91
3140106	8272	BRUSHY CR	83.4	30.4	10 - 71	0.0	0.0 - 0.0	10.1	3.4 - 21.9	1.1	0.4 - 3.3	6.0	2.0 - 18.0	0.0	0.0 - 0.0	13.2	4.7 - 32.4	0.88
3140106	8273	DYAS CR	70.4	21.3	7 - 39	0.0	0.0 - 0.0	5.7	1.5 - 11.2	0.5	0.1 - 1.4	3.0	0.9 - 7.8	0.0	0.0 - 0.0	12.1	4.2 - 25.7	0.88
3140106	8274	MCCURTIN CR	50.3	32.6	11 - 69	0.0	0.0 - 0.0	14.6	5.0 - 33.5	0.7	0.2 - 2.3	4.4	1.4 - 9.7	0.0	0.0 - 0.0	12.9	4.4 - 32.0	0.91
3140106	8275	STYX R	76.9	45.1	15 - 101	0.0	0.0 - 0.0	8.5	2.7 - 18.0	2.6	0.6 - 7.6	18.7	5.6 - 44.3	0.0	0.0 - 0.0	15.3	5.2 - 38.2	0.99
3140106	8276	BELLEFONTAINE (	71.5	25.2	9 - 73	0.0	0.0 - 0.0	4.2	1.4 - 11.5	1.2	0.4 - 4.2	5.6	1.7 - 21.1	0.0	0.0 - 0.0	14.1	4.6 - 44.0	0.99
3140106	8277	STYX R	150.7	41.9	13 - 94	0.0	0.0 - 0.0	9.0	3.1 - 19.8	2.4	0.7 - 6.7	15.9	4.5 - 47.1	0.0	0.0 - 0.0	14.5	4.9 - 36.4	0.99
3140106	8278	STYX R	133.1	43.7	15 - 96	15.9	5.3 - 37.9	10.3	3.7 - 21.3	0.8	0.3 - 2.5	2.8	0.9 - 6.9	0.0	0.0 - 0.0	14.0	4.9 - 32.2	0.97
3140106	8279	HOLLINGER CR	241.9	30.9	12 - 61	0.0	0.0 - 0.0	9.4	3.2 - 19.5	1.2	0.4 - 2.9	6.6	2.4 - 14.9	0.0	0.0 - 0.0	13.7	4.9 - 28.8	0.97
3140106	8280	BLACKWATER CR	354.7	80.1	27 - 151	2.7	0.9 - 5.3	11.3	3.7 - 19.1	5.7	1.5 - 15.4	44.5	14.8 - 102.6	0.0	0.0 - 0.0	16.0	5.5 - 31.7	1.00
3140107	8281	BAYOU MARCUS (	79.2	141.9	43 - 256	10.0	3.3 - 20.4	124.6	36.6 - 230.6	0.1	0.0 - 0.3	0.5	0.1 - 1.2	0.0	0.0 - 0.0	6.7	2.2 - 13.9	1.00
3140107	8282	ELEVENMILE CR	129.7	235.1	92 - 512	147.6	57.5 - 319.4	69.7	25.5 - 154.5	1.1	0.3 - 2.6	6.9	2.4 - 18.2	0.0	0.0 - 0.0	9.8	4.1 - 22.4	1.00
3140107	8283	WOLF CR	69.8	119.4	40 - 295	19.2	6.2 - 45.8	35.0	12.1 - 75.7	5.6	1.8 - 13.2	41.9	13.0 - 120.9	0.0	0.0 - 0.0	17.7	5.8 - 42.0	1.00
3140107	8284	INTRACOASTAL W	44.7	166.4	54 - 364	101.7	33.4 - 246.8	39.3	12.4 - 77.6	1.4	0.4 - 3.8	9.1	2.7 - 29.9	0.0	0.0 - 0.0	15.0	4.7 - 30.5	1.00
3140201	8285	CHOCTAWHATCH	1.1	53.8	17 - 128	0.0	0.0 - 0.0	18.4	5.6 - 42.2	6.7	2.1 - 20.0	10.6	3.5 - 27.8	0.0	0.0 - 0.0	18.1	5.4 - 47.7	0.95
3140201	8286	CHOCTAWHATCH	118.5	51.0	21 - 118	0.0	0.0 - 0.0	7.2	2.7 - 16.7	11.6	3.8 - 39.4	19.6	7.3 - 64.5	0.0	0.0 - 0.0	12.6	5.1 - 31.1	0.95
3140201	8287	BARNES CR	41.1	54.9	19 - 101	0.0	0.0 - 0.0	7.6	2.4 - 15.0	14.2	4.3 - 36.1	23.9	7.0 - 62.0	0.0	0.0 - 0.0	9.1	2.7 - 19.5	0.92
3140201	8288	CHOCTAWHATCH	8.5	43.0	14 - 111	0.0	0.0 - 0.0	4.9	1.5 - 13.4	9.1	2.6 - 27.7	15.5	5.1 - 46.5	0.0	0.0 - 0.0	13.5	4.3 - 38.2	0.92
3140201	8289	CHOCTAWHATCH	17.5	38.6	12 - 92	0.0	0.0 - 0.0	6.3	1.9 - 13.4	7.1	2.2 - 17.4	11.7	3.5 - 28.0	0.0	0.0 - 0.0	13.4	3.7 - 35.3	0.92
3140201	8290	CHOCTAWHATCH	6.5	52.1	17 - 117	0.0	0.0 - 0.0	7.6	2.4 - 17.4	12.7	3.7 - 34.8	21.1	7.4 - 54.2	0.0	0.0 - 0.0	10.7	3.5 - 30.4	0.91
3140201	8291	HURRICANE CR	141.2	58.6	20 - 97	0.0	0.0 - 0.0	10.1	3.5 - 17.7	13.7	3.8 - 30.7	23.3	7.2 - 48.0	0.0	0.0 - 0.0	11.5	3.7 - 25.4	0.91
3140201	8292	CHOCTAWHATCH	9.8	27.9	10 - 60	0.0	0.0 - 0.0	2.2	0.7 - 4.8	4.9	1.5 - 13.7	9.8	3.5 - 29.2	0.0	0.0 - 0.0	11.0	3.6 - 27.3	0.91
3140201	8293	PATES CR	49.4	55.9	20 - 107	0.0	0.0 - 0.0	6.5	2.2 - 12.5	10.3	3.3 - 27.0	27.2	8.9 - 65.6	0.0	0.0 - 0.0	11.8	4.2 - 25.1	0.90
3140201	8294	CHOCTAWHATCH	30.1	28.9	10 - 58	0.0	0.0 - 0.0	6.7	2.4 - 13.7	4.0	1.3 - 11.9	10.1	3.0 - 23.8	0.0	0.0 - 0.0	8.2	3.1 - 19.7	0.90
3140201	8295	LITTLE CHOCTAW	138.9	44.4	14 - 111	0.0	0.0 - 0.0	9.1	2.7 - 24.4	6.4	2.0 - 18.5	20.3	6.3 - 67.2	0.0	0.0 - 0.0	8.6	2.9 - 23.8	0.90
3140201	8296	BEAR CR	65.5	63.1	24 - 122	0.0	0.0 - 0.0	7.8	2.8 - 16.3	13.4	4.7 - 39.8	30.0	10.9 - 74.3	0.0	0.0 - 0.0	11.9	4.8 - 29.2	0.79
3140201	8297	LITTLE CHOCTAW	37.3	147.5	49 - 328	95.3	31.6 - 218.0	16.9	5.7 - 38.0	7.8	2.6 - 20.6	18.7	5.6 - 47.9	0.0	0.0 - 0.0	8.8	3.1 - 21.9	0.79
3140201	8298	NEWTON CR	100.1	103.2	35 - 263	33.5	12.9 - 83.7	37.1	11.3 - 83.6	5.9	1.8 - 17.2	16.3	5.8 - 49.2	0.0	0.0 - 0.0	10.3	3.3 - 23.8	0.75
3140201	8299	LITTLE CHOCTAW	67.9	76.7	26 - 150	0.0	0.0 - 0.0	53.0	16.9 - 112.3	3.6	0.9 - 8.8	11.7	3.7 - 28.5	0.0	0.0 - 0.0	8.4	2.9 - 19.7	0.75
3140201	8300	CHOCTAWHATCH	115.6	36.7	14 - 77	0.0	0.0 - 0.0	19.8	6.9 - 43.7	3.3	1.1 - 9.4	7.0	2.7 - 20.7	0.0	0.0 - 0.0	6.6	2.5 - 16.2	0.90
3140201	8301	CHOCTAWHATCH	21.9	32.9	10 - 68	0.0	0.0 - 0.0	9.8	3.0 - 20.8	5.5	1.5 - 15.2	12.1	3.9 - 29.5	0.0	0.0 - 0.0	5.5	1.8 - 12.3	0.87
3140201	8302	CHOCTAWHATCH	89.5	38.3	12 - 80	0.0	0.0 - 0.0	9.4	2.4 - 20.5	6.9	1.8 - 20.2	14.8	4.2 - 36.2	0.0	0.0 - 0.0	7.3	2.4 - 16.4	0.87
3140201	8303	BLACKWOOD CR	116.5	49.5	20 - 103	0.0	0.0 - 0.0	11.0	4.5 - 27.5	5.9	2.0 - 16.1	23.2	8.2 - 62.8	0.0	0.0 - 0.0	9.4	3.5 - 25.8	0.85
3140201	8304	CHOCTAWHATCH	139.2	29.4	10 - 71	0.0	0.0 - 0.0	5.4	1.7 - 12.3	4.2	1.3 - 14.2	12.9	4.2 - 37.6	0.0	0.0 - 0.0	6.9	2.3 - 18.5	0.85
3140201	8305	POOR CR	52.8	25.7	11 - 61	0.0	0.0 - 0.0	7.1	2.8 - 15.6	1.7	0.5 - 4.8	8.7	3.4 - 24.5	0.0	0.0 - 0.0	8.1	3.2 - 22.1	0.81
3140201	8306	CHOCTAWHATCH	55.1	20.1	7 - 53	0.0	0.0 - 0.0	3.0	1.1 - 6.5	2.2	0.7 - 6.2	7.0	2.1 - 21.0	0.0	0.0 - 0.0	8.0	2.7 - 21.0	0.81

8-digit HUC	ID	Name	Area	Catchment Yield		Point sources		Developed Land		Manure		Agricultural Land		Phosphate Mines		Soil parent rock		Frac
				kg/km^2	90% CI	kg/km^2	90% CI	kg/km^2	90% CI	kg/km^2	90% CI	kg/km^2	90% CI	kg/km^2	90% CI	kg/km^2	90% CI	
3140201	8307	CHOCTAWHATCH	125.3	17.9	6 - 44	0.0	0.0 - 0.0	5.2	1.6 - 13.2	1.1	0.3 - 2.7	5.4	1.8 - 14.2	0.0	0.0 - 0.0	6.2	2.1 - 14.2	0.79
3140201	8308	PINEY WOODS CR	51.7	18.9	7 - 38	0.0	0.0 - 0.0	5.3	1.7 - 12.9	1.9	0.6 - 4.7	6.2	1.9 - 14.8	0.0	0.0 - 0.0	5.5	2.0 - 13.2	0.74
3140201	8309	CHOCTAWHATCH	164.9	26.7	6 - 61	0.0	0.0 - 0.0	5.0	1.2 - 11.1	4.1	1.0 - 10.3	11.4	2.6 - 31.9	0.0	0.0 - 0.0	6.2	1.6 - 15.5	0.74
3140201	8310	PANTHER CR	26.0	14.5	5 - 28	0.0	0.0 - 0.0	4.5	1.6 - 9.3	0.9	0.3 - 2.0	2.9	1.1 - 8.4	0.0	0.0 - 0.0	6.2	2.1 - 12.9	0.79
3140201	8311	CHOCTAWHATCH	68.5	22.0	7 - 49	0.0	0.0 - 0.0	5.6	1.6 - 10.8	3.3	1.0 - 9.6	7.1	2.0 - 17.7	0.0	0.0 - 0.0	6.0	2.2 - 15.1	0.87
3140201	8312	CHOCTAWHATCH	93.9	14.9	4 - 31	0.0	0.0 - 0.0	4.2	1.2 - 9.6	1.7	0.5 - 4.6	3.6	1.0 - 9.8	0.0	0.0 - 0.0	5.5	1.7 - 14.2	0.84
3140201	8313	BEAR CR	91.2	16.0	6 - 35	0.0	0.0 - 0.0	4.0	1.5 - 8.8	2.0	0.8 - 5.0	5.1	1.8 - 13.9	0.0	0.0 - 0.0	5.0	2.1 - 10.4	0.80
3140201	8314	CHOCTAWHATCH	27.3	13.7	6 - 35	0.0	0.0 - 0.0	3.1	1.1 - 8.0	2.0	0.7 - 6.2	4.2	1.6 - 12.2	0.0	0.0 - 0.0	4.5	2.0 - 12.8	0.80
3140201	8315	CHOCTAWHATCH	54.9	23.1	7 - 49	0.0	0.0 - 0.0	6.1	1.9 - 13.3	3.4	1.1 - 10.0	8.8	2.9 - 27.9	0.0	0.0 - 0.0	4.8	1.7 - 12.4	0.77
3140201	8316	LINDSEY CR	104.7	22.1	8 - 51	0.0	0.0 - 0.0	5.4	1.7 - 12.1	2.9	0.9 - 8.9	7.7	2.5 - 21.3	0.0	0.0 - 0.0	6.0	2.2 - 13.7	0.73
3140201	8317	CHOCTAWHATCH	86.6	21.8	8 - 43	0.0	0.0 - 0.0	4.8	1.5 - 10.1	2.8	0.9 - 7.4	7.1	2.3 - 20.0	0.0	0.0 - 0.0	7.1	2.4 - 15.2	0.73
3140201	8318	SKIES CR	94.1	23.3	8 - 42	0.0	0.0 - 0.0	5.6	1.8 - 10.3	3.7	1.0 - 10.6	9.6	3.3 - 21.8	0.0	0.0 - 0.0	4.5	1.4 - 9.8	0.77
3140201	8319	JUDY CR	86.0	19.0	6 - 40	0.0	0.0 - 0.0	8.2	2.6 - 15.2	2.0	0.6 - 5.0	4.0	1.3 - 10.2	0.0	0.0 - 0.0	4.9	1.4 - 11.7	0.84
3140201	8320	LITTLE JUDY CR	78.0	17.4	5 - 37	0.0	0.0 - 0.0	4.3	1.2 - 9.8	2.5	0.8 - 9.0	6.2	2.0 - 17.6	0.0	0.0 - 0.0	4.3	1.3 - 10.9	0.76
3140201	8321	JUDY CR	133.2	14.9	5 - 32	0.0	0.0 - 0.0	4.5	1.4 - 9.3	1.9	0.6 - 5.3	4.4	1.4 - 11.0	0.0	0.0 - 0.0	4.0	1.5 - 9.1	0.76
3140201	8322	HURRICANE CR	72.7	59.1	22 - 145	21.7	6.6 - 52.5	23.4	8.2 - 55.4	2.1	0.6 - 6.7	4.5	1.4 - 11.3	0.0	0.0 - 0.0	7.3	2.5 - 18.3	0.87
3140201	8323	CLAYBANK CR	146.7	53.7	15 - 121	5.1	1.7 - 11.4	22.0	5.7 - 51.6	6.8	1.7 - 17.7	11.9	3.6 - 32.0	0.0	0.0 - 0.0	7.8	2.6 - 18.9	0.91
3140201	8324	CLAYBANK CR	9.9	38.1	14 - 68	0.0	0.0 - 0.0	30.4	11.0 - 58.7	0.6	0.2 - 1.3	1.3	0.5 - 3.3	0.0	0.0 - 0.0	5.7	2.0 - 12.2	0.81
3140201	8325	CLAYBANK CR	51.0	8.1	3 - 14	0.0	0.0 - 0.0	4.5	1.3 - 8.4	0.3	0.1 - 0.8	0.7	0.2 - 1.6	0.0	0.0 - 0.0	2.5	0.9 - 5.3	0.79
3140201	8326	BEAR CR	93.0	22.8	8 - 46	0.0	0.0 - 0.0	10.1	3.7 - 22.4	2.5	0.8 - 6.2	5.4	2.0 - 13.6	0.0	0.0 - 0.0	4.8	1.9 - 11.6	0.31
3140201	8327	CLAYBANK CR	94.0	17.8	6 - 42	0.0	0.0 - 0.0	5.5	1.6 - 13.3	2.5	0.7 - 7.1	5.1	1.5 - 11.7	0.0	0.0 - 0.0	4.7	1.7 - 11.5	0.31
3140201	8328	STEEP HEAD CR	56.9	16.3	5 - 36	0.0	0.0 - 0.0	7.4	2.2 - 16.8	1.2	0.4 - 3.6	2.0	0.7 - 5.8	0.0	0.0 - 0.0	5.7	2.0 - 14.4	0.79
3140201	8329	BOWLES CR	76.3	20.1	6 - 53	0.0	0.0 - 0.0	7.2	2.0 - 18.3	2.7	0.8 - 9.2	3.2	0.9 - 10.8	0.0	0.0 - 0.0	7.0	1.9 - 19.5	0.68
3140201	8330	STEEP HEAD CR	33.5	29.6	10 - 78	0.0	0.0 - 0.0	7.6	2.6 - 17.9	6.9	2.1 - 19.3	8.1	2.5 - 26.6	0.0	0.0 - 0.0	7.0	2.6 - 17.7	0.68
3140201	8331	*A	52.7	72.8	24 - 160	21.8	7.4 - 59.9	35.2	10.8 - 76.1	4.3	1.3 - 11.6	5.5	1.7 - 16.0	0.0	0.0 - 0.0	6.0	2.0 - 16.3	0.81
3140201	8332	WILKESON CR	21.2	51.4	15 - 107	0.0	0.0 - 0.0	10.7	2.9 - 25.2	12.0	3.4 - 35.5	19.1	6.2 - 51.6	0.0	0.0 - 0.0	9.6	2.8 - 23.1	0.92
3140201	8333	WILKESON CR	27.5	52.7	16 - 114	0.0	0.0 - 0.0	7.7	2.6 - 17.2	16.3	4.2 - 51.4	20.5	7.1 - 46.4	0.0	0.0 - 0.0	8.2	3.0 - 19.8	0.86
3140201	8334	BELL CR	45.8	49.7	17 - 81	0.0	0.0 - 0.0	6.4	2.1 - 12.2	16.2	5.2 - 36.4	18.6	7.0 - 40.1	0.0	0.0 - 0.0	8.5	2.8 - 15.7	0.86
3140201	8335	DOUBLE BRIDGES	57.7	58.8	19 - 107	0.0	0.0 - 0.0	11.8	3.7 - 23.6	12.3	3.8 - 34.5	21.1	8.2 - 48.9	0.0	0.0 - 0.0	13.6	4.8 - 31.2	0.95
3140201	8336	BEAVERDAM CR	67.1	56.6	21 - 110	0.0	0.0 - 0.0	7.9	2.8 - 16.9	14.5	4.4 - 37.1	21.2	7.3 - 62.5	0.0	0.0 - 0.0	13.0	4.4 - 27.5	0.93
3140201	8337	DOUBLE BRIDGES	23.8	60.4	18 - 141	0.0	0.0 - 0.0	7.4	2.3 - 15.6	14.8	4.2 - 43.8	25.1	7.9 - 69.0	0.0	0.0 - 0.0	13.1	4.5 - 29.9	0.93
3140201	8338	DOUBLE BRIDGES	82.0	157.4	54 - 412	104.6	34.1 - 254.3	6.7	2.2 - 16.2	14.5	4.2 - 45.2	18.8	5.0 - 52.1	0.0	0.0 - 0.0	12.8	4.6 - 32.8	0.92
3140201	8339	DOUBLE BRIDGES	102.6	65.0	25 - 139	10.8	4.1 - 24.1	15.9	6.1 - 37.4	13.0	4.3 - 38.2	15.3	6.5 - 46.2	0.0	0.0 - 0.0	10.1	3.7 - 25.4	0.88
3140201	8340	LITTLE DOUBLE B	62.2	48.6	16 - 97	0.0	0.0 - 0.0	6.8	2.3 - 15.3	14.9	4.3 - 44.8	17.2	5.5 - 43.2	0.0	0.0 - 0.0	9.7	3.7 - 23.2	0.88
3140201	8341	TIGHT EYE CR	111.2	58.7	18 - 139	0.0	0.0 - 0.0	7.7	2.1 - 15.8	17.1	5.1 - 54.0	21.8	6.0 - 53.8	0.0	0.0 - 0.0	12.0	3.6 - 35.0	0.92
3140202	8342	PEA R	48.1	60.8	19 - 115	11.9	3.9 - 25.6	20.0	6.1 - 45.7	5.8	1.6 - 16.7	10.2	3.1 - 27.1	0.0	0.0 - 0.0	12.9	4.7 - 30.6	0.95
3140202	8343	SANDY CR	68.4	53.7	17 - 95	0.0	0.0 - 0.0	8.9	2.6 - 17.3	12.0	2.9 - 30.6	20.6	6.4 - 45.8	0.0	0.0 - 0.0	12.2	3.9 - 24.3	0.94
3140202	8344	PEA R	252.5	35.7	13 - 62	0.0	0.0 - 0.0	5.4	2.1 - 9.3	4.5	1.5 - 11.6	9.7	3.7 - 24.0	0.0	0.0 - 0.0	16.1	6.5 - 33.7	0.94
3140202	8345	PEA R	109.4	64.4	20 - 135	0.0	0.0 - 0.0	8.9	2.8 - 20.2	14.7	4.5 - 50.5	25.0	8.3 - 76.8	0.0	0.0 - 0.0	15.8	5.3 - 41.6	0.93

8-digit HUC	ID	Name	Area	Catchment Yield		Point sources		Developed Land		Manure		Agricultural Land		Phosphate Mines		Soil parent rock		Frac
				kg/km^2	90% CI	kg/km^2	90% CI	kg/km^2	90% CI	kg/km^2	90% CI	kg/km^2	90% CI	kg/km^2	90% CI	kg/km^2	90% CI	
3140202	8346	HOLLY MILL CR	59.5	59.7	19 - 129	0.0	0.0 - 0.0	7.2	2.3 - 16.9	19.0	6.4 - 52.2	22.5	7.0 - 66.0	0.0	0.0 - 0.0	10.9	3.6 - 27.4	0.91
3140202	8347	PEA R	113.1	74.7	23 - 145	0.0	0.0 - 0.0	10.7	3.1 - 20.4	21.0	5.5 - 54.3	27.4	8.1 - 77.5	0.0	0.0 - 0.0	15.7	5.6 - 40.2	0.91
3140202	8348	PEA R	1.6	108.3	38 - 196	0.0	0.0 - 0.0	0.0	0.0 - 0.0	38.7	11.8 - 89.3	46.0	16.9 - 93.1	0.0	0.0 - 0.0	23.7	8.1 - 47.1	0.90
3140202	8349	PEA R	33.0	55.9	17 - 115	0.0	0.0 - 0.0	6.5	1.9 - 13.2	17.5	5.2 - 52.9	20.2	5.7 - 46.8	0.0	0.0 - 0.0	11.6	3.7 - 25.3	0.90
3140202	8350	BUCKS MILL CR	80.7	31.4	11 - 60	0.0	0.0 - 0.0	7.0	2.5 - 15.6	7.3	2.2 - 20.0	8.5	2.9 - 21.6	0.0	0.0 - 0.0	8.6	3.0 - 19.2	0.89
3140202	8351	PEA R	63.6	29.0	10 - 68	0.0	0.0 - 0.0	7.0	2.4 - 15.6	6.6	2.1 - 18.6	7.7	2.4 - 21.0	0.0	0.0 - 0.0	7.8	3.0 - 19.2	0.89
3140202	8352	PEA R	3.7	17.6	6 - 31	0.0	0.0 - 0.0	11.4	3.7 - 20.8	0.2	0.1 - 0.6	0.3	0.1 - 0.9	0.0	0.0 - 0.0	5.7	2.0 - 12.3	0.88
3140202	8353	PEA R	95.4	28.2	10 - 52	0.0	0.0 - 0.0	8.0	2.9 - 16.1	5.6	1.9 - 14.1	6.6	2.6 - 16.1	0.0	0.0 - 0.0	7.9	2.9 - 18.5	0.88
3140202	8354	PEA R	152.9	27.8	8 - 59	0.0	0.0 - 0.0	5.8	1.8 - 13.6	6.4	1.9 - 19.1	7.6	1.9 - 20.7	0.0	0.0 - 0.0	7.9	2.4 - 20.3	0.86
3140202	8355	PEA R	59.5	31.9	10 - 66	0.0	0.0 - 0.0	6.8	2.0 - 13.7	8.1	2.0 - 21.7	10.6	3.1 - 26.9	0.0	0.0 - 0.0	6.3	2.2 - 14.0	0.84
3140202	8356	PEA R	87.3	27.2	8 - 51	0.0	0.0 - 0.0	5.0	1.5 - 10.9	5.3	1.5 - 12.5	9.3	3.1 - 25.9	0.0	0.0 - 0.0	7.5	2.2 - 17.5	0.82
3140202	8358	BIG CR	79.6	22.8	7 - 51	0.0	0.0 - 0.0	5.6	1.8 - 13.2	2.9	0.9 - 7.3	7.7	2.3 - 20.4	0.0	0.0 - 0.0	6.5	2.3 - 15.9	0.80
3140202	8359	PEA R	10.4	33.8	11 - 75	0.0	0.0 - 0.0	4.1	1.3 - 9.3	6.2	1.6 - 18.2	9.7	3.1 - 31.5	0.0	0.0 - 0.0	13.8	4.5 - 34.2	0.80
3140202	8360	PEA R	30.9	37.6	13 - 84	0.0	0.0 - 0.0	3.2	1.1 - 7.7	8.4	2.5 - 28.4	13.4	4.6 - 38.1	0.0	0.0 - 0.0	12.6	4.2 - 33.6	0.79
3140202	8361	PEA CR	144.9	24.6	8 - 59	0.0	0.0 - 0.0	4.4	1.4 - 11.1	3.4	1.0 - 9.6	8.8	2.6 - 22.3	0.0	0.0 - 0.0	8.1	2.4 - 20.9	0.78
3140202	8362	PEA CR	74.8	21.0	8 - 44	0.0	0.0 - 0.0	6.7	2.3 - 14.3	2.2	0.7 - 6.0	5.9	2.0 - 14.7	0.0	0.0 - 0.0	6.3	2.5 - 14.6	0.70
3140202	8363	STINKING CR	51.6	15.1	5 - 38	0.0	0.0 - 0.0	3.6	1.3 - 8.3	1.4	0.4 - 4.0	3.6	1.2 - 10.3	0.0	0.0 - 0.0	6.5	2.3 - 15.8	0.70
3140202	8364	PEA R	54.1	23.5	9 - 42	0.0	0.0 - 0.0	5.1	1.8 - 9.8	3.7	1.2 - 9.8	5.8	2.2 - 13.9	0.0	0.0 - 0.0	8.9	3.3 - 21.0	0.78
3140202	8365	PEA R	79.1	13.9	5 - 26	0.0	0.0 - 0.0	3.4	1.0 - 7.1	0.6	0.2 - 1.8	2.1	0.7 - 4.7	0.0	0.0 - 0.0	7.8	2.3 - 17.0	0.75
3140202	8366	PEA R	4.4	27.3	10 - 60	0.0	0.0 - 0.0	3.0	1.1 - 6.9	1.8	0.6 - 5.1	3.8	1.4 - 9.3	0.0	0.0 - 0.0	18.7	7.0 - 44.3	0.73
3140202	8367	PEA R	2.3	24.5	8 - 72	0.0	0.0 - 0.0	1.6	0.4 - 4.0	1.0	0.3 - 3.6	3.3	1.0 - 8.9	0.0	0.0 - 0.0	18.6	6.7 - 60.9	0.72
3140202	8368	*A	29.4	20.3	7 - 43	0.0	0.0 - 0.0	3.9	1.2 - 8.6	1.2	0.3 - 3.4	5.7	1.5 - 13.9	0.0	0.0 - 0.0	9.6	3.4 - 21.9	0.71
3140202	8369	LITTLE INDIAN CR	67.5	22.6	8 - 46	0.0	0.0 - 0.0	4.2	1.3 - 9.5	1.6	0.5 - 4.3	7.9	2.6 - 20.6	0.0	0.0 - 0.0	8.9	3.3 - 18.9	0.72
3140202	8370	BIG SANDY CR	47.0	20.0	6 - 37	0.0	0.0 - 0.0	5.6	1.4 - 10.6	0.9	0.2 - 2.1	4.2	1.2 - 9.2	0.0	0.0 - 0.0	9.3	2.7 - 18.1	0.73
3140202	8371	PEROTE CR	65.7	14.6	5 - 34	0.0	0.0 - 0.0	3.9	1.2 - 9.6	0.6	0.2 - 1.7	2.5	0.8 - 6.1	0.0	0.0 - 0.0	7.6	2.7 - 21.3	0.75
3140202	8372	BUCKHORN CR	121.8	23.2	8 - 45	0.0	0.0 - 0.0	5.4	1.9 - 11.2	4.4	1.4 - 11.9	6.7	2.1 - 16.8	0.0	0.0 - 0.0	6.7	2.4 - 13.8	0.79
3140202	8373	RICHLAND CR	131.4	29.5	12 - 61	0.0	0.0 - 0.0	6.9	2.1 - 14.3	6.0	2.2 - 14.8	8.7	3.1 - 25.8	0.0	0.0 - 0.0	7.9	3.4 - 17.8	0.80
3140202	8374	BOWDEN MILL CR	49.1	21.1	7 - 43	0.0	0.0 - 0.0	5.4	1.8 - 10.3	4.6	1.4 - 11.6	6.6	1.9 - 15.8	0.0	0.0 - 0.0	4.5	1.6 - 10.8	0.82
3140202	8375	*B	57.9	32.0	9 - 56	0.0	0.0 - 0.0	6.7	2.1 - 13.8	9.0	2.4 - 21.0	11.9	3.1 - 31.2	0.0	0.0 - 0.0	4.4	1.4 - 9.5	0.84
3140202	8376	*C	14.0	16.5	6 - 37	0.0	0.0 - 0.0	6.8	2.4 - 16.2	1.8	0.5 - 5.3	2.0	0.6 - 5.7	0.0	0.0 - 0.0	6.0	2.2 - 13.8	0.86
3140202	8377	BIG CR	9.2	53.8	23 - 121	0.0	0.0 - 0.0	21.0	8.3 - 47.1	11.1	4.0 - 33.5	11.5	4.6 - 30.1	0.0	0.0 - 0.0	10.1	4.1 - 23.1	0.88
3140202	8378	BIG CR	11.5	25.7	8 - 50	0.0	0.0 - 0.0	5.0	1.7 - 11.3	5.7	1.6 - 15.8	6.2	1.9 - 16.9	0.0	0.0 - 0.0	8.7	2.9 - 21.4	0.87
3140202	8379	WHITEWATER CR	183.3	22.4	8 - 40	0.0	0.0 - 0.0	5.9	2.0 - 12.0	4.7	1.5 - 15.0	5.8	1.7 - 14.2	0.0	0.0 - 0.0	6.0	2.1 - 15.7	0.86
3140202	8380	MIMS CR	45.9	46.0	16 - 129	10.5	3.8 - 29.9	9.7	3.5 - 27.4	8.3	2.4 - 30.7	12.1	4.0 - 38.4	0.0	0.0 - 0.0	5.4	1.8 - 14.8	0.79
3140202	8381	WHITEWATER CR	6.5	34.3	12 - 81	0.0	0.0 - 0.0	5.9	1.9 - 14.1	7.6	2.2 - 21.6	11.1	3.7 - 31.0	0.0	0.0 - 0.0	9.7	4.0 - 23.1	0.79
3140202	8382	WHITEWATER CR	85.5	32.7	11 - 67	0.0	0.0 - 0.0	7.6	2.6 - 18.6	7.1	2.3 - 20.4	10.3	3.5 - 25.7	0.0	0.0 - 0.0	7.7	2.6 - 18.9	0.78
3140202	8383	WALNUT CR	120.0	70.0	22 - 121	37.5	11.4 - 70.9	14.3	4.3 - 25.7	4.7	1.4 - 13.4	6.8	2.3 - 16.6	0.0	0.0 - 0.0	6.8	2.3 - 15.2	0.78
3140202	8384	SILERS CR	17.0	26.4	9 - 48	0.0	0.0 - 0.0	7.3	2.7 - 13.6	5.3	1.7 - 15.1	6.3	2.0 - 18.0	0.0	0.0 - 0.0	7.5	2.7 - 17.7	0.86
3140202	8385	SILERS CR	85.2	25.9	8 - 60	0.0	0.0 - 0.0	4.9	1.8 - 12.2	5.9	1.8 - 15.9	7.7	2.8 - 19.5	0.0	0.0 - 0.0	7.4	2.6 - 16.3	0.84

8-digit HUC	ID	Name	Area	Catchment Yield		Point sources		Developed Land		Manure		Agricultural Land		Phosphate Mines		Soil parent rock		Frac
				kg/km^2	90% CI	kg/km^2	90% CI	kg/km^2	90% CI	kg/km^2	90% CI	kg/km^2	90% CI	kg/km^2	90% CI	kg/km^2	90% CI	
3140202	8386	SILERS CR	104.1	29.5	10 - 77	0.0	0.0 - 0.0	8.8	2.8 - 21.9	5.7	1.7 - 15.1	8.3	2.7 - 25.8	0.0	0.0 - 0.0	6.7	2.4 - 21.2	0.79
3140202	8387	BLUFF CR	29.0	38.3	12 - 88	0.0	0.0 - 0.0	6.3	1.8 - 13.6	10.2	2.8 - 26.4	14.5	4.6 - 39.6	0.0	0.0 - 0.0	7.4	2.7 - 19.2	0.79
3140202	8388	PEA CR	58.6	15.0	5 - 28	0.0	0.0 - 0.0	4.2	1.4 - 8.6	2.1	0.6 - 4.8	2.4	0.8 - 5.7	0.0	0.0 - 0.0	6.3	2.3 - 14.6	0.84
3140202	8389	PEA CR	63.1	15.4	4 - 41	0.0	0.0 - 0.0	4.9	1.3 - 12.2	2.3	0.5 - 6.9	2.6	0.7 - 7.8	0.0	0.0 - 0.0	5.6	1.7 - 17.3	0.87
3140202	8390	BEAVER DAM	80.6	25.2	8 - 64	0.0	0.0 - 0.0	9.3	3.1 - 23.0	4.6	1.4 - 13.8	5.5	1.5 - 14.6	0.0	0.0 - 0.0	5.7	1.9 - 15.2	0.88
3140202	8391	HAYS CR	44.8	52.1	17 - 131	0.0	0.0 - 0.0	8.3	2.6 - 23.2	15.2	4.3 - 51.7	18.0	5.5 - 50.2	0.0	0.0 - 0.0	10.6	3.7 - 29.5	0.90
3140202	8392	PAGES CR	32.4	62.3	20 - 140	0.0	0.0 - 0.0	8.0	2.7 - 19.4	18.1	5.7 - 54.3	24.2	7.4 - 65.0	0.0	0.0 - 0.0	12.0	3.9 - 30.3	0.90
3140202	8393	FLAT CR	16.2	51.0	16 - 136	0.0	0.0 - 0.0	8.4	2.5 - 19.0	10.1	2.6 - 33.9	17.1	4.5 - 59.6	0.0	0.0 - 0.0	15.4	4.7 - 42.0	0.93
3140202	8394	FLAT CR	18.7	55.6	21 - 110	0.0	0.0 - 0.0	6.5	2.4 - 14.3	13.0	4.2 - 30.9	21.9	7.8 - 64.2	0.0	0.0 - 0.0	14.1	5.5 - 32.7	0.92
3140202	8395	FLAT CR	132.6	52.0	16 - 97	0.0	0.0 - 0.0	9.0	2.4 - 19.6	9.8	2.5 - 28.9	17.9	5.9 - 44.8	0.0	0.0 - 0.0	15.3	5.1 - 35.7	0.91
3140202	8396	PANTHER CR	84.3	39.9	11 - 74	0.0	0.0 - 0.0	6.5	1.8 - 12.2	5.0	1.3 - 13.1	10.8	3.3 - 26.1	0.0	0.0 - 0.0	17.6	5.2 - 40.6	0.91
3140202	8397	EIGHTMILE CR	302.4	40.7	15 - 79	0.0	0.0 - 0.0	6.3	2.3 - 12.0	5.7	1.8 - 13.1	13.1	4.7 - 31.0	0.0	0.0 - 0.0	15.7	5.2 - 34.5	0.92
3140202	8398	PEA R	150.9	20.8	6 - 33	0.0	0.0 - 0.0	5.2	1.5 - 10.3	1.4	0.4 - 3.4	6.8	2.1 - 14.8	0.0	0.0 - 0.0	7.4	2.3 - 18.1	0.71
3140203	8399	CHOCTAWHATCH	51.8	6.1	2 - 13	0.0	0.0 - 0.0	0.5	0.2 - 1.0	0.1	0.0 - 0.2	0.0	0.0 - 0.0	0.0	0.0 - 0.0	5.5	2.0 - 14.0	1.00
3140203	8400	PINE LOG CR	16.0	10.4	3 - 22	0.0	0.0 - 0.0	2.5	0.8 - 4.6	0.1	0.0 - 0.2	0.0	0.0 - 0.0	0.0	0.0 - 0.0	7.8	2.7 - 16.2	1.00
3140203	8401	OTTER CR	36.8	18.9	6 - 53	0.0	0.0 - 0.0	7.4	2.3 - 23.7	0.1	0.0 - 0.3	0.0	0.0 - 0.0	0.0	0.0 - 0.0	11.4	4.0 - 33.1	0.98
3140203	8402	PINE LOG CR	90.3	17.7	7 - 38	0.0	0.0 - 0.0	6.8	2.4 - 16.3	0.2	0.1 - 0.6	0.6	0.2 - 1.8	0.0	0.0 - 0.0	10.0	3.9 - 22.0	0.98
3140203	8403	BOTHERATION CR	58.0	18.2	6 - 41	0.0	0.0 - 0.0	5.7	1.7 - 12.7	0.2	0.1 - 0.5	0.0	0.0 - 0.0	0.0	0.0 - 0.0	12.2	3.7 - 28.7	0.91
3140203	8404	BEAR BAY CR	207.3	22.4	8 - 44	0.0	0.0 - 0.0	9.9	3.3 - 22.8	0.5	0.1 - 1.2	0.5	0.2 - 1.1	0.0	0.0 - 0.0	11.5	4.3 - 24.5	0.91
3140203	8405	CHOCTAWHATCH	58.2	10.9	4 - 21	0.0	0.0 - 0.0	3.0	1.1 - 6.3	0.2	0.1 - 0.6	0.6	0.2 - 1.5	0.0	0.0 - 0.0	7.1	2.8 - 13.9	1.00
3140203	8406	CHOCTAWHATCH	19.6	11.4	4 - 22	0.0	0.0 - 0.0	2.7	0.8 - 5.7	0.1	0.0 - 0.3	0.0	0.0 - 0.0	0.0	0.0 - 0.0	8.6	2.8 - 16.2	0.99
3140203	8407	HOLMES CR	314.4	25.1	7 - 44	0.0	0.0 - 0.0	4.3	1.1 - 8.9	1.0	0.2 - 3.0	4.6	1.3 - 10.0	0.0	0.0 - 0.0	15.3	4.6 - 33.0	0.99
3140203	8408	HARD LABOR CR	11.0	28.8	9 - 49	0.0	0.0 - 0.0	4.0	1.1 - 7.6	1.5	0.4 - 3.7	6.5	2.0 - 14.6	0.0	0.0 - 0.0	16.8	5.4 - 34.6	0.93
3140203	8409	HARD LABOR CR	149.7	27.3	8 - 67	0.0	0.0 - 0.0	7.8	2.5 - 16.8	1.0	0.3 - 3.4	4.9	1.5 - 13.4	0.0	0.0 - 0.0	13.7	4.0 - 37.5	0.92
3140203	8410	FLAT CR	65.3	35.3	10 - 90	0.0	0.0 - 0.0	7.0	2.0 - 18.1	1.9	0.5 - 5.3	11.0	3.2 - 31.8	0.0	0.0 - 0.0	15.4	4.3 - 44.6	0.92
3140203	8411	HOLMES CR	145.3	43.2	13 - 87	7.1	2.2 - 17.7	8.7	2.3 - 19.0	3.3	0.9 - 8.7	9.4	2.6 - 27.7	0.0	0.0 - 0.0	14.7	3.9 - 34.1	0.93
3140203	8412	ALLIGATOR CR	269.3	55.4	22 - 103	6.7	2.4 - 13.5	10.3	3.5 - 19.5	3.6	1.2 - 9.0	20.3	7.7 - 46.6	0.0	0.0 - 0.0	14.5	5.4 - 29.9	0.92
3140203	8413	HOLMES CR	245.9	51.4	19 - 120	1.5	0.5 - 3.8	8.0	2.7 - 19.4	7.9	2.4 - 23.1	20.0	7.1 - 61.6	0.0	0.0 - 0.0	14.1	5.4 - 37.4	0.92
3140203	8414	CHOCTAWHATCH	13.7	8.7	3 - 21	0.0	0.0 - 0.0	0.3	0.1 - 0.7	0.0	0.0 - 0.1	0.0	0.0 - 0.0	0.0	0.0 - 0.0	8.4	2.8 - 20.9	0.99
3140203	8415	CHOCTAWHATCH	37.5	13.1	4 - 28	0.0	0.0 - 0.0	2.0	0.6 - 4.7	0.6	0.1 - 2.2	2.4	0.7 - 6.1	0.0	0.0 - 0.0	8.1	2.4 - 20.0	0.98
3140203	8416	CHOCTAWHATCH	6.0	22.4	8 - 59	0.0	0.0 - 0.0	0.2	0.1 - 0.5	0.1	0.0 - 0.3	0.5	0.1 - 1.2	0.0	0.0 - 0.0	21.6	8.1 - 57.9	0.98
3140203	8417	CHOCTAWHATCH	81.5	32.0	10 - 68	0.0	0.0 - 0.0	3.6	1.2 - 8.3	3.2	1.0 - 9.2	5.7	1.9 - 15.2	0.0	0.0 - 0.0	19.4	6.0 - 47.1	0.98
3140203	8418	*A	94.4	25.7	8 - 67	0.0	0.0 - 0.0	5.9	1.7 - 15.2	1.2	0.3 - 3.5	3.6	1.1 - 9.6	0.0	0.0 - 0.0	15.0	5.1 - 38.2	0.97
3140203	8419	CHOCTAWHATCH	144.5	29.4	10 - 53	0.0	0.0 - 0.0	6.1	2.1 - 12.1	3.3	1.1 - 7.7	5.8	1.8 - 15.1	0.0	0.0 - 0.0	14.2	5.3 - 28.6	0.97
3140203	8420	WRIGHTS CR	104.5	28.3	10 - 56	0.0	0.0 - 0.0	3.6	1.2 - 6.8	5.0	1.6 - 13.0	7.6	2.5 - 18.4	0.0	0.0 - 0.0	12.2	4.3 - 28.3	0.97
3140203	8421	WRIGHTS CR	224.0	50.4	18 - 107	0.0	0.0 - 0.0	5.9	2.0 - 13.1	12.0	3.5 - 32.7	19.4	6.7 - 50.1	0.0	0.0 - 0.0	13.2	5.0 - 30.7	0.94
3140203	8422	TENMILE CR	126.3	40.5	13 - 99	0.0	0.0 - 0.0	4.9	1.5 - 11.8	8.7	2.6 - 30.4	14.0	4.1 - 36.7	0.0	0.0 - 0.0	12.8	3.9 - 32.1	0.94
3140203	8423	CHOCTAWHATCH	92.9	25.1	9 - 48	0.0	0.0 - 0.0	2.1	0.7 - 4.0	3.5	1.2 - 9.2	5.6	1.7 - 13.9	0.0	0.0 - 0.0	13.8	5.1 - 31.3	0.97
3140203	8424	CHOCTAWHATCH	42.8	26.6	9 - 66	0.0	0.0 - 0.0	3.5	1.2 - 8.5	3.9	1.2 - 13.4	6.0	2.0 - 16.1	0.0	0.0 - 0.0	13.2	4.7 - 35.9	0.96

8-digit HUC	ID	Name	Area	Catchment Yield		Point sources		Developed Land		Manure		Agricultural Land		Phosphate Mines		Soil parent rock		Frac
				kg/km^2	90% CI	kg/km^2	90% CI	kg/km^2	90% CI	kg/km^2	90% CI	kg/km^2	90% CI	kg/km^2	90% CI	kg/km^2	90% CI	
3140203	8425	E PITTMAN CR	81.4	47.5	16 - 107	0.0	0.0 - 0.0	5.4	1.7 - 13.3	11.4	3.2 - 35.2	18.5	6.4 - 52.2	0.0	0.0 - 0.0	12.2	4.5 - 28.2	0.96
3140203	8427	CHOCTAWHATCHI	39.7	51.1	23 - 127	0.0	0.0 - 0.0	5.1	2.1 - 13.9	11.7	3.7 - 41.7	19.1	7.6 - 65.2	0.0	0.0 - 0.0	15.2	6.7 - 42.9	0.96
3140203	8428	SPRING CR	131.4	65.0	19 - 162	0.0	0.0 - 0.0	8.6	2.1 - 19.8	15.9	4.3 - 41.3	27.1	8.8 - 70.9	0.0	0.0 - 0.0	13.4	4.3 - 32.8	0.95
3140203	8429	CHOCTAWHATCHI	6.3	33.6	12 - 81	0.0	0.0 - 0.0	4.8	1.6 - 12.2	6.0	1.7 - 18.0	10.7	3.7 - 27.2	0.0	0.0 - 0.0	12.1	4.5 - 31.6	0.95
3140203	8430	*B	53.9	41.1	13 - 94	0.0	0.0 - 0.0	6.7	2.1 - 16.7	8.3	2.1 - 23.7	13.6	4.5 - 34.0	0.0	0.0 - 0.0	12.6	3.6 - 29.7	0.96
3140203	8431	W PITTMAN CR	127.1	35.1	14 - 86	0.0	0.0 - 0.0	5.6	2.0 - 12.9	5.7	2.1 - 18.1	9.4	3.3 - 26.3	0.0	0.0 - 0.0	14.3	5.2 - 34.5	0.96
3140203	8432	SANDY CR	49.7	21.5	7 - 51	0.0	0.0 - 0.0	6.2	1.9 - 14.9	1.3	0.4 - 3.6	2.6	0.9 - 7.5	0.0	0.0 - 0.0	11.4	3.5 - 25.0	0.98
3140203	8433	BLUE CR	105.3	32.1	10 - 66	0.0	0.0 - 0.0	5.0	1.7 - 9.5	4.9	1.4 - 14.0	8.2	2.5 - 19.5	0.0	0.0 - 0.0	14.1	4.1 - 30.6	0.95
3140203	8434	SANDY CR	100.0	29.2	11 - 51	0.0	0.0 - 0.0	9.6	3.6 - 18.2	2.8	0.9 - 7.8	4.3	1.5 - 10.5	0.0	0.0 - 0.0	12.4	4.5 - 26.6	0.95
3140203	8435	SANDY CR	69.9	38.7	14 - 99	0.0	0.0 - 0.0	6.4	2.2 - 15.7	3.2	1.1 - 10.0	10.1	3.4 - 24.9	0.0	0.0 - 0.0	19.0	6.9 - 47.5	0.90
3140203	8436	*C	57.1	53.0	20 - 89	0.0	0.0 - 0.0	29.9	10.6 - 52.0	1.4	0.4 - 3.0	4.8	1.5 - 12.7	0.0	0.0 - 0.0	16.9	5.7 - 33.2	0.90
3140203	8437	*A	223.3	18.4	6 - 47	0.0	0.0 - 0.0	9.3	3.3 - 24.6	0.8	0.2 - 2.5	2.4	0.8 - 6.0	0.0	0.0 - 0.0	5.9	1.7 - 17.2	0.98
3140203	8438	SEVEN RUNS CR	76.0	34.0	11 - 72	0.0	0.0 - 0.0	6.8	2.5 - 15.5	4.4	1.2 - 12.9	15.7	5.2 - 46.8	0.0	0.0 - 0.0	7.0	2.4 - 18.2	0.98
3140203	8439	BIG CYPRESS CR	29.4	19.5	7 - 47	0.0	0.0 - 0.0	2.9	1.0 - 7.0	0.7	0.2 - 2.1	1.7	0.5 - 5.4	0.0	0.0 - 0.0	14.2	5.4 - 37.7	0.99
3140301	8440	CONECUH R	91.9	24.2	8 - 42	0.0	0.0 - 0.0	2.7	0.9 - 4.9	2.6	0.7 - 6.5	9.0	2.7 - 20.4	0.0	0.0 - 0.0	9.9	3.1 - 21.7	0.94
3140301	8441	*E	34.3	28.6	8 - 56	0.0	0.0 - 0.0	10.8	3.2 - 21.1	3.7	1.2 - 9.9	7.9	2.5 - 19.7	0.0	0.0 - 0.0	6.2	2.2 - 13.5	0.93
3140301	8442	CONECUH R	51.9	25.8	9 - 54	0.0	0.0 - 0.0	4.8	1.6 - 10.8	4.4	1.3 - 12.1	9.8	3.3 - 26.6	0.0	0.0 - 0.0	6.8	2.6 - 15.7	0.93
3140301	8443	CONECUH R	121.2	46.8	17 - 112	16.7	5.6 - 41.6	7.7	2.5 - 18.2	5.5	1.9 - 19.3	11.9	4.4 - 37.4	0.0	0.0 - 0.0	5.0	1.7 - 14.4	0.93
3140301	8444	CONECUH R	46.7	45.6	15 - 103	1.2	0.4 - 2.7	26.9	9.4 - 62.5	4.4	1.3 - 13.0	9.1	2.8 - 23.0	0.0	0.0 - 0.0	4.1	1.3 - 10.6	0.92
3140301	8445	CONECUH R	201.2	21.5	7 - 51	0.0	0.0 - 0.0	5.6	1.7 - 13.8	3.5	1.0 - 9.7	7.4	2.3 - 20.9	0.0	0.0 - 0.0	4.9	1.6 - 12.0	0.87
3140301	8446	CONECUH R	50.8	29.8	10 - 70	0.0	0.0 - 0.0	3.4	1.0 - 7.3	6.6	2.1 - 18.4	8.7	2.7 - 22.9	0.0	0.0 - 0.0	11.0	4.1 - 27.6	0.61
3140301	8447	CONECUH R	3.6	15.1	5 - 43	0.0	0.0 - 0.0	0.4	0.1 - 1.0	0.2	0.0 - 0.5	0.0	0.0 - 0.0	0.0	0.0 - 0.0	14.6	5.1 - 41.0	0.60
3140301	8448	CONECUH R	24.6	27.1	10 - 57	0.0	0.0 - 0.0	6.2	1.9 - 13.4	5.4	1.6 - 15.7	5.7	1.9 - 13.1	0.0	0.0 - 0.0	9.9	3.6 - 24.5	0.60
3140301	8449	CONECUH R	16.7	19.8	7 - 48	0.0	0.0 - 0.0	5.1	1.5 - 10.7	3.1	0.8 - 7.3	3.5	1.0 - 8.8	0.0	0.0 - 0.0	8.2	2.8 - 23.2	0.60
3140301	8450	CONECUH R	131.1	24.8	8 - 49	0.0	0.0 - 0.0	5.9	1.9 - 13.1	5.2	1.6 - 12.2	5.8	1.7 - 13.5	0.0	0.0 - 0.0	7.9	2.7 - 17.3	0.60
3140301	8451	CONECUH R	11.0	56.3	22 - 122	0.0	0.0 - 0.0	6.0	2.2 - 13.6	14.1	4.9 - 42.8	20.8	7.3 - 54.0	0.0	0.0 - 0.0	15.4	5.9 - 39.3	0.59
3140301	8452	CONECUH R	321.7	31.1	11 - 69	0.0	0.0 - 0.0	6.7	2.2 - 15.1	7.0	2.2 - 21.4	9.7	3.2 - 26.4	0.0	0.0 - 0.0	7.7	2.8 - 19.5	0.58
3140301	8453	CONECUH R	35.8	41.1	15 - 91	0.0	0.0 - 0.0	17.9	6.6 - 40.2	5.3	1.7 - 13.5	7.9	2.9 - 20.3	0.0	0.0 - 0.0	9.9	4.2 - 23.5	0.56
3140301	8454	CONECUH R	122.8	19.8	7 - 45	0.0	0.0 - 0.0	5.0	1.6 - 12.0	2.9	0.8 - 8.5	4.3	1.5 - 12.4	0.0	0.0 - 0.0	7.6	2.8 - 19.5	0.55
3140301	8455	CONECUH R	287.9	20.7	8 - 45	0.0	0.0 - 0.0	5.8	2.1 - 13.4	1.2	0.3 - 4.0	5.2	1.8 - 11.1	0.0	0.0 - 0.0	8.5	2.7 - 20.5	0.53
3140301	8456	LOG CR	52.5	20.0	8 - 33	0.0	0.0 - 0.0	3.3	1.3 - 5.6	3.5	1.1 - 8.6	5.6	2.1 - 12.1	0.0	0.0 - 0.0	7.7	3.0 - 15.0	0.53
3140301	8457	MANNINGS CR	154.6	16.7	6 - 41	0.0	0.0 - 0.0	3.2	1.0 - 7.2	2.2	0.7 - 6.4	3.6	1.2 - 10.0	0.0	0.0 - 0.0	7.7	2.7 - 19.1	0.55
3140301	8458	BEEMAN CR	78.1	19.4	6 - 39	0.0	0.0 - 0.0	6.2	1.9 - 12.0	2.2	0.7 - 5.4	3.1	1.0 - 8.4	0.0	0.0 - 0.0	7.9	2.5 - 18.0	0.56
3140301	8459	*A	21.2	38.0	12 - 80	0.0	0.0 - 0.0	5.7	1.7 - 12.6	12.2	3.8 - 32.5	12.8	3.6 - 32.9	0.0	0.0 - 0.0	7.3	2.4 - 17.1	0.58
3140301	8460	PROVIDENCE CR	43.6	44.3	13 - 96	0.0	0.0 - 0.0	8.3	2.4 - 18.8	13.7	3.8 - 44.8	13.8	3.9 - 39.4	0.0	0.0 - 0.0	8.5	2.6 - 21.4	0.59
3140301	8461	DRY CR	52.7	23.4	8 - 59	0.0	0.0 - 0.0	6.4	2.0 - 14.6	5.1	1.5 - 14.5	5.2	1.7 - 15.0	0.0	0.0 - 0.0	6.6	2.0 - 17.9	0.60
3140301	8462	BRUSHY CR	26.1	25.1	8 - 51	0.0	0.0 - 0.0	5.1	1.6 - 11.3	6.6	1.7 - 19.8	6.5	2.1 - 15.3	0.0	0.0 - 0.0	6.9	2.6 - 16.4	0.60
3140301	8463	BUCK CR	30.5	27.0	8 - 64	0.0	0.0 - 0.0	7.1	2.0 - 18.3	6.2	1.6 - 18.1	6.4	1.9 - 18.8	0.0	0.0 - 0.0	7.3	2.2 - 20.9	0.60
3140301	8464	*B	17.0	17.8	6 - 37	0.0	0.0 - 0.0	4.0	1.3 - 8.7	3.3	1.1 - 8.5	3.4	1.2 - 8.9	0.0	0.0 - 0.0	7.1	2.6 - 17.5	0.60

8-digit HUC	ID	Name	Area	Catchment Yield		Point sources		Developed Land		Manure		Agricultural Land		Phosphate Mines		Soil parent rock		Frac
				kg/km^2	90% CI	kg/km^2	90% CI	kg/km^2	90% CI	kg/km^2	90% CI	kg/km^2	90% CI	kg/km^2	90% CI	kg/km^2	90% CI	
3140301	8465	HORNET CR	20.0	21.0	7 - 56	0.0	0.0 - 0.0	6.1	2.1 - 18.6	3.9	1.1 - 11.7	4.1	1.4 - 12.6	0.0	0.0 - 0.0	6.8	2.3 - 19.5	0.61
3140301	8466	*C	47.5	26.2	8 - 43	0.0	0.0 - 0.0	8.4	2.5 - 14.4	4.7	1.2 - 10.7	10.0	2.8 - 21.3	0.0	0.0 - 0.0	3.1	0.9 - 6.1	0.92
3140301	8467	*D	33.3	18.1	6 - 34	0.0	0.0 - 0.0	4.3	1.4 - 8.4	3.3	1.0 - 8.1	7.0	2.2 - 18.8	0.0	0.0 - 0.0	3.5	1.2 - 7.9	0.93
3140302	8468	PASTALIGA R	12.1	60.6	17 - 128	0.0	0.0 - 0.0	7.5	2.1 - 16.6	14.9	3.5 - 39.1	32.9	10.1 - 83.2	0.0	0.0 - 0.0	5.3	1.5 - 13.0	0.87
3140302	8469	PASTALIGA R	154.2	24.0	10 - 53	0.0	0.0 - 0.0	4.2	1.7 - 8.6	3.9	1.5 - 11.5	8.2	2.9 - 27.1	0.0	0.0 - 0.0	7.7	3.1 - 17.9	0.87
3140302	8470	PASTALIGA R	26.2	15.9	5 - 31	0.0	0.0 - 0.0	2.9	0.9 - 5.6	2.1	0.7 - 5.5	2.5	0.7 - 5.5	0.0	0.0 - 0.0	8.5	2.8 - 18.7	0.85
3140302	8471	PASTALIGA R	108.5	21.7	8 - 36	0.0	0.0 - 0.0	4.3	1.6 - 8.3	4.6	1.5 - 10.5	4.7	1.8 - 12.0	0.0	0.0 - 0.0	8.0	2.8 - 16.8	0.84
3140302	8472	PASTALIGA R	7.3	18.9	8 - 43	0.0	0.0 - 0.0	0.4	0.2 - 0.9	4.3	1.6 - 10.6	4.1	1.5 - 11.0	0.0	0.0 - 0.0	10.2	4.1 - 25.8	0.82
3140302	8473	PASTALIGA R	106.2	41.9	16 - 85	0.0	0.0 - 0.0	10.4	3.8 - 20.3	10.8	3.5 - 24.7	11.1	4.3 - 29.1	0.0	0.0 - 0.0	9.6	3.1 - 20.3	0.82
3140302	8474	PASTALIGA R	69.2	32.9	11 - 58	0.0	0.0 - 0.0	5.4	1.7 - 9.2	8.6	2.4 - 22.9	9.5	3.2 - 22.1	0.0	0.0 - 0.0	9.4	3.1 - 19.6	0.79
3140302	8475	PASTALIGA R	35.2	25.3	9 - 61	0.0	0.0 - 0.0	4.0	1.2 - 9.2	5.6	1.6 - 18.3	6.1	1.9 - 15.5	0.0	0.0 - 0.0	9.7	3.0 - 25.6	0.77
3140302	8476	OLUSTEE CR	123.3	21.6	6 - 46	0.0	0.0 - 0.0	4.5	1.4 - 9.6	2.9	0.8 - 8.6	5.8	1.6 - 16.4	0.0	0.0 - 0.0	8.4	2.2 - 20.4	0.74
3140302	8477	PASTALIGA R	45.4	18.5	7 - 34	0.0	0.0 - 0.0	4.6	1.8 - 9.1	2.1	0.6 - 5.8	2.9	1.0 - 6.8	0.0	0.0 - 0.0	8.8	2.9 - 21.3	0.74
3140302	8478	WEAVER MILL CR	32.5	20.8	7 - 43	0.0	0.0 - 0.0	4.7	1.7 - 9.5	1.9	0.6 - 5.0	6.7	1.9 - 17.9	0.0	0.0 - 0.0	7.6	2.6 - 16.3	0.67
3140302	8479	PASTALIGA R	38.9	22.1	8 - 37	0.0	0.0 - 0.0	5.2	1.7 - 9.7	2.2	0.6 - 5.9	7.3	2.6 - 16.6	0.0	0.0 - 0.0	7.4	2.9 - 14.5	0.67
3140302	8480	BLUE CR	61.8	26.7	9 - 55	0.0	0.0 - 0.0	6.2	2.2 - 14.8	5.3	1.6 - 14.4	5.7	2.0 - 14.2	0.0	0.0 - 0.0	9.6	3.4 - 21.3	0.77
3140302	8481	DRY CR	33.0	16.5	5 - 39	0.0	0.0 - 0.0	4.7	1.5 - 10.3	2.0	0.5 - 6.9	2.4	0.7 - 6.3	0.0	0.0 - 0.0	7.3	2.3 - 21.2	0.72
3140302	8482	BLUE CR	64.8	21.9	6 - 38	0.0	0.0 - 0.0	6.7	2.0 - 13.5	3.5	0.9 - 8.0	4.8	1.3 - 9.9	0.0	0.0 - 0.0	6.9	2.1 - 14.7	0.72
3140302	8483	POND CR	41.6	26.7	8 - 51	0.0	0.0 - 0.0	5.9	1.7 - 12.0	6.8	1.6 - 17.0	7.1	2.0 - 16.7	0.0	0.0 - 0.0	6.8	2.5 - 15.7	0.79
3140302	8484	LITTLE PATSALIG.	38.3	52.4	17 - 91	0.6	0.2 - 1.2	6.2	1.9 - 11.2	17.1	5.1 - 35.9	17.8	5.7 - 40.4	0.0	0.0 - 0.0	10.7	3.5 - 24.8	0.82
3140302	8485	LITTLE PATSALIG.	217.5	27.4	8 - 62	0.0	0.0 - 0.0	6.2	2.1 - 13.9	6.6	1.5 - 17.7	6.7	2.3 - 18.0	0.0	0.0 - 0.0	7.9	2.4 - 20.7	0.80
3140302	8486	SILOM CR	48.5	40.6	15 - 88	0.0	0.0 - 0.0	6.7	2.4 - 14.7	12.5	4.2 - 35.6	13.3	4.9 - 39.6	0.0	0.0 - 0.0	8.0	3.1 - 21.2	0.80
3140302	8487	HORSE CR	31.4	40.3	12 - 72	0.0	0.0 - 0.0	5.6	1.5 - 11.8	14.0	4.0 - 39.3	14.4	4.1 - 36.7	0.0	0.0 - 0.0	6.3	1.9 - 15.4	0.82
3140302	8488	PINEY WOODS CR	82.0	21.2	7 - 59	0.0	0.0 - 0.0	5.3	1.6 - 13.6	4.4	1.4 - 14.0	4.9	1.6 - 14.0	0.0	0.0 - 0.0	6.6	1.9 - 17.8	0.84
3140302	8489	SAWYER CR	48.9	18.6	5 - 38	0.0	0.0 - 0.0	4.0	1.3 - 8.8	2.5	0.8 - 5.5	3.9	1.0 - 10.3	0.0	0.0 - 0.0	8.2	2.5 - 17.3	0.85
3140302	8490	BUCK CR	55.9	30.0	11 - 58	0.0	0.0 - 0.0	8.2	2.9 - 15.8	5.8	1.8 - 15.9	12.4	4.5 - 29.4	0.0	0.0 - 0.0	3.7	1.5 - 8.1	0.87
3140302	8491	OLUSTEE CR	36.0	19.9	8 - 44	0.0	0.0 - 0.0	4.3	1.6 - 9.4	1.9	0.6 - 5.4	6.7	2.5 - 16.2	0.0	0.0 - 0.0	7.0	2.4 - 15.4	0.68
3140302	8492	GREENBRIER CR	37.5	25.2	8 - 62	0.0	0.0 - 0.0	6.1	2.0 - 14.1	2.6	0.7 - 8.4	9.1	3.2 - 24.2	0.0	0.0 - 0.0	7.3	2.2 - 18.7	0.68
3140303	8493	SEPULGA R	78.3	16.5	5 - 29	0.0	0.0 - 0.0	3.4	1.1 - 6.9	0.7	0.2 - 1.9	6.1	2.0 - 15.6	0.0	0.0 - 0.0	6.3	2.2 - 14.5	0.94
3140303	8494	SEPULGA R	61.9	15.1	5 - 33	0.0	0.0 - 0.0	2.9	1.0 - 5.6	1.2	0.4 - 3.4	5.8	1.8 - 15.9	0.0	0.0 - 0.0	5.2	1.8 - 12.9	0.94
3140303	8495	SEPULGA R	17.6	11.1	4 - 32	0.0	0.0 - 0.0	1.9	0.7 - 5.5	1.3	0.4 - 4.6	3.9	1.5 - 12.7	0.0	0.0 - 0.0	4.0	1.4 - 13.3	0.93
3140303	8496	PIGEON CR	94.6	18.6	7 - 45	0.0	0.0 - 0.0	6.0	2.0 - 16.2	2.0	0.6 - 5.6	6.4	2.2 - 18.4	0.0	0.0 - 0.0	4.2	1.4 - 10.9	0.92
3140303	8497	PIGEON CR	529.6	31.5	10 - 65	0.0	0.0 - 0.0	5.8	1.8 - 13.0	6.6	1.7 - 16.6	10.5	3.3 - 25.7	0.0	0.0 - 0.0	8.5	2.7 - 18.8	0.90
3140303	8498	PIGEON CR	6.7	28.7	10 - 59	0.0	0.0 - 0.0	2.4	0.8 - 5.1	4.2	1.3 - 12.9	7.7	2.4 - 16.0	0.0	0.0 - 0.0	14.4	4.3 - 34.2	0.82
3140303	8499	THREE RUN CR	122.7	24.3	8 - 40	0.0	0.0 - 0.0	5.2	1.8 - 10.8	4.0	1.0 - 9.5	6.1	1.8 - 13.2	0.0	0.0 - 0.0	8.9	3.0 - 17.9	0.81
3140303	8500	PIGEON CR	102.4	27.6	10 - 52	0.0	0.0 - 0.0	8.2	2.8 - 18.9	3.8	0.9 - 11.4	7.0	2.4 - 19.1	0.0	0.0 - 0.0	8.6	3.0 - 18.4	0.81
3140303	8501	HALLS CR	50.7	24.0	8 - 55	0.0	0.0 - 0.0	6.5	2.0 - 13.1	3.2	0.9 - 8.7	5.3	1.6 - 13.7	0.0	0.0 - 0.0	9.0	3.2 - 22.2	0.82
3140303	8502	REEDY CR	38.0	25.4	10 - 60	0.0	0.0 - 0.0	7.5	2.8 - 15.5	2.9	1.0 - 7.7	11.4	3.7 - 28.5	0.0	0.0 - 0.0	3.6	1.2 - 8.3	0.90
3140303	8503	SEPULGA R	224.4	21.6	7 - 61	0.0	0.0 - 0.0	6.1	1.9 - 16.8	2.1	0.6 - 5.7	8.5	2.8 - 25.4	0.0	0.0 - 0.0	4.9	1.5 - 14.4	0.92

8-digit HUC	ID	Name	Area	Catchment Yield		Point sources		Developed Land		Manure		Agricultural Land		Phosphate Mines		Soil parent rock		Frac
				kg/km^2	90% CI	kg/km^2	90% CI	kg/km^2	90% CI	kg/km^2	90% CI	kg/km^2	90% CI	kg/km^2	90% CI	kg/km^2	90% CI	
3140303	8504	PERSIMMON CR	58.9	29.4	9 - 72	0.0	0.0 - 0.0	8.7	2.6 - 22.3	3.9	1.1 - 10.4	7.3	2.1 - 22.0	0.0	0.0 - 0.0	9.4	2.8 - 23.0	0.88
3140303	8505	PERSIMMON CR	26.0	24.8	9 - 47	0.0	0.0 - 0.0	5.8	2.0 - 13.5	3.3	0.9 - 8.5	5.4	2.0 - 12.6	0.0	0.0 - 0.0	10.3	3.5 - 22.4	0.87
3140303	8506	PERSIMMON CR	45.9	34.0	12 - 79	0.0	0.0 - 0.0	7.2	2.6 - 17.3	6.0	1.8 - 17.8	9.5	3.3 - 23.8	0.0	0.0 - 0.0	11.3	4.2 - 29.6	0.86
3140303	8507	PERSIMMON CR	41.2	25.4	9 - 47	0.0	0.0 - 0.0	3.1	1.1 - 6.5	4.9	1.5 - 11.0	8.4	2.6 - 22.5	0.0	0.0 - 0.0	9.1	2.7 - 21.3	0.83
3140303	8508	PERSIMMON CR	117.7	50.6	15 - 104	15.7	4.9 - 36.1	14.6	4.4 - 28.7	4.1	1.1 - 11.4	6.8	2.0 - 17.0	0.0	0.0 - 0.0	9.5	3.1 - 21.3	0.79
3140303	8509	STALLINGS CR	81.3	29.9	11 - 67	0.0	0.0 - 0.0	9.4	3.1 - 20.3	4.5	1.5 - 14.2	7.3	2.5 - 20.5	0.0	0.0 - 0.0	8.7	3.3 - 20.0	0.79
3140303	8510	MUSCLE CR	37.3	29.0	9 - 74	0.0	0.0 - 0.0	5.6	1.5 - 14.1	5.6	1.4 - 16.8	9.4	2.6 - 27.7	0.0	0.0 - 0.0	8.4	2.6 - 22.3	0.83
3140303	8511	ROCKY CR	148.4	27.5	9 - 63	0.0	0.0 - 0.0	8.8	2.5 - 20.2	3.9	1.0 - 12.7	6.5	1.8 - 18.6	0.0	0.0 - 0.0	8.3	2.7 - 20.5	0.86
3140303	8512	PANTHER CR	146.4	25.8	9 - 42	0.0	0.0 - 0.0	6.7	2.2 - 13.0	4.1	1.4 - 10.4	6.8	2.1 - 16.4	0.0	0.0 - 0.0	8.1	2.8 - 15.3	0.87
3140303	8513	SEPULGA R	52.7	18.2	6 - 42	0.0	0.0 - 0.0	4.1	1.2 - 8.7	1.3	0.4 - 3.5	5.4	1.9 - 14.2	0.0	0.0 - 0.0	7.5	2.5 - 15.3	0.88
3140303	8514	LONG CR	223.6	23.2	9 - 44	0.0	0.0 - 0.0	4.1	1.4 - 7.5	3.3	1.0 - 6.8	7.1	2.5 - 17.9	0.0	0.0 - 0.0	8.7	3.5 - 17.1	0.86
3140303	8515	SEPULGA R	173.2	16.7	6 - 47	0.0	0.0 - 0.0	2.1	0.7 - 5.1	0.9	0.3 - 2.5	5.7	1.9 - 16.7	0.0	0.0 - 0.0	8.1	3.0 - 22.5	0.86
3140303	8516	OLD TOWN CR	94.7	20.3	7 - 50	0.0	0.0 - 0.0	6.6	2.4 - 15.2	1.3	0.5 - 3.2	8.4	2.6 - 25.6	0.0	0.0 - 0.0	4.1	1.4 - 9.4	0.93
3140303	8517	BOTTLE CR	21.1	14.9	6 - 36	0.0	0.0 - 0.0	4.3	1.3 - 11.1	0.9	0.3 - 2.2	5.5	2.1 - 15.4	0.0	0.0 - 0.0	4.3	1.7 - 11.2	0.94
3140303	8518	BOTTLE CR	46.9	19.4	6 - 45	0.0	0.0 - 0.0	5.1	1.5 - 10.6	1.4	0.4 - 3.5	9.0	2.8 - 25.7	0.0	0.0 - 0.0	4.0	1.4 - 9.1	0.90
3140303	8519	*A	39.9	21.8	7 - 50	0.0	0.0 - 0.0	5.2	1.6 - 12.8	1.7	0.5 - 5.8	11.1	3.5 - 32.5	0.0	0.0 - 0.0	3.8	1.1 - 9.6	0.90
3140304	8520	CONECUH R	21.9	38.7	12 - 82	0.0	0.0 - 0.0	8.3	2.3 - 18.4	1.1	0.3 - 2.7	15.0	4.7 - 37.3	0.0	0.0 - 0.0	14.3	4.5 - 28.6	0.97
3140304	8521	CONECUH R	65.6	671.7	189 - 1592	643.4	179.5 - 1560.4	6.1	1.9 - 11.5	0.9	0.2 - 2.4	6.6	2.2 - 17.4	0.0	0.0 - 0.0	14.8	4.5 - 32.3	0.97
3140304	8522	CONECUH R	374.7	28.5	8 - 74	0.0	0.0 - 0.0	6.3	1.6 - 16.3	0.9	0.2 - 3.1	9.4	2.8 - 25.7	0.0	0.0 - 0.0	11.9	3.7 - 30.7	0.97
3140304	8523	CONECUH R	64.3	19.8	6 - 54	0.0	0.0 - 0.0	5.4	1.5 - 14.1	0.5	0.1 - 1.4	4.9	1.2 - 14.0	0.0	0.0 - 0.0	9.1	3.0 - 24.1	0.95
3140304	8524	SMITH CR	28.7	19.2	6 - 47	0.0	0.0 - 0.0	4.6	1.3 - 9.9	0.8	0.2 - 2.2	7.9	2.5 - 22.9	0.0	0.0 - 0.0	5.9	2.0 - 16.0	0.95
3140304	8525	MURDER CR	37.4	72.4	23 - 131	42.0	13.6 - 76.1	17.4	4.7 - 41.2	0.7	0.2 - 1.9	3.3	1.0 - 7.4	0.0	0.0 - 0.0	9.0	2.5 - 18.0	0.97
3140304	8526	MURDER CR	374.5	23.1	9 - 52	0.0	0.0 - 0.0	5.6	1.8 - 11.9	1.2	0.4 - 3.3	9.6	3.3 - 27.1	0.0	0.0 - 0.0	6.7	2.5 - 15.4	0.96
3140304	8527	MURDER CR	12.5	28.6	9 - 66	0.0	0.0 - 0.0	6.5	2.0 - 15.8	2.3	0.7 - 7.6	13.6	4.3 - 32.8	0.0	0.0 - 0.0	6.2	2.2 - 15.5	0.93
3140304	8528	MURDER CR	60.3	30.9	10 - 74	0.0	0.0 - 0.0	8.6	2.8 - 20.6	2.3	0.7 - 6.4	14.9	4.7 - 40.1	0.0	0.0 - 0.0	5.0	1.7 - 11.7	0.93
3140304	8529	*A	112.9	30.6	10 - 58	8.0	2.9 - 17.6	9.3	2.9 - 22.0	1.2	0.4 - 3.8	7.8	2.5 - 23.4	0.0	0.0 - 0.0	4.2	1.6 - 10.2	0.91
3140304	8530	MURDER CR	27.8	41.7	15 - 86	0.0	0.0 - 0.0	27.7	10.2 - 63.9	1.1	0.4 - 2.9	7.4	2.4 - 19.4	0.0	0.0 - 0.0	5.5	2.1 - 13.3	0.91
3140304	8531	CANE CR	82.8	26.4	7 - 67	0.0	0.0 - 0.0	6.7	2.0 - 15.9	2.1	0.5 - 6.5	13.1	3.1 - 35.2	0.0	0.0 - 0.0	4.5	1.4 - 11.7	0.90
3140304	8532	MURDER CR	345.1	17.3	5 - 42	0.0	0.0 - 0.0	3.6	1.1 - 9.0	1.2	0.3 - 3.8	7.8	2.2 - 22.4	0.0	0.0 - 0.0	4.7	1.8 - 9.5	0.90
3140304	8533	*B	36.0	22.2	6 - 47	0.0	0.0 - 0.0	6.0	1.5 - 13.0	1.6	0.4 - 4.5	9.8	2.8 - 25.9	0.0	0.0 - 0.0	4.8	1.5 - 10.7	0.93
3140304	8534	PANTHER CR	73.9	31.0	10 - 61	0.0	0.0 - 0.0	6.9	2.0 - 14.9	2.4	0.6 - 7.5	15.3	4.7 - 36.3	0.0	0.0 - 0.0	6.4	2.1 - 13.9	0.93
3140304	8535	BURNT CORN CR	65.6	33.9	12 - 84	0.0	0.0 - 0.0	17.3	5.8 - 43.4	0.8	0.3 - 2.5	7.3	2.7 - 20.9	0.0	0.0 - 0.0	8.6	3.1 - 21.2	0.96
3140304	8536	*C	55.4	22.5	9 - 62	0.0	0.0 - 0.0	5.0	2.0 - 11.2	1.1	0.4 - 3.9	9.1	3.4 - 33.8	0.0	0.0 - 0.0	7.2	2.8 - 19.6	0.95
3140304	8537	BURNT CORN CR	86.2	26.4	9 - 64	0.0	0.0 - 0.0	5.5	1.8 - 12.5	1.2	0.4 - 3.3	11.3	3.2 - 30.9	0.0	0.0 - 0.0	8.3	2.9 - 20.9	0.95
3140304	8538	BURNT CORN CR	191.7	20.4	6 - 43	0.0	0.0 - 0.0	4.7	1.5 - 10.0	1.5	0.4 - 4.4	9.3	2.9 - 23.2	0.0	0.0 - 0.0	4.9	1.5 - 12.3	0.91
3140304	8539	*D	85.9	30.7	11 - 67	0.5	0.2 - 1.1	7.1	2.5 - 15.6	2.2	0.8 - 5.7	14.4	5.2 - 34.2	0.0	0.0 - 0.0	6.3	2.1 - 15.6	0.91
3140304	8540	*E	35.1	50.3	16 - 96	0.0	0.0 - 0.0	11.1	3.8 - 23.3	2.5	0.7 - 6.3	24.9	7.3 - 53.5	0.0	0.0 - 0.0	11.8	4.0 - 27.2	0.97
3140304	8541	LITTLE ESCAMBIA	50.5	35.4	9 - 72	0.0	0.0 - 0.0	9.9	2.9 - 22.1	1.8	0.5 - 5.0	12.7	3.9 - 29.8	0.0	0.0 - 0.0	11.0	3.6 - 24.3	0.97
3140304	8542	LITTLE ESCAMBIA	29.9	22.9	8 - 47	0.0	0.0 - 0.0	2.9	0.9 - 6.5	1.8	0.6 - 4.5	9.7	2.8 - 25.2	0.0	0.0 - 0.0	8.5	3.3 - 19.5	0.95

8-digit HUC	ID	Name	Area	Catchment Yield		Point sources		Developed Land		Manure		Agricultural Land		Phosphate Mines		Soil parent rock		Frac
				kg/km^2	90% CI	kg/km^2	90% CI	kg/km^2	90% CI	kg/km^2	90% CI	kg/km^2	90% CI	kg/km^2	90% CI	kg/km^2	90% CI	
3140304	8543	LITTLE ESCAMBIA	83.8	17.2	6 - 29	0.0	0.0 - 0.0	4.2	1.4 - 7.6	0.8	0.2 - 2.3	2.8	0.9 - 6.2	0.0	0.0 - 0.0	9.4	3.1 - 21.7	0.93
3140304	8544	LITTLE ESCAMBIA	60.4	24.3	7 - 58	0.0	0.0 - 0.0	3.5	1.0 - 9.5	1.6	0.4 - 4.7	10.9	3.6 - 26.9	0.0	0.0 - 0.0	8.3	2.5 - 20.6	0.88
3140304	8545	DEAN CR	20.8	20.6	6 - 41	0.0	0.0 - 0.0	4.3	1.3 - 9.6	0.7	0.2 - 1.7	7.0	2.2 - 17.5	0.0	0.0 - 0.0	8.6	3.0 - 18.8	0.88
3140304	8546	NARROW GAP CR	57.5	24.0	9 - 46	0.0	0.0 - 0.0	6.4	2.4 - 11.7	0.9	0.3 - 2.4	8.0	3.3 - 21.1	0.0	0.0 - 0.0	8.7	3.2 - 17.3	0.93
3140304	8547	HALL CR	68.8	28.9	10 - 70	0.0	0.0 - 0.0	6.8	2.4 - 15.8	1.3	0.4 - 3.6	10.5	3.2 - 33.0	0.0	0.0 - 0.0	10.3	4.0 - 26.0	0.95
3140305	8548	ESCAMBIA R	342.5	53.3	19 - 120	8.6	2.8 - 19.6	26.2	8.5 - 56.8	0.8	0.3 - 2.3	7.1	2.7 - 19.9	0.0	0.0 - 0.0	10.6	3.5 - 28.8	1.00
3140305	8549	ESCAMBIA R	154.4	41.7	15 - 92	0.0	0.0 - 0.0	10.5	3.6 - 22.3	1.4	0.4 - 4.0	18.6	6.0 - 52.6	0.0	0.0 - 0.0	11.2	3.4 - 21.7	0.99
3140305	8550	ESCAMBIA R	60.7	36.4	13 - 79	0.0	0.0 - 0.0	7.7	2.6 - 18.4	1.3	0.4 - 3.3	16.7	5.1 - 45.1	0.0	0.0 - 0.0	10.8	3.8 - 22.7	0.98
3140305	8551	ESCAMBIA R	87.1	41.2	14 - 75	2.9	1.1 - 6.0	13.3	4.2 - 27.9	1.6	0.4 - 4.5	12.8	4.4 - 32.2	0.0	0.0 - 0.0	10.6	3.9 - 26.5	0.98
3140305	8552	ESCAMBIA R	34.0	39.8	14 - 75	0.0	0.0 - 0.0	14.3	4.6 - 26.2	1.0	0.3 - 2.3	13.8	4.4 - 37.1	0.0	0.0 - 0.0	10.7	4.0 - 21.1	0.98
3140305	8553	BIG ESCAMBIA CR	161.3	40.7	14 - 79	0.0	0.0 - 0.0	12.3	3.9 - 24.7	2.1	0.6 - 5.2	16.6	5.1 - 36.2	0.0	0.0 - 0.0	9.8	3.3 - 21.0	0.98
3140305	8554	BIG ESCAMBIA CR	60.5	32.3	12 - 72	0.0	0.0 - 0.0	6.2	1.9 - 14.9	1.7	0.5 - 4.5	15.7	5.7 - 39.4	0.0	0.0 - 0.0	8.7	2.9 - 20.4	0.94
3140305	8555	BIG ESCAMBIA CR	78.6	29.2	9 - 60	0.0	0.0 - 0.0	4.1	1.2 - 8.1	1.5	0.4 - 4.0	14.2	4.1 - 34.9	0.0	0.0 - 0.0	9.4	2.7 - 22.3	0.93
3140305	8556	*A	153.2	21.6	6 - 56	0.0	0.0 - 0.0	2.4	0.7 - 5.9	1.7	0.4 - 4.6	10.5	3.0 - 30.8	0.0	0.0 - 0.0	7.0	2.2 - 18.0	0.90
3140305	8557	BIG ESCAMBIA CR	28.3	34.8	15 - 61	0.0	0.0 - 0.0	3.1	1.2 - 5.8	3.0	1.1 - 7.1	19.1	6.9 - 41.0	0.0	0.0 - 0.0	9.5	3.8 - 22.1	0.90
3140305	8558	CORLEY CR	65.2	50.4	17 - 111	0.0	0.0 - 0.0	7.8	2.5 - 18.4	4.8	1.4 - 13.7	29.9	10.3 - 82.8	0.0	0.0 - 0.0	8.1	2.3 - 20.7	0.88
3140305	8559	BIG ESCAMBIA CR	85.5	68.2	25 - 149	0.0	0.0 - 0.0	11.7	4.1 - 25.9	6.2	1.8 - 20.8	38.6	12.3 - 106.0	0.0	0.0 - 0.0	11.6	4.1 - 29.3	0.88
3140305	8560	ROBINSON CR	40.3	30.6	9 - 64	0.0	0.0 - 0.0	2.1	0.8 - 4.8	2.0	0.5 - 4.9	17.7	4.7 - 47.7	0.0	0.0 - 0.0	8.8	2.4 - 20.4	0.93
3140305	8561	SIZEMORE CR	21.4	25.9	10 - 48	0.0	0.0 - 0.0	9.1	3.4 - 18.5	1.3	0.4 - 3.1	8.3	2.7 - 20.3	0.0	0.0 - 0.0	7.3	2.8 - 15.8	0.94
3140305	8562	WET WEATHER CR	131.9	53.2	21 - 111	2.9	1.1 - 6.5	7.2	3.0 - 15.3	2.9	1.0 - 7.4	29.1	10.7 - 71.0	0.0	0.0 - 0.0	11.1	4.6 - 24.3	0.92
3140305	8563	SIZEMORE CR	64.5	61.1	21 - 109	0.0	0.0 - 0.0	11.8	3.8 - 22.6	3.4	1.1 - 8.6	34.5	11.3 - 76.7	0.0	0.0 - 0.0	11.4	4.3 - 23.6	0.92
3140305	8564	CANOE CR	117.1	50.7	14 - 106	0.0	0.0 - 0.0	9.9	2.9 - 19.2	3.0	0.8 - 7.1	26.4	7.3 - 72.8	0.0	0.0 - 0.0	11.4	3.3 - 24.9	0.98
3140305	8565	MITCHELL CR	35.2	29.6	10 - 56	0.0	0.0 - 0.0	10.2	3.8 - 19.9	1.3	0.4 - 2.9	6.5	2.3 - 14.9	0.0	0.0 - 0.0	11.7	4.2 - 26.3	0.98
3140305	8566	PINE BARREN CR	78.0	29.8	10 - 65	0.0	0.0 - 0.0	9.7	3.4 - 21.4	1.3	0.4 - 3.7	4.7	1.7 - 11.9	0.0	0.0 - 0.0	14.1	4.4 - 34.8	0.99
3140305	8567	PINE BARREN CR	110.4	64.4	22 - 127	0.0	0.0 - 0.0	15.7	4.9 - 31.5	4.0	1.3 - 11.2	32.1	10.7 - 69.7	0.0	0.0 - 0.0	12.7	4.6 - 27.7	0.93
3140305	8568	BLUE WATER CR	51.9	56.6	17 - 132	0.0	0.0 - 0.0	9.2	3.0 - 20.8	4.0	1.0 - 11.5	30.7	9.0 - 86.0	0.0	0.0 - 0.0	12.6	4.7 - 27.8	0.93
3150101	8569	CONSAUGA R	267.2	63.6	23 - 181	0.0	0.0 - 0.0	25.0	9.0 - 76.4	10.6	3.4 - 34.2	10.9	3.7 - 31.3	0.0	0.0 - 0.0	17.1	6.1 - 44.7	0.31
3150101	8570	HOLLY CR	61.9	51.9	17 - 98	0.0	0.0 - 0.0	10.9	3.6 - 21.1	7.3	2.0 - 18.4	11.8	4.3 - 26.7	0.0	0.0 - 0.0	21.9	7.3 - 48.6	0.30
3150101	8571	ROCK CR	56.5	38.4	14 - 94	0.0	0.0 - 0.0	8.2	2.9 - 16.8	4.1	1.2 - 13.7	7.0	2.5 - 16.2	0.0	0.0 - 0.0	19.0	7.0 - 49.8	0.29
3150101	8572	HOLLY CR	182.1	66.6	22 - 121	25.0	8.2 - 52.3	12.0	3.3 - 26.0	4.1	1.2 - 10.4	7.1	2.3 - 17.5	0.0	0.0 - 0.0	18.4	5.7 - 43.9	0.29
3150101	8573	CONSAUGA R	17.2	97.3	30 - 190	0.0	0.0 - 0.0	17.4	5.0 - 40.4	21.9	5.6 - 53.4	31.1	9.2 - 74.3	0.0	0.0 - 0.0	26.9	7.6 - 70.0	0.30
3150101	8574	CONSAUGA R	55.1	95.8	33 - 200	0.0	0.0 - 0.0	28.9	8.9 - 61.3	17.6	5.6 - 54.5	28.0	9.1 - 69.8	0.0	0.0 - 0.0	21.3	7.0 - 49.9	0.30
3150101	8575	MILL CR	109.5	64.7	22 - 186	0.0	0.0 - 0.0	19.5	6.2 - 54.5	10.3	3.4 - 32.5	18.1	6.6 - 53.4	0.0	0.0 - 0.0	16.8	5.9 - 47.4	0.29
3150101	8576	CONSAUGA R	83.4	80.0	25 - 147	0.0	0.0 - 0.0	12.8	4.0 - 26.6	21.0	6.1 - 54.9	25.4	7.8 - 62.3	0.0	0.0 - 0.0	20.7	6.5 - 44.0	0.29
3150101	8577	SUMAC CR	36.8	67.0	26 - 166	0.0	0.0 - 0.0	15.0	6.1 - 36.6	12.8	4.5 - 42.4	22.3	9.1 - 59.9	0.0	0.0 - 0.0	16.9	6.9 - 47.5	0.28
3150101	8578	SUMAC CR	34.5	26.7	9 - 60	0.0	0.0 - 0.0	4.3	1.4 - 10.2	2.8	0.8 - 7.3	4.9	1.7 - 13.7	0.0	0.0 - 0.0	14.8	5.1 - 31.4	0.26
3150101	8579	*A	28.7	29.3	9 - 56	0.0	0.0 - 0.0	8.1	2.4 - 16.5	2.6	0.7 - 6.2	4.4	1.4 - 10.8	0.0	0.0 - 0.0	14.2	4.5 - 30.9	0.26
3150101	8580	CONSAUGA R	18.8	83.9	33 - 192	0.0	0.0 - 0.0	8.3	3.2 - 18.7	21.7	7.3 - 60.5	29.2	9.8 - 82.8	0.0	0.0 - 0.0	24.8	10.2 - 63.0	0.28
3150101	8581	CONSAUGA R	31.0	103.6	37 - 207	0.0	0.0 - 0.0	9.4	2.9 - 17.0	25.9	8.1 - 72.2	45.6	15.0 - 109.4	0.0	0.0 - 0.0	22.6	7.5 - 55.3	0.28

8-digit HUC	ID	Name	Area	Catchment Yield		Point sources		Developed Land		Manure		Agricultural Land		Phosphate Mines		Soil parent rock		Frac
				kg/km^2	90% CI	kg/km^2	90% CI	kg/km^2	90% CI	kg/km^2	90% CI	kg/km^2	90% CI	kg/km^2	90% CI	kg/km^2	90% CI	
3150101	8582	CONSAUGA R	95.4	34.1	10 - 67	0.0	0.0 - 0.0	7.9	2.3 - 17.4	4.4	1.2 - 13.0	8.1	2.4 - 19.8	0.0	0.0 - 0.0	13.8	4.2 - 31.4	0.27
3150101	8583	CONSAUGA R	93.1	18.8	7 - 42	0.0	0.0 - 0.0	1.2	0.4 - 2.7	0.2	0.1 - 0.6	0.3	0.1 - 0.9	0.0	0.0 - 0.0	17.2	6.4 - 39.9	0.25
3150101	8584	JACKS CR	132.0	18.3	7 - 36	0.0	0.0 - 0.0	1.3	0.5 - 2.9	0.0	0.0 - 0.1	0.0	0.0 - 0.1	0.0	0.0 - 0.0	16.9	6.3 - 33.4	0.25
3150101	8585	MILL CR	7.2	71.2	25 - 143	0.0	0.0 - 0.0	7.1	2.1 - 16.5	19.3	6.5 - 51.7	25.7	8.2 - 63.3	0.0	0.0 - 0.0	19.2	6.3 - 42.6	0.27
3150101	8586	OLD FORT CR	25.4	60.9	21 - 157	0.0	0.0 - 0.0	7.9	2.6 - 19.4	14.6	4.6 - 41.2	23.3	8.0 - 63.4	0.0	0.0 - 0.0	15.1	5.3 - 41.0	0.25
3150101	8587	MILL CR	31.1	65.0	23 - 152	0.0	0.0 - 0.0	6.5	2.2 - 12.3	19.2	5.5 - 46.0	24.0	8.5 - 53.5	0.0	0.0 - 0.0	15.4	5.2 - 32.9	0.25
3150101	8588	SUGAR CR	57.3	59.7	20 - 107	0.0	0.0 - 0.0	5.0	1.7 - 9.7	16.9	4.9 - 37.8	21.4	7.5 - 49.8	0.0	0.0 - 0.0	16.5	5.2 - 33.8	0.28
3150101	8589	COAHULLA R	12.6	75.5	28 - 149	0.0	0.0 - 0.0	21.4	7.8 - 46.4	16.7	5.2 - 39.2	18.5	6.4 - 48.1	0.0	0.0 - 0.0	19.0	7.3 - 40.4	0.30
3150101	8590	COAHULLA R	311.1	72.3	22 - 144	0.0	0.0 - 0.0	13.8	3.4 - 27.9	17.5	4.3 - 47.5	21.0	6.5 - 46.5	3.6	0.9 - 11.6	16.3	4.7 - 39.0	0.29
3150101	8591	MILL CR	35.3	108.3	38 - 253	0.0	0.0 - 0.0	83.9	30.6 - 198.1	3.1	1.0 - 11.3	3.4	1.2 - 11.2	0.0	0.0 - 0.0	17.8	6.1 - 43.7	0.29
3150101	8592	HAIG CR	30.2	50.7	20 - 88	0.0	0.0 - 0.0	23.1	8.5 - 44.2	6.2	2.1 - 16.7	6.5	2.4 - 14.6	0.0	0.0 - 0.0	14.9	5.7 - 31.5	0.27
3150101	8593	MILL CR	76.5	51.0	18 - 125	0.0	0.0 - 0.0	27.6	9.2 - 61.4	4.9	1.4 - 15.0	5.6	1.9 - 15.7	0.0	0.0 - 0.0	12.8	4.2 - 36.1	0.27
3150102	8594	COOSAWATTEE R	91.7	113.1	32 - 320	0.0	0.0 - 0.0	12.8	3.9 - 31.2	42.9	12.7 - 139.4	37.2	13.5 - 110.9	0.0	0.0 - 0.0	20.1	6.0 - 62.2	0.31
3150102	8595	SALACOA CR	5.6	164.8	62 - 283	0.0	0.0 - 0.0	12.6	4.4 - 26.8	71.5	23.9 - 233.7	61.0	21.4 - 138.0	0.0	0.0 - 0.0	19.7	7.3 - 41.9	0.31
3150102	8596	SALACOA CR	9.8	111.1	30 - 270	0.0	0.0 - 0.0	8.0	2.8 - 19.8	43.1	14.0 - 117.4	36.5	10.8 - 94.0	0.0	0.0 - 0.0	23.6	8.2 - 56.4	0.30
3150102	8597	PINE LOG CR	76.8	105.2	38 - 189	0.0	0.0 - 0.0	15.6	5.8 - 32.5	39.6	12.8 - 104.2	33.3	12.1 - 84.4	0.0	0.0 - 0.0	16.7	5.9 - 34.7	0.30
3150102	8598	CEDAR CR	78.6	52.8	17 - 142	0.0	0.0 - 0.0	7.9	2.9 - 18.6	14.6	3.9 - 42.7	15.7	5.7 - 44.8	0.0	0.0 - 0.0	14.5	4.6 - 42.1	0.28
3150102	8599	PINE LOG CR	174.5	38.7	13 - 71	0.0	0.0 - 0.0	5.9	1.9 - 11.8	7.3	2.1 - 20.5	11.4	4.0 - 23.9	0.0	0.0 - 0.0	14.1	4.7 - 29.7	0.28
3150102	8600	SALACOA CR	243.8	39.0	11 - 101	0.4	0.1 - 1.0	7.7	2.0 - 19.5	8.8	2.4 - 22.4	6.7	2.1 - 17.0	0.0	0.0 - 0.0	15.4	4.1 - 37.8	0.30
3150102	8601	LICK CR	38.3	49.4	16 - 86	0.0	0.0 - 0.0	7.5	2.4 - 15.2	14.5	4.6 - 32.9	11.8	3.9 - 24.9	0.0	0.0 - 0.0	15.7	5.3 - 34.8	0.30
3150102	8602	COOSAWATTEE R	182.9	44.6	14 - 94	0.0	0.0 - 0.0	7.2	2.2 - 13.7	9.7	2.6 - 28.4	9.7	3.4 - 25.7	0.0	0.0 - 0.0	18.0	5.7 - 45.2	0.31
3150102	8603	SCARECORN CR	73.8	59.3	16 - 134	0.0	0.0 - 0.0	9.5	3.1 - 21.1	26.7	6.7 - 74.7	9.7	2.6 - 27.9	0.0	0.0 - 0.0	13.3	4.3 - 31.4	0.20
3150102	8604	TALKING ROCK C	235.3	50.3	16 - 90	0.0	0.0 - 0.0	10.3	3.2 - 21.3	18.8	5.6 - 47.3	5.0	1.6 - 12.7	0.0	0.0 - 0.0	16.1	5.0 - 33.9	0.20
3150102	8605	COOSAWATTEE R	45.3	533.4	177 - 1478	454.1	147.6 - 1286.9	29.0	9.1 - 73.0	25.2	7.6 - 75.1	4.6	1.7 - 14.7	0.0	0.0 - 0.0	20.5	6.9 - 53.1	0.20
3150102	8606	CARTECAY R	53.3	53.3	22 - 119	0.0	0.0 - 0.0	13.6	5.5 - 31.5	18.2	5.8 - 54.9	3.4	1.2 - 8.8	0.0	0.0 - 0.0	18.0	7.3 - 46.2	0.20
3150102	8607	CLEAR CR	64.1	49.6	17 - 113	0.0	0.0 - 0.0	9.2	3.2 - 21.3	20.8	6.6 - 57.5	3.8	1.3 - 10.6	0.0	0.0 - 0.0	15.7	5.3 - 42.9	0.19
3150102	8608	CARTECAY R	233.5	50.8	13 - 128	0.0	0.0 - 0.0	8.4	2.2 - 22.2	21.9	4.9 - 66.3	4.0	1.0 - 10.6	0.0	0.0 - 0.0	16.4	4.8 - 47.1	0.19
3150102	8609	ELLIJAY R	121.5	54.0	19 - 114	0.0	0.0 - 0.0	13.1	4.4 - 30.1	19.1	5.2 - 55.8	3.6	1.4 - 10.3	0.0	0.0 - 0.0	18.1	7.1 - 42.5	0.20
3150102	8610	ELLIJAY R	75.3	36.4	12 - 85	0.0	0.0 - 0.0	13.4	4.1 - 30.9	5.5	1.7 - 13.9	1.7	0.4 - 4.9	0.0	0.0 - 0.0	15.8	5.0 - 38.8	0.19
3150102	8611	BOARDTOWN CR	45.7	43.4	18 - 104	0.0	0.0 - 0.0	6.7	2.7 - 16.2	15.0	5.7 - 45.2	3.0	1.3 - 8.6	0.0	0.0 - 0.0	18.6	7.0 - 55.0	0.19
3150102	8612	MOUNTAINTOWN	105.4	49.1	16 - 121	0.0	0.0 - 0.0	10.7	3.2 - 24.2	17.8	5.7 - 58.3	3.3	1.0 - 10.6	0.0	0.0 - 0.0	17.4	6.0 - 43.3	0.20
3150102	8613	E MOUNTAINTOW	38.5	31.2	12 - 85	0.0	0.0 - 0.0	3.2	1.1 - 8.2	7.4	2.6 - 23.8	1.5	0.5 - 5.5	0.0	0.0 - 0.0	19.1	7.7 - 57.0	0.19
3150102	8614	MOUNTAINTOWN	45.6	30.0	12 - 51	0.0	0.0 - 0.0	2.6	0.8 - 4.8	6.8	2.3 - 15.9	1.2	0.4 - 3.1	0.0	0.0 - 0.0	19.4	7.3 - 35.9	0.19
3150102	8615	TALIS CR	44.6	35.5	14 - 80	0.0	0.0 - 0.0	9.2	3.2 - 22.1	7.6	2.6 - 21.2	1.2	0.5 - 3.5	0.0	0.0 - 0.0	17.5	6.3 - 45.7	0.20
3150102	8616	COOSAWATTEE R	31.0	63.5	19 - 141	0.0	0.0 - 0.0	11.5	3.7 - 27.8	29.8	8.2 - 76.9	5.5	1.8 - 14.5	0.0	0.0 - 0.0	16.7	5.5 - 40.3	0.20
3150103	8617	OOSTANAULA R	209.4	68.1	20 - 121	0.0	0.0 - 0.0	23.6	6.9 - 49.9	7.9	2.0 - 17.8	17.3	4.9 - 39.8	0.0	0.0 - 0.0	19.3	6.7 - 43.4	0.33
3150103	8618	OOSTANAULA R	75.0	47.7	13 - 86	0.0	0.0 - 0.0	6.0	1.7 - 11.6	10.2	3.0 - 21.8	16.7	4.8 - 39.3	0.0	0.0 - 0.0	14.8	4.5 - 36.7	0.32
3150103	8619	OOSTANAULA R	133.0	78.0	29 - 145	0.0	0.0 - 0.0	9.7	3.6 - 20.1	27.2	8.3 - 71.8	22.7	8.7 - 47.5	0.0	0.0 - 0.0	18.4	5.9 - 43.5	0.32
3150103	8620	OOSTANAULA R	25.4	85.5	31 - 170	0.0	0.0 - 0.0	11.4	3.9 - 22.3	29.8	9.9 - 81.6	25.1	9.5 - 61.1	0.0	0.0 - 0.0	19.2	6.9 - 42.0	0.31

8-digit HUC	ID	Name	Area	Catchment Yield		Point sources		Developed Land		Manure		Agricultural Land		Phosphate Mines		Soil parent rock		Frac
				kg/km^2	90% CI	kg/km^2	90% CI	kg/km^2	90% CI	kg/km^2	90% CI	kg/km^2	90% CI	kg/km^2	90% CI	kg/km^2	90% CI	
3150103	8621	OOTHKALOOGA C	168.7	84.3	31 - 167	8.5	3.2 - 17.4	25.5	8.8 - 53.1	16.4	5.5 - 38.9	16.6	6.2 - 43.5	0.0	0.0 - 0.0	17.4	6.8 - 38.7	0.31
3150103	8622	OOSTANAULA R	89.9	768.9	263 - 1922	675.0	236.0 - 1752.1	26.3	8.5 - 65.1	25.2	7.0 - 69.6	21.8	7.3 - 66.5	0.0	0.0 - 0.0	20.7	5.9 - 52.1	0.31
3150103	8623	*A	44.6	62.6	24 - 137	0.0	0.0 - 0.0	6.9	2.3 - 14.9	22.0	7.5 - 63.2	18.9	6.5 - 46.5	0.0	0.0 - 0.0	14.8	5.2 - 34.2	0.31
3150103	8624	JOHNS CR	116.5	22.1	7 - 46	0.0	0.0 - 0.0	2.1	0.7 - 4.9	3.9	1.2 - 11.3	6.1	1.9 - 15.1	0.0	0.0 - 0.0	10.0	3.5 - 24.6	0.32
3150103	8625	ARMUCHEE CR	71.4	33.2	12 - 82	0.0	0.0 - 0.0	8.6	2.8 - 20.2	4.1	1.4 - 11.3	9.8	3.5 - 24.7	0.0	0.0 - 0.0	10.7	4.0 - 23.9	0.32
3150103	8626	ARMUCHEE CR	295.8	29.7	10 - 65	0.0	0.0 - 0.0	3.9	1.3 - 8.2	5.0	1.4 - 13.1	10.2	3.5 - 22.3	0.0	0.0 - 0.0	10.6	3.3 - 24.6	0.31
3150103	8627	LITTLE ARMUCHE	4.5	57.8	19 - 107	0.0	0.0 - 0.0	11.0	3.3 - 22.3	10.2	2.8 - 27.8	25.3	7.9 - 58.9	0.0	0.0 - 0.0	11.3	3.9 - 25.5	0.31
3150103	8628	LITTLE ARMUCHE	146.6	27.4	9 - 46	0.0	0.0 - 0.0	5.4	1.9 - 10.4	2.5	0.8 - 5.8	10.1	3.2 - 25.3	0.0	0.0 - 0.0	9.4	3.3 - 20.3	0.31
3150103	8629	HEATH CR	62.2	17.3	5 - 31	0.0	0.0 - 0.0	2.9	0.8 - 5.9	1.8	0.5 - 5.0	4.2	1.3 - 10.1	0.0	0.0 - 0.0	8.4	2.6 - 18.5	0.31
3150104	8630	ETOWAH R	216.0	64.6	23 - 115	0.0	0.0 - 0.0	28.0	8.1 - 51.3	5.7	1.6 - 14.6	13.4	4.6 - 28.4	0.0	0.0 - 0.0	17.6	6.8 - 37.2	0.33
3150104	8631	SPRING CR	97.6	51.6	17 - 113	0.0	0.0 - 0.0	8.3	2.9 - 17.3	8.8	2.3 - 25.8	18.2	5.0 - 50.0	0.0	0.0 - 0.0	16.4	5.3 - 38.2	0.32
3150104	8632	ETOWAH R	135.7	51.0	21 - 113	0.0	0.0 - 0.0	8.5	3.0 - 21.6	9.0	3.3 - 29.7	16.5	6.1 - 39.0	0.0	0.0 - 0.0	17.0	6.7 - 45.7	0.32
3150104	8633	ETOWAH R	191.1	50.0	15 - 81	0.0	0.0 - 0.0	11.4	3.3 - 19.0	9.0	2.5 - 24.5	14.5	4.3 - 37.4	0.0	0.0 - 0.0	15.1	5.1 - 31.6	0.32
3150104	8634	EUHARLEE CR	68.6	55.8	20 - 97	0.0	0.0 - 0.0	10.0	3.5 - 19.9	12.7	4.0 - 30.8	21.6	7.3 - 55.4	0.0	0.0 - 0.0	11.6	4.0 - 24.4	0.32
3150104	8635	EUHARLEE CR	316.9	55.4	17 - 132	6.8	2.1 - 16.4	11.7	3.8 - 27.4	7.3	2.0 - 18.0	16.3	5.4 - 42.1	0.0	0.0 - 0.0	13.3	4.2 - 30.1	0.31
3150104	8636	HILLS CR	71.9	21.7	6 - 51	0.0	0.0 - 0.0	3.4	0.8 - 7.1	3.3	0.8 - 9.3	7.2	2.0 - 18.5	0.0	0.0 - 0.0	7.9	2.1 - 21.2	0.31
3150104	8637	ETOWAH R	13.3	59.7	23 - 122	0.0	0.0 - 0.0	22.0	8.2 - 49.9	9.9	3.0 - 28.7	15.7	5.6 - 43.3	0.0	0.0 - 0.0	12.1	4.6 - 29.9	0.32
3150104	8638	RACCOON CR	143.2	18.5	7 - 34	0.0	0.0 - 0.0	3.2	1.0 - 6.8	2.8	0.8 - 8.4	5.0	1.8 - 11.0	0.0	0.0 - 0.0	7.5	2.7 - 14.9	0.32
3150104	8639	ETOWAH R	187.4	329.2	118 - 681	258.6	96.1 - 598.7	33.0	10.0 - 75.2	8.3	2.2 - 22.4	13.6	5.1 - 31.5	0.0	0.0 - 0.0	15.7	5.4 - 36.6	0.32
3150104	8640	ETOWAH R	36.1	36.2	12 - 74	0.0	0.0 - 0.0	10.5	3.5 - 21.0	6.1	1.6 - 16.6	10.7	3.4 - 26.3	0.0	0.0 - 0.0	8.9	3.0 - 20.8	0.31
3150104	8641	PUMPKINVINE CR	567.7	43.8	14 - 114	1.3	0.4 - 3.1	27.1	8.4 - 65.2	1.4	0.5 - 3.7	3.3	1.0 - 8.1	0.0	0.0 - 0.0	10.7	3.6 - 29.8	0.31
3150104	8642	ETOWAH R	35.1	89.9	34 - 202	35.7	14.8 - 82.0	31.2	11.6 - 79.1	4.1	1.2 - 14.3	6.3	2.0 - 19.5	0.0	0.0 - 0.0	12.5	5.2 - 28.3	0.31
3150104	8643	LITTLE R	109.6	72.4	20 - 183	1.7	0.5 - 4.0	45.4	12.9 - 112.3	3.5	1.0 - 10.1	4.7	1.3 - 12.7	0.0	0.0 - 0.0	17.1	4.8 - 42.5	0.13
3150104	8644	COPPER SANDY CI	63.2	65.8	20 - 127	0.0	0.0 - 0.0	42.6	11.9 - 80.5	2.6	0.8 - 8.0	5.9	2.0 - 15.9	0.0	0.0 - 0.0	14.6	4.5 - 30.5	0.12
3150104	8645	LITTLE R	54.3	53.3	20 - 147	0.0	0.0 - 0.0	12.1	4.2 - 34.8	12.4	3.9 - 38.9	11.4	4.3 - 30.9	0.0	0.0 - 0.0	17.4	6.9 - 48.0	0.12
3150104	8646	CANTON CR	54.6	67.2	19 - 139	0.0	0.0 - 0.0	27.3	8.0 - 60.8	12.0	3.3 - 31.8	9.3	2.7 - 22.9	0.0	0.0 - 0.0	18.6	6.3 - 39.7	0.13
3150104	8647	ETOWAH R	88.6	48.7	16 - 124	0.0	0.0 - 0.0	20.3	5.9 - 51.9	7.2	2.1 - 22.4	5.6	1.9 - 17.3	0.0	0.0 - 0.0	15.6	5.3 - 40.8	0.13
3150104	8648	ETOWAH R	67.5	55.7	15 - 115	0.0	0.0 - 0.0	16.0	4.3 - 32.0	12.3	3.2 - 34.3	9.5	2.9 - 26.1	0.0	0.0 - 0.0	17.9	4.3 - 42.3	0.13
3150104	8649	ETOWAH R	74.5	42.3	16 - 93	0.0	0.0 - 0.0	9.7	3.7 - 20.2	9.9	3.3 - 28.1	7.2	2.4 - 17.6	0.0	0.0 - 0.0	15.5	5.7 - 39.8	0.13
3150104	8650	SETTINGDOWN CI	130.9	75.9	24 - 181	0.0	0.0 - 0.0	23.9	7.4 - 61.6	20.6	5.8 - 59.4	13.9	4.4 - 43.2	0.0	0.0 - 0.0	17.5	5.2 - 42.8	0.12
3150104	8651	ETOWAH R	119.7	41.0	12 - 85	0.0	0.0 - 0.0	8.4	2.4 - 17.4	12.9	3.4 - 37.5	7.0	1.8 - 19.0	0.0	0.0 - 0.0	12.6	3.8 - 26.6	0.12
3150104	8652	ETOWAH R	6.9	14.8	5 - 37	0.0	0.0 - 0.0	6.0	2.0 - 14.6	0.5	0.2 - 1.6	0.1	0.0 - 0.4	0.0	0.0 - 0.0	8.2	2.9 - 22.5	0.12
3150104	8653	ETOWAH R	358.6	38.7	13 - 92	0.0	0.0 - 0.0	9.1	2.9 - 20.5	8.7	2.9 - 24.9	3.8	1.3 - 10.7	0.0	0.0 - 0.0	17.1	6.1 - 47.8	0.12
3150104	8654	SHOAL CR	96.4	35.3	11 - 80	0.0	0.0 - 0.0	12.1	3.2 - 28.6	9.8	2.5 - 30.9	3.8	1.1 - 9.5	0.0	0.0 - 0.0	9.6	3.3 - 24.1	0.12
3150104	8655	AMICALOLA CR	55.3	32.1	10 - 62	0.0	0.0 - 0.0	9.3	2.8 - 20.4	8.9	2.5 - 21.5	3.6	1.2 - 8.8	0.0	0.0 - 0.0	10.3	3.3 - 21.9	0.12
3150104	8656	COCHRANS CR	78.8	44.3	16 - 86	0.0	0.0 - 0.0	8.1	2.9 - 15.8	16.9	5.2 - 54.0	6.7	2.4 - 18.2	0.0	0.0 - 0.0	12.5	4.3 - 25.8	0.12
3150104	8657	AMICALOLA CR	14.2	29.1	10 - 60	0.0	0.0 - 0.0	9.4	3.0 - 19.2	6.1	1.6 - 13.5	2.6	0.8 - 6.0	0.0	0.0 - 0.0	11.1	4.0 - 26.9	0.12
3150104	8658	LITTLE AMICALOI	34.3	31.8	10 - 75	0.0	0.0 - 0.0	8.3	2.6 - 19.3	6.0	1.6 - 16.8	2.3	0.7 - 6.6	0.0	0.0 - 0.0	15.1	5.0 - 35.1	0.11
3150104	8659	AMICALOLA CR	70.1	21.9	7 - 51	0.0	0.0 - 0.0	5.6	1.8 - 12.7	0.9	0.3 - 2.4	0.4	0.1 - 0.9	0.0	0.0 - 0.0	15.1	5.4 - 36.9	0.11

8-digit HUC	ID	Name	Area	Catchment Yield		Point sources		Developed Land		Manure		Agricultural Land		Phosphate Mines		Soil parent rock		Frac
				kg/km^2	90% CI	kg/km^2	90% CI	kg/km^2	90% CI	kg/km^2	90% CI	kg/km^2	90% CI	kg/km^2	90% CI	kg/km^2	90% CI	
3150104	8660	LONG SWAMP CR	60.5	36.5	13 - 63	0.0	0.0 - 0.0	9.6	3.3 - 19.5	8.2	2.7 - 20.1	3.8	1.4 - 9.3	0.0	0.0 - 0.0	14.9	5.4 - 33.1	0.13
3150104	8661	LONG SWAMP CR,	62.0	36.8	12 - 70	0.0	0.0 - 0.0	13.6	4.2 - 26.2	5.7	1.7 - 16.1	1.9	0.6 - 4.0	0.0	0.0 - 0.0	15.6	5.5 - 38.3	0.12
3150104	8662	LONG SWAMP CR	76.9	38.0	12 - 85	0.0	0.0 - 0.0	13.1	3.8 - 33.6	5.4	1.6 - 16.2	2.0	0.6 - 6.1	0.0	0.0 - 0.0	17.5	5.9 - 48.5	0.12
3150104	8663	MOUNTAIN CR	35.1	46.2	15 - 77	0.0	0.0 - 0.0	17.5	5.4 - 33.8	7.1	2.1 - 19.8	5.7	1.9 - 12.4	0.0	0.0 - 0.0	15.9	5.6 - 31.2	0.13
3150104	8664	MOUNTAIN CR	23.2	47.6	14 - 105	0.0	0.0 - 0.0	21.6	6.1 - 48.8	7.1	1.9 - 20.4	3.3	0.9 - 8.8	0.0	0.0 - 0.0	15.6	3.9 - 38.5	0.12
3150104	8665	MOUNTAIN CR	5.9	70.8	24 - 149	0.0	0.0 - 0.0	14.2	4.7 - 29.8	31.1	9.5 - 75.3	12.3	4.6 - 27.2	0.0	0.0 - 0.0	13.2	4.8 - 27.7	0.11
3150104	8666	MOUNTAIN CR	17.5	99.4	35 - 231	31.1	10.2 - 71.0	28.8	10.1 - 69.6	18.4	5.8 - 62.8	6.8	2.2 - 18.7	0.0	0.0 - 0.0	14.3	4.7 - 35.0	0.11
3150104	8667	SHARP CR	56.7	59.4	22 - 111	0.0	0.0 - 0.0	22.0	8.2 - 42.2	17.1	5.5 - 43.0	6.0	2.1 - 14.0	0.0	0.0 - 0.0	14.3	5.4 - 33.1	0.11
3150104	8668	ROCK CR	24.2	39.6	12 - 95	0.0	0.0 - 0.0	9.0	2.8 - 20.9	11.7	3.2 - 38.5	4.6	1.3 - 12.7	0.0	0.0 - 0.0	14.2	4.6 - 33.7	0.11
3150104	8669	BLUFF CR	33.1	33.4	13 - 85	0.0	0.0 - 0.0	11.0	3.7 - 27.1	5.3	1.6 - 17.1	3.7	1.4 - 10.6	0.0	0.0 - 0.0	13.4	4.5 - 37.9	0.12
3150104	8670	SHOAL CR	175.2	26.8	7 - 59	0.0	0.0 - 0.0	8.1	2.2 - 17.9	3.6	1.0 - 9.9	2.7	0.9 - 6.5	0.0	0.0 - 0.0	12.3	3.9 - 28.3	0.13
3150104	8671	ETOWAH R	36.3	323.1	90 - 821	280.9	77.8 - 724.8	22.2	5.7 - 48.9	2.9	0.7 - 9.2	2.1	0.7 - 6.1	0.0	0.0 - 0.0	15.0	3.8 - 38.7	0.13
3150105	8672	COOSA R	79.7	93.2	36 - 187	18.5	7.0 - 41.5	5.7	1.8 - 11.4	9.6	3.1 - 23.7	37.4	13.3 - 93.2	0.0	0.0 - 0.0	22.0	8.6 - 51.0	0.49
3150105	8673	TERRAPIN CR	329.1	40.7	16 - 76	0.0	0.0 - 0.0	6.3	2.3 - 12.5	4.0	1.4 - 12.0	15.2	6.3 - 43.3	0.0	0.0 - 0.0	15.3	6.1 - 36.2	0.49
3150105	8674	NANCES CR	71.9	70.9	33 - 114	20.3	8.3 - 37.3	12.9	5.0 - 22.8	5.8	2.2 - 13.7	16.4	6.8 - 34.3	0.0	0.0 - 0.0	15.5	6.7 - 30.2	0.47
3150105	8675	TERRAPIN CR	152.3	22.4	7 - 46	0.0	0.0 - 0.0	3.2	0.8 - 7.3	2.3	0.6 - 6.5	6.3	1.9 - 17.5	0.0	0.0 - 0.0	10.7	3.2 - 28.8	0.47
3150105	8676	WALLACE CR	120.3	19.6	7 - 51	0.0	0.0 - 0.0	3.5	1.1 - 9.8	4.7	1.5 - 13.5	2.8	0.9 - 10.7	0.0	0.0 - 0.0	8.7	3.3 - 22.5	0.44
3150105	8677	LITTLE TERRAPIN	41.6	18.4	7 - 38	0.0	0.0 - 0.0	2.8	1.1 - 6.1	3.6	1.1 - 10.4	2.8	1.0 - 6.3	0.0	0.0 - 0.0	9.2	3.6 - 19.2	0.44
3150105	8678	COOSA R	26.7	92.5	31 - 220	0.0	0.0 - 0.0	26.0	8.2 - 67.4	8.5	2.6 - 20.8	35.6	11.2 - 83.1	0.0	0.0 - 0.0	22.4	7.9 - 62.3	0.49
3150105	8679	CEDAR CR	149.0	39.7	12 - 83	1.3	0.4 - 3.2	7.0	2.0 - 14.8	4.5	1.1 - 12.8	9.6	2.9 - 23.1	0.0	0.0 - 0.0	17.3	5.1 - 41.0	0.33
3150105	8680	CEDAR CR	142.4	112.0	43 - 220	54.9	21.1 - 120.5	23.4	7.9 - 43.4	5.5	1.7 - 13.8	12.4	4.2 - 28.9	0.0	0.0 - 0.0	15.8	5.6 - 34.4	0.31
3150105	8681	CEDAR CR	109.5	40.8	13 - 66	0.0	0.0 - 0.0	8.9	3.0 - 16.5	5.8	1.7 - 15.8	13.0	4.4 - 34.1	0.0	0.0 - 0.0	13.1	4.4 - 29.1	0.28
3150105	8682	*A	56.8	43.8	15 - 83	0.0	0.0 - 0.0	8.2	2.9 - 15.9	6.6	2.1 - 16.6	15.4	5.2 - 35.6	0.0	0.0 - 0.0	13.5	4.1 - 29.0	0.28
3150105	8683	LAKE CR	89.1	51.9	16 - 99	0.0	0.0 - 0.0	9.9	3.1 - 21.2	7.7	2.2 - 22.9	19.0	6.1 - 46.7	0.0	0.0 - 0.0	15.3	4.8 - 35.0	0.31
3150105	8684	COOSA R	163.2	60.5	20 - 152	23.1	7.3 - 65.1	6.3	2.2 - 16.3	5.0	1.5 - 13.8	11.5	3.8 - 34.9	0.0	0.0 - 0.0	14.6	4.7 - 39.0	0.33
3150105	8685	COOSA R	128.8	237.6	83 - 419	185.5	64.8 - 329.8	18.4	5.7 - 35.7	4.8	1.6 - 11.2	11.8	4.4 - 24.6	0.0	0.0 - 0.0	17.1	6.0 - 34.5	0.33
3150105	8686	BEACH CR	65.4	41.9	14 - 94	0.0	0.0 - 0.0	15.3	5.0 - 36.3	3.7	1.1 - 10.9	8.2	2.5 - 23.6	0.0	0.0 - 0.0	14.7	5.5 - 37.6	0.33
3150105	8687	CHATOOGA R	81.9	37.8	14 - 90	0.0	0.0 - 0.0	7.4	2.6 - 16.4	3.3	1.2 - 9.9	13.3	4.2 - 38.0	0.0	0.0 - 0.0	13.7	5.1 - 31.4	0.33
3150105	8688	WICKERS CR	32.3	25.0	8 - 54	0.0	0.0 - 0.0	6.4	1.9 - 13.6	1.4	0.3 - 3.1	5.3	1.8 - 13.6	0.0	0.0 - 0.0	11.9	3.6 - 29.1	0.32
3150105	8689	CHATOOGA R	148.1	59.4	21 - 110	3.8	1.2 - 6.8	14.4	5.0 - 28.4	4.4	1.3 - 11.8	18.9	7.1 - 40.5	0.0	0.0 - 0.0	17.9	5.9 - 39.5	0.32
3150105	8690	CANE CR	101.4	47.5	16 - 108	0.0	0.0 - 0.0	10.9	3.6 - 23.0	8.2	2.2 - 23.0	14.4	4.8 - 37.5	0.0	0.0 - 0.0	14.0	5.4 - 36.8	0.30
3150105	8691	CHATOOGA R	24.7	49.4	15 - 99	0.0	0.0 - 0.0	7.8	2.4 - 15.5	4.4	1.4 - 12.1	20.1	6.0 - 50.1	0.0	0.0 - 0.0	17.0	5.5 - 37.8	0.30
3150105	8692	CHATOOGA R	57.7	30.5	13 - 58	0.0	0.0 - 0.0	4.8	1.9 - 9.6	5.5	1.9 - 14.4	10.3	4.3 - 29.4	0.0	0.0 - 0.0	10.0	3.7 - 22.5	0.30
3150105	8693	CHATOOGA R	72.7	122.5	37 - 219	48.8	14.8 - 94.4	25.8	6.3 - 46.4	11.3	3.1 - 23.2	19.4	6.0 - 45.0	0.0	0.0 - 0.0	17.2	5.0 - 39.0	0.29
3150105	8694	DUCK CR	83.9	41.1	13 - 100	0.0	0.0 - 0.0	7.1	2.3 - 18.2	7.5	2.1 - 21.1	13.1	3.8 - 36.7	0.0	0.0 - 0.0	13.4	4.3 - 34.6	0.29
3150105	8695	TEOLGA CR	59.5	43.5	16 - 76	0.0	0.0 - 0.0	8.1	2.7 - 16.4	4.2	1.4 - 9.4	17.3	5.7 - 38.5	0.0	0.0 - 0.0	13.9	4.8 - 30.6	0.30
3150105	8696	MILLS CR	40.1	30.0	10 - 65	0.0	0.0 - 0.0	3.1	0.9 - 7.0	3.0	1.0 - 7.9	11.8	3.9 - 32.6	0.0	0.0 - 0.0	12.1	4.5 - 28.3	0.33
3150105	8697	MILLS CR	127.8	44.8	15 - 105	0.0	0.0 - 0.0	8.8	3.1 - 21.7	4.4	1.5 - 13.5	18.2	6.3 - 46.7	0.0	0.0 - 0.0	13.3	4.9 - 35.7	0.30
3150105	8698	CULSTIGH CR	21.1	39.2	13 - 82	0.0	0.0 - 0.0	7.8	2.5 - 17.9	3.6	1.1 - 8.3	14.3	4.4 - 32.4	0.0	0.0 - 0.0	13.6	4.8 - 30.9	0.30

8-digit HUC	ID	Name	Area	Catchment Yield		Point sources		Developed Land		Manure		Agricultural Land		Phosphate Mines		Soil parent rock		Frac
				kg/km^2	90% CI	kg/km^2	90% CI	kg/km^2	90% CI	kg/km^2	90% CI	kg/km^2	90% CI	kg/km^2	90% CI	kg/km^2	90% CI	
3150105	8699	LITTLE R	34.4	32.6	10 - 61	0.0	0.0 - 0.0	6.0	1.9 - 11.7	9.4	2.5 - 27.7	11.3	3.1 - 29.7	0.0	0.0 - 0.0	5.8	2.0 - 12.5	0.33
3150105	8700	LITTLE R	115.5	29.2	12 - 54	0.0	0.0 - 0.0	5.2	2.2 - 10.3	9.2	3.0 - 23.6	8.6	2.8 - 21.8	0.0	0.0 - 0.0	6.2	2.5 - 14.7	0.32
3150105	8701	LITTLE R	140.8	19.1	6 - 39	0.0	0.0 - 0.0	4.7	1.5 - 11.0	3.6	1.0 - 9.3	4.7	1.4 - 11.5	0.0	0.0 - 0.0	6.1	1.7 - 13.3	0.31
3150105	8702	LITTLE R, W FK	126.9	20.9	7 - 50	0.1	0.0 - 0.3	3.8	1.2 - 8.4	5.2	1.5 - 14.4	5.7	1.6 - 17.0	0.0	0.0 - 0.0	6.0	1.8 - 15.7	0.31
3150105	8703	BEAR CR	35.9	38.6	14 - 80	0.0	0.0 - 0.0	6.2	2.4 - 15.2	13.8	4.8 - 40.4	12.9	4.4 - 37.4	0.0	0.0 - 0.0	5.7	2.4 - 12.6	0.32
3150105	8704	JOHNNIE CR	53.3	40.7	14 - 101	0.0	0.0 - 0.0	7.4	2.5 - 16.5	13.8	4.6 - 43.3	14.2	4.1 - 41.3	0.0	0.0 - 0.0	5.3	1.8 - 14.8	0.33
3150106	8706	TALLADEGA CR	435.4	40.2	15 - 93	7.3	2.6 - 17.8	10.8	3.6 - 24.0	2.4	0.7 - 6.5	7.3	2.5 - 21.0	0.0	0.0 - 0.0	12.4	4.3 - 28.5	0.68
3150106	8707	COOSA R	59.8	285.8	109 - 534	227.3	86.4 - 426.3	14.5	5.3 - 28.1	3.2	1.0 - 8.0	25.1	9.4 - 59.0	0.0	0.0 - 0.0	15.6	5.6 - 32.5	0.68
3150106	8708	*A	37.7	41.5	13 - 92	0.0	0.0 - 0.0	5.3	1.6 - 10.7	4.5	1.2 - 12.7	15.8	4.8 - 44.4	0.0	0.0 - 0.0	15.8	5.1 - 33.3	0.67
3150106	8709	COOSA R	62.8	45.5	13 - 103	0.0	0.0 - 0.0	8.0	2.2 - 21.9	2.5	0.7 - 7.8	21.8	6.2 - 57.2	0.0	0.0 - 0.0	13.1	3.9 - 34.3	0.67
3150106	8710	COOSA R	289.1	38.3	16 - 80	0.0	0.0 - 0.0	11.4	4.4 - 26.3	3.5	1.2 - 8.5	9.2	3.5 - 25.9	0.0	0.0 - 0.0	14.3	5.2 - 34.0	0.67
3150106	8711	CHOCCOLOCCO CI	87.7	53.8	15 - 94	0.0	0.0 - 0.0	14.0	3.9 - 27.8	4.6	1.1 - 12.9	17.7	4.8 - 32.4	0.0	0.0 - 0.0	17.5	5.1 - 34.5	0.58
3150106	8712	CHOCCOLOCCO CI	15.5	53.6	20 - 143	0.0	0.0 - 0.0	5.5	1.9 - 13.0	6.2	1.7 - 22.2	25.8	8.3 - 64.9	0.0	0.0 - 0.0	16.1	5.7 - 46.2	0.57
3150106	8713	CHEAHA CR	16.3	66.2	26 - 139	0.0	0.0 - 0.0	7.3	2.6 - 16.0	7.6	2.7 - 23.0	31.5	10.7 - 83.8	0.0	0.0 - 0.0	19.8	7.9 - 46.0	0.56
3150106	8714	KELLY CR	85.0	54.0	19 - 135	0.0	0.0 - 0.0	11.9	4.5 - 24.7	5.1	1.4 - 15.0	20.9	7.2 - 55.7	0.0	0.0 - 0.0	16.1	6.2 - 43.6	0.55
3150106	8715	CHEAHA CR	192.9	24.4	9 - 43	0.0	0.0 - 0.0	5.2	1.7 - 10.0	1.8	0.5 - 3.9	6.9	2.2 - 17.7	0.0	0.0 - 0.0	10.6	3.7 - 21.8	0.55
3150106	8716	CHOCCOLOCCO CI	54.5	66.7	25 - 151	0.0	0.0 - 0.0	14.3	5.0 - 31.0	6.8	2.1 - 21.3	26.3	9.5 - 63.9	0.0	0.0 - 0.0	19.2	7.5 - 46.4	0.56
3150106	8717	SALT CR	70.1	19.4	7 - 44	0.0	0.0 - 0.0	3.9	1.2 - 9.3	1.2	0.4 - 3.6	4.0	1.3 - 10.5	0.0	0.0 - 0.0	10.2	3.8 - 28.9	0.55
3150106	8718	CHOCCOLOCCO CI	276.0	128.8	47 - 291	64.0	22.2 - 149.4	40.2	13.0 - 94.7	2.5	0.7 - 6.9	7.8	2.5 - 23.4	0.0	0.0 - 0.0	14.3	4.7 - 35.6	0.55
3150106	8719	HILLABEE CR	55.4	14.2	6 - 41	0.0	0.0 - 0.0	3.4	1.2 - 9.7	0.3	0.1 - 1.2	0.6	0.3 - 2.2	0.0	0.0 - 0.0	9.8	3.8 - 30.1	0.53
3150106	8720	CHOCCOLOCCO CI	155.7	42.8	18 - 98	0.0	0.0 - 0.0	8.8	3.3 - 20.9	5.1	1.7 - 13.7	14.1	5.2 - 37.4	0.0	0.0 - 0.0	14.9	5.6 - 35.2	0.53
3150106	8721	SHOAL CR	100.5	5.6	2 - 11	0.0	0.0 - 0.0	1.3	0.4 - 2.9	0.1	0.0 - 0.4	0.3	0.1 - 0.7	0.0	0.0 - 0.0	3.9	1.4 - 9.1	0.49
3150106	8722	CHOCCOLOCCO CI	140.9	27.1	10 - 70	0.0	0.0 - 0.0	3.4	1.3 - 8.3	3.1	1.1 - 9.0	8.9	3.1 - 26.9	0.0	0.0 - 0.0	11.6	4.9 - 30.3	0.49
3150106	8723	COOSA R	65.6	91.9	36 - 165	31.5	12.2 - 65.9	27.5	10.9 - 55.4	5.6	1.7 - 17.0	11.8	4.0 - 29.1	0.0	0.0 - 0.0	15.5	6.3 - 35.3	0.58
3150106	8724	BLUE EYE CR	77.2	75.6	29 - 145	14.7	5.7 - 36.3	18.6	7.2 - 38.4	5.4	1.8 - 14.1	20.6	6.8 - 56.7	0.0	0.0 - 0.0	16.4	5.4 - 37.7	0.58
3150106	8725	COOSA R	43.7	46.2	16 - 99	0.0	0.0 - 0.0	11.5	3.9 - 22.9	4.3	1.3 - 10.7	13.4	4.8 - 33.4	0.0	0.0 - 0.0	16.9	5.5 - 39.5	0.58
3150106	8726	COOSA R	69.0	31.8	10 - 74	0.0	0.0 - 0.0	4.3	1.4 - 9.1	4.6	1.3 - 13.0	11.5	3.4 - 34.9	0.0	0.0 - 0.0	11.5	3.6 - 29.5	0.58
3150106	8727	COOSA R	35.9	43.4	14 - 85	0.0	0.0 - 0.0	6.2	2.1 - 13.1	6.5	1.8 - 18.2	17.7	5.8 - 43.9	0.0	0.0 - 0.0	13.0	4.0 - 29.0	0.57
3150106	8728	CANE CR	86.5	26.6	9 - 54	0.0	0.0 - 0.0	5.7	2.0 - 13.0	2.3	0.7 - 6.6	5.6	1.7 - 16.1	0.0	0.0 - 0.0	12.9	4.6 - 29.2	0.57
3150106	8729	*B	27.6	44.0	16 - 109	0.0	0.0 - 0.0	30.6	11.4 - 76.1	0.5	0.2 - 1.7	0.6	0.2 - 1.6	0.0	0.0 - 0.0	12.3	4.1 - 32.0	0.52
3150106	8730	CANE CR	126.7	60.1	20 - 131	9.3	3.4 - 24.0	29.5	9.6 - 64.6	2.4	0.8 - 6.7	5.9	2.1 - 14.4	0.0	0.0 - 0.0	13.1	4.3 - 35.4	0.52
3150106	8731	COOSA R	16.2	39.8	10 - 96	0.0	0.0 - 0.0	4.8	1.3 - 13.0	6.6	1.6 - 19.0	17.3	4.5 - 53.9	0.0	0.0 - 0.0	11.1	3.1 - 31.0	0.57
3150106	8732	TALLASSEEHATCI	18.9	31.6	9 - 55	0.0	0.0 - 0.0	8.3	2.5 - 17.3	3.5	0.9 - 8.5	8.7	2.5 - 20.9	0.0	0.0 - 0.0	11.0	3.4 - 23.7	0.57
3150106	8733	TALLASSEEHATCI	27.9	34.7	10 - 65	0.0	0.0 - 0.0	5.9	1.8 - 13.2	4.7	1.3 - 14.6	12.3	3.4 - 26.9	0.0	0.0 - 0.0	11.8	3.5 - 31.2	0.56
3150106	8734	*C	47.8	72.3	27 - 137	0.0	0.0 - 0.0	15.3	5.7 - 32.4	10.5	3.6 - 27.0	30.0	10.5 - 72.0	0.0	0.0 - 0.0	16.5	5.9 - 39.1	0.55
3150106	8735	TALLASSEEHATCI	38.4	55.4	17 - 125	0.0	0.0 - 0.0	9.8	2.9 - 20.0	8.2	2.2 - 19.4	24.0	7.0 - 71.0	0.0	0.0 - 0.0	13.3	4.0 - 32.0	0.55
3150106	8736	TALLASSEEHATCI	38.1	49.5	16 - 116	0.0	0.0 - 0.0	12.3	4.0 - 28.0	5.9	1.5 - 15.8	15.7	4.8 - 43.6	0.0	0.0 - 0.0	15.7	4.8 - 35.3	0.52
3150106	8737	*D	59.4	38.5	11 - 78	0.0	0.0 - 0.0	14.5	4.9 - 32.6	2.9	0.9 - 8.8	7.2	2.5 - 17.8	0.0	0.0 - 0.0	13.8	4.5 - 30.1	0.49
3150106	8738	TALLASSEEHATCI	134.0	64.7	22 - 157	19.4	7.4 - 45.8	19.2	6.8 - 44.7	3.4	1.1 - 9.6	9.9	3.5 - 25.8	0.0	0.0 - 0.0	12.7	5.0 - 33.1	0.49

8-digit HUC	ID	Name	Area	Catchment Yield kg/km^2	Catchment Yield 90% CI	Point sources kg/km^2	Point sources 90% CI	Developed Land kg/km^2	Developed Land 90% CI	Manure kg/km^2	Manure 90% CI	Agricultural Land kg/km^2	Agricultural Land 90% CI	Phosphate Mines kg/km^2	Phosphate Mines 90% CI	Soil parent rock kg/km^2	Soil parent rock 90% CI	Frac
3150106	8739	*E	35.0	36.0	12 - 92	0.0	0.0 - 0.0	7.8	2.6 - 18.0	4.8	1.6 - 14.0	14.3	4.6 - 44.2	0.0	0.0 - 0.0	9.1	3.0 - 23.8	0.52
3150106	8740	OHATCHEE	206.1	32.9	11 - 65	0.0	0.0 - 0.0	7.3	2.6 - 12.8	3.9	1.2 - 10.2	10.8	3.2 - 23.4	0.0	0.0 - 0.0	10.8	3.5 - 25.3	0.56
3150106	8741	COOSA R	45.6	16.9	7 - 43	0.0	0.0 - 0.0	4.0	1.6 - 11.2	1.8	0.6 - 6.8	3.6	1.4 - 11.0	0.0	0.0 - 0.0	7.5	3.0 - 19.3	0.57
3150106	8742	COOSA R	30.3	31.0	9 - 57	0.0	0.0 - 0.0	9.2	2.5 - 17.4	3.1	0.9 - 7.1	7.9	2.3 - 18.2	0.0	0.0 - 0.0	10.9	3.5 - 26.5	0.50
3150106	8743	COOSA R	31.3	45.2	16 - 113	0.0	0.0 - 0.0	12.9	4.4 - 30.6	6.9	2.2 - 26.3	13.9	4.9 - 41.7	0.0	0.0 - 0.0	11.5	4.3 - 30.0	0.50
3150106	8744	COOSA R	97.7	275.8	96 - 647	179.5	59.6 - 458.7	29.7	10.1 - 79.4	13.4	4.0 - 42.0	28.0	8.9 - 71.0	0.0	0.0 - 0.0	25.2	8.7 - 59.9	0.50
3150106	8745	COOSA R	65.8	89.7	37 - 168	1.4	0.6 - 3.1	54.7	20.1 - 109.3	5.5	1.8 - 15.9	11.2	3.9 - 28.2	0.0	0.0 - 0.0	16.8	6.4 - 39.7	0.50
3150106	8746	BIG COVE CR	73.2	50.9	18 - 91	0.0	0.0 - 0.0	14.8	5.3 - 28.7	7.5	2.6 - 19.7	14.9	5.0 - 32.9	0.0	0.0 - 0.0	13.7	5.3 - 26.7	0.50
3150106	8747	COOSA R	201.5	67.5	26 - 178	0.0	0.0 - 0.0	9.2	3.3 - 23.3	13.2	4.1 - 39.8	27.7	9.9 - 90.1	0.0	0.0 - 0.0	17.5	7.0 - 46.5	0.50
3150106	8748	BALL PLAY CR	168.7	40.5	12 - 90	0.0	0.0 - 0.0	4.6	1.3 - 10.5	4.8	1.4 - 13.7	13.6	4.1 - 43.4	0.0	0.0 - 0.0	17.5	4.9 - 48.3	0.49
3150106	8749	COOSA R	9.7	53.6	16 - 111	0.0	0.0 - 0.0	7.6	2.0 - 16.8	5.3	1.4 - 12.1	21.0	6.1 - 46.0	0.0	0.0 - 0.0	19.6	6.0 - 47.0	0.49
3150106	8750	BIG WILLIS CR	2.8	83.3	28 - 160	0.0	0.0 - 0.0	50.3	15.0 - 97.3	1.5	0.4 - 4.4	3.4	1.3 - 10.2	0.0	0.0 - 0.0	28.1	9.2 - 67.5	0.50
3150106	8751	BLACK CR	166.9	35.3	9 - 73	0.0	0.0 - 0.0	20.3	5.1 - 43.1	3.3	1.1 - 6.8	6.3	1.5 - 15.7	0.0	0.0 - 0.0	5.3	1.6 - 10.3	0.50
3150106	8752	BIG WILLIS CR	204.8	77.3	24 - 166	22.4	8.4 - 52.2	28.7	7.4 - 60.3	5.4	1.5 - 14.6	10.0	3.2 - 23.4	0.0	0.0 - 0.0	10.7	3.7 - 26.9	0.50
3150106	8753	BIG WILLIS CR	24.3	30.9	12 - 74	0.0	0.0 - 0.0	6.8	2.3 - 14.8	5.1	1.6 - 14.7	10.9	3.8 - 28.7	0.0	0.0 - 0.0	8.1	2.8 - 19.1	0.48
3150106	8754	BIG WILLIS CR	2.0	41.6	12 - 79	0.0	0.0 - 0.0	7.4	2.1 - 15.9	8.5	2.4 - 25.6	18.3	4.7 - 42.8	0.0	0.0 - 0.0	7.4	2.5 - 17.7	0.47
3150106	8755	BIG WILLIS CR	72.3	50.2	16 - 139	0.0	0.0 - 0.0	9.3	3.6 - 27.8	14.9	5.3 - 43.5	15.6	5.6 - 56.9	0.0	0.0 - 0.0	10.4	3.9 - 27.7	0.46
3150106	8756	BIG WILLIS CR	343.2	64.7	24 - 158	14.8	5.1 - 34.0	15.3	5.4 - 40.1	13.0	4.2 - 44.5	11.8	4.1 - 29.1	0.0	0.0 - 0.0	9.9	3.9 - 22.8	0.43
3150106	8757	*F	30.7	39.9	13 - 99	0.0	0.0 - 0.0	4.6	1.4 - 12.4	15.0	4.5 - 52.0	14.0	4.2 - 35.1	0.0	0.0 - 0.0	6.4	2.1 - 17.5	0.43
3150106	8758	*G	20.4	35.1	12 - 83	0.0	0.0 - 0.0	7.2	2.4 - 16.8	7.2	2.3 - 19.5	12.1	4.2 - 37.0	0.0	0.0 - 0.0	8.6	3.1 - 20.6	0.46
3150106	8759	*H	31.0	26.6	10 - 63	0.0	0.0 - 0.0	5.4	1.7 - 13.7	4.8	1.6 - 12.0	9.8	3.6 - 28.7	0.0	0.0 - 0.0	6.6	2.4 - 16.0	0.47
3150106	8760	*I	48.0	34.6	13 - 66	0.0	0.0 - 0.0	11.8	4.0 - 24.5	5.2	1.8 - 12.9	10.7	4.0 - 26.3	0.0	0.0 - 0.0	7.0	2.6 - 15.9	0.48
3150106	8761	BIG CANOE CR	132.7	47.9	16 - 100	0.0	0.0 - 0.0	9.5	3.1 - 21.5	7.3	2.1 - 20.8	14.7	5.3 - 44.2	0.0	0.0 - 0.0	16.4	5.4 - 36.4	0.50
3150106	8762	LITTLE CANOE CR	85.7	36.1	14 - 91	0.0	0.0 - 0.0	5.6	2.0 - 14.3	6.2	1.8 - 20.0	12.8	4.2 - 39.2	0.0	0.0 - 0.0	11.4	4.1 - 31.5	0.49
3150106	8763	BIG CANOE CR	135.1	48.9	15 - 114	0.0	0.0 - 0.0	9.0	2.9 - 21.1	8.3	2.4 - 21.2	17.0	5.8 - 44.7	0.0	0.0 - 0.0	14.5	4.4 - 38.4	0.49
3150106	8764	BIG CANOE CR	127.0	32.3	10 - 62	0.0	0.0 - 0.0	4.8	1.5 - 9.9	5.7	1.7 - 15.8	10.9	3.3 - 28.4	0.0	0.0 - 0.0	10.8	3.6 - 27.2	0.46
3150106	8765	*J	111.5	34.6	10 - 60	0.0	0.0 - 0.0	9.2	2.7 - 18.6	4.7	1.2 - 12.4	8.8	2.5 - 21.3	0.0	0.0 - 0.0	11.9	3.6 - 26.9	0.46
3150106	8766	BEAVER CR	93.7	43.1	16 - 96	0.0	0.0 - 0.0	6.8	2.3 - 17.2	8.0	2.7 - 23.0	15.8	5.6 - 47.5	0.0	0.0 - 0.0	12.6	4.9 - 33.7	0.50
3150106	8767	SHOAL CR	86.0	27.9	9 - 72	0.0	0.0 - 0.0	2.7	0.8 - 7.2	5.3	1.6 - 13.7	10.7	3.5 - 29.3	0.0	0.0 - 0.0	9.2	3.4 - 24.7	0.50
3150106	8768	TROUT CR	75.3	18.9	7 - 50	0.3	0.1 - 0.9	4.8	1.7 - 11.5	2.7	0.8 - 8.5	4.6	1.6 - 14.8	0.0	0.0 - 0.0	6.5	2.3 - 19.3	0.57
3150106	8769	BROKEN ARROW (	142.2	20.4	7 - 37	0.0	0.0 - 0.0	3.8	1.3 - 7.2	3.6	1.2 - 8.4	6.1	2.0 - 13.3	0.0	0.0 - 0.0	6.9	2.4 - 14.9	0.58
3150106	8770	KELLEY CR	37.2	36.8	11 - 74	0.0	0.0 - 0.0	6.5	2.0 - 16.0	4.7	1.3 - 15.1	12.5	4.1 - 29.5	0.0	0.0 - 0.0	13.1	4.4 - 28.7	0.67
3150106	8771	KELLEY CR	19.3	13.2	4 - 26	0.0	0.0 - 0.0	2.7	0.8 - 5.3	1.3	0.4 - 3.6	2.7	1.0 - 6.8	0.0	0.0 - 0.0	6.4	2.2 - 14.4	0.62
3150106	8772	*K	47.4	19.4	5 - 39	0.0	0.0 - 0.0	6.4	2.0 - 14.4	2.7	0.7 - 6.9	4.9	1.4 - 12.5	0.0	0.0 - 0.0	5.4	1.4 - 13.3	0.58
3150106	8773	KELLEY CR	80.3	44.6	17 - 80	0.2	0.1 - 0.4	12.2	4.2 - 25.1	7.2	2.0 - 20.7	14.7	5.5 - 36.5	0.0	0.0 - 0.0	10.3	3.8 - 24.0	0.58
3150106	8774	SHOAL CR	82.1	13.8	5 - 26	0.0	0.0 - 0.0	4.5	1.6 - 8.7	0.6	0.2 - 1.8	3.3	1.2 - 7.8	0.0	0.0 - 0.0	5.3	2.0 - 12.5	0.62
3150106	8775	EASTRABOGA CR	73.5	75.3	28 - 145	0.0	0.0 - 0.0	34.6	12.3 - 79.5	5.4	1.7 - 14.4	19.7	7.1 - 47.9	0.0	0.0 - 0.0	15.6	5.8 - 35.3	0.57
3150107	8776	COOSA R	34.3	150.8	56 - 276	103.7	36.1 - 206.3	13.3	4.2 - 25.6	1.9	0.6 - 5.2	12.9	3.9 - 32.1	0.0	0.0 - 0.0	19.0	7.2 - 38.1	0.79
3150107	8777	LITTLE WEOKA CI	35.3	18.0	6 - 41	0.0	0.0 - 0.0	5.1	1.6 - 10.5	1.2	0.4 - 3.2	3.8	1.3 - 9.6	0.0	0.0 - 0.0	8.0	2.6 - 18.4	0.71

8-digit HUC	ID	Name	Area	Catchment Yield kg/km^2	Catchment Yield 90% CI	Point sources kg/km^2	Point sources 90% CI	Developed Land kg/km^2	Developed Land 90% CI	Manure kg/km^2	Manure 90% CI	Agricultural Land kg/km^2	Agricultural Land 90% CI	Phosphate Mines kg/km^2	Phosphate Mines 90% CI	Soil parent rock kg/km^2	Soil parent rock 90% CI	Frac
3150107	8778	WEOKA CR	66.8	15.6	5 - 34	0.0	0.0 - 0.0	4.5	1.3 - 11.3	0.7	0.2 - 2.2	1.6	0.5 - 4.6	0.0	0.0 - 0.0	8.8	2.8 - 20.9	0.71
3150107	8779	COOSA R	19.7	15.6	5 - 32	0.0	0.0 - 0.0	7.0	2.4 - 14.0	0.6	0.2 - 1.6	0.6	0.2 - 1.6	0.0	0.0 - 0.0	7.5	2.8 - 18.5	0.76
3150107	8780	SWAMP CR	177.3	17.5	6 - 41	0.0	0.0 - 0.0	5.6	1.6 - 12.2	0.5	0.2 - 1.4	1.5	0.4 - 4.4	0.0	0.0 - 0.0	9.9	3.2 - 25.8	0.72
3150107	8781	HATCHET CR	56.6	15.2	5 - 31	0.0	0.0 - 0.0	5.9	1.8 - 12.0	0.4	0.1 - 1.0	0.4	0.1 - 1.3	0.0	0.0 - 0.0	8.5	2.5 - 19.1	0.72
3150107	8783	JACKS CR	71.3	16.3	5 - 33	0.0	0.0 - 0.0	4.4	1.3 - 8.5	0.5	0.1 - 1.2	0.8	0.3 - 2.0	0.0	0.0 - 0.0	10.6	3.2 - 22.5	0.69
3150107	8784	SOCAPATOY CR	124.5	24.3	9 - 63	2.5	0.8 - 5.9	8.6	2.8 - 19.9	0.6	0.2 - 1.6	2.3	0.8 - 7.1	0.0	0.0 - 0.0	10.3	3.3 - 25.3	0.69
3150107	8785	HATCHET CR	411.8	16.6	6 - 34	0.0	0.0 - 0.0	4.6	1.5 - 9.8	1.8	0.6 - 5.0	2.2	0.8 - 5.3	0.0	0.0 - 0.0	7.9	2.5 - 18.6	0.69
3150107	8786	COOSA R	1.6	17.4	7 - 32	0.0	0.0 - 0.0	8.4	3.0 - 15.5	0.3	0.1 - 0.8	0.4	0.2 - 1.0	0.0	0.0 - 0.0	8.4	3.3 - 18.2	0.72
3150107	8787	COOSA R	9.4	28.6	10 - 62	0.0	0.0 - 0.0	7.5	2.3 - 15.3	0.6	0.2 - 1.8	4.4	1.4 - 11.0	0.0	0.0 - 0.0	16.1	6.0 - 37.8	0.68
3150107	8788	PECKERWOOD CR	76.7	20.4	7 - 41	0.0	0.0 - 0.0	3.7	1.1 - 8.3	0.9	0.2 - 2.3	4.1	1.1 - 12.4	0.0	0.0 - 0.0	11.7	3.6 - 27.9	0.68
3150107	8789	COOSA R	16.0	36.8	12 - 69	0.0	0.0 - 0.0	9.8	3.0 - 20.1	2.2	0.7 - 5.4	7.7	2.2 - 18.8	0.0	0.0 - 0.0	17.1	5.4 - 39.6	0.68
3150107	8790	CEDAR CR	127.5	46.4	14 - 99	0.0	0.0 - 0.0	6.0	1.7 - 13.6	4.6	1.4 - 12.2	18.2	5.1 - 42.0	0.0	0.0 - 0.0	17.7	5.1 - 51.8	0.68
3150107	8791	COOSA R	153.3	41.0	21 - 75	0.0	0.0 - 0.1	7.3	3.3 - 16.2	2.1	0.8 - 5.4	17.6	8.0 - 39.4	0.0	0.0 - 0.0	14.0	6.4 - 27.6	0.68
3150107	8792	COOSA R	94.8	40.3	12 - 66	0.0	0.0 - 0.0	6.0	1.8 - 11.0	2.9	0.8 - 9.2	16.2	4.8 - 35.7	0.0	0.0 - 0.0	15.2	5.1 - 31.5	0.68
3150107	8793	TALLASSEEHATCI	83.9	70.5	21 - 129	0.0	0.0 - 0.0	19.5	6.9 - 36.0	6.1	2.0 - 12.6	25.2	7.7 - 60.8	0.0	0.0 - 0.0	19.8	6.2 - 43.7	0.68
3150107	8794	TALLASSEEHATCI	31.5	69.5	21 - 134	0.0	0.0 - 0.0	11.3	3.3 - 23.9	7.5	2.1 - 22.4	31.7	10.0 - 84.1	0.0	0.0 - 0.0	19.0	6.0 - 47.2	0.67
3150107	8795	*A	53.9	128.3	38 - 298	49.3	13.7 - 117.5	48.4	16.6 - 105.1	2.5	0.8 - 6.5	9.3	2.6 - 31.6	0.0	0.0 - 0.0	18.8	5.6 - 44.8	0.66
3150107	8796	TALLASSEEHATCI	11.2	92.7	32 - 193	0.0	0.0 - 0.0	13.6	4.9 - 27.8	10.9	3.9 - 26.8	46.6	14.3 - 109.2	0.0	0.0 - 0.0	21.7	7.7 - 47.1	0.66
3150107	8797	TALLASSEEHATCI	9.7	169.5	52 - 334	0.0	0.0 - 0.0	21.5	7.2 - 39.4	4.8	1.5 - 11.1	17.3	5.0 - 38.4	108.1	28.5 - 309.5	17.7	5.7 - 40.7	0.66
3150107	8798	CROOKED CR	43.5	42.9	13 - 121	0.0	0.0 - 0.0	17.4	5.4 - 48.9	1.8	0.6 - 6.0	5.4	1.9 - 18.0	6.9	1.9 - 26.1	11.3	3.6 - 33.2	0.64
3150107	8799	TALLASSEEHATCI	89.2	13.1	5 - 25	0.0	0.0 - 0.0	4.2	1.6 - 8.0	0.2	0.1 - 0.6	0.1	0.0 - 0.3	0.0	0.0 - 0.0	8.6	3.6 - 18.1	0.64
3150107	8800	WEEWOKA CR	116.1	47.8	17 - 95	0.0	0.0 - 0.0	8.1	2.8 - 17.7	4.9	1.3 - 17.0	19.8	7.0 - 49.6	0.0	0.0 - 0.0	15.0	5.7 - 31.3	0.67
3150107	8802	YELLOWLEAF CR	58.0	44.9	19 - 104	0.0	0.0 - 0.0	6.7	2.4 - 14.7	2.0	0.7 - 5.9	21.5	7.9 - 56.3	0.0	0.0 - 0.0	14.7	5.2 - 35.2	0.68
3150107	8803	*B	40.3	54.7	17 - 103	0.0	0.0 - 0.0	8.5	3.0 - 15.8	2.7	0.8 - 6.0	30.9	9.4 - 70.7	0.0	0.0 - 0.0	12.6	4.3 - 28.1	0.67
3150107	8804	YELLOWLEAF CR	46.0	23.8	9 - 55	0.0	0.0 - 0.0	4.2	1.4 - 9.5	0.9	0.3 - 2.2	3.9	1.3 - 9.2	0.0	0.0 - 0.0	14.8	5.2 - 36.6	0.67
3150107	8805	MUDDY PRONG	74.7	19.7	7 - 34	0.0	0.0 - 0.0	6.7	2.2 - 14.1	0.6	0.2 - 1.7	3.8	1.3 - 10.1	0.0	0.0 - 0.0	8.6	2.8 - 17.3	0.66
3150107	8806	YELLOWLEAF CR	267.8	23.7	9 - 47	0.1	0.0 - 0.1	11.1	3.9 - 23.7	0.8	0.2 - 2.0	4.1	1.3 - 9.8	0.0	0.0 - 0.0	7.7	2.6 - 15.7	0.66
3150107	8807	BEESWAX CR	58.1	28.5	11 - 68	0.0	0.0 - 0.0	5.5	1.9 - 12.7	1.2	0.4 - 3.4	8.7	3.2 - 23.7	0.0	0.0 - 0.0	13.1	4.8 - 32.9	0.68
3150107	8808	WAXAHATCHEE C	2.8	28.1	11 - 62	0.0	0.0 - 0.0	0.5	0.2 - 1.2	1.4	0.4 - 4.0	5.6	2.1 - 13.7	0.0	0.0 - 0.0	20.7	7.6 - 45.6	0.67
3150107	8809	WAXAHATCHEE C	38.8	30.5	12 - 55	0.0	0.0 - 0.0	8.3	2.8 - 17.0	0.7	0.2 - 1.7	3.5	1.2 - 8.0	0.0	0.0 - 0.0	18.0	6.8 - 36.3	0.66
3150107	8810	WAXAHATCHEE C	121.0	33.4	12 - 71	8.2	3.1 - 18.0	8.6	2.8 - 18.0	0.8	0.2 - 2.1	4.5	1.6 - 10.2	0.0	0.0 - 0.0	11.4	4.1 - 24.2	0.64
3150107	8811	WAXAHATCHEE C	93.1	35.9	12 - 66	0.0	0.0 - 0.0	13.1	4.4 - 27.3	1.0	0.3 - 2.6	7.8	2.4 - 17.5	0.0	0.0 - 0.0	14.0	4.7 - 31.6	0.64
3150107	8812	STUMPS CR	175.2	32.2	13 - 81	0.0	0.0 - 0.0	9.7	3.6 - 23.9	1.3	0.4 - 3.8	6.6	2.4 - 18.4	0.0	0.0 - 0.0	14.6	5.3 - 41.1	0.66
3150107	8813	WASH CR	39.8	23.0	7 - 46	0.0	0.0 - 0.0	3.2	0.9 - 6.7	1.5	0.4 - 4.0	3.1	0.8 - 7.4	0.0	0.0 - 0.0	15.2	4.8 - 34.3	0.67
3150107	8814	YELLOWLEAF CR	123.0	21.8	7 - 54	0.0	0.0 - 0.0	5.7	1.7 - 13.6	1.6	0.4 - 4.5	5.6	1.6 - 14.3	0.0	0.0 - 0.0	8.9	2.7 - 24.5	0.72
3150107	8815	CHESTNUT CR	194.9	25.9	9 - 66	0.0	0.0 - 0.0	9.8	3.3 - 23.4	1.7	0.6 - 5.2	10.3	3.3 - 30.9	0.0	0.0 - 0.0	4.1	1.2 - 10.9	0.76
3150107	8816	EMAUHEE CR	80.1	21.8	8 - 48	0.5	0.2 - 1.1	8.0	2.7 - 18.4	0.9	0.3 - 2.9	2.3	0.8 - 6.0	0.0	0.0 - 0.0	10.1	3.2 - 22.9	0.66
3150108	8817	LITTLE TALLAPOO	33.6	21.6	9 - 53	0.0	0.0 - 0.0	6.3	2.3 - 16.5	3.4	1.2 - 11.0	4.0	1.5 - 11.7	0.0	0.0 - 0.0	7.9	3.2 - 20.9	0.32
3150108	8818	WEDOWEE CR	134.0	42.4	12 - 92	0.0	0.0 - 0.0	7.9	2.2 - 17.7	10.9	2.7 - 28.7	14.6	4.4 - 41.1	0.0	0.0 - 0.0	9.1	2.8 - 21.3	0.31

8-digit HUC	ID	Name	Area	Catchment Yield		Point sources		Developed Land		Manure		Agricultural Land		Phosphate Mines		Soil parent rock		Frac
				kg/km^2	90% CI	kg/km^2	90% CI	kg/km^2	90% CI	kg/km^2	90% CI	kg/km^2	90% CI	kg/km^2	90% CI	kg/km^2	90% CI	
3150108	8819	LITTLE TALLAPOO	2.9	16.8	6 - 37	0.0	0.0 - 0.0	4.8	1.6 - 11.6	2.4	0.7 - 6.2	1.8	0.5 - 4.8	0.0	0.0 - 0.0	7.9	3.0 - 20.2	0.31
3150108	8820	LITTLE TALLAPOO	43.2	37.9	15 - 76	0.0	0.0 - 0.0	8.6	3.2 - 18.6	8.9	3.2 - 28.6	11.9	4.6 - 29.6	0.0	0.0 - 0.0	8.5	3.4 - 20.1	0.31
3150108	8821	BEAR CR	49.8	40.8	16 - 84	0.0	0.0 - 0.0	7.0	2.6 - 17.5	10.8	3.4 - 32.2	14.8	4.9 - 37.5	0.0	0.0 - 0.0	8.2	3.4 - 20.8	0.31
3150108	8822	LITTLE TALLAPOO	128.7	37.7	13 - 80	0.0	0.0 - 0.0	6.2	2.1 - 13.0	9.8	2.9 - 27.5	12.3	3.8 - 33.4	0.0	0.0 - 0.0	9.5	3.4 - 25.1	0.31
3150108	8823	CANE CR	37.8	47.0	17 - 87	0.0	0.0 - 0.0	7.5	2.7 - 14.9	12.4	3.7 - 30.6	16.5	5.4 - 36.4	0.0	0.0 - 0.0	10.7	3.2 - 24.1	0.30
3150108	8824	LITTLE TALLAPOO	1.5	142.9	50 - 297	0.0	0.0 - 0.0	3.5	1.3 - 7.8	49.1	15.7 - 132.0	72.2	23.8 - 192.0	0.0	0.0 - 0.0	18.1	5.8 - 46.7	0.30
3150108	8825	*A	58.8	51.5	18 - 94	0.0	0.0 - 0.0	6.8	2.3 - 12.7	15.0	4.3 - 40.8	20.1	6.6 - 49.2	0.0	0.0 - 0.0	9.7	3.9 - 20.7	0.29
3150108	8826	LITTLE TALLAPOO	157.6	66.3	25 - 136	0.0	0.0 - 0.0	8.0	3.3 - 16.2	24.1	7.4 - 62.9	23.9	8.4 - 52.0	0.0	0.0 - 0.0	10.3	4.0 - 23.4	0.29
3150108	8827	LITTLE TALLAPOO	159.0	59.8	21 - 121	0.0	0.0 - 0.0	8.2	3.1 - 19.7	20.6	6.1 - 55.3	21.3	7.3 - 53.5	0.0	0.0 - 0.0	9.6	3.4 - 21.8	0.28
3150108	8828	BUFFALO CR	71.1	63.1	26 - 152	0.0	0.0 - 0.0	27.1	10.9 - 67.2	13.4	4.8 - 35.1	13.6	4.7 - 37.7	0.0	0.0 - 0.0	9.1	3.5 - 22.4	0.27
3150108	8829	LITTLE TALLAPOO	49.1	53.8	21 - 116	0.0	0.0 - 0.0	10.0	3.9 - 19.2	16.5	5.5 - 46.2	16.7	7.0 - 44.0	0.0	0.0 - 0.0	10.7	4.2 - 24.3	0.27
3150108	8830	LITTLE TALLAPOO	269.0	58.2	17 - 121	1.9	0.6 - 4.1	21.8	6.4 - 50.1	11.7	2.9 - 38.5	11.9	4.2 - 32.4	0.0	0.0 - 0.0	10.8	3.3 - 28.0	0.26
3150108	8831	*B	91.2	51.2	18 - 135	4.8	1.6 - 13.0	14.8	4.9 - 38.1	10.3	3.1 - 40.2	11.1	3.5 - 34.9	0.0	0.0 - 0.0	10.3	3.9 - 28.0	0.26
3150108	8832	BIG INDIAN CR	122.4	49.7	17 - 101	2.5	0.9 - 6.2	8.9	3.1 - 19.1	14.5	4.5 - 44.7	14.8	5.2 - 39.3	0.0	0.0 - 0.0	8.9	3.0 - 21.7	0.28
3150108	8833	TURKEY CR	56.8	46.3	17 - 107	0.0	0.0 - 0.0	15.0	5.6 - 35.4	10.4	2.9 - 31.5	10.5	3.8 - 30.2	0.0	0.0 - 0.0	10.5	3.9 - 25.6	0.24
3150108	8834	BIG INDIAN CR	10.3	28.1	9 - 62	0.0	0.0 - 0.0	6.1	2.1 - 14.7	7.0	2.1 - 18.5	6.1	2.2 - 16.0	0.0	0.0 - 0.0	9.0	2.8 - 22.2	0.24
3150108	8835	PINEY CR	71.3	30.8	11 - 78	0.0	0.0 - 0.0	7.2	2.4 - 16.7	7.0	2.2 - 22.6	8.8	3.1 - 24.1	0.0	0.0 - 0.0	7.9	2.7 - 20.6	0.31
3150108	8836	TALLAPOOSA R	273.1	24.9	10 - 58	0.0	0.0 - 0.0	5.7	2.1 - 12.0	4.9	1.7 - 14.3	5.7	2.1 - 17.0	0.0	0.0 - 0.0	8.7	3.1 - 21.5	0.32
3150108	8837	TALLAPOOSA R	47.5	26.1	8 - 55	0.0	0.0 - 0.0	6.6	1.9 - 15.1	5.8	1.7 - 18.9	4.6	1.2 - 13.2	0.0	0.0 - 0.0	9.1	2.8 - 24.5	0.31
3150108	8838	TALLAPOOSA R	7.3	40.3	13 - 89	0.0	0.0 - 0.0	6.7	2.1 - 15.8	11.6	3.8 - 28.9	9.4	3.2 - 21.1	0.0	0.0 - 0.0	12.6	4.6 - 30.4	0.31
3150108	8839	DYNE CR	65.0	27.1	8 - 46	0.0	0.0 - 0.0	3.6	1.1 - 7.4	8.0	2.4 - 21.6	6.0	1.8 - 12.9	0.0	0.0 - 0.0	9.5	3.1 - 20.2	0.31
3150108	8840	TALLAPOOSA R	119.8	35.1	14 - 62	0.0	0.0 - 0.0	9.7	3.4 - 20.0	7.9	2.6 - 19.7	6.7	2.8 - 17.3	0.0	0.0 - 0.0	10.8	4.8 - 21.9	0.31
3150108	8841	TALLAPOOSA R	62.3	34.8	12 - 77	0.0	0.0 - 0.0	6.0	2.1 - 12.3	10.2	3.0 - 28.8	8.7	3.1 - 20.8	0.0	0.0 - 0.0	9.8	3.5 - 24.2	0.30
3150108	8842	SILAS CR	45.6	46.7	17 - 114	0.0	0.0 - 0.0	7.7	2.7 - 17.9	15.8	4.6 - 47.8	13.8	3.8 - 33.7	0.0	0.0 - 0.0	9.4	3.8 - 22.6	0.30
3150108	8843	TALLAPOOSA R	51.2	41.8	13 - 75	0.0	0.0 - 0.0	7.5	2.5 - 14.9	12.9	3.3 - 28.8	10.8	3.2 - 27.5	0.0	0.0 - 0.0	10.5	3.4 - 21.0	0.30
3150108	8844	OWEN CR	21.0	27.1	9 - 50	0.0	0.0 - 0.0	8.5	2.5 - 18.4	5.2	1.5 - 13.4	4.1	1.4 - 9.7	0.0	0.0 - 0.0	9.4	3.3 - 18.9	0.29
3150108	8845	TALLAPOOSA R	42.4	33.0	12 - 73	0.0	0.0 - 0.0	5.5	1.8 - 11.5	10.0	3.2 - 28.3	7.3	2.5 - 20.2	0.0	0.0 - 0.0	10.2	3.5 - 25.0	0.29
3150108	8846	KELLEY CR	38.8	32.5	9 - 52	0.0	0.0 - 0.0	7.2	2.1 - 11.7	7.4	2.0 - 16.5	8.1	2.1 - 17.8	0.0	0.0 - 0.0	9.8	3.0 - 18.0	0.29
3150108	8847	TALLAPOOSA R	99.1	28.0	9 - 49	0.0	0.0 - 0.0	6.6	2.2 - 11.7	6.6	1.8 - 16.2	4.9	1.3 - 11.9	0.0	0.0 - 0.0	9.9	3.1 - 21.0	0.29
3150108	8848	BEACH CR	95.4	32.4	12 - 70	0.0	0.0 - 0.0	11.5	4.1 - 27.1	3.6	1.0 - 10.4	7.6	2.5 - 19.8	0.0	0.0 - 0.0	9.8	3.5 - 24.8	0.28
3150108	8849	TALLAPOOSA R	143.8	28.8	11 - 54	1.0	0.4 - 2.2	6.6	2.3 - 13.5	4.3	1.5 - 11.7	8.1	3.5 - 20.0	0.0	0.0 - 0.0	8.8	3.5 - 20.3	0.28
3150108	8850	LITTLE R	95.0	41.4	12 - 112	3.5	1.0 - 9.2	8.6	2.4 - 25.1	5.6	1.4 - 16.9	12.3	3.5 - 38.1	0.0	0.0 - 0.0	11.4	3.0 - 34.3	0.26
3150108	8851	TALLAPOOSA R	129.4	28.8	10 - 61	0.0	0.0 - 0.0	5.9	1.9 - 12.0	4.2	1.2 - 10.6	9.3	2.8 - 25.3	0.0	0.0 - 0.0	9.4	3.2 - 21.6	0.26
3150108	8852	BROOKS CR	44.6	35.9	12 - 91	0.0	0.0 - 0.0	9.3	3.2 - 23.4	6.2	2.0 - 19.7	9.7	3.4 - 29.0	0.0	0.0 - 0.0	10.7	3.5 - 30.0	0.25
3150108	8853	TALLAPOOSA R	80.7	32.2	11 - 62	0.0	0.0 - 0.0	7.8	2.7 - 15.9	5.2	1.6 - 13.0	10.7	3.3 - 24.3	0.0	0.0 - 0.0	8.6	3.0 - 21.1	0.25
3150108	8854	CANE CR	162.3	22.3	7 - 45	0.0	0.0 - 0.0	6.3	2.1 - 13.4	4.3	1.2 - 12.7	3.0	0.9 - 7.7	0.0	0.0 - 0.0	8.6	3.0 - 21.4	0.30
3150108	8855	CHULAFINNEE CR	71.1	32.9	12 - 71	0.0	0.0 - 0.0	6.8	2.4 - 15.4	8.7	2.5 - 25.9	7.4	2.7 - 19.9	0.0	0.0 - 0.0	10.0	3.9 - 24.0	0.31
3150108	8856	KETCHEPEDRAKE	135.0	21.7	9 - 45	0.0	0.0 - 0.0	3.8	1.4 - 7.8	3.6	1.3 - 8.8	3.6	1.4 - 7.9	0.0	0.0 - 0.0	10.7	4.0 - 24.1	0.31
3150109	8857	SANDY CR	11.2	26.2	8 - 52	0.0	0.0 - 0.0	7.9	2.2 - 16.9	0.6	0.2 - 1.5	3.6	1.1 - 8.3	0.0	0.0 - 0.0	14.1	4.3 - 32.5	0.34

8-digit HUC	ID	Name	Area	Catchment Yield		Point sources		Developed Land		Manure		Agricultural Land		Phosphate Mines		Soil parent rock		Frac
				kg/km^2	90% CI	kg/km^2	90% CI	kg/km^2	90% CI	kg/km^2	90% CI	kg/km^2	90% CI	kg/km^2	90% CI	kg/km^2	90% CI	
3150109	8858	SANDY CR, S FK	86.0	29.7	9 - 68	0.0	0.0 - 0.0	7.0	2.0 - 16.6	1.5	0.4 - 4.6	9.5	2.7 - 24.7	0.0	0.0 - 0.0	11.7	3.6 - 29.5	0.33
3150109	8859	SANDY CR, S FK	84.4	22.2	6 - 54	0.0	0.0 - 0.0	5.1	1.5 - 12.5	1.4	0.4 - 4.6	5.2	1.5 - 14.6	0.0	0.0 - 0.0	10.6	3.1 - 24.6	0.30
3150109	8860	LITTLE SANDY CR	88.0	22.3	7 - 49	0.0	0.0 - 0.0	4.1	1.2 - 8.6	1.5	0.4 - 4.1	6.2	1.9 - 18.5	0.0	0.0 - 0.0	10.5	3.8 - 23.8	0.30
3150109	8861	SANDY CR, N FK	27.1	45.5	14 - 108	9.2	3.1 - 21.2	14.4	3.9 - 30.6	1.2	0.4 - 3.4	5.7	1.8 - 16.3	0.0	0.0 - 0.0	15.1	5.3 - 36.4	0.33
3150109	8862	*A	40.2	20.3	8 - 45	0.0	0.0 - 0.0	4.7	1.6 - 9.6	0.8	0.3 - 2.2	3.1	1.1 - 7.7	0.0	0.0 - 0.0	11.7	4.3 - 29.2	0.30
3150109	8863	SANDY CR, N FK	73.9	21.2	7 - 44	0.0	0.0 - 0.0	4.3	1.4 - 8.7	0.6	0.2 - 1.4	2.4	0.8 - 5.2	0.0	0.0 - 0.0	13.9	4.5 - 30.3	0.30
3150109	8864	BUCK CR	52.2	30.0	9 - 75	0.0	0.0 - 0.0	8.9	2.9 - 21.1	1.1	0.3 - 3.7	6.8	1.9 - 19.7	0.0	0.0 - 0.0	13.2	4.2 - 31.5	0.34
3150109	8865	TALLAPOOSA R	60.7	17.0	6 - 35	0.0	0.0 - 0.0	4.7	1.7 - 9.7	0.6	0.2 - 1.8	3.1	1.1 - 7.7	0.0	0.0 - 0.0	8.6	3.3 - 20.0	0.34
3150109	8866	TALLAPOOSA R	26.8	15.5	5 - 27	0.0	0.0 - 0.0	5.0	1.6 - 10.0	0.6	0.2 - 1.5	1.7	0.6 - 3.9	0.0	0.0 - 0.0	8.1	2.6 - 17.0	0.34
3150109	8867	EAGLE CR	16.6	21.0	7 - 54	0.0	0.0 - 0.0	5.3	1.6 - 14.0	0.8	0.2 - 2.9	5.9	2.0 - 18.5	0.0	0.0 - 0.0	9.0	3.1 - 25.8	0.33
3150109	8868	TALLAPOOSA R	58.6	21.4	6 - 41	0.0	0.0 - 0.0	4.1	1.1 - 7.8	0.8	0.2 - 1.7	3.6	1.1 - 8.0	0.0	0.0 - 0.0	12.9	3.9 - 29.9	0.33
3150109	8869	COUNTY LINE CR	41.2	22.8	9 - 48	0.0	0.0 - 0.0	4.0	1.4 - 8.2	0.8	0.3 - 2.6	4.7	1.6 - 14.3	0.0	0.0 - 0.0	13.2	4.7 - 30.7	0.33
3150109	8871	CHATAHOSPEE CR	41.2	20.0	7 - 40	0.0	0.0 - 0.0	4.1	1.2 - 9.3	0.8	0.3 - 2.9	2.9	1.0 - 7.0	0.0	0.0 - 0.0	12.1	4.3 - 28.2	0.33
3150109	8872	CHATAHOSPEE CR	10.7	25.6	10 - 62	0.0	0.0 - 0.0	4.0	1.4 - 9.5	2.0	0.6 - 6.8	8.0	3.0 - 23.0	0.0	0.0 - 0.0	11.6	4.5 - 26.3	0.32
3150109	8873	LITTLE CHATAHO	91.4	28.9	9 - 69	0.0	0.0 - 0.0	4.7	1.3 - 11.6	1.9	0.5 - 5.4	11.1	3.4 - 26.6	0.0	0.0 - 0.0	11.2	3.7 - 26.5	0.32
3150109	8874	CHATAHOSPEE CR	30.0	24.4	8 - 61	0.0	0.0 - 0.0	3.7	1.1 - 7.6	1.8	0.5 - 5.5	7.6	2.6 - 20.5	0.0	0.0 - 0.0	11.4	4.1 - 29.2	0.32
3150109	8875	FINLEY CR	48.4	43.9	14 - 87	9.3	3.1 - 20.5	7.9	2.5 - 15.7	2.1	0.6 - 5.6	13.3	4.2 - 29.8	0.0	0.0 - 0.0	11.3	3.5 - 25.3	0.30
3150109	8876	CHATAHOSPEE CR	19.4	32.2	11 - 77	0.0	0.0 - 0.0	8.9	2.7 - 21.3	1.8	0.5 - 5.1	10.0	3.3 - 24.6	0.0	0.0 - 0.0	11.5	4.0 - 29.7	0.30
3150109	8877	ALLEN CR	67.5	34.1	10 - 85	0.0	0.0 - 0.0	6.2	1.7 - 15.4	2.3	0.7 - 6.8	14.8	4.6 - 37.6	0.0	0.0 - 0.0	10.9	3.4 - 27.8	0.32
3150109	8878	TALLAPOOSA R	76.1	16.3	5 - 29	0.0	0.0 - 0.0	4.2	1.4 - 7.5	0.8	0.3 - 2.0	3.1	1.0 - 6.3	0.0	0.0 - 0.0	8.2	2.7 - 15.2	0.33
3150109	8879	HIGH PINE CR	203.1	38.1	13 - 80	2.1	0.7 - 5.3	8.6	3.0 - 20.3	6.9	2.1 - 20.7	10.3	3.2 - 23.5	0.0	0.0 - 0.0	10.2	3.5 - 24.5	0.33
3150109	8880	TALLAPOOSA R	9.9	17.0	6 - 45	0.0	0.0 - 0.0	5.9	2.1 - 15.1	0.8	0.3 - 2.4	1.9	0.6 - 5.4	0.0	0.0 - 0.0	8.4	3.0 - 26.2	0.33
3150109	8881	TALLAPOOSA R	48.3	23.2	8 - 54	0.0	0.0 - 0.0	6.2	2.1 - 15.0	3.7	1.1 - 11.9	3.9	1.2 - 12.8	0.0	0.0 - 0.0	9.4	3.1 - 25.0	0.33
3150109	8882	TALLAPOOSA R	67.0	17.3	5 - 41	0.0	0.0 - 0.0	3.5	1.0 - 7.7	2.7	0.8 - 7.5	2.2	0.6 - 6.6	0.0	0.0 - 0.0	8.9	2.6 - 20.4	0.33
3150109	8883	CORNHOUSE CR	12.6	19.8	6 - 37	0.0	0.0 - 0.0	3.4	1.0 - 7.4	2.7	0.8 - 6.2	2.7	0.9 - 6.7	0.0	0.0 - 0.0	11.1	3.5 - 23.5	0.33
3150109	8884	CORNHOUSE CR	84.0	30.8	9 - 66	0.0	0.0 - 0.0	5.2	1.5 - 10.0	6.8	1.9 - 20.7	7.3	2.3 - 20.0	0.0	0.0 - 0.0	11.5	4.0 - 26.2	0.32
3150109	8885	WILD CAT CR	49.0	29.7	12 - 67	0.0	0.0 - 0.0	5.9	2.2 - 12.5	6.3	2.2 - 19.3	7.3	2.9 - 18.5	0.0	0.0 - 0.0	10.2	3.4 - 26.8	0.32
3150109	8886	TALLAPOOSA R	28.0	11.7	4 - 25	0.0	0.0 - 0.0	1.3	0.5 - 2.8	0.9	0.3 - 2.5	0.9	0.3 - 2.4	0.0	0.0 - 0.0	8.5	3.0 - 18.8	0.33
3150109	8887	TALLAPOOSA R	155.1	30.5	10 - 74	0.0	0.0 - 0.0	6.7	2.2 - 16.9	6.6	1.8 - 17.5	7.9	2.6 - 23.0	0.0	0.0 - 0.0	9.4	3.2 - 23.4	0.32
3150109	8888	CROOKED CR	11.8	13.6	4 - 32	0.0	0.0 - 0.0	2.4	0.7 - 5.1	1.1	0.3 - 3.2	1.1	0.4 - 2.8	0.0	0.0 - 0.0	9.0	2.9 - 21.3	0.32
3150109	8889	CROOKED CR	145.3	38.8	13 - 73	6.2	2.0 - 13.0	8.0	2.4 - 15.7	6.9	2.0 - 19.4	8.1	2.6 - 20.6	0.0	0.0 - 0.0	9.6	3.0 - 22.6	0.32
3150109	8890	WESOBULGA CR	97.4	22.4	8 - 50	0.0	0.0 - 0.0	6.1	2.0 - 13.8	4.3	1.4 - 11.3	4.3	1.4 - 10.5	0.0	0.0 - 0.0	7.8	2.2 - 20.4	0.32
3150109	8891	CEDAR CR	26.9	20.7	7 - 51	0.0	0.0 - 0.0	6.1	1.8 - 14.2	3.0	0.8 - 10.5	3.0	1.0 - 8.6	0.0	0.0 - 0.0	8.6	3.0 - 23.7	0.33
3150109	8892	*B	29.4	25.4	9 - 43	7.6	3.0 - 15.1	5.4	2.0 - 9.5	2.2	0.8 - 5.5	2.1	0.7 - 5.4	0.0	0.0 - 0.0	8.1	2.9 - 17.3	0.33
3150109	8893	EMUCKFAW CR	168.6	15.7	5 - 40	0.0	0.0 - 0.0	4.2	1.2 - 10.5	1.2	0.3 - 3.3	2.1	0.6 - 5.3	0.0	0.0 - 0.0	8.2	2.9 - 21.5	0.34
3150109	8894	*C	46.9	21.5	9 - 43	0.0	0.0 - 0.0	7.0	2.9 - 15.2	0.8	0.3 - 2.3	4.6	1.8 - 11.4	0.0	0.0 - 0.0	9.0	3.7 - 22.5	0.34
3150109	8895	HILLABEE CR	45.3	18.9	7 - 31	0.0	0.0 - 0.0	7.5	2.8 - 13.9	0.5	0.1 - 1.2	1.3	0.5 - 2.9	0.0	0.0 - 0.0	9.6	3.5 - 19.4	0.33
3150109	8896	ENITACHOPCO CR	295.8	25.4	9 - 47	0.0	0.0 - 0.0	5.8	2.0 - 13.1	5.2	1.7 - 14.1	5.5	1.8 - 17.0	0.0	0.0 - 0.0	9.0	3.2 - 20.3	0.32
3150109	8897	HILLABEE CR	188.1	20.6	8 - 42	0.0	0.0 - 0.0	4.8	1.8 - 10.4	3.1	1.2 - 8.4	3.0	1.1 - 7.0	0.0	0.0 - 0.0	9.8	3.6 - 22.7	0.32

8-digit HUC	ID	Name	Area	Catchment Yield		Point sources		Developed Land		Manure		Agricultural Land		Phosphate Mines		Soil parent rock		Frac
				kg/km2	90% CI	kg/km2	90% CI	kg/km2	90% CI	kg/km2	90% CI	kg/km2	90% CI	kg/km2	90% CI	kg/km2	90% CI	
3150109	8898	HACKNEY CR	9.4	16.7	6 - 39	0.0	0.0 - 0.0	4.8	1.6 - 11.1	0.6	0.2 - 2.1	2.6	1.0 - 6.8	0.0	0.0 - 0.0	8.7	3.1 - 21.8	0.33
3150109	8899	HACKNEY CR	17.1	22.6	8 - 55	0.0	0.0 - 0.0	7.2	2.3 - 16.6	0.9	0.2 - 2.3	4.6	1.7 - 13.1	0.0	0.0 - 0.0	9.9	3.3 - 25.1	0.32
3150109	8900	TOWN CR	45.8	20.7	8 - 52	0.0	0.0 - 0.0	5.8	2.3 - 14.4	0.7	0.2 - 2.5	3.2	1.1 - 7.6	0.0	0.0 - 0.0	11.1	4.2 - 30.1	0.32
3150109	8901	CHIKASANOXEE C	196.8	24.5	9 - 58	0.0	0.0 - 0.0	5.4	1.9 - 11.3	1.9	0.6 - 6.1	6.4	2.2 - 15.5	0.0	0.0 - 0.0	10.7	4.0 - 27.0	0.33
3150109	8902	TALLAPOOSA R	31.4	19.7	6 - 56	0.0	0.0 - 0.0	6.4	2.0 - 17.2	1.0	0.3 - 3.8	4.2	1.3 - 14.1	0.0	0.0 - 0.0	8.0	2.6 - 23.0	0.33
3150110	8903	TALLAPOOSA R	229.9	57.4	17 - 135	0.0	0.0 - 0.0	23.0	6.8 - 56.7	4.0	1.1 - 11.0	17.7	5.5 - 45.2	0.0	0.0 - 0.0	12.7	4.2 - 31.8	0.79
3150110	8904	TALLAPOOSA R	2.4	80.9	21 - 197	0.0	0.0 - 0.0	6.2	1.8 - 15.0	11.4	3.1 - 35.8	47.7	11.6 - 152.3	0.0	0.0 - 0.0	15.6	5.1 - 44.2	0.79
3150110	8905	MILLER CR	70.8	88.0	26 - 175	0.0	0.0 - 0.0	11.5	3.2 - 25.4	10.4	2.7 - 35.0	37.2	10.1 - 100.7	0.0	0.0 - 0.0	28.9	8.6 - 64.6	0.79
3150110	8906	TALLAPOOSA R	18.1	28.3	8 - 59	0.0	0.0 - 0.0	4.3	1.3 - 8.5	2.1	0.6 - 6.0	11.5	3.1 - 26.9	0.0	0.0 - 0.0	10.3	3.2 - 25.5	0.79
3150110	8907	LINE CR	49.9	43.6	19 - 100	0.0	0.0 - 0.0	5.1	2.0 - 10.4	4.5	1.7 - 14.5	19.3	8.4 - 51.1	0.0	0.0 - 0.0	14.7	5.6 - 37.9	0.78
3150110	8908	JOHNSONS CR	118.8	128.8	45 - 280	0.0	0.0 - 0.0	2.1	0.7 - 4.9	15.4	4.5 - 49.8	56.5	18.7 - 149.4	0.0	0.0 - 0.0	54.8	18.1 - 132.9	0.76
3150110	8909	LINE CR	11.3	22.3	9 - 41	0.0	0.0 - 0.0	2.6	0.9 - 4.9	1.0	0.3 - 2.8	8.4	2.9 - 21.5	0.0	0.0 - 0.0	10.3	3.7 - 21.5	0.76
3150110	8910	LINE CR	6.0	56.7	18 - 107	0.0	0.0 - 0.0	1.5	0.4 - 2.9	5.2	1.5 - 12.0	22.6	6.7 - 49.1	0.0	0.0 - 0.0	27.5	8.7 - 56.8	0.75
3150110	8911	PANTHER CR	70.3	60.5	21 - 114	0.0	0.0 - 0.0	1.5	0.5 - 2.7	5.7	1.8 - 16.2	26.6	9.2 - 60.9	0.0	0.0 - 0.0	26.8	9.3 - 56.0	0.73
3150110	8912	LINE CR	196.1	35.4	11 - 84	0.0	0.0 - 0.0	3.0	0.9 - 7.1	2.5	0.7 - 7.2	12.2	3.5 - 31.6	0.0	0.0 - 0.0	17.8	5.1 - 44.5	0.73
3150110	8913	OLD TOWN CR	42.6	20.7	7 - 42	0.0	0.0 - 0.0	2.0	0.6 - 4.6	0.7	0.2 - 1.9	5.5	1.7 - 12.1	0.0	0.0 - 0.0	12.5	4.6 - 27.9	0.75
3150110	8914	BUGHALL CR	44.2	59.0	18 - 142	0.0	0.0 - 0.0	2.3	0.7 - 5.4	4.7	1.3 - 14.8	24.7	7.2 - 68.4	0.0	0.0 - 0.0	27.3	8.5 - 73.4	0.71
3150110	8915	BUGHALL CR	118.1	26.0	8 - 51	0.0	0.0 - 0.0	3.4	0.9 - 8.3	1.4	0.4 - 4.2	6.8	1.9 - 17.7	0.0	0.0 - 0.0	14.3	4.3 - 34.3	0.66
3150110	8916	SLAUGHTER CR	102.6	39.1	14 - 80	0.0	0.0 - 0.0	4.4	1.7 - 10.4	2.8	0.8 - 8.5	13.6	4.3 - 35.2	0.0	0.0 - 0.0	18.2	7.1 - 42.1	0.66
3150110	8917	OLD TOWN CR	73.4	66.7	24 - 151	14.2	4.9 - 31.4	3.1	1.0 - 7.8	3.7	1.0 - 9.9	24.4	8.4 - 56.6	0.0	0.0 - 0.0	21.3	7.2 - 54.5	0.71
3150110	8918	TALLAPOOSA R	36.0	17.2	6 - 41	0.0	0.0 - 0.0	2.6	0.9 - 6.3	1.4	0.4 - 4.5	8.8	2.9 - 24.0	0.0	0.0 - 0.0	4.5	1.7 - 11.1	0.78
3150110	8919	CUBAHATCHEE	372.2	24.5	9 - 46	0.0	0.0 - 0.0	3.3	1.1 - 6.8	1.2	0.3 - 2.7	7.8	2.7 - 19.1	0.0	0.0 - 0.0	12.2	4.3 - 25.9	0.78
3150110	8920	TALLAPOOSA R	24.8	47.1	16 - 89	0.0	0.0 - 0.0	2.8	0.8 - 5.2	4.2	1.1 - 10.9	28.8	9.8 - 64.4	0.0	0.0 - 0.0	11.3	3.6 - 22.0	0.78
3150110	8921	CALEBEE CR	396.9	24.5	7 - 58	2.8	0.8 - 6.8	5.1	1.6 - 12.2	0.9	0.3 - 2.7	6.7	2.0 - 17.4	0.0	0.0 - 0.0	8.9	2.8 - 21.8	0.78
3150110	8922	TALLAPOOSA R	1.3	94.4	36 - 215	0.0	0.0 - 0.0	0.0	0.0 - 0.0	9.9	3.4 - 29.8	69.3	26.2 - 162.2	0.0	0.0 - 0.0	15.2	5.5 - 37.1	0.78
3150110	8923	TALLAPOOSA R	6.9	83.6	29 - 204	0.0	0.0 - 0.0	2.5	0.8 - 7.5	7.9	2.3 - 20.6	58.3	19.2 - 162.1	0.0	0.0 - 0.0	14.9	4.8 - 39.9	0.78
3150110	8924	TALLAPOOSA R	24.0	34.9	14 - 73	0.0	0.0 - 0.0	9.6	3.5 - 19.7	2.3	0.8 - 5.6	15.9	5.6 - 38.3	0.0	0.0 - 0.0	7.0	2.4 - 18.1	0.78
3150110	8925	UPAHEE CR	245.5	23.6	8 - 58	0.0	0.0 - 0.0	9.3	2.9 - 22.5	1.1	0.3 - 3.9	8.9	2.9 - 23.7	0.0	0.0 - 0.0	4.3	1.4 - 12.3	0.78
3150110	8926	UPAHEE CR	36.2	31.2	10 - 101	0.0	0.0 - 0.0	24.6	7.3 - 81.8	0.3	0.1 - 1.1	2.5	0.7 - 8.5	0.0	0.0 - 0.0	3.9	1.3 - 11.4	0.75
3150110	8927	OPINTLOCCO CR	265.3	15.4	5 - 37	0.0	0.0 - 0.0	2.1	0.7 - 4.7	0.6	0.2 - 1.7	5.5	1.4 - 16.0	0.0	0.0 - 0.0	7.3	2.3 - 18.6	0.74
3150110	8928	CHEWACLA CR	386.4	36.4	13 - 102	10.7	4.0 - 36.4	13.8	4.7 - 35.9	0.6	0.2 - 2.1	6.1	2.1 - 17.7	0.0	0.0 - 0.0	5.2	1.9 - 15.8	0.74
3150110	8929	*A	162.0	20.9	7 - 58	0.0	0.0 - 0.0	8.6	2.6 - 25.2	0.8	0.2 - 2.4	7.9	2.5 - 23.2	0.0	0.0 - 0.0	3.6	1.0 - 8.7	0.75
3150110	8930	TALLAPOOSA R	43.2	73.1	26 - 177	32.9	11.2 - 87.6	12.6	3.8 - 28.0	2.4	0.7 - 7.3	19.6	5.7 - 54.8	0.0	0.0 - 0.0	5.7	1.8 - 13.8	0.78
3150110	8931	STONE CR	41.3	30.4	10 - 56	0.0	0.0 - 0.0	10.2	3.3 - 23.3	1.6	0.5 - 3.4	14.2	3.9 - 36.1	0.0	0.0 - 0.0	4.5	1.3 - 9.7	0.77
3150110	8932	TALLAPOOSA R	59.6	29.2	11 - 57	0.0	0.0 - 0.0	8.8	3.3 - 19.4	1.5	0.5 - 4.4	10.7	3.6 - 29.6	0.0	0.0 - 0.0	8.2	2.8 - 17.6	0.77
3150110	8933	SOUGAHATCHEE (	253.2	18.4	7 - 38	0.0	0.0 - 0.0	3.1	1.1 - 6.0	0.7	0.2 - 2.2	4.1	1.4 - 9.7	0.0	0.0 - 0.0	10.4	3.6 - 23.5	0.76
3150110	8934	SOUGAHATCHEE (	186.4	42.8	15 - 91	14.3	4.8 - 32.6	16.6	5.5 - 38.1	0.4	0.1 - 1.0	3.2	1.0 - 8.1	0.0	0.0 - 0.0	8.3	2.9 - 19.7	0.70
3150110	8935	*B	123.1	26.4	8 - 57	0.0	0.0 - 0.0	4.5	1.5 - 8.7	1.1	0.3 - 2.7	8.6	2.6 - 21.9	0.0	0.0 - 0.0	12.3	4.1 - 27.3	0.70
3150110	8936	TALLAPOOSA R	9.9	13.7	5 - 25	0.0	0.0 - 0.0	0.0	0.0 - 0.0	0.3	0.1 - 0.7	1.3	0.4 - 3.0	0.0	0.0 - 0.0	12.1	4.0 - 23.3	0.76

8-digit HUC	ID	Name	Area	Catchment Yield		Point sources		Developed Land		Manure		Agricultural Land		Phosphate Mines		Soil parent rock		Frac
				kg/km^2	90% CI	kg/km^2	90% CI	kg/km^2	90% CI	kg/km^2	90% CI	kg/km^2	90% CI	kg/km^2	90% CI	kg/km^2	90% CI	
3150110	8937	TALLAPOOSA R	12.2	11.3	3 - 28	0.0	0.0 - 0.0	1.8	0.5 - 4.3	0.1	0.0 - 0.3	0.1	0.0 - 0.4	0.0	0.0 - 0.0	9.3	2.8 - 22.4	0.76
3150110	8938	*C	76.0	20.5	6 - 36	0.0	0.0 - 0.0	5.6	1.6 - 11.6	0.7	0.2 - 1.6	4.1	1.2 - 9.2	0.0	0.0 - 0.0	10.1	3.4 - 19.5	0.76
3150110	8939	TALLAPOOSA R	2.1	22.9	7 - 46	0.0	0.0 - 0.0	14.2	3.8 - 30.2	0.5	0.1 - 1.0	0.7	0.2 - 1.8	0.0	0.0 - 0.0	7.6	2.2 - 17.0	0.76
3150110	8940	CHANNAHATCHEI	100.0	15.0	5 - 26	0.0	0.0 - 0.0	4.4	1.6 - 9.0	0.9	0.2 - 2.2	3.2	1.0 - 7.7	0.0	0.0 - 0.0	6.5	2.5 - 14.0	0.76
3150110	8941	*D	95.4	33.9	12 - 68	0.0	0.0 - 0.0	6.0	2.1 - 11.0	2.8	0.9 - 7.9	18.1	6.0 - 44.0	0.0	0.0 - 0.0	7.0	2.5 - 14.5	0.78
3150110	8942	TUMKEEHATCHEI	99.0	20.1	6 - 42	0.0	0.0 - 0.0	4.3	1.4 - 9.4	1.5	0.4 - 3.6	9.6	3.0 - 27.8	0.0	0.0 - 0.0	4.6	1.4 - 10.5	0.78
3150110	8943	CHUBBEHATCHEE	189.3	14.5	5 - 28	0.0	0.0 - 0.0	4.8	1.6 - 9.3	1.0	0.3 - 2.2	5.5	1.8 - 12.6	0.0	0.0 - 0.0	3.3	1.0 - 7.1	0.79
3150201	8944	ALABAMA R	146.1	99.5	29 - 210	6.7	2.1 - 14.5	17.4	5.0 - 34.4	5.6	1.5 - 14.9	35.0	10.6 - 83.4	0.0	0.0 - 0.0	34.8	10.5 - 81.3	0.87
3150201	8945	ALABAMA R	112.5	85.2	33 - 194	0.0	0.0 - 0.0	12.9	5.1 - 30.0	6.1	1.8 - 17.5	38.3	15.5 - 109.8	0.0	0.0 - 0.0	27.8	9.8 - 76.3	0.87
3150201	8946	OLD TOWN CR	80.4	55.1	20 - 107	0.0	0.0 - 0.0	6.7	2.2 - 13.8	3.1	1.1 - 7.9	19.1	6.1 - 51.3	0.0	0.0 - 0.0	26.2	9.3 - 65.6	0.86
3150201	8947	ALABAMA R	27.5	1004.0	309 - 2278	933.2	292.1 - 2142.(	12.3	3.4 - 27.5	4.8	1.2 - 15.9	30.2	8.7 - 68.1	0.0	0.0 - 0.0	23.5	7.3 - 66.4	0.86
3150201	8948	ALABAMA R	1.2	46.4	13 - 130	0.0	0.0 - 0.0	0.0	0.0 - 0.0	3.3	0.8 - 13.4	19.4	5.7 - 57.3	0.0	0.0 - 0.0	23.7	6.3 - 76.0	0.86
3150201	8949	ALABAMA R	88.5	70.5	25 - 163	0.0	0.0 - 0.0	4.2	1.4 - 9.7	6.5	1.8 - 18.5	36.5	11.9 - 107.0	0.0	0.0 - 0.0	23.4	8.2 - 56.8	0.86
3150201	8950	OLD TOWN CR	69.2	61.2	21 - 108	0.0	0.0 - 0.0	4.2	1.5 - 7.9	8.0	2.5 - 22.4	20.0	7.6 - 45.0	0.0	0.0 - 0.0	29.0	9.6 - 66.0	0.86
3150201	8951	ALABAMA R	1.0	63.7	19 - 146	0.0	0.0 - 0.0	13.2	3.8 - 31.7	4.0	1.0 - 12.1	19.3	5.9 - 54.4	0.0	0.0 - 0.0	27.3	8.9 - 62.6	0.86
3150201	8952	BIG SWAMP CR	141.7	80.3	29 - 184	0.0	0.0 - 0.0	6.3	2.3 - 14.2	14.1	4.5 - 37.6	30.7	10.8 - 89.7	0.0	0.0 - 0.0	29.2	10.3 - 89.9	0.86
3150201	8954	BUCK CR	28.3	107.9	40 - 214	0.0	0.0 - 0.0	5.5	2.1 - 11.6	21.5	6.7 - 69.8	47.0	16.9 - 112.8	0.0	0.0 - 0.0	33.9	13.2 - 92.9	0.80
3150201	8955	PANTHER CR	65.6	99.4	39 - 226	0.0	0.0 - 0.0	4.8	1.6 - 9.8	17.4	5.6 - 54.7	37.3	13.6 - 124.9	0.0	0.0 - 0.0	39.8	17.7 - 96.5	0.80
3150201	8956	BIG SWAMP CR	11.3	88.5	30 - 158	0.0	0.0 - 0.0	2.1	0.7 - 4.1	13.5	4.4 - 35.3	29.6	9.8 - 61.7	0.0	0.0 - 0.0	43.3	16.9 - 94.9	0.81
3150201	8957	*A	83.2	92.3	32 - 251	0.0	0.0 - 0.0	4.6	1.5 - 12.2	13.4	3.5 - 43.3	29.5	9.9 - 75.8	0.0	0.0 - 0.0	44.8	15.2 - 118.9	0.80
3150201	8958	BIG SWAMP CR	157.7	113.8	34 - 226	0.0	0.0 - 0.0	5.5	1.6 - 10.8	18.3	5.1 - 41.9	40.1	12.6 - 95.1	0.0	0.0 - 0.0	49.8	15.6 - 105.7	0.80
3150201	8959	LAKE CR	11.1	118.2	46 - 225	0.0	0.0 - 0.0	1.4	0.5 - 2.7	18.7	5.6 - 47.3	41.7	14.0 - 102.2	0.0	0.0 - 0.0	56.4	19.6 - 114.4	0.75
3150201	8960	LAKE CR	62.7	36.1	13 - 77	0.0	0.0 - 0.0	3.5	1.2 - 7.6	4.9	1.6 - 16.1	10.4	3.9 - 24.6	0.0	0.0 - 0.0	17.4	6.7 - 45.0	0.72
3150201	8961	DEPOSIT CR	46.2	36.1	11 - 73	0.0	0.0 - 0.0	2.7	1.0 - 5.6	5.2	1.5 - 12.1	11.7	4.1 - 27.0	0.0	0.0 - 0.0	16.5	5.5 - 40.1	0.72
3150201	8962	BIG SWAMP CR	14.1	125.5	41 - 301	0.0	0.0 - 0.0	2.2	0.7 - 5.2	19.9	5.7 - 66.7	44.6	13.9 - 132.8	0.0	0.0 - 0.0	58.8	19.1 - 181.4	0.75
3150201	8963	BIG SWAMP CR	14.2	74.4	25 - 151	0.0	0.0 - 0.0	6.2	2.2 - 13.4	10.7	3.5 - 28.5	22.7	7.9 - 57.8	0.0	0.0 - 0.0	34.8	10.9 - 73.7	0.72
3150201	8964	CHERRY CR	140.7	49.2	15 - 94	0.0	0.0 - 0.0	4.6	1.3 - 10.4	6.6	1.7 - 15.7	14.2	3.8 - 35.7	0.0	0.0 - 0.0	23.8	6.7 - 49.3	0.72
3150201	8965	ALABAMA R	227.2	33.8	11 - 85	0.0	0.0 - 0.0	3.6	1.1 - 9.5	3.8	1.2 - 9.7	16.4	5.0 - 43.4	0.0	0.0 - 0.0	10.0	3.1 - 29.1	0.86
3150201	8966	ALABAMA R	163.3	59.3	22 - 115	0.0	0.0 - 0.0	3.1	1.1 - 6.7	8.7	2.8 - 26.8	33.1	11.5 - 85.5	0.0	0.0 - 0.0	14.4	4.6 - 35.2	0.80
3150201	8967	TALLAWASSEE CI	88.7	78.5	24 - 201	0.0	0.0 - 0.0	5.5	1.7 - 13.7	11.5	3.0 - 34.3	25.3	8.8 - 72.0	0.0	0.0 - 0.0	36.3	10.7 - 93.4	0.80
3150201	8968	ALABAMA R	13.0	69.7	20 - 132	0.0	0.0 - 0.0	10.6	3.0 - 22.4	7.1	2.1 - 17.1	24.7	6.8 - 57.9	0.0	0.0 - 0.0	27.4	8.7 - 59.9	0.80
3150201	8969	PINTALLA CR	61.6	176.2	59 - 338	76.6	27.6 - 165.8	7.5	2.2 - 15.9	13.3	3.7 - 36.7	33.8	11.1 - 80.7	0.0	0.0 - 0.0	44.9	16.4 - 94.1	0.80
3150201	8970	STEER CR	106.0	142.6	46 - 269	0.0	0.0 - 0.0	4.1	1.3 - 7.8	26.2	8.4 - 60.5	57.4	16.8 - 135.1	0.0	0.0 - 0.0	55.0	18.0 - 114.9	0.77
3150201	8971	PINTALLA CR	63.5	163.3	45 - 303	0.0	0.0 - 0.0	6.0	2.0 - 11.3	22.4	6.0 - 60.3	70.1	22.4 - 165.9	0.0	0.0 - 0.0	64.8	18.6 - 133.6	0.77
3150201	8972	PINCHONY CR	230.2	55.8	20 - 119	0.0	0.0 - 0.0	4.6	1.5 - 9.6	8.6	2.8 - 29.5	19.9	6.9 - 50.9	0.0	0.0 - 0.0	22.7	7.7 - 54.3	0.74
3150201	8973	PINTALLA CR	71.3	164.2	56 - 320	0.0	0.0 - 0.0	3.9	1.4 - 8.7	21.1	5.6 - 57.1	74.6	26.0 - 177.8	0.0	0.0 - 0.0	64.6	23.2 - 147.2	0.74
3150201	8974	ALABAMA R	54.8	57.1	19 - 124	0.0	0.0 - 0.0	6.4	2.0 - 12.9	5.3	1.5 - 15.1	31.2	9.2 - 81.5	0.0	0.0 - 0.0	14.4	5.0 - 32.1	0.80
3150201	8975	CATOMA CR	180.4	272.7	93 - 527	143.1	46.7 - 316.1	31.2	11.5 - 60.4	11.9	3.8 - 39.8	42.6	13.9 - 111.3	0.0	0.0 - 0.0	43.9	13.7 - 100.4	0.80
3150201	8976	RAMER CR	217.8	88.2	31 - 182	0.0	0.0 - 0.0	3.0	1.0 - 6.3	10.3	2.9 - 33.4	36.7	12.1 - 85.8	0.0	0.0 - 0.0	38.2	14.3 - 96.4	0.76

8-digit HUC	ID	Name	Area	Catchment Yield		Point sources		Developed Land		Manure		Agricultural Land		Phosphate Mines		Soil parent rock		Frac
				kg/km^2	90% CI	kg/km^2	90% CI	kg/km^2	90% CI	kg/km^2	90% CI	kg/km^2	90% CI	kg/km^2	90% CI	kg/km^2	90% CI	
3150201	8977	CATOMA CR	69.7	134.2	43 - 261	0.0	0.0 - 0.0	18.5	6.5 - 32.3	10.7	3.0 - 27.5	38.0	11.7 - 82.2	0.0	0.0 - 0.0	67.1	21.4 - 148.5	0.76
3150201	8978	CATOMA CR	262.7	29.4	11 - 55	0.4	0.2 - 0.8	3.0	1.1 - 6.0	2.4	0.7 - 5.6	8.5	3.3 - 23.7	0.0	0.0 - 0.0	15.1	5.5 - 33.3	0.73
3150201	8979	LITTLE CATOMA C	142.8	107.4	33 - 243	0.0	0.0 - 0.0	3.3	1.0 - 7.8	12.1	3.7 - 42.7	42.5	16.0 - 100.5	0.0	0.0 - 0.0	49.4	14.7 - 129.9	0.73
3150201	8980	ALABAMA R	16.4	52.9	21 - 119	0.0	0.0 - 0.0	4.9	2.0 - 11.8	5.0	1.8 - 12.7	27.4	11.1 - 74.9	0.0	0.0 - 0.0	15.6	6.4 - 38.6	0.80
3150201	8981	ALABAMA R	17.8	68.3	22 - 170	0.0	0.0 - 0.0	6.7	2.1 - 14.3	8.2	2.2 - 24.9	38.9	12.9 - 109.9	0.0	0.0 - 0.0	14.5	5.0 - 37.9	0.80
3150201	8982	*B	19.7	98.9	38 - 206	0.0	0.0 - 0.0	25.5	8.2 - 54.1	12.2	3.8 - 31.0	44.1	15.8 - 116.7	0.0	0.0 - 0.0	17.3	6.9 - 40.8	0.80
3150201	8983	*C	7.7	79.5	31 - 154	0.0	0.0 - 0.0	34.3	12.5 - 71.9	6.5	2.2 - 16.6	21.3	6.9 - 52.2	0.0	0.0 - 0.0	17.5	6.2 - 43.3	0.75
3150201	8984	*D	32.8	94.3	32 - 226	0.0	0.0 - 0.0	55.6	18.9 - 135.0	5.3	1.5 - 14.2	18.5	5.7 - 58.0	0.0	0.0 - 0.0	14.9	6.2 - 40.1	0.75
3150201	8985	ALABAMA R	13.8	36.3	12 - 82	0.0	0.0 - 0.0	3.0	1.0 - 6.2	2.5	0.8 - 8.8	15.4	4.7 - 38.2	0.0	0.0 - 0.0	15.3	5.1 - 43.0	0.80
3150201	8986	ALABAMA R	84.1	48.4	17 - 96	0.0	0.0 - 0.0	6.5	2.4 - 14.0	4.3	1.3 - 11.8	28.3	9.6 - 67.7	0.0	0.0 - 0.0	9.2	3.2 - 22.2	0.80
3150201	8987	MORTAR CR	32.3	94.3	38 - 205	38.1	16.3 - 82.1	16.2	5.7 - 34.5	4.1	1.3 - 11.9	27.1	10.0 - 69.2	0.0	0.0 - 0.0	8.9	3.4 - 18.7	0.80
3150201	8988	MORTAR CR	131.9	24.9	10 - 60	0.0	0.0 - 0.0	8.0	2.7 - 20.5	1.8	0.6 - 5.2	11.6	4.4 - 32.9	0.0	0.0 - 0.0	3.4	1.3 - 8.5	0.78
3150201	8989	*E	68.4	30.0	10 - 83	0.0	0.0 - 0.0	8.4	2.9 - 23.4	2.4	0.7 - 7.8	15.5	5.6 - 47.8	0.0	0.0 - 0.0	3.7	1.3 - 9.9	0.78
3150201	8990	AUTAUGA CR	57.4	511.1	181 - 923	463.7	161.5 - 855.1	26.7	8.0 - 51.4	1.7	0.5 - 4.1	10.6	3.9 - 21.8	0.0	0.0 - 0.0	8.4	3.0 - 18.3	0.80
3150201	8991	BRIDGE CR	98.3	17.5	6 - 37	0.0	0.0 - 0.0	6.4	2.4 - 13.6	1.1	0.3 - 3.6	6.6	2.1 - 17.1	0.0	0.0 - 0.0	3.4	1.1 - 9.3	0.76
3150201	8992	AUTAUGA CR	159.4	22.9	8 - 50	0.0	0.0 - 0.0	6.7	2.1 - 15.2	1.8	0.5 - 5.2	10.9	3.7 - 28.1	0.0	0.0 - 0.0	3.5	1.2 - 8.5	0.76
3150201	8993	SWIFT CR	404.9	20.3	5 - 41	0.0	0.0 - 0.0	4.8	1.2 - 9.8	1.5	0.3 - 4.3	9.3	2.3 - 25.5	0.0	0.0 - 0.0	4.7	1.1 - 11.6	0.80
3150201	8994	LITTLE MULBERR	314.5	17.4	6 - 33	0.0	0.0 - 0.0	4.4	1.5 - 8.0	1.2	0.4 - 3.0	7.3	2.6 - 17.2	0.0	0.0 - 0.0	4.5	1.6 - 11.1	0.86
3150201	8995	MULBERRY CR	9.3	40.9	11 - 70	0.0	0.0 - 0.0	6.7	1.8 - 12.0	3.2	0.8 - 7.4	20.0	5.8 - 40.5	0.0	0.0 - 0.0	11.1	3.3 - 21.4	0.86
3150201	8996	BUCK CR	108.9	12.4	4 - 30	0.0	0.0 - 0.0	3.1	0.9 - 8.3	0.5	0.1 - 1.7	3.3	1.0 - 9.2	0.0	0.0 - 0.0	5.5	1.5 - 15.5	0.86
3150201	8997	MULBERRY CR	71.0	28.8	10 - 54	0.0	0.0 - 0.0	5.2	1.9 - 9.3	2.0	0.6 - 4.5	12.7	4.4 - 29.2	0.0	0.0 - 0.0	8.8	3.0 - 16.9	0.86
3150201	8998	MULBERRY CR	59.3	25.4	8 - 48	0.0	0.0 - 0.0	8.5	2.7 - 17.9	1.6	0.4 - 3.9	10.6	3.3 - 27.7	0.0	0.0 - 0.0	4.7	1.6 - 9.4	0.80
3150201	8999	MULBERRY CR,E F	11.0	12.0	4 - 19	0.0	0.0 - 0.0	3.6	1.1 - 5.6	0.4	0.1 - 0.9	2.2	0.7 - 4.4	0.0	0.0 - 0.0	5.8	1.9 - 11.4	0.78
3150201	9000	MULBERRY CR,E F	78.2	21.5	10 - 50	0.0	0.0 - 0.0	4.5	2.0 - 10.8	1.6	0.6 - 4.7	10.4	4.5 - 32.3	0.0	0.0 - 0.0	5.0	2.2 - 12.4	0.77
3150201	9001	MULBERRY CR,MI	172.0	35.7	14 - 67	0.0	0.0 - 0.0	10.0	3.7 - 17.4	2.9	0.9 - 7.9	18.0	6.5 - 41.0	0.0	0.0 - 0.0	4.8	1.9 - 10.9	0.77
3150201	9002	MULBERRY CR	94.9	20.5	7 - 44	0.0	0.0 - 0.0	5.5	1.8 - 12.2	1.3	0.4 - 4.3	8.8	2.6 - 24.9	0.0	0.0 - 0.0	5.0	1.7 - 14.3	0.78
3150201	9003	*F	44.7	12.9	4 - 33	0.0	0.0 - 0.0	3.0	1.0 - 7.1	0.7	0.2 - 2.1	4.6	1.6 - 13.5	0.0	0.0 - 0.0	4.5	1.7 - 11.5	0.80
3150201	9004	VALLEY CR	267.1	44.8	13 - 84	16.1	4.8 - 35.5	13.1	4.0 - 25.2	0.9	0.3 - 2.2	5.7	1.6 - 12.5	0.0	0.0 - 0.0	9.0	2.9 - 21.4	0.87
3150202	9005	CAHABA R	13.8	55.0	17 - 152	0.0	0.0 - 0.0	6.6	2.0 - 18.9	2.2	0.6 - 7.8	12.8	3.5 - 35.6	0.0	0.0 - 0.0	33.5	10.4 - 95.3	0.87
3150202	9006	CHILDERS CR	72.3	71.6	28 - 150	0.0	0.0 - 0.0	8.4	2.9 - 22.2	5.0	1.6 - 14.3	31.2	11.5 - 84.6	0.0	0.0 - 0.0	27.0	11.1 - 68.2	0.86
3150202	9007	CAHABA R	69.1	84.3	31 - 239	0.0	0.0 - 0.0	6.6	2.3 - 16.6	5.9	1.9 - 19.6	36.5	12.2 - 114.1	0.0	0.0 - 0.0	35.3	13.1 - 99.1	0.86
3150202	9008	OAKMULGEE CR	93.0	27.3	10 - 59	0.0	0.0 - 0.0	5.2	1.8 - 13.0	1.5	0.5 - 3.7	9.7	3.3 - 26.0	0.0	0.0 - 0.0	10.8	4.1 - 27.6	0.85
3150202	9009	LITTLE OAKMULG	118.8	9.0	3 - 19	0.0	0.0 - 0.0	3.3	1.1 - 6.2	0.2	0.0 - 0.4	1.0	0.3 - 2.3	0.0	0.0 - 0.0	4.5	1.4 - 10.2	0.83
3150202	9010	OAKMULGEE CR	123.3	13.3	5 - 28	0.0	0.0 - 0.0	4.3	1.5 - 9.2	0.5	0.1 - 1.2	3.0	1.0 - 7.8	0.0	0.0 - 0.0	5.4	1.9 - 13.7	0.83
3150202	9011	OAKMULGEE CR	212.1	14.2	5 - 32	0.0	0.0 - 0.0	4.7	1.7 - 10.6	0.7	0.2 - 2.1	4.6	1.5 - 12.9	0.0	0.0 - 0.0	4.3	1.5 - 11.5	0.79
3150202	9012	BEAVERDAM CR	87.0	11.2	4 - 22	0.0	0.0 - 0.0	4.8	1.5 - 9.7	0.3	0.1 - 0.7	1.9	0.7 - 4.4	0.0	0.0 - 0.0	4.2	1.4 - 9.1	0.79
3150202	9013	CAHABA R	77.3	29.0	11 - 72	0.0	0.0 - 0.0	3.6	1.3 - 9.7	1.5	0.5 - 4.6	10.1	3.7 - 25.2	0.0	0.0 - 0.0	13.8	4.7 - 38.0	0.85
3150202	9014	CAHABA R	25.2	30.6	11 - 66	0.0	0.0 - 0.0	4.7	1.7 - 9.3	1.3	0.4 - 3.9	9.0	2.7 - 24.6	0.0	0.0 - 0.0	15.6	5.4 - 38.9	0.84
3150202	9015	WATERS CR	56.1	16.0	5 - 39	0.0	0.0 - 0.0	4.9	1.5 - 11.0	0.6	0.2 - 1.6	3.8	1.3 - 11.0	0.0	0.0 - 0.0	6.7	2.2 - 18.5	0.84

8-digit HUC	ID	Name	Area	Catchment Yield		Point sources		Developed Land		Manure		Agricultural Land		Phosphate Mines		Soil parent rock		Frac
				kg/km^2	90% CI	kg/km^2	90% CI	kg/km^2	90% CI	kg/km^2	90% CI	kg/km^2	90% CI	kg/km^2	90% CI	kg/km^2	90% CI	
3150202	9016	CAHABA R	415.6	18.0	6 - 37	0.0	0.0 - 0.0	4.6	1.5 - 9.4	0.8	0.2 - 2.2	5.2	1.6 - 14.3	0.0	0.0 - 0.0	7.4	2.4 - 15.9	0.84
3150202	9017	CAHABA R	60.8	17.1	6 - 42	0.0	0.0 - 0.0	4.1	1.3 - 8.8	0.6	0.2 - 2.3	4.5	1.5 - 11.5	0.0	0.0 - 0.0	7.9	2.7 - 19.8	0.82
3150202	9018	CAHABA R	77.5	24.3	8 - 51	0.0	0.0 - 0.0	5.1	1.6 - 10.9	1.3	0.4 - 4.2	9.8	3.3 - 27.5	0.0	0.0 - 0.0	8.1	2.7 - 19.6	0.81
3150202	9019	SANDY CR	77.6	17.6	6 - 36	0.0	0.0 - 0.0	7.1	2.6 - 13.6	0.6	0.2 - 1.6	4.7	1.6 - 10.8	0.0	0.0 - 0.0	5.2	1.8 - 12.7	0.81
3150202	9021	CAHABA R	10.9	188.5	61 - 392	110.6	38.0 - 229.2	28.8	9.2 - 59.3	3.8	1.1 - 9.3	29.5	8.8 - 70.5	0.0	0.0 - 0.0	15.8	5.8 - 34.1	0.81
3150202	9022	CAHABA R	65.7	22.4	8 - 44	0.0	0.0 - 0.0	4.6	1.6 - 9.6	1.1	0.3 - 3.0	4.8	1.7 - 13.1	0.0	0.0 - 0.0	11.9	4.1 - 28.3	0.80
3150202	9023	LITTLE CAHABA R	7.9	13.9	5 - 30	0.0	0.0 - 0.0	1.4	0.5 - 3.0	0.7	0.2 - 2.0	1.3	0.4 - 3.3	0.0	0.0 - 0.0	10.5	3.9 - 25.1	0.79
3150202	9024	SIXMILE CR	214.7	20.3	8 - 45	0.0	0.0 - 0.0	5.3	1.9 - 14.0	1.2	0.4 - 3.5	7.0	2.6 - 20.8	0.0	0.0 - 0.0	6.8	2.4 - 15.5	0.79
3150202	9025	LITTLE CAHABA R	85.3	23.5	8 - 60	0.0	0.0 - 0.0	5.5	2.0 - 12.8	1.4	0.4 - 4.1	5.9	2.0 - 15.7	0.0	0.0 - 0.0	10.7	3.3 - 29.5	0.79
3150202	9026	MAHAN CR	168.7	32.8	12 - 61	0.0	0.0 - 0.0	7.0	2.4 - 11.5	2.3	0.7 - 5.8	12.9	4.7 - 31.2	0.0	0.0 - 0.0	10.6	3.7 - 20.8	0.75
3150202	9027	SHOAL CR	230.4	43.6	14 - 102	2.8	1.0 - 6.5	15.8	5.3 - 36.5	1.3	0.4 - 4.4	11.8	3.8 - 30.8	0.0	0.0 - 0.0	12.0	4.2 - 31.3	0.75
3150202	9028	CAHABA R	242.6	16.8	6 - 29	0.0	0.0 - 0.0	7.0	2.5 - 14.8	0.5	0.2 - 1.2	1.7	0.6 - 4.1	0.0	0.0 - 0.0	7.5	2.8 - 14.4	0.79
3150202	9029	CAHABA R	183.5	14.3	5 - 27	0.0	0.0 - 0.0	5.6	1.8 - 10.0	0.4	0.1 - 1.2	2.3	0.7 - 5.2	0.0	0.0 - 0.0	6.0	2.3 - 13.1	0.77
3150202	9030	*A	15.7	269.7	102 - 553	186.3	70.4 - 387.8	71.6	26.8 - 149.3	0.3	0.1 - 0.8	1.5	0.5 - 3.5	0.0	0.0 - 0.0	10.1	3.7 - 25.9	0.73
3150202	9031	*B	99.3	180.8	65 - 346	124.8	48.1 - 247.3	41.5	14.9 - 78.5	0.5	0.2 - 1.3	3.5	1.3 - 7.4	0.0	0.0 - 0.0	10.4	3.8 - 23.3	0.71
3150202	9032	*A	5.6	119.9	39 - 256	0.0	0.0 - 0.0	102.8	32.9 - 225.7	0.1	0.0 - 0.5	0.9	0.3 - 2.5	0.0	0.0 - 0.0	16.1	4.9 - 39.7	0.71
3150202	9033	CAHABA R	158.2	232.2	83 - 492	146.9	53.3 - 325.1	76.5	26.3 - 179.3	0.2	0.1 - 0.5	1.9	0.6 - 4.5	0.0	0.0 - 0.0	6.7	2.4 - 15.9	0.73
3150202	9034	*C	126.4	62.6	17 - 128	55.8	15.1 - 115.0	4.5	1.1 - 9.3	0.2	0.0 - 0.4	0.6	0.2 - 1.5	0.0	0.0 - 0.0	1.4	0.4 - 3.1	0.69
3150202	9035	CAHABA R	22.0	72.8	20 - 188	0.0	0.0 - 0.0	66.0	18.3 - 173.2	0.1	0.0 - 0.2	0.7	0.2 - 2.0	0.0	0.0 - 0.0	6.1	2.0 - 15.9	0.69
3150202	9036	BIG BLACK CR	106.0	36.3	13 - 73	14.1	5.1 - 28.8	6.2	2.2 - 13.7	2.9	1.0 - 7.7	5.0	1.5 - 11.9	0.0	0.0 - 0.0	8.1	2.6 - 19.8	0.64
3150202	9037	*D	134.9	62.6	22 - 107	25.1	9.0 - 48.7	21.7	6.9 - 41.8	0.9	0.3 - 2.5	6.4	2.3 - 14.4	0.0	0.0 - 0.0	8.6	3.3 - 19.5	0.64
3150202	9038	SHADES CR	361.6	56.2	18 - 99	0.0	0.0 - 0.0	39.9	13.1 - 80.7	0.7	0.2 - 2.1	5.5	1.7 - 13.6	0.0	0.0 - 0.0	10.1	3.3 - 20.8	0.77
3150202	9039	SHULTZ CR	35.4	12.0	5 - 27	0.0	0.0 - 0.0	3.6	1.4 - 9.1	0.2	0.1 - 0.5	0.9	0.3 - 2.3	0.0	0.0 - 0.0	7.3	3.0 - 17.9	0.80
3150202	9040	HILL CR	107.7	18.5	6 - 43	0.0	0.0 - 0.0	6.5	2.1 - 14.5	0.8	0.2 - 2.4	3.8	1.1 - 10.1	0.0	0.0 - 0.0	7.4	2.6 - 16.4	0.78
3150202	9041	SHULTZ CR	50.3	11.8	4 - 23	0.0	0.0 - 0.0	3.2	1.2 - 7.3	0.3	0.1 - 1.0	2.6	0.9 - 5.7	0.0	0.0 - 0.0	5.7	2.0 - 14.3	0.78
3150202	9042	HAYSOP CR	116.6	19.0	7 - 34	0.0	0.0 - 0.0	7.7	2.5 - 13.1	0.7	0.2 - 1.8	5.1	1.7 - 13.1	0.0	0.0 - 0.0	5.5	2.2 - 11.9	0.81
3150202	9043	AFFOHEE CR	104.8	12.4	4 - 27	0.0	0.0 - 0.0	3.8	1.3 - 9.0	0.4	0.1 - 1.2	3.0	0.9 - 8.2	0.0	0.0 - 0.0	5.1	1.3 - 14.3	0.81
3150202	9044	BLUE OUTEE CR	117.7	11.4	4 - 24	0.0	0.0 - 0.0	3.7	1.2 - 8.4	0.3	0.1 - 0.8	2.2	0.8 - 5.3	0.0	0.0 - 0.0	5.2	1.9 - 14.4	0.82
3150202	9045	RICE CR	87.2	32.3	11 - 78	0.0	0.0 - 0.0	9.0	3.1 - 20.7	1.5	0.5 - 3.9	10.2	3.0 - 23.8	0.0	0.0 - 0.0	11.6	4.2 - 30.2	0.84
3150203	9046	ALABAMA R	58.8	29.0	10 - 72	0.0	0.0 - 0.0	3.6	1.4 - 9.7	3.0	0.9 - 9.5	10.5	3.8 - 30.4	0.0	0.0 - 0.0	11.8	4.1 - 36.1	0.95
3150203	9047	ALABAMA R	76.2	35.5	11 - 71	0.0	0.0 - 0.0	3.3	0.9 - 7.8	4.2	1.2 - 12.2	14.1	4.7 - 35.2	0.0	0.0 - 0.0	13.9	4.3 - 32.6	0.95
3150203	9048	PURSLEY CR	1.8	23.3	7 - 63	0.0	0.0 - 0.0	2.4	0.8 - 6.0	0.5	0.1 - 1.7	1.4	0.5 - 4.1	0.0	0.0 - 0.0	19.0	6.3 - 56.3	0.95
3150203	9049	GRAVEL CR	77.7	12.7	4 - 25	0.0	0.0 - 0.0	2.6	1.0 - 5.8	0.6	0.2 - 1.4	2.1	0.7 - 5.5	0.0	0.0 - 0.0	7.5	2.6 - 16.5	0.94
3150203	9050	PURSLEY CR	191.5	23.6	9 - 53	0.0	0.0 - 0.0	5.4	1.9 - 11.9	1.7	0.6 - 4.9	5.6	1.8 - 14.9	0.0	0.0 - 0.0	10.9	4.0 - 29.3	0.94
3150203	9051	ALABAMA R	82.0	235.3	82 - 525	185.4	64.5 - 431.0	4.0	1.3 - 9.9	3.8	1.1 - 10.4	12.2	4.1 - 31.7	0.0	0.0 - 0.0	29.9	10.0 - 67.5	0.95
3150203	9052	ALABAMA R	28.8	32.1	11 - 79	0.0	0.0 - 0.0	3.0	1.0 - 6.5	1.5	0.4 - 4.5	4.9	1.5 - 13.1	0.0	0.0 - 0.0	22.7	7.5 - 59.8	0.94
3150203	9053	ALABAMA R	4.9	71.8	25 - 197	0.0	0.0 - 0.0	2.1	0.7 - 4.8	4.6	1.4 - 14.8	15.8	4.8 - 44.0	0.0	0.0 - 0.0	49.3	16.4 - 143.5	0.94
3150203	9054	ROCKWEST CR	67.9	40.6	13 - 102	0.0	0.0 - 0.0	9.7	3.2 - 22.0	3.7	1.0 - 10.1	12.3	4.0 - 31.1	0.0	0.0 - 0.0	14.8	4.6 - 40.8	0.94
3150203	9055	ALABAMA R	42.3	57.9	19 - 147	0.0	0.0 - 0.0	5.7	1.7 - 13.0	6.6	1.9 - 20.4	21.5	6.8 - 57.4	0.0	0.0 - 0.0	24.1	7.1 - 71.5	0.94

8-digit HUC	ID	Name	Area	Catchment Yield		Point sources		Developed Land		Manure		Agricultural Land		Phosphate Mines		Soil parent rock		Frac
				kg/km2	90% CI	kg/km2	90% CI	kg/km2	90% CI	kg/km2	90% CI	kg/km2	90% CI	kg/km2	90% CI	kg/km2	90% CI	
3150203	9056	PINE BARRON CR	180.5	41.7	16 - 85	0.0	0.0 - 0.0	3.7	1.3 - 7.3	3.4	1.1 - 8.6	13.2	4.5 - 32.5	0.0	0.0 - 0.0	21.4	7.5 - 53.0	0.87
3150203	9057	PRAIRIE CR	65.3	27.6	10 - 43	0.0	0.0 - 0.0	1.7	0.6 - 2.9	3.1	1.1 - 7.7	10.3	3.5 - 21.1	0.0	0.0 - 0.0	12.5	4.4 - 23.6	0.79
3150203	9058	PINE BARRON CR	12.5	31.8	11 - 61	0.0	0.0 - 0.0	5.4	1.7 - 12.2	2.6	0.7 - 6.6	8.6	3.1 - 20.8	0.0	0.0 - 0.0	15.2	4.9 - 32.2	0.79
3150203	9059	BEAR CR	197.4	15.5	6 - 29	0.0	0.0 - 0.0	2.7	0.9 - 5.4	0.9	0.2 - 2.4	3.1	1.0 - 7.2	0.0	0.0 - 0.0	8.8	2.9 - 18.8	0.75
3150203	9060	PINE BARRON CR	30.4	25.5	9 - 59	0.0	0.0 - 0.0	4.6	1.6 - 11.5	1.4	0.4 - 4.9	5.0	1.7 - 14.1	0.0	0.0 - 0.0	14.4	5.2 - 36.9	0.75
3150203	9061	TURKEY CR	63.1	17.5	6 - 38	0.0	0.0 - 0.0	3.8	1.1 - 8.4	1.1	0.3 - 2.9	4.1	1.3 - 11.5	0.0	0.0 - 0.0	8.5	2.4 - 19.6	0.73
3150203	9062	PINE BARRON CR	112.1	25.3	10 - 49	0.0	0.0 - 0.0	2.4	0.8 - 4.9	2.8	1.1 - 6.0	7.9	3.0 - 17.3	0.0	0.0 - 0.0	12.1	5.2 - 25.9	0.73
3150203	9063	STURDEVANT CR	138.8	17.0	6 - 55	0.0	0.0 - 0.0	3.1	0.9 - 8.7	2.1	0.6 - 7.0	5.5	1.8 - 21.4	0.0	0.0 - 0.0	6.3	2.1 - 21.0	0.63
3150203	9064	PINE BARRON CR	74.9	22.6	8 - 47	0.0	0.0 - 0.0	5.1	1.5 - 10.3	4.1	1.2 - 9.8	6.7	2.3 - 18.0	0.0	0.0 - 0.0	6.8	2.1 - 15.0	0.63
3150203	9065	ALABAMA R	4.5	79.0	25 - 167	0.0	0.0 - 0.0	3.6	1.1 - 7.4	3.6	0.9 - 9.8	22.7	7.2 - 59.0	0.0	0.0 - 0.0	49.0	14.2 - 124.8	0.87
3150203	9066	ALABAMA R	7.2	48.1	16 - 97	0.0	0.0 - 0.0	2.9	0.9 - 6.1	1.3	0.4 - 3.2	7.7	2.5 - 17.8	0.0	0.0 - 0.0	36.2	11.4 - 72.4	0.87
3150203	9067	ALABAMA R	197.2	38.0	14 - 73	0.0	0.0 - 0.0	4.0	1.5 - 8.2	1.7	0.6 - 4.9	10.7	3.9 - 24.0	0.0	0.0 - 0.0	21.5	7.8 - 48.6	0.87
3150203	9068	CEDAR CR	27.8	66.6	21 - 124	0.0	0.0 - 0.0	4.2	1.2 - 8.2	4.0	1.2 - 10.8	24.5	7.9 - 61.5	0.0	0.0 - 0.0	33.8	10.4 - 71.0	0.87
3150203	9069	CEDAR CR	104.6	42.4	15 - 92	0.0	0.0 - 0.0	3.7	1.3 - 6.9	2.0	0.6 - 5.8	12.2	4.4 - 30.9	0.0	0.0 - 0.0	24.4	8.2 - 61.0	0.85
3150203	9070	CEDAR CR	198.0	38.8	12 - 86	0.0	0.0 - 0.0	4.1	1.4 - 8.7	3.9	1.1 - 11.4	13.1	4.6 - 34.0	0.0	0.0 - 0.0	17.7	5.6 - 44.8	0.82
3150203	9071	WOLF CR	118.1	17.4	6 - 33	0.0	0.0 - 0.0	3.0	1.1 - 5.8	1.9	0.6 - 4.4	3.2	1.1 - 8.2	0.0	0.0 - 0.0	9.3	3.3 - 19.1	0.77
3150203	9072	CEDAR CR	280.4	19.5	5 - 41	0.0	0.0 - 0.0	2.9	0.8 - 5.8	1.7	0.4 - 4.9	3.2	0.9 - 7.6	0.0	0.0 - 0.0	11.7	3.2 - 26.7	0.77
3150203	9073	DRY CEDAR CR	334.6	29.1	9 - 56	0.0	0.0 - 0.0	4.0	1.3 - 7.4	2.7	0.8 - 7.5	6.8	2.1 - 15.8	0.0	0.0 - 0.0	15.6	4.8 - 38.0	0.82
3150203	9074	MUSH CR	155.5	41.9	14 - 80	0.0	0.0 - 0.0	5.3	1.8 - 12.4	2.8	0.8 - 7.5	13.2	4.7 - 30.9	0.0	0.0 - 0.0	20.5	6.5 - 42.1	0.85
3150203	9075	ALABAMA R	1.1	80.3	26 - 182	0.0	0.0 - 0.0	1.2	0.4 - 2.9	5.1	1.5 - 17.6	37.5	10.9 - 108.5	0.0	0.0 - 0.0	36.5	11.1 - 91.3	0.87
3150203	9076	ALABAMA R	94.1	60.4	26 - 114	0.0	0.0 - 0.0	4.8	1.8 - 9.5	3.4	1.2 - 9.6	21.3	8.8 - 53.9	0.0	0.0 - 0.0	31.0	12.7 - 76.1	0.87
3150203	9077	BIG SWAMP CR	130.4	63.4	23 - 130	0.0	0.0 - 0.0	5.1	1.5 - 11.2	3.7	1.2 - 9.7	23.2	9.0 - 56.6	0.0	0.0 - 0.0	31.5	10.6 - 76.3	0.87
3150203	9078	BOGUE CHITTO CR	105.2	74.3	29 - 183	0.0	0.0 - 0.0	5.0	1.7 - 12.0	3.1	0.9 - 9.7	19.5	7.2 - 48.9	0.0	0.0 - 0.0	46.7	18.3 - 142.0	0.87
3150203	9079	BOGUE CHITTO CR	85.1	126.5	43 - 262	0.0	0.0 - 0.0	6.4	2.1 - 14.0	7.9	2.1 - 26.1	49.5	17.4 - 134.9	0.0	0.0 - 0.0	62.8	23.5 - 154.1	0.84
3150203	9080	CHANEY CR	115.8	137.3	50 - 307	0.0	0.0 - 0.0	4.7	1.6 - 9.1	9.7	2.8 - 26.2	60.4	20.2 - 147.0	0.0	0.0 - 0.0	62.4	22.3 - 146.1	0.83
3150203	9081	BOGUE CHITTO CR	11.7	101.0	32 - 212	0.0	0.0 - 0.0	3.0	1.0 - 6.0	4.2	1.2 - 14.1	27.7	9.4 - 59.8	0.0	0.0 - 0.0	66.1	23.4 - 174.6	0.83
3150203	9082	BOGUE CHITTO CR	63.3	132.7	49 - 328	0.0	0.0 - 0.0	4.2	1.6 - 9.0	9.0	2.8 - 26.3	55.7	20.2 - 161.6	0.0	0.0 - 0.0	63.8	22.4 - 156.2	0.82
3150203	9083	BOGUE CHITTO CR	124.7	46.6	15 - 90	0.0	0.0 - 0.0	5.4	1.6 - 11.1	2.8	0.9 - 7.9	18.3	5.9 - 41.6	0.0	0.0 - 0.0	20.0	6.1 - 43.2	0.78
3150203	9084	WASHINGTON TR	89.0	95.8	31 - 229	0.0	0.0 - 0.0	3.7	1.2 - 8.7	7.1	2.1 - 21.8	45.8	13.4 - 116.5	0.0	0.0 - 0.0	39.2	12.8 - 102.8	0.78
3150203	9085	MUD CR	234.9	130.3	49 - 365	0.0	0.0 - 0.0	6.0	2.4 - 18.1	8.9	2.5 - 35.0	57.1	19.3 - 173.8	0.0	0.0 - 0.0	58.3	24.2 - 169.7	0.82
3150203	9086	BEAR CR	91.9	108.5	38 - 240	0.0	0.0 - 0.0	4.0	1.3 - 9.8	7.8	2.2 - 21.8	48.5	16.7 - 144.5	0.0	0.0 - 0.0	48.3	16.8 - 105.5	0.84
3150203	9087	CHILATCHEE CR	395.4	58.2	21 - 146	0.0	0.0 - 0.0	3.1	1.1 - 7.3	2.7	0.7 - 7.6	15.5	4.8 - 43.6	0.0	0.0 - 0.0	37.0	12.6 - 99.0	0.87
3150203	9088	DIXON CR	108.2	35.5	13 - 91	0.0	0.0 - 0.0	1.9	0.6 - 4.7	1.9	0.6 - 5.0	6.5	2.2 - 16.8	0.0	0.0 - 0.0	25.2	9.4 - 66.0	0.94
3150203	9089	BEAVER CR	11.4	50.9	21 - 110	0.0	0.0 - 0.0	2.6	0.9 - 6.1	5.0	1.6 - 14.9	15.8	5.1 - 41.6	0.0	0.0 - 0.0	27.5	10.6 - 72.7	0.94
3150203	9090	RED CR	78.5	26.1	10 - 71	0.0	0.0 - 0.0	2.9	1.1 - 8.9	0.7	0.2 - 2.2	2.3	0.9 - 6.1	0.0	0.0 - 0.0	20.3	7.7 - 51.3	0.94
3150203	9091	BEAVER CR	16.3	44.5	17 - 100	0.0	0.0 - 0.0	2.5	0.9 - 5.5	1.2	0.4 - 3.0	4.2	1.6 - 10.2	0.0	0.0 - 0.0	36.6	13.4 - 86.7	0.94
3150203	9092	GOOSE CR	155.1	30.0	11 - 59	0.0	0.0 - 0.0	3.7	1.2 - 7.2	1.1	0.3 - 2.8	4.3	1.1 - 10.2	0.0	0.0 - 0.0	21.0	8.2 - 45.0	0.93
3150203	9093	TURKEY CR	168.2	19.9	7 - 38	0.9	0.3 - 1.9	6.4	2.3 - 12.1	0.7	0.3 - 2.0	2.7	1.0 - 5.9	0.0	0.0 - 0.0	9.2	3.5 - 21.0	0.93
3150203	9094	JAMES CR	41.9	17.6	7 - 32	0.0	0.0 - 0.0	4.0	1.4 - 8.1	0.4	0.2 - 0.9	1.8	0.7 - 4.6	0.0	0.0 - 0.0	11.3	4.6 - 23.8	0.91

8-digit HUC	ID	Name	Area	Catchment Yield		Point sources		Developed Land		Manure		Agricultural Land		Phosphate Mines		Soil parent rock		Frac
				kg/km^2	90% CI	kg/km^2	90% CI	kg/km^2	90% CI	kg/km^2	90% CI	kg/km^2	90% CI	kg/km^2	90% CI	kg/km^2	90% CI	
3150203	9095	BAPTIZING CR	2.6	41.8	15 - 96	0.0	0.0 - 0.0	3.1	1.0 - 6.8	4.8	1.4 - 12.8	17.2	6.0 - 41.8	0.0	0.0 - 0.0	16.8	6.4 - 46.9	0.91
3150203	9096	BAPTIZING CR	110.5	18.7	8 - 41	0.0	0.0 - 0.0	4.0	1.7 - 9.6	1.0	0.4 - 3.1	4.1	1.8 - 10.5	0.0	0.0 - 0.0	9.5	4.2 - 23.9	0.90
3150203	9097	DRY CR	83.8	15.6	5 - 28	0.0	0.0 - 0.0	4.0	1.1 - 8.2	0.8	0.2 - 1.7	3.7	0.9 - 8.8	0.0	0.0 - 0.0	7.1	2.3 - 14.3	0.90
3150203	9098	BEAR CR	136.5	10.8	3 - 22	0.0	0.0 - 0.0	2.1	0.6 - 3.9	0.2	0.1 - 0.5	0.6	0.2 - 1.6	0.0	0.0 - 0.0	7.9	2.3 - 20.2	0.95
3150203	9099	MCCALLS CR	113.0	25.9	9 - 41	0.0	0.0 - 0.0	5.5	2.0 - 9.4	2.9	0.9 - 6.7	9.7	3.3 - 20.2	0.0	0.0 - 0.0	7.8	2.3 - 17.1	0.95
3150204	9100	ALABAMA R	33.6	27.7	9 - 60	0.0	0.0 - 0.0	0.0	0.0 - 0.0	0.3	0.1 - 0.7	2.1	0.6 - 4.9	0.0	0.0 - 0.0	25.3	8.1 - 55.6	0.99
3150204	9101	PINE LOG CR	215.3	15.2	4 - 28	0.0	0.0 - 0.0	2.4	0.6 - 5.2	0.5	0.1 - 1.4	3.4	0.9 - 8.8	0.0	0.0 - 0.0	8.9	2.5 - 18.9	0.99
3150204	9102	ALABAMA R	179.8	27.0	11 - 59	0.0	0.0 - 0.0	1.5	0.6 - 2.9	0.5	0.2 - 1.5	3.7	1.5 - 9.5	0.0	0.0 - 0.0	21.3	8.2 - 58.0	0.99
3150204	9103	*A	99.4	20.7	8 - 40	0.0	0.0 - 0.0	3.3	1.2 - 6.3	0.6	0.2 - 1.4	4.7	1.7 - 10.9	0.0	0.0 - 0.0	12.1	4.4 - 26.3	0.99
3150204	9104	ALABAMA R	13.5	39.3	14 - 98	0.0	0.0 - 0.0	1.1	0.4 - 2.7	1.1	0.4 - 3.4	7.5	2.7 - 17.8	0.0	0.0 - 0.0	29.7	10.0 - 81.1	0.99
3150204	9105	LITTLE R	373.3	35.9	15 - 72	0.0	0.0 - 0.0	4.6	1.8 - 10.2	2.4	0.8 - 5.8	18.1	6.6 - 44.8	0.0	0.0 - 0.0	10.7	4.2 - 22.2	0.99
3150204	9106	ALABAMA R	104.6	19.6	8 - 39	0.0	0.0 - 0.0	5.7	2.3 - 13.2	0.6	0.2 - 1.8	3.2	1.1 - 7.4	0.0	0.0 - 0.0	10.0	3.7 - 24.6	0.99
3150204	9107	*B	56.6	23.9	9 - 47	0.0	0.0 - 0.0	4.8	1.5 - 9.7	1.5	0.4 - 3.6	9.5	3.1 - 24.2	0.0	0.0 - 0.0	8.0	2.7 - 16.9	0.98
3150204	9108	ALABAMA R	12.0	42.3	14 - 96	0.0	0.0 - 0.0	2.9	0.9 - 6.1	1.4	0.4 - 3.9	8.1	2.9 - 19.4	0.0	0.0 - 0.0	30.0	9.3 - 76.6	0.98
3150204	9109	ALABAMA R	12.1	36.5	13 - 77	0.0	0.0 - 0.0	3.5	1.2 - 8.0	0.9	0.3 - 2.7	4.4	1.8 - 11.1	0.0	0.0 - 0.0	27.7	9.4 - 65.1	0.98
3150204	9110	WALLERS CR	88.6	25.7	8 - 57	0.0	0.0 - 0.0	3.6	1.1 - 8.5	2.0	0.5 - 5.0	12.6	4.1 - 30.6	0.0	0.0 - 0.0	7.6	2.0 - 17.3	0.98
3150204	9111	ALABAMA R	103.4	18.5	8 - 37	0.0	0.0 - 0.0	2.2	0.9 - 4.0	1.0	0.3 - 2.2	5.7	2.0 - 14.5	0.0	0.0 - 0.0	9.7	4.0 - 23.6	0.98
3150204	9113	LOVETTS CR	104.0	35.2	13 - 76	0.0	0.0 - 0.0	3.8	1.5 - 7.8	2.9	1.0 - 7.5	18.2	5.9 - 47.2	0.0	0.0 - 0.0	10.3	4.0 - 22.8	0.98
3150204	9114	RANDONS CR	20.5	19.2	6 - 44	0.0	0.0 - 0.0	2.4	0.8 - 5.9	1.5	0.4 - 4.3	8.5	2.6 - 23.7	0.0	0.0 - 0.0	6.8	2.4 - 15.3	0.98
3150204	9115	BEAR CR	49.8	42.4	14 - 100	0.0	0.0 - 0.0	7.8	2.6 - 16.9	3.4	1.2 - 10.5	21.5	6.5 - 66.4	0.0	0.0 - 0.0	9.7	3.6 - 23.8	0.95
3150204	9116	RANDONS CR	72.7	27.9	11 - 68	0.0	0.0 - 0.0	6.3	2.2 - 16.1	2.1	0.8 - 6.7	13.3	4.9 - 35.6	0.0	0.0 - 0.0	6.2	2.4 - 15.4	0.95
3150204	9117	ALABAMA R	360.6	19.7	7 - 46	0.0	0.0 - 0.0	2.6	0.8 - 5.8	1.1	0.3 - 3.4	6.0	1.9 - 15.8	0.0	0.0 - 0.0	10.1	3.4 - 26.2	0.98
3150204	9118	LIMESTONE CR	188.8	34.5	12 - 61	6.1	2.1 - 12.9	11.4	3.7 - 23.6	1.2	0.3 - 3.1	7.5	2.2 - 17.5	0.0	0.0 - 0.0	8.4	2.8 - 19.1	0.98
3150204	9120	LIMESTONE CR	100.2	36.9	12 - 73	11.0	3.8 - 24.5	9.5	3.2 - 20.1	1.4	0.4 - 3.8	8.8	2.8 - 21.0	0.0	0.0 - 0.0	6.3	2.4 - 16.3	0.94
3150204	9121	WALKERS CR	45.2	13.0	5 - 33	0.0	0.0 - 0.0	3.2	1.1 - 7.1	0.5	0.1 - 1.6	2.9	1.2 - 8.2	0.0	0.0 - 0.0	6.5	2.6 - 16.1	0.94
3150204	9122	BRUSHEY CR	130.6	15.5	6 - 32	0.0	0.0 - 0.0	5.0	1.8 - 10.4	0.6	0.2 - 1.7	3.6	1.2 - 10.4	0.0	0.0 - 0.0	6.3	2.3 - 15.2	0.95
3150204	9123	ALABAMA R	2.7	39.1	13 - 97	0.0	0.0 - 0.0	0.0	0.0 - 0.0	0.8	0.2 - 2.6	3.5	1.1 - 10.0	0.0	0.0 - 0.0	34.8	11.6 - 90.8	0.98
3150204	9124	BIG FLAT CR	71.8	25.2	10 - 60	0.0	0.0 - 0.0	2.2	0.8 - 5.4	1.4	0.5 - 3.9	8.8	3.1 - 24.2	0.0	0.0 - 0.0	12.8	4.8 - 31.8	0.98
3150204	9126	BIG FLAT CR	143.9	12.9	5 - 27	0.0	0.0 - 0.0	2.1	0.7 - 4.8	0.4	0.1 - 1.3	2.6	1.0 - 6.1	0.0	0.0 - 0.0	7.7	2.6 - 18.6	0.97
3150204	9127	ROBINSON CR	180.5	17.3	7 - 42	0.4	0.2 - 1.0	3.7	1.5 - 8.5	0.7	0.2 - 2.1	4.2	1.6 - 12.5	0.0	0.0 - 0.0	8.3	3.1 - 22.0	0.93
3150204	9128	BIG FLAT CR	197.1	17.1	6 - 35	0.0	0.0 - 0.0	2.0	0.6 - 4.8	0.7	0.2 - 1.7	4.2	1.4 - 11.3	0.0	0.0 - 0.0	10.2	3.2 - 23.1	0.93
3150204	9129	BIG FLAT CR	81.0	11.9	4 - 23	0.0	0.0 - 0.0	2.3	0.7 - 4.1	0.2	0.1 - 0.5	0.9	0.3 - 2.2	0.0	0.0 - 0.0	8.5	2.7 - 17.5	0.84
3150204	9130	*C	43.3	12.7	4 - 23	0.0	0.0 - 0.0	3.1	1.0 - 5.7	0.3	0.1 - 0.7	1.3	0.4 - 3.1	0.0	0.0 - 0.0	7.9	2.5 - 16.7	0.84
3150204	9131	HOLLY MILL CR	31.8	20.5	6 - 44	0.0	0.0 - 0.0	6.0	1.8 - 13.4	1.2	0.4 - 3.7	7.7	2.3 - 21.5	0.0	0.0 - 0.0	5.6	1.8 - 13.2	0.97
3150204	9132	BRADLEY MILL CI	46.2	18.8	8 - 48	0.0	0.0 - 0.0	4.4	1.6 - 10.9	1.1	0.4 - 3.4	7.0	2.8 - 22.0	0.0	0.0 - 0.0	6.4	2.4 - 17.3	0.97
3150204	9133	ALABAMA R	39.8	18.6	5 - 39	0.0	0.0 - 0.0	1.6	0.4 - 3.4	0.9	0.2 - 2.3	5.6	1.5 - 13.6	0.0	0.0 - 0.0	10.6	3.2 - 24.9	0.98
3150204	9134	ALABAMA R	286.3	17.1	5 - 34	0.0	0.0 - 0.0	2.5	0.8 - 5.8	0.6	0.2 - 1.4	3.4	0.9 - 8.5	0.0	0.0 - 0.0	10.5	2.7 - 22.0	0.95
3150204	9135	TALLAHATCHEE C	104.0	19.3	7 - 41	0.0	0.0 - 0.0	3.1	1.1 - 6.7	1.2	0.4 - 3.1	5.1	1.5 - 12.8	0.0	0.0 - 0.0	9.9	3.5 - 25.9	0.95
3150204	9137	SILVER CR	90.7	7.9	3 - 21	0.0	0.0 - 0.0	2.5	0.9 - 6.4	0.1	0.0 - 0.2	0.5	0.2 - 1.4	0.0	0.0 - 0.0	4.8	1.9 - 15.0	0.95

8-digit HUC	ID	Name	Area	Catchment Yield		Point sources		Developed Land		Manure		Agricultural Land		Phosphate Mines		Soil parent rock		Frac
				kg/km^2	90% CI	kg/km^2	90% CI	kg/km^2	90% CI	kg/km^2	90% CI	kg/km^2	90% CI	kg/km^2	90% CI	kg/km^2	90% CI	
3150204	9138	LITTLE REEDY CR	24.3	21.7	7 - 44	0.0	0.0 - 0.0	7.7	2.6 - 15.2	1.0	0.3 - 2.5	5.6	1.6 - 13.4	0.0	0.0 - 0.0	7.3	2.3 - 17.0	0.98
3150204	9139	SIZEMORE CR	40.2	10.3	4 - 25	0.0	0.0 - 0.0	4.6	1.5 - 10.7	0.5	0.1 - 1.2	2.5	0.8 - 7.2	0.0	0.0 - 0.0	2.8	1.0 - 6.3	0.96
3150204	9140	LITTLE REEDY CR	35.1	12.5	5 - 24	0.0	0.0 - 0.0	4.0	1.5 - 8.4	0.7	0.2 - 1.8	3.6	1.1 - 8.7	0.0	0.0 - 0.0	4.1	1.5 - 9.5	0.96
3160101	9141	TOMBIGBEE R	71.8	80.9	26 - 175	0.0	0.0 - 0.0	17.5	5.2 - 34.5	3.6	1.0 - 11.1	36.6	11.6 - 98.9	0.0	0.0 - 0.0	23.2	7.6 - 57.7	0.86
3160101	9142	TOMBIGBEE R	89.0	61.2	21 - 116	0.1	0.0 - 0.1	19.6	7.1 - 36.4	1.6	0.4 - 5.2	14.7	4.4 - 32.9	0.0	0.0 - 0.0	25.3	8.6 - 58.2	0.86
3160101	9143	TOMBIGBEE R	43.8	91.3	32 - 170	0.0	0.0 - 0.0	41.7	13.4 - 88.1	2.4	0.7 - 5.6	23.0	7.8 - 58.5	0.0	0.0 - 0.0	24.2	8.9 - 49.3	0.85
3160101	9144	TOMBIGBEE R	203.7	80.0	29 - 158	0.3	0.1 - 0.6	5.8	2.1 - 10.5	5.4	1.8 - 14.0	42.0	15.1 - 108.8	0.0	0.0 - 0.0	26.6	9.7 - 63.3	0.85
3160101	9145	TOMBIGBEE R	60.6	221.2	74 - 531	116.3	40.9 - 266.5	12.9	4.0 - 30.3	6.9	2.1 - 25.4	53.0	17.9 - 138.3	0.0	0.0 - 0.0	32.2	9.7 - 99.0	0.84
3160101	9146	TOMBIGBEE R	191.5	64.5	21 - 119	0.0	0.0 - 0.0	5.5	1.9 - 9.9	3.7	1.2 - 7.5	28.7	8.9 - 64.6	0.0	0.0 - 0.0	26.6	8.4 - 58.0	0.84
3160101	9147	WEAVER CR	147.0	48.9	14 - 94	0.0	0.0 - 0.0	5.8	1.8 - 11.1	3.0	0.8 - 7.0	23.3	7.2 - 55.3	0.0	0.0 - 0.0	16.7	5.1 - 40.3	0.83
3160101	9148	TOMBIGBEE R	64.2	137.0	53 - 271	24.9	10.0 - 53.1	28.4	9.7 - 53.0	5.9	2.0 - 18.3	43.6	14.6 - 107.7	0.0	0.0 - 0.0	34.2	11.3 - 85.8	0.83
3160101	9149	TOMBIGBEE R	109.2	86.4	28 - 182	0.0	0.0 - 0.0	7.0	2.1 - 15.0	6.0	1.9 - 15.6	43.3	13.8 - 107.7	0.0	0.0 - 0.0	30.1	9.7 - 75.6	0.82
3160101	9150	TOMBIGBEE R	16.1	42.8	16 - 99	0.0	0.0 - 0.0	2.7	1.0 - 5.9	2.1	0.7 - 6.5	11.8	4.2 - 29.2	0.0	0.0 - 0.0	26.2	10.3 - 76.0	0.82
3160101	9151	BULL MOUNTAIN	114.1	65.0	23 - 135	0.4	0.1 - 0.9	7.5	2.6 - 16.7	5.6	1.9 - 19.1	33.9	11.9 - 78.7	0.0	0.0 - 0.0	17.6	6.6 - 47.7	0.81
3160101	9152	JIMS CR	63.3	14.5	5 - 26	0.0	0.0 - 0.0	6.4	2.1 - 11.7	0.6	0.2 - 1.5	1.7	0.6 - 3.8	0.0	0.0 - 0.0	5.9	2.0 - 11.2	0.80
3160101	9153	BULL MOUNTAIN	80.0	26.2	12 - 51	0.0	0.0 - 0.0	7.8	3.4 - 17.0	3.2	1.3 - 10.9	8.5	3.3 - 20.4	0.0	0.0 - 0.0	6.6	3.1 - 17.0	0.80
3160101	9154	BULL MOUNTAIN	103.9	24.5	10 - 67	0.0	0.0 - 0.0	9.9	3.8 - 25.5	2.4	0.9 - 6.8	6.5	2.3 - 19.2	0.0	0.0 - 0.0	5.7	2.5 - 16.7	0.78
3160101	9155	BULL MOUNTAIN	234.9	18.5	7 - 33	0.0	0.0 - 0.0	4.3	1.6 - 8.3	3.8	1.2 - 9.7	5.0	1.5 - 12.3	0.0	0.0 - 0.0	5.4	1.8 - 12.5	0.75
3160101	9156	*A	70.2	26.8	7 - 64	0.0	0.0 - 0.0	6.5	1.9 - 14.7	6.4	1.7 - 20.1	8.8	2.6 - 28.1	0.0	0.0 - 0.0	5.1	1.4 - 14.4	0.75
3160101	9157	GUM CR	245.8	29.9	9 - 58	0.0	0.0 - 0.0	7.7	2.5 - 15.7	4.7	1.5 - 12.8	10.1	3.0 - 29.6	0.0	0.0 - 0.0	7.3	2.3 - 18.7	0.78
3160101	9158	TOMBIGBEE R	174.4	48.1	14 - 87	8.1	2.4 - 16.3	13.6	4.2 - 28.2	4.3	1.2 - 11.5	11.5	3.6 - 29.3	0.0	0.0 - 0.0	10.6	3.1 - 24.1	0.81
3160101	9159	TOMBIGBEE R	20.2	77.8	25 - 164	3.0	1.0 - 6.8	9.8	2.8 - 20.8	10.2	2.9 - 31.4	29.2	8.3 - 80.1	0.0	0.0 - 0.0	25.6	9.1 - 66.2	0.80
3160101	9160	CUMMINGS CR	81.4	23.8	9 - 58	0.0	0.0 - 0.0	10.0	3.8 - 21.7	1.7	0.6 - 4.7	4.6	1.5 - 13.1	0.0	0.0 - 0.0	7.6	2.9 - 19.6	0.79
3160101	9161	TOMBIGBEE R	11.8	60.0	20 - 116	0.0	0.0 - 0.0	1.0	0.3 - 2.3	5.8	1.7 - 16.4	15.8	5.0 - 41.6	0.0	0.0 - 0.0	37.4	12.3 - 96.2	0.78
3160101	9162	TOMBIGBEE R	10.7	79.2	30 - 166	0.0	0.0 - 0.0	2.0	0.8 - 4.2	9.8	3.0 - 26.4	26.6	9.4 - 69.4	0.0	0.0 - 0.0	40.8	14.8 - 98.1	0.77
3160101	9163	RED BUD CR	155.4	21.1	8 - 42	0.0	0.0 - 0.0	5.7	2.1 - 12.2	1.1	0.3 - 3.1	4.7	1.6 - 11.1	0.0	0.0 - 0.0	9.7	3.2 - 23.4	0.77
3160101	9164	RED BUD CR	63.2	25.6	10 - 52	0.0	0.0 - 0.0	6.7	2.4 - 15.0	1.3	0.4 - 4.0	8.5	3.3 - 21.6	0.0	0.0 - 0.0	9.1	3.8 - 21.6	0.74
3160101	9165	MACKEYS CR	244.2	23.5	8 - 41	0.1	0.0 - 0.1	8.8	2.7 - 17.3	0.7	0.2 - 1.6	4.9	1.8 - 12.9	0.0	0.0 - 0.0	9.0	2.9 - 16.4	0.74
3160101	9166	TOMBIGBEE R	1.7	193.8	73 - 474	0.0	0.0 - 0.0	10.1	3.4 - 23.3	40.0	12.1 - 103.3	105.3	37.5 - 308.6	0.0	0.0 - 0.0	38.3	13.4 - 90.0	0.77
3160101	9167	TOMBIGBEE R	5.3	91.7	26 - 159	0.0	0.0 - 0.0	5.4	1.7 - 10.3	7.9	2.4 - 19.6	35.6	10.8 - 81.1	0.0	0.0 - 0.0	42.7	13.5 - 95.8	0.77
3160101	9168	LITTLE BROWN CI	68.3	27.6	10 - 55	0.0	0.0 - 0.0	5.3	1.8 - 10.8	0.8	0.2 - 2.4	9.2	3.1 - 18.2	0.0	0.0 - 0.0	12.2	4.0 - 28.9	0.76
3160101	9169	TOMBIGBEE R	67.6	31.6	9 - 76	0.0	0.0 - 0.0	7.4	2.2 - 21.2	1.2	0.3 - 3.1	12.5	3.2 - 32.3	0.0	0.0 - 0.0	10.4	3.2 - 27.4	0.76
3160101	9170	BIG BROWN CR	28.2	61.6	18 - 165	0.1	0.0 - 0.1	7.9	2.7 - 17.2	3.5	1.1 - 10.2	32.8	9.0 - 107.5	0.0	0.0 - 0.0	17.3	5.2 - 43.7	0.77
3160101	9171	BIG BROWN CR	12.0	73.9	26 - 168	0.0	0.0 - 0.0	6.3	2.1 - 13.9	3.9	1.1 - 10.0	42.1	14.1 - 102.4	0.0	0.0 - 0.0	21.6	7.2 - 50.4	0.68
3160101	9172	BIG BROWN CR	142.2	41.3	18 - 87	0.0	0.0 - 0.0	7.2	2.9 - 14.2	0.9	0.4 - 2.4	10.2	4.1 - 26.6	0.0	0.0 - 0.0	23.1	9.7 - 50.5	0.64
3160101	9173	YOUNGS CR	20.3	19.6	6 - 54	0.0	0.0 - 0.0	4.5	1.6 - 14.5	0.4	0.1 - 1.2	4.7	1.3 - 14.2	0.0	0.0 - 0.0	9.9	3.0 - 31.5	0.64
3160101	9174	HURRICANE CR	54.4	20.5	6 - 56	0.0	0.0 - 0.0	3.7	0.9 - 11.0	0.7	0.2 - 3.1	7.9	2.1 - 23.2	0.0	0.0 - 0.0	8.2	2.1 - 27.6	0.68
3160101	9175	DONIVAN CR	35.9	63.5	21 - 143	0.0	0.0 - 0.0	4.6	1.5 - 10.8	10.1	2.9 - 30.6	33.2	11.2 - 80.5	0.0	0.0 - 0.0	15.5	4.8 - 39.8	0.77
3160101	9176	CASEY CR	33.6	17.6	5 - 54	0.0	0.0 - 0.0	2.7	0.7 - 10.3	0.6	0.2 - 2.4	6.1	1.5 - 19.7	0.0	0.0 - 0.0	8.2	2.6 - 24.1	0.21

8-digit HUC	ID	Name	Area	Catchment Yield		Point sources		Developed Land		Manure		Agricultural Land		Phosphate Mines		Soil parent rock		Frac
				kg/km^2	90% CI	kg/km^2	90% CI	kg/km^2	90% CI	kg/km^2	90% CI	kg/km^2	90% CI	kg/km^2	90% CI	kg/km^2	90% CI	
3160101	9177	DONIVAN CR	36.9	17.5	5 - 66	0.0	0.0 - 0.0	3.0	0.9 - 11.8	0.5	0.1 - 1.8	4.9	1.4 - 18.0	0.0	0.0 - 0.0	9.1	2.8 - 34.5	0.21
3160101	9178	TWENTYMILE CR	131.1	129.3	46 - 300	0.0	0.0 - 0.0	6.5	2.2 - 14.2	16.6	5.3 - 39.9	62.9	21.2 - 155.9	0.0	0.0 - 0.0	43.2	14.5 - 102.9	0.78
3160101	9179	TWENTYMILE CR	241.7	146.7	46 - 303	0.1	0.0 - 0.3	9.0	2.7 - 16.7	6.3	1.9 - 15.6	65.9	21.2 - 153.7	0.0	0.0 - 0.0	65.3	21.6 - 151.1	0.72
3160101	9180	CAMPBELLTOWN	3.7	59.4	24 - 128	0.0	0.0 - 0.0	2.4	0.8 - 5.1	3.0	0.9 - 7.6	25.0	9.1 - 54.7	0.0	0.0 - 0.0	29.1	11.0 - 72.4	0.72
3160101	9181	OKEELALA CR	33.6	68.6	23 - 214	0.0	0.0 - 0.0	4.5	1.3 - 12.9	3.0	0.9 - 11.2	29.8	9.8 - 105.5	0.0	0.0 - 0.0	31.3	10.1 - 97.7	0.59
3160101	9182	CAMPBELLTOWN	50.7	64.3	20 - 183	0.0	0.0 - 0.0	4.1	1.1 - 12.9	3.4	0.9 - 12.4	29.3	7.6 - 86.9	0.0	0.0 - 0.0	27.4	8.2 - 89.0	0.59
3160101	9183	MANTACHIE CR	183.5	77.0	25 - 155	0.1	0.0 - 0.2	8.0	2.6 - 16.6	11.0	2.9 - 30.4	35.2	11.5 - 93.8	0.0	0.0 - 0.0	22.7	7.5 - 59.6	0.80
3160101	9184	BOGUEFALA CR	1.7	42.6	12 - 98	0.0	0.0 - 0.0	0.0	0.0 - 0.0	1.1	0.3 - 3.8	6.3	1.9 - 14.5	0.0	0.0 - 0.0	35.3	9.8 - 94.4	0.82
3160101	9185	BOGUEFALA CR	143.6	58.2	19 - 113	0.7	0.3 - 1.5	7.6	2.7 - 14.1	6.3	2.1 - 15.9	24.4	7.6 - 58.8	0.0	0.0 - 0.0	19.1	6.7 - 43.4	0.81
3160101	9186	BOGUEGABA CR	61.0	83.6	34 - 212	0.0	0.0 - 0.0	7.8	3.0 - 19.0	9.5	2.9 - 30.2	42.6	15.2 - 128.0	0.0	0.0 - 0.0	23.7	8.2 - 68.2	0.81
3160101	9187	MATUBBY CR	315.1	183.9	54 - 407	1.6	0.5 - 3.9	6.1	1.8 - 12.4	13.3	3.6 - 39.9	91.1	26.8 - 247.3	0.0	0.0 - 0.0	71.7	19.7 - 173.3	0.84
3160101	9188	JAMES CR	116.0	187.7	55 - 395	0.0	0.0 - 0.0	9.4	2.6 - 18.9	12.9	3.3 - 36.5	98.6	32.4 - 259.7	0.0	0.0 - 0.0	66.7	21.6 - 161.6	0.84
3160101	9189	TOWN CR	36.8	77.4	26 - 193	0.0	0.0 - 0.0	3.5	1.0 - 8.7	6.5	2.2 - 22.4	38.1	12.6 - 111.3	0.0	0.0 - 0.0	29.4	10.6 - 86.7	0.85
3160101	9190	HARRY KETTLE C	62.1	114.2	42 - 329	0.3	0.1 - 0.7	3.2	1.1 - 7.4	7.6	2.2 - 24.0	55.2	18.7 - 147.3	0.0	0.0 - 0.0	48.0	17.6 - 148.9	0.82
3160101	9191	TOWN CR	12.7	340.7	99 - 907	10.9	4.0 - 23.1	4.1	1.3 - 8.5	34.4	7.8 - 122.3	189.8	56.7 - 606.0	0.0	0.0 - 0.0	101.6	29.0 - 268.8	0.82
3160101	9192	TOWN CR	35.8	257.1	80 - 688	0.0	0.0 - 0.0	2.9	0.9 - 6.1	18.9	4.4 - 64.9	144.0	43.3 - 372.5	0.0	0.0 - 0.0	91.3	23.6 - 272.0	0.79
3160101	9193	*B	38.9	270.0	97 - 648	0.0	0.0 - 0.0	2.5	0.8 - 5.1	20.1	5.4 - 52.4	144.9	53.8 - 394.1	0.0	0.0 - 0.0	102.4	31.1 - 254.8	0.79
3160101	9194	TOMBIGBEE R	56.6	65.1	22 - 173	0.0	0.0 - 0.0	4.7	1.6 - 11.1	9.5	3.0 - 31.4	25.4	8.9 - 76.3	0.0	0.0 - 0.0	25.4	9.0 - 75.4	0.79
3160102	9195	TOWN CR	149.4	120.4	38 - 267	0.0	0.0 - 0.0	5.5	1.6 - 12.3	8.8	2.8 - 29.9	56.9	19.4 - 134.8	0.0	0.0 - 0.0	49.3	15.9 - 124.9	0.82
3160102	9196	TOWN CR	29.5	240.7	77 - 533	0.0	0.0 - 0.0	11.4	3.6 - 26.8	15.0	4.0 - 55.0	122.3	35.2 - 270.5	0.0	0.0 - 0.0	91.9	28.4 - 225.9	0.80
3160102	9197	TOWN CR	110.2	135.7	49 - 274	0.0	0.0 - 0.0	7.8	2.9 - 14.9	7.5	2.4 - 21.5	62.0	20.4 - 162.4	0.0	0.0 - 0.0	58.5	20.2 - 126.7	0.80
3160102	9198	*A	84.2	64.4	20 - 117	0.8	0.2 - 1.7	13.4	4.5 - 27.5	2.6	0.7 - 7.0	21.8	7.3 - 46.5	0.0	0.0 - 0.0	25.8	8.4 - 56.7	0.78
3160102	9199	TOWN CR	13.9	1096.9	414 - 2939	908.6	343.1 - 2462.3	68.5	24.3 - 168.7	4.2	1.3 - 13.4	34.4	12.6 - 104.0	0.0	0.0 - 0.0	81.3	28.2 - 238.9	0.78
3160102	9200	EUCLAUTUBBA CI	102.1	92.7	29 - 177	8.5	2.7 - 18.9	23.2	6.9 - 52.3	2.7	0.7 - 6.6	22.0	6.2 - 61.1	0.0	0.0 - 0.0	36.3	12.0 - 77.8	0.77
3160102	9201	FLAT CR	58.7	152.1	42 - 394	0.0	0.0 - 0.0	7.8	2.3 - 19.6	9.5	2.3 - 29.0	77.9	18.9 - 211.4	0.0	0.0 - 0.0	56.8	17.7 - 162.9	0.75
3160102	9202	TISHOMINGO CR	100.3	141.5	44 - 316	0.0	0.0 - 0.0	4.9	1.5 - 11.2	8.4	2.3 - 23.0	67.7	20.5 - 171.7	0.0	0.0 - 0.0	60.6	19.5 - 158.6	0.75
3160102	9203	YONABA CR	141.0	158.4	51 - 361	0.1	0.0 - 0.2	36.3	12.4 - 89.6	5.9	1.5 - 18.9	47.4	15.1 - 129.4	0.0	0.0 - 0.0	68.8	22.6 - 175.3	0.77
3160102	9204	LAMP CR	67.2	152.8	50 - 332	0.0	0.0 - 0.0	5.5	1.8 - 12.5	9.5	2.6 - 26.5	73.2	23.9 - 170.3	0.0	0.0 - 0.0	64.7	22.5 - 170.5	0.74
3160102	9205	YONABA CR	8.6	226.3	76 - 563	0.0	0.0 - 0.0	3.0	1.1 - 7.4	15.9	4.3 - 55.7	127.7	42.4 - 377.7	0.0	0.0 - 0.0	79.6	28.3 - 204.4	0.74
3160102	9206	BRIDGE CR	40.1	121.3	46 - 250	0.0	0.0 - 0.0	6.3	2.1 - 12.7	7.2	2.4 - 19.1	50.1	17.3 - 128.6	0.0	0.0 - 0.0	57.8	23.1 - 133.8	0.71
3160102	9207	YONABA CR	85.1	71.4	21 - 182	0.3	0.1 - 0.6	5.2	1.4 - 11.7	3.6	1.0 - 10.4	24.9	7.1 - 65.6	0.0	0.0 - 0.0	37.5	10.7 - 92.7	0.71
3160102	9208	COONEWAH CR	63.8	244.2	96 - 516	0.7	0.3 - 1.7	30.0	11.4 - 65.6	12.8	4.3 - 36.7	105.7	38.7 - 260.0	0.0	0.0 - 0.0	95.0	37.9 - 248.8	0.80
3160102	9209	LITTLE COONEWA	32.5	195.6	67 - 477	0.0	0.0 - 0.0	38.2	13.1 - 87.4	7.7	2.2 - 23.6	64.0	21.5 - 179.9	0.0	0.0 - 0.0	85.8	27.4 - 247.2	0.69
3160102	9210	COONEWAH CR	74.2	130.1	45 - 269	0.0	0.0 - 0.0	6.4	2.1 - 15.2	6.4	2.0 - 19.0	60.4	19.1 - 152.9	0.0	0.0 - 0.0	57.0	20.3 - 139.3	0.69
3160102	9211	CHIWAPA CR	2.1	408.4	127 - 817	0.0	0.0 - 0.0	0.0	0.0 - 0.0	29.4	7.3 - 89.4	227.2	69.2 - 551.8	0.0	0.0 - 0.0	151.9	44.6 - 350.5	0.80
3160102	9212	CHIWAPA CR	132.6	206.5	75 - 396	1.6	0.6 - 3.1	6.4	2.1 - 12.1	12.2	3.4 - 30.7	107.7	37.4 - 224.1	0.0	0.0 - 0.0	78.6	26.9 - 169.4	0.80
3160102	9213	MUBBY CR	7.4	212.3	72 - 437	0.0	0.0 - 0.0	4.7	1.5 - 10.5	11.1	3.2 - 29.7	121.8	43.2 - 309.0	0.0	0.0 - 0.0	74.7	23.8 - 178.4	0.72
3160102	9214	MUBBY CR	48.3	67.7	23 - 144	0.0	0.0 - 0.1	5.0	1.5 - 10.5	2.7	0.8 - 7.7	29.4	10.5 - 78.2	0.0	0.0 - 0.0	30.6	12.0 - 79.4	0.69
3160102	9215	MILLER CR	42.7	63.7	20 - 126	0.0	0.0 - 0.0	5.9	1.7 - 15.1	2.2	0.6 - 5.2	24.3	7.0 - 62.8	0.0	0.0 - 0.0	31.1	9.3 - 73.6	0.69

8-digit HUC	ID	Name	Area	Catchment Yield		Point sources		Developed Land		Manure		Agricultural Land		Phosphate Mines		Soil parent rock		Frac
				kg/km^2	90% CI	kg/km^2	90% CI	kg/km^2	90% CI	kg/km^2	90% CI	kg/km^2	90% CI	kg/km^2	90% CI	kg/km^2	90% CI	
3160102	9216	CHIWAPA CR	41.9	93.3	32 - 196	0.0	0.0 - 0.0	2.6	0.8 - 5.4	3.5	0.9 - 13.2	37.5	12.2 - 93.5	0.0	0.0 - 0.0	49.8	16.3 - 118.9	0.72
3160102	9217	CHIWAPA CR	10.4	61.0	24 - 156	0.0	0.0 - 0.0	8.6	2.8 - 21.4	2.0	0.6 - 6.2	21.3	7.5 - 65.3	0.0	0.0 - 0.0	29.1	11.8 - 73.2	0.65
3160102	9218	*B	106.2	75.5	23 - 146	4.2	1.4 - 8.4	8.3	2.3 - 19.2	2.5	0.8 - 6.0	26.7	9.3 - 64.7	0.0	0.0 - 0.0	33.8	10.9 - 68.8	0.65
3160102	9220	TUBBALUBBA CR	45.7	236.9	75 - 587	0.0	0.0 - 0.0	6.1	2.0 - 12.3	14.4	4.1 - 49.6	118.5	40.4 - 316.0	0.0	0.0 - 0.0	97.8	29.5 - 237.1	0.80
3160102	9221	TALLABINNELA C	147.3	196.9	54 - 397	0.0	0.0 - 0.0	6.2	1.9 - 13.2	15.8	4.1 - 40.1	93.6	25.6 - 223.8	0.0	0.0 - 0.0	81.3	23.7 - 191.4	0.80
3160103	9222	BUTTAHATCHEE I	167.0	73.6	28 - 144	0.8	0.3 - 1.5	6.6	2.2 - 13.2	4.7	1.4 - 13.1	41.0	14.3 - 97.2	0.0	0.0 - 0.0	20.6	7.2 - 42.6	0.85
3160103	9223	BUTTAHATCHEE I	93.2	24.0	7 - 41	0.0	0.0 - 0.0	5.5	1.6 - 10.6	1.6	0.4 - 3.3	7.4	2.1 - 15.8	0.0	0.0 - 0.0	9.6	2.8 - 20.0	0.81
3160103	9224	BOGUE CR	54.8	31.1	9 - 56	3.8	1.2 - 8.3	11.7	3.3 - 24.5	1.9	0.6 - 5.5	7.8	2.3 - 16.5	0.0	0.0 - 0.0	5.8	2.0 - 14.2	0.80
3160103	9225	BUTTAHATCHEE I	37.5	20.9	6 - 42	0.0	0.0 - 0.0	9.4	3.2 - 19.0	1.3	0.4 - 3.1	5.3	1.3 - 12.8	0.0	0.0 - 0.0	4.9	1.5 - 11.8	0.80
3160103	9226	BEAVER CR	232.7	24.1	9 - 41	0.9	0.3 - 1.7	10.5	3.7 - 19.3	2.5	0.8 - 5.7	4.5	1.7 - 11.5	0.0	0.0 - 0.0	5.6	2.0 - 12.4	0.80
3160103	9227	BUTTAHATCHEE I	227.9	26.1	8 - 55	3.9	1.2 - 9.6	6.7	2.1 - 15.8	2.6	0.7 - 8.3	6.0	1.8 - 16.2	0.0	0.0 - 0.0	6.8	2.2 - 16.8	0.80
3160103	9228	WOODS CR	74.9	23.0	8 - 57	0.0	0.0 - 0.0	5.9	1.8 - 15.6	4.6	1.5 - 13.0	6.3	2.0 - 16.0	0.0	0.0 - 0.0	6.1	1.7 - 15.8	0.77
3160103	9229	BUTTAHATCHEE I	39.8	51.1	14 - 123	0.0	0.0 - 0.0	33.2	8.6 - 78.6	4.2	1.1 - 12.6	6.3	1.9 - 17.9	0.0	0.0 - 0.0	7.4	2.2 - 18.7	0.77
3160103	9230	BUTTAHATCHEE I	15.1	43.0	15 - 89	0.0	0.0 - 0.0	21.3	6.6 - 50.4	5.7	1.7 - 15.8	8.7	3.0 - 22.0	0.0	0.0 - 0.0	7.3	2.4 - 17.0	0.76
3160103	9231	BUTTAHATCHEE I	19.6	54.3	18 - 131	0.0	0.0 - 0.0	15.6	5.1 - 37.1	12.9	3.8 - 35.8	18.3	5.2 - 45.5	0.0	0.0 - 0.0	7.5	2.5 - 17.7	0.75
3160103	9232	BUTTAHATCHEE I	55.9	21.7	6 - 48	0.0	0.0 - 0.0	7.4	2.0 - 16.1	3.2	0.8 - 8.8	4.4	1.2 - 11.6	0.0	0.0 - 0.0	6.8	2.0 - 16.6	0.74
3160103	9233	BUTTAHATCHEE I	29.7	29.3	10 - 69	0.0	0.0 - 0.0	5.3	1.9 - 12.0	8.0	2.4 - 24.4	9.9	3.2 - 27.5	0.0	0.0 - 0.0	6.1	1.8 - 16.0	0.72
3160103	9234	BUTTAHATCHEE I	88.3	29.0	10 - 57	0.0	0.0 - 0.0	7.8	2.7 - 16.0	7.3	2.3 - 22.0	8.4	2.5 - 24.4	0.0	0.0 - 0.0	5.6	1.9 - 14.6	0.70
3160103	9235	*A	101.2	29.0	10 - 65	0.0	0.0 - 0.0	10.2	3.0 - 23.6	6.0	1.8 - 18.3	7.9	2.3 - 21.3	0.0	0.0 - 0.0	4.9	1.5 - 12.2	0.70
3160103	9236	BARN CR	53.1	19.2	7 - 41	0.0	0.0 - 0.0	5.9	2.2 - 12.5	3.5	1.2 - 9.6	4.3	1.5 - 10.5	0.0	0.0 - 0.0	5.4	2.0 - 14.5	0.72
3160103	9237	CAMP CR	47.6	21.5	7 - 41	0.0	0.0 - 0.0	6.8	2.4 - 14.6	3.8	1.2 - 10.3	5.5	1.9 - 14.6	0.0	0.0 - 0.0	5.5	1.9 - 13.8	0.74
3160103	9238	CLIFTY CR	120.7	32.3	9 - 66	0.0	0.0 - 0.0	8.4	2.2 - 18.0	7.7	2.1 - 21.8	10.8	3.3 - 26.4	0.0	0.0 - 0.0	5.4	1.6 - 13.1	0.75
3160103	9239	WILLIAMS CR	76.9	21.7	8 - 45	0.0	0.0 - 0.0	7.0	2.2 - 15.2	3.8	1.0 - 10.0	5.3	1.7 - 14.7	0.0	0.0 - 0.0	5.6	2.0 - 13.0	0.76
3160103	9240	SIPSEY CR	57.6	23.6	8 - 48	0.0	0.0 - 0.0	4.6	1.6 - 10.6	1.2	0.4 - 4.4	9.6	3.1 - 24.8	0.0	0.0 - 0.0	8.1	2.6 - 20.9	0.81
3160103	9241	SIPSEY CR	242.3	24.4	8 - 50	0.0	0.0 - 0.0	6.1	1.9 - 14.0	3.7	1.1 - 9.3	8.6	2.7 - 24.6	0.0	0.0 - 0.0	6.1	2.0 - 13.5	0.79
3160103	9242	SIPSEY CR	80.9	36.8	12 - 86	0.0	0.0 - 0.0	13.8	4.0 - 31.6	7.2	2.4 - 15.7	10.1	3.6 - 22.9	0.0	0.0 - 0.0	5.7	2.1 - 13.3	0.73
3160103	9243	HURRICANE CR	95.1	32.8	9 - 67	0.0	0.0 - 0.0	9.6	3.1 - 21.5	7.1	2.1 - 20.8	10.0	3.1 - 28.3	0.0	0.0 - 0.0	6.1	2.0 - 17.5	0.73
3160103	9244	SPLUNGE CR	104.0	15.2	5 - 38	0.0	0.0 - 0.0	5.3	1.4 - 13.6	0.7	0.2 - 1.8	3.0	0.9 - 10.3	0.0	0.0 - 0.0	6.3	1.9 - 15.9	0.79
3160104	9245	TIBBEE CR	62.1	161.1	50 - 328	0.0	0.0 - 0.0	5.9	1.6 - 12.3	13.8	3.7 - 44.1	80.3	26.0 - 182.0	0.0	0.0 - 0.0	61.1	19.6 - 141.5	0.86
3160104	9246	TIBBEE CR	13.6	174.0	67 - 379	0.0	0.0 - 0.0	3.7	1.4 - 8.9	7.2	2.2 - 27.7	40.4	16.1 - 132.7	0.0	0.0 - 0.0	122.7	46.3 - 327.7	0.85
3160104	9247	*A	45.3	254.4	76 - 624	0.0	0.0 - 0.0	7.2	2.3 - 15.2	22.0	4.7 - 70.4	126.9	34.2 - 392.1	0.0	0.0 - 0.0	98.4	27.7 - 257.9	0.84
3160104	9248	TIBBEE CR	107.8	1068.9	384 - 2164	853.5	300.2 - 1829.9	20.0	6.5 - 40.7	15.1	4.6 - 42.5	77.3	26.1 - 208.9	0.0	0.0 - 0.0	103.0	37.6 - 224.0	0.84
3160104	9249	CHUQUATONCHEI	180.1	244.6	72 - 635	0.0	0.0 - 0.0	5.6	1.6 - 11.5	19.4	4.9 - 68.5	116.8	35.8 - 376.4	0.0	0.0 - 0.0	102.7	30.0 - 286.0	0.83
3160104	9250	CHUQUATONCHEI	546.7	164.7	50 - 356	0.0	0.0 - 0.0	4.7	1.6 - 9.5	14.8	4.0 - 44.7	78.2	23.7 - 217.4	0.0	0.0 - 0.0	67.1	19.4 - 154.4	0.81
3160104	9251	HOULKA CR	108.1	171.0	56 - 318	0.0	0.0 - 0.0	4.9	1.6 - 9.6	15.4	3.9 - 36.9	82.7	27.1 - 191.8	0.0	0.0 - 0.0	68.0	21.8 - 142.1	0.81
3160104	9252	LONG CR	74.9	117.8	33 - 225	0.9	0.2 - 1.9	4.7	1.4 - 9.2	10.4	2.5 - 26.9	49.6	17.0 - 128.6	0.0	0.0 - 0.0	52.2	15.1 - 122.8	0.79
3160104	9253	HOULKA CR	55.6	109.7	34 - 250	0.0	0.0 - 0.0	5.6	1.7 - 12.0	9.0	2.4 - 26.8	45.4	14.7 - 124.2	0.0	0.0 - 0.0	49.7	16.2 - 141.2	0.79
3160104	9254	HOULKA CR	208.6	200.8	51 - 426	5.1	1.4 - 13.3	6.5	1.8 - 15.7	17.9	4.0 - 45.4	85.4	24.8 - 206.9	0.0	0.0 - 0.0	85.8	21.5 - 235.9	0.76
3160104	9256	CHICO CR	54.6	496.0	163 - 1119	21.6	8.4 - 47.9	21.6	7.8 - 47.9	40.7	11.4 - 112.0	190.0	58.2 - 434.8	0.0	0.0 - 0.0	222.2	68.9 - 625.0	0.76

8-digit HUC	ID	Name	Area	Catchment Yield		Point sources		Developed Land		Manure		Agricultural Land		Phosphate Mines		Soil parent rock		Frac
				kg/km^2	90% CI	kg/km^2	90% CI	kg/km^2	90% CI	kg/km^2	90% CI	kg/km^2	90% CI	kg/km^2	90% CI	kg/km^2	90% CI	
3160104	9257	CANE CR	21.8	295.5	100 - 754	0.0	0.0 - 0.0	5.1	1.9 - 11.3	22.5	5.8 - 69.1	108.0	35.5 - 293.6	0.0	0.0 - 0.0	159.8	51.1 - 430.4	0.76
3160104	9258	CHEMEN CR	53.7	434.5	140 - 1149	0.0	0.0 - 0.0	6.7	2.4 - 13.8	39.8	10.0 - 129.8	184.4	54.4 - 548.9	0.0	0.0 - 0.0	203.7	65.5 - 649.3	0.70
3160104	9259	LINE CR	78.2	210.2	62 - 461	0.0	0.0 - 0.0	5.0	1.6 - 10.1	19.4	4.8 - 51.3	104.7	31.2 - 264.1	0.0	0.0 - 0.0	81.2	23.6 - 200.2	0.83
3160104	9260	LINE CR	119.2	112.7	31 - 270	0.0	0.0 - 0.0	4.8	1.6 - 11.6	7.6	2.0 - 19.9	43.4	15.5 - 108.6	0.0	0.0 - 0.0	56.9	15.4 - 144.6	0.82
3160104	9262	LINE CR	46.4	92.3	32 - 201	0.0	0.0 - 0.0	5.4	1.9 - 11.6	4.2	1.0 - 13.0	24.9	8.9 - 56.3	0.0	0.0 - 0.0	57.7	19.9 - 141.8	0.79
3160104	9263	STANDING REED (	1.7	361.0	93 - 878	0.0	0.0 - 0.0	8.1	2.4 - 17.3	33.3	8.8 - 91.1	192.4	51.0 - 505.9	0.0	0.0 - 0.0	127.3	32.1 - 342.0	0.77
3160104	9264	STANDING REED (	44.6	109.8	33 - 257	0.1	0.0 - 0.2	4.9	1.5 - 10.9	7.2	2.1 - 18.4	37.9	10.3 - 96.7	0.0	0.0 - 0.0	59.7	17.9 - 150.3	0.77
3160104	9265	LITTLE CANE CR	62.8	222.8	75 - 438	0.0	0.0 - 0.0	5.7	2.0 - 10.8	17.4	4.9 - 43.5	88.5	31.2 - 196.5	0.0	0.0 - 0.0	111.2	36.7 - 264.0	0.77
3160104	9266	LINE CR	49.8	197.3	65 - 465	0.0	0.0 - 0.0	6.3	2.0 - 13.5	13.9	4.0 - 50.2	70.7	24.4 - 218.5	0.0	0.0 - 0.0	106.4	40.2 - 272.7	0.77
3160104	9267	*B	84.2	157.7	54 - 373	0.0	0.0 - 0.0	5.0	1.4 - 12.3	12.4	3.9 - 54.9	61.3	21.0 - 182.5	0.0	0.0 - 0.0	78.9	25.4 - 208.0	0.79
3160104	9268	JOHNSON CR	54.6	146.8	53 - 339	2.2	0.7 - 4.6	4.0	1.5 - 8.1	8.1	2.4 - 20.5	43.2	14.3 - 102.5	0.0	0.0 - 0.0	89.3	30.5 - 207.8	0.79
3160104	9269	TRIM CANE CR	12.9	151.8	45 - 293	0.0	0.0 - 0.0	2.3	0.7 - 4.3	9.8	2.6 - 28.8	48.0	14.6 - 111.0	0.0	0.0 - 0.0	91.7	27.5 - 204.8	0.82
3160104	9270	SUN CR	160.5	143.5	49 - 299	0.0	0.0 - 0.0	4.7	1.7 - 10.4	8.7	2.5 - 23.7	46.1	13.9 - 114.1	0.0	0.0 - 0.0	84.1	27.7 - 206.9	0.81
3160104	9271	TRIM CANE CR	236.3	186.4	50 - 558	0.0	0.0 - 0.0	10.9	3.0 - 26.1	12.4	2.7 - 43.9	57.3	13.7 - 148.9	0.0	0.0 - 0.0	105.8	28.3 - 327.0	0.81
3160104	9272	SELF CR	18.5	151.5	51 - 393	0.0	0.0 - 0.0	5.0	1.6 - 10.3	10.5	3.1 - 29.7	45.2	14.2 - 124.6	0.0	0.0 - 0.0	90.8	28.3 - 251.4	0.71
3160104	9273	TRIM CANE CR	35.6	77.0	23 - 185	0.0	0.0 - 0.0	4.4	1.2 - 10.0	3.5	1.0 - 9.7	16.1	4.6 - 46.0	0.0	0.0 - 0.0	53.0	15.6 - 132.7	0.71
3160104	9274	CATALPA CR	88.1	213.1	72 - 546	0.1	0.0 - 0.2	8.0	2.7 - 15.4	13.4	3.6 - 40.4	96.0	30.7 - 258.0	0.0	0.0 - 0.0	95.7	27.4 - 256.4	0.85
3160104	9275	SAND CR	100.8	167.1	52 - 370	0.0	0.0 - 0.0	16.2	4.8 - 36.8	12.1	3.0 - 35.2	56.7	18.1 - 130.5	0.0	0.0 - 0.0	82.0	24.9 - 201.4	0.79
3160104	9276	CATALPA CR	150.8	248.4	101 - 522	47.9	19.0 - 104.0	15.9	5.6 - 31.0	15.1	5.8 - 42.6	85.2	33.5 - 212.5	0.0	0.0 - 0.0	84.3	30.6 - 204.9	0.79
3160104	9277	CANE CR	33.3	383.2	131 - 908	0.0	0.0 - 0.0	7.7	2.4 - 13.8	33.5	9.9 - 81.2	157.7	49.5 - 381.8	0.0	0.0 - 0.0	184.3	57.0 - 535.8	0.70
3160105	9278	LUXAPALLILA CR	86.5	262.4	91 - 601	161.0	53.8 - 392.4	36.5	12.8 - 82.4	6.8	2.0 - 18.9	33.8	12.5 - 79.9	0.0	0.0 - 0.0	24.4	8.7 - 57.8	0.86
3160105	9279	MAGBY CR	126.9	36.0	13 - 95	0.0	0.0 - 0.1	14.0	4.9 - 38.7	4.8	1.4 - 15.1	8.6	2.7 - 22.5	0.0	0.0 - 0.0	8.5	3.0 - 23.1	0.86
3160105	9280	LUXAPALLILA CR	2.6	174.5	67 - 485	6.8	2.5 - 19.7	132.3	52.7 - 367.3	0.5	0.2 - 1.8	5.3	1.8 - 15.2	0.0	0.0 - 0.0	29.6	10.9 - 92.3	0.86
3160105	9281	LUXAPALLILA CR	805.5	27.2	12 - 52	0.6	0.2 - 1.3	8.4	3.6 - 21.2	3.5	1.4 - 9.7	8.8	3.8 - 19.1	0.0	0.0 - 0.0	5.9	2.6 - 13.6	0.85
3160105	9282	YELLOW CR	68.9	65.5	24 - 173	0.0	0.0 - 0.0	8.4	3.1 - 22.1	4.4	1.5 - 14.3	41.9	13.7 - 134.1	0.0	0.0 - 0.0	10.8	4.6 - 30.8	0.85
3160105	9283	MUD CR	129.8	19.2	7 - 36	0.0	0.0 - 0.0	6.0	2.2 - 12.9	1.6	0.5 - 4.0	6.5	2.2 - 14.6	0.0	0.0 - 0.0	5.1	1.5 - 12.4	0.84
3160105	9284	YELLOW CR	26.3	30.6	10 - 61	0.0	0.0 - 0.0	5.7	1.7 - 13.0	2.9	0.9 - 9.9	14.4	4.3 - 41.9	0.0	0.0 - 0.0	7.7	2.6 - 22.2	0.84
3160105	9285	YELLOW CR	103.0	25.3	9 - 59	0.0	0.0 - 0.0	8.0	2.8 - 19.8	2.1	0.7 - 7.2	8.8	2.9 - 24.7	0.0	0.0 - 0.0	6.4	2.2 - 16.7	0.83
3160105	9286	HELL'S CR	205.6	15.8	6 - 38	0.0	0.0 - 0.0	6.7	2.3 - 14.7	0.9	0.3 - 3.1	3.5	1.2 - 9.0	0.0	0.0 - 0.0	4.6	1.6 - 12.8	0.80
3160105	9287	YELLOW CR	203.3	15.6	6 - 32	0.0	0.0 - 0.0	8.0	3.0 - 17.2	0.6	0.2 - 1.7	2.6	0.8 - 7.7	0.0	0.0 - 0.0	4.3	1.8 - 9.6	0.80
3160105	9288	WILSON CR	7.3	40.6	12 - 101	0.0	0.0 - 0.0	8.7	2.8 - 21.9	4.1	1.2 - 12.0	19.5	5.8 - 57.4	0.0	0.0 - 0.0	8.4	2.2 - 22.9	0.83
3160105	9289	WILSON CR	106.1	33.0	10 - 62	0.0	0.0 - 0.0	7.6	2.3 - 16.1	3.6	1.4 - 9.6	15.2	4.2 - 41.6	0.0	0.0 - 0.0	6.6	2.3 - 15.8	0.82
3160105	9290	CUT BANK CR	75.6	24.1	9 - 50	0.0	0.0 - 0.0	4.7	1.7 - 9.7	1.8	0.5 - 5.2	7.7	2.6 - 18.2	0.0	0.0 - 0.0	10.0	3.4 - 22.2	0.82
3160106	9291	TOMBIGBEE R	330.5	56.3	20 - 108	0.0	0.0 - 0.0	4.6	1.5 - 10.7	3.4	1.0 - 9.3	17.6	5.2 - 43.0	0.0	0.0 - 0.0	30.6	10.2 - 71.1	0.91
3160106	9292	TAYLOR CR	80.6	138.2	49 - 307	0.0	0.0 - 0.0	3.0	1.0 - 6.5	10.1	2.6 - 31.7	55.5	19.3 - 149.7	0.0	0.0 - 0.0	69.6	23.5 - 157.0	0.90
3160106	9293	TOMBIGBEE R	122.1	125.5	43 - 328	0.0	0.0 - 0.0	6.6	2.3 - 17.2	11.8	3.7 - 36.9	54.5	18.7 - 141.5	0.0	0.0 - 0.0	52.7	18.0 - 148.8	0.90
3160106	9294	TOMBIGBEE R	129.0	98.9	37 - 270	0.0	0.0 - 0.0	5.7	2.2 - 13.9	7.8	2.3 - 29.0	39.1	14.6 - 123.9	0.0	0.0 - 0.0	46.3	15.8 - 124.8	0.90
3160106	9295	BRUSH CR	159.3	50.9	18 - 150	0.0	0.0 - 0.0	5.0	2.0 - 11.8	3.4	1.0 - 10.1	19.0	6.4 - 56.6	0.0	0.0 - 0.0	23.4	8.6 - 62.4	0.89
3160106	9296	TOMBIGBEE R	2.5	39.3	12 - 80	0.0	0.0 - 0.0	1.6	0.5 - 2.9	1.4	0.3 - 3.9	7.5	2.2 - 17.6	0.0	0.0 - 0.0	28.8	9.2 - 69.2	0.89

8-digit HUC	ID	Name	Area	Catchment Yield		Point sources		Developed Land		Manure		Agricultural Land		Phosphate Mines		Soil parent rock		Frac
				kg/km^2	90% CI	kg/km^2	90% CI	kg/km^2	90% CI	kg/km^2	90% CI	kg/km^2	90% CI	kg/km^2	90% CI	kg/km^2	90% CI	
3160106	9297	TRUSSELLS CR	201.8	28.9	8 - 82	0.0	0.0 - 0.0	3.3	1.0 - 7.9	1.2	0.3 - 4.1	6.9	2.1 - 22.7	0.0	0.0 - 0.0	17.5	4.7 - 47.2	0.89
3160106	9298	TOMBIGBEE R	40.5	62.1	22 - 162	0.0	0.0 - 0.0	5.7	1.9 - 14.8	3.8	1.0 - 11.8	19.4	6.3 - 53.0	0.0	0.0 - 0.0	33.2	11.1 - 93.8	0.89
3160106	9299	TOMBIGBEE R	76.6	89.1	33 - 229	0.0	0.0 - 0.0	3.9	1.6 - 7.8	5.7	1.7 - 19.7	32.0	11.8 - 90.1	0.0	0.0 - 0.0	47.5	15.3 - 159.7	0.89
3160106	9300	TOMBIGBEE R	27.3	55.1	19 - 121	0.0	0.0 - 0.0	3.7	1.3 - 7.9	2.5	0.8 - 6.5	12.7	4.2 - 30.7	0.0	0.0 - 0.0	36.1	12.0 - 89.2	0.89
3160106	9301	TOMBIGBEE R	62.0	56.8	21 - 118	0.0	0.0 - 0.0	4.6	1.7 - 10.3	3.8	1.3 - 10.0	18.5	7.0 - 51.7	0.0	0.0 - 0.0	30.0	11.6 - 74.1	0.89
3160106	9302	TOMBIGBEE R	54.8	141.0	60 - 275	0.0	0.0 - 0.0	4.0	1.6 - 8.3	43.3	15.8 - 113.0	52.5	21.0 - 124.9	0.0	0.0 - 0.0	41.2	17.7 - 100.0	0.88
3160106	9303	LUBBUB CR	13.7	31.7	11 - 67	0.0	0.0 - 0.0	2.3	0.9 - 5.0	5.5	1.7 - 19.6	6.4	2.0 - 15.5	0.0	0.0 - 0.0	17.5	6.0 - 40.8	0.88
3160106	9304	LUBBUB CR	128.8	26.7	10 - 73	2.3	0.9 - 6.3	5.5	1.9 - 14.1	5.2	1.7 - 16.9	6.2	2.3 - 18.3	0.0	0.0 - 0.0	7.6	2.5 - 21.3	0.87
3160106	9305	BEAR CR	31.7	13.9	4 - 27	0.0	0.0 - 0.0	2.6	0.8 - 5.6	2.1	0.6 - 6.2	2.4	0.8 - 6.2	0.0	0.0 - 0.0	6.9	2.1 - 15.9	0.82
3160106	9306	LUBBUB CR	4.5	21.1	7 - 49	0.0	0.0 - 0.0	4.7	1.7 - 8.6	4.1	1.3 - 12.0	5.1	1.7 - 12.2	0.0	0.0 - 0.0	7.2	2.3 - 16.1	0.82
3160106	9307	LITTLE LUBBUB C	73.7	15.2	4 - 38	0.0	0.0 - 0.0	4.7	1.4 - 10.3	2.0	0.5 - 5.9	2.3	0.7 - 6.8	0.0	0.0 - 0.0	6.2	1.8 - 15.6	0.82
3160106	9308	LUBBUB CR	302.0	21.9	7 - 52	0.0	0.0 - 0.0	7.2	2.5 - 16.0	4.6	1.4 - 12.8	5.3	1.7 - 12.3	0.0	0.0 - 0.0	4.8	1.5 - 12.4	0.82
3160106	9309	BLUBBER CR	134.3	29.2	10 - 57	0.9	0.3 - 2.0	8.7	3.1 - 17.5	5.7	1.9 - 16.5	6.6	2.5 - 17.2	0.0	0.0 - 0.0	7.3	2.4 - 17.9	0.87
3160106	9310	TOMBIGBEE R	28.1	106.9	33 - 206	0.0	0.0 - 0.0	3.8	1.3 - 7.9	29.6	8.7 - 77.0	35.5	10.6 - 80.4	0.0	0.0 - 0.0	38.0	11.1 - 83.9	0.88
3160106	9311	TOMBIGBEE R	93.0	94.0	31 - 178	0.0	0.0 - 0.0	4.2	1.4 - 10.2	17.3	5.0 - 53.5	36.0	13.5 - 93.2	0.0	0.0 - 0.0	36.5	11.4 - 83.4	0.88
3160106	9312	BIG CR	138.1	25.1	9 - 59	0.0	0.0 - 0.0	5.6	2.0 - 12.7	6.1	2.0 - 17.6	7.3	2.5 - 20.5	0.0	0.0 - 0.0	6.1	2.1 - 15.7	0.87
3160106	9313	TOMBIGBEE R	73.2	96.2	36 - 193	0.0	0.0 - 0.0	4.9	1.6 - 10.5	8.9	2.9 - 22.8	38.4	13.8 - 101.8	0.0	0.0 - 0.0	44.0	16.1 - 107.2	0.87
3160106	9316	TOMBIGBEE R	38.7	40.0	18 - 91	0.0	0.0 - 0.0	5.6	2.0 - 13.7	3.5	1.1 - 9.3	13.2	5.4 - 35.2	0.0	0.0 - 0.0	17.7	7.4 - 43.5	0.87
3160106	9318	KINCAIDE CR	21.8	85.8	24 - 198	0.0	0.0 - 0.0	6.7	2.1 - 13.7	4.5	1.0 - 12.4	45.4	12.0 - 115.9	0.0	0.0 - 0.0	29.2	7.8 - 75.7	0.87
3160106	9319	KINCAIDE CR	95.3	21.8	9 - 42	0.0	0.0 - 0.0	6.2	2.4 - 14.1	3.6	1.2 - 11.3	5.2	1.8 - 14.0	0.0	0.0 - 0.0	6.8	2.7 - 17.0	0.85
3160106	9320	NASH CR	44.5	30.4	11 - 55	0.0	0.0 - 0.0	6.9	2.4 - 12.7	4.5	1.4 - 10.9	9.3	3.1 - 21.7	0.0	0.0 - 0.0	9.8	3.5 - 20.8	0.85
3160106	9321	TOMBIGBEE R	68.4	616.1	177 - 1348	499.0	148.8 - 1089.2	2.9	0.9 - 6.1	5.1	1.4 - 16.4	52.9	16.7 - 130.4	0.0	0.0 - 0.0	56.1	15.6 - 130.3	0.87
3160106	9322	TOMBIGBEE R	134.2	58.0	19 - 160	8.1	2.9 - 21.2	9.3	2.9 - 24.0	5.2	1.6 - 14.3	20.1	6.4 - 58.8	0.0	0.0 - 0.0	15.4	4.8 - 44.1	0.87
3160106	9323	MCCOWERS CR	8.4	46.5	17 - 98	0.0	0.0 - 0.0	4.0	1.3 - 9.6	1.9	0.6 - 5.6	20.5	7.5 - 57.1	0.0	0.0 - 0.0	20.0	8.4 - 49.1	0.87
3160106	9324	MAYO SLOUGH	132.4	191.7	64 - 411	2.6	0.9 - 7.1	6.6	2.1 - 18.1	9.6	2.6 - 34.2	97.5	32.4 - 275.1	0.0	0.0 - 0.0	75.4	25.6 - 203.5	0.84
3160106	9325	MCCOWERS CR	126.0	190.7	47 - 450	0.0	0.0 - 0.0	3.6	1.1 - 9.1	9.9	2.3 - 28.9	99.5	30.7 - 321.2	0.0	0.0 - 0.0	77.7	20.9 - 186.5	0.84
3160106	9326	BIG DITCH	119.7	189.6	57 - 355	0.0	0.0 - 0.0	4.0	1.2 - 7.6	10.9	2.3 - 30.5	96.0	31.0 - 216.4	0.0	0.0 - 0.0	78.7	20.7 - 197.3	0.87
3160106	9327	BROKEN PUMPKIN	86.6	203.9	60 - 505	0.0	0.0 - 0.0	2.7	0.8 - 6.5	15.6	4.3 - 42.3	108.3	28.4 - 274.0	0.0	0.0 - 0.0	77.3	20.8 - 208.1	0.87
3160106	9328	BOGUE CHITTO CI	10.6	242.1	77 - 612	0.0	0.0 - 0.0	3.4	1.2 - 8.2	86.5	25.2 - 270.0	99.1	32.9 - 295.4	0.0	0.0 - 0.0	53.0	18.8 - 160.4	0.88
3160106	9329	*B	43.8	231.2	77 - 436	0.0	0.0 - 0.0	4.4	1.5 - 8.8	58.8	19.1 - 160.7	92.1	30.3 - 240.8	0.0	0.0 - 0.0	75.9	26.2 - 167.1	0.89
3160106	9330	*A	79.7	86.5	27 - 170	0.0	0.0 - 0.0	3.1	0.9 - 6.1	6.9	2.1 - 19.1	38.5	11.0 - 92.4	0.0	0.0 - 0.0	38.0	12.2 - 87.0	0.89
3160106	9331	FACTORY CR	98.8	129.7	39 - 235	0.0	0.0 - 0.0	3.8	1.1 - 6.6	13.0	3.6 - 32.4	58.9	15.7 - 142.4	0.0	0.0 - 0.0	54.1	16.5 - 105.2	0.90
3160106	9332	SNEADS CR	86.6	18.3	7 - 35	0.0	0.0 - 0.0	4.9	1.9 - 9.6	3.8	1.3 - 10.3	4.5	1.5 - 10.9	0.0	0.0 - 0.0	5.2	2.1 - 12.5	0.76
3160106	9333	BEAR CR	231.5	24.7	10 - 63	0.0	0.0 - 0.0	7.3	2.8 - 19.9	6.0	2.1 - 15.7	7.1	2.7 - 20.3	0.0	0.0 - 0.0	4.3	1.8 - 11.7	0.76
3160107	9334	SIPSEY R	108.6	37.8	13 - 95	0.0	0.0 - 0.0	4.7	1.7 - 11.3	8.1	2.4 - 26.3	11.9	4.4 - 34.9	0.0	0.0 - 0.0	13.2	4.2 - 38.7	0.88
3160107	9335	BRUSH CR	142.9	18.7	7 - 47	0.0	0.0 - 0.0	4.6	1.9 - 13.0	1.0	0.4 - 2.8	5.5	2.0 - 15.3	0.0	0.0 - 0.0	7.6	2.7 - 21.8	0.87
3160107	9336	SIPSEY R	155.5	19.7	7 - 47	0.0	0.0 - 0.0	4.0	1.2 - 9.3	2.9	0.8 - 9.9	4.4	1.4 - 12.0	0.0	0.0 - 0.0	8.4	3.1 - 23.8	0.87
3160107	9337	SIPSEY R	230.9	20.0	6 - 41	0.0	0.0 - 0.0	4.9	1.5 - 10.2	2.3	0.8 - 6.3	7.4	2.2 - 17.9	0.0	0.0 - 0.0	5.4	1.8 - 13.4	0.85
3160107	9338	SIPSEY R	88.9	15.4	6 - 30	0.0	0.0 - 0.0	4.2	1.6 - 8.9	1.3	0.4 - 3.2	5.0	1.9 - 12.6	0.0	0.0 - 0.0	4.9	1.8 - 11.9	0.83

8-digit HUC	ID	Name	Area	Catchment Yield		Point sources		Developed Land		Manure		Agricultural Land		Phosphate Mines		Soil parent rock		Frac
				kg/km^2	90% CI	kg/km^2	90% CI	kg/km^2	90% CI	kg/km^2	90% CI	kg/km^2	90% CI	kg/km^2	90% CI	kg/km^2	90% CI	
3160107	9339	SIPSEY R	180.9	15.5	5 - 33	0.0	0.0 - 0.0	3.8	1.2 - 8.2	1.4	0.4 - 3.5	5.1	1.8 - 13.2	0.0	0.0 - 0.0	5.1	1.8 - 14.4	0.82
3160107	9340	SIPSEY R	11.7	26.6	9 - 71	0.0	0.0 - 0.0	6.7	2.0 - 16.8	3.6	1.1 - 11.6	8.3	2.4 - 24.2	0.0	0.0 - 0.0	8.0	3.0 - 22.4	0.80
3160107	9341	DAVIS CR	86.0	12.0	4 - 25	0.0	0.0 - 0.0	4.6	1.7 - 9.7	0.7	0.2 - 1.9	1.8	0.7 - 4.5	0.0	0.0 - 0.0	4.9	1.7 - 11.9	0.79
3160107	9342	SIPSEY R	299.2	33.4	14 - 74	7.5	3.2 - 16.9	9.3	3.6 - 23.4	2.8	0.9 - 7.4	7.0	2.6 - 17.7	0.0	0.0 - 0.0	6.8	2.6 - 15.6	0.79
3160107	9343	BOXES CR	72.1	20.9	7 - 51	0.0	0.0 - 0.0	4.5	1.5 - 10.4	3.0	0.9 - 9.0	5.0	1.9 - 13.0	0.0	0.0 - 0.0	8.5	2.7 - 23.9	0.74
3160107	9344	SIPSEY R	51.8	27.3	10 - 49	0.0	0.0 - 0.0	11.1	3.9 - 20.5	3.1	0.9 - 8.2	7.3	2.4 - 18.9	0.0	0.0 - 0.0	5.9	2.1 - 12.6	0.74
3160107	9345	BARREN CR	27.4	22.4	8 - 42	0.0	0.0 - 0.0	6.0	2.0 - 12.4	2.2	0.7 - 6.1	5.0	1.8 - 11.2	0.0	0.0 - 0.0	9.2	3.5 - 19.6	0.73
3160107	9346	SIPSEY R	37.7	30.7	11 - 84	0.0	0.0 - 0.0	6.2	2.2 - 15.8	4.3	1.2 - 12.5	10.7	3.4 - 32.3	0.0	0.0 - 0.0	9.5	3.2 - 24.7	0.73
3160107	9347	NEW R	199.2	27.9	10 - 57	0.0	0.0 - 0.0	10.7	3.9 - 22.2	5.2	1.7 - 12.3	3.8	1.3 - 8.9	0.0	0.0 - 0.0	8.2	3.0 - 20.6	0.71
3160107	9348	LITTLE NEW R	133.7	33.0	12 - 73	0.6	0.2 - 1.5	11.9	4.1 - 29.6	5.7	2.0 - 16.0	7.0	2.1 - 19.3	0.0	0.0 - 0.0	7.7	3.1 - 17.7	0.71
3160107	9349	BEAR CR	66.8	10.6	3 - 25	0.0	0.0 - 0.0	4.0	1.3 - 9.2	0.7	0.2 - 2.0	1.8	0.6 - 4.7	0.0	0.0 - 0.0	4.2	1.4 - 9.4	0.80
3160107	9350	DUNN CR	60.1	16.2	5 - 31	0.0	0.0 - 0.0	4.1	1.4 - 8.8	3.0	0.9 - 7.2	4.9	1.5 - 12.5	0.0	0.0 - 0.0	4.3	1.4 - 9.6	0.82
3160107	9351	BOX CR	48.2	13.0	4 - 24	0.0	0.0 - 0.0	5.7	1.7 - 12.5	0.9	0.2 - 2.3	2.4	0.8 - 6.3	0.0	0.0 - 0.0	4.0	1.3 - 8.0	0.83
3160107	9352	BOX CR	34.5	15.8	5 - 32	0.0	0.0 - 0.0	4.8	1.5 - 10.7	1.9	0.6 - 4.8	2.3	0.9 - 5.5	0.0	0.0 - 0.0	6.8	2.1 - 16.1	0.85
3160108	9353	NOXUBEE R	13.1	82.3	26 - 171	0.0	0.0 - 0.0	5.7	1.7 - 13.5	7.2	2.0 - 18.5	33.1	11.3 - 91.4	0.0	0.0 - 0.0	36.2	11.4 - 88.1	0.89
3160108	9354	NOXUBEE R	96.2	114.0	45 - 275	0.0	0.0 - 0.0	4.6	1.6 - 9.7	8.4	2.5 - 30.1	37.7	14.4 - 106.5	0.0	0.0 - 0.0	63.3	22.6 - 191.7	0.89
3160108	9355	WOODWARDS CR	178.3	201.5	70 - 470	0.2	0.1 - 0.5	2.9	1.0 - 6.0	28.4	8.3 - 86.6	98.3	29.3 - 265.4	0.0	0.0 - 0.0	71.7	23.1 - 210.4	0.88
3160108	9356	NOXUBEE R	394.3	131.4	50 - 307	0.4	0.2 - 0.9	3.9	1.5 - 8.2	7.8	2.5 - 22.2	49.2	17.3 - 129.2	0.0	0.0 - 0.0	70.0	24.9 - 174.4	0.88
3160108	9357	PLUM CR	87.2	219.9	65 - 428	0.0	0.0 - 0.0	3.9	1.3 - 7.0	17.1	4.6 - 40.1	115.9	31.4 - 252.9	0.0	0.0 - 0.0	83.0	25.0 - 181.6	0.84
3160108	9358	NOXUBEE R	8.9	87.2	28 - 227	0.0	0.0 - 0.0	0.8	0.3 - 1.8	1.1	0.4 - 3.1	7.9	2.2 - 22.1	0.0	0.0 - 0.0	77.4	25.5 - 220.0	0.84
3160108	9359	NOXUBEE R	36.8	173.5	49 - 376	18.7	5.5 - 48.6	15.1	4.2 - 34.0	6.8	1.8 - 20.9	47.7	14.8 - 123.3	0.0	0.0 - 0.0	85.1	23.1 - 207.1	0.83
3160108	9360	HORSE HUNTERS (	75.9	231.2	78 - 484	0.0	0.0 - 0.0	7.6	2.6 - 14.5	17.9	5.3 - 55.4	122.6	39.0 - 291.1	0.0	0.0 - 0.0	83.1	24.5 - 200.0	0.82
3160108	9361	NOXUBEE R	5.9	173.8	61 - 392	0.0	0.0 - 0.0	2.2	0.8 - 5.2	11.0	3.1 - 32.4	77.4	25.1 - 216.5	0.0	0.0 - 0.0	83.1	27.4 - 247.7	0.82
3160108	9362	JOES CR	86.2	177.7	54 - 355	4.5	1.3 - 10.4	5.3	1.5 - 11.6	12.4	3.4 - 39.4	87.3	26.2 - 198.9	0.0	0.0 - 0.0	68.1	20.1 - 159.0	0.82
3160108	9363	NOXUBEE R	56.7	171.9	66 - 364	0.0	0.0 - 0.0	2.8	1.1 - 6.5	9.1	3.0 - 26.1	62.1	21.5 - 182.1	0.0	0.0 - 0.0	97.9	38.3 - 260.1	0.82
3160108	9364	NOXUBEE R	29.7	147.2	55 - 335	0.0	0.0 - 0.0	2.8	1.0 - 6.1	4.1	1.3 - 10.9	27.7	9.4 - 78.4	0.0	0.0 - 0.0	112.6	45.0 - 283.9	0.81
3160108	9365	NOXUBEE R	263.5	167.1	53 - 390	0.0	0.0 - 0.0	4.1	1.2 - 9.0	10.0	2.9 - 27.8	58.6	18.0 - 157.4	0.0	0.0 - 0.0	94.3	29.8 - 222.9	0.80
3160108	9366	CHINCHAHOMA C	54.9	239.8	74 - 496	0.1	0.0 - 0.2	4.6	1.4 - 9.1	22.8	5.4 - 62.7	102.8	34.7 - 233.9	0.0	0.0 - 0.0	109.5	31.1 - 251.2	0.77
3160108	9367	CHINCHAHOMA C	133.9	173.0	41 - 351	0.0	0.0 - 0.0	6.0	1.6 - 12.3	8.4	2.3 - 24.6	38.4	9.8 - 82.0	0.0	0.0 - 0.0	120.1	30.1 - 257.4	0.76
3160108	9368	DRY CR	160.3	116.0	33 - 276	0.0	0.0 - 0.0	4.2	1.0 - 8.6	5.1	1.3 - 15.8	22.9	5.8 - 52.4	0.0	0.0 - 0.0	83.8	25.2 - 188.6	0.76
3160108	9369	NOXUBEE R	12.7	147.2	41 - 330	0.0	0.0 - 0.0	4.7	1.3 - 8.3	2.8	0.6 - 6.9	17.8	5.0 - 43.0	0.0	0.0 - 0.0	121.9	32.5 - 295.7	0.77
3160108	9370	SAND CR	140.0	50.7	18 - 113	0.0	0.0 - 0.1	4.8	1.8 - 10.7	4.3	1.3 - 13.0	14.4	4.8 - 38.3	0.0	0.0 - 0.0	27.2	9.6 - 76.9	0.48
3160108	9371	NOXUBEE R	89.9	57.5	19 - 116	0.0	0.0 - 0.0	3.8	1.2 - 7.0	3.0	0.8 - 8.1	13.6	4.5 - 30.9	0.0	0.0 - 0.0	37.1	11.4 - 88.3	0.48
3160108	9373	NOXUBEE R	72.1	28.9	11 - 74	0.0	0.0 - 0.0	3.8	1.2 - 9.4	1.3	0.4 - 3.8	3.6	1.2 - 9.8	0.0	0.0 - 0.0	20.3	8.3 - 59.6	0.45
3160108	9374	LITTLE NOXUBEE	35.6	32.2	13 - 71	0.0	0.0 - 0.0	3.7	1.6 - 7.8	1.0	0.3 - 2.9	4.2	1.5 - 9.6	0.0	0.0 - 0.0	23.3	8.8 - 60.4	0.45
3160108	9375	MILLER	100.2	35.3	12 - 97	0.0	0.0 - 0.0	5.2	1.6 - 13.7	2.0	0.5 - 6.9	7.5	2.2 - 20.2	0.0	0.0 - 0.0	20.6	7.3 - 60.0	0.45
3160108	9376	YELLOW CR	116.3	89.9	28 - 184	0.0	0.0 - 0.0	3.1	1.1 - 6.5	2.4	0.8 - 6.3	11.6	3.7 - 28.9	0.0	0.0 - 0.0	72.8	23.7 - 172.7	0.80
3160108	9377	HASHUQUA CR	376.2	61.8	22 - 140	0.0	0.0 - 0.1	4.5	1.4 - 9.0	2.3	0.7 - 5.9	12.9	4.2 - 30.2	0.0	0.0 - 0.0	42.0	14.9 - 110.1	0.81
3160108	9378	MACEDONIA CR	201.3	89.1	28 - 193	0.0	0.0 - 0.0	4.6	1.3 - 9.4	3.0	0.7 - 8.2	20.6	6.5 - 52.5	0.0	0.0 - 0.0	60.9	19.2 - 140.5	0.83

8-digit HUC	ID	Name	Area	Catchment Yield kg/km^2	Catchment Yield 90% CI	Point sources kg/km^2	Point sources 90% CI	Developed Land kg/km^2	Developed Land 90% CI	Manure kg/km^2	Manure 90% CI	Agricultural Land kg/km^2	Agricultural Land 90% CI	Phosphate Mines kg/km^2	Phosphate Mines 90% CI	Soil parent rock kg/km^2	Soil parent rock 90% CI	Frac
3160108	9379	BODKA CR	170.7	112.6	45 - 220	0.0	0.0 - 0.0	3.6	1.5 - 7.2	10.8	3.3 - 32.5	48.0	19.5 - 121.3	0.0	0.0 - 0.0	50.2	19.5 - 116.4	0.89
3160108	9380	BODKA CR	7.6	96.6	31 - 197	0.0	0.0 - 0.0	3.2	1.0 - 6.1	2.1	0.5 - 4.8	7.7	2.4 - 15.8	0.0	0.0 - 0.0	83.7	26.3 - 174.6	0.83
3160108	9381	BODKA CR	44.8	136.1	47 - 332	0.0	0.0 - 0.0	3.0	1.0 - 7.2	11.8	4.0 - 33.2	47.3	17.0 - 140.2	0.0	0.0 - 0.0	74.1	25.9 - 205.6	0.81
3160108	9382	*A	172.0	108.2	35 - 241	0.0	0.0 - 0.0	5.7	1.9 - 10.6	6.0	1.8 - 15.7	24.9	7.9 - 68.3	0.0	0.0 - 0.0	71.7	21.9 - 189.3	0.81
3160108	9383	BIG SCOOBA CR	276.7	105.7	34 - 222	0.6	0.2 - 1.3	4.8	1.3 - 9.9	4.4	1.2 - 10.9	18.6	5.5 - 39.8	0.0	0.0 - 0.0	77.4	24.4 - 188.4	0.83
3160109	9384	BLACK WARRIOR	36.2	10.9	4 - 26	0.0	0.0 - 0.0	0.9	0.3 - 2.1	0.1	0.0 - 0.5	0.2	0.1 - 0.6	0.0	0.0 - 0.0	9.6	3.6 - 23.3	0.60
3160109	9385	BLACK WARRIOR	232.7	21.3	8 - 43	0.0	0.0 - 0.0	8.6	3.0 - 16.9	1.3	0.4 - 4.1	3.1	1.1 - 6.8	0.0	0.0 - 0.0	8.4	3.1 - 22.4	0.59
3160109	9386	BLACK WARRIOR	23.5	26.5	10 - 66	0.0	0.0 - 0.0	6.6	2.5 - 16.2	3.3	1.0 - 8.8	5.6	1.6 - 18.9	0.0	0.0 - 0.0	11.0	4.3 - 26.9	0.58
3160109	9387	BLACK WARRIOR	64.6	24.6	7 - 56	0.0	0.0 - 0.0	6.4	2.0 - 14.2	4.0	1.0 - 10.5	6.8	1.9 - 18.2	0.0	0.0 - 0.0	7.4	2.2 - 20.0	0.58
3160109	9388	BLACK WARRIOR	100.9	25.0	9 - 51	0.0	0.0 - 0.0	5.1	1.8 - 11.1	5.6	1.9 - 15.6	7.4	2.6 - 18.4	0.0	0.0 - 0.0	7.0	2.7 - 15.2	0.57
3160109	9389	BLACK WARRIOR	175.4	36.8	13 - 81	0.0	0.0 - 0.0	5.9	2.1 - 12.1	13.6	4.5 - 37.0	12.3	3.9 - 31.2	0.0	0.0 - 0.0	5.1	1.6 - 10.8	0.56
3160109	9390	BLACK WARRIOR	187.7	30.0	9 - 71	0.2	0.1 - 0.6	6.8	2.0 - 16.0	9.1	2.6 - 33.2	7.8	2.5 - 19.1	0.0	0.0 - 0.0	6.1	1.9 - 16.6	0.55
3160109	9391	BLACK WARRIOR	1.8	50.7	17 - 145	0.0	0.0 - 0.0	9.3	2.9 - 24.6	18.1	5.5 - 59.6	18.3	5.8 - 60.0	0.0	0.0 - 0.0	5.0	1.7 - 13.9	0.53
3160109	9392	BLACK WARRIOR	4.4	45.9	14 - 100	0.0	0.0 - 0.0	6.3	2.0 - 12.5	15.8	5.1 - 42.1	18.0	5.0 - 45.1	0.0	0.0 - 0.0	5.9	2.0 - 14.8	0.53
3160109	9393	COPELAND CR	62.7	55.3	17 - 125	6.1	2.2 - 15.7	9.9	3.1 - 25.1	14.0	4.3 - 42.5	17.9	5.0 - 50.2	0.0	0.0 - 0.0	7.3	2.8 - 18.0	0.53
3160109	9394	BLACK WARRIOR	4.5	49.7	17 - 100	0.0	0.0 - 0.0	7.8	2.7 - 16.1	18.9	6.1 - 48.1	18.1	6.5 - 47.6	0.0	0.0 - 0.0	4.9	1.7 - 11.4	0.53
3160109	9395	BLACK WARRIOR	126.8	60.0	19 - 132	0.8	0.3 - 1.8	8.2	2.5 - 17.9	24.3	7.1 - 67.6	21.7	6.8 - 63.3	0.0	0.0 - 0.0	5.1	1.6 - 12.0	0.52
3160109	9396	BLACK WARRIOR	6.8	60.6	18 - 150	0.0	0.0 - 0.0	17.1	5.1 - 48.4	18.2	4.1 - 56.6	20.0	6.5 - 59.2	0.0	0.0 - 0.0	5.3	1.6 - 13.7	0.49
3160109	9397	BLACK WARRIOR	35.7	50.7	17 - 104	0.0	0.0 - 0.0	11.5	3.7 - 23.7	15.1	3.9 - 40.4	18.5	5.1 - 50.2	0.0	0.0 - 0.0	5.6	1.7 - 13.2	0.48
3160109	9398	*A	26.6	60.0	20 - 119	0.0	0.0 - 0.0	6.2	2.1 - 11.4	27.5	8.3 - 64.9	21.5	6.4 - 48.5	0.0	0.0 - 0.0	4.9	1.7 - 10.8	0.48
3160109	9399	TIBB CR	61.8	58.7	18 - 138	0.0	0.0 - 0.0	14.1	4.4 - 35.3	21.2	6.1 - 58.3	18.7	7.0 - 56.4	0.0	0.0 - 0.0	4.7	1.4 - 11.9	0.46
3160109	9400	*A	44.7	60.4	18 - 135	0.0	0.0 - 0.0	8.2	2.3 - 16.7	26.5	7.5 - 68.8	21.1	6.0 - 57.7	0.0	0.0 - 0.0	4.6	1.5 - 12.5	0.46
3160109	9401	PAN CR	29.1	70.3	22 - 161	0.0	0.0 - 0.0	9.4	2.8 - 21.1	32.3	10.9 - 76.8	23.9	6.8 - 73.3	0.0	0.0 - 0.0	4.7	1.3 - 12.2	0.49
3160109	9402	DUCK CR	163.0	63.6	23 - 116	0.0	0.0 - 0.0	9.4	3.3 - 21.5	28.4	9.2 - 58.8	21.0	7.2 - 47.6	0.0	0.0 - 0.0	4.7	1.7 - 10.7	0.52
3160109	9403	BROGLEN R	79.6	66.2	20 - 144	0.0	0.0 - 0.0	12.1	3.9 - 32.8	28.2	7.8 - 77.4	20.8	6.4 - 57.1	0.0	0.0 - 0.0	5.1	1.8 - 11.7	0.53
3160109	9404	BRINDLEY CR	66.8	65.0	17 - 151	0.0	0.0 - 0.0	9.6	2.5 - 21.6	29.0	7.1 - 99.3	21.5	6.1 - 56.6	0.0	0.0 - 0.0	4.8	1.4 - 11.7	0.50
3160109	9405	EIGHTMILE CR	32.5	269.8	86 - 736	189.5	61.4 - 541.9	47.0	16.0 - 120.5	16.3	4.3 - 53.4	11.9	3.6 - 37.6	0.0	0.0 - 0.0	5.2	1.5 - 13.7	0.50
3160109	9406	EIGHTMILE CR	80.6	28.5	8 - 69	0.0	0.0 - 0.0	5.9	1.9 - 15.5	11.5	3.1 - 31.5	8.9	2.7 - 26.4	0.0	0.0 - 0.0	2.3	0.7 - 6.4	0.47
3160109	9407	BRIDGE CR	29.0	62.7	23 - 140	0.0	0.0 - 0.0	37.4	13.8 - 89.2	12.2	4.3 - 35.7	8.8	3.4 - 25.0	0.0	0.0 - 0.0	4.2	1.6 - 10.7	0.47
3160109	9408	*B	48.0	72.9	23 - 127	0.0	0.0 - 0.0	19.0	6.4 - 37.9	28.0	7.2 - 80.8	20.4	6.3 - 48.1	0.0	0.0 - 0.0	5.5	1.8 - 11.5	0.53
3160109	9409	MARRIOTT CR	67.8	27.9	10 - 61	0.0	0.0 - 0.1	7.6	2.6 - 17.5	9.5	3.0 - 24.5	6.6	2.1 - 19.3	0.0	0.0 - 0.0	4.1	1.4 - 8.9	0.55
3160109	9410	DORSEY CR	76.9	29.1	8 - 64	0.0	0.0 - 0.0	4.6	1.4 - 9.4	12.2	3.3 - 34.0	7.1	2.1 - 19.6	0.0	0.0 - 0.0	5.2	1.5 - 11.9	0.56
3160109	9411	BLACKWATER CR	1.8	29.5	13 - 69	0.0	0.0 - 0.0	16.7	6.4 - 41.5	1.8	0.6 - 6.5	2.5	1.0 - 6.2	0.0	0.0 - 0.0	8.6	3.8 - 19.8	0.58
3160109	9412	BLACKWATER CR	103.9	24.5	8 - 48	0.0	0.0 - 0.0	5.5	1.6 - 11.2	4.3	1.3 - 10.0	7.6	2.2 - 19.8	0.0	0.0 - 0.0	7.1	2.4 - 13.9	0.58
3160109	9413	BLACKWATER CR	227.1	20.4	7 - 40	0.0	0.0 - 0.1	7.8	2.8 - 19.1	3.7	1.1 - 10.4	3.7	1.2 - 10.5	0.0	0.0 - 0.0	5.1	1.8 - 10.6	0.50
3160109	9414	POLEY CR	42.4	48.9	15 - 84	0.2	0.0 - 0.4	22.1	6.6 - 39.2	6.7	2.0 - 14.9	12.0	3.5 - 29.5	0.0	0.0 - 0.0	7.9	2.8 - 19.3	0.58
3160109	9415	CANE CR	186.7	76.0	24 - 200	34.4	10.9 - 95.8	21.9	6.7 - 58.3	3.9	1.2 - 12.8	6.7	2.2 - 22.1	0.0	0.0 - 0.0	9.1	3.0 - 23.6	0.58
3160109	9416	LOST CR	16.6	14.6	7 - 28	0.0	0.0 - 0.0	3.2	1.2 - 6.8	0.8	0.2 - 2.1	1.4	0.5 - 3.3	0.0	0.0 - 0.0	9.1	4.2 - 19.7	0.59
3160109	9417	LOST CR	531.3	25.1	9 - 58	0.0	0.0 - 0.0	6.2	1.9 - 15.1	3.8	1.1 - 10.5	5.9	1.9 - 15.8	0.0	0.0 - 0.0	9.1	3.0 - 21.3	0.59

8-digit HUC	ID	Name	Area	Catchment Yield		Point sources		Developed Land		Manure		Agricultural Land		Phosphate Mines		Soil parent rock		Frac
				kg/km^2	90% CI	kg/km^2	90% CI	kg/km^2	90% CI	kg/km^2	90% CI	kg/km^2	90% CI	kg/km^2	90% CI	kg/km^2	90% CI	
3160109	9418	WOLF CR	352.4	14.7	5 - 28	0.0	0.0 - 0.0	2.2	0.6 - 4.6	1.2	0.4 - 3.5	1.7	0.5 - 4.2	0.0	0.0 - 0.0	9.6	3.1 - 20.9	0.59
3160110	9419	BLACK WARRIOR	75.2	22.7	8 - 39	0.0	0.0 - 0.0	5.7	1.9 - 11.2	5.0	1.6 - 14.1	5.8	2.0 - 13.6	0.0	0.0 - 0.0	6.2	2.3 - 12.3	0.57
3160110	9420	BLACK WARRIOR	60.3	26.0	9 - 44	0.0	0.0 - 0.0	4.9	1.7 - 9.0	6.0	1.9 - 13.6	9.9	3.2 - 23.9	0.0	0.0 - 0.0	5.2	1.7 - 10.9	0.56
3160110	9421	BLEVENS CR	40.6	53.6	19 - 107	0.0	0.0 - 0.0	9.3	3.1 - 21.3	21.2	6.9 - 62.4	17.8	5.5 - 45.1	0.0	0.0 - 0.0	5.3	2.1 - 13.3	0.18
3160110	9422	JONES CR	30.2	42.9	15 - 98	0.0	0.0 - 0.0	4.8	1.5 - 12.2	19.0	6.0 - 50.2	13.7	4.3 - 34.9	0.0	0.0 - 0.0	5.4	1.9 - 12.8	0.17
3160110	9423	BLEVENS CR	33.9	33.1	11 - 70	0.0	0.0 - 0.0	4.2	1.3 - 8.5	13.7	4.1 - 38.4	9.9	3.1 - 25.9	0.0	0.0 - 0.0	5.3	1.9 - 14.2	0.17
3160110	9424	BRUSHY FK	79.3	37.9	11 - 86	0.1	0.0 - 0.2	6.7	2.0 - 15.1	12.6	3.6 - 32.8	13.5	3.9 - 38.8	0.0	0.0 - 0.0	5.0	1.7 - 11.9	0.18
3160110	9425	CASPEY CR	62.9	10.7	3 - 24	0.0	0.0 - 0.0	2.0	0.6 - 5.4	2.1	0.6 - 5.6	2.3	0.7 - 6.5	0.0	0.0 - 0.0	4.3	1.4 - 10.5	0.18
3160110	9426	BLACK WARRIOR	1.7	9.8	3 - 22	0.0	0.0 - 0.0	5.1	1.6 - 12.1	0.1	0.0 - 0.4	0.0	0.0 - 0.0	0.0	0.0 - 0.0	4.6	1.7 - 11.1	0.18
3160110	9427	RUSH CR	35.9	7.7	3 - 16	0.0	0.0 - 0.0	2.6	1.0 - 5.9	0.4	0.1 - 1.2	0.4	0.1 - 1.0	0.0	0.0 - 0.0	4.4	1.4 - 10.1	0.17
3160110	9428	BLACK WARRIOR	28.7	14.2	5 - 27	0.0	0.0 - 0.0	4.7	1.7 - 9.4	2.3	0.7 - 5.6	2.8	0.9 - 7.3	0.0	0.0 - 0.0	4.4	1.6 - 9.7	0.17
3160110	9429	BRUSHY CR	56.3	6.9	3 - 16	0.0	0.0 - 0.0	1.7	0.6 - 4.1	0.2	0.1 - 0.7	0.6	0.2 - 1.7	0.0	0.0 - 0.0	4.4	1.7 - 9.4	0.17
3160110	9430	LEWIS SMITH L	32.2	9.3	3 - 16	0.0	0.0 - 0.0	3.3	1.0 - 6.4	0.6	0.1 - 1.4	1.1	0.3 - 2.2	0.0	0.0 - 0.0	4.4	1.2 - 8.9	0.17
3160110	9431	BLACK WARRIOR	22.4	12.5	5 - 29	0.0	0.0 - 0.0	4.9	1.8 - 10.6	1.4	0.5 - 4.1	1.5	0.6 - 4.4	0.0	0.0 - 0.0	4.6	1.9 - 11.0	0.18
3160110	9432	BLACK WARRIOR	36.2	7.2	3 - 14	0.0	0.0 - 0.0	2.4	0.8 - 4.5	0.1	0.0 - 0.4	0.1	0.0 - 0.1	0.0	0.0 - 0.0	4.6	1.7 - 10.5	0.18
3160110	9433	BORDEN CR	97.2	6.2	2 - 13	0.0	0.0 - 0.0	1.5	0.5 - 3.2	0.1	0.0 - 0.3	0.2	0.1 - 0.5	0.0	0.0 - 0.0	4.4	1.6 - 9.9	0.17
3160110	9434	BLACK WARRIOR	29.6	5.3	2 - 9	0.0	0.0 - 0.0	0.7	0.2 - 1.1	0.0	0.0 - 0.0	0.0	0.0 - 0.0	0.0	0.0 - 0.0	4.6	1.5 - 8.6	0.17
3160110	9435	MATTOX CR	55.6	6.2	2 - 14	0.0	0.0 - 0.0	1.6	0.5 - 3.1	0.0	0.0 - 0.1	0.1	0.0 - 0.2	0.0	0.0 - 0.0	4.5	1.4 - 10.5	0.16
3160110	9436	HUBBARD CR	49.0	15.7	6 - 37	0.0	0.0 - 0.0	4.4	1.6 - 10.1	3.2	1.1 - 8.1	3.6	1.1 - 10.9	0.0	0.0 - 0.0	4.6	1.6 - 12.1	0.16
3160110	9437	CANEY CR	48.0	10.1	4 - 25	0.0	0.0 - 0.0	2.3	0.9 - 5.4	1.5	0.5 - 5.4	1.8	0.6 - 5.2	0.0	0.0 - 0.0	4.5	1.6 - 11.4	0.18
3160110	9438	SANDY CR	41.6	30.2	9 - 71	0.0	0.0 - 0.0	8.3	3.0 - 18.9	8.7	2.6 - 26.2	9.2	2.9 - 30.4	0.0	0.0 - 0.0	4.0	1.6 - 9.7	0.18
3160110	9439	CLEAR CR, RIGHT	81.4	29.3	11 - 57	0.0	0.0 - 0.0	7.0	2.6 - 13.6	8.7	3.1 - 21.5	8.9	3.2 - 21.1	0.0	0.0 - 0.0	4.7	1.9 - 9.6	0.17
3160110	9440	CLEAR CR	103.8	30.9	10 - 54	0.0	0.0 - 0.0	11.4	3.3 - 23.5	7.4	2.4 - 16.6	7.3	2.4 - 17.5	0.0	0.0 - 0.0	4.7	1.7 - 10.7	0.17
3160111	9441	BLACK WARRIOR	91.3	15.9	5 - 34	0.0	0.0 - 0.0	5.2	1.8 - 13.2	0.4	0.1 - 0.9	1.3	0.4 - 4.0	0.0	0.0 - 0.0	9.0	3.2 - 21.1	0.60
3160111	9442	SHORT CR	51.3	16.4	6 - 37	0.0	0.0 - 0.0	6.9	2.8 - 16.2	0.3	0.1 - 0.7	0.9	0.4 - 2.0	0.0	0.0 - 0.0	8.3	3.2 - 19.8	0.59
3160111	9443	BLACK WARRIOR	5.5	22.7	7 - 40	0.0	0.0 - 0.0	13.4	3.6 - 25.7	0.3	0.1 - 0.7	0.3	0.1 - 0.7	0.0	0.0 - 0.0	8.7	3.1 - 17.9	0.59
3160111	9444	VILLAGE CR	254.2	78.1	24 - 158	10.6	3.7 - 20.8	54.0	16.8 - 110.5	0.3	0.1 - 0.9	1.8	0.5 - 4.1	0.0	0.0 - 0.0	11.3	3.6 - 26.3	0.59
3160111	9445	BLACK WARRIOR	36.8	26.8	9 - 50	0.0	0.0 - 0.0	14.1	4.6 - 31.5	0.9	0.2 - 2.5	3.3	1.1 - 9.3	0.0	0.0 - 0.0	8.5	3.0 - 20.4	0.59
3160111	9446	FIVEMILE CR	9.5	12.6	4 - 26	0.0	0.0 - 0.0	3.2	1.1 - 6.4	0.8	0.2 - 2.3	0.2	0.1 - 0.5	0.0	0.0 - 0.0	8.4	3.0 - 18.3	0.58
3160111	9447	BLACK WARRIOR	20.0	18.9	5 - 39	0.0	0.0 - 0.0	6.4	1.6 - 13.9	0.6	0.1 - 1.7	3.6	1.0 - 9.9	0.0	0.0 - 0.0	8.3	2.4 - 20.0	0.58
3160111	9448	CANE CR	42.0	17.8	6 - 32	0.0	0.0 - 0.0	8.0	2.5 - 16.3	0.6	0.2 - 1.4	1.2	0.4 - 2.8	0.0	0.0 - 0.0	8.0	2.6 - 15.9	0.58
3160111	9449	BLACK WARRIOR	20.2	27.4	9 - 67	0.0	0.0 - 0.0	15.0	4.9 - 43.0	0.8	0.2 - 2.5	6.0	1.9 - 17.2	0.0	0.0 - 0.0	5.6	2.0 - 13.5	0.58
3160111	9450	CROOKED CR	47.3	27.9	9 - 57	0.0	0.0 - 0.0	16.8	5.1 - 34.0	0.4	0.1 - 1.2	2.6	0.7 - 6.3	0.0	0.0 - 0.0	8.2	2.7 - 18.2	0.57
3160111	9451	BLACK WARRIOR	76.6	27.1	9 - 70	0.0	0.0 - 0.0	8.1	2.4 - 18.4	2.8	0.8 - 8.9	9.7	3.4 - 27.8	0.0	0.0 - 0.0	6.6	2.2 - 20.4	0.57
3160111	9452	TURKEY CR	16.7	42.2	18 - 93	0.3	0.1 - 0.8	23.9	9.2 - 52.2	1.0	0.4 - 3.1	8.0	3.3 - 22.4	0.0	0.0 - 0.0	8.9	3.8 - 23.4	0.56
3160111	9453	CUNNINGHAM CR	65.2	25.4	7 - 51	0.0	0.0 - 0.0	14.2	3.9 - 30.8	0.4	0.1 - 1.1	2.6	0.8 - 7.1	0.0	0.0 - 0.0	8.2	2.4 - 18.1	0.54
3160111	9454	TURKEY CR	133.8	69.9	23 - 129	27.8	9.3 - 52.4	22.1	7.4 - 46.5	0.9	0.3 - 2.5	7.3	2.4 - 17.4	0.0	0.0 - 0.0	11.8	3.5 - 26.3	0.54
3160111	9455	BLACK WARRIOR	84.7	38.6	10 - 72	0.0	0.0 - 0.0	12.9	3.4 - 27.5	6.6	1.4 - 16.3	12.2	2.8 - 31.8	0.0	0.0 - 0.0	6.9	2.3 - 15.3	0.56
3160111	9456	SELF CR	2.2	18.2	6 - 46	0.0	0.0 - 0.0	0.6	0.2 - 1.3	0.8	0.3 - 2.1	8.0	2.6 - 24.9	0.0	0.0 - 0.0	8.7	2.9 - 19.6	0.55

8-digit HUC	ID	Name	Area	Catchment Yield		Point sources		Developed Land		Manure		Agricultural Land		Phosphate Mines		Soil parent rock		Frac
				kg/km^2	90% CI	kg/km^2	90% CI	kg/km^2	90% CI	kg/km^2	90% CI	kg/km^2	90% CI	kg/km^2	90% CI	kg/km^2	90% CI	
3160111	9457	SELF CR	48.4	31.7	10 - 67	0.0	0.0 - 0.0	10.6	2.7 - 24.8	1.0	0.2 - 3.0	9.2	2.9 - 24.8	0.0	0.0 - 0.0	10.9	2.9 - 22.9	0.54
3160111	9458	GURLEY CR	94.0	31.2	12 - 70	0.0	0.0 - 0.0	9.4	3.1 - 21.5	3.9	1.2 - 11.0	7.9	2.8 - 20.3	0.0	0.0 - 0.0	10.0	3.6 - 26.3	0.54
3160111	9459	BLACK WARRIOR	212.9	35.6	10 - 71	0.0	0.0 - 0.1	6.7	1.9 - 13.0	9.7	2.3 - 26.5	12.3	3.2 - 29.0	0.0	0.0 - 0.0	6.9	2.2 - 16.3	0.55
3160111	9460	LITTLE WARRIOR	16.1	43.2	15 - 96	0.0	0.0 - 0.0	11.8	3.9 - 24.4	10.5	3.3 - 31.0	13.7	4.4 - 37.4	0.0	0.0 - 0.0	7.2	2.6 - 18.0	0.53
3160111	9461	LITTLE WARRIOR	262.0	13.2	4 - 29	0.0	0.0 - 0.0	2.4	0.7 - 5.3	3.4	0.9 - 11.4	4.3	1.2 - 11.9	0.0	0.0 - 0.0	3.1	0.9 - 7.4	0.52
3160111	9462	CALVERT PRONG	220.2	43.9	16 - 118	0.0	0.0 - 0.0	9.8	3.5 - 25.2	11.5	3.7 - 36.5	14.7	5.3 - 42.6	0.0	0.0 - 0.0	7.8	3.1 - 18.4	0.52
3160111	9463	BLACK WARRIOR	35.6	48.7	14 - 92	0.0	0.0 - 0.0	6.3	1.9 - 10.8	15.4	4.3 - 44.7	20.4	5.2 - 53.1	0.0	0.0 - 0.0	6.7	2.1 - 14.7	0.53
3160111	9464	*A	51.4	58.9	23 - 130	0.0	0.0 - 0.0	11.4	4.4 - 24.4	17.8	5.0 - 46.6	22.1	8.6 - 57.2	0.0	0.0 - 0.0	7.7	2.7 - 17.8	0.53
3160111	9465	BLACK WARRIOR	38.4	36.1	11 - 80	0.0	0.0 - 0.0	6.1	1.9 - 13.4	10.4	2.8 - 30.9	13.7	4.0 - 33.6	0.0	0.0 - 0.0	6.0	1.7 - 15.4	0.53
3160111	9466	BLACK WARRIOR	81.5	49.3	17 - 89	0.0	0.0 - 0.0	5.8	2.0 - 11.6	16.3	5.0 - 42.0	21.2	6.7 - 49.1	0.0	0.0 - 0.0	6.0	1.9 - 12.5	0.52
3160111	9467	WYNNVILLE CR	69.9	53.1	18 - 116	0.0	0.0 - 0.0	9.2	3.0 - 21.8	15.8	5.5 - 43.3	21.0	5.8 - 53.5	0.0	0.0 - 0.0	7.1	2.2 - 17.1	0.50
3160111	9468	BLACK WARRIOR	32.0	49.4	16 - 117	0.0	0.0 - 0.0	8.4	2.5 - 21.5	15.5	4.5 - 49.6	20.5	5.7 - 58.2	0.0	0.0 - 0.0	5.1	1.6 - 12.5	0.50
3160111	9469	BLACK WARRIOR	4.0	47.9	17 - 95	0.0	0.0 - 0.0	8.6	2.7 - 16.7	15.0	4.7 - 41.6	19.5	5.9 - 45.6	0.0	0.0 - 0.0	4.8	1.7 - 11.6	0.49
3160111	9470	BLACK WARRIOR	32.0	50.6	17 - 119	0.0	0.0 - 0.0	10.0	3.3 - 24.3	15.5	4.7 - 45.2	20.2	6.0 - 56.4	0.0	0.0 - 0.0	5.0	1.7 - 12.8	0.48
3160111	9471	BLACK WARRIOR	30.9	35.1	11 - 81	0.0	0.0 - 0.0	5.2	1.7 - 11.9	7.5	2.4 - 20.9	13.0	3.4 - 34.3	0.0	0.0 - 0.0	9.4	3.2 - 23.9	0.46
3160111	9472	BLACK WARRIOR	120.2	30.5	9 - 55	0.0	0.0 - 0.0	6.9	2.1 - 14.1	6.1	1.7 - 17.8	11.4	3.4 - 27.8	0.0	0.0 - 0.0	6.0	2.0 - 10.8	0.44
3160111	9473	BRISTOW CR	67.0	44.4	13 - 97	0.0	0.0 - 0.0	6.0	1.7 - 12.9	9.3	2.6 - 22.8	19.5	5.9 - 45.4	0.0	0.0 - 0.0	9.6	3.0 - 23.0	0.44
3160111	9474	CLEAR CR	74.3	98.2	32 - 166	52.1	16.5 - 97.1	12.7	4.1 - 20.7	12.0	3.5 - 26.8	16.8	5.5 - 35.0	0.0	0.0 - 0.0	4.6	1.4 - 10.1	0.46
3160111	9475	BIG MUD CR	52.4	59.8	23 - 150	0.0	0.0 - 0.0	8.0	2.7 - 20.5	22.5	7.8 - 66.0	24.5	9.0 - 78.2	0.0	0.0 - 0.0	4.7	1.8 - 11.8	0.48
3160111	9476	SLAB CR	171.6	61.1	24 - 121	0.0	0.0 - 0.0	18.0	6.2 - 37.4	18.4	6.5 - 55.1	19.8	6.9 - 50.1	0.0	0.0 - 0.0	4.9	1.7 - 12.1	0.49
3160111	9477	GRAVES CR	43.1	241.2	85 - 462	178.2	60.1 - 348.8	8.6	2.6 - 16.2	19.9	6.0 - 51.6	25.3	7.9 - 60.6	0.0	0.0 - 0.0	9.1	3.0 - 20.0	0.52
3160112	9478	BLACK WARRIOR	62.5	72.7	25 - 183	0.0	0.0 - 0.0	61.3	20.3 - 154.8	1.0	0.3 - 3.1	3.9	1.3 - 10.1	0.0	0.0 - 0.0	6.4	2.5 - 17.1	0.77
3160112	9479	BLACK WARRIOR	7.6	38.6	13 - 78	0.0	0.0 - 0.0	28.3	9.1 - 59.8	0.8	0.3 - 2.2	1.9	0.7 - 4.3	0.0	0.0 - 0.0	7.6	3.0 - 15.8	0.77
3160112	9480	HURRICANE R	21.5	39.1	10 - 70	0.0	0.0 - 0.0	27.1	6.3 - 51.1	0.8	0.2 - 2.6	2.7	0.9 - 5.8	0.0	0.0 - 0.0	8.4	2.2 - 17.5	0.76
3160112	9481	*A	46.7	42.3	14 - 103	0.0	0.0 - 0.0	32.0	10.6 - 76.4	1.2	0.3 - 3.2	4.6	1.5 - 13.6	0.0	0.0 - 0.0	4.6	1.5 - 12.1	0.74
3160112	9482	HURRICANE R	104.1	23.3	9 - 61	0.0	0.0 - 0.0	11.0	4.4 - 26.8	1.3	0.5 - 4.3	3.5	1.3 - 10.1	0.0	0.0 - 0.0	7.4	3.0 - 19.7	0.74
3160112	9483	*B	49.2	32.9	12 - 61	0.0	0.0 - 0.0	17.6	6.7 - 39.6	1.7	0.5 - 4.0	4.3	1.5 - 9.6	0.0	0.0 - 0.0	9.3	3.3 - 21.5	0.66
3160112	9484	HURRICANE R	78.7	15.3	5 - 33	0.0	0.0 - 0.0	6.6	1.9 - 14.7	0.5	0.1 - 1.4	1.0	0.3 - 2.4	0.0	0.0 - 0.0	7.3	2.0 - 19.0	0.66
3160112	9485	BLACK WARRIOR	289.1	12.5	4 - 28	0.0	0.0 - 0.0	3.2	1.2 - 7.5	0.7	0.2 - 1.7	0.8	0.3 - 2.2	0.0	0.0 - 0.0	7.8	2.7 - 20.7	0.76
3160112	9486	DAVIS CR	281.2	15.5	5 - 27	0.0	0.0 - 0.0	5.4	1.6 - 10.3	0.6	0.1 - 1.5	1.3	0.4 - 3.3	0.0	0.0 - 0.0	8.3	2.6 - 16.5	0.72
3160112	9487	BLACK WARRIOR	9.2	10.6	4 - 27	0.0	0.0 - 0.0	0.0	0.0 - 0.0	0.2	0.1 - 0.5	0.0	0.0 - 0.0	0.0	0.0 - 0.0	10.4	4.2 - 26.5	0.72
3160112	9488	BLACK WARRIOR	30.9	10.4	4 - 23	0.0	0.0 - 0.0	1.5	0.6 - 3.2	0.1	0.0 - 0.4	0.4	0.1 - 0.9	0.0	0.0 - 0.0	8.4	3.0 - 19.9	0.72
3160112	9489	BLACK WARRIOR	164.2	12.9	4 - 27	0.0	0.0 - 0.0	2.3	0.8 - 4.1	0.3	0.1 - 0.8	0.7	0.2 - 1.8	0.0	0.0 - 0.0	9.6	3.3 - 24.2	0.60
3160112	9490	VALLEY CR	14.3	14.0	5 - 30	0.0	0.0 - 0.0	3.2	0.9 - 7.0	0.3	0.1 - 0.8	1.6	0.5 - 4.3	0.0	0.0 - 0.0	9.0	2.9 - 20.5	0.60
3160112	9491	MUD CR	137.5	12.1	5 - 25	0.0	0.0 - 0.0	2.8	1.0 - 5.9	0.2	0.0 - 0.4	0.8	0.3 - 2.1	0.0	0.0 - 0.0	8.3	3.4 - 19.1	0.59
3160112	9492	VALLEY CR	42.2	15.6	6 - 34	0.0	0.0 - 0.0	5.3	1.9 - 9.9	0.2	0.1 - 0.5	1.3	0.5 - 3.6	0.0	0.0 - 0.0	8.8	3.1 - 20.8	0.59
3160112	9493	VALLEY CR	380.0	243.0	77 - 453	161.8	53.5 - 344.3	60.1	18.5 - 118.2	0.4	0.1 - 0.8	3.1	1.0 - 7.1	0.0	0.0 - 0.0	17.7	5.9 - 39.8	0.58
3160112	9494	ROCK CR	85.3	36.1	14 - 67	0.0	0.0 - 0.0	24.7	9.1 - 50.5	0.3	0.1 - 0.7	2.5	0.9 - 6.9	0.0	0.0 - 0.0	8.6	3.3 - 22.3	0.58
3160112	9495	BLACK WARRIOR	24.9	12.4	4 - 24	0.0	0.0 - 0.0	2.2	0.7 - 4.0	0.2	0.0 - 0.4	0.3	0.1 - 0.5	0.0	0.0 - 0.0	9.7	2.9 - 19.8	0.60

8-digit HUC	ID	Name	Area	Catchment Yield		Point sources		Developed Land		Manure		Agricultural Land		Phosphate Mines		Soil parent rock		Frac
				kg/km^2	90% CI	kg/km^2	90% CI	kg/km^2	90% CI	kg/km^2	90% CI	kg/km^2	90% CI	kg/km^2	90% CI	kg/km^2	90% CI	
3160112	9496	BIG YELLOW CR	171.8	12.0	4 - 22	0.0	0.0 - 0.0	1.5	0.4 - 3.2	0.8	0.2 - 2.2	1.1	0.3 - 2.7	0.0	0.0 - 0.0	8.6	2.9 - 18.0	0.60
3160112	9497	BLUE CR	108.1	12.2	4 - 24	0.0	0.0 - 0.0	1.7	0.5 - 3.5	0.8	0.2 - 2.2	1.1	0.3 - 2.4	0.0	0.0 - 0.0	8.5	2.7 - 19.8	0.72
3160112	9498	NORTH R	32.3	11.3	4 - 21	0.0	0.0 - 0.0	6.9	2.3 - 14.8	0.4	0.1 - 1.1	1.3	0.4 - 3.0	0.0	0.0 - 0.0	2.7	1.0 - 5.4	0.77
3160112	9499	NORTH R	77.0	18.4	6 - 45	0.0	0.0 - 0.0	7.5	2.6 - 18.3	1.1	0.3 - 3.6	3.8	1.2 - 12.1	0.0	0.0 - 0.0	5.9	2.1 - 14.9	0.34
3160112	9500	NORTH R	100.0	16.1	6 - 48	0.0	0.0 - 0.0	3.8	1.2 - 9.5	1.3	0.4 - 4.2	3.6	1.2 - 10.9	0.0	0.0 - 0.0	7.4	2.6 - 21.9	0.33
3160112	9501	CRIPPLE CR	45.6	15.7	6 - 33	0.0	0.0 - 0.0	3.1	1.1 - 6.7	1.5	0.5 - 3.8	3.2	1.1 - 7.8	0.0	0.0 - 0.0	7.9	3.0 - 18.5	0.32
3160112	9502	NORTH R	93.7	22.2	7 - 57	0.0	0.0 - 0.0	3.9	1.2 - 9.4	2.4	0.6 - 6.4	7.9	2.3 - 23.1	0.0	0.0 - 0.0	8.0	2.9 - 21.1	0.32
3160112	9503	PYRO CR	63.7	11.1	4 - 28	0.0	0.0 - 0.0	1.4	0.4 - 3.5	0.5	0.2 - 1.5	0.5	0.1 - 1.6	0.0	0.0 - 0.0	8.7	3.1 - 20.9	0.31
3160112	9504	NORTH R	431.2	19.0	5 - 38	0.4	0.1 - 0.9	5.1	1.5 - 10.7	1.9	0.4 - 4.5	4.1	1.0 - 10.3	0.0	0.0 - 0.0	7.6	2.3 - 17.0	0.31
3160112	9505	BINION CR	189.2	15.8	5 - 28	0.0	0.0 - 0.0	5.2	1.5 - 9.1	1.1	0.3 - 2.6	4.3	1.3 - 10.9	0.0	0.0 - 0.0	5.2	1.7 - 11.1	0.33
3160112	9506	CARROLL CR	65.4	24.4	8 - 60	0.0	0.0 - 0.0	10.9	3.5 - 26.2	1.6	0.5 - 6.1	6.5	2.4 - 17.2	0.0	0.0 - 0.0	5.5	1.9 - 15.2	0.34
3160113	9507	BLACK WARRIOR	125.9	114.2	43 - 230	0.0	0.0 - 0.0	10.2	3.6 - 20.2	10.8	3.5 - 27.9	49.0	18.6 - 114.5	0.0	0.0 - 0.0	44.2	14.7 - 111.1	0.91
3160113	9508	BIG PRARIE CR	11.8	136.5	41 - 321	0.0	0.0 - 0.0	6.3	2.1 - 13.4	15.3	4.3 - 40.1	67.2	17.3 - 165.6	0.0	0.0 - 0.0	47.7	15.9 - 116.1	0.91
3160113	9509	BIG PRARIE CR	5.6	130.7	39 - 315	0.0	0.0 - 0.0	0.8	0.2 - 1.7	14.1	3.4 - 49.7	63.5	18.1 - 182.6	0.0	0.0 - 0.0	52.4	17.1 - 134.1	0.91
3160113	9510	BIG PRARIE CR	45.9	165.7	57 - 322	0.0	0.0 - 0.0	5.5	1.8 - 10.6	18.5	5.1 - 53.1	81.9	27.0 - 208.2	0.0	0.0 - 0.0	59.7	20.8 - 126.9	0.90
3160113	9511	COTTONWOOD CR	116.5	149.5	47 - 310	0.4	0.1 - 1.1	7.6	2.5 - 15.9	15.5	4.4 - 47.5	71.2	21.6 - 176.1	0.0	0.0 - 0.0	54.9	18.1 - 145.9	0.88
3160113	9512	BIG PRARIE CR	151.8	142.2	48 - 325	0.0	0.0 - 0.0	3.0	1.0 - 6.0	13.5	4.1 - 44.1	69.0	20.9 - 205.0	0.0	0.0 - 0.0	56.8	18.7 - 138.0	0.88
3160113	9513	BIG PRARIE CR	87.7	34.8	12 - 62	0.0	0.0 - 0.0	4.6	1.6 - 8.3	2.1	0.7 - 4.5	12.3	3.7 - 28.1	0.0	0.0 - 0.0	15.8	4.8 - 32.9	0.84
3160113	9514	WHITSIT CR	65.2	120.2	36 - 237	0.0	0.0 - 0.0	4.0	1.3 - 7.8	13.3	3.5 - 43.3	59.5	19.5 - 138.3	0.0	0.0 - 0.0	43.4	13.7 - 99.9	0.84
3160113	9515	LITTLE PRAIRIE C	85.7	129.7	45 - 273	0.0	0.0 - 0.0	6.7	2.2 - 14.3	14.1	3.9 - 43.4	62.4	22.1 - 165.1	0.0	0.0 - 0.0	46.5	15.3 - 107.4	0.90
3160113	9516	BIG GERMAN CR	96.0	102.7	32 - 186	0.0	0.0 - 0.0	4.2	1.3 - 7.5	11.6	2.8 - 35.1	51.6	17.8 - 105.2	0.0	0.0 - 0.0	35.4	11.3 - 76.7	0.91
3160113	9517	BLACK WARRIOR	403.3	72.1	21 - 140	0.0	0.0 - 0.0	4.8	1.5 - 9.7	6.0	1.7 - 17.7	30.3	10.5 - 74.9	0.0	0.0 - 0.0	31.1	8.3 - 74.7	0.91
3160113	9518	BIG BRUSH CR	118.3	21.4	9 - 48	0.0	0.0 - 0.0	4.8	1.8 - 9.9	1.6	0.5 - 4.0	7.0	2.7 - 20.7	0.0	0.0 - 0.0	8.1	3.6 - 19.9	0.82
3160113	9519	COLWELL CR	52.9	40.3	11 - 76	17.8	4.6 - 35.5	10.5	3.0 - 21.4	1.0	0.3 - 2.4	4.5	1.4 - 10.9	0.0	0.0 - 0.0	6.4	1.7 - 13.2	0.77
3160113	9520	BIG BRUSH CR	48.8	15.6	6 - 27	0.0	0.0 - 0.0	5.2	1.7 - 10.0	0.8	0.3 - 2.1	3.6	1.3 - 8.3	0.0	0.0 - 0.0	6.0	2.2 - 12.9	0.77
3160113	9521	POLECAT CR	73.9	10.3	3 - 19	0.0	0.0 - 0.0	2.3	0.7 - 4.4	0.3	0.1 - 0.9	2.0	0.6 - 4.8	0.0	0.0 - 0.0	5.7	1.7 - 13.9	0.74
3160113	9522	BIG BRUSH CR	12.6	14.9	5 - 37	0.0	0.0 - 0.0	5.0	1.8 - 12.4	0.6	0.2 - 1.9	3.1	1.0 - 8.7	0.0	0.0 - 0.0	6.1	2.0 - 15.2	0.74
3160113	9523	BIG BRUSH CR	2.4	14.3	4 - 25	0.0	0.0 - 0.0	6.1	1.7 - 12.5	0.5	0.1 - 1.4	1.4	0.4 - 3.4	0.0	0.0 - 0.0	6.4	1.9 - 14.5	0.73
3160113	9524	BIG BRUSH CR	107.3	10.4	3 - 20	0.0	0.0 - 0.0	3.7	1.1 - 7.7	0.2	0.1 - 0.6	1.4	0.4 - 3.6	0.0	0.0 - 0.0	5.1	1.7 - 11.8	0.72
3160113	9525	LITTLE BRUSH CR	42.5	15.1	5 - 35	0.0	0.0 - 0.0	5.2	1.8 - 14.3	0.7	0.2 - 2.0	3.3	1.2 - 9.7	0.0	0.0 - 0.0	5.9	2.0 - 14.4	0.72
3160113	9526	SPARKS CR	55.6	14.9	6 - 36	0.0	0.0 - 0.0	4.4	1.7 - 10.6	0.8	0.3 - 2.3	3.6	1.4 - 10.9	0.0	0.0 - 0.0	6.0	2.3 - 14.6	0.73
3160113	9527	BLACK WARRIOR	8.5	29.9	9 - 70	0.0	0.0 - 0.0	2.0	0.6 - 4.1	0.6	0.2 - 1.9	4.0	1.2 - 9.2	0.0	0.0 - 0.0	23.3	7.1 - 55.9	0.82
3160113	9528	BLACK WARRIOR	65.2	24.7	9 - 53	0.0	0.0 - 0.0	3.4	1.2 - 6.8	1.1	0.4 - 3.4	5.3	1.9 - 12.5	0.0	0.0 - 0.0	15.0	5.3 - 37.5	0.82
3160113	9529	FIVEMILE CR	301.4	11.7	5 - 28	0.0	0.0 - 0.0	3.4	1.3 - 8.3	0.6	0.2 - 1.7	2.7	0.9 - 7.2	0.0	0.0 - 0.0	5.1	1.9 - 12.4	0.82
3160113	9530	BLACK WARRIOR	248.4	20.4	6 - 43	0.0	0.0 - 0.0	4.9	1.5 - 11.1	1.5	0.3 - 3.9	6.5	1.5 - 17.6	0.0	0.0 - 0.0	7.5	2.1 - 19.0	0.82
3160113	9531	ELLIOTTS CR	124.5	25.6	10 - 62	0.0	0.0 - 0.0	5.5	2.0 - 11.6	2.3	0.7 - 5.9	10.2	3.9 - 30.4	0.0	0.0 - 0.0	7.6	2.9 - 19.3	0.81
3160113	9532	BLACK WARRIOR	18.8	34.4	10 - 72	0.0	0.0 - 0.0	1.5	0.4 - 3.3	2.9	0.8 - 7.9	12.4	3.3 - 33.4	0.0	0.0 - 0.0	17.6	4.6 - 39.5	0.81
3160113	9533	BLACK WARRIOR	36.3	80.5	28 - 171	0.0	0.0 - 0.0	5.4	1.8 - 11.0	9.8	2.8 - 26.0	38.4	13.8 - 105.8	0.0	0.0 - 0.0	26.9	8.5 - 58.1	0.81
3160113	9534	BIG SANDY CR	460.6	11.3	4 - 25	0.0	0.0 - 0.0	3.5	1.2 - 8.8	0.6	0.2 - 1.9	2.3	0.7 - 6.0	0.0	0.0 - 0.0	4.9	1.6 - 15.4	0.81

8-digit HUC	ID	Name	Area	Catchment Yield		Point sources		Developed Land		Manure		Agricultural Land		Phosphate Mines		Soil parent rock		Frac
				kg/km^2	90% CI	kg/km^2	90% CI	kg/km^2	90% CI	kg/km^2	90% CI	kg/km^2	90% CI	kg/km^2	90% CI	kg/km^2	90% CI	
3160113	9535	BLACK WARRIOR	313.2	145.9	45 - 269	82.5	24.8 - 166.3	32.7	10.7 - 64.7	4.0	1.1 - 11.2	15.7	5.3 - 35.0	0.0	0.0 - 0.0	11.0	3.2 - 27.1	0.81
3160113	9536	BLACK WARRIOR	2.3	135.4	47 - 276	0.0	0.0 - 0.0	5.0	1.8 - 12.0	22.0	7.1 - 79.0	80.5	25.6 - 178.4	0.0	0.0 - 0.0	27.8	10.1 - 62.3	0.80
3160113	9537	BLACK WARRIOR	11.1	107.4	38 - 222	0.0	0.0 - 0.0	51.3	17.8 - 103.5	8.3	2.9 - 19.3	32.2	11.2 - 76.2	0.0	0.0 - 0.0	15.6	5.6 - 35.7	0.80
3160113	9538	BLACK WARRIOR	2.6	1375.3	450 - 3472	1271.4	403.2 - 3566.(	84.7	25.5 - 197.7	2.8	0.8 - 9.1	9.9	3.0 - 30.3	0.0	0.0 - 0.0	6.5	2.0 - 17.8	0.80
3160113	9539	*A	41.4	42.3	15 - 77	0.0	0.0 - 0.0	30.9	10.5 - 59.6	1.4	0.5 - 3.0	5.4	1.7 - 11.6	0.0	0.0 - 0.0	4.6	1.6 - 9.8	0.80
3160113	9540	*B	24.4	65.1	20 - 160	0.0	0.0 - 0.0	35.2	10.2 - 90.8	4.9	1.4 - 12.8	19.4	6.4 - 52.0	0.0	0.0 - 0.0	5.6	1.6 - 14.4	0.80
3160113	9541	*C	92.1	16.2	5 - 35	0.0	0.0 - 0.0	6.5	2.1 - 13.7	1.1	0.3 - 2.7	4.5	1.4 - 12.6	0.0	0.0 - 0.0	4.0	1.4 - 8.9	0.80
3160113	9542	GANT CR	93.7	22.4	7 - 36	0.0	0.0 - 0.0	5.6	1.8 - 10.6	2.0	0.6 - 5.5	7.9	2.7 - 17.6	0.0	0.0 - 0.0	6.8	2.4 - 14.3	0.81
3160113	9543	MINTER CR	84.1	18.3	6 - 38	0.0	0.0 - 0.0	7.0	2.0 - 16.1	0.6	0.2 - 1.5	3.5	1.0 - 9.1	0.0	0.0 - 0.0	7.3	2.8 - 16.3	0.82
3160201	9544	TOMBIGBEE R	19.2	28.6	9 - 54	0.0	0.0 - 0.0	3.3	1.0 - 6.3	0.2	0.0 - 0.5	1.2	0.3 - 3.0	0.0	0.0 - 0.0	23.9	7.5 - 48.4	0.94
3160201	9545	TOMBIGBEE R	8.9	19.4	7 - 44	0.0	0.0 - 0.0	2.7	0.9 - 7.0	0.2	0.0 - 0.5	0.8	0.3 - 2.2	0.0	0.0 - 0.0	15.7	5.7 - 39.3	0.94
3160201	9546	TOMBIGBEE R	28.6	25.8	9 - 57	0.0	0.0 - 0.0	3.2	1.0 - 7.2	1.2	0.3 - 3.2	6.3	1.8 - 18.4	0.0	0.0 - 0.0	15.1	4.8 - 38.3	0.93
3160201	9547	TOMBIGBEE R	125.7	15.6	5 - 35	0.0	0.0 - 0.0	2.4	0.7 - 6.1	0.4	0.1 - 1.0	1.9	0.6 - 5.2	0.0	0.0 - 0.0	11.0	3.7 - 26.1	0.93
3160201	9548	BASHI CR	38.5	21.0	5 - 42	0.0	0.0 - 0.0	2.1	0.6 - 4.6	0.5	0.1 - 1.1	2.5	0.6 - 6.4	0.0	0.0 - 0.0	15.8	4.3 - 33.8	0.93
3160201	9549	TALLAHATTA CR	92.2	12.1	4 - 20	0.0	0.0 - 0.0	1.8	0.6 - 3.4	0.3	0.1 - 0.7	1.7	0.6 - 4.0	0.0	0.0 - 0.0	8.2	2.8 - 16.5	0.90
3160201	9550	BASHI CR	219.8	13.8	5 - 28	0.0	0.0 - 0.0	2.5	0.9 - 5.4	0.5	0.1 - 1.2	2.4	0.9 - 6.6	0.0	0.0 - 0.0	8.5	3.3 - 17.5	0.90
3160201	9551	TOMBIGBEE R	77.8	23.2	6 - 47	0.0	0.0 - 0.0	2.7	0.9 - 6.0	1.2	0.3 - 3.2	5.9	1.6 - 14.4	0.0	0.0 - 0.0	13.4	3.8 - 28.8	0.93
3160201	9552	TOMBIGBEE R	2.4	21.1	7 - 44	0.0	0.0 - 0.0	2.1	0.7 - 4.1	0.1	0.0 - 0.3	0.0	0.0 - 0.0	0.0	0.0 - 0.0	18.8	6.4 - 40.8	0.93
3160201	9553	TOMBIGBEE R	22.9	19.2	6 - 44	0.0	0.0 - 0.0	3.7	1.1 - 7.6	0.7	0.2 - 1.8	3.4	1.2 - 8.6	0.0	0.0 - 0.0	11.4	3.7 - 27.6	0.93
3160201	9554	TOMBIGBEE R	45.2	22.9	8 - 56	0.0	0.0 - 0.0	2.5	0.9 - 5.7	1.7	0.5 - 5.3	7.5	2.7 - 20.9	0.0	0.0 - 0.0	11.2	3.7 - 27.2	0.93
3160201	9555	HORSE CR	117.2	19.1	7 - 40	0.0	0.0 - 0.0	3.7	1.3 - 7.1	0.9	0.3 - 2.3	3.8	1.2 - 10.2	0.0	0.0 - 0.0	10.8	3.6 - 24.6	0.93
3160201	9556	HORSE CR	151.3	19.5	7 - 33	0.0	0.0 - 0.0	4.7	1.4 - 8.7	1.2	0.4 - 2.6	5.5	1.8 - 11.5	0.0	0.0 - 0.0	8.1	3.0 - 15.8	0.87
3160201	9557	SWEETWATER CR	99.7	16.6	6 - 44	0.0	0.0 - 0.0	4.3	1.5 - 11.4	0.7	0.2 - 2.3	3.3	1.3 - 9.9	0.0	0.0 - 0.0	8.2	3.1 - 24.8	0.87
3160201	9558	TOMBIGBEE R	54.7	39.0	13 - 71	0.0	0.0 - 0.0	4.6	1.3 - 9.7	3.6	0.9 - 7.8	16.0	4.4 - 35.1	0.0	0.0 - 0.0	14.9	4.7 - 29.6	0.93
3160201	9559	TOMBIGBEE R	73.7	24.1	7 - 60	0.0	0.0 - 0.0	3.6	1.0 - 8.3	1.0	0.3 - 2.5	4.5	1.4 - 13.1	0.0	0.0 - 0.0	14.9	4.3 - 40.1	0.93
3160201	9560	BEAVER CR	249.2	21.8	7 - 44	0.0	0.0 - 0.0	2.8	0.9 - 5.6	1.0	0.3 - 2.3	4.4	1.4 - 12.7	0.0	0.0 - 0.0	13.6	4.6 - 31.0	0.92
3160201	9561	TOMBIGBEE R	56.4	55.4	16 - 115	0.0	0.0 - 0.0	11.1	3.2 - 25.1	1.0	0.3 - 2.8	4.2	1.2 - 10.7	0.0	0.0 - 0.0	39.1	10.8 - 93.6	0.92
3160201	9562	TOMBIGBEE R	64.9	48.2	16 - 135	0.0	0.0 - 0.0	1.8	0.6 - 4.5	0.7	0.2 - 2.3	3.5	1.2 - 10.6	0.0	0.0 - 0.0	42.2	14.5 - 123.0	0.92
3160201	9563	CHICKASAW BOGU	135.3	62.7	23 - 134	0.0	0.0 - 0.0	5.1	1.8 - 10.9	3.2	1.1 - 9.2	14.4	4.5 - 36.3	0.0	0.0 - 0.0	40.0	13.9 - 94.5	0.92
3160201	9564	CHICKASAW BOGU	55.1	74.2	23 - 164	0.0	0.0 - 0.0	9.0	2.8 - 17.9	2.3	0.6 - 7.3	10.3	2.9 - 25.1	0.0	0.0 - 0.0	52.7	15.3 - 124.0	0.90
3160201	9565	ATKINS CR	112.4	48.4	17 - 120	0.0	0.0 - 0.0	2.7	0.8 - 6.9	3.2	0.8 - 10.0	14.3	4.2 - 42.2	0.0	0.0 - 0.0	28.2	9.6 - 85.1	0.88
3160201	9566	CHICKASAW BOGU	1.1	144.1	42 - 295	0.0	0.0 - 0.0	0.0	0.0 - 0.0	13.0	3.3 - 34.3	57.3	16.9 - 136.3	0.0	0.0 - 0.0	73.9	20.6 - 189.5	0.88
3160201	9567	CHICKASAW BOGU	6.7	101.3	30 - 311	0.0	0.0 - 0.0	1.2	0.4 - 2.9	5.3	1.4 - 17.9	21.6	7.6 - 58.6	0.0	0.0 - 0.0	73.3	21.1 - 209.7	0.88
3160201	9568	CHICKASAW BOGU	10.3	57.9	24 - 117	0.0	0.0 - 0.0	3.5	1.3 - 7.3	1.5	0.5 - 4.1	7.8	2.8 - 18.6	0.0	0.0 - 0.0	45.0	18.5 - 86.9	0.86
3160201	9569	CHICKASAW BOGU	180.3	42.9	14 - 101	0.0	0.0 - 0.0	3.0	1.0 - 7.9	2.5	0.7 - 7.3	11.2	3.7 - 33.3	0.0	0.0 - 0.0	26.2	8.2 - 65.0	0.84
3160201	9570	DRY CR	93.4	109.4	38 - 291	0.0	0.0 - 0.0	3.4	1.0 - 9.4	11.3	2.8 - 39.3	50.9	16.9 - 165.2	0.0	0.0 - 0.0	43.7	13.5 - 125.4	0.84
3160201	9571	LITTLE CRY CR	64.5	135.7	44 - 279	0.0	0.0 - 0.0	3.0	1.0 - 7.6	14.5	4.1 - 40.4	65.0	20.4 - 189.7	0.0	0.0 - 0.0	53.1	18.3 - 131.8	0.86
3160201	9572	POWELL CR	176.2	133.4	49 - 250	0.0	0.0 - 0.0	3.7	1.1 - 7.1	14.2	3.9 - 49.4	63.6	22.0 - 140.6	0.0	0.0 - 0.0	52.0	18.8 - 132.0	0.88
3160201	9573	BARTON CR	52.4	57.5	16 - 124	0.0	0.0 - 0.0	3.2	0.9 - 6.4	2.1	0.5 - 6.2	9.4	2.7 - 21.7	0.0	0.0 - 0.0	42.7	11.2 - 102.7	0.90

8-digit HUC	ID	Name	Area	Catchment Yield		Point sources		Developed Land		Manure		Agricultural Land		Phosphate Mines		Soil parent rock		Frac
				kg/km^2	90% CI	kg/km^2	90% CI	kg/km^2	90% CI	kg/km^2	90% CI	kg/km^2	90% CI	kg/km^2	90% CI	kg/km^2	90% CI	
3160201	9574	TOMBIGBEE R	80.0	70.1	22 - 168	0.0	0.0 - 0.0	3.3	0.9 - 7.7	3.1	0.9 - 10.0	13.7	4.4 - 43.7	0.0	0.0 - 0.0	50.0	15.3 - 132.9	0.92
3160201	9575	TOMBIGBEE R	212.5	87.4	31 - 192	0.0	0.0 - 0.0	4.8	1.6 - 11.6	5.5	1.5 - 16.9	24.8	7.9 - 66.1	0.0	0.0 - 0.0	52.4	18.9 - 131.6	0.92
3160201	9576	TOMBIGBEE R	225.9	104.3	27 - 265	34.2	8.9 - 85.4	9.5	2.6 - 23.4	4.0	1.0 - 10.5	18.4	5.0 - 52.6	0.0	0.0 - 0.0	38.2	9.5 - 105.3	0.92
3160201	9577	COTOHAGA CR	82.7	24.3	9 - 62	0.0	0.0 - 0.0	3.6	1.1 - 8.9	0.3	0.1 - 0.8	1.2	0.3 - 3.4	0.0	0.0 - 0.0	19.3	7.0 - 49.8	0.92
3160201	9578	KINTERBISH CR	360.6	15.3	5 - 34	0.0	0.0 - 0.0	3.9	1.1 - 8.5	0.4	0.1 - 1.2	1.9	0.5 - 4.6	0.0	0.0 - 0.0	9.1	2.9 - 21.1	0.92
3160201	9579	TUCKABUM CR	100.4	22.3	8 - 42	0.0	0.0 - 0.0	4.9	1.6 - 10.4	0.8	0.3 - 2.1	3.6	1.1 - 9.1	0.0	0.0 - 0.0	13.0	4.8 - 28.1	0.93
3160201	9580	VAUGHAN CR	65.6	13.8	4 - 36	0.0	0.0 - 0.0	4.1	1.3 - 9.9	0.4	0.1 - 1.0	1.6	0.5 - 4.4	0.0	0.0 - 0.0	7.8	2.4 - 20.2	0.93
3160201	9581	MELON CR	27.6	13.7	5 - 25	0.0	0.0 - 0.0	4.9	1.5 - 9.9	0.4	0.1 - 0.9	1.6	0.6 - 4.3	0.0	0.0 - 0.0	6.8	2.3 - 14.7	0.93
3160201	9582	WAHALAK CR	188.2	15.3	5 - 36	0.0	0.0 - 0.0	5.5	1.6 - 14.1	0.5	0.1 - 1.2	2.0	0.6 - 5.0	0.0	0.0 - 0.0	7.3	2.4 - 17.1	0.93
3160201	9583	TALLAWAMPA CR	124.2	10.6	3 - 23	0.0	0.0 - 0.0	3.5	1.0 - 8.0	0.2	0.0 - 0.5	0.9	0.3 - 2.4	0.0	0.0 - 0.0	6.0	2.1 - 12.5	0.93
3160201	9584	BOGUELOOSA CR	11.1	35.0	14 - 83	0.0	0.0 - 0.0	2.9	0.9 - 6.1	0.1	0.0 - 0.4	1.1	0.4 - 2.9	0.0	0.0 - 0.0	30.9	11.9 - 80.2	0.93
3160201	9585	SURVEYOURS CR	84.8	13.7	5 - 28	0.0	0.0 - 0.0	4.8	1.9 - 11.0	0.5	0.2 - 1.7	2.3	0.8 - 6.6	0.0	0.0 - 0.0	6.0	2.2 - 14.2	0.92
3160201	9587	BOGUELOOSA CR	54.0	24.4	10 - 65	0.0	0.0 - 0.0	6.9	2.4 - 17.1	1.8	0.6 - 5.6	8.1	3.0 - 21.8	0.0	0.0 - 0.0	7.7	2.9 - 20.2	0.92
3160201	9588	BOGUELOOSA CR	151.8	14.4	6 - 25	0.0	0.0 - 0.0	5.4	1.9 - 11.2	0.5	0.2 - 1.4	2.6	1.0 - 6.1	0.0	0.0 - 0.0	5.9	2.3 - 13.7	0.90
3160201	9589	PUSS CUSS CR	42.2	18.9	6 - 37	0.0	0.0 - 0.0	6.0	2.0 - 12.2	1.2	0.4 - 3.7	5.2	1.7 - 13.7	0.0	0.0 - 0.0	6.5	2.2 - 15.0	0.90
3160201	9590	OKATUPPA CR	214.9	10.2	4 - 23	0.0	0.0 - 0.0	2.7	0.9 - 6.4	0.4	0.1 - 1.1	1.6	0.5 - 4.8	0.0	0.0 - 0.0	5.5	1.9 - 13.1	0.87
3160201	9591	PUSS CUSS CR	140.5	19.3	5 - 47	0.0	0.0 - 0.0	3.7	1.0 - 9.6	1.3	0.3 - 4.5	5.7	1.5 - 17.1	0.0	0.0 - 0.0	8.6	2.1 - 26.1	0.87
3160201	9592	SOUWILPA CR	108.4	14.8	6 - 28	0.0	0.0 - 0.0	5.9	2.2 - 11.8	0.6	0.2 - 1.5	2.6	0.9 - 5.6	0.0	0.0 - 0.0	5.8	2.2 - 14.0	0.92
3160201	9593	TURKEY CR	142.5	14.0	5 - 25	0.0	0.0 - 0.0	3.6	1.2 - 6.6	0.5	0.2 - 1.2	2.4	0.8 - 5.4	0.0	0.0 - 0.0	7.4	2.6 - 14.9	0.94
3160201	9594	YANTLEY CR	229.2	14.6	6 - 27	0.0	0.0 - 0.0	3.8	1.2 - 7.0	0.7	0.2 - 1.6	3.0	1.1 - 5.9	0.0	0.0 - 0.0	7.1	2.7 - 16.1	0.90
3160201	9595	TUCKABUM CR	43.1	14.2	4 - 28	0.0	0.0 - 0.0	3.2	1.0 - 6.5	0.4	0.1 - 1.2	1.7	0.6 - 4.0	0.0	0.0 - 0.0	8.8	2.6 - 20.0	0.90
3160201	9596	TUCKABUM CR	103.6	16.7	6 - 34	0.0	0.0 - 0.0	5.6	1.7 - 12.8	0.7	0.2 - 2.3	3.2	1.1 - 7.5	0.0	0.0 - 0.0	7.1	2.3 - 16.5	0.85
3160201	9597	BOGUELICHITTO C	168.3	14.1	5 - 27	0.0	0.0 - 0.0	4.1	1.5 - 8.6	0.5	0.2 - 1.3	2.4	0.8 - 5.9	0.0	0.0 - 0.0	7.1	2.7 - 17.6	0.85
3160202	9598	SUCARNOCHEE R	107.5	54.4	19 - 108	0.0	0.0 - 0.0	5.6	2.2 - 12.5	1.7	0.5 - 4.2	7.8	2.7 - 19.7	0.0	0.0 - 0.0	39.3	13.6 - 94.4	0.92
3160202	9599	*A	66.8	69.4	24 - 140	0.0	0.0 - 0.0	4.5	1.6 - 9.0	4.0	1.1 - 14.4	18.7	5.5 - 45.9	0.0	0.0 - 0.0	42.2	13.9 - 103.9	0.91
3160202	9600	SUCARNOCHEE R	114.0	50.9	18 - 152	0.0	0.0 - 0.0	5.7	1.7 - 17.5	1.0	0.3 - 3.0	4.3	1.4 - 13.6	0.0	0.0 - 0.0	39.9	13.8 - 122.5	0.91
3160202	9601	SUCARNOCHEE R	388.6	97.5	29 - 249	0.0	0.0 - 0.0	5.3	1.5 - 11.6	7.2	2.0 - 21.6	32.3	10.1 - 93.7	0.0	0.0 - 0.0	52.7	15.5 - 130.8	0.89
3160202	9602	SUCARNOCHEE R	62.6	84.7	29 - 176	0.0	0.0 - 0.0	4.5	1.6 - 8.8	5.9	1.8 - 18.0	24.1	7.9 - 59.6	0.0	0.0 - 0.0	50.2	17.4 - 108.6	0.84
3160202	9603	SUCARNOCHEE R	23.3	98.2	31 - 187	0.0	0.0 - 0.0	7.6	2.5 - 16.2	4.9	1.4 - 13.4	19.1	5.8 - 43.6	0.0	0.0 - 0.0	66.7	21.7 - 156.2	0.82
3160202	9604	STRAIGHT CR	52.4	25.7	10 - 50	0.0	0.0 - 0.0	4.6	1.6 - 10.2	1.2	0.4 - 3.3	4.5	1.6 - 11.5	0.0	0.0 - 0.0	15.5	5.2 - 34.2	0.76
3160202	9605	SUCARNOCHEE R	35.5	24.6	8 - 52	0.0	0.0 - 0.0	7.8	2.4 - 16.3	0.8	0.3 - 2.1	3.1	1.1 - 6.7	0.0	0.0 - 0.0	12.9	4.2 - 31.3	0.76
3160202	9606	SUCARNOCHEE R	68.7	24.6	10 - 60	0.0	0.0 - 0.0	4.4	1.6 - 9.7	2.1	0.8 - 5.2	8.3	2.8 - 24.5	0.0	0.0 - 0.0	9.8	3.7 - 27.2	0.71
3160202	9607	*B	99.2	28.0	8 - 52	0.0	0.0 - 0.0	5.6	1.9 - 12.2	2.0	0.6 - 5.6	8.1	2.8 - 18.2	0.0	0.0 - 0.0	12.3	3.5 - 25.7	0.71
3160202	9608	BLACKWATER CR	4.4	98.8	33 - 211	0.0	0.0 - 0.0	10.2	3.1 - 22.4	3.7	1.1 - 10.8	17.6	5.3 - 40.6	0.0	0.0 - 0.0	67.3	20.9 - 155.3	0.82
3160202	9609	PAWTICFAW CR	59.6	36.4	15 - 88	0.0	0.0 - 0.0	5.4	2.2 - 12.2	1.4	0.5 - 5.0	5.7	2.0 - 16.7	0.0	0.0 - 0.0	23.9	9.7 - 67.5	0.81
3160202	9610	PARKER CR	30.8	21.2	7 - 40	0.0	0.0 - 0.0	5.8	1.7 - 12.7	0.9	0.2 - 2.2	3.7	1.2 - 9.0	0.0	0.0 - 0.0	10.8	3.7 - 20.6	0.76
3160202	9611	PAWTICFAW CR	165.3	28.7	10 - 52	0.0	0.0 - 0.0	6.2	1.9 - 13.3	2.4	0.7 - 6.8	9.7	3.1 - 22.7	0.0	0.0 - 0.0	10.4	3.4 - 25.2	0.76
3160202	9612	BLACKWATER CR	147.0	25.5	9 - 45	0.0	0.0 - 0.0	6.1	2.1 - 11.4	1.5	0.5 - 3.5	6.0	2.0 - 13.9	0.0	0.0 - 0.0	12.0	4.1 - 27.1	0.81
3160202	9613	PONTA CR	114.3	31.4	12 - 84	0.0	0.0 - 0.0	3.8	1.3 - 8.5	1.8	0.6 - 6.4	7.5	2.8 - 24.0	0.0	0.0 - 0.0	18.3	7.2 - 50.6	0.84

8-digit HUC	ID	Name	Area	Catchment Yield		Point sources		Developed Land		Manure		Agricultural Land		Phosphate Mines		Soil parent rock		Frac
				kg/km^2	90% CI	kg/km^2	90% CI	kg/km^2	90% CI	kg/km^2	90% CI	kg/km^2	90% CI	kg/km^2	90% CI	kg/km^2	90% CI	
3160202	9614	BIG REED CR	60.0	28.0	9 - 57	0.0	0.0 - 0.0	10.5	3.1 - 21.3	1.6	0.4 - 4.5	6.1	1.9 - 15.8	0.0	0.0 - 0.0	9.9	3.6 - 21.0	0.79
3160202	9615	PONTA CR	242.9	29.3	11 - 68	0.0	0.0 - 0.0	9.4	3.5 - 22.3	1.5	0.5 - 4.7	5.9	2.0 - 14.0	0.0	0.0 - 0.0	12.4	5.1 - 31.8	0.79
3160202	9616	ALAMUCHEE CR	147.8	49.6	18 - 99	2.0	0.7 - 4.6	7.7	2.5 - 16.8	1.5	0.5 - 4.5	6.8	2.2 - 16.8	0.0	0.0 - 0.0	31.7	11.2 - 74.1	0.89
3160202	9617	TOOMSUBA CR	223.0	22.2	9 - 52	0.0	0.0 - 0.0	8.0	2.9 - 19.6	0.8	0.3 - 2.3	3.4	1.3 - 8.8	0.0	0.0 - 0.0	10.0	3.6 - 23.9	0.85
3160202	9618	ALAMUCHEE CR	209.9	19.4	7 - 43	0.0	0.0 - 0.0	6.7	2.2 - 14.4	0.8	0.2 - 2.5	3.1	1.1 - 7.5	0.0	0.0 - 0.0	8.9	3.4 - 21.2	0.85
3160203	9619	TOMBIGBEE R	15.4	26.9	9 - 70	0.0	0.0 - 0.0	0.2	0.1 - 0.6	0.2	0.0 - 0.5	1.0	0.3 - 3.2	0.0	0.0 - 0.0	25.5	9.1 - 67.1	0.99
3160203	9620	TOMBIGBEE R	57.4	37.1	14 - 77	0.0	0.0 - 0.0	3.0	1.1 - 6.2	1.7	0.6 - 4.8	4.7	1.7 - 10.9	0.0	0.0 - 0.0	27.7	10.5 - 62.2	0.99
3160203	9621	TOMBIGBEE R	3.7	28.4	9 - 81	0.0	0.0 - 0.0	0.0	0.0 - 0.0	0.0	0.0 - 0.0	0.0	0.0 - 0.0	0.0	0.0 - 0.0	28.4	9.4 - 80.7	0.99
3160203	9622	*A	114.6	23.4	7 - 56	0.0	0.0 - 0.0	1.7	0.5 - 3.7	0.3	0.1 - 0.9	1.3	0.5 - 3.6	0.0	0.0 - 0.0	20.1	5.8 - 51.6	0.99
3160203	9623	TOMBIGBEE R	24.6	176.4	60 - 474	133.3	45.0 - 370.9	7.0	2.3 - 18.6	2.4	0.6 - 7.7	4.8	1.6 - 14.3	0.0	0.0 - 0.0	29.0	9.7 - 86.6	0.99
3160203	9624	TOMBIGBEE R	115.7	20.1	7 - 50	0.0	0.0 - 0.0	2.0	0.6 - 4.6	0.5	0.2 - 1.9	2.1	0.7 - 6.4	0.0	0.0 - 0.0	15.5	5.0 - 41.2	0.99
3160203	9625	TOMBIGBEE R	56.4	10.6	4 - 31	0.0	0.0 - 0.0	3.4	1.1 - 9.7	0.3	0.1 - 1.1	1.4	0.6 - 4.7	0.0	0.0 - 0.0	5.5	2.3 - 15.8	0.98
3160203	9626	BASSETT CR	61.5	249.6	77 - 589	224.3	69.9 - 553.8	18.1	5.2 - 48.5	0.2	0.1 - 0.7	1.3	0.4 - 3.9	0.0	0.0 - 0.0	5.6	1.7 - 13.3	0.98
3160203	9627	BASSETT CR	219.6	12.6	3 - 29	0.0	0.0 - 0.0	4.5	1.2 - 9.3	0.6	0.1 - 1.6	3.3	0.8 - 9.1	0.0	0.0 - 0.0	4.1	1.1 - 11.3	0.96
3160203	9628	BASSETT CR	58.5	12.1	4 - 26	0.0	0.0 - 0.0	1.5	0.5 - 2.9	0.3	0.1 - 0.7	1.3	0.4 - 3.4	0.0	0.0 - 0.0	8.9	2.7 - 22.5	0.90
3160203	9629	LITTLE BASSETT (	87.4	11.3	4 - 23	0.0	0.0 - 0.0	4.1	1.5 - 8.7	0.3	0.1 - 0.9	1.9	0.6 - 5.5	0.0	0.0 - 0.0	5.0	1.9 - 10.6	0.86
3160203	9630	BASSETT CR	145.2	26.6	10 - 49	4.1	1.4 - 8.8	12.7	4.5 - 24.4	0.5	0.2 - 1.5	2.9	0.9 - 6.5	0.0	0.0 - 0.0	6.3	2.0 - 12.6	0.86
3160203	9631	*B	28.6	23.3	7 - 61	0.0	0.0 - 0.0	15.6	4.9 - 42.6	0.7	0.2 - 2.5	3.7	0.9 - 13.2	0.0	0.0 - 0.0	3.2	1.0 - 9.3	0.90
3160203	9632	RABBIT CR	84.1	15.4	6 - 36	0.0	0.0 - 0.0	6.6	2.3 - 15.9	0.8	0.3 - 2.0	4.1	1.5 - 11.6	0.0	0.0 - 0.0	3.9	1.6 - 9.1	0.96
3160203	9633	TOMBIGBEE R	97.1	25.8	9 - 57	0.0	0.0 - 0.0	11.8	4.1 - 26.1	1.3	0.4 - 3.2	5.0	1.7 - 12.4	0.0	0.0 - 0.0	7.8	2.9 - 20.5	0.98
3160203	9634	JACKSON CR	30.5	18.8	6 - 36	0.0	0.0 - 0.0	3.8	1.3 - 8.0	0.5	0.1 - 1.3	2.8	0.9 - 6.7	0.0	0.0 - 0.0	11.7	4.1 - 27.0	0.98
3160203	9635	JACKSON CR	153.7	12.2	4 - 28	0.0	0.0 - 0.0	5.7	1.8 - 13.5	0.4	0.1 - 1.3	2.3	0.8 - 5.8	0.0	0.0 - 0.0	3.7	1.4 - 8.6	0.94
3160203	9636	TATTILABA	106.4	8.5	3 - 23	0.0	0.0 - 0.0	2.6	0.9 - 7.4	0.2	0.1 - 0.6	1.0	0.3 - 3.1	0.0	0.0 - 0.0	4.7	1.7 - 13.4	0.94
3160203	9637	TOMBIGBEE R	17.9	17.0	6 - 45	0.0	0.0 - 0.0	3.9	1.3 - 10.0	1.4	0.4 - 5.1	3.6	1.2 - 10.0	0.0	0.0 - 0.0	8.2	2.8 - 25.9	0.98
3160203	9638	TOMBIGBEE R	101.4	20.9	8 - 43	0.0	0.0 - 0.0	2.1	0.7 - 3.9	1.0	0.3 - 3.1	3.7	1.4 - 8.8	0.0	0.0 - 0.0	14.1	5.2 - 33.3	0.98
3160203	9639	SATILPA CR	5.0	22.3	9 - 54	0.0	0.0 - 0.0	1.3	0.5 - 3.5	0.1	0.0 - 0.3	0.7	0.3 - 2.3	0.0	0.0 - 0.0	20.1	8.0 - 49.5	0.98
3160203	9640	SATILPA CR	74.3	14.2	4 - 31	0.0	0.0 - 0.0	3.7	1.0 - 7.6	0.4	0.1 - 1.2	2.3	0.7 - 5.7	0.0	0.0 - 0.0	7.7	2.4 - 19.2	0.97
3160203	9641	SATILPA CR	9.4	13.6	5 - 24	0.0	0.0 - 0.0	5.1	1.7 - 10.6	0.5	0.1 - 1.3	2.6	0.8 - 6.0	0.0	0.0 - 0.0	5.4	1.7 - 10.7	0.93
3160203	9642	SATILPA CR	85.7	8.4	3 - 20	0.0	0.0 - 0.0	2.0	0.6 - 4.9	0.2	0.0 - 0.4	0.8	0.2 - 2.1	0.0	0.0 - 0.0	5.4	2.0 - 11.7	0.91
3160203	9643	*C	39.7	10.5	3 - 19	0.0	0.0 - 0.0	4.4	1.3 - 9.0	0.1	0.0 - 0.3	0.4	0.1 - 1.0	0.0	0.0 - 0.0	5.6	1.6 - 11.6	0.83
3160203	9644	SATILPA CR	75.4	8.6	3 - 19	0.0	0.0 - 0.0	2.3	0.7 - 5.6	0.2	0.1 - 0.6	1.2	0.4 - 3.2	0.0	0.0 - 0.0	4.9	1.7 - 14.7	0.83
3160203	9645	*D	107.9	7.3	3 - 16	0.0	0.0 - 0.0	2.3	0.8 - 4.7	0.1	0.0 - 0.4	0.7	0.2 - 1.8	0.0	0.0 - 0.0	4.3	1.8 - 10.8	0.91
3160203	9646	HARRIS CR	78.2	9.6	3 - 19	0.0	0.0 - 0.0	2.6	0.9 - 5.1	0.4	0.1 - 0.9	1.9	0.7 - 4.8	0.0	0.0 - 0.0	4.7	1.8 - 10.7	0.93
3160203	9647	*E	30.2	15.6	5 - 39	0.0	0.0 - 0.0	6.7	1.8 - 17.0	0.8	0.2 - 2.3	4.1	1.1 - 10.0	0.0	0.0 - 0.0	3.9	1.1 - 9.4	0.97
3160203	9648	TOMBIGBEE R	14.1	28.2	10 - 65	0.0	0.0 - 0.0	1.0	0.4 - 2.4	0.3	0.1 - 0.8	1.8	0.7 - 4.4	0.0	0.0 - 0.0	25.1	8.8 - 63.1	0.98
3160203	9649	TOMBIGBEE R	9.7	34.7	13 - 85	0.0	0.0 - 0.0	11.3	4.2 - 27.2	1.8	0.6 - 5.8	8.7	2.9 - 23.9	0.0	0.0 - 0.0	12.9	4.9 - 34.4	0.98
3160203	9650	ESTIS MILL CR	86.8	12.3	4 - 29	0.0	0.0 - 0.0	4.9	1.6 - 10.9	0.5	0.1 - 1.4	2.7	0.9 - 8.2	0.0	0.0 - 0.0	4.2	1.4 - 10.9	0.94
3160203	9651	TOMBIGBEE R	3.6	26.1	7 - 59	0.0	0.0 - 0.0	4.8	1.3 - 9.3	0.5	0.1 - 1.5	1.7	0.5 - 3.9	0.0	0.0 - 0.0	19.1	5.3 - 50.0	0.94
3160203	9652	SEYOYOH CR	60.9	15.4	5 - 31	0.0	0.0 - 0.0	2.2	0.7 - 4.5	0.3	0.1 - 0.7	1.2	0.4 - 3.1	0.0	0.0 - 0.0	11.7	4.1 - 24.7	0.98

8-digit HUC	ID	Name	Area	Catchment Yield kg/km^2	Catchment Yield 90% CI	Point sources kg/km^2	Point sources 90% CI	Developed Land kg/km^2	Developed Land 90% CI	Manure kg/km^2	Manure 90% CI	Agricultural Land kg/km^2	Agricultural Land 90% CI	Phosphate Mines kg/km^2	Phosphate Mines 90% CI	Soil parent rock kg/km^2	Soil parent rock 90% CI	Frac
3160203	9653	SANTA BOGUE CR	83.4	13.8	6 - 36	0.0	0.0 - 0.0	1.7	0.5 - 4.1	1.0	0.3 - 2.9	2.7	0.9 - 7.7	0.0	0.0 - 0.0	8.4	3.2 - 23.0	0.98
3160203	9654	SANTA BOGUE CR	21.3	16.0	5 - 44	0.0	0.0 - 0.0	3.3	1.1 - 8.9	1.3	0.4 - 3.8	3.0	0.9 - 9.5	0.0	0.0 - 0.0	8.4	2.7 - 24.2	0.94
3160203	9655	SANTA BOGUE CR	20.0	17.3	6 - 40	0.0	0.0 - 0.0	2.7	0.9 - 5.9	2.1	0.7 - 5.8	5.1	1.6 - 13.7	0.0	0.0 - 0.0	7.4	3.1 - 17.6	0.92
3160203	9656	TAYLORS CR, N PI	12.1	19.0	5 - 35	0.0	0.0 - 0.0	2.2	0.6 - 4.2	2.8	0.6 - 6.6	7.2	2.1 - 16.9	0.0	0.0 - 0.0	6.8	2.0 - 14.1	0.92
3160203	9657	DUNBAR CR	4.3	8.8	2 - 22	0.0	0.0 - 0.0	0.0	0.0 - 0.0	0.4	0.1 - 1.4	1.1	0.3 - 2.6	0.0	0.0 - 0.0	7.3	2.0 - 17.9	0.89
3160203	9658	DUNBAR CR	57.8	20.6	8 - 48	0.0	0.0 - 0.0	4.6	1.7 - 9.5	2.3	0.7 - 5.9	6.8	2.4 - 17.9	0.0	0.0 - 0.0	7.0	2.6 - 18.7	0.87
3160203	9659	MILL CR	74.4	22.7	8 - 55	0.0	0.0 - 0.0	6.2	1.9 - 14.4	3.0	0.9 - 8.2	7.6	2.6 - 21.0	0.0	0.0 - 0.0	6.0	2.2 - 16.5	0.87
3160203	9660	TAYLORS CR, N PI	135.5	15.3	5 - 44	0.0	0.0 - 0.0	3.7	1.1 - 8.5	1.4	0.4 - 5.1	3.4	1.0 - 9.2	0.0	0.0 - 0.0	6.9	2.2 - 22.1	0.89
3160203	9661	PINE BARREN CR	71.5	13.0	4 - 32	0.0	0.0 - 0.0	3.1	0.8 - 7.0	1.1	0.3 - 3.1	2.3	0.7 - 7.3	0.0	0.0 - 0.0	6.5	2.2 - 15.5	0.94
3160203	9662	TAULER CR	13.3	17.1	5 - 37	0.0	0.0 - 0.0	1.8	0.6 - 3.6	0.3	0.1 - 0.9	0.8	0.3 - 2.1	0.0	0.0 - 0.0	14.2	3.8 - 33.5	0.98
3160203	9663	ELIAS CR	43.2	19.0	7 - 35	0.0	0.0 - 0.0	4.5	1.4 - 10.2	2.2	0.7 - 6.9	5.3	1.8 - 11.7	0.0	0.0 - 0.0	7.0	2.5 - 15.1	0.93
3160203	9664	TAULER CR	80.1	17.4	7 - 44	0.0	0.0 - 0.0	3.1	1.1 - 7.6	1.9	0.6 - 5.5	4.6	1.8 - 15.0	0.0	0.0 - 0.0	7.7	3.1 - 24.7	0.93
3160203	9665	BASSETTS CR	68.9	39.7	11 - 79	0.0	0.0 - 0.0	4.9	1.4 - 9.6	4.9	1.2 - 13.5	11.8	3.2 - 25.6	0.0	0.0 - 0.0	18.3	5.6 - 40.4	0.98
3160203	9666	BASSETTS CR	409.9	25.4	10 - 52	0.0	0.0 - 0.0	6.6	2.5 - 15.6	2.9	1.0 - 7.5	7.1	2.8 - 19.6	0.0	0.0 - 0.0	8.8	3.4 - 18.2	0.96
3160203	9667	ARMSTRONG CR	87.2	19.4	7 - 38	0.0	0.0 - 0.0	5.6	2.0 - 12.0	1.5	0.4 - 3.8	3.6	1.2 - 9.6	0.0	0.0 - 0.0	8.7	3.4 - 18.3	0.96
3160203	9668	LEWIS CR	231.7	26.7	11 - 57	0.0	0.0 - 0.0	6.5	2.6 - 15.4	2.1	0.8 - 5.9	5.0	2.0 - 12.5	0.0	0.0 - 0.0	13.1	5.5 - 31.7	0.99
3160203	9669	BILBO CR	3.6	28.3	9 - 76	0.0	0.0 - 0.0	0.8	0.3 - 1.5	0.0	0.0 - 0.0	0.0	0.0 - 0.0	0.0	0.0 - 0.0	27.5	8.8 - 77.0	0.99
3160203	9670	BILBO CR	209.8	28.9	9 - 71	0.0	0.0 - 0.0	8.9	2.8 - 20.9	2.7	0.9 - 7.5	6.4	1.9 - 18.5	0.0	0.0 - 0.0	10.9	3.3 - 28.3	0.98
3160203	9671	BATES CR	220.9	26.3	10 - 52	0.0	0.0 - 0.0	6.3	2.4 - 14.5	2.5	0.9 - 6.5	6.1	2.6 - 17.0	0.0	0.0 - 0.0	11.3	4.4 - 25.7	0.98
3160203	9672	*F	86.5	31.5	11 - 87	0.0	0.0 - 0.0	7.9	2.6 - 20.5	3.0	1.0 - 8.4	8.5	2.4 - 24.3	0.0	0.0 - 0.0	12.1	4.7 - 35.1	0.99
3160204	9674	TENSAW R	9.7	11.9	3 - 31	0.0	0.0 - 0.0	5.6	1.4 - 14.8	0.0	0.0 - 0.0	0.0	0.0 - 0.0	0.0	0.0 - 0.0	6.4	2.0 - 17.9	1.00
3160204	9675	BAY MINETTE CR	17.5	30.3	10 - 61	0.0	0.0 - 0.0	20.7	6.6 - 44.3	0.1	0.0 - 0.2	0.4	0.1 - 1.2	0.0	0.0 - 0.0	9.2	3.2 - 21.4	0.98
3160204	9676	SHILBY CR	31.4	20.8	8 - 44	0.0	0.0 - 0.0	7.2	2.4 - 15.6	0.4	0.1 - 1.5	2.2	0.8 - 7.5	0.0	0.0 - 0.0	11.0	4.0 - 25.2	0.93
3160204	9677	BAY MINETTE CR	140.0	25.8	10 - 57	0.0	0.0 - 0.0	11.9	3.9 - 26.1	0.6	0.2 - 1.4	3.6	1.3 - 8.1	0.0	0.0 - 0.0	9.7	3.7 - 26.2	0.93
3160204	9678	TENSAW R	199.6	16.6	7 - 31	0.0	0.0 - 0.0	3.5	1.2 - 6.5	0.3	0.1 - 0.8	1.9	0.6 - 5.2	0.0	0.0 - 0.0	10.9	4.4 - 21.3	0.98
3160204	9679	TENSAW R	304.6	15.9	5 - 39	0.0	0.0 - 0.0	2.9	0.9 - 6.7	0.5	0.1 - 1.2	2.5	0.7 - 7.0	0.0	0.0 - 0.0	10.0	3.0 - 26.4	0.90
3160204	9680	MIDDLE R	80.1	5.7	2 - 17	0.0	0.0 - 0.0	0.2	0.1 - 0.6	0.0	0.0 - 0.0	0.0	0.0 - 0.0	0.0	0.0 - 0.0	5.5	2.2 - 16.9	0.90
3160204	9681	MOBILE R	83.3	27.3	7 - 50	0.0	0.0 - 0.0	6.1	1.5 - 12.9	1.2	0.3 - 2.9	7.6	1.9 - 18.7	0.0	0.0 - 0.0	12.4	3.4 - 25.4	0.99
3160204	9682	MOBILE R	10.0	21.2	5 - 53	0.0	0.0 - 0.0	6.8	1.7 - 19.4	0.7	0.2 - 2.4	4.2	0.9 - 13.9	0.0	0.0 - 0.0	9.4	2.4 - 25.5	1.00
3160204	9683	MOBILE R	2.9	12.6	3 - 38	0.0	0.0 - 0.0	0.7	0.1 - 2.8	1.1	0.2 - 4.5	6.1	1.0 - 21.4	0.0	0.0 - 0.0	4.7	1.0 - 14.9	1.00
3160204	9684	MOBILE R	22.2	18.9	7 - 39	0.0	0.0 - 0.0	0.0	0.0 - 0.0	0.1	0.0 - 0.3	0.6	0.2 - 1.3	0.0	0.0 - 0.0	18.3	6.5 - 38.4	1.00
3160204	9685	MOBILE R	303.3	23.8	8 - 61	8.8	2.9 - 23.0	4.7	1.4 - 11.6	0.7	0.2 - 2.4	1.7	0.6 - 4.8	0.0	0.0 - 0.0	7.8	2.5 - 21.8	1.00
3160204	9686	MOBILE R	11.2	15.0	4 - 37	0.0	0.0 - 0.0	0.0	0.0 - 0.0	1.5	0.4 - 5.1	7.9	1.9 - 21.2	0.0	0.0 - 0.0	5.6	1.5 - 14.5	0.99
3160204	9687	CEDAR CR	59.7	22.6	7 - 54	0.0	0.0 - 0.0	6.3	1.9 - 14.7	0.7	0.2 - 2.3	3.9	0.9 - 9.8	0.0	0.0 - 0.0	11.7	3.6 - 30.9	0.99
3160204	9688	CEDAR CR	36.9	20.7	7 - 43	0.0	0.0 - 0.0	4.5	1.4 - 9.7	1.0	0.3 - 2.3	5.5	1.9 - 14.8	0.0	0.0 - 0.0	9.7	3.4 - 22.6	0.93
3160204	9689	CEDAR CR	64.6	28.0	10 - 59	0.0	0.0 - 0.0	7.9	2.6 - 17.9	1.7	0.6 - 4.5	7.0	2.2 - 19.7	0.0	0.0 - 0.0	11.4	3.9 - 25.7	0.89
3160204	9690	LITTLE CR	34.7	20.8	8 - 58	0.0	0.0 - 0.0	3.6	1.2 - 10.5	0.9	0.3 - 2.8	4.5	1.6 - 13.2	0.0	0.0 - 0.0	11.8	4.7 - 29.8	0.89
3160204	9691	BULL BK	39.5	18.3	6 - 34	0.0	0.0 - 0.0	3.3	1.0 - 6.4	0.8	0.3 - 1.8	2.2	0.7 - 4.7	0.0	0.0 - 0.0	12.1	4.1 - 24.6	0.93
3160204	9692	BAYOU SARA	198.1	41.7	16 - 72	7.2	2.9 - 13.6	17.0	6.0 - 32.0	1.4	0.5 - 3.8	3.5	1.3 - 6.9	0.0	0.0 - 0.0	12.5	4.7 - 25.9	1.00

8-digit HUC	ID	Name	Area	Catchment Yield		Point sources		Developed Land		Manure		Agricultural Land		Phosphate Mines		Soil parent rock		Frac
				kg/km^2	90% CI	kg/km^2	90% CI	kg/km^2	90% CI	kg/km^2	90% CI	kg/km^2	90% CI	kg/km^2	90% CI	kg/km^2	90% CI	
3160204	9693	CHICKASAW CR	227.6	146.9	47 - 268	104.6	35.5 - 212.0	29.8	9.1 - 51.4	0.7	0.2 - 1.9	3.1	1.0 - 6.5	0.0	0.0 - 0.0	8.6	2.9 - 17.0	1.00
3160204	9694	CHICKASAW CR	229.3	18.5	6 - 47	0.0	0.0 - 0.1	5.6	1.7 - 15.2	0.8	0.2 - 2.9	2.1	0.6 - 6.3	0.0	0.0 - 0.0	10.0	3.2 - 26.2	0.94
3160204	9695	GALLOP CR	59.3	20.1	7 - 40	0.0	0.0 - 0.0	6.3	2.3 - 13.5	0.8	0.3 - 2.7	3.6	1.2 - 9.2	0.0	0.0 - 0.0	9.3	3.5 - 21.9	0.94
3160204	9696	THREEMILE CR	64.3	269.6	76 - 505	251.1	72.5 - 489.6	13.1	3.1 - 29.9	0.1	0.0 - 0.2	0.4	0.1 - 1.0	0.0	0.0 - 0.0	5.0	1.2 - 12.7	1.00
3160205	9697	MAGNOLIA R	85.6	99.3	36 - 235	0.0	0.0 - 0.0	22.6	7.7 - 51.1	6.9	2.4 - 25.2	55.7	19.0 - 159.8	0.0	0.0 - 0.0	14.1	5.4 - 37.0	1.00
3160205	9698	FISH R	19.9	83.5	27 - 176	0.0	0.0 - 0.0	19.2	5.6 - 38.4	5.9	1.6 - 15.1	45.8	13.8 - 116.0	0.0	0.0 - 0.0	12.6	3.9 - 28.9	1.00
3160205	9699	FISH R	64.9	82.6	27 - 192	0.0	0.0 - 0.0	28.2	9.2 - 62.9	4.8	1.2 - 13.0	35.1	11.9 - 97.6	0.0	0.0 - 0.0	14.5	4.7 - 41.4	1.00
3160205	9700	POLECAT CR	76.2	86.0	28 - 163	0.0	0.0 - 0.0	17.7	5.3 - 39.9	6.0	1.6 - 17.3	49.1	15.8 - 115.3	0.0	0.0 - 0.0	13.2	5.0 - 32.8	0.99
3160205	9701	FISH R	204.1	72.2	25 - 161	1.0	0.4 - 2.4	19.7	6.6 - 44.9	4.4	1.3 - 13.9	33.1	10.8 - 100.3	0.0	0.0 - 0.0	14.0	4.8 - 37.8	0.99
3160205	9702	FISH R, WATERHO	29.9	87.0	34 - 178	0.0	0.0 - 0.0	20.1	6.9 - 42.5	6.3	2.0 - 15.5	47.1	17.6 - 128.4	0.0	0.0 - 0.0	13.4	4.9 - 29.8	1.00
3160205	9703	DOG R	86.5	18.0	4 - 54	0.0	0.0 - 0.0	13.9	3.0 - 47.4	0.0	0.0 - 0.1	0.1	0.0 - 0.2	0.0	0.0 - 0.0	4.0	1.1 - 11.3	1.00
3160205	9704	HALLS MILL CR	83.0	90.7	33 - 198	0.0	0.0 - 0.0	77.4	27.2 - 169.4	0.5	0.1 - 1.4	2.6	1.0 - 7.1	0.0	0.0 - 0.0	10.2	3.4 - 21.1	1.00
3160205	9705	FOWL R	24.8	41.4	14 - 79	0.4	0.1 - 0.8	10.9	3.6 - 21.1	2.5	0.7 - 7.4	12.3	3.7 - 31.4	0.0	0.0 - 0.0	15.4	5.4 - 35.4	1.00
3160205	9706	*A	23.3	66.0	23 - 162	0.0	0.0 - 0.0	27.5	10.2 - 63.6	4.1	1.5 - 10.4	17.7	5.5 - 48.3	0.0	0.0 - 0.0	16.8	6.1 - 43.2	0.98
3160205	9707	FOWL R	81.9	58.8	20 - 106	0.0	0.0 - 0.0	21.4	6.5 - 40.6	4.1	1.3 - 9.2	19.7	5.9 - 46.9	0.0	0.0 - 0.0	13.6	4.6 - 28.9	0.98
3170001	9708	OKATIBBEE CR	123.5	22.6	7 - 48	0.0	0.0 - 0.0	8.0	2.6 - 16.0	1.2	0.4 - 3.2	4.4	1.2 - 11.5	0.0	0.0 - 0.0	9.0	3.1 - 21.5	0.86
3170001	9709	SOWASHEE CR	222.8	126.0	42 - 241	60.4	19.6 - 123.4	42.9	12.8 - 88.1	2.2	0.6 - 5.1	8.6	3.0 - 19.4	0.0	0.0 - 0.0	11.8	3.6 - 24.7	0.84
3170001	9710	OKATIBBEE CR	48.7	55.9	17 - 122	0.7	0.2 - 1.8	31.5	8.8 - 68.8	1.9	0.5 - 5.4	7.0	2.0 - 19.0	0.0	0.0 - 0.0	14.7	4.9 - 36.8	0.84
3170001	9711	*A	57.8	40.5	15 - 71	1.9	0.7 - 4.4	11.3	3.7 - 24.1	3.1	1.1 - 9.4	12.3	4.3 - 32.9	0.0	0.0 - 0.0	11.9	4.5 - 27.9	0.82
3170001	9712	OKATIBBEE CR	136.0	42.2	14 - 105	0.0	0.0 - 0.0	9.2	3.1 - 22.3	4.1	1.2 - 11.5	15.7	4.6 - 44.9	0.0	0.0 - 0.0	13.2	4.8 - 36.8	0.82
3170001	9713	CHICKASAWHAY (	99.3	39.3	14 - 82	0.0	0.0 - 0.0	6.9	2.1 - 15.1	3.9	1.1 - 10.1	15.9	5.2 - 36.1	0.0	0.0 - 0.0	12.6	4.0 - 26.1	0.30
3170001	9714	OKATIBBEE CR	166.8	41.7	13 - 85	0.0	0.0 - 0.0	6.4	2.2 - 12.9	7.9	2.5 - 21.1	14.2	4.4 - 35.5	0.0	0.0 - 0.0	13.2	4.0 - 31.3	0.30
3170001	9715	CHUNKY R	151.4	23.0	8 - 42	0.0	0.0 - 0.0	5.7	1.7 - 10.9	1.5	0.4 - 3.8	3.4	1.1 - 7.9	0.0	0.0 - 0.0	12.4	4.0 - 25.4	0.86
3170001	9716	TALLAHATTA CR	232.8	23.3	9 - 46	0.0	0.0 - 0.0	4.9	1.8 - 10.4	4.4	1.4 - 11.7	5.3	1.8 - 14.7	0.0	0.0 - 0.0	8.6	3.4 - 20.4	0.84
3170001	9717	CHUNKY R	65.3	43.3	17 - 105	0.2	0.1 - 0.4	8.1	2.9 - 18.2	9.0	2.6 - 25.7	8.7	2.6 - 23.1	0.0	0.0 - 0.0	17.2	6.5 - 43.1	0.84
3170001	9718	CHUNKY CR	3.8	112.4	37 - 234	0.0	0.0 - 0.0	15.1	4.4 - 33.7	28.7	8.0 - 86.2	26.7	8.4 - 66.5	0.0	0.0 - 0.0	41.9	15.7 - 104.3	0.81
3170001	9719	CHUNKY CR	33.6	68.0	22 - 135	0.0	0.0 - 0.0	6.0	2.1 - 13.5	19.0	6.0 - 61.2	16.0	5.5 - 46.0	0.0	0.0 - 0.0	27.0	8.7 - 75.0	0.81
3170001	9720	TALLASHUA CR	42.3	43.2	12 - 79	0.0	0.0 - 0.0	3.8	1.1 - 7.0	8.4	1.9 - 20.6	6.7	1.8 - 14.2	0.0	0.0 - 0.0	24.4	7.2 - 50.4	0.79
3170001	9721	TALLASHUA CR	161.7	47.2	16 - 96	0.0	0.0 - 0.0	6.0	1.8 - 12.8	15.6	4.6 - 42.4	11.7	3.7 - 29.8	0.0	0.0 - 0.0	13.9	4.9 - 32.2	0.75
3170001	9722	*B	52.8	58.1	19 - 131	0.0	0.0 - 0.0	7.4	2.6 - 17.1	18.2	5.9 - 59.4	15.0	4.4 - 44.1	0.0	0.0 - 0.0	17.6	5.6 - 39.9	0.75
3170001	9723	CHUNKY CR	138.7	64.6	22 - 142	2.2	0.7 - 4.9	8.2	2.8 - 21.1	18.9	6.0 - 53.8	15.8	5.4 - 41.3	0.0	0.0 - 0.0	19.6	6.5 - 46.7	0.79
3170001	9724	OCKAHATTA CR	123.0	93.5	37 - 190	2.7	1.0 - 5.9	7.8	2.8 - 16.8	30.6	10.2 - 91.1	26.4	9.0 - 57.8	0.0	0.0 - 0.0	26.0	9.8 - 59.2	0.81
3170001	9725	POTTERCHITTO CI	125.7	85.5	25 - 159	0.6	0.2 - 1.4	6.4	1.7 - 12.7	27.8	6.7 - 58.8	23.5	6.9 - 53.4	0.0	0.0 - 0.0	27.3	8.8 - 60.0	0.81
3170001	9726	TURKEY CR	61.7	70.9	24 - 161	0.0	0.0 - 0.0	5.3	1.9 - 12.2	16.6	5.2 - 41.1	14.8	4.5 - 46.4	0.0	0.0 - 0.0	34.2	12.2 - 98.4	0.76
3170001	9727	POTTERCHITTO CI	1.4	30.1	10 - 67	0.0	0.0 - 0.0	1.0	0.4 - 2.2	0.0	0.0 - 0.0	0.0	0.0 - 0.0	0.0	0.0 - 0.0	29.0	9.4 - 66.9	0.76
3170001	9728	RISER CR	91.4	130.1	51 - 315	8.2	3.3 - 22.6	14.1	5.6 - 39.0	35.5	12.3 - 99.6	30.8	11.0 - 104.4	0.0	0.0 - 0.0	41.4	15.5 - 110.1	0.76
3170001	9729	TARLOW CR	70.0	107.7	38 - 207	0.0	0.0 - 0.0	7.3	2.6 - 13.4	31.5	9.5 - 69.0	27.8	10.3 - 55.1	0.0	0.0 - 0.0	41.1	15.2 - 92.0	0.76
3170002	9731	BUCKATONNA R	61.8	83.8	30 - 214	0.0	0.0 - 0.0	9.6	3.1 - 25.8	33.1	11.5 - 108.5	22.3	8.2 - 79.6	0.0	0.0 - 0.0	18.8	7.1 - 50.8	0.94
3170002	9732	LITTLE RED R	173.0	31.4	10 - 58	0.0	0.0 - 0.0	5.6	1.9 - 10.4	7.8	2.4 - 19.0	11.7	3.7 - 29.6	0.0	0.0 - 0.0	6.3	2.0 - 13.1	0.93

8-digit HUC	ID	Name	Area	Catchment Yield		Point sources		Developed Land		Manure		Agricultural Land		Phosphate Mines		Soil parent rock		Frac
				kg/km^2	90% CI	kg/km^2	90% CI	kg/km^2	90% CI	kg/km^2	90% CI	kg/km^2	90% CI	kg/km^2	90% CI	kg/km^2	90% CI	
3170002	9733	BUCKATONNA R	209.5	40.5	13 - 73	0.0	0.0 - 0.0	6.5	2.1 - 12.6	13.8	4.0 - 32.0	10.3	2.6 - 25.2	0.0	0.0 - 0.0	9.8	3.3 - 24.0	0.93
3170002	9734	BUCKATONNA R	338.8	45.5	17 - 102	0.0	0.0 - 0.0	4.5	1.5 - 8.3	8.1	2.6 - 23.6	12.3	4.2 - 30.2	0.0	0.0 - 0.0	20.5	7.7 - 46.4	0.91
3170002	9735	BUCKATONNA R	54.3	27.5	10 - 70	0.0	0.0 - 0.0	4.8	1.5 - 10.9	1.7	0.5 - 4.6	5.2	1.6 - 15.1	0.0	0.0 - 0.0	15.9	5.1 - 41.8	0.87
3170002	9736	ROCKY CR	81.9	11.5	4 - 25	0.0	0.0 - 0.0	3.3	1.0 - 6.7	0.5	0.2 - 1.3	1.4	0.5 - 3.5	0.0	0.0 - 0.0	6.3	2.2 - 14.7	0.85
3170002	9737	BUCKATONNA R	9.6	31.2	11 - 65	0.0	0.0 - 0.0	1.2	0.4 - 2.2	0.2	0.0 - 0.3	0.5	0.2 - 1.1	0.0	0.0 - 0.0	29.3	10.6 - 64.9	0.85
3170002	9738	BUCKATONNA R	33.7	18.2	6 - 34	0.0	0.0 - 0.0	3.0	1.0 - 6.5	1.6	0.5 - 4.4	4.9	1.7 - 12.2	0.0	0.0 - 0.0	8.7	3.1 - 19.6	0.84
3170002	9739	HURRICANE CR	97.5	20.3	7 - 52	0.0	0.0 - 0.0	5.5	1.8 - 14.2	1.6	0.5 - 4.6	5.7	1.8 - 17.7	0.0	0.0 - 0.0	7.6	2.5 - 19.3	0.81
3170002	9740	BUCKATONNA R	193.1	25.3	10 - 63	0.0	0.0 - 0.0	6.6	2.6 - 16.2	1.8	0.7 - 4.9	7.0	2.7 - 18.5	0.0	0.0 - 0.0	9.9	4.5 - 26.7	0.81
3170002	9741	LONG CR	211.1	27.7	9 - 51	0.0	0.0 - 0.0	6.6	2.1 - 13.0	2.0	0.5 - 4.8	7.0	2.2 - 18.5	0.0	0.0 - 0.0	12.1	3.4 - 26.0	0.84
3170002	9742	HANGING MOSS C	64.4	41.0	13 - 104	0.0	0.0 - 0.0	5.3	1.5 - 11.7	3.3	0.8 - 9.0	10.3	3.1 - 25.6	0.0	0.0 - 0.0	22.2	7.5 - 73.5	0.87
3170002	9743	DRY CR	68.0	100.2	34 - 215	0.0	0.0 - 0.0	2.5	0.9 - 7.0	32.3	10.2 - 90.0	37.1	11.2 - 119.5	0.0	0.0 - 0.0	28.3	10.6 - 72.8	0.91
3170002	9744	CHICKASAWHAY	226.6	69.6	27 - 128	7.6	3.0 - 14.9	16.7	5.7 - 32.5	20.6	7.1 - 50.1	13.5	4.9 - 31.4	0.0	0.0 - 0.0	11.2	4.4 - 21.7	0.94
3170002	9745	CHICKASAWHAY	355.1	39.4	11 - 70	0.0	0.0 - 0.0	7.2	1.9 - 14.2	10.2	2.4 - 32.1	8.8	2.6 - 22.4	0.0	0.0 - 0.0	13.2	4.0 - 30.5	0.92
3170002	9746	CHICKASAWHAY	180.5	51.3	19 - 107	0.0	0.0 - 0.0	4.4	1.4 - 8.8	3.8	1.2 - 11.5	12.3	3.7 - 30.5	0.0	0.0 - 0.0	30.8	12.3 - 65.3	0.90
3170002	9747	ARCHUSA CR	157.7	33.9	12 - 73	0.0	0.0 - 0.0	8.0	3.0 - 19.3	2.1	0.7 - 6.1	6.5	2.3 - 16.5	0.0	0.0 - 0.0	17.3	6.2 - 39.3	0.89
3170002	9748	CHICKASAWHAY	15.4	124.3	37 - 272	70.6	21.8 - 160.8	12.7	3.4 - 29.8	1.8	0.4 - 5.2	6.8	1.7 - 17.9	0.0	0.0 - 0.0	32.4	9.9 - 77.8	0.89
3170002	9749	CHICKASAWHAY	142.5	50.6	17 - 96	2.7	0.9 - 5.3	9.9	3.2 - 19.7	3.1	0.9 - 8.3	9.9	2.9 - 22.3	0.0	0.0 - 0.0	25.0	8.1 - 54.9	0.89
3170002	9750	SOUINLOVEY CR	20.6	44.1	14 - 104	0.0	0.0 - 0.0	4.9	1.6 - 10.7	3.6	1.1 - 12.1	11.4	2.9 - 29.8	0.0	0.0 - 0.0	24.1	8.4 - 60.4	0.89
3170002	9751	GORDON CR	30.9	38.7	13 - 83	0.0	0.0 - 0.0	7.9	2.5 - 16.9	2.3	0.7 - 6.4	7.0	2.4 - 19.3	0.0	0.0 - 0.0	21.4	7.5 - 52.1	0.88
3170002	9752	SOUINLOVEY CR	492.2	58.3	19 - 106	0.0	0.0 - 0.0	4.1	1.3 - 8.7	13.0	4.2 - 38.6	14.7	5.1 - 35.3	0.0	0.0 - 0.0	26.5	8.1 - 62.3	0.88
3170002	9753	SHUBUTA CR	43.8	75.3	24 - 177	5.6	2.0 - 12.7	8.2	2.6 - 18.3	5.1	1.5 - 14.1	16.2	5.1 - 39.7	0.0	0.0 - 0.0	40.2	13.2 - 107.4	0.90
3170002	9754	CANE CR	135.9	44.1	16 - 82	0.3	0.1 - 0.6	4.4	1.4 - 8.9	4.5	1.6 - 12.8	11.0	3.6 - 26.8	0.0	0.0 - 0.0	23.9	8.9 - 50.4	0.89
3170002	9755	SHUBUTA CR	196.4	34.6	13 - 79	0.0	0.0 - 0.0	3.7	1.2 - 7.4	2.8	0.9 - 9.9	6.0	2.1 - 16.6	0.0	0.0 - 0.0	22.2	8.3 - 54.6	0.89
3170002	9756	YELLOW CR	142.8	40.9	15 - 94	0.0	0.0 - 0.0	9.3	3.7 - 22.4	14.2	4.5 - 41.8	9.1	3.7 - 26.1	0.0	0.0 - 0.0	8.3	3.2 - 19.8	0.92
3170003	9757	CHICKASAWHAY	135.5	29.1	10 - 68	0.0	0.0 - 0.0	8.4	2.8 - 20.9	2.2	0.7 - 6.1	3.7	1.3 - 9.8	0.0	0.0 - 0.0	14.7	5.4 - 34.2	0.98
3170003	9758	CHICKASAWHAY	189.3	40.8	14 - 95	4.5	1.8 - 11.0	8.4	3.0 - 21.5	5.4	2.0 - 14.4	6.9	2.4 - 20.1	0.0	0.0 - 0.0	15.6	5.8 - 39.7	0.97
3170003	9759	CHICKASAWHAY	21.0	64.2	25 - 145	0.0	0.0 - 0.0	5.5	2.1 - 11.2	27.7	9.8 - 78.7	17.6	6.2 - 38.7	0.0	0.0 - 0.0	13.4	5.2 - 34.0	0.94
3170003	9760	BIG CR	376.8	35.1	14 - 80	0.0	0.0 - 0.1	6.1	2.1 - 13.0	12.1	4.0 - 32.5	7.9	2.8 - 22.4	0.0	0.0 - 0.0	9.0	3.8 - 18.8	0.94
3170003	9761	BIG CR	29.6	27.3	9 - 51	0.0	0.0 - 0.0	7.8	2.4 - 15.7	4.4	1.1 - 13.6	4.5	1.6 - 11.1	0.0	0.0 - 0.0	10.6	3.3 - 24.6	0.97
3170003	9762	BIG CR	88.9	33.1	12 - 59	0.0	0.0 - 0.0	9.4	3.1 - 19.4	5.9	1.9 - 16.5	9.4	2.9 - 22.6	0.0	0.0 - 0.0	8.4	2.8 - 19.4	0.95
3170003	9763	MASON CR	120.5	35.1	13 - 59	0.0	0.0 - 0.0	7.0	2.5 - 13.0	4.8	1.4 - 11.3	7.4	2.5 - 15.6	0.0	0.0 - 0.0	15.9	5.6 - 30.0	0.92
3170003	9764	BIG CR	185.6	35.7	13 - 80	0.0	0.0 - 0.0	8.0	3.1 - 19.6	6.0	1.7 - 16.9	9.3	3.2 - 25.1	0.0	0.0 - 0.0	12.4	5.0 - 31.7	0.92
3170003	9765	BRUSHY CR	57.7	40.0	16 - 92	0.0	0.0 - 0.0	7.5	2.7 - 17.7	8.4	2.7 - 24.4	13.0	4.8 - 37.4	0.0	0.0 - 0.0	11.2	4.4 - 25.8	0.95
3170004	9767	LEAF R	279.9	81.1	28 - 177	21.0	7.5 - 50.2	12.1	4.3 - 25.4	20.4	5.9 - 49.8	17.8	6.3 - 48.6	0.0	0.0 - 0.0	9.8	3.6 - 22.5	0.93
3170004	9768	LEAF R	16.1	60.0	20 - 115	0.0	0.0 - 0.0	4.4	1.4 - 9.6	25.8	7.4 - 82.4	19.7	5.9 - 51.5	0.0	0.0 - 0.0	10.3	3.2 - 25.5	0.90
3170004	9769	BIG CR	112.4	87.2	29 - 174	0.0	0.0 - 0.0	7.9	2.5 - 15.4	37.8	11.7 - 105.1	28.1	9.4 - 77.5	0.0	0.0 - 0.0	13.4	4.4 - 28.4	0.90
3170004	9770	ETEHOMO CR	145.3	63.7	24 - 142	2.2	0.8 - 5.1	9.0	3.3 - 20.0	23.4	8.4 - 64.9	19.1	6.8 - 55.4	0.0	0.0 - 0.0	10.0	4.0 - 24.9	0.86
3170004	9771	LITTLE CR	44.9	57.3	25 - 131	0.0	0.0 - 0.0	7.4	3.1 - 16.3	27.1	10.9 - 68.1	13.5	5.1 - 34.9	0.0	0.0 - 0.0	9.3	3.6 - 22.8	0.86
3170004	9772	LEAF R	91.2	68.7	21 - 144	0.0	0.0 - 0.0	8.4	2.5 - 17.2	27.3	8.9 - 69.7	21.7	6.7 - 56.6	0.0	0.0 - 0.0	11.3	3.7 - 27.5	0.90

8-digit HUC	ID	Name	Area	Catchment Yield		Point sources		Developed Land		Manure		Agricultural Land		Phosphate Mines		Soil parent rock		Frac
				kg/km^2	90% CI	kg/km^2	90% CI	kg/km^2	90% CI	kg/km^2	90% CI	kg/km^2	90% CI	kg/km^2	90% CI	kg/km^2	90% CI	
3170004	9773	LEAF R	85.9	72.2	26 - 189	0.9	0.3 - 2.6	7.2	2.5 - 17.5	32.3	10.9 - 120.5	16.5	5.9 - 45.6	0.0	0.0 - 0.0	15.4	6.1 - 49.1	0.88
3170004	9774	LEAF R	95.1	49.6	20 - 95	0.0	0.0 - 0.0	4.7	1.7 - 8.6	22.8	7.8 - 59.5	10.2	4.5 - 24.5	0.0	0.0 - 0.0	11.9	4.4 - 29.1	0.86
3170004	9775	W TALLAHALA CF	173.0	66.3	22 - 160	0.0	0.0 - 0.0	4.8	1.6 - 11.4	27.7	7.4 - 85.0	16.4	5.7 - 42.5	0.0	0.0 - 0.0	17.3	5.4 - 45.0	0.84
3170004	9776	W TALLAHALA CF	19.1	75.5	23 - 151	0.0	0.0 - 0.0	1.6	0.5 - 3.3	14.5	4.1 - 34.7	8.4	2.7 - 18.3	0.0	0.0 - 0.0	51.1	15.5 - 107.6	0.77
3170004	9777	QUARTERLIAH CR	89.6	117.0	38 - 223	0.0	0.0 - 0.0	2.2	0.7 - 4.2	32.5	9.0 - 81.6	28.3	9.8 - 61.1	0.0	0.0 - 0.0	54.1	16.5 - 111.3	0.74
3170004	9778	W TALLAHALA CF	115.2	97.4	38 - 172	0.0	0.0 - 0.0	1.7	0.7 - 3.3	24.4	8.0 - 83.0	17.0	5.8 - 35.1	0.0	0.0 - 0.0	54.3	21.5 - 124.2	0.74
3170004	9779	LEAF R	35.6	94.7	29 - 253	0.0	0.0 - 0.0	6.6	2.2 - 17.4	50.4	14.9 - 162.1	22.2	6.9 - 79.7	0.0	0.0 - 0.0	15.5	6.1 - 46.9	0.84
3170004	9780	ICHUSA CR	127.3	78.8	26 - 155	1.8	0.6 - 4.0	4.6	1.6 - 9.5	35.7	10.5 - 99.9	15.7	5.4 - 43.5	0.0	0.0 - 0.0	21.1	6.8 - 44.2	0.83
3170004	9781	LEAF R	250.7	52.0	18 - 102	0.0	0.0 - 0.0	4.8	1.6 - 11.0	22.1	6.2 - 66.8	10.1	3.1 - 26.1	0.0	0.0 - 0.0	15.0	5.0 - 35.6	0.83
3170004	9782	LEAF R	91.1	131.3	32 - 307	0.0	0.0 - 0.0	4.6	1.3 - 12.0	43.7	11.2 - 131.6	26.6	9.9 - 87.9	0.0	0.0 - 0.0	56.5	16.0 - 130.8	0.75
3170004	9783	TALLOBOGUE CR	75.0	127.8	44 - 254	0.0	0.0 - 0.0	6.6	2.3 - 11.8	49.1	15.8 - 163.8	28.5	11.0 - 75.0	0.0	0.0 - 0.0	43.6	15.5 - 114.2	0.75
3170004	9784	FISHER CR	71.7	87.7	30 - 165	9.4	3.4 - 18.3	9.2	3.5 - 18.0	41.0	13.1 - 109.1	18.2	6.8 - 40.2	0.0	0.0 - 0.0	10.0	3.6 - 20.2	0.86
3170004	9785	OAKOHAY CR	638.4	71.1	27 - 154	0.1	0.0 - 0.2	6.3	1.9 - 14.6	36.0	11.2 - 97.3	17.3	6.0 - 45.4	0.0	0.0 - 0.0	11.4	4.5 - 31.1	0.88
3170004	9786	OAKEY WOODS CI	151.3	62.2	19 - 111	0.0	0.0 - 0.0	9.6	2.8 - 18.7	20.9	5.6 - 59.7	19.5	6.5 - 48.5	0.0	0.0 - 0.0	12.2	3.8 - 28.6	0.90
3170004	9787	BOWIE R	223.6	74.6	24 - 171	14.1	4.6 - 36.8	28.3	8.3 - 72.9	8.0	2.2 - 26.8	13.1	4.5 - 41.7	0.0	0.0 - 0.0	11.2	3.8 - 26.5	0.93
3170004	9788	OKATOMA CR	74.5	81.1	33 - 155	0.0	0.0 - 0.0	9.9	3.8 - 20.6	30.9	10.8 - 91.1	27.0	11.2 - 60.4	0.0	0.0 - 0.0	13.3	5.4 - 28.6	0.91
3170004	9789	BLACKLEY CR	42.6	73.0	24 - 191	0.0	0.0 - 0.0	8.0	2.5 - 24.3	31.8	9.9 - 92.3	20.4	6.6 - 59.1	0.0	0.0 - 0.0	12.7	4.6 - 35.8	0.85
3170004	9790	OKATOMA CR	370.1	98.3	39 - 221	2.0	0.7 - 5.1	11.9	4.7 - 26.6	43.3	15.0 - 133.9	28.2	10.5 - 87.9	0.0	0.0 - 0.0	13.0	4.8 - 34.2	0.85
3170004	9791	BOWIE R	112.2	66.8	27 - 145	0.0	0.0 - 0.0	8.3	3.1 - 19.6	22.4	7.7 - 72.8	23.6	10.0 - 59.8	0.0	0.0 - 0.0	12.5	4.8 - 30.6	0.91
3170004	9792	TERRIBLE CR	116.6	54.0	19 - 125	0.0	0.0 - 0.0	7.9	2.8 - 20.4	18.3	5.8 - 48.7	17.7	6.2 - 47.9	0.0	0.0 - 0.0	10.1	3.8 - 25.5	0.86
3170004	9793	BOWIE R	142.2	60.3	20 - 143	0.0	0.0 - 0.0	8.7	2.5 - 19.9	13.7	4.0 - 38.8	24.2	7.5 - 60.5	0.0	0.0 - 0.0	13.8	4.6 - 34.7	0.86
3170004	9794	DRY CR	54.4	119.9	32 - 218	0.0	0.0 - 0.0	8.1	2.1 - 14.3	41.4	11.3 - 96.8	40.8	11.3 - 87.9	0.0	0.0 - 0.0	29.5	8.9 - 67.7	0.83
3170004	9795	BOWIE CR	195.7	71.2	25 - 126	0.0	0.0 - 0.0	7.3	2.6 - 15.3	25.4	7.8 - 70.3	23.7	8.8 - 58.7	0.0	0.0 - 0.0	14.8	4.9 - 33.0	0.83
3170005	9796	LEAF R	193.9	27.3	8 - 67	0.0	0.0 - 0.0	6.8	2.0 - 16.6	2.4	0.6 - 7.0	2.8	0.8 - 9.2	0.0	0.0 - 0.0	15.2	4.9 - 38.3	0.98
3170005	9797	ATKINSON CR	150.2	21.1	7 - 38	0.0	0.0 - 0.0	7.3	2.2 - 14.1	2.8	1.0 - 7.1	4.7	1.6 - 11.1	0.0	0.0 - 0.0	6.2	2.1 - 12.9	0.97
3170005	9798	LEAF R	31.6	35.2	12 - 78	1.2	0.4 - 2.7	11.4	3.5 - 24.0	3.6	1.2 - 10.6	5.9	2.1 - 15.3	0.0	0.0 - 0.0	13.1	4.5 - 30.3	0.97
3170005	9799	GAINES CR	144.5	51.0	18 - 113	0.0	0.0 - 0.0	7.8	2.3 - 17.2	8.7	2.7 - 24.4	18.4	5.9 - 43.5	0.0	0.0 - 0.0	16.1	5.2 - 40.9	0.96
3170005	9800	SAND HILL CR	80.0	79.8	23 - 152	0.0	0.0 - 0.0	9.8	2.7 - 20.1	20.0	5.9 - 50.2	29.2	8.6 - 70.1	0.0	0.0 - 0.0	20.8	6.2 - 46.7	0.87
3170005	9801	PINEY WOODS CR	144.3	41.4	17 - 102	0.0	0.0 - 0.0	6.8	2.7 - 17.5	9.9	3.3 - 32.8	11.3	4.2 - 29.4	0.0	0.0 - 0.0	13.4	5.1 - 37.5	0.87
3170005	9802	LEAF R	76.8	22.6	7 - 42	0.0	0.0 - 0.0	6.9	2.3 - 15.3	2.3	0.7 - 6.1	4.9	1.6 - 13.5	0.0	0.0 - 0.0	8.6	2.9 - 18.1	0.96
3170005	9803	THOMPSON CR	380.7	25.4	8 - 54	0.7	0.2 - 1.8	6.3	1.9 - 12.9	4.1	1.2 - 11.9	6.6	1.7 - 18.0	0.0	0.0 - 0.0	7.7	2.3 - 19.2	0.96
3170005	9804	LITTLE THOMPSOI	97.1	31.0	10 - 76	0.0	0.0 - 0.0	5.6	1.7 - 13.0	11.1	3.4 - 33.9	7.4	2.2 - 20.5	0.0	0.0 - 0.0	6.9	2.1 - 17.0	0.83
3170005	9805	THOMPSON CR	107.9	56.2	18 - 125	0.0	0.0 - 0.0	6.8	2.0 - 15.1	24.3	6.7 - 65.4	15.8	5.2 - 39.6	0.0	0.0 - 0.0	9.4	3.2 - 20.4	0.83
3170005	9806	LEAF R	111.0	25.5	10 - 43	2.2	0.9 - 4.2	9.8	3.7 - 18.2	1.8	0.6 - 3.9	3.7	1.4 - 7.6	0.0	0.0 - 0.0	8.1	3.0 - 14.9	0.96
3170005	9807	BOGUE HOMO	84.0	33.6	13 - 78	0.0	0.0 - 0.0	6.5	2.2 - 15.1	4.7	1.6 - 14.3	10.5	3.7 - 26.4	0.0	0.0 - 0.0	11.8	4.5 - 32.8	0.95
3170005	9808	BOGUE HOMO	64.5	32.2	11 - 59	0.0	0.0 - 0.0	7.6	2.5 - 15.6	5.2	1.5 - 11.0	10.4	3.6 - 24.4	0.0	0.0 - 0.0	8.9	3.1 - 18.5	0.94
3170005	9809	TIGER CR	145.9	20.9	7 - 38	0.0	0.0 - 0.0	3.9	1.3 - 8.4	5.0	1.6 - 12.7	3.7	1.2 - 9.3	0.0	0.0 - 0.0	8.2	2.8 - 16.7	0.92
3170005	9810	BOGUE HOMO	414.0	49.0	18 - 121	0.1	0.0 - 0.1	5.6	2.1 - 14.1	19.6	6.4 - 58.8	15.1	5.4 - 45.6	0.0	0.0 - 0.0	8.7	3.4 - 21.9	0.92
3170005	9811	LITTLE BOGUE HC	123.6	47.5	16 - 130	0.0	0.0 - 0.0	5.2	1.9 - 16.0	16.2	4.6 - 54.9	13.5	5.1 - 35.7	0.0	0.0 - 0.0	12.6	4.1 - 40.8	0.69

8-digit HUC	ID	Name	Area	Catchment Yield		Point sources		Developed Land		Manure		Agricultural Land		Phosphate Mines		Soil parent rock		Frac
				kg/km2	90% CI	kg/km2	90% CI	kg/km2	90% CI	kg/km2	90% CI	kg/km2	90% CI	kg/km2	90% CI	kg/km2	90% CI	
3170005	9812	BOGUE HOMO	186.2	45.3	12 - 78	0.7	0.2 - 1.4	11.3	3.1 - 20.9	10.5	2.2 - 26.8	10.4	2.9 - 21.7	0.0	0.0 - 0.0	12.3	3.5 - 27.7	0.69
3170005	9813	BUCK CR	80.4	36.7	12 - 66	0.0	0.0 - 0.0	7.1	2.2 - 13.0	9.7	3.1 - 26.5	12.5	3.7 - 32.6	0.0	0.0 - 0.0	7.4	2.5 - 17.1	0.94
3170005	9814	LEAF R	69.8	27.8	7 - 59	1.5	0.4 - 3.1	10.1	2.5 - 19.6	2.0	0.5 - 5.5	4.2	1.1 - 11.7	0.0	0.0 - 0.0	9.9	2.8 - 23.2	0.95
3170005	9815	TALLAHALA CR	69.5	303.4	103 - 657	256.4	85.7 - 564.1	5.7	2.0 - 10.8	8.4	2.6 - 26.6	19.2	6.4 - 45.7	0.0	0.0 - 0.0	13.7	4.6 - 31.4	0.95
3170005	9816	TALLAHALA CR	36.2	67.5	22 - 143	15.2	4.9 - 35.5	7.2	2.3 - 14.9	18.8	4.9 - 47.3	14.4	4.2 - 35.2	0.0	0.0 - 0.0	12.0	3.8 - 26.4	0.90
3170005	9817	TALLAHALA CR	173.9	158.2	48 - 333	64.6	20.2 - 135.9	31.2	9.4 - 64.2	25.9	7.4 - 68.2	21.6	6.5 - 49.4	0.0	0.0 - 0.0	14.9	4.6 - 36.2	0.89
3170005	9818	TALLATTAH CR	80.8	21.8	7 - 42	0.0	0.0 - 0.0	5.3	1.8 - 11.1	4.0	1.3 - 10.8	4.6	1.4 - 11.3	0.0	0.0 - 0.0	7.9	2.6 - 17.4	0.83
3170005	9819	TALLAHALA CR	284.6	32.6	12 - 79	0.0	0.0 - 0.0	3.8	1.5 - 9.3	7.1	2.1 - 22.4	8.1	2.9 - 21.0	0.0	0.0 - 0.0	13.6	4.9 - 34.8	0.83
3170005	9820	MCVAY CR	14.5	35.7	14 - 88	0.0	0.0 - 0.0	3.4	1.2 - 8.0	6.4	2.0 - 19.9	8.1	2.8 - 21.0	0.0	0.0 - 0.0	17.9	7.1 - 50.0	0.77
3170005	9821	TALLAHALA CR	107.0	55.1	20 - 166	0.0	0.0 - 0.0	2.7	0.9 - 8.9	9.3	3.1 - 30.7	10.7	3.5 - 37.3	0.0	0.0 - 0.0	32.4	12.0 - 90.5	0.77
3170005	9822	TALLAHOMA CR	195.7	94.5	32 - 163	2.1	0.8 - 4.4	15.5	4.9 - 28.5	35.7	11.8 - 81.4	28.2	10.3 - 65.4	0.0	0.0 - 0.0	13.1	4.3 - 31.1	0.89
3170005	9823	TALLAHOMA CR	307.0	45.6	15 - 87	0.0	0.0 - 0.0	5.3	1.7 - 11.0	12.0	3.7 - 27.7	13.4	4.4 - 31.0	0.0	0.0 - 0.0	14.9	5.1 - 37.4	0.84
3170005	9824	TARAPIN CR	50.5	44.5	14 - 99	0.0	0.0 - 0.0	8.4	2.5 - 19.1	13.0	3.4 - 35.0	13.6	4.6 - 39.4	0.0	0.0 - 0.0	9.6	2.3 - 23.2	0.84
3170005	9825	ROCKY CR	89.4	101.9	32 - 214	2.8	0.9 - 7.0	16.9	5.5 - 38.2	38.3	11.1 - 102.6	30.1	10.3 - 83.1	0.0	0.0 - 0.0	13.9	4.5 - 33.2	0.90
3170005	9826	LEAF R	48.7	33.0	10 - 70	0.0	0.0 - 0.0	11.5	3.5 - 26.6	3.1	0.8 - 8.3	6.6	1.7 - 18.0	0.0	0.0 - 0.0	11.8	3.3 - 30.5	0.95
3170005	9827	REESE CR	46.0	66.3	20 - 120	0.4	0.1 - 0.7	7.9	2.6 - 13.6	14.0	4.0 - 32.7	33.2	9.7 - 65.9	0.0	0.0 - 0.0	10.9	3.7 - 20.7	0.94
3170005	9828	LEAF R	109.3	52.6	19 - 109	12.1	4.5 - 28.5	13.1	4.5 - 27.8	5.4	1.5 - 13.5	12.3	4.3 - 27.9	0.0	0.0 - 0.0	9.8	3.5 - 22.7	0.94
3170006	9829	PASCAGOULA R	167.6	34.3	13 - 74	0.0	0.0 - 0.0	8.5	2.9 - 21.6	1.7	0.5 - 5.3	3.7	1.2 - 11.5	0.0	0.0 - 0.0	20.4	7.2 - 53.0	1.00
3170006	9830	PASCAGOULA R	93.7	25.8	10 - 70	1.5	0.6 - 3.6	4.6	1.6 - 12.6	1.9	0.7 - 5.8	5.2	1.9 - 15.6	0.0	0.0 - 0.0	12.5	4.7 - 34.2	0.99
3170006	9831	BIG CEDAR R	185.2	43.5	16 - 108	2.3	0.9 - 6.2	10.2	3.3 - 31.1	4.0	1.3 - 10.9	15.9	6.1 - 41.9	0.0	0.0 - 0.0	11.1	4.0 - 27.3	0.99
3170006	9832	PASCAGOULA R	78.5	43.9	16 - 107	0.0	0.0 - 0.0	5.8	1.9 - 14.8	4.8	1.5 - 13.0	19.1	6.6 - 58.5	0.0	0.0 - 0.0	14.2	5.0 - 34.5	0.99
3170006	9833	WHITE CR	53.4	44.6	15 - 90	0.0	0.0 - 0.0	11.2	3.4 - 24.2	4.5	1.4 - 12.1	18.5	6.0 - 45.0	0.0	0.0 - 0.0	10.5	3.4 - 23.6	0.99
3170006	9834	PASCAGOULA R	49.5	34.7	12 - 83	0.0	0.0 - 0.0	3.4	1.0 - 8.1	1.5	0.5 - 4.1	5.5	1.7 - 14.6	0.0	0.0 - 0.0	24.4	8.5 - 63.0	0.99
3170006	9835	BIG CR	144.8	30.7	10 - 72	0.0	0.0 - 0.0	13.1	4.0 - 30.7	1.9	0.6 - 5.5	6.3	2.0 - 17.8	0.0	0.0 - 0.0	9.4	3.0 - 24.6	0.98
3170006	9836	PASCAGOULA R	33.5	21.0	7 - 44	0.0	0.0 - 0.0	4.2	1.4 - 8.9	1.1	0.3 - 3.2	0.7	0.2 - 1.8	0.0	0.0 - 0.0	15.0	5.3 - 38.5	0.98
3170006	9837	PASCAGOULA R	76.2	19.8	8 - 46	0.0	0.0 - 0.0	3.5	1.1 - 8.2	0.8	0.2 - 2.3	1.1	0.4 - 3.0	0.0	0.0 - 0.0	14.4	5.4 - 37.7	0.98
3170006	9838	WHISKEY CR	152.4	12.1	4 - 26	0.0	0.0 - 0.0	3.8	1.3 - 8.5	0.4	0.1 - 1.2	0.4	0.1 - 1.0	0.0	0.0 - 0.0	7.5	2.7 - 16.3	0.98
3170006	9839	MOUNGERS CR	53.9	54.1	19 - 107	0.0	0.0 - 0.0	12.2	4.3 - 23.6	1.9	0.6 - 5.4	4.0	1.3 - 9.4	0.0	0.0 - 0.0	36.0	12.0 - 79.9	1.00
3170006	9840	MOUNGERS CR	10.8	35.0	11 - 66	5.1	1.5 - 10.9	5.7	1.9 - 10.7	1.2	0.3 - 3.8	0.4	0.1 - 1.1	0.0	0.0 - 0.0	22.6	7.0 - 54.3	0.98
3170006	9841	MOUNGERS CR	264.3	25.2	9 - 44	0.2	0.1 - 0.5	7.9	2.5 - 16.5	1.3	0.4 - 3.6	1.7	0.5 - 4.2	0.0	0.0 - 0.0	13.9	4.8 - 26.0	0.96
3170006	9842	BLUFF CR	30.1	51.1	15 - 95	0.0	0.0 - 0.0	13.8	3.7 - 27.7	5.9	1.6 - 12.8	16.7	4.5 - 43.4	0.0	0.0 - 0.0	14.6	4.2 - 28.7	0.96
3170006	9843	BAYOU CASTELLE	29.1	40.6	13 - 92	0.0	0.0 - 0.0	15.8	4.9 - 36.8	2.8	0.8 - 7.5	1.4	0.4 - 3.6	0.0	0.0 - 0.0	20.6	7.1 - 47.6	0.98
3170007	9844	BLACK CR	17.9	11.1	4 - 27	0.0	0.0 - 0.0	1.0	0.4 - 2.4	0.1	0.0 - 0.2	0.1	0.0 - 0.2	0.0	0.0 - 0.0	9.9	3.3 - 25.5	0.99
3170007	9845	BLACK CR	184.6	21.9	8 - 40	0.0	0.0 - 0.0	4.4	1.6 - 7.9	1.3	0.4 - 3.4	2.3	0.8 - 5.4	0.0	0.0 - 0.0	13.9	4.8 - 29.5	0.99
3170007	9846	HICKORY CR	80.2	11.8	4 - 24	0.0	0.0 - 0.0	2.9	1.0 - 6.4	0.2	0.0 - 0.4	0.2	0.1 - 0.5	0.0	0.0 - 0.0	8.6	3.1 - 19.0	0.95
3170007	9847	BLACK CR	10.5	10.9	4 - 22	0.0	0.0 - 0.0	2.0	0.7 - 3.7	0.1	0.0 - 0.4	0.0	0.0 - 0.0	0.0	0.0 - 0.0	8.7	2.8 - 20.2	0.95
3170007	9848	CYPRESS CR	21.8	14.5	6 - 27	0.0	0.0 - 0.0	5.1	1.9 - 9.8	0.7	0.2 - 2.1	1.4	0.5 - 3.8	0.0	0.0 - 0.0	7.2	2.8 - 14.8	0.94
3170007	9849	BLACK CR	25.4	16.1	5 - 35	0.0	0.0 - 0.0	3.3	0.9 - 7.1	0.7	0.2 - 1.9	0.9	0.3 - 2.4	0.0	0.0 - 0.0	11.2	3.4 - 27.4	0.94
3170007	9850	BLACK CR	28.6	19.0	7 - 33	0.0	0.0 - 0.0	3.9	1.4 - 8.5	2.1	0.7 - 4.9	3.3	1.1 - 8.8	0.0	0.0 - 0.0	9.8	3.5 - 19.3	0.94

8-digit HUC	ID	Name	Area	Catchment Yield kg/km²	Catchment Yield 90% CI	Point sources kg/km²	Point sources 90% CI	Developed Land kg/km²	Developed Land 90% CI	Manure kg/km²	Manure 90% CI	Agricultural Land kg/km²	Agricultural Land 90% CI	Phosphate Mines kg/km²	Phosphate Mines 90% CI	Soil parent rock kg/km²	Soil parent rock 90% CI	Frac
3170007	9851	PIERCES CR	67.8	14.2	5 - 34	0.0	0.0 - 0.0	5.5	1.8 - 13.4	1.2	0.4 - 3.0	0.3	0.1 - 0.7	0.0	0.0 - 0.0	7.3	2.8 - 17.3	0.93
3170007	9852	BLACK CR	52.8	17.3	6 - 40	0.0	0.0 - 0.0	4.8	1.6 - 11.5	1.4	0.5 - 4.0	1.4	0.5 - 3.6	0.0	0.0 - 0.0	9.8	3.5 - 24.9	0.93
3170007	9853	POPLAR CR	35.6	34.3	13 - 82	0.0	0.0 - 0.0	17.8	6.0 - 43.9	3.2	1.1 - 8.1	2.5	0.9 - 7.1	0.0	0.0 - 0.0	10.7	4.2 - 26.2	0.92
3170007	9854	POPLAR CR	59.1	37.6	14 - 104	0.0	0.0 - 0.0	27.9	8.9 - 76.6	0.7	0.2 - 1.8	0.6	0.2 - 1.8	0.0	0.0 - 0.0	8.4	3.2 - 22.9	0.90
3170007	9855	WALLS CR	70.2	30.2	12 - 64	0.0	0.0 - 0.1	14.6	5.3 - 33.6	2.5	0.7 - 6.5	5.5	1.9 - 15.0	0.0	0.0 - 0.0	7.7	2.7 - 19.4	0.90
3170007	9856	BLACK CR	163.6	27.5	9 - 68	0.0	0.0 - 0.0	6.5	1.9 - 14.7	4.1	1.2 - 12.2	6.3	1.6 - 19.1	0.0	0.0 - 0.0	10.6	3.7 - 29.1	0.92
3170007	9857	BLACK CR	113.9	34.3	14 - 70	0.0	0.0 - 0.0	12.3	5.1 - 26.6	4.7	1.6 - 11.2	8.1	2.9 - 21.5	0.0	0.0 - 0.0	9.3	3.8 - 20.0	0.90
3170007	9858	SANDY RUN CR	52.4	37.2	12 - 75	0.5	0.2 - 1.0	13.0	4.4 - 28.7	5.4	1.8 - 17.1	8.1	2.6 - 21.5	0.0	0.0 - 0.0	10.3	3.3 - 21.6	0.87
3170007	9859	BLACK CR	73.4	39.2	11 - 103	0.0	0.0 - 0.0	9.3	2.6 - 24.0	9.0	2.5 - 28.5	12.6	4.1 - 36.4	0.0	0.0 - 0.0	8.3	2.2 - 27.6	0.87
3170007	9860	PERKINS CR	50.1	52.0	17 - 95	0.9	0.3 - 1.9	24.6	8.0 - 48.5	6.4	1.9 - 16.9	9.8	2.9 - 27.7	0.0	0.0 - 0.0	10.2	3.7 - 20.1	0.85
3170007	9861	BLACK CR	254.9	46.2	14 - 114	0.0	0.0 - 0.1	7.6	2.2 - 15.9	10.8	3.0 - 25.1	16.7	4.7 - 49.7	0.0	0.0 - 0.0	11.0	3.6 - 30.0	0.85
3170007	9862	LITTLE BLACK CR	235.6	39.6	13 - 91	1.7	0.6 - 4.2	10.8	3.6 - 22.9	7.7	2.3 - 20.4	11.1	3.7 - 29.5	0.0	0.0 - 0.0	8.2	2.7 - 18.7	0.90
3170007	9863	BEAVERDAM CR	171.1	18.8	6 - 44	0.0	0.0 - 0.0	5.6	2.2 - 11.6	1.4	0.4 - 4.4	2.6	0.8 - 7.0	0.0	0.0 - 0.0	9.3	3.2 - 24.7	0.94
3170007	9864	RED CR	127.6	20.6	7 - 35	0.0	0.0 - 0.0	6.5	2.2 - 11.8	1.0	0.3 - 2.5	0.5	0.2 - 1.1	0.0	0.0 - 0.0	12.7	4.4 - 21.6	0.99
3170007	9865	BLUFF CR	102.9	25.4	7 - 53	0.0	0.0 - 0.0	6.0	1.6 - 13.5	3.2	0.9 - 8.1	7.3	1.9 - 21.2	0.0	0.0 - 0.0	8.9	2.5 - 20.8	0.96
3170007	9866	RED CR	168.4	27.8	10 - 59	0.0	0.0 - 0.0	6.7	2.2 - 15.2	3.6	1.0 - 10.4	7.9	2.8 - 19.8	0.0	0.0 - 0.0	9.6	3.2 - 23.3	0.96
3170007	9867	FLINT CR	102.9	22.5	7 - 55	2.1	0.7 - 5.3	4.9	1.5 - 10.4	2.9	0.8 - 7.5	7.2	1.9 - 24.4	0.0	0.0 - 0.0	5.4	1.7 - 13.7	0.94
3170007	9868	RED CR	48.0	36.2	13 - 64	0.0	0.0 - 0.0	13.1	4.4 - 26.6	4.2	1.1 - 10.4	9.4	3.4 - 24.7	0.0	0.0 - 0.0	9.6	3.4 - 21.5	0.94
3170007	9869	RED CR	38.3	48.0	16 - 91	10.6	3.5 - 23.1	18.4	6.2 - 37.5	3.3	1.1 - 9.6	5.9	1.9 - 14.8	0.0	0.0 - 0.0	9.8	3.1 - 21.5	0.92
3170007	9870	RED CR	101.4	22.7	7 - 57	0.0	0.0 - 0.0	6.1	2.1 - 14.7	2.4	0.6 - 7.1	4.1	1.3 - 12.3	0.0	0.0 - 0.0	10.0	3.2 - 25.2	0.91
3170007	9871	RED CR, DOUBLE l	84.5	25.8	11 - 48	0.0	0.0 - 0.0	5.2	1.9 - 10.0	3.3	1.2 - 9.4	6.7	2.4 - 17.0	0.0	0.0 - 0.0	10.6	3.8 - 23.0	0.89
3170007	9872	RED CR	289.6	38.3	13 - 61	1.5	0.5 - 2.8	8.9	2.9 - 16.2	5.8	1.7 - 14.9	11.8	3.6 - 25.0	0.0	0.0 - 0.0	10.3	3.4 - 20.6	0.89
3170007	9873	KIRBY CR	66.2	27.8	10 - 63	0.0	0.0 - 0.0	6.4	2.3 - 15.0	3.8	1.2 - 10.4	7.5	2.6 - 22.0	0.0	0.0 - 0.0	10.0	3.8 - 27.2	0.91
3170007	9874	TENMILE CR	64.6	24.5	9 - 53	0.1	0.0 - 0.2	12.1	3.9 - 26.4	1.5	0.5 - 4.2	2.8	1.0 - 6.7	0.0	0.0 - 0.0	8.0	3.0 - 19.8	0.92
3170008	9875	ESCATAWPA R	40.5	415.3	159 - 921	323.9	125.4 - 772.6	44.7	17.3 - 91.5	1.0	0.3 - 2.9	1.7	0.5 - 4.5	0.0	0.0 - 0.0	44.1	14.6 - 106.6	1.00
3170008	9876	ESCATAWPA R	40.5	27.5	9 - 70	0.7	0.2 - 1.8	7.0	2.0 - 18.6	0.6	0.2 - 2.2	1.2	0.3 - 3.6	0.0	0.0 - 0.0	18.0	6.2 - 45.8	0.99
3170008	9877	FRANKLIN CR	97.4	58.0	22 - 120	0.0	0.0 - 0.0	13.9	5.8 - 31.2	5.2	1.8 - 13.4	27.6	11.1 - 73.5	0.0	0.0 - 0.0	11.1	4.9 - 24.9	0.99
3170008	9878	ESCATAWPA R	2.0	39.2	11 - 87	0.0	0.0 - 0.0	1.0	0.3 - 2.1	3.8	1.0 - 10.7	11.5	2.9 - 30.3	0.0	0.0 - 0.0	22.9	6.8 - 57.4	0.99
3170008	9879	JACKSON CR	107.0	51.0	16 - 97	0.0	0.0 - 0.0	8.9	2.8 - 17.6	5.1	1.6 - 13.2	26.3	8.3 - 61.9	0.0	0.0 - 0.0	10.7	3.4 - 21.5	0.99
3170008	9880	ESCATAWPA R	16.2	29.4	11 - 63	0.0	0.0 - 0.0	4.3	1.5 - 10.4	2.2	0.7 - 6.7	4.1	1.3 - 11.8	0.0	0.0 - 0.0	18.7	6.6 - 45.3	0.99
3170008	9881	BIG CR	44.0	47.8	15 - 85	0.0	0.0 - 0.0	3.1	1.0 - 5.9	5.3	1.7 - 11.6	24.5	7.4 - 58.5	0.0	0.0 - 0.0	14.9	5.1 - 29.1	0.98
3170008	9882	MILLR CR	28.0	33.8	11 - 77	0.0	0.0 - 0.0	6.4	2.2 - 14.4	2.8	0.9 - 7.3	14.4	4.1 - 41.6	0.0	0.0 - 0.0	10.2	3.7 - 26.7	0.95
3170008	9883	*A	28.6	52.4	17 - 122	0.0	0.0 - 0.0	7.5	2.5 - 16.1	5.3	1.8 - 14.3	27.5	9.6 - 68.3	0.0	0.0 - 0.0	12.1	4.6 - 32.0	0.90
3170008	9884	MILLR CR	62.2	57.7	20 - 142	0.0	0.0 - 0.0	27.8	10.0 - 62.3	3.0	0.9 - 9.0	15.2	5.4 - 40.4	0.0	0.0 - 0.0	11.8	4.1 - 30.9	0.90
3170008	9885	BIG CR	8.9	21.8	8 - 44	0.0	0.0 - 0.0	4.7	1.7 - 10.2	1.8	0.6 - 4.7	5.3	1.9 - 13.7	0.0	0.0 - 0.0	10.0	3.7 - 23.2	0.95
3170008	9886	BIG CR	95.6	40.5	17 - 78	0.0	0.0 - 0.0	18.8	6.8 - 39.0	1.9	0.7 - 5.7	8.9	3.0 - 23.2	0.0	0.0 - 0.0	10.9	4.0 - 24.5	0.94
3170008	9887	PASTURE CR	26.9	51.0	18 - 128	0.0	0.0 - 0.0	6.4	2.0 - 15.4	5.4	1.7 - 18.8	29.8	11.4 - 76.0	0.0	0.0 - 0.0	9.3	3.6 - 24.7	0.94
3170008	9888	ESCATAWPA R	123.9	45.1	15 - 107	0.0	0.0 - 0.0	5.3	1.7 - 12.7	6.1	1.8 - 17.9	19.1	5.9 - 53.1	0.0	0.0 - 0.0	14.7	5.2 - 38.4	0.98
3170008	9889	FLAT CR	52.7	41.4	16 - 104	0.0	0.0 - 0.0	5.6	2.0 - 14.1	4.3	1.3 - 11.1	21.2	7.8 - 59.9	0.0	0.0 - 0.0	10.3	4.2 - 25.6	0.97

8-digit HUC	ID	Name	Area	Catchment Yield		Point sources		Developed Land		Manure		Agricultural Land		Phosphate Mines		Soil parent rock		Frac
				kg/km^2	90% CI	kg/km^2	90% CI	kg/km^2	90% CI	kg/km^2	90% CI	kg/km^2	90% CI	kg/km^2	90% CI	kg/km^2	90% CI	
3170008	9890	ESCATAWPA R	97.7	56.0	19 - 127	0.0	0.0 - 0.0	7.4	2.5 - 14.9	6.7	1.8 - 19.4	26.2	7.9 - 76.8	0.0	0.0 - 0.0	15.8	5.7 - 43.2	0.97
3170008	9891	ESCATAWPA R	79.2	22.7	9 - 48	0.0	0.0 - 0.0	2.3	0.8 - 4.3	1.6	0.5 - 4.4	3.8	1.2 - 8.8	0.0	0.0 - 0.0	15.0	5.7 - 42.6	0.95
3170008	9892	PUPPY CR	109.1	31.7	11 - 71	0.0	0.0 - 0.0	6.7	2.4 - 16.0	2.1	0.7 - 6.1	9.1	3.1 - 22.6	0.0	0.0 - 0.0	13.8	4.9 - 35.7	0.94
3170008	9893	ESCATAWPA R	56.4	25.4	8 - 60	0.0	0.0 - 0.0	2.5	0.8 - 6.5	4.2	1.1 - 11.4	6.8	2.2 - 17.1	0.0	0.0 - 0.0	11.9	3.6 - 31.5	0.94
3170008	9894	ESCATAWPA R	40.9	26.7	7 - 66	0.0	0.0 - 0.0	6.4	1.9 - 14.1	1.7	0.4 - 5.3	5.3	1.5 - 14.0	0.0	0.0 - 0.0	13.4	3.9 - 36.6	0.93
3170008	9895	BENNETT CR	76.9	34.1	12 - 73	0.0	0.0 - 0.0	6.5	2.4 - 15.3	2.4	0.8 - 7.9	11.6	3.6 - 33.6	0.0	0.0 - 0.0	13.6	5.1 - 33.4	0.93
3170008	9896	ESCATAWPA R	66.8	24.5	8 - 45	0.0	0.0 - 0.0	5.5	1.6 - 10.6	3.1	0.8 - 7.5	6.7	2.3 - 14.6	0.0	0.0 - 0.0	9.2	3.1 - 20.2	0.93
3170008	9897	ESCATAWPA R	103.2	23.2	8 - 48	0.0	0.0 - 0.0	6.5	2.1 - 14.7	1.9	0.6 - 5.3	4.6	1.5 - 11.7	0.0	0.0 - 0.0	10.2	3.4 - 25.6	0.92
3170008	9898	PINE BRANCH CR	105.3	20.2	7 - 49	0.0	0.0 - 0.0	6.2	2.2 - 13.1	1.6	0.6 - 4.3	4.1	1.4 - 11.2	0.0	0.0 - 0.0	8.2	3.1 - 20.5	0.89
3170008	9899	ESCATAWPA R	60.5	26.4	8 - 64	0.0	0.0 - 0.0	6.2	2.2 - 14.4	3.4	1.0 - 9.3	8.2	2.8 - 21.9	0.0	0.0 - 0.0	8.6	3.0 - 25.4	0.89
3170008	9900	ESCATAWPA R	209.8	23.8	8 - 64	0.0	0.0 - 0.0	6.4	2.0 - 17.1	3.2	0.9 - 9.2	7.3	2.5 - 21.1	0.0	0.0 - 0.0	6.9	2.1 - 19.7	0.87
3170008	9901	BRUSHY CR	61.7	28.9	9 - 77	0.0	0.0 - 0.0	11.5	3.9 - 30.8	4.1	1.4 - 11.5	6.6	2.2 - 18.3	0.0	0.0 - 0.0	6.7	2.2 - 20.2	0.87
3170008	9902	POND CR	98.8	27.0	9 - 56	0.0	0.0 - 0.0	5.9	2.0 - 12.3	3.4	0.9 - 9.5	7.6	2.5 - 20.3	0.0	0.0 - 0.0	10.1	3.3 - 23.8	0.92
3170008	9903	NOBODIES CR	37.4	25.3	8 - 64	0.0	0.0 - 0.0	4.2	1.3 - 10.5	3.7	1.1 - 12.2	7.3	2.1 - 21.9	0.0	0.0 - 0.0	10.1	3.2 - 30.4	0.93
3170008	9904	BRUSHY CR	154.6	34.8	11 - 80	0.0	0.0 - 0.0	7.1	1.9 - 15.7	6.0	1.5 - 13.8	11.7	3.6 - 30.8	0.0	0.0 - 0.0	9.9	3.2 - 23.1	0.95
3170008	9905	BLACK CR	166.8	29.7	12 - 57	0.0	0.0 - 0.0	6.0	2.2 - 11.8	2.9	0.9 - 8.2	6.1	2.0 - 14.4	0.0	0.0 - 0.0	14.7	5.5 - 31.6	0.99
3170009	9906	OLD FOREST BAY(	116.3	42.2	13 - 87	0.0	0.0 - 0.0	16.6	5.1 - 34.4	2.6	0.8 - 7.6	5.5	1.5 - 15.7	0.0	0.0 - 0.0	17.6	5.9 - 47.0	1.00
3170009	9907	TCHOUTACABOUI	47.4	62.7	22 - 121	1.0	0.3 - 2.1	39.8	12.5 - 80.8	1.0	0.3 - 2.9	4.7	1.7 - 11.2	0.0	0.0 - 0.0	16.1	5.2 - 37.6	1.00
3170009	9908	CYPRESS CR	23.7	59.4	17 - 119	0.0	0.0 - 0.0	36.3	10.0 - 72.9	3.4	0.9 - 9.9	8.6	2.6 - 23.7	0.0	0.0 - 0.0	11.2	3.5 - 29.0	0.99
3170009	9909	TCHOUTACABOUI	5.4	26.9	9 - 47	0.0	0.0 - 0.0	9.6	2.8 - 20.1	1.5	0.4 - 3.9	5.1	1.6 - 12.8	0.0	0.0 - 0.0	10.7	3.8 - 25.1	0.99
3170009	9911	BAYOU COSTAPIA	81.8	108.4	35 - 233	67.8	23.1 - 164.0	11.8	3.5 - 26.7	3.4	0.9 - 10.8	7.2	2.2 - 20.0	0.0	0.0 - 0.0	18.2	5.7 - 51.1	0.98
3170009	9912	TCHOUTACABOUI	196.6	20.8	8 - 51	0.0	0.0 - 0.0	5.3	1.7 - 14.4	0.5	0.2 - 1.5	0.9	0.3 - 2.5	0.0	0.0 - 0.0	14.2	5.7 - 33.1	0.98
3170009	9913	TUXACHANIE CR	270.2	20.5	7 - 41	0.0	0.0 - 0.0	5.5	1.6 - 13.1	0.6	0.2 - 1.8	1.3	0.4 - 3.4	0.0	0.0 - 0.0	13.1	4.6 - 34.2	0.98
3170009	9914	BILOXI R	61.5	44.8	14 - 89	0.1	0.0 - 0.1	25.0	7.9 - 57.3	1.1	0.3 - 3.2	6.0	1.9 - 17.8	0.0	0.0 - 0.0	12.5	4.0 - 26.1	1.00
3170009	9915	BILOXI R	59.4	23.0	7 - 40	0.0	0.0 - 0.0	5.2	1.6 - 9.2	1.1	0.3 - 2.9	5.8	2.1 - 12.4	0.0	0.0 - 0.0	10.9	3.3 - 22.9	0.98
3170009	9916	SAUCER CR	120.5	28.1	9 - 63	0.0	0.0 - 0.0	10.6	3.3 - 24.8	1.2	0.4 - 3.8	4.0	1.2 - 9.8	0.0	0.0 - 0.0	12.2	3.8 - 28.3	0.95
3170009	9917	BILOXI R	11.1	35.9	14 - 87	0.0	0.0 - 0.0	14.3	5.7 - 33.6	1.8	0.6 - 4.4	7.8	2.7 - 22.3	0.0	0.0 - 0.0	12.1	5.3 - 28.9	0.95
3170009	9918	LITTLE BILOXI R	197.5	28.5	10 - 58	0.5	0.2 - 1.1	7.9	2.2 - 17.4	2.1	0.6 - 6.2	7.7	2.6 - 19.5	0.0	0.0 - 0.0	10.3	3.7 - 25.1	0.98
3170009	9919	WOLF R	161.7	44.1	13 - 78	0.0	0.0 - 0.1	11.8	3.2 - 23.4	2.4	0.6 - 6.5	12.6	3.5 - 32.9	0.0	0.0 - 0.0	17.3	5.3 - 43.6	1.00
3170009	9920	MURDER CR	83.6	33.8	10 - 76	0.0	0.0 - 0.0	3.8	1.1 - 7.9	4.5	1.2 - 13.6	14.1	4.1 - 39.5	0.0	0.0 - 0.0	11.4	3.5 - 24.0	0.93
3170009	9921	WOLF R	322.3	28.6	9 - 56	0.1	0.0 - 0.2	6.0	2.0 - 12.4	3.6	1.1 - 10.6	7.4	2.8 - 19.2	0.0	0.0 - 0.0	11.6	3.9 - 25.3	0.93
3170009	9922	JOURDAN R	19.9	496.2	145 - 1346	297.5	99.4 - 867.7	94.9	26.2 - 263.6	1.2	0.3 - 4.2	0.7	0.2 - 2.2	0.0	0.0 - 0.0	101.8	28.3 - 326.7	1.00
3170009	9923	JOURDAN R	17.3	257.7	92 - 559	99.1	38.1 - 224.8	17.1	6.1 - 39.3	7.5	2.5 - 24.0	29.8	12.1 - 79.1	0.0	0.0 - 0.0	104.2	37.1 - 264.0	0.99
3170009	9924	ROTTEN BAYOU	151.4	46.9	15 - 88	0.0	0.0 - 0.0	15.5	5.0 - 31.1	2.7	0.8 - 7.7	12.1	4.1 - 29.5	0.0	0.0 - 0.0	16.6	5.2 - 35.4	0.99
3170009	9925	JOURDAN R	77.9	67.4	25 - 151	1.4	0.5 - 3.2	12.7	4.7 - 30.6	3.9	1.3 - 13.7	14.7	5.7 - 39.1	0.0	0.0 - 0.0	34.7	11.3 - 88.3	0.99
3170009	9926	BAYOU BACON	154.3	44.1	16 - 79	0.2	0.1 - 0.4	6.9	2.5 - 13.6	3.9	1.3 - 10.4	16.6	5.5 - 37.4	0.0	0.0 - 0.0	16.6	6.7 - 31.9	0.97
3170009	9927	JOURDAN R	64.0	41.3	13 - 90	0.0	0.0 - 0.0	5.0	1.6 - 11.8	3.0	0.9 - 7.8	7.1	2.1 - 16.8	0.0	0.0 - 0.0	26.2	8.6 - 64.3	0.97
3170009	9928	JOURDAN R	7.0	33.4	13 - 73	0.0	0.0 - 0.0	4.5	1.6 - 9.4	2.1	0.7 - 5.8	1.6	0.5 - 3.6	0.0	0.0 - 0.0	25.2	9.1 - 65.2	0.95
3170009	9929	HICKORY CR	122.3	39.8	13 - 99	0.0	0.0 - 0.0	5.3	1.7 - 13.5	3.9	1.2 - 13.0	17.3	5.5 - 52.3	0.0	0.0 - 0.0	13.4	4.4 - 33.4	0.94

8-digit HUC	ID	Name	Area	Catchment Yield kg/km²	Catchment Yield 90% CI	Point sources kg/km²	Point sources 90% CI	Developed Land kg/km²	Developed Land 90% CI	Manure kg/km²	Manure 90% CI	Agricultural Land kg/km²	Agricultural Land 90% CI	Phosphate Mines kg/km²	Phosphate Mines 90% CI	Soil parent rock kg/km²	Soil parent rock 90% CI	Frac
3170009	9930	CATAHOULA CR	91.7	49.2	16 - 113	0.0	0.0 - 0.0	4.4	1.3 - 9.7	5.8	1.4 - 16.0	24.3	7.4 - 56.0	0.0	0.0 - 0.0	14.7	4.8 - 30.8	0.94
3170009	9931	MILL CR	66.9	62.8	21 - 145	0.0	0.0 - 0.0	7.5	2.5 - 17.4	7.8	2.3 - 22.8	31.7	10.9 - 76.1	0.0	0.0 - 0.0	15.8	4.8 - 40.4	0.95
3170009	9932	BAYOU LA CROIX	202.9	65.3	18 - 155	0.1	0.0 - 0.2	17.8	5.4 - 38.7	2.6	0.7 - 7.9	3.0	1.0 - 7.4	0.0	0.0 - 0.0	41.8	11.5 - 108.5	0.99
3170009	9933	BERNARD BAYOU	12.8	1507.0	476 - 3172	1380.3	450.4 - 2982.9	88.3	23.3 - 178.6	0.3	0.1 - 0.6	0.3	0.1 - 0.7	0.0	0.0 - 0.0	37.8	11.1 - 95.4	1.00
3170009	9934	BERNARD BAYOU	90.2	141.3	44 - 325	72.7	23.3 - 155.1	39.5	12.4 - 98.1	2.4	0.7 - 6.3	15.7	4.5 - 40.0	0.0	0.0 - 0.0	10.9	3.5 - 25.4	0.98
3170009	9935	TURKEY CR	84.7	68.8	26 - 141	0.4	0.1 - 0.9	34.3	11.8 - 77.1	1.8	0.6 - 5.3	9.5	3.0 - 25.4	0.0	0.0 - 0.0	22.7	7.9 - 46.6	0.98
3180001	9936	PEARL R	21.5	182.7	65 - 414	0.0	0.0 - 0.0	7.2	2.4 - 17.5	62.8	19.5 - 203.9	47.7	14.9 - 112.6	0.0	0.0 - 0.0	65.2	21.2 - 167.5	0.41
3180001	9937	TUSCOLAMETA CI	8.1	151.8	52 - 361	0.0	0.0 - 0.0	6.9	2.2 - 13.9	45.7	14.1 - 157.9	36.6	12.9 - 100.9	0.0	0.0 - 0.0	62.6	21.0 - 176.7	0.40
3180001	9938	CALUCTA CR	138.0	178.8	66 - 401	0.0	0.0 - 0.0	5.9	2.1 - 13.2	73.3	24.7 - 198.7	51.0	18.3 - 148.0	0.0	0.0 - 0.0	48.6	17.8 - 122.7	0.40
3180001	9939	TUSCOLAMETA CI	102.8	137.8	44 - 295	0.0	0.0 - 0.0	7.4	2.3 - 18.3	49.8	15.8 - 144.1	37.7	12.6 - 86.5	0.0	0.0 - 0.0	42.9	14.8 - 120.0	0.40
3180001	9940	SHOCKALOO CR	165.4	200.1	76 - 381	0.0	0.0 - 0.0	5.0	1.7 - 10.7	87.7	26.9 - 209.5	53.3	18.9 - 123.6	0.0	0.0 - 0.0	54.1	18.1 - 116.0	0.39
3180001	9941	TUSCOLAMETA CI	51.4	112.4	29 - 228	3.8	1.0 - 8.6	10.0	3.1 - 20.7	36.0	8.0 - 100.9	27.8	8.0 - 73.8	0.0	0.0 - 0.0	34.8	8.7 - 82.2	0.39
3180001	9942	TALABOGUE CR	42.6	204.9	65 - 461	0.0	0.0 - 0.0	7.0	2.1 - 15.9	94.8	27.7 - 270.9	58.7	16.5 - 146.1	0.0	0.0 - 0.0	44.5	12.6 - 112.0	0.38
3180001	9943	LITTLE R	31.2	257.9	67 - 594	0.0	0.0 - 0.0	6.9	2.0 - 17.2	125.6	31.3 - 351.7	75.9	19.8 - 196.3	0.0	0.0 - 0.0	49.4	13.3 - 131.3	0.36
3180001	9944	TALABOGUE CR	67.9	174.9	57 - 393	0.0	0.0 - 0.0	7.9	2.8 - 19.2	70.3	21.6 - 184.4	41.7	14.7 - 100.4	0.0	0.0 - 0.0	55.1	19.0 - 136.1	0.36
3180001	9945	TUSCOLAMETA CI	24.3	148.3	48 - 283	0.0	0.0 - 0.0	5.2	1.8 - 10.8	59.0	17.3 - 182.1	36.5	12.2 - 93.6	0.0	0.0 - 0.0	47.6	16.0 - 120.5	0.38
3180001	9946	HONTOKALO CR	165.6	245.0	89 - 535	63.6	22.0 - 165.2	11.0	3.7 - 24.6	79.1	25.8 - 224.0	47.5	16.6 - 115.7	0.0	0.0 - 0.0	43.8	15.4 - 111.8	0.38
3180001	9947	TUSCOLAMETA CI	1.9	455.5	159 - 944	0.0	0.0 - 0.0	0.0	0.0 - 0.0	242.5	79.2 - 587.9	142.8	46.7 - 311.1	0.0	0.0 - 0.0	70.2	23.3 - 151.4	0.38
3180001	9948	TUSCOLAMETA CI	221.9	131.2	38 - 309	0.4	0.1 - 1.0	6.3	1.8 - 13.9	53.5	16.5 - 185.1	35.6	10.1 - 103.4	0.0	0.0 - 0.0	35.4	11.3 - 87.1	0.38
3180001	9949	TUSCOLAMETA CI	90.3	133.4	51 - 260	0.0	0.0 - 0.0	6.9	2.6 - 14.1	47.3	15.0 - 130.6	40.7	15.1 - 90.8	0.0	0.0 - 0.0	38.5	13.5 - 91.4	0.35
3180001	9950	CONEHATTA CR	60.7	88.5	30 - 201	0.0	0.0 - 0.0	5.8	2.0 - 14.0	24.0	7.5 - 86.3	20.9	6.2 - 50.1	0.0	0.0 - 0.0	37.8	12.8 - 111.3	0.35
3180001	9951	BRUSHY CR	29.6	85.0	26 - 218	0.0	0.0 - 0.0	5.4	1.7 - 12.2	20.8	5.5 - 63.9	17.3	5.4 - 50.0	0.0	0.0 - 0.0	41.5	12.0 - 121.7	0.33
3180001	9952	CONEHATTA CR	49.0	107.4	35 - 249	0.0	0.0 - 0.0	7.0	2.3 - 16.0	38.4	11.9 - 114.8	33.5	10.8 - 83.3	0.0	0.0 - 0.0	28.5	10.1 - 82.5	0.33
3180001	9953	SIPSEY CR	237.1	134.9	42 - 324	21.6	7.2 - 56.1	7.6	2.2 - 18.9	45.9	12.5 - 137.3	33.1	10.8 - 92.2	0.0	0.0 - 0.0	26.8	7.5 - 70.2	0.38
3180001	9954	PEARL R	198.3	123.4	41 - 214	4.8	1.8 - 9.8	13.5	4.7 - 26.3	40.7	13.2 - 93.7	31.7	10.1 - 72.9	0.0	0.0 - 0.0	32.7	12.4 - 73.4	0.40
3180001	9955	PEARL R	1.5	112.7	34 - 216	0.0	0.0 - 0.0	0.0	0.0 - 0.0	16.7	4.6 - 40.4	16.1	4.7 - 36.5	0.0	0.0 - 0.0	79.9	24.2 - 172.8	0.40
3180001	9956	STANDING PINE C	154.3	116.3	44 - 246	0.0	0.0 - 0.0	7.5	2.9 - 17.3	45.1	13.5 - 122.8	35.0	12.5 - 95.7	0.0	0.0 - 0.0	28.6	10.4 - 68.2	0.40
3180001	9957	PEARL R	129.1	81.4	27 - 154	0.0	0.0 - 0.1	5.4	1.9 - 11.2	27.2	7.5 - 68.8	21.1	6.5 - 46.2	0.0	0.0 - 0.0	27.6	8.7 - 65.2	0.40
3180001	9958	BEASHA CR	143.2	83.4	30 - 160	0.0	0.0 - 0.0	6.6	2.3 - 12.2	32.5	10.5 - 81.1	23.7	8.7 - 55.3	0.0	0.0 - 0.0	20.6	7.2 - 46.7	0.39
3180001	9959	PEARL R	46.1	54.6	18 - 123	0.0	0.0 - 0.0	7.3	2.2 - 17.3	15.8	4.7 - 48.2	11.5	3.3 - 33.1	0.0	0.0 - 0.0	20.0	7.1 - 50.3	0.39
3180001	9960	PEARL R	31.3	45.2	16 - 94	0.0	0.0 - 0.0	4.0	1.3 - 8.7	11.7	3.6 - 35.6	9.0	3.4 - 20.7	0.0	0.0 - 0.0	20.4	7.4 - 44.5	0.39
3180001	9961	KENTAWAH CR	129.3	87.2	30 - 230	1.2	0.4 - 3.7	20.2	7.2 - 50.5	28.9	8.2 - 92.3	21.4	7.0 - 62.1	0.0	0.0 - 0.0	15.5	5.8 - 44.8	0.38
3180001	9962	COONSHUCK CR	51.7	82.3	33 - 178	0.0	0.0 - 0.0	8.2	3.3 - 18.6	30.4	11.2 - 86.4	22.6	8.9 - 54.5	0.0	0.0 - 0.0	21.1	7.9 - 47.0	0.37
3180001	9963	FULLTON CR	70.0	59.1	22 - 112	0.0	0.0 - 0.0	8.4	2.5 - 17.3	19.2	5.9 - 55.1	14.0	4.7 - 38.3	0.0	0.0 - 0.0	17.5	6.4 - 35.9	0.35
3180001	9964	COONSHUCK CR	48.2	60.1	20 - 128	0.0	0.0 - 0.0	6.5	2.1 - 15.1	24.6	6.9 - 59.9	17.7	5.9 - 50.9	0.0	0.0 - 0.0	11.3	4.2 - 24.6	0.35
3180001	9965	CUSHTUSIA CR	132.7	83.4	26 - 166	0.0	0.0 - 0.0	6.6	2.1 - 13.3	36.3	10.4 - 86.3	26.4	7.4 - 63.2	0.0	0.0 - 0.0	14.1	4.3 - 31.0	0.37
3180001	9967	PEARL R	25.1	86.6	32 - 174	0.0	0.0 - 0.0	4.2	1.6 - 8.8	31.6	10.2 - 80.8	23.5	8.1 - 59.7	0.0	0.0 - 0.0	27.4	10.7 - 80.0	0.38
3180001	9968	PEARL R	176.9	89.2	24 - 237	3.7	1.1 - 9.8	8.4	2.0 - 21.6	35.5	7.9 - 112.3	26.2	7.2 - 77.8	0.0	0.0 - 0.0	15.4	4.6 - 39.6	0.38
3180001	9969	PEARL R	53.5	99.9	28 - 176	0.0	0.0 - 0.0	4.6	1.5 - 8.7	34.1	9.0 - 77.7	42.0	13.2 - 91.2	0.0	0.0 - 0.0	19.2	6.0 - 38.0	0.37

8-digit HUC	ID	Name	Area	Catchment Yield		Point sources		Developed Land		Manure		Agricultural Land		Phosphate Mines		Soil parent rock		Frac
				kg/km^2	90% CI	kg/km^2	90% CI	kg/km^2	90% CI	kg/km^2	90% CI	kg/km^2	90% CI	kg/km^2	90% CI	kg/km^2	90% CI	
3180001	9970	BOGUE CHITTO	333.2	82.2	24 - 160	0.0	0.0 - 0.0	6.2	1.8 - 14.3	25.1	6.5 - 70.7	32.7	9.7 - 87.1	0.0	0.0 - 0.0	18.2	6.2 - 40.4	0.37
3180001	9971	PEARL R	16.3	73.1	24 - 138	0.0	0.0 - 0.0	5.3	1.7 - 11.2	14.6	4.5 - 36.3	35.5	11.8 - 85.1	0.0	0.0 - 0.0	17.7	5.6 - 42.6	0.37
3180001	9972	PEARL R	158.7	82.3	23 - 151	0.0	0.0 - 0.0	6.2	1.8 - 11.7	12.1	3.2 - 31.5	45.1	13.2 - 91.7	0.0	0.0 - 0.0	18.9	5.9 - 38.0	0.37
3180001	9973	NANWAYA CR	143.7	77.8	26 - 202	0.0	0.0 - 0.0	8.4	2.7 - 19.3	10.2	3.0 - 31.0	39.9	12.7 - 114.0	0.0	0.0 - 0.0	19.3	6.2 - 54.6	0.34
3180001	9974	PEARL R	59.0	128.0	41 - 302	14.1	4.4 - 37.6	13.8	4.7 - 37.7	15.1	4.2 - 43.7	58.1	17.7 - 185.4	0.0	0.0 - 0.0	26.8	9.2 - 83.9	0.34
3180001	9975	TALLAHAGA CR	311.5	84.8	35 - 191	4.0	1.6 - 8.2	9.1	3.3 - 21.1	9.9	3.3 - 24.2	38.7	15.0 - 96.2	0.0	0.0 - 0.0	23.1	9.2 - 54.6	0.37
3180001	9976	NOXAPATER CR	134.7	49.5	19 - 114	0.3	0.1 - 0.8	5.7	1.8 - 13.6	8.1	2.6 - 25.0	21.5	7.5 - 57.6	0.0	0.0 - 0.0	13.9	5.6 - 38.7	0.37
3180001	9977	PINISHOOK CR	127.4	44.9	15 - 109	0.0	0.0 - 0.0	5.6	1.9 - 13.4	9.1	2.9 - 26.3	15.6	5.4 - 43.6	0.0	0.0 - 0.0	14.5	4.5 - 39.2	0.38
3180001	9978	HURRICANE CR	59.0	57.4	19 - 131	0.0	0.0 - 0.0	6.2	1.8 - 16.3	19.5	6.0 - 55.5	16.2	5.2 - 45.2	0.0	0.0 - 0.0	15.5	4.9 - 37.4	0.38
3180001	9979	LUKFAPA CR	53.4	34.6	12 - 83	0.0	0.0 - 0.0	5.9	1.8 - 13.2	6.8	2.0 - 18.7	5.2	1.9 - 13.4	0.0	0.0 - 0.0	16.7	6.6 - 38.5	0.39
3180001	9980	LOBUTCHA CR	49.8	110.3	31 - 259	0.0	0.0 - 0.0	6.3	1.9 - 16.3	41.5	10.4 - 116.7	31.9	9.6 - 74.7	0.0	0.0 - 0.0	30.6	9.0 - 85.0	0.40
3180001	9981	LOBUTCHA CR	149.6	59.7	16 - 123	0.0	0.0 - 0.0	6.3	1.6 - 12.8	17.5	4.5 - 50.7	15.0	4.3 - 40.1	0.0	0.0 - 0.0	21.0	5.8 - 49.6	0.39
3180001	9982	LOBUTCHA CR	449.6	33.8	13 - 86	0.0	0.0 - 0.0	5.5	1.6 - 13.5	2.7	0.8 - 7.6	8.1	3.0 - 22.1	0.0	0.0 - 0.0	17.4	6.1 - 47.8	0.38
3180001	9983	PAILEY CR	79.7	51.0	15 - 87	0.0	0.0 - 0.0	5.7	1.6 - 10.3	5.5	1.2 - 14.1	15.2	4.7 - 34.8	0.0	0.0 - 0.0	24.6	7.4 - 49.8	0.38
3180001	9984	COBBS CR	88.9	412.9	131 - 921	302.8	96.8 - 701.4	8.3	2.8 - 17.2	42.4	13.0 - 103.2	32.4	10.3 - 82.7	0.0	0.0 - 0.0	26.9	8.9 - 71.7	0.39
3180001	9985	YOCKANOOKANY	162.0	101.3	30 - 203	0.0	0.0 - 0.0	8.3	2.7 - 19.4	28.2	7.7 - 66.2	28.6	8.6 - 65.4	0.0	0.0 - 0.0	36.2	9.5 - 86.4	0.41
3180001	9986	TIBBEY CR	142.0	29.4	10 - 65	0.0	0.0 - 0.0	5.5	2.0 - 11.7	4.4	1.1 - 11.7	9.1	2.9 - 23.5	0.0	0.0 - 0.0	10.4	3.4 - 25.6	0.37
3180001	9987	BESA CHITO CR	198.7	48.8	15 - 86	1.3	0.4 - 2.7	8.6	2.4 - 15.9	7.6	2.0 - 19.0	16.7	6.0 - 36.2	0.0	0.0 - 0.0	14.6	4.1 - 31.8	0.37
3180002	9988	STRONG R	26.2	53.1	21 - 105	0.0	0.0 - 0.0	5.7	2.3 - 13.6	21.6	7.9 - 60.7	11.8	3.9 - 29.4	0.0	0.0 - 0.0	14.1	5.3 - 32.1	0.90
3180002	9989	STRONG R	358.4	67.1	21 - 137	0.4	0.1 - 0.9	6.7	2.5 - 14.3	26.4	7.4 - 77.1	16.5	6.2 - 40.6	0.0	0.0 - 0.0	17.2	5.3 - 43.4	0.89
3180002	9990	SELLER'S CR	88.9	69.9	25 - 152	0.7	0.3 - 1.5	14.4	5.0 - 32.4	25.2	7.8 - 73.4	15.2	6.0 - 46.0	0.0	0.0 - 0.0	14.5	5.8 - 39.7	0.87
3180002	9993	STRONG R	302.9	61.4	23 - 161	0.0	0.0 - 0.0	6.0	2.1 - 17.9	24.2	7.3 - 86.7	18.9	7.3 - 57.5	0.0	0.0 - 0.0	12.3	5.1 - 38.3	0.86
3180002	9994	STRONG R	5.8	277.1	87 - 574	0.0	0.0 - 0.0	8.4	2.7 - 18.5	166.7	50.9 - 524.5	76.0	22.4 - 228.9	0.0	0.0 - 0.0	26.1	9.6 - 58.8	0.82
3180002	9995	STRONG R	125.9	76.8	22 - 184	0.0	0.0 - 0.0	5.1	1.4 - 12.7	37.3	10.1 - 115.5	17.1	5.2 - 45.9	0.0	0.0 - 0.0	17.2	5.0 - 44.7	0.81
3180002	9996	CANEY CR	98.2	76.1	22 - 164	0.0	0.0 - 0.0	4.1	1.3 - 8.0	21.1	5.8 - 55.1	11.9	3.3 - 28.5	0.0	0.0 - 0.0	39.0	13.6 - 92.1	0.76
3180002	9997	STRONG R	164.1	148.0	45 - 386	18.0	5.8 - 39.0	7.1	2.3 - 16.5	49.1	13.2 - 165.6	28.7	7.9 - 70.2	0.0	0.0 - 0.0	45.1	12.9 - 132.5	0.76
3180002	9998	RASBERRY CR	80.2	103.4	32 - 197	0.0	0.0 - 0.0	5.8	1.7 - 11.2	44.8	11.9 - 117.6	34.9	10.4 - 78.4	0.0	0.0 - 0.0	17.9	5.9 - 42.4	0.81
3180002	9999	PURUIS CR	78.6	71.3	24 - 170	0.0	0.0 - 0.0	4.4	1.6 - 10.2	19.5	6.2 - 65.1	31.7	9.7 - 89.0	0.0	0.0 - 0.0	15.7	5.2 - 39.0	0.82
3180002	10000	CAMPBELL CR	151.6	57.7	22 - 100	0.0	0.0 - 0.0	3.5	1.2 - 6.7	16.0	5.2 - 37.6	21.8	7.8 - 56.7	0.0	0.0 - 0.0	16.4	6.4 - 34.0	0.86
3180002	10001	DABBS CR	159.3	51.1	16 - 123	0.4	0.1 - 1.0	5.9	1.7 - 15.3	11.7	3.1 - 33.8	14.0	4.1 - 37.1	0.0	0.0 - 0.0	19.1	5.9 - 49.7	0.87
3180002	10002	BIG CR	133.0	49.4	15 - 126	0.0	0.0 - 0.0	5.2	1.7 - 14.0	19.3	5.4 - 62.4	12.0	4.0 - 35.5	0.0	0.0 - 0.0	12.8	3.8 - 33.3	0.89
3180002	10003	PEARL R	7.3	80.7	29 - 143	17.7	6.0 - 36.0	17.3	5.7 - 35.9	15.4	4.4 - 35.5	14.0	4.5 - 35.0	0.0	0.0 - 0.0	16.3	6.1 - 38.8	0.90
3180002	10004	LIMESTONE CR	101.0	42.6	14 - 111	0.0	0.0 - 0.0	6.0	1.8 - 13.9	13.3	3.7 - 41.2	8.3	2.6 - 23.5	0.0	0.0 - 0.0	14.9	5.1 - 43.6	0.90
3180002	10005	PEARL R	839.3	162.1	48 - 359	5.8	1.9 - 12.8	11.2	3.3 - 22.6	29.5	7.5 - 94.9	62.6	19.7 - 183.9	0.0	0.0 - 0.0	52.9	15.3 - 138.9	0.90
3180002	10006	RICHLAND CR	143.6	234.2	84 - 536	0.0	0.0 - 0.0	49.1	17.5 - 109.3	22.5	6.7 - 66.0	38.2	12.5 - 110.3	0.0	0.0 - 0.0	124.5	45.2 - 296.2	0.87
3180002	10007	TUMBALOO CR	116.5	121.1	38 - 263	0.1	0.0 - 0.2	9.0	3.2 - 21.9	23.0	6.5 - 70.9	37.4	10.9 - 107.1	0.0	0.0 - 0.0	51.6	16.7 - 124.6	0.82
3180002	10008	RICHLAND CR	95.1	151.5	44 - 304	0.0	0.0 - 0.0	17.3	5.6 - 37.9	25.6	6.5 - 71.8	42.6	11.8 - 121.4	0.0	0.0 - 0.0	66.1	21.0 - 161.9	0.82
3180002	10009	PEARL R	359.4	276.7	92 - 727	1.5	0.6 - 3.6	88.0	28.1 - 229.6	8.2	2.3 - 23.7	25.2	8.3 - 78.9	0.0	0.0 - 0.0	153.8	48.3 - 520.1	0.87
3180002	10010	PELAHATCHIE CR	46.8	66.7	22 - 177	0.0	0.0 - 0.0	2.1	0.7 - 5.9	11.9	3.4 - 39.4	20.1	7.1 - 58.2	0.0	0.0 - 0.0	32.5	10.0 - 106.6	0.40

8-digit HUC	ID	Name	Area	Catchment Yield		Point sources		Developed Land		Manure		Agricultural Land		Phosphate Mines		Soil parent rock		Frac
				kg/km^2	90% CI	kg/km^2	90% CI	kg/km^2	90% CI	kg/km^2	90% CI	kg/km^2	90% CI	kg/km^2	90% CI	kg/km^2	90% CI	
3180002	10011	CLEAR CR	99.6	179.0	53 - 395	0.0	0.0 - 0.0	4.2	1.3 - 9.8	39.7	9.2 - 109.8	60.7	19.9 - 172.4	0.0	0.0 - 0.0	74.4	21.2 - 195.0	0.40
3180002	10012	COFFEE BOGUE	127.4	201.3	71 - 436	0.0	0.0 - 0.0	5.7	1.9 - 13.5	81.7	26.9 - 214.7	51.6	17.8 - 133.2	0.0	0.0 - 0.0	62.3	21.4 - 188.0	0.41
3180002	10013	PEARL R	81.1	123.4	44 - 269	0.0	0.0 - 0.0	7.1	2.6 - 15.0	27.6	8.9 - 85.1	36.0	11.3 - 96.4	0.0	0.0 - 0.0	52.7	17.8 - 131.4	0.41
3180002	10014	EUTACUTACHEE C	60.7	68.5	25 - 123	0.0	0.0 - 0.0	7.3	2.7 - 13.2	16.5	5.6 - 42.7	27.1	10.5 - 57.5	0.0	0.0 - 0.0	17.5	6.1 - 43.1	0.39
3180002	10015	PELAHATCHIE CR	209.5	123.5	46 - 286	2.6	0.9 - 6.7	9.3	3.2 - 23.3	37.2	13.2 - 103.8	38.5	11.6 - 101.1	0.0	0.0 - 0.0	35.9	14.3 - 93.5	0.39
3180003	10017	HOLIDAY CR	230.8	44.1	17 - 90	0.7	0.3 - 1.6	7.6	2.6 - 15.5	9.3	2.9 - 27.9	15.5	5.0 - 38.9	0.0	0.0 - 0.0	11.0	4.2 - 25.3	0.94
3180003	10018	PEARL R	113.8	37.1	13 - 88	0.0	0.0 - 0.0	9.2	2.8 - 23.3	6.5	2.3 - 20.1	9.6	3.4 - 25.5	0.0	0.0 - 0.0	11.7	4.2 - 32.0	0.94
3180003	10019	GREENS CR	158.8	45.7	13 - 87	0.0	0.0 - 0.0	6.2	1.7 - 12.6	8.1	2.0 - 18.6	17.0	5.7 - 42.0	0.0	0.0 - 0.0	14.4	4.3 - 31.8	0.93
3180003	10020	PEARL R	31.3	27.8	9 - 50	0.0	0.0 - 0.0	6.1	1.9 - 11.0	4.3	1.1 - 11.6	5.5	1.9 - 14.7	0.0	0.0 - 0.0	11.9	3.6 - 27.2	0.93
3180003	10021	PEARL R	10.6	55.7	22 - 113	0.0	0.0 - 0.0	4.5	1.7 - 8.3	14.2	4.8 - 33.3	18.3	7.3 - 45.5	0.0	0.0 - 0.0	18.7	7.0 - 40.1	0.93
3180003	10022	WHITE SAND CR	34.6	62.3	21 - 145	0.0	0.0 - 0.0	6.9	2.3 - 14.1	17.2	5.3 - 45.7	21.6	7.0 - 56.1	0.0	0.0 - 0.0	16.6	6.0 - 42.1	0.93
3180003	10023	WHITE SAND CR	20.9	35.7	9 - 73	0.0	0.0 - 0.0	6.6	1.9 - 13.4	5.5	1.4 - 13.8	11.3	3.5 - 25.1	0.0	0.0 - 0.0	12.4	3.0 - 28.1	0.90
3180003	10024	LITTLE WHITE SAI	123.5	36.5	13 - 75	0.0	0.0 - 0.0	7.5	2.6 - 15.5	5.4	1.6 - 12.4	11.3	3.8 - 26.5	0.0	0.0 - 0.0	12.3	4.3 - 29.7	0.87
3180003	10025	WHITE SAND CR	137.8	52.5	18 - 138	4.1	1.3 - 10.6	11.7	3.7 - 29.3	8.1	2.3 - 25.4	17.0	5.6 - 48.2	0.0	0.0 - 0.0	11.5	4.1 - 35.3	0.87
3180003	10026	DRY CR	44.1	43.4	13 - 104	0.0	0.0 - 0.0	8.2	2.7 - 22.1	8.3	2.2 - 23.5	14.9	4.1 - 38.0	0.0	0.0 - 0.0	11.9	4.4 - 25.6	0.90
3180003	10027	PEARL R	152.2	63.7	21 - 162	0.3	0.1 - 0.7	7.3	2.5 - 18.6	16.3	4.7 - 48.8	20.1	6.6 - 58.7	0.0	0.0 - 0.0	19.8	6.8 - 51.0	0.93
3180003	10028	SILVER CR	67.6	54.9	19 - 114	0.0	0.0 - 0.0	6.4	1.9 - 13.1	14.5	4.2 - 37.9	18.6	5.3 - 43.5	0.0	0.0 - 0.0	15.4	5.2 - 42.9	0.92
3180003	10029	HOOKER HOLLOW	41.2	36.7	14 - 69	0.0	0.0 - 0.0	7.6	2.6 - 16.9	5.7	1.9 - 17.0	11.6	4.3 - 25.7	0.0	0.0 - 0.0	11.8	4.5 - 27.6	0.87
3180003	10030	SILVER CR	50.4	55.3	17 - 104	1.1	0.3 - 2.2	9.7	3.3 - 21.4	12.8	3.9 - 32.9	16.5	4.9 - 40.7	0.0	0.0 - 0.0	15.2	4.7 - 35.3	0.87
3180003	10031	SILVER CR, E PROI	149.0	53.0	17 - 114	0.0	0.0 - 0.0	6.4	2.0 - 13.1	15.9	4.1 - 49.5	19.4	5.3 - 51.4	0.0	0.0 - 0.0	11.4	3.2 - 30.9	0.81
3180003	10032	SILVER CR	124.4	107.4	35 - 232	0.3	0.1 - 0.8	7.5	2.5 - 17.8	45.7	13.6 - 113.9	33.6	10.8 - 98.3	0.0	0.0 - 0.0	20.2	7.0 - 50.0	0.81
3180003	10033	PEARL R	14.5	74.3	24 - 153	0.0	0.0 - 0.0	5.0	1.6 - 9.9	21.9	6.5 - 56.4	27.7	8.8 - 79.2	0.0	0.0 - 0.0	19.7	6.6 - 49.0	0.92
3180003	10034	PEARL R	4.7	144.4	59 - 363	0.0	0.0 - 0.0	24.6	9.1 - 57.7	44.1	15.1 - 126.7	54.7	20.3 - 158.5	0.0	0.0 - 0.0	21.0	8.6 - 57.0	0.92
3180003	10035	CROOKED CR	68.1	62.3	21 - 120	0.0	0.0 - 0.0	7.3	2.5 - 16.6	14.0	3.8 - 44.7	17.1	5.2 - 48.5	0.0	0.0 - 0.0	23.8	7.5 - 57.0	0.91
3180003	10036	PEARL R	2.0	66.7	21 - 147	0.0	0.0 - 0.0	10.3	3.8 - 23.5	16.2	5.0 - 52.3	22.8	8.5 - 66.4	0.0	0.0 - 0.0	17.5	6.0 - 43.5	0.91
3180003	10037	PEARL R	50.3	48.4	16 - 85	0.0	0.0 - 0.0	4.8	1.6 - 9.1	10.5	2.8 - 28.8	13.2	3.9 - 30.6	0.0	0.0 - 0.0	19.9	6.5 - 43.9	0.91
3180003	10038	BAHALA CR	70.3	76.7	23 - 150	0.0	0.0 - 0.0	5.4	1.6 - 11.4	18.4	5.1 - 47.5	26.5	7.9 - 66.1	0.0	0.0 - 0.0	26.3	7.5 - 65.1	0.91
3180003	10039	BAHALA CR	220.5	111.3	41 - 252	2.1	0.8 - 5.1	7.0	2.7 - 15.9	20.7	6.6 - 67.8	46.1	16.1 - 113.9	0.0	0.0 - 0.0	35.3	12.7 - 89.0	0.88
3180003	10040	LITTLE BAHALA C	135.1	63.6	20 - 145	0.0	0.0 - 0.0	9.4	3.4 - 23.2	11.9	3.6 - 36.4	23.7	7.4 - 65.4	0.0	0.0 - 0.0	18.6	5.2 - 50.3	0.88
3180003	10041	FAIR R	343.8	60.5	19 - 112	0.0	0.0 - 0.0	6.3	2.1 - 12.5	12.9	4.0 - 30.5	22.3	6.0 - 48.4	0.0	0.0 - 0.0	19.0	5.7 - 51.3	0.91
3180003	10042	HALLS CR	117.3	101.1	33 - 202	2.5	0.9 - 5.6	8.4	2.5 - 18.5	28.9	8.8 - 72.0	36.3	11.5 - 97.7	0.0	0.0 - 0.0	25.0	8.4 - 61.9	0.92
3180003	10043	TILTON CR	103.9	48.6	16 - 107	0.0	0.0 - 0.0	7.7	2.6 - 16.6	10.9	3.2 - 28.8	13.5	3.7 - 39.2	0.0	0.0 - 0.0	16.5	5.5 - 38.9	0.93
3180004	10044	PEARL R	321.0	79.6	26 - 202	13.5	4.3 - 33.7	13.6	3.8 - 41.3	4.0	1.1 - 11.3	12.1	3.3 - 33.7	0.0	0.0 - 0.0	36.4	11.2 - 95.9	1.00
3180004	10045	HOBOLOCHITO R	27.4	124.8	50 - 257	36.1	13.9 - 76.3	19.4	7.3 - 41.0	8.6	2.8 - 25.3	28.2	10.4 - 67.2	0.0	0.0 - 0.0	32.5	12.3 - 84.0	0.99
3180004	10046	E HOBOLOCHITO I	299.2	47.3	17 - 94	1.6	0.6 - 3.7	13.5	4.7 - 29.5	4.4	1.4 - 12.4	14.8	4.6 - 37.6	0.0	0.0 - 0.0	12.9	4.4 - 30.0	0.97
3180004	10047	HOBOLOCHITO R	349.1	60.5	22 - 137	0.2	0.1 - 0.5	8.3	3.0 - 19.2	8.0	2.3 - 24.7	29.1	10.3 - 76.4	0.0	0.0 - 0.0	14.9	5.5 - 40.0	0.97
3180004	10048	WHITE SAND CR	67.0	38.2	14 - 75	0.0	0.0 - 0.0	4.9	1.8 - 9.5	5.0	1.5 - 14.3	17.3	5.5 - 40.1	0.0	0.0 - 0.0	11.0	4.1 - 22.9	0.87
3180004	10049	HOBOLOCHITO R	182.2	33.5	13 - 77	0.0	0.0 - 0.0	2.0	0.7 - 4.9	4.9	1.8 - 13.6	15.2	5.5 - 39.5	0.0	0.0 - 0.0	11.5	4.6 - 27.7	0.87
3180004	10050	PEARL R	6.1	74.4	21 - 151	0.0	0.0 - 0.0	4.0	1.1 - 7.5	6.9	1.6 - 16.6	16.8	4.4 - 35.0	0.0	0.0 - 0.0	46.7	13.4 - 104.9	0.99

8-digit HUC	ID	Name	Area	Catchment Yield kg/km^2	Catchment Yield 90% CI	Point sources kg/km^2	Point sources 90% CI	Developed Land kg/km^2	Developed Land 90% CI	Manure kg/km^2	Manure 90% CI	Agricultural Land kg/km^2	Agricultural Land 90% CI	Phosphate Mines kg/km^2	Phosphate Mines 90% CI	Soil parent rock kg/km^2	Soil parent rock 90% CI	Frac
3180004	10051	PEARL R	320.5	53.2	19 - 114	0.0	0.0 - 0.0	9.1	3.0 - 18.7	7.4	2.2 - 20.6	21.5	6.7 - 60.1	0.0	0.0 - 0.0	15.2	4.7 - 36.8	0.98
3180004	10052	PEARL R	6.0	24.9	7 - 61	0.0	0.0 - 0.0	3.1	1.1 - 6.5	0.9	0.3 - 2.3	1.9	0.6 - 4.6	0.0	0.0 - 0.0	19.1	5.1 - 48.6	0.97
3180004	10054	CLEAR CR	106.6	26.8	9 - 57	0.0	0.0 - 0.1	8.0	2.6 - 16.5	3.5	1.1 - 10.9	4.1	1.5 - 11.9	0.0	0.0 - 0.0	11.1	4.1 - 26.8	0.96
3180004	10055	PEARL R	184.0	48.5	19 - 110	0.0	0.0 - 0.0	7.7	2.6 - 17.1	12.1	3.9 - 31.6	16.6	5.3 - 50.9	0.0	0.0 - 0.0	12.2	4.4 - 33.7	0.96
3180004	10056	LOWER LITTLE CR	131.6	34.1	10 - 71	0.0	0.0 - 0.0	8.0	2.6 - 17.6	6.5	1.8 - 16.6	8.0	2.6 - 20.3	0.0	0.0 - 0.0	11.6	3.9 - 26.4	0.95
3180004	10057	LOWER LITTLE CR	150.7	22.6	9 - 47	0.0	0.0 - 0.0	7.0	2.4 - 13.3	3.9	1.2 - 9.8	4.5	1.5 - 12.9	0.0	0.0 - 0.0	7.3	2.9 - 19.1	0.88
3180004	10058	GULLY CR	59.8	27.4	9 - 50	0.0	0.0 - 0.0	6.3	2.3 - 12.3	6.0	1.8 - 15.0	8.4	2.6 - 18.5	0.0	0.0 - 0.0	6.7	2.5 - 15.1	0.88
3180004	10059	PEARL R	43.9	58.0	27 - 111	0.0	0.0 - 0.0	9.9	3.9 - 23.6	15.3	5.4 - 40.8	21.2	9.2 - 46.9	0.0	0.0 - 0.0	11.6	4.9 - 25.4	0.95
3180004	10060	UPPER LITTLE CR	88.3	41.3	13 - 91	0.0	0.0 - 0.0	7.7	2.5 - 18.6	8.8	2.4 - 27.2	11.6	4.0 - 28.6	0.0	0.0 - 0.0	13.1	4.5 - 32.2	0.95
3180004	10061	UPPER LITTLE CR	192.6	48.6	14 - 110	0.0	0.0 - 0.0	8.4	2.5 - 17.9	11.0	3.0 - 32.7	15.9	5.7 - 37.4	0.0	0.0 - 0.0	13.2	4.6 - 30.8	0.87
3180004	10062	BEAVERDAM CR	49.3	49.3	16 - 111	0.0	0.0 - 0.0	9.5	3.0 - 22.1	12.5	3.9 - 33.7	17.8	5.9 - 50.4	0.0	0.0 - 0.0	9.4	3.6 - 22.0	0.87
3180004	10063	PEARL R	3.5	48.1	19 - 91	0.0	0.0 - 0.0	10.5	4.1 - 20.3	9.1	2.8 - 26.7	12.2	4.8 - 27.9	0.0	0.0 - 0.0	16.3	6.0 - 35.2	0.95
3180004	10064	PEARL R	272.5	67.3	21 - 145	9.7	3.5 - 25.8	18.3	5.2 - 45.9	11.6	3.3 - 32.6	16.5	4.8 - 43.0	0.0	0.0 - 0.0	11.1	3.9 - 28.5	0.95
3180004	10065	HARPER'S CR	34.8	37.5	12 - 74	0.0	0.0 - 0.0	7.5	2.3 - 14.3	8.8	2.7 - 26.3	12.1	3.9 - 30.0	0.0	0.0 - 0.0	9.0	2.7 - 23.0	0.94
3180004	10066	PEARL R	47.7	36.2	10 - 66	0.0	0.0 - 0.0	9.6	2.9 - 16.1	7.7	1.7 - 22.3	11.1	3.2 - 31.4	0.0	0.0 - 0.0	7.8	2.5 - 17.2	0.94
3180004	10067	TENMILE CR	102.7	60.5	22 - 105	0.0	0.0 - 0.0	8.9	2.9 - 17.6	16.2	6.1 - 47.3	22.6	6.9 - 55.3	0.0	0.0 - 0.0	12.8	5.0 - 24.1	0.95
3180004	10069	BOGUE LUSA CR	202.3	32.3	10 - 78	0.0	0.0 - 0.0	14.4	4.5 - 36.2	3.9	1.1 - 13.1	7.9	2.4 - 20.9	0.0	0.0 - 0.0	6.0	2.0 - 17.0	0.97
3180004	10070	WEST PEARL R	234.9	6.6	2 - 18	0.0	0.0 - 0.0	1.9	0.5 - 5.1	0.3	0.1 - 1.3	0.9	0.2 - 2.5	0.0	0.0 - 0.0	3.5	0.9 - 10.8	1.00
3180004	10071	WEST PEARL R	30.7	19.4	7 - 43	0.0	0.0 - 0.0	0.7	0.3 - 1.5	0.0	0.0 - 0.0	0.0	0.0 - 0.0	0.0	0.0 - 0.0	18.7	6.3 - 40.8	0.97
3180004	10072	BOGUE CHITTO R	52.1	36.1	16 - 69	0.0	0.0 - 0.0	7.6	3.4 - 16.3	3.9	1.5 - 13.4	14.8	6.4 - 41.9	0.0	0.0 - 0.0	9.7	3.7 - 25.0	0.97
3180004	10073	BOGUE CHITTO R	68.5	41.7	14 - 79	0.0	0.0 - 0.0	4.3	1.4 - 8.5	5.3	1.7 - 11.6	22.7	6.9 - 52.2	0.0	0.0 - 0.0	9.5	2.8 - 19.5	0.96
3180004	10074	TALISHEEK CR	62.1	23.3	9 - 44	0.0	0.0 - 0.0	5.3	2.0 - 12.1	2.7	0.9 - 7.3	8.4	2.8 - 19.3	0.0	0.0 - 0.0	6.9	2.7 - 17.4	0.96
3180005	10075	BOGUE CHITO R	22.4	25.8	9 - 50	0.0	0.0 - 0.0	8.2	2.8 - 17.7	2.3	0.6 - 6.2	10.8	3.7 - 27.6	0.0	0.0 - 0.0	4.6	1.5 - 12.1	0.95
3180005	10076	LAWRENCE CR	161.2	72.1	25 - 188	0.0	0.0 - 0.0	13.2	3.9 - 36.9	10.4	3.4 - 33.8	27.0	8.7 - 72.3	0.0	0.0 - 0.0	21.4	7.8 - 56.3	0.93
3180005	10077	BOGUE CHITO R	69.4	56.6	20 - 120	0.0	0.0 - 0.0	22.8	7.7 - 48.7	9.6	3.0 - 30.3	24.2	8.1 - 61.0	0.0	0.0 - 0.0	0.0	0.0 - 0.0	0.93
3180005	10078	HAYS CR	110.5	81.5	23 - 225	0.0	0.0 - 0.0	9.5	2.4 - 30.8	16.9	4.8 - 50.8	35.9	10.2 - 122.7	0.0	0.0 - 0.0	19.2	6.8 - 52.3	0.92
3180005	10079	BOGUE CHITO R	6.1	43.2	14 - 92	0.0	0.0 - 0.0	9.9	2.9 - 23.7	12.1	3.5 - 35.0	21.3	7.1 - 49.7	0.0	0.0 - 0.0	0.0	0.0 - 0.0	0.92
3180005	10080	BOGUE CHITO R	123.4	87.5	31 - 213	0.0	0.0 - 0.0	10.7	3.8 - 27.8	20.7	6.8 - 62.4	39.4	14.2 - 109.7	0.0	0.0 - 0.0	16.7	5.7 - 45.1	0.91
3180005	10081	MAGEES CR	559.1	113.4	36 - 224	0.6	0.2 - 1.2	10.5	3.1 - 20.5	33.1	10.4 - 90.3	46.3	14.3 - 110.1	0.0	0.0 - 0.0	22.8	7.5 - 56.4	0.90
3180005	10082	BOGUE CHITO R	293.6	84.2	30 - 142	0.0	0.0 - 0.0	9.7	3.4 - 18.3	21.9	6.6 - 46.4	31.3	10.1 - 64.3	0.0	0.0 - 0.0	21.2	7.5 - 52.5	0.90
3180005	10083	TOPISAW CR	113.3	102.8	34 - 244	0.0	0.0 - 0.0	10.0	3.5 - 22.5	26.5	8.5 - 77.9	38.3	12.1 - 109.8	0.0	0.0 - 0.0	28.0	9.7 - 71.6	0.87
3180005	10084	E TOPISAW CR	151.0	143.3	44 - 327	0.0	0.0 - 0.0	8.9	3.2 - 20.2	39.6	12.4 - 106.3	60.1	18.9 - 150.8	0.0	0.0 - 0.0	34.7	10.6 - 75.6	0.85
3180005	10085	W TOPISAW CR	117.8	121.8	44 - 226	0.0	0.0 - 0.0	8.0	2.6 - 16.2	26.0	7.8 - 79.3	50.5	16.9 - 116.2	0.0	0.0 - 0.0	37.4	13.2 - 100.1	0.85
3180005	10086	BOGUE CHITO R	794.5	184.8	50 - 388	28.0	8.5 - 61.0	17.3	4.8 - 33.5	34.0	8.1 - 127.7	63.2	17.2 - 169.8	0.0	0.0 - 0.0	42.2	11.8 - 108.5	0.87
3180005	10087	SILVER CR	14.1	84.7	32 - 185	0.0	0.0 - 0.0	11.1	3.7 - 24.4	16.7	5.3 - 49.0	43.8	15.6 - 107.8	0.0	0.0 - 0.0	13.1	5.1 - 32.8	0.91
3180005	10088	SILVER CR	132.4	95.1	29 - 172	0.0	0.0 - 0.0	8.9	2.3 - 16.8	21.9	5.6 - 49.2	42.6	12.1 - 98.2	0.0	0.0 - 0.0	21.7	7.4 - 47.7	0.90
3180005	10089	LITTLE SILVER CR	105.2	88.7	27 - 213	0.0	0.0 - 0.0	8.3	2.6 - 19.3	16.6	4.7 - 59.4	42.9	13.2 - 135.2	0.0	0.0 - 0.0	20.8	6.8 - 49.6	0.90
3080102	10090	OKLAWAHA R	136.5	0.2	0 - 0	0.0	0.0 - 0.0	0.1	0.0 - 0.2	0.0	0.0 - 0.0	0.0	0.0 - 0.1	0.0	0.0 - 0.0	0.1	0.0 - 0.2	0.21
3080102	10091	OKLAWAHA R	112.3	27.2	9 - 55	2.2	0.7 - 4.1	10.8	3.5 - 23.3	0.4	0.1 - 1.0	2.2	0.7 - 5.2	0.0	0.0 - 0.0	11.7	4.1 - 27.6	0.21

8-digit HUC	ID	Name	Area	Catchment Yield		Point sources		Developed Land		Manure		Agricultural Land		Phosphate Mines		Soil parent rock		Frac
				kg/km^2	90% CI	kg/km^2	90% CI	kg/km^2	90% CI	kg/km^2	90% CI	kg/km^2	90% CI	kg/km^2	90% CI	kg/km^2	90% CI	
3080102	10092	*B	201.8	10.1	3 - 23	0.0	0.0 - 0.0	1.7	0.5 - 4.5	0.5	0.1 - 1.6	2.3	0.7 - 7.5	0.0	0.0 - 0.0	5.5	1.8 - 13.8	0.21
3080103	10094	LITTLE HAW CR	171.7	4.7	2 - 13	0.0	0.0 - 0.0	0.6	0.2 - 1.7	0.2	0.1 - 0.7	0.7	0.2 - 2.5	0.0	0.0 - 0.0	3.2	1.0 - 9.2	0.89
3080103	10095	HAW CR	58.0	0.6	0 - 2	0.0	0.0 - 0.0	0.0	0.0 - 0.2	0.0	0.0 - 0.1	0.1	0.0 - 0.5	0.0	0.0 - 0.0	0.4	0.1 - 1.4	0.94
3080103	10096	*C	414.9	20.4	7 - 46	0.0	0.0 - 0.0	3.6	1.1 - 8.3	0.6	0.2 - 1.9	3.3	1.1 - 8.9	0.0	0.0 - 0.0	12.8	4.2 - 28.0	0.94
3080101	10098	ST JOHNS R	179.1	1.7	0 - 5	0.0	0.0 - 0.0	0.6	0.1 - 2.3	0.0	0.0 - 0.1	0.2	0.0 - 0.5	0.0	0.0 - 0.0	0.9	0.2 - 2.6	0.95
3080101	10099	ST JOHNS R	361.5	10.9	4 - 22	0.0	0.0 - 0.0	2.3	0.8 - 5.3	0.2	0.1 - 0.6	0.6	0.2 - 1.7	0.0	0.0 - 0.0	7.8	2.8 - 17.9	0.99
3080103	10100	DUNNS CR	115.8	1.0	0 - 4	0.0	0.0 - 0.0	0.2	0.0 - 1.1	0.0	0.0 - 0.1	0.1	0.0 - 0.5	0.0	0.0 - 0.0	0.6	0.1 - 2.4	0.99
3080103	10101	ETONIA CR	20.5	592.5	195 - 1625	590.7	194.6 - 1624.6	0.4	0.1 - 1.6	0.0	0.0 - 0.2	0.1	0.0 - 0.5	0.0	0.0 - 0.0	1.2	0.2 - 4.5	1.00
3080103	10102	SIXMILE CR	284.1	17.5	6 - 34	0.0	0.0 - 0.0	1.8	0.5 - 4.4	0.4	0.1 - 1.1	4.7	1.5 - 11.7	0.0	0.0 - 0.0	10.7	3.7 - 21.7	1.00
3080103	10103	JULINGTON CR	65.3	55.7	21 - 137	8.9	3.1 - 22.6	34.3	11.7 - 78.0	0.3	0.1 - 0.7	0.1	0.1 - 0.3	0.0	0.0 - 0.0	12.2	4.2 - 33.2	1.00
3080103	10104	DURBIN CR	212.7	19.9	8 - 39	0.0	0.0 - 0.0	5.0	1.8 - 10.1	0.4	0.1 - 1.1	0.7	0.3 - 1.5	0.1	0.0 - 0.1	13.8	5.6 - 29.7	1.00
3080103	10105	ORTEGA R	180.4	55.3	18 - 125	2.5	0.9 - 5.8	39.9	12.7 - 90.5	0.9	0.3 - 2.8	1.0	0.4 - 2.8	0.0	0.0 - 0.0	10.9	3.3 - 27.1	1.00
3080103	10106	CEDAR CR	116.9	45.3	15 - 99	0.0	0.0 - 0.0	30.5	9.8 - 66.6	1.3	0.3 - 3.8	1.7	0.5 - 4.6	0.0	0.0 - 0.0	11.9	4.1 - 34.9	1.00
3080103	10107	TROUT R	201.5	32.1	10 - 61	0.0	0.0 - 0.0	14.4	4.1 - 27.8	2.6	0.8 - 6.6	2.9	0.9 - 6.8	0.0	0.0 - 0.0	12.1	3.4 - 29.7	1.00
3080103	10108	*A	115.3	81.6	26 - 172	10.4	3.4 - 23.2	57.2	17.9 - 122.1	0.7	0.2 - 1.9	0.7	0.2 - 1.9	0.0	0.0 - 0.0	12.5	3.8 - 31.3	1.00
3080103	10109	INTRACOASTAL W	14.3	0.1	0 - 1	0.0	0.0 - 0.0	0.0	0.0 - 0.1	0.0	0.0 - 0.0	0.0	0.0 - 0.0	0.0	0.0 - 0.0	0.1	0.0 - 0.5	1.00
3080103	10110	INTRACOASTAL W	221.0	21.5	7 - 61	5.5	1.7 - 14.6	11.5	3.8 - 31.5	0.1	0.0 - 0.4	0.1	0.0 - 0.3	0.0	0.0 - 0.0	4.2	1.2 - 12.3	1.00
3010103	10112	SMITH R	10.9	26.2	7 - 54	0.0	0.0 - 0.0	11.9	3.4 - 26.7	0.8	0.2 - 2.3	3.7	1.0 - 8.9	0.0	0.0 - 0.0	9.8	3.1 - 23.1	0.29
3010102	10113	DIFFICULT CR	185.7	30.6	10 - 53	0.0	0.0 - 0.0	3.3	1.0 - 6.0	2.6	0.7 - 6.7	11.9	3.7 - 27.7	0.0	0.0 - 0.0	12.9	4.2 - 29.5	0.37
3010102	10114	ROANOKE R	124.9	31.1	10 - 70	0.0	0.0 - 0.0	3.4	1.0 - 6.9	3.4	1.0 - 10.9	13.4	5.0 - 36.0	0.0	0.0 - 0.0	10.9	4.1 - 26.8	0.37
3050104	10116	SANDERS CR	108.6	11.6	4 - 22	0.0	0.0 - 0.0	3.4	1.0 - 6.2	1.1	0.3 - 2.6	2.0	0.6 - 4.7	0.0	0.0 - 0.0	5.1	1.7 - 10.7	0.42
3040201	10117	BLACK CR	320.5	7.7	2 - 22	0.0	0.0 - 0.0	2.0	0.6 - 5.4	0.5	0.1 - 1.6	1.8	0.6 - 5.6	0.0	0.0 - 0.0	3.5	1.0 - 10.0	0.74
3130001	10118	LITTLE R, E FK	49.6	107.5	36 - 265	0.0	0.0 - 0.0	14.1	4.6 - 40.6	50.3	15.2 - 156.1	21.9	6.9 - 58.9	0.0	0.0 - 0.0	21.1	6.8 - 58.6	0.04
3150109	10119	HILLABEE CR	125.6	25.9	9 - 62	0.0	0.0 - 0.0	11.0	4.4 - 26.9	0.7	0.2 - 1.8	2.6	1.0 - 6.8	0.0	0.0 - 0.0	11.6	4.1 - 30.2	0.34
3150109	10120	TALLAPOOSA R	3.6	13.6	6 - 30	0.0	0.0 - 0.0	1.6	0.6 - 3.4	0.1	0.0 - 0.2	0.0	0.0 - 0.0	0.0	0.0 - 0.0	12.0	4.9 - 27.9	0.34
3150107	10121	WALNUT CR	156.0	37.8	12 - 73	0.0	0.0 - 0.0	15.2	3.9 - 31.6	2.6	0.7 - 6.8	13.9	4.5 - 31.0	0.0	0.0 - 0.0	6.0	1.9 - 12.7	0.72
3070101	10122	BIG CEDAR CR	89.7	18.3	6 - 37	0.0	0.0 - 0.0	4.4	1.3 - 10.9	0.7	0.2 - 1.8	1.0	0.4 - 2.3	0.0	0.0 - 0.0	12.3	4.3 - 31.0	0.54
3180003	10123	PEARL R	464.2	112.9	34 - 216	36.8	11.8 - 79.6	6.9	2.1 - 14.6	18.8	5.7 - 47.2	28.2	7.9 - 68.5	0.0	0.0 - 0.0	22.3	7.5 - 52.2	0.91
3100205	10124	MILL CR	179.5	135.9	52 - 312	7.6	2.7 - 18.1	44.3	16.2 - 98.3	4.8	1.5 - 16.0	10.5	3.9 - 26.1	41.9	15.2 - 148.8	26.8	10.0 - 66.6	0.91
3050201	10125	COOPER R	34.7	637.8	229 - 1346	603.2	215.8 - 1299.0	22.8	6.9 - 47.8	0.1	0.0 - 0.2	0.4	0.1 - 1.0	0.0	0.0 - 0.0	11.3	3.3 - 26.2	1.00
3050201	10126	WANDO R	331.7	5.0	2 - 12	0.1	0.0 - 0.2	1.8	0.5 - 4.9	0.0	0.0 - 0.1	0.6	0.2 - 1.9	0.0	0.0 - 0.0	2.4	0.8 - 6.7	1.00
3050202	10127	ASHLEY R	134.7	31.1	13 - 62	14.1	5.9 - 32.0	8.3	2.5 - 17.5	0.1	0.0 - 0.5	0.8	0.3 - 2.2	0.0	0.0 - 0.0	7.7	2.7 - 21.4	1.00
3050202	10128	*B	10.5	0.7	0 - 3	0.0	0.0 - 0.0	0.2	0.0 - 1.3	0.0	0.0 - 0.0	0.0	0.0 - 0.2	0.0	0.0 - 0.0	0.5	0.1 - 2.1	1.00
3060204	10130	JERICO R	12.0	9.9	3 - 26	0.0	0.0 - 0.0	0.4	0.1 - 1.2	0.0	0.0 - 0.1	0.1	0.0 - 0.3	0.0	0.0 - 0.0	9.4	2.7 - 23.6	1.00
3060204	10131	SAPELO R	479.6	23.4	7 - 44	0.0	0.0 - 0.0	2.5	0.7 - 4.7	0.3	0.1 - 0.6	0.9	0.2 - 2.0	0.0	0.0 - 0.0	19.7	5.5 - 37.7	1.00
3070106	10132	ALTAMAHA R	8.5	5.5	1 - 19	0.0	0.0 - 0.0	0.4	0.1 - 1.4	0.0	0.0 - 0.0	0.1	0.0 - 0.2	0.0	0.0 - 0.0	5.1	0.9 - 17.6	1.00
3070203	10133	INTRACOASTAL W	79.6	39.0	13 - 79	0.1	0.0 - 0.1	26.0	8.2 - 55.3	0.1	0.0 - 0.2	0.6	0.2 - 1.4	0.0	0.0 - 0.0	12.3	3.8 - 27.6	1.00
3070203	10134	TURTLE R	75.5	22.9	8 - 59	0.0	0.0 - 0.0	2.0	0.7 - 4.3	0.3	0.1 - 0.9	0.9	0.3 - 2.1	0.0	0.0 - 0.0	19.7	6.5 - 52.7	1.00
3070203	10135	LITTLE SATILLA R	639.2	14.6	5 - 36	0.0	0.0 - 0.0	1.7	0.5 - 3.9	0.3	0.1 - 1.0	0.8	0.2 - 2.3	0.0	0.0 - 0.0	11.8	3.9 - 29.2	1.00

8-digit HUC	ID	Name	Area	Catchment Yield		Point sources		Developed Land		Manure		Agricultural Land		Phosphate Mines		Soil parent rock		Frac
				kg/km^2	90% CI	kg/km^2	90% CI	kg/km^2	90% CI	kg/km^2	90% CI	kg/km^2	90% CI	kg/km^2	90% CI	kg/km^2	90% CI	
3070201	10136	SATILLA R	762.6	13.0	4 - 29	0.4	0.1 - 0.8	2.1	0.6 - 4.7	0.1	0.0 - 0.4	0.5	0.2 - 1.3	0.0	0.0 - 0.0	9.8	3.2 - 23.9	1.00
3070203	10137	CROOKED R	118.3	11.1	3 - 24	0.0	0.0 - 0.0	1.2	0.3 - 2.9	0.0	0.0 - 0.1	0.1	0.0 - 0.3	0.0	0.0 - 0.0	9.8	2.6 - 22.5	1.00
3010205	10138	NORTH LANDING [illegible]	73.5	23.1	7 - 56	0.0	0.0 - 0.0	0.5	0.1 - 1.3	0.2	0.0 - 0.4	12.7	3.5 - 37.9	0.0	0.0 - 0.0	9.7	2.8 - 24.8	1.00
3040204	10139	LITTLE PEE DEE R	17.9	24.7	7 - 65	0.0	0.0 - 0.0	0.2	0.1 - 0.4	0.8	0.2 - 2.6	3.2	1.1 - 8.8	0.0	0.0 - 0.0	20.5	6.1 - 58.2	0.99
3010107	10140	CONOCONNARA S	836.5	38.5	12 - 68	1.6	0.5 - 3.1	3.0	0.9 - 5.4	3.8	1.1 - 8.3	20.1	5.8 - 42.0	0.0	0.0 - 0.0	10.0	2.9 - 21.6	0.98
3080101	10141	ST JOHNS R	367.5	7.2	2 - 15	0.0	0.0 - 0.0	1.0	0.3 - 2.3	0.3	0.1 - 0.8	0.4	0.1 - 1.2	0.0	0.0 - 0.0	5.5	1.6 - 12.9	0.98
3080101	10142	SALT SPRING RUN	232.4	10.8	3 - 30	0.0	0.0 - 0.0	2.6	0.8 - 7.0	0.3	0.1 - 1.2	0.2	0.1 - 0.7	0.0	0.0 - 0.0	7.6	2.2 - 21.2	0.98
3080103	10143	BLACK CR	66.8	63.2	21 - 161	31.3	10.8 - 86.0	14.4	4.5 - 33.0	1.4	0.4 - 4.1	4.4	1.5 - 12.0	0.0	0.0 - 0.0	11.6	3.6 - 35.1	1.00
3080103	10144	CLARKES CR	53.3	17.2	6 - 32	0.0	0.0 - 0.0	5.9	2.1 - 13.0	0.8	0.3 - 2.3	1.4	0.4 - 3.1	0.0	0.0 - 0.0	8.9	3.6 - 21.3	1.00
3080103	10145	DEEP CR	211.3	11.2	4 - 24	0.5	0.1 - 1.1	1.3	0.4 - 3.4	0.2	0.1 - 0.8	3.4	0.9 - 9.2	0.0	0.0 - 0.0	5.8	1.9 - 14.0	1.00
3130003	10146	MARINGO CR	103.2	16.9	6 - 31	0.0	0.0 - 0.0	6.6	2.0 - 12.8	0.7	0.2 - 1.8	6.9	2.5 - 15.0	0.0	0.0 - 0.0	2.6	0.8 - 5.1	0.35
3130003	10147	UCHEE CR	85.6	21.2	6 - 55	0.0	0.0 - 0.0	6.8	2.1 - 14.7	0.8	0.2 - 1.9	6.7	2.2 - 18.5	0.0	0.0 - 0.0	6.9	2.3 - 19.2	0.35
3080102	10148	OKLAWAHA R	336.2	11.5	4 - 25	1.8	0.7 - 3.8	2.3	0.7 - 4.7	0.2	0.1 - 0.7	0.7	0.3 - 1.8	0.0	0.0 - 0.0	6.5	2.4 - 16.8	0.99
3020201	10149	FLAT R	20.7	35.8	11 - 76	0.0	0.0 - 0.0	5.1	1.5 - 12.1	2.3	0.6 - 7.2	15.9	5.1 - 44.1	0.0	0.0 - 0.0	12.6	4.3 - 30.2	0.20
3070103	10150	BIG FLAT CR	98.8	45.4	14 - 86	0.8	0.2 - 1.8	13.7	4.0 - 29.4	5.6	1.5 - 15.0	13.0	3.9 - 37.4	0.0	0.0 - 0.0	12.5	3.9 - 27.1	0.54
3070106	10151	*B	46.6	21.0	8 - 43	0.0	0.0 - 0.0	4.4	1.5 - 10.0	1.3	0.4 - 3.7	7.8	2.8 - 20.1	0.0	0.0 - 0.0	7.5	2.8 - 17.8	0.83
3070106	10152	*D	77.6	19.5	7 - 47	0.0	0.0 - 0.0	4.1	1.4 - 10.9	1.1	0.4 - 3.1	6.6	2.1 - 18.5	0.0	0.0 - 0.0	7.8	2.9 - 19.7	0.85
3040203	10153	BIG SWAMP	87.5	44.8	15 - 84	0.0	0.0 - 0.0	4.9	1.5 - 10.5	12.8	3.9 - 38.2	18.2	5.6 - 47.0	0.0	0.0 - 0.0	8.9	2.8 - 20.1	0.80
3040203	10154	LITTLE MARSH SW	137.0	41.4	18 - 77	0.8	0.3 - 2.0	5.0	2.0 - 10.5	10.8	3.6 - 24.7	16.2	6.5 - 41.2	0.0	0.0 - 0.0	8.5	3.0 - 21.2	0.80
3170004	10155	OTAKOOCHA CR	43.9	59.1	18 - 156	0.0	0.0 - 0.0	1.3	0.4 - 3.1	4.0	1.0 - 10.4	4.6	1.4 - 13.0	0.0	0.0 - 0.0	49.2	14.5 - 135.9	0.77
3180002	10156	PEARL R	84.4	114.7	34 - 267	0.0	0.0 - 0.0	6.9	2.2 - 16.2	15.9	4.1 - 50.7	30.5	9.1 - 92.4	0.0	0.0 - 0.0	61.4	17.4 - 153.6	0.41
3180002	10157	FANNEGUSHA CR	312.4	234.0	78 - 550	0.1	0.0 - 0.2	4.4	1.6 - 9.7	83.2	24.6 - 221.3	75.8	28.9 - 245.2	0.0	0.0 - 0.0	70.6	26.2 - 194.8	0.41
3180002	10158	PELAHATCHIE CR	127.5	135.5	46 - 270	0.1	0.0 - 0.2	6.8	2.3 - 14.6	24.9	6.9 - 61.0	41.0	14.4 - 99.5	0.0	0.0 - 0.0	62.7	19.0 - 152.0	0.41
3180002	10159	PEARL R	38.2	148.2	50 - 368	0.0	0.0 - 0.0	35.1	11.7 - 83.6	3.1	0.9 - 11.0	28.2	8.5 - 73.9	0.0	0.0 - 0.0	81.9	27.5 - 233.2	0.86
3180002	10160	PEARL R	135.9	168.1	47 - 426	0.0	0.0 - 0.0	9.7	3.0 - 23.9	13.1	3.6 - 41.8	28.5	7.7 - 85.7	0.0	0.0 - 0.0	116.8	33.6 - 317.2	0.41
3180002	10161	PEARL R	3.2	77.8	26 - 228	0.0	0.0 - 0.0	7.5	2.3 - 16.7	0.0	0.0 - 0.1	0.0	0.0 - 0.0	0.0	0.0 - 0.0	70.3	21.9 - 226.6	0.41
3180002	10163	PELAHATCHIE CR	87.4	145.4	41 - 288	0.0	0.0 - 0.0	32.6	9.2 - 66.7	13.7	3.4 - 34.8	23.1	6.2 - 66.3	0.0	0.0 - 0.0	76.1	20.5 - 158.6	0.41
3170001	10164	OKATIBBEE CR	14.7	39.9	12 - 94	0.0	0.0 - 0.0	3.0	0.9 - 6.5	2.9	0.8 - 8.2	11.7	3.1 - 31.2	0.0	0.0 - 0.0	22.4	6.8 - 61.4	0.31
3170001	10165	OKATIBBEE CR	116.4	14.5	5 - 27	0.0	0.0 - 0.0	3.0	0.9 - 5.7	1.3	0.3 - 3.6	5.2	1.4 - 12.0	0.0	0.0 - 0.0	5.0	1.4 - 12.5	0.80
3160108	10166	NOXUBEE R	110.8	108.5	38 - 244	0.0	0.0 - 0.0	3.8	1.3 - 9.3	4.4	1.3 - 11.7	18.4	6.8 - 50.0	0.0	0.0 - 0.0	81.8	27.6 - 202.3	0.50
3160108	10167	NOXUBEE R	34.7	66.1	25 - 151	0.0	0.0 - 0.0	1.8	0.7 - 3.7	1.4	0.5 - 4.1	6.3	2.1 - 16.0	0.0	0.0 - 0.0	56.7	19.8 - 145.8	0.77
3160110	10168	CLEAR CR	168.2	20.6	6 - 48	0.0	0.0 - 0.0	6.2	1.8 - 14.9	5.2	1.4 - 14.6	4.7	1.5 - 12.9	0.0	0.0 - 0.0	4.5	1.3 - 11.8	0.18
3160110	10169	BLACK WARRIOR	13.1	9.9	3 - 17	0.0	0.0 - 0.0	4.8	1.5 - 9.6	0.2	0.1 - 0.7	0.3	0.1 - 0.6	0.0	0.0 - 0.0	4.6	1.7 - 7.8	0.18
3160110	10170	BLACK WARRIOR	81.5	15.6	6 - 40	0.0	0.0 - 0.0	4.3	1.5 - 11.9	3.3	1.0 - 9.3	3.6	1.1 - 9.5	0.0	0.0 - 0.0	4.4	1.4 - 12.7	0.18
3160110	10171	BRUSHY FK	37.1	36.6	13 - 74	0.0	0.0 - 0.0	6.4	2.2 - 12.8	12.2	4.0 - 34.4	13.4	4.6 - 33.4	0.0	0.0 - 0.0	4.4	1.6 - 10.5	0.18
3160110	10172	CROOKED CR	179.7	49.8	16 - 116	0.0	0.0 - 0.0	6.7	2.1 - 15.4	22.0	6.4 - 65.2	16.1	5.0 - 42.1	0.0	0.0 - 0.0	4.9	1.8 - 11.7	0.18
3160110	10173	RYAN CR	174.2	65.4	20 - 130	0.0	0.0 - 0.0	21.4	6.5 - 45.5	22.4	6.3 - 64.4	16.3	4.8 - 36.5	0.0	0.0 - 0.0	5.2	1.8 - 12.4	0.18
3160110	10174	BLACK WARRIOR	4.5	5.2	2 - 13	0.0	0.0 - 0.0	1.8	0.6 - 5.1	0.5	0.2 - 1.8	0.8	0.3 - 2.6	0.0	0.0 - 0.0	2.0	0.8 - 5.7	0.56
3160110	10175	BLACK WARRIOR	16.2	32.2	9 - 76	0.0	0.0 - 0.0	8.4	2.5 - 21.1	8.6	2.3 - 23.9	9.5	2.9 - 24.8	0.0	0.0 - 0.0	5.8	2.2 - 14.0	0.18

8-digit HUC	ID	Name	Area	Catchment Yield		Point sources		Developed Land		Manure		Agricultural Land		Phosphate Mines		Soil parent rock		Frac
				kg/km^2	90% CI	kg/km^2	90% CI	kg/km^2	90% CI	kg/km^2	90% CI	kg/km^2	90% CI	kg/km^2	90% CI	kg/km^2	90% CI	
3160110	10176	BRUSHY FK	41.2	43.8	14 - 92	0.0	0.0 - 0.0	7.0	2.4 - 14.2	17.0	5.1 - 48.1	14.0	4.7 - 32.6	0.0	0.0 - 0.0	5.8	2.0 - 14.5	0.18
3160110	10177	BLACK WARRIOR	98.6	29.3	9 - 56	0.1	0.0 - 0.1	7.5	2.7 - 15.5	7.7	2.2 - 20.4	9.2	2.9 - 21.3	0.0	0.0 - 0.0	4.8	1.3 - 10.8	0.18
3160110	10178	BLACK WARRIOR	22.8	20.3	7 - 38	0.0	0.0 - 0.0	5.2	1.6 - 11.7	4.9	1.3 - 14.3	4.9	1.6 - 11.6	0.0	0.0 - 0.0	5.3	1.7 - 12.5	0.18
3160110	10179	CLEAR CR	37.5	13.0	5 - 32	0.0	0.0 - 0.0	4.5	1.6 - 12.5	2.2	0.7 - 7.3	1.2	0.4 - 4.1	0.0	0.0 - 0.0	5.1	1.9 - 13.7	0.18
3160110	10180	BLACK WARRIOR	165.0	22.1	7 - 43	0.0	0.0 - 0.0	7.6	2.4 - 16.7	4.7	1.3 - 10.7	4.9	1.7 - 12.6	0.0	0.0 - 0.0	5.0	1.6 - 10.9	0.18
3160110	10181	BLACK WARRIOR	57.8	19.1	6 - 53	0.0	0.0 - 0.0	6.1	2.1 - 17.1	4.0	1.2 - 11.7	4.2	1.4 - 12.0	0.0	0.0 - 0.0	4.8	1.5 - 12.3	0.18
3160110	10182	BRUSHY FK	42.9	35.4	11 - 80	0.0	0.0 - 0.0	5.7	1.7 - 12.3	11.8	3.2 - 30.1	13.1	3.3 - 33.3	0.0	0.0 - 0.0	4.8	1.6 - 12.5	0.18
3160110	10183	CROOKED CR	53.1	38.1	13 - 98	0.0	0.0 - 0.0	5.8	2.1 - 15.4	14.7	4.8 - 44.8	12.9	4.2 - 37.5	0.0	0.0 - 0.0	4.7	1.8 - 13.1	0.18
3160110	10184	RYAN CR	280.6	49.9	18 - 99	0.0	0.0 - 0.0	7.7	2.9 - 15.5	21.4	7.1 - 56.8	15.5	6.1 - 32.7	0.0	0.0 - 0.0	5.2	2.0 - 12.6	0.18
3150107	10185	COOSA R	2.7	24.4	8 - 50	0.0	0.0 - 0.0	7.7	2.4 - 17.3	0.4	0.1 - 1.3	2.5	0.8 - 6.7	0.0	0.0 - 0.0	13.7	4.8 - 29.9	0.68
3150107	10186	WAXAHATCHEE C	49.6	31.9	10 - 64	0.0	0.0 - 0.0	6.3	1.8 - 14.3	1.3	0.4 - 3.7	5.7	1.8 - 14.3	0.0	0.0 - 0.0	18.7	6.0 - 44.3	0.68
3150107	10187	PAINT CR	74.8	13.6	4 - 30	0.0	0.0 - 0.0	5.0	1.6 - 10.7	0.6	0.2 - 1.8	0.1	0.0 - 0.3	0.0	0.0 - 0.0	7.9	2.7 - 20.7	0.68
3150107	10188	COOSA R	3.7	20.2	6 - 40	0.0	0.0 - 0.0	5.8	1.6 - 11.8	0.4	0.1 - 1.0	1.0	0.3 - 2.6	0.0	0.0 - 0.0	12.9	4.1 - 29.4	0.72
3150107	10189	COOSA R	17.1	19.7	7 - 54	0.0	0.0 - 0.0	4.1	1.3 - 9.5	0.6	0.2 - 1.9	0.1	0.0 - 0.2	0.0	0.0 - 0.0	14.9	5.8 - 39.6	0.68
3150107	10190	COOSA R	44.2	22.1	8 - 44	0.0	0.0 - 0.0	4.8	1.7 - 9.4	0.4	0.1 - 0.9	1.5	0.5 - 3.8	0.0	0.0 - 0.0	15.4	5.5 - 32.5	0.68
3150107	10191	WAXAHATCHEE C	8.9	26.3	10 - 54	0.0	0.0 - 0.0	6.4	2.3 - 13.5	0.8	0.3 - 2.1	0.5	0.2 - 1.2	0.0	0.0 - 0.0	18.6	6.4 - 40.0	0.68
3150107	10192	PAINT CR	18.6	16.0	6 - 32	0.0	0.0 - 0.0	6.6	2.2 - 17.3	0.1	0.0 - 0.4	0.3	0.1 - 0.8	0.0	0.0 - 0.0	8.9	3.0 - 20.9	0.68
3150105	10193	YELLOW CR	82.3	33.9	11 - 71	0.0	0.0 - 0.0	7.0	2.4 - 16.3	8.7	2.7 - 25.5	13.3	4.1 - 39.5	0.0	0.0 - 0.0	4.9	1.6 - 12.8	0.33
3150105	10194	LITTLE R	47.3	34.4	10 - 68	0.0	0.0 - 0.0	6.9	2.2 - 12.8	6.7	2.0 - 19.0	14.2	4.0 - 33.9	0.0	0.0 - 0.0	6.6	2.0 - 13.6	0.33
3150105	10195	CHATOOGA R	51.6	46.9	19 - 85	0.0	0.0 - 0.0	8.0	3.2 - 15.9	4.8	1.6 - 13.9	18.2	6.9 - 40.2	0.0	0.0 - 0.0	15.9	6.5 - 33.1	0.33
3150105	10196	COOSA R	32.4	37.9	14 - 79	0.0	0.0 - 0.0	5.1	2.1 - 10.2	3.5	1.1 - 7.9	11.8	4.4 - 28.6	0.0	0.0 - 0.0	17.5	6.3 - 44.0	0.33
3150105	10197	SPRING CR	136.0	45.1	18 - 105	0.0	0.0 - 0.0	5.5	2.1 - 13.1	5.5	1.9 - 13.7	18.6	7.3 - 53.5	0.0	0.0 - 0.0	15.5	6.3 - 48.0	0.33
3150105	10199	COOSA R	21.2	75.1	25 - 224	0.0	0.0 - 0.0	14.1	4.8 - 36.9	6.7	1.8 - 27.2	27.3	8.4 - 96.3	0.0	0.0 - 0.0	26.9	10.2 - 91.8	0.33
3150105	10200	COOSA R	12.3	64.0	18 - 146	0.0	0.0 - 0.0	10.4	2.9 - 26.7	4.4	1.1 - 13.2	17.6	4.7 - 43.9	0.0	0.0 - 0.0	31.5	9.5 - 80.1	0.33
3150105	10201	COOSA R	20.0	84.2	28 - 183	0.0	0.0 - 0.0	28.2	9.2 - 58.4	6.4	2.0 - 19.0	26.5	7.9 - 62.3	0.0	0.0 - 0.0	23.1	7.8 - 63.3	0.33
3150105	10202	YELLOW CR	13.3	35.4	12 - 98	0.0	0.0 - 0.0	9.0	3.1 - 26.6	2.3	0.6 - 6.8	8.8	2.9 - 27.7	0.0	0.0 - 0.0	15.3	5.4 - 45.2	0.33
3150105	10203	LITTLE R	124.5	36.3	12 - 81	0.0	0.0 - 0.0	6.3	2.1 - 13.7	3.3	1.0 - 9.4	12.3	3.6 - 31.1	0.0	0.0 - 0.0	14.4	4.8 - 39.8	0.33
3150105	10204	CHATOOGA R	33.9	51.4	16 - 119	0.0	0.0 - 0.0	12.8	3.7 - 27.6	3.7	1.0 - 10.0	15.0	4.9 - 38.7	0.0	0.0 - 0.0	19.9	6.1 - 48.2	0.33
3150105	10205	COOSA R	134.9	57.1	24 - 152	0.0	0.0 - 0.0	6.1	2.1 - 15.9	6.2	1.9 - 19.4	20.4	8.3 - 58.1	0.0	0.0 - 0.0	24.4	10.0 - 66.2	0.33
3150105	10206	SPRING CR	117.9	60.5	19 - 105	0.0	0.0 - 0.0	7.4	2.4 - 14.1	6.4	1.7 - 16.8	25.6	7.5 - 55.2	0.0	0.0 - 0.0	21.0	5.8 - 47.4	0.33
3170008	10207	BIG CR	68.5	36.7	13 - 69	0.0	0.0 - 0.0	15.8	5.1 - 30.6	1.8	0.5 - 5.1	8.6	2.5 - 20.8	0.0	0.0 - 0.0	10.5	3.1 - 23.4	0.22
3170008	10208	BIG CR	129.8	11.0	4 - 21	0.0	0.0 - 0.0	5.0	1.7 - 10.5	0.5	0.2 - 1.4	2.7	0.9 - 7.2	0.0	0.0 - 0.0	2.8	0.9 - 6.5	0.92
3170006	10210	ESCATAWPA R	30.9	184.7	61 - 449	0.0	0.0 - 0.0	57.6	18.4 - 131.2	0.2	0.1 - 0.8	0.3	0.1 - 0.9	0.0	0.0 - 0.0	126.6	39.2 - 376.0	1.00
3130003	10214	CHATTAHOOCHEE	341.1	19.9	7 - 33	0.0	0.0 - 0.0	3.5	1.2 - 6.4	0.9	0.3 - 2.2	6.6	2.0 - 16.0	0.0	0.0 - 0.0	8.9	2.9 - 18.7	0.36
3130003	10215	BARBOUR CR	245.9	11.2	4 - 22	0.0	0.0 - 0.0	3.4	1.1 - 6.8	0.5	0.1 - 1.3	1.4	0.4 - 3.2	0.0	0.0 - 0.0	5.9	2.2 - 12.1	0.36
3130003	10216	PATAULA CR	221.2	13.9	5 - 25	0.0	0.0 - 0.0	2.3	0.8 - 4.5	0.3	0.1 - 0.7	2.8	1.1 - 6.0	0.0	0.0 - 0.0	8.5	3.1 - 19.9	0.36
3130003	10217	CHATTAHOOCHEE	195.3	15.0	6 - 34	0.0	0.0 - 0.0	3.1	1.0 - 7.3	0.4	0.1 - 1.2	3.9	1.3 - 11.2	0.0	0.0 - 0.0	7.6	2.7 - 18.7	0.61
3130003	10218	CHATTAHOOCHEE	216.7	24.6	9 - 64	0.0	0.0 - 0.0	6.4	2.1 - 17.0	1.5	0.5 - 4.6	7.7	2.6 - 21.1	0.0	0.0 - 0.0	9.0	2.9 - 27.9	0.36
3130003	10219	CHATTAHOOCHEE	189.1	45.0	15 - 100	20.3	6.4 - 46.1	10.5	3.4 - 24.3	0.9	0.2 - 2.4	4.2	1.5 - 12.8	0.0	0.0 - 0.0	8.9	3.4 - 23.0	0.36

8-digit HUC	ID	Name	Area	Catchment Yield		Point sources		Developed Land		Manure		Agricultural Land		Phosphate Mines		Soil parent rock		Frac
				kg/km^2	90% CI	kg/km^2	90% CI	kg/km^2	90% CI	kg/km^2	90% CI	kg/km^2	90% CI	kg/km^2	90% CI	kg/km^2	90% CI	
3130004	10220	CHATTAHOOCHEE	1.8	54.8	22 - 148	0.0	0.0 - 0.0	11.9	4.2 - 30.2	3.2	1.1 - 10.2	18.9	6.2 - 59.2	0.0	0.0 - 0.0	20.9	8.8 - 57.6	0.61
3130003	10221	COWIKEE CR, N FK	35.6	34.8	10 - 85	0.0	0.0 - 0.0	8.5	2.5 - 18.2	4.2	1.1 - 12.2	10.9	3.2 - 29.5	0.0	0.0 - 0.0	11.2	3.6 - 29.9	0.36
3130003	10222	CHATTAHOOCHEE	52.5	27.7	8 - 58	0.0	0.0 - 0.0	4.5	1.2 - 10.0	3.3	0.8 - 10.1	9.4	3.2 - 21.9	0.0	0.0 - 0.0	10.6	2.8 - 26.2	0.36
3130003	10223	BARBOUR CR	164.2	19.9	7 - 35	0.0	0.0 - 0.0	7.9	2.5 - 15.5	1.2	0.4 - 2.9	3.3	1.1 - 7.9	0.0	0.0 - 0.0	7.5	2.6 - 16.9	0.36
3130003	10224	PATAULA CR	39.5	26.3	9 - 52	0.0	0.0 - 0.0	4.4	1.5 - 9.5	0.8	0.3 - 1.9	10.7	3.4 - 23.4	0.0	0.0 - 0.0	10.4	3.5 - 23.2	0.36
3130007	10225	KINCHAFOONEE C	30.0	87.4	35 - 186	0.0	0.0 - 0.0	69.9	27.9 - 145.1	0.4	0.1 - 1.0	3.1	1.2 - 7.8	0.0	0.0 - 0.0	14.0	5.7 - 32.2	0.60
3130007	10226	MUCKALEE CR	152.2	39.5	14 - 97	0.0	0.0 - 0.0	7.6	3.0 - 17.3	1.7	0.5 - 5.9	19.1	6.5 - 53.7	0.0	0.0 - 0.0	11.1	4.0 - 29.4	0.60
3130006	10227	FLINT R	32.9	25.4	9 - 64	0.0	0.0 - 0.0	2.3	0.7 - 5.0	1.2	0.4 - 3.6	13.2	4.5 - 33.8	0.0	0.0 - 0.0	8.8	3.1 - 23.8	0.60
3130006	10228	PINEY WOODS CR	131.8	29.4	11 - 53	0.0	0.0 - 0.0	8.9	3.2 - 17.7	0.8	0.3 - 2.3	9.5	3.3 - 22.9	0.0	0.0 - 0.0	10.2	3.8 - 23.0	0.60
3130006	10229	FLINT R	38.0	50.2	15 - 101	0.0	0.0 - 0.0	33.4	10.0 - 72.0	0.6	0.2 - 1.6	6.3	1.8 - 15.6	0.0	0.0 - 0.0	9.8	2.8 - 20.1	0.60
3130007	10230	KINCHAFOONEE C	1.8	71.1	25 - 151	0.0	0.0 - 0.0	60.5	20.9 - 133.1	0.1	0.0 - 0.3	1.0	0.3 - 2.5	0.0	0.0 - 0.0	9.5	3.1 - 25.8	0.60
3130007	10231	KINCHAFOONEE C	1.0	86.0	28 - 169	0.0	0.0 - 0.0	66.8	19.2 - 142.1	0.1	0.0 - 0.3	0.8	0.2 - 2.4	0.0	0.0 - 0.0	18.3	6.4 - 45.6	0.60
3130007	10232	MUCKALEE CR	1.7	43.4	15 - 78	0.0	0.0 - 0.0	12.0	4.0 - 21.8	1.3	0.3 - 3.2	9.1	3.3 - 20.4	0.0	0.0 - 0.0	21.1	6.8 - 49.9	0.60
3130006	10233	FLINT R	32.9	35.4	13 - 90	0.0	0.0 - 0.0	4.0	1.4 - 10.2	1.4	0.4 - 4.3	19.4	6.1 - 52.7	0.0	0.0 - 0.0	10.7	4.0 - 26.4	0.60
3130006	10235	LIME CR	7.7	34.4	13 - 89	0.0	0.0 - 0.0	0.9	0.3 - 1.9	2.9	1.0 - 9.6	20.6	7.0 - 51.9	0.0	0.0 - 0.0	9.9	3.5 - 24.2	0.49
3130006	10236	FLINT R	29.2	34.1	11 - 86	0.0	0.0 - 0.0	3.0	1.0 - 7.5	2.6	0.6 - 7.1	18.7	6.4 - 51.8	0.0	0.0 - 0.0	9.8	3.2 - 25.8	0.49
3130006	10237	LIMESTONE CR	54.3	29.3	13 - 70	0.0	0.0 - 0.0	3.3	1.2 - 6.7	1.9	0.7 - 6.0	16.0	6.5 - 41.4	0.0	0.0 - 0.0	8.1	3.1 - 20.0	0.49
3130006	10238	GUM CR	220.2	42.2	14 - 79	12.8	3.9 - 26.6	9.7	2.6 - 18.6	0.8	0.2 - 2.5	10.9	3.6 - 22.4	0.0	0.0 - 0.0	8.0	2.6 - 17.8	0.49
3130006	10239	CEDAR CR	129.0	32.8	12 - 80	0.0	0.0 - 0.0	9.5	3.3 - 22.9	0.7	0.2 - 2.4	14.3	5.0 - 36.4	0.0	0.0 - 0.0	8.3	2.6 - 23.5	0.49
3130006	10240	SWIFT CR	229.6	27.6	10 - 58	0.0	0.0 - 0.0	4.6	1.6 - 11.0	1.0	0.3 - 2.7	13.1	4.3 - 35.4	0.0	0.0 - 0.0	8.9	2.9 - 19.3	0.49
3130006	10242	FLINT R	32.5	23.0	6 - 50	0.0	0.0 - 0.0	7.8	2.0 - 17.3	0.5	0.1 - 1.4	4.8	1.4 - 12.8	0.0	0.0 - 0.0	9.9	2.5 - 25.6	0.49
3130006	10243	FLINT R	65.9	32.2	10 - 71	0.0	0.0 - 0.0	5.5	1.4 - 12.2	1.6	0.4 - 3.6	15.9	5.1 - 44.5	0.0	0.0 - 0.0	9.2	2.6 - 20.2	0.49
3130006	10244	FLINT R	56.0	33.6	12 - 69	0.0	0.0 - 0.0	4.4	1.4 - 9.2	1.8	0.5 - 4.8	18.3	5.9 - 45.6	0.0	0.0 - 0.0	9.1	2.7 - 19.9	0.49
3130006	10245	FLINT R	16.9	38.6	12 - 96	0.0	0.0 - 0.0	2.0	0.7 - 4.7	2.7	0.7 - 8.9	23.8	7.0 - 62.1	0.0	0.0 - 0.0	10.1	3.2 - 25.8	0.49
3130006	10250	CEDAR CR	4.6	31.3	12 - 60	0.0	0.0 - 0.0	6.3	2.1 - 12.4	0.8	0.2 - 2.3	13.9	4.6 - 30.9	0.0	0.0 - 0.0	10.3	3.5 - 22.7	0.49
3130006	10251	SWIFT CR	6.0	30.3	11 - 69	0.0	0.0 - 0.0	14.9	5.0 - 30.8	0.6	0.2 - 1.5	5.2	1.8 - 12.2	0.0	0.0 - 0.0	9.7	3.4 - 21.7	0.49
3130002	10252	HALAWAKEE CR	212.5	36.9	11 - 75	4.7	1.4 - 10.6	9.3	3.1 - 21.4	1.1	0.4 - 3.0	8.4	2.4 - 19.8	0.0	0.0 - 0.0	13.4	4.0 - 31.9	0.30
3130002	10253	OSANIPPA CR	323.0	26.7	7 - 62	0.0	0.0 - 0.0	6.9	2.0 - 15.9	1.3	0.3 - 4.1	7.3	2.3 - 20.8	0.0	0.0 - 0.0	11.2	3.5 - 29.9	0.30
3130002	10255	CHATTAHOOCHEE	4.8	28.4	10 - 58	0.0	0.0 - 0.0	8.5	2.7 - 18.7	0.3	0.1 - 0.8	1.1	0.4 - 2.6	0.0	0.0 - 0.0	18.6	6.6 - 43.9	0.34
3130002	10256	CHATTAHOOCHEE	7.6	30.8	10 - 69	0.0	0.0 - 0.0	6.7	2.2 - 12.1	0.1	0.0 - 0.2	0.3	0.1 - 0.6	0.0	0.0 - 0.0	23.7	7.9 - 58.2	0.30
3130002	10257	HALAWAKEE CR	37.5	28.2	9 - 54	0.0	0.0 - 0.0	7.1	2.1 - 14.4	0.6	0.2 - 1.8	4.4	1.3 - 11.0	0.0	0.0 - 0.0	16.0	5.3 - 39.2	0.30
3130002	10258	OSANIPPA CR	3.4	32.9	12 - 62	0.0	0.0 - 0.0	11.2	4.2 - 23.4	0.5	0.2 - 1.4	3.4	1.1 - 7.5	0.0	0.0 - 0.0	17.8	6.6 - 38.4	0.30
3130002	10259	CHATTAHOOCHEE	2.3	32.0	10 - 65	0.0	0.0 - 0.0	7.7	2.6 - 14.8	0.2	0.1 - 0.5	2.2	0.6 - 5.6	0.0	0.0 - 0.0	21.9	6.9 - 51.9	0.30
3130002	10260	WEHADKEE CR	162.5	36.1	12 - 67	0.0	0.0 - 0.0	5.5	1.7 - 12.1	8.6	2.6 - 24.1	11.1	3.6 - 27.7	0.0	0.0 - 0.0	10.9	3.6 - 24.2	0.18
3130002	10261	NEW R	4.9	21.5	9 - 53	0.0	0.0 - 0.0	4.5	1.9 - 10.4	2.7	0.9 - 7.4	3.1	1.2 - 9.4	0.0	0.0 - 0.0	11.1	4.3 - 25.9	0.18
3130002	10262	YELLOWJACKET C	253.1	28.6	10 - 51	0.0	0.0 - 0.0	7.6	2.7 - 14.8	1.5	0.4 - 4.0	7.4	2.4 - 18.4	0.0	0.0 - 0.0	12.2	3.7 - 26.2	0.18
3130002	10263	CHATTAHOOCHEE	56.9	18.7	5 - 42	0.0	0.0 - 0.0	3.5	0.9 - 8.3	0.6	0.2 - 1.8	4.1	1.2 - 9.8	0.0	0.0 - 0.0	10.5	3.1 - 25.9	0.30
3130002	10264	CHATTAHOOCHEE	163.6	26.0	9 - 49	0.0	0.0 - 0.0	7.5	2.3 - 16.1	1.7	0.5 - 4.6	3.7	1.2 - 8.3	0.0	0.0 - 0.0	13.0	4.6 - 29.2	0.18
3130002	10265	CHATTAHOOCHEE	2.6	22.7	7 - 50	0.0	0.0 - 0.0	0.5	0.1 - 1.0	0.8	0.2 - 2.2	3.5	1.0 - 9.0	0.0	0.0 - 0.0	17.8	5.0 - 42.2	0.18

8-digit HUC	ID	Name	Area	Catchment Yield		Point sources		Developed Land		Manure		Agricultural Land		Phosphate Mines		Soil parent rock		Frac
				kg/km^2	90% CI	kg/km^2	90% CI	kg/km^2	90% CI	kg/km^2	90% CI	kg/km^2	90% CI	kg/km^2	90% CI	kg/km^2	90% CI	
3130002	10266	WEHADKEE CR	233.1	30.8	11 - 57	0.0	0.0 - 0.0	7.1	2.7 - 15.1	3.1	0.9 - 9.9	7.7	2.9 - 19.5	0.0	0.0 - 0.0	12.9	4.8 - 28.2	0.18
3130002	10267	CHATTAHOOCHEE	99.6	26.4	8 - 52	0.0	0.0 - 0.0	5.3	2.0 - 12.4	4.2	1.3 - 11.7	4.9	1.6 - 10.8	0.0	0.0 - 0.0	12.1	3.6 - 26.2	0.18
3130002	10268	CHATTAHOOCHEE	1.2	26.4	10 - 49	0.0	0.0 - 0.0	1.1	0.4 - 2.2	4.5	1.5 - 11.7	5.9	2.5 - 13.5	0.0	0.0 - 0.0	15.0	5.8 - 35.1	0.18
3130002	10269	NEW R	62.6	22.2	8 - 43	0.0	0.0 - 0.0	4.3	1.4 - 10.2	2.9	0.9 - 8.8	3.4	1.1 - 11.1	0.0	0.0 - 0.0	11.4	4.1 - 26.8	0.18
3130002	10270	YELLOWJACKET C	270.9	31.9	11 - 65	0.0	0.0 - 0.0	12.4	3.6 - 25.3	1.5	0.4 - 4.4	5.7	1.8 - 13.9	0.0	0.0 - 0.0	12.4	3.9 - 31.8	0.18
3150109	10272	TALLAPOOSA R	536.6	9.0	3 - 17	0.0	0.0 - 0.0	3.3	1.0 - 6.8	0.2	0.1 - 0.6	0.8	0.2 - 1.8	0.0	0.0 - 0.0	4.6	1.6 - 9.7	0.76
3150109	10273	TALLAPOOSA R	347.5	106.6	37 - 200	81.4	27.0 - 167.1	11.7	3.7 - 22.8	0.5	0.1 - 1.4	2.3	0.7 - 5.8	0.0	0.0 - 0.0	10.7	3.7 - 23.9	0.34
3150109	10274	SANDY CR	40.3	25.0	7 - 56	0.0	0.0 - 0.0	8.8	2.3 - 20.0	0.3	0.1 - 1.0	1.2	0.3 - 3.3	0.0	0.0 - 0.0	14.7	4.2 - 36.8	0.34
3150107	10275	WEOGUFKA CR	334.6	19.2	7 - 48	0.0	0.0 - 0.0	6.9	2.4 - 17.3	0.9	0.3 - 2.7	3.8	1.4 - 13.0	0.0	0.0 - 0.0	7.5	2.8 - 20.0	0.72
3150107	10276	HATCHET CR	17.8	13.1	5 - 25	0.0	0.0 - 0.0	3.5	1.2 - 6.5	0.6	0.2 - 1.5	0.2	0.1 - 0.7	0.0	0.0 - 0.0	8.8	3.4 - 18.2	0.72
3150107	10277	COOSA R	94.7	14.7	5 - 37	0.0	0.0 - 0.0	4.8	1.5 - 10.6	0.5	0.1 - 1.8	0.7	0.2 - 2.0	0.0	0.0 - 0.0	8.6	2.9 - 21.7	0.72
3150107	10278	COOSA R	83.6	17.0	5 - 31	0.0	0.0 - 0.0	5.1	1.4 - 9.7	1.0	0.2 - 2.9	3.8	1.2 - 8.8	0.0	0.0 - 0.0	7.1	1.9 - 14.8	0.76
3150107	10279	HATCHET CR	28.0	13.2	5 - 32	0.0	0.0 - 0.0	4.2	1.5 - 10.8	0.2	0.1 - 0.6	0.1	0.0 - 0.4	0.0	0.0 - 0.0	8.7	3.1 - 21.5	0.72
3150107	10280	COOSA R	6.9	18.4	6 - 46	0.0	0.0 - 0.0	3.8	1.1 - 8.4	0.8	0.2 - 2.3	0.3	0.1 - 1.0	0.0	0.0 - 0.0	13.5	4.4 - 38.4	0.72
3150107	10281	WEOGUFKA CR	15.0	15.8	5 - 38	0.0	0.0 - 0.0	6.0	1.8 - 15.3	0.8	0.2 - 2.8	0.4	0.1 - 1.3	0.0	0.0 - 0.0	8.6	2.8 - 21.4	0.72
3150107	10282	HATCHET CR	27.0	13.3	5 - 29	0.0	0.0 - 0.0	5.6	2.2 - 11.6	0.3	0.1 - 1.0	0.1	0.1 - 0.4	0.0	0.0 - 0.0	7.3	2.7 - 18.7	0.72
3150107	10284	COOSA R	147.6	16.3	6 - 30	0.0	0.0 - 0.0	3.8	1.4 - 7.5	0.5	0.2 - 1.5	2.2	0.7 - 5.6	0.0	0.0 - 0.0	9.7	3.6 - 23.3	0.76
3150107	10285	SHOAL CR	69.8	20.5	8 - 41	0.0	0.0 - 0.0	5.7	2.2 - 12.7	1.4	0.5 - 4.0	9.2	3.6 - 24.2	0.0	0.0 - 0.0	4.2	1.8 - 9.6	0.76
3150107	10286	WEOKA CR	104.1	17.3	5 - 48	0.0	0.0 - 0.0	4.5	1.3 - 11.3	0.6	0.2 - 1.9	1.8	0.5 - 5.0	0.0	0.0 - 0.0	10.3	3.4 - 30.5	0.76
3150107	10287	SOFKAHATCHEE C	119.1	16.5	6 - 30	0.0	0.0 - 0.0	4.5	1.4 - 9.4	1.1	0.3 - 3.0	4.7	1.6 - 10.2	0.0	0.0 - 0.0	6.1	2.2 - 13.6	0.76
3150107	10288	COOSA R	4.2	24.6	9 - 51	0.0	0.0 - 0.0	2.5	0.8 - 4.5	0.5	0.2 - 1.4	3.9	1.4 - 8.6	0.0	0.0 - 0.0	17.7	6.6 - 43.2	0.79
3150107	10289	COOSA R	13.3	40.5	12 - 64	0.1	0.0 - 0.1	12.1	3.7 - 22.0	2.2	0.6 - 5.8	14.4	3.6 - 30.3	0.0	0.0 - 0.0	11.7	3.7 - 21.8	0.76
3150107	10290	COOSA R	2.8	28.7	10 - 58	0.0	0.0 - 0.0	3.8	1.2 - 8.4	2.2	0.7 - 5.7	14.4	5.0 - 34.3	0.0	0.0 - 0.0	8.3	3.1 - 18.1	0.76
3150107	10291	COOSA R	9.6	32.2	11 - 87	0.0	0.0 - 0.0	7.1	2.4 - 18.9	1.8	0.5 - 5.7	9.4	3.2 - 28.8	0.0	0.0 - 0.0	14.0	4.4 - 39.6	0.76
3150107	10292	SHOAL CR	7.0	30.0	10 - 59	0.0	0.0 - 0.0	10.7	3.5 - 20.3	1.4	0.4 - 3.6	9.8	3.0 - 21.8	0.0	0.0 - 0.0	8.2	2.8 - 18.1	0.76
3150107	10293	WEOKA CR	20.8	30.0	9 - 67	0.0	0.0 - 0.0	8.5	2.3 - 18.0	2.2	0.6 - 6.1	10.6	3.1 - 26.1	0.0	0.0 - 0.0	8.8	2.8 - 20.6	0.76
3150107	10294	SOFKAHATCHEE C	7.6	24.9	8 - 48	0.0	0.0 - 0.0	7.9	2.4 - 17.2	1.4	0.4 - 4.1	6.6	2.2 - 15.9	0.0	0.0 - 0.0	9.0	2.6 - 21.1	0.76
3130011	10295	INTRACOASTAL W	72.2	11.7	4 - 25	0.0	0.0 - 0.0	0.2	0.1 - 0.3	0.0	0.0 - 0.0	0.0	0.0 - 0.0	0.0	0.0 - 0.0	11.5	4.2 - 24.3	0.91
3130011	10296	INTRACOASTAL W	209.8	9.3	3 - 26	0.0	0.0 - 0.0	0.3	0.1 - 0.8	0.0	0.0 - 0.0	0.0	0.0 - 0.0	0.0	0.0 - 0.0	9.0	3.2 - 26.0	0.91
3130011	10297	APALACHICOLA R	30.9	6.4	1 - 16	0.0	0.0 - 0.0	0.0	0.0 - 0.0	0.0	0.0 - 0.0	0.0	0.0 - 0.0	0.0	0.0 - 0.0	6.4	1.5 - 16.5	1.00
3130012	10298	CHIPOLA R	35.6	29.1	9 - 73	0.0	0.0 - 0.0	5.4	1.5 - 12.7	0.4	0.1 - 1.6	1.8	0.5 - 5.5	0.0	0.0 - 0.0	21.5	6.8 - 53.3	0.71
3130012	10299	CYPRESS CR	225.0	28.1	10 - 73	0.0	0.0 - 0.0	6.3	2.3 - 14.2	0.4	0.1 - 1.1	1.9	0.6 - 5.7	0.0	0.0 - 0.0	19.6	6.3 - 51.8	0.71
3130012	10300	CHIPOLA R	141.4	19.9	7 - 42	0.0	0.0 - 0.0	3.7	1.1 - 8.7	0.1	0.0 - 0.3	1.9	0.6 - 5.3	0.0	0.0 - 0.0	14.2	4.8 - 30.2	0.98
3130012	10301	CHIPOLA R	23.9	29.6	10 - 56	0.0	0.0 - 0.0	3.9	1.3 - 8.0	0.2	0.1 - 0.5	0.0	0.0 - 0.1	0.0	0.0 - 0.0	25.5	8.3 - 53.7	0.71
3120003	10303	OCHOCKONEE R	213.6	63.2	20 - 106	0.0	0.0 - 0.0	24.3	7.2 - 41.3	0.5	0.1 - 1.3	3.1	0.9 - 6.5	0.0	0.0 - 0.0	35.3	11.6 - 64.1	0.61
3120003	10304	LITTLE CR	231.8	37.7	13 - 79	2.3	0.7 - 5.6	14.0	4.1 - 29.6	0.6	0.2 - 1.6	4.6	1.4 - 13.3	0.0	0.0 - 0.0	16.2	5.6 - 40.7	0.61
3120003	10306	OCKLAWAHA CR	78.5	15.0	5 - 28	0.0	0.0 - 0.0	4.9	1.7 - 9.7	0.3	0.1 - 0.7	1.0	0.3 - 2.3	0.0	0.0 - 0.0	8.9	2.7 - 17.9	0.61
3120003	10307	OCHOCKONEE R	67.2	11.8	3 - 29	0.0	0.0 - 0.0	4.6	1.4 - 10.9	0.1	0.0 - 0.4	0.4	0.1 - 1.3	0.0	0.0 - 0.0	6.7	2.1 - 17.6	0.95
3120003	10308	OCHOCKONEE R	24.1	21.7	8 - 46	0.0	0.0 - 0.0	11.0	4.1 - 24.6	0.2	0.1 - 0.8	1.3	0.4 - 3.4	0.0	0.0 - 0.0	9.2	3.0 - 23.9	0.61

8-digit HUC	ID	Name	Area	Catchment Yield		Point sources		Developed Land		Manure		Agricultural Land		Phosphate Mines		Soil parent rock		Frac
				kg/km^2	90% CI	kg/km^2	90% CI	kg/km^2	90% CI	kg/km^2	90% CI	kg/km^2	90% CI	kg/km^2	90% CI	kg/km^2	90% CI	
3120003	10309	OCHOCKONEE R	14.4	20.6	7 - 35	0.0	0.0 - 0.0	9.3	2.9 - 16.9	0.2	0.1 - 0.5	1.3	0.5 - 3.3	0.0	0.0 - 0.0	9.7	3.5 - 20.4	0.61
3120003	10310	OCHOCKONEE R	63.0	19.4	6 - 44	0.0	0.0 - 0.0	6.8	2.0 - 14.6	0.2	0.0 - 0.5	1.0	0.3 - 2.4	0.0	0.0 - 0.0	11.4	3.2 - 34.6	0.61
3120003	10311	LITTLE CR	10.5	24.1	9 - 56	0.0	0.0 - 0.0	2.8	1.0 - 6.0	0.1	0.0 - 0.4	0.7	0.2 - 2.1	0.0	0.0 - 0.0	20.5	7.2 - 49.0	0.61
3120003	10312	BEAR CR	11.5	20.3	6 - 50	0.0	0.0 - 0.0	5.7	1.9 - 12.8	0.3	0.1 - 0.8	1.4	0.5 - 3.5	0.0	0.0 - 0.0	12.9	3.8 - 34.1	0.61
3120003	10313	OCKLAWAHA CR	19.6	26.4	9 - 71	0.0	0.0 - 0.0	11.8	4.1 - 33.3	1.0	0.3 - 3.6	5.3	1.8 - 15.8	0.0	0.0 - 0.0	8.2	2.5 - 21.7	0.61
3130004	10314	CHATTAHOOCHEE	189.2	42.1	14 - 98	0.0	0.0 - 0.0	6.2	2.0 - 14.2	3.5	1.0 - 10.7	20.7	6.4 - 60.3	0.0	0.0 - 0.0	11.7	3.8 - 28.5	0.63
3130010	10315	FISHPOND DRAIN	356.9	55.1	20 - 114	0.0	0.0 - 0.0	8.3	2.9 - 16.4	3.9	1.2 - 12.3	30.1	11.0 - 74.4	0.0	0.0 - 0.0	12.8	4.6 - 28.3	0.63
3130010	10316	SPRING CR	218.4	46.0	14 - 94	1.3	0.5 - 2.6	7.2	2.2 - 15.4	2.7	0.9 - 7.3	23.1	6.4 - 55.4	0.0	0.0 - 0.0	11.6	4.0 - 27.8	0.63
3130008	10317	FLINT R	121.5	54.9	18 - 132	11.0	3.6 - 26.3	13.3	4.2 - 30.9	1.6	0.5 - 4.7	11.6	3.9 - 28.9	0.0	0.0 - 0.0	17.3	5.6 - 45.0	0.63
3130008	10318	SPRING CR	18.5	23.9	7 - 56	0.0	0.0 - 0.0	5.5	1.6 - 12.5	0.8	0.2 - 2.3	4.4	1.2 - 11.4	0.0	0.0 - 0.0	13.2	3.6 - 34.8	0.98
3130004	10321	CHATTAHOOCHEE	392.2	39.8	13 - 72	0.0	0.0 - 0.0	7.0	2.2 - 13.2	3.1	0.9 - 7.2	15.5	4.4 - 31.1	0.0	0.0 - 0.0	14.2	4.5 - 27.0	0.63
3130010	10322	FISHPOND DRAIN	19.3	34.3	14 - 74	0.0	0.0 - 0.0	15.1	5.8 - 34.8	0.8	0.3 - 2.6	2.8	0.9 - 7.6	0.0	0.0 - 0.0	15.5	6.8 - 36.8	0.63
3130010	10323	SPRING CR	111.3	44.2	15 - 129	0.0	0.0 - 0.0	4.7	1.4 - 12.3	2.7	0.7 - 9.2	19.1	6.3 - 62.5	0.0	0.0 - 0.0	17.7	5.0 - 54.2	0.63
3130008	10324	FLINT R	182.9	32.8	13 - 80	0.0	0.0 - 0.0	3.1	1.1 - 5.8	1.4	0.5 - 4.8	10.4	3.8 - 26.9	0.0	0.0 - 0.0	17.9	7.4 - 46.4	0.63
3120001	10325	WARD CR	261.2	39.5	14 - 72	0.0	0.0 - 0.0	7.5	2.8 - 14.3	1.3	0.4 - 3.4	4.5	1.5 - 10.8	0.0	0.0 - 0.0	26.1	9.5 - 58.1	0.90
3120001	10326	WARD CR	197.1	41.8	14 - 107	0.0	0.0 - 0.0	7.8	2.5 - 20.3	1.0	0.3 - 2.8	4.5	1.6 - 12.6	0.0	0.0 - 0.0	28.4	9.7 - 73.0	0.92
3150102	10328	TALKING ROCK C	57.2	32.0	10 - 60	0.0	0.0 - 0.0	5.3	1.9 - 11.0	9.6	3.1 - 27.9	2.3	0.7 - 5.5	0.0	0.0 - 0.0	14.8	5.4 - 33.1	0.20
3150102	10329	COOSAWATTEE R	1.6	17.8	6 - 48	0.0	0.0 - 0.0	3.0	0.9 - 7.5	0.6	0.2 - 2.2	1.2	0.4 - 3.4	0.0	0.0 - 0.0	12.9	4.1 - 39.6	0.28
3150102	10330	COOSAWATTEE R	69.2	30.4	11 - 73	0.0	0.0 - 0.0	5.6	2.5 - 12.2	3.5	1.1 - 12.9	0.9	0.3 - 2.8	0.0	0.0 - 0.0	20.4	6.7 - 51.5	0.20
3150102	10331	TALKING ROCK C	6.4	30.3	9 - 62	0.0	0.0 - 0.0	12.5	3.6 - 26.4	0.7	0.2 - 1.9	0.4	0.1 - 1.0	0.0	0.0 - 0.0	16.7	5.5 - 42.2	0.20
3150104	10333	LITTLE R	140.5	79.1	28 - 179	1.3	0.4 - 3.1	42.0	15.5 - 100.2	12.1	3.8 - 31.0	9.6	3.1 - 24.8	0.0	0.0 - 0.0	14.1	5.2 - 34.3	0.13
3150104	10334	NOONDAY CR	127.2	116.5	37 - 237	17.6	5.4 - 43.6	82.8	25.1 - 167.9	0.7	0.2 - 1.7	2.0	0.6 - 5.8	0.0	0.0 - 0.0	13.4	4.0 - 29.8	0.13
3150104	10335	ETOWAH R	225.5	24.3	6 - 53	10.5	2.8 - 25.5	6.0	1.7 - 13.8	0.6	0.1 - 1.4	0.6	0.2 - 1.8	0.0	0.0 - 0.0	6.6	1.5 - 15.1	0.31
3150104	10336	LITTLE R	39.2	78.1	22 - 161	0.0	0.0 - 0.0	56.7	17.1 - 129.8	2.3	0.6 - 6.0	1.7	0.5 - 4.8	0.0	0.0 - 0.0	17.3	4.9 - 37.4	0.13
3150104	10337	ETOWAH R	56.3	37.6	13 - 97	0.0	0.0 - 0.0	20.4	7.2 - 53.3	1.7	0.5 - 4.8	1.1	0.4 - 3.2	0.0	0.0 - 0.0	14.5	4.2 - 38.6	0.13
3150104	10338	NOONDAY CR	3.5	87.8	29 - 178	0.0	0.0 - 0.0	64.7	20.8 - 153.6	0.4	0.1 - 1.0	0.2	0.1 - 0.5	0.0	0.0 - 0.0	22.5	6.9 - 51.0	0.13
3150104	10339	LITTLE R	20.1	89.0	36 - 192	0.0	0.0 - 0.0	58.8	22.7 - 127.8	4.9	1.7 - 13.8	3.8	1.4 - 9.4	0.0	0.0 - 0.0	21.5	8.9 - 51.8	0.13
3130001	10340	CHESTATEE R	54.4	35.7	11 - 65	0.0	0.0 - 0.0	12.4	3.5 - 26.1	5.7	1.9 - 16.6	2.8	0.9 - 6.9	0.0	0.0 - 0.0	14.8	4.5 - 35.4	0.04
3130001	10341	LITTLE R, W FK	6.5	119.3	40 - 256	0.0	0.0 - 0.0	17.2	5.8 - 40.0	59.8	16.5 - 152.5	27.4	10.4 - 73.6	0.0	0.0 - 0.0	14.9	5.4 - 34.3	0.04
3130001	10342	CHATTAHOOCHEE	129.0	72.0	26 - 137	0.0	0.0 - 0.0	12.6	4.4 - 24.5	31.9	11.4 - 89.0	13.8	4.9 - 31.7	0.0	0.0 - 0.0	13.7	5.3 - 30.6	0.04
3130001	10343	FLAT CR	20.0	274.9	93 - 597	160.8	57.5 - 377.2	97.9	32.9 - 232.0	5.1	1.6 - 15.6	2.3	0.8 - 6.4	0.0	0.0 - 0.0	8.8	3.2 - 18.2	0.04
3130001	10344	CHATTAHOOCHEE	262.2	16.7	5 - 38	0.0	0.0 - 0.1	6.6	1.9 - 16.6	3.6	1.1 - 11.2	2.2	0.7 - 5.7	0.0	0.0 - 0.0	4.3	1.6 - 10.1	0.16
3130001	10345	CHATTAHOOCHEE	3.4	57.8	23 - 107	0.0	0.0 - 0.0	16.6	5.8 - 34.6	3.1	1.0 - 7.0	1.6	0.5 - 3.8	0.0	0.0 - 0.0	36.5	14.5 - 76.9	0.04
3130001	10346	CHATTAHOOCHEE	66.3	187.9	59 - 450	109.9	32.9 - 277.0	44.7	12.2 - 107.1	8.9	2.9 - 24.5	3.6	1.2 - 10.0	0.0	0.0 - 0.0	20.8	6.3 - 52.5	0.04
3130001	10347	LITTLE R, E FK	111.8	85.5	27 - 179	0.0	0.0 - 0.0	17.3	5.5 - 35.4	30.9	9.1 - 75.2	14.4	4.4 - 38.0	0.0	0.0 - 0.0	22.9	7.2 - 51.0	0.04
3130001	10348	CHESTATEE R	183.3	71.8	23 - 127	0.0	0.0 - 0.0	21.0	6.8 - 37.6	19.9	5.8 - 47.5	9.0	2.7 - 20.1	0.0	0.0 - 0.0	21.9	7.4 - 48.3	0.04
3130001	10349	LITTLE R, W FK	7.7	84.8	26 - 160	0.0	0.0 - 0.0	22.5	6.8 - 48.2	32.8	9.9 - 81.3	14.3	4.2 - 35.8	0.0	0.0 - 0.0	15.3	4.2 - 37.9	0.04
3130001	10350	CHATTAHOOCHEE	29.8	70.7	20 - 133	0.0	0.0 - 0.0	51.4	13.9 - 103.4	5.8	1.6 - 16.6	2.2	0.7 - 5.6	0.0	0.0 - 0.0	11.2	3.4 - 23.5	0.04
3130001	10351	FLAT CR	54.4	85.7	31 - 171	0.0	0.0 - 0.0	46.1	16.0 - 96.0	19.2	6.0 - 62.5	8.2	2.9 - 21.5	0.0	0.0 - 0.0	12.2	4.5 - 30.6	0.04

8-digit HUC	ID	Name	Area	Catchment Yield		Point sources		Developed Land		Manure		Agricultural Land		Phosphate Mines		Soil parent rock		Frac
				kg/km^2	90% CI	kg/km^2	90% CI	kg/km^2	90% CI	kg/km^2	90% CI	kg/km^2	90% CI	kg/km^2	90% CI	kg/km^2	90% CI	
3060102	10353	TALLULAH R	150.9	18.8	6 - 40	0.0	0.0 - 0.0	5.8	1.8 - 12.4	0.3	0.1 - 1.0	0.3	0.1 - 1.0	0.0	0.0 - 0.0	12.3	3.8 - 30.3	0.09
3060102	10354	TUGALOO R	15.8	35.4	11 - 59	0.0	0.0 - 0.0	13.5	4.7 - 24.9	5.7	1.7 - 13.8	3.3	1.1 - 7.7	0.0	0.0 - 0.0	12.8	4.3 - 28.2	0.13
3060102	10355	CHAUGA R	16.0	30.5	12 - 67	0.0	0.0 - 0.0	7.8	2.9 - 19.3	7.0	2.2 - 17.4	6.1	2.3 - 14.2	0.0	0.0 - 0.0	9.5	3.8 - 25.9	0.13
3060102	10356	ROCK CR	29.7	47.7	13 - 102	0.0	0.0 - 0.0	12.5	3.4 - 29.1	13.5	3.5 - 41.6	8.1	2.0 - 21.8	0.0	0.0 - 0.0	13.6	4.1 - 31.3	0.13
3060102	10357	CHOESTEA CR	55.4	41.5	15 - 70	0.9	0.3 - 1.8	9.1	3.2 - 18.3	9.9	3.3 - 28.0	10.5	3.6 - 23.9	0.0	0.0 - 0.0	11.1	3.9 - 23.0	0.13
3060102	10358	LITTLE CHOESTOI	19.0	53.7	16 - 94	0.0	0.0 - 0.0	5.9	1.6 - 11.2	15.4	4.6 - 39.6	17.9	5.4 - 40.6	0.0	0.0 - 0.0	14.5	4.2 - 34.2	0.13
3060101	10359	CONECROSS CR	46.9	59.8	22 - 124	0.0	0.0 - 0.0	6.9	2.4 - 15.2	17.3	5.6 - 49.3	19.4	7.5 - 51.7	0.0	0.0 - 0.0	16.2	6.4 - 35.5	0.13
3060101	10360	SENECA CR	21.9	56.6	19 - 134	0.0	0.0 - 0.0	27.6	9.1 - 74.4	6.8	1.9 - 17.4	7.0	2.0 - 21.4	0.0	0.0 - 0.0	15.2	5.4 - 43.2	0.13
3060101	10361	SENECA R	31.4	27.8	8 - 60	0.0	0.0 - 0.0	11.4	3.5 - 24.6	1.4	0.4 - 4.1	2.1	0.7 - 5.4	0.0	0.0 - 0.0	12.9	4.0 - 30.7	0.13
3060101	10362	SIXMILE CR	33.7	33.9	10 - 75	0.0	0.0 - 0.0	8.6	2.5 - 20.0	2.1	0.6 - 4.9	10.3	3.2 - 27.1	0.0	0.0 - 0.0	12.9	4.0 - 31.7	0.13
3060101	10363	TWELVEMILE CR	21.8	46.0	18 - 80	5.8	2.1 - 12.1	16.2	6.6 - 31.1	2.0	0.7 - 4.7	8.6	2.8 - 20.1	0.0	0.0 - 0.0	13.4	5.1 - 25.2	0.13
3060101	10364	EIGHTEENMILE CI	29.3	53.1	18 - 104	13.0	4.2 - 30.1	13.1	4.5 - 29.1	2.4	0.7 - 6.9	6.1	1.9 - 15.4	0.0	0.0 - 0.0	18.6	6.7 - 40.6	0.13
3060101	10365	THREE AND TWEN	230.4	50.1	19 - 94	0.0	0.0 - 0.0	11.3	4.4 - 22.9	5.4	1.6 - 15.4	17.2	6.1 - 54.1	0.0	0.0 - 0.0	16.2	5.9 - 35.8	0.13
3060101	10366	SIX AND TWENTY	84.9	49.5	17 - 98	4.5	1.5 - 10.2	11.4	3.7 - 25.0	4.8	1.3 - 13.6	14.1	4.5 - 31.2	0.0	0.0 - 0.0	14.8	5.1 - 37.7	0.13
3060101	10367	SENECA R	40.9	44.0	14 - 83	0.0	0.0 - 0.0	6.3	1.9 - 12.0	3.5	0.9 - 9.5	10.0	3.1 - 20.9	0.0	0.0 - 0.0	24.2	7.2 - 50.6	0.13
3060101	10368	THREE AND TWEN	11.1	55.9	18 - 120	8.3	2.5 - 19.9	9.0	2.9 - 20.9	4.0	1.0 - 10.4	13.0	4.1 - 33.8	0.0	0.0 - 0.0	21.6	7.1 - 54.7	0.13
3060101	10369	SENECA R	21.0	50.3	17 - 97	2.3	0.8 - 5.1	15.0	5.1 - 29.5	3.4	0.9 - 10.6	8.3	2.9 - 20.4	0.0	0.0 - 0.0	21.3	6.9 - 44.1	0.13
3060101	10370	SENECA R	13.2	38.9	14 - 90	0.0	0.0 - 0.0	7.4	2.6 - 17.8	2.4	0.7 - 5.9	6.0	2.2 - 17.0	0.0	0.0 - 0.0	23.2	8.1 - 55.9	0.13
3060101	10371	SENECA R	21.7	30.2	10 - 74	0.0	0.0 - 0.0	1.7	0.6 - 4.1	3.9	1.2 - 13.3	4.8	1.6 - 13.3	0.0	0.0 - 0.0	19.7	6.5 - 50.1	0.13
3060101	10372	SENECA R	53.0	267.0	83 - 540	207.1	66.7 - 441.9	33.4	11.4 - 68.3	4.4	1.3 - 10.4	6.4	2.0 - 15.6	0.0	0.0 - 0.0	15.7	5.2 - 33.3	0.13
3060101	10373	SENECA R	14.8	34.1	12 - 73	0.0	0.0 - 0.0	15.6	5.1 - 31.8	1.3	0.4 - 3.7	0.7	0.2 - 1.9	0.0	0.0 - 0.0	16.6	5.2 - 41.7	0.13
3060102	10374	CHAUGA R	13.7	55.8	18 - 118	0.0	0.0 - 0.0	6.5	2.1 - 13.9	15.1	4.8 - 56.8	12.3	4.4 - 29.8	0.0	0.0 - 0.0	22.0	7.1 - 54.8	0.13
3060102	10375	CHAUGA R	391.2	70.9	25 - 157	0.1	0.0 - 0.1	11.0	3.9 - 25.4	22.8	6.6 - 68.9	17.8	6.7 - 47.2	0.0	0.0 - 0.0	19.3	6.5 - 47.7	0.13
3060102	10378	CHAUGA R	29.0	33.7	13 - 72	0.0	0.0 - 0.0	7.9	3.0 - 18.4	6.0	2.0 - 17.1	3.5	1.3 - 10.0	0.0	0.0 - 0.0	16.3	6.0 - 37.5	0.13
3060103	10379	SENECA R	191.1	22.1	7 - 66	0.0	0.0 - 0.0	3.6	1.1 - 10.4	6.8	1.8 - 25.4	5.1	1.6 - 16.6	0.0	0.0 - 0.0	6.6	2.2 - 17.4	0.41
3060102	10380	TUGALOO R	6.2	32.4	9 - 75	0.0	0.0 - 0.0	4.8	1.3 - 12.2	5.7	1.5 - 15.5	4.5	1.4 - 15.6	0.0	0.0 - 0.0	17.3	4.8 - 43.6	0.13
3060102	10381	CHAUGA R	5.2	33.6	11 - 70	0.0	0.0 - 0.0	13.7	5.0 - 31.3	5.7	1.6 - 17.0	4.0	1.3 - 9.6	0.0	0.0 - 0.0	10.2	3.8 - 26.8	0.13
3060102	10382	ROCK CR	1.0	35.0	10 - 75	0.0	0.0 - 0.0	13.5	3.6 - 32.0	2.3	0.6 - 7.7	1.4	0.4 - 3.8	0.0	0.0 - 0.0	17.8	5.3 - 44.5	0.13
3060102	10383	CHOESTEA CR	13.2	24.7	9 - 47	0.0	0.0 - 0.0	2.6	0.9 - 5.2	3.3	0.9 - 10.2	2.5	0.8 - 6.2	0.0	0.0 - 0.0	16.2	6.0 - 38.9	0.13
3060102	10384	LITTLE CHOESTOI	12.4	52.6	17 - 111	0.0	0.0 - 0.0	14.5	4.7 - 31.4	10.7	2.9 - 27.8	11.9	4.0 - 29.6	0.0	0.0 - 0.0	15.5	6.0 - 35.3	0.13
3060101	10385	CONECROSS CR	35.5	54.2	16 - 129	0.0	0.0 - 0.0	5.0	1.6 - 13.0	11.1	2.9 - 35.9	13.7	4.3 - 45.9	0.0	0.0 - 0.0	24.4	6.5 - 67.4	0.13
3060101	10386	SENECA CR	12.7	38.8	14 - 82	0.0	0.0 - 0.0	7.8	2.3 - 16.7	6.2	1.9 - 17.6	5.6	1.9 - 15.1	0.0	0.0 - 0.0	19.3	6.8 - 46.4	0.13
3060101	10387	SENECA R	12.9	24.9	8 - 54	0.0	0.0 - 0.0	7.4	2.6 - 14.1	1.4	0.4 - 4.1	1.1	0.3 - 2.6	0.0	0.0 - 0.0	14.9	4.7 - 33.6	0.13
3060101	10388	SIXMILE CR	3.1	24.9	8 - 55	0.0	0.0 - 0.0	8.3	2.7 - 18.5	0.4	0.1 - 1.1	0.2	0.1 - 0.5	0.0	0.0 - 0.0	16.0	5.8 - 38.2	0.13
3060101	10389	TWELVEMILE CR	21.2	170.5	46 - 367	131.5	35.3 - 281.9	23.3	5.6 - 48.7	0.7	0.2 - 1.9	1.4	0.4 - 3.5	0.0	0.0 - 0.0	13.5	3.7 - 29.1	0.13
3060101	10390	EIGHTEENMILE CI	5.9	43.4	15 - 120	0.0	0.0 - 0.0	11.5	4.0 - 35.7	3.7	1.1 - 12.3	9.0	3.0 - 24.9	0.0	0.0 - 0.0	19.1	7.5 - 53.2	0.13
3060101	10391	THREE AND TWEN	8.7	47.6	19 - 79	0.0	0.0 - 0.0	16.4	5.6 - 30.6	3.7	1.1 - 8.0	9.5	3.6 - 20.0	0.0	0.0 - 0.0	18.1	6.9 - 33.4	0.13
3060101	10392	SIX AND TWENTY	97.3	69.1	22 - 122	0.7	0.2 - 1.4	37.0	11.7 - 73.8	3.9	1.2 - 11.1	11.6	3.6 - 28.1	0.0	0.0 - 0.0	15.9	5.5 - 37.0	0.13
3060102	10393	EASTANOLEE CR	27.0	44.3	14 - 98	0.0	0.0 - 0.0	12.9	3.9 - 31.4	10.6	3.1 - 30.4	5.0	1.4 - 14.8	0.0	0.0 - 0.0	15.8	4.5 - 38.1	0.13

8-digit HUC	ID	Name	Area	Catchment Yield		Point sources		Developed Land		Manure		Agricultural Land		Phosphate Mines		Soil parent rock		Frac
				kg/km^2	90% CI	kg/km^2	90% CI	kg/km^2	90% CI	kg/km^2	90% CI	kg/km^2	90% CI	kg/km^2	90% CI	kg/km^2	90% CI	
3060102	10394	EASTANOLEE CR	66.0	105.0	30 - 205	23.6	7.2 - 53.2	29.3	8.0 - 59.8	22.7	6.3 - 57.7	13.9	3.6 - 41.7	0.0	0.0 - 0.0	15.4	4.9 - 38.5	0.13
3060102	10395	LITTLE BEAVERD	14.7	46.6	18 - 119	0.0	0.0 - 0.0	5.2	1.9 - 13.0	3.5	1.1 - 12.0	10.6	3.8 - 30.9	0.0	0.0 - 0.0	27.3	10.8 - 73.9	0.13
3060102	10396	LITTLE BEAVERD	49.5	60.8	25 - 116	0.0	0.0 - 0.0	9.5	3.8 - 20.0	12.0	3.7 - 29.7	23.6	9.2 - 55.0	0.0	0.0 - 0.0	15.8	5.4 - 36.9	0.13
3060103	10397	ROCKY R	13.3	34.5	11 - 92	0.0	0.0 - 0.0	6.6	2.1 - 16.4	3.5	1.0 - 11.6	9.4	2.8 - 26.7	0.0	0.0 - 0.0	14.9	4.5 - 47.1	0.15
3060103	10398	ROCKY R	57.8	14.4	5 - 24	0.0	0.0 - 0.0	3.2	1.0 - 6.0	1.4	0.4 - 3.2	4.2	1.5 - 8.4	0.0	0.0 - 0.0	5.6	2.0 - 11.5	0.39
3060101	10399	WHITEWATER R	91.9	60.9	20 - 111	2.7	0.9 - 5.5	23.1	8.5 - 43.9	0.2	0.1 - 0.5	1.6	0.5 - 3.9	0.0	0.0 - 0.0	33.4	10.7 - 67.0	0.01
3060101	10400	TOXAWAY R	128.1	41.0	13 - 75	0.7	0.2 - 1.3	11.7	3.4 - 21.3	0.3	0.1 - 0.8	1.4	0.4 - 3.6	0.0	0.0 - 0.0	26.9	8.1 - 55.5	0.01
3060101	10401	EASTATOE CR	111.0	23.0	8 - 57	0.0	0.0 - 0.1	4.6	1.5 - 10.5	0.3	0.1 - 1.0	1.2	0.4 - 3.3	0.0	0.0 - 0.0	16.8	6.0 - 39.5	0.01
3060101	10402	CANE CR	86.7	46.5	16 - 103	0.0	0.0 - 0.0	15.7	5.0 - 34.3	10.1	3.1 - 31.6	10.6	3.3 - 29.2	0.0	0.0 - 0.0	10.1	3.8 - 25.8	0.04
3060101	10403	CROOKED CR	16.4	53.9	19 - 113	0.0	0.0 - 0.0	9.9	3.1 - 21.8	16.7	4.9 - 44.3	18.5	5.4 - 55.4	0.0	0.0 - 0.0	8.9	3.0 - 19.6	0.04
3060101	10404	LITTLE R	43.5	30.0	9 - 59	0.0	0.0 - 0.0	8.6	2.6 - 16.9	5.8	1.5 - 15.4	5.6	1.8 - 14.8	0.0	0.0 - 0.0	10.0	3.4 - 21.6	0.04
3060101	10405	KEOWEE R	42.8	29.9	11 - 57	0.0	0.0 - 0.0	10.5	3.5 - 21.8	3.6	1.2 - 9.8	3.9	1.3 - 9.6	0.0	0.0 - 0.0	12.0	4.5 - 23.4	0.04
3060101	10406	KEOWEE R	1.0	12.5	4 - 24	0.0	0.0 - 0.0	3.5	1.1 - 7.3	1.9	0.4 - 4.5	1.9	0.5 - 4.8	0.0	0.0 - 0.0	5.2	1.7 - 11.1	0.12
3060101	10407	TOXAWAY R	3.8	7.0	3 - 14	0.0	0.0 - 0.0	0.8	0.3 - 1.6	1.4	0.4 - 3.9	1.4	0.4 - 3.1	0.0	0.0 - 0.0	3.4	1.4 - 8.2	0.04
3060101	10408	TOXAWAY R	198.6	37.2	13 - 72	0.3	0.1 - 0.6	3.8	1.5 - 7.4	0.9	0.3 - 2.5	1.4	0.5 - 3.9	0.0	0.0 - 0.0	30.8	10.6 - 63.3	0.01
3060101	10409	CANE CR	1.4	26.1	8 - 59	0.0	0.0 - 0.0	11.0	3.5 - 27.4	2.6	0.8 - 9.9	1.9	0.7 - 5.7	0.0	0.0 - 0.0	10.5	3.2 - 27.6	0.04
3060101	10410	CANE CR	26.2	32.1	10 - 60	0.0	0.0 - 0.0	6.4	2.1 - 11.5	6.2	1.8 - 15.6	6.2	2.2 - 16.8	0.0	0.0 - 0.0	13.3	4.1 - 31.5	0.04
3060101	10411	WHITEWATER R	1.9	30.5	10 - 65	0.0	0.0 - 0.0	0.0	0.0 - 0.0	0.1	0.0 - 0.3	0.0	0.0 - 0.0	0.0	0.0 - 0.0	30.3	9.7 - 64.6	0.01
3060101	10413	EASTATOE CR	15.5	37.7	13 - 72	0.0	0.0 - 0.0	15.5	4.9 - 32.8	0.7	0.2 - 1.6	3.5	0.9 - 8.3	0.0	0.0 - 0.0	18.1	5.9 - 42.2	0.01
3060101	10414	CANE CR	44.7	47.9	18 - 85	0.0	0.0 - 0.0	23.5	8.1 - 47.3	4.1	1.3 - 11.5	4.0	1.3 - 8.9	0.0	0.0 - 0.0	16.3	5.6 - 33.5	0.04
3060101	10415	CROOKED CR	9.8	38.1	13 - 84	0.0	0.0 - 0.0	9.4	2.8 - 19.1	6.0	1.6 - 17.9	5.6	2.2 - 15.2	0.0	0.0 - 0.0	17.1	5.5 - 39.5	0.04
3060101	10416	LITTLE R	9.1	40.3	14 - 69	0.0	0.0 - 0.0	24.7	7.6 - 47.0	1.8	0.5 - 4.3	1.1	0.4 - 2.5	0.0	0.0 - 0.0	12.8	4.7 - 26.2	0.04
3060101	10417	KEOWEE R	106.3	23.1	7 - 62	0.0	0.0 - 0.0	6.7	2.0 - 18.4	1.1	0.3 - 3.0	2.7	0.8 - 7.0	0.0	0.0 - 0.0	12.7	3.9 - 36.2	0.04
3050109	10418	SALADA R	42.4	25.8	10 - 65	0.0	0.0 - 0.0	7.4	2.9 - 18.9	2.1	0.6 - 6.1	5.5	1.8 - 13.8	0.0	0.0 - 0.0	10.8	4.5 - 28.1	0.06
3050109	10419	REEDY R	6.6	26.2	9 - 65	0.0	0.0 - 0.0	2.6	0.9 - 5.6	2.0	0.6 - 5.8	5.7	1.9 - 14.6	0.0	0.0 - 0.0	15.8	5.9 - 43.2	0.06
3050109	10420	RABON CR	98.1	28.9	9 - 64	0.0	0.0 - 0.0	4.5	1.4 - 10.8	2.4	0.6 - 7.4	7.4	2.1 - 20.6	0.0	0.0 - 0.0	14.5	4.4 - 36.2	0.06
3050109	10421	CANE CR	58.8	25.7	8 - 56	0.0	0.0 - 0.0	5.2	1.8 - 11.5	2.1	0.6 - 5.6	5.7	1.8 - 15.9	0.0	0.0 - 0.0	12.8	4.2 - 30.5	0.06
3050109	10422	LITTLE R	2.5	30.2	11 - 63	0.0	0.0 - 0.0	3.5	1.1 - 7.5	3.9	1.2 - 10.8	8.9	2.9 - 20.4	0.0	0.0 - 0.0	13.9	4.7 - 32.0	0.10
3050109	10424	SALADA R	27.2	37.7	12 - 76	0.0	0.0 - 0.0	13.6	4.3 - 28.5	2.0	0.6 - 5.6	6.7	2.2 - 16.5	0.0	0.0 - 0.0	15.5	5.0 - 35.2	0.06
3050109	10425	REEDY R	38.2	34.3	12 - 73	0.0	0.0 - 0.0	7.6	2.6 - 16.8	2.6	0.7 - 9.4	7.6	2.8 - 19.6	0.0	0.0 - 0.0	16.5	5.6 - 46.1	0.06
3050109	10426	SALADA R	51.6	25.4	10 - 67	0.0	0.0 - 0.0	6.6	2.5 - 16.5	1.9	0.6 - 5.5	4.8	1.9 - 14.4	0.0	0.0 - 0.0	12.2	4.5 - 34.6	0.06
3050109	10427	REEDY R	16.9	34.4	12 - 72	0.0	0.0 - 0.0	5.8	1.6 - 12.6	2.9	0.9 - 8.3	7.4	2.5 - 17.3	0.0	0.0 - 0.0	18.2	6.2 - 42.9	0.06
3050109	10428	RABON CR	4.5	37.5	13 - 71	0.0	0.0 - 0.0	4.8	1.6 - 10.0	3.4	1.0 - 9.1	10.6	3.2 - 24.6	0.0	0.0 - 0.0	18.7	6.2 - 41.4	0.06
3050109	10429	CANE CR	26.0	31.0	10 - 72	0.0	0.0 - 0.0	8.6	2.9 - 19.5	2.0	0.5 - 5.8	4.0	1.2 - 10.9	0.0	0.0 - 0.0	16.3	5.6 - 42.0	0.06
3070103	10430	SOUTH R	108.7	32.4	11 - 71	0.0	0.0 - 0.0	6.7	2.0 - 15.1	2.1	0.5 - 5.3	8.6	2.9 - 22.0	0.0	0.0 - 0.0	15.0	5.0 - 36.1	0.58
3070103	10431	YELLOW R	188.5	43.1	15 - 110	0.0	0.0 - 0.0	22.1	8.0 - 52.3	1.9	0.6 - 6.7	6.5	2.2 - 19.3	0.0	0.0 - 0.0	12.6	4.7 - 38.8	0.58
3070103	10432	TUSSAHAW CR	190.2	33.1	11 - 61	0.0	0.0 - 0.0	6.9	2.2 - 14.2	1.9	0.6 - 4.7	9.9	3.5 - 26.3	0.0	0.0 - 0.0	14.5	5.0 - 32.4	0.58
3070103	10433	ALCOVY R	90.4	36.8	13 - 68	0.0	0.0 - 0.0	11.8	4.0 - 23.5	2.5	0.8 - 6.1	9.4	3.2 - 20.4	0.0	0.0 - 0.0	13.1	4.8 - 28.3	0.58
3070103	10434	BEAR CR	90.0	36.7	13 - 100	0.0	0.0 - 0.0	5.4	2.1 - 14.5	4.1	1.3 - 12.5	14.3	5.4 - 44.8	0.0	0.0 - 0.0	12.9	5.3 - 35.8	0.58

8-digit HUC	ID	Name	Area	Catchment Yield kg/km^2	Catchment Yield 90% CI	Point sources kg/km^2	Point sources 90% CI	Developed Land kg/km^2	Developed Land 90% CI	Manure kg/km^2	Manure 90% CI	Agricultural Land kg/km^2	Agricultural Land 90% CI	Phosphate Mines kg/km^2	Phosphate Mines 90% CI	Soil parent rock kg/km^2	Soil parent rock 90% CI	Frac
3070103	10435	SOUTH R	14.1	21.2	6 - 50	0.0	0.0 - 0.0	4.4	1.3 - 9.8	1.0	0.3 - 3.1	2.8	1.0 - 7.2	0.0	0.0 - 0.0	13.1	4.0 - 36.1	0.81
3070103	10436	SOUTH R	4.6	41.3	11 - 86	0.0	0.0 - 0.0	16.0	4.6 - 35.1	0.6	0.1 - 1.7	1.4	0.4 - 3.2	0.0	0.0 - 0.0	23.3	6.1 - 57.7	0.58
3070103	10437	SOUTH R	6.3	29.9	10 - 60	0.0	0.0 - 0.0	13.4	4.3 - 28.3	1.1	0.3 - 3.3	3.0	1.0 - 8.2	0.0	0.0 - 0.0	12.4	3.9 - 29.2	0.58
3070103	10438	ALCOVY R	49.9	37.1	14 - 84	0.0	0.0 - 0.0	10.1	3.9 - 24.4	5.4	1.8 - 14.5	5.6	2.2 - 16.0	0.0	0.0 - 0.0	16.0	6.1 - 37.0	0.58
3070103	10439	SOUTH R	7.1	34.6	11 - 74	0.0	0.0 - 0.0	10.5	3.1 - 24.9	1.3	0.4 - 4.1	7.3	2.4 - 19.2	0.0	0.0 - 0.0	15.5	4.8 - 36.0	0.58
3070103	10440	YELLOW R	6.7	21.4	7 - 42	0.0	0.0 - 0.0	5.6	1.7 - 10.6	1.6	0.4 - 3.8	3.4	1.2 - 8.4	0.0	0.0 - 0.0	10.8	3.7 - 24.0	0.58
3070103	10441	TUSSAHAW CR	27.3	34.5	10 - 65	0.0	0.0 - 0.0	8.9	2.3 - 20.2	1.3	0.4 - 3.2	8.8	2.5 - 19.6	0.0	0.0 - 0.0	15.5	5.1 - 38.7	0.58
3070103	10442	ALCOVY R	2.6	30.2	11 - 81	0.0	0.0 - 0.0	2.1	0.7 - 4.9	1.9	0.6 - 5.9	7.6	2.6 - 22.8	0.0	0.0 - 0.0	18.6	6.3 - 51.0	0.58
3070103	10443	BEAR CR	1.4	20.5	7 - 40	0.0	0.0 - 0.0	0.0	0.0 - 0.0	1.3	0.4 - 3.4	1.3	0.4 - 3.3	0.0	0.0 - 0.0	17.8	5.9 - 39.4	0.58
3070101	10444	LITTLE R	76.4	30.0	11 - 52	2.4	0.9 - 4.2	9.3	3.4 - 17.2	2.1	0.7 - 5.4	3.4	1.1 - 7.7	0.0	0.0 - 0.0	12.9	4.4 - 28.1	0.54
3070101	10445	MURDER CR	38.8	20.7	6 - 48	0.0	0.0 - 0.0	5.5	1.8 - 12.2	1.1	0.3 - 3.6	1.7	0.5 - 4.8	0.0	0.0 - 0.0	12.3	3.7 - 34.3	0.54
3070101	10446	LITTLE CEDAR CR	42.2	19.8	7 - 46	0.0	0.0 - 0.0	5.3	1.8 - 13.1	1.0	0.3 - 3.3	2.6	0.9 - 6.3	0.0	0.0 - 0.0	10.9	3.6 - 27.9	0.54
3070101	10447	TAYLOR CR	59.5	16.5	5 - 36	0.0	0.0 - 0.0	2.1	0.6 - 4.9	1.1	0.3 - 3.5	2.6	0.6 - 6.3	0.0	0.0 - 0.0	10.8	3.5 - 25.7	0.54
3070101	10448	ROOTY CR	101.5	33.6	13 - 89	1.5	0.5 - 4.5	6.6	2.4 - 17.6	4.7	1.5 - 13.5	9.4	3.8 - 31.1	0.0	0.0 - 0.0	11.4	4.2 - 31.9	0.54
3070101	10449	CROOKED CR	81.0	25.5	9 - 59	0.0	0.0 - 0.0	3.4	1.2 - 7.8	3.7	1.3 - 11.6	7.3	2.7 - 20.7	0.0	0.0 - 0.0	11.0	4.1 - 25.1	0.54
3070101	10450	OCONEE R	13.7	11.7	4 - 23	0.0	0.0 - 0.0	2.6	0.9 - 5.2	0.7	0.2 - 1.8	0.4	0.1 - 1.0	0.0	0.0 - 0.0	8.0	2.8 - 16.4	0.54
3070101	10451	NEEL CR	2.7	21.2	8 - 64	0.0	0.0 - 0.0	4.2	1.3 - 9.7	0.3	0.1 - 1.1	0.4	0.1 - 1.3	0.0	0.0 - 0.0	16.3	6.0 - 54.4	0.54
3070101	10452	OCONEE R	140.5	16.1	7 - 39	0.0	0.0 - 0.0	4.3	1.6 - 10.5	0.5	0.2 - 1.7	1.4	0.5 - 3.8	0.0	0.0 - 0.0	9.9	3.9 - 26.3	0.88
3070101	10454	OCONEE R	25.7	27.1	7 - 46	0.0	0.0 - 0.0	8.2	2.0 - 15.0	0.9	0.2 - 3.0	1.4	0.4 - 3.1	0.0	0.0 - 0.0	16.6	4.5 - 32.1	0.54
3070101	10455	OCONEE R	117.5	18.9	6 - 34	0.0	0.0 - 0.0	3.7	1.0 - 6.6	0.6	0.2 - 1.5	1.1	0.3 - 2.3	0.0	0.0 - 0.0	13.5	4.2 - 28.6	0.54
3070101	10456	LITTLE R	57.7	29.3	9 - 68	0.0	0.0 - 0.0	12.3	3.7 - 28.6	1.4	0.4 - 3.8	2.2	0.6 - 5.7	0.0	0.0 - 0.0	13.3	4.0 - 31.8	0.54
3070101	10457	LITTLE R	32.1	25.5	9 - 53	0.0	0.0 - 0.0	8.8	3.0 - 20.0	1.3	0.4 - 3.8	1.2	0.4 - 2.9	0.0	0.0 - 0.0	14.1	4.4 - 32.2	0.54
3070101	10458	BIG CEDAR CR	7.7	22.2	7 - 61	0.0	0.0 - 0.0	5.3	1.6 - 16.7	1.9	0.6 - 7.1	1.7	0.5 - 5.4	0.0	0.0 - 0.0	13.3	4.1 - 35.2	0.54
3070101	10459	BIG CEDAR CR	5.6	30.1	11 - 67	0.0	0.0 - 0.0	11.5	3.9 - 25.2	0.9	0.3 - 2.7	0.6	0.2 - 1.6	0.0	0.0 - 0.0	17.0	5.6 - 38.8	0.54
3070101	10460	LITTLE R	6.2	30.9	12 - 61	0.0	0.0 - 0.0	10.5	3.3 - 22.1	2.3	0.7 - 6.0	4.0	1.2 - 8.9	0.0	0.0 - 0.0	14.1	4.6 - 29.3	0.54
3070101	10461	MURDER CR	1.0	20.0	8 - 41	0.0	0.0 - 0.0	2.1	0.8 - 4.7	1.1	0.4 - 2.8	0.6	0.2 - 1.7	0.0	0.0 - 0.0	16.2	6.0 - 33.9	0.54
3070101	10463	TAYLOR CR	2.7	17.4	7 - 39	0.0	0.0 - 0.0	3.7	1.5 - 8.5	0.6	0.2 - 2.0	1.3	0.5 - 3.2	0.0	0.0 - 0.0	11.7	4.6 - 26.6	0.54
3070101	10464	ROOTY CR	18.3	26.7	9 - 53	0.0	0.0 - 0.0	9.0	3.0 - 17.6	1.4	0.4 - 3.1	1.8	0.5 - 4.0	0.0	0.0 - 0.0	14.5	4.7 - 30.7	0.54
3070101	10465	CROOKED CR	16.9	25.1	7 - 73	0.0	0.0 - 0.0	7.4	2.1 - 17.0	1.4	0.4 - 5.0	2.6	0.8 - 7.0	0.0	0.0 - 0.0	13.6	4.1 - 37.4	0.54
3070101	10466	OCONEE R	27.2	21.4	6 - 49	0.0	0.0 - 0.0	6.6	1.8 - 14.2	0.5	0.1 - 1.5	0.5	0.2 - 1.2	0.0	0.0 - 0.0	13.8	3.3 - 34.4	0.54
3070101	10467	NEEL CR	7.8	20.3	7 - 49	0.0	0.0 - 0.0	2.7	1.0 - 6.9	0.5	0.2 - 1.6	2.8	1.0 - 8.1	0.0	0.0 - 0.0	14.3	5.3 - 36.0	0.54
3060103	10468	FISHING CR	190.8	27.1	9 - 57	0.0	0.0 - 0.0	4.0	1.2 - 9.4	4.2	1.2 - 11.3	6.3	2.0 - 15.6	0.0	0.0 - 0.0	12.6	4.5 - 28.6	0.42
3060103	10469	SOAP CR	94.2	32.6	12 - 60	0.0	0.0 - 0.0	5.7	2.0 - 10.5	3.7	1.2 - 8.3	10.0	3.4 - 25.2	0.0	0.0 - 0.0	13.1	4.8 - 30.7	0.42
3060105	10470	LITTLE R	5.5	32.1	9 - 73	0.0	0.0 - 0.0	3.5	0.9 - 7.5	0.4	0.1 - 1.1	3.0	0.8 - 8.0	0.0	0.0 - 0.0	25.2	7.5 - 68.9	0.42
3060103	10471	SAVANNAH R	27.0	17.3	5 - 40	0.0	0.0 - 0.0	4.9	1.7 - 11.9	0.7	0.2 - 2.1	1.4	0.4 - 3.5	0.0	0.0 - 0.0	10.3	3.0 - 26.5	0.42
3060103	10472	PISTOL CR	53.9	27.8	8 - 65	0.0	0.0 - 0.0	3.1	0.9 - 7.8	4.4	1.2 - 12.8	6.9	2.1 - 19.8	0.0	0.0 - 0.0	13.3	3.9 - 34.5	0.42
3060103	10473	NEWFORD CR	76.2	28.1	8 - 61	0.0	0.0 - 0.0	3.5	1.0 - 6.9	4.3	1.3 - 11.7	7.0	1.8 - 18.9	0.0	0.0 - 0.0	13.3	3.8 - 31.6	0.42
3060103	10474	LITTLE R	39.4	19.6	6 - 34	0.0	0.0 - 0.0	4.7	1.5 - 9.9	0.6	0.2 - 1.4	2.4	0.7 - 5.7	0.0	0.0 - 0.0	11.9	3.6 - 24.6	0.42
3060103	10475	LONG CANE CR	196.3	18.7	7 - 36	0.0	0.0 - 0.0	3.8	1.4 - 7.3	0.5	0.2 - 1.3	1.4	0.5 - 3.0	0.0	0.0 - 0.0	12.9	4.4 - 27.4	0.42

8-digit HUC	ID	Name	Area	Catchment Yield		Point sources		Developed Land		Manure		Agricultural Land		Phosphate Mines		Soil parent rock		Frac
				kg/km^2	90% CI	kg/km^2	90% CI	kg/km^2	90% CI	kg/km^2	90% CI	kg/km^2	90% CI	kg/km^2	90% CI	kg/km^2	90% CI	
3060103	10476	SAVANNAH R	18.2	16.2	6 - 34	0.0	0.0 - 0.0	4.4	1.6 - 9.1	0.2	0.1 - 0.5	1.2	0.4 - 2.8	0.0	0.0 - 0.0	10.4	3.9 - 26.6	0.92
3060103	10477	SAVANNAH R	1.0	26.6	9 - 73	0.0	0.0 - 0.0	1.1	0.4 - 2.7	0.2	0.1 - 0.7	0.0	0.0 - 0.0	0.0	0.0 - 0.0	25.3	8.9 - 69.6	0.42
3060103	10478	LITTLE R	79.0	37.1	12 - 79	0.0	0.0 - 0.0	16.2	5.2 - 34.2	0.6	0.1 - 1.8	2.6	0.8 - 7.2	0.0	0.0 - 0.0	17.7	5.3 - 45.0	0.42
3060103	10479	SAVANNAH R	124.1	24.4	7 - 53	0.0	0.0 - 0.0	5.6	1.7 - 13.3	0.7	0.2 - 2.5	1.7	0.5 - 3.7	0.0	0.0 - 0.0	16.4	5.1 - 39.5	0.42
3060103	10480	SAVANNAH R	33.6	27.0	8 - 65	0.0	0.0 - 0.0	3.2	0.9 - 7.0	0.5	0.1 - 1.7	1.2	0.3 - 3.2	0.0	0.0 - 0.0	22.1	6.6 - 53.5	0.42
3060103	10482	SAVANNAH R	230.8	22.2	7 - 38	0.0	0.0 - 0.0	5.1	1.7 - 9.4	1.1	0.3 - 2.5	4.6	1.5 - 9.9	0.0	0.0 - 0.0	11.4	3.8 - 25.9	0.42
3060104	10484	BROAD R	6.3	25.5	8 - 61	0.0	0.0 - 0.0	3.8	1.2 - 7.4	0.4	0.1 - 1.0	0.0	0.0 - 0.0	0.0	0.0 - 0.0	21.3	6.4 - 55.7	0.42
3060103	10485	FISHING CR	116.5	25.0	8 - 66	0.0	0.0 - 0.0	4.0	1.3 - 10.2	2.7	0.8 - 8.0	4.5	1.5 - 12.5	0.0	0.0 - 0.0	13.7	4.2 - 38.8	0.42
3060103	10486	SOAP CR	76.8	42.3	15 - 96	4.6	1.7 - 11.4	8.1	2.5 - 19.6	3.8	1.2 - 10.5	13.1	4.1 - 40.3	0.0	0.0 - 0.0	12.7	4.1 - 30.3	0.42
3060105	10487	LITTLE R	274.5	32.0	10 - 78	0.0	0.0 - 0.0	4.9	1.4 - 10.7	1.9	0.6 - 4.7	7.7	2.3 - 20.5	0.0	0.0 - 0.0	17.6	5.2 - 46.3	0.42
3060103	10488	SAVANNAH R	61.7	22.0	7 - 53	0.0	0.0 - 0.0	3.3	1.1 - 7.7	0.8	0.2 - 2.8	1.3	0.4 - 2.9	0.0	0.0 - 0.0	16.6	5.3 - 49.3	0.42
3060103	10489	PISTOL CR	7.5	25.8	10 - 64	0.0	0.0 - 0.0	6.4	2.2 - 16.8	0.6	0.2 - 1.4	1.7	0.6 - 5.0	0.0	0.0 - 0.0	17.1	6.3 - 42.6	0.42
3060103	10490	NEWFORD CR	9.6	25.7	8 - 56	0.0	0.0 - 0.0	7.2	2.3 - 15.2	1.0	0.3 - 2.8	1.8	0.6 - 4.2	0.0	0.0 - 0.0	15.7	5.1 - 41.6	0.42
3060103	10491	LITTLE R	71.8	24.1	8 - 62	0.0	0.0 - 0.0	4.8	1.6 - 11.9	0.6	0.2 - 1.9	2.1	0.7 - 5.6	0.0	0.0 - 0.0	16.6	5.5 - 48.7	0.42
3060103	10492	LONG CANE CR	8.5	30.7	10 - 78	0.0	0.0 - 0.0	4.0	1.2 - 10.6	1.3	0.4 - 4.2	6.4	1.9 - 18.4	0.0	0.0 - 0.0	18.9	6.9 - 52.7	0.42
3060204	10493	LITTLE OGEECHEI	367.2	36.7	15 - 90	12.8	4.7 - 34.1	8.4	3.0 - 19.7	0.4	0.1 - 1.3	2.4	0.8 - 7.7	0.0	0.0 - 0.0	12.6	4.8 - 35.6	1.00
3050109	10495	SALADA R	34.9	35.5	12 - 73	0.0	0.0 - 0.0	5.3	1.8 - 12.4	7.1	2.1 - 18.7	9.4	2.8 - 23.6	0.0	0.0 - 0.0	13.7	4.9 - 33.4	0.12
3050109	10496	BEAVER DAM CR	73.6	40.8	14 - 88	0.0	0.0 - 0.0	4.5	1.5 - 9.0	10.1	2.9 - 27.7	15.1	5.0 - 40.8	0.0	0.0 - 0.0	11.1	3.6 - 26.8	0.12
3050109	10497	BUSH R	295.7	61.5	20 - 155	18.3	5.4 - 47.2	13.0	3.8 - 34.0	7.3	1.7 - 20.5	12.2	3.6 - 30.9	0.0	0.0 - 0.0	10.7	3.4 - 29.6	0.12
3050109	10498	CAMPING CR	37.3	32.5	10 - 74	0.0	0.0 - 0.0	8.8	2.8 - 20.3	5.4	1.6 - 19.8	7.6	2.7 - 20.1	0.0	0.0 - 0.0	10.6	3.6 - 25.5	0.12
3050109	10499	HOLLOW CR	51.9	34.5	11 - 71	0.0	0.0 - 0.0	5.7	1.7 - 12.8	5.4	1.4 - 15.8	9.2	3.0 - 20.6	0.0	0.0 - 0.0	14.2	4.6 - 34.3	0.12
3050109	10500	HORSE CR	20.9	30.8	10 - 67	0.0	0.0 - 0.0	7.3	2.4 - 16.4	4.8	1.6 - 12.1	8.6	2.4 - 23.1	0.0	0.0 - 0.0	10.1	3.5 - 23.2	0.12
3050109	10501	WEST CR	56.7	32.9	11 - 65	0.0	0.0 - 0.0	11.9	3.8 - 25.2	4.1	1.5 - 9.9	5.4	1.7 - 16.0	0.0	0.0 - 0.0	11.5	4.1 - 26.1	0.12
3050109	10502	CLOUDS CR	169.8	33.5	10 - 59	1.1	0.4 - 2.4	4.9	1.6 - 9.1	7.5	2.3 - 20.3	9.7	3.5 - 26.0	0.0	0.0 - 0.0	10.3	3.0 - 23.7	0.12
3050109	10504	BIG CR	138.0	46.3	14 - 99	0.0	0.0 - 0.0	5.2	1.7 - 11.6	12.0	3.5 - 31.9	15.9	5.1 - 38.9	0.0	0.0 - 0.0	13.1	3.7 - 33.2	0.12
3050109	10505	SALADA R	266.1	11.5	4 - 27	0.0	0.0 - 0.0	3.3	0.9 - 8.1	0.8	0.2 - 2.7	1.5	0.4 - 3.8	0.0	0.0 - 0.0	5.9	1.7 - 17.2	0.42
3050109	10506	SALADA R	1.0	38.8	11 - 89	0.0	0.0 - 0.0	0.0	0.0 - 0.0	0.0	0.0 - 0.0	0.0	0.0 - 0.0	0.0	0.0 - 0.0	38.8	11.2 - 89.4	0.12
3050109	10507	SALADA R	154.8	37.8	14 - 77	0.0	0.0 - 0.0	6.8	2.5 - 13.8	6.3	2.1 - 20.5	8.6	2.8 - 18.7	0.0	0.0 - 0.0	16.1	5.1 - 43.5	0.12
3050109	10508	SALADA R	54.4	37.4	14 - 84	0.0	0.0 - 0.0	5.8	2.0 - 12.7	7.9	2.5 - 22.7	10.7	4.2 - 28.1	0.0	0.0 - 0.0	12.9	4.2 - 29.9	0.12
3050109	10509	SALADA R	14.4	33.6	10 - 69	0.0	0.0 - 0.0	2.1	0.6 - 4.0	8.8	2.1 - 22.2	12.5	3.5 - 32.3	0.0	0.0 - 0.0	10.3	2.8 - 21.7	0.12
3050109	10510	LITTLE SALUDA R	6.1	36.9	12 - 77	0.0	0.0 - 0.0	11.2	3.5 - 24.6	5.4	1.6 - 14.3	7.0	2.2 - 16.0	0.0	0.0 - 0.0	13.3	4.4 - 37.3	0.12
3050109	10511	LITTLE SALUDA R	74.8	52.6	17 - 110	0.0	0.0 - 0.0	6.0	1.8 - 13.9	14.5	4.5 - 41.8	19.0	6.5 - 58.9	0.0	0.0 - 0.0	13.2	4.3 - 32.7	0.12
3050109	10512	HOLLOW CR	41.3	41.2	14 - 96	0.0	0.0 - 0.0	8.5	3.1 - 20.8	5.7	2.1 - 16.8	10.4	3.8 - 30.4	0.0	0.0 - 0.0	16.5	5.3 - 48.7	0.12
3050109	10513	SALADA R	21.9	23.6	8 - 47	0.0	0.0 - 0.0	3.4	1.1 - 6.7	3.3	0.9 - 7.8	4.0	1.5 - 10.5	0.0	0.0 - 0.0	13.0	4.8 - 30.6	0.12
3050109	10514	BEAVER DAM CR	2.4	11.2	4 - 24	0.0	0.0 - 0.0	3.3	1.0 - 7.1	0.5	0.1 - 1.5	0.4	0.1 - 0.9	0.0	0.0 - 0.0	7.1	2.3 - 17.2	0.12
3050109	10515	BUSH R	9.5	25.3	8 - 46	0.0	0.0 - 0.0	4.1	1.4 - 8.6	4.2	1.3 - 11.9	5.7	1.8 - 13.5	0.0	0.0 - 0.0	11.2	4.2 - 27.3	0.12
3050109	10516	CAMPING CR	69.6	41.8	13 - 93	0.1	0.0 - 0.2	8.5	2.6 - 18.9	6.5	1.8 - 19.3	10.0	3.4 - 24.4	0.0	0.0 - 0.0	16.7	5.1 - 41.9	0.12
3050109	10517	HOLLOW CR	7.4	34.4	13 - 71	0.0	0.0 - 0.0	11.0	3.7 - 24.2	2.7	0.8 - 7.4	4.8	1.6 - 11.4	0.0	0.0 - 0.0	15.9	6.0 - 35.4	0.12
3050109	10518	HORSE CR	16.7	42.9	14 - 99	0.0	0.0 - 0.0	11.6	3.4 - 28.4	6.4	1.7 - 17.4	11.3	3.6 - 28.8	0.0	0.0 - 0.0	13.6	4.3 - 31.0	0.12

8-digit HUC	ID	Name	Area	Catchment Yield		Point sources		Developed Land		Manure		Agricultural Land		Phosphate Mines		Soil parent rock		Frac
				kg/km^2	90% CI	kg/km^2	90% CI	kg/km^2	90% CI	kg/km^2	90% CI	kg/km^2	90% CI	kg/km^2	90% CI	kg/km^2	90% CI	
3050109	10519	WEST CR	65.9	45.5	15 - 92	0.0	0.0 - 0.0	6.8	2.2 - 15.3	10.4	2.9 - 26.3	13.1	3.8 - 33.0	0.0	0.0 - 0.0	15.2	4.9 - 34.2	0.12
3050109	10520	LITTLE SALUDA R	12.5	59.1	17 - 128	0.0	0.0 - 0.0	4.4	1.3 - 10.1	16.9	4.6 - 43.8	22.5	6.7 - 60.7	0.0	0.0 - 0.0	15.3	5.3 - 39.1	0.12
3050109	10521	BIG CR	11.1	53.6	20 - 128	0.0	0.0 - 0.0	6.3	2.3 - 13.4	14.1	4.1 - 39.2	18.3	6.9 - 48.2	0.0	0.0 - 0.0	15.0	5.4 - 42.8	0.12
3050111	10522	SANTEE R	101.3	22.7	8 - 53	0.0	0.0 - 0.0	2.7	0.9 - 5.9	0.5	0.2 - 1.4	10.7	3.4 - 30.6	0.0	0.0 - 0.0	8.7	3.2 - 22.7	0.44
3050111	10523	SPRING GROVE CF	90.5	15.6	6 - 33	1.1	0.4 - 2.4	2.1	0.8 - 4.3	1.3	0.4 - 3.1	6.7	2.4 - 16.6	0.0	0.0 - 0.0	4.4	1.7 - 10.3	0.44
3050111	10524	HALFWAY SWAMI	177.9	24.6	7 - 58	2.1	0.7 - 5.1	6.1	1.7 - 15.7	0.5	0.1 - 1.6	10.7	3.4 - 29.5	0.0	0.0 - 0.0	5.2	1.9 - 13.9	0.44
3050111	10525	JACKS CR	57.8	35.0	11 - 71	0.0	0.0 - 0.0	5.5	1.6 - 11.4	4.2	1.2 - 11.7	18.9	5.9 - 44.6	0.0	0.0 - 0.0	6.4	1.9 - 15.5	0.44
3050111	10527	SANTEE R	34.8	10.4	4 - 25	0.0	0.0 - 0.0	1.6	0.6 - 3.7	0.4	0.1 - 1.3	1.9	0.6 - 5.2	0.0	0.0 - 0.0	6.5	2.2 - 18.3	0.98
3050111	10528	SANTEE R	93.7	27.5	8 - 70	0.0	0.0 - 0.0	4.3	1.3 - 10.3	2.1	0.6 - 7.0	12.9	4.0 - 38.7	0.0	0.0 - 0.0	8.1	2.9 - 21.1	0.44
3050111	10529	SANTEE R	8.4	24.3	7 - 51	0.0	0.0 - 0.0	0.7	0.2 - 1.5	0.3	0.1 - 0.9	7.0	2.2 - 19.4	0.0	0.0 - 0.0	16.2	4.8 - 38.9	0.44
3050111	10530	SANTEE R	573.3	32.0	11 - 75	0.0	0.0 - 0.0	5.6	1.8 - 13.2	2.7	0.9 - 8.0	12.9	5.3 - 31.6	0.0	0.0 - 0.0	10.7	3.9 - 30.8	0.44
3050111	10531	SANTEE R	39.6	23.5	8 - 55	0.0	0.0 - 0.0	1.2	0.4 - 2.4	0.7	0.2 - 1.7	11.9	4.1 - 27.3	0.0	0.0 - 0.0	9.7	3.0 - 23.6	0.44
3050111	10532	SPRING GROVE CF	5.9	14.3	5 - 36	0.0	0.0 - 0.0	0.0	0.0 - 0.0	0.0	0.0 - 0.0	0.0	0.0 - 0.0	0.0	0.0 - 0.0	14.3	4.5 - 36.3	0.44
3050111	10533	HALFWAY SWAMI	22.2	25.1	10 - 52	0.0	0.0 - 0.0	3.7	1.3 - 8.4	0.5	0.2 - 1.5	11.7	4.1 - 27.9	0.0	0.0 - 0.0	9.2	3.6 - 24.0	0.44
3050201	10534	COOPER RIVER W	41.6	9.6	3 - 24	0.0	0.0 - 0.0	0.1	0.0 - 0.2	0.1	0.0 - 0.1	0.8	0.3 - 2.3	0.0	0.0 - 0.0	8.6	2.8 - 22.4	0.36
3050201	10535	COOPER RIVER W	254.9	5.4	1 - 11	0.1	0.0 - 0.2	0.5	0.1 - 1.0	0.0	0.0 - 0.1	0.8	0.2 - 2.1	0.0	0.0 - 0.0	4.0	1.0 - 8.7	0.94
3050111	10536	JACKS CR	82.2	35.4	12 - 79	0.0	0.0 - 0.0	2.4	0.7 - 6.7	4.3	1.2 - 13.0	19.0	5.6 - 51.5	0.0	0.0 - 0.0	9.7	3.3 - 24.1	0.44
3030005	10540	TOWN CR	349.5	14.8	5 - 31	0.0	0.0 - 0.0	2.0	0.6 - 5.5	2.0	0.6 - 5.8	4.3	1.4 - 10.0	0.0	0.0 - 0.0	6.4	2.0 - 15.3	1.00
3030005	10541	CAPE FEAR R	2.8	6.7	2 - 15	0.0	0.0 - 0.0	5.6	1.5 - 12.4	0.0	0.0 - 0.0	0.0	0.0 - 0.1	0.0	0.0 - 0.0	1.1	0.3 - 2.7	1.00
3040206	10542	WACCAMAW R	104.0	9.0	3 - 23	0.0	0.0 - 0.0	1.1	0.3 - 2.6	1.5	0.4 - 4.4	2.3	0.6 - 7.5	0.0	0.0 - 0.0	4.0	1.1 - 10.1	0.79
3040206	10543	WACCAMAW R	61.8	8.6	2 - 17	0.0	0.0 - 0.0	1.0	0.3 - 2.2	1.2	0.3 - 3.1	1.7	0.5 - 4.0	0.0	0.0 - 0.0	4.7	1.3 - 10.5	0.81
3050104	10544	BEAVER CR	111.4	14.2	5 - 38	0.0	0.0 - 0.0	1.6	0.5 - 4.1	0.7	0.2 - 2.0	1.1	0.4 - 3.3	0.0	0.0 - 0.0	10.9	3.6 - 30.8	0.32
3050104	10545	CATAWBA R	19.8	10.7	3 - 24	0.0	0.0 - 0.0	0.3	0.1 - 0.6	0.2	0.0 - 0.5	0.3	0.1 - 0.7	0.0	0.0 - 0.0	10.0	3.0 - 23.3	0.32
3050104	10546	DUTCHMAN'S CR	31.3	16.6	6 - 42	0.0	0.0 - 0.0	1.3	0.4 - 3.5	0.9	0.2 - 2.2	2.3	0.8 - 5.9	0.0	0.0 - 0.0	12.1	3.9 - 33.3	0.32
3050104	10547	WHITE OAK CR	42.6	12.1	4 - 29	0.0	0.0 - 0.0	2.1	0.8 - 5.1	0.5	0.2 - 1.7	0.9	0.4 - 2.7	0.0	0.0 - 0.0	8.6	3.0 - 20.7	0.32
3050104	10548	FLAT ROCK CR	37.5	7.2	2 - 15	0.0	0.0 - 0.0	2.1	0.7 - 4.5	0.2	0.1 - 0.6	0.4	0.1 - 0.9	0.0	0.0 - 0.0	4.5	1.4 - 10.1	0.32
3050104	10549	SAWNEYS CR	57.2	17.2	6 - 37	0.0	0.0 - 0.0	2.5	0.8 - 4.9	1.3	0.4 - 3.1	2.8	0.8 - 7.0	0.0	0.0 - 0.0	10.6	3.9 - 23.8	0.32
3050104	10550	BIG WATEREE CR	150.4	18.7	6 - 46	0.0	0.0 - 0.0	2.1	0.6 - 5.0	1.3	0.4 - 4.8	4.0	1.4 - 11.3	0.0	0.0 - 0.0	11.3	3.6 - 28.5	0.32
3050104	10551	LITTLE WATEREE	23.4	16.4	5 - 34	0.0	0.0 - 0.0	5.1	1.5 - 9.9	0.2	0.0 - 0.5	0.4	0.1 - 0.9	0.0	0.0 - 0.0	10.7	3.5 - 23.8	0.32
3050104	10553	CATAWBA R	11.3	11.5	4 - 21	0.0	0.0 - 0.0	2.9	0.9 - 5.2	0.8	0.2 - 2.0	1.6	0.5 - 3.5	0.0	0.0 - 0.0	6.3	2.1 - 13.1	0.32
3050104	10555	CATAWBA R	21.5	23.6	9 - 50	0.0	0.0 - 0.0	5.5	2.2 - 12.7	0.7	0.2 - 2.2	1.2	0.5 - 2.6	0.0	0.0 - 0.0	16.2	6.1 - 39.8	0.32
3050104	10556	CATAWBA R	52.7	22.4	7 - 54	0.0	0.0 - 0.0	4.2	1.5 - 9.2	0.6	0.2 - 1.4	1.1	0.4 - 2.9	0.0	0.0 - 0.0	16.6	5.3 - 42.3	0.32
3050104	10557	CATAWBA R	50.5	18.0	6 - 42	0.1	0.0 - 0.2	3.1	1.0 - 6.4	0.3	0.1 - 1.0	0.5	0.2 - 1.3	0.0	0.0 - 0.0	14.1	4.2 - 34.3	0.32
3050104	10558	CATAWBA R	87.1	15.0	4 - 43	0.0	0.0 - 0.0	1.7	0.5 - 4.7	0.4	0.1 - 1.5	0.7	0.2 - 2.3	0.0	0.0 - 0.0	12.2	3.8 - 36.2	0.32
3050104	10559	BIG WATEREE CR	20.8	18.8	5 - 41	0.0	0.0 - 0.0	5.5	1.7 - 11.6	0.5	0.1 - 1.4	0.7	0.2 - 1.7	0.0	0.0 - 0.0	12.2	3.1 - 27.7	0.32
3050104	10560	BEAVER CR	27.3	21.8	8 - 48	0.0	0.0 - 0.0	5.8	2.0 - 12.3	0.6	0.2 - 2.0	1.2	0.4 - 3.7	0.0	0.0 - 0.0	14.2	5.3 - 37.4	0.32
3050104	10561	CATAWBA R	20.7	15.7	5 - 38	0.0	0.0 - 0.0	3.1	1.1 - 7.7	0.6	0.2 - 1.8	1.4	0.4 - 4.0	0.0	0.0 - 0.0	10.6	3.5 - 26.3	0.32
3050104	10562	DUTCHMAN'S CR	8.6	20.9	7 - 47	0.0	0.0 - 0.0	3.7	1.3 - 8.3	0.4	0.1 - 1.3	1.0	0.3 - 2.6	0.0	0.0 - 0.0	15.8	5.6 - 39.6	0.32
3050104	10563	WHITE OAK CR	3.7	29.3	11 - 74	1.5	0.5 - 3.5	5.0	1.7 - 12.3	0.7	0.2 - 2.1	1.9	0.6 - 5.0	0.0	0.0 - 0.0	20.2	7.0 - 52.2	0.32

8-digit HUC	ID	Name	Area	Catchment Yield		Point sources		Developed Land		Manure		Agricultural Land		Phosphate Mines		Soil parent rock		Frac
				kg/km^2	90% CI	kg/km^2	90% CI	kg/km^2	90% CI	kg/km^2	90% CI	kg/km^2	90% CI	kg/km^2	90% CI	kg/km^2	90% CI	
3050104	10566	BIG WATEREE CR	1.1	19.5	7 - 34	0.0	0.0 - 0.0	5.8	2.1 - 12.1	0.8	0.2 - 2.2	1.3	0.5 - 2.4	0.0	0.0 - 0.0	11.6	4.1 - 21.4	0.32
3050103	10568	CATAWBA R	1.3	14.0	5 - 32	0.0	0.0 - 0.0	0.0	0.0 - 0.0	0.7	0.3 - 2.2	0.0	0.0 - 0.0	0.0	0.0 - 0.0	13.2	4.7 - 32.8	0.29
3050103	10569	CATAWBA R	1.5	24.6	8 - 44	0.0	0.0 - 0.0	10.3	3.2 - 20.9	0.6	0.2 - 1.5	2.2	0.7 - 5.1	0.0	0.0 - 0.0	11.5	4.0 - 23.3	0.31
3050103	10570	CATAWBA R	54.7	27.7	9 - 64	0.0	0.0 - 0.0	6.3	1.8 - 17.6	1.8	0.5 - 5.4	4.1	1.2 - 11.0	0.0	0.0 - 0.0	15.5	4.7 - 39.8	0.29
3050101	10571	LINVILLE R	1.1	38.0	12 - 94	0.0	0.0 - 0.0	12.2	3.9 - 28.3	2.8	0.8 - 8.9	9.2	3.3 - 25.0	0.0	0.0 - 0.0	13.8	4.7 - 37.1	0.01
3050101	10572	CATAWBA R, N FK	222.4	12.7	4 - 28	0.0	0.0 - 0.1	4.0	1.2 - 8.1	0.3	0.1 - 1.0	1.0	0.3 - 2.7	0.0	0.0 - 0.0	7.3	2.3 - 18.7	0.01
3050101	10573	CATAWBA R	68.8	31.9	10 - 60	3.8	1.2 - 7.6	12.0	3.7 - 24.6	1.5	0.4 - 3.2	4.7	1.4 - 10.3	0.0	0.0 - 0.0	9.9	3.3 - 18.5	0.01
3050101	10575	CATAWBA R	63.8	16.2	5 - 35	0.0	0.0 - 0.0	4.7	1.6 - 10.8	0.5	0.1 - 1.1	1.1	0.4 - 2.8	0.0	0.0 - 0.0	9.9	3.5 - 21.5	0.01
3050101	10576	LINVILLE R	64.2	18.2	6 - 32	0.0	0.0 - 0.0	4.0	1.3 - 7.3	0.6	0.2 - 1.5	1.6	0.5 - 3.5	0.0	0.0 - 0.0	12.0	3.9 - 25.1	0.01
3050101	10578	CATAWBA R	5.1	44.8	17 - 95	0.0	0.0 - 0.0	25.1	8.3 - 54.6	1.7	0.5 - 4.3	4.8	1.6 - 12.6	0.0	0.0 - 0.0	13.2	4.9 - 32.6	0.01
3050105	10579	S PACOLET R	19.6	71.3	30 - 131	0.0	0.0 - 0.0	22.1	9.5 - 43.4	3.7	1.2 - 11.6	26.3	11.3 - 58.9	0.0	0.0 - 0.0	19.2	6.8 - 41.8	0.08
3050105	10580	S PACOLET R	87.2	13.8	5 - 34	0.0	0.0 - 0.0	4.7	1.5 - 12.3	0.6	0.2 - 1.7	3.9	1.1 - 11.6	0.0	0.0 - 0.0	4.6	1.4 - 12.2	0.29
3050105	10581	BROAD R	176.5	23.7	9 - 42	0.0	0.0 - 0.0	7.3	2.7 - 14.0	0.6	0.2 - 1.5	2.7	1.0 - 6.6	0.0	0.0 - 0.0	13.1	5.2 - 27.6	0.08
3050105	10582	BROAD R	69.3	8.5	3 - 21	0.0	0.0 - 0.0	3.6	1.2 - 8.0	0.1	0.0 - 0.3	0.4	0.1 - 1.2	0.0	0.0 - 0.0	4.4	1.6 - 11.7	0.28
3050101	10583	CATAWBA R	117.8	253.9	96 - 511	198.8	73.4 - 440.6	40.0	14.4 - 72.4	0.5	0.2 - 1.3	2.5	0.8 - 5.5	0.0	0.0 - 0.0	12.1	4.8 - 30.2	0.20
3050101	10584	CATAWBA CR	75.9	59.1	18 - 125	0.0	0.0 - 0.0	40.7	11.8 - 87.9	1.2	0.4 - 3.9	5.2	1.7 - 12.3	0.0	0.0 - 0.0	12.0	3.8 - 29.8	0.20
3050102	10585	CATAWBA R, S FK	72.2	92.3	34 - 198	26.2	9.1 - 58.4	46.7	15.1 - 98.1	0.8	0.2 - 2.6	3.4	1.2 - 10.1	0.0	0.0 - 0.0	15.3	5.4 - 38.3	0.20
3050101	10587	ALLISON CR	122.2	35.2	10 - 87	0.0	0.0 - 0.0	9.4	2.5 - 24.2	2.8	0.8 - 8.8	12.3	3.3 - 35.7	0.0	0.0 - 0.0	10.7	3.7 - 25.8	0.20
3050101	10588	CATAWBA R	13.5	73.1	25 - 152	37.6	13.3 - 77.5	21.4	7.2 - 49.5	0.9	0.3 - 2.9	3.6	1.1 - 8.1	0.0	0.0 - 0.0	9.5	3.0 - 24.8	0.28
3050101	10589	CATAWBA R	35.6	37.4	12 - 90	0.0	0.0 - 0.0	16.9	5.6 - 41.4	1.1	0.3 - 3.1	5.0	1.5 - 14.4	0.0	0.0 - 0.0	14.5	4.7 - 34.1	0.20
3050101	10590	CATAWBA R	57.9	41.4	17 - 103	5.9	2.2 - 15.8	14.1	5.5 - 31.0	1.3	0.4 - 4.5	6.1	2.3 - 15.6	0.0	0.0 - 0.0	13.9	5.2 - 39.7	0.20
3050101	10591	CATAWBA R	2.8	24.5	10 - 56	0.0	0.0 - 0.0	1.4	0.4 - 3.3	1.0	0.3 - 3.3	4.0	1.2 - 11.6	0.0	0.0 - 0.0	18.0	7.3 - 49.2	0.20
3050101	10593	CATAWBA R	18.4	38.5	11 - 80	0.0	0.0 - 0.0	21.0	6.3 - 48.1	0.5	0.2 - 1.3	3.4	1.1 - 10.5	0.0	0.0 - 0.0	13.6	4.1 - 31.9	0.20
3050101	10594	CATAWBA CR	6.5	28.6	10 - 57	0.0	0.0 - 0.0	6.0	2.0 - 13.1	2.1	0.6 - 5.1	8.7	2.9 - 25.5	0.0	0.0 - 0.0	11.8	4.0 - 28.9	0.20
3050101	10596	CROWDERS CR	20.1	38.9	13 - 95	0.0	0.0 - 0.0	16.1	4.8 - 40.0	1.8	0.5 - 5.1	7.4	2.5 - 22.4	0.0	0.0 - 0.0	13.6	4.6 - 34.5	0.20
3050101	10597	ALLISON CR	51.9	38.8	14 - 88	0.0	0.0 - 0.0	12.8	4.3 - 28.1	2.6	0.8 - 8.4	11.0	3.9 - 33.7	0.0	0.0 - 0.0	12.4	4.9 - 29.5	0.20
3050101	10598	CATAWBA R	157.4	42.7	15 - 88	0.0	0.0 - 0.0	7.4	2.4 - 15.8	6.2	1.9 - 17.3	16.9	6.5 - 43.5	0.0	0.0 - 0.0	12.3	5.1 - 30.9	0.06
3050101	10599	CATAWBA R	526.5	16.0	5 - 39	0.5	0.1 - 1.2	6.6	2.1 - 17.8	1.0	0.3 - 3.0	3.1	1.0 - 8.7	0.0	0.0 - 0.0	4.8	1.4 - 12.6	0.17
3040103	10600	YADKIN R	25.2	27.3	10 - 55	0.0	0.0 - 0.0	8.2	3.1 - 17.0	1.6	0.5 - 4.8	5.9	2.2 - 13.2	0.0	0.0 - 0.0	11.7	4.5 - 31.3	0.48
3040103	10601	TOWN CR	116.0	40.9	14 - 95	0.1	0.0 - 0.3	22.6	7.5 - 57.1	1.5	0.5 - 4.3	7.3	2.8 - 17.9	0.0	0.0 - 0.0	9.3	3.4 - 26.6	0.48
3040103	10602	SECOND CR	102.1	36.7	12 - 81	0.0	0.0 - 0.0	8.0	2.6 - 19.0	2.9	0.9 - 10.5	15.3	5.4 - 44.1	0.0	0.0 - 0.0	10.5	3.3 - 27.8	0.48
3040103	10603	SWEARING CR	100.7	48.2	14 - 102	0.0	0.0 - 0.0	25.9	6.9 - 63.4	2.4	0.6 - 5.7	9.4	2.8 - 24.0	0.0	0.0 - 0.0	10.6	3.4 - 21.8	0.48
3040103	10604	ABBOTTS CR	57.2	198.7	65 - 424	159.8	54.2 - 397.8	16.0	4.3 - 37.7	2.8	0.7 - 6.7	10.4	3.4 - 26.3	0.0	0.0 - 0.0	9.7	3.4 - 23.5	0.48
3040103	10605	FOURMILE CR	20.1	13.0	4 - 27	0.0	0.0 - 0.0	3.5	1.4 - 7.8	0.8	0.2 - 2.4	2.3	0.9 - 5.2	0.0	0.0 - 0.0	6.4	2.1 - 17.0	0.66
3040103	10606	YADKIN R	29.8	13.7	5 - 28	0.0	0.0 - 0.0	1.8	0.7 - 3.5	0.9	0.3 - 2.1	4.6	1.7 - 10.8	0.0	0.0 - 0.0	6.5	2.6 - 15.0	0.66
3040103	10607	YADKIN R	5.7	31.5	9 - 77	0.0	0.0 - 0.0	0.2	0.1 - 0.5	1.8	0.5 - 5.8	8.9	2.5 - 24.5	0.0	0.0 - 0.0	20.7	5.8 - 55.6	0.48
3040103	10608	YADKIN R	5.3	42.7	17 - 88	0.0	0.0 - 0.0	7.9	2.9 - 18.2	2.7	0.9 - 9.4	13.8	5.5 - 37.3	0.0	0.0 - 0.0	18.3	7.0 - 46.6	0.48
3040103	10609	YADKIN R	14.9	37.5	14 - 76	0.0	0.0 - 0.0	8.3	2.9 - 16.7	2.9	0.8 - 8.6	10.6	3.5 - 25.6	0.0	0.0 - 0.0	15.8	5.6 - 41.0	0.48
3040103	10610	YADKIN R	3.2	38.1	14 - 86	0.0	0.0 - 0.0	12.7	4.9 - 28.1	1.7	0.5 - 5.3	6.0	2.3 - 14.3	0.0	0.0 - 0.0	17.7	6.3 - 41.9	0.48

8-digit HUC	ID	Name	Area	Catchment Yield		Point sources		Developed Land		Manure		Agricultural Land		Phosphate Mines		Soil parent rock		Frac
				kg/km^2	90% CI	kg/km^2	90% CI	kg/km^2	90% CI	kg/km^2	90% CI	kg/km^2	90% CI	kg/km^2	90% CI	kg/km^2	90% CI	
3040103	10611	YADKIN R	102.5	42.5	15 - 107	1.0	0.3 - 2.4	8.2	2.7 - 19.3	4.1	1.2 - 14.3	16.4	5.5 - 42.5	0.0	0.0 - 0.0	12.8	4.5 - 33.5	0.48
3040103	10612	TOWN CR	83.5	34.1	12 - 74	0.0	0.0 - 0.0	7.5	2.5 - 15.5	2.4	0.7 - 6.8	11.8	4.3 - 29.9	0.0	0.0 - 0.0	12.5	4.4 - 30.2	0.48
3040103	10613	SECOND CR	38.0	31.3	11 - 63	0.0	0.0 - 0.0	4.5	1.5 - 10.1	2.5	0.6 - 6.3	12.6	4.7 - 28.4	0.0	0.0 - 0.0	11.8	3.8 - 29.4	0.48
3040103	10614	SWEARING CR	28.2	34.4	13 - 76	2.0	0.7 - 3.7	7.7	2.7 - 16.9	2.9	1.0 - 7.9	10.9	4.1 - 26.1	0.0	0.0 - 0.0	10.9	3.9 - 26.8	0.48
3040103	10615	ABBOTTS CR	74.8	33.6	12 - 70	0.9	0.3 - 2.0	9.2	3.1 - 21.8	2.4	0.7 - 6.4	7.9	2.8 - 20.1	0.0	0.0 - 0.0	13.2	4.3 - 33.1	0.48
3040103	10616	FOURMILE CR	15.8	15.9	5 - 32	0.0	0.0 - 0.0	1.2	0.4 - 2.5	1.2	0.4 - 3.2	3.5	1.2 - 7.8	0.0	0.0 - 0.0	10.0	3.3 - 24.3	0.66
3010103	10617	SMITH R	3.6	15.9	5 - 37	0.0	0.0 - 0.0	2.1	0.7 - 4.4	0.7	0.2 - 2.0	2.0	0.7 - 4.5	0.0	0.0 - 0.0	11.1	3.6 - 29.5	0.08
3010103	10618	GOBLINTOWN CR	62.3	20.1	6 - 49	0.0	0.0 - 0.0	4.2	1.5 - 10.9	1.1	0.3 - 3.7	3.1	1.2 - 7.1	0.0	0.0 - 0.0	11.7	3.4 - 28.9	0.08
3010103	10619	SMITH R	25.2	5.3	1 - 13	0.0	0.0 - 0.0	0.7	0.2 - 1.7	0.1	0.0 - 0.4	0.5	0.2 - 1.7	0.0	0.0 - 0.0	3.9	1.0 - 10.0	0.28
3010103	10620	GOBLINTOWN CR	8.1	19.8	6 - 48	0.0	0.0 - 0.0	2.7	0.9 - 6.2	0.3	0.1 - 0.8	0.4	0.2 - 1.1	0.0	0.0 - 0.0	16.5	5.2 - 42.8	0.08
3010103	10621	SMITH R	6.0	16.5	5 - 42	0.0	0.0 - 0.0	2.9	1.0 - 8.1	0.4	0.1 - 1.4	1.2	0.4 - 3.4	0.0	0.0 - 0.0	12.0	3.8 - 35.1	0.08
3010101	10622	ROANOKE R	72.5	19.5	6 - 47	0.0	0.0 - 0.0	8.7	2.6 - 21.3	0.7	0.2 - 2.2	2.2	0.7 - 6.5	0.0	0.0 - 0.0	7.9	2.2 - 21.5	0.05
3010101	10623	GILLS CR	86.8	28.8	8 - 45	0.0	0.0 - 0.0	2.6	0.7 - 5.1	4.0	0.9 - 10.8	12.8	3.8 - 26.0	0.0	0.0 - 0.0	9.4	2.4 - 19.8	0.05
3010101	10624	BLACKWATER R	35.7	30.4	12 - 57	0.0	0.0 - 0.0	3.4	1.2 - 6.6	4.6	1.7 - 11.3	14.4	5.2 - 34.0	0.0	0.0 - 0.0	8.0	3.2 - 17.3	0.05
3010101	10625	ROANOKE R	19.7	23.5	7 - 50	0.0	0.0 - 0.0	2.1	0.7 - 4.7	1.3	0.3 - 3.5	5.0	1.5 - 11.8	0.0	0.0 - 0.0	15.1	4.5 - 33.9	0.23
3010101	10626	PIGG R	134.9	26.8	10 - 54	0.0	0.0 - 0.0	4.3	1.5 - 9.4	2.2	0.7 - 5.7	10.4	3.9 - 27.9	0.0	0.0 - 0.0	10.0	3.6 - 24.0	0.23
3010101	10627	ROANOKE R	146.5	16.5	6 - 30	0.0	0.0 - 0.0	2.0	0.6 - 4.0	1.3	0.4 - 2.9	4.8	1.9 - 11.8	0.0	0.0 - 0.0	8.5	2.8 - 18.0	0.32
3010101	10628	ROANOKE R	49.6	6.1	2 - 13	0.0	0.0 - 0.0	0.7	0.2 - 1.3	0.5	0.1 - 1.2	1.6	0.4 - 3.9	0.0	0.0 - 0.0	3.4	1.0 - 8.3	0.23
3010101	10629	BLACKWATER R	64.6	31.2	11 - 64	0.0	0.0 - 0.0	4.8	1.5 - 10.8	3.4	1.0 - 10.8	11.1	3.7 - 27.7	0.0	0.0 - 0.0	11.9	3.8 - 32.2	0.05
3010101	10630	ROANOKE R	317.0	26.9	9 - 49	0.0	0.0 - 0.0	5.8	1.8 - 11.8	2.7	0.8 - 6.8	8.5	2.5 - 20.3	0.0	0.0 - 0.0	9.9	3.3 - 20.6	0.05
3010101	10631	GILLS CR	23.6	29.5	9 - 74	0.0	0.0 - 0.0	3.7	1.3 - 9.1	3.9	1.2 - 11.4	12.1	3.8 - 41.1	0.0	0.0 - 0.0	9.8	3.1 - 28.9	0.05
3010101	10632	BLACKWATER R	85.3	35.7	13 - 69	0.0	0.0 - 0.0	4.1	1.4 - 8.7	5.1	1.5 - 15.8	16.5	6.0 - 36.0	0.0	0.0 - 0.0	9.9	3.0 - 23.6	0.05
3010101	10633	ROANOKE R	2.5	20.0	7 - 54	0.0	0.0 - 0.0	2.1	0.7 - 6.7	1.1	0.3 - 4.0	5.4	2.0 - 15.1	0.0	0.0 - 0.0	11.3	4.0 - 33.6	0.23
3010104	10635	DAN R	12.0	33.4	12 - 63	0.0	0.0 - 0.0	1.5	0.5 - 3.1	2.3	0.7 - 6.0	7.6	2.5 - 17.5	0.0	0.0 - 0.0	22.0	7.2 - 43.7	0.37
3010102	10636	JONATHAN CR	67.1	35.5	12 - 88	0.0	0.0 - 0.0	2.8	0.8 - 6.6	2.4	0.7 - 6.4	14.2	4.8 - 39.0	0.0	0.0 - 0.0	16.1	5.0 - 36.7	0.37
3010102	10637	GRASSY CR	2.9	25.7	7 - 55	0.0	0.0 - 0.0	4.4	1.2 - 10.0	1.1	0.3 - 3.0	6.5	1.6 - 16.9	0.0	0.0 - 0.0	13.6	3.5 - 31.9	0.37
3010102	10638	LITTLE ISLAND CR	13.9	19.5	6 - 44	0.0	0.0 - 0.0	3.8	1.1 - 8.4	0.6	0.2 - 1.6	9.1	2.7 - 24.3	0.0	0.0 - 0.0	6.0	2.1 - 15.2	0.37
3010102	10639	BLUESTONE CR	135.8	37.3	14 - 93	0.0	0.0 - 0.0	3.3	1.1 - 8.2	4.2	1.5 - 15.0	15.7	5.8 - 41.5	0.0	0.0 - 0.0	14.1	5.2 - 38.6	0.37
3010102	10640	LITTLE BLUESTON	92.6	46.9	12 - 82	5.4	1.4 - 10.3	4.4	1.1 - 8.2	4.4	0.9 - 11.1	18.0	4.8 - 39.3	0.0	0.0 - 0.0	14.7	3.7 - 31.8	0.37
3010102	10641	BUTCHER CR	60.6	36.2	12 - 78	0.0	0.0 - 0.0	4.4	1.4 - 10.7	3.1	0.8 - 10.4	12.1	4.1 - 30.8	0.0	0.0 - 0.0	16.6	5.5 - 43.2	0.37
3010102	10642	CROOKED RUN CR	38.6	17.2	6 - 44	0.0	0.0 - 0.0	4.4	1.5 - 10.2	0.6	0.2 - 1.9	6.3	2.2 - 16.5	0.0	0.0 - 0.0	6.0	2.0 - 16.3	0.37
3010102	10643	ROANOKE R	19.4	11.7	4 - 31	0.0	0.0 - 0.0	0.8	0.3 - 2.2	1.1	0.3 - 3.8	4.3	1.3 - 11.6	0.0	0.0 - 0.0	5.5	1.8 - 14.8	0.69
3010102	10644	ROANOKE R	94.2	25.0	8 - 54	0.0	0.0 - 0.0	1.3	0.4 - 3.3	2.0	0.5 - 6.1	8.8	2.9 - 23.5	0.0	0.0 - 0.0	12.8	4.1 - 35.0	0.37
3010102	10645	ROANOKE R	27.1	29.6	9 - 60	0.0	0.0 - 0.0	1.7	0.5 - 3.3	2.1	0.7 - 5.9	7.9	2.6 - 19.5	0.0	0.0 - 0.0	17.8	5.5 - 42.6	0.37
3010102	10646	ROANOKE R	6.5	35.3	11 - 72	0.0	0.0 - 0.0	0.6	0.2 - 1.1	1.5	0.4 - 4.1	5.1	1.5 - 10.5	0.0	0.0 - 0.0	28.2	8.6 - 61.0	0.37
3010102	10647	ROANOKE R	69.9	34.1	13 - 71	2.3	0.8 - 5.2	6.5	2.2 - 15.5	2.2	0.6 - 6.6	7.1	2.7 - 21.0	0.0	0.0 - 0.0	16.0	5.7 - 39.6	0.37
3010102	10648	BLUESTONE CR	39.2	48.0	14 - 112	0.0	0.0 - 0.0	3.4	1.1 - 7.8	5.0	1.3 - 15.6	19.7	5.7 - 52.0	0.0	0.0 - 0.0	19.9	5.9 - 50.1	0.37
3010102	10649	ROANOKE R	80.0	40.8	14 - 72	0.0	0.0 - 0.0	3.8	1.1 - 7.0	4.4	1.4 - 12.3	17.1	5.5 - 32.2	0.0	0.0 - 0.0	15.6	5.2 - 36.2	0.37
3010102	10650	ROANOKE R	44.9	36.1	10 - 92	0.0	0.0 - 0.0	3.0	0.9 - 6.2	3.4	0.9 - 11.8	10.0	2.9 - 23.4	0.0	0.0 - 0.0	19.7	6.9 - 55.7	0.37

8-digit HUC	ID	Name	Area	Catchment Yield		Point sources		Developed Land		Manure		Agricultural Land		Phosphate Mines		Soil parent rock		Frac
				kg/km^2	90% CI	kg/km^2	90% CI	kg/km^2	90% CI	kg/km^2	90% CI	kg/km^2	90% CI	kg/km^2	90% CI	kg/km^2	90% CI	
3010102	10651	GRASSY CR	145.8	37.0	13 - 77	0.0	0.0 - 0.0	3.4	1.0 - 6.7	3.0	0.9 - 8.9	12.8	3.8 - 24.2	0.0	0.0 - 0.0	17.8	6.3 - 44.1	0.37
3010104	10652	DAN R	1.1	21.4	6 - 45	0.0	0.0 - 0.0	0.0	0.0 - 0.0	0.6	0.2 - 1.4	1.6	0.4 - 3.9	0.0	0.0 - 0.0	19.3	5.4 - 43.5	0.37
3010102	10653	JONATHAN CR	28.9	48.6	17 - 101	0.0	0.0 - 0.0	2.3	0.8 - 4.6	5.1	1.4 - 16.3	21.0	7.5 - 48.5	0.0	0.0 - 0.0	20.3	6.3 - 49.6	0.37
3010102	10654	GRASSY CR	12.3	38.1	14 - 84	0.0	0.0 - 0.0	3.1	1.1 - 7.4	2.1	0.6 - 6.3	14.7	5.1 - 37.7	0.0	0.0 - 0.0	18.1	6.1 - 47.1	0.37
3010102	10655	LITTLE ISLAND CF	14.4	29.3	11 - 62	0.0	0.0 - 0.0	3.0	1.1 - 6.3	1.8	0.5 - 5.2	6.9	2.5 - 16.3	0.0	0.0 - 0.0	17.6	6.6 - 43.7	0.37
3010102	10656	BLUESTONE CR	8.1	32.1	11 - 63	0.0	0.0 - 0.0	4.2	1.4 - 7.4	2.8	0.9 - 8.9	10.3	3.5 - 24.7	0.0	0.0 - 0.0	14.8	4.9 - 37.8	0.37
3010102	10657	LITTLE BLUESTON	2.5	48.7	15 - 120	0.0	0.0 - 0.0	2.8	0.8 - 6.2	4.6	1.1 - 14.2	16.8	5.1 - 44.4	0.0	0.0 - 0.0	24.6	7.6 - 60.2	0.37
3010102	10658	BUTCHER CR	65.8	38.6	14 - 74	0.0	0.0 - 0.0	2.5	0.9 - 5.1	3.4	1.0 - 9.3	13.7	5.2 - 29.9	0.0	0.0 - 0.0	18.9	7.1 - 44.5	0.37
3010102	10659	CROOKED RUN CF	258.5	30.7	10 - 59	6.1	1.9 - 15.8	8.8	2.9 - 20.1	0.7	0.2 - 2.1	6.8	2.2 - 15.3	0.0	0.0 - 0.0	8.3	2.7 - 22.1	0.37
3010106	10660	ROANOKE R	6.6	20.7	8 - 48	0.0	0.0 - 0.0	1.0	0.4 - 2.1	2.2	0.7 - 7.1	8.0	2.9 - 21.8	0.0	0.0 - 0.0	9.5	3.5 - 26.1	0.70
3010106	10661	BLUE MUD CR	15.0	23.3	8 - 54	0.0	0.0 - 0.0	6.0	2.0 - 15.8	2.2	0.6 - 7.5	6.7	2.5 - 17.9	0.0	0.0 - 0.0	8.5	2.8 - 25.0	0.70
3010106	10662	ROANOKE R	264.5	216.0	67 - 448	162.0	53.2 - 359.5	0.4	0.1 - 0.7	10.8	2.8 - 31.7	28.5	8.9 - 65.4	0.0	0.0 - 0.0	14.4	4.5 - 36.0	0.98
3010106	10663	ROANOKE R	8.5	23.7	7 - 57	0.0	0.0 - 0.0	9.3	2.6 - 20.9	1.0	0.3 - 2.8	2.8	0.9 - 6.7	0.0	0.0 - 0.0	10.6	3.3 - 27.9	0.70
3010106	10664	BLUE MUD CR	23.8	23.3	8 - 54	0.0	0.0 - 0.0	4.2	1.2 - 10.8	3.1	0.8 - 9.2	7.1	2.4 - 20.9	0.0	0.0 - 0.0	9.0	3.1 - 23.6	0.70
3010106	10665	ROANOKE R	807.2	16.8	5 - 33	0.0	0.0 - 0.0	4.2	1.4 - 8.5	1.5	0.5 - 3.5	4.4	1.4 - 10.4	0.0	0.0 - 0.0	6.8	2.1 - 15.8	0.95
3010205	10666	NORTH R	123.8	22.9	7 - 57	0.0	0.0 - 0.0	0.1	0.0 - 0.2	0.1	0.0 - 0.2	16.7	4.7 - 47.2	0.0	0.0 - 0.0	6.0	1.7 - 16.9	1.00
3010205	10667	PASQUOTUNK R	160.7	26.1	8 - 54	0.1	0.0 - 0.2	0.7	0.2 - 1.3	0.1	0.0 - 0.2	16.4	4.6 - 40.6	0.0	0.0 - 0.0	8.9	2.9 - 21.6	1.00
3010205	10670	PERQUIMANS R	198.2	24.8	7 - 52	0.0	0.0 - 0.0	0.2	0.0 - 0.4	2.1	0.6 - 5.7	16.0	4.7 - 36.8	0.0	0.0 - 0.0	6.6	2.1 - 16.2	1.00
3010205	10671	PERQUIMANS R	1.1	97.4	27 - 274	0.0	0.0 - 0.0	8.0	2.3 - 19.0	8.1	2.2 - 24.5	53.7	15.1 - 185.7	0.0	0.0 - 0.0	27.6	7.2 - 80.7	1.00
3010205	10672	SCUPPERNONG R	469.6	3.5	1 - 10	0.2	0.1 - 0.4	0.2	0.0 - 0.6	0.1	0.0 - 0.5	1.9	0.4 - 6.5	0.0	0.0 - 0.0	1.1	0.3 - 3.3	1.00
3010205	10674	ALLIGATOR R	2.0	0.3	0 - 1	0.0	0.0 - 0.0	0.0	0.0 - 0.0	0.0	0.0 - 0.0	0.0	0.0 - 0.0	0.0	0.0 - 0.0	0.3	0.0 - 1.2	1.00
3020103	10676	TAR R	2.2	36.0	13 - 63	0.0	0.0 - 0.0	14.7	4.4 - 25.7	1.3	0.4 - 3.4	6.7	2.1 - 15.4	0.0	0.0 - 0.0	13.3	4.9 - 29.7	1.00
3020104	10677	DURHAM CR	143.5	26.9	8 - 72	0.0	0.0 - 0.0	1.2	0.4 - 2.5	0.3	0.1 - 0.7	2.0	0.6 - 5.3	15.5	4.1 - 52.4	7.8	2.4 - 20.8	1.00
3020104	10678	SOUTH CR	204.7	16.3	4 - 48	0.7	0.3 - 1.6	0.4	0.1 - 1.2	0.4	0.1 - 1.5	2.7	0.7 - 7.1	9.0	2.0 - 38.3	3.1	0.9 - 8.4	1.00
3020104	10679	INTRACOASTAL W	30.2	1.4	0 - 4	0.0	0.0 - 0.0	0.1	0.0 - 0.4	0.0	0.0 - 0.0	0.1	0.0 - 0.2	0.0	0.0 - 0.0	1.2	0.3 - 3.6	1.00
3020104	10680	PANTEGO CR	10.5	6.5	2 - 17	0.0	0.0 - 0.0	1.4	0.4 - 3.8	0.3	0.1 - 1.2	1.8	0.4 - 6.0	0.0	0.0 - 0.0	3.0	0.9 - 8.6	1.00
3020104	10681	PUNGO R	605.7	0.6	0 - 2	0.2	0.1 - 0.5	0.0	0.0 - 0.1	0.0	0.0 - 0.1	0.2	0.0 - 1.4	0.0	0.0 - 0.0	0.1	0.0 - 0.7	1.00
3020204	10693	UPPER BROAD CR	150.4	14.7	4 - 41	0.0	0.0 - 0.0	1.5	0.4 - 3.6	1.1	0.3 - 2.8	4.6	1.4 - 13.0	0.0	0.0 - 0.0	7.6	1.9 - 19.4	1.00
3020204	10694	TRENT R	14.2	61.5	22 - 153	1.3	0.6 - 3.8	43.5	16.0 - 114.7	1.2	0.3 - 3.6	3.4	1.4 - 10.5	0.0	0.0 - 0.0	12.1	4.0 - 33.1	1.00
3030001	10700	SOUTHWEST CR	224.8	26.3	7 - 55	0.8	0.2 - 1.6	6.1	1.7 - 13.1	6.0	1.4 - 13.4	6.3	1.7 - 16.5	0.0	0.0 - 0.0	7.1	2.2 - 15.8	1.00
3030001	10701	NEW R	112.7	47.5	17 - 81	4.0	1.4 - 7.7	18.2	6.3 - 37.8	8.7	2.6 - 20.5	9.2	3.6 - 20.5	0.0	0.0 - 0.0	7.5	2.8 - 15.0	1.00
3020106	10705	HUNTERS CR	142.6	2.0	0 - 7	0.0	0.0 - 0.0	0.2	0.0 - 0.6	0.1	0.0 - 0.5	0.1	0.0 - 0.3	0.0	0.0 - 0.0	1.6	0.4 - 5.8	0.93
3020106	10706	WHITE OAK R	16.5	59.7	19 - 123	7.0	1.9 - 14.6	3.6	1.1 - 8.2	19.5	5.6 - 49.9	24.2	7.0 - 57.9	0.0	0.0 - 0.0	5.3	1.7 - 12.4	1.00
3020106	10707	HUNTERS CR	44.0	1.1	0 - 3	0.0	0.0 - 0.0	0.0	0.0 - 0.0	0.0	0.0 - 0.0	0.0	0.0 - 0.0	0.0	0.0 - 0.0	1.1	0.3 - 3.3	0.96
3020106	10709	NEWPORT R	408.9	11.8	4 - 24	2.4	0.8 - 5.5	4.5	1.4 - 10.9	0.3	0.1 - 0.9	1.8	0.6 - 5.6	0.0	0.0 - 0.0	2.7	0.9 - 6.2	1.00
3040103	10711	YADKIN R	339.0	31.5	11 - 68	0.8	0.3 - 2.0	4.8	1.7 - 9.8	3.0	1.0 - 8.6	10.6	4.1 - 27.9	0.0	0.0 - 0.0	12.3	4.2 - 33.7	0.70
3040103	10712	YADKIN R	132.3	28.0	10 - 58	0.0	0.0 - 0.0	6.7	2.4 - 12.7	2.9	0.9 - 8.0	4.2	1.4 - 10.4	0.0	0.0 - 0.0	14.3	4.4 - 37.9	0.70
3040104	10713	PEE DEE R	13.3	13.3	5 - 29	0.0	0.0 - 0.0	6.7	2.3 - 16.4	0.3	0.1 - 0.9	0.2	0.1 - 0.5	0.0	0.0 - 0.0	6.1	2.3 - 14.6	0.71
3040104	10714	PEE DEE R	349.3	37.8	14 - 84	2.5	1.0 - 6.0	8.0	2.9 - 18.2	5.9	1.9 - 16.4	8.4	2.9 - 24.5	0.0	0.0 - 0.0	13.0	5.2 - 35.0	0.79

8-digit HUC	ID	Name	Area	Catchment Yield		Point sources		Developed Land		Manure		Agricultural Land		Phosphate Mines		Soil parent rock		Frac
				kg/km^2	90% CI	kg/km^2	90% CI	kg/km^2	90% CI	kg/km^2	90% CI	kg/km^2	90% CI	kg/km^2	90% CI	kg/km^2	90% CI	
3010103	10715	W BELEWS CR	21.8	29.7	12 - 69	0.0	0.0 - 0.0	7.2	2.4 - 15.4	1.4	0.5 - 4.2	10.6	4.0 - 25.3	0.0	0.0 - 0.0	10.4	3.9 - 28.1	0.04
3010103	10716	BELEWS CR	48.6	42.1	16 - 115	6.5	2.3 - 18.6	15.7	5.6 - 36.2	1.3	0.4 - 3.6	9.5	3.5 - 27.4	0.0	0.0 - 0.0	9.2	3.0 - 25.7	0.04
3010103	10717	E BELEWS CR	23.9	35.5	12 - 75	0.9	0.3 - 2.4	15.9	5.2 - 36.5	1.3	0.4 - 3.2	9.1	3.0 - 21.9	0.0	0.0 - 0.0	8.3	3.0 - 20.2	0.04
3010103	10718	BELEWS CR	9.7	3.3	1 - 9	0.0	0.0 - 0.0	0.4	0.1 - 0.9	0.1	0.0 - 0.3	0.5	0.2 - 1.5	0.0	0.0 - 0.0	2.3	0.6 - 6.3	0.31
3010103	10719	BELEWS CR	28.4	25.3	9 - 48	0.0	0.0 - 0.0	3.1	1.1 - 7.0	1.2	0.3 - 3.4	8.4	3.0 - 21.4	0.0	0.0 - 0.0	12.6	4.0 - 27.6	0.04
3010103	10720	W BELEWS CR	17.4	20.3	7 - 47	0.0	0.0 - 0.0	5.4	1.9 - 12.4	0.7	0.2 - 2.1	3.5	1.1 - 8.8	0.0	0.0 - 0.0	10.8	4.3 - 27.2	0.04
3010103	10721	BELEWS CR	5.8	32.1	10 - 71	0.0	0.0 - 0.0	5.1	1.7 - 11.8	1.6	0.5 - 5.0	13.6	4.2 - 31.7	0.0	0.0 - 0.0	11.8	3.5 - 30.5	0.04
3010103	10722	E BELEWS CR	25.9	29.6	11 - 56	0.0	0.0 - 0.0	8.2	2.7 - 16.9	1.5	0.5 - 3.9	9.2	3.2 - 20.7	0.0	0.0 - 0.0	10.6	4.1 - 24.3	0.04
3030002	10723	HAW R, REEDY FK	4.8	11.9	4 - 20	0.0	0.0 - 0.0	2.4	0.7 - 4.9	0.7	0.2 - 1.6	4.3	1.3 - 10.7	0.0	0.0 - 0.0	4.4	1.3 - 10.5	0.08
3030002	10724	HAW R, REEDY FK	89.6	11.5	4 - 24	0.0	0.0 - 0.0	6.1	1.8 - 12.9	0.4	0.1 - 1.1	2.1	0.7 - 5.7	0.0	0.0 - 0.0	3.0	1.1 - 7.7	0.31
3010104	10725	HYCO R, REEDY FI	41.9	23.4	7 - 41	0.0	0.0 - 0.0	3.3	0.9 - 5.6	1.4	0.4 - 4.1	7.3	2.1 - 16.2	0.0	0.0 - 0.0	11.4	3.3 - 25.4	0.10
3010104	10726	HYCO R	147.1	23.9	9 - 50	0.0	0.0 - 0.0	3.3	1.1 - 7.3	1.8	0.6 - 4.4	9.1	3.1 - 23.2	0.0	0.0 - 0.0	9.7	3.6 - 22.0	0.10
3010104	10727	S HYCO CR	207.4	26.4	9 - 54	0.0	0.0 - 0.1	3.3	1.1 - 7.2	2.0	0.6 - 5.3	11.3	4.1 - 30.3	0.0	0.0 - 0.0	9.8	3.2 - 22.5	0.10
3010104	10728	HYCO R	91.3	7.9	2 - 18	0.0	0.0 - 0.0	1.2	0.3 - 2.8	0.5	0.1 - 1.4	2.5	0.7 - 6.3	0.0	0.0 - 0.0	3.7	1.1 - 9.4	0.33
3010104	10729	HYCO R	51.7	20.1	9 - 40	0.0	0.0 - 0.0	3.2	1.2 - 6.4	1.0	0.4 - 2.7	4.9	2.1 - 10.1	0.0	0.0 - 0.0	11.0	4.5 - 23.0	0.10
3010104	10731	HYCO R	1.4	12.3	4 - 24	0.0	0.0 - 0.0	0.0	0.0 - 0.0	0.9	0.2 - 2.6	1.3	0.4 - 3.1	0.0	0.0 - 0.0	10.1	2.7 - 21.6	0.10
3010104	10732	S HYCO CR	2.7	18.3	6 - 36	0.0	0.0 - 0.0	3.3	1.0 - 6.2	0.6	0.2 - 1.5	2.7	0.9 - 6.0	0.0	0.0 - 0.0	11.8	4.0 - 25.2	0.10
3100208	10733	WITHLACOOCHEE	59.6	27.1	10 - 60	0.0	0.0 - 0.0	13.6	4.5 - 29.6	1.1	0.4 - 3.6	1.9	0.6 - 5.3	0.0	0.0 - 0.0	10.5	3.3 - 27.1	1.00
3100208	10734	WITHLACOOCHEE	1.7	39.2	14 - 78	0.0	0.0 - 0.0	18.0	5.8 - 40.3	0.2	0.0 - 0.4	0.6	0.2 - 1.7	0.0	0.0 - 0.0	20.4	7.0 - 45.9	0.97
3100208	10735	WITHLACOOCHEE	247.7	31.9	10 - 65	0.0	0.0 - 0.0	16.3	4.5 - 33.4	1.3	0.4 - 3.6	5.1	1.5 - 13.5	0.0	0.0 - 0.1	9.3	2.9 - 21.5	0.46
3100208	10736	WITHLACOOCHEE	110.4	25.8	7 - 47	0.0	0.0 - 0.0	12.1	3.2 - 25.0	1.0	0.3 - 2.4	2.8	0.7 - 7.0	0.0	0.0 - 0.0	9.8	2.8 - 24.4	0.97
3100202	10737	MANATEE R	1.8	0.5	0 - 2	0.0	0.0 - 0.0	0.0	0.0 - 0.1	0.1	0.0 - 0.4	0.2	0.0 - 1.2	0.0	0.0 - 0.0	0.2	0.0 - 1.1	1.00
3100202	10738	BRADEN R	253.8	111.6	44 - 260	0.0	0.0 - 0.0	38.2	15.3 - 91.9	8.2	2.9 - 25.4	22.5	9.1 - 58.1	0.0	0.0 - 0.0	42.8	14.8 - 125.5	1.00
3100102	10742	MYAKKA R	1.4	86.8	33 - 216	0.0	0.0 - 0.0	16.8	6.6 - 36.6	2.2	0.7 - 6.7	0.0	0.0 - 0.0	0.0	0.0 - 0.0	67.8	24.9 - 173.5	1.00
3100102	10743	*A	171.1	77.4	22 - 186	0.0	0.0 - 0.1	52.3	14.1 - 130.6	0.7	0.2 - 2.0	1.0	0.4 - 2.8	0.0	0.0 - 0.0	23.4	7.8 - 68.1	1.00
3100101	10748	PEACE R	4.0	0.2	0 - 1	0.0	0.0 - 0.0	0.0	0.0 - 0.1	0.0	0.0 - 0.1	0.0	0.0 - 0.2	0.0	0.0 - 0.0	0.1	0.0 - 0.6	1.00
3100101	10749	SHELL CR	27.1	81.2	24 - 193	0.0	0.0 - 0.0	19.5	6.2 - 47.1	7.9	1.9 - 23.4	30.3	8.3 - 85.3	0.0	0.0 - 0.0	23.5	7.2 - 63.5	1.00
3080101	10765	ST JOHNS R	51.9	14.8	4 - 37	0.0	0.0 - 0.0	1.3	0.4 - 3.8	1.5	0.4 - 4.5	6.6	1.8 - 19.9	0.0	0.0 - 0.0	5.3	1.5 - 13.7	0.84
3080101	10766	ST JOHNS R	139.3	2.7	0 - 11	0.0	0.0 - 0.0	0.1	0.0 - 0.4	0.2	0.0 - 1.2	1.0	0.1 - 5.5	0.0	0.0 - 0.0	1.4	0.2 - 6.0	0.86
3080101	10767	ST JOHNS R	25.5	1.4	0 - 6	0.0	0.0 - 0.0	0.0	0.0 - 0.0	0.1	0.0 - 0.7	0.5	0.0 - 2.7	0.0	0.0 - 0.0	0.8	0.1 - 3.1	0.87
3080101	10768	ST JOHNS R	235.3	55.6	15 - 110	0.3	0.1 - 0.7	7.1	2.1 - 14.3	5.0	1.2 - 13.4	20.7	5.8 - 55.6	0.0	0.0 - 0.0	22.4	6.6 - 54.4	0.84
3080101	10769	ST JOHNS R	12.1	9.9	3 - 30	0.0	0.0 - 0.0	0.0	0.0 - 0.1	0.2	0.0 - 1.0	0.7	0.2 - 2.3	0.0	0.0 - 0.0	9.0	2.5 - 28.1	0.86
3080101	10770	ST JOHNS R	63.7	4.5	1 - 13	0.0	0.0 - 0.0	0.5	0.1 - 1.7	0.0	0.0 - 0.1	0.1	0.0 - 0.3	0.0	0.0 - 0.0	3.9	1.0 - 11.6	0.87
3080101	10795	ST JOHNS R	308.9	28.7	10 - 72	0.0	0.0 - 0.0	5.2	1.5 - 13.0	0.3	0.1 - 0.8	1.0	0.3 - 2.7	0.0	0.0 - 0.0	22.3	7.8 - 60.5	0.90
3080101	10796	ECOHLOCKHATCI	162.4	56.6	19 - 108	0.0	0.0 - 0.0	12.4	4.2 - 23.2	4.0	1.1 - 12.8	12.6	4.0 - 31.6	0.0	0.0 - 0.0	27.6	8.8 - 66.2	0.91
3080101	10797	ST JOHNS R	57.3	93.1	26 - 276	0.0	0.0 - 0.0	0.1	0.0 - 0.3	4.7	1.0 - 17.6	13.8	4.2 - 44.5	0.0	0.0 - 0.0	74.5	19.7 - 240.3	0.91
3080101	10798	ST JOHNS R	89.4	49.9	15 - 138	0.0	0.0 - 0.0	4.1	1.1 - 9.7	1.1	0.3 - 3.4	4.5	1.5 - 13.3	0.0	0.0 - 0.0	40.2	12.5 - 117.2	0.92
3010203	10804	CHOWAN R	1.0	15.3	6 - 39	0.0	0.0 - 0.0	0.0	0.0 - 0.0	0.0	0.0 - 0.0	0.0	0.0 - 0.0	0.0	0.0 - 0.0	15.3	5.6 - 38.7	1.00
3010203	10805	SALMON CR	82.7	64.9	24 - 170	0.0	0.0 - 0.0	1.9	0.7 - 4.5	12.4	4.0 - 36.7	32.2	11.9 - 86.9	0.0	0.0 - 0.0	18.4	6.0 - 49.3	1.00

8-digit HUC	ID	Name	Area	Catchment Yield		Point sources		Developed Land		Manure		Agricultural Land		Phosphate Mines		Soil parent rock		Frac
				kg/km^2	90% CI	kg/km^2	90% CI	kg/km^2	90% CI	kg/km^2	90% CI	kg/km^2	90% CI	kg/km^2	90% CI	kg/km^2	90% CI	
6010101	16715	HOLSTON R, N FK	29.4	59.4	21 - 136	0.0	0.0 - 0.0	23.8	9.1 - 58.9	6.7	2.0 - 19.4	18.2	5.8 - 44.7	0.0	0.0 - 0.0	10.7	3.8 - 31.8	0.12
6010101	16716	HOLSTON R, N FK	20.6	39.9	14 - 70	1.5	0.6 - 3.2	18.7	7.5 - 35.2	3.1	0.9 - 8.5	9.0	3.5 - 20.4	0.0	0.0 - 0.0	7.7	2.5 - 16.9	0.12
6010101	16717	HOLSTON R, N FK	216.6	18.7	6 - 41	0.0	0.0 - 0.0	4.0	1.3 - 7.8	2.4	0.8 - 6.7	6.5	2.2 - 14.8	0.0	0.0 - 0.0	5.9	2.0 - 16.7	0.12
6010101	16718	ABRAMS CR	59.7	23.2	8 - 42	0.0	0.0 - 0.0	3.9	1.3 - 7.7	3.9	1.0 - 8.7	8.6	2.8 - 19.5	0.0	0.0 - 0.0	6.7	2.3 - 14.5	0.12
6010101	16719	HOLSTON R, N FK	177.6	17.9	6 - 41	0.0	0.0 - 0.0	2.1	0.7 - 4.9	2.8	0.7 - 9.9	6.5	2.1 - 17.1	0.0	0.0 - 0.0	6.4	2.1 - 15.7	0.12
6010101	16720	GARRETT CR	14.7	30.5	10 - 62	0.0	0.0 - 0.0	5.7	1.6 - 14.8	5.2	1.4 - 12.5	11.9	3.5 - 28.1	0.0	0.0 - 0.0	7.7	2.4 - 17.4	0.11
6010101	16721	HOLSTON R, N FK	26.3	20.3	6 - 55	0.0	0.0 - 0.0	1.9	0.6 - 4.8	3.3	0.9 - 9.7	7.8	2.4 - 20.5	0.0	0.0 - 0.0	7.3	2.2 - 21.5	0.11
6010101	16722	HOLSTON R, N FK	196.6	23.8	9 - 49	0.0	0.0 - 0.0	1.9	0.7 - 4.5	4.1	1.3 - 11.7	9.6	3.5 - 24.6	0.0	0.0 - 0.0	8.1	2.9 - 19.1	0.11
6010101	16723	HOLSTON R, N FK	3.2	13.9	6 - 35	0.0	0.0 - 0.0	2.1	0.8 - 5.1	1.6	0.6 - 5.1	3.9	1.4 - 11.7	0.0	0.0 - 0.0	6.3	2.6 - 15.8	0.11
6010101	16724	STONEMILL CR	26.7	26.2	8 - 68	0.0	0.0 - 0.0	2.1	0.6 - 5.3	4.6	1.4 - 14.4	10.6	3.0 - 29.5	0.0	0.0 - 0.0	8.8	2.8 - 24.3	0.11
6010101	16725	HOLSTON R, N FK	3.7	10.3	4 - 23	0.0	0.0 - 0.0	2.3	0.8 - 5.0	0.7	0.2 - 1.7	1.2	0.4 - 3.2	0.0	0.0 - 0.0	6.2	2.1 - 15.5	0.11
6010101	16726	HOLSTON R, N FK	148.3	25.1	8 - 60	0.0	0.0 - 0.0	2.5	0.8 - 6.5	4.3	1.4 - 13.0	11.4	4.0 - 32.3	0.0	0.0 - 0.0	6.9	2.2 - 17.7	0.10
6010101	16727	HOLSTON R, N FK	112.5	28.8	10 - 56	0.0	0.0 - 0.0	2.9	1.1 - 6.1	4.8	1.6 - 13.3	14.0	4.5 - 34.7	0.0	0.0 - 0.0	7.1	2.7 - 15.4	0.10
6010101	16728	LICK CR	82.4	6.6	2 - 14	0.0	0.0 - 0.0	1.5	0.5 - 3.4	0.4	0.1 - 1.2	1.1	0.4 - 3.0	0.0	0.0 - 0.0	3.6	1.2 - 8.4	0.10
6010101	16729	LAUREL CR	159.9	9.0	3 - 16	0.0	0.0 - 0.0	1.7	0.6 - 3.2	0.9	0.3 - 2.1	2.4	0.7 - 5.8	0.0	0.0 - 0.0	4.0	1.4 - 7.8	0.10
6010101	16730	TUMBLING CR	72.2	8.6	4 - 21	0.0	0.0 - 0.0	1.0	0.3 - 2.2	0.4	0.1 - 1.1	0.9	0.3 - 2.5	0.0	0.0 - 0.0	6.3	2.8 - 15.1	0.11
6010101	16731	BRUMLEY CR	55.6	7.3	2 - 14	0.0	0.0 - 0.0	0.5	0.2 - 1.1	0.7	0.2 - 1.9	1.5	0.5 - 4.2	0.0	0.0 - 0.0	4.5	1.3 - 9.6	0.11
6010101	16732	BIG MOCCASIN CF	246.4	31.8	9 - 60	1.2	0.4 - 2.6	5.7	1.9 - 11.7	4.7	1.5 - 11.6	13.0	3.7 - 30.0	0.0	0.0 - 0.0	7.3	2.4 - 15.3	0.12
6010101	16733	POSSUM CR	96.4	25.2	8 - 47	0.0	0.0 - 0.0	4.0	1.1 - 7.7	3.9	0.9 - 7.9	10.8	3.0 - 24.9	0.0	0.0 - 0.0	6.5	2.0 - 14.0	0.12
6010102	16734	HOLSTON R, S FK	7.2	83.2	27 - 190	0.0	0.0 - 0.0	66.7	21.5 - 158.7	1.5	0.4 - 4.7	3.9	1.3 - 9.5	0.0	0.0 - 0.0	11.1	3.7 - 27.9	0.12
6010102	16735	HOLSTON R, S FK	12.0	826.2	263 - 1748	750.7	237.8 - 1622.5	62.0	18.8 - 135.3	0.7	0.2 - 1.9	2.0	0.7 - 4.6	0.0	0.0 - 0.0	10.9	3.7 - 24.5	0.12
6010102	16736	HORSE CR	122.5	39.5	15 - 87	0.0	0.0 - 0.0	8.3	3.1 - 18.4	5.9	2.1 - 18.1	15.9	5.6 - 42.7	0.0	0.0 - 0.0	9.4	3.5 - 22.8	0.12
6010102	16737	HOLSTON R, S FK	94.7	48.9	18 - 111	0.0	0.0 - 0.0	16.6	5.6 - 37.7	6.8	2.2 - 19.7	16.9	6.5 - 40.0	0.0	0.0 - 0.0	8.6	2.9 - 20.7	0.12
6010102	16738	HOLSTON R, S FK	111.7	75.7	21 - 120	18.7	6.0 - 32.1	16.0	4.4 - 30.1	9.2	2.5 - 23.2	21.7	6.0 - 48.7	0.0	0.0 - 0.0	10.0	3.1 - 20.1	0.09
6010102	16739	HOLSTON R, S FK	202.9	38.3	13 - 85	0.1	0.0 - 0.2	6.6	2.4 - 13.0	5.6	1.5 - 15.7	15.8	6.1 - 38.9	0.0	0.0 - 0.0	10.3	3.2 - 27.1	0.07
6010102	16740	HOLSTON R, S FK	40.0	47.5	18 - 125	0.0	0.0 - 0.0	6.7	2.5 - 16.1	7.5	2.4 - 24.3	21.5	7.7 - 60.4	0.0	0.0 - 0.0	11.8	4.3 - 34.1	0.07
6010102	16741	HOLSTON R, S FK	34.7	31.5	10 - 76	0.0	0.0 - 0.0	3.2	1.0 - 7.3	6.1	1.7 - 20.1	13.7	4.0 - 35.8	0.0	0.0 - 0.0	8.5	2.8 - 20.6	0.03
6010102	16742	WHITETOP LAURE	17.2	26.8	9 - 47	0.0	0.0 - 0.0	6.6	2.1 - 14.7	3.5	1.0 - 8.4	8.0	2.8 - 17.1	0.0	0.0 - 0.0	8.8	2.8 - 17.5	0.03
6010102	16743	BEAVERDAM CR	143.9	15.2	6 - 27	0.0	0.0 - 0.0	2.5	1.0 - 4.7	1.1	0.4 - 2.8	3.2	1.2 - 7.3	0.0	0.0 - 0.0	8.4	3.1 - 18.6	0.03
6010102	16744	WHITETOP LAURE	5.0	19.1	7 - 48	0.0	0.0 - 0.0	11.0	4.0 - 27.7	0.3	0.1 - 1.0	0.7	0.2 - 2.0	0.0	0.0 - 0.0	7.1	2.6 - 18.4	0.03
6010102	16745	LAUREL CR	35.7	12.4	4 - 26	0.0	0.0 - 0.0	2.8	0.9 - 6.2	0.6	0.2 - 1.8	1.7	0.5 - 4.1	0.0	0.0 - 0.0	7.2	2.2 - 18.1	0.03
6010102	16746	LAUREL CR	45.6	19.8	7 - 37	0.0	0.0 - 0.0	4.2	1.2 - 8.3	2.1	0.6 - 4.9	5.7	2.0 - 12.0	0.0	0.0 - 0.0	7.8	2.7 - 15.7	0.03
6010102	16747	LAUREL CR, GENT	27.5	12.7	5 - 25	0.0	0.0 - 0.0	1.0	0.4 - 1.9	0.7	0.2 - 2.0	2.1	0.7 - 5.6	0.0	0.0 - 0.0	8.9	3.4 - 19.9	0.03
6010102	16748	WHITETOP LAURE	6.9	10.6	4 - 30	0.0	0.0 - 0.0	4.9	1.9 - 14.4	0.0	0.0 - 0.2	0.1	0.0 - 0.3	0.0	0.0 - 0.0	5.6	2.2 - 16.9	0.03
6010102	16749	WHITETOP LAURE	117.9	14.1	5 - 39	0.0	0.0 - 0.0	2.7	0.9 - 6.6	0.7	0.2 - 1.8	1.8	0.5 - 4.8	0.0	0.0 - 0.0	8.9	3.0 - 25.8	0.03
6010102	16750	STRAIGHT CR	21.0	8.1	3 - 18	0.0	0.0 - 0.0	1.5	0.5 - 3.1	0.1	0.0 - 0.1	0.2	0.1 - 0.5	0.0	0.0 - 0.0	6.4	2.3 - 14.6	0.03
6010102	16751	HOLSTON R, S FK	166.7	32.0	9 - 77	0.0	0.0 - 0.0	2.7	0.7 - 6.3	6.1	1.8 - 16.7	14.7	4.0 - 37.2	0.0	0.0 - 0.0	8.6	3.1 - 23.1	0.03
6010102	16752	HOLSTON R, MIDD	9.1	42.5	14 - 75	0.0	0.0 - 0.0	9.2	2.8 - 19.2	7.0	2.0 - 17.0	18.8	6.2 - 44.4	0.0	0.0 - 0.0	7.5	2.3 - 16.3	0.03
6010102	16753	HOLSTON R, MIDD	158.4	38.3	14 - 84	12.5	4.5 - 26.9	8.7	2.9 - 19.3	2.8	0.9 - 7.4	7.3	2.5 - 17.1	0.0	0.0 - 0.0	6.9	2.7 - 17.3	0.03

8-digit HUC	ID	Name	Area	Catchment Yield		Point sources		Developed Land		Manure		Agricultural Land		Phosphate Mines		Soil parent rock		Frac
				kg/km^2	90% CI	kg/km^2	90% CI	kg/km^2	90% CI	kg/km^2	90% CI	kg/km^2	90% CI	kg/km^2	90% CI	kg/km^2	90% CI	
6010102	16755	NICKS CR	15.6	13.7	4 - 27	0.0	0.0 - 0.0	1.8	0.5 - 3.8	1.2	0.3 - 3.1	3.1	1.0 - 7.9	0.0	0.0 - 0.0	7.5	2.1 - 16.7	0.03
6010102	16756	HOLSTON R, MIDD	78.1	28.0	12 - 73	0.0	0.0 - 0.0	5.9	2.3 - 15.2	4.0	1.3 - 11.9	10.8	4.2 - 29.0	0.0	0.0 - 0.0	7.3	2.6 - 20.2	0.03
6010102	16757	BEAR CR	38.8	9.0	3 - 22	0.0	0.0 - 0.0	1.6	0.5 - 4.1	0.8	0.2 - 2.4	2.2	0.6 - 5.1	0.0	0.0 - 0.0	4.4	1.4 - 11.4	0.03
6010102	16758	WALKER CR	40.6	12.9	5 - 28	0.0	0.0 - 0.0	2.9	1.0 - 6.2	1.7	0.5 - 4.5	4.5	1.5 - 12.9	0.0	0.0 - 0.0	3.9	1.2 - 8.5	0.03
6010102	16759	SINKING CR	67.4	55.0	20 - 139	0.0	0.0 - 0.0	16.3	5.9 - 41.3	7.4	2.2 - 21.6	18.5	6.4 - 54.3	0.0	0.0 - 0.0	12.8	4.7 - 32.3	0.07
6010102	16760	BEAVER CR	100.4	55.1	18 - 104	0.0	0.0 - 0.0	14.8	4.8 - 28.7	6.8	2.0 - 15.6	19.9	7.8 - 57.9	0.0	0.0 - 0.0	13.6	4.3 - 28.8	0.07
6010102	16761	CEDAR CR	23.6	52.9	20 - 109	0.0	0.0 - 0.0	31.2	10.8 - 68.7	2.8	1.0 - 7.4	7.9	3.1 - 19.3	0.0	0.0 - 0.0	10.9	3.8 - 23.9	0.07
6010102	16762	BEAVER CR	46.4	59.4	19 - 129	0.0	0.0 - 0.0	28.5	8.8 - 67.1	5.5	1.7 - 15.6	13.4	4.7 - 32.1	0.0	0.0 - 0.0	12.1	4.2 - 27.9	0.07
6010102	16763	FALL CR	34.8	50.2	17 - 96	0.0	0.0 - 0.0	13.4	3.8 - 28.1	6.8	1.6 - 19.2	19.9	6.9 - 40.3	0.0	0.0 - 0.0	10.1	2.6 - 22.6	0.09
6010102	16764	REEDY CR	154.5	163.1	66 - 407	113.5	46.5 - 285.8	22.6	7.7 - 51.6	4.5	1.4 - 12.5	12.7	5.2 - 34.3	0.0	0.0 - 0.0	9.9	3.9 - 26.7	0.12
6010103	16765	*A	43.0	220.5	81 - 515	148.3	55.8 - 339.0	51.7	17.8 - 124.3	3.4	1.0 - 9.5	6.9	2.2 - 17.9	0.0	0.0 - 0.0	10.0	3.7 - 24.6	0.07
6010103	16766	WATAUGA R	71.4	42.6	14 - 75	0.0	0.0 - 0.0	16.6	5.7 - 36.7	4.1	1.1 - 11.7	11.6	4.2 - 26.2	0.0	0.0 - 0.0	10.4	3.7 - 21.7	0.07
6010103	16767	BUFFALO CR	99.6	31.4	15 - 76	0.0	0.0 - 0.0	11.6	4.9 - 27.2	2.5	0.9 - 8.8	8.7	3.5 - 22.9	0.0	0.0 - 0.0	8.6	3.9 - 20.8	0.07
6010103	16768	WATAUGA R	56.1	137.7	50 - 336	94.3	32.7 - 228.6	22.0	7.4 - 52.0	3.0	1.0 - 8.1	9.2	3.4 - 25.8	0.0	0.0 - 0.0	9.2	3.4 - 20.6	0.07
6010103	16769	DOE R	29.3	40.5	15 - 70	0.0	0.0 - 0.0	23.0	8.2 - 45.3	2.0	0.6 - 5.3	6.3	2.2 - 13.4	0.0	0.0 - 0.0	9.1	3.2 - 22.9	0.07
6010103	16770	DOE R	2.9	32.7	12 - 84	0.0	0.0 - 0.0	17.8	6.1 - 47.4	1.4	0.5 - 3.7	4.0	1.5 - 11.0	0.0	0.0 - 0.0	9.6	3.6 - 23.3	0.07
6010103	16771	TIGER CR	83.6	17.0	6 - 35	0.0	0.0 - 0.0	4.2	1.4 - 9.2	0.7	0.2 - 1.9	2.1	0.8 - 6.1	0.0	0.0 - 0.0	10.0	3.8 - 24.7	0.07
6010103	16772	DOE R	172.1	19.2	6 - 47	0.0	0.0 - 0.0	4.4	1.5 - 8.9	0.7	0.2 - 2.2	2.3	0.8 - 5.5	0.0	0.0 - 0.0	11.8	3.3 - 29.8	0.07
6010103	16773	DOE R, LAUREL FI	68.0	14.6	5 - 37	0.0	0.0 - 0.0	3.3	1.2 - 8.6	0.5	0.2 - 1.2	1.2	0.4 - 2.9	0.0	0.0 - 0.0	9.6	3.3 - 26.9	0.07
6010103	16774	WATAUGA R	22.5	29.3	9 - 49	0.0	0.0 - 0.0	13.1	3.7 - 24.1	2.2	0.5 - 6.3	7.1	2.0 - 16.3	0.0	0.0 - 0.0	7.0	2.2 - 12.1	0.07
6010103	16775	WATAUGA R	41.8	22.5	6 - 48	0.0	0.0 - 0.0	5.0	1.5 - 12.7	2.3	0.6 - 5.8	7.1	2.1 - 19.9	0.0	0.0 - 0.0	8.0	2.3 - 19.3	0.07
6010103	16776	WATAUGA R	146.5	27.5	11 - 48	2.1	0.8 - 4.3	9.6	3.7 - 18.0	1.0	0.3 - 2.4	2.9	1.1 - 7.2	0.0	0.0 - 0.0	11.9	4.5 - 25.4	0.03
6010103	16778	ROAN CR	106.3	18.1	5 - 33	0.0	0.0 - 0.0	2.3	0.6 - 4.5	2.0	0.5 - 4.9	5.4	1.4 - 12.0	0.0	0.0 - 0.0	8.4	2.5 - 18.5	0.03
6010103	16779	FORGE CR	104.8	21.7	7 - 41	0.0	0.0 - 0.0	4.7	1.4 - 9.6	1.3	0.4 - 4.0	3.4	1.2 - 8.6	0.0	0.0 - 0.0	12.3	4.3 - 27.5	0.03
6010103	16780	ROAN CR	74.3	40.9	12 - 87	13.7	3.9 - 33.9	8.0	2.2 - 18.8	2.5	0.7 - 7.1	6.8	2.2 - 17.5	0.0	0.0 - 0.0	9.9	2.9 - 22.3	0.03
6010103	16781	DOE CR	111.1	19.5	6 - 39	0.0	0.0 - 0.0	3.1	1.0 - 6.1	2.0	0.5 - 5.7	5.4	1.6 - 13.6	0.0	0.0 - 0.0	9.0	2.4 - 19.9	0.03
6010103	16782	STONY CR	157.9	16.1	5 - 31	0.0	0.0 - 0.0	3.4	1.0 - 6.2	1.2	0.4 - 3.6	3.7	1.2 - 9.1	0.0	0.0 - 0.0	7.8	2.5 - 16.9	0.07
6010104	16783	HOLSTON R	75.5	141.3	46 - 241	70.1	24.9 - 130.4	43.6	13.7 - 85.6	3.5	0.9 - 9.0	11.3	3.1 - 23.3	0.0	0.0 - 0.0	12.8	3.7 - 25.4	0.26
6010104	16784	HOLSTON R	216.5	61.3	23 - 138	2.8	1.1 - 6.5	8.7	3.1 - 21.0	12.0	3.8 - 31.1	25.4	8.1 - 61.6	0.0	0.0 - 0.0	12.4	4.9 - 30.0	0.25
6010104	16785	HOLSTON R	162.1	47.0	14 - 103	0.0	0.0 - 0.0	7.2	2.0 - 17.3	10.3	2.5 - 26.4	19.5	5.4 - 53.7	0.0	0.0 - 0.0	10.1	3.2 - 23.2	0.25
6010104	16786	LOUDERBACK CR	54.8	35.4	12 - 70	0.0	0.0 - 0.0	3.4	1.2 - 6.4	6.4	1.8 - 17.8	16.3	5.3 - 40.4	0.0	0.0 - 0.0	9.4	3.2 - 21.7	0.13
6010104	16787	HOLSTON R	18.5	56.3	14 - 116	0.0	0.0 - 0.0	4.7	1.3 - 9.4	11.4	2.7 - 25.1	27.9	6.8 - 74.2	0.0	0.0 - 0.0	12.3	3.4 - 27.5	0.13
6010104	16788	BEECH CR	136.7	21.6	8 - 56	0.0	0.0 - 0.0	2.7	1.0 - 6.7	3.1	0.9 - 9.1	7.9	2.7 - 22.6	0.0	0.0 - 0.0	7.9	2.7 - 21.5	0.13
6010104	16790	HOLSTON R	199.3	54.2	17 - 122	0.0	0.0 - 0.0	6.7	2.0 - 13.8	10.0	2.7 - 33.2	26.3	8.6 - 70.3	0.0	0.0 - 0.0	11.2	3.5 - 27.8	0.13
6010104	16791	BIG CR	138.7	23.5	8 - 55	0.0	0.0 - 0.0	4.4	1.7 - 10.7	4.5	1.4 - 12.2	7.1	2.3 - 19.5	0.0	0.0 - 0.0	7.4	2.6 - 18.2	0.13
6010104	16792	RICHLAND CR	171.2	27.4	9 - 70	1.2	0.4 - 3.1	6.6	2.0 - 16.7	3.9	1.1 - 10.7	7.9	2.8 - 22.6	0.0	0.0 - 0.0	7.7	2.7 - 20.3	0.25
6010104	16793	BIG FLAT CR	177.9	43.7	15 - 125	1.1	0.4 - 3.5	9.0	3.1 - 26.0	6.7	2.1 - 25.7	15.4	5.2 - 46.8	0.0	0.0 - 0.0	11.3	3.6 - 31.8	0.25
6010105	16794	FRENCH BROAD R	53.0	62.0	20 - 117	0.0	0.0 - 0.0	8.3	2.7 - 16.5	11.5	2.9 - 34.5	29.5	9.5 - 73.2	0.0	0.0 - 0.0	12.7	4.1 - 29.7	0.15
6010105	16795	FRENCH BROAD R	13.3	11.7	4 - 23	0.0	0.0 - 0.0	2.2	0.7 - 4.7	0.8	0.3 - 2.4	2.2	0.8 - 5.1	0.0	0.0 - 0.0	6.4	2.2 - 13.8	0.15

8-digit HUC	ID	Name	Area	Catchment Yield		Point sources		Developed Land		Manure		Agricultural Land		Phosphate Mines		Soil parent rock		Frac
				kg/km^2	90% CI	kg/km^2	90% CI	kg/km^2	90% CI	kg/km^2	90% CI	kg/km^2	90% CI	kg/km^2	90% CI	kg/km^2	90% CI	
6010105	16796	BIG CR	1.1	23.8	8 - 47	0.0	0.0 - 0.0	3.1	0.9 - 5.6	3.4	1.0 - 9.2	9.3	2.8 - 19.8	0.0	0.0 - 0.0	8.0	2.6 - 18.3	0.15
6010105	16797	BIG CR, GULF FK	124.7	8.1	3 - 18	0.0	0.0 - 0.0	2.3	0.8 - 4.4	0.3	0.1 - 1.0	0.8	0.3 - 2.0	0.0	0.0 - 0.0	4.7	1.8 - 11.7	0.15
6010105	16798	BIG CR, TRAIL FK	83.7	9.5	3 - 18	0.0	0.0 - 0.0	2.5	0.7 - 4.6	0.5	0.1 - 1.1	1.2	0.4 - 2.4	0.0	0.0 - 0.0	5.3	1.8 - 11.1	0.15
6010105	16799	FRENCH BROAD R	46.4	11.2	3 - 22	0.0	0.0 - 0.0	2.6	0.9 - 5.6	0.6	0.2 - 1.5	1.4	0.4 - 3.7	0.0	0.0 - 0.0	6.6	2.0 - 16.2	0.15
6010105	16800	WOLF CR	28.9	8.2	3 - 19	0.0	0.0 - 0.0	1.8	0.6 - 4.0	0.0	0.0 - 0.1	0.1	0.0 - 0.2	0.0	0.0 - 0.0	6.3	2.2 - 14.6	0.15
6010105	16801	FRENCH BROAD R	29.7	10.0	4 - 25	0.0	0.0 - 0.0	1.6	0.5 - 4.7	0.5	0.2 - 1.8	1.3	0.4 - 4.0	0.0	0.0 - 0.0	6.4	2.5 - 15.5	0.15
6010105	16802	FRENCH BROAD R	76.2	10.3	4 - 23	1.7	0.6 - 3.4	3.1	1.1 - 6.9	0.3	0.1 - 0.8	1.0	0.3 - 2.5	0.0	0.0 - 0.0	4.2	1.6 - 10.2	0.15
6010105	16803	SPRING CR	185.7	13.5	5 - 30	0.0	0.0 - 0.0	2.9	1.1 - 6.0	0.3	0.1 - 0.9	1.1	0.3 - 2.7	0.0	0.0 - 0.0	9.2	3.1 - 20.8	0.15
6010105	16804	FRENCH BROAD R	12.3	3.5	1 - 9	0.0	0.0 - 0.0	0.1	0.0 - 0.2	0.0	0.0 - 0.0	0.0	0.0 - 0.0	0.0	0.0 - 0.0	3.4	1.1 - 8.5	0.15
6010105	16805	FRENCH BROAD R	120.0	13.2	4 - 24	0.2	0.1 - 0.4	3.0	1.0 - 5.9	0.5	0.2 - 1.4	1.8	0.6 - 4.0	0.0	0.0 - 0.0	7.7	2.4 - 16.6	0.15
6010105	16806	FRENCH BROAD R	79.0	25.7	8 - 62	5.4	1.7 - 13.0	4.2	1.4 - 9.1	1.3	0.4 - 3.1	4.4	1.3 - 12.1	0.0	0.0 - 0.0	10.4	3.5 - 26.4	0.15
6010105	16807	FRENCH BROAD R	13.8	23.4	8 - 52	0.0	0.0 - 0.0	3.8	1.1 - 8.4	2.1	0.6 - 6.5	7.3	2.2 - 17.6	0.0	0.0 - 0.0	10.2	3.4 - 24.5	0.15
6010105	16808	SANDY MUSH CR	3.1	19.0	7 - 35	0.0	0.0 - 0.0	1.0	0.3 - 2.0	1.5	0.4 - 4.1	5.3	2.0 - 11.2	0.0	0.0 - 0.0	11.2	3.8 - 23.9	0.14
6010105	16809	SANDY MUSH CR	124.9	18.5	7 - 48	0.0	0.0 - 0.0	2.6	1.0 - 6.1	1.3	0.4 - 3.8	4.7	1.6 - 12.7	0.0	0.0 - 0.0	10.0	3.9 - 26.6	0.14
6010105	16810	TURKEY CR	79.0	23.3	7 - 54	0.0	0.0 - 0.0	3.0	0.9 - 8.1	2.2	0.6 - 7.0	7.9	2.5 - 21.7	0.0	0.0 - 0.0	10.3	3.3 - 25.9	0.14
6010105	16811	FRENCH BROAD R	16.8	23.9	7 - 50	0.0	0.0 - 0.0	4.9	1.3 - 10.3	2.0	0.5 - 4.4	7.1	2.2 - 18.5	0.0	0.0 - 0.0	10.0	3.1 - 20.6	0.14
6010105	16812	FRENCH BROAD R	2.3	19.6	6 - 41	0.0	0.0 - 0.0	4.5	1.4 - 9.7	1.2	0.3 - 3.4	5.1	1.6 - 12.1	0.0	0.0 - 0.0	8.8	2.5 - 20.5	0.14
6010105	16813	NEWFOUND CR	90.0	25.5	10 - 65	0.1	0.1 - 0.4	4.9	1.8 - 13.1	2.6	0.8 - 7.9	9.6	3.1 - 26.0	0.0	0.0 - 0.0	8.3	3.0 - 22.2	0.14
6010105	16815	FRENCH BROAD R	5.5	49.3	17 - 111	0.0	0.0 - 0.0	40.6	13.5 - 95.2	0.7	0.2 - 1.9	3.3	1.1 - 8.1	0.0	0.0 - 0.0	4.7	1.6 - 11.8	0.14
6010105	16816	HOMINY CR	91.5	42.5	15 - 114	0.2	0.1 - 0.6	25.6	8.4 - 73.0	1.3	0.4 - 3.6	4.8	1.5 - 14.3	0.0	0.0 - 0.0	10.6	3.4 - 28.6	0.14
6010105	16817	HOMINY CR	78.9	27.4	9 - 59	0.1	0.0 - 0.1	9.1	3.0 - 17.7	1.8	0.6 - 4.6	5.3	1.8 - 12.1	0.0	0.0 - 0.0	11.1	4.2 - 24.8	0.13
6010105	16818	S HOMINY CR	99.1	21.7	7 - 44	0.0	0.0 - 0.0	5.8	1.6 - 13.6	1.1	0.3 - 2.7	4.0	1.1 - 10.4	0.0	0.0 - 0.0	10.8	3.0 - 23.1	0.13
6010105	16819	FRENCH BROAD R	160.8	36.0	14 - 95	0.8	0.3 - 2.0	21.1	8.1 - 59.7	1.3	0.4 - 4.3	5.1	1.8 - 14.6	0.0	0.0 - 0.0	7.7	2.6 - 22.2	0.14
6010105	16820	FRENCH BROAD R	3.8	73.7	26 - 147	0.0	0.0 - 0.0	6.9	2.5 - 13.1	7.8	2.7 - 19.2	47.5	15.5 - 110.9	0.0	0.0 - 0.0	11.5	3.6 - 21.8	0.14
6010105	16822	MILLS R	25.7	47.6	17 - 114	2.2	0.7 - 6.0	12.4	4.1 - 28.7	3.2	0.9 - 9.7	19.0	6.6 - 55.2	0.0	0.0 - 0.0	10.9	3.8 - 27.0	0.14
6010105	16823	MILLS R	59.9	18.4	7 - 34	0.0	0.0 - 0.1	3.2	1.1 - 6.5	0.1	0.0 - 0.3	0.5	0.2 - 1.4	0.0	0.0 - 0.0	14.6	5.2 - 28.2	0.13
6010105	16824	MILLS R, S FK	107.7	22.8	9 - 48	0.0	0.0 - 0.0	3.2	1.0 - 6.6	0.2	0.1 - 0.5	1.1	0.4 - 2.9	0.0	0.0 - 0.0	18.2	6.6 - 40.7	0.13
6010105	16825	FRENCH BROAD R	12.9	72.7	27 - 161	2.4	0.8 - 5.5	46.1	16.9 - 96.5	1.6	0.5 - 4.4	8.8	3.1 - 23.3	0.0	0.0 - 0.0	13.9	5.1 - 33.1	0.14
6010105	16826	BOYLSTON CR	41.4	40.9	14 - 95	0.0	0.0 - 0.0	10.7	3.7 - 24.7	2.6	0.9 - 7.5	15.7	5.4 - 44.4	0.0	0.0 - 0.0	11.9	4.2 - 36.5	0.14
6010105	16827	FRENCH BROAD R	138.0	61.7	19 - 151	2.2	0.6 - 5.0	26.1	8.0 - 61.2	2.3	0.7 - 6.6	13.1	4.4 - 42.7	0.0	0.0 - 0.0	18.0	6.1 - 44.4	0.14
6010105	16828	FRENCH BROAD R	1.4	4036.8	1450 - 8543	3994.6	1438.2 - 8443.	13.9	3.9 - 25.3	1.9	0.6 - 5.2	9.7	2.8 - 24.3	0.0	0.0 - 0.0	16.7	5.6 - 32.7	0.13
6010105	16829	DAVIDSON R	131.3	40.0	13 - 89	0.0	0.0 - 0.1	12.7	4.5 - 29.8	0.3	0.1 - 0.9	1.5	0.5 - 4.5	0.0	0.0 - 0.0	25.5	9.0 - 58.7	0.13
6010105	16830	FRENCH BROAD R	103.1	74.7	25 - 201	3.9	1.5 - 11.1	36.7	12.8 - 94.5	1.9	0.6 - 5.1	10.6	3.5 - 27.7	0.0	0.0 - 0.0	21.6	7.6 - 56.3	0.13
6010105	16831	CATHEY'S CR	38.4	42.5	18 - 109	0.0	0.0 - 0.0	9.7	3.4 - 26.0	1.0	0.3 - 3.0	5.6	2.0 - 17.1	0.0	0.0 - 0.0	26.2	11.4 - 70.3	0.13
6010105	16832	FRENCH BROAD R	36.0	63.8	19 - 123	0.0	0.0 - 0.0	17.5	5.5 - 35.6	3.3	1.0 - 8.8	18.1	5.4 - 42.6	0.0	0.0 - 0.0	24.9	8.0 - 56.5	0.13
6010105	16833	FRENCH BROAD R	1.3	200.8	68 - 404	101.8	35.0 - 243.3	54.0	17.5 - 112.5	3.2	0.9 - 7.5	14.9	4.5 - 42.1	0.0	0.0 - 0.0	27.0	9.0 - 54.9	0.13
6010105	16834	FRENCH BROAD R	98.8	42.9	17 - 102	0.0	0.0 - 0.0	8.9	3.1 - 19.8	0.5	0.2 - 1.5	2.6	0.9 - 8.5	0.0	0.0 - 0.0	30.9	11.3 - 76.1	0.13
6010105	16835	FRENCH BROAD R	76.3	48.7	18 - 148	0.0	0.0 - 0.0	13.8	4.9 - 41.2	0.9	0.3 - 3.2	5.1	1.8 - 17.4	0.0	0.0 - 0.0	28.9	10.8 - 88.6	0.13
6010105	16836	FRENCH BROAD R	20.4	54.3	20 - 117	0.0	0.0 - 0.0	22.2	8.0 - 50.7	0.9	0.3 - 2.6	4.5	1.5 - 11.7	0.0	0.0 - 0.0	26.7	8.9 - 58.6	0.13

8-digit HUC	ID	Name	Area	Catchment Yield		Point sources		Developed Land		Manure		Agricultural Land		Phosphate Mines		Soil parent rock		Frac
				kg/km^2	90% CI	kg/km^2	90% CI	kg/km^2	90% CI	kg/km^2	90% CI	kg/km^2	90% CI	kg/km^2	90% CI	kg/km^2	90% CI	
6010105	16837	FRENCH BROAD R	66.4	36.6	13 - 81	0.0	0.0 - 0.0	9.4	2.8 - 21.2	0.4	0.1 - 1.0	2.2	0.7 - 6.5	0.0	0.0 - 0.0	24.6	9.5 - 57.8	0.13
6010105	16838	LITTLE R	155.6	40.0	15 - 99	0.1	0.0 - 0.1	12.4	4.8 - 29.1	1.0	0.3 - 2.7	5.5	2.0 - 16.2	0.0	0.0 - 0.0	21.1	8.5 - 62.7	0.13
6010105	16839	MUD CR	38.8	418.6	171 - 997	367.6	152.8 - 873.0	31.3	11.7 - 74.0	1.1	0.4 - 3.3	6.6	2.4 - 16.9	0.0	0.0 - 0.0	12.0	4.6 - 31.3	0.14
6010105	16840	MUD CR	136.8	88.5	26 - 160	0.6	0.2 - 1.2	59.5	16.8 - 117.6	1.8	0.5 - 4.6	10.9	2.9 - 27.3	0.0	0.0 - 0.0	15.7	5.2 - 32.4	0.13
6010105	16841	CLEAR CR	114.7	43.5	13 - 77	0.7	0.2 - 1.3	14.2	3.8 - 24.6	2.2	0.6 - 5.7	13.7	4.0 - 32.9	0.0	0.0 - 0.0	12.7	4.0 - 28.4	0.13
6010105	16842	CANE CR	17.9	59.1	21 - 128	0.0	0.0 - 0.0	36.2	12.5 - 87.8	2.2	0.6 - 5.4	12.0	4.0 - 27.1	0.0	0.0 - 0.0	8.8	3.3 - 23.7	0.14
6010105	16843	HOOPER CR	42.4	21.2	8 - 55	0.0	0.0 - 0.0	5.1	1.8 - 13.6	1.0	0.3 - 3.8	5.8	1.7 - 15.8	0.0	0.0 - 0.0	9.3	3.1 - 23.9	0.14
6010105	16844	CANE CR	166.0	26.2	9 - 66	0.3	0.1 - 0.8	10.3	3.0 - 25.1	1.6	0.4 - 5.5	5.9	1.9 - 18.1	0.0	0.0 - 0.0	8.1	2.6 - 21.9	0.14
6010105	16845	SWANNONA R	101.4	36.0	11 - 67	0.0	0.0 - 0.0	29.0	9.0 - 58.1	0.3	0.1 - 0.9	1.1	0.4 - 2.6	0.0	0.0 - 0.0	5.4	1.8 - 13.6	0.14
6010105	16846	SWANNONA R	10.9	35.9	13 - 72	0.0	0.0 - 0.0	25.0	8.2 - 53.6	1.3	0.4 - 3.6	4.4	1.6 - 10.1	0.0	0.0 - 0.0	5.3	1.9 - 12.2	0.13
6010105	16847	SWANNONA R	28.4	33.3	12 - 85	0.0	0.0 - 0.0	23.8	9.0 - 61.2	0.4	0.1 - 1.3	1.4	0.4 - 3.9	0.0	0.0 - 0.0	7.7	2.6 - 24.6	0.13
6010105	16848	SWANNONA R	56.6	35.7	11 - 66	0.0	0.0 - 0.0	25.4	7.3 - 54.0	0.3	0.1 - 0.6	1.1	0.3 - 2.4	0.0	0.0 - 0.0	8.9	3.0 - 16.0	0.12
6010105	16849	SWANNONA R, N F	82.6	9.6	3 - 25	0.0	0.0 - 0.0	2.7	1.0 - 6.4	0.2	0.1 - 0.5	0.7	0.2 - 1.7	0.0	0.0 - 0.0	6.1	1.9 - 17.9	0.12
6010105	16850	BEETREE CR	36.0	14.0	5 - 31	0.0	0.0 - 0.0	3.7	1.1 - 8.9	0.2	0.0 - 0.5	0.6	0.2 - 1.4	0.0	0.0 - 0.0	9.5	3.2 - 22.3	0.13
6010105	16851	BULL CR	27.7	15.6	6 - 34	0.0	0.0 - 0.0	5.5	2.0 - 11.3	0.5	0.2 - 1.2	1.8	0.6 - 4.4	0.0	0.0 - 0.0	7.8	2.9 - 18.3	0.13
6010105	16852	FLAT CR	64.3	23.7	8 - 42	2.8	0.9 - 5.7	6.3	1.8 - 12.8	1.5	0.5 - 4.1	5.5	1.7 - 15.1	0.0	0.0 - 0.0	7.6	2.3 - 17.0	0.14
6010105	16853	IVY CR	105.4	21.0	7 - 35	0.0	0.0 - 0.0	4.3	1.4 - 7.3	1.6	0.4 - 3.6	5.6	1.8 - 11.4	0.0	0.0 - 0.0	9.5	3.3 - 18.2	0.15
6010105	16854	IVY CR	14.2	19.7	6 - 52	0.0	0.0 - 0.0	4.9	1.4 - 12.2	1.2	0.3 - 3.4	4.4	1.2 - 12.8	0.0	0.0 - 0.0	9.2	2.6 - 23.2	0.14
6010105	16855	IVY CR	155.8	12.1	4 - 31	0.0	0.0 - 0.0	2.6	0.8 - 6.5	0.5	0.1 - 1.4	1.7	0.6 - 5.0	0.0	0.0 - 0.0	7.3	2.3 - 21.7	0.13
6010105	16856	PAINT FORK CR	20.3	23.7	8 - 53	0.0	0.0 - 0.0	6.7	2.5 - 16.8	1.7	0.6 - 5.2	5.8	2.0 - 16.2	0.0	0.0 - 0.0	9.4	3.3 - 23.5	0.13
6010105	16857	PAINT FORK CR	37.3	13.5	4 - 27	0.0	0.0 - 0.0	2.0	0.6 - 4.5	0.7	0.2 - 2.2	2.5	0.7 - 6.6	0.0	0.0 - 0.0	8.3	2.5 - 18.8	0.13
6010105	16858	CALIFORNIA CR	64.0	22.0	8 - 46	0.0	0.0 - 0.0	5.3	1.6 - 12.7	1.4	0.4 - 3.9	4.7	1.7 - 12.5	0.0	0.0 - 0.0	10.6	3.9 - 26.4	0.13
6010105	16859	GABRIEL CR	18.9	51.6	17 - 119	29.0	9.7 - 65.1	6.5	2.2 - 14.8	1.5	0.4 - 4.6	5.3	1.9 - 13.7	0.0	0.0 - 0.0	9.2	3.0 - 25.3	0.14
6010105	16860	WALNUT CR	44.2	18.8	6 - 43	0.0	0.0 - 0.0	4.3	1.5 - 8.6	1.0	0.3 - 2.8	3.3	1.0 - 9.0	0.0	0.0 - 0.0	10.2	2.8 - 25.7	0.15
6010105	16861	BIG LAUREL CR	25.0	6.2	2 - 11	0.0	0.0 - 0.0	3.0	0.9 - 5.2	0.0	0.0 - 0.0	0.1	0.0 - 0.2	0.0	0.0 - 0.0	3.0	0.9 - 5.6	0.15
6010105	16862	BIG LAUREL CR	175.5	19.0	7 - 34	0.0	0.0 - 0.1	4.0	1.3 - 7.6	0.6	0.2 - 1.6	1.7	0.6 - 3.8	0.0	0.0 - 0.0	12.6	4.6 - 27.7	0.14
6010105	16863	SHELTON LAUREL	142.8	7.1	3 - 14	0.1	0.0 - 0.2	1.7	0.6 - 3.6	0.2	0.1 - 0.5	0.7	0.2 - 1.7	0.0	0.0 - 0.0	4.4	1.6 - 9.1	0.14
6010105	16864	PAINT CR	64.7	8.2	3 - 20	0.0	0.0 - 0.0	1.5	0.5 - 3.9	0.2	0.0 - 0.5	0.3	0.1 - 0.7	0.0	0.0 - 0.0	6.2	2.2 - 15.5	0.15
6010105	16865	LONG CR	31.6	22.1	9 - 62	0.0	0.0 - 0.0	2.9	1.0 - 7.9	3.2	1.1 - 10.5	8.3	2.7 - 27.6	0.0	0.0 - 0.0	7.7	3.1 - 21.3	0.15
6010106	16867	PIGEON R	6.4	59.8	23 - 116	0.0	0.0 - 0.0	6.5	2.4 - 12.6	11.6	3.7 - 35.7	29.1	11.2 - 65.4	0.0	0.0 - 0.0	12.7	4.6 - 31.3	0.15
6010106	16868	SINKING CR	39.5	34.4	13 - 59	0.0	0.0 - 0.0	16.0	5.7 - 31.7	2.2	0.7 - 6.4	5.5	2.0 - 11.5	0.0	0.0 - 0.0	10.7	3.9 - 23.8	0.15
6010106	16869	PIGEON R	9.1	556.7	221 - 1233	478.1	182.5 - 1066.3	47.8	16.3 - 99.9	5.3	1.6 - 14.9	13.3	4.3 - 33.9	0.0	0.0 - 0.0	12.1	4.4 - 31.0	0.15
6010106	16870	COSBY CR	7.9	40.7	13 - 75	0.0	0.0 - 0.0	15.5	5.2 - 35.2	3.8	1.2 - 9.2	9.6	3.5 - 21.3	0.0	0.0 - 0.0	11.8	3.8 - 28.4	0.15
6010106	16871	BOGARD CR	22.5	28.8	9 - 57	0.0	0.0 - 0.0	3.2	1.1 - 6.3	3.2	1.0 - 8.2	8.6	2.8 - 18.1	0.0	0.0 - 0.0	13.7	4.6 - 34.0	0.14
6010106	16872	COSBY CR	43.5	16.7	6 - 36	0.0	0.0 - 0.0	4.8	1.8 - 10.9	1.2	0.4 - 2.9	3.0	1.2 - 7.8	0.0	0.0 - 0.0	7.6	2.9 - 17.1	0.14
6010106	16873	INDIAN CAMP CR	24.6	17.5	6 - 37	0.0	0.0 - 0.0	4.2	1.3 - 10.1	0.2	0.1 - 0.5	0.5	0.1 - 1.2	0.0	0.0 - 0.0	12.5	4.5 - 27.8	0.11
6010106	16874	COSBY CR	53.8	12.3	4 - 30	0.0	0.0 - 0.0	2.7	0.8 - 6.3	0.2	0.1 - 0.5	0.5	0.1 - 1.3	0.0	0.0 - 0.0	9.0	2.8 - 25.5	0.11
6010106	16875	PIGEON R	101.3	11.2	4 - 21	0.0	0.0 - 0.0	4.3	1.4 - 9.2	0.4	0.1 - 0.9	0.8	0.3 - 2.0	0.0	0.0 - 0.0	5.7	2.0 - 12.0	0.15
6010106	16876	BIG CR	94.9	9.0	3 - 20	0.0	0.0 - 0.0	1.0	0.3 - 2.2	0.0	0.0 - 0.1	0.1	0.0 - 0.1	0.0	0.0 - 0.0	7.9	2.9 - 18.5	0.14

8-digit HUC	ID	Name	Area	Catchment Yield		Point sources		Developed Land		Manure		Agricultural Land		Phosphate Mines		Soil parent rock		Frac
				kg/km^2	90% CI	kg/km^2	90% CI	kg/km^2	90% CI	kg/km^2	90% CI	kg/km^2	90% CI	kg/km^2	90% CI	kg/km^2	90% CI	
6010106	16877	PIGEON R	113.9	9.6	4 - 26	0.5	0.1 - 1.3	2.5	0.9 - 5.9	0.0	0.0 - 0.1	0.1	0.0 - 0.3	0.0	0.0 - 0.0	6.5	2.4 - 19.1	0.14
6010106	16878	CATALOOCHEE CI	32.0	8.5	2 - 18	0.0	0.0 - 0.0	0.8	0.3 - 1.4	0.0	0.0 - 0.0	0.0	0.0 - 0.0	0.0	0.0 - 0.0	7.7	2.2 - 16.4	0.14
6010106	16879	PIGEON R	31.7	12.4	4 - 23	0.0	0.0 - 0.0	4.6	1.6 - 8.6	0.4	0.1 - 0.9	0.8	0.3 - 1.8	0.0	0.0 - 0.0	6.6	2.3 - 15.4	0.14
6010106	16881	JONATHAN'S CR	119.5	49.0	18 - 117	19.6	7.5 - 48.3	11.3	3.6 - 27.1	1.8	0.6 - 4.8	4.8	1.6 - 12.3	0.0	0.0 - 0.0	11.4	4.3 - 27.8	0.13
6010106	16882	JONATHAN'S CR	37.7	23.0	7 - 42	0.0	0.0 - 0.0	10.6	3.3 - 20.4	0.4	0.1 - 1.2	1.1	0.3 - 2.6	0.0	0.0 - 0.0	10.9	4.0 - 22.7	0.12
6010106	16883	CAMPBELL CR	21.3	12.1	5 - 21	0.0	0.0 - 0.0	2.3	0.9 - 4.0	0.4	0.1 - 0.9	0.9	0.3 - 2.5	0.0	0.0 - 0.0	8.6	3.1 - 17.0	0.12
6010106	16884	PIGEON R	10.7	19.3	7 - 47	0.0	0.0 - 0.0	3.2	1.1 - 8.2	2.6	0.8 - 7.7	6.7	2.2 - 19.7	0.0	0.0 - 0.0	6.7	2.7 - 16.9	0.13
6010106	16885	PIGEON R	32.3	132.4	52 - 313	104.1	40.2 - 250.5	6.5	2.2 - 13.0	3.6	1.1 - 9.8	9.2	3.4 - 23.9	0.0	0.0 - 0.0	9.1	3.3 - 19.6	0.13
6010106	16886	RICHLAND CR	90.6	42.1	14 - 80	0.0	0.0 - 0.0	25.5	7.3 - 47.0	1.7	0.4 - 5.0	4.4	1.3 - 10.1	0.0	0.0 - 0.0	10.6	3.4 - 21.4	0.13
6010106	16887	RICHLAND CR	34.5	38.4	12 - 86	0.2	0.1 - 0.4	19.8	6.2 - 48.8	1.3	0.4 - 4.5	3.1	1.0 - 7.4	0.0	0.0 - 0.0	13.9	4.6 - 34.7	0.10
6010106	16888	ALLEN CR	51.8	20.0	6 - 50	0.0	0.0 - 0.0	7.0	2.3 - 18.4	0.3	0.1 - 0.9	1.0	0.3 - 2.6	0.0	0.0 - 0.0	11.7	3.8 - 29.4	0.10
6010106	16889	PIGEON R	15.6	41.6	15 - 112	0.0	0.0 - 0.0	19.7	7.4 - 47.1	2.7	0.9 - 8.5	6.8	2.1 - 19.6	0.0	0.0 - 0.0	12.4	4.0 - 32.4	0.13
6010106	16890	PIGEON R	10.1	50.3	16 - 95	0.0	0.0 - 0.0	27.8	8.5 - 58.9	2.8	0.8 - 9.0	7.3	2.4 - 20.4	0.0	0.0 - 0.0	12.4	4.2 - 27.5	0.13
6010106	16891	PIGEON R, W FK	167.1	24.0	8 - 61	0.1	0.0 - 0.2	5.1	1.8 - 13.4	0.8	0.2 - 2.5	1.9	0.6 - 6.0	0.0	0.0 - 0.0	16.1	4.9 - 42.9	0.12
6010106	16892	PIGEON R, E FK	136.9	27.8	10 - 65	0.3	0.1 - 0.6	6.7	2.7 - 12.9	0.9	0.3 - 2.3	2.1	0.8 - 5.1	0.0	0.0 - 0.0	17.9	6.9 - 44.6	0.12
6010106	16893	BEAVERDAM CR	29.5	26.0	10 - 55	0.0	0.0 - 0.0	8.7	3.0 - 17.5	1.8	0.5 - 4.8	4.7	1.6 - 11.9	0.0	0.0 - 0.0	10.8	3.8 - 25.5	0.13
6010106	16894	CRABTREE CR	67.2	21.8	8 - 52	0.0	0.0 - 0.0	2.4	0.9 - 5.6	2.7	0.8 - 7.3	6.6	2.1 - 16.9	0.0	0.0 - 0.0	10.1	4.0 - 26.2	0.13
6010106	16895	SPRING CR	66.5	22.0	8 - 46	0.2	0.1 - 0.5	3.2	1.0 - 6.9	1.9	0.6 - 5.3	4.7	1.6 - 11.2	0.0	0.0 - 0.0	12.0	3.9 - 28.7	0.13
6010107	16896	FRENCH BROAD R	59.9	51.1	18 - 95	0.0	0.0 - 0.0	15.7	5.7 - 32.3	4.7	1.5 - 12.6	15.5	4.9 - 34.7	0.0	0.0 - 0.0	15.1	5.2 - 35.2	0.26
6010107	16897	FRENCH BROAD R	11.6	50.5	14 - 106	0.0	0.0 - 0.0	3.5	0.9 - 7.8	8.6	2.1 - 23.4	26.2	7.9 - 74.1	0.0	0.0 - 0.0	12.2	3.8 - 28.3	0.25
6010107	16898	HAPPY CR	4.4	68.9	23 - 119	0.0	0.0 - 0.0	7.2	2.6 - 12.8	13.0	4.0 - 34.3	37.7	12.7 - 74.7	0.0	0.0 - 0.0	11.0	3.9 - 24.8	0.25
6010107	16899	HAPPY CR	45.7	59.9	20 - 133	0.0	0.0 - 0.0	12.5	4.2 - 22.6	9.1	2.6 - 21.8	27.1	8.8 - 67.8	0.0	0.0 - 0.0	11.3	3.6 - 26.4	0.25
6010107	16900	KNOB CR	66.3	30.0	10 - 74	0.0	0.0 - 0.0	4.0	1.2 - 9.8	2.9	0.8 - 10.4	8.6	2.8 - 23.3	0.0	0.0 - 0.0	14.5	4.3 - 41.2	0.25
6010107	16901	FRENCH BROAD R	18.9	57.6	20 - 137	0.0	0.0 - 0.0	6.9	2.4 - 17.1	9.9	2.7 - 33.5	29.1	9.7 - 73.0	0.0	0.0 - 0.0	11.7	4.1 - 29.9	0.25
6010107	16902	LITTLE PIGEON R	3.4	1666.5	482 - 3774	1609.1	464.1 - 3796.(	17.9	4.2 - 42.7	7.4	2.1 - 17.6	21.2	7.1 - 57.1	0.0	0.0 - 0.0	10.9	3.1 - 29.2	0.25
6010107	16903	GISTS CR	52.9	30.2	9 - 62	0.0	0.0 - 0.0	5.4	1.5 - 11.8	3.2	0.9 - 9.2	9.1	2.8 - 24.2	0.0	0.0 - 0.0	12.5	4.0 - 27.9	0.25
6010107	16904	LITTLE PIGEON R	20.3	47.9	14 - 90	0.0	0.0 - 0.0	13.0	4.0 - 26.2	6.1	1.4 - 20.4	17.6	4.9 - 35.8	0.0	0.0 - 0.0	11.1	3.1 - 26.1	0.25
6010107	16905	LITTLE PIGEON R,	24.1	60.6	17 - 129	0.0	0.0 - 0.0	27.2	7.3 - 53.5	5.4	1.5 - 15.8	15.7	4.9 - 42.5	0.0	0.0 - 0.0	12.3	4.0 - 27.8	0.25
6010107	16906	WALDEN CR	23.8	306.4	96 - 630	263.2	83.1 - 578.4	22.5	6.3 - 47.4	1.7	0.5 - 4.6	4.9	1.3 - 12.6	0.0	0.0 - 0.0	14.2	4.8 - 34.0	0.24
6010107	16907	WALDEN CR	63.2	20.3	6 - 39	0.0	0.0 - 0.0	3.9	1.1 - 7.9	0.7	0.2 - 1.6	1.9	0.6 - 4.6	0.0	0.0 - 0.0	13.8	4.1 - 29.7	0.22
6010107	16908	COVE CR	50.7	34.7	12 - 81	0.0	0.0 - 0.0	10.9	3.6 - 24.5	3.1	0.9 - 8.5	9.2	3.0 - 23.7	0.0	0.0 - 0.0	11.5	3.9 - 27.0	0.22
6010107	16909	LITTLE PIGEON R,	80.6	80.4	30 - 156	43.4	16.4 - 89.5	22.0	7.3 - 48.5	0.5	0.1 - 1.2	1.4	0.5 - 3.5	0.0	0.0 - 0.0	13.1	4.6 - 30.4	0.24
6010107	16910	LITTLE PIGEON R,	109.8	24.2	8 - 59	0.0	0.0 - 0.0	9.2	3.2 - 19.5	0.0	0.0 - 0.0	0.0	0.0 - 0.1	0.0	0.0 - 0.0	14.9	4.9 - 33.2	0.22
6010107	16911	DUDLEY CR	39.0	27.4	9 - 68	0.0	0.0 - 0.0	13.6	5.0 - 32.2	0.1	0.0 - 0.4	0.2	0.1 - 0.7	0.0	0.0 - 0.0	13.4	4.5 - 32.5	0.22
6010107	16912	LITTLE PIGEON R	55.9	46.7	17 - 82	0.0	0.0 - 0.0	15.9	5.6 - 34.9	4.9	1.6 - 14.0	14.2	5.3 - 38.3	0.0	0.0 - 0.0	11.7	4.1 - 25.8	0.25
6010107	16913	LITTLE PIGEON R	22.7	55.4	19 - 129	0.0	0.0 - 0.0	8.1	2.5 - 19.3	7.0	2.1 - 21.0	20.7	6.7 - 52.6	0.0	0.0 - 0.0	19.5	7.0 - 54.7	0.24
6010107	16914	BIRD CR	41.9	23.5	9 - 56	0.0	0.0 - 0.0	11.2	3.8 - 29.8	0.8	0.3 - 2.6	2.2	0.8 - 6.4	0.0	0.0 - 0.0	9.4	3.7 - 20.7	0.23
6010107	16915	LITTLE PIGEON R	36.6	16.0	5 - 28	0.0	0.0 - 0.0	4.1	1.3 - 8.1	0.6	0.2 - 1.7	1.8	0.6 - 4.0	0.0	0.0 - 0.0	9.4	2.7 - 20.6	0.23
6010107	16916	LITTLE PIGEON R	33.9	16.7	5 - 44	0.0	0.0 - 0.0	4.2	1.4 - 10.2	0.3	0.1 - 0.8	0.7	0.3 - 2.2	0.0	0.0 - 0.0	11.6	3.5 - 31.5	0.22

8-digit HUC	ID	Name	Area	Catchment Yield		Point sources		Developed Land		Manure		Agricultural Land		Phosphate Mines		Soil parent rock		Frac
				kg/km^2	90% CI	kg/km^2	90% CI	kg/km^2	90% CI	kg/km^2	90% CI	kg/km^2	90% CI	kg/km^2	90% CI	kg/km^2	90% CI	
6010107	16917	LITTLE PIGEON R	46.6	17.8	6 - 41	0.0	0.0 - 0.0	0.1	0.0 - 0.2	0.0	0.0 - 0.0	0.1	0.0 - 0.1	0.0	0.0 - 0.0	17.6	6.0 - 41.2	0.20
6010107	16918	LITTLE PIGEON R,	52.4	16.2	6 - 38	0.0	0.0 - 0.0	0.0	0.0 - 0.0	0.0	0.0 - 0.0	0.0	0.0 - 0.0	0.0	0.0 - 0.0	16.2	6.3 - 37.8	0.20
6010107	16919	WEBB CR	47.4	23.1	8 - 43	2.8	1.0 - 5.1	8.2	2.9 - 17.1	0.1	0.0 - 0.3	0.3	0.1 - 0.6	0.0	0.0 - 0.0	11.7	3.8 - 24.5	0.22
6010107	16920	LITTLE EAST FK	63.0	34.1	10 - 74	0.0	0.0 - 0.0	6.1	1.9 - 12.5	4.3	1.3 - 11.7	12.7	4.6 - 36.2	0.0	0.0 - 0.0	11.1	4.3 - 25.5	0.24
6010107	16921	DUNN CR	87.9	14.4	5 - 37	0.0	0.0 - 0.0	3.1	1.0 - 8.0	0.5	0.1 - 1.3	1.3	0.4 - 3.8	0.0	0.0 - 0.1	9.5	3.2 - 24.1	0.22
6010107	16922	WILHITE CR	31.9	21.7	7 - 45	2.4	0.7 - 5.1	9.1	2.7 - 19.3	0.3	0.1 - 0.8	0.8	0.3 - 2.1	0.0	0.0 - 0.0	9.0	2.7 - 22.5	0.22
6010107	16923	FRENCH BROAD R	38.7	52.4	17 - 116	0.0	0.0 - 0.0	5.9	1.7 - 13.0	9.0	2.2 - 28.6	26.5	8.5 - 68.8	0.0	0.0 - 0.0	11.1	3.6 - 26.9	0.25
6010107	16924	FRENCH BROAD R	81.7	55.5	21 - 129	1.6	0.6 - 3.7	4.0	1.3 - 8.7	9.9	2.5 - 31.7	24.5	9.6 - 71.3	0.0	0.0 - 0.0	15.5	4.9 - 38.2	0.15
6010107	16925	DUMPLIN CR	126.3	55.0	16 - 112	0.0	0.0 - 0.0	8.5	2.8 - 18.9	10.7	3.1 - 28.7	22.7	6.7 - 50.7	0.0	0.0 - 0.0	13.1	4.2 - 30.7	0.25
6010108	16926	NOLICHUCKY R	11.5	45.5	16 - 72	0.0	0.0 - 0.0	1.6	0.4 - 2.7	9.4	3.1 - 20.6	20.8	7.7 - 40.6	0.0	0.0 - 0.0	13.8	4.0 - 26.0	0.15
6010108	16927	NOLICHUCKY R	74.6	60.4	23 - 105	0.0	0.0 - 0.0	6.8	2.2 - 12.6	12.2	3.7 - 27.6	28.0	10.1 - 57.5	0.0	0.0 - 0.0	13.5	4.2 - 30.8	0.15
6010108	16929	NOLICHUCKY R	38.3	47.1	14 - 109	0.0	0.0 - 0.0	4.2	1.2 - 8.5	8.9	2.3 - 26.0	21.8	7.1 - 56.9	0.0	0.0 - 0.0	12.3	3.8 - 28.7	0.15
6010108	16930	NOLICHUCKY R	154.8	54.1	18 - 118	0.0	0.0 - 0.0	4.6	1.5 - 10.8	11.1	3.2 - 34.2	27.4	8.0 - 73.3	0.0	0.0 - 0.0	11.0	3.4 - 25.5	0.15
6010108	16931	NOLICHUCKY R	16.6	59.9	18 - 117	0.0	0.0 - 0.0	5.4	1.7 - 11.3	12.8	3.6 - 31.4	31.9	10.0 - 71.4	0.0	0.0 - 0.0	9.8	2.9 - 21.9	0.15
6010108	16932	MEADOW CR	49.2	34.3	12 - 81	0.0	0.0 - 0.0	3.0	1.1 - 7.1	6.7	2.3 - 19.2	16.4	5.8 - 42.6	0.0	0.0 - 0.0	8.2	2.8 - 20.7	0.15
6010108	16934	COVE CR	76.7	21.9	8 - 41	0.0	0.0 - 0.0	2.8	0.9 - 5.0	3.4	1.2 - 8.7	8.4	3.2 - 20.7	0.0	0.0 - 0.0	7.2	2.5 - 17.2	0.15
6010108	16935	NOLICHUCKY R	427.1	59.0	19 - 122	11.2	4.3 - 22.7	8.7	2.9 - 19.8	8.6	2.2 - 30.6	21.0	7.0 - 48.0	0.0	0.0 - 0.0	9.5	3.3 - 24.4	0.15
6010108	16936	NOLICHUCKY R	405.3	38.7	12 - 93	2.8	0.9 - 6.6	5.2	1.6 - 13.0	6.9	2.0 - 21.4	15.0	4.2 - 42.2	0.0	0.0 - 0.1	8.9	3.0 - 20.6	0.14
6010108	16937	NOLICHUCKY R	19.9	141.5	41 - 374	114.0	33.7 - 305.3	17.6	4.5 - 41.1	0.3	0.1 - 1.1	1.3	0.4 - 4.3	0.0	0.0 - 0.0	8.2	2.5 - 21.8	0.14
6010108	16938	S INDIAN CR	205.2	17.8	6 - 42	0.0	0.0 - 0.0	4.6	1.4 - 10.5	0.3	0.1 - 0.9	1.3	0.4 - 4.0	0.0	0.0 - 0.0	11.6	4.0 - 27.4	0.14
6010108	16939	NOLICHUCKY R	99.9	10.1	4 - 24	0.0	0.0 - 0.0	1.8	0.6 - 4.0	0.1	0.0 - 0.4	0.5	0.2 - 1.1	0.0	0.0 - 0.0	7.6	2.9 - 16.8	0.14
6010108	16940	SAM'S CR	60.0	20.3	8 - 45	0.0	0.0 - 0.0	4.8	1.8 - 10.2	0.3	0.1 - 0.9	1.0	0.4 - 2.3	0.0	0.0 - 0.0	14.2	5.7 - 37.2	0.13
6010108	16941	RICE CR	41.7	31.1	10 - 61	0.0	0.0 - 0.0	7.5	2.5 - 16.1	0.8	0.3 - 1.9	2.8	0.9 - 7.5	0.0	0.0 - 0.0	20.0	6.7 - 42.7	0.13
6010108	16942	SAM'S CR	149.7	21.8	7 - 39	0.0	0.0 - 0.0	5.1	1.6 - 11.1	1.1	0.3 - 3.3	4.0	1.3 - 11.0	0.0	0.0 - 0.0	11.6	3.8 - 28.0	0.13
6010108	16943	NOLICHUCKY R	65.4	15.6	6 - 36	0.1	0.0 - 0.2	2.2	0.8 - 5.3	0.4	0.1 - 1.0	1.4	0.5 - 3.6	0.0	0.0 - 0.0	11.5	4.3 - 28.9	0.13
6010108	16944	NOLICHUCKY R	1.6	15.7	4 - 33	0.0	0.0 - 0.0	0.9	0.3 - 2.1	0.5	0.1 - 1.3	2.4	0.7 - 5.9	0.0	0.0 - 0.0	11.9	3.3 - 25.8	0.13
6010108	16945	CANE R	213.2	21.5	6 - 54	3.9	1.2 - 10.2	4.5	1.4 - 11.2	0.7	0.2 - 1.9	2.5	0.8 - 8.1	0.0	0.0 - 0.0	10.0	2.7 - 24.9	0.13
6010108	16946	N TOE R	44.9	18.6	6 - 43	0.0	0.0 - 0.0	4.2	1.3 - 8.9	0.7	0.2 - 2.0	2.6	0.9 - 7.2	0.0	0.0 - 0.0	11.0	3.5 - 31.7	0.13
6010108	16947	N TOE R	28.3	18.7	7 - 38	0.0	0.0 - 0.0	5.8	1.9 - 13.2	1.1	0.3 - 3.2	4.1	1.3 - 10.4	0.0	0.0 - 0.0	7.7	2.9 - 19.2	0.13
6010108	16948	S TOE R	116.7	25.4	7 - 52	0.0	0.0 - 0.0	10.8	3.0 - 23.8	1.2	0.3 - 3.6	3.9	1.2 - 10.5	0.0	0.0 - 0.0	9.6	2.8 - 20.7	0.13
6010108	16949	N TOE R	11.8	18.6	6 - 41	0.0	0.0 - 0.0	6.7	1.9 - 13.6	1.1	0.3 - 3.0	4.4	1.3 - 11.8	0.0	0.0 - 0.0	6.4	2.3 - 17.5	0.13
6010108	16950	BIG CRABTREE CF	63.1	19.8	7 - 43	0.0	0.0 - 0.0	6.0	2.1 - 14.0	0.8	0.2 - 2.3	3.4	1.2 - 8.7	0.0	0.0 - 0.0	9.7	3.4 - 22.6	0.13
6010108	16951	N TOE R	401.1	23.5	6 - 46	1.6	0.5 - 3.5	8.0	2.5 - 15.9	0.4	0.1 - 1.0	3.2	0.8 - 7.6	0.0	0.0 - 0.0	10.3	2.7 - 24.7	0.13
6010108	16952	CANE CR	79.6	18.5	6 - 48	1.0	0.3 - 2.6	4.7	1.5 - 12.6	0.6	0.2 - 2.2	3.2	0.9 - 9.4	0.0	0.0 - 0.0	8.9	2.8 - 27.2	0.13
6010108	16953	BIG ROCK CR	165.6	17.2	6 - 33	0.0	0.0 - 0.0	2.8	0.8 - 5.9	0.5	0.1 - 1.1	2.3	0.8 - 5.5	0.0	0.0 - 0.0	11.6	3.9 - 23.8	0.13
6010108	16954	N INDIAN CR	156.0	18.6	7 - 35	0.0	0.0 - 0.0	7.0	2.5 - 15.1	0.5	0.2 - 1.2	2.2	0.7 - 6.0	0.0	0.0 - 0.0	9.0	3.0 - 17.9	0.14
6010108	16955	BIG LIMESTONE C	3.9	63.9	21 - 134	0.0	0.0 - 0.0	2.9	1.1 - 6.4	15.3	4.6 - 40.9	35.9	12.6 - 90.5	0.0	0.0 - 0.0	9.9	3.7 - 22.6	0.14
6010108	16956	BIG LIMESTONE C	145.8	60.1	23 - 122	0.0	0.0 - 0.0	6.5	2.3 - 13.6	13.8	4.5 - 37.5	30.3	11.4 - 80.5	0.0	0.0 - 0.0	9.5	3.9 - 24.7	0.14
6010108	16957	CARSON CR	54.5	61.9	22 - 135	0.0	0.0 - 0.0	4.4	1.5 - 10.2	14.1	4.0 - 43.6	33.5	10.8 - 89.0	0.0	0.0 - 0.0	10.0	3.6 - 24.4	0.14

8-digit HUC	ID	Name	Area	Catchment Yield		Point sources		Developed Land		Manure		Agricultural Land		Phosphate Mines		Soil parent rock		Frac
				kg/km^2	90% CI	kg/km^2	90% CI	kg/km^2	90% CI	kg/km^2	90% CI	kg/km^2	90% CI	kg/km^2	90% CI	kg/km^2	90% CI	
6010108	16958	PIGEON CR	18.4	56.1	19 - 116	0.0	0.0 - 0.0	7.2	2.5 - 17.3	11.3	3.5 - 29.7	28.2	9.7 - 63.1	0.0	0.0 - 0.0	9.4	3.3 - 24.4	0.15
6010108	16959	LITTLE CHUCKY C	108.9	55.1	19 - 116	0.0	0.0 - 0.0	5.7	1.7 - 14.8	11.3	3.1 - 34.7	27.5	10.1 - 67.3	0.0	0.0 - 0.0	10.5	3.4 - 26.9	0.15
6010108	16960	LICK CR	139.0	76.9	24 - 182	0.0	0.0 - 0.0	5.3	1.6 - 11.6	17.1	4.9 - 59.8	41.9	12.3 - 117.9	0.0	0.0 - 0.0	12.5	4.0 - 31.8	0.15
6010108	16961	LICK CR	119.4	100.4	31 - 231	17.8	6.1 - 47.8	5.5	1.8 - 15.2	18.3	5.2 - 55.5	44.5	13.4 - 155.1	0.0	0.0 - 0.0	14.4	4.9 - 41.1	0.14
6010108	16962	HOOVER CR	114.7	57.5	20 - 129	0.0	0.0 - 0.0	7.8	2.3 - 18.5	11.0	3.5 - 31.2	27.0	9.8 - 68.7	0.0	0.0 - 0.0	11.8	3.7 - 31.1	0.13
6010108	16963	LICK CR	82.6	57.0	20 - 137	2.0	0.7 - 5.1	4.6	1.5 - 10.5	11.1	3.1 - 30.9	27.3	9.3 - 79.1	0.0	0.0 - 0.0	12.0	3.8 - 30.0	0.13
6010108	16964	LICK CR	39.9	49.1	14 - 125	0.0	0.0 - 0.0	5.1	1.5 - 11.5	9.8	2.6 - 28.4	23.5	7.4 - 59.4	0.0	0.0 - 0.0	10.7	3.1 - 29.7	0.12
6010108	16965	HORSE CR	147.8	48.7	14 - 106	0.0	0.0 - 0.0	5.4	1.7 - 12.5	9.6	2.6 - 27.7	23.0	6.7 - 55.2	0.0	0.0 - 0.0	10.6	2.6 - 25.5	0.12
6010108	16966	GAP CR	48.6	40.1	13 - 96	0.0	0.0 - 0.0	3.3	0.9 - 7.6	7.8	2.4 - 23.7	19.2	6.6 - 47.6	0.0	0.0 - 0.0	9.8	3.1 - 24.6	0.14
6010108	16967	BENT CR	127.5	55.8	21 - 125	0.0	0.0 - 0.0	4.9	1.7 - 11.2	12.3	4.2 - 38.0	28.7	10.2 - 74.4	0.0	0.0 - 0.0	9.9	3.9 - 26.7	0.15
6010108	16968	LONG CR	91.2	60.4	20 - 141	0.0	0.0 - 0.0	7.9	2.8 - 17.9	13.4	4.0 - 35.6	28.0	9.9 - 78.3	0.0	0.0 - 0.0	11.1	3.7 - 27.4	0.15
6010201	16969	TENNESSEE R	97.9	48.7	18 - 101	0.0	0.0 - 0.0	11.4	3.9 - 24.5	4.4	1.4 - 12.7	15.4	6.2 - 39.5	0.0	0.0 - 0.0	17.4	6.4 - 44.7	0.30
6010201	16970	PAINT ROCK CR	78.8	40.8	13 - 68	0.0	0.0 - 0.0	5.2	1.8 - 9.1	5.4	1.5 - 17.0	17.0	5.3 - 38.7	0.0	0.0 - 0.0	13.3	4.7 - 28.7	0.30
6010201	16971	TENNESSEE R	65.7	46.8	14 - 113	0.0	0.0 - 0.0	7.0	1.8 - 18.2	6.8	1.5 - 22.1	18.8	5.0 - 56.3	0.0	0.0 - 0.0	14.3	4.0 - 41.4	0.30
6010201	16972	POND CR	96.9	72.1	24 - 123	0.0	0.0 - 0.0	10.4	3.3 - 20.7	14.3	3.9 - 34.7	33.9	11.6 - 76.4	0.0	0.0 - 0.0	13.6	4.5 - 26.4	0.30
6010201	16973	TENNESSEE R	69.2	54.5	16 - 104	0.0	0.0 - 0.0	13.8	4.4 - 28.2	8.1	2.0 - 23.5	18.9	5.9 - 42.5	0.0	0.0 - 0.0	13.8	4.1 - 36.8	0.30
6010201	16974	SWEETWATER CR	162.7	75.2	20 - 163	1.1	0.3 - 2.9	15.0	4.5 - 33.2	13.3	3.7 - 31.4	32.3	9.7 - 85.0	0.0	0.0 - 0.0	13.4	4.2 - 31.6	0.30
6010201	16975	TENNESSEE R	92.2	269.2	98 - 539	200.2	73.9 - 448.4	24.9	7.9 - 51.5	8.9	2.6 - 25.9	20.6	6.9 - 46.7	0.0	0.0 - 0.0	14.5	5.4 - 35.3	0.30
6010201	16977	TENNESSEE R	1.0	80.4	27 - 149	0.0	0.0 - 0.0	50.5	16.7 - 96.7	3.3	1.0 - 9.4	7.2	2.5 - 16.5	0.0	0.0 - 0.0	19.4	6.9 - 44.0	0.30
6010201	16978	TENNESSEE R	7.3	56.5	16 - 135	0.0	0.0 - 0.0	17.1	4.5 - 39.3	5.0	1.3 - 15.5	18.9	5.9 - 49.5	0.0	0.0 - 0.0	15.4	4.4 - 43.6	0.30
6010201	16979	LITTLE R	223.5	76.0	24 - 155	0.0	0.0 - 0.0	34.1	10.6 - 76.0	6.7	1.9 - 20.1	21.1	7.1 - 56.2	0.0	0.0 - 0.0	14.1	4.3 - 32.5	0.26
6010201	16980	LITTLE R	17.9	82.0	30 - 159	0.0	0.0 - 0.0	14.4	5.1 - 29.3	12.9	4.1 - 34.7	40.5	12.5 - 101.6	0.0	0.0 - 0.0	14.2	4.9 - 29.7	0.25
6010201	16981	CROOKED CR	83.3	49.8	16 - 100	0.0	0.0 - 0.0	8.1	3.0 - 16.4	7.2	2.1 - 20.3	22.5	8.4 - 58.4	0.0	0.0 - 0.0	12.1	4.1 - 28.4	0.25
6010201	16982	LITTLE R	7.4	81.3	25 - 184	0.0	0.0 - 0.0	10.4	3.3 - 28.2	13.8	4.0 - 41.7	43.0	12.9 - 108.0	0.0	0.0 - 0.0	14.1	4.8 - 33.1	0.25
6010201	16983	LITTLE R	55.1	28.5	11 - 59	0.0	0.0 - 0.0	6.8	2.6 - 15.3	1.9	0.5 - 5.3	5.8	2.2 - 15.8	0.0	0.0 - 0.0	14.0	5.2 - 30.2	0.25
6010201	16984	HESSE CR	69.6	19.7	7 - 45	0.0	0.0 - 0.0	4.5	1.5 - 10.4	0.7	0.2 - 1.7	2.1	0.7 - 4.8	0.0	0.0 - 0.0	12.4	4.1 - 29.9	0.24
6010201	16985	LITTLE R	97.7	28.9	11 - 52	0.0	0.0 - 0.0	10.2	3.7 - 20.2	1.6	0.5 - 3.8	4.9	1.6 - 12.2	0.0	0.0 - 0.0	12.2	4.4 - 27.1	0.24
6010201	16986	ELLEJOY CR	101.8	51.5	19 - 111	0.0	0.0 - 0.0	5.1	1.7 - 11.4	7.5	2.1 - 21.0	23.1	8.1 - 57.0	0.0	0.0 - 0.0	15.9	6.0 - 35.7	0.25
6010201	16987	NAILS CR	47.2	62.7	23 - 125	0.0	0.0 - 0.0	11.5	4.1 - 25.6	8.9	2.8 - 22.1	27.8	9.9 - 69.3	0.0	0.0 - 0.0	14.6	5.6 - 30.8	0.25
6010201	16988	TENNESSEE R	240.3	599.3	223 - 1221	504.6	184.2 - 1096.3	77.2	27.3 - 139.4	1.2	0.4 - 2.9	3.6	1.3 - 7.9	0.0	0.0 - 0.0	12.7	4.7 - 28.8	0.26
6010201	16989	TOWN CR	25.7	90.4	29 - 192	0.0	0.0 - 0.0	53.7	16.4 - 123.0	6.9	2.3 - 20.7	16.1	5.3 - 37.2	0.0	0.0 - 0.0	13.7	4.4 - 32.5	0.30
6010201	16990	PINEY CR	1.1	8.9	3 - 19	0.0	0.0 - 0.0	0.0	0.0 - 0.0	0.0	0.0 - 0.0	0.0	0.0 - 0.0	0.0	0.0 - 0.0	8.9	2.7 - 18.8	0.30
6010201	16992	PINEY CR	58.6	30.3	12 - 70	0.0	0.0 - 0.0	8.5	3.0 - 19.7	2.3	0.7 - 6.1	4.3	1.3 - 11.4	0.0	0.0 - 0.0	15.2	6.2 - 33.3	0.29
6010201	16993	MAMMY'S CR	37.3	35.0	11 - 63	0.0	0.0 - 0.0	10.0	3.0 - 19.0	2.7	0.8 - 6.7	5.2	1.6 - 12.4	0.0	0.0 - 0.0	17.1	5.3 - 34.9	0.29
6010201	16994	FALL CR	57.4	26.6	9 - 46	0.0	0.0 - 0.0	6.6	2.4 - 12.9	1.0	0.3 - 2.4	1.6	0.6 - 4.1	0.0	0.0 - 0.0	17.4	6.2 - 34.1	0.30
6010201	16995	WHITES CR	21.5	26.0	8 - 65	0.0	0.0 - 0.0	4.4	1.3 - 10.3	2.5	0.8 - 7.4	4.1	1.3 - 10.9	0.0	0.0 - 0.0	15.0	4.6 - 36.2	0.30
6010201	16996	ALLOWAY CR	79.4	31.9	12 - 74	0.0	0.0 - 0.0	3.0	0.9 - 6.7	3.3	0.9 - 8.2	9.9	3.3 - 26.1	0.0	0.0 - 0.0	15.7	5.7 - 40.3	0.28
6010201	16997	WHITES CR	44.0	23.1	8 - 55	0.0	0.0 - 0.0	2.3	0.8 - 4.8	1.4	0.4 - 4.0	3.8	1.3 - 10.0	0.0	0.0 - 0.0	15.6	5.6 - 38.4	0.28
6010201	16998	PINEY R	24.3	29.7	11 - 74	0.0	0.0 - 0.0	5.2	1.8 - 12.9	2.7	0.9 - 8.3	9.1	3.1 - 25.4	0.0	0.0 - 0.0	12.7	4.8 - 36.3	0.30

8-digit HUC	ID	Name	Area	Catchment Yield		Point sources		Developed Land		Manure		Agricultural Land		Phosphate Mines		Soil parent rock		Frac
				kg/km^2	90% CI	kg/km^2	90% CI	kg/km^2	90% CI	kg/km^2	90% CI	kg/km^2	90% CI	kg/km^2	90% CI	kg/km^2	90% CI	
6010201	16999	PINEY R	24.1	20.9	7 - 50	0.0	0.0 - 0.0	2.8	0.9 - 7.8	1.1	0.3 - 3.4	1.8	0.7 - 5.1	0.0	0.0 - 0.0	15.1	5.3 - 40.4	0.29
6010201	17000	SOAK CR	33.6	20.4	7 - 39	0.0	0.0 - 0.0	3.1	1.0 - 5.6	1.8	0.5 - 4.5	0.4	0.1 - 1.1	0.0	0.0 - 0.0	15.1	4.8 - 30.6	0.29
6010201	17001	PINEY CR	14.7	28.6	8 - 53	0.0	0.0 - 0.0	8.2	2.1 - 16.1	3.2	0.8 - 7.5	4.3	1.2 - 11.0	0.0	0.0 - 0.0	12.9	4.5 - 27.6	0.30
6010201	17002	DUSKIN CR	25.7	30.8	11 - 57	0.0	0.0 - 0.0	9.1	3.1 - 17.2	4.2	1.3 - 9.7	2.5	0.8 - 5.4	0.0	0.0 - 0.0	15.1	5.5 - 31.7	0.29
6010201	17003	PINEY CR	27.9	30.0	10 - 74	0.0	0.0 - 0.0	6.9	2.0 - 14.8	3.8	1.0 - 10.0	4.1	1.4 - 12.4	0.0	0.0 - 0.0	15.1	5.6 - 42.8	0.29
6010201	17004	MOCCASIN CR	41.4	28.2	13 - 76	0.0	0.0 - 0.0	5.7	2.5 - 13.9	3.3	1.2 - 11.2	3.9	1.7 - 10.8	0.0	0.0 - 0.0	15.3	6.6 - 40.0	0.28
6010201	17005	PINEY CR	48.8	29.0	8 - 71	0.0	0.0 - 0.0	4.4	1.3 - 13.4	2.5	0.7 - 8.1	7.0	2.0 - 22.3	0.0	0.0 - 0.0	15.0	4.6 - 34.4	0.28
6010202	17006	NANTAHALA R	27.2	16.7	5 - 37	0.0	0.0 - 0.0	3.4	1.0 - 10.3	0.1	0.0 - 0.3	0.5	0.1 - 1.5	0.0	0.0 - 0.0	12.7	3.9 - 29.1	0.14
6010202	17007	WHITE OAK CR	65.6	19.7	9 - 40	0.1	0.0 - 0.2	5.5	2.1 - 12.7	0.2	0.1 - 0.7	1.2	0.4 - 3.0	0.0	0.0 - 0.0	12.6	5.2 - 27.5	0.14
6010202	17008	BURNINGTOWN CI	68.7	20.8	7 - 43	0.0	0.0 - 0.0	3.2	0.9 - 6.8	0.4	0.1 - 1.1	1.8	0.5 - 5.4	0.0	0.0 - 0.0	15.3	5.3 - 35.5	0.14
6010202	17009	LITTLE TENNESSE	40.0	27.1	10 - 54	0.0	0.0 - 0.0	7.0	2.2 - 15.2	1.0	0.3 - 2.8	4.7	1.6 - 10.6	0.0	0.0 - 0.0	14.5	4.6 - 34.0	0.14
6010202	17010	LITTLE TENNESSE	54.5	39.3	12 - 100	0.0	0.0 - 0.0	9.1	2.7 - 22.3	1.9	0.5 - 5.7	8.9	2.5 - 27.1	0.0	0.0 - 0.0	19.4	6.5 - 50.3	0.14
6010202	17011	LITTLE TENNESSE	17.9	168.1	52 - 383	97.7	29.6 - 240.4	39.1	12.3 - 85.0	1.9	0.6 - 4.7	9.0	2.7 - 23.3	0.0	0.0 - 0.0	20.4	6.3 - 46.8	0.13
6010202	17012	LITTLE TENNESSE	3.3	58.5	20 - 114	0.0	0.0 - 0.0	21.7	6.4 - 45.9	2.5	0.8 - 6.5	13.2	4.3 - 33.9	0.0	0.0 - 0.0	21.1	7.1 - 52.1	0.13
6010202	17013	CARTOOGECHAUI	52.2	46.7	15 - 117	0.0	0.0 - 0.0	16.6	5.9 - 40.6	1.9	0.5 - 5.4	9.3	2.8 - 27.9	0.0	0.0 - 0.0	19.0	6.9 - 53.1	0.13
6010202	17014	CARTOOGECHAUI	35.9	27.6	10 - 56	0.0	0.0 - 0.0	5.0	1.6 - 11.1	0.3	0.1 - 0.8	1.4	0.5 - 3.4	0.0	0.0 - 0.0	20.9	7.6 - 48.2	0.12
6010202	17015	JONES CR	60.5	40.0	13 - 84	0.0	0.0 - 0.0	9.8	3.2 - 19.3	0.9	0.3 - 1.9	4.4	1.4 - 13.0	0.0	0.0 - 0.0	24.9	8.3 - 55.6	0.12
6010202	17016	LITTLE TENNESSE	151.6	40.7	17 - 97	0.0	0.0 - 0.1	10.1	3.4 - 23.7	1.4	0.5 - 4.3	6.5	2.2 - 20.1	0.0	0.0 - 0.0	22.6	8.9 - 50.7	0.13
6010202	17017	LITTLE TENNESSE	175.6	70.9	23 - 149	0.5	0.2 - 1.2	17.3	5.9 - 38.5	6.9	2.2 - 16.1	9.1	2.5 - 23.1	0.0	0.0 - 0.0	37.0	13.9 - 84.4	0.13
6010202	17018	MIDDLE CR	34.7	43.6	17 - 102	0.0	0.0 - 0.0	8.6	2.9 - 20.7	1.5	0.4 - 4.4	5.9	2.1 - 17.7	0.0	0.0 - 0.0	27.6	10.2 - 67.9	0.13
6010202	17019	GULLASAJA R	102.7	31.5	13 - 82	0.3	0.1 - 0.8	9.4	3.4 - 25.6	0.7	0.2 - 2.4	3.6	1.3 - 10.4	0.0	0.0 - 0.0	17.4	6.8 - 45.0	0.13
6010202	17020	GULLASAJA R	92.8	74.1	26 - 149	4.4	1.6 - 10.2	33.1	11.3 - 68.0	0.4	0.1 - 1.1	2.1	0.6 - 5.6	0.0	0.0 - 0.0	34.1	13.1 - 81.7	0.13
6010202	17021	WALNUT CR	45.0	30.2	10 - 82	0.0	0.0 - 0.0	5.0	1.5 - 12.0	0.1	0.0 - 0.5	0.7	0.2 - 2.7	0.0	0.0 - 0.0	24.3	8.1 - 63.2	0.13
6010202	17022	WATAUGA CR	44.4	38.8	10 - 74	0.0	0.0 - 0.0	11.8	2.9 - 23.8	1.4	0.3 - 3.6	6.6	1.8 - 16.0	0.0	0.0 - 0.0	19.0	5.0 - 41.6	0.13
6010202	17023	COWEE CR	67.4	22.7	8 - 39	0.0	0.0 - 0.0	4.2	1.4 - 8.2	0.5	0.2 - 1.3	2.2	0.8 - 5.3	0.0	0.0 - 0.0	15.8	5.5 - 32.1	0.14
6010203	17024	TUCKASEGEE R	31.9	21.6	6 - 41	0.1	0.0 - 0.2	11.7	3.1 - 22.3	0.3	0.1 - 1.0	2.1	0.6 - 5.8	0.0	0.0 - 0.0	7.4	2.4 - 17.7	0.14
6010203	17025	TUCKASEGEE R	1.9	29.3	10 - 68	0.0	0.0 - 0.0	16.0	5.2 - 38.0	0.6	0.2 - 1.6	4.5	1.5 - 12.3	0.0	0.0 - 0.0	8.1	2.6 - 23.6	0.14
6010203	17026	TUCKASEGEE R	5.9	36.7	11 - 66	0.0	0.0 - 0.0	24.8	6.9 - 47.3	0.2	0.1 - 0.5	1.4	0.3 - 3.1	0.0	0.0 - 0.0	10.2	3.0 - 23.5	0.14
6010203	17027	CONNELLY CR	35.4	14.7	5 - 35	0.3	0.1 - 0.8	4.2	1.6 - 10.1	0.1	0.0 - 0.4	0.9	0.3 - 2.5	0.0	0.0 - 0.0	9.2	3.5 - 23.9	0.14
6010203	17028	TUCKASEGEE R	101.7	20.7	8 - 51	0.0	0.0 - 0.1	9.8	3.2 - 24.3	0.2	0.1 - 0.7	2.2	0.8 - 6.7	0.0	0.0 - 0.0	8.4	3.4 - 20.7	0.14
6010203	17029	TUCKASEGEE R	6.3	393.9	126 - 876	356.1	112.7 - 825.8	19.3	6.5 - 49.4	0.3	0.1 - 0.8	2.3	0.7 - 6.5	0.0	0.0 - 0.0	16.0	5.5 - 41.4	0.14
6010203	17030	SAVANNAH CR	104.9	26.3	9 - 51	0.0	0.0 - 0.0	7.0	2.7 - 12.7	0.2	0.1 - 0.7	2.0	0.8 - 4.4	0.0	0.0 - 0.0	17.1	5.8 - 35.7	0.13
6010203	17031	TUCKASEGEE R	36.0	34.2	11 - 70	0.0	0.0 - 0.0	14.2	4.5 - 29.3	0.4	0.1 - 1.2	4.0	1.3 - 10.3	0.0	0.0 - 0.0	15.5	5.2 - 36.7	0.13
6010203	17032	CULLOWHEE CR	60.5	25.7	9 - 58	0.0	0.0 - 0.0	7.9	2.4 - 17.2	0.2	0.1 - 0.6	1.9	0.6 - 4.8	0.0	0.0 - 0.0	15.7	5.9 - 36.6	0.13
6010203	17033	TUCKASEGEE R	22.5	25.1	9 - 61	0.0	0.0 - 0.0	8.3	3.0 - 20.4	0.2	0.1 - 0.5	1.7	0.6 - 4.8	0.0	0.0 - 0.0	14.9	5.3 - 38.2	0.13
6010203	17034	TUCKASEGEE R	12.9	24.0	9 - 54	0.0	0.0 - 0.0	6.3	2.2 - 15.6	0.4	0.1 - 1.3	4.3	1.6 - 11.5	0.0	0.0 - 0.0	13.0	4.7 - 33.5	0.13
6010203	17035	TUCKASEGEE R, V	151.6	14.1	5 - 31	0.1	0.0 - 0.3	3.9	1.2 - 8.4	0.1	0.0 - 0.4	1.4	0.4 - 3.8	0.0	0.0 - 0.0	8.5	2.9 - 19.7	0.13
6010203	17036	TUCKASEGEE R	105.3	26.8	10 - 59	0.0	0.0 - 0.0	4.5	1.6 - 9.9	0.2	0.1 - 0.5	1.9	0.7 - 4.4	0.0	0.0 - 0.0	20.2	8.9 - 43.9	0.13
6010203	17037	TUCKASEGEE R	5.3	41.5	14 - 110	0.0	0.0 - 0.0	4.5	1.4 - 11.5	0.1	0.0 - 0.3	1.3	0.5 - 4.1	0.0	0.0 - 0.0	35.5	11.5 - 90.6	0.11

8-digit HUC	ID	Name	Area	Catchment Yield		Point sources		Developed Land		Manure		Agricultural Land		Phosphate Mines		Soil parent rock		Frac
				kg/km^2	90% CI	kg/km^2	90% CI	kg/km^2	90% CI	kg/km^2	90% CI	kg/km^2	90% CI	kg/km^2	90% CI	kg/km^2	90% CI	
6010203	17038	TUCKASEGEE R	31.1	49.4	17 - 102	0.0	0.0 - 0.0	6.5	1.9 - 15.5	0.2	0.0 - 0.4	1.4	0.4 - 3.8	0.0	0.0 - 0.0	41.4	15.2 - 94.2	0.11
6010203	17039	TANASEE CR	32.6	44.5	13 - 118	0.0	0.0 - 0.0	6.5	2.0 - 16.0	0.5	0.2 - 1.5	4.3	1.1 - 14.3	0.0	0.0 - 0.0	33.1	11.0 - 90.1	0.11
6010203	17040	WOLF CR	41.9	30.0	10 - 61	0.0	0.0 - 0.0	3.9	1.3 - 8.5	0.2	0.0 - 0.4	1.5	0.5 - 3.9	0.0	0.0 - 0.0	24.5	7.9 - 50.6	0.11
6010203	17041	TUCKASEGEE R, C	133.0	21.2	7 - 45	0.0	0.0 - 0.0	3.2	1.1 - 6.7	0.1	0.0 - 0.3	1.1	0.3 - 2.8	0.0	0.0 - 0.0	16.8	5.7 - 37.6	0.13
6010203	17042	DARK RIDGE CR	152.4	27.5	9 - 51	1.3	0.4 - 2.6	11.3	3.5 - 22.2	0.1	0.0 - 0.3	1.4	0.4 - 3.6	0.0	0.0 - 0.0	13.3	4.4 - 30.6	0.14
6010203	17043	OCONALUFTEE R	40.0	82.1	29 - 178	54.6	19.8 - 126.3	13.9	4.3 - 28.9	0.4	0.1 - 1.1	3.4	1.0 - 8.2	0.0	0.0 - 0.0	9.9	3.9 - 20.8	0.14
6010203	17044	SOCO CR	100.0	18.4	7 - 33	0.0	0.0 - 0.0	8.2	2.4 - 15.3	0.1	0.0 - 0.1	0.5	0.2 - 1.1	0.0	0.0 - 0.0	9.6	3.4 - 20.5	0.14
6010203	17045	OCONALUFTEE R	16.4	28.5	8 - 66	0.0	0.0 - 0.0	15.6	4.6 - 40.1	0.2	0.1 - 0.8	1.9	0.6 - 5.5	0.0	0.0 - 0.0	10.8	3.4 - 27.2	0.14
6010203	17046	OCONALUFTEE R,	70.6	14.1	5 - 28	0.0	0.0 - 0.0	4.3	1.5 - 9.0	0.0	0.0 - 0.1	0.2	0.1 - 0.5	0.0	0.0 - 0.0	9.5	3.8 - 21.4	0.13
6010203	17047	OCONALUFTEE R,	63.2	11.0	4 - 26	0.0	0.0 - 0.0	0.7	0.2 - 1.7	0.0	0.0 - 0.0	0.0	0.0 - 0.1	0.0	0.0 - 0.0	10.3	3.3 - 25.3	0.13
6010203	17048	OCONALUFTEE R,	59.7	14.7	5 - 32	0.0	0.0 - 0.0	1.3	0.4 - 2.7	0.0	0.0 - 0.1	0.2	0.1 - 0.5	0.0	0.0 - 0.0	13.3	4.9 - 31.4	0.13
6010203	17049	OCONALUFTEE R	135.7	13.5	5 - 28	0.1	0.0 - 0.2	3.4	1.2 - 7.1	0.0	0.0 - 0.0	0.1	0.0 - 0.3	0.0	0.0 - 0.0	9.8	3.9 - 22.1	0.13
6010203	17050	COOPER CR	20.1	12.2	5 - 30	0.0	0.0 - 0.0	2.7	0.9 - 7.4	0.1	0.0 - 0.2	0.5	0.2 - 1.8	0.0	0.0 - 0.0	8.9	3.1 - 21.9	0.14
6010203	17051	DEEP CR	12.5	27.7	9 - 43	0.0	0.0 - 0.0	14.6	4.4 - 26.8	0.4	0.1 - 0.9	3.1	1.0 - 6.9	0.0	0.0 - 0.0	9.6	3.4 - 17.2	0.14
6010203	17052	INDIAN CR	21.6	10.2	3 - 22	0.0	0.0 - 0.0	1.7	0.5 - 3.4	0.0	0.0 - 0.0	0.0	0.0 - 0.0	0.0	0.0 - 0.0	8.5	2.9 - 19.3	0.13
6010203	17053	DEEP CR	80.7	10.4	3 - 28	0.0	0.0 - 0.0	1.1	0.3 - 2.8	0.0	0.0 - 0.0	0.0	0.0 - 0.0	0.0	0.0 - 0.0	9.4	2.7 - 25.7	0.13
6010204	17054	LITTLE TENNESSE	86.8	39.7	14 - 72	0.0	0.0 - 0.0	12.1	4.3 - 26.5	5.2	1.5 - 13.6	11.8	4.0 - 30.3	0.0	0.0 - 0.0	10.6	3.9 - 26.3	0.30
6010204	17055	FORK CR	123.0	71.6	28 - 159	0.0	0.0 - 0.0	9.9	3.5 - 20.9	13.7	4.6 - 35.8	34.1	12.4 - 90.9	0.0	0.0 - 0.0	13.9	5.3 - 34.6	0.22
6010204	17056	LITTLE TENNESSE	4.5	56.1	20 - 135	0.0	0.0 - 0.0	25.3	7.9 - 60.5	3.3	1.0 - 10.9	8.1	2.4 - 19.7	0.0	0.0 - 0.0	19.4	6.1 - 55.5	0.22
6010204	17057	BAT CR	80.3	74.9	25 - 158	12.9	4.2 - 27.3	13.9	4.9 - 32.8	9.1	2.7 - 26.7	23.1	7.2 - 57.5	0.0	0.0 - 0.0	16.0	5.1 - 38.1	0.22
6010204	17058	LITTLE TENNESSE	2.6	32.7	10 - 81	0.0	0.0 - 0.0	1.0	0.3 - 2.1	3.2	0.8 - 8.6	7.6	1.9 - 20.6	0.0	0.0 - 0.0	20.9	6.7 - 52.4	0.22
6010204	17059	LITTLE TENNESSE	63.1	72.0	20 - 144	5.9	2.0 - 13.6	14.2	3.8 - 27.8	10.7	2.5 - 27.4	26.0	7.1 - 62.7	0.0	0.0 - 0.0	15.1	4.4 - 31.9	0.22
6010204	17060	TELICO R	12.7	33.5	13 - 85	0.0	0.0 - 0.0	3.3	1.1 - 8.4	2.8	0.9 - 8.5	6.7	2.6 - 21.4	0.0	0.0 - 0.0	20.6	8.0 - 54.1	0.21
6010204	17061	TELICO R	218.9	43.7	15 - 100	1.5	0.5 - 3.1	6.4	2.0 - 14.7	4.9	1.4 - 19.2	12.1	4.0 - 38.8	0.0	0.0 - 0.0	18.9	6.3 - 50.0	0.21
6010204	17062	TELICO R	28.6	22.3	6 - 41	0.0	0.0 - 0.0	7.1	2.1 - 13.9	0.4	0.1 - 1.0	0.8	0.3 - 2.1	0.0	0.0 - 0.0	14.0	4.1 - 31.5	0.20
6010204	17063	*A	30.6	17.0	5 - 41	0.0	0.0 - 0.0	4.3	1.3 - 10.3	0.2	0.1 - 0.7	0.1	0.0 - 0.4	0.0	0.0 - 0.0	12.4	3.8 - 29.5	0.19
6010204	17064	TELICO R	54.8	18.1	6 - 39	0.0	0.0 - 0.0	5.4	1.6 - 11.9	0.1	0.0 - 0.4	0.3	0.1 - 0.9	0.0	0.0 - 0.0	12.2	3.9 - 27.4	0.19
6010204	17065	BALD R	56.7	13.4	5 - 28	0.0	0.0 - 0.0	0.9	0.3 - 1.7	0.0	0.0 - 0.0	0.0	0.0 - 0.0	0.0	0.0 - 0.0	12.5	4.5 - 27.1	0.19
6010204	17066	TELICO R	133.4	17.3	6 - 33	0.0	0.0 - 0.0	2.1	0.6 - 4.5	0.0	0.0 - 0.0	0.0	0.0 - 0.0	0.0	0.0 - 0.0	15.2	5.4 - 29.7	0.19
6010204	17067	SMOKEY RUN	23.4	22.8	7 - 42	0.0	0.0 - 0.0	7.7	2.6 - 13.4	0.5	0.2 - 1.2	1.3	0.4 - 2.7	0.0	0.0 - 0.0	13.4	4.7 - 28.0	0.20
6010204	17068	BALLPLAY CR	69.2	25.5	9 - 76	0.0	0.0 - 0.0	3.9	1.3 - 10.3	1.8	0.5 - 6.0	4.1	1.3 - 11.2	0.0	0.0 - 0.0	15.7	5.6 - 45.6	0.21
6010204	17070	LITTLE TENNESSE	96.9	28.5	10 - 58	0.0	0.0 - 0.0	5.7	1.9 - 12.4	2.2	0.6 - 5.4	5.4	1.6 - 13.7	0.0	0.0 - 0.0	15.2	5.6 - 32.6	0.21
6010204	17071	CITICO R	184.7	12.7	4 - 26	0.0	0.0 - 0.0	2.4	0.8 - 5.2	0.1	0.0 - 0.4	0.2	0.1 - 0.5	0.0	0.0 - 0.0	10.0	3.6 - 22.7	0.21
6010204	17072	LITTLE TENNESSE	53.9	19.5	7 - 44	0.0	0.0 - 0.0	5.4	1.7 - 13.3	0.5	0.1 - 1.4	1.0	0.3 - 2.6	0.0	0.0 - 0.0	12.7	4.1 - 33.0	0.21
6010204	17073	LITTLE TENNESSE	150.4	13.3	4 - 31	0.0	0.0 - 0.0	2.9	0.8 - 6.6	0.1	0.0 - 0.3	0.1	0.0 - 0.4	0.0	0.0 - 0.0	10.1	2.8 - 25.8	0.20
6010204	17074	CHEOAH R	25.7	15.5	5 - 29	0.0	0.0 - 0.0	7.3	2.4 - 16.3	0.1	0.0 - 0.2	0.0	0.0 - 0.1	0.0	0.0 - 0.0	8.2	2.6 - 17.3	0.20
6010204	17075	DEEP CR	12.6	13.9	5 - 31	0.0	0.0 - 0.0	3.4	1.2 - 7.1	0.0	0.0 - 0.0	0.0	0.0 - 0.0	0.0	0.0 - 0.0	10.5	3.7 - 24.8	0.20
6010204	17076	CHEOAH R	2.7	18.2	5 - 38	0.0	0.0 - 0.0	11.7	3.0 - 24.5	0.1	0.0 - 0.3	0.0	0.0 - 0.0	0.0	0.0 - 0.0	6.4	2.0 - 15.4	0.20
6010204	17077	CHEOAH R	30.0	15.7	5 - 34	0.0	0.0 - 0.0	6.4	2.0 - 13.8	0.2	0.1 - 0.6	0.6	0.2 - 1.5	0.0	0.0 - 0.0	8.4	3.1 - 21.0	0.20

8-digit HUC	ID	Name	Area	Catchment Yield		Point sources		Developed Land		Manure		Agricultural Land		Phosphate Mines		Soil parent rock		Frac
				kg/km^2	90% CI	kg/km^2	90% CI	kg/km^2	90% CI	kg/km^2	90% CI	kg/km^2	90% CI	kg/km^2	90% CI	kg/km^2	90% CI	
6010204	17078	TULULAH CR	103.6	22.0	7 - 42	0.0	0.0 - 0.0	9.4	2.6 - 20.6	0.4	0.1 - 1.0	1.2	0.4 - 2.9	0.0	0.0 - 0.0	11.0	3.9 - 24.7	0.11
6010204	17079	SWEETWATER CR	37.3	19.2	7 - 38	0.0	0.0 - 0.0	8.4	2.6 - 16.7	0.5	0.1 - 1.4	1.8	0.5 - 4.4	0.0	0.0 - 0.0	8.6	3.2 - 18.5	0.11
6010204	17080	YELLOW CR	35.5	13.9	5 - 35	0.0	0.0 - 0.0	4.5	1.4 - 11.3	0.2	0.1 - 0.7	0.7	0.2 - 2.3	0.0	0.0 - 0.0	8.5	3.0 - 25.7	0.20
6010204	17081	LITTLE TENNESSE	97.0	13.7	5 - 27	1.0	0.4 - 2.6	4.8	1.8 - 10.9	0.0	0.0 - 0.1	0.1	0.0 - 0.2	0.0	0.0 - 0.0	7.7	2.6 - 18.1	0.20
6010204	17082	ABRAMS CR	1.0	28.7	9 - 53	0.0	0.0 - 0.0	14.1	4.9 - 28.4	0.0	0.0 - 0.0	0.0	0.0 - 0.0	0.0	0.0 - 0.0	14.6	4.8 - 30.3	0.20
6010204	17083	PANTHER CR	27.7	15.5	6 - 41	0.0	0.0 - 0.0	0.5	0.2 - 1.0	0.0	0.0 - 0.0	0.0	0.0 - 0.0	0.0	0.0 - 0.0	15.0	5.4 - 40.1	0.20
6010204	17084	ABRAMS CR	104.5	17.7	7 - 40	0.0	0.0 - 0.0	3.2	1.2 - 6.8	0.2	0.1 - 0.7	0.7	0.2 - 2.1	0.0	0.0 - 0.0	13.6	5.6 - 35.1	0.20
6010204	17085	MILL CR	43.5	18.4	6 - 47	0.0	0.0 - 0.0	0.6	0.2 - 1.5	0.2	0.1 - 0.6	0.6	0.2 - 1.5	0.0	0.0 - 0.0	17.0	5.9 - 44.1	0.20
6010204	17086	ABRAMS CR	49.9	32.6	11 - 83	0.0	0.0 - 0.0	3.8	1.2 - 8.7	2.7	0.8 - 7.0	8.9	2.7 - 26.4	0.0	0.0 - 0.0	17.2	5.8 - 40.5	0.20
6010204	17087	NINEMILE CR	155.8	49.1	15 - 91	0.0	0.0 - 0.0	7.0	2.0 - 13.4	7.2	2.0 - 19.9	22.0	7.3 - 55.2	0.0	0.0 - 0.0	13.0	4.2 - 26.1	0.21
6010204	17088	BAKER CR	114.2	82.3	28 - 173	0.0	0.0 - 0.0	9.1	3.1 - 19.1	15.1	4.8 - 37.6	43.1	13.7 - 101.7	0.0	0.0 - 0.0	15.0	4.7 - 37.8	0.22
6010204	17089	TELICO R	20.8	61.0	20 - 130	0.0	0.0 - 0.0	6.9	2.4 - 15.7	10.4	2.9 - 37.9	27.0	9.1 - 74.6	0.0	0.0 - 0.0	16.7	4.9 - 40.7	0.21
6010204	17090	NOTCHY CR	88.3	66.6	24 - 163	0.0	0.0 - 0.0	6.5	2.3 - 16.8	11.8	3.4 - 38.6	29.9	9.9 - 88.0	0.0	0.0 - 0.0	18.5	6.2 - 51.7	0.21
6010205	17091	BIG WAR CR	123.9	25.7	7 - 52	0.0	0.0 - 0.0	6.2	1.9 - 12.6	4.6	1.5 - 12.5	5.0	1.4 - 12.9	0.0	0.0 - 0.0	10.0	2.8 - 22.7	0.11
6010205	17092	CLINCH R	250.8	23.6	8 - 53	1.3	0.5 - 2.8	7.4	2.6 - 17.3	3.0	1.0 - 9.3	2.8	1.0 - 6.9	0.0	0.0 - 0.0	9.1	3.0 - 22.8	0.11
6010205	17093	CLINCH R	39.6	28.2	10 - 57	0.0	0.0 - 0.0	6.9	2.4 - 14.3	4.2	1.1 - 12.7	3.0	1.0 - 7.2	0.0	0.0 - 0.0	14.2	4.8 - 32.0	0.11
6010205	17094	CLINCH R	149.1	24.3	9 - 55	0.0	0.0 - 0.0	5.6	2.1 - 12.0	3.0	1.0 - 6.8	5.2	1.9 - 13.4	0.0	0.0 - 0.0	10.6	3.6 - 27.1	0.11
6010205	17095	COPPER CR	346.2	39.5	14 - 100	0.0	0.0 - 0.0	4.9	1.7 - 13.5	6.4	1.8 - 23.8	18.1	6.8 - 52.4	0.0	0.0 - 0.0	10.1	3.3 - 32.8	0.10
6010205	17096	CLINCH R	2.8	25.7	8 - 53	0.0	0.0 - 0.0	8.3	2.6 - 17.9	1.5	0.5 - 4.4	5.0	1.6 - 13.4	0.0	0.0 - 0.0	10.9	3.4 - 28.1	0.10
6010205	17097	CLINCH R	72.8	35.5	13 - 69	0.0	0.0 - 0.0	5.1	1.7 - 10.7	4.8	1.4 - 12.4	13.8	4.3 - 37.1	0.0	0.0 - 0.0	11.8	4.1 - 27.4	0.10
6010205	17098	CLINCH R	25.4	24.3	7 - 45	0.0	0.0 - 0.0	3.1	1.0 - 6.3	2.6	0.7 - 6.5	7.1	2.1 - 16.6	0.0	0.0 - 0.0	11.5	3.2 - 22.2	0.10
6010205	17099	CLINCH R	153.7	23.7	8 - 48	0.0	0.0 - 0.0	4.4	1.5 - 10.1	2.6	0.7 - 6.8	6.7	2.4 - 17.7	0.0	0.0 - 0.0	10.0	3.4 - 25.4	0.10
6010205	17100	CLINCH R	15.1	13.2	5 - 23	0.0	0.0 - 0.0	3.5	1.2 - 6.2	1.4	0.4 - 3.7	2.5	0.8 - 4.9	0.0	0.0 - 0.0	5.7	1.9 - 12.0	0.10
6010205	17101	CLINCH R	110.3	22.7	6 - 40	0.0	0.0 - 0.0	6.9	1.8 - 15.7	2.7	0.7 - 6.8	5.9	1.9 - 14.3	0.0	0.0 - 0.0	7.2	2.0 - 18.2	0.10
6010205	17102	CLINCH R	63.5	34.0	11 - 69	0.0	0.0 - 0.0	4.7	1.3 - 9.8	5.9	1.7 - 13.9	15.2	4.7 - 34.2	0.0	0.0 - 0.0	8.3	2.5 - 18.6	0.10
6010205	17103	CLINCH R	26.1	28.7	8 - 65	0.0	0.0 - 0.0	4.2	1.2 - 8.7	4.7	1.2 - 14.0	12.4	3.5 - 32.2	0.0	0.0 - 0.0	7.3	2.1 - 19.0	0.10
6010205	17104	BIG CEDAR CR	234.6	41.4	12 - 71	2.2	0.7 - 4.1	6.3	2.0 - 12.6	6.4	1.7 - 15.1	16.9	5.3 - 40.2	0.0	0.0 - 0.0	9.5	3.0 - 19.5	0.09
6010205	17105	CLINCH R	30.8	31.7	10 - 80	0.0	0.0 - 0.0	4.9	1.6 - 11.5	4.7	1.4 - 14.9	12.5	4.5 - 33.7	0.0	0.0 - 0.0	9.5	3.1 - 26.3	0.09
6010205	17106	CLINCH R	22.1	44.9	15 - 88	0.0	0.0 - 0.0	7.1	2.0 - 13.5	7.1	1.9 - 17.4	19.9	6.6 - 42.7	0.0	0.0 - 0.0	10.8	3.6 - 27.6	0.09
6010205	17107	LITTLE R	320.0	34.5	11 - 79	0.0	0.0 - 0.0	3.5	1.1 - 8.7	6.3	1.9 - 17.6	16.5	5.3 - 45.7	0.0	0.0 - 0.0	8.2	3.1 - 23.9	0.09
6010205	17108	CLINCH R	10.2	30.6	11 - 62	0.0	0.0 - 0.0	9.9	3.6 - 21.9	3.4	1.1 - 9.1	8.6	2.8 - 21.5	0.0	0.0 - 0.0	8.8	3.1 - 19.9	0.09
6010205	17109	CLINCH R	28.7	29.9	10 - 76	0.0	0.0 - 0.0	5.1	1.7 - 13.2	4.3	1.5 - 15.1	11.9	3.9 - 32.0	0.0	0.0 - 0.0	8.6	2.9 - 22.5	0.09
6010205	17110	CLINCH R	28.9	25.5	9 - 47	0.0	0.0 - 0.0	13.3	4.5 - 26.9	1.8	0.6 - 4.5	4.8	1.5 - 13.3	0.0	0.0 - 0.0	5.6	2.0 - 11.8	0.09
6010205	17111	CLINCH R	24.5	138.9	46 - 273	112.5	38.8 - 222.7	14.7	4.6 - 26.7	1.5	0.4 - 3.4	4.1	1.3 - 10.3	0.0	0.0 - 0.0	6.1	1.9 - 13.3	0.09
6010205	17112	CLINCH R	8.1	42.8	14 - 94	0.0	0.0 - 0.0	29.6	9.2 - 68.9	1.0	0.3 - 2.6	3.1	1.0 - 7.6	0.0	0.0 - 0.0	9.0	3.2 - 19.9	0.09
6010205	17114	CLINCH R	77.8	33.5	12 - 55	0.0	0.0 - 0.0	8.2	2.9 - 15.7	4.5	1.5 - 12.7	12.0	4.6 - 28.6	0.0	0.0 - 0.0	8.9	3.3 - 18.2	0.08
6010205	17115	CLINCH R, BUNDY	32.0	34.3	11 - 70	0.0	0.0 - 0.0	4.6	1.5 - 8.0	6.1	1.6 - 18.5	15.2	5.2 - 37.8	0.0	0.0 - 0.0	8.4	2.9 - 18.8	0.07
6010205	17116	CLINCH R	124.8	41.9	16 - 93	7.1	2.6 - 16.8	9.6	3.4 - 20.8	5.7	1.7 - 16.4	11.4	4.4 - 26.5	0.0	0.0 - 0.0	8.2	3.0 - 20.0	0.07
6010205	17117	GREASY CR	87.5	19.6	8 - 50	0.0	0.0 - 0.0	6.5	2.4 - 17.6	1.8	0.6 - 5.9	4.7	1.8 - 12.9	0.0	0.0 - 0.0	6.6	2.4 - 19.2	0.08

8-digit HUC	ID	Name	Area	Catchment Yield		Point sources		Developed Land		Manure		Agricultural Land		Phosphate Mines		Soil parent rock		Frac
				kg/km^2	90% CI	kg/km^2	90% CI	kg/km^2	90% CI	kg/km^2	90% CI	kg/km^2	90% CI	kg/km^2	90% CI	kg/km^2	90% CI	
6010205	17118	MIDDLE CR	32.8	16.3	5 - 34	0.0	0.0 - 0.0	5.9	1.8 - 12.8	0.9	0.3 - 2.6	2.2	0.8 - 5.8	0.0	0.0 - 0.0	7.2	2.6 - 17.2	0.08
6010205	17119	BIG CR	41.2	13.2	5 - 25	0.0	0.0 - 0.0	6.9	2.6 - 15.9	0.5	0.2 - 1.9	1.1	0.4 - 2.6	0.0	0.0 - 0.0	4.7	1.9 - 9.2	0.09
6010205	17120	MUDLICK CR	19.0	14.2	4 - 31	0.0	0.0 - 0.0	6.0	1.8 - 14.0	0.8	0.2 - 2.3	2.2	0.7 - 5.6	0.0	0.0 - 0.0	5.2	1.7 - 11.1	0.09
6010205	17121	MILL CR	12.4	19.6	7 - 37	0.0	0.0 - 0.0	8.9	3.0 - 18.2	1.5	0.5 - 3.8	4.3	1.3 - 10.2	0.0	0.0 - 0.0	4.9	1.7 - 10.7	0.09
6010205	17122	SWORDS CR	5.6	30.9	12 - 67	0.0	0.0 - 0.0	12.1	4.8 - 28.0	2.9	1.0 - 8.4	8.3	2.8 - 22.6	0.0	0.0 - 0.0	7.5	2.9 - 18.7	0.09
6010205	17123	SWORDS CR	28.6	18.5	6 - 44	0.0	0.0 - 0.0	9.4	3.0 - 23.5	1.2	0.4 - 3.0	3.0	1.0 - 7.6	0.0	0.0 - 0.0	5.0	1.6 - 11.6	0.09
6010205	17124	HESS CR	25.1	18.9	5 - 32	0.0	0.0 - 0.0	8.7	2.6 - 16.1	1.6	0.5 - 3.8	3.8	1.2 - 11.5	0.0	0.0 - 0.0	4.8	1.8 - 9.6	0.09
6010205	17125	LEWIS CR	54.3	21.2	7 - 43	0.0	0.0 - 0.0	8.0	2.5 - 17.2	2.1	0.6 - 4.8	5.8	1.8 - 15.0	0.0	0.0 - 0.0	5.2	1.7 - 13.2	0.09
6010205	17126	CHANEY CR	86.5	13.6	4 - 26	0.0	0.0 - 0.0	5.0	1.6 - 10.2	1.4	0.4 - 4.3	2.8	0.8 - 6.8	0.0	0.0 - 0.0	4.4	1.4 - 12.1	0.10
6010205	17127	LICK CR	74.1	18.4	6 - 46	0.0	0.0 - 0.0	9.3	3.4 - 21.8	1.1	0.4 - 3.3	2.6	0.9 - 7.6	0.0	0.0 - 0.0	5.5	2.0 - 17.4	0.10
6010205	17128	GUEST R	260.7	39.1	18 - 79	13.9	6.2 - 28.9	13.4	5.6 - 31.9	0.4	0.2 - 1.0	4.9	2.0 - 12.4	0.0	0.0 - 0.0	6.4	3.1 - 13.8	0.10
6010205	17129	CLINCH R, CORDE	42.7	13.9	5 - 26	0.0	0.0 - 0.0	5.9	1.9 - 12.8	0.2	0.1 - 0.6	1.0	0.3 - 2.4	0.0	0.0 - 0.0	6.7	2.4 - 14.4	0.10
6010205	17130	STONY CR	109.9	16.4	5 - 31	0.0	0.0 - 0.0	3.8	1.4 - 8.1	1.2	0.3 - 3.3	2.6	0.8 - 6.8	0.0	0.0 - 0.0	8.7	2.8 - 19.7	0.10
6010205	17131	COVE CR	64.0	28.5	8 - 73	0.0	0.0 - 0.0	3.9	1.0 - 9.1	3.6	0.8 - 9.5	10.7	3.2 - 31.8	0.0	0.0 - 0.0	10.3	3.3 - 27.4	0.10
6010205	17132	STOCK CR	74.5	18.1	5 - 35	0.0	0.0 - 0.0	4.3	1.2 - 8.0	1.4	0.4 - 3.7	3.3	0.9 - 7.5	0.0	0.0 - 0.0	9.1	2.6 - 20.4	0.10
6010205	17133	CLINCH R, N FK	222.3	19.6	6 - 41	0.0	0.0 - 0.0	7.1	2.2 - 14.7	1.8	0.5 - 5.0	1.9	0.5 - 5.5	0.0	0.0 - 0.0	8.8	2.6 - 20.8	0.11
6010205	17134	BLACKWATER CR	115.9	17.3	6 - 33	0.0	0.0 - 0.0	4.5	1.6 - 9.0	2.3	0.7 - 8.4	2.7	1.0 - 6.3	0.0	0.0 - 0.0	7.8	2.5 - 19.4	0.11
6010206	17135	POWELL R	61.0	32.7	10 - 63	0.0	0.0 - 0.0	7.5	2.3 - 14.2	6.6	1.9 - 16.4	6.1	1.9 - 18.1	0.0	0.0 - 0.0	12.5	4.1 - 31.6	0.11
6010206	17136	RUSSELL CR	44.4	77.9	23 - 132	23.2	7.2 - 46.8	19.3	5.4 - 37.0	10.0	3.0 - 24.9	12.1	3.8 - 24.7	0.0	0.0 - 0.0	13.2	3.7 - 28.2	0.10
6010206	17137	POWELL R	216.8	36.4	12 - 80	0.0	0.0 - 0.0	5.9	1.8 - 13.6	8.7	2.7 - 25.5	9.9	3.2 - 25.8	0.0	0.0 - 0.0	11.8	4.1 - 31.8	0.10
6010206	17138	POWELL R	66.3	45.2	16 - 80	0.0	0.0 - 0.0	5.6	1.8 - 10.3	10.0	3.0 - 26.8	14.6	4.6 - 33.9	0.0	0.0 - 0.0	15.0	5.2 - 33.3	0.10
6010206	17139	POWELL R	16.3	60.4	20 - 152	0.0	0.0 - 0.0	5.6	1.8 - 15.0	14.3	4.2 - 51.6	23.0	7.6 - 71.6	0.0	0.0 - 0.0	17.5	6.2 - 47.3	0.10
6010206	17140	WALLEN CR	122.1	28.5	9 - 52	0.0	0.0 - 0.0	5.3	1.5 - 10.5	5.9	1.8 - 17.8	5.3	1.9 - 12.0	0.0	0.0 - 0.0	12.0	3.8 - 23.3	0.10
6010206	17141	POWELL R	152.9	41.6	14 - 113	0.0	0.0 - 0.0	11.6	3.9 - 27.8	8.3	2.2 - 30.5	9.7	3.0 - 27.7	0.0	0.0 - 0.0	12.0	3.8 - 35.2	0.10
6010206	17142	POWELL R	118.0	39.9	14 - 76	16.1	5.8 - 33.1	10.2	3.3 - 20.2	3.2	1.1 - 9.4	1.9	0.7 - 4.7	0.0	0.0 - 0.0	8.4	3.1 - 19.2	0.09
6010206	17143	*A	113.4	29.3	10 - 70	0.0	0.0 - 0.0	17.7	6.3 - 37.6	0.4	0.1 - 1.2	2.1	0.7 - 6.1	0.0	0.0 - 0.0	9.2	3.0 - 20.7	0.09
6010206	17144	POWELL R	42.4	17.2	5 - 29	0.0	0.0 - 0.0	9.8	2.7 - 17.0	0.1	0.0 - 0.3	0.0	0.0 - 0.0	0.0	0.0 - 0.0	7.3	2.3 - 14.6	0.09
6010206	17145	POWELL R	158.0	13.5	5 - 24	0.0	0.0 - 0.0	4.9	1.8 - 10.0	0.3	0.1 - 0.8	2.0	0.6 - 5.1	0.0	0.0 - 0.0	6.3	2.2 - 12.9	0.08
6010206	17146	MUD LICK CR	86.0	10.4	3 - 20	0.0	0.0 - 0.0	3.6	1.1 - 7.4	0.1	0.0 - 0.2	0.0	0.0 - 0.0	0.0	0.0 - 0.0	6.7	2.0 - 15.2	0.08
6010206	17147	POWELL R, N FK	233.0	21.1	7 - 42	1.4	0.5 - 3.3	10.8	3.5 - 23.4	1.1	0.3 - 2.8	0.4	0.1 - 0.9	0.0	0.0 - 0.0	7.3	2.5 - 17.5	0.09
6010206	17148	HARDY CR	1.2	72.3	22 - 145	0.0	0.0 - 0.0	11.2	3.8 - 20.0	15.4	4.5 - 42.8	30.7	10.4 - 74.0	0.0	0.0 - 0.0	15.0	5.4 - 38.0	0.10
6010206	17149	DRY CR	68.7	26.0	8 - 46	0.0	0.0 - 0.0	4.9	1.6 - 10.1	5.5	1.4 - 15.1	6.4	1.9 - 16.0	0.0	0.0 - 0.0	9.2	2.7 - 21.0	0.10
6010206	17150	HARDY CR	42.4	24.9	8 - 61	0.0	0.0 - 0.0	6.4	2.0 - 13.6	4.4	1.2 - 13.2	5.3	1.7 - 14.7	0.0	0.0 - 0.0	8.9	2.8 - 26.2	0.10
6010206	17151	MARTIN CR	62.9	30.7	10 - 54	0.0	0.0 - 0.0	5.8	1.7 - 10.7	6.7	1.8 - 17.8	8.3	2.8 - 19.4	0.0	0.0 - 0.0	9.9	3.4 - 22.0	0.10
6010206	17152	INDIAN CR	172.4	29.7	11 - 78	0.0	0.0 - 0.0	7.0	2.4 - 16.6	5.7	1.8 - 17.2	8.0	3.1 - 20.5	0.0	0.0 - 0.0	9.0	3.0 - 22.4	0.11
6010207	17153	CLINCH R	21.7	133.4	40 - 264	61.1	17.8 - 128.0	48.0	14.5 - 101.4	1.8	0.5 - 4.3	5.8	1.7 - 17.7	0.0	0.0 - 0.0	16.7	5.3 - 31.0	0.30
6010207	17154	CLINCH R	41.6	50.7	18 - 89	0.0	0.0 - 0.0	16.3	5.1 - 34.6	4.3	1.2 - 11.2	15.2	4.5 - 33.5	0.0	0.0 - 0.0	14.9	5.4 - 33.5	0.30
6010207	17155	CLINCH R	118.7	47.3	16 - 79	0.1	0.0 - 0.2	15.3	5.3 - 26.5	4.3	1.4 - 10.5	12.9	4.3 - 26.9	0.0	0.0 - 0.0	14.6	4.8 - 30.0	0.30
6010207	17156	HICKORY CR	24.5	53.9	19 - 140	0.0	0.0 - 0.0	23.3	7.9 - 57.0	4.4	1.5 - 15.3	12.6	4.4 - 36.2	0.0	0.0 - 0.0	13.7	4.7 - 42.3	0.30

8-digit HUC	ID	Name	Area	Catchment Yield		Point sources		Developed Land		Manure		Agricultural Land		Phosphate Mines		Soil parent rock		Frac
				kg/km^2	90% CI	kg/km^2	90% CI	kg/km^2	90% CI	kg/km^2	90% CI	kg/km^2	90% CI	kg/km^2	90% CI	kg/km^2	90% CI	
6010207	17157	CLINCH R	5.5	33.7	11 - 80	0.0	0.0 - 0.0	8.2	2.8 - 19.6	2.6	0.8 - 7.1	8.4	2.9 - 20.9	0.0	0.0 - 0.0	14.5	5.0 - 33.9	0.30
6010207	17158	BULLRUN CR	272.3	45.2	17 - 115	1.6	0.5 - 3.7	14.4	5.2 - 35.9	4.6	1.4 - 12.5	10.6	3.4 - 27.6	0.0	0.0 - 0.0	14.0	4.6 - 34.5	0.26
6010207	17159	CLINCH R	122.3	86.6	25 - 207	19.2	5.7 - 42.3	40.4	11.1 - 102.9	2.7	0.8 - 6.6	9.8	3.0 - 29.3	0.0	0.0 - 0.0	14.5	3.9 - 32.9	0.26
6010207	17160	HINDS CR	17.3	62.8	17 - 130	0.0	0.0 - 0.0	17.5	5.0 - 36.9	6.2	1.5 - 15.2	24.4	7.9 - 50.6	0.0	0.0 - 0.0	14.7	4.1 - 34.3	0.26
6010207	17161	HINDS CR	103.9	42.8	13 - 82	0.0	0.0 - 0.0	11.6	4.1 - 23.4	4.1	1.2 - 11.3	11.1	3.4 - 24.5	0.0	0.0 - 0.0	16.0	5.0 - 38.9	0.25
6010207	17162	BUFFALO CR	49.2	67.3	21 - 170	3.4	1.0 - 8.1	21.8	6.2 - 50.3	6.1	1.6 - 16.2	22.0	6.5 - 60.9	0.0	0.0 - 0.0	14.0	4.1 - 33.8	0.25
6010207	17163	CLINCH R	175.0	39.2	15 - 64	4.1	1.6 - 6.7	17.1	5.7 - 30.0	1.8	0.5 - 4.7	5.7	2.0 - 13.6	0.0	0.0 - 0.0	10.6	3.5 - 21.2	0.26
6010207	17164	POPLAR CR	10.0	83.6	28 - 195	0.0	0.0 - 0.0	60.4	19.6 - 136.5	1.6	0.4 - 4.9	5.0	1.4 - 12.9	0.0	0.0 - 0.0	16.5	5.9 - 39.4	0.30
6010207	17165	POPLAR CR, BRUS	77.3	56.3	21 - 118	0.0	0.0 - 0.0	38.6	14.1 - 80.7	0.9	0.3 - 2.8	3.0	1.1 - 8.6	0.0	0.0 - 0.0	13.8	4.5 - 36.9	0.30
6010207	17166	POPLAR CR	263.1	33.9	16 - 69	2.3	1.0 - 5.3	12.7	4.9 - 26.8	2.0	0.7 - 4.7	6.8	2.8 - 17.9	0.0	0.0 - 0.0	10.1	4.4 - 25.0	0.30
6010208	17167	EMORY R	183.8	39.1	14 - 78	0.0	0.0 - 0.0	18.3	6.1 - 41.6	2.2	0.7 - 5.9	6.7	2.2 - 16.5	0.0	0.0 - 0.0	11.9	3.6 - 25.4	0.30
6010208	17168	EMORY R	16.8	40.1	14 - 92	0.0	0.0 - 0.0	15.3	4.4 - 35.7	3.2	0.8 - 8.7	8.4	2.8 - 21.1	0.0	0.0 - 0.0	13.1	4.2 - 32.5	0.30
6010208	17169	EMORY R	5.4	21.1	6 - 41	0.0	0.0 - 0.0	5.2	1.5 - 9.4	0.6	0.1 - 1.5	0.7	0.2 - 1.6	0.0	0.0 - 0.0	14.7	4.2 - 32.0	0.29
6010208	17170	EMORY R, CROOK	168.4	33.1	12 - 73	5.0	2.0 - 11.5	10.4	3.4 - 21.8	2.0	0.6 - 5.6	4.8	1.7 - 12.9	0.0	0.0 - 0.0	10.9	3.9 - 25.5	0.29
6010208	17171	EMORY R	67.7	21.5	8 - 60	0.0	0.0 - 0.0	5.2	1.9 - 12.8	0.5	0.2 - 1.5	0.5	0.2 - 1.3	0.0	0.0 - 0.0	15.4	5.4 - 45.3	0.29
6010208	17172	EMORY R	238.7	18.8	7 - 35	0.0	0.0 - 0.0	6.3	2.1 - 11.8	0.9	0.3 - 2.3	1.6	0.6 - 4.8	0.0	0.0 - 0.0	10.1	3.5 - 22.5	0.29
6010208	17173	OBED R	19.1	25.9	7 - 58	0.0	0.0 - 0.0	6.1	1.7 - 13.9	2.2	0.7 - 6.7	4.4	1.1 - 12.5	0.0	0.0 - 0.0	13.2	4.2 - 33.2	0.29
6010208	17174	CLEAR CR	39.4	27.2	9 - 56	0.0	0.0 - 0.0	9.2	3.1 - 20.2	2.1	0.7 - 5.6	3.3	1.1 - 9.2	0.0	0.0 - 0.0	12.6	4.6 - 29.5	0.29
6010208	17175	WHITE CR	131.5	25.1	8 - 40	0.0	0.0 - 0.0	7.2	2.4 - 13.6	2.4	0.7 - 5.9	3.8	1.2 - 7.6	0.0	0.0 - 0.0	11.6	3.6 - 24.2	0.27
6010208	17176	CLEAR CR	249.3	30.2	9 - 64	0.0	0.0 - 0.0	5.8	1.9 - 11.9	4.1	1.2 - 11.7	7.7	2.5 - 18.1	0.0	0.0 - 0.0	12.6	3.8 - 29.4	0.27
6010208	17177	OBED R	14.5	18.7	7 - 48	0.0	0.0 - 0.0	2.4	0.8 - 6.4	1.1	0.4 - 3.9	1.0	0.4 - 3.0	0.0	0.0 - 0.0	14.3	5.1 - 38.4	0.29
6010208	17178	OBED R	31.0	24.4	9 - 54	0.0	0.0 - 0.0	7.2	2.4 - 17.4	1.5	0.5 - 4.4	1.9	0.6 - 5.6	0.0	0.0 - 0.0	13.8	4.7 - 36.3	0.29
6010208	17179	OBED R	324.1	57.2	23 - 147	11.9	4.3 - 30.3	17.1	6.0 - 44.3	3.4	1.1 - 10.8	10.5	3.4 - 29.2	0.0	0.0 - 0.0	14.3	4.7 - 34.2	0.28
6010208	17180	OTTER CR	52.3	30.1	10 - 62	0.0	0.0 - 0.0	10.8	3.1 - 24.0	1.7	0.5 - 4.8	3.1	0.8 - 8.4	0.0	0.0 - 0.0	14.5	5.3 - 31.9	0.28
6010208	17181	DADDY'S CR	208.5	32.9	9 - 71	0.0	0.0 - 0.0	12.9	3.5 - 29.3	1.9	0.5 - 5.0	2.9	0.8 - 7.6	0.0	0.0 - 0.0	15.2	4.5 - 36.5	0.29
6010208	17182	BYRD CR	75.8	57.4	17 - 115	0.0	0.0 - 0.0	29.2	9.5 - 63.5	3.7	0.9 - 9.6	10.5	2.9 - 25.9	0.0	0.0 - 0.0	13.9	3.9 - 32.2	0.27
6010208	17183	DADDY'S CR	73.6	38.9	15 - 98	0.0	0.0 - 0.0	10.5	3.5 - 25.9	4.2	1.4 - 15.0	11.1	4.3 - 31.3	0.0	0.0 - 0.0	13.1	4.6 - 37.2	0.27
6010208	17184	BASSES CR	40.0	51.6	16 - 131	0.0	0.0 - 0.0	22.4	6.6 - 56.0	3.8	1.0 - 10.1	10.4	2.7 - 36.4	0.0	0.0 - 0.0	15.1	4.0 - 34.1	0.25
6010208	17185	DADDY'S CR	57.7	41.2	14 - 82	0.0	0.0 - 0.0	8.3	2.8 - 16.8	5.0	1.3 - 11.4	14.4	4.8 - 35.7	0.0	0.0 - 0.0	13.5	4.4 - 32.3	0.25
6010208	17186	CRAB ORCHARD C	124.5	25.1	8 - 49	0.0	0.0 - 0.0	5.6	1.9 - 12.2	1.9	0.6 - 5.6	3.2	1.0 - 7.5	0.0	0.0 - 0.0	14.4	4.8 - 31.2	0.29
6010208	17187	CLIFTY CR	52.2	31.2	12 - 79	0.0	0.0 - 0.0	9.3	3.2 - 21.3	2.7	0.8 - 8.3	5.1	1.7 - 13.4	0.0	0.0 - 0.0	14.1	4.9 - 37.5	0.30
6020001	17188	TENNESSEE R	338.4	21.4	9 - 39	0.0	0.0 - 0.0	5.9	2.3 - 12.7	2.0	0.7 - 4.8	4.2	1.5 - 10.5	0.0	0.0 - 0.0	9.3	3.6 - 20.8	0.44
6020001	17189	TENNESSEE R	96.0	2784.1	994 - 6271	2718.6	966.8 - 6131.3	48.6	19.0 - 108.0	1.1	0.3 - 3.1	3.2	1.1 - 9.8	0.0	0.0 - 0.0	12.6	4.5 - 29.0	0.43
6020001	17190	LOOKOUT CR	494.1	41.7	17 - 103	1.0	0.4 - 2.4	22.4	9.3 - 50.1	3.7	1.2 - 9.9	5.8	1.9 - 17.3	0.0	0.0 - 0.0	8.9	3.5 - 21.6	0.43
6020001	17191	LOOKOUT CR	180.0	34.8	12 - 87	0.0	0.0 - 0.0	8.9	3.0 - 20.4	8.2	2.7 - 22.2	8.9	3.0 - 24.6	0.0	0.0 - 0.0	8.9	3.7 - 21.8	0.37
6020001	17192	TENNESSEE R	51.8	193.6	78 - 475	67.7	26.6 - 193.0	102.3	40.1 - 247.8	0.5	0.2 - 1.7	1.2	0.4 - 3.2	0.0	0.0 - 0.0	21.8	7.3 - 60.6	0.43
6020001	17193	CHICKAMAUGA C	160.5	117.7	36 - 227	10.5	3.7 - 22.0	85.4	25.6 - 172.8	1.4	0.4 - 3.6	3.1	1.0 - 7.0	0.0	0.0 - 0.0	17.4	5.5 - 38.3	0.43
6020001	17194	W CHICKAMAUGA	113.7	105.6	40 - 172	18.8	8.1 - 35.2	29.7	10.1 - 56.0	14.8	5.0 - 32.4	22.3	7.9 - 55.7	0.0	0.0 - 0.0	20.0	6.7 - 41.2	0.41
6020001	17195	W CHICKAMAUGA	263.8	46.4	17 - 90	0.0	0.0 - 0.0	6.9	2.5 - 14.2	10.2	3.1 - 27.7	18.0	6.4 - 43.3	0.0	0.0 - 0.0	11.3	4.3 - 24.7	0.38

8-digit HUC	ID	Name	Area	Catchment Yield		Point sources		Developed Land		Manure		Agricultural Land		Phosphate Mines		Soil parent rock		Frac
				kg/km^2	90% CI	kg/km^2	90% CI	kg/km^2	90% CI	kg/km^2	90% CI	kg/km^2	90% CI	kg/km^2	90% CI	kg/km^2	90% CI	
6020001	17196	CRAWFISH CR	27.6	49.8	19 - 119	0.0	0.0 - 0.0	9.3	3.1 - 22.7	8.2	2.7 - 23.2	13.5	5.1 - 34.8	0.0	0.0 - 0.0	18.9	7.1 - 48.9	0.38
6020001	17197	E CHICKAMAUGA	37.7	88.9	28 - 202	0.0	0.0 - 0.0	64.6	19.9 - 154.2	2.2	0.6 - 6.0	6.2	2.1 - 14.6	0.0	0.0 - 0.0	15.9	5.5 - 40.5	0.41
6020001	17198	PEAVINE CR	89.2	81.4	28 - 189	0.0	0.0 - 0.0	22.2	8.0 - 53.0	16.1	5.2 - 49.6	25.2	8.6 - 65.5	0.0	0.0 - 0.0	17.9	5.9 - 45.5	0.41
6020001	17199	E CHICKAMAUGA	89.3	74.4	27 - 132	9.9	3.6 - 20.6	27.4	9.0 - 56.6	9.1	2.8 - 22.5	15.5	5.3 - 34.7	0.0	0.0 - 0.0	12.6	4.5 - 29.0	0.41
6020001	17200	LITTLE CHICKAM/	122.7	70.0	26 - 165	0.0	0.0 - 0.0	12.3	4.5 - 27.0	16.9	5.7 - 46.3	25.7	9.8 - 62.5	0.0	0.0 - 0.0	15.0	5.4 - 40.2	0.39
6020001	17201	E CHICKAMAUGA	18.9	40.9	18 - 81	0.0	0.0 - 0.0	17.5	7.3 - 37.7	5.6	1.9 - 17.6	7.8	2.8 - 20.2	0.0	0.0 - 0.0	10.0	4.1 - 20.6	0.39
6020001	17202	E CHICKAMAUGA	164.6	51.4	17 - 108	0.0	0.0 - 0.0	11.5	3.9 - 24.4	12.2	3.8 - 36.5	14.6	5.4 - 36.0	0.0	0.0 - 0.0	13.1	4.5 - 28.7	0.39
6020001	17203	TIGER CR	110.6	54.2	17 - 96	0.0	0.0 - 0.0	8.1	2.5 - 15.9	13.1	4.1 - 30.2	18.5	5.2 - 42.9	0.0	0.0 - 0.0	14.5	5.0 - 31.3	0.39
6020001	17204	TENNESSEE R	19.0	306.2	120 - 859	184.8	71.3 - 487.1	100.2	37.3 - 312.1	0.8	0.3 - 2.4	2.8	1.2 - 8.6	0.0	0.0 - 0.0	17.6	7.4 - 50.3	0.43
6020001	17205	TENNESSEE R	1.0	52.4	19 - 112	0.0	0.0 - 0.0	18.5	5.3 - 42.3	0.7	0.2 - 1.9	2.7	0.8 - 6.5	0.0	0.0 - 0.0	30.6	11.1 - 78.3	0.42
6020001	17206	TENNESSEE R	1.8	53.3	19 - 134	0.0	0.0 - 0.0	5.1	1.8 - 11.7	6.8	2.1 - 25.7	26.3	9.6 - 77.1	0.0	0.0 - 0.0	15.1	5.0 - 37.5	0.37
6020001	17207	TENNESSEE R	94.9	77.5	25 - 143	4.4	1.5 - 9.5	5.8	1.7 - 11.0	8.8	2.6 - 21.8	34.1	10.9 - 78.3	0.0	0.0 - 0.0	24.5	7.0 - 50.7	0.37
6020001	17208	DECATEUR CR	64.9	51.2	21 - 89	0.0	0.0 - 0.0	8.7	3.2 - 16.6	6.5	2.2 - 13.5	19.3	7.0 - 41.9	0.0	0.0 - 0.0	16.6	6.2 - 35.8	0.37
6020001	17209	TENNESSEE R	59.4	62.4	24 - 127	0.0	0.0 - 0.0	8.5	3.0 - 18.0	6.7	2.4 - 22.3	28.6	9.7 - 73.9	0.0	0.0 - 0.0	18.6	6.9 - 44.3	0.37
6020001	17210	TENNESSEE R	24.0	46.5	17 - 96	0.0	0.0 - 0.0	3.2	1.0 - 6.5	5.1	1.5 - 14.0	19.7	6.1 - 45.1	0.0	0.0 - 0.0	18.5	6.8 - 44.2	0.37
6020001	17211	SEWEE CR	75.2	48.5	19 - 97	0.0	0.0 - 0.0	6.6	2.4 - 15.4	6.6	2.2 - 19.6	19.5	7.0 - 48.7	0.0	0.0 - 0.0	15.9	5.9 - 37.8	0.37
6020001	17212	BLACK ANKLE CR	84.2	51.6	17 - 110	0.0	0.0 - 0.0	6.0	1.8 - 13.2	9.7	2.6 - 25.5	22.6	7.3 - 54.1	0.0	0.0 - 0.0	13.4	5.0 - 30.9	0.35
6020001	17213	SEWEE CR	11.9	51.1	18 - 111	0.0	0.0 - 0.0	10.3	3.5 - 20.7	6.7	1.7 - 19.5	18.5	6.1 - 46.7	0.0	0.0 - 0.0	15.6	6.0 - 36.5	0.35
6020001	17214	LITTLE SEEWEE C	77.3	46.0	14 - 105	0.0	0.0 - 0.0	5.3	1.5 - 12.4	7.3	1.9 - 23.0	20.1	5.8 - 50.5	0.0	0.0 - 0.0	13.3	3.8 - 34.3	0.34
6020001	17215	TENNESSEE R	68.0	54.6	17 - 124	0.0	0.0 - 0.0	8.1	2.6 - 18.8	6.0	1.6 - 16.5	22.7	7.0 - 63.6	0.0	0.0 - 0.0	17.9	5.7 - 42.4	0.37
6020001	17216	CLEAR CR	57.4	42.8	13 - 86	0.0	0.0 - 0.0	6.5	2.0 - 12.6	4.0	1.1 - 10.7	18.6	5.7 - 47.1	0.0	0.0 - 0.0	13.7	4.2 - 31.9	0.37
6020001	17217	RICHLAND CR	14.2	60.6	26 - 115	0.0	0.0 - 0.0	18.8	7.2 - 42.0	4.5	1.6 - 14.1	20.9	9.4 - 46.6	0.0	0.0 - 0.0	16.4	6.8 - 37.9	0.37
6020001	17218	LITTLE RICHLANE	56.7	53.1	18 - 96	0.0	0.0 - 0.0	19.6	6.8 - 38.3	3.4	1.0 - 9.2	15.2	5.1 - 36.7	0.0	0.0 - 0.0	14.9	5.0 - 31.7	0.36
6020001	17219	RICHLAND CR	24.2	57.6	24 - 125	0.0	0.0 - 0.0	32.4	12.7 - 75.9	2.0	0.7 - 5.6	8.6	2.9 - 20.6	0.0	0.0 - 0.0	14.6	6.0 - 31.6	0.36
6020001	17220	RICHLAND CR	1.7	9.9	4 - 25	0.0	0.0 - 0.0	0.0	0.0 - 0.0	0.5	0.2 - 1.5	0.0	0.0 - 0.0	0.0	0.0 - 0.0	9.5	3.8 - 24.3	0.36
6020001	17221	LAUREL CR	14.5	29.8	10 - 73	0.0	0.0 - 0.0	6.1	2.0 - 14.0	2.3	0.7 - 5.7	6.6	2.0 - 16.0	0.0	0.0 - 0.0	14.8	4.8 - 34.0	0.35
6020001	17222	RICHLAND CR	66.9	43.9	14 - 102	0.0	0.0 - 0.0	6.6	1.9 - 15.3	6.9	2.2 - 17.0	15.4	4.9 - 45.4	0.0	0.0 - 0.0	15.0	4.8 - 36.9	0.35
6020001	17223	TIGUES CR	35.5	50.9	16 - 134	0.0	0.0 - 0.0	9.4	3.6 - 22.7	5.7	2.1 - 15.4	20.7	6.2 - 63.6	0.0	0.0 - 0.0	15.1	5.4 - 44.2	0.36
6020001	17224	ROARING CR	22.7	31.7	11 - 75	0.0	0.0 - 0.0	8.5	3.0 - 19.7	3.2	1.1 - 9.0	9.2	3.0 - 25.3	0.0	0.0 - 0.0	10.7	3.6 - 24.5	0.36
6020001	17225	ROARING CR	111.8	41.2	14 - 94	0.0	0.0 - 0.0	7.9	2.7 - 17.1	5.0	1.6 - 14.3	14.6	5.0 - 39.3	0.0	0.0 - 0.0	13.7	4.4 - 34.2	0.35
6020001	17226	MCGILL CR	35.4	29.7	11 - 58	0.0	0.0 - 0.0	3.4	1.2 - 6.6	3.7	1.1 - 9.4	8.1	2.6 - 17.2	0.0	0.0 - 0.0	14.5	4.9 - 33.7	0.35
6020001	17227	ROCK CR	101.6	27.1	8 - 50	0.0	0.0 - 0.0	4.3	1.2 - 8.5	3.2	0.9 - 8.0	5.5	1.7 - 14.3	0.0	0.0 - 0.0	14.2	4.3 - 29.4	0.36
6020001	17228	SODDY CR	31.3	23.9	8 - 46	0.0	0.0 - 0.0	2.3	0.7 - 4.5	1.6	0.4 - 5.6	4.0	1.2 - 12.1	0.0	0.0 - 0.0	15.9	5.4 - 35.8	0.35
6020001	17229	GRAY CR	40.6	29.2	9 - 65	0.0	0.0 - 0.0	4.0	1.3 - 9.2	2.1	0.5 - 5.9	7.5	2.3 - 19.5	0.0	0.0 - 0.0	15.6	4.5 - 33.0	0.35
6020001	17230	N CHICKAMUGA C	76.4	81.9	30 - 161	0.0	0.0 - 0.0	57.3	20.0 - 119.4	2.6	0.8 - 8.1	7.8	2.7 - 20.7	0.0	0.0 - 0.0	14.2	5.3 - 28.2	0.42
6020001	17231	N CHICKAMUGA C	58.9	46.0	16 - 81	0.0	0.0 - 0.0	26.9	9.3 - 53.2	1.9	0.6 - 4.1	5.1	1.6 - 13.8	0.0	0.0 - 0.0	12.1	3.9 - 29.5	0.41
6020001	17232	COOPER CR	27.7	25.1	8 - 53	0.0	0.0 - 0.0	2.8	1.0 - 6.0	1.5	0.5 - 3.3	5.4	1.8 - 14.3	0.0	0.0 - 0.0	15.5	5.3 - 36.4	0.40
6020001	17233	N CHICKAMUGA C	1.9	16.6	5 - 39	0.0	0.0 - 0.0	0.0	0.0 - 0.0	0.3	0.1 - 0.8	0.3	0.1 - 0.9	0.0	0.0 - 0.0	16.0	5.3 - 37.8	0.40
6020001	17234	CAIN CR	5.0	16.1	6 - 35	0.0	0.0 - 0.0	0.0	0.0 - 0.0	0.4	0.1 - 0.9	0.1	0.0 - 0.3	0.0	0.0 - 0.0	15.6	5.4 - 33.8	0.40

8-digit HUC	ID	Name	Area	Catchment Yield		Point sources		Developed Land		Manure		Agricultural Land		Phosphate Mines		Soil parent rock		Frac
				kg/km^2	90% CI	kg/km^2	90% CI	kg/km^2	90% CI	kg/km^2	90% CI	kg/km^2	90% CI	kg/km^2	90% CI	kg/km^2	90% CI	
6020001	17235	CAIN CR	18.3	18.1	6 - 48	0.0	0.0 - 0.0	1.5	0.5 - 3.6	0.3	0.1 - 0.9	0.9	0.3 - 2.3	0.0	0.0 - 0.0	15.4	5.2 - 40.4	0.39
6020001	17236	MOSSY CR	33.0	18.2	6 - 42	0.0	0.0 - 0.0	1.1	0.4 - 2.5	0.5	0.1 - 1.5	0.7	0.2 - 1.8	0.0	0.0 - 0.0	15.9	5.8 - 39.8	0.39
6020001	17237	N CHICKAMUGA C	51.2	23.6	8 - 42	0.0	0.0 - 0.0	4.4	1.4 - 8.0	0.9	0.3 - 2.1	2.0	0.6 - 4.4	0.0	0.0 - 0.0	16.2	5.3 - 33.5	0.40
6020001	17238	PALLING WATER (	35.1	31.6	9 - 62	0.0	0.0 - 0.0	10.3	3.0 - 18.7	1.8	0.5 - 4.3	5.2	1.2 - 15.2	0.0	0.0 - 0.0	14.4	4.2 - 36.9	0.41
6020001	17239	SUCK CR	60.2	25.5	8 - 45	0.0	0.0 - 0.0	6.4	2.0 - 12.2	0.9	0.3 - 2.1	2.2	0.6 - 5.3	0.0	0.0 - 0.0	16.0	4.9 - 33.7	0.43
6020002	17240	LICK CR	32.2	53.2	15 - 105	0.0	0.0 - 0.0	6.1	1.8 - 12.8	11.6	3.3 - 27.4	19.0	4.8 - 50.4	0.0	0.0 - 0.0	16.6	4.8 - 36.5	0.37
6020002	17241	HIWASSEE R	2.8	49.9	17 - 124	0.0	0.0 - 0.0	8.9	2.9 - 19.0	7.4	2.1 - 23.0	10.1	3.4 - 25.6	0.0	0.0 - 0.0	23.5	7.6 - 61.8	0.37
6020002	17242	HIWASSEE R	6.0	29.0	10 - 62	0.0	0.0 - 0.0	3.6	1.3 - 7.6	4.2	1.3 - 9.4	5.1	1.5 - 12.8	0.0	0.0 - 0.0	16.2	5.4 - 43.5	0.37
6020002	17243	CANDIES CR	288.1	60.8	18 - 143	0.0	0.0 - 0.0	15.0	4.1 - 34.7	13.4	3.5 - 33.4	16.6	5.0 - 40.5	0.0	0.0 - 0.0	15.9	5.0 - 39.4	0.37
6020002	17244	HIWASSEE R	1.0	96.6	27 - 197	0.0	0.0 - 0.0	1.5	0.5 - 3.2	27.4	6.5 - 77.7	32.5	9.8 - 68.5	0.0	0.0 - 0.0	35.2	11.4 - 82.8	0.37
6020002	17245	HIWASSEE R	10.1	36.6	14 - 60	0.0	0.0 - 0.0	6.5	2.0 - 11.7	6.4	2.0 - 13.5	8.8	3.0 - 18.4	0.0	0.0 - 0.0	14.9	5.9 - 28.1	0.37
6020002	17247	S MOUSE CR	101.8	98.1	30 - 196	0.0	0.0 - 0.0	56.4	17.0 - 118.0	11.2	3.1 - 31.8	14.2	4.7 - 33.7	0.0	0.0 - 0.0	16.3	6.0 - 35.9	0.37
6020002	17248	HIWASSEE R	26.5	257.7	80 - 684	171.4	55.8 - 461.3	40.9	12.9 - 107.3	12.1	3.4 - 42.7	18.0	5.2 - 61.7	0.0	0.0 - 0.0	15.2	5.2 - 42.9	0.37
6020002	17249	HIWASSEE R	13.1	48.6	14 - 84	0.0	0.0 - 0.0	6.4	1.8 - 12.9	9.0	2.3 - 25.3	13.2	3.2 - 42.0	0.0	0.0 - 0.0	19.9	5.7 - 49.5	0.36
6020002	17250	CHATATA CR	84.9	71.1	23 - 116	0.0	0.0 - 0.0	17.3	5.4 - 30.6	18.0	5.1 - 47.0	22.4	6.3 - 43.9	0.0	0.0 - 0.0	13.4	4.3 - 27.1	0.36
6020002	17251	HIWASSEE R	28.5	47.0	14 - 84	0.0	0.0 - 0.0	4.8	1.6 - 10.0	10.4	2.8 - 29.1	14.6	4.3 - 30.8	0.0	0.0 - 0.0	17.2	5.6 - 33.6	0.36
6020002	17252	S CHESTUEE CR	104.1	58.1	22 - 104	0.0	0.0 - 0.0	8.5	3.1 - 16.6	14.4	4.7 - 35.1	19.3	6.9 - 41.2	0.0	0.0 - 0.0	15.9	6.4 - 38.3	0.36
6020002	17253	HIWASSEE R	7.4	86.8	31 - 199	0.0	0.0 - 0.0	3.6	1.1 - 7.5	22.5	6.1 - 67.3	42.1	13.9 - 113.6	0.0	0.0 - 0.0	18.5	6.2 - 40.6	0.36
6020002	17254	HIWASSEE R	5.6	79.4	26 - 181	0.0	0.0 - 0.0	4.4	1.4 - 9.4	19.0	5.8 - 44.9	37.6	12.3 - 91.8	0.0	0.0 - 0.0	18.5	6.0 - 44.0	0.36
6020002	17255	HIWASSEE R	15.7	80.0	28 - 187	0.0	0.0 - 0.0	7.6	2.5 - 20.7	19.5	5.7 - 62.1	37.3	12.0 - 104.9	0.0	0.0 - 0.0	15.5	5.8 - 36.6	0.36
6020002	17256	HIWASSEE R	553.0	22.9	8 - 46	0.0	0.0 - 0.0	5.9	2.1 - 12.5	1.4	0.4 - 4.0	2.7	0.9 - 6.4	0.0	0.0 - 0.0	12.8	4.3 - 31.4	0.36
6020002	17257	HIWASSEE R	1.1	5.9	2 - 14	0.0	0.0 - 0.0	0.0	0.0 - 0.0	0.0	0.0 - 0.1	0.0	0.0 - 0.0	0.0	0.0 - 0.0	5.9	1.9 - 13.7	0.35
6020002	17258	NOTTELY R	72.7	30.4	10 - 60	0.0	0.0 - 0.0	9.5	3.1 - 20.3	1.1	0.3 - 2.5	2.1	0.7 - 6.7	0.0	0.0 - 0.0	17.6	5.8 - 40.8	0.13
6020002	17259	TOWN CR	46.3	23.7	7 - 61	0.0	0.0 - 0.0	5.9	1.7 - 13.8	1.4	0.4 - 4.0	2.9	0.9 - 8.5	0.0	0.0 - 0.0	13.5	4.6 - 36.5	0.13
6020002	17260	HIWASSEE R	1.8	658.7	194 - 1746	636.8	187.4 - 1789.3	14.5	4.4 - 35.6	0.2	0.1 - 0.6	0.5	0.1 - 1.2	0.0	0.0 - 0.0	6.8	2.2 - 18.3	0.33
6020002	17261	HIWASSEE R	5.6	43.7	14 - 107	0.0	0.0 - 0.0	32.9	10.2 - 81.7	0.5	0.1 - 1.5	1.5	0.5 - 4.3	0.0	0.0 - 0.0	8.8	2.7 - 23.8	0.33
6020002	17262	HIWASSEE R	7.5	25.9	8 - 48	0.0	0.0 - 0.0	11.0	3.2 - 23.0	1.5	0.4 - 3.7	4.4	1.3 - 10.4	0.0	0.0 - 0.0	9.0	3.2 - 18.1	0.33
6020002	17263	BRASSTOWN CR	217.2	26.8	10 - 65	0.8	0.3 - 1.9	8.0	2.4 - 21.2	1.4	0.5 - 3.6	4.0	1.4 - 10.9	0.0	0.0 - 0.0	12.6	4.8 - 32.6	0.32
6020002	17264	HIWASSEE R	47.5	20.5	7 - 60	0.0	0.0 - 0.0	8.5	2.9 - 25.1	0.8	0.3 - 2.7	2.8	0.8 - 11.3	0.0	0.0 - 0.0	8.4	3.1 - 21.2	0.32
6020002	17265	HIWASSEE R	15.9	12.6	5 - 29	0.0	0.0 - 0.0	3.0	1.2 - 7.1	0.4	0.1 - 1.3	1.3	0.4 - 3.8	0.0	0.0 - 0.0	7.9	2.9 - 20.3	0.32
6020002	17266	HIWASSEE R	44.7	53.8	19 - 129	2.5	0.9 - 6.1	14.4	4.6 - 33.2	3.3	1.1 - 9.0	11.4	4.0 - 33.4	0.0	0.0 - 0.0	22.2	7.9 - 52.8	0.32
6020002	17267	HIWASSEE R	119.2	31.8	10 - 61	0.0	0.0 - 0.0	8.2	2.5 - 15.3	0.8	0.2 - 1.9	2.1	0.6 - 4.7	0.0	0.0 - 0.0	20.7	6.3 - 41.4	0.10
6020002	17268	HIGHTOWN CR	85.9	32.4	13 - 81	0.0	0.0 - 0.0	7.8	2.5 - 21.5	1.1	0.4 - 3.3	2.9	1.1 - 8.2	0.0	0.0 - 0.0	20.6	8.1 - 53.7	0.10
6020002	17269	TUSQUITEE CR	112.2	20.7	6 - 47	0.0	0.0 - 0.0	4.2	1.1 - 9.7	0.6	0.1 - 1.4	1.9	0.5 - 4.8	0.0	0.0 - 0.0	14.1	4.3 - 33.0	0.32
6020002	17270	FIRES CR	59.8	15.2	5 - 29	0.0	0.0 - 0.0	2.6	0.9 - 5.4	0.0	0.0 - 0.1	0.1	0.0 - 0.3	0.0	0.0 - 0.0	12.5	4.5 - 26.2	0.32
6020002	17271	PEACHTREE CR	47.8	20.9	7 - 47	0.0	0.0 - 0.0	7.2	2.3 - 16.8	1.0	0.3 - 3.1	3.5	1.1 - 11.1	0.0	0.0 - 0.0	9.1	3.4 - 21.6	0.33
6020002	17272	VALLEY R	30.3	32.9	12 - 84	0.0	0.0 - 0.0	21.2	7.0 - 50.8	0.5	0.2 - 1.6	1.7	0.5 - 5.6	0.0	0.0 - 0.0	9.4	3.6 - 28.6	0.33
6020002	17273	PHILLIPS CR	23.8	21.8	8 - 37	0.0	0.0 - 0.0	9.5	3.2 - 17.0	0.4	0.1 - 0.9	1.2	0.3 - 2.6	0.0	0.0 - 0.0	10.8	3.6 - 22.6	0.31
6020002	17274	VALLEY R	88.4	22.0	9 - 51	0.0	0.0 - 0.0	9.9	3.4 - 23.1	0.3	0.1 - 0.8	0.9	0.3 - 2.7	0.0	0.0 - 0.0	10.9	4.2 - 29.0	0.31

8-digit HUC	ID	Name	Area	Catchment Yield		Point sources		Developed Land		Manure		Agricultural Land		Phosphate Mines		Soil parent rock		Frac
				kg/km^2	90% CI	kg/km^2	90% CI	kg/km^2	90% CI	kg/km^2	90% CI	kg/km^2	90% CI	kg/km^2	90% CI	kg/km^2	90% CI	
6020002	17275	SHULER CR	50.1	8.8	3 - 22	0.0	0.0 - 0.0	2.2	0.8 - 5.4	0.0	0.0 - 0.0	0.0	0.0 - 0.1	0.0	0.0 - 0.0	6.6	2.6 - 17.0	0.35
6020002	17276	CONASAUGA CR	272.2	50.7	17 - 82	5.2	1.8 - 10.8	9.9	3.4 - 18.5	5.5	1.6 - 12.8	11.9	3.6 - 31.1	0.0	0.0 - 0.0	18.2	6.0 - 30.8	0.36
6020002	17277	CHESTUEE CR	344.0	69.1	23 - 136	0.9	0.3 - 2.1	9.4	3.4 - 21.1	12.5	3.7 - 32.8	29.4	9.9 - 77.1	0.0	0.0 - 0.0	16.8	5.1 - 43.2	0.36
6020002	17278	OOSTANAULA CR	181.7	92.8	30 - 226	20.8	7.1 - 55.0	14.0	4.6 - 31.2	11.3	3.7 - 34.4	26.3	8.3 - 68.9	0.0	0.0 - 0.0	20.4	7.0 - 51.3	0.36
6020002	17279	SPRING CR	2.4	43.3	16 - 122	0.0	0.0 - 0.0	13.2	4.3 - 36.7	6.2	2.2 - 19.8	6.3	2.2 - 16.5	0.0	0.0 - 0.0	17.5	6.2 - 58.9	0.37
6020002	17280	N MOUSE CR	195.3	81.2	24 - 208	9.3	3.0 - 25.3	22.9	6.1 - 58.8	10.3	2.6 - 30.3	23.9	6.9 - 72.1	0.0	0.0 - 0.0	14.8	5.1 - 37.2	0.36
6020002	17281	SPRING CR	45.7	61.3	18 - 132	0.0	0.0 - 0.0	7.1	2.2 - 16.9	12.1	3.6 - 43.8	28.0	8.5 - 71.1	0.0	0.0 - 0.0	14.1	4.7 - 32.4	0.36
6020002	17282	ROGERS CR	144.8	37.6	15 - 61	0.0	0.0 - 0.0	5.3	2.1 - 9.8	6.3	2.3 - 14.0	12.7	4.7 - 28.1	0.0	0.0 - 0.0	13.3	5.0 - 25.7	0.37
6020002	17283	PRICE CR	17.6	42.9	13 - 90	0.0	0.0 - 0.0	5.4	1.8 - 10.8	5.3	1.6 - 13.6	15.5	4.9 - 39.4	0.0	0.0 - 0.0	16.7	5.5 - 40.6	0.37
6020003	17284	OCOEE R	71.2	53.8	20 - 99	2.8	0.9 - 5.3	9.4	3.2 - 16.7	9.6	3.0 - 23.9	18.5	6.7 - 42.9	0.0	0.0 - 0.0	13.5	4.8 - 33.6	0.36
6020003	17285	COOKSON CR	33.1	26.6	7 - 46	0.0	0.0 - 0.0	3.5	1.0 - 6.6	3.2	0.8 - 7.5	6.1	1.6 - 12.9	0.0	0.0 - 0.0	13.7	3.9 - 29.5	0.36
6020003	17286	OCOEE R	12.5	38.9	10 - 95	0.0	0.0 - 0.0	6.5	1.8 - 16.3	6.2	1.4 - 20.1	12.0	3.3 - 36.9	0.0	0.0 - 0.0	14.3	3.9 - 33.3	0.36
6020003	17287	OCOEE R	4.1	25.2	9 - 53	0.0	0.0 - 0.0	8.9	3.1 - 20.3	2.4	0.8 - 5.5	3.0	0.9 - 8.2	0.0	0.0 - 0.0	11.0	3.9 - 23.1	0.29
6020003	17288	FIGHTINGTOWN C	94.0	33.0	11 - 65	0.0	0.0 - 0.0	13.1	4.0 - 28.4	3.5	1.0 - 8.5	4.5	1.5 - 9.6	0.0	0.0 - 0.0	11.9	4.3 - 32.6	0.29
6020003	17289	PATTERSON CR	38.3	28.0	11 - 52	0.0	0.0 - 0.0	6.8	2.3 - 14.1	2.2	0.7 - 5.1	2.9	1.0 - 7.1	0.0	0.0 - 0.0	16.2	6.0 - 35.1	0.27
6020003	17290	FIGHTINGTOWN C	52.4	24.3	9 - 58	0.0	0.0 - 0.0	4.7	1.6 - 10.1	1.1	0.3 - 2.6	1.2	0.4 - 3.5	0.0	0.0 - 0.0	17.3	6.0 - 46.0	0.27
6020003	17291	OCOEE R	55.7	40.6	12 - 88	6.1	2.2 - 14.7	15.1	4.1 - 32.7	3.1	0.9 - 9.6	5.3	1.5 - 13.8	0.0	0.0 - 0.0	10.9	3.6 - 27.1	0.29
6020003	17292	OCOEE R	59.6	52.0	18 - 107	9.9	3.8 - 22.7	13.2	4.2 - 34.2	4.9	1.5 - 16.7	6.8	2.1 - 19.7	0.0	0.0 - 0.0	17.1	6.2 - 36.7	0.29
6020003	17293	OCOEE R	141.7	38.8	14 - 76	0.0	0.0 - 0.0	15.4	5.2 - 28.8	4.3	1.3 - 11.1	5.7	1.7 - 14.8	0.0	0.0 - 0.0	13.3	4.8 - 30.8	0.28
6020003	17294	BIG CR	31.2	25.4	9 - 52	0.0	0.0 - 0.0	5.3	1.7 - 12.5	2.3	0.7 - 6.7	1.0	0.3 - 2.5	0.0	0.0 - 0.0	16.7	6.0 - 38.9	0.15
6020003	17295	OCOEE R	5.0	33.4	9 - 56	0.0	0.0 - 0.0	8.7	2.3 - 17.1	2.7	0.7 - 6.4	4.2	1.3 - 8.7	0.0	0.0 - 0.0	17.9	4.5 - 35.7	0.15
6020003	17296	OCOEE R	150.3	23.9	9 - 48	0.0	0.0 - 0.0	4.3	1.3 - 8.6	1.4	0.4 - 3.6	1.9	0.6 - 4.6	0.0	0.0 - 0.0	16.2	5.7 - 31.9	0.15
6020003	17297	OCOEE R	42.1	20.8	9 - 44	0.0	0.0 - 0.0	3.2	1.1 - 7.3	0.7	0.2 - 1.9	0.9	0.3 - 2.5	0.0	0.0 - 0.0	16.0	6.3 - 36.4	0.15
6020003	17298	COOPER CR	214.8	31.5	9 - 67	0.0	0.0 - 0.0	6.1	1.9 - 14.6	0.8	0.2 - 2.5	1.6	0.5 - 4.0	0.0	0.0 - 0.0	23.0	7.0 - 53.8	0.15
6020003	17299	HOTHOUSE CR	67.5	28.6	8 - 53	0.0	0.0 - 0.0	8.3	2.3 - 15.9	2.8	0.8 - 8.0	4.2	1.3 - 11.4	0.0	0.0 - 0.0	13.4	4.1 - 27.9	0.29
6020003	17300	N POTATOE CR	38.9	28.9	10 - 55	0.0	0.0 - 0.0	9.0	2.9 - 19.3	3.4	1.1 - 7.2	6.3	2.0 - 17.0	0.0	0.0 - 0.0	10.3	3.4 - 22.4	0.29
6020004	17301	SEQUATCHIE R	76.5	66.8	25 - 162	0.0	0.0 - 0.0	17.5	5.9 - 45.6	9.0	2.5 - 26.4	23.2	9.3 - 62.5	0.0	0.0 - 0.0	17.2	6.1 - 39.4	0.44
6020004	17302	SEQUATCHIE R	109.6	60.1	26 - 105	0.0	0.0 - 0.0	12.9	5.2 - 25.6	8.9	3.0 - 22.2	23.7	9.7 - 71.2	0.0	0.0 - 0.0	14.6	6.4 - 34.8	0.44
6020004	17303	SEQUATCHIE R	18.1	70.6	22 - 160	0.0	0.0 - 0.0	6.4	1.6 - 16.4	12.7	3.3 - 34.0	35.2	10.7 - 95.9	0.0	0.0 - 0.0	16.2	5.7 - 44.4	0.42
6020004	17304	SEQUATCHIE R	34.5	51.1	17 - 125	0.0	0.0 - 0.0	6.2	2.3 - 13.4	5.8	1.8 - 18.2	22.9	7.0 - 58.5	0.0	0.0 - 0.0	16.2	5.1 - 41.9	0.42
6020004	17305	SEQUATCHIE R	60.4	74.6	26 - 142	15.4	5.3 - 30.9	15.8	5.1 - 31.5	5.7	1.4 - 14.4	23.1	7.9 - 55.9	0.0	0.0 - 0.0	14.7	5.0 - 30.9	0.41
6020004	17306	SEQUATCHIE R	93.4	51.6	16 - 106	0.0	0.0 - 0.0	8.3	2.8 - 18.2	7.2	1.9 - 21.6	22.1	6.4 - 51.3	0.0	0.0 - 0.0	14.0	4.5 - 31.7	0.40
6020004	17307	SEQUATCHIE R	392.3	49.8	17 - 88	1.6	0.5 - 3.0	7.0	2.4 - 12.2	8.6	2.7 - 21.7	20.7	7.0 - 44.8	0.0	0.0 - 0.0	12.0	4.0 - 23.8	0.39
6020004	17308	MAISE CR	46.8	36.8	13 - 73	0.0	0.0 - 0.0	7.3	2.6 - 16.0	5.0	1.4 - 13.6	11.5	3.8 - 31.9	0.0	0.0 - 0.0	13.0	5.2 - 29.2	0.39
6020004	17309	BIG BRUSH CR	13.8	73.5	22 - 223	0.0	0.0 - 0.0	26.4	8.0 - 73.8	6.9	1.6 - 24.8	27.4	9.4 - 86.8	0.0	0.0 - 0.0	12.8	3.9 - 39.4	0.40
6020004	17310	BIG BRUSH CR	124.6	21.5	6 - 42	0.0	0.0 - 0.0	3.2	1.0 - 6.5	1.4	0.4 - 3.5	2.3	0.7 - 5.2	0.0	0.0 - 0.0	14.6	4.4 - 29.4	0.39
6020004	17311	LITTLE BRUSH CR	40.8	27.0	8 - 58	0.0	0.0 - 0.0	7.1	1.7 - 14.7	1.7	0.5 - 3.8	3.6	1.0 - 9.9	0.0	0.0 - 0.0	14.6	4.7 - 32.0	0.39
6020004	17312	WOODCOCK CR	69.7	29.5	12 - 54	0.0	0.0 - 0.0	6.4	2.4 - 12.5	2.5	0.8 - 5.9	8.1	2.9 - 19.9	0.0	0.0 - 0.0	12.6	4.8 - 27.0	0.41
6020004	17313	HICKS CR	60.7	25.0	7 - 56	0.0	0.0 - 0.0	4.0	1.2 - 8.7	1.6	0.4 - 4.9	5.1	1.7 - 12.2	0.0	0.0 - 0.0	14.3	4.3 - 34.8	0.42

8-digit HUC	ID	Name	Area	Catchment Yield		Point sources		Developed Land		Manure		Agricultural Land		Phosphate Mines		Soil parent rock		Frac
				kg/km^2	90% CI	kg/km^2	90% CI	kg/km^2	90% CI	kg/km^2	90% CI	kg/km^2	90% CI	kg/km^2	90% CI	kg/km^2	90% CI	
6020004	17314	GRIFFITH CR	28.6	38.1	15 - 83	0.0	0.0 - 0.0	10.7	4.2 - 24.8	3.9	1.4 - 10.6	10.3	3.4 - 24.7	0.0	0.0 - 0.0	13.3	5.0 - 33.5	0.42
6020004	17315	LITTLE SEQUATCH	97.1	36.7	15 - 81	0.0	0.0 - 0.0	7.3	2.5 - 19.6	5.1	1.8 - 14.3	12.7	4.7 - 29.9	0.0	0.0 - 0.0	11.6	4.4 - 28.9	0.44
6020004	17316	LITTLE SEQUATCH	2.3	10.5	3 - 22	0.0	0.0 - 0.0	0.0	0.0 - 0.0	0.0	0.0 - 0.0	0.0	0.0 - 0.0	0.0	0.0 - 0.0	10.5	2.9 - 22.4	0.42
6020004	17317	POCKET CR	40.9	24.8	8 - 50	0.0	0.0 - 0.0	4.9	1.6 - 10.3	1.8	0.6 - 4.4	3.7	1.1 - 11.1	0.0	0.0 - 0.0	14.4	4.7 - 35.0	0.41
6020004	17318	LITTLE SEQUATCH	24.1	21.1	7 - 37	0.0	0.0 - 0.0	3.2	1.1 - 6.2	1.1	0.4 - 2.5	2.5	0.8 - 6.5	0.0	0.0 - 0.0	14.2	4.5 - 30.7	0.41
6020004	17319	LAUREL CR	32.1	17.4	6 - 43	0.0	0.0 - 0.0	1.9	0.6 - 4.1	0.9	0.3 - 2.6	1.5	0.5 - 4.2	0.0	0.0 - 0.0	13.0	4.5 - 36.5	0.40
6020004	17320	LITTLE SEQUATCH	113.0	23.4	9 - 41	0.0	0.0 - 0.0	4.4	1.6 - 8.4	2.6	0.9 - 6.5	4.0	1.3 - 9.5	0.0	0.0 - 0.0	12.3	4.6 - 25.1	0.40
6020004	17321	INDIAN CR	31.3	18.0	6 - 31	0.0	0.0 - 0.0	3.2	1.1 - 6.0	1.2	0.3 - 2.6	2.9	1.0 - 6.5	0.0	0.0 - 0.0	10.8	3.5 - 20.8	0.42
6030001	17322	SHORT CR	114.1	142.0	37 - 273	75.7	19.0 - 160.7	30.1	7.2 - 60.3	15.4	3.5 - 41.1	16.0	3.7 - 42.8	0.0	0.0 - 0.0	4.7	1.3 - 10.9	0.44
6030001	17323	SHORT CR	93.4	47.9	15 - 91	0.0	0.0 - 0.0	12.1	4.1 - 23.7	12.5	3.5 - 30.9	18.7	5.5 - 43.5	0.0	0.0 - 0.0	4.6	1.6 - 10.1	0.41
6030001	17324	CROSS CR	83.9	54.1	18 - 90	0.0	0.0 - 0.0	8.6	3.1 - 15.2	21.3	6.9 - 55.4	19.8	7.1 - 43.3	0.0	0.0 - 0.0	4.4	1.5 - 8.8	0.41
6030001	17325	SCARHAM CR	83.8	52.4	17 - 101	0.0	0.0 - 0.0	7.0	2.3 - 13.4	20.7	5.7 - 53.6	20.0	6.3 - 49.6	0.0	0.0 - 0.0	4.7	1.4 - 8.6	0.44
6030001	17326	WHIPPOORWILL C	10.9	43.5	14 - 92	0.0	0.0 - 0.0	7.7	2.5 - 18.1	15.2	4.6 - 38.6	15.9	5.3 - 44.0	0.0	0.0 - 0.0	4.7	1.4 - 12.6	0.44
6030001	17327	TOWN CR	228.7	54.1	19 - 101	0.0	0.0 - 0.0	8.9	3.1 - 18.0	21.0	6.2 - 46.7	19.5	6.7 - 48.0	0.0	0.0 - 0.0	4.7	1.8 - 9.5	0.44
6030001	17328	TOWN CR	123.2	49.8	16 - 119	0.0	0.0 - 0.0	7.8	2.7 - 22.4	19.0	6.0 - 49.4	17.6	5.7 - 51.3	0.0	0.0 - 0.0	5.3	1.6 - 14.2	0.40
6030001	17329	BENGIS CR	66.3	52.9	16 - 108	0.0	0.0 - 0.0	8.1	2.6 - 14.9	20.5	6.1 - 55.1	19.2	5.6 - 53.4	0.0	0.0 - 0.0	5.0	1.7 - 11.7	0.40
6030001	17330	BLACK OAK CR	73.7	56.2	15 - 116	0.0	0.0 - 0.0	8.0	2.5 - 17.0	22.6	5.9 - 56.5	20.9	5.5 - 55.9	0.0	0.0 - 0.0	4.6	1.3 - 11.5	0.44
6030001	17331	S SAUTY CR	6.9	54.6	18 - 104	0.0	0.0 - 0.0	7.5	2.3 - 15.4	18.1	5.0 - 56.7	23.7	8.3 - 60.4	0.0	0.0 - 0.0	5.3	1.8 - 12.2	0.43
6030001	17332	PINEY CR	114.7	57.2	16 - 124	0.2	0.1 - 0.5	11.1	3.5 - 24.7	20.9	5.8 - 56.7	20.1	6.1 - 61.8	0.0	0.0 - 0.0	4.8	1.2 - 11.6	0.42
6030001	17333	S SAUTY CR	43.1	47.9	18 - 102	0.0	0.0 - 0.0	8.1	3.0 - 17.8	13.7	4.7 - 38.2	21.2	6.8 - 56.8	0.0	0.0 - 0.0	4.9	1.9 - 11.7	0.42
6030001	17334	KIRBY CR	98.6	44.3	13 - 97	0.0	0.0 - 0.0	9.9	2.8 - 19.4	7.5	2.2 - 23.4	21.6	6.1 - 52.6	0.0	0.0 - 0.0	5.2	1.8 - 12.0	0.43
6030001	17335	TENNESSEE R	5.3	20.0	7 - 42	0.0	0.0 - 0.0	0.0	0.0 - 0.0	1.6	0.5 - 4.2	4.0	1.3 - 10.1	0.0	0.0 - 0.0	14.4	5.3 - 36.5	0.44
6030001	17336	COON CR	229.7	29.9	10 - 62	0.0	0.0 - 0.0	5.2	1.7 - 12.3	7.4	2.4 - 22.0	11.5	3.9 - 30.7	0.0	0.0 - 0.0	5.8	2.0 - 13.1	0.44
6030001	17337	TENNESSEE R	27.4	77.6	28 - 166	0.0	0.0 - 0.0	4.0	1.4 - 8.1	11.9	3.7 - 36.0	32.8	11.4 - 85.0	0.0	0.0 - 0.0	28.9	10.3 - 67.1	0.44
6030001	17338	TENNESSEE R	53.2	174.8	69 - 393	97.3	38.6 - 244.7	8.3	3.1 - 17.9	11.4	3.6 - 36.4	31.9	11.1 - 89.5	0.0	0.0 - 0.0	25.7	9.3 - 63.6	0.44
6030001	17339	TENNESSEE R	8.7	49.9	17 - 121	0.0	0.0 - 0.0	6.4	1.9 - 15.2	4.2	1.2 - 11.6	9.8	2.5 - 24.8	0.0	0.0 - 0.0	29.5	9.8 - 78.7	0.44
6030001	17340	LONG ISLAND CR	28.5	18.3	7 - 37	0.0	0.0 - 0.0	1.3	0.5 - 2.5	2.3	0.8 - 5.4	6.5	2.2 - 14.0	0.0	0.0 - 0.0	8.2	3.3 - 17.8	0.44
6030001	17341	MILLER CR	125.5	35.0	12 - 75	0.0	0.0 - 0.0	8.0	2.5 - 17.9	8.3	2.6 - 22.7	13.3	4.3 - 34.2	0.0	0.0 - 0.0	5.5	1.9 - 14.7	0.43
6030001	17342	LONG ISLAND CR	65.6	32.7	12 - 64	0.0	0.0 - 0.0	7.8	2.5 - 15.4	5.5	1.8 - 13.8	13.9	4.3 - 34.1	0.0	0.0 - 0.0	5.6	1.9 - 13.3	0.43
6030001	17343	TENNESSEE R	43.5	94.6	34 - 218	17.3	6.4 - 45.4	15.4	4.9 - 41.1	9.6	3.3 - 30.2	27.8	9.2 - 71.9	0.0	0.0 - 0.0	24.4	8.8 - 70.8	0.44
6030001	17344	TENNESSEE R	18.7	118.5	43 - 250	41.5	15.0 - 93.1	32.6	11.0 - 79.6	8.9	2.8 - 20.5	24.2	9.3 - 58.0	0.0	0.0 - 0.0	11.3	3.8 - 23.7	0.44
6030001	17345	TENNESSEE R	21.4	101.1	31 - 231	24.8	7.9 - 65.0	21.4	6.8 - 47.2	10.8	3.3 - 36.3	28.1	8.3 - 83.3	0.0	0.0 - 0.0	16.2	5.3 - 44.9	0.44
6030001	17346	BATTLE CR	29.0	42.2	13 - 87	0.0	0.0 - 0.0	20.0	5.8 - 45.8	3.0	0.8 - 10.2	7.2	2.3 - 19.0	0.0	0.0 - 0.0	12.0	3.7 - 27.3	0.44
6030001	17347	BATTLE CR	32.9	33.5	10 - 69	0.0	0.0 - 0.0	8.1	2.3 - 18.1	3.9	1.0 - 10.6	9.5	3.2 - 22.8	0.0	0.0 - 0.0	12.1	3.8 - 27.8	0.43
6030001	17348	BIG FIERY GIZZAR	136.7	29.8	11 - 61	0.0	0.0 - 0.0	7.9	2.8 - 17.9	3.0	1.0 - 8.6	5.4	1.8 - 13.8	0.0	0.0 - 0.0	13.5	4.7 - 30.9	0.41
6030001	17349	BATTLE CR	62.8	23.8	9 - 48	0.0	0.0 - 0.0	4.0	1.5 - 9.4	2.1	0.7 - 5.9	5.8	1.9 - 19.2	0.0	0.0 - 0.0	11.8	4.7 - 28.4	0.41
6030001	17350	BATTLE CR	35.3	33.8	11 - 69	0.0	0.0 - 0.0	12.8	4.1 - 28.6	3.0	0.8 - 7.0	5.4	1.8 - 14.2	0.0	0.0 - 0.0	12.5	3.8 - 26.1	0.37
6030001	17351	BATTLE CR, W FK	60.9	27.7	12 - 73	0.0	0.0 - 0.0	9.2	3.4 - 20.9	1.6	0.6 - 5.0	3.8	1.2 - 10.4	0.0	0.0 - 0.0	13.2	5.0 - 37.1	0.37
6030001	17352	SWEDEN CR	80.9	22.3	8 - 53	0.0	0.0 - 0.0	2.1	0.7 - 4.3	2.4	0.8 - 6.4	6.5	1.9 - 17.3	0.0	0.0 - 0.0	11.3	4.4 - 27.6	0.43

8-digit HUC	ID	Name	Area	Catchment Yield		Point sources		Developed Land		Manure		Agricultural Land		Phosphate Mines		Soil parent rock		Frac
				kg/km^2	90% CI	kg/km^2	90% CI	kg/km^2	90% CI	kg/km^2	90% CI	kg/km^2	90% CI	kg/km^2	90% CI	kg/km^2	90% CI	
6030001	17353	JONES CR	57.8	45.1	15 - 99	0.0	0.0 - 0.0	12.5	3.8 - 30.1	5.3	1.5 - 14.2	15.4	4.8 - 40.1	0.0	0.0 - 0.0	11.9	4.2 - 27.9	0.44
6030001	17354	WIDOW'S CR	109.9	49.4	16 - 100	0.0	0.0 - 0.0	10.3	3.3 - 22.0	6.3	1.8 - 17.9	17.8	5.3 - 47.9	0.0	0.0 - 0.0	15.0	4.9 - 36.4	0.44
6030001	17355	CROW CR	78.2	61.1	15 - 159	0.0	0.0 - 0.0	11.0	2.9 - 33.0	9.5	2.5 - 28.7	27.0	7.8 - 75.9	0.0	0.0 - 0.0	13.7	3.7 - 40.3	0.44
6030001	17356	CROW CR	412.6	23.5	8 - 50	0.0	0.0 - 0.0	2.7	1.0 - 6.1	2.6	0.9 - 6.9	6.2	1.8 - 19.2	0.0	0.0 - 0.0	12.0	4.5 - 27.9	0.44
6030001	17357	BIG COON CR	7.3	33.7	12 - 57	0.0	0.0 - 0.0	2.8	1.1 - 4.9	5.3	1.8 - 12.1	15.9	5.8 - 37.9	0.0	0.0 - 0.0	9.7	3.7 - 19.9	0.44
6030001	17358	LITTLE COON CR	80.0	22.0	9 - 49	0.0	0.0 - 0.0	1.7	0.6 - 4.3	2.5	0.8 - 8.7	6.6	2.5 - 15.3	0.0	0.0 - 0.0	11.3	4.5 - 24.5	0.43
6030001	17359	BIG COON CR	132.6	20.9	7 - 40	0.0	0.0 - 0.0	2.1	0.7 - 4.1	2.4	0.7 - 5.8	6.3	2.1 - 14.4	0.0	0.0 - 0.0	10.1	3.7 - 23.8	0.43
6030001	17360	MUD CR	210.6	58.7	18 - 133	0.0	0.0 - 0.0	3.5	1.0 - 7.6	10.5	2.8 - 26.7	30.1	8.4 - 80.5	0.0	0.0 - 0.0	14.7	4.7 - 35.0	0.44
6030002	17361	W FLINT CR	252.0	72.6	23 - 167	0.0	0.0 - 0.0	8.1	2.6 - 20.3	16.3	4.6 - 55.3	38.4	11.6 - 97.1	0.0	0.0 - 0.0	9.8	3.5 - 24.1	0.55
6030002	17362	ELAM CR	79.6	103.2	37 - 227	0.0	0.0 - 0.0	6.6	2.1 - 17.7	23.6	6.6 - 70.7	56.3	20.0 - 146.9	0.0	0.0 - 0.0	16.6	5.7 - 40.7	0.48
6030002	17363	W FLINT CR	95.9	36.2	10 - 89	0.0	0.0 - 0.0	3.7	1.0 - 8.6	7.3	1.9 - 17.4	17.4	4.8 - 50.9	0.0	0.0 - 0.0	7.8	2.3 - 18.8	0.48
6030002	17364	FLINT CR	11.8	56.9	19 - 118	0.0	0.0 - 0.0	10.4	3.4 - 23.9	12.4	3.6 - 34.4	28.2	9.3 - 72.6	0.0	0.0 - 0.0	5.9	2.0 - 15.7	0.55
6030002	17365	NO BUSINESS CR	94.8	124.8	42 - 290	0.0	0.0 - 0.0	6.4	2.2 - 16.8	30.4	8.2 - 82.3	69.1	21.8 - 202.2	0.0	0.0 - 0.0	18.8	6.4 - 51.3	0.54
6030002	17366	FLINT CR	231.2	94.4	31 - 196	11.5	3.6 - 26.7	12.3	3.7 - 22.8	18.5	5.3 - 59.8	40.2	13.1 - 104.8	0.0	0.0 - 0.0	11.9	4.1 - 27.9	0.54
6030002	17367	FLINT CR	74.3	61.1	18 - 129	0.0	0.0 - 0.0	6.9	2.1 - 16.5	15.6	4.1 - 41.1	28.6	7.7 - 80.1	0.0	0.0 - 0.0	10.0	3.3 - 22.0	0.51
6030002	17368	FLINT CR	98.8	49.0	17 - 95	0.1	0.0 - 0.2	11.4	4.3 - 22.1	16.7	5.6 - 38.9	15.8	5.1 - 41.1	0.0	0.0 - 0.0	5.1	1.9 - 10.6	0.49
6030002	17369	MILL CR	51.6	25.8	8 - 70	0.0	0.0 - 0.0	4.6	1.5 - 11.7	5.1	1.5 - 15.7	11.4	3.4 - 35.3	0.0	0.0 - 0.0	4.6	1.4 - 12.1	0.49
6030002	17370	CEDAR CR	69.5	78.9	25 - 163	0.0	0.0 - 0.0	9.7	3.1 - 21.2	17.7	4.9 - 43.3	39.4	12.5 - 86.0	0.0	0.0 - 0.0	12.1	4.4 - 27.8	0.51
6030002	17371	COTACO CR	215.5	60.6	20 - 127	0.0	0.0 - 0.0	6.6	2.3 - 12.7	13.5	3.9 - 34.2	30.1	10.7 - 78.4	0.0	0.0 - 0.0	10.4	3.7 - 25.7	0.55
6030002	17372	SIXMILE CR	42.3	34.8	10 - 71	0.0	0.0 - 0.0	4.3	1.2 - 9.3	6.9	2.1 - 20.5	15.8	4.6 - 36.5	0.0	0.0 - 0.0	7.9	2.6 - 17.0	0.52
6030002	17373	COTACO CR	11.7	56.6	20 - 128	0.0	0.0 - 0.0	8.0	3.0 - 17.5	11.1	3.0 - 28.9	25.0	7.8 - 73.3	0.0	0.0 - 0.0	12.5	4.6 - 31.9	0.52
6030002	17374	COTACO CR, W FK	19.5	33.0	10 - 62	0.0	0.0 - 0.0	6.1	1.7 - 12.9	6.5	1.9 - 13.7	14.3	4.8 - 36.1	0.0	0.0 - 0.0	6.1	1.7 - 13.5	0.52
6030002	17375	COTACO CR, W FK	70.3	31.0	11 - 61	0.0	0.0 - 0.0	5.4	1.8 - 12.7	7.0	2.1 - 18.9	13.6	4.5 - 32.2	0.0	0.0 - 0.0	4.9	1.8 - 11.6	0.51
6030002	17376	MUD CR	50.0	43.1	12 - 118	0.0	0.0 - 0.0	6.2	1.9 - 16.1	13.0	3.8 - 41.1	19.1	5.6 - 58.5	0.0	0.0 - 0.0	4.9	1.5 - 14.1	0.51
6030002	17377	COTACO CR	203.5	38.8	14 - 72	0.0	0.0 - 0.0	10.3	3.6 - 18.9	9.7	3.1 - 25.6	13.6	4.3 - 31.3	0.0	0.0 - 0.0	5.2	1.9 - 11.3	0.52
6030002	17378	TENNESSEE R	25.0	192.7	59 - 429	120.0	39.0 - 286.3	3.3	0.9 - 6.8	11.3	3.1 - 33.3	29.7	8.7 - 79.2	0.0	0.0 - 0.0	28.5	9.2 - 73.9	0.55
6030002	17379	TENNESSEE R	78.5	159.7	61 - 273	90.4	33.5 - 177.2	18.9	6.4 - 32.4	8.3	2.8 - 22.8	23.4	7.6 - 54.9	0.0	0.0 - 0.0	18.7	6.7 - 41.2	0.55
6030002	17380	TENNESSEE R	52.0	47.3	16 - 116	0.0	0.0 - 0.0	7.8	2.4 - 18.0	6.3	1.7 - 19.4	20.1	6.5 - 54.1	0.0	0.0 - 0.0	13.2	4.5 - 33.5	0.55
6030002	17381	TENNESSEE R	49.3	31.9	11 - 52	0.0	0.0 - 0.0	5.0	1.6 - 8.6	7.2	2.2 - 17.4	12.7	4.1 - 30.7	0.0	0.0 - 0.0	7.1	2.2 - 13.9	0.55
6030002	17382	PAINT ROCK CR	433.1	49.4	18 - 128	0.0	0.0 - 0.0	6.0	2.1 - 14.7	10.0	3.2 - 29.4	22.2	7.2 - 60.4	0.0	0.0 - 0.0	11.1	4.3 - 29.6	0.55
6030002	17383	PAINT ROCK CR	39.2	30.3	8 - 70	0.0	0.0 - 0.0	2.0	0.5 - 4.6	4.8	1.2 - 14.2	14.3	3.7 - 41.8	0.0	0.0 - 0.0	9.3	2.7 - 23.0	0.52
6030002	17384	GUESS CR	87.4	26.6	10 - 63	0.0	0.0 - 0.0	3.2	1.2 - 7.1	3.4	1.0 - 9.2	9.8	3.2 - 25.8	0.0	0.0 - 0.0	10.1	4.0 - 23.9	0.51
6030002	17385	PAINT ROCK CR	17.8	30.0	11 - 51	0.0	0.0 - 0.0	2.4	0.8 - 4.6	3.9	1.2 - 10.0	11.4	4.6 - 24.1	0.0	0.0 - 0.0	12.2	4.4 - 26.7	0.51
6030002	17386	PAINT ROCK CR	94.5	19.0	7 - 43	0.0	0.0 - 0.0	1.3	0.5 - 2.9	2.3	0.8 - 6.7	6.5	2.3 - 16.6	0.0	0.0 - 0.0	8.9	2.9 - 20.9	0.50
6030002	17387	PAINT ROCK CR	67.7	19.3	6 - 47	0.1	0.0 - 0.3	1.8	0.5 - 4.7	2.2	0.6 - 6.6	6.1	1.9 - 14.3	0.0	0.0 - 0.0	9.1	2.7 - 26.2	0.48
6030002	17388	PAINT ROCK CR	398.5	16.4	6 - 30	0.0	0.0 - 0.0	1.7	0.6 - 3.6	1.6	0.5 - 4.1	4.0	1.4 - 10.3	0.0	0.0 - 0.0	9.1	3.3 - 21.6	0.46
6030002	17389	CLEAR CR	48.3	20.2	6 - 36	0.0	0.0 - 0.0	1.5	0.5 - 2.9	2.5	0.7 - 6.6	7.3	2.4 - 16.4	0.0	0.0 - 0.0	8.8	3.2 - 20.3	0.52
6030002	17390	FLINT R	256.2	64.6	23 - 164	3.5	1.2 - 9.7	12.5	4.3 - 36.5	3.6	1.2 - 10.9	30.0	9.9 - 79.5	0.0	0.0 - 0.0	14.9	5.2 - 41.5	0.55
6030002	17391	HURRICANE CR	186.1	44.6	11 - 103	0.0	0.0 - 0.0	5.8	1.5 - 12.9	2.8	0.7 - 8.7	23.3	6.0 - 55.9	0.0	0.0 - 0.0	12.7	3.4 - 31.6	0.53

8-digit HUC	ID	Name	Area	Catchment Yield		Point sources		Developed Land		Manure		Agricultural Land		Phosphate Mines		Soil parent rock		Frac
				kg/km^2	90% CI	kg/km^2	90% CI	kg/km^2	90% CI	kg/km^2	90% CI	kg/km^2	90% CI	kg/km^2	90% CI	kg/km^2	90% CI	
6030002	17392	FLINT R	54.5	59.2	17 - 140	0.0	0.0 - 0.0	9.6	2.8 - 22.0	3.7	0.9 - 10.1	30.9	9.0 - 87.4	0.0	0.0 - 0.0	15.0	4.7 - 37.1	0.53
6030002	17393	FLINT R	27.2	86.8	30 - 168	0.0	0.0 - 0.0	9.7	3.2 - 19.4	5.9	1.9 - 15.7	49.6	16.9 - 116.8	0.0	0.0 - 0.0	21.5	7.3 - 50.9	0.51
6030002	17394	FLINT R, MOUNTA	363.5	179.0	60 - 416	0.0	0.0 - 0.0	11.0	3.5 - 25.8	30.1	8.3 - 94.0	100.6	35.8 - 280.1	0.0	0.0 - 0.0	37.2	11.3 - 95.7	0.50
6030002	17395	FLINT R, MOUNTA	217.0	88.9	28 - 180	0.0	0.0 - 0.0	6.4	1.8 - 14.0	10.2	3.0 - 29.7	51.8	15.8 - 124.1	0.0	0.0 - 0.0	20.7	6.5 - 44.5	0.50
6030002	17396	FLINT R, BRIER FK	41.8	117.2	42 - 231	0.0	0.0 - 0.0	16.3	5.7 - 29.7	8.1	2.7 - 25.5	68.3	24.3 - 156.4	0.0	0.0 - 0.0	24.6	8.0 - 58.0	0.51
6030002	17397	FLINT R, BRIER FK	131.3	145.9	43 - 319	0.0	0.0 - 0.0	10.7	2.7 - 20.7	14.1	3.4 - 36.1	89.8	26.7 - 197.3	0.0	0.0 - 0.0	31.3	9.7 - 70.8	0.50
6030002	17398	BEAVERDAM CR	113.1	106.0	44 - 241	0.0	0.0 - 0.0	13.2	5.1 - 30.4	7.4	2.7 - 23.2	61.3	23.3 - 152.0	0.0	0.0 - 0.0	24.1	10.6 - 62.2	0.50
6030002	17399	ALDRIDGE CR	60.6	82.7	23 - 186	0.0	0.0 - 0.0	60.3	17.6 - 131.2	1.2	0.3 - 3.5	9.7	3.0 - 22.0	0.0	0.0 - 0.0	11.6	3.5 - 27.1	0.55
6030002	17400	INDIAN CR	24.7	79.2	29 - 144	0.0	0.0 - 0.0	21.7	6.8 - 45.0	3.1	1.0 - 8.2	24.0	8.7 - 55.5	0.0	0.0 - 0.0	30.5	11.6 - 69.0	0.55
6030002	17401	INDIAN CR, HUNT[illegible]	20.2	78.6	25 - 153	0.0	0.0 - 0.0	35.8	10.4 - 81.4	2.2	0.7 - 6.9	16.8	4.8 - 43.2	0.0	0.0 - 0.0	23.9	7.0 - 61.3	0.54
6030002	17402	INDIAN CR, HUNT[illegible]	152.7	256.6	95 - 551	140.0	50.2 - 292.2	90.5	30.3 - 188.9	1.0	0.3 - 2.8	7.7	2.5 - 17.9	0.0	0.0 - 0.0	17.4	5.7 - 42.6	0.52
6030002	17403	*A	41.5	130.2	45 - 246	17.2	5.6 - 41.1	80.9	27.5 - 157.9	1.3	0.3 - 3.5	11.4	4.4 - 27.8	0.0	0.0 - 0.0	19.3	6.3 - 51.5	0.52
6030002	17404	INDIAN CR	183.4	94.5	30 - 190	0.0	0.0 - 0.0	37.0	11.4 - 78.2	4.0	1.1 - 10.4	33.8	10.3 - 83.8	0.0	0.0 - 0.0	19.6	6.4 - 50.0	0.54
6030002	17405	LIMESTONE CR	87.0	161.1	53 - 403	0.0	0.0 - 0.0	8.5	2.7 - 20.0	17.8	4.9 - 57.1	95.1	27.5 - 265.2	0.0	0.0 - 0.0	39.7	11.7 - 109.7	0.49
6030002	17406	LITTLE LIMESTON	86.4	201.9	58 - 486	0.0	0.0 - 0.0	8.4	2.6 - 20.8	28.0	7.6 - 85.2	118.8	33.0 - 311.3	0.0	0.0 - 0.0	46.7	12.9 - 124.4	0.49
6030003	17407	ELK R	27.1	792.4	264 - 1807	0.0	0.0 - 0.0	13.0	4.4 - 28.5	19.8	5.4 - 54.5	50.0	16.5 - 126.8	0.0	0.0 - 0.0	709.6	235.5 - 1678	0.54
6030003	17408	ELK R	1.0	469.8	144 - 1016	0.0	0.0 - 0.0	0.0	0.0 - 0.0	29.9	8.6 - 90.2	77.6	23.4 - 183.1	0.0	0.0 - 0.0	362.4	111.8 - 855.[illegible]	0.53
6030003	17409	KELLY CR	39.2	201.0	75 - 437	0.0	0.0 - 0.0	6.8	2.3 - 14.8	12.3	3.6 - 32.0	31.7	11.2 - 84.0	0.0	0.0 - 0.0	150.3	58.2 - 359.5	0.53
6030003	17410	ELK R	17.6	329.3	96 - 755	0.0	0.0 - 0.0	4.5	1.5 - 9.2	15.6	3.2 - 48.0	39.5	12.4 - 113.1	0.0	0.0 - 0.0	269.7	84.5 - 623.5	0.53
6030003	17411	ELK R	33.9	269.9	86 - 705	0.0	0.0 - 0.0	5.4	1.9 - 13.1	9.5	2.8 - 30.5	25.5	8.5 - 67.6	0.0	0.0 - 0.0	229.4	70.9 - 598.5	0.53
6030003	17412	COLDWATER CR	79.7	172.6	71 - 366	0.0	0.0 - 0.0	7.0	2.3 - 14.1	10.5	3.5 - 26.7	27.9	10.5 - 62.8	0.0	0.0 - 0.0	127.3	48.8 - 275.5	0.53
6030003	17413	ELK R	29.2	392.2	149 - 782	0.0	0.0 - 0.0	6.1	2.3 - 11.6	9.4	3.1 - 20.7	24.2	9.3 - 52.4	0.0	0.0 - 0.0	352.4	128.4 - 747.[illegible]	0.53
6030003	17414	ELK R	92.9	653.2	184 - 1457	0.0	0.0 - 0.0	6.7	1.8 - 12.5	17.5	5.3 - 52.5	46.9	14.8 - 109.9	0.0	0.0 - 0.0	582.1	168.7 - 1443	0.53
6030003	17415	ELK R	15.7	874.5	268 - 2058	0.0	0.0 - 0.0	44.7	13.9 - 99.4	16.4	4.5 - 46.9	42.7	12.5 - 114.9	0.0	0.0 - 0.0	770.6	225.2 - 1839	0.52
6030003	17416	ELK R	74.8	548.2	190 - 1152	29.0	10.2 - 70.1	10.2	3.2 - 22.0	14.8	4.5 - 40.2	39.3	13.7 - 104.1	0.0	0.0 - 0.0	454.9	166.1 - 987.[illegible]	0.51
6030003	17417	ELK R	144.7	298.5	122 - 797	0.0	0.0 - 0.0	6.2	2.6 - 15.4	11.5	4.1 - 33.4	28.7	12.1 - 85.4	0.0	0.0 - 0.0	252.1	98.4 - 704.3	0.51
6030003	17418	BEAN'S CR	54.2	150.4	49 - 369	0.0	0.0 - 0.0	6.2	2.0 - 14.8	24.1	7.4 - 72.6	55.3	17.4 - 162.0	0.0	0.0 - 0.0	64.8	20.7 - 176.1	0.49
6030003	17419	BEAN'S CR	125.7	97.9	30 - 210	0.0	0.0 - 0.0	7.0	2.2 - 15.2	21.9	5.8 - 69.0	48.9	14.2 - 123.1	0.0	0.0 - 0.0	20.1	6.4 - 50.9	0.45
6030003	17420	*A	42.0	112.6	42 - 258	0.0	0.0 - 0.0	8.4	2.9 - 19.6	26.3	8.5 - 100.4	58.4	21.1 - 171.8	0.0	0.0 - 0.0	19.5	7.1 - 47.8	0.45
6030003	17421	ELK R	104.1	262.0	98 - 576	0.0	0.0 - 0.0	7.0	2.4 - 14.6	22.5	7.2 - 60.0	42.6	12.2 - 130.9	0.0	0.0 - 0.0	189.9	71.5 - 447.9	0.49
6030003	17422	ELK R	29.1	145.8	57 - 329	0.0	0.0 - 0.0	8.4	2.8 - 20.7	31.7	10.5 - 101.1	79.1	30.3 - 194.8	0.0	0.0 - 0.0	26.6	9.8 - 66.7	0.09
6030003	17423	MUD CR	51.7	55.2	19 - 119	0.0	0.0 - 0.0	7.4	2.5 - 18.9	10.9	3.7 - 29.8	24.2	8.2 - 72.6	0.0	0.0 - 0.0	12.7	5.3 - 30.6	0.09
6030003	17424	ELK R	67.1	89.5	28 - 170	0.0	0.0 - 0.0	8.4	2.8 - 14.9	17.7	5.1 - 44.5	46.8	15.6 - 113.6	0.0	0.0 - 0.0	16.6	6.1 - 34.5	0.09
6030003	17425	DRY CR	56.1	56.5	18 - 142	9.5	3.3 - 24.1	12.9	4.1 - 29.9	7.9	2.7 - 20.6	12.5	3.5 - 33.4	0.0	0.0 - 0.0	13.7	4.4 - 37.2	0.09
6030003	17426	ELK R	16.4	122.3	31 - 220	0.0	0.0 - 0.0	9.6	2.7 - 18.7	34.9	7.8 - 103.8	56.4	14.1 - 131.8	0.0	0.0 - 0.0	21.4	5.3 - 53.7	0.09
6030003	17427	DRY CR	66.6	34.5	11 - 89	0.0	0.0 - 0.0	4.5	1.4 - 12.2	6.0	1.7 - 19.0	9.3	2.6 - 27.4	0.0	0.0 - 0.0	14.7	4.3 - 36.9	0.09
6030003	17428	ELK R	89.2	41.0	13 - 104	0.0	0.0 - 0.0	3.5	1.0 - 8.2	8.6	2.5 - 23.0	13.7	4.5 - 36.7	0.0	0.0 - 0.0	15.1	5.0 - 37.3	0.09
6030003	17429	BEAN CR	54.1	65.0	22 - 146	0.0	0.0 - 0.0	5.0	1.5 - 13.3	10.2	3.5 - 31.2	35.5	12.6 - 92.0	0.0	0.0 - 0.0	14.2	4.9 - 33.7	0.09
6030003	17430	MULBERRY CR	20.7	797.8	307 - 1927	0.0	0.0 - 0.0	5.2	1.8 - 13.0	17.6	5.7 - 45.9	47.5	18.4 - 113.2	0.0	0.0 - 0.0	727.4	273.5 - 1914	0.51

8-digit HUC	ID	Name	Area	Catchment Yield		Point sources		Developed Land		Manure		Agricultural Land		Phosphate Mines		Soil parent rock		Frac
				kg/km^2	90% CI	kg/km^2	90% CI	kg/km^2	90% CI	kg/km^2	90% CI	kg/km^2	90% CI	kg/km^2	90% CI	kg/km^2	90% CI	
6030003	17431	MULBERRY CR, E	138.1	534.7	167 - 1201	15.2	4.7 - 32.4	8.4	2.8 - 16.5	16.1	4.6 - 40.7	30.4	9.6 - 86.8	0.0	0.0 - 0.0	464.5	139.1 - 1004	0.49
6030003	17432	MULBERRY CR, W	107.3	560.5	180 - 1302	0.0	0.0 - 0.0	6.1	1.9 - 11.7	11.9	3.4 - 33.7	24.9	7.7 - 53.5	0.0	0.0 - 0.0	517.5	162.8 - 1257	0.49
6030003	17433	NORRIS CR	122.4	577.4	177 - 1222	0.0	0.0 - 0.0	11.8	3.4 - 25.0	8.7	2.6 - 23.5	23.0	7.0 - 60.4	0.0	0.0 - 0.0	533.8	163.2 - 1148	0.51
6030003	17434	CANE CR	70.1	783.9	295 - 1683	0.0	0.0 - 0.0	9.2	3.2 - 18.5	16.2	4.9 - 49.0	43.1	14.2 - 125.8	0.0	0.0 - 0.0	715.4	266.0 - 1615	0.52
6030003	17435	PLEASANT VALLE	132.0	578.2	195 - 1461	1.1	0.4 - 3.0	8.2	3.0 - 20.7	8.6	2.3 - 26.6	23.9	8.8 - 64.2	0.0	0.0 - 0.0	536.4	190.2 - 1379	0.48
6030003	17436	CANE CR	30.5	624.1	175 - 1494	0.0	0.0 - 0.0	4.9	1.3 - 11.4	10.0	2.4 - 30.1	28.2	7.7 - 72.6	0.0	0.0 - 0.0	581.0	160.1 - 1396	0.48
6030003	17437	SWAN CR	132.2	663.6	260 - 1557	0.0	0.0 - 0.0	5.6	1.9 - 12.8	10.3	3.5 - 33.4	27.7	9.9 - 73.6	0.0	0.0 - 0.0	619.9	244.7 - 1496	0.53
6030003	17438	BRADSHAW CR	182.6	344.5	109 - 822	0.0	0.0 - 0.0	6.4	1.9 - 13.9	6.9	1.7 - 21.5	17.8	4.8 - 47.3	0.0	0.0 - 0.0	313.4	98.7 - 756.5	0.53
6030003	17439	INDIAN CR	68.9	223.6	74 - 480	0.0	0.0 - 0.0	9.1	3.0 - 18.2	8.9	2.5 - 23.9	22.3	6.7 - 58.1	0.0	0.0 - 0.0	183.3	60.2 - 422.6	0.53
6030004	17440	ELK R	145.8	643.6	196 - 1519	0.0	0.0 - 0.0	6.2	2.0 - 14.2	10.4	2.9 - 31.9	33.9	9.3 - 99.3	0.9	0.3 - 3.4	592.3	182.2 - 1513	0.55
6030004	17441	MILL CR	24.6	737.8	205 - 1392	16.9	4.5 - 34.2	6.2	1.4 - 14.0	9.3	2.1 - 28.1	40.4	10.6 - 93.8	0.0	0.0 - 0.0	665.0	185.9 - 1321	0.54
6030004	17442	ELK R	8.9	954.7	337 - 1908	0.0	0.0 - 0.0	3.8	1.2 - 6.9	18.4	4.8 - 47.4	46.6	15.1 - 95.6	0.0	0.0 - 0.0	886.0	306.7 - 1861	0.54
6030004	17443	ELK R	48.2	516.3	194 - 1330	0.0	0.0 - 0.0	10.8	3.6 - 24.2	12.6	4.2 - 39.7	38.2	13.4 - 100.5	0.0	0.0 - 0.0	454.7	165.5 - 1215	0.54
6030004	17444	RICHLAND CR	50.3	705.5	265 - 1999	0.0	0.0 - 0.0	6.8	2.3 - 16.2	15.9	5.1 - 49.9	40.0	13.8 - 108.1	0.0	0.0 - 0.0	642.8	246.5 - 1780	0.54
6030004	17445	BUCHANON CR	109.6	245.9	99 - 624	0.0	0.0 - 0.0	7.8	3.0 - 17.7	8.9	2.9 - 20.0	22.3	8.1 - 65.2	0.0	0.0 - 0.0	206.9	81.9 - 553.7	0.53
6030004	17446	RICHLAND CR	159.0	616.6	222 - 1275	0.0	0.0 - 0.0	17.1	5.5 - 32.0	12.3	3.5 - 28.6	31.0	9.4 - 77.6	2.5	0.8 - 6.9	553.7	192.7 - 1216	0.53
6030004	17447	RICHLAND CR	51.0	536.6	173 - 1051	0.0	0.0 - 0.0	5.0	1.5 - 10.3	13.5	3.9 - 32.0	33.6	10.8 - 96.9	35.6	10.4 - 112.2	448.8	146.6 - 936.	0.50
6030004	17448	RICHLAND CR	35.5	769.7	244 - 1718	107.6	36.7 - 214.8	8.3	2.5 - 16.2	16.8	5.0 - 44.1	42.0	12.9 - 106.2	2.4	0.7 - 6.4	592.4	184.5 - 1302	0.49
6030004	17449	RICHLAND CR	244.3	640.4	209 - 1439	0.0	0.0 - 0.0	6.9	2.1 - 17.3	12.0	3.7 - 31.0	32.9	10.2 - 88.5	36.1	10.6 - 114.4	552.5	173.7 - 1240	0.48
6030004	17450	LYNN CR	17.1	759.0	247 - 1888	0.0	0.0 - 0.0	8.1	3.0 - 15.4	18.9	5.9 - 53.1	47.1	15.6 - 125.3	0.0	0.0 - 0.0	684.9	216.6 - 1752	0.48
6030004	17451	ROBERTSON FK CI	75.0	601.4	184 - 1377	0.0	0.0 - 0.0	6.3	1.6 - 16.7	11.0	2.7 - 26.7	29.4	8.9 - 78.9	8.4	2.2 - 27.0	546.3	166.4 - 1421	0.46
6030004	17452	LYNN CR	46.1	491.3	132 - 1246	0.0	0.0 - 0.0	6.4	1.8 - 16.2	10.0	2.6 - 33.5	25.7	6.7 - 66.7	38.5	8.4 - 115.0	410.6	111.3 - 1036	0.46
6030004	17453	BIG CR	74.4	522.0	162 - 890	0.0	0.0 - 0.0	5.7	1.7 - 11.1	11.0	3.0 - 23.8	27.6	8.4 - 61.3	22.1	6.2 - 61.8	455.7	141.9 - 827.	0.49
6030004	17454	YOKLEY CR	52.3	279.1	95 - 505	0.0	0.0 - 0.0	4.7	1.5 - 10.0	6.6	2.1 - 17.3	16.9	5.8 - 41.7	37.2	10.6 - 121.0	213.7	72.2 - 393.1	0.45
6030004	17455	BIG CR	98.5	119.2	33 - 229	0.0	0.0 - 0.0	4.5	1.3 - 8.4	8.3	2.1 - 18.2	22.8	5.9 - 54.5	2.4	0.6 - 8.1	81.2	23.3 - 161.0	0.45
6030004	17456	DRY WEAKLEY CI	3.5	1209.5	407 - 2759	0.0	0.0 - 0.0	9.8	2.8 - 20.6	29.0	7.9 - 75.1	73.3	24.8 - 167.3	168.7	49.6 - 460.7	928.8	315.5 - 2383	0.50
6030004	17457	DRY WEAKLEY CI	149.4	153.9	51 - 307	0.0	0.0 - 0.0	5.9	2.0 - 12.8	8.2	2.8 - 19.8	21.7	6.5 - 49.3	0.0	0.0 - 0.0	118.1	37.9 - 260.1	0.50
6030004	17458	AGNEW CR	35.6	250.3	92 - 677	0.0	0.0 - 0.0	5.5	2.0 - 15.7	7.4	2.5 - 21.0	19.0	6.5 - 66.5	0.9	0.3 - 3.3	217.5	78.0 - 664.5	0.50
6030004	17459	SHOAL CR	37.6	242.6	98 - 551	0.0	0.0 - 0.0	5.4	1.8 - 10.7	5.5	1.7 - 14.8	25.1	8.5 - 58.8	0.0	0.0 - 0.0	206.6	79.4 - 467.1	0.55
6030004	17460	SHOAL CR, E FK	66.3	151.4	51 - 362	0.0	0.0 - 0.0	5.9	1.7 - 12.2	6.0	1.6 - 17.7	15.1	4.6 - 36.5	0.0	0.0 - 0.0	124.5	41.7 - 309.0	0.51
6030004	17461	SHOAL CR, W FK	52.3	113.7	41 - 230	0.0	0.0 - 0.0	7.3	2.5 - 15.1	8.4	2.6 - 28.4	21.3	7.9 - 53.3	0.0	0.0 - 0.0	76.8	28.5 - 170.3	0.51
6030004	17462	SUGAR CR, E FK	23.2	75.1	23 - 161	0.0	0.0 - 0.0	8.1	2.5 - 16.4	9.2	2.6 - 24.0	24.1	6.7 - 59.2	0.0	0.0 - 0.0	33.7	10.2 - 79.0	0.52
6030004	17463	SHANNON CR	47.8	73.4	26 - 151	0.0	0.0 - 0.0	7.5	2.6 - 17.1	8.5	2.8 - 22.2	19.7	6.7 - 53.3	0.0	0.0 - 0.0	37.7	12.3 - 86.9	0.50
6030004	17464	SUGAR CR, E FK	96.7	67.4	19 - 150	0.0	0.0 - 0.0	5.9	1.7 - 14.7	7.7	2.0 - 22.6	22.3	6.3 - 64.6	0.0	0.0 - 0.0	31.4	8.6 - 76.4	0.50
6030004	17465	SUGAR CR, W FK	131.4	85.2	25 - 160	0.0	0.0 - 0.0	7.5	2.3 - 16.0	10.9	2.9 - 28.0	33.0	8.7 - 89.7	0.0	0.0 - 0.0	33.6	8.8 - 70.5	0.52
6030004	17466	RICHLAND CR	1.0	1063.6	397 - 2817	0.0	0.0 - 0.0	0.0	0.0 - 0.0	35.4	12.2 - 96.9	90.9	30.2 - 221.1	0.0	0.0 - 0.0	937.4	354.7 - 2503	0.50
6030004	17467	PIGEON ROOST CI	60.7	484.3	167 - 988	0.0	0.0 - 0.0	9.0	2.7 - 18.4	9.0	2.6 - 22.5	22.5	7.4 - 65.7	55.0	15.5 - 170.0	388.9	137.6 - 846.	0.50
6030005	17468	CANEY CR	148.0	38.1	13 - 86	0.2	0.1 - 0.5	7.9	2.5 - 18.8	1.1	0.3 - 3.7	7.4	2.4 - 17.8	0.0	0.0 - 0.0	21.5	7.1 - 47.5	0.65
6030005	17469	YELLOW CR	222.2	38.3	11 - 70	1.6	0.5 - 3.8	10.5	3.1 - 21.5	1.6	0.4 - 4.2	12.0	3.4 - 32.0	0.0	0.0 - 0.0	12.6	3.8 - 25.8	0.68

8-digit HUC	ID	Name	Area	Catchment Yield		Point sources		Developed Land		Manure		Agricultural Land		Phosphate Mines		Soil parent rock		Frac
				kg/km^2	90% CI	kg/km^2	90% CI	kg/km^2	90% CI	kg/km^2	90% CI	kg/km^2	90% CI	kg/km^2	90% CI	kg/km^2	90% CI	
6030005	17470	DRY CR	47.0	11.8	4 - 31	0.0	0.0 - 0.0	2.5	0.9 - 6.0	0.6	0.2 - 1.8	2.2	0.7 - 5.9	0.0	0.0 - 0.0	6.5	2.5 - 16.1	0.51
6030005	17471	CANE CR	23.9	16.2	7 - 47	0.0	0.0 - 0.0	2.4	0.8 - 6.7	2.1	0.7 - 7.3	6.7	2.4 - 22.3	0.0	0.0 - 0.0	4.9	2.0 - 14.3	0.51
6030005	17472	MILL CR	26.9	32.0	12 - 66	0.0	0.0 - 0.0	4.6	1.5 - 9.3	5.0	1.8 - 13.2	18.7	6.6 - 44.9	0.0	0.0 - 0.0	3.7	1.3 - 8.6	0.55
6030005	17473	LITTLE BEAR CR	15.2	29.7	11 - 55	0.0	0.0 - 0.0	6.5	2.1 - 12.5	3.8	1.2 - 11.1	14.4	5.1 - 34.1	0.0	0.0 - 0.0	5.0	1.8 - 11.5	0.55
6030005	17474	SMITH CR	24.9	23.7	8 - 39	0.0	0.0 - 0.0	5.8	1.9 - 10.9	2.9	0.9 - 5.9	10.3	3.2 - 22.7	0.0	0.0 - 0.0	4.7	1.7 - 8.1	0.53
6030005	17475	LITTLE BEAR CR	20.7	24.2	7 - 61	0.2	0.0 - 0.4	6.5	1.8 - 16.4	3.0	0.8 - 7.9	10.6	3.5 - 30.3	0.0	0.0 - 0.0	4.0	1.1 - 12.1	0.53
6030005	17476	COOK CR	29.3	27.1	9 - 46	0.0	0.0 - 0.0	4.8	1.5 - 9.0	4.2	1.1 - 9.7	11.1	3.8 - 21.6	0.0	0.0 - 0.0	7.0	2.3 - 14.9	0.51
6030005	17477	LITTLE BEAR CR	48.7	56.9	22 - 91	0.0	0.0 - 0.0	10.1	3.5 - 17.4	12.8	4.0 - 33.4	25.1	9.1 - 51.4	0.0	0.0 - 0.0	9.0	3.5 - 16.5	0.51
6030005	17478	SPRING CR	25.5	87.9	25 - 183	0.0	0.0 - 0.0	10.5	3.3 - 23.7	12.5	3.7 - 32.7	45.9	12.1 - 119.9	0.0	0.0 - 0.0	18.9	5.8 - 46.0	0.55
6030005	17479	SPRING CR	107.0	54.4	16 - 109	0.0	0.0 - 0.0	6.0	2.0 - 15.0	15.1	3.7 - 36.9	23.9	7.0 - 66.2	0.0	0.0 - 0.0	9.5	3.3 - 19.6	0.52
6030005	17480	*A	26.5	60.4	22 - 151	0.0	0.0 - 0.0	3.7	1.2 - 9.3	9.1	3.0 - 29.0	33.2	10.7 - 86.6	0.0	0.0 - 0.0	14.4	5.3 - 34.4	0.52
6030005	17481	*B	99.4	109.9	34 - 277	0.0	0.0 - 0.0	13.6	3.6 - 36.9	16.6	4.3 - 46.5	60.6	22.1 - 171.3	0.0	0.0 - 0.0	19.1	6.3 - 48.2	0.55
6030005	17482	TENNESSEE R	27.0	159.4	51 - 409	0.0	0.0 - 0.0	111.6	34.0 - 275.7	0.6	0.2 - 2.3	2.8	0.8 - 7.4	0.0	0.0 - 0.0	44.3	14.2 - 121.9	0.57
6030005	17483	TOWN CR	11.5	82.7	22 - 197	0.0	0.0 - 0.0	8.4	2.3 - 19.8	16.8	4.5 - 40.4	49.4	13.0 - 121.6	0.0	0.0 - 0.0	8.1	2.2 - 20.6	0.51
6030005	17484	MUD CR	126.1	128.8	37 - 280	0.0	0.0 - 0.0	6.0	1.8 - 13.7	49.5	13.6 - 145.0	53.8	16.5 - 150.8	0.0	0.0 - 0.0	19.4	7.1 - 53.9	0.50
6030005	17485	TOWN CR	200.5	86.9	31 - 196	0.0	0.0 - 0.0	6.7	2.2 - 16.9	19.9	5.5 - 54.4	45.3	16.5 - 116.7	0.0	0.0 - 0.0	14.9	5.1 - 36.7	0.50
6030005	17486	*C	33.2	89.2	33 - 212	0.0	0.0 - 0.0	7.0	2.5 - 14.6	20.3	5.7 - 69.1	48.0	17.0 - 122.1	0.0	0.0 - 0.0	13.9	4.3 - 37.9	0.51
6030005	17487	*D	51.4	60.1	20 - 130	0.0	0.0 - 0.0	7.0	2.4 - 14.8	13.6	4.6 - 41.9	32.9	9.5 - 89.3	0.0	0.0 - 0.0	6.7	2.5 - 16.4	0.51
6030005	17488	BIG NANCE CR	21.8	25.6	9 - 58	0.0	0.0 - 0.0	5.2	1.5 - 11.0	4.6	1.3 - 11.4	9.4	2.9 - 25.3	0.0	0.0 - 0.0	6.5	2.0 - 14.8	0.51
6030005	17489	BORDEN CR	66.7	103.2	32 - 235	0.0	0.0 - 0.0	8.0	2.9 - 16.5	22.9	6.6 - 64.1	55.3	16.7 - 141.5	0.0	0.0 - 0.0	17.0	5.9 - 46.3	0.50
6030005	17490	*E	31.1	126.9	42 - 243	0.0	0.0 - 0.0	8.7	2.7 - 19.7	29.0	9.5 - 74.9	68.3	22.8 - 207.2	0.0	0.0 - 0.0	21.0	6.1 - 46.9	0.47
6030005	17491	BORDEN CR	93.6	133.1	49 - 259	14.6	5.4 - 29.3	12.9	4.0 - 27.3	25.3	7.3 - 72.1	60.8	20.0 - 140.8	0.0	0.0 - 0.0	19.4	7.0 - 45.1	0.47
6030005	17492	BIG NANCE CR	105.7	65.9	21 - 120	0.1	0.0 - 0.3	9.1	2.6 - 18.7	14.0	3.8 - 33.7	32.9	9.6 - 81.1	0.0	0.0 - 0.0	9.7	2.9 - 21.3	0.50
6030005	17493	MILL CR	41.4	177.5	55 - 383	0.0	0.0 - 0.0	11.7	3.5 - 22.3	15.2	3.9 - 35.5	78.9	25.1 - 177.2	0.0	0.0 - 0.0	71.6	21.0 - 193.3	0.53
6030005	17494	BLUEWATER CR	28.0	143.4	44 - 336	0.0	0.0 - 0.0	9.6	3.1 - 24.4	12.0	3.7 - 35.8	62.3	18.2 - 165.6	0.0	0.0 - 0.0	59.5	19.2 - 160.9	0.53
6030005	17495	BLUEWATER CR	229.0	121.9	39 - 220	0.0	0.0 - 0.0	8.5	2.9 - 14.9	13.5	4.7 - 34.7	47.8	13.9 - 100.3	0.0	0.0 - 0.0	52.1	19.9 - 116.4	0.52
6030005	17496	RICHARDSON CR	28.1	142.7	44 - 315	0.0	0.0 - 0.0	13.4	4.4 - 26.7	11.4	3.3 - 31.9	59.1	17.9 - 150.1	0.0	0.0 - 0.0	58.8	18.1 - 146.9	0.52
6030005	17497	SHOAL CR	63.7	110.4	34 - 202	0.0	0.0 - 0.0	9.0	2.7 - 17.8	9.8	2.0 - 23.6	34.4	10.0 - 83.6	0.0	0.0 - 0.0	57.2	17.9 - 115.3	0.53
6030005	17498	SHOAL CR	35.6	75.9	25 - 151	0.0	0.0 - 0.0	6.2	2.0 - 12.5	6.1	1.7 - 17.4	18.3	5.8 - 44.4	0.0	0.0 - 0.0	45.2	15.6 - 101.7	0.53
6030005	17499	SHOAL CR	54.0	85.8	28 - 189	0.0	0.0 - 0.0	9.6	2.9 - 23.9	8.6	2.4 - 25.0	26.2	9.0 - 83.3	0.0	0.0 - 0.0	41.3	12.8 - 88.5	0.52
6030005	17500	SHOAL CR	114.6	69.4	23 - 166	2.5	0.8 - 6.3	7.2	2.3 - 16.6	5.9	1.7 - 16.4	17.6	5.1 - 46.0	0.0	0.0 - 0.0	36.3	11.2 - 104.7	0.51
6030005	17502	SHOAL CR	75.8	160.4	50 - 270	0.0	0.0 - 0.0	34.2	10.7 - 69.4	18.7	5.3 - 46.2	56.5	15.8 - 132.4	0.0	0.0 - 0.0	51.1	17.8 - 117.4	0.47
6030005	17503	CROWSON CR	64.5	164.0	53 - 321	0.0	0.0 - 0.0	17.4	6.0 - 40.0	23.0	7.3 - 59.9	70.0	22.5 - 163.8	0.0	0.0 - 0.0	53.6	17.5 - 122.1	0.47
6030005	17504	POND CR	52.2	151.0	58 - 332	0.0	0.0 - 0.0	15.2	6.0 - 32.6	21.8	7.4 - 55.5	65.9	22.1 - 184.0	0.0	0.0 - 0.0	48.1	17.8 - 116.4	0.47
6030005	17505	KNOB CR	143.0	64.6	24 - 115	0.0	0.0 - 0.0	5.6	2.0 - 10.0	6.9	2.2 - 15.0	20.7	5.8 - 49.7	0.0	0.0 - 0.0	31.4	11.8 - 62.8	0.51
6030005	17506	CHISOLM CR	9.7	79.5	24 - 173	0.0	0.0 - 0.0	4.6	1.3 - 10.7	7.6	2.2 - 20.8	22.7	7.4 - 53.5	0.0	0.0 - 0.0	44.5	14.1 - 106.3	0.52
6030005	17507	CHISOLM CR	113.4	66.7	25 - 119	0.0	0.0 - 0.0	6.0	2.1 - 10.7	5.8	1.9 - 14.0	18.1	6.4 - 45.2	0.0	0.0 - 0.0	36.8	14.1 - 87.2	0.51
6030005	17508	FACTORY CR	56.1	66.7	21 - 134	0.0	0.0 - 0.0	4.1	1.3 - 9.3	3.9	1.2 - 10.8	12.5	4.1 - 30.6	0.0	0.0 - 0.0	46.3	14.5 - 101.7	0.51
6030005	17509	FACTORY CR	82.3	61.6	20 - 129	0.0	0.0 - 0.0	5.5	1.9 - 11.7	3.1	0.8 - 7.8	9.2	3.1 - 24.4	0.0	0.0 - 0.0	43.9	14.4 - 101.8	0.47

8-digit HUC	ID	Name	Area	Catchment Yield		Point sources		Developed Land		Manure		Agricultural Land		Phosphate Mines		Soil parent rock		Frac
				kg/km^2	90% CI	kg/km^2	90% CI	kg/km^2	90% CI	kg/km^2	90% CI	kg/km^2	90% CI	kg/km^2	90% CI	kg/km^2	90% CI	
6030005	17510	SHAWNETTE CR	48.7	72.9	23 - 134	0.0	0.0 - 0.0	7.8	2.5 - 15.1	5.2	1.3 - 10.1	18.7	5.7 - 42.1	0.0	0.0 - 0.0	41.2	13.1 - 88.5	0.47
6030005	17511	HOLLY CR	50.2	67.8	23 - 133	0.0	0.0 - 0.0	5.2	1.8 - 10.2	3.6	1.3 - 8.8	11.3	3.9 - 26.6	0.0	0.0 - 0.0	47.7	15.7 - 103.1	0.53
6030005	17512	BUTLER CR	157.2	84.5	26 - 252	0.0	0.0 - 0.0	6.5	2.2 - 17.8	7.1	1.7 - 22.3	26.1	7.4 - 80.5	0.0	0.0 - 0.0	44.6	12.9 - 132.0	0.53
6030005	17513	CYPRESS CR	63.3	454.8	138 - 860	299.0	93.8 - 547.1	62.8	19.3 - 129.9	6.1	1.7 - 16.6	30.3	9.2 - 68.7	0.0	0.0 - 0.0	56.6	19.1 - 120.3	0.57
6030005	17514	LITTLE CYPRESS C	134.2	119.5	39 - 292	0.6	0.2 - 1.5	14.0	4.8 - 33.6	10.3	3.0 - 30.7	46.5	17.0 - 141.9	0.0	0.0 - 0.0	48.1	14.0 - 145.4	0.56
6030005	17515	CYPRESS CR	26.0	146.6	48 - 352	0.0	0.0 - 0.0	12.2	3.2 - 28.4	10.9	3.5 - 37.0	56.0	16.4 - 160.8	0.0	0.0 - 0.0	67.5	24.4 - 182.6	0.56
6030005	17516	CYPRESS CR	40.7	174.9	61 - 384	0.0	0.0 - 0.0	10.3	3.5 - 24.9	15.9	4.9 - 44.1	83.0	28.3 - 207.0	0.0	0.0 - 0.0	65.7	21.4 - 161.2	0.55
6030005	17517	CYPRESS CR, GRE	114.8	102.3	37 - 282	0.0	0.0 - 0.0	5.9	2.1 - 14.5	10.8	3.3 - 39.5	51.6	19.8 - 170.3	0.0	0.0 - 0.0	34.0	10.9 - 94.6	0.53
6030005	17518	CYPRESS CR	128.4	97.1	30 - 270	0.0	0.0 - 0.0	8.7	2.7 - 23.8	9.5	2.6 - 32.5	42.4	13.4 - 131.4	0.0	0.0 - 0.0	36.6	10.4 - 96.5	0.53
6030005	17519	BURCHAM CR	46.1	170.3	53 - 341	0.0	0.0 - 0.0	11.4	3.7 - 23.9	15.8	4.4 - 39.5	81.1	23.5 - 211.2	0.0	0.0 - 0.0	62.0	20.1 - 159.1	0.55
6030005	17520	SECOND CR	64.7	45.6	16 - 90	0.0	0.0 - 0.0	3.0	1.1 - 6.1	3.8	1.2 - 9.6	15.2	4.7 - 39.6	0.0	0.0 - 0.0	23.6	8.3 - 56.4	0.52
6030005	17521	GRASSY CR	31.8	30.3	10 - 78	0.0	0.0 - 0.0	2.1	0.7 - 5.3	3.0	0.9 - 8.5	11.9	3.9 - 31.6	0.0	0.0 - 0.0	13.3	4.4 - 35.2	0.52
6030006	17522	CRIPPLE DEER CR	21.9	30.9	11 - 77	0.0	0.0 - 0.0	5.8	2.0 - 12.6	2.6	0.8 - 6.8	11.2	3.9 - 32.9	0.0	0.0 - 0.0	11.3	3.8 - 28.6	0.57
6030006	17523	LITTLE CRIPPLE D	49.6	45.6	18 - 102	0.2	0.1 - 0.4	8.8	3.2 - 19.0	2.8	0.9 - 7.6	20.7	8.3 - 59.7	0.0	0.0 - 0.0	13.1	5.0 - 32.1	0.54
6030006	17524	CRIPPLE DEER CR	74.7	34.7	13 - 72	0.0	0.0 - 0.0	7.7	2.6 - 17.3	2.0	0.6 - 5.2	14.4	4.6 - 33.6	0.0	0.0 - 0.0	10.7	3.8 - 22.9	0.54
6030006	17525	BEAR CR	56.1	32.9	11 - 71	0.0	0.0 - 0.0	6.5	2.0 - 13.7	3.5	1.1 - 11.4	13.8	5.4 - 34.3	0.0	0.0 - 0.0	9.1	3.1 - 24.8	0.57
6030006	17526	BEAR CR	12.3	46.9	16 - 107	0.0	0.0 - 0.0	12.2	4.1 - 30.2	4.5	1.5 - 15.8	17.5	6.0 - 44.6	0.0	0.0 - 0.0	12.6	4.6 - 32.7	0.57
6030006	17527	BEAR CR	452.0	47.7	16 - 84	1.1	0.4 - 2.2	8.0	2.6 - 15.1	10.7	3.2 - 29.4	17.7	5.7 - 36.2	0.0	0.0 - 0.0	10.2	3.4 - 22.9	0.56
6030006	17529	BEAR CR	99.5	27.0	9 - 57	0.0	0.0 - 0.0	8.4	2.5 - 16.3	6.7	1.9 - 18.0	8.6	2.8 - 24.2	0.0	0.0 - 0.0	3.3	1.0 - 7.5	0.49
6030006	17530	BEAR CR	173.4	40.7	12 - 83	0.0	0.0 - 0.0	9.9	2.9 - 22.4	12.2	3.4 - 32.4	13.5	4.0 - 35.5	0.0	0.0 - 0.0	5.0	1.5 - 12.4	0.25
6030006	17531	LITTLE BEAR CR	25.8	56.2	20 - 150	0.0	0.0 - 0.0	9.8	3.5 - 23.5	18.9	5.6 - 67.8	21.4	7.6 - 69.4	0.0	0.0 - 0.0	6.1	2.2 - 16.5	0.25
6030006	17532	TURKEY CR	22.4	51.1	19 - 120	0.0	0.0 - 0.0	4.6	1.8 - 11.3	19.9	7.0 - 62.8	21.2	7.6 - 61.7	0.0	0.0 - 0.0	5.4	2.0 - 13.0	0.24
6030006	17533	LITTLE BEAR CR	59.4	49.8	16 - 116	0.0	0.0 - 0.0	5.7	1.8 - 13.4	18.9	5.1 - 50.6	19.7	6.5 - 55.5	0.0	0.0 - 0.0	5.4	1.9 - 14.5	0.24
6030006	17534	BLUFF CR	29.6	32.5	10 - 64	0.0	0.0 - 0.0	10.6	3.3 - 22.9	7.7	2.3 - 20.7	8.5	2.8 - 20.4	0.0	0.0 - 0.0	5.8	2.0 - 14.0	0.49
6030006	17535	LITTLE BEAR CR	96.6	36.8	12 - 84	0.0	0.0 - 0.0	5.2	1.5 - 11.4	6.9	2.1 - 18.7	11.6	3.2 - 31.6	0.0	0.0 - 0.0	13.1	4.2 - 33.8	0.56
6030006	17536	LITTLE BEAR CR	232.1	15.6	5 - 31	0.0	0.0 - 0.0	2.8	1.0 - 6.0	4.2	1.1 - 12.0	4.5	1.4 - 11.2	0.0	0.0 - 0.0	4.0	1.2 - 10.8	0.55
6030006	17537	CEDAR CR	142.1	11.1	3 - 23	0.0	0.0 - 0.0	2.1	0.6 - 4.9	2.3	0.7 - 6.0	2.7	0.8 - 7.4	0.0	0.0 - 0.0	4.0	1.2 - 9.7	0.55
6030006	17538	LICK CR	25.4	30.5	11 - 56	0.0	0.0 - 0.0	6.0	2.0 - 12.3	7.2	2.5 - 16.5	8.1	2.7 - 18.6	0.0	0.0 - 0.0	9.1	3.4 - 20.1	0.21
6030006	17539	CEDAR CR	100.9	27.2	8 - 65	0.0	0.0 - 0.0	5.9	1.7 - 13.4	5.3	1.4 - 14.9	6.1	1.9 - 14.8	0.0	0.0 - 0.0	10.0	3.1 - 25.4	0.21
6030006	17540	CEDAR CR	77.0	49.8	15 - 99	0.0	0.0 - 0.0	7.2	2.4 - 14.2	15.1	3.9 - 40.2	16.0	4.3 - 39.0	0.0	0.0 - 0.0	11.6	3.4 - 26.6	0.20
6030006	17541	CEDAR CR	79.1	37.4	15 - 71	0.0	0.0 - 0.0	4.1	1.7 - 8.6	12.3	4.6 - 30.5	12.6	4.7 - 32.9	0.0	0.0 - 0.0	8.4	3.7 - 17.5	0.19
6030006	17542	HARRIS CR	67.1	183.2	59 - 403	56.9	18.1 - 128.1	31.1	9.7 - 71.6	37.1	12.0 - 104.8	39.3	12.7 - 90.0	0.0	0.0 - 0.0	18.9	6.6 - 45.2	0.19
6030006	17543	DUNKIN CR	33.1	23.4	10 - 58	0.0	0.0 - 0.0	5.1	1.9 - 11.7	3.7	1.3 - 9.9	4.0	1.5 - 10.5	0.0	0.0 - 0.0	10.7	4.2 - 27.2	0.20
6030006	17544	ROCK CR	138.1	17.7	6 - 37	0.0	0.0 - 0.0	3.8	1.1 - 8.1	1.0	0.3 - 2.7	3.5	1.2 - 9.2	0.0	0.0 - 0.0	9.4	3.4 - 24.4	0.57
6030006	17545	BUZZARD ROOST	102.2	18.3	6 - 32	0.0	0.0 - 0.0	3.2	1.1 - 6.0	1.2	0.4 - 2.8	4.6	1.4 - 10.9	0.0	0.0 - 0.0	9.3	2.9 - 18.2	0.55
6030006	17546	BROWN'S CR	31.7	45.7	18 - 82	0.0	0.0 - 0.0	8.2	3.2 - 16.8	4.9	1.7 - 12.9	18.2	6.2 - 44.0	0.0	0.0 - 0.0	14.4	5.7 - 30.1	0.55
6040001	17547	CUB CR	90.4	52.7	21 - 151	0.0	0.0 - 0.0	3.8	1.6 - 8.9	4.4	1.5 - 12.5	16.7	6.7 - 47.4	0.0	0.0 - 0.0	27.8	10.2 - 81.5	0.60
6040001	17548	CUB CR, SOLPHER	66.7	63.0	25 - 179	0.0	0.0 - 0.0	4.0	1.5 - 10.9	6.1	2.1 - 18.7	24.1	8.9 - 80.4	0.0	0.0 - 0.0	28.8	10.3 - 88.7	0.60
6040001	17549	BEECH R	137.2	94.3	29 - 203	6.6	2.0 - 16.2	9.6	2.7 - 21.4	7.3	1.9 - 23.6	31.8	10.3 - 87.1	0.0	0.0 - 0.0	39.1	11.4 - 98.6	0.64

8-digit HUC	ID	Name	Area	Catchment Yield		Point sources		Developed Land		Manure		Agricultural Land		Phosphate Mines		Soil parent rock		Frac
				kg/km^2	90% CI	kg/km^2	90% CI	kg/km^2	90% CI	kg/km^2	90% CI	kg/km^2	90% CI	kg/km^2	90% CI	kg/km^2	90% CI	
6040001	17551	BIG CR	46.5	80.4	25 - 197	0.0	0.0 - 0.0	5.1	1.6 - 11.9	9.4	2.7 - 33.9	34.5	10.1 - 100.2	0.0	0.0 - 0.0	31.4	9.5 - 84.2	0.62
6040001	17552	BEECH R	20.6	155.7	62 - 295	0.0	0.0 - 0.0	6.0	2.3 - 12.9	20.7	7.0 - 59.7	79.5	30.0 - 176.1	0.0	0.0 - 0.0	49.5	20.3 - 121.0	0.62
6040001	17553	BROWNS CR	63.8	61.4	21 - 158	0.0	0.0 - 0.0	4.2	1.3 - 10.6	5.9	1.9 - 15.7	21.9	6.9 - 59.3	0.0	0.0 - 0.0	29.4	9.6 - 92.9	0.61
6040001	17554	BEECH R	7.8	229.5	82 - 491	0.0	0.0 - 0.0	10.1	3.7 - 20.2	32.7	9.8 - 112.4	127.4	44.2 - 308.5	0.0	0.0 - 0.0	59.2	21.1 - 159.1	0.61
6040001	17555	BEECH R	76.5	79.5	25 - 192	0.0	0.0 - 0.0	5.7	1.7 - 14.9	9.2	2.5 - 30.5	30.8	8.3 - 87.1	0.0	0.0 - 0.0	33.8	10.4 - 89.3	0.60
6040001	17556	BEECH R	168.2	102.2	40 - 220	14.8	6.0 - 30.5	14.1	5.1 - 30.4	9.4	3.2 - 32.2	32.8	11.4 - 84.7	0.0	0.0 - 0.0	31.2	11.2 - 86.5	0.58
6040001	17557	PINEY CR	54.2	50.3	18 - 107	0.0	0.0 - 0.0	6.1	2.2 - 12.3	4.3	1.2 - 12.3	14.4	4.5 - 36.1	0.0	0.0 - 0.0	25.5	8.7 - 61.7	0.58
6040001	17558	CANE CR	57.7	122.9	38 - 277	0.0	0.0 - 0.0	3.0	0.8 - 7.3	18.0	4.8 - 60.2	66.7	20.0 - 207.3	0.0	0.0 - 0.0	35.1	11.0 - 77.7	0.60
6040001	17559	FLAT CR	44.3	102.0	38 - 176	0.0	0.0 - 0.0	3.6	1.3 - 7.2	13.0	4.0 - 35.6	49.5	17.6 - 97.7	0.0	0.0 - 0.0	36.0	11.6 - 79.7	0.62
6040001	17560	RUSHING CR	67.3	90.0	31 - 196	3.6	1.3 - 7.2	4.0	1.4 - 8.0	8.0	2.1 - 22.6	35.9	14.1 - 82.9	0.0	0.0 - 0.0	38.5	13.4 - 86.8	0.64
6040001	17561	WHITES CR	64.4	45.5	15 - 93	0.0	0.0 - 0.0	1.9	0.6 - 3.9	2.9	0.9 - 6.6	11.6	3.8 - 27.8	0.0	0.0 - 0.0	29.1	10.1 - 60.0	0.65
6040001	17562	TENNESSEE R	92.5	125.8	44 - 249	0.0	0.0 - 0.0	5.1	1.9 - 9.4	4.6	1.3 - 11.4	20.1	6.2 - 43.9	0.0	0.0 - 0.0	96.0	34.6 - 194.9	0.65
6040001	17563	TENNESSEE R	81.5	89.9	26 - 196	0.0	0.0 - 0.0	14.1	3.8 - 32.2	4.7	1.2 - 13.8	23.8	6.7 - 65.9	0.0	0.0 - 0.0	47.3	13.5 - 116.9	0.64
6040001	17564	TURNBO CR	53.5	53.2	18 - 109	0.0	0.0 - 0.0	5.1	1.5 - 11.7	5.0	1.6 - 12.2	23.3	7.3 - 51.4	0.0	0.0 - 0.0	19.8	7.1 - 49.2	0.64
6040001	17565	TENNESSEE R	3.7	63.3	23 - 158	0.0	0.0 - 0.0	5.3	1.8 - 12.7	7.3	2.2 - 20.9	37.0	13.0 - 122.7	0.0	0.0 - 0.0	13.7	5.1 - 38.3	0.64
6040001	17566	TENNESSEE R	12.5	57.9	21 - 131	0.0	0.0 - 0.0	3.7	0.9 - 9.2	7.3	2.1 - 22.1	36.7	12.4 - 97.5	0.0	0.0 - 0.0	10.3	3.6 - 26.8	0.64
6040001	17567	STEWMAN CR	74.4	65.9	22 - 161	0.0	0.0 - 0.0	4.3	1.5 - 8.4	6.0	1.9 - 17.2	29.6	8.7 - 83.9	0.0	0.0 - 0.0	26.0	9.5 - 66.6	0.64
6040001	17568	TENNESSEE R	1.4	41.0	14 - 100	0.0	0.0 - 0.0	1.0	0.3 - 2.5	3.8	1.2 - 11.1	26.3	8.9 - 71.6	0.0	0.0 - 0.0	10.0	3.3 - 24.7	0.64
6040001	17569	TENNESSEE R	11.9	54.4	15 - 100	0.0	0.0 - 0.0	3.5	1.0 - 7.4	4.9	1.3 - 12.9	31.0	8.4 - 71.4	0.0	0.0 - 0.0	15.0	4.3 - 35.6	0.64
6040001	17570	DOE CR	83.8	85.7	27 - 192	0.0	0.0 - 0.0	4.1	1.3 - 10.9	10.1	2.9 - 30.4	43.1	12.5 - 117.2	0.0	0.0 - 0.0	28.4	8.8 - 72.4	0.64
6040001	17571	TENNESSEE R	18.5	126.9	43 - 266	0.0	0.0 - 0.0	7.2	2.3 - 18.0	8.8	2.5 - 27.2	74.5	25.9 - 189.6	0.0	0.0 - 0.0	36.4	12.0 - 89.6	0.64
6040001	17572	WHITE OAK CR	15.3	84.7	26 - 177	0.0	0.0 - 0.0	4.0	1.2 - 8.2	5.6	1.7 - 15.9	51.0	16.5 - 123.0	0.0	0.0 - 0.0	24.1	7.3 - 60.7	0.64
6040001	17573	FLATS CR	34.4	96.6	36 - 226	0.0	0.0 - 0.0	5.4	2.0 - 10.8	5.9	1.9 - 18.1	41.5	12.9 - 119.0	0.0	0.0 - 0.0	43.8	15.5 - 109.3	0.63
6040001	17574	WHITE OAK CR	19.3	77.1	19 - 201	0.0	0.0 - 0.0	3.7	0.9 - 8.9	3.7	1.0 - 11.9	31.2	6.3 - 81.0	0.0	0.0 - 0.0	38.6	10.3 - 109.4	0.63
6040001	17575	WHITE OAK CR	3.2	132.4	43 - 315	0.0	0.0 - 0.0	0.0	0.0 - 0.0	2.6	0.7 - 6.6	20.0	6.4 - 58.1	0.0	0.0 - 0.0	109.8	35.5 - 273.3	0.63
6040001	17576	HURRICANE CR	37.2	117.1	37 - 244	0.0	0.0 - 0.0	4.9	1.7 - 12.8	11.2	3.8 - 27.5	59.1	19.8 - 150.1	0.0	0.0 - 0.0	42.0	13.7 - 98.1	0.62
6040001	17577	WHITE OAK CR	3.2	155.7	47 - 422	0.0	0.0 - 0.0	0.0	0.0 - 0.0	2.4	0.7 - 8.7	18.3	5.8 - 52.4	0.0	0.0 - 0.0	135.1	40.7 - 389.3	0.62
6040001	17578	MIDDLETON CR	149.8	80.5	27 - 212	0.0	0.0 - 0.0	6.1	2.1 - 14.5	7.1	2.2 - 19.1	36.4	10.5 - 103.9	0.0	0.0 - 0.0	30.8	10.5 - 84.7	0.61
6040001	17579	WHITE OAK CR	144.8	69.3	22 - 175	0.0	0.0 - 0.0	5.3	1.7 - 13.2	3.8	1.1 - 9.4	29.5	10.2 - 76.9	0.0	0.0 - 0.0	30.7	9.2 - 84.1	0.61
6040001	17580	MUD CR	76.2	109.2	31 - 244	0.0	0.0 - 0.0	5.3	1.5 - 10.7	6.0	1.5 - 16.9	53.2	14.3 - 151.0	0.0	0.0 - 0.0	44.7	12.5 - 112.1	0.63
6040001	17581	TENNESSEE R	32.1	77.2	24 - 172	0.0	0.0 - 0.0	3.2	1.0 - 7.2	5.8	1.7 - 18.8	51.0	15.6 - 132.5	0.0	0.0 - 0.0	17.3	5.9 - 45.3	0.64
6040001	17582	TENNESSEE R	201.2	152.5	53 - 410	0.0	0.0 - 0.0	11.6	3.6 - 31.8	10.5	3.5 - 33.0	93.9	29.0 - 291.7	0.0	0.0 - 0.0	36.5	12.1 - 107.6	0.64
6040001	17583	SNAKE CR	3.7	100.9	37 - 212	0.0	0.0 - 0.0	11.4	4.1 - 23.8	5.8	1.8 - 14.6	45.5	16.5 - 104.5	0.0	0.0 - 0.0	38.3	14.1 - 86.1	0.64
6040001	17584	SNAKE CR	226.8	91.4	38 - 175	2.3	0.9 - 4.7	6.7	2.2 - 12.8	5.0	1.7 - 11.5	44.7	18.0 - 106.4	0.0	0.0 - 0.0	32.8	13.2 - 80.9	0.63
6040001	17585	OWL CR	101.0	104.3	36 - 200	0.0	0.0 - 0.0	5.7	1.9 - 10.8	5.8	1.5 - 18.2	50.6	16.1 - 113.3	0.0	0.0 - 0.0	42.1	13.7 - 101.5	0.63
6040001	17586	TENNESSEE R	13.8	121.4	41 - 291	0.0	0.0 - 0.0	6.3	2.2 - 14.6	7.8	2.1 - 26.2	68.2	20.7 - 184.8	0.0	0.0 - 0.0	39.1	13.0 - 97.3	0.64
6040001	17587	LICK CR	101.0	118.9	42 - 228	0.0	0.0 - 0.0	6.5	2.4 - 12.0	5.4	1.4 - 14.2	48.0	13.9 - 116.4	0.0	0.0 - 0.0	59.0	21.4 - 125.3	0.64
6040001	17588	TENNESSEE R	38.9	94.8	28 - 216	0.0	0.0 - 0.0	8.1	2.3 - 17.7	5.7	1.4 - 16.5	49.9	15.9 - 128.1	0.0	0.0 - 0.0	31.1	8.6 - 84.8	0.64
6040001	17589	CHAMBERS CR	104.0	64.2	21 - 140	0.0	0.0 - 0.0	5.2	1.6 - 11.3	2.8	0.9 - 7.7	23.6	8.2 - 62.0	0.0	0.0 - 0.0	32.6	10.6 - 77.2	0.64

8-digit HUC	ID	Name	Area	Catchment Yield		Point sources		Developed Land		Manure		Agricultural Land		Phosphate Mines		Soil parent rock		Frac
				kg/km^2	90% CI	kg/km^2	90% CI	kg/km^2	90% CI	kg/km^2	90% CI	kg/km^2	90% CI	kg/km^2	90% CI	kg/km^2	90% CI	
6040001	17590	WALDROP CR	59.7	84.1	31 - 225	0.0	0.0 - 0.0	7.1	3.0 - 16.5	4.1	1.4 - 11.5	34.7	11.7 - 106.9	0.0	0.0 - 0.0	38.2	14.4 - 90.2	0.59
6040001	17591	CHAMBERS CR	59.1	93.2	33 - 175	0.0	0.0 - 0.0	6.7	2.3 - 13.0	4.7	1.3 - 12.4	33.3	12.9 - 82.6	0.0	0.0 - 0.0	48.5	15.5 - 96.7	0.59
6040001	17592	TENNESSEE R	19.1	1249.0	423 - 2788	1181.7	401.9 - 2669.4	12.0	3.4 - 22.8	2.3	0.7 - 6.2	19.8	6.1 - 44.9	0.0	0.0 - 0.0	33.2	11.8 - 76.3	0.64
6040001	17593	HORSE CR	21.3	98.1	34 - 217	0.0	0.0 - 0.0	6.1	2.2 - 13.0	7.9	2.2 - 25.8	70.5	25.1 - 186.0	0.0	0.0 - 0.0	13.6	4.3 - 32.0	0.64
6040001	17594	HORSE CR	95.6	63.5	19 - 118	0.0	0.0 - 0.0	14.3	4.0 - 29.8	3.5	0.9 - 10.0	31.4	9.6 - 75.2	0.0	0.0 - 0.0	14.2	4.3 - 31.9	0.63
6040001	17595	HOLLAND CR	40.7	32.4	10 - 59	0.0	0.0 - 0.0	5.0	1.5 - 10.4	1.9	0.5 - 4.8	16.1	4.8 - 36.1	0.0	0.0 - 0.0	9.3	2.6 - 19.0	0.56
6040001	17596	HORSE CR	173.7	33.2	11 - 66	0.0	0.0 - 0.0	3.5	1.2 - 7.1	2.1	0.6 - 5.1	14.9	4.9 - 37.3	0.0	0.0 - 0.0	12.6	4.1 - 27.4	0.56
6040001	17597	TURKEY CR	161.4	42.9	14 - 107	9.6	3.2 - 25.8	4.6	1.5 - 10.4	1.6	0.4 - 4.8	14.5	4.8 - 43.7	0.0	0.0 - 0.0	12.6	4.1 - 32.9	0.63
6040001	17598	INDIAN CR	69.4	44.8	14 - 106	0.0	0.0 - 0.0	4.1	1.2 - 8.8	3.1	0.9 - 9.0	27.3	8.4 - 69.4	0.0	0.0 - 0.0	10.3	3.3 - 24.4	0.64
6040001	17599	INDIAN CR, SMITH	47.2	44.2	17 - 103	0.0	0.0 - 0.0	3.2	1.1 - 9.3	1.6	0.5 - 6.2	8.2	3.0 - 21.1	0.0	0.0 - 0.0	31.1	11.3 - 79.1	0.62
6040001	17600	INDIAN CR	124.5	53.3	19 - 119	0.0	0.0 - 0.0	3.4	1.2 - 7.4	2.6	0.7 - 6.8	14.0	4.8 - 35.5	0.0	0.0 - 0.0	33.3	12.2 - 78.2	0.62
6040001	17601	HARDIN CR	54.3	41.5	14 - 78	0.0	0.0 - 0.0	2.8	0.9 - 7.1	1.9	0.6 - 4.2	14.8	4.8 - 34.2	0.0	0.0 - 0.0	21.9	7.8 - 50.6	0.64
6040001	17602	HARDIN CR	145.5	58.4	21 - 129	0.0	0.0 - 0.0	5.6	1.9 - 12.1	2.6	0.9 - 7.3	9.2	3.1 - 23.0	0.0	0.0 - 0.0	41.0	13.3 - 103.3	0.59
6040001	17603	EAGLE CR	55.1	53.8	22 - 132	0.0	0.0 - 0.0	3.8	1.5 - 8.9	2.9	1.1 - 6.6	10.9	4.2 - 25.7	0.0	0.0 - 0.0	36.2	13.7 - 98.1	0.59
6040001	17604	BEECH CR	152.2	92.3	39 - 179	0.0	0.0 - 0.0	4.1	1.5 - 8.1	2.2	0.8 - 6.0	7.5	2.7 - 19.7	0.0	0.0 - 0.0	78.5	31.1 - 165.2	0.64
6040001	17605	WEATHERFORD C	130.8	58.2	18 - 116	0.0	0.0 - 0.0	5.1	1.6 - 10.4	4.6	1.6 - 13.7	16.0	4.7 - 37.2	0.0	0.0 - 0.0	32.5	9.9 - 71.5	0.57
6040001	17606	INDIAN CR	222.5	60.5	23 - 124	0.0	0.0 - 0.0	4.7	1.6 - 9.8	3.6	1.3 - 10.6	10.8	3.8 - 29.2	0.0	0.0 - 0.0	41.4	16.0 - 94.2	0.57
6040002	17607	DUCK R	34.1	974.2	345 - 2140	0.0	0.0 - 0.0	5.3	1.8 - 9.4	13.3	3.9 - 32.4	37.7	13.0 - 83.6	0.0	0.0 - 0.0	917.9	326.0 - 2069	0.59
6040002	17609	FOUNTAIN CR	62.6	713.3	233 - 1424	0.0	0.0 - 0.0	4.2	1.3 - 8.1	13.8	4.1 - 39.6	41.6	13.2 - 89.4	19.0	5.4 - 56.4	634.7	215.0 - 1377	0.58
6040002	17610	FOUNTAIN CR	72.2	618.0	239 - 1925	0.0	0.0 - 0.0	4.7	1.7 - 11.7	10.3	3.3 - 33.8	30.9	10.9 - 95.0	43.4	14.7 - 168.9	528.7	204.4 - 1633	0.53
6040002	17611	GLOBE CR	91.6	662.9	213 - 1315	0.0	0.0 - 0.0	6.3	1.9 - 12.2	11.9	3.4 - 36.5	34.7	11.2 - 82.6	5.6	1.7 - 16.8	604.5	187.0 - 1327	0.53
6040002	17612	SILVER CR	40.2	356.3	101 - 781	0.0	0.0 - 0.0	6.8	1.9 - 11.5	11.5	3.1 - 32.0	32.9	8.3 - 79.1	0.0	0.0 - 0.0	305.0	87.0 - 721.5	0.58
6040002	17613	DUCK R	45.6	402.0	135 - 945	0.0	0.0 - 0.0	4.3	1.5 - 10.0	10.0	3.0 - 30.7	28.4	9.3 - 70.4	0.0	0.0 - 0.0	359.4	116.5 - 897.4	0.59
6040002	17614	CEDAR CR	50.7	172.9	62 - 350	0.0	0.0 - 0.0	5.0	1.9 - 9.3	13.0	3.8 - 34.6	36.5	12.6 - 87.6	0.0	0.0 - 0.0	118.4	42.7 - 250.7	0.58
6040002	17615	DUCK R	40.6	227.5	82 - 507	0.0	0.0 - 0.0	4.1	1.3 - 9.8	6.9	2.1 - 21.4	18.8	5.4 - 54.7	0.0	0.0 - 0.0	197.7	72.1 - 444.2	0.58
6040002	17616	DUCK R	44.3	193.4	62 - 485	0.0	0.0 - 0.0	5.4	2.0 - 12.6	12.3	3.3 - 35.3	34.3	12.1 - 94.2	0.0	0.0 - 0.0	141.5	45.2 - 373.5	0.57
6040002	17617	DUCK R	15.3	181.7	58 - 361	0.0	0.0 - 0.0	4.0	1.4 - 9.1	11.3	3.0 - 34.7	32.4	11.3 - 70.3	0.0	0.0 - 0.0	134.0	44.2 - 282.1	0.57
6040002	17618	BIG ROCK CR	1.4	188.6	56 - 474	0.0	0.0 - 0.0	9.2	2.8 - 22.2	8.6	2.6 - 29.4	25.2	8.0 - 79.1	0.0	0.0 - 0.0	145.7	47.3 - 370.9	0.57
6040002	17619	BIG ROCK CR	164.2	410.4	153 - 888	22.0	8.9 - 51.1	17.5	7.2 - 37.3	12.1	3.7 - 39.5	34.8	12.8 - 80.6	0.0	0.0 - 0.0	324.0	119.3 - 835.[illegible]	0.56
6040002	17620	E ROCK CR	28.1	186.7	63 - 389	0.0	0.0 - 0.0	5.8	2.1 - 11.8	14.8	4.3 - 37.3	43.2	15.4 - 101.2	0.0	0.0 - 0.0	122.9	42.4 - 271.9	0.56
6040002	17621	BELFAST CR	32.8	300.9	100 - 603	0.0	0.0 - 0.0	7.9	2.8 - 14.9	12.2	3.8 - 41.5	34.6	12.9 - 77.1	0.0	0.0 - 0.0	246.1	77.2 - 527.2	0.52
6040002	17622	E ROCK CR	86.2	262.3	94 - 605	0.0	0.0 - 0.0	5.1	1.6 - 11.5	20.4	5.2 - 71.4	47.9	15.1 - 137.8	0.0	0.0 - 0.0	188.9	63.4 - 456.4	0.52
6040002	17623	DUCK R	73.6	207.8	76 - 424	3.6	1.4 - 6.4	5.1	1.9 - 9.7	17.7	5.2 - 42.4	46.0	14.8 - 90.6	0.0	0.0 - 0.0	135.6	49.6 - 298.1	0.57
6040002	17624	DUCK R	9.4	261.1	91 - 672	0.0	0.0 - 0.0	8.1	3.0 - 23.1	21.8	7.1 - 59.0	60.0	20.7 - 167.5	0.0	0.0 - 0.0	171.2	61.0 - 465.7	0.56
6040002	17625	DUCK R	6.2	247.4	83 - 465	0.0	0.0 - 0.0	4.2	1.6 - 7.8	25.6	7.2 - 76.1	53.9	18.4 - 120.1	0.0	0.0 - 0.0	163.7	55.6 - 367.4	0.56
6040002	17626	DUCK R	34.8	214.2	67 - 492	0.0	0.0 - 0.0	4.5	1.4 - 10.0	23.2	5.8 - 65.4	45.5	12.8 - 130.4	0.0	0.0 - 0.0	141.0	44.4 - 336.3	0.56
6040002	17627	SINKING CR	80.1	310.2	96 - 639	0.0	0.0 - 0.0	4.3	1.3 - 10.1	22.6	5.9 - 67.9	44.8	13.2 - 104.8	0.0	0.0 - 0.0	238.6	72.0 - 556.4	0.55
6040002	17628	DUCK R	14.6	241.7	67 - 607	0.0	0.0 - 0.0	5.4	1.5 - 14.2	30.0	8.4 - 86.7	58.7	14.9 - 173.5	0.0	0.0 - 0.0	147.6	42.6 - 403.2	0.55
6040002	17629	DUCK R	45.4	237.3	82 - 524	0.0	0.0 - 0.0	10.5	3.3 - 23.0	28.6	7.4 - 82.1	56.4	20.5 - 161.0	0.0	0.0 - 0.0	141.8	45.8 - 326.4	0.54

8-digit HUC	ID	Name	Area	Catchment Yield		Point sources		Developed Land		Manure		Agricultural Land		Phosphate Mines		Soil parent rock		Frac
				kg/km^2	90% CI	kg/km^2	90% CI	kg/km^2	90% CI	kg/km^2	90% CI	kg/km^2	90% CI	kg/km^2	90% CI	kg/km^2	90% CI	
6040002	17630	SUGAR CR	19.8	212.2	83 - 441	0.0	0.0 - 0.0	4.8	2.0 - 10.4	30.1	9.9 - 86.6	58.4	22.0 - 147.5	0.0	0.0 - 0.0	118.9	46.6 - 318.1	0.54
6040002	17631	DUCK R	4.8	336.2	122 - 815	0.0	0.0 - 0.0	2.3	0.8 - 5.4	50.8	16.5 - 177.9	100.4	35.7 - 243.6	0.0	0.0 - 0.0	182.6	59.7 - 543.3	0.54
6040002	17632	FLAT CR	72.4	525.3	146 - 1322	0.0	0.0 - 0.0	6.6	1.9 - 14.5	25.8	7.1 - 77.0	50.5	16.8 - 112.0	0.0	0.0 - 0.0	442.4	125.2 - 1155	0.54
6040002	17633	DUCK R	264.4	512.9	158 - 1007	63.7	20.0 - 152.4	18.4	6.3 - 40.7	23.8	7.6 - 68.2	47.0	14.1 - 112.6	0.0	0.0 - 0.0	360.0	104.1 - 787.(	0.54
6040002	17634	THOMPSON CR	51.8	97.0	34 - 234	0.0	0.0 - 0.0	7.6	2.9 - 19.0	20.7	5.6 - 63.9	40.2	14.2 - 111.4	0.0	0.0 - 0.0	28.4	9.9 - 78.6	0.52
6040002	17635	DUCK R	2.5	358.9	105 - 686	0.0	0.0 - 0.0	1.5	0.5 - 3.0	51.4	13.0 - 140.8	103.8	34.6 - 243.1	0.0	0.0 - 0.0	202.2	66.3 - 398.0	0.52
6040002	17636	DUCK R	285.3	46.6	14 - 103	0.0	0.0 - 0.0	8.4	2.6 - 17.9	6.2	1.9 - 16.5	16.9	4.6 - 43.0	0.0	0.0 - 0.0	15.1	4.5 - 36.9	0.52
6040002	17637	DUCK R	321.1	217.7	79 - 488	10.9	4.0 - 22.7	15.1	5.4 - 33.4	30.3	9.5 - 89.9	108.6	38.8 - 268.0	0.0	0.0 - 0.0	52.8	17.0 - 157.6	0.26
6040002	17638	WATERACE CR	8.8	149.3	54 - 327	0.0	0.0 - 0.0	9.8	3.6 - 21.3	28.8	9.0 - 77.0	56.2	18.8 - 160.0	0.0	0.0 - 0.0	54.5	20.8 - 125.5	0.52
6040002	17639	WATERACE CR, G.	51.9	100.4	36 - 217	0.0	0.0 - 0.0	6.7	2.2 - 14.0	24.5	7.3 - 70.4	48.3	16.4 - 131.0	0.0	0.0 - 0.0	21.0	7.0 - 50.0	0.51
6040002	17640	WATERACE CR, G.	170.4	63.9	23 - 136	0.0	0.0 - 0.0	7.1	2.7 - 15.1	10.1	3.5 - 32.8	27.7	9.5 - 76.8	0.0	0.0 - 0.0	18.9	6.5 - 48.8	0.47
6040002	17641	WATERACE CR	104.4	104.6	41 - 220	2.3	0.8 - 5.6	6.9	2.7 - 15.8	24.4	8.9 - 68.1	48.1	16.5 - 130.5	0.0	0.0 - 0.0	23.0	8.9 - 58.1	0.51
6040002	17642	FALL CR	110.8	209.8	62 - 384	1.4	0.4 - 3.2	6.7	2.1 - 12.8	28.9	6.7 - 82.4	56.3	15.6 - 147.4	0.0	0.0 - 0.0	116.5	33.6 - 267.2	0.54
6040002	17643	NORTH FK CR	16.5	198.1	70 - 482	0.0	0.0 - 0.0	3.7	1.3 - 8.6	19.0	5.6 - 61.3	36.0	13.0 - 96.5	0.0	0.0 - 0.0	139.3	46.0 - 377.3	0.56
6040002	17645	NORTH FK CR	15.3	246.0	82 - 543	0.0	0.0 - 0.0	10.4	3.3 - 21.4	31.6	10.0 - 83.8	60.7	20.4 - 154.9	0.0	0.0 - 0.0	143.2	48.6 - 374.8	0.54
6040002	17646	NORTH FK CR	76.2	285.8	83 - 582	0.0	0.0 - 0.0	6.2	1.8 - 12.8	32.4	6.9 - 88.4	79.2	24.7 - 201.1	0.0	0.0 - 0.0	167.9	54.1 - 390.0	0.52
6040002	17647	ALEXANDER CR	49.0	204.8	65 - 518	0.0	0.0 - 0.0	5.0	1.6 - 11.2	26.0	7.2 - 94.7	58.9	19.5 - 174.7	0.0	0.0 - 0.0	115.0	35.6 - 296.0	0.52
6040002	17648	WEAKLEY CR	41.1	217.8	71 - 489	0.0	0.0 - 0.0	8.9	3.0 - 19.4	31.8	8.6 - 104.6	61.8	20.1 - 200.7	0.0	0.0 - 0.0	115.3	38.6 - 285.4	0.54
6040002	17649	CLEM CR	39.4	193.0	64 - 440	0.0	0.0 - 0.0	7.1	2.2 - 15.9	24.0	6.7 - 65.8	47.8	14.3 - 125.9	0.0	0.0 - 0.0	114.2	37.2 - 284.4	0.54
6040002	17650	WILSON CR	42.1	250.6	68 - 543	0.0	0.0 - 0.0	4.9	1.5 - 9.2	30.4	8.1 - 91.3	67.9	20.1 - 176.6	0.0	0.0 - 0.0	147.4	43.7 - 320.5	0.56
6040002	17651	SPRING CR	64.8	309.2	96 - 669	0.0	0.0 - 0.0	9.1	2.8 - 18.3	15.2	4.7 - 46.4	47.1	14.3 - 113.8	0.0	0.0 - 0.0	237.7	79.4 - 540.2	0.56
6040002	17652	CANEY CR	76.1	202.3	73 - 508	0.0	0.0 - 0.0	5.9	2.0 - 14.9	12.9	3.4 - 49.6	37.2	13.1 - 100.1	0.0	0.0 - 0.0	146.2	53.5 - 374.6	0.57
6040002	17653	FLAT CR	128.1	281.2	95 - 610	0.0	0.0 - 0.0	4.2	1.4 - 8.8	11.2	3.6 - 37.6	33.5	12.1 - 97.8	0.0	0.0 - 0.0	232.2	77.3 - 533.8	0.57
6040003	17654	DUCK R	11.1	52.9	16 - 130	0.0	0.0 - 0.0	0.4	0.1 - 0.8	3.1	0.8 - 9.2	7.0	1.8 - 17.2	0.0	0.0 - 0.0	42.4	12.7 - 114.3	0.64
6040003	17655	DUCK R	15.4	88.2	30 - 213	0.0	0.0 - 0.0	4.2	1.6 - 9.5	9.7	2.7 - 28.9	25.2	8.3 - 62.5	0.0	0.0 - 0.0	49.1	17.0 - 130.7	0.64
6040003	17656	DUCK R	46.9	59.6	19 - 125	0.0	0.0 - 0.0	2.0	0.5 - 3.8	4.0	1.0 - 8.5	10.4	3.4 - 24.9	0.0	0.0 - 0.0	43.2	13.7 - 99.5	0.64
6040003	17657	DUCK R	22.4	60.4	19 - 154	0.0	0.0 - 0.0	1.3	0.3 - 2.7	4.6	1.4 - 13.3	12.5	3.8 - 32.0	0.0	0.0 - 0.0	42.0	13.0 - 105.8	0.64
6040003	17658	DUCK R	132.7	44.6	13 - 88	0.0	0.0 - 0.0	1.8	0.5 - 3.7	2.3	0.7 - 5.6	5.2	1.5 - 12.2	0.0	0.0 - 0.0	35.4	9.9 - 73.2	0.64
6040003	17659	BEAVERDAM CR	211.7	43.5	16 - 101	0.0	0.0 - 0.0	2.5	0.9 - 6.1	2.7	0.9 - 8.0	6.1	2.0 - 15.0	0.0	0.0 - 0.0	32.2	11.5 - 80.3	0.63
6040003	17660	DUCK R	53.6	45.4	16 - 81	0.0	0.0 - 0.0	1.1	0.4 - 2.1	2.7	0.9 - 5.5	6.5	2.3 - 14.2	0.0	0.0 - 0.0	35.1	12.4 - 66.1	0.63
6040003	17661	DUCK R	152.6	65.5	19 - 115	2.6	0.8 - 5.1	6.8	2.1 - 13.4	5.4	1.6 - 15.3	13.8	3.3 - 32.6	0.3	0.1 - 0.9	36.6	9.7 - 71.2	0.63
6040003	17662	BIG SWAN CR	17.8	59.4	17 - 150	0.0	0.0 - 0.0	5.9	1.5 - 15.2	5.4	1.3 - 15.8	10.6	3.2 - 29.3	0.7	0.2 - 3.1	36.8	10.8 - 100.7	0.62
6040003	17663	BIG SWAN CR	157.0	36.0	11 - 91	0.0	0.0 - 0.0	1.9	0.6 - 4.5	1.7	0.5 - 5.2	4.2	1.4 - 10.6	1.5	0.5 - 4.2	26.7	8.3 - 76.0	0.54
6040003	17664	LITTLE SWAN CR	68.5	38.4	13 - 90	0.0	0.0 - 0.0	6.2	1.9 - 16.2	1.3	0.4 - 3.9	6.4	2.1 - 15.3	0.0	0.0 - 0.0	24.5	7.9 - 64.7	0.26
6040003	17665	BIG SWAN CR	110.9	31.2	9 - 65	0.0	0.0 - 0.0	2.8	0.8 - 5.1	0.9	0.2 - 2.2	4.4	1.1 - 10.0	0.0	0.0 - 0.0	23.1	7.1 - 54.0	0.26
6040003	17666	BLUE BUCK CR	46.7	35.4	11 - 80	0.0	0.0 - 0.0	2.1	0.7 - 4.1	1.3	0.4 - 3.1	1.5	0.5 - 3.2	5.5	1.4 - 15.4	25.1	6.8 - 62.9	0.54
6040003	17667	DUCK R	88.3	78.9	27 - 148	0.0	0.0 - 0.0	3.6	1.1 - 6.8	6.4	1.8 - 17.2	16.8	5.7 - 40.2	0.2	0.1 - 0.5	51.9	18.0 - 104.8	0.62
6040003	17668	DUCK R	127.5	496.8	188 - 1227	0.0	0.0 - 0.0	4.1	1.5 - 11.5	9.0	2.7 - 25.8	23.9	8.4 - 72.3	77.2	24.3 - 247.2	382.5	146.3 - 1012	0.62
6040003	17669	CATHEY'S CR	122.8	202.3	67 - 459	0.0	0.0 - 0.0	2.9	1.1 - 6.9	4.6	1.4 - 13.7	14.0	4.8 - 38.7	0.0	0.0 - 0.0	180.8	60.6 - 434.8	0.61

8-digit HUC	ID	Name	Area	Catchment Yield		Point sources		Developed Land		Manure		Agricultural Land		Phosphate Mines		Soil parent rock		Frac
				kg/km^2	90% CI	kg/km^2	90% CI	kg/km^2	90% CI	kg/km^2	90% CI	kg/km^2	90% CI	kg/km^2	90% CI	kg/km^2	90% CI	
6040003	17671	BIG BIGBY CR	83.4	714.0	273 - 1655	0.0	0.0 - 0.0	4.7	1.5 - 10.7	15.3	4.7 - 41.6	47.0	17.7 - 126.4	18.1	5.8 - 48.4	628.9	227.1 - 1497	0.61
6040003	17672	BIG BIGBY CR	23.1	565.8	175 - 1136	0.0	0.0 - 0.0	5.4	1.8 - 11.5	12.9	3.9 - 37.6	38.3	12.4 - 84.3	30.8	8.6 - 110.4	478.4	154.3 - 961.[illegible]	0.40
6040003	17673	BIG BIGBY CR	44.9	139.7	48 - 344	0.0	0.0 - 0.0	1.8	0.5 - 3.9	2.3	0.7 - 6.7	7.7	2.4 - 19.2	0.0	0.0 - 0.0	127.9	45.1 - 335.1	0.27
6040003	17674	DRY CR	77.4	183.5	63 - 395	2.3	0.9 - 5.1	4.6	1.4 - 10.5	3.7	1.2 - 9.0	11.2	4.1 - 27.7	7.8	2.3 - 24.7	153.8	53.4 - 355.9	0.27
6040003	17675	SCOTT'S CR	105.7	630.1	218 - 1439	6.9	2.6 - 18.2	9.9	3.6 - 21.5	7.0	2.2 - 21.1	21.6	7.0 - 64.6	98.4	27.7 - 287.8	486.4	170.9 - 1179	0.40
6040003	17676	DUCK R	135.0	296.5	106 - 660	0.0	0.0 - 0.0	4.2	1.3 - 9.4	6.3	1.9 - 19.2	17.0	5.8 - 43.8	31.6	9.7 - 87.0	237.4	85.3 - 574.5	0.61
6040003	17677	DUCK R	96.7	1069.9	316 - 2308	20.8	6.6 - 45.5	11.8	3.6 - 23.1	15.3	4.1 - 34.5	46.5	14.3 - 115.7	114.6	38.8 - 256.1	860.9	262.9 - 1991	0.60
6040003	17678	DUCK R	6.8	2605.6	891 - 5706	1348.6	470.5 - 3332.8	3.5	1.1 - 7.8	16.4	4.9 - 40.9	51.1	15.9 - 128.8	120.6	37.0 - 351.3	1065.5	386.5 - 2550	0.60
6040003	17679	LITTLE BIGBY CR	120.1	569.9	223 - 1404	0.0	0.0 - 0.0	15.5	5.9 - 38.8	7.7	2.6 - 22.9	23.2	7.8 - 67.1	58.1	20.0 - 202.5	465.4	176.7 - 1194	0.60
6040003	17680	DUCK R	10.7	1918.1	634 - 3817	0.0	0.0 - 0.0	47.7	16.3 - 103.0	11.2	3.6 - 30.7	32.0	10.2 - 77.7	725.0	219.4 - 2025	1102.2	362.6 - 2398	0.60
6040003	17681	DUCK R	11.2	1242.1	449 - 2617	0.0	0.0 - 0.0	98.6	34.8 - 204.1	2.9	0.9 - 10.2	7.2	2.3 - 17.3	94.6	31.7 - 245.6	1038.9	367.1 - 2346	0.60
6040003	17682	LYTLE CR	27.0	957.1	278 - 1734	0.0	0.0 - 0.0	23.5	6.7 - 38.6	16.3	4.5 - 38.3	48.0	14.4 - 103.0	48.4	11.2 - 120.3	821.0	242.6 - 1513	0.59
6040003	17683	DUCK R	3.8	1125.7	355 - 2610	0.0	0.0 - 0.0	56.2	18.5 - 116.4	13.2	3.5 - 38.1	39.9	11.3 - 109.6	0.0	0.0 - 0.0	1016.3	324.9 - 2428	0.59
6040003	17684	DUCK R	3.7	1197.1	384 - 2461	0.0	0.0 - 0.0	7.1	2.4 - 14.3	21.0	5.8 - 55.8	59.5	18.5 - 140.2	0.0	0.0 - 0.0	1109.5	361.8 - 2349	0.59
6040003	17685	BEAR CR	29.4	903.7	273 - 1895	0.0	0.0 - 0.0	14.0	4.5 - 27.9	11.5	3.3 - 28.9	36.0	12.6 - 92.7	199.2	53.4 - 627.7	643.0	220.1 - 1578	0.59
6040003	17686	RUTHERFORD CR	74.5	1018.9	326 - 2197	0.8	0.2 - 1.6	11.3	3.5 - 25.2	14.3	3.8 - 40.2	42.8	13.3 - 98.4	149.3	42.7 - 379.0	800.4	262.9 - 1829	0.60
6040003	17687	RUTHERFORD CR	50.6	972.5	337 - 2295	38.6	13.6 - 96.5	16.5	5.6 - 36.2	19.9	6.0 - 61.4	60.0	17.8 - 160.3	9.9	3.2 - 30.3	827.6	286.0 - 2038	0.59
6040003	17688	RUTHERFORD CR	63.1	594.5	215 - 1335	0.0	0.0 - 0.0	7.3	2.2 - 16.8	15.8	4.5 - 48.0	47.9	15.6 - 125.5	0.0	0.0 - 0.0	523.5	188.6 - 1270	0.59
6040003	17689	AENON CR	39.4	649.6	215 - 1423	0.0	0.0 - 0.0	28.6	8.4 - 72.2	16.1	4.2 - 52.6	47.8	12.8 - 141.7	0.0	0.0 - 0.0	557.1	182.8 - 1349	0.59
6040003	17690	CARTER'S CR	73.2	489.4	152 - 1097	0.0	0.0 - 0.0	9.6	3.2 - 21.5	11.1	3.6 - 28.0	33.4	10.5 - 83.9	4.9	1.3 - 13.9	430.4	130.5 - 990.[illegible]	0.59
6040003	17691	TURKEY CR	44.2	365.5	110 - 858	0.0	0.0 - 0.0	3.1	0.9 - 6.5	4.6	1.1 - 11.8	12.6	3.6 - 32.3	93.3	24.1 - 335.9	251.9	74.8 - 560.8	0.60
6040003	17692	LEIPER'S CR	70.8	237.0	76 - 681	0.0	0.0 - 0.0	3.0	0.9 - 9.9	4.5	1.2 - 14.5	13.0	3.7 - 39.6	20.9	5.0 - 85.5	195.6	59.4 - 570.2	0.60
6040003	17693	LICK CR	38.8	47.1	18 - 113	0.0	0.0 - 0.0	3.6	1.3 - 9.3	3.6	1.1 - 10.5	7.2	2.8 - 20.4	0.1	0.0 - 0.3	32.6	12.4 - 82.4	0.62
6040003	17694	DOG CR	24.7	40.5	14 - 90	0.0	0.0 - 0.0	2.8	0.8 - 7.4	3.3	0.9 - 8.0	8.5	2.8 - 20.0	0.0	0.0 - 0.0	26.0	9.3 - 66.0	0.49
6040003	17695	LICK CR	1.6	59.5	21 - 128	0.0	0.0 - 0.0	8.2	2.5 - 17.6	7.0	2.2 - 17.2	11.2	4.2 - 27.4	0.0	0.0 - 0.0	33.0	10.8 - 74.3	0.49
6040003	17696	LICK CR	6.9	46.5	15 - 117	0.0	0.0 - 0.0	2.5	0.8 - 6.9	5.1	1.4 - 16.6	2.8	0.9 - 8.6	0.0	0.0 - 0.0	36.0	12.4 - 89.2	0.46
6040003	17697	LICK CR	87.7	30.4	10 - 67	0.0	0.0 - 0.0	2.5	0.8 - 5.6	2.5	0.7 - 6.2	5.3	1.7 - 12.3	0.0	0.0 - 0.0	20.1	6.9 - 51.6	0.41
6040003	17698	LICK CR, BARREN	8.4	52.9	22 - 118	0.0	0.0 - 0.0	5.3	2.0 - 10.5	4.9	1.6 - 11.7	9.7	3.6 - 24.2	0.0	0.0 - 0.0	33.0	12.6 - 72.0	0.41
6040003	17699	YOUNGER CR	44.6	51.8	18 - 106	0.0	0.0 - 0.0	4.4	1.6 - 10.2	5.5	1.9 - 17.2	13.9	4.3 - 36.0	0.0	0.0 - 0.0	28.1	9.3 - 64.4	0.34
6040003	17700	LICK CR, BARREN	19.4	61.2	20 - 120	0.0	0.0 - 0.0	5.0	1.4 - 10.8	6.3	1.7 - 21.0	13.6	4.3 - 34.9	0.0	0.0 - 0.0	36.3	11.2 - 91.7	0.34
6040003	17701	TATUM CR	30.6	41.2	13 - 84	0.0	0.0 - 0.0	3.6	1.3 - 7.5	3.4	1.0 - 10.2	5.8	2.0 - 16.0	0.0	0.0 - 0.0	28.4	8.4 - 62.8	0.46
6040003	17702	PINEY R	83.7	62.0	19 - 142	0.0	0.0 - 0.0	3.5	1.0 - 8.3	5.8	1.5 - 15.5	13.9	4.1 - 34.3	0.0	0.0 - 0.0	38.7	11.6 - 96.9	0.63
6040003	17703	MILL CR	88.3	50.7	18 - 115	0.0	0.0 - 0.0	5.0	1.6 - 11.3	4.5	1.4 - 13.0	10.0	3.4 - 27.0	0.0	0.0 - 0.0	31.2	11.0 - 74.1	0.61
6040003	17704	PINEY R	69.0	47.8	17 - 100	0.0	0.0 - 0.0	2.9	0.9 - 6.6	4.2	1.2 - 10.9	8.5	2.5 - 20.0	0.0	0.0 - 0.0	32.2	11.2 - 81.4	0.61
6040003	17705	BIG SPRING CR	60.0	61.6	21 - 133	0.0	0.0 - 0.0	5.7	2.1 - 13.0	6.9	2.2 - 18.5	16.5	5.2 - 43.6	0.0	0.0 - 0.0	32.5	10.8 - 77.2	0.60
6040003	17706	PINEY R	2.7	94.5	35 - 205	0.0	0.0 - 0.0	3.9	1.4 - 7.6	12.9	4.6 - 34.0	31.0	11.5 - 79.5	0.0	0.0 - 0.0	46.7	18.5 - 106.1	0.60
6040003	17707	PINEY R	16.2	48.0	18 - 94	0.0	0.0 - 0.0	4.5	1.6 - 9.1	3.7	1.2 - 9.5	4.4	1.5 - 12.0	0.0	0.0 - 0.0	35.3	13.4 - 78.3	0.60
6040003	17708	BEAR CR	58.3	74.6	27 - 188	0.0	0.0 - 0.0	6.1	2.0 - 15.1	8.1	2.3 - 30.2	17.6	6.1 - 42.7	0.0	0.0 - 0.0	42.8	15.2 - 108.8	0.57
6040003	17709	PINEY R	129.6	101.0	34 - 181	0.0	0.0 - 0.0	12.0	4.1 - 25.3	10.2	2.9 - 25.1	28.9	9.9 - 69.0	0.0	0.0 - 0.0	50.0	18.0 - 110.3	0.57

8-digit HUC	ID	Name	Area	Catchment Yield		Point sources		Developed Land		Manure		Agricultural Land		Phosphate Mines		Soil parent rock		Frac
				kg/km^2	90% CI	kg/km^2	90% CI	kg/km^2	90% CI	kg/km^2	90% CI	kg/km^2	90% CI	kg/km^2	90% CI	kg/km^2	90% CI	
6040003	17710	GARNER CR	70.5	74.8	21 - 157	0.0	0.0 - 0.0	5.0	1.7 - 10.7	7.7	2.1 - 19.4	18.5	6.0 - 47.6	0.0	0.0 - 0.0	43.5	14.3 - 103.3	0.60
6040003	17711	SUGAR CR	82.2	33.2	12 - 78	0.0	0.0 - 0.0	3.1	0.9 - 6.8	0.8	0.3 - 2.0	1.2	0.3 - 2.9	0.0	0.0 - 0.0	28.1	9.7 - 76.3	0.64
6040003	17712	TUMBLING CR	133.8	40.6	14 - 86	0.0	0.0 - 0.0	1.7	0.5 - 3.8	2.7	0.8 - 8.0	4.4	1.5 - 11.2	0.0	0.0 - 0.0	31.7	10.6 - 78.9	0.64
6040003	17713	HURRICANE CR	206.6	57.0	17 - 128	0.0	0.0 - 0.0	3.1	1.0 - 7.0	4.6	1.5 - 11.0	9.5	3.0 - 22.7	0.2	0.0 - 0.7	39.7	11.3 - 102.3	0.64
6040003	17714	BLUE CR	124.3	62.4	23 - 140	5.0	1.6 - 12.7	3.7	1.3 - 9.0	4.7	1.5 - 15.9	11.1	3.6 - 30.9	0.0	0.0 - 0.0	37.7	14.5 - 97.8	0.64
6040003	17715	DUCK R	15.5	78.9	29 - 153	0.0	0.0 - 0.0	1.8	0.6 - 3.6	8.0	2.5 - 19.2	21.1	7.1 - 47.6	0.0	0.0 - 0.0	48.1	18.2 - 96.5	0.65
6040004	17716	BUFFALO R	236.8	63.9	24 - 129	1.4	0.5 - 3.1	3.6	1.2 - 7.2	4.4	1.3 - 11.6	12.9	4.4 - 33.2	0.0	0.0 - 0.0	41.5	15.1 - 93.7	0.64
6040004	17717	BUFFALO R	31.7	56.1	20 - 141	0.0	0.0 - 0.0	4.1	1.4 - 9.4	1.7	0.6 - 5.1	7.4	2.5 - 20.9	0.0	0.0 - 0.0	42.8	15.5 - 106.2	0.62
6040004	17718	BUFFALO R	16.8	48.1	21 - 100	0.0	0.0 - 0.0	4.3	1.5 - 8.6	1.3	0.4 - 3.0	3.1	1.0 - 7.6	0.0	0.0 - 0.0	39.4	17.1 - 92.1	0.61
6040004	17719	BUFFALO R	57.6	69.9	23 - 188	7.9	2.6 - 22.7	7.0	2.1 - 18.9	2.2	0.6 - 7.6	8.0	2.6 - 27.9	0.0	0.0 - 0.0	44.7	14.0 - 122.3	0.61
6040004	17720	BUFFALO R	57.1	57.1	21 - 128	0.0	0.0 - 0.0	3.5	1.3 - 7.2	2.7	0.8 - 8.5	10.4	3.4 - 32.2	0.0	0.0 - 0.0	40.4	14.0 - 90.7	0.60
6040004	17721	BUFFALO R	10.8	72.0	25 - 154	0.0	0.0 - 0.0	10.3	3.6 - 26.2	4.5	1.4 - 13.8	20.2	7.1 - 57.5	0.0	0.0 - 0.0	37.1	12.7 - 89.7	0.59
6040004	17722	GREEN R	156.4	82.2	28 - 159	2.6	0.9 - 5.6	11.2	3.8 - 23.7	5.8	1.8 - 13.5	16.1	5.1 - 38.8	0.0	0.0 - 0.0	46.4	15.3 - 104.2	0.57
6040004	17723	BUFFALO R	65.4	72.4	27 - 171	0.0	0.0 - 0.0	4.5	1.6 - 9.9	5.7	1.7 - 17.1	18.9	6.2 - 48.9	0.0	0.0 - 0.0	43.4	15.7 - 104.7	0.57
6040004	17724	FORTYEIGHT CR	144.2	69.7	28 - 162	0.0	0.0 - 0.0	6.2	2.5 - 15.7	4.5	1.5 - 15.8	13.5	4.6 - 34.8	0.0	0.0 - 0.0	45.5	17.6 - 118.4	0.56
6040004	17725	BUFFALO R	44.2	72.3	23 - 147	0.0	0.0 - 0.0	4.2	1.2 - 9.3	5.0	1.4 - 13.1	18.3	5.4 - 43.1	0.0	0.0 - 0.0	44.8	13.9 - 98.8	0.56
6040004	17727	BUFFALO R	39.2	55.6	20 - 160	0.0	0.0 - 0.0	4.0	1.1 - 11.7	2.6	0.7 - 8.6	10.1	3.2 - 33.2	0.0	0.0 - 0.0	39.0	13.4 - 113.4	0.55
6040004	17728	LITTLE BUFFALO	1.3	108.4	33 - 240	0.0	0.0 - 0.0	2.2	0.6 - 5.4	8.8	2.2 - 23.7	47.3	12.8 - 134.8	0.0	0.0 - 0.0	50.2	17.3 - 114.7	0.54
6040004	17729	BRUSH CR	36.2	48.8	20 - 125	0.0	0.0 - 0.0	2.6	0.9 - 6.3	2.8	0.9 - 9.2	8.5	3.1 - 27.4	0.8	0.3 - 3.1	34.1	14.3 - 93.6	0.53
6040004	17731	LITTLE BUFFALO	102.6	62.6	19 - 142	0.0	0.0 - 0.0	5.1	1.6 - 11.9	5.0	1.5 - 15.1	15.9	4.7 - 44.3	0.0	0.0 - 0.0	36.5	11.1 - 84.8	0.53
6040004	17732	CHIEF CR	60.5	65.9	20 - 152	0.0	0.0 - 0.0	5.3	1.6 - 11.0	6.0	1.9 - 14.6	17.9	5.1 - 45.4	0.0	0.0 - 0.0	36.8	12.4 - 98.5	0.53
6040004	17733	BUFFALO R	3.7	70.2	26 - 139	0.0	0.0 - 0.0	10.8	3.6 - 21.5	3.1	1.1 - 8.8	16.6	5.3 - 38.2	0.0	0.0 - 0.0	39.6	16.1 - 90.9	0.54
6040004	17734	BUFFALO R	81.6	53.4	16 - 138	0.0	0.0 - 0.0	3.6	1.2 - 8.9	3.1	1.0 - 9.8	12.3	3.8 - 35.8	0.0	0.0 - 0.0	34.3	11.2 - 99.3	0.53
6040004	17735	BUFFALO R	13.9	65.6	24 - 139	0.0	0.0 - 0.0	4.3	1.4 - 8.9	6.6	1.9 - 17.4	19.2	6.5 - 53.7	2.6	0.7 - 8.4	33.0	11.7 - 70.9	0.50
6040004	17736	WALKER CR	64.8	115.8	37 - 205	0.0	0.0 - 0.0	6.9	2.0 - 13.1	16.9	4.7 - 40.6	51.8	18.6 - 113.8	0.0	0.0 - 0.0	40.1	11.2 - 84.4	0.48
6040004	17737	BUFFALO R	93.6	111.6	43 - 223	0.0	0.0 - 0.0	8.2	3.4 - 18.0	14.9	5.4 - 36.2	45.2	15.0 - 129.5	0.0	0.0 - 0.0	43.3	18.0 - 101.5	0.48
6040004	17738	SAW CR	34.3	82.7	32 - 205	0.0	0.0 - 0.0	12.5	4.7 - 29.3	7.8	2.3 - 30.6	24.2	8.6 - 63.8	0.0	0.0 - 0.0	38.2	14.7 - 109.0	0.50
6040004	17739	GRINDERS CR	41.4	74.9	28 - 143	0.0	0.0 - 0.0	4.4	1.5 - 9.6	5.7	1.8 - 15.1	28.6	8.9 - 61.7	0.0	0.0 - 0.0	36.2	11.8 - 68.1	0.53
6040004	17740	ROCKHOUSE CR	52.3	86.6	28 - 177	14.5	5.0 - 30.8	12.9	4.3 - 27.8	3.5	1.0 - 9.1	18.4	5.5 - 47.0	0.0	0.0 - 0.0	37.4	11.7 - 88.8	0.55
6040004	17741	TRACE CR	64.2	50.9	13 - 94	0.0	0.0 - 0.0	4.4	1.1 - 9.3	2.3	0.5 - 5.1	10.8	2.9 - 24.9	0.0	0.0 - 0.0	33.4	9.0 - 75.9	0.55
6040004	17742	SINKING CR	43.6	53.2	17 - 130	0.0	0.0 - 0.0	3.0	1.0 - 7.0	2.1	0.6 - 6.0	8.2	2.5 - 23.8	0.0	0.0 - 0.0	39.9	13.2 - 99.6	0.59
6040004	17743	HURRICANE CR	37.9	43.8	16 - 92	0.0	0.0 - 0.0	1.4	0.4 - 2.8	0.7	0.2 - 1.7	1.7	0.6 - 4.1	0.0	0.0 - 0.0	40.0	14.7 - 84.1	0.60
6040004	17744	COON CR	57.2	48.5	15 - 104	0.0	0.0 - 0.0	3.8	1.2 - 8.1	0.8	0.2 - 2.5	2.0	0.5 - 6.3	0.0	0.0 - 0.0	41.9	13.0 - 104.2	0.61
6040004	17745	BRUSH CR	41.4	52.3	19 - 140	7.2	2.6 - 21.2	2.6	0.9 - 6.4	0.8	0.3 - 2.6	1.1	0.3 - 3.4	0.0	0.0 - 0.0	40.7	15.0 - 110.0	0.61
6040004	17746	CANE CR	220.8	48.0	23 - 87	0.0	0.0 - 0.0	3.2	1.3 - 6.1	1.7	0.6 - 4.3	5.5	2.1 - 11.2	0.2	0.1 - 0.4	37.5	16.8 - 76.1	0.62
6040005	17747	W SANDY CR, MC	209.1	132.3	38 - 250	19.1	6.2 - 37.6	11.7	3.7 - 23.5	13.9	3.4 - 34.9	50.0	14.1 - 115.9	0.0	0.0 - 0.0	37.7	10.8 - 90.5	0.64
6040005	17748	W SANDY CR	157.4	104.6	37 - 245	0.0	0.0 - 0.0	6.5	2.3 - 16.1	13.5	4.2 - 42.6	48.7	17.0 - 148.2	0.0	0.0 - 0.0	35.9	12.7 - 104.4	0.64
6040005	17749	BIG SANDY CR	12.4	85.2	27 - 171	0.0	0.0 - 0.0	6.5	2.1 - 12.6	9.7	3.1 - 23.7	40.1	12.1 - 101.1	0.0	0.0 - 0.0	29.0	10.4 - 73.6	0.64
6040005	17750	BEAR CR	75.1	68.0	22 - 121	0.0	0.0 - 0.0	3.3	1.0 - 5.9	8.2	2.2 - 18.1	29.8	9.0 - 67.6	0.0	0.0 - 0.0	26.6	8.4 - 56.1	0.63

8-digit HUC	ID	Name	Area	Catchment Yield		Point sources		Developed Land		Manure		Agricultural Land		Phosphate Mines		Soil parent rock		Frac
				kg/km^2	90% CI	kg/km^2	90% CI	kg/km^2	90% CI	kg/km^2	90% CI	kg/km^2	90% CI	kg/km^2	90% CI	kg/km^2	90% CI	
6040005	17751	BIG SANDY CR	12.4	154.8	51 - 327	0.0	0.0 - 0.0	2.8	0.8 - 5.6	17.0	4.7 - 41.7	74.5	22.1 - 195.9	0.0	0.0 - 0.0	60.5	17.6 - 146.0	0.63
6040005	17752	BIG SANDY CR	356.9	81.3	26 - 205	0.7	0.2 - 1.7	5.5	1.8 - 13.8	5.3	1.5 - 15.8	31.1	10.9 - 81.3	0.0	0.0 - 0.0	38.7	11.9 - 113.1	0.63
6040005	17753	BIG SANDY CR	100.5	95.5	39 - 203	0.0	0.0 - 0.0	4.7	1.4 - 10.9	6.1	2.0 - 15.4	43.0	16.3 - 97.7	0.0	0.0 - 0.0	41.6	14.8 - 110.8	0.59
6040005	17754	ROAN CR	32.5	154.6	41 - 372	0.0	0.0 - 0.0	5.8	1.6 - 12.0	11.5	2.7 - 37.9	89.3	24.8 - 255.7	0.0	0.0 - 0.0	47.9	12.1 - 138.7	0.56
6040005	17755	BIG SANDY CR	192.3	94.6	33 - 219	0.0	0.0 - 0.0	5.6	2.1 - 13.5	11.7	3.3 - 30.1	47.2	17.9 - 119.0	0.0	0.0 - 0.0	30.1	10.4 - 73.7	0.56
6040005	17756	MAPLE CR	33.3	52.4	18 - 121	0.0	0.0 - 0.0	3.4	1.2 - 8.3	1.5	0.4 - 4.2	8.2	2.9 - 23.9	0.0	0.0 - 0.0	39.3	12.4 - 105.3	0.59
6040005	17757	DRY CR	27.0	118.2	39 - 241	0.0	0.0 - 0.0	4.8	1.5 - 12.8	12.3	3.4 - 34.0	45.9	13.9 - 104.8	0.0	0.0 - 0.0	55.2	17.8 - 140.6	0.63
6040005	17758	RUSHING CR	59.7	183.1	60 - 460	0.0	0.0 - 0.0	7.3	2.5 - 17.9	23.8	5.6 - 80.3	93.9	30.2 - 277.8	0.0	0.0 - 0.0	58.1	17.3 - 166.5	0.64
6040006	17759	CLARK'S R	141.3	233.1	64 - 650	6.5	2.0 - 16.5	18.9	5.2 - 42.4	2.7	0.6 - 9.1	69.5	18.8 - 198.2	0.0	0.0 - 0.0	135.6	36.0 - 391.8	1.00
6040006	17760	CLARK'S R, W FK	2.8	302.2	87 - 873	0.0	0.0 - 0.0	1.7	0.7 - 3.7	1.9	0.5 - 6.4	47.1	14.9 - 141.9	0.0	0.0 - 0.0	251.6	70.7 - 802.4	0.98
6040006	17761	CAMP CR	42.7	153.8	55 - 336	0.0	0.0 - 0.0	3.4	1.3 - 6.4	16.8	5.4 - 59.3	77.1	30.8 - 180.2	0.0	0.0 - 0.0	56.4	19.6 - 170.7	0.97
6040006	17762	CLARK'S R, W FK	139.8	171.4	47 - 411	0.9	0.3 - 2.2	4.0	1.2 - 9.7	23.0	6.0 - 57.2	74.4	20.5 - 204.3	0.0	0.0 - 0.0	69.0	19.5 - 187.1	0.97
6040006	17763	TRACE CR	22.6	148.0	47 - 318	0.0	0.0 - 0.0	4.2	1.3 - 10.4	24.6	6.9 - 70.4	77.3	23.9 - 194.2	0.0	0.0 - 0.0	41.9	12.9 - 104.1	0.93
6040006	17764	CLARK'S R, W FK	88.5	142.4	39 - 300	0.0	0.0 - 0.0	4.0	1.2 - 8.7	22.6	5.2 - 70.0	71.6	21.4 - 178.4	0.0	0.0 - 0.0	44.2	13.4 - 122.9	0.93
6040006	17765	CLARK'S R, W FK	190.7	192.7	71 - 439	0.0	0.0 - 0.0	5.2	1.8 - 11.2	24.6	8.5 - 84.1	109.3	39.2 - 283.3	0.0	0.0 - 0.0	53.6	19.8 - 142.6	0.91
6040006	17766	SOLDIER CR	51.5	210.8	61 - 473	0.0	0.0 - 0.0	4.4	1.3 - 9.3	25.0	6.5 - 79.9	100.2	29.9 - 237.5	0.0	0.0 - 0.0	81.1	25.4 - 221.4	0.91
6040006	17767	CLARK'S R	101.3	172.9	53 - 383	0.2	0.0 - 0.4	4.2	1.2 - 9.0	15.5	3.9 - 45.4	65.5	20.1 - 161.5	0.0	0.0 - 0.0	87.5	25.5 - 209.6	0.98
6040006	17768	SMITH CR	61.4	183.1	53 - 467	0.0	0.0 - 0.0	5.7	1.6 - 13.3	22.2	6.3 - 65.6	87.7	23.6 - 230.4	0.0	0.0 - 0.0	67.4	19.4 - 193.3	0.95
6040006	17769	CLARK'S R	373.8	246.0	103 - 514	44.8	17.1 - 100.7	10.6	3.7 - 22.4	22.2	7.7 - 68.4	101.0	38.2 - 271.9	0.0	0.0 - 0.0	67.4	27.8 - 185.2	0.95
6040006	17770	CLARK'S R	127.6	383.2	107 - 952	0.5	0.2 - 1.3	18.5	5.7 - 39.6	45.0	10.2 - 135.2	200.9	55.3 - 544.1	0.0	0.0 - 0.0	118.2	34.3 - 327.7	0.86
6040006	17771	CLARK'S R, E FK	75.9	403.6	144 - 965	0.0	0.0 - 0.0	6.8	2.3 - 14.4	49.7	14.7 - 141.5	252.4	83.6 - 658.0	0.0	0.0 - 0.0	94.7	29.3 - 239.1	0.81
6040006	17772	CLARK'S R	35.1	358.7	123 - 853	0.0	0.0 - 0.0	6.0	2.2 - 13.7	46.1	13.7 - 159.0	223.7	73.7 - 591.3	0.0	0.0 - 0.0	82.9	24.7 - 218.6	0.81
6040006	17773	CLAYTON CR	42.7	474.3	148 - 954	0.1	0.0 - 0.2	8.2	2.7 - 15.6	53.2	13.4 - 157.8	269.9	82.2 - 608.0	0.0	0.0 - 0.0	142.9	40.6 - 336.6	0.86
6040006	17774	TENNESSEE R	3.8	267.7	86 - 712	0.6	0.2 - 1.5	15.1	4.6 - 34.1	15.7	4.1 - 49.7	92.4	28.5 - 281.4	0.0	0.0 - 0.0	143.9	42.2 - 392.9	1.00
6020001	17775	SEWEE CR	69.7	63.4	26 - 115	0.0	0.0 - 0.0	5.8	2.1 - 10.8	9.1	2.7 - 21.9	29.0	10.4 - 64.1	0.0	0.0 - 0.0	19.4	8.1 - 44.6	0.34
6010201	17776	RILEY CR	36.6	52.3	18 - 119	0.0	0.0 - 0.0	7.0	2.2 - 15.3	6.2	1.9 - 17.3	20.5	7.4 - 52.9	0.0	0.0 - 0.0	18.7	6.9 - 49.8	0.30
6010102	17777	HOLSTON R, S FK	92.8	39.2	12 - 75	0.0	0.0 - 0.0	6.0	1.6 - 13.8	6.9	1.9 - 20.3	15.7	4.9 - 46.0	0.0	0.0 - 0.0	10.6	3.0 - 26.8	0.03
6010102	17778	SPRING CR	37.5	59.1	20 - 144	0.0	0.0 - 0.0	9.9	3.3 - 26.0	10.9	3.1 - 31.2	25.2	8.5 - 61.0	0.0	0.0 - 0.0	13.1	4.4 - 34.1	0.03
6010102	17779	HOLSTON R, S FK	133.8	6.9	2 - 14	0.0	0.0 - 0.0	0.8	0.3 - 1.7	0.3	0.1 - 1.2	0.8	0.2 - 1.5	0.0	0.0 - 0.0	5.0	1.5 - 11.6	0.07
6010102	17780	HOLSTON R, S FK	91.5	85.7	32 - 224	27.3	9.7 - 70.8	15.3	5.8 - 39.5	9.0	2.9 - 26.1	20.7	7.1 - 65.0	0.0	0.0 - 0.0	13.3	4.8 - 35.3	0.03
6010102	17781	SPRING CR	7.5	59.2	19 - 117	0.0	0.0 - 0.0	5.9	1.7 - 13.8	12.6	3.3 - 33.6	29.4	9.4 - 73.9	0.0	0.0 - 0.0	11.3	3.9 - 29.5	0.03
6010102	17782	HOLSTON R, S FK	7.5	57.8	16 - 153	0.0	0.0 - 0.0	3.8	1.1 - 10.6	10.3	2.5 - 36.9	30.5	9.1 - 89.9	0.0	0.0 - 0.0	13.2	3.5 - 39.6	0.07
6010103	17783	BOONE'S CR	41.9	60.6	20 - 128	0.0	0.0 - 0.0	16.8	6.1 - 38.9	10.9	3.3 - 28.9	23.8	7.6 - 55.4	0.0	0.0 - 0.0	9.1	3.2 - 24.6	0.07
6010103	17784	WATAUGA R	4.6	42.5	15 - 90	0.0	0.0 - 0.0	4.4	1.6 - 8.6	8.0	2.3 - 27.2	17.5	5.7 - 45.7	0.0	0.0 - 0.0	12.6	4.8 - 30.4	0.07
6010102	17785	HOLSTON R, S FK	3.7	33.9	12 - 84	0.0	0.0 - 0.0	12.0	3.9 - 29.4	4.6	1.5 - 15.1	8.9	3.1 - 27.3	0.0	0.0 - 0.0	8.5	2.8 - 23.0	0.09
6010103	17787	WATAUGA R	62.3	52.3	15 - 103	0.0	0.0 - 0.0	9.3	2.6 - 19.1	9.3	2.5 - 21.0	23.0	7.0 - 58.3	0.0	0.0 - 0.0	10.7	2.8 - 25.0	0.07
6010102	17788	HOLSTON R, S FK	86.2	53.0	19 - 106	0.0	0.0 - 0.0	9.7	3.4 - 21.1	8.3	2.6 - 22.7	24.0	8.5 - 63.2	0.0	0.0 - 0.0	11.1	4.0 - 26.9	0.07
6010103	17789	BOONE'S CR	2.0	70.7	26 - 138	0.0	0.0 - 0.0	29.5	10.5 - 60.2	10.3	2.9 - 30.9	21.4	7.7 - 55.8	0.0	0.0 - 0.0	9.5	3.4 - 22.9	0.07
6010103	17790	WATAUGA R	76.8	125.6	40 - 275	60.9	18.9 - 154.0	33.4	10.5 - 74.2	6.4	1.8 - 18.1	14.5	4.6 - 35.9	0.0	0.0 - 0.0	10.5	3.4 - 25.3	0.07

8-digit HUC	ID	Name	Area	Catchment Yield		Point sources		Developed Land		Manure		Agricultural Land		Phosphate Mines		Soil parent rock		Frac
				kg/km^2	90% CI	kg/km^2	90% CI	kg/km^2	90% CI	kg/km^2	90% CI	kg/km^2	90% CI	kg/km^2	90% CI	kg/km^2	90% CI	
6010103	17791	ROAN CR	2.8	10.2	3 - 25	0.0	0.0 - 0.0	0.9	0.3 - 2.0	0.1	0.0 - 0.2	0.0	0.0 - 0.0	0.0	0.0 - 0.0	9.2	2.9 - 23.3	0.03
6010103	17792	WATAUGA R	211.2	22.0	7 - 57	1.3	0.4 - 3.8	5.1	1.6 - 13.1	1.1	0.3 - 3.6	3.5	1.1 - 10.8	0.0	0.0 - 0.0	11.0	3.7 - 34.6	0.03
6010103	17793	ELK R	207.0	21.9	9 - 56	3.4	1.2 - 9.9	5.7	2.0 - 13.6	0.3	0.1 - 1.1	2.0	0.6 - 5.7	0.0	0.0 - 0.0	10.5	4.1 - 26.8	0.03
6010103	17794	WATAUGA R	104.3	8.3	3 - 19	0.0	0.0 - 0.0	1.7	0.5 - 4.3	0.4	0.1 - 1.0	1.0	0.4 - 2.6	0.0	0.0 - 0.0	5.2	1.7 - 11.7	0.07
6010103	17795	WATAUGA R	3.1	24.2	9 - 60	0.0	0.0 - 0.0	5.3	1.9 - 13.0	1.2	0.4 - 3.7	3.1	1.0 - 7.6	0.0	0.0 - 0.0	14.6	4.8 - 37.4	0.03
6010103	17796	ROAN CR	31.1	18.0	7 - 36	0.0	0.0 - 0.0	2.5	0.9 - 5.5	1.7	0.6 - 5.0	4.3	1.6 - 13.2	0.0	0.0 - 0.0	9.6	3.7 - 22.1	0.03
6010103	17797	WATAUGA R	8.4	21.1	8 - 40	0.0	0.0 - 0.0	3.6	1.3 - 7.7	1.9	0.5 - 5.0	4.9	1.6 - 11.1	0.0	0.0 - 0.0	10.7	3.7 - 27.3	0.03
6010103	17798	ELK R	6.6	23.2	10 - 53	0.0	0.0 - 0.0	6.8	2.7 - 16.3	1.0	0.4 - 3.1	3.2	1.1 - 8.0	0.0	0.0 - 0.0	12.2	5.3 - 30.8	0.03
6010104	17799	POOR VALLEY CR	111.3	15.1	6 - 31	0.0	0.0 - 0.0	6.7	2.5 - 15.2	1.7	0.6 - 5.1	2.5	0.8 - 7.0	0.0	0.0 - 0.0	4.2	1.7 - 9.2	0.13
6010104	17800	HOLSTON R	224.6	45.2	15 - 91	6.9	2.4 - 14.9	8.6	2.6 - 17.2	6.7	1.9 - 20.9	12.8	4.3 - 31.7	0.0	0.0 - 0.0	10.1	3.4 - 24.7	0.13
6010104	17801	HOLSTON R	629.8	31.5	13 - 59	8.0	3.3 - 17.1	7.2	2.4 - 15.7	3.6	1.1 - 8.8	6.8	2.7 - 17.8	0.0	0.0 - 0.0	5.8	2.0 - 13.3	0.25
6010104	17802	POOR VALLEY CR	25.8	43.6	14 - 86	0.0	0.0 - 0.0	8.7	2.9 - 19.7	8.6	2.8 - 28.5	14.2	4.5 - 35.7	0.0	0.0 - 0.0	12.1	4.1 - 31.1	0.13
6010104	17803	HOLSTON R	26.5	20.6	7 - 42	0.0	0.0 - 0.0	6.0	2.1 - 12.8	2.3	0.7 - 6.7	2.5	0.9 - 6.4	0.0	0.0 - 0.0	9.6	3.0 - 26.5	0.13
6010205	17804	INDIAN CR	55.4	28.0	12 - 55	0.0	0.0 - 0.0	5.8	2.4 - 12.0	4.7	1.7 - 15.9	4.6	1.8 - 11.1	0.0	0.0 - 0.0	12.8	5.3 - 28.4	0.11
6010205	17805	CLINCH R	18.4	22.8	8 - 43	0.0	0.0 - 0.0	4.7	1.4 - 9.5	3.3	0.9 - 8.5	2.9	0.8 - 6.7	0.0	0.0 - 0.0	11.9	3.9 - 26.2	0.11
6010205	17806	CLINCH R	7.2	29.0	8 - 64	0.0	0.0 - 0.0	8.6	2.5 - 19.0	4.1	1.1 - 14.8	3.7	1.1 - 9.5	0.0	0.0 - 0.0	12.6	3.2 - 30.8	0.11
6010205	17807	BIG SYCAMORE CI	90.5	19.4	6 - 34	0.0	0.0 - 0.0	4.4	1.4 - 8.0	3.0	0.8 - 8.7	4.3	1.7 - 9.1	0.0	0.0 - 0.0	7.7	2.5 - 15.1	0.11
6010205	17808	LITTLE SYCAMOR	45.5	38.5	12 - 74	0.0	0.0 - 0.0	4.8	1.4 - 10.8	9.6	2.7 - 26.4	12.0	3.3 - 31.6	0.0	0.0 - 0.0	12.0	3.8 - 27.1	0.11
6010205	17809	CLINCH R	356.5	19.8	6 - 48	4.2	1.4 - 10.3	8.7	2.7 - 19.3	0.8	0.2 - 2.1	1.3	0.4 - 3.5	0.0	0.0 - 0.0	4.8	1.6 - 10.7	0.25
6010205	17810	CLINCH R	530.4	35.7	13 - 84	0.0	0.0 - 0.0	7.6	2.6 - 19.3	5.3	1.6 - 13.1	8.3	2.5 - 21.3	0.0	0.0 - 0.0	14.5	5.9 - 37.4	0.11
6010205	17811	CLINCH R	94.8	32.0	13 - 65	0.0	0.0 - 0.0	6.5	2.2 - 13.3	5.9	2.2 - 16.0	6.8	2.6 - 16.3	0.0	0.0 - 0.0	12.7	5.2 - 32.3	0.11
6010205	17812	POWELL R	7.9	38.0	12 - 67	0.0	0.0 - 0.0	15.7	4.8 - 29.0	2.6	0.8 - 6.8	4.1	1.2 - 8.9	0.0	0.0 - 0.0	15.6	5.3 - 35.6	0.11
6010205	17813	BIG SYCAMORE CI	45.1	42.7	12 - 77	0.0	0.0 - 0.0	10.7	3.0 - 22.3	7.9	2.2 - 20.0	9.9	2.7 - 25.1	0.0	0.0 - 0.0	14.2	4.1 - 32.8	0.11
6010205	17814	BIG SYCAMORE CI	2.3	21.2	7 - 42	0.0	0.0 - 0.0	6.1	1.9 - 13.0	0.7	0.2 - 2.0	0.6	0.2 - 1.4	0.0	0.0 - 0.0	13.7	4.6 - 30.5	0.11
6010205	17816	INDIAN CR	15.9	43.5	15 - 79	0.0	0.0 - 0.0	12.6	4.5 - 26.2	7.5	2.3 - 19.5	8.1	2.4 - 21.3	0.0	0.0 - 0.0	15.3	5.4 - 30.8	0.11
6010206	17817	POWELL R	273.2	35.7	10 - 66	1.0	0.3 - 2.0	8.8	2.7 - 17.8	6.2	1.7 - 16.9	7.8	2.5 - 15.9	0.0	0.0 - 0.0	11.8	3.4 - 25.5	0.11
6010206	17818	DAVIS CR	167.2	43.7	15 - 94	0.0	0.0 - 0.0	9.1	2.6 - 22.6	7.5	1.8 - 20.4	14.9	5.1 - 38.4	0.0	0.0 - 0.0	12.1	3.8 - 27.7	0.11
6010206	17819	POWELL R	141.9	35.8	12 - 86	0.0	0.0 - 0.0	13.4	4.6 - 33.0	3.1	0.9 - 8.8	5.2	1.6 - 15.4	0.0	0.0 - 0.0	14.0	4.5 - 35.5	0.11
6010206	17820	POWELL R	92.9	25.9	9 - 72	0.0	0.0 - 0.0	8.9	2.8 - 23.6	2.0	0.6 - 7.1	2.2	0.7 - 5.6	0.0	0.0 - 0.0	12.8	4.3 - 35.6	0.11
6010207	17823	BEAVER CR	233.1	128.7	49 - 312	48.9	18.3 - 117.4	47.3	16.7 - 116.6	5.1	1.6 - 15.8	13.4	4.9 - 33.1	0.0	0.0 - 0.0	14.2	5.0 - 35.6	0.26
6010207	17824	CLINCH R	55.1	40.9	15 - 82	0.0	0.0 - 0.0	11.4	3.9 - 25.5	3.7	1.3 - 10.0	11.5	3.6 - 29.7	0.0	0.0 - 0.0	14.3	5.2 - 34.8	0.30
6010207	17825	CLINCH R	28.4	55.2	21 - 111	0.0	0.0 - 0.0	28.1	9.2 - 54.7	2.3	0.7 - 5.6	7.5	2.7 - 18.3	0.0	0.0 - 0.0	17.3	6.9 - 37.2	0.26
6010207	17826	BEAVER CR	1.7	41.9	13 - 71	0.0	0.0 - 0.0	15.6	5.1 - 29.0	3.0	0.9 - 7.6	8.7	2.3 - 17.7	0.0	0.0 - 0.0	14.5	4.2 - 28.7	0.26
6010201	17827	*B	19.5	112.0	43 - 273	0.0	0.0 - 0.0	80.1	32.2 - 191.5	4.1	1.4 - 10.5	13.5	4.6 - 38.7	0.0	0.0 - 0.0	14.4	4.9 - 41.5	0.26
6010201	17828	TENNESSEE R	124.0	64.3	26 - 108	0.0	0.0 - 0.0	27.5	10.5 - 51.9	5.4	1.9 - 12.8	17.0	6.1 - 40.4	0.0	0.0 - 0.0	14.4	5.9 - 29.5	0.26
6010201	17829	*A	44.5	57.9	20 - 138	0.0	0.0 - 0.0	6.7	2.2 - 16.0	9.8	2.8 - 27.5	25.2	8.7 - 70.1	0.0	0.0 - 0.0	16.2	5.0 - 40.2	0.26
6010201	17830	TENNESSEE R	56.3	58.6	22 - 139	0.2	0.1 - 0.5	23.7	8.9 - 56.3	6.8	2.1 - 17.2	15.5	6.1 - 36.3	0.0	0.0 - 0.0	12.3	5.0 - 33.8	0.30
6010201	17831	TENNESSEE R	100.1	177.6	51 - 376	116.3	33.2 - 244.5	9.0	2.4 - 17.7	8.8	2.0 - 21.8	26.4	7.2 - 63.6	0.0	0.0 - 0.0	17.1	4.5 - 35.2	0.26
6010201	17832	*B	44.8	109.4	37 - 232	0.0	0.0 - 0.0	81.1	26.6 - 171.3	3.2	1.0 - 8.5	10.3	3.6 - 27.1	0.0	0.0 - 0.0	14.9	5.0 - 34.4	0.26

8-digit HUC	ID	Name	Area	Catchment Yield		Point sources		Developed Land		Manure		Agricultural Land		Phosphate Mines		Soil parent rock		Frac
				kg/km^2	90% CI	kg/km^2	90% CI	kg/km^2	90% CI	kg/km^2	90% CI	kg/km^2	90% CI	kg/km^2	90% CI	kg/km^2	90% CI	
6010201	17833	TENNESSEE R	98.2	101.2	37 - 240	0.0	0.0 - 0.0	80.3	28.5 - 188.5	1.7	0.5 - 5.1	5.1	1.9 - 14.9	0.0	0.0 - 0.0	14.2	5.1 - 37.2	0.26
6010201	17834	*A	1.7	41.1	14 - 82	0.0	0.0 - 0.0	6.2	1.9 - 12.8	6.5	2.3 - 16.1	13.2	4.3 - 33.2	0.0	0.0 - 0.0	15.2	5.9 - 33.3	0.26
6010201	17835	TENNESSEE R	24.0	51.7	16 - 113	0.0	0.0 - 0.0	18.6	5.7 - 38.3	4.0	1.3 - 10.0	13.6	4.7 - 34.5	0.0	0.0 - 0.0	15.5	5.2 - 36.2	0.30
6010201	17836	WHITES CR	50.1	86.4	28 - 161	36.4	12.5 - 73.1	26.7	7.9 - 54.8	2.3	0.7 - 5.9	7.9	2.4 - 19.0	0.0	0.0 - 0.0	13.1	4.5 - 31.3	0.30
6010201	17837	PINEY R	41.2	62.9	23 - 120	0.0	0.0 - 0.0	23.3	7.5 - 45.7	4.3	1.3 - 11.2	20.4	6.6 - 47.7	0.0	0.0 - 0.0	14.9	5.4 - 35.2	0.30
6010201	17839	TENNESSEE R	214.5	49.0	17 - 132	1.9	0.6 - 5.3	17.1	5.7 - 43.9	3.1	0.8 - 9.2	9.8	3.3 - 33.3	0.0	0.0 - 0.0	17.1	6.5 - 51.8	0.30
6010201	17840	WHITES CR	52.6	50.5	15 - 102	0.0	0.0 - 0.0	13.8	4.4 - 27.6	3.3	0.9 - 9.4	14.6	4.1 - 36.7	0.0	0.0 - 0.0	18.8	5.6 - 44.5	0.30
6010201	17841	PINEY R	72.6	74.5	25 - 199	13.6	4.5 - 37.3	9.0	2.9 - 22.3	5.5	1.4 - 16.3	26.5	8.6 - 89.1	0.0	0.0 - 0.0	19.9	6.5 - 57.0	0.30
6010201	17842	TENNESSEE R	73.6	35.9	11 - 72	0.0	0.0 - 0.0	7.6	2.3 - 16.6	1.7	0.5 - 3.9	5.5	1.6 - 14.2	0.0	0.0 - 0.0	21.1	6.3 - 45.7	0.30
6020001	17843	ROARING CR	22.4	39.9	14 - 82	0.0	0.0 - 0.0	7.9	2.8 - 17.8	4.5	1.5 - 12.1	12.7	4.4 - 34.5	0.0	0.0 - 0.0	14.9	5.7 - 34.4	0.37
6020001	17844	POSSUM CR	85.4	31.7	10 - 54	0.0	0.0 - 0.0	7.5	2.9 - 14.7	3.3	1.1 - 7.7	7.2	2.2 - 15.0	0.0	0.0 - 0.0	13.7	4.1 - 27.5	0.37
6020001	17845	SODDY CR	96.1	34.9	12 - 71	0.0	0.0 - 0.0	12.7	4.0 - 23.9	2.5	0.8 - 6.5	6.0	1.9 - 15.4	0.0	0.0 - 0.0	13.7	4.8 - 30.3	0.37
6020001	17846	LONG SAVANNAH	247.9	54.5	17 - 105	0.0	0.0 - 0.0	16.3	4.7 - 33.4	6.3	1.8 - 19.2	17.5	5.7 - 40.2	0.0	0.0 - 0.0	14.4	4.3 - 40.0	0.37
6020002	17847	HIWASSEE R	156.0	61.0	25 - 116	0.0	0.0 - 0.0	6.9	2.7 - 15.2	9.6	3.1 - 25.8	24.5	9.1 - 62.0	0.0	0.0 - 0.0	20.0	8.1 - 39.9	0.37
6020001	17848	TENNESSEE R	54.3	46.4	16 - 99	0.7	0.2 - 1.5	28.2	9.5 - 60.5	0.9	0.3 - 2.2	2.5	0.8 - 6.4	0.0	0.0 - 0.0	14.1	5.2 - 32.3	0.42
6020001	17849	TENNESSEE R	99.8	54.8	21 - 109	0.0	0.0 - 0.0	20.9	6.8 - 41.9	4.0	1.2 - 9.8	10.9	3.9 - 24.8	0.0	0.0 - 0.0	19.0	7.5 - 44.1	0.37
6020001	17850	TENNESSEE R	8.6	39.7	14 - 80	0.0	0.0 - 0.0	4.5	1.4 - 10.1	4.2	1.1 - 11.9	9.8	3.9 - 23.0	0.0	0.0 - 0.0	21.2	7.9 - 43.5	0.37
6020001	17851	TENNESSEE R	72.4	44.0	17 - 111	0.0	0.0 - 0.0	4.4	1.7 - 10.1	6.0	1.9 - 17.2	17.2	6.2 - 60.7	0.0	0.0 - 0.0	16.5	6.6 - 43.3	0.37
6020001	17852	TENNESSEE R	60.8	43.5	17 - 84	0.0	0.0 - 0.0	6.5	2.3 - 14.9	5.0	1.8 - 14.4	15.8	5.4 - 48.7	0.0	0.0 - 0.0	16.2	6.0 - 36.6	0.37
6020001	17853	TENNESSEE R	24.5	167.3	49 - 359	114.8	32.0 - 254.3	16.2	4.5 - 36.3	3.7	1.0 - 8.0	15.9	4.3 - 40.8	0.0	0.0 - 0.0	16.7	4.9 - 41.5	0.37
6020001	17854	ROARING CR	4.1	30.3	11 - 62	0.0	0.0 - 0.0	5.0	1.6 - 10.6	2.9	0.8 - 8.2	8.0	2.7 - 19.4	0.0	0.0 - 0.0	14.4	5.0 - 33.0	0.37
6020001	17857	LONG SAVANNAH	21.6	37.1	11 - 94	0.0	0.0 - 0.0	15.6	4.7 - 40.8	1.4	0.4 - 3.9	3.6	1.0 - 10.6	0.0	0.0 - 0.0	16.5	6.2 - 52.5	0.37
6020003	17859	BAKER CR	13.9	16.0	6 - 37	0.0	0.0 - 0.0	2.4	0.8 - 5.0	0.1	0.0 - 0.3	0.2	0.1 - 0.6	0.0	0.0 - 0.0	13.2	4.7 - 32.1	0.30
6020003	17860	GREASY CR	59.9	17.0	7 - 35	0.0	0.0 - 0.0	3.8	1.2 - 8.3	0.4	0.1 - 1.0	0.7	0.2 - 1.5	0.0	0.0 - 0.0	12.2	4.8 - 27.1	0.30
6020003	17861	OCOEE R	245.1	19.1	7 - 39	0.0	0.0 - 0.1	4.6	1.4 - 10.9	0.4	0.1 - 1.1	0.7	0.2 - 1.8	0.0	0.0 - 0.0	13.4	5.3 - 30.4	0.30
6020003	17863	OCOEE R	57.6	19.7	7 - 45	0.0	0.0 - 0.0	4.2	1.5 - 9.0	0.0	0.0 - 0.1	0.0	0.0 - 0.1	0.0	0.0 - 0.0	15.5	5.7 - 36.8	0.30
6020003	17864	BAKER CR	4.5	28.1	11 - 60	0.0	0.0 - 0.0	8.1	2.8 - 16.5	0.4	0.1 - 1.1	0.6	0.2 - 1.5	0.0	0.0 - 0.0	19.0	7.5 - 45.2	0.30
6020002	17867	S SHOAL CR	49.9	12.3	5 - 27	0.5	0.2 - 1.3	4.2	1.4 - 9.8	0.3	0.1 - 0.7	0.8	0.3 - 2.4	0.0	0.0 - 0.0	6.5	2.4 - 14.9	0.33
6020002	17868	N SHOAL CR	22.3	11.7	4 - 26	0.0	0.0 - 0.0	3.7	1.3 - 7.9	0.2	0.0 - 0.5	0.4	0.1 - 1.1	0.0	0.0 - 0.0	7.5	2.9 - 18.9	0.33
6020002	17869	COOK CR	76.8	15.7	6 - 35	0.0	0.0 - 0.0	4.1	1.6 - 7.9	0.2	0.1 - 0.6	0.8	0.3 - 2.2	0.0	0.0 - 0.0	10.6	4.2 - 26.3	0.33
6020002	17870	HANGING DOG CR	104.0	15.5	6 - 31	0.0	0.0 - 0.0	4.7	1.7 - 9.7	0.2	0.1 - 0.6	0.8	0.3 - 2.1	0.0	0.0 - 0.0	9.7	3.5 - 22.1	0.33
6020002	17871	NOTTELY R	187.6	24.9	7 - 56	0.0	0.0 - 0.0	9.4	2.7 - 21.3	1.4	0.4 - 3.9	3.3	0.9 - 8.7	0.0	0.0 - 0.0	10.8	3.1 - 26.8	0.33
6020002	17872	HIWASSEE R	34.5	10.7	3 - 20	0.0	0.0 - 0.0	4.8	1.4 - 10.2	0.2	0.1 - 0.5	0.5	0.1 - 1.3	0.0	0.0 - 0.0	5.3	1.6 - 11.4	0.35
6020002	17873	HIWASSEE R	2.4	9.0	3 - 18	0.0	0.0 - 0.0	2.3	0.7 - 4.3	0.0	0.0 - 0.1	0.0	0.0 - 0.0	0.0	0.0 - 0.0	6.6	2.1 - 16.6	0.33
6020002	17874	HIWASSEE R	5.7	13.0	4 - 29	0.0	0.0 - 0.0	5.9	1.7 - 13.6	0.1	0.0 - 0.3	0.3	0.1 - 0.8	0.0	0.0 - 0.0	6.7	2.1 - 15.8	0.33
6020002	17876	HIWASSEE R	20.4	12.2	3 - 31	0.7	0.2 - 1.9	5.6	1.5 - 14.0	0.0	0.0 - 0.1	0.1	0.0 - 0.4	0.0	0.0 - 0.0	5.8	1.8 - 17.7	0.33
6020002	17877	HIWASSEE R	10.6	7.0	2 - 17	0.0	0.0 - 0.0	1.1	0.4 - 2.4	0.0	0.0 - 0.1	0.0	0.0 - 0.1	0.0	0.0 - 0.0	5.8	2.0 - 14.5	0.33
6020002	17879	COOK CR	2.5	7.5	3 - 14	0.0	0.0 - 0.0	0.0	0.0 - 0.0	0.2	0.1 - 0.5	1.0	0.3 - 2.3	0.0	0.0 - 0.0	6.3	2.3 - 12.3	0.33
6020002	17880	HANGING DOG CR	2.6	17.4	5 - 38	0.0	0.0 - 0.0	10.0	2.8 - 20.7	0.3	0.1 - 0.7	0.6	0.2 - 1.5	0.0	0.0 - 0.0	6.5	2.2 - 14.8	0.33

8-digit HUC	ID	Name	Area	Catchment Yield		Point sources		Developed Land		Manure		Agricultural Land		Phosphate Mines		Soil parent rock		Frac
				kg/km^2	90% CI	kg/km^2	90% CI	kg/km^2	90% CI	kg/km^2	90% CI	kg/km^2	90% CI	kg/km^2	90% CI	kg/km^2	90% CI	
6020002	17881	NOTTELY R	2.7	6.9	2 - 16	0.0	0.0 - 0.0	0.0	0.0 - 0.0	0.1	0.0 - 0.2	0.2	0.1 - 0.5	0.0	0.0 - 0.0	6.6	2.1 - 15.8	0.33
6020002	17882	HIWASSEE R	163.8	12.6	3 - 22	0.0	0.0 - 0.0	5.4	1.4 - 9.7	0.3	0.1 - 0.7	0.9	0.2 - 2.4	0.0	0.0 - 0.0	5.9	1.9 - 15.7	0.33
6020002	17883	YOUNGCANE CR	73.9	34.1	10 - 61	0.0	0.0 - 0.0	12.7	4.3 - 25.3	3.3	1.0 - 7.4	7.1	1.9 - 16.3	0.0	0.0 - 0.0	11.0	3.6 - 23.5	0.14
6020002	17884	NOTTELY R	130.3	36.6	13 - 73	0.0	0.0 - 0.0	16.5	5.5 - 32.3	2.2	0.8 - 5.9	4.5	1.3 - 11.3	0.0	0.0 - 0.0	13.3	4.3 - 31.5	0.14
6020002	17885	COOSE CR	57.6	35.8	13 - 95	0.0	0.0 - 0.0	14.2	5.2 - 32.1	2.5	0.8 - 7.4	5.3	1.7 - 17.7	0.0	0.0 - 0.0	13.7	4.5 - 35.6	0.14
6020002	17886	NOTTELY R	119.9	15.5	5 - 34	0.0	0.0 - 0.0	5.5	1.5 - 12.8	1.2	0.3 - 4.0	2.4	0.7 - 6.2	0.0	0.0 - 0.0	6.3	2.1 - 16.0	0.32
6020002	17887	YOUNGCANE CR	9.5	29.2	9 - 62	0.0	0.0 - 0.0	13.0	4.1 - 28.2	2.4	0.7 - 6.6	3.3	1.2 - 8.0	0.0	0.0 - 0.0	10.4	3.4 - 23.4	0.14
6020002	17888	NOTTELY R	1.6	63.5	21 - 151	0.0	0.0 - 0.0	34.7	11.0 - 86.8	4.0	1.1 - 11.1	6.7	2.1 - 18.8	0.0	0.0 - 0.0	18.1	5.7 - 45.0	0.14
6020002	17890	NOTTELY R	42.9	46.6	15 - 131	0.0	0.0 - 0.0	19.0	6.1 - 52.8	2.6	0.8 - 7.2	5.0	1.6 - 15.6	0.0	0.0 - 0.0	19.9	6.5 - 58.9	0.14
6020003	17891	OCOEE R	43.0	22.6	7 - 51	0.0	0.0 - 0.0	6.9	1.8 - 18.9	0.3	0.1 - 0.9	0.4	0.1 - 1.0	0.0	0.0 - 0.0	15.0	5.0 - 40.0	0.15
6020003	17892	OCOEE R	106.1	14.9	5 - 37	0.0	0.0 - 0.0	4.0	1.2 - 10.0	1.2	0.3 - 4.3	1.6	0.5 - 4.8	0.0	0.0 - 0.0	8.1	2.5 - 22.8	0.28
6020002	17893	SHOOTING CR	109.4	34.5	12 - 62	0.0	0.0 - 0.0	8.1	2.5 - 14.9	1.1	0.4 - 2.9	4.0	1.2 - 9.1	0.0	0.0 - 0.0	21.4	7.4 - 41.5	0.10
6020002	17895	HIWASSEE R	3.6	15.4	5 - 38	0.0	0.0 - 0.0	2.3	0.8 - 6.5	1.2	0.3 - 3.8	4.6	1.4 - 13.1	0.0	0.0 - 0.0	7.3	2.3 - 18.9	0.31
6020002	17896	SHOOTING CR	22.2	37.4	12 - 70	0.0	0.0 - 0.0	12.1	3.6 - 23.8	2.1	0.6 - 6.0	6.9	2.2 - 16.9	0.0	0.0 - 0.0	16.2	5.4 - 38.0	0.10
6020002	17897	HIWASSEE R	151.1	47.4	18 - 94	5.4	2.0 - 11.2	14.1	4.5 - 25.6	2.1	0.6 - 5.0	5.4	1.9 - 13.2	0.0	0.0 - 0.0	20.4	7.8 - 44.9	0.10
6010202	17898	NANTAHALA R	152.1	35.6	13 - 80	0.0	0.0 - 0.0	4.4	1.5 - 10.5	0.1	0.0 - 0.2	0.2	0.1 - 0.8	0.0	0.0 - 0.0	30.9	10.9 - 68.7	0.08
6010202	17899	JARRETT CR	15.5	34.8	11 - 81	0.0	0.0 - 0.0	8.3	2.8 - 18.9	0.0	0.0 - 0.1	0.1	0.0 - 0.2	0.0	0.0 - 0.0	26.3	7.5 - 69.4	0.08
6010202	17900	NANTAHALA R	48.9	11.9	3 - 26	0.0	0.0 - 0.0	2.7	0.7 - 5.6	0.1	0.0 - 0.1	0.2	0.1 - 0.6	0.0	0.0 - 0.0	8.9	2.7 - 20.5	0.13
6010202	17901	NANTAHALA R	14.3	22.6	7 - 63	0.0	0.0 - 0.0	5.1	1.6 - 13.6	0.0	0.0 - 0.1	0.0	0.0 - 0.0	0.0	0.0 - 0.0	17.5	5.5 - 53.4	0.08
6010202	17902	JARRETT CR	4.1	25.8	8 - 60	0.0	0.0 - 0.0	8.9	2.9 - 19.8	0.0	0.0 - 0.1	0.1	0.0 - 0.4	0.0	0.0 - 0.0	16.7	5.7 - 44.1	0.08
6010202	17903	LITTLE TENNESSE	1.4	17.4	6 - 38	0.0	0.0 - 0.0	10.9	3.7 - 24.6	0.1	0.0 - 0.4	0.6	0.2 - 1.7	0.0	0.0 - 0.0	5.7	2.1 - 13.1	0.14
6010202	17904	NANTAHALA R	106.2	16.2	5 - 34	0.0	0.0 - 0.0	6.5	2.1 - 13.5	0.1	0.0 - 0.1	0.3	0.1 - 0.8	0.0	0.0 - 0.0	9.3	3.3 - 21.2	0.14
6010202	17905	LITTLE TENNESSE	37.9	14.4	4 - 39	0.0	0.0 - 0.0	7.9	2.6 - 21.3	0.1	0.0 - 0.3	0.6	0.2 - 1.7	0.0	0.0 - 0.0	5.8	1.7 - 17.9	0.14
6010202	17906	ALARKA CR	81.3	13.1	4 - 23	0.0	0.0 - 0.0	4.7	1.4 - 9.1	0.1	0.0 - 0.3	0.7	0.2 - 1.7	0.0	0.0 - 0.0	7.6	2.4 - 15.4	0.14
6010203	17907	TUCKASEGEE R	52.1	30.7	12 - 80	4.9	1.8 - 15.1	15.9	5.5 - 41.8	0.2	0.1 - 0.6	1.2	0.5 - 4.1	0.0	0.0 - 0.0	8.5	3.5 - 23.1	0.14
6010203	17908	NOLAND CR	54.1	10.5	3 - 29	0.0	0.0 - 0.0	0.6	0.2 - 1.5	0.0	0.0 - 0.0	0.0	0.0 - 0.0	0.0	0.0 - 0.0	9.9	3.1 - 28.3	0.14
6010203	17909	FORNEY CR	73.0	10.2	4 - 22	0.0	0.0 - 0.0	0.0	0.0 - 0.0	0.0	0.0 - 0.0	0.0	0.0 - 0.0	0.0	0.0 - 0.0	10.1	3.8 - 21.9	0.14
6010202	17910	LITTLE TENNESSE	18.9	12.2	5 - 29	0.0	0.0 - 0.0	3.4	1.2 - 8.1	0.1	0.0 - 0.2	0.1	0.0 - 0.3	0.0	0.0 - 0.0	8.7	3.5 - 21.0	0.14
6010202	17911	TUCKASEGEE R	4.0	9.0	3 - 20	0.0	0.0 - 0.0	0.0	0.0 - 0.0	0.0	0.0 - 0.0	0.0	0.0 - 0.0	0.0	0.0 - 0.0	9.0	2.8 - 20.2	0.14
6010203	17912	TUCKASEGEE R	3.8	8.4	3 - 20	0.0	0.0 - 0.0	0.0	0.0 - 0.0	0.0	0.0 - 0.0	0.0	0.0 - 0.0	0.0	0.0 - 0.0	8.4	2.7 - 20.0	0.14
6010203	17913	TUCKASEGEE R	9.4	7.3	2 - 16	0.0	0.0 - 0.0	1.5	0.4 - 3.4	0.0	0.0 - 0.0	0.0	0.0 - 0.1	0.0	0.0 - 0.0	5.8	2.0 - 13.4	0.14
6010202	17914	LITTLE TENNESSE	1.9	8.5	2 - 19	0.0	0.0 - 0.0	0.6	0.2 - 1.4	0.1	0.0 - 0.4	0.1	0.0 - 0.4	0.0	0.0 - 0.0	7.6	1.9 - 18.3	0.14
6010202	17915	LITTLE TENNESSE	4.0	12.3	4 - 24	0.0	0.0 - 0.0	6.0	1.9 - 11.7	0.1	0.0 - 0.2	0.4	0.1 - 1.1	0.0	0.0 - 0.0	5.8	1.8 - 13.7	0.14
6010202	17916	NANTAHALA R	17.6	18.4	6 - 47	0.4	0.1 - 1.1	11.4	3.7 - 27.3	0.1	0.0 - 0.2	0.5	0.2 - 1.4	0.0	0.0 - 0.0	6.0	1.8 - 15.4	0.14
6010202	17917	LITTLE TENNESSE	5.9	13.2	5 - 27	0.0	0.0 - 0.0	7.2	2.5 - 15.4	0.1	0.0 - 0.3	0.4	0.1 - 1.0	0.0	0.0 - 0.0	5.5	2.1 - 13.4	0.14
6010202	17918	ALARKA CR	9.0	15.0	5 - 28	0.0	0.0 - 0.0	6.9	2.3 - 14.4	0.1	0.0 - 0.2	0.4	0.1 - 1.1	0.0	0.0 - 0.0	7.7	2.8 - 17.0	0.14
6010203	17919	TUCKASEGEE R	5.3	11.1	3 - 22	0.0	0.0 - 0.0	5.5	1.6 - 11.9	0.0	0.0 - 0.1	0.1	0.0 - 0.4	0.0	0.0 - 0.0	5.5	1.6 - 12.6	0.14
6010203	17921	FORNEY CR	5.6	6.1	2 - 13	0.0	0.0 - 0.0	0.0	0.0 - 0.0	0.0	0.0 - 0.0	0.0	0.0 - 0.0	0.0	0.0 - 0.0	6.1	2.0 - 13.5	0.14
6010204	17922	TULULAH CR	1.2	469.9	171 - 1243	419.2	153.5 - 1104.5	25.1	10.1 - 62.1	3.3	1.1 - 9.0	9.7	3.5 - 25.5	0.0	0.0 - 0.0	12.6	5.0 - 33.2	0.11

8-digit HUC	ID	Name	Area	Catchment Yield		Point sources		Developed Land		Manure		Agricultural Land		Phosphate Mines		Soil parent rock		Frac
				kg/km^2	90% CI	kg/km^2	90% CI	kg/km^2	90% CI	kg/km^2	90% CI	kg/km^2	90% CI	kg/km^2	90% CI	kg/km^2	90% CI	
6010204	17923	SNOWBIRD CR	111.1	17.4	7 - 34	0.0	0.0 - 0.0	3.4	1.3 - 6.8	0.1	0.0 - 0.2	0.3	0.1 - 0.8	0.0	0.0 - 0.0	13.6	4.9 - 28.3	0.11
6010204	17924	TULULAH CR	26.1	18.0	6 - 40	0.0	0.0 - 0.0	7.8	2.7 - 18.2	0.3	0.1 - 1.1	1.1	0.3 - 2.6	0.0	0.0 - 0.0	8.8	2.9 - 20.0	0.11
6010204	17925	SNOWBIRD CR	8.8	15.9	5 - 30	0.0	0.0 - 0.0	5.1	1.6 - 9.4	0.2	0.1 - 0.5	0.6	0.2 - 1.6	0.0	0.0 - 0.0	10.0	3.4 - 22.3	0.11
6010204	17926	TULULAH CR	157.5	9.8	3 - 23	0.0	0.0 - 0.0	2.4	0.8 - 5.5	0.1	0.0 - 0.2	0.2	0.1 - 0.7	0.0	0.0 - 0.0	7.0	2.4 - 18.5	0.20
6010202	17927	EAGLE CR	67.8	9.7	3 - 27	0.0	0.0 - 0.0	0.0	0.0 - 0.1	0.0	0.0 - 0.0	0.0	0.0 - 0.0	0.0	0.0 - 0.0	9.6	2.5 - 27.0	0.14
6010202	17928	HAZEL CR	111.2	10.0	3 - 24	0.0	0.0 - 0.0	0.0	0.0 - 0.0	0.0	0.0 - 0.0	0.0	0.0 - 0.0	0.0	0.0 - 0.0	10.0	2.8 - 24.5	0.14
6010202	17929	LITTLE TENNESSE	4.5	11.8	4 - 27	0.0	0.0 - 0.0	2.7	0.9 - 6.6	0.1	0.0 - 0.2	0.6	0.2 - 1.4	0.0	0.0 - 0.0	8.5	3.0 - 19.3	0.20
6010202	17930	LITTLE TENNESSE	11.2	12.8	4 - 26	0.0	0.0 - 0.0	2.1	0.7 - 4.4	0.1	0.0 - 0.2	0.2	0.1 - 0.5	0.0	0.0 - 0.0	10.4	3.3 - 23.4	0.14
6010202	17931	LITTLE TENNESSE	205.4	11.6	5 - 23	0.0	0.0 - 0.0	2.8	1.0 - 5.7	0.2	0.1 - 0.7	0.7	0.2 - 2.0	0.0	0.0 - 0.0	7.9	3.2 - 18.6	0.14
6010202	17932	EAGLE CR	10.4	9.1	3 - 21	0.0	0.0 - 0.0	0.0	0.0 - 0.0	0.0	0.0 - 0.1	0.1	0.0 - 0.3	0.0	0.0 - 0.0	9.0	3.4 - 20.8	0.14
6010202	17933	HAZEL CR	21.3	6.7	2 - 20	0.0	0.0 - 0.0	0.0	0.0 - 0.0	0.0	0.0 - 0.0	0.1	0.0 - 0.4	0.0	0.0 - 0.0	6.6	2.2 - 19.8	0.14
6030003	17934	BRADLEY CR	123.4	118.9	35 - 204	0.0	0.0 - 0.0	8.8	2.8 - 16.3	18.5	5.2 - 44.0	65.8	18.8 - 131.0	0.0	0.0 - 0.0	25.8	8.1 - 52.4	0.09
6030003	17935	ELK R	19.7	158.7	53 - 389	0.0	0.0 - 0.0	7.1	2.4 - 16.6	37.0	11.6 - 121.8	84.1	28.2 - 229.8	0.0	0.0 - 0.0	30.5	9.7 - 84.6	0.09
6030003	17937	ELK R	1.1	157.5	49 - 411	0.0	0.0 - 0.0	1.3	0.4 - 3.1	34.9	9.2 - 129.2	77.5	25.7 - 218.6	0.0	0.0 - 0.0	43.9	15.6 - 133.9	0.09
6030003	17938	ELK R	100.7	99.4	34 - 190	42.2	13.9 - 88.2	4.2	1.2 - 8.3	10.5	2.7 - 31.8	24.0	7.3 - 56.3	0.0	0.0 - 0.0	18.3	5.5 - 41.7	0.20
6030003	17939	ROCK CR	98.8	280.5	108 - 534	55.7	22.1 - 108.1	36.7	14.6 - 74.8	37.5	13.8 - 96.2	87.1	31.3 - 200.8	0.0	0.0 - 0.0	63.5	21.7 - 154.5	0.21
6030003	17940	ELK R	38.4	163.7	46 - 345	0.0	0.0 - 0.0	14.1	3.9 - 30.2	29.1	7.4 - 75.7	64.2	20.2 - 155.5	0.0	0.0 - 0.0	56.3	14.8 - 133.4	0.21
6030003	17941	WAGNER CR	39.7	118.0	38 - 262	8.7	2.7 - 20.0	12.4	4.4 - 27.4	24.4	7.6 - 77.5	52.8	18.0 - 129.5	0.0	0.0 - 0.0	19.6	6.1 - 52.1	0.21
6030003	17942	BOILING FK CR	137.2	170.8	57 - 337	110.3	38.9 - 227.1	8.3	2.7 - 14.6	12.4	3.5 - 31.5	26.9	8.0 - 60.5	0.0	0.0 - 0.0	12.9	4.2 - 27.1	0.21
6030003	17943	DRY CR	36.9	113.7	38 - 247	0.0	0.0 - 0.0	7.9	2.5 - 16.1	26.5	7.8 - 68.8	58.2	18.0 - 152.1	0.0	0.0 - 0.0	21.1	6.9 - 46.9	0.21
6030003	17944	HURRICANE CR	68.4	196.4	70 - 411	0.0	0.0 - 0.0	8.4	2.7 - 17.9	20.7	6.3 - 51.0	38.5	13.4 - 96.9	0.0	0.0 - 0.0	128.7	45.4 - 309.4	0.21
6030003	17945	ELK R	54.7	105.6	43 - 210	0.0	0.0 - 0.0	2.6	1.0 - 6.0	4.6	1.7 - 12.7	9.5	3.6 - 23.3	0.0	0.0 - 0.0	88.9	36.3 - 195.4	0.48
6030003	17946	BOILING FK CR	8.5	237.8	72 - 549	113.3	35.3 - 284.2	12.7	3.6 - 31.2	24.1	5.9 - 77.3	52.4	15.8 - 143.9	0.0	0.0 - 0.0	35.1	11.0 - 86.7	0.21
6030003	17947	BOILING FK CR	2.6	107.4	45 - 274	0.0	0.0 - 0.0	37.2	14.0 - 84.5	13.4	4.6 - 43.5	32.2	12.5 - 113.2	0.0	0.0 - 0.0	24.6	8.9 - 64.2	0.21
6030003	17948	ROCK CR	5.6	188.8	69 - 422	0.0	0.0 - 0.0	28.9	9.4 - 63.7	28.3	8.7 - 90.6	62.7	23.0 - 157.0	0.0	0.0 - 0.0	68.9	26.3 - 187.3	0.21
6030003	17949	ELK R	35.0	196.4	62 - 466	0.0	0.0 - 0.0	17.8	6.0 - 42.6	40.5	10.7 - 139.2	91.5	28.6 - 223.5	0.0	0.0 - 0.0	46.6	14.1 - 127.5	0.21
6030003	17950	ELK R	47.7	132.8	45 - 272	0.0	0.0 - 0.0	18.5	5.5 - 44.7	25.6	8.2 - 71.3	56.7	15.9 - 144.7	0.0	0.0 - 0.0	32.0	10.9 - 74.4	0.21
6030003	17951	WAGNER CR	9.3	131.8	52 - 301	0.0	0.0 - 0.0	51.8	19.2 - 120.8	18.9	7.1 - 58.5	41.6	14.8 - 118.7	0.0	0.0 - 0.0	19.5	7.8 - 50.4	0.21
6030003	17952	BOILING FK CR	11.3	127.3	34 - 252	0.0	0.0 - 0.0	57.7	14.3 - 123.1	16.3	4.6 - 48.9	35.4	9.6 - 87.5	0.0	0.0 - 0.0	17.9	5.2 - 42.9	0.21
6030003	17953	DRY CR	21.3	123.5	40 - 282	0.0	0.0 - 0.0	24.1	8.4 - 57.9	24.6	7.5 - 74.0	55.1	17.0 - 141.3	0.0	0.0 - 0.0	19.8	6.5 - 52.6	0.21
6030003	17954	HURRICANE CR	14.2	313.5	105 - 927	0.0	0.0 - 0.0	4.1	1.4 - 9.9	11.0	3.6 - 31.6	24.4	9.5 - 77.1	0.0	0.0 - 0.0	274.0	90.2 - 811.3	0.21
6030003	17955	ELK R	78.6	185.0	62 - 362	0.0	0.0 - 0.0	8.2	2.8 - 18.0	24.0	8.0 - 62.3	53.6	18.3 - 137.7	0.0	0.0 - 0.0	99.2	28.7 - 241.8	0.21
6030001	17956	TENNESSEE R	37.7	63.1	21 - 130	3.1	0.9 - 7.1	14.2	4.8 - 30.1	6.9	2.2 - 21.1	19.0	6.1 - 44.9	0.0	0.0 - 0.0	20.0	6.6 - 43.9	0.45
6030001	17957	JONES CR	188.8	43.9	12 - 91	0.0	0.0 - 0.0	7.9	2.4 - 16.9	10.5	3.0 - 26.7	20.0	5.1 - 59.7	0.0	0.0 - 0.0	5.4	1.6 - 12.4	0.45
6030001	17958	N SAUTY CR	129.9	59.1	19 - 120	0.0	0.0 - 0.0	4.0	1.2 - 9.3	10.4	2.7 - 27.4	30.3	8.9 - 76.8	0.0	0.0 - 0.0	14.4	4.8 - 37.8	0.45
6030001	17959	S SAUTY CR	60.0	41.2	12 - 83	0.0	0.0 - 0.0	5.5	1.9 - 10.7	13.5	3.7 - 32.3	17.2	5.5 - 43.1	0.0	0.0 - 0.0	5.0	1.5 - 11.7	0.45
6030001	17960	TOWN CR	23.8	29.5	10 - 54	0.0	0.0 - 0.0	3.7	1.1 - 7.7	9.5	2.6 - 23.5	10.3	3.0 - 22.1	0.0	0.0 - 0.0	5.9	2.2 - 12.5	0.45
6030001	17961	SHORT CR	9.6	42.5	14 - 82	0.0	0.0 - 0.0	5.6	1.7 - 11.6	15.7	4.6 - 39.2	16.2	5.0 - 38.8	0.0	0.0 - 0.0	5.0	1.7 - 10.1	0.45
6030001	17962	BIG SPRING CR	132.9	55.7	18 - 112	0.0	0.0 - 0.0	9.2	3.0 - 20.0	18.2	5.3 - 40.9	20.0	5.9 - 50.6	0.0	0.0 - 0.0	8.3	3.0 - 19.3	0.45

8-digit HUC	ID	Name	Area	Catchment Yield		Point sources		Developed Land		Manure		Agricultural Land		Phosphate Mines		Soil parent rock		Frac
				kg/km2	90% CI	kg/km2	90% CI	kg/km2	90% CI	kg/km2	90% CI	kg/km2	90% CI	kg/km2	90% CI	kg/km2	90% CI	
6030001	17963	BROWN CR	123.8	71.9	24 - 191	0.0	0.0 - 0.0	5.7	1.8 - 12.6	24.7	6.9 - 68.6	27.3	9.0 - 77.4	0.0	0.0 - 0.0	14.3	4.5 - 34.2	0.45
6030002	17964	TENNESSEE R	161.5	49.8	15 - 106	0.0	0.0 - 0.0	8.2	2.9 - 19.5	17.5	5.9 - 50.4	18.2	5.3 - 54.8	0.0	0.0 - 0.0	5.9	2.2 - 13.2	0.55
6030001	17965	TENNESSEE R	6.8	44.9	16 - 123	0.0	0.0 - 0.0	12.5	4.2 - 32.1	0.0	0.0 - 0.0	0.0	0.0 - 0.0	0.0	0.0 - 0.0	32.4	11.0 - 92.2	0.45
6030001	17966	TENNESSEE R	14.4	54.7	17 - 138	0.0	0.0 - 0.0	15.1	4.8 - 38.4	8.1	2.1 - 24.1	8.1	2.5 - 25.2	0.0	0.0 - 0.0	23.4	8.2 - 59.8	0.45
6030001	17967	TENNESSEE R	10.4	24.6	8 - 53	0.0	0.0 - 0.0	0.6	0.2 - 1.2	0.8	0.3 - 2.1	2.5	0.9 - 6.0	0.0	0.0 - 0.0	20.6	6.6 - 45.0	0.45
6030001	17968	JONES CR	15.6	26.9	10 - 47	0.0	0.0 - 0.0	4.3	1.4 - 8.1	3.8	1.3 - 9.4	11.1	4.0 - 27.4	0.0	0.0 - 0.0	7.6	3.0 - 15.1	0.45
6030001	17969	N SAUTY CR	64.9	50.6	19 - 101	0.0	0.0 - 0.0	13.3	4.7 - 28.0	6.6	1.9 - 21.4	16.9	6.1 - 39.6	0.0	0.0 - 0.0	13.8	5.0 - 35.0	0.45
6030001	17970	S SAUTY CR	103.5	40.8	15 - 70	0.0	0.0 - 0.0	4.6	1.6 - 8.4	9.8	3.2 - 24.3	17.7	6.4 - 45.3	0.0	0.0 - 0.0	8.7	3.2 - 15.5	0.45
6030001	17971	TOWN CR	38.7	44.8	14 - 93	0.0	0.0 - 0.0	6.6	2.0 - 15.2	11.8	3.1 - 36.8	12.4	3.9 - 31.7	0.0	0.0 - 0.0	14.0	4.1 - 34.6	0.45
6030001	17972	TENNESSEE R	8.5	44.1	14 - 102	11.5	4.0 - 25.0	4.5	1.2 - 10.4	3.3	0.9 - 9.2	3.9	1.3 - 9.6	0.0	0.0 - 0.0	20.8	6.5 - 51.9	0.45
6030001	17973	SHORT CR	39.7	45.9	15 - 80	0.0	0.0 - 0.0	8.8	3.1 - 17.2	15.3	4.3 - 39.2	16.0	4.8 - 36.9	0.0	0.0 - 0.0	5.8	1.7 - 12.4	0.45
6030001	17974	BIG SPRING CR	48.4	135.8	47 - 328	84.9	29.3 - 221.2	28.0	8.6 - 58.1	7.2	2.1 - 19.4	7.3	2.5 - 19.1	0.0	0.0 - 0.0	8.4	3.1 - 21.5	0.45
6030001	17975	BROWN CR	38.1	64.6	25 - 148	0.0	0.0 - 0.0	10.3	3.9 - 25.0	16.1	5.7 - 47.4	16.6	5.9 - 48.6	0.0	0.0 - 0.0	21.7	8.1 - 59.5	0.45
6030001	17976	TENNESSEE R	131.8	28.0	9 - 71	0.0	0.0 - 0.0	4.6	1.3 - 11.4	8.1	2.4 - 24.9	8.4	2.5 - 26.1	0.0	0.0 - 0.0	7.0	2.5 - 19.0	0.55
6030002	17977	TENNESSEE R, MII	146.9	189.9	72 - 399	88.6	34.0 - 192.4	37.0	14.0 - 94.7	5.4	1.7 - 14.7	38.0	14.4 - 100.0	0.0	0.0 - 0.0	21.0	7.8 - 48.1	0.55
6030002	17979	PINEY CR	242.4	152.3	64 - 285	0.0	0.0 - 0.0	12.0	4.5 - 22.1	16.9	5.7 - 52.1	86.0	32.9 - 201.8	0.0	0.0 - 0.0	37.4	16.0 - 85.7	0.55
6030002	17980	LIMESTONE CR	217.9	138.0	42 - 305	0.4	0.1 - 1.0	19.2	5.4 - 47.7	12.8	3.4 - 37.0	74.4	23.3 - 215.7	0.0	0.0 - 0.0	31.3	9.8 - 77.5	0.55
6030002	17981	LIMESTONE CR, M	80.9	98.0	43 - 189	0.0	0.0 - 0.0	10.5	4.4 - 21.0	11.2	4.0 - 28.3	56.4	23.1 - 140.3	0.0	0.0 - 0.0	19.8	8.9 - 45.2	0.55
6030002	17982	FLINT CR	35.6	53.3	22 - 100	0.0	0.0 - 0.0	11.8	4.5 - 23.1	9.4	3.1 - 27.9	20.9	7.4 - 49.1	0.0	0.0 - 0.0	11.2	4.1 - 25.4	0.55
6030002	17983	SWAN CR	141.4	142.6	53 - 360	0.0	0.0 - 0.0	28.7	10.8 - 71.5	12.7	3.9 - 36.6	66.2	21.1 - 162.7	0.4	0.1 - 1.3	34.6	11.7 - 92.8	0.55
6030002	17984	MUD CR	30.7	134.2	52 - 248	0.0	0.0 - 0.0	8.1	2.9 - 15.5	15.5	5.4 - 37.4	80.2	30.6 - 174.4	0.0	0.0 - 0.0	30.4	10.3 - 67.8	0.55
6030002	17985	ROUND ISLAND CI	129.3	178.8	67 - 331	18.6	7.9 - 37.3	8.9	3.2 - 18.4	18.3	6.0 - 48.3	95.9	31.2 - 216.9	0.0	0.0 - 0.0	37.0	13.5 - 82.3	0.55
6030002	17986	MALLARD CR	99.5	126.7	47 - 238	0.0	0.0 - 0.0	6.8	2.3 - 12.3	27.2	8.2 - 60.1	65.2	20.9 - 142.9	0.0	0.0 - 0.0	27.6	10.0 - 61.4	0.55
6030004	17987	BIG CR	40.9	169.5	60 - 315	0.0	0.0 - 0.0	9.5	3.3 - 20.4	17.7	5.0 - 43.6	92.0	32.1 - 218.2	0.0	0.0 - 0.0	50.2	18.6 - 98.9	0.55
6030004	17988	SUGAR CR	156.7	164.6	51 - 465	0.0	0.0 - 0.0	6.2	2.0 - 15.4	7.5	2.2 - 24.0	30.0	9.5 - 90.7	0.0	0.0 - 0.0	121.0	43.3 - 343.1	0.55
6030004	17989	ELK R	92.5	260.4	100 - 549	0.0	0.0 - 0.0	5.9	1.9 - 13.0	9.8	3.1 - 27.6	50.7	15.3 - 118.6	0.0	0.0 - 0.0	193.9	72.2 - 463.6	0.55
6030004	17990	ANDERSON CR	133.2	156.5	51 - 382	0.0	0.0 - 0.0	8.6	2.9 - 24.3	13.9	4.6 - 42.6	67.7	22.3 - 185.1	0.0	0.0 - 0.0	66.3	21.2 - 171.0	0.55
6030002	17991	FIRST CR	79.2	141.3	52 - 307	0.0	0.0 - 0.0	11.8	3.7 - 26.5	12.7	4.1 - 36.0	65.4	22.6 - 165.6	0.0	0.0 - 0.0	51.4	20.2 - 123.6	0.55
6030002	17992	SECOND CR	152.1	135.1	50 - 279	0.0	0.0 - 0.0	7.5	2.5 - 17.3	13.7	4.1 - 33.9	56.3	19.6 - 138.3	0.0	0.0 - 0.0	57.5	22.4 - 122.8	0.55
6030005	17993	BIG NANCE CR	137.3	508.7	173 - 1097	386.1	138.6 - 843.8	11.4	3.6 - 25.2	26.6	7.5 - 68.2	59.7	20.2 - 148.2	0.0	0.0 - 0.0	24.9	8.6 - 57.6	0.55
6030005	17994	TOWN CR	238.7	89.8	31 - 232	0.0	0.0 - 0.0	5.7	1.8 - 14.8	17.1	5.4 - 54.5	50.4	16.0 - 157.9	0.0	0.0 - 0.0	16.6	5.6 - 45.5	0.55
6030005	17995	BLUEWATER CR	36.5	133.4	46 - 329	0.0	0.0 - 0.0	13.7	4.9 - 34.0	10.5	3.5 - 35.9	53.3	18.0 - 147.4	0.0	0.0 - 0.0	55.9	18.3 - 157.1	0.55
6030005	17996	SHOAL CR	151.3	114.6	36 - 289	0.0	0.0 - 0.0	15.9	4.4 - 46.8	7.1	1.8 - 23.5	36.0	11.0 - 106.9	0.0	0.0 - 0.0	55.7	17.3 - 178.9	0.55
6030002	17997	LIMESTONE CR	12.0	87.5	29 - 178	0.0	0.0 - 0.0	7.8	2.7 - 15.4	8.8	2.7 - 22.4	42.7	14.0 - 130.8	0.0	0.0 - 0.0	28.1	9.1 - 74.2	0.55
6030004	17999	ELK R	66.3	81.9	29 - 176	0.0	0.0 - 0.0	6.4	2.4 - 14.0	6.1	2.0 - 17.8	32.0	10.8 - 78.9	0.0	0.0 - 0.0	37.4	12.5 - 91.2	0.55
6030004	18000	ELK R	18.4	214.4	68 - 534	0.0	0.0 - 0.0	7.5	2.2 - 18.7	7.9	2.5 - 23.0	40.7	11.5 - 116.4	0.0	0.0 - 0.0	158.3	50.7 - 405.1	0.55
6030005	18001	TENNESSEE R	102.7	115.2	40 - 210	0.0	0.0 - 0.0	11.0	4.0 - 21.0	8.6	2.9 - 19.6	41.3	13.8 - 93.1	0.0	0.0 - 0.0	54.3	18.2 - 122.0	0.55
6030005	18002	TENNESSEE R	4.0	65.8	25 - 146	0.0	0.0 - 0.0	1.2	0.4 - 2.3	2.6	0.8 - 7.9	10.4	3.4 - 26.2	0.0	0.0 - 0.0	51.6	19.4 - 124.2	0.55
6030005	18003	TENNESSEE R	3.3	76.6	28 - 171	0.0	0.0 - 0.0	0.5	0.2 - 1.0	4.4	1.5 - 12.2	11.3	4.1 - 28.6	0.0	0.0 - 0.0	60.5	21.3 - 159.1	0.55

8-digit HUC	ID	Name	Area	Catchment Yield		Point sources		Developed Land		Manure		Agricultural Land		Phosphate Mines		Soil parent rock		Frac
				kg/km^2	90% CI	kg/km^2	90% CI	kg/km^2	90% CI	kg/km^2	90% CI	kg/km^2	90% CI	kg/km^2	90% CI	kg/km^2	90% CI	
6030004	18005	ELK R	29.9	81.1	28 - 150	0.0	0.0 - 0.0	9.5	3.1 - 18.9	7.4	2.3 - 21.1	37.7	12.3 - 84.3	0.0	0.0 - 0.0	26.5	8.6 - 59.8	0.55
6030002	18007	TENNESSEE R	20.2	65.9	26 - 166	0.0	0.0 - 0.0	4.8	1.7 - 12.2	14.1	4.4 - 40.5	31.7	11.8 - 87.7	0.0	0.0 - 0.0	15.2	5.8 - 38.6	0.55
6030002	18008	PINEY CR	2.6	62.7	29 - 158	0.0	0.0 - 0.0	4.7	2.0 - 10.2	8.1	2.8 - 26.9	21.8	9.2 - 68.6	0.0	0.0 - 0.0	28.2	12.2 - 75.9	0.55
6030002	18009	LIMESTONE CR	1.2	81.9	27 - 227	0.0	0.0 - 0.0	0.0	0.0 - 0.0	8.8	2.2 - 28.9	34.8	9.9 - 103.2	0.0	0.0 - 0.0	38.2	13.3 - 121.6	0.55
6030002	18010	LIMESTONE CR, M	10.9	96.6	38 - 202	0.0	0.0 - 0.0	2.6	1.0 - 6.1	11.3	3.9 - 32.2	56.7	21.9 - 137.8	0.0	0.0 - 0.0	25.9	9.5 - 66.8	0.55
6030002	18011	TENNESSEE R	4.8	82.0	24 - 193	0.0	0.0 - 0.0	10.9	3.2 - 24.7	10.5	3.1 - 35.4	25.0	7.7 - 64.7	0.0	0.0 - 0.0	35.7	10.9 - 89.4	0.55
6030002	18012	FLINT CR	79.7	120.8	40 - 264	0.0	0.0 - 0.0	52.1	17.2 - 103.5	13.2	4.5 - 38.3	29.5	9.3 - 74.9	0.0	0.0 - 0.0	26.0	9.9 - 60.8	0.55
6030002	18013	TENNESSEE R	8.6	994.0	356 - 2008	915.9	333.4 - 1861.5	41.0	12.9 - 85.4	0.0	0.0 - 0.0	0.0	0.0 - 0.0	0.0	0.0 - 0.0	37.1	12.1 - 80.3	0.55
6030002	18014	TENNESSEE R	7.2	98.4	32 - 230	0.0	0.0 - 0.0	32.2	10.8 - 79.9	0.0	0.0 - 0.0	0.0	0.0 - 0.0	28.9	8.5 - 88.7	37.3	12.7 - 99.6	0.55
6030002	18015	SWAN CR	30.8	100.4	31 - 199	0.0	0.0 - 0.0	5.8	2.0 - 12.4	10.9	2.9 - 26.2	56.8	17.3 - 113.8	0.0	0.0 - 0.0	26.9	7.3 - 63.2	0.55
6030002	18016	MUD CR	5.6	54.2	17 - 135	0.0	0.0 - 0.0	2.0	0.7 - 4.5	3.1	0.8 - 10.1	11.8	3.4 - 41.1	0.0	0.0 - 0.0	37.3	12.6 - 108.9	0.55
6030002	18017	ROUND ISLAND CI	6.8	85.3	26 - 182	0.0	0.0 - 0.0	1.4	0.4 - 2.9	8.0	2.0 - 20.9	39.4	12.1 - 98.5	0.0	0.0 - 0.0	36.5	9.7 - 85.6	0.55
6030002	18018	TENNESSEE R	122.5	130.8	48 - 298	0.0	0.0 - 0.0	15.7	5.4 - 36.8	24.1	7.1 - 65.4	60.6	22.3 - 154.7	0.0	0.0 - 0.0	30.4	10.8 - 81.0	0.55
6030002	18019	MALLARD CR	9.2	98.3	28 - 172	0.0	0.0 - 0.0	9.0	2.6 - 17.4	16.8	4.7 - 41.6	40.8	12.9 - 81.3	0.0	0.0 - 0.0	31.6	8.6 - 67.3	0.55
6030004	18020	SUGAR CR	5.0	210.8	79 - 482	0.0	0.0 - 0.0	4.6	1.6 - 11.2	3.2	1.0 - 9.3	17.8	6.2 - 50.2	0.0	0.0 - 0.0	185.2	67.4 - 407.3	0.55
6030004	18021	ELK R	6.8	269.0	87 - 520	0.0	0.0 - 0.0	3.2	1.1 - 5.6	3.1	0.9 - 7.4	16.1	5.2 - 39.8	0.0	0.0 - 0.0	246.6	81.0 - 506.4	0.55
6030004	18022	ANDERSON CR	16.9	108.9	35 - 263	0.0	0.0 - 0.0	8.6	2.8 - 20.9	7.8	2.2 - 26.4	40.5	13.1 - 119.8	0.0	0.0 - 0.0	52.0	16.8 - 139.8	0.55
6030004	18023	BIG CR	3.2	137.2	53 - 343	0.0	0.0 - 0.0	11.3	3.8 - 31.5	5.0	1.6 - 14.6	26.3	8.5 - 86.1	0.0	0.0 - 0.0	94.7	35.4 - 243.6	0.55
6030002	18024	FIRST CR	5.2	81.1	25 - 207	0.0	0.0 - 0.0	0.3	0.1 - 0.8	3.7	1.1 - 13.0	18.6	5.1 - 55.4	0.0	0.0 - 0.0	58.5	16.6 - 169.7	0.55
6030002	18025	SECOND CR	4.9	88.1	28 - 220	0.0	0.0 - 0.0	8.7	3.1 - 26.2	3.7	1.2 - 11.1	17.8	5.0 - 54.9	0.0	0.0 - 0.0	57.9	19.0 - 160.7	0.55
6030005	18026	TOWN CR	28.1	118.2	45 - 268	0.0	0.0 - 0.0	5.5	2.0 - 11.2	18.9	6.0 - 52.4	66.1	23.0 - 176.2	0.0	0.0 - 0.0	27.7	9.5 - 70.5	0.55
6030005	18027	BLUEWATER CR	2.2	100.4	35 - 258	0.0	0.0 - 0.0	5.7	2.0 - 13.7	5.7	1.9 - 16.2	27.7	9.0 - 84.3	0.0	0.0 - 0.0	61.3	21.1 - 193.9	0.55
6030005	18028	SHOAL CR	1.4	95.9	29 - 246	0.0	0.0 - 0.0	17.7	4.8 - 43.9	1.7	0.4 - 4.6	10.8	3.1 - 31.5	0.0	0.0 - 0.0	65.7	19.7 - 180.2	0.55
6030005	18029	TENNESSEE R	83.2	101.2	37 - 244	0.0	0.0 - 0.0	20.0	6.7 - 45.3	10.0	3.3 - 29.2	37.0	12.5 - 90.6	0.0	0.0 - 0.0	34.2	12.1 - 88.4	0.57
6030005	18030	TENNESSEE R	7.1	103.5	34 - 267	0.0	0.0 - 0.0	40.0	13.2 - 100.0	5.9	1.5 - 22.7	28.8	10.0 - 88.3	0.0	0.0 - 0.0	28.8	8.6 - 77.0	0.57
6030005	18031	SPRING CR	125.9	155.7	60 - 314	27.0	10.0 - 70.5	53.7	18.8 - 116.7	11.2	3.5 - 33.6	40.8	15.5 - 97.3	0.0	0.0 - 0.0	22.9	8.2 - 56.9	0.57
6030005	18032	LITTLE BEAR CR	17.4	62.8	20 - 134	0.0	0.0 - 0.0	18.3	5.5 - 38.0	6.7	1.7 - 19.5	24.7	7.4 - 65.2	0.0	0.0 - 0.0	13.1	4.2 - 33.6	0.57
6030005	18033	SINKING CR	112.8	99.5	29 - 239	0.0	0.0 - 0.0	8.0	2.0 - 17.4	11.4	2.8 - 34.3	59.3	17.1 - 168.2	0.0	0.0 - 0.0	20.8	6.1 - 54.6	0.57
6030005	18034	CANE CR	67.6	40.0	14 - 84	0.0	0.0 - 0.0	6.0	2.0 - 11.3	5.0	1.5 - 13.4	18.2	5.3 - 47.8	0.0	0.0 - 0.0	10.8	3.3 - 24.0	0.57
6030005	18035	BLUFF CR	49.3	31.5	12 - 61	0.0	0.0 - 0.0	5.5	1.9 - 11.4	1.9	0.6 - 4.7	9.8	3.3 - 23.2	0.0	0.0 - 0.0	14.4	5.1 - 35.1	0.57
6030005	18036	SECOND CR	86.7	21.4	8 - 51	0.0	0.0 - 0.0	3.8	1.3 - 9.0	1.0	0.3 - 3.2	5.9	1.8 - 16.2	0.0	0.0 - 0.0	10.7	4.1 - 25.8	0.57
6030006	18037	BUZZARD ROOST	48.9	30.0	9 - 55	0.0	0.0 - 0.0	8.2	2.6 - 16.6	2.2	0.6 - 5.5	8.0	2.4 - 20.2	0.0	0.0 - 0.0	11.6	3.6 - 26.5	0.57
6030006	18038	BEAR CR	50.1	34.7	10 - 89	0.0	0.0 - 0.0	10.1	2.8 - 25.7	2.0	0.5 - 5.8	10.0	2.9 - 30.1	0.0	0.0 - 0.0	12.6	3.8 - 34.6	0.57
6030005	18039	YELLOW CR	58.1	32.2	14 - 73	0.0	0.0 - 0.0	10.3	4.1 - 22.9	1.3	0.5 - 3.6	9.0	3.6 - 24.8	0.0	0.0 - 0.0	11.6	5.0 - 27.1	0.65
6030005	18040	TENNESSEE R	117.0	27.2	9 - 60	0.0	0.0 - 0.0	6.3	1.8 - 14.6	0.5	0.1 - 1.5	4.0	1.4 - 9.6	0.0	0.0 - 0.0	16.4	5.7 - 38.5	0.64
6030005	18041	TENNESSEE R	187.9	34.0	11 - 76	2.8	1.0 - 6.6	9.0	2.5 - 18.7	0.7	0.2 - 1.8	4.5	1.4 - 12.1	0.0	0.0 - 0.0	17.0	5.5 - 38.6	0.57
6030005	18042	BEAR CR	1.0	35.8	11 - 83	0.0	0.0 - 0.0	1.7	0.5 - 3.7	0.0	0.0 - 0.0	0.0	0.0 - 0.0	0.0	0.0 - 0.0	34.1	10.4 - 82.3	0.57
6030005	18043	TENNESSEE R	16.6	24.7	9 - 52	0.0	0.0 - 0.0	4.8	1.5 - 10.2	0.1	0.0 - 0.4	0.6	0.2 - 1.5	0.0	0.0 - 0.0	19.1	6.6 - 47.7	0.57
6030005	18044	TENNESSEE R	102.8	24.3	9 - 62	0.0	0.0 - 0.0	4.0	1.4 - 10.5	0.6	0.2 - 1.7	3.0	1.1 - 8.1	0.0	0.0 - 0.0	16.6	6.0 - 45.3	0.57

8-digit HUC	ID	Name	Area	Catchment Yield		Point sources		Developed Land		Manure		Agricultural Land		Phosphate Mines		Soil parent rock		Frac
				kg/km^2	90% CI	kg/km^2	90% CI	kg/km^2	90% CI	kg/km^2	90% CI	kg/km^2	90% CI	kg/km^2	90% CI	kg/km^2	90% CI	
6030005	18045	TENNESSEE R	193.7	114.7	37 - 194	44.6	15.2 - 82.9	8.1	2.5 - 14.9	8.1	2.5 - 21.5	33.5	8.2 - 61.5	0.0	0.0 - 0.0	20.4	7.1 - 40.3	0.57
6030005	18046	TENNESSEE R	5.6	63.6	18 - 185	0.0	0.0 - 0.0	13.9	3.8 - 40.2	4.3	1.1 - 14.6	19.6	5.6 - 60.7	0.0	0.0 - 0.0	25.8	7.4 - 80.1	0.57
6030005	18047	TENNESSEE R	33.6	58.1	19 - 107	0.0	0.0 - 0.0	12.0	3.8 - 25.9	5.6	1.9 - 14.7	24.0	8.1 - 54.6	0.0	0.0 - 0.0	16.5	5.6 - 35.6	0.57
6030005	18048	TENNESSEE R	12.3	54.4	20 - 92	0.0	0.0 - 0.0	4.4	1.6 - 8.4	4.6	1.5 - 11.5	21.6	7.8 - 50.7	0.0	0.0 - 0.0	23.8	8.5 - 49.5	0.57
6030006	18049	BEAR CR	130.6	33.8	12 - 61	0.0	0.0 - 0.0	7.6	2.5 - 15.4	1.0	0.3 - 2.6	5.4	1.8 - 11.6	0.0	0.0 - 0.0	19.8	6.7 - 38.2	0.57
6030005	18050	TENNESSEE R	30.7	154.5	55 - 322	61.3	19.9 - 130.3	7.5	2.3 - 14.3	10.6	3.5 - 30.1	54.6	17.9 - 133.1	0.0	0.0 - 0.0	20.6	6.7 - 53.6	0.57
6030005	18051	SPRING CR	1.5	43.8	17 - 106	0.0	0.0 - 0.0	3.1	1.1 - 7.9	1.7	0.6 - 5.5	5.2	1.7 - 15.2	0.0	0.0 - 0.0	33.9	12.9 - 89.4	0.57
6030005	18052	LITTLE BEAR CR	2.4	39.8	15 - 99	0.0	0.0 - 0.0	12.2	4.9 - 31.5	1.4	0.4 - 5.3	4.1	1.5 - 13.0	0.0	0.0 - 0.0	22.1	8.5 - 58.1	0.57
6030005	18053	SINKING CR	1.1	42.7	14 - 87	0.0	0.0 - 0.0	0.0	0.0 - 0.0	0.9	0.2 - 1.9	5.9	1.9 - 11.6	0.0	0.0 - 0.0	36.0	11.7 - 82.8	0.57
6030005	18054	CANE CR	17.1	64.7	23 - 133	0.0	0.0 - 0.0	8.4	2.8 - 18.2	8.2	2.4 - 21.5	29.4	9.0 - 77.1	0.0	0.0 - 0.0	18.7	7.5 - 44.4	0.57
6030005	18055	BLUFF CR	1.1	32.0	11 - 81	0.0	0.0 - 0.0	0.0	0.0 - 0.0	0.2	0.1 - 0.6	1.2	0.4 - 3.1	0.0	0.0 - 0.0	30.6	10.7 - 80.9	0.57
6030005	18056	SECOND CR	54.6	24.1	8 - 44	0.0	0.0 - 0.0	6.2	1.8 - 13.2	1.0	0.3 - 3.0	5.9	1.7 - 17.2	0.0	0.0 - 0.0	11.0	3.7 - 27.9	0.57
6030006	18057	BUZZARD ROOST	1.1	25.5	8 - 61	0.0	0.0 - 0.0	0.0	0.0 - 0.0	2.5	0.8 - 7.4	8.6	3.1 - 25.1	0.0	0.0 - 0.0	14.5	4.1 - 36.8	0.57
6030006	18058	BEAR CR	2.2	15.7	6 - 32	0.0	0.0 - 0.0	0.8	0.3 - 1.9	0.2	0.1 - 0.6	0.7	0.2 - 1.8	0.0	0.0 - 0.0	14.0	5.1 - 29.4	0.57
6030005	18059	YELLOW CR	83.4	32.1	11 - 76	0.1	0.0 - 0.3	9.3	3.1 - 23.0	0.5	0.2 - 1.5	3.7	1.4 - 12.0	0.0	0.0 - 0.0	18.4	6.9 - 42.4	0.64
6040001	18060	CEDAR CR	91.3	59.0	24 - 131	0.0	0.0 - 0.0	3.2	1.3 - 6.4	1.8	0.6 - 5.2	7.6	2.7 - 19.9	0.0	0.0 - 0.0	46.5	16.9 - 116.7	0.65
6040001	18061	CYPRESS CR	51.0	47.7	16 - 90	0.0	0.0 - 0.0	3.4	1.2 - 6.9	1.4	0.4 - 3.3	5.6	1.8 - 12.6	0.0	0.0 - 0.0	37.3	12.9 - 77.4	0.65
6040001	18062	BEECH R	34.7	52.7	22 - 129	0.0	0.0 - 0.0	4.0	1.5 - 9.8	2.7	0.9 - 7.5	11.8	4.4 - 32.6	0.0	0.0 - 0.0	34.3	13.2 - 92.5	0.65
6040001	18063	LICK CR	72.3	44.5	15 - 94	0.0	0.0 - 0.0	1.9	0.7 - 3.9	1.0	0.3 - 2.9	3.4	0.9 - 7.9	0.0	0.0 - 0.0	38.3	12.8 - 95.7	0.65
6040001	18064	CUB CR	61.1	60.4	20 - 128	0.0	0.0 - 0.0	3.4	1.2 - 7.7	3.8	1.1 - 10.2	16.1	5.3 - 37.0	0.0	0.0 - 0.0	37.1	13.0 - 81.2	0.65
6040001	18065	TOM'S CR	74.5	50.7	17 - 116	0.0	0.0 - 0.0	2.2	0.7 - 4.3	1.3	0.3 - 3.1	4.9	1.7 - 11.8	0.5	0.1 - 1.6	41.9	13.8 - 103.8	0.65
6040001	18066	ROAN CR	1.2	148.7	54 - 362	0.0	0.0 - 0.0	3.6	1.1 - 7.4	7.7	2.2 - 23.0	33.9	11.7 - 85.5	0.0	0.0 - 0.0	103.5	36.5 - 286.6	0.65
6040001	18067	ROAN CR	39.6	46.3	16 - 133	0.0	0.0 - 0.0	1.1	0.4 - 3.0	1.0	0.4 - 3.5	3.7	1.4 - 11.6	0.0	0.0 - 0.0	40.5	13.8 - 120.7	0.64
6040001	18068	CROOKED CR	42.5	49.5	16 - 111	0.0	0.0 - 0.0	1.6	0.5 - 4.0	1.2	0.4 - 4.0	4.4	1.4 - 12.5	0.0	0.0 - 0.0	42.2	13.2 - 100.0	0.65
6040001	18069	*A	71.1	60.4	22 - 159	0.0	0.0 - 0.0	5.1	1.6 - 12.0	3.5	1.2 - 10.7	14.6	6.3 - 42.6	0.0	0.0 - 0.0	37.2	13.6 - 111.2	0.65
6040005	18070	BIRDSONG CR	168.1	58.4	19 - 120	0.0	0.0 - 0.0	3.9	1.3 - 7.6	5.4	1.5 - 12.8	19.9	6.1 - 46.8	0.0	0.0 - 0.0	29.3	9.4 - 66.8	0.65
6040005	18071	TRACE CR	92.6	94.2	31 - 216	19.3	6.4 - 48.1	11.8	4.0 - 26.0	6.6	2.3 - 20.0	16.3	5.3 - 58.6	0.0	0.0 - 0.0	40.2	13.0 - 96.4	0.65
6040005	18072	DRY CR	19.2	40.2	13 - 90	0.0	0.0 - 0.0	1.6	0.4 - 3.2	1.3	0.4 - 3.4	1.4	0.4 - 3.2	0.0	0.0 - 0.0	35.9	11.5 - 83.0	0.65
6040005	18073	BIG RICHLAND CR	133.2	42.5	14 - 89	0.0	0.0 - 0.0	2.9	1.0 - 5.9	3.4	1.1 - 9.8	5.3	1.9 - 14.6	0.0	0.0 - 0.0	30.9	9.7 - 72.1	0.65
6040005	18074	LITTLE RICHLAND	52.5	41.8	14 - 98	0.0	0.0 - 0.0	2.6	0.9 - 5.5	2.9	0.9 - 7.9	4.8	1.4 - 13.2	0.0	0.0 - 0.0	31.5	10.2 - 74.7	0.65
6040005	18075	WHITEOAK CR	246.3	43.8	14 - 109	0.0	0.0 - 0.0	2.8	0.9 - 6.2	3.9	1.3 - 13.8	5.4	1.7 - 12.5	0.0	0.0 - 0.0	31.7	10.7 - 81.9	0.65
6040005	18076	CANE CR	47.8	49.5	14 - 95	0.0	0.0 - 0.0	4.1	1.2 - 8.4	6.1	1.7 - 12.2	6.9	2.0 - 15.6	0.0	0.0 - 0.0	32.4	8.9 - 69.1	0.65
6040005	18077	HURRICANE CR	58.6	41.4	13 - 97	0.0	0.0 - 0.0	3.5	0.9 - 7.4	3.3	0.9 - 10.0	4.8	1.6 - 14.0	0.0	0.0 - 0.0	29.9	9.3 - 76.4	0.65
6040005	18078	STANDING ROCK (	66.0	44.4	17 - 106	0.0	0.0 - 0.0	2.5	0.9 - 5.6	2.5	0.7 - 7.0	7.0	2.2 - 20.0	0.0	0.0 - 0.0	32.5	12.2 - 88.5	0.65
6040005	18079	BLOOD R	181.4	121.8	44 - 272	0.0	0.0 - 0.0	4.2	1.5 - 8.5	15.4	4.5 - 41.9	63.6	22.7 - 174.8	0.0	0.0 - 0.0	38.5	12.7 - 96.6	0.65
6040005	18080	BIG SANDY CR	102.6	155.5	58 - 321	2.4	1.0 - 5.3	5.9	2.6 - 11.8	16.3	5.0 - 55.1	64.0	22.5 - 134.1	0.0	0.0 - 0.0	66.8	25.3 - 169.0	0.65
6040005	18081	W SANDY CR	10.1	98.5	36 - 203	0.0	0.0 - 0.0	2.5	0.8 - 5.2	10.1	3.2 - 26.6	38.1	13.7 - 99.1	0.0	0.0 - 0.0	47.7	16.1 - 123.3	0.65
6040005	18082	TENNESSEE R	638.4	39.0	13 - 81	0.4	0.1 - 0.9	1.6	0.5 - 3.2	2.4	0.7 - 5.8	10.2	2.8 - 24.1	0.0	0.0 - 0.0	24.5	7.6 - 62.7	1.00
6040005	18083	TENNESSEE R	267.1	48.9	15 - 113	0.0	0.0 - 0.0	1.6	0.5 - 3.4	0.7	0.2 - 2.3	2.7	0.9 - 7.5	0.0	0.0 - 0.0	43.8	13.9 - 112.4	0.65

8-digit HUC	ID	Name	Area	Catchment Yield		Point sources		Developed Land		Manure		Agricultural Land		Phosphate Mines		Soil parent rock		Frac
				kg/km^2	90% CI	kg/km^2	90% CI	kg/km^2	90% CI	kg/km^2	90% CI	kg/km^2	90% CI	kg/km^2	90% CI	kg/km^2	90% CI	
6040005	18084	BIG SANDY CR	130.7	62.6	25 - 120	0.0	0.0 - 0.0	4.9	1.6 - 10.0	4.4	1.5 - 13.0	14.2	4.7 - 32.6	0.0	0.0 - 0.0	39.0	14.4 - 93.6	0.65
6040005	18085	TENNESSEE R	2.3	80.5	30 - 208	0.0	0.0 - 0.0	0.0	0.0 - 0.0	0.0	0.0 - 0.0	0.0	0.0 - 0.0	0.0	0.0 - 0.0	80.5	29.7 - 208.5	0.65
6040005	18086	TENNESSEE R	166.4	51.1	22 - 112	0.0	0.0 - 0.0	1.7	0.6 - 3.9	1.4	0.5 - 3.7	4.3	1.4 - 9.6	0.0	0.0 - 0.0	43.7	18.1 - 98.2	0.65
6040005	18087	TENNESSEE R	13.9	45.9	13 - 99	0.0	0.0 - 0.0	1.7	0.6 - 3.1	0.4	0.1 - 0.7	0.9	0.3 - 1.8	0.0	0.0 - 0.0	42.9	12.3 - 93.7	0.65
6040005	18088	TENNESSEE R	50.2	41.4	13 - 84	0.0	0.0 - 0.0	1.8	0.5 - 3.2	0.8	0.2 - 1.8	1.9	0.5 - 4.3	0.0	0.0 - 0.0	37.0	11.1 - 81.6	0.65
6040005	18089	TENNESSEE R	135.6	50.3	18 - 138	0.0	0.0 - 0.0	1.3	0.5 - 3.2	1.5	0.5 - 4.4	3.8	1.6 - 10.2	0.0	0.0 - 0.0	43.7	15.6 - 127.4	0.65
6040005	18090	TENNESSEE R	78.6	51.2	17 - 115	0.0	0.0 - 0.0	1.6	0.5 - 3.1	1.7	0.5 - 3.7	4.8	1.5 - 12.9	0.0	0.0 - 0.0	43.2	13.5 - 107.2	0.65
6040005	18091	TENNESSEE R	31.8	195.3	71 - 417	143.6	49.5 - 333.7	2.8	1.0 - 6.9	1.2	0.4 - 3.8	2.7	1.1 - 8.0	0.0	0.0 - 0.0	44.9	17.7 - 93.1	0.65
6040005	18092	LITTLE RICHLAND	2.7	70.8	24 - 177	0.0	0.0 - 0.0	0.0	0.0 - 0.0	1.4	0.4 - 4.1	1.8	0.6 - 4.9	0.0	0.0 - 0.0	67.6	22.4 - 167.6	0.65
6040001	18093	CEDAR CR	1.8	146.8	55 - 306	0.0	0.0 - 0.0	4.7	1.8 - 11.3	7.2	2.3 - 24.8	35.4	11.8 - 101.9	0.0	0.0 - 0.0	99.4	34.7 - 257.6	0.65
6040001	18094	TENNESSEE R	17.6	84.4	28 - 178	0.0	0.0 - 0.0	2.3	0.7 - 4.9	5.0	1.5 - 14.4	23.1	6.6 - 53.2	0.0	0.0 - 0.0	54.0	18.0 - 112.9	0.65
6040001	18095	TENNESSEE R	1.4	137.4	50 - 362	0.0	0.0 - 0.0	5.0	1.8 - 15.4	6.5	1.8 - 20.9	31.0	10.0 - 99.1	0.0	0.0 - 0.0	95.0	34.5 - 249.3	0.65
6040001	18097	BEECH R	3.8	54.3	18 - 127	0.0	0.0 - 0.0	3.3	0.9 - 7.9	2.4	0.7 - 6.3	10.5	3.3 - 29.2	0.0	0.0 - 0.0	38.1	12.5 - 90.9	0.65
6040001	18098	LICK CR	1.7	108.5	38 - 277	0.0	0.0 - 0.0	0.0	0.0 - 0.0	0.8	0.2 - 3.2	4.2	1.2 - 11.8	0.0	0.0 - 0.0	103.4	36.6 - 269.8	0.65
6040001	18099	TENNESSEE R	4.5	96.1	35 - 188	0.0	0.0 - 0.0	0.3	0.1 - 0.6	4.1	1.3 - 9.6	20.4	7.0 - 48.1	0.0	0.0 - 0.0	71.3	24.3 - 160.8	0.65
6040001	18100	CUB CR	5.3	50.9	15 - 106	0.0	0.0 - 0.0	0.0	0.0 - 0.0	2.6	0.7 - 6.8	13.2	3.1 - 36.7	0.0	0.0 - 0.0	35.0	11.0 - 75.4	0.65
6040001	18101	TENNESSEE R	120.2	50.6	17 - 125	0.0	0.0 - 0.0	3.5	1.2 - 7.9	2.0	0.6 - 6.3	6.9	2.3 - 17.2	0.0	0.0 - 0.0	38.2	12.5 - 111.5	0.65
6040001	18102	TOM'S CR	9.1	51.1	19 - 113	0.0	0.0 - 0.0	2.1	0.7 - 4.3	1.5	0.4 - 4.4	5.4	1.8 - 14.9	0.0	0.0 - 0.0	42.1	14.8 - 98.8	0.65
6040001	18103	TENNESSEE R	3.7	59.1	21 - 110	0.0	0.0 - 0.0	2.6	0.9 - 4.9	3.8	1.2 - 8.4	17.7	5.4 - 42.8	0.0	0.0 - 0.0	35.1	11.5 - 77.9	0.65
6040001	18104	CROOKED CR	2.4	166.4	63 - 374	0.0	0.0 - 0.0	3.0	1.0 - 6.4	8.0	2.5 - 23.4	41.3	13.5 - 113.0	0.0	0.0 - 0.0	114.2	41.1 - 297.0	0.65
6040005	18105	TENNESSEE R	232.4	153.8	49 - 359	22.3	7.6 - 53.6	13.8	4.9 - 28.0	9.8	2.9 - 33.8	34.0	10.9 - 87.5	0.0	0.0 - 0.0	74.0	23.7 - 202.8	0.65
6040001	18106	TENNESSEE R	31.9	73.6	22 - 132	0.0	0.0 - 0.0	1.6	0.5 - 2.8	2.9	0.7 - 5.8	7.7	2.2 - 15.6	0.0	0.0 - 0.0	61.4	17.4 - 121.1	0.65
6040001	18107	TENNESSEE R	167.3	46.8	14 - 105	0.0	0.0 - 0.0	1.8	0.5 - 4.2	1.1	0.3 - 3.2	3.2	0.8 - 7.5	0.0	0.0 - 0.0	40.7	12.5 - 96.4	0.65
6040003	18108	DUCK R	56.2	86.7	29 - 252	0.0	0.0 - 0.0	4.8	1.5 - 11.4	7.4	2.0 - 28.0	19.7	7.6 - 64.8	0.0	0.0 - 0.0	54.8	17.7 - 181.6	0.65
6040005	18109	BIRDSONG CR	71.7	67.3	20 - 156	0.0	0.0 - 0.0	3.9	1.3 - 10.0	3.0	0.8 - 8.5	10.8	2.9 - 27.7	0.0	0.0 - 0.0	49.5	14.5 - 113.2	0.65
6040005	18110	TRACE CR	10.9	56.2	21 - 140	0.0	0.0 - 0.0	0.6	0.2 - 1.6	0.5	0.1 - 1.3	1.1	0.4 - 3.1	0.0	0.0 - 0.0	54.0	19.6 - 133.7	0.65
6040005	18111	DRY CR	2.2	73.9	24 - 167	0.0	0.0 - 0.0	1.3	0.4 - 2.7	0.1	0.0 - 0.3	0.6	0.2 - 1.4	0.0	0.0 - 0.0	72.0	22.8 - 164.8	0.65
6040005	18112	BIG RICHLAND CR	8.7	48.8	14 - 98	0.0	0.0 - 0.0	1.6	0.5 - 2.9	0.7	0.2 - 1.8	1.5	0.5 - 3.0	0.0	0.0 - 0.0	45.1	12.9 - 98.1	0.65
6040005	18113	LITTLE RICHLAND	1.2	65.1	25 - 171	0.0	0.0 - 0.0	1.2	0.4 - 2.7	0.1	0.0 - 0.2	0.9	0.4 - 2.4	0.0	0.0 - 0.0	62.9	24.2 - 169.1	0.65
6040005	18114	WHITEOAK CR	32.7	46.4	15 - 113	0.0	0.0 - 0.0	0.7	0.2 - 1.4	1.5	0.4 - 4.6	1.9	0.6 - 5.4	0.0	0.0 - 0.0	42.4	14.8 - 109.0	0.65
6040005	18115	CANE CR	13.9	53.3	18 - 119	0.0	0.0 - 0.0	2.8	1.0 - 6.2	2.8	0.9 - 7.9	3.3	1.2 - 9.5	0.0	0.0 - 0.0	44.3	14.5 - 107.4	0.65
6040005	18116	HURRICANE CR	10.2	45.5	15 - 104	0.0	0.0 - 0.0	1.0	0.3 - 2.0	0.3	0.1 - 0.7	0.2	0.1 - 0.5	0.0	0.0 - 0.0	44.0	14.7 - 100.3	0.65
6040005	18117	STANDING ROCK C	11.1	50.4	15 - 107	0.0	0.0 - 0.0	0.7	0.2 - 1.5	0.2	0.1 - 0.5	0.3	0.1 - 0.7	0.0	0.0 - 0.0	49.1	14.9 - 105.0	0.65
6040005	18118	BLOOD R	154.9	70.4	24 - 171	0.0	0.0 - 0.0	3.1	1.0 - 7.1	5.6	1.5 - 20.3	25.9	8.5 - 63.2	0.0	0.0 - 0.0	35.8	10.9 - 91.7	0.65
6040005	18119	BIG SANDY CR	86.0	80.4	28 - 214	0.0	0.0 - 0.0	3.8	1.2 - 11.3	6.1	2.0 - 17.7	22.4	8.0 - 74.1	0.0	0.0 - 0.0	48.1	16.3 - 138.9	0.65
6040005	18120	W SANDY CR	24.2	122.9	43 - 242	0.0	0.0 - 0.0	5.2	1.7 - 10.1	14.2	4.4 - 36.3	51.5	16.9 - 139.5	0.0	0.0 - 0.0	52.1	16.3 - 118.3	0.65
6010107	18121	FRENCH BROAD R	66.2	55.5	19 - 121	5.8	2.0 - 12.3	7.9	2.5 - 18.0	8.6	2.5 - 25.0	16.8	5.0 - 40.3	0.0	0.0 - 0.0	16.3	4.9 - 40.0	0.15
6010107	18122	BALL CR	8.7	31.7	11 - 55	0.0	0.0 - 0.0	8.8	2.7 - 18.2	3.9	1.2 - 10.3	7.2	2.4 - 15.6	0.0	0.0 - 0.0	11.8	4.1 - 25.5	0.15
6010107	18123	FRENCH BROAD R	91.0	64.4	20 - 129	0.0	0.0 - 0.0	6.8	1.9 - 16.0	14.5	4.0 - 46.3	27.7	8.8 - 73.6	0.0	0.0 - 0.0	15.5	4.6 - 37.3	0.15

8-digit HUC	ID	Name	Area	Catchment Yield		Point sources		Developed Land		Manure		Agricultural Land		Phosphate Mines		Soil parent rock		Frac
				kg/km^2	90% CI	kg/km^2	90% CI	kg/km^2	90% CI	kg/km^2	90% CI	kg/km^2	90% CI	kg/km^2	90% CI	kg/km^2	90% CI	
6010107	18124	BALL CR	19.1	35.6	11 - 66	0.0	0.0 - 0.0	4.0	1.2 - 8.3	5.9	1.4 - 18.2	10.2	3.4 - 22.5	0.0	0.0 - 0.0	15.5	5.0 - 35.8	0.15
6010107	18125	FRENCH BROAD R	296.3	29.0	10 - 58	0.8	0.3 - 1.6	3.8	1.1 - 7.7	5.2	1.5 - 15.7	10.8	3.5 - 25.7	0.0	0.0 - 0.0	8.6	2.9 - 19.6	0.25
6040001	18126	TENNESSEE R	14.5	59.8	24 - 119	0.0	0.0 - 0.0	1.7	0.7 - 3.5	2.0	0.7 - 5.0	4.9	1.8 - 11.5	0.0	0.0 - 0.0	51.2	20.3 - 113.5	0.65
6040006	18127	TENNESSEE R	330.9	177.2	67 - 382	0.3	0.1 - 0.6	10.2	3.6 - 19.8	10.1	3.1 - 28.7	74.7	26.9 - 193.9	0.0	0.0 - 0.0	81.9	29.8 - 190.7	1.00
6040001	18128	*A	1.2	48.6	21 - 170	0.0	0.0 - 0.0	0.0	0.0 - 0.0	0.0	0.0 - 0.0	0.0	0.0 - 0.0	0.0	0.0 - 0.0	48.6	20.9 - 169.9	0.65
6040001	18129	TENNESSEE R	25.2	40.3	12 - 99	0.0	0.0 - 0.0	2.4	0.6 - 4.7	1.1	0.3 - 3.2	4.2	1.2 - 9.6	0.0	0.0 - 0.0	32.7	9.3 - 83.2	0.65
6040001	18130	TENNESSEE R	65.4	53.2	20 - 105	0.0	0.0 - 0.0	3.0	1.0 - 6.3	1.4	0.4 - 4.2	5.7	2.2 - 12.7	0.0	0.0 - 0.0	43.1	15.3 - 95.8	0.65
6040001	18131	TENNESSEE R	83.7	56.0	18 - 112	0.0	0.0 - 0.0	2.1	0.6 - 4.7	2.0	0.6 - 6.2	8.8	3.1 - 19.5	0.0	0.0 - 0.0	43.0	13.3 - 92.0	0.65
6030002	18132	TENNESSEE R	5.5	73.8	28 - 202	0.0	0.0 - 0.0	4.4	1.5 - 10.5	2.4	0.8 - 8.4	7.2	2.6 - 20.5	0.0	0.0 - 0.0	59.8	21.7 - 175.6	0.55
6030002	18133	TENNESSEE R	94.0	121.7	36 - 241	0.0	0.0 - 0.0	9.2	2.9 - 19.4	22.0	5.4 - 69.9	56.8	15.5 - 155.2	0.0	0.0 - 0.0	33.6	9.1 - 78.8	0.55
6030002	18134	TENNESSEE R	119.1	107.0	42 - 256	0.0	0.0 - 0.0	5.9	2.1 - 13.6	12.4	3.6 - 37.9	56.3	21.3 - 152.7	0.0	0.0 - 0.0	32.3	11.5 - 74.6	0.55
6030002	18135	TENNESSEE R	142.8	353.0	122 - 651	232.0	77.6 - 469.3	50.0	15.9 - 95.3	10.4	3.2 - 26.0	31.5	9.3 - 73.2	0.0	0.0 - 0.0	29.1	10.3 - 63.4	0.55
6030002	18136	TENNESSEE R	78.4	87.9	29 - 165	0.0	0.0 - 0.0	6.9	2.2 - 13.8	18.6	5.1 - 58.0	43.7	14.1 - 122.5	0.0	0.0 - 0.0	18.8	5.9 - 40.9	0.55
6030001	18137	TENNESSEE R	138.1	47.7	17 - 101	0.0	0.0 - 0.0	5.9	2.0 - 12.5	11.9	3.8 - 31.2	12.5	4.1 - 32.4	0.0	0.0 - 0.0	17.4	6.3 - 40.0	0.45
6030001	18138	TENNESSEE R	39.7	40.7	11 - 116	0.0	0.0 - 0.0	5.6	1.7 - 14.6	8.5	2.3 - 26.7	14.1	3.8 - 42.0	0.0	0.0 - 0.0	12.5	3.4 - 38.8	0.45
6030001	18139	TENNESSEE R	307.9	69.3	26 - 161	17.2	6.1 - 40.7	12.8	4.2 - 30.9	6.7	2.3 - 17.5	19.3	7.1 - 48.8	0.0	0.0 - 0.0	13.3	5.0 - 35.3	0.45
6030005	18140	BIG NANCE CR	3.8	75.5	23 - 170	0.0	0.0 - 0.0	11.3	3.6 - 26.7	7.9	2.3 - 23.9	18.9	6.4 - 53.1	0.0	0.0 - 0.0	37.4	11.1 - 87.9	0.55
3010201	65040	NOTTOWAY R	3.6	33.2	11 - 88	0.0	0.0 - 0.0	0.0	0.0 - 0.0	2.9	0.9 - 8.6	20.5	6.4 - 63.3	0.0	0.0 - 0.0	9.8	3.4 - 22.2	0.94
3010202	65041	BLACKWATER R	138.3	43.5	14 - 91	0.0	0.0 - 0.0	0.8	0.3 - 1.8	7.2	2.2 - 20.8	25.1	8.4 - 61.8	0.0	0.0 - 0.0	10.4	3.3 - 27.6	0.93
3010204	65042	MEHERIN R	237.1	18.1	7 - 43	0.0	0.0 - 0.0	5.0	1.7 - 12.6	0.6	0.2 - 1.9	5.8	2.1 - 16.2	0.0	0.0 - 0.0	6.6	2.2 - 16.8	0.89
3010104	65043	DAN R	104.4	27.8	9 - 69	0.0	0.0 - 0.0	4.0	1.2 - 9.4	2.4	0.7 - 6.8	10.3	3.1 - 31.7	0.0	0.0 - 0.0	11.1	3.8 - 26.2	0.36
3010106	65044	ROANOKE R	40.1	465.7	162 - 1067	404.1	142.8 - 968.4	19.4	6.1 - 41.0	8.5	2.5 - 23.4	21.9	7.3 - 53.4	0.0	0.0 - 0.0	11.9	4.1 - 29.6	0.95
3020103	65045	TAR R	42.9	80.7	28 - 136	0.0	0.0 - 0.0	13.0	4.2 - 23.9	15.9	4.4 - 45.7	38.7	13.3 - 80.3	0.0	0.0 - 0.0	13.2	4.4 - 30.5	0.95
3020202	65046	NEUSE R	21.8	98.2	40 - 269	0.0	0.0 - 0.0	41.4	15.0 - 101.1	12.4	4.1 - 40.3	13.2	4.8 - 39.0	0.0	0.0 - 0.0	31.1	12.7 - 89.1	0.97
3020203	65047	CONTENTNEA CR	227.2	95.9	32 - 171	0.7	0.2 - 1.3	6.9	2.1 - 14.8	46.6	14.4 - 119.1	27.1	9.2 - 60.9	0.0	0.0 - 0.0	14.6	4.6 - 28.5	0.95
3030005	65048	CAPE FEAR R	176.7	27.4	10 - 58	0.1	0.0 - 0.1	1.8	0.7 - 4.1	14.1	4.5 - 32.6	5.8	1.9 - 15.7	0.0	0.0 - 0.0	5.6	2.3 - 15.1	0.98
3040101	65049	YADKIN R	139.2	35.9	11 - 81	0.2	0.1 - 0.5	6.4	2.0 - 14.5	3.7	0.9 - 9.9	13.9	3.9 - 37.2	0.0	0.0 - 0.0	11.7	3.2 - 32.0	0.47
3040105	65050	ROCKY R	2.3	54.5	18 - 120	0.0	0.0 - 0.0	1.4	0.4 - 2.9	20.5	5.8 - 64.8	16.7	5.0 - 43.3	0.0	0.0 - 0.0	15.9	5.3 - 34.9	0.77
3040201	65051	PEE DEE R	105.4	22.5	6 - 42	0.0	0.0 - 0.0	5.3	1.6 - 10.9	5.0	1.5 - 14.0	3.1	1.0 - 7.4	0.0	0.0 - 0.0	9.1	2.7 - 22.9	0.83
3050112	65055	SANTEE R	2.4	50.7	17 - 110	0.0	0.0 - 0.0	2.8	1.0 - 5.9	2.9	0.8 - 10.0	23.9	7.8 - 72.0	0.0	0.0 - 0.0	21.0	6.0 - 53.6	0.98
3050205	65056	EDISTO R	10.6	36.8	12 - 93	0.0	0.0 - 0.0	2.5	0.9 - 5.6	1.5	0.5 - 4.3	8.2	2.8 - 23.8	0.0	0.0 - 0.0	24.7	8.0 - 71.1	0.93
3060106	65057	UPPER THREE RUN	27.4	17.5	7 - 37	0.0	0.0 - 0.0	9.7	3.7 - 20.2	0.8	0.3 - 2.1	1.8	0.7 - 4.0	0.0	0.0 - 0.0	5.1	1.9 - 11.4	0.75
3060109	65058	SAVANNAH R	2.5	36.9	12 - 78	0.0	0.0 - 0.0	2.7	0.8 - 6.6	0.5	0.1 - 1.6	8.3	2.7 - 23.9	0.0	0.0 - 0.0	25.3	8.0 - 61.9	0.98
3060202	65059	OGEECHEE R	320.1	23.1	8 - 62	0.0	0.0 - 0.0	4.0	1.3 - 11.2	0.8	0.3 - 2.2	8.1	2.8 - 24.2	0.0	0.0 - 0.0	10.2	3.3 - 28.8	0.96
3070103	65060	FALLING CR	1.1	15.9	5 - 37	0.0	0.0 - 0.0	2.7	1.0 - 6.9	0.0	0.0 - 0.0	0.0	0.0 - 0.0	0.0	0.0 - 0.0	13.2	4.5 - 31.3	0.78
3070106	65061	ALTAMAHA R	25.1	8.0	2 - 18	0.0	0.0 - 0.0	6.1	1.6 - 14.7	0.1	0.0 - 0.2	0.4	0.1 - 1.1	0.0	0.0 - 0.0	1.4	0.4 - 3.6	0.99
3070106	65062	ALTAMAHA R	226.4	8.0	3 - 17	0.0	0.0 - 0.0	1.6	0.5 - 3.6	0.2	0.1 - 0.6	0.7	0.2 - 1.7	0.0	0.0 - 0.0	5.4	2.0 - 13.5	1.00
3070204	65064	ST MARYS R	3.4	24.2	8 - 45	0.0	0.0 - 0.0	3.3	1.1 - 6.8	3.2	0.9 - 8.2	3.5	1.1 - 8.1	0.0	0.0 - 0.0	14.3	4.3 - 30.6	0.88
3080102	65065	OKLAWAHA R	788.1	19.6	6 - 38	0.0	0.0 - 0.0	8.5	2.4 - 19.1	0.8	0.2 - 1.9	2.6	0.8 - 6.3	0.0	0.0 - 0.0	7.8	2.5 - 16.4	0.21

8-digit HUC	ID	Name	Area	Catchment Yield		Point sources		Developed Land		Manure		Agricultural Land		Phosphate Mines		Soil parent rock		Frac
				kg/km^2	90% CI	kg/km^2	90% CI	kg/km^2	90% CI	kg/km^2	90% CI	kg/km^2	90% CI	kg/km^2	90% CI	kg/km^2	90% CI	
3080103	65066	ST JOHNS R	6.4	1.1	0 - 4	0.0	0.0 - 0.0	0.2	0.0 - 0.9	0.0	0.0 - 0.0	0.0	0.0 - 0.0	0.0	0.0 - 0.0	0.9	0.2 - 3.5	0.99
3080203	65067	MAIN CANAL	684.7	179.0	57 - 381	0.5	0.1 - 1.0	31.9	10.2 - 68.8	12.4	3.3 - 42.5	78.6	23.7 - 225.5	0.0	0.0 - 0.0	55.6	17.4 - 132.6	0.99
3100102	65072	MYAKKA R	2.2	84.5	26 - 215	0.0	0.0 - 0.0	7.9	2.2 - 18.7	12.3	3.1 - 44.2	28.8	7.9 - 79.6	0.0	0.0 - 0.0	35.5	10.8 - 91.9	0.93
3110102	65078	STEINHATCHEE R	41.2	17.5	6 - 31	0.0	0.0 - 0.0	4.8	1.8 - 9.1	0.3	0.1 - 1.0	1.0	0.3 - 2.2	0.0	0.0 - 0.0	11.3	3.9 - 24.2	0.97
3110103	65079	AUCILLA R	27.8	19.5	6 - 43	0.0	0.0 - 0.0	7.6	2.2 - 17.5	0.3	0.1 - 1.0	0.3	0.1 - 0.7	0.4	0.1 - 1.0	10.9	3.5 - 26.3	0.98
3120001	65080	ST MARKS R	898.1	53.8	18 - 109	0.0	0.0 - 0.0	30.6	10.0 - 68.1	0.6	0.2 - 1.6	3.8	1.3 - 10.2	0.0	0.0 - 0.0	18.8	6.4 - 41.6	0.98
3120003	65081	SOPCHOPPY CR	296.3	19.8	6 - 37	0.0	0.0 - 0.0	1.9	0.6 - 4.0	0.0	0.0 - 0.1	0.1	0.0 - 0.1	0.0	0.0 - 0.0	17.8	5.4 - 37.4	0.93
3120003	65082	OCHOCKONEE R	36.0	49.9	16 - 89	0.0	0.0 - 0.0	20.8	6.7 - 41.7	0.5	0.1 - 1.4	4.1	1.3 - 10.4	0.0	0.0 - 0.0	24.4	8.6 - 54.0	0.60
3130004	65083	CHATTAHOOCHEE	8.6	37.8	11 - 80	0.0	0.0 - 0.0	5.1	1.5 - 11.9	2.5	0.7 - 8.4	18.7	6.0 - 51.6	0.0	0.0 - 0.0	11.5	3.8 - 29.1	0.61
3130012	65085	CHIPOLA R	269.3	37.7	13 - 68	0.0	0.0 - 0.0	8.8	3.2 - 15.9	2.0	0.6 - 4.9	9.8	3.4 - 22.2	0.0	0.0 - 0.0	17.1	6.4 - 37.6	0.69
3140101	65086	ECONFINA CR	301.1	22.1	7 - 62	0.0	0.0 - 0.0	10.3	3.3 - 27.3	0.3	0.1 - 1.1	1.3	0.4 - 3.9	0.0	0.0 - 0.0	10.2	2.9 - 31.3	0.47
3140203	65087	CHOCTAWHATCH	1.5	31.9	9 - 55	0.0	0.0 - 0.0	23.0	6.2 - 43.6	0.7	0.2 - 1.8	1.9	0.5 - 4.6	0.0	0.0 - 0.0	6.3	2.1 - 13.5	0.99
3140305	65090	ESCAMBIA R	2.8	42.0	15 - 103	0.0	0.0 - 0.0	17.4	6.4 - 42.4	1.1	0.3 - 3.3	2.9	1.0 - 9.5	0.0	0.0 - 0.0	20.6	7.5 - 54.5	0.98
3140106	65091	PERDIDO R	110.3	36.0	12 - 99	0.0	0.0 - 0.0	8.6	2.8 - 21.3	1.9	0.6 - 6.4	12.0	4.6 - 37.0	0.0	0.0 - 0.0	13.5	4.4 - 40.5	0.97
3150201	65092	ALABAMA R	237.8	160.7	50 - 329	91.4	29.1 - 207.5	36.6	12.1 - 81.8	3.5	1.1 - 9.8	18.2	5.3 - 43.4	0.0	0.0 - 0.0	11.0	3.8 - 27.0	0.80
3150204	65094	ALABAMA R	3.1	122.6	35 - 245	0.0	0.0 - 0.0	8.5	2.2 - 15.9	10.9	2.8 - 32.0	68.2	18.4 - 157.9	0.0	0.0 - 0.0	35.0	11.3 - 81.2	0.98
3160106	65095	TOMBIGBEE R	5.7	69.4	25 - 166	0.0	0.0 - 0.0	6.2	2.1 - 14.5	5.9	1.7 - 15.5	27.4	8.6 - 85.8	0.0	0.0 - 0.0	29.9	10.5 - 68.2	0.89
3160110	65096	BLACK WARRIOR	5.0	8.0	3 - 18	0.0	0.0 - 0.0	3.2	1.1 - 7.1	0.0	0.0 - 0.0	0.0	0.0 - 0.0	0.0	0.0 - 0.0	4.7	1.8 - 11.4	0.17
3170006	65097	PASCAGOULA R	18.6	21.3	8 - 45	0.0	0.0 - 0.0	3.6	1.3 - 7.4	1.3	0.4 - 3.6	2.2	0.7 - 6.8	0.0	0.0 - 0.0	14.2	5.5 - 31.0	0.98
3170007	65098	CYPRESS CR	137.7	13.8	4 - 26	0.0	0.0 - 0.0	5.4	1.4 - 10.8	0.2	0.1 - 0.7	0.2	0.0 - 0.5	0.0	0.0 - 0.0	8.0	2.5 - 17.3	0.92
3180005	65101	BOGUE CHITO R	372.7	35.2	13 - 74	0.0	0.0 - 0.0	8.6	3.2 - 17.4	6.4	1.9 - 18.2	15.5	5.2 - 35.5	0.0	0.0 - 0.0	4.6	1.7 - 10.7	0.95
6010105	65131	FRENCH BROAD R	7.7	22.8	9 - 46	0.0	0.0 - 0.0	5.8	2.0 - 12.3	1.6	0.5 - 4.7	6.0	2.3 - 14.2	0.0	0.0 - 0.0	9.3	3.8 - 21.0	0.15
6010107	65133	FRENCH BROAD R	149.3	48.8	19 - 87	0.0	0.0 - 0.0	7.9	3.0 - 17.3	7.1	2.3 - 20.4	20.4	7.1 - 46.8	0.0	0.0 - 0.0	13.3	4.8 - 29.2	0.25
6010104	65134	HOLSTON R	99.9	62.7	22 - 122	0.0	0.0 - 0.0	24.0	7.8 - 48.2	6.7	2.0 - 16.3	19.5	6.3 - 44.2	0.0	0.0 - 0.0	12.5	4.1 - 25.3	0.25
6010201	65135	LITTLE R	273.9	17.9	7 - 33	0.0	0.0 - 0.0	0.7	0.3 - 1.4	0.0	0.0 - 0.0	0.0	0.0 - 0.0	0.0	0.0 - 0.0	17.2	6.5 - 32.3	0.24
6010207	65136	CLINCH R	31.0	52.5	16 - 98	0.0	0.0 - 0.0	18.5	5.7 - 42.7	6.2	2.0 - 16.6	13.3	3.3 - 32.9	0.0	0.0 - 0.0	14.5	5.2 - 35.2	0.30
6010201	65137	TENNESSEE R	18.1	45.7	17 - 88	0.0	0.0 - 0.0	6.3	2.2 - 12.6	3.5	1.2 - 9.2	13.3	4.6 - 32.1	0.0	0.0 - 0.0	22.6	8.5 - 49.0	0.30
6030001	65138	TENNESSEE R	8.9	52.6	18 - 131	0.0	0.0 - 0.0	26.5	9.0 - 64.8	3.8	1.2 - 11.6	9.6	3.2 - 26.7	0.0	0.0 - 0.0	12.7	4.6 - 40.2	0.44
6040004	65140	BUFFALO R	61.8	69.0	25 - 159	0.0	0.0 - 0.0	7.1	2.0 - 15.5	4.0	1.2 - 10.1	15.9	5.3 - 41.1	0.0	0.0 - 0.0	42.1	14.6 - 110.5	0.59
6040006	65141	TENNESSEE R	314.8	194.7	58 - 453	54.5	17.7 - 159.1	9.3	2.8 - 21.1	14.7	3.7 - 43.9	65.0	19.5 - 160.2	0.0	0.0 - 0.0	51.1	14.6 - 143.6	1.00
3130001	65490	CHATTAHOOCHEE	49.0	96.9	26 - 196	0.0	0.0 - 0.0	82.7	21.9 - 168.2	0.6	0.2 - 1.7	4.5	1.1 - 11.2	0.0	0.0 - 0.0	9.1	2.7 - 21.0	0.17
3130010	65495	AYCOCKS CR	68.9	60.1	23 - 129	0.0	0.0 - 0.0	6.3	2.4 - 13.2	3.8	1.2 - 11.1	36.5	12.4 - 103.8	0.0	0.0 - 0.0	13.5	5.3 - 29.4	0.60
3130011	65496	APALACHICOLA R	219.6	26.9	9 - 68	0.0	0.0 - 0.0	3.6	1.0 - 8.1	0.0	0.0 - 0.1	0.1	0.0 - 0.2	0.0	0.0 - 0.0	23.2	7.6 - 58.3	1.00
3020101	65497	DEVILS CRADLE C	34.9	28.7	11 - 52	0.0	0.0 - 0.0	5.4	1.7 - 11.0	3.1	1.0 - 7.3	12.0	4.1 - 28.0	0.0	0.0 - 0.0	8.1	3.0 - 15.8	0.65
3020103	65498	*A	58.5	89.5	28 - 200	0.0	0.0 - 0.0	5.1	1.7 - 10.9	19.6	5.2 - 55.2	48.1	16.8 - 113.3	0.0	0.0 - 0.0	16.6	5.2 - 49.3	0.93
3070105	65515	TURNPIKE CR	124.6	17.6	6 - 38	0.0	0.0 - 0.0	4.4	1.4 - 9.5	0.8	0.2 - 2.2	5.9	2.1 - 17.0	0.0	0.0 - 0.0	6.6	2.1 - 15.3	0.89
3110204	65518	LITTLE R	130.0	36.0	11 - 93	0.0	0.0 - 0.0	5.1	1.5 - 12.8	2.2	0.7 - 6.2	18.0	5.6 - 53.0	0.0	0.0 - 0.0	10.7	3.4 - 26.0	0.68
3110203	65519	WITHLACOOCHEE	31.5	33.3	10 - 60	0.0	0.0 - 0.0	10.9	3.5 - 21.3	0.9	0.2 - 2.1	7.3	2.2 - 18.2	0.0	0.0 - 0.0	14.3	4.7 - 27.9	0.87
3040206	65608	WACCAMAW R	247.7	246.8	80 - 533	216.1	72.6 - 464.9	11.2	3.8 - 21.0	0.4	0.1 - 1.4	0.4	0.1 - 0.8	0.0	0.0 - 0.0	18.7	6.2 - 38.9	1.00

8-digit HUC	ID	Name	Area	Catchment Yield		Point sources		Developed Land		Manure		Agricultural Land		Phosphate Mines		Soil parent rock		Frac
				kg/km^2	90% CI	kg/km^2	90% CI	kg/km^2	90% CI	kg/km^2	90% CI	kg/km^2	90% CI	kg/km^2	90% CI	kg/km^2	90% CI	
3040207	65609	*C	1.1	2.9	1 - 8	0.0	0.0 - 0.0	0.0	0.0 - 0.0	0.0	0.0 - 0.0	0.0	0.0 - 0.0	0.0	0.0 - 0.0	2.9	0.9 - 7.7	1.00
3050202	65610	INTRACOASTAL W	41.9	7.9	2 - 22	7.9	2.2 - 21.9	0.0	0.0 - 0.0	0.0	0.0 - 0.0	0.0	0.0 - 0.0	0.0	0.0 - 0.0	0.0	0.0 - 0.5	1.00
3130008	65730	FLINT R	69.1	31.2	10 - 53	0.0	0.0 - 0.0	6.1	2.1 - 12.5	4.1	1.1 - 11.2	10.1	3.2 - 22.7	0.0	0.0 - 0.0	10.9	3.8 - 21.9	0.62
3010106	65740	ROANOKE R	529.0	60.3	21 - 120	18.0	5.9 - 37.3	6.5	2.0 - 13.8	6.5	2.0 - 17.0	19.9	6.4 - 47.8	0.0	0.0 - 0.0	9.4	2.9 - 26.6	0.97
3050208	65742	COOSAWHATCHIE	47.4	33.9	14 - 79	0.0	0.0 - 0.0	7.0	2.8 - 15.9	0.3	0.1 - 0.8	8.1	3.1 - 22.0	0.0	0.0 - 0.0	18.5	6.7 - 50.4	0.93
3080101	65743	ST JOHNS R	414.1	1.1	0 - 4	0.0	0.0 - 0.0	0.2	0.0 - 0.7	0.0	0.0 - 0.1	0.1	0.0 - 0.5	0.0	0.0 - 0.0	0.8	0.2 - 2.5	0.96
3050201	65744	COOPER RIVER W	101.8	23.2	6 - 40	0.0	0.0 - 0.0	3.9	1.2 - 7.3	0.8	0.2 - 2.0	11.6	3.5 - 25.1	0.0	0.0 - 0.0	6.9	1.9 - 16.4	0.36
3080201	65745	SPRUCE CR	61.4	55.2	16 - 113	0.0	0.0 - 0.0	36.7	11.4 - 76.8	0.4	0.1 - 1.1	2.2	0.7 - 6.3	0.0	0.0 - 0.0	15.9	5.5 - 40.4	1.00
3180004	65746	PEARL R	386.1	49.7	16 - 91	0.0	0.0 - 0.0	6.7	1.9 - 13.5	7.5	2.1 - 21.1	18.2	5.5 - 42.0	0.0	0.0 - 0.0	17.3	5.5 - 37.2	0.97
3020201	65747	ENO R	15.6	69.7	25 - 146	0.0	0.0 - 0.0	49.3	17.9 - 110.4	1.2	0.4 - 3.2	4.6	1.7 - 12.8	0.0	0.0 - 0.0	14.7	5.1 - 38.7	0.24
3020201	65749	LITTLE R	16.5	40.6	13 - 77	0.0	0.0 - 0.0	18.6	5.0 - 35.3	1.5	0.4 - 3.1	9.3	2.8 - 23.5	0.0	0.0 - 0.0	11.2	3.6 - 26.0	0.27
3020201	65750	FLAT R	10.8	23.3	8 - 46	0.0	0.0 - 0.0	3.3	1.1 - 6.2	1.8	0.5 - 4.4	7.4	2.5 - 19.9	0.0	0.0 - 0.0	10.7	3.9 - 22.4	0.29
3020203	65752	CONTENTNEA CR	52.4	29.1	9 - 86	0.0	0.0 - 0.0	4.6	1.3 - 13.0	4.0	1.2 - 15.5	14.5	4.7 - 43.8	0.0	0.0 - 0.0	5.9	1.8 - 19.4	0.81
3020203	65753	NAHUNTA SWAMI	203.1	72.6	23 - 142	0.1	0.0 - 0.2	5.9	1.8 - 11.8	34.0	9.4 - 100.3	24.4	7.9 - 51.0	0.0	0.0 - 0.0	8.2	2.6 - 20.1	0.90
3020203	65754	CONTENTNEA CR	27.1	127.0	43 - 299	8.6	3.2 - 22.7	6.7	2.1 - 15.2	59.3	18.9 - 155.2	34.9	10.3 - 94.1	0.0	0.0 - 0.0	17.4	5.9 - 51.2	0.96
3020202	65755	NEUSE R	107.4	54.9	21 - 95	0.0	0.0 - 0.0	3.9	1.3 - 7.6	11.2	3.2 - 28.5	29.7	10.4 - 62.8	0.0	0.0 - 0.0	10.1	3.5 - 22.7	0.99
3030002	65756	LANE CR	15.2	29.3	11 - 59	0.0	0.0 - 0.0	3.4	1.2 - 6.6	2.2	0.7 - 5.4	9.6	3.3 - 23.8	0.0	0.0 - 0.0	14.2	5.0 - 35.8	0.34
3030002	65757	HAW R	169.3	26.7	8 - 47	0.2	0.0 - 0.3	3.1	0.8 - 5.2	6.0	1.6 - 14.3	6.1	2.0 - 14.3	0.0	0.0 - 0.0	11.3	3.1 - 23.7	0.40
3030002	65758	MORGAN CR	23.0	32.2	11 - 65	0.0	0.0 - 0.0	5.7	1.9 - 12.0	2.5	0.8 - 5.7	10.6	3.4 - 23.9	0.0	0.0 - 0.0	13.4	4.9 - 31.4	0.34
3030003	65759	DEEP R	25.2	19.5	6 - 44	0.0	0.0 - 0.0	5.9	1.8 - 13.5	3.0	0.8 - 8.0	2.8	0.9 - 8.1	0.0	0.0 - 0.0	7.8	2.3 - 20.2	0.88
3050102	65760	INDIAN CR	23.0	46.4	15 - 87	0.0	0.0 - 0.0	9.4	2.7 - 19.4	4.3	1.4 - 11.9	19.5	6.0 - 45.3	0.0	0.0 - 0.0	13.2	4.5 - 31.6	0.19
3050109	65761	SALADA R	57.2	473.0	129 - 871	388.5	101.7 - 745.9	67.0	20.5 - 125.0	0.8	0.2 - 2.0	1.7	0.6 - 4.0	0.0	0.0 - 0.0	15.0	4.3 - 31.2	0.43
3050110	65762	CONGAREE R	3.8	85.9	25 - 203	0.0	0.0 - 0.0	79.1	23.1 - 188.7	0.0	0.0 - 0.0	0.1	0.0 - 0.4	0.0	0.0 - 0.0	6.6	2.1 - 16.4	0.43
3050204	65763	MCTIER CR	42.0	17.7	5 - 40	0.0	0.0 - 0.0	5.5	1.6 - 14.3	1.7	0.4 - 5.3	3.9	1.1 - 9.9	0.0	0.0 - 0.0	6.6	2.0 - 16.3	0.60
3060102	65764	CHATOOGA R	83.2	30.0	12 - 55	0.0	0.0 - 0.0	3.6	1.4 - 7.2	2.6	0.8 - 7.5	3.0	1.1 - 7.8	0.0	0.0 - 0.0	20.7	7.9 - 43.3	0.11
3060104	65765	BROAD R	2.4	71.4	28 - 162	0.0	0.0 - 0.0	5.6	2.1 - 13.6	16.9	5.4 - 53.4	25.1	9.9 - 74.7	0.0	0.0 - 0.0	23.7	8.4 - 65.7	0.41
3060109	65767	SAVANNAH R	22.6	26.9	8 - 54	0.0	0.0 - 0.0	3.6	1.1 - 8.1	0.5	0.2 - 1.7	6.3	2.1 - 15.8	0.0	0.0 - 0.0	16.5	4.9 - 40.0	0.98
3060202	65768	OGEECHEE R	29.7	14.9	4 - 24	0.0	0.0 - 0.0	5.4	1.4 - 9.2	0.2	0.0 - 0.4	2.2	0.6 - 5.0	0.0	0.0 - 0.0	7.1	2.0 - 12.6	0.96
3060202	65769	BLACK CR	57.2	35.3	11 - 96	0.0	0.0 - 0.0	7.1	1.8 - 19.7	1.2	0.3 - 4.0	13.4	4.0 - 38.1	0.0	0.0 - 0.0	13.6	4.3 - 36.5	0.93
3070103	65770	YELLOW R	93.4	105.2	34 - 186	12.4	3.7 - 24.4	69.3	22.5 - 130.8	0.2	0.1 - 0.7	1.9	0.6 - 3.9	0.0	0.0 - 0.0	21.3	7.6 - 41.9	0.50
3070103	65771	YELLOW R	169.5	54.7	18 - 136	5.0	1.6 - 14.8	32.6	10.5 - 73.2	0.5	0.1 - 1.5	4.4	1.3 - 15.1	0.0	0.0 - 0.0	12.3	3.6 - 35.4	0.53
3070103	65772	BIG HAYNES CR	45.5	74.3	22 - 178	0.0	0.0 - 0.0	50.5	14.9 - 127.0	0.8	0.2 - 2.6	6.7	1.9 - 20.1	0.0	0.0 - 0.0	16.3	4.7 - 42.3	0.44
3070103	65773	BRUSHY FK CR	20.9	63.5	22 - 153	0.0	0.0 - 0.0	35.5	12.3 - 73.5	1.4	0.4 - 3.8	11.0	3.4 - 34.1	0.0	0.0 - 0.0	15.7	5.8 - 35.2	0.42
3070103	65774	OCMULGEE R	141.6	35.5	12 - 68	0.0	0.0 - 0.0	17.2	6.1 - 36.9	1.4	0.4 - 3.5	2.2	0.7 - 5.4	0.0	0.0 - 0.0	14.8	5.3 - 33.9	0.85
3070104	65775	BIG CR	67.4	23.5	7 - 59	0.0	0.0 - 0.0	3.3	1.0 - 8.3	0.5	0.1 - 1.4	13.3	4.1 - 44.1	0.0	0.0 - 0.0	6.5	2.4 - 17.1	0.88
3070104	65776	OCMULGEE R	184.9	19.0	7 - 40	0.0	0.0 - 0.0	3.4	1.1 - 7.2	0.6	0.2 - 1.8	6.0	2.0 - 15.1	0.0	0.0 - 0.0	9.0	2.8 - 22.9	0.96
3070101	65777	LITTLE R	4.8	28.2	8 - 59	0.0	0.0 - 0.0	8.5	2.5 - 18.0	2.1	0.5 - 6.9	3.1	0.9 - 7.0	0.0	0.0 - 0.0	14.5	4.1 - 38.6	0.52
3070101	65778	MURDER CR	82.1	20.9	7 - 40	0.0	0.0 - 0.0	4.4	1.4 - 8.8	1.7	0.5 - 5.6	2.5	0.9 - 6.0	0.0	0.0 - 0.0	12.3	4.2 - 26.5	0.53
3070102	65779	OCONEE R	4.2	15.7	6 - 30	0.0	0.0 - 0.0	2.7	1.1 - 5.9	0.4	0.1 - 1.2	2.0	0.7 - 4.9	0.0	0.0 - 0.0	10.6	4.1 - 23.4	0.92

8-digit HUC	ID	Name	Area	Catchment Yield		Point sources		Developed Land		Manure		Agricultural Land		Phosphate Mines		Soil parent rock		Frac
				kg/km^2	90% CI	kg/km^2	90% CI	kg/km^2	90% CI	kg/km^2	90% CI	kg/km^2	90% CI	kg/km^2	90% CI	kg/km^2	90% CI	
3100101	65784	PEACE R	42.4	1861.9	662 - 4656	0.0	0.0 - 0.0	25.9	8.6 - 48.3	7.7	1.7 - 29.6	8.4	2.7 - 21.2	1759.4	623.9 - 4930	60.4	20.3 - 179.9	0.79
3100101	65785	PEACE R	67.7	105.2	39 - 209	0.0	0.0 - 0.0	16.1	5.5 - 35.2	14.9	4.9 - 38.9	35.7	12.4 - 89.6	0.0	0.0 - 0.0	38.5	14.3 - 87.4	0.92
3100101	65786	CHARLIE CR	96.4	128.4	44 - 319	0.0	0.0 - 0.0	2.2	0.8 - 5.0	24.0	6.5 - 73.4	60.1	20.8 - 157.6	0.0	0.0 - 0.0	42.1	14.4 - 116.5	0.94
3100101	65787	PEACE R	140.6	149.8	52 - 351	0.0	0.0 - 0.0	7.7	2.6 - 17.7	12.6	4.0 - 36.9	42.0	14.2 - 119.6	46.4	14.7 - 144.5	41.1	14.5 - 110.4	0.98
3100101	65788	JOSHUA CR	169.9	115.2	33 - 254	0.0	0.0 - 0.0	8.7	2.6 - 19.4	16.0	4.4 - 44.3	55.5	17.4 - 133.1	3.1	0.9 - 8.0	31.9	7.8 - 79.7	0.97
3100101	65789	HORSE CR	146.4	1026.7	348 - 2596	0.0	0.0 - 0.0	2.7	1.0 - 5.4	10.1	3.3 - 28.7	24.7	9.0 - 57.2	946.4	329.4 - 2759	42.8	17.2 - 94.4	0.82
3100101	65790	HORSE CR	236.1	96.5	30 - 269	0.0	0.0 - 0.0	4.5	1.3 - 11.2	12.9	3.0 - 42.9	40.1	13.4 - 107.3	0.0	0.0 - 0.0	38.8	11.6 - 120.4	0.95
3100101	65791	PRAIRIE CR	705.6	55.2	17 - 112	0.0	0.0 - 0.0	0.7	0.2 - 1.4	9.1	2.3 - 22.7	32.1	9.8 - 71.5	0.1	0.0 - 0.3	13.2	3.8 - 34.2	0.94
3100102	65793	MYAKKA R	265.7	61.4	21 - 111	0.0	0.0 - 0.0	5.6	1.8 - 10.7	7.1	2.1 - 18.1	19.7	6.9 - 46.2	0.0	0.0 - 0.0	29.0	9.3 - 56.3	0.93
3100202	65794	MANATEE R	17.9	72.4	23 - 139	0.0	0.0 - 0.0	0.9	0.3 - 1.8	8.0	2.3 - 18.0	25.3	8.4 - 53.9	0.0	0.0 - 0.0	38.2	11.6 - 89.6	0.23
3100203	65795	LITTLE MANATEE	136.7	121.5	44 - 297	43.8	14.8 - 97.6	3.4	1.1 - 7.0	10.9	3.0 - 28.4	15.8	5.0 - 41.2	0.0	0.0 - 0.0	47.6	16.4 - 120.4	0.84
3100203	65796	LITTLE MANATEE	41.3	97.4	34 - 246	0.0	0.0 - 0.0	19.6	6.7 - 45.2	10.9	3.0 - 30.8	23.3	8.3 - 63.6	0.0	0.0 - 0.0	43.6	13.9 - 124.8	0.94
3100206	65797	BULLFROG CR	81.5	75.6	29 - 161	0.0	0.0 - 0.0	9.6	3.1 - 20.6	11.6	3.8 - 38.0	26.4	9.3 - 62.2	0.0	0.0 - 0.0	28.1	9.7 - 69.3	0.98
3100204	65798	ALAFIA R, N PRON	18.1	322.4	125 - 749	0.0	0.0 - 0.0	18.0	6.9 - 48.5	6.8	2.0 - 24.5	14.2	5.5 - 40.6	248.1	97.2 - 723.2	35.3	13.1 - 99.0	0.91
3100204	65799	ALAFIA R, S PRON	276.7	532.1	183 - 1033	287.1	100.0 - 627.7	2.6	0.8 - 4.8	7.6	2.4 - 21.4	4.6	1.5 - 10.7	190.5	66.4 - 574.2	39.7	13.2 - 87.4	0.90
3100205	65801	BLACKWATER CR	12.2	381.5	117 - 974	0.0	0.0 - 0.0	1.7	0.6 - 4.8	21.4	5.0 - 71.8	49.0	13.6 - 148.8	251.1	81.0 - 793.7	58.3	17.0 - 155.7	0.85
3100205	65802	HILLSBOROUGH R	5.3	65.2	19 - 173	0.0	0.0 - 0.0	2.7	1.0 - 5.7	6.7	1.5 - 22.6	14.7	4.5 - 48.9	0.0	0.0 - 0.0	41.2	12.2 - 125.7	0.89
3100205	65803	HILLSBOROUGH R	24.2	71.9	22 - 159	0.0	0.0 - 0.0	18.9	6.3 - 42.6	4.0	1.1 - 12.0	9.0	3.1 - 28.1	0.0	0.0 - 0.0	40.0	12.9 - 104.5	0.94
3100205	65804	TROUT CR	365.9	82.4	30 - 153	0.2	0.1 - 0.4	16.5	5.4 - 29.7	10.1	3.1 - 25.9	23.1	8.7 - 51.9	0.0	0.0 - 0.0	32.6	11.5 - 70.9	0.92
3100207	65806	ANCLOTE R	267.7	75.3	29 - 172	0.0	0.0 - 0.0	19.6	7.2 - 42.0	6.8	2.6 - 19.4	15.1	5.3 - 40.4	0.0	0.0 - 0.0	33.8	12.8 - 86.4	0.98
3100208	65807	WITHLACOOCHEE	792.2	41.9	14 - 79	0.0	0.0 - 0.0	5.8	2.1 - 11.5	2.2	0.7 - 6.0	12.8	4.3 - 28.8	0.0	0.0 - 0.0	21.1	7.7 - 46.6	0.07
3110203	65809	WITHLACOOCHEE	120.2	129.3	52 - 333	91.4	37.9 - 227.1	7.0	2.7 - 17.4	1.9	0.7 - 5.5	12.6	4.1 - 38.1	0.0	0.0 - 0.0	16.4	6.5 - 43.0	0.87
3110203	65810	WITHLACOOCHEE	93.8	36.0	10 - 65	0.0	0.0 - 0.0	5.3	1.6 - 10.9	1.8	0.5 - 3.9	6.6	1.7 - 14.0	0.0	0.0 - 0.0	22.3	6.3 - 48.0	0.89
3120003	65811	OCHOCKONEE R	4.3	30.3	11 - 60	0.0	0.0 - 0.0	2.8	1.0 - 5.4	1.1	0.3 - 2.7	3.5	1.2 - 8.1	0.0	0.0 - 0.0	22.9	8.2 - 45.2	0.58
3130001	65812	CHATTAHOOCHEE	130.3	121.9	44 - 214	26.2	9.6 - 52.5	26.9	9.1 - 52.7	42.1	12.7 - 104.5	15.0	5.0 - 35.4	0.0	0.0 - 0.0	11.7	4.0 - 25.0	0.04
3130001	65813	LITTLE R, W FK	47.8	111.2	38 - 263	0.0	0.0 - 0.0	13.0	4.3 - 27.4	51.4	16.0 - 158.7	23.0	7.8 - 65.1	0.0	0.0 - 0.0	23.7	8.6 - 61.3	0.04
3130001	65814	SWANNEE CR	23.7	77.7	24 - 162	0.0	0.0 - 0.0	65.1	19.1 - 137.2	0.2	0.0 - 0.4	1.0	0.3 - 3.5	0.0	0.0 - 0.0	11.4	3.1 - 32.1	0.15
3130001	65815	PEACHTREE CR, S	39.6	161.0	55 - 383	0.0	0.0 - 0.0	113.9	39.7 - 260.0	0.0	0.0 - 0.1	0.3	0.1 - 0.9	0.0	0.0 - 0.0	46.7	14.7 - 112.0	0.17
3130002	65816	SWEETWATER CR	48.0	127.4	41 - 242	0.0	0.0 - 0.0	86.7	26.6 - 178.1	0.4	0.1 - 0.8	3.3	1.0 - 7.4	0.0	0.0 - 0.0	37.1	12.6 - 74.9	0.17
3130002	65817	CHATTAHOOCHEE	93.1	38.5	14 - 93	0.0	0.0 - 0.0	17.8	5.7 - 40.0	1.0	0.3 - 3.0	5.5	1.8 - 14.5	0.0	0.0 - 0.0	14.2	5.3 - 37.4	0.17
3130002	65818	SNAKE CR	92.6	35.4	11 - 76	0.0	0.0 - 0.0	9.1	2.8 - 22.0	8.5	2.6 - 24.5	7.9	2.2 - 23.2	0.0	0.0 - 0.0	9.9	2.9 - 23.7	0.17
3130002	65819	CHATTAHOOCHEE	9.9	43.4	16 - 82	0.0	0.0 - 0.0	9.6	3.3 - 20.1	5.6	2.0 - 15.0	14.5	5.1 - 41.4	0.0	0.0 - 0.0	13.7	4.8 - 30.1	0.18
3130002	65820	NEW R	73.2	21.1	6 - 39	0.0	0.0 - 0.0	3.3	1.0 - 7.5	1.7	0.5 - 5.5	5.1	1.5 - 11.9	0.0	0.0 - 0.0	11.0	3.1 - 26.3	0.18
3130003	65821	CHATTAHOOCHEE	10.1	2164.2	757 - 4548	2144.3	748.6 - 4511.3	2.7	0.9 - 5.4	0.3	0.1 - 0.8	3.8	1.3 - 10.7	0.0	0.0 - 0.0	13.0	4.1 - 28.6	0.36
3130004	65822	CHATTAHOOCHEE	148.4	43.8	16 - 88	0.0	0.0 - 0.0	6.4	2.3 - 11.9	3.0	0.9 - 9.2	23.5	8.0 - 58.6	0.0	0.0 - 0.0	10.8	3.4 - 26.3	0.63
3130005	65823	FLINT R	401.8	46.8	17 - 104	1.3	0.5 - 3.0	19.4	6.5 - 46.6	1.3	0.4 - 3.6	9.4	3.1 - 24.1	0.0	0.0 - 0.0	15.4	5.1 - 36.9	0.42
3130005	65824	FLINT R	41.6	28.0	12 - 64	0.0	0.0 - 0.0	5.5	2.4 - 13.4	2.8	0.9 - 8.1	6.4	2.4 - 18.2	0.0	0.0 - 0.0	13.2	5.1 - 35.7	0.46
3130006	65825	FLINT R	1.8	44.2	19 - 91	0.0	0.0 - 0.0	26.2	11.3 - 59.1	2.6	1.0 - 7.1	5.5	1.9 - 14.0	0.0	0.0 - 0.0	10.0	4.2 - 24.7	0.48
3130006	65826	LIME CR	160.5	30.3	11 - 78	0.0	0.0 - 0.0	4.3	1.5 - 9.8	2.3	0.7 - 6.3	15.8	5.1 - 43.9	0.0	0.0 - 0.0	7.9	3.0 - 23.3	0.46

8-digit HUC	ID	Name	Area	Catchment Yield		Point sources		Developed Land		Manure		Agricultural Land		Phosphate Mines		Soil parent rock		Frac
				kg/km^2	90% CI	kg/km^2	90% CI	kg/km^2	90% CI	kg/km^2	90% CI	kg/km^2	90% CI	kg/km^2	90% CI	kg/km^2	90% CI	
3130007	65827	MUCKALEE CR	48.4	40.7	14 - 101	0.0	0.0 - 0.0	5.3	1.8 - 12.7	1.9	0.6 - 5.4	22.3	7.0 - 60.3	0.0	0.0 - 0.0	11.1	3.7 - 27.3	0.58
3130008	65828	FLINT R	17.1	99.3	37 - 205	0.0	0.0 - 0.0	88.1	32.6 - 176.8	0.1	0.0 - 0.3	1.1	0.4 - 2.5	0.0	0.0 - 0.0	10.0	3.5 - 22.5	0.61
3130008	65829	FLINT R	1.8	53.1	20 - 105	0.0	0.0 - 0.0	28.4	10.2 - 58.0	2.1	0.7 - 5.2	11.7	3.8 - 29.5	0.0	0.0 - 0.0	10.9	3.9 - 23.5	0.62
3130008	65830	FLINT R	320.2	40.6	12 - 94	0.0	0.0 - 0.0	9.3	3.0 - 21.2	2.1	0.6 - 7.1	15.2	4.4 - 37.8	0.0	0.0 - 0.0	13.9	4.2 - 37.3	0.63
3150104	65831	ETOWAH R	39.3	58.7	20 - 133	0.0	0.0 - 0.0	10.3	3.2 - 24.0	12.8	4.0 - 43.4	21.7	6.5 - 58.9	0.0	0.0 - 0.0	13.9	4.7 - 41.7	0.32
3150105	65832	COOSA R	10.5	101.4	39 - 172	0.0	0.0 - 0.0	6.0	2.2 - 10.7	15.1	4.8 - 45.8	42.4	14.4 - 90.6	0.0	0.0 - 0.0	37.8	12.9 - 85.2	0.33
3150105	65833	CHATOOGA R	92.1	138.7	39 - 283	84.2	24.2 - 181.8	21.0	6.2 - 45.9	3.8	1.0 - 10.8	12.9	3.7 - 32.0	0.0	0.0 - 0.0	16.9	4.8 - 39.2	0.31
3150105	65834	CHATOOGA R	1.8	49.2	17 - 94	0.0	0.0 - 0.0	7.2	2.3 - 13.7	4.1	1.3 - 11.2	19.3	6.9 - 46.9	0.0	0.0 - 0.0	18.6	6.6 - 41.0	0.33
3150201	65835	*C	34.3	127.1	40 - 286	0.0	0.0 - 0.0	110.5	36.5 - 253.0	0.4	0.1 - 1.1	1.6	0.6 - 5.2	0.0	0.0 - 0.0	14.6	5.0 - 35.5	0.69
3150201	65836	PINTALLA CR	150.8	39.1	15 - 101	0.0	0.0 - 0.0	3.5	1.2 - 8.2	4.0	1.2 - 12.0	12.0	3.8 - 32.8	0.0	0.0 - 0.0	19.6	7.0 - 54.7	0.69
3150202	65837	CAHABA R	118.1	43.0	13 - 75	4.9	1.6 - 9.0	27.7	7.7 - 49.4	0.4	0.1 - 1.0	3.3	1.0 - 8.3	0.0	0.0 - 0.0	6.8	2.1 - 13.7	0.68
3150202	65838	*A	65.6	56.2	16 - 94	0.0	0.0 - 0.0	40.7	10.9 - 72.3	0.5	0.1 - 1.0	4.6	1.2 - 10.3	0.0	0.0 - 0.0	10.5	3.1 - 19.7	0.69
3150202	65839	CAHABA R	37.5	36.3	12 - 81	0.0	0.0 - 0.0	14.8	4.5 - 28.5	1.3	0.4 - 3.9	8.8	2.8 - 21.1	0.0	0.0 - 0.0	11.4	3.9 - 31.5	0.80
3160106	65840	BOGUE CHITTO CI	137.1	199.8	66 - 395	0.0	0.0 - 0.0	2.9	0.9 - 5.3	21.3	6.0 - 63.8	104.2	32.3 - 244.3	0.0	0.0 - 0.0	71.4	24.5 - 182.4	0.86
3160111	65841	BLACK WARRIOR	4.7	61.7	21 - 122	0.0	0.0 - 0.0	13.2	4.3 - 29.4	18.4	6.0 - 42.5	23.7	8.0 - 56.7	0.0	0.0 - 0.0	6.4	2.2 - 14.2	0.52
3160204	65842	THREEMILE CR	25.5	15.5	4 - 49	0.0	0.0 - 0.0	11.6	2.6 - 38.0	0.1	0.0 - 0.3	0.3	0.1 - 1.2	0.0	0.0 - 0.0	3.4	0.8 - 10.2	0.89
6010105	65843	FRENCH BROAD R	76.8	32.4	12 - 78	0.0	0.0 - 0.0	5.7	2.0 - 13.0	5.1	1.5 - 16.9	12.8	4.2 - 37.6	0.0	0.0 - 0.0	8.9	3.3 - 23.2	0.15
6010108	65844	NOLICHUCKY R	20.3	77.3	25 - 167	0.0	0.0 - 0.0	4.6	1.5 - 11.1	17.8	4.1 - 57.8	42.6	14.5 - 115.8	0.0	0.0 - 0.0	12.4	4.0 - 33.2	0.15
6010102	65845	HOLSTON R, MIDE	284.8	48.5	17 - 97	2.3	0.7 - 4.6	7.8	2.5 - 15.2	8.8	2.5 - 19.3	21.2	6.5 - 50.8	0.0	0.0 - 0.0	8.4	2.8 - 17.1	0.03
6010205	65846	CLINCH R	8.8	20.0	5 - 42	0.0	0.0 - 0.0	3.8	1.1 - 7.4	2.6	0.6 - 7.4	2.6	0.8 - 5.9	0.0	0.0 - 0.0	11.0	3.1 - 24.5	0.11
6010208	65847	CLEAR CR	31.4	29.4	9 - 62	0.0	0.0 - 0.0	8.6	2.8 - 16.6	2.9	0.8 - 8.0	4.8	1.4 - 11.7	0.0	0.0 - 0.0	13.1	3.9 - 30.7	0.28
6020001	65848	TENNESSEE R	2.9	97.6	36 - 186	0.0	0.0 - 0.0	65.0	23.8 - 128.0	4.1	1.3 - 10.5	12.1	3.7 - 28.2	0.0	0.0 - 0.0	16.4	5.9 - 37.5	0.43
6030001	65849	WHIPPOORWILL C	142.8	54.8	19 - 126	0.0	0.0 - 0.0	9.5	3.1 - 21.2	21.1	7.6 - 59.7	19.7	6.1 - 52.7	0.0	0.0 - 0.0	4.4	1.8 - 10.7	0.43
6030002	65850	FLINT R	79.6	114.9	39 - 286	14.9	5.3 - 38.7	27.7	8.5 - 65.0	5.5	1.6 - 15.8	46.1	13.4 - 140.9	0.0	0.0 - 0.0	20.6	7.1 - 53.1	0.52
6030003	65851	CANE CR	41.1	771.9	255 - 1669	0.0	0.0 - 0.0	7.3	2.0 - 17.4	16.5	4.8 - 46.8	44.1	12.5 - 127.6	0.0	0.0 - 0.0	704.0	240.9 - 1722	0.49
6040002	65852	NORTH FK CR	1.8	261.6	88 - 449	0.0	0.0 - 0.0	11.4	3.8 - 24.3	35.9	9.4 - 93.6	70.0	22.1 - 145.5	0.0	0.0 - 0.0	144.2	46.2 - 315.8	0.54
3110202	65895	ALPAHA R	439.6	37.9	11 - 95	0.9	0.3 - 2.6	6.5	1.8 - 14.1	1.2	0.3 - 3.4	5.5	1.5 - 17.5	0.8	0.2 - 2.4	22.9	7.4 - 54.4	0.94
3030003	65973	HICKORY CR	19.2	31.3	9 - 64	0.0	0.0 - 0.0	24.4	7.4 - 48.6	0.3	0.1 - 0.7	1.5	0.4 - 3.3	0.0	0.0 - 0.0	5.1	1.5 - 12.6	0.61
6010105	65992	FRENCH BROAD R	28.7	55.5	18 - 115	0.0	0.0 - 0.0	46.4	15.4 - 94.8	0.4	0.1 - 1.2	1.7	0.5 - 4.3	0.0	0.0 - 0.0	6.9	2.1 - 14.6	0.14
3020103	66016	TAR R	230.9	33.2	10 - 74	0.0	0.0 - 0.0	4.3	1.1 - 9.2	6.1	1.7 - 16.5	15.4	4.6 - 40.2	0.0	0.0 - 0.0	7.4	2.1 - 16.2	0.97
3050203	66050	EDISTO R, N FK	1.5	23.2	8 - 51	0.0	0.0 - 0.0	8.2	2.7 - 18.1	1.3	0.4 - 3.8	6.0	2.0 - 16.5	0.0	0.0 - 0.0	7.7	2.5 - 20.0	0.82
3050109	66079	SALADA R	1.1	40.0	13 - 76	0.0	0.0 - 0.0	14.0	4.7 - 28.1	5.7	1.6 - 17.7	7.0	2.2 - 16.9	0.0	0.0 - 0.0	13.5	4.3 - 30.8	0.12
3060101	66084	TWELVEMILE CR	2.1	64.9	24 - 126	0.0	0.0 - 0.0	42.8	14.7 - 87.5	2.9	1.0 - 8.7	10.0	3.8 - 24.3	0.0	0.0 - 0.0	9.1	3.5 - 21.2	0.13
3020202	66111	BEAR CR	163.2	83.7	29 - 169	0.0	0.0 - 0.0	7.9	2.5 - 18.0	36.5	10.7 - 87.3	26.7	9.2 - 74.5	0.0	0.0 - 0.0	12.7	4.8 - 30.2	0.93
3050103	66115	FISHING CR	67.7	26.2	9 - 49	0.0	0.0 - 0.0	5.5	1.5 - 10.2	1.6	0.5 - 4.0	6.1	2.0 - 12.6	0.0	0.0 - 0.0	13.0	4.3 - 27.6	0.29
3020203	66119	LITTLE CONTENTI	398.7	63.7	20 - 136	4.3	1.3 - 10.2	6.4	1.8 - 14.6	19.2	5.0 - 50.0	21.8	7.1 - 55.3	0.0	0.0 - 0.0	12.1	3.3 - 29.9	0.92
3050110	66120	GILLS CR	28.8	77.3	21 - 179	0.0	0.0 - 0.0	67.5	17.9 - 158.4	0.0	0.0 - 0.2	0.6	0.2 - 1.5	0.0	0.0 - 0.0	9.1	3.0 - 27.2	0.40
3050206	66121	COW CASTLE CR	79.8	36.3	11 - 69	0.0	0.0 - 0.0	7.4	2.1 - 15.1	3.5	1.0 - 9.2	19.5	5.9 - 44.9	0.0	0.0 - 0.0	6.0	2.0 - 14.0	0.72
3070106	66122	ALTAMAHA R	45.9	1195.4	406 - 3124	1193.0	405.5 - 3119.1	1.6	0.4 - 4.0	0.0	0.0 - 0.1	0.0	0.0 - 0.1	0.0	0.0 - 0.0	0.8	0.2 - 1.7	0.99

8-digit HUC	ID	Name	Area	Catchment Yield		Point sources		Developed Land		Manure		Agricultural Land		Phosphate Mines		Soil parent rock		Frac
				kg/km^2	90% CI	kg/km^2	90% CI	kg/km^2	90% CI	kg/km^2	90% CI	kg/km^2	90% CI	kg/km^2	90% CI	kg/km^2	90% CI	
3100101	66127	SHELL CR	6.0	80.1	26 - 162	0.0	0.0 - 0.0	25.2	8.8 - 59.1	6.5	1.8 - 22.0	26.0	9.1 - 75.3	0.0	0.0 - 0.0	22.4	7.5 - 56.1	0.99
3170008	66128	BIG CR	70.8	37.3	14 - 83	0.0	0.0 - 0.0	5.5	1.9 - 12.1	2.9	1.1 - 8.1	14.7	5.5 - 33.9	0.0	0.0 - 0.0	14.2	5.1 - 34.0	0.22
3150203	66129	ALABAMA R	251.9	51.9	17 - 129	0.0	0.0 - 0.1	4.1	1.4 - 10.9	2.9	0.9 - 7.6	9.4	3.0 - 25.3	0.0	0.0 - 0.0	35.5	13.3 - 85.4	0.87
3150106	66130	BIG CANOE CR	128.1	52.3	17 - 90	0.3	0.1 - 0.5	9.1	3.0 - 18.2	9.4	2.9 - 22.3	19.1	6.0 - 44.4	0.0	0.0 - 0.0	14.3	4.7 - 31.4	0.47
3150106	66131	BIG WILLIS CR	18.3	25.5	9 - 50	0.0	0.0 - 0.0	6.0	2.0 - 12.6	3.6	1.0 - 8.7	7.4	2.3 - 18.4	0.0	0.0 - 0.0	8.5	3.0 - 19.0	0.47
3160109	66132	BLACKWATER CR	241.4	32.0	12 - 59	0.0	0.0 - 0.0	6.5	2.2 - 13.1	6.7	2.2 - 16.5	11.9	3.7 - 26.6	0.0	0.0 - 0.0	6.9	2.3 - 15.9	0.54
3160113	66133	BLACK WARRIOR	44.0	36.2	14 - 75	0.0	0.0 - 0.0	4.8	1.8 - 10.6	2.0	0.5 - 6.9	10.2	3.8 - 24.7	0.0	0.0 - 0.0	19.2	6.9 - 44.5	0.82
3150202	66134	CAHABA R	9.3	12.8	5 - 28	0.0	0.0 - 0.0	3.8	1.4 - 7.6	0.3	0.1 - 0.9	2.6	1.0 - 7.4	0.0	0.0 - 0.0	6.0	2.4 - 14.7	0.74
3150202	66135	CAHABA R	1.1	18.2	7 - 39	0.0	0.0 - 0.0	0.0	0.0 - 0.0	0.3	0.1 - 0.9	2.7	0.8 - 7.4	0.0	0.0 - 0.0	15.2	6.1 - 38.2	0.81
3150201	66136	CATOMA CR	73.4	140.6	43 - 286	0.0	0.0 - 0.0	45.6	14.4 - 95.0	6.7	1.5 - 19.6	24.7	6.5 - 58.3	0.0	0.0 - 0.0	63.6	18.5 - 147.9	0.77
3150202	66137	CAHABA R	54.9	122.1	40 - 261	0.0	0.0 - 0.0	4.4	1.5 - 8.5	9.5	3.0 - 26.2	61.0	17.9 - 146.0	0.0	0.0 - 0.0	47.1	17.0 - 93.1	0.85
3130003	66138	CHATTAHOOCHEE	17.7	94.8	35 - 209	0.0	0.0 - 0.0	80.7	29.0 - 195.0	0.1	0.0 - 0.4	0.7	0.2 - 1.9	0.0	0.0 - 0.0	13.3	5.2 - 32.1	0.35
3150105	66139	COOSA R	66.8	33.4	11 - 77	0.0	0.0 - 0.0	10.8	3.8 - 26.8	2.5	0.8 - 7.7	10.5	3.5 - 28.1	0.0	0.0 - 0.0	9.6	3.6 - 25.1	0.49
3150107	66141	COOSA R	128.7	33.3	13 - 59	0.0	0.0 - 0.0	11.2	3.8 - 21.6	1.6	0.5 - 4.1	10.4	3.8 - 25.8	0.0	0.0 - 0.0	10.2	3.7 - 20.3	0.79
3160113	66142	ELLIOTTS CR	83.6	11.6	4 - 25	0.0	0.0 - 0.0	3.5	1.1 - 7.0	0.7	0.2 - 1.9	3.0	1.0 - 7.1	0.0	0.0 - 0.0	4.5	1.5 - 10.3	0.75
3160111	66143	FIVEMILE CR	104.4	42.6	12 - 76	0.1	0.0 - 0.1	29.5	8.7 - 58.9	0.7	0.2 - 1.6	3.8	1.2 - 8.6	0.0	0.0 - 0.0	8.5	2.9 - 18.9	0.57
3160111	66144	FIVEMILE CR	157.2	246.3	97 - 553	166.9	61.9 - 390.2	63.8	25.2 - 127.6	0.3	0.1 - 0.8	2.0	0.8 - 4.4	0.0	0.0 - 0.0	13.3	4.7 - 31.8	0.54
3150107	66145	HATCHET CR	90.0	16.4	5 - 30	0.0	0.0 - 0.0	6.8	2.2 - 11.6	0.5	0.1 - 1.2	0.9	0.3 - 2.1	0.0	0.0 - 0.0	8.2	2.3 - 18.0	0.70
3150106	66146	KELLEY CR	279.0	25.6	9 - 44	0.0	0.0 - 0.0	6.7	2.1 - 12.5	2.7	0.8 - 6.7	7.4	2.4 - 15.4	0.0	0.0 - 0.0	8.8	3.2 - 16.5	0.65
3160111	66147	BLACK WARRIOR	37.0	13.0	4 - 28	0.0	0.0 - 0.0	3.4	1.1 - 7.3	0.5	0.1 - 1.3	0.5	0.2 - 1.3	0.0	0.0 - 0.0	8.6	2.5 - 19.9	0.59
3160111	66148	BLACK WARRIOR	99.9	25.6	8 - 49	0.0	0.0 - 0.0	6.5	2.1 - 14.3	1.6	0.5 - 3.8	11.5	3.4 - 30.5	0.0	0.0 - 0.0	5.9	1.7 - 12.0	0.58
3150108	66149	LITTLE TALLAPOO	5.5	93.0	23 - 206	0.0	0.0 - 0.0	8.5	2.2 - 19.2	29.8	6.3 - 102.3	41.4	11.0 - 95.9	0.0	0.0 - 0.0	13.3	3.4 - 33.6	0.30
3150105	66150	LITTLE R	23.8	25.4	9 - 61	0.0	0.0 - 0.0	6.9	2.5 - 16.9	1.8	0.5 - 6.0	6.7	2.0 - 20.3	0.0	0.0 - 0.0	9.9	3.3 - 24.7	0.33
3150201	66151	MULBERRY CR	140.2	19.2	7 - 41	0.0	0.0 - 0.0	4.9	1.6 - 10.8	1.3	0.4 - 4.4	8.0	2.6 - 20.0	0.0	0.0 - 0.0	5.0	1.7 - 13.2	0.83
3150203	66152	PINE BARRON CR	58.9	32.2	9 - 72	0.0	0.0 - 0.0	4.2	1.1 - 9.3	2.6	0.6 - 7.6	8.6	2.4 - 24.1	0.0	0.0 - 0.0	16.8	4.4 - 37.9	0.78
3130003	66153	COWIKEE CR, S FK	306.5	15.3	7 - 40	0.0	0.0 - 0.0	4.4	1.8 - 12.2	1.1	0.4 - 3.8	3.1	1.3 - 9.9	0.0	0.0 - 0.0	6.7	2.9 - 20.5	0.35
3150108	66154	TALLAPOOSA R	225.3	37.5	12 - 80	1.9	0.6 - 4.0	10.5	3.3 - 23.2	5.8	1.6 - 16.0	9.9	2.9 - 20.9	0.0	0.0 - 0.0	9.4	3.3 - 20.6	0.29
3100204	66155	ALAFIA R	6.8	75.4	26 - 162	0.0	0.0 - 0.0	51.9	16.7 - 116.9	0.5	0.1 - 1.3	1.2	0.4 - 2.8	0.0	0.0 - 0.0	21.8	6.8 - 57.6	0.97
3100205	66156	HILLSBOROUGH R	219.3	56.4	19 - 127	0.0	0.0 - 0.0	41.9	14.7 - 106.6	0.3	0.1 - 1.1	0.6	0.2 - 1.7	0.0	0.0 - 0.0	13.6	5.0 - 35.6	0.99
3100204	66157	ALAFIA R	82.4	94.1	33 - 167	0.5	0.2 - 1.1	39.3	12.4 - 92.8	5.1	1.6 - 14.2	10.9	3.9 - 28.1	0.0	0.0 - 0.0	38.3	13.5 - 83.2	0.98
3080102	66159	OKLAWAHA R	124.4	8.1	3 - 18	0.0	0.0 - 0.0	1.0	0.3 - 2.2	0.2	0.1 - 0.7	0.2	0.1 - 0.5	0.0	0.0 - 0.0	6.6	2.1 - 15.3	0.64
3140106	66160	PERDIDO R	26.4	26.8	10 - 56	0.0	0.0 - 0.0	7.5	2.5 - 15.6	1.1	0.3 - 3.0	4.2	1.6 - 10.0	0.0	0.0 - 0.0	13.9	5.0 - 34.3	0.96
3140104	66161	BIG COLDWATER	81.8	27.2	10 - 53	0.0	0.0 - 0.0	9.5	3.4 - 18.3	0.5	0.2 - 1.5	6.3	2.1 - 14.9	0.0	0.0 - 0.0	10.8	3.5 - 27.7	0.98
3140103	66162	YELLOW R	512.1	32.3	11 - 67	0.0	0.0 - 0.0	9.2	2.8 - 19.8	2.6	0.8 - 6.7	10.0	3.0 - 28.6	0.0	0.0 - 0.0	10.6	3.6 - 23.2	0.96
3100102	66163	MYAKKA R	129.2	79.3	32 - 189	0.0	0.0 - 0.0	21.0	7.6 - 54.9	4.2	1.4 - 11.2	8.9	3.3 - 25.3	0.0	0.0 - 0.0	45.2	18.4 - 118.0	0.97
3100202	66164	MANATEE R	137.0	95.0	28 - 225	0.0	0.0 - 0.0	5.7	1.9 - 11.6	11.8	3.1 - 39.8	37.9	12.0 - 101.7	0.0	0.0 - 0.0	39.7	12.0 - 117.0	0.23
3100208	66165	WITHLACOOCHEE	154.1	26.1	8 - 58	0.0	0.0 - 0.0	12.7	4.3 - 28.9	0.6	0.1 - 1.6	2.4	0.8 - 7.1	0.4	0.1 - 1.0	10.0	3.2 - 28.0	0.09
3120003	66166	OCHOCKONEE R	130.5	34.3	15 - 76	0.0	0.0 - 0.0	4.7	1.7 - 10.4	0.2	0.1 - 0.6	1.3	0.5 - 3.3	0.0	0.0 - 0.0	28.1	12.6 - 76.7	0.97
3140102	66167	ALAQUA CR	100.8	21.8	9 - 45	0.0	0.0 - 0.0	13.1	5.0 - 30.3	0.5	0.2 - 1.3	0.6	0.2 - 1.6	0.0	0.0 - 0.0	7.6	3.1 - 16.2	0.92

8-digit HUC	ID	Name	Area	Catchment Yield		Point sources		Developed Land		Manure		Agricultural Land		Phosphate Mines		Soil parent rock		Frac
				kg/km^2	90% CI	kg/km^2	90% CI	kg/km^2	90% CI	kg/km^2	90% CI	kg/km^2	90% CI	kg/km^2	90% CI	kg/km^2	90% CI	
3130011	66168	APALACHICOLA R	1.6	21.8	6 - 39	0.0	0.0 - 0.0	8.4	2.6 - 17.8	0.4	0.1 - 1.0	2.5	0.7 - 5.5	0.0	0.0 - 0.0	10.4	3.0 - 24.2	0.98
3140104	66169	BLACKWATER R	8.0	39.2	14 - 79	0.0	0.0 - 0.0	9.8	3.6 - 21.5	1.6	0.5 - 5.6	15.8	5.2 - 41.4	0.0	0.0 - 0.0	12.0	4.7 - 25.2	0.92
3140106	66170	BRUSHY CR	70.1	96.2	32 - 172	23.8	7.8 - 53.1	29.1	10.4 - 54.8	2.9	0.8 - 8.2	27.5	7.9 - 56.5	0.0	0.0 - 0.0	12.8	4.3 - 26.2	0.88
3130012	66171	CHIPOLA R	46.9	32.7	11 - 60	0.0	0.0 - 0.0	6.9	2.1 - 13.0	0.4	0.1 - 1.2	2.7	0.9 - 6.1	0.0	0.0 - 0.0	22.6	7.9 - 50.2	0.71
3100204	66172	ALAFIA R	9.7	79.2	24 - 151	0.0	0.0 - 0.0	48.8	17.1 - 90.1	0.9	0.3 - 2.5	1.3	0.4 - 3.4	0.0	0.0 - 0.0	28.2	9.1 - 71.4	0.95
3100208	66173	WITHLACOOCHEE	168.8	59.5	19 - 153	0.0	0.0 - 0.0	11.2	3.4 - 27.6	7.3	2.0 - 25.3	17.4	5.7 - 43.4	0.0	0.0 - 0.0	23.5	7.6 - 68.4	0.08
3110202	66174	GRAND BAY CR	138.0	38.0	15 - 75	0.0	0.0 - 0.0	7.7	2.8 - 15.0	0.7	0.2 - 2.3	9.2	3.3 - 22.0	0.1	0.0 - 0.3	20.3	7.9 - 46.2	0.92
3080103	66176	BLACK CR	23.0	34.1	10 - 64	0.0	0.0 - 0.0	20.8	6.0 - 42.3	0.6	0.2 - 1.7	1.3	0.4 - 3.4	0.0	0.0 - 0.0	11.5	3.5 - 24.7	0.97
3110102	66177	ECONFINA R	547.4	13.5	4 - 39	0.0	0.0 - 0.0	1.8	0.5 - 4.7	0.5	0.2 - 1.5	1.2	0.3 - 3.9	0.0	0.0 - 0.0	10.0	3.2 - 27.7	0.92
3110102	66178	FENHOLLOWAY R	757.5	63.9	24 - 149	51.1	18.9 - 123.8	3.1	1.1 - 5.9	0.5	0.2 - 1.4	0.7	0.3 - 1.6	0.0	0.0 - 0.0	8.5	3.0 - 19.1	0.94
3110206	66179	NEW R	40.7	40.6	15 - 114	0.0	0.0 - 0.0	5.1	1.8 - 12.6	6.7	2.3 - 19.3	11.8	4.7 - 31.1	0.0	0.0 - 0.0	17.0	6.2 - 53.5	0.86
3100101	66180	PEACE R	126.7	2187.1	770 - 5123	289.8	105.9 - 619.8	13.8	5.2 - 29.8	9.4	2.8 - 28.6	3.7	1.3 - 10.3	1811.3	640.9 - 460[illegible]	59.1	19.8 - 152.7	0.83
3110206	66181	SANTE FE R	14.6	39.3	13 - 80	0.0	0.0 - 0.0	8.0	2.3 - 18.2	4.3	1.4 - 13.8	11.6	3.5 - 29.4	0.0	0.0 - 0.0	15.4	5.7 - 43.1	0.91
3110206	66182	SANTE FE R	44.2	37.5	14 - 76	0.0	0.0 - 0.0	6.6	2.0 - 14.9	2.9	0.9 - 11.3	9.6	3.0 - 23.0	0.0	0.0 - 0.0	18.4	6.7 - 46.0	0.93
3110206	66183	SANTE FE R	337.5	41.4	14 - 84	0.0	0.0 - 0.0	9.8	3.4 - 22.1	3.7	1.1 - 9.1	11.0	3.7 - 28.3	0.0	0.0 - 0.0	16.9	5.1 - 40.6	0.94
3110206	66184	SANTE FE R	460.4	34.4	13 - 92	0.0	0.0 - 0.0	7.1	2.7 - 16.9	3.5	1.1 - 10.7	10.2	3.7 - 30.2	0.0	0.0 - 0.0	13.7	4.7 - 40.0	0.96
3110201	66185	SUWANNEE R	118.2	38.2	11 - 74	0.2	0.1 - 0.6	12.2	3.4 - 27.3	1.2	0.3 - 3.5	2.5	0.7 - 6.1	0.1	0.0 - 0.4	22.0	6.1 - 47.3	0.90
3110201	66186	SUWANNEE R	140.8	50.7	19 - 101	0.0	0.0 - 0.0	7.8	2.6 - 18.5	3.4	1.2 - 9.1	5.6	2.0 - 13.6	12.9	4.0 - 40.6	21.1	7.8 - 50.0	0.92
3110205	66187	SUWANNEE R	3.7	4948.8	1874 - 11906	4909.5	1857.3 - 1181[illegible]	27.9	9.8 - 63.9	2.1	0.8 - 5.7	2.3	0.8 - 6.4	0.0	0.0 - 0.0	7.0	2.7 - 19.1	0.94
3110205	66188	SUWANNEE R	654.9	28.0	9 - 65	0.0	0.0 - 0.0	6.9	2.3 - 16.5	5.9	1.5 - 16.4	8.0	2.4 - 22.4	0.0	0.0 - 0.0	7.1	2.5 - 16.2	0.96
3110205	66189	SUWANNEE R	1154.9	37.6	12 - 66	0.0	0.0 - 0.0	9.2	2.7 - 16.9	7.5	2.2 - 20.0	10.6	3.0 - 22.8	0.0	0.0 - 0.0	10.3	3.5 - 20.0	0.97
3110205	66190	SUWANNEE R	182.4	27.1	9 - 51	0.0	0.0 - 0.0	8.8	2.9 - 17.0	3.6	1.0 - 8.6	8.4	2.9 - 22.9	0.0	0.0 - 0.0	6.4	2.2 - 14.4	0.98
3110205	66191	SUWANNEE R	39.4	35.7	13 - 66	0.0	0.0 - 0.0	17.8	6.2 - 34.0	2.0	0.6 - 4.8	7.7	2.7 - 18.2	0.0	0.0 - 0.0	8.1	3.0 - 16.8	0.99
3110205	66192	SUWANNEE R	642.6	19.7	7 - 44	0.0	0.0 - 0.0	5.7	1.8 - 12.2	2.1	0.6 - 5.6	6.6	2.1 - 20.2	0.0	0.0 - 0.0	5.3	1.9 - 13.8	1.00
3080201	66193	*D	80.8	46.4	14 - 110	0.0	0.0 - 0.0	18.0	5.0 - 39.4	1.8	0.5 - 4.0	1.9	0.6 - 5.0	0.0	0.0 - 0.0	24.7	7.5 - 66.2	0.92
3100202	66194	MANATEE R	36.6	86.7	31 - 168	0.0	0.0 - 0.0	3.1	1.2 - 6.4	11.2	3.8 - 26.6	36.1	11.3 - 84.5	0.0	0.0 - 0.0	36.3	13.2 - 89.9	0.23
3110203	66195	WITHLACOOCHEE	147.1	99.2	35 - 233	51.3	17.9 - 138.0	9.4	2.9 - 24.1	1.0	0.3 - 3.5	6.6	2.2 - 19.1	0.0	0.0 - 0.0	30.9	10.2 - 81.7	0.91
3060102	66196	TALLULAH R	76.3	42.1	14 - 109	0.0	0.0 - 0.0	8.8	2.7 - 22.6	2.5	0.7 - 7.2	2.7	0.8 - 9.1	0.0	0.0 - 0.0	28.1	9.7 - 75.5	0.03
3060106	66198	SAVANNAH R	5.4	13.2	4 - 28	0.0	0.0 - 0.0	0.1	0.0 - 0.2	0.1	0.0 - 0.4	0.2	0.0 - 0.4	0.0	0.0 - 0.0	12.8	3.6 - 28.0	0.95
3060108	66199	BRIER CR	678.8	22.7	7 - 50	0.0	0.0 - 0.0	6.1	1.9 - 14.6	0.9	0.2 - 2.8	8.3	2.3 - 21.3	0.0	0.0 - 0.0	7.3	2.2 - 20.3	0.93
3060201	66200	WILLIAMSON SWA	287.7	22.6	7 - 47	0.0	0.0 - 0.0	4.0	0.9 - 9.6	0.9	0.2 - 2.7	7.0	2.1 - 16.2	0.0	0.0 - 0.0	10.7	3.8 - 28.4	0.64
3070102	66201	OCONEE R	72.8	42.9	13 - 108	0.0	0.0 - 0.0	18.6	5.7 - 49.5	2.0	0.5 - 8.6	4.9	1.5 - 13.6	0.0	0.0 - 0.0	17.4	5.3 - 47.7	0.88
3070103	66202	TOBESOFKEE R	158.2	37.1	14 - 74	0.0	0.0 - 0.0	18.1	6.5 - 39.0	1.0	0.4 - 3.2	5.3	2.2 - 13.3	0.0	0.0 - 0.0	12.7	4.5 - 31.3	0.41
3070103	66203	OCMULGEE R	34.6	19.9	8 - 41	0.0	0.0 - 0.0	2.4	0.8 - 5.5	0.6	0.2 - 1.4	4.8	1.7 - 12.9	0.0	0.0 - 0.0	12.1	4.2 - 27.2	0.87
3070201	66204	SATILLA R	191.7	31.6	10 - 60	0.0	0.0 - 0.0	6.1	2.1 - 12.4	3.4	0.9 - 9.0	12.8	4.1 - 33.0	0.0	0.0 - 0.0	9.3	3.0 - 22.2	0.87
3070202	66205	BIG SATILLA CR	17.1	20.6	7 - 55	0.0	0.0 - 0.0	3.4	1.2 - 9.3	0.7	0.2 - 2.3	6.2	2.1 - 18.4	0.0	0.0 - 0.0	10.3	3.6 - 31.9	0.88
3070201	66206	SATILLA R	78.8	11.1	3 - 29	0.0	0.0 - 0.0	2.0	0.6 - 5.0	1.2	0.3 - 3.1	1.5	0.4 - 4.8	0.0	0.0 - 0.0	6.4	1.9 - 16.8	0.94
3110203	66208	WITHLACOOCHEE	45.4	29.7	9 - 56	0.0	0.0 - 0.0	6.0	1.9 - 11.9	1.2	0.4 - 2.9	4.1	1.2 - 10.5	0.0	0.0 - 0.0	18.4	5.9 - 44.0	0.91
3130005	66209	FLINT R	47.3	43.9	13 - 77	0.0	0.0 - 0.0	7.1	2.1 - 12.6	3.9	1.1 - 9.8	17.7	5.3 - 36.0	0.0	0.0 - 0.0	15.2	4.7 - 32.8	0.44

8-digit HUC	ID	Name	Area	Catchment Yield		Point sources		Developed Land		Manure		Agricultural Land		Phosphate Mines		Soil parent rock		Frac
				kg/km^2	90% CI	kg/km^2	90% CI	kg/km^2	90% CI	kg/km^2	90% CI	kg/km^2	90% CI	kg/km^2	90% CI	kg/km^2	90% CI	
3130007	66211	KINCHAFOONEE C	309.4	32.4	10 - 74	0.0	0.0 - 0.0	3.7	1.2 - 7.5	1.0	0.3 - 3.1	17.8	5.2 - 48.1	0.0	0.0 - 0.0	9.9	2.9 - 24.6	0.58
3130009	66212	PACHITLA CR	5.3	48.5	16 - 93	0.0	0.0 - 0.0	7.9	2.4 - 15.9	2.8	0.8 - 7.2	22.8	8.4 - 60.3	0.0	0.0 - 0.0	15.0	5.0 - 37.6	0.57
3130009	66213	ICHAWAYNOCHAW	11.1	44.4	16 - 85	0.0	0.0 - 0.0	4.6	1.4 - 9.6	3.3	0.8 - 8.8	21.8	7.2 - 52.5	0.0	0.0 - 0.0	14.8	4.6 - 34.2	0.60
3130009	66214	ICHAWAYNOCHAW	186.9	35.7	11 - 70	0.0	0.0 - 0.0	6.2	1.7 - 11.4	2.6	0.7 - 6.1	16.7	5.1 - 39.6	0.0	0.0 - 0.0	10.3	3.0 - 24.8	0.62
3130010	66215	SPRING CR	190.7	44.1	15 - 86	0.0	0.0 - 0.0	5.3	1.7 - 11.2	2.9	0.9 - 6.1	25.2	9.3 - 61.4	0.0	0.0 - 0.0	10.8	3.4 - 23.8	0.61
3130001	66216	CHATTAHOOCHEE	10.0	76.8	30 - 133	0.0	0.0 - 0.0	46.3	17.5 - 92.9	7.2	2.3 - 15.8	2.6	0.9 - 6.1	0.0	0.0 - 0.0	20.7	7.2 - 41.6	0.04
3130001	66218	CHESTATEE R	44.7	56.8	18 - 112	0.0	0.0 - 0.0	13.3	4.0 - 27.8	18.0	4.7 - 52.4	10.1	3.7 - 25.5	0.0	0.0 - 0.0	15.4	5.1 - 36.3	0.04
3130001	66219	CHATTAHOOCHEE	19.2	59.3	21 - 149	0.0	0.0 - 0.0	17.5	6.4 - 42.6	8.2	2.4 - 28.2	5.3	1.6 - 15.1	0.0	0.0 - 0.0	28.4	10.0 - 77.5	0.04
3130001	66220	CHATTAHOOCHEE	32.3	87.7	32 - 175	0.0	0.0 - 0.0	53.0	18.7 - 119.2	11.9	3.5 - 38.6	9.3	3.3 - 26.3	0.0	0.0 - 0.0	13.5	4.9 - 31.1	0.17
3130002	66221	CHATTAHOOCHEE	54.0	131.4	44 - 320	0.0	0.0 - 0.0	95.1	31.3 - 215.4	0.5	0.1 - 1.6	2.8	0.9 - 6.7	0.0	0.0 - 0.0	32.9	11.2 - 84.7	0.17
3130002	66222	CHATTAHOOCHEE	387.4	38.7	12 - 80	0.0	0.0 - 0.0	6.6	2.0 - 13.1	9.4	2.5 - 28.4	11.7	3.4 - 30.7	0.0	0.0 - 0.0	11.0	3.5 - 25.6	0.18
3130003	66224	CHATTAHOOCHEE	9.0	6567.6	2197 - 16033	6507.0	2174.9 - 1589[illegible]	33.7	12.4 - 86.4	1.7	0.5 - 4.7	12.8	5.1 - 29.6	0.0	0.0 - 0.0	12.4	4.0 - 33.7	0.35
3130003	66225	UPATOI CR	5.1	18.5	6 - 35	0.0	0.0 - 0.0	7.1	2.3 - 15.4	0.3	0.1 - 0.8	2.0	0.6 - 5.4	0.0	0.0 - 0.0	9.2	3.0 - 21.5	0.35
3130003	66226	CHATTAHOOCHEE	3.8	12.0	4 - 25	0.0	0.0 - 0.0	2.4	0.8 - 4.7	0.0	0.0 - 0.0	0.2	0.1 - 0.4	0.0	0.0 - 0.0	9.4	3.3 - 21.9	0.36
3130004	66227	CHATTAHOOCHEE	3.1	61.3	25 - 146	0.0	0.0 - 0.0	21.2	7.9 - 56.8	4.0	1.4 - 13.8	21.7	8.8 - 65.3	0.0	0.0 - 0.0	14.4	6.1 - 33.2	0.61
3160102	66228	TOWN CR	18.5	223.0	75 - 494	0.0	0.0 - 0.0	6.4	2.0 - 12.5	12.8	3.8 - 35.8	98.9	31.4 - 280.4	0.0	0.0 - 0.0	105.0	30.9 - 254.2	0.81
3160103	66229	BUTTAHATCHEE R	114.3	24.4	7 - 51	0.0	0.0 - 0.0	4.4	1.4 - 9.8	1.1	0.3 - 2.8	8.5	2.6 - 20.0	0.0	0.0 - 0.0	10.4	3.2 - 23.5	0.82
3160105	66230	LUXAPALLILA CR	126.4	123.6	48 - 300	0.2	0.1 - 0.4	13.2	4.6 - 29.6	7.0	2.4 - 22.4	69.7	26.6 - 188.9	0.0	0.0 - 0.0	33.5	12.0 - 98.7	0.86
3160106	66231	COAL FIRE CR	340.6	15.7	6 - 27	0.0	0.0 - 0.0	5.5	2.0 - 11.6	2.4	0.8 - 6.2	3.1	1.0 - 7.2	0.0	0.0 - 0.0	4.7	1.5 - 10.0	0.87
3160202	66232	SUCARNOCHEE R	96.5	57.5	17 - 109	4.5	1.5 - 8.4	7.3	2.3 - 14.8	2.0	0.6 - 4.8	8.2	2.7 - 19.7	0.0	0.0 - 0.0	35.5	10.4 - 78.7	0.80
3170004	66233	LEAF R	16.8	82.2	34 - 195	0.0	0.0 - 0.0	7.5	2.8 - 16.1	27.7	9.6 - 79.3	26.2	10.0 - 66.9	0.0	0.0 - 0.0	20.8	7.4 - 55.3	0.89
3170004	66234	BOWIE R	170.1	57.4	18 - 102	0.2	0.1 - 0.5	8.3	2.7 - 16.6	15.8	4.7 - 37.7	19.3	6.1 - 45.4	0.0	0.0 - 0.0	13.8	4.5 - 31.1	0.88
3170004	66235	OKATOMA CR	245.4	159.0	59 - 383	90.0	35.8 - 233.4	14.8	4.9 - 35.9	21.6	7.1 - 55.3	20.9	7.7 - 56.7	0.0	0.0 - 0.0	11.8	4.4 - 30.5	0.89
3170005	66236	LEAF R	182.7	191.9	50 - 415	114.2	30.6 - 277.1	47.8	12.9 - 105.3	5.4	1.3 - 17.7	12.8	3.2 - 37.9	0.0	0.0 - 0.0	11.9	3.1 - 31.1	0.94
3170005	66237	TALLAHALA CR	260.5	50.9	20 - 91	0.1	0.0 - 0.2	7.0	2.5 - 14.6	15.5	5.1 - 44.8	18.5	6.7 - 43.1	0.0	0.0 - 0.0	9.9	3.7 - 23.9	0.93
3170003	66238	CHICKASAWHAY R	518.7	43.1	15 - 82	0.3	0.1 - 0.8	8.0	2.5 - 16.3	8.0	2.5 - 19.5	10.3	3.4 - 26.3	0.0	0.0 - 0.0	16.5	5.9 - 42.4	0.96
3170007	66239	BLACK CR	111.0	14.1	5 - 26	0.0	0.0 - 0.0	4.0	1.5 - 8.7	0.7	0.2 - 2.0	1.1	0.4 - 3.1	0.0	0.0 - 0.0	8.2	2.9 - 17.5	0.96
3170007	66240	RED CR	81.2	17.3	6 - 41	0.0	0.0 - 0.0	6.6	2.3 - 15.1	0.9	0.3 - 2.9	0.8	0.3 - 2.3	0.0	0.0 - 0.0	8.9	3.4 - 21.1	0.97
3170006	66241	PASCAGOULA R	11.9	16.2	5 - 40	0.0	0.0 - 0.0	2.4	0.6 - 5.9	0.0	0.0 - 0.0	0.0	0.0 - 0.0	0.0	0.0 - 0.0	13.8	3.9 - 36.1	0.99
3170008	66242	ESCATAWPA R	164.8	36.4	12 - 77	0.0	0.0 - 0.0	10.1	3.5 - 22.7	3.0	1.0 - 8.2	11.4	3.4 - 30.5	0.0	0.0 - 0.0	11.9	4.1 - 28.3	0.96
3170009	66243	BILOXI R	245.7	25.2	8 - 49	0.0	0.0 - 0.0	7.2	2.1 - 15.3	1.9	0.5 - 4.8	4.2	1.3 - 10.0	0.0	0.0 - 0.0	11.9	3.3 - 29.3	0.94
3170009	66244	WOLF R	398.0	28.6	8 - 76	0.0	0.0 - 0.0	5.2	1.6 - 11.3	2.8	0.8 - 9.1	9.2	2.4 - 26.4	0.0	0.0 - 0.0	11.4	3.2 - 29.1	0.97
3180001	66245	PEARL R	45.7	49.7	16 - 94	0.0	0.0 - 0.0	6.2	2.1 - 13.6	14.6	4.5 - 38.9	11.3	3.7 - 24.0	0.0	0.0 - 0.0	17.6	5.4 - 42.2	0.39
3180001	66246	YOCKANOOKANY	759.3	56.9	19 - 133	0.4	0.1 - 1.0	8.6	2.9 - 19.0	6.3	1.9 - 17.2	15.1	5.1 - 37.0	0.0	0.0 - 0.0	26.5	9.7 - 69.7	0.40
3180002	66247	PEARL R	121.0	942.4	330 - 2106	683.7	222.5 - 1649.[illegible]	48.0	15.6 - 108.2	15.8	4.2 - 46.7	37.2	11.1 - 101.5	0.0	0.0 - 0.0	157.6	49.7 - 494.5	0.88
3180002	66248	STRONG R	7.1	77.4	21 - 142	0.0	0.0 - 0.0	22.8	6.4 - 40.7	23.3	6.1 - 57.8	14.5	4.2 - 32.8	0.0	0.0 - 0.0	16.8	4.3 - 43.7	0.87
3180003	66249	PEARL R	82.6	82.8	27 - 193	0.0	0.0 - 0.0	13.1	4.5 - 30.3	23.1	6.5 - 62.2	28.1	8.4 - 82.3	0.0	0.0 - 0.0	18.5	5.8 - 42.1	0.92
3180004	66250	PUSHEPATAPA CR	514.2	70.2	26 - 180	0.0	0.0 - 0.0	10.7	4.1 - 26.4	15.5	4.7 - 50.0	25.9	8.1 - 79.7	0.0	0.0 - 0.0	18.1	6.5 - 45.6	0.97
3050105	66251	SECOND BROAD R	76.5	23.2	8 - 54	0.0	0.0 - 0.0	4.7	1.4 - 10.6	1.3	0.4 - 4.4	5.0	1.6 - 12.9	0.0	0.0 - 0.0	12.2	4.3 - 26.9	0.29

8-digit HUC	ID	Name	Area	Catchment Yield		Point sources		Developed Land		Manure		Agricultural Land		Phosphate Mines		Soil parent rock		Frac
				kg/km^2	90% CI	kg/km^2	90% CI	kg/km^2	90% CI	kg/km^2	90% CI	kg/km^2	90% CI	kg/km^2	90% CI	kg/km^2	90% CI	
3050105	66252	BROAD R	14.5	34.0	11 - 80	0.0	0.0 - 0.0	5.7	1.8 - 12.3	3.7	0.9 - 10.6	12.5	4.1 - 33.9	0.0	0.0 - 0.0	12.1	3.8 - 31.9	0.32
3030002	66253	BUFFALO CR	95.7	259.2	89 - 492	170.7	57.7 - 371.4	71.7	25.4 - 145.5	0.4	0.1 - 1.0	2.4	0.9 - 5.0	0.0	0.0 - 0.0	14.0	4.4 - 31.0	0.31
3030002	66254	*A	112.8	276.4	105 - 677	192.1	71.0 - 471.7	65.2	24.3 - 152.8	0.8	0.2 - 2.6	4.9	1.7 - 14.6	0.0	0.0 - 0.0	13.3	4.8 - 36.1	0.32
3030002	66255	HAW R	51.2	156.0	44 - 290	92.3	25.0 - 191.3	45.5	14.8 - 100.2	1.9	0.5 - 4.8	7.1	2.0 - 18.3	0.0	0.0 - 0.0	9.2	2.7 - 19.9	0.37
3030002	66256	NEW HOPE CR	196.6	78.7	27 - 202	21.3	6.9 - 57.4	35.6	10.7 - 104.5	1.3	0.4 - 4.4	5.6	1.7 - 18.4	0.0	0.0 - 0.0	15.0	4.8 - 41.5	0.36
3030002	66257	NORTHEAST CR	55.5	119.3	45 - 262	55.5	20.2 - 130.4	47.4	18.7 - 110.7	0.7	0.2 - 2.3	2.9	1.0 - 7.7	0.0	0.0 - 0.0	12.8	4.5 - 33.5	0.36
3030002	66258	MORGAN CR	93.7	75.7	26 - 172	38.5	14.0 - 83.6	20.5	5.8 - 46.6	1.6	0.4 - 4.0	4.7	1.6 - 10.3	0.0	0.0 - 0.0	10.4	3.2 - 26.6	0.37
3030003	66259	HICKORY CR	37.7	75.3	24 - 161	0.0	0.0 - 0.0	54.1	16.6 - 114.9	1.2	0.4 - 3.4	7.2	2.4 - 17.7	0.0	0.0 - 0.0	12.8	4.3 - 28.4	0.27
3030003	66260	DEEP R	167.0	293.5	90 - 756	235.3	70.1 - 611.3	39.2	13.1 - 107.2	1.9	0.6 - 5.8	7.5	2.3 - 22.2	0.0	0.0 - 0.0	9.6	3.1 - 24.3	0.67
3030004	66261	CAPE FEAR R	17.6	29.3	9 - 60	0.0	0.0 - 0.0	14.7	4.4 - 29.2	4.8	1.2 - 12.2	5.9	1.8 - 15.3	0.0	0.0 - 0.0	4.0	1.4 - 9.0	0.90
3030004	66262	UPPER LITTLE R	200.0	26.3	9 - 69	0.0	0.0 - 0.0	5.7	1.9 - 14.0	6.2	1.9 - 17.5	7.9	2.3 - 22.7	0.0	0.0 - 0.0	6.4	2.5 - 18.0	0.90
3030004	66263	CAPE FEAR R	41.9	68.4	26 - 132	29.9	11.5 - 61.8	15.0	5.7 - 37.0	8.2	2.8 - 20.6	10.8	3.6 - 26.1	0.0	0.0 - 0.0	4.6	1.7 - 10.6	0.91
3030005	66265	CAPE FEAR R	329.7	112.3	35 - 261	86.3	27.6 - 209.5	3.2	0.9 - 8.9	7.5	2.0 - 19.2	7.4	2.1 - 22.4	0.0	0.0 - 0.0	7.9	2.6 - 17.1	0.99
3030006	66266	BLACK R	55.6	54.0	20 - 127	0.0	0.0 - 0.0	3.5	1.3 - 8.0	27.5	9.1 - 87.5	9.3	3.4 - 21.7	0.0	0.0 - 0.0	13.7	5.3 - 42.6	0.93
3030007	66267	NE CAPE FEAR R	154.0	85.0	27 - 181	0.0	0.0 - 0.0	3.0	1.0 - 6.2	58.3	15.9 - 166.5	14.5	4.6 - 32.3	0.0	0.0 - 0.0	9.2	3.2 - 21.6	0.89
3050101	66268	CATAWBA R	79.7	23.7	7 - 61	0.0	0.0 - 0.0	6.2	1.8 - 15.1	1.4	0.4 - 4.3	4.5	1.3 - 13.5	0.0	0.0 - 0.0	11.6	3.6 - 31.0	0.01
3050101	66269	LINVILLE R	168.7	27.1	10 - 55	3.5	1.3 - 7.4	11.2	4.3 - 24.6	0.3	0.1 - 0.6	2.1	0.7 - 5.8	0.0	0.0 - 0.0	10.0	3.6 - 24.4	0.01
3050102	66270	CATAWBA R, S FK	64.0	17.6	6 - 45	0.1	0.0 - 0.1	3.1	1.2 - 7.4	0.5	0.2 - 1.5	1.7	0.6 - 5.0	0.0	0.0 - 0.0	12.2	4.0 - 33.3	0.14
3050102	66271	LITTLE LONG CR	81.9	47.8	15 - 97	0.0	0.0 - 0.0	12.5	3.7 - 26.4	4.1	1.2 - 13.0	17.9	6.2 - 42.3	0.0	0.0 - 0.0	13.3	4.4 - 34.5	0.18
3050102	66272	CATAWBA R, S FK	30.2	98.5	32 - 172	48.0	15.3 - 101.7	29.9	9.6 - 56.0	1.1	0.4 - 2.8	4.5	1.3 - 10.6	0.0	0.0 - 0.0	14.9	5.1 - 33.7	0.20
3050103	66273	SUGAR CR	31.2	91.8	22 - 196	0.0	0.0 - 0.0	70.8	16.8 - 156.1	0.5	0.1 - 1.5	3.7	0.9 - 9.5	0.0	0.0 - 0.0	16.8	4.5 - 43.1	0.27
3050103	66274	LITTLE SUGAR CR	128.8	282.4	99 - 662	182.1	59.2 - 434.4	84.4	31.2 - 213.4	0.0	0.0 - 0.1	0.3	0.1 - 0.9	0.0	0.0 - 0.0	15.5	5.3 - 44.2	0.27
3050103	66275	MCALPINE CR	99.6	85.9	30 - 202	1.9	0.7 - 5.1	71.9	24.4 - 169.9	0.2	0.1 - 0.6	1.4	0.5 - 3.7	0.0	0.0 - 0.0	10.5	3.6 - 27.3	0.25
3050103	66276	TWELVEMILE CR	193.8	64.7	17 - 139	0.0	0.0 - 0.0	9.7	2.7 - 22.4	20.3	5.7 - 52.6	20.2	5.6 - 44.7	0.0	0.0 - 0.0	14.5	4.9 - 36.5	0.27
6010105	66277	FRENCH BROAD R	2.6	94.5	27 - 196	0.0	0.0 - 0.0	53.0	14.9 - 110.2	2.2	0.6 - 5.3	11.0	2.9 - 30.9	0.0	0.0 - 0.0	28.4	8.8 - 65.5	0.13
6010105	66278	FRENCH BROAD R	72.6	1157.9	345 - 2649	1121.9	336.9 - 2567.4	28.4	8.7 - 70.9	0.6	0.1 - 1.7	2.1	0.6 - 5.9	0.0	0.0 - 0.0	4.9	1.3 - 13.0	0.14
6010105	66279	FRENCH BROAD R	112.6	20.7	7 - 43	0.0	0.0 - 0.0	8.4	3.0 - 19.8	1.1	0.4 - 3.1	4.1	1.5 - 11.2	0.0	0.0 - 0.0	7.0	2.5 - 18.2	0.14
6010106	66280	PIGEON R	31.0	37.7	12 - 78	0.0	0.0 - 0.0	13.7	4.2 - 30.7	3.2	0.9 - 8.7	8.3	2.4 - 20.1	0.0	0.0 - 0.0	12.5	4.2 - 32.6	0.13
6010106	66281	PIGEON R	43.3	618.2	270 - 1232	583.3	249.4 - 1184.8	14.5	5.9 - 28.8	2.6	0.9 - 7.7	6.7	2.8 - 14.7	0.0	0.0 - 0.0	11.1	4.4 - 24.8	0.13
6010106	66282	CATALOOCHEE CI	127.6	9.3	3 - 21	0.0	0.0 - 0.0	0.6	0.2 - 1.4	0.0	0.0 - 0.1	0.1	0.0 - 0.2	0.0	0.0 - 0.0	8.6	2.8 - 20.3	0.13
6010106	66283	PIGEON R	21.9	13.5	5 - 26	0.0	0.0 - 0.0	5.4	1.7 - 10.9	0.8	0.2 - 2.0	1.9	0.6 - 4.9	0.0	0.0 - 0.0	5.3	1.8 - 13.5	0.13
6010108	66284	S TOE R	111.9	17.0	6 - 35	0.0	0.0 - 0.0	3.7	1.3 - 8.3	0.2	0.1 - 0.7	0.8	0.2 - 1.9	0.0	0.0 - 0.0	12.2	4.2 - 25.4	0.12
6020002	66285	HIWASSEE R	41.7	23.2	8 - 54	0.0	0.0 - 0.0	10.1	3.0 - 25.0	0.9	0.3 - 2.4	2.9	0.9 - 8.4	0.0	0.0 - 0.0	9.3	3.5 - 22.5	0.33
6020002	66286	VALLEY R	162.5	34.0	12 - 63	6.8	2.5 - 14.0	11.4	4.3 - 24.0	0.8	0.3 - 1.8	2.8	1.0 - 7.7	0.0	0.0 - 0.0	12.2	4.4 - 27.5	0.32
3040203	66287	DROWNING CR	6.6	43.2	14 - 91	0.0	0.0 - 0.0	6.6	2.1 - 12.9	12.3	3.6 - 33.7	7.5	2.5 - 20.2	0.0	0.0 - 0.0	16.8	5.6 - 41.4	0.73
3040203	66288	LUMBER R	242.4	27.4	8 - 70	3.0	0.9 - 7.9	6.3	2.0 - 14.2	2.8	0.8 - 8.3	5.2	1.5 - 14.0	0.0	0.0 - 0.0	10.2	3.3 - 30.0	0.78
3040203	66289	LUMBER R	3.0	30.9	7 - 70	0.0	0.0 - 0.0	6.0	1.6 - 12.6	2.4	0.5 - 6.5	3.0	0.8 - 6.9	0.0	0.0 - 0.0	19.6	5.0 - 44.6	0.91
3040206	66290	WACCAMAW R	28.3	34.3	10 - 75	0.0	0.0 - 0.0	1.4	0.4 - 3.2	6.7	1.7 - 19.9	13.9	4.0 - 33.4	0.0	0.0 - 0.0	12.4	3.3 - 30.3	0.87
3020201	66291	ENO R	216.0	44.5	16 - 81	2.6	0.9 - 5.1	20.7	6.7 - 40.5	1.5	0.4 - 4.0	6.6	2.1 - 15.7	0.0	0.0 - 0.0	13.1	4.3 - 35.5	0.28

8-digit HUC	ID	Name	Area	Catchment Yield		Point sources		Developed Land		Manure		Agricultural Land		Phosphate Mines		Soil parent rock		Frac
				kg/km^2	90% CI	kg/km^2	90% CI	kg/km^2	90% CI	kg/km^2	90% CI	kg/km^2	90% CI	kg/km^2	90% CI	kg/km^2	90% CI	
3020201	66293	NEUSE R	102.9	29.2	9 - 52	0.1	0.0 - 0.2	14.5	4.3 - 28.2	0.6	0.2 - 1.9	5.0	1.6 - 11.8	0.0	0.0 - 0.0	9.0	2.9 - 21.5	0.31
3020201	66294	CRABTREE CR	218.3	91.9	34 - 176	33.8	12.8 - 71.1	40.2	14.7 - 88.5	0.5	0.2 - 1.4	4.8	1.8 - 13.8	0.0	0.0 - 0.0	12.6	4.6 - 33.2	0.79
3020201	66295	NEUSE R	356.0	186.9	64 - 450	153.8	51.6 - 375.2	11.3	4.0 - 25.7	3.5	1.1 - 9.6	10.7	3.7 - 29.0	0.0	0.0 - 0.0	7.5	2.9 - 17.1	0.89
3020202	66296	NEUSE R	36.8	63.6	23 - 122	11.2	4.0 - 24.1	15.4	5.4 - 32.4	15.8	5.5 - 34.9	11.2	3.9 - 27.9	0.0	0.0 - 0.0	10.2	3.8 - 24.0	0.94
3020204	66297	TRENT R	117.4	28.7	10 - 57	0.0	0.0 - 0.0	1.7	0.6 - 3.8	12.4	3.8 - 32.3	8.8	3.1 - 23.1	0.0	0.0 - 0.0	5.8	2.0 - 13.5	0.93
6010103	66298	WATAUGA R, BRU	90.8	25.1	8 - 58	0.1	0.0 - 0.2	7.3	2.1 - 16.2	1.7	0.5 - 4.8	5.0	1.6 - 13.8	0.0	0.0 - 0.0	11.2	3.4 - 27.8	0.03
3020101	66300	TAR R	212.7	84.2	26 - 142	36.1	12.2 - 70.6	19.1	5.5 - 35.7	6.2	1.8 - 14.9	14.5	4.4 - 29.6	0.0	0.0 - 0.0	8.2	2.4 - 18.0	0.92
3020102	66301	FISHING CR	221.1	27.1	9 - 48	0.0	0.0 - 0.0	5.8	1.9 - 10.8	3.7	1.1 - 10.3	12.0	3.8 - 26.1	0.0	0.0 - 0.0	5.6	1.9 - 13.5	0.87
3020103	66302	CONETOE CR	28.3	76.4	30 - 141	0.0	0.0 - 0.0	5.1	1.7 - 10.4	16.5	5.2 - 42.5	40.2	13.8 - 87.7	0.0	0.0 - 0.0	14.5	5.1 - 31.6	0.97
3020103	66303	CONETOE CR	49.0	104.6	34 - 255	0.0	0.0 - 0.0	7.6	2.3 - 18.6	22.9	6.8 - 57.6	56.1	18.6 - 134.0	0.0	0.0 - 0.0	17.9	5.8 - 45.8	0.92
3030001	66304	NEW R	197.3	34.5	11 - 58	1.6	0.5 - 2.9	3.1	0.9 - 5.4	12.2	3.6 - 26.7	12.8	4.4 - 27.3	0.0	0.0 - 0.0	4.8	1.7 - 8.7	0.96
3040101	66305	ELK CR	73.0	16.1	5 - 36	0.0	0.0 - 0.0	1.8	0.6 - 3.9	2.6	0.7 - 6.8	1.5	0.5 - 3.9	0.0	0.0 - 0.0	10.2	3.2 - 26.9	0.21
3040103	66306	ABBOTTS CR	12.8	62.8	18 - 120	0.0	0.0 - 0.0	48.4	13.7 - 107.0	0.8	0.2 - 2.0	2.6	0.7 - 7.0	0.0	0.0 - 0.0	11.1	3.3 - 21.9	0.46
3040103	66307	ABBOTTS CR	1.2	37.8	11 - 72	0.0	0.0 - 0.0	5.5	1.8 - 11.3	4.4	1.3 - 11.5	16.3	4.8 - 43.3	0.0	0.0 - 0.0	11.6	3.8 - 25.2	0.48
3040104	66308	PEE DEE R	3.8	126.4	34 - 295	0.0	0.0 - 0.0	2.7	0.8 - 5.9	65.1	14.3 - 202.8	41.8	11.5 - 116.9	0.0	0.0 - 0.0	16.8	4.3 - 44.8	0.79
3040104	66309	LITTLE R	428.8	35.3	10 - 75	7.0	2.2 - 16.8	4.8	1.5 - 10.4	6.8	1.9 - 18.7	3.9	1.2 - 10.6	0.0	0.0 - 0.0	12.7	4.3 - 32.7	0.78
3050107	66310	S TYGER R	203.7	86.3	31 - 178	26.5	9.1 - 61.2	27.5	9.6 - 59.4	1.9	0.5 - 4.6	12.1	4.3 - 35.0	0.0	0.0 - 0.0	18.3	6.7 - 46.0	0.29
3050107	66311	M TYGER R	170.2	80.8	25 - 203	22.6	7.0 - 62.0	21.4	6.6 - 52.5	2.2	0.6 - 6.0	14.4	4.3 - 49.8	0.0	0.0 - 0.0	20.2	6.2 - 55.6	0.20
3050105	66313	BROAD R	51.1	41.7	12 - 81	0.0	0.0 - 0.0	8.9	2.5 - 15.8	3.9	1.1 - 10.0	16.9	4.8 - 45.2	0.0	0.0 - 0.0	12.0	3.7 - 28.0	0.32
3050105	66314	BROAD R	5.6	24.5	8 - 48	0.0	0.0 - 0.0	3.5	1.1 - 7.0	1.9	0.5 - 4.5	5.9	2.0 - 15.0	0.0	0.0 - 0.0	13.2	4.1 - 28.1	0.33
3050106	66315	BROAD R	144.4	19.4	7 - 48	0.0	0.0 - 0.0	2.4	0.8 - 5.5	0.6	0.2 - 2.4	2.6	0.8 - 7.4	0.0	0.0 - 0.0	13.9	4.6 - 34.0	0.34
3050106	66316	BROAD R	43.8	19.8	7 - 48	0.0	0.0 - 0.0	2.9	1.0 - 6.5	0.7	0.2 - 2.2	1.5	0.6 - 4.4	0.0	0.0 - 0.0	14.6	5.3 - 36.3	0.35
3050107	66317	TYGER R	37.3	20.4	7 - 49	0.0	0.0 - 0.0	2.3	0.8 - 5.5	0.6	0.2 - 1.6	2.9	1.0 - 7.8	0.0	0.0 - 0.0	14.6	4.9 - 38.9	0.34
3050108	66318	ENOREE R	179.8	22.1	8 - 55	0.0	0.0 - 0.0	3.2	1.1 - 7.7	0.8	0.3 - 2.8	2.7	1.1 - 7.0	0.0	0.0 - 0.0	15.4	5.7 - 41.0	0.33
3050105	66319	N PACOLET R	199.8	51.0	19 - 95	3.5	1.3 - 8.2	12.8	5.0 - 23.9	2.1	0.7 - 5.1	13.7	4.8 - 35.2	0.0	0.0 - 0.0	18.9	6.4 - 40.0	0.29
3050105	66320	PACOLET R	96.7	70.8	26 - 134	17.3	6.1 - 37.0	18.0	6.1 - 39.4	2.7	0.9 - 7.1	17.0	5.5 - 38.1	0.0	0.0 - 0.0	15.9	5.6 - 35.1	0.31
3050105	66321	LAWSONS FORK C	12.9	95.5	28 - 227	11.8	3.4 - 33.3	49.7	15.0 - 117.2	2.2	0.6 - 6.5	14.7	3.9 - 45.0	0.0	0.0 - 0.0	17.1	4.9 - 43.9	0.25
3050106	66322	BROAD R	18.7	19.4	6 - 41	0.0	0.0 - 0.0	3.4	1.1 - 6.5	1.1	0.3 - 2.8	1.6	0.5 - 3.5	0.0	0.0 - 0.0	13.3	4.1 - 30.7	0.40
3050105	66323	PACOLET R	54.0	60.1	16 - 146	10.3	2.8 - 28.7	28.9	7.4 - 72.5	1.1	0.3 - 3.5	6.4	1.9 - 17.9	0.0	0.0 - 0.0	13.4	3.8 - 35.3	0.31
3050106	66324	BROAD R	21.0	92.1	31 - 211	0.0	0.0 - 0.0	82.5	27.5 - 194.1	0.0	0.0 - 0.1	0.4	0.1 - 1.1	0.0	0.0 - 0.0	9.2	3.4 - 21.5	0.43
3050110	66325	CONGAREE R	10.0	20.5	6 - 48	0.0	0.0 - 0.0	0.3	0.1 - 0.5	0.1	0.0 - 0.3	1.0	0.4 - 2.1	0.0	0.0 - 0.0	19.1	5.9 - 46.7	0.43
3050207	66326	SALKEHATCHIE R	44.3	16.7	5 - 37	0.0	0.0 - 0.0	2.1	0.6 - 4.8	0.3	0.1 - 0.8	3.8	1.3 - 11.1	0.0	0.0 - 0.0	10.6	2.9 - 24.2	0.87
3050103	66327	CATAWBA R	67.3	65.8	21 - 120	0.0	0.0 - 0.0	49.0	16.1 - 101.2	1.1	0.3 - 2.9	4.6	1.5 - 11.4	0.0	0.0 - 0.0	11.1	3.5 - 26.4	0.28
3050103	66328	ROCKY CR	49.0	30.9	10 - 68	5.2	1.9 - 12.5	7.5	2.6 - 15.6	1.7	0.5 - 4.4	5.9	1.6 - 15.7	0.0	0.0 - 0.0	10.6	3.6 - 23.6	0.32
3050104	66329	WATEREE R	22.1	19.0	7 - 44	0.0	0.0 - 0.0	0.7	0.2 - 1.5	0.3	0.1 - 0.8	2.7	1.0 - 6.5	0.0	0.0 - 0.0	15.3	5.5 - 35.2	0.43
3050103	66330	MCALPINE CR	55.3	2383.1	781 - 6081	2280.3	736.9 - 5998.4	87.1	29.1 - 233.4	0.1	0.0 - 0.3	0.5	0.2 - 1.4	0.0	0.0 - 0.0	15.0	5.2 - 42.0	0.27
3050203	66331	EDISTO R, N FK	29.2	31.0	10 - 66	0.0	0.0 - 0.0	2.6	0.9 - 6.1	2.8	0.7 - 7.7	14.3	4.8 - 40.1	0.0	0.0 - 0.0	11.3	3.2 - 30.1	0.86
3050204	66332	EDISTO R, S FK	226.4	25.8	9 - 67	0.0	0.0 - 0.0	4.3	1.3 - 10.5	2.3	0.7 - 7.3	12.1	4.5 - 36.2	0.0	0.0 - 0.0	7.1	2.5 - 17.6	0.86
3050203	66333	EDISTO R, N FK	74.3	17.4	6 - 41	0.0	0.0 - 0.0	3.5	1.1 - 8.5	1.3	0.4 - 4.0	5.8	1.9 - 13.9	0.0	0.0 - 0.0	6.8	2.5 - 17.7	0.82

8-digit HUC	ID	Name	Area	Catchment Yield		Point sources		Developed Land		Manure		Agricultural Land		Phosphate Mines		Soil parent rock		Frac
				kg/km^2	90% CI	kg/km^2	90% CI	kg/km^2	90% CI	kg/km^2	90% CI	kg/km^2	90% CI	kg/km^2	90% CI	kg/km^2	90% CI	
3050205	66334	EDISTO R	282.5	34.9	16 - 84	0.0	0.0 - 0.0	2.3	0.9 - 5.9	1.0	0.3 - 2.8	4.6	1.8 - 14.1	0.0	0.0 - 0.0	27.1	11.2 - 68.6	0.96
3040206	66335	WACCAMAW R	141.5	72.0	22 - 159	23.7	7.4 - 52.1	20.6	6.1 - 51.2	1.9	0.5 - 5.1	9.4	2.7 - 22.2	0.0	0.0 - 0.0	16.4	4.6 - 37.7	0.97
3040201	66336	PEE DEE R	242.1	28.2	9 - 55	0.0	0.0 - 0.0	7.1	2.2 - 15.0	2.5	0.9 - 6.1	6.6	2.0 - 16.0	0.0	0.0 - 0.0	12.0	3.9 - 25.8	0.84
3040201	66337	PEE DEE R	1.8	44.6	14 - 114	0.0	0.0 - 0.0	15.9	5.2 - 39.1	1.4	0.4 - 5.0	14.4	4.5 - 42.2	0.0	0.0 - 0.0	12.9	4.6 - 33.1	0.85
3040201	66338	PEE DEE R	128.1	22.9	8 - 43	0.0	0.0 - 0.0	1.3	0.4 - 2.6	1.2	0.4 - 3.5	8.4	3.0 - 21.5	0.0	0.0 - 0.0	11.9	4.0 - 27.7	0.86
3040202	66339	LYNCHES R	55.2	32.3	11 - 83	0.0	0.0 - 0.0	5.0	1.8 - 11.4	1.4	0.4 - 4.0	17.1	5.5 - 39.4	0.0	0.0 - 0.0	8.8	2.9 - 25.1	0.83
3040204	66340	LITTLE PEE DEE R	44.4	40.5	14 - 77	0.0	0.0 - 0.0	4.4	1.3 - 9.3	3.4	0.8 - 9.8	20.5	7.0 - 43.5	0.0	0.0 - 0.0	12.4	3.9 - 33.1	0.96
3040205	66341	BLACK R	2.1	30.3	11 - 87	0.0	0.0 - 0.0	10.5	3.7 - 26.5	0.5	0.2 - 2.0	1.6	0.6 - 4.6	0.0	0.0 - 0.0	17.7	6.3 - 53.4	0.90
3180002	66342	PEARL R	79.4	132.0	44 - 321	0.7	0.2 - 1.7	6.8	2.4 - 17.1	8.4	2.3 - 27.8	26.8	8.2 - 81.9	0.0	0.0 - 0.0	89.4	29.0 - 223.8	0.41
3050207	66343	SALKEHATCHIE R	11.7	23.6	9 - 62	0.0	0.0 - 0.0	3.2	1.2 - 7.7	0.4	0.2 - 1.6	9.7	3.8 - 28.4	0.0	0.0 - 0.0	10.3	4.0 - 27.9	0.84
3040201	66344	PEE DEE R	155.4	31.1	11 - 75	0.1	0.1 - 0.3	2.1	0.7 - 6.0	1.6	0.5 - 4.6	14.1	5.5 - 35.7	0.0	0.0 - 0.0	13.1	4.5 - 35.4	0.99
3040201	66345	PEE DEE R	43.3	16.5	6 - 35	0.0	0.0 - 0.0	0.9	0.3 - 2.1	0.5	0.2 - 1.3	5.3	2.0 - 13.4	0.0	0.0 - 0.0	9.8	3.8 - 25.0	0.88
3040204	66347	LITTLE PEE DEE R	4.5	49.2	16 - 103	0.0	0.0 - 0.0	4.4	1.4 - 9.8	2.4	0.7 - 7.4	19.7	6.6 - 45.4	0.0	0.0 - 0.0	22.6	7.0 - 53.3	0.98
3040202	66348	LITTLE FORK CR	39.9	25.7	8 - 57	3.0	1.0 - 7.3	8.0	2.4 - 22.8	1.4	0.4 - 4.2	5.1	1.6 - 12.7	0.0	0.0 - 0.0	8.0	2.6 - 18.3	0.68
3040201	66349	BLACK CR	102.9	26.3	8 - 52	0.0	0.0 - 0.0	10.6	3.3 - 20.2	1.1	0.4 - 3.3	6.8	2.1 - 17.8	0.0	0.0 - 0.0	7.8	2.6 - 22.4	0.76
3040202	66350	LYNCHES R	284.8	49.8	15 - 104	14.6	4.1 - 33.4	3.5	1.1 - 8.2	1.6	0.4 - 4.9	20.1	6.0 - 49.9	0.0	0.0 - 0.0	9.9	3.2 - 24.1	0.86
3040201	66351	PEE DEE R	70.3	71.9	26 - 146	46.3	16.7 - 96.3	1.0	0.3 - 2.0	0.9	0.2 - 2.4	5.2	1.7 - 11.4	0.0	0.0 - 0.0	18.6	6.0 - 46.4	0.87
3040204	66352	LITTLE PEE DEE R	48.1	30.3	11 - 55	0.0	0.0 - 0.0	1.3	0.4 - 2.1	1.6	0.4 - 3.6	7.8	2.9 - 19.1	0.0	0.0 - 0.0	19.6	7.2 - 42.1	0.99
3040205	66353	TIMBER CR	263.0	21.6	7 - 45	0.0	0.0 - 0.0	3.6	1.3 - 7.4	1.1	0.3 - 2.9	7.0	2.4 - 18.7	0.0	0.0 - 0.0	9.9	3.0 - 23.6	0.75
3050109	66355	REEDY R	125.2	103.0	34 - 261	0.1	0.0 - 0.3	81.6	26.7 - 217.9	0.5	0.1 - 1.9	3.2	1.0 - 8.1	0.0	0.0 - 0.0	17.6	6.0 - 45.8	0.05
3050109	66356	REEDY R	1.1	32.8	10 - 57	0.0	0.0 - 0.0	4.2	1.2 - 7.0	2.5	0.6 - 6.7	6.4	1.9 - 12.1	0.0	0.0 - 0.0	19.7	6.0 - 39.6	0.06
3050109	66357	SALADA R	4.5	26.0	8 - 72	0.0	0.0 - 0.0	6.6	2.1 - 16.1	3.4	0.9 - 10.3	3.2	0.8 - 9.6	0.0	0.0 - 0.0	12.9	4.2 - 37.7	0.12
3050109	66358	LITTLE SALUDA R	8.3	144.0	52 - 244	76.7	27.2 - 146.3	30.1	10.6 - 52.5	9.8	2.7 - 19.6	12.6	4.4 - 24.3	0.0	0.0 - 0.0	14.8	4.8 - 28.1	0.11
3050109	66359	REEDY R	158.3	107.6	36 - 230	27.2	8.8 - 68.9	58.2	19.2 - 115.8	0.9	0.3 - 2.2	5.9	2.0 - 17.2	0.0	0.0 - 0.0	15.4	4.9 - 38.9	0.06
3050109	66360	MID SALUDA R	56.6	24.0	8 - 56	0.0	0.0 - 0.0	4.9	1.4 - 11.5	0.2	0.1 - 0.5	1.3	0.4 - 4.1	0.0	0.0 - 0.0	17.6	5.9 - 44.5	0.03
3050109	66361	BUSH R	9.4	27.8	8 - 68	0.0	0.0 - 0.0	5.7	1.7 - 15.3	4.1	1.1 - 14.2	5.8	1.8 - 16.9	0.0	0.0 - 0.0	12.3	3.6 - 38.3	0.12
3050109	66362	LITTLE SALUDA R	1.7	74.2	23 - 123	0.0	0.0 - 0.0	5.5	1.9 - 10.0	23.0	5.9 - 49.6	30.3	9.4 - 61.9	0.0	0.0 - 0.0	15.4	4.0 - 31.6	0.12
3050109	66363	SALADA R	42.6	319.9	113 - 769	290.8	103.0 - 696.8	9.6	3.1 - 21.5	2.3	0.7 - 6.8	6.0	2.0 - 16.0	0.0	0.0 - 0.0	11.3	3.8 - 29.3	0.06
3050109	66364	SALADA R	14.8	41.3	14 - 116	0.0	0.0 - 0.0	16.4	5.4 - 38.7	2.6	0.8 - 9.6	5.6	1.9 - 16.1	0.0	0.0 - 0.0	16.8	5.7 - 70.6	0.06
3050109	66365	GROVE CR	52.8	108.4	33 - 252	50.3	15.8 - 119.5	35.2	9.6 - 69.4	1.0	0.3 - 3.3	5.9	1.8 - 15.4	0.0	0.0 - 0.0	16.1	4.7 - 38.5	0.05
3050109	66366	SALADA R	107.7	17.2	5 - 47	0.0	0.0 - 0.0	4.2	1.1 - 10.7	1.3	0.3 - 3.7	2.7	0.8 - 8.1	0.0	0.0 - 0.0	9.0	2.4 - 25.8	0.12
3050109	66367	SALADA R	20.6	22.0	8 - 39	0.0	0.0 - 0.0	5.4	2.2 - 12.2	2.0	0.7 - 4.6	1.8	0.6 - 4.4	0.0	0.0 - 0.0	12.8	4.8 - 27.6	0.12
3050109	66368	LITTLE R	25.5	29.1	10 - 69	0.0	0.0 - 0.0	4.8	1.6 - 12.0	5.3	1.6 - 14.1	6.8	2.1 - 19.4	0.0	0.0 - 0.0	12.2	4.1 - 30.9	0.12
3050109	66369	S RABON CR	72.0	42.8	12 - 96	0.0	0.0 - 0.0	12.4	3.8 - 27.9	2.8	0.9 - 7.0	15.2	4.6 - 42.2	0.0	0.0 - 0.0	12.5	4.0 - 31.8	0.06
3060101	66370	CONECROSS CR	192.9	75.3	23 - 157	21.1	6.8 - 48.1	18.5	6.2 - 42.8	11.1	3.3 - 31.4	12.1	4.2 - 32.0	0.0	0.0 - 0.0	12.4	4.5 - 28.6	0.12
3060101	66371	TWELVEMILE CR	7.1	37.1	13 - 66	0.0	0.0 - 0.0	9.7	3.5 - 18.9	2.6	0.8 - 6.9	10.0	3.5 - 23.1	0.0	0.0 - 0.0	14.8	5.0 - 32.9	0.13
3060103	66372	SAVANNAH R	2.8	33.8	11 - 84	0.0	0.0 - 0.0	4.3	1.3 - 9.8	5.5	1.6 - 17.2	3.9	1.4 - 10.1	0.0	0.0 - 0.0	20.2	6.1 - 61.4	0.41
3060106	66373	SAVANNAH R	155.8	81.1	22 - 185	54.6	15.8 - 124.9	2.9	0.9 - 6.7	1.0	0.3 - 2.9	14.3	4.0 - 40.1	0.0	0.0 - 0.0	8.3	2.4 - 21.7	0.96
3060101	66374	EIGHTEENMILE CI	13.4	80.7	30 - 170	0.0	0.0 - 0.0	61.4	21.5 - 127.4	1.2	0.4 - 4.0	3.7	1.3 - 9.7	0.0	0.0 - 0.0	14.4	5.7 - 37.8	0.12

8-digit HUC	ID	Name	Area	Catchment Yield		Point sources		Developed Land		Manure		Agricultural Land		Phosphate Mines		Soil parent rock		Frac
				kg/km^2	90% CI	kg/km^2	90% CI	kg/km^2	90% CI	kg/km^2	90% CI	kg/km^2	90% CI	kg/km^2	90% CI	kg/km^2	90% CI	
3060101	66375	TWELVEMILE CR	39.6	40.9	14 - 90	0.8	0.2 - 1.8	11.4	3.8 - 25.8	2.5	0.7 - 7.3	11.5	3.9 - 26.4	0.0	0.0 - 0.0	14.8	4.9 - 32.9	0.13
3060106	66377	SAVANNAH R	246.4	263.1	100 - 687	204.1	78.4 - 524.4	42.9	14.0 - 104.2	0.9	0.3 - 2.3	3.4	1.1 - 9.2	0.0	0.0 - 0.0	11.8	4.6 - 32.6	0.93
3060106	66378	FOURMILE CR	42.2	16.1	5 - 38	0.0	0.0 - 0.0	10.3	3.5 - 23.3	0.1	0.0 - 0.4	0.0	0.0 - 0.0	0.0	0.0 - 0.0	5.7	2.0 - 13.7	0.80
3060106	66379	LOWER THREE RU	159.6	8.7	3 - 21	0.0	0.0 - 0.0	1.0	0.3 - 2.2	0.1	0.0 - 0.3	1.0	0.4 - 2.3	0.0	0.0 - 0.0	6.6	2.4 - 16.9	0.07
3060103	66380	ROCKY R	20.5	40.8	13 - 91	0.0	0.0 - 0.0	7.8	2.5 - 16.4	4.9	1.4 - 13.5	14.0	4.4 - 39.3	0.0	0.0 - 0.0	14.2	4.9 - 35.9	0.15
6010102	66382	BEAVER CR	45.1	69.5	20 - 125	0.0	0.0 - 0.0	46.4	13.0 - 90.1	3.3	0.9 - 8.1	9.6	2.8 - 21.3	0.0	0.0 - 0.0	10.2	3.2 - 23.0	0.06
6020002	66383	HIWASSEE R	1.6	10999.2	3491 - 26200	########	3480.6 - 2611	15.3	4.4 - 29.2	3.7	1.0 - 9.2	4.8	1.4 - 11.7	0.0	0.0 - 0.0	14.6	4.5 - 34.6	0.37
6020001	66384	TENNESSEE R	39.4	12.9	3 - 27	0.0	0.0 - 0.0	1.3	0.3 - 2.5	0.3	0.1 - 0.9	0.7	0.2 - 1.6	0.0	0.0 - 0.0	10.6	2.6 - 24.5	0.43
6010202	66385	LITTLE TENNESSE	75.9	13.1	5 - 24	0.0	0.0 - 0.0	4.2	1.6 - 8.5	0.1	0.0 - 0.3	0.5	0.2 - 1.4	0.0	0.0 - 0.0	8.3	3.0 - 17.3	0.14
6010104	66386	HOLSTON R	119.4	58.0	17 - 115	11.2	3.6 - 25.6	14.4	4.3 - 33.8	6.3	1.7 - 16.2	16.4	4.7 - 51.5	0.0	0.0 - 0.0	9.7	2.8 - 23.6	0.12
6010106	66387	PIGEON R	87.0	35.9	11 - 66	0.0	0.0 - 0.0	9.8	3.2 - 19.2	4.7	1.3 - 13.5	11.8	3.7 - 28.9	0.0	0.0 - 0.0	9.6	3.0 - 22.8	0.15
6010206	66388	POWELL R	7.1	34.8	10 - 66	0.0	0.0 - 0.0	13.2	4.0 - 25.5	6.5	2.0 - 19.0	4.6	1.3 - 10.6	0.0	0.0 - 0.0	10.5	3.6 - 25.6	0.11
6020004	66389	SEQUATCHIE R	22.3	63.8	24 - 173	0.0	0.0 - 0.0	10.0	3.5 - 25.0	11.0	3.3 - 34.9	29.8	9.6 - 83.7	0.0	0.0 - 0.0	13.0	4.4 - 37.7	0.42
6010208	66390	EMORY R	39.7	29.2	10 - 79	0.0	0.0 - 0.0	12.1	4.4 - 29.7	1.5	0.4 - 4.1	3.5	1.0 - 10.5	0.0	0.0 - 0.0	12.1	3.8 - 35.4	0.29
6020002	66391	HIWASSEE R	1.5	89.8	26 - 158	0.0	0.0 - 0.0	53.0	15.6 - 97.5	8.5	2.4 - 20.5	11.5	3.5 - 32.8	0.0	0.0 - 0.0	16.7	5.5 - 37.8	0.36
6020003	66392	OCOEE R	1.5	26.9	10 - 67	0.0	0.0 - 0.0	10.0	4.1 - 22.9	0.0	0.0 - 0.0	0.0	0.0 - 0.0	0.0	0.0 - 0.0	16.9	6.5 - 54.4	0.36
6010205	66393	CLINCH R	131.0	24.3	7 - 54	0.0	0.0 - 0.0	5.8	1.6 - 12.9	3.4	0.9 - 8.7	8.8	2.6 - 24.5	0.0	0.0 - 0.0	6.4	1.9 - 15.6	0.10
6010102	66394	BEAVER CR	69.3	57.1	17 - 102	0.0	0.0 - 0.0	13.2	3.8 - 27.2	10.0	2.6 - 28.2	23.5	7.7 - 51.6	0.0	0.0 - 0.0	10.4	3.3 - 24.0	0.06
6010101	66395	HOLSTON R, N FK	97.6	30.8	12 - 69	3.5	1.5 - 8.1	5.4	1.9 - 12.8	3.8	1.3 - 9.7	9.6	3.5 - 24.2	0.0	0.0 - 0.0	8.4	3.8 - 20.9	0.11
6010101	66396	HOLSTON R, N FK	38.8	39.2	13 - 68	0.0	0.0 - 0.0	2.8	0.9 - 4.8	7.2	2.1 - 17.5	18.3	5.4 - 38.1	0.0	0.0 - 0.0	10.9	3.3 - 22.5	0.10
6010102	66397	HOLSTON R, S FK	14.1	31.1	10 - 60	0.0	0.0 - 0.0	3.2	1.2 - 6.8	5.7	1.9 - 17.1	12.9	4.6 - 26.2	0.0	0.0 - 0.0	9.3	3.0 - 25.0	0.03
6010102	66398	HOLSTON R, S FK	199.0	20.7	7 - 49	0.0	0.0 - 0.0	3.3	1.1 - 6.3	2.6	0.7 - 6.7	6.8	2.0 - 16.2	0.0	0.0 - 0.0	8.2	2.7 - 19.8	0.03
3120003	66399	TELOGIA CR	314.5	30.7	9 - 79	0.0	0.0 - 0.0	7.7	2.2 - 16.2	0.9	0.3 - 2.6	7.3	2.0 - 19.5	0.0	0.0 - 0.0	14.8	4.6 - 38.9	0.89
3080101	66400	BLACK WATER CR	80.0	9.3	3 - 19	0.0	0.0 - 0.0	1.8	0.4 - 4.4	0.5	0.1 - 1.4	2.9	0.7 - 7.4	0.0	0.0 - 0.0	4.1	1.2 - 9.5	0.91
3080101	66401	ALEXANDER SPRI	553.2	10.8	4 - 18	0.0	0.0 - 0.0	1.7	0.5 - 3.2	0.6	0.2 - 1.4	1.9	0.6 - 4.9	0.0	0.0 - 0.0	6.6	2.2 - 12.4	0.89
3080103	66402	BLACK CR, N FK	82.7	54.4	16 - 101	0.0	0.0 - 0.0	33.2	9.6 - 67.0	0.9	0.2 - 2.4	1.5	0.4 - 3.6	0.0	0.0 - 0.0	18.7	6.5 - 41.0	0.96
3080101	66403	ST JOHNS R	38.7	7.8	2 - 27	0.0	0.0 - 0.0	0.0	0.0 - 0.1	0.9	0.2 - 3.9	3.7	0.7 - 15.4	0.0	0.0 - 0.0	3.1	0.7 - 11.6	0.83
3060109	66404	EBENEZER CR	135.6	29.2	8 - 61	6.0	1.7 - 11.9	5.9	1.7 - 11.7	0.6	0.2 - 1.5	5.3	1.8 - 11.7	0.0	0.0 - 0.0	11.3	3.0 - 28.9	0.96
3130002	66405	CHATTAHOOCHEE	140.8	23.1	8 - 40	0.0	0.0 - 0.0	5.1	1.8 - 9.0	1.4	0.4 - 3.4	4.2	1.4 - 8.9	0.0	0.0 - 0.0	12.3	4.6 - 24.7	0.18
3170005	66406	LEAF R	14.1	30.1	11 - 65	0.0	0.0 - 0.0	13.2	4.4 - 29.4	2.0	0.7 - 6.1	3.1	1.0 - 7.3	0.0	0.0 - 0.0	11.8	4.1 - 30.4	0.97
3170001	66407	OKATIBBEE CR	21.5	27.9	11 - 48	0.0	0.0 - 0.0	14.8	5.5 - 29.5	1.0	0.3 - 2.6	3.8	1.4 - 9.4	0.0	0.0 - 0.0	8.3	3.3 - 16.5	0.84
3040105	66408	ROCKY R	9.1	61.2	23 - 131	0.0	0.0 - 0.0	2.5	0.8 - 4.8	21.1	6.5 - 56.2	21.8	7.1 - 50.1	0.0	0.0 - 0.0	15.9	5.3 - 35.2	0.78
3050103	66409	CATAWBA R	62.6	23.7	6 - 53	0.0	0.0 - 0.0	5.3	1.7 - 12.5	1.3	0.3 - 4.1	3.8	1.2 - 10.0	0.0	0.0 - 0.0	13.3	3.5 - 31.8	0.29
3050104	66410	WATEREE R	44.4	47.1	17 - 129	21.6	7.8 - 61.0	6.0	2.1 - 17.0	2.6	0.8 - 9.6	5.1	1.9 - 14.0	0.0	0.0 - 0.0	11.7	3.8 - 37.5	0.42
3050103	66411	FISHING CR	3.5	31.6	13 - 64	0.0	0.0 - 0.0	15.7	5.4 - 32.8	0.8	0.3 - 2.3	2.7	1.0 - 7.5	0.0	0.0 - 0.0	12.4	4.7 - 35.1	0.29
3050103	66412	CATAWBA R	2.2	27.1	10 - 55	0.0	0.0 - 0.0	12.3	4.1 - 25.2	0.2	0.1 - 0.4	0.8	0.3 - 1.9	0.0	0.0 - 0.0	13.8	5.0 - 34.5	0.32
3050103	66413	CATAWBA R	13.5	20.6	8 - 43	0.0	0.0 - 0.0	7.5	2.8 - 15.0	0.7	0.3 - 2.3	2.6	0.9 - 7.0	0.0	0.0 - 0.0	9.8	3.9 - 23.4	0.32
3050109	66414	SALADA R	13.7	72.0	22 - 148	0.0	0.0 - 0.0	38.2	10.3 - 83.7	3.7	1.1 - 9.9	11.0	3.5 - 26.7	0.0	0.0 - 0.0	19.1	6.2 - 42.4	0.05
3050109	66415	SALADA R	102.2	39.0	12 - 101	0.0	0.0 - 0.0	12.1	3.5 - 28.0	2.3	0.6 - 6.5	11.0	3.6 - 31.6	0.0	0.0 - 0.0	13.5	4.3 - 33.3	0.04

8-digit HUC	ID	Name	Area	Catchment Yield		Point sources		Developed Land		Manure		Agricultural Land		Phosphate Mines		Soil parent rock		Frac
				kg/km^2	90% CI	kg/km^2	90% CI	kg/km^2	90% CI	kg/km^2	90% CI	kg/km^2	90% CI	kg/km^2	90% CI	kg/km^2	90% CI	
3060106	66416	UPPER THREE RUN	20.9	9.3	3 - 22	0.0	0.0 - 0.0	1.4	0.5 - 3.2	0.2	0.1 - 0.8	0.0	0.0 - 0.0	0.0	0.0 - 0.0	7.7	2.4 - 19.7	0.91
3040205	66417	BLACK R	219.8	40.8	14 - 105	12.2	4.4 - 30.5	6.8	2.4 - 15.9	0.9	0.3 - 3.0	13.8	4.4 - 38.9	0.0	0.0 - 0.0	7.2	2.3 - 21.2	0.90
3040205	66419	BLACK R	515.3	25.2	9 - 43	0.0	0.0 - 0.0	3.4	1.3 - 6.5	1.0	0.3 - 2.7	11.0	3.8 - 24.7	0.0	0.0 - 0.0	9.8	3.3 - 23.4	0.96
3020201	66420	MOUNTAIN CR	20.7	39.9	17 - 102	0.0	0.0 - 0.0	7.8	3.0 - 18.4	2.4	0.9 - 7.3	17.2	6.4 - 48.8	0.0	0.0 - 0.0	12.5	5.1 - 32.2	0.25
3020201	66421	FLAT R	8.3	19.9	5 - 48	0.0	0.0 - 0.0	2.7	0.8 - 6.0	0.7	0.2 - 1.8	4.1	1.3 - 11.2	0.0	0.0 - 0.0	12.4	3.3 - 33.9	0.20
3110201	66422	SUWANNEE R	57.4	10.2	3 - 21	0.0	0.0 - 0.0	2.8	0.9 - 6.2	0.0	0.0 - 0.1	0.1	0.0 - 0.1	0.0	0.0 - 0.0	7.3	2.4 - 17.6	0.86
3020201	66423	FLAT R	57.2	28.3	10 - 58	0.0	0.0 - 0.0	4.6	1.6 - 10.8	1.6	0.5 - 4.5	10.4	3.7 - 26.6	0.0	0.0 - 0.0	11.7	3.7 - 29.4	0.20
3040205	66424	BLACK R	267.1	29.1	11 - 57	0.0	0.0 - 0.0	4.9	1.6 - 9.1	1.1	0.3 - 2.9	15.4	5.5 - 32.9	0.0	0.0 - 0.0	7.6	2.6 - 17.5	0.92
3070204	66426	ST MARYS R, M PR	290.8	9.6	4 - 25	0.0	0.0 - 0.0	1.9	0.7 - 4.6	0.1	0.0 - 0.3	0.1	0.0 - 0.2	0.0	0.0 - 0.0	7.5	2.6 - 19.6	0.82
3080101	66427	ST JOHNS R	43.3	79.5	19 - 221	0.0	0.0 - 0.0	1.4	0.4 - 2.9	0.0	0.0 - 0.1	0.0	0.0 - 0.0	0.0	0.0 - 0.0	78.1	18.3 - 221.3	0.90
3100205	66428	HILLSBOROUGH R	212.8	80.4	25 - 199	0.0	0.0 - 0.0	21.5	6.7 - 51.3	6.5	1.7 - 20.1	17.4	5.1 - 50.1	5.2	1.5 - 14.7	29.9	8.6 - 76.4	0.87
3100206	66429	ROCKY CR	51.7	18.8	6 - 37	0.0	0.0 - 0.0	9.8	2.6 - 22.2	0.5	0.1 - 1.4	1.1	0.3 - 2.8	0.0	0.0 - 0.0	7.4	2.3 - 16.1	0.97
3060106	66437	SAVANNAH R	23.2	203.4	71 - 465	147.3	48.9 - 353.2	40.3	14.6 - 88.4	0.2	0.1 - 0.7	2.8	0.9 - 7.1	0.0	0.0 - 0.0	12.7	4.4 - 33.0	0.92
3130002	66442	CHATTAHOOCHEE	23.4	25.7	11 - 56	0.0	0.0 - 0.0	10.2	3.8 - 20.6	0.6	0.2 - 1.6	1.8	0.7 - 4.5	0.0	0.0 - 0.0	13.2	4.8 - 32.4	0.30
3160106	66443	TOMBIGBEE R	7.6	33.6	10 - 77	0.0	0.0 - 0.0	2.0	0.7 - 4.9	7.0	1.9 - 20.6	8.0	2.5 - 20.4	0.0	0.0 - 0.0	16.6	4.5 - 39.6	0.87
3130006	66446	FLINT R	145.4	26.3	8 - 62	0.0	0.0 - 0.0	2.7	0.7 - 6.4	1.8	0.4 - 6.9	11.8	3.2 - 29.7	0.0	0.0 - 0.0	10.0	2.4 - 27.4	0.49
3080101	66450	0	18.3	41.3	16 - 82	0.0	0.0 - 0.0	0.9	0.3 - 1.7	2.1	0.6 - 5.5	10.3	3.5 - 24.1	0.0	0.0 - 0.0	28.1	10.3 - 61.2	0.80
3080101	66451	0	648.9	2.5	0 - 10	0.0	0.0 - 0.0	0.1	0.0 - 0.4	0.3	0.0 - 1.3	1.2	0.2 - 6.0	0.0	0.0 - 0.0	0.9	0.2 - 3.4	0.82
3080102	66452	0	210.6	19.7	7 - 39	0.0	0.0 - 0.0	3.3	1.0 - 6.9	1.7	0.5 - 4.6	6.3	2.0 - 18.0	0.0	0.0 - 0.0	8.3	3.0 - 19.2	0.59
3080102	66453	0	4.9	62.4	17 - 144	0.0	0.0 - 0.0	1.4	0.4 - 3.1	6.0	1.6 - 17.8	12.6	3.8 - 34.9	0.0	0.0 - 0.0	42.5	11.9 - 112.7	0.58
3080102	66454	0	81.3	10.8	3 - 27	0.0	0.0 - 0.0	0.5	0.1 - 1.4	0.4	0.1 - 1.1	0.4	0.1 - 1.4	0.0	0.0 - 0.0	9.5	2.7 - 22.6	0.57
3080102	66455	0	139.9	38.9	13 - 90	0.0	0.0 - 0.0	2.7	0.9 - 4.9	3.1	0.8 - 7.5	4.9	1.6 - 12.3	0.0	0.0 - 0.0	28.2	9.3 - 71.7	0.58
3080102	66456	0	128.2	46.7	13 - 116	0.0	0.0 - 0.0	16.8	5.1 - 40.6	1.2	0.3 - 4.0	1.0	0.3 - 3.1	0.2	0.1 - 0.7	27.4	8.4 - 78.6	0.55
3080101	66457	ST JOHNS R	39.6	9.3	2 - 25	0.0	0.0 - 0.0	0.2	0.0 - 0.5	0.9	0.2 - 3.4	3.9	0.8 - 12.7	0.0	0.0 - 0.0	4.2	1.0 - 11.9	0.83
3060101	66458	EIGHTEENMILE CR	109.1	50.1	18 - 122	1.0	0.3 - 2.9	23.3	7.3 - 52.9	2.2	0.6 - 6.7	8.9	2.7 - 21.9	0.0	0.0 - 0.0	14.6	4.8 - 39.0	0.11
3110201	66459	SUWANNEE R, M F	185.0	7.2	2 - 14	0.0	0.0 - 0.0	1.3	0.4 - 2.6	0.3	0.1 - 0.9	0.7	0.2 - 1.6	0.0	0.0 - 0.0	5.0	1.7 - 10.5	0.76
3110201	66460	SUWANNEE CR	39.6	0.3	0 - 1	0.0	0.0 - 0.0	0.0	0.0 - 0.1	0.0	0.0 - 0.0	0.0	0.0 - 0.0	0.0	0.0 - 0.0	0.3	0.1 - 0.7	0.84
3080101	66461	0	30.6	0.1	0 - 1	0.0	0.0 - 0.0	0.0	0.0 - 0.0	0.0	0.0 - 0.1	0.1	0.0 - 0.5	0.0	0.0 - 0.0	0.1	0.0 - 0.7	0.79
3080102	66462	0	1802.0	40.5	14 - 63	2.9	1.1 - 5.1	11.9	3.9 - 21.8	2.8	0.8 - 6.9	8.3	2.8 - 20.0	0.0	0.0 - 0.0	14.6	5.6 - 29.1	0.55
3010203	81014	CHOWAN R	10.1	19.8	6 - 38	0.0	0.0 - 0.0	0.2	0.1 - 0.5	3.0	0.9 - 7.8	7.9	2.6 - 21.3	0.0	0.0 - 0.0	8.6	2.5 - 22.0	1.00
3010205	81015	CURRITUNK SOUN	177.7	1.6	0 - 5	0.0	0.0 - 0.0	0.0	0.0 - 0.2	0.0	0.0 - 0.0	0.8	0.1 - 3.2	0.0	0.0 - 0.0	0.7	0.1 - 1.9	1.00
3010205	81016	NORTH R	19.9	0.0	0 - 0	0.0	0.0 - 0.0	0.0	0.0 - 0.0	0.0	0.0 - 0.0	0.0	0.0 - 0.0	0.0	0.0 - 0.0	0.0	0.0 - 0.0	1.00
3010205	81017	ALBEMARLE SOUI	126.5	63.7	21 - 123	0.0	0.0 - 0.0	0.0	0.0 - 0.1	0.3	0.1 - 0.7	25.3	8.2 - 53.3	0.0	0.0 - 0.0	38.0	11.3 - 90.0	1.00
3010205	81018	LITTLE R	165.7	120.1	33 - 302	0.0	0.0 - 0.0	0.3	0.1 - 0.7	6.4	1.9 - 19.0	79.2	20.8 - 203.7	0.0	0.0 - 0.0	34.1	10.3 - 86.9	1.00
3010205	81019	PERQUIMANS R	76.1	93.0	34 - 209	3.2	1.2 - 8.8	2.0	0.7 - 4.5	6.7	2.3 - 18.9	49.9	17.0 - 146.2	0.0	0.0 - 0.0	31.2	11.2 - 86.4	1.00
3010205	81020	ALBEMARLE SOUI	656.8	60.1	19 - 106	0.0	0.0 - 0.0	1.1	0.4 - 2.1	5.1	1.5 - 11.0	31.7	9.6 - 64.6	0.0	0.0 - 0.0	22.2	7.1 - 51.4	1.00
3010205	81021	ALBEMARLE SOUI	56.7	48.7	20 - 102	0.0	0.0 - 0.0	0.2	0.1 - 0.5	9.1	3.2 - 25.4	23.4	8.0 - 58.0	0.0	0.0 - 0.0	16.0	5.7 - 40.4	1.00
3010205	81022	ALBEMARLE SOUI	304.0	23.4	7 - 51	0.0	0.0 - 0.0	0.8	0.2 - 2.1	0.7	0.2 - 2.7	6.8	1.8 - 15.3	0.0	0.0 - 0.0	15.0	4.2 - 39.4	1.00
3010205	81023	SCUPPERNONG R	111.9	6.4	2 - 17	5.0	1.5 - 13.4	0.1	0.0 - 0.5	0.0	0.0 - 0.0	0.7	0.1 - 2.7	0.0	0.0 - 0.0	0.7	0.1 - 2.5	1.00

8-digit HUC	ID	Name	Area	Catchment Yield		Point sources		Developed Land		Manure		Agricultural Land		Phosphate Mines		Soil parent rock		Frac
				kg/km^2	90% CI	kg/km^2	90% CI	kg/km^2	90% CI	kg/km^2	90% CI	kg/km^2	90% CI	kg/km^2	90% CI	kg/km^2	90% CI	
3010205	81024	ALBEMARLE SOU	275.8	2.9	1 - 7	0.0	0.0 - 0.0	0.1	0.0 - 0.3	0.0	0.0 - 0.0	0.2	0.1 - 0.7	0.0	0.0 - 0.0	2.6	0.7 - 6.3	1.00
3010205	81025	ALLIGATOR R	41.8	0.1	0 - 1	0.0	0.0 - 0.0	0.0	0.0 - 0.1	0.0	0.0 - 0.0	0.0	0.0 - 0.2	0.0	0.0 - 0.0	0.1	0.0 - 0.5	1.00
3020104	81026	PAMLICO R	55.1	0.1	0 - 0	0.0	0.0 - 0.0	0.0	0.0 - 0.0	0.0	0.0 - 0.0	0.0	0.0 - 0.1	0.0	0.0 - 0.0	0.1	0.0 - 0.3	1.00
3020104	81027	PUNGO R	112.8	20.9	6 - 52	0.0	0.0 - 0.0	1.0	0.3 - 2.4	1.5	0.3 - 4.5	9.0	2.3 - 25.1	0.0	0.0 - 0.0	9.6	2.6 - 24.7	1.00
3020104	81028	PAMLICO R	53.2	0.7	0 - 3	0.0	0.0 - 0.0	0.0	0.0 - 0.1	0.0	0.0 - 0.0	0.1	0.0 - 0.3	0.0	0.0 - 0.0	0.6	0.1 - 2.7	1.00
3020104	81029	PAMLICO R	227.3	47.2	14 - 114	0.0	0.0 - 0.0	5.9	1.6 - 12.5	3.1	0.9 - 8.3	18.3	5.5 - 50.9	0.0	0.0 - 0.0	19.8	5.8 - 51.3	1.00
3020105	81030	PAMLICO SOUND	646.7	0.0	0 - 0	0.0	0.0 - 0.0	0.0	0.0 - 0.0	0.0	0.0 - 0.0	0.0	0.0 - 0.0	0.0	0.0 - 0.0	0.0	0.0 - 0.0	1.00
3020105	81031	PAMLICO SOUND	1.1	0.1	0 - 1	0.0	0.0 - 0.0	0.0	0.0 - 0.0	0.0	0.0 - 0.0	0.0	0.0 - 0.0	0.0	0.0 - 0.0	0.1	0.0 - 0.6	1.00
3020105	81032	PAMLICO SOUND	223.5	3.5	1 - 9	0.0	0.0 - 0.0	0.0	0.0 - 0.1	0.0	0.0 - 0.0	0.8	0.2 - 2.9	0.0	0.0 - 0.0	2.7	0.7 - 7.3	1.00
3020105	81033	PAMLICO SOUND	371.3	0.5	0 - 2	0.0	0.0 - 0.0	0.0	0.0 - 0.0	0.0	0.0 - 0.0	0.1	0.0 - 0.5	0.0	0.0 - 0.0	0.4	0.1 - 1.5	1.00
3020105	81034	PAMLICO SOUND	126.0	0.9	0 - 4	0.0	0.0 - 0.0	0.0	0.0 - 0.2	0.0	0.0 - 0.0	0.3	0.0 - 1.3	0.0	0.0 - 0.0	0.6	0.1 - 2.8	1.00
3020105	81035	PAMLICO SOUND	346.9	14.9	4 - 38	0.0	0.0 - 0.0	0.9	0.3 - 2.5	0.2	0.1 - 0.8	6.9	2.0 - 22.2	0.0	0.0 - 0.0	6.9	2.0 - 17.5	1.00
3020204	81037	NEUSE R	486.9	19.2	7 - 40	0.0	0.0 - 0.0	1.7	0.6 - 3.9	0.2	0.0 - 0.5	6.2	2.1 - 15.8	0.0	0.0 - 0.0	11.1	3.8 - 24.8	1.00
3020204	81038	NEUSE R	28.9	488.6	173 - 1176	429.0	155.1 - 1039.5	45.3	14.9 - 105.9	0.7	0.2 - 1.8	1.5	0.5 - 3.6	0.0	0.0 - 0.0	12.2	4.4 - 28.5	1.00
3030001	81039	ONSLOW BAY	863.7	5.0	2 - 11	0.4	0.1 - 0.9	1.4	0.4 - 3.8	0.8	0.2 - 2.5	0.7	0.2 - 1.7	0.0	0.0 - 0.0	1.8	0.6 - 4.3	1.00
3030001	81040	ONSLOW BAY	408.5	11.4	3 - 22	0.3	0.1 - 0.6	8.2	2.1 - 17.4	0.1	0.0 - 0.3	0.5	0.1 - 1.0	0.0	0.0 - 0.0	2.4	0.7 - 4.9	1.00
3040207	81041	LONG BAY	111.7	0.1	0 - 1	0.0	0.0 - 0.0	0.0	0.0 - 0.2	0.0	0.0 - 0.0	0.0	0.0 - 0.0	0.0	0.0 - 0.0	0.1	0.0 - 0.5	1.00
3040207	81042	LONG BAY	486.2	4.1	1 - 10	0.0	0.0 - 0.0	2.6	0.7 - 7.3	0.0	0.0 - 0.0	0.0	0.0 - 0.0	0.0	0.0 - 0.0	1.5	0.5 - 3.7	1.00
3040207	81043	LONG BAY	434.6	0.6	0 - 2	0.0	0.0 - 0.0	0.1	0.0 - 0.4	0.0	0.0 - 0.0	0.0	0.0 - 0.0	0.0	0.0 - 0.0	0.5	0.1 - 2.2	1.00
3050112	81045	ATLANTIC OCEAN	32.7	0.0	0 - 0	0.0	0.0 - 0.0	0.0	0.0 - 0.0	0.0	0.0 - 0.0	0.0	0.0 - 0.0	0.0	0.0 - 0.0	0.0	0.0 - 0.3	1.00
3050112	81046	ATLANTIC OCEAN	5.4	0.0	0 - 0	0.0	0.0 - 0.0	0.0	0.0 - 0.0	0.0	0.0 - 0.0	0.0	0.0 - 0.0	0.0	0.0 - 0.0	0.0	0.0 - 0.3	1.00
3050201	81048	COOPER R	15.5	11.0	4 - 27	10.5	4.3 - 27.0	0.0	0.0 - 0.2	0.0	0.0 - 0.0	0.1	0.0 - 0.6	0.0	0.0 - 0.0	0.4	0.0 - 2.0	1.00
3050202	81049	ATLANTIC OCEAN	146.2	0.0	0 - 0	0.0	0.0 - 0.0	0.0	0.0 - 0.0	0.0	0.0 - 0.0	0.0	0.0 - 0.0	0.0	0.0 - 0.0	0.0	0.0 - 0.3	1.00
3050202	81050	ASHLEY R	40.7	24.9	7 - 62	0.0	0.0 - 0.0	13.0	3.6 - 32.9	0.0	0.0 - 0.1	0.5	0.1 - 1.4	0.0	0.0 - 0.0	11.4	3.5 - 34.6	1.00
3050205	81051	ATLANTIC OCEAN	7.8	0.0	0 - 0	0.0	0.0 - 0.0	0.0	0.0 - 0.0	0.0	0.0 - 0.0	0.0	0.0 - 0.0	0.0	0.0 - 0.0	0.0	0.0 - 0.2	1.00
3050205	81052	ATLANTIC OCEAN	159.2	0.1	0 - 1	0.0	0.0 - 0.0	0.0	0.0 - 0.0	0.0	0.0 - 0.0	0.0	0.0 - 0.0	0.0	0.0 - 0.0	0.1	0.0 - 1.0	1.00
3050208	81053	ST HELENA SOUN	72.0	0.1	0 - 1	0.0	0.0 - 0.0	0.0	0.0 - 0.0	0.0	0.0 - 0.0	0.0	0.0 - 0.0	0.0	0.0 - 0.0	0.1	0.0 - 1.0	1.00
3050208	81054	PORT ROYAL SOU	1.4	0.0	0 - 0	0.0	0.0 - 0.0	0.0	0.0 - 0.0	0.0	0.0 - 0.0	0.0	0.0 - 0.0	0.0	0.0 - 0.0	0.0	0.0 - 0.3	1.00
3050208	81056	PORT ROYAL SOU	81.1	0.4	0 - 2	0.0	0.0 - 0.0	0.0	0.0 - 0.0	0.0	0.0 - 0.0	0.0	0.0 - 0.1	0.0	0.0 - 0.0	0.3	0.1 - 1.9	1.00
3050208	81057	PORT ROYAL SOU	296.4	10.5	4 - 19	7.9	3.0 - 16.9	0.4	0.1 - 1.2	0.0	0.0 - 0.1	0.3	0.1 - 1.1	0.0	0.0 - 0.0	1.9	0.4 - 5.3	1.00
3050208	81059	PORT ROYAL SOU	563.1	5.3	2 - 11	5.1	1.6 - 11.6	0.0	0.0 - 0.1	0.0	0.0 - 0.0	0.0	0.0 - 0.0	0.0	0.0 - 0.0	0.2	0.0 - 1.2	1.00
3050208	81060	PORT ROYAL SOU	25.1	0.0	0 - 0	0.0	0.0 - 0.0	0.0	0.0 - 0.0	0.0	0.0 - 0.0	0.0	0.0 - 0.0	0.0	0.0 - 0.0	0.0	0.0 - 0.4	1.00
3060204	81062	TYBEE ROADS	12.9	108.2	39 - 286	103.0	36.3 - 273.3	0.2	0.1 - 1.0	0.0	0.0 - 0.0	0.0	0.0 - 0.1	0.0	0.0 - 0.0	4.9	1.1 - 20.3	1.00
3060204	81063	TYBEE ROADS	1.7	2.7	0 - 9	0.0	0.0 - 0.0	0.1	0.0 - 0.5	0.0	0.0 - 0.0	0.0	0.0 - 0.2	0.0	0.0 - 0.0	2.6	0.5 - 8.7	1.00
3060204	81064	LITTLE OGEECHE	15.6	2.6	0 - 10	0.0	0.0 - 0.0	0.0	0.0 - 0.0	0.0	0.0 - 0.0	0.0	0.0 - 0.0	0.0	0.0 - 0.0	2.6	0.4 - 10.0	1.00
3060204	81065	OSSABAW SOUND	82.7	5.8	1 - 20	0.0	0.0 - 0.0	0.0	0.0 - 0.0	0.0	0.0 - 0.0	0.0	0.0 - 0.0	0.0	0.0 - 0.0	5.8	1.0 - 20.4	1.00
3060204	81066	MEDWAY R	32.2	8.4	2 - 22	0.0	0.0 - 0.0	0.1	0.0 - 0.3	0.0	0.0 - 0.0	0.0	0.0 - 0.0	0.0	0.0 - 0.0	8.3	2.0 - 22.0	1.00
3060204	81067	DOBOY SOUND	91.2	2.7	0 - 9	0.0	0.0 - 0.0	0.0	0.0 - 0.0	0.0	0.0 - 0.0	0.0	0.0 - 0.0	0.0	0.0 - 0.0	2.7	0.4 - 8.9	1.00
3060204	81068	ALTAMAHA SOUN	81.1	6.7	2 - 21	0.0	0.0 - 0.0	0.2	0.0 - 0.5	0.0	0.0 - 0.0	0.0	0.0 - 0.1	0.0	0.0 - 0.0	6.5	1.5 - 20.6	1.00

8-digit HUC	ID	Name	Area	Catchment Yield		Point sources		Developed Land		Manure		Agricultural Land		Phosphate Mines		Soil parent rock		Frac
				kg/km^2	90% CI	kg/km^2	90% CI	kg/km^2	90% CI	kg/km^2	90% CI	kg/km^2	90% CI	kg/km^2	90% CI	kg/km^2	90% CI	
3070106	81069	ALTAMAHA R	12.5	7.9	1 - 25	0.0	0.0 - 0.0	0.0	0.0 - 0.0	0.0	0.0 - 0.0	0.0	0.0 - 0.0	0.0	0.0 - 0.0	7.9	1.5 - 24.7	1.00
3070203	81073	ST SIMONS SOUNI	157.5	10.3	2 - 31	0.0	0.0 - 0.0	0.8	0.2 - 2.2	0.0	0.0 - 0.0	0.1	0.0 - 0.5	0.0	0.0 - 0.0	9.4	2.1 - 30.1	1.00
3070204	81074	ATLANTIC OCEAN	85.8	174.4	63 - 411	170.7	64.1 - 407.2	1.6	0.4 - 4.7	0.1	0.0 - 0.3	0.0	0.0 - 0.1	0.0	0.0 - 0.0	2.0	0.6 - 4.8	1.00
3070205	81075	NASSAU SOUND	58.6	3.7	1 - 8	1.6	0.6 - 4.1	0.6	0.1 - 2.4	0.0	0.0 - 0.2	0.0	0.0 - 0.1	0.0	0.0 - 0.0	1.3	0.3 - 5.1	1.00
3070205	81076	NASSAU SOUND	127.4	0.7	0 - 2	0.0	0.0 - 0.0	0.1	0.0 - 0.3	0.0	0.0 - 0.1	0.0	0.0 - 0.0	0.0	0.0 - 0.0	0.6	0.1 - 1.8	1.00
3080103	81088	ST JOHNS R	127.9	32.9	10 - 78	0.0	0.0 - 0.0	4.5	1.3 - 10.8	1.2	0.3 - 3.5	2.9	0.8 - 7.6	0.0	0.0 - 0.0	24.3	7.1 - 62.5	1.00
3080103	81091	ST JOHNS R	53.8	51.3	17 - 118	0.0	0.0 - 0.0	11.0	3.7 - 27.2	1.9	0.6 - 5.2	5.4	1.8 - 13.4	0.0	0.0 - 0.0	33.0	10.1 - 87.9	1.00
3080201	81093	ATLANTIC OCEAN	62.6	44.4	14 - 106	0.0	0.0 - 0.0	17.3	5.8 - 37.2	0.2	0.0 - 0.4	0.4	0.1 - 1.0	0.0	0.0 - 0.0	26.5	7.8 - 63.2	1.00
3080201	81094	ATLANTIC OCEAN	1051.0	36.5	11 - 106	0.0	0.0 - 0.1	3.2	0.9 - 9.3	0.0	0.0 - 0.1	0.1	0.0 - 0.3	0.0	0.0 - 0.0	33.2	9.5 - 97.2	1.00
3080201	81095	ATLANTIC OCEAN	2.8	59.6	21 - 117	0.0	0.0 - 0.0	44.0	15.4 - 86.1	0.1	0.0 - 0.2	0.3	0.1 - 0.7	0.0	0.0 - 0.0	15.2	5.5 - 40.2	1.00
3080202	81096	MOSQUITO LAGOO	485.5	28.9	10 - 77	0.7	0.3 - 2.1	9.4	2.9 - 25.3	0.1	0.0 - 0.3	0.4	0.1 - 1.1	0.0	0.0 - 0.0	18.3	6.6 - 51.3	1.00
3080202	81097	INDIAN R	449.3	25.0	9 - 62	0.8	0.3 - 2.0	7.7	2.7 - 18.2	0.4	0.1 - 1.1	1.6	0.6 - 4.0	0.0	0.0 - 0.0	14.5	5.2 - 39.6	1.00
3080202	81098	INDIAN R	460.3	65.9	20 - 147	1.6	0.5 - 4.5	43.6	12.3 - 97.3	0.1	0.0 - 0.3	0.2	0.1 - 0.5	0.0	0.0 - 0.0	20.4	7.3 - 51.6	1.00
3100101	81126	PEACE R	44.9	60.8	22 - 159	0.0	0.0 - 0.0	38.5	13.7 - 93.8	1.1	0.3 - 3.9	3.5	1.2 - 8.8	0.0	0.0 - 0.0	17.7	5.8 - 50.0	1.00
3100101	81128	PEACE R	96.2	37.2	12 - 87	0.0	0.0 - 0.0	23.8	7.2 - 61.6	0.0	0.0 - 0.1	0.0	0.0 - 0.0	0.0	0.0 - 0.0	13.4	4.6 - 35.7	1.00
3100102	81129	PEACE R	65.8	727.8	293 - 1558	648.8	255.9 - 1372.2	50.5	20.0 - 114.3	1.2	0.4 - 4.0	3.5	1.3 - 9.6	0.0	0.0 - 0.0	23.8	9.9 - 65.6	1.00
3100103	81131	CHARLOTTE HARI	57.2	65.1	25 - 164	0.0	0.0 - 0.0	7.0	2.2 - 15.5	0.0	0.0 - 0.0	0.0	0.0 - 0.0	0.0	0.0 - 0.0	58.0	22.7 - 153.4	1.00
3100103	81132	CHARLOTTE HARI	36.4	64.6	19 - 175	0.0	0.0 - 0.0	7.8	2.3 - 18.7	0.0	0.0 - 0.1	0.0	0.0 - 0.0	0.0	0.0 - 0.0	56.8	15.5 - 159.1	1.00
3100103	81133	CHARLOTTE HARI	55.2	45.3	14 - 123	0.0	0.0 - 0.0	0.0	0.0 - 0.0	0.3	0.1 - 0.9	0.9	0.3 - 2.4	0.0	0.0 - 0.0	44.2	13.9 - 122.5	1.00
3100101	81136	CHARLOTTE HARI	28.8	73.5	19 - 183	0.0	0.0 - 0.0	22.8	7.5 - 56.3	0.0	0.0 - 0.0	0.0	0.0 - 0.0	0.0	0.0 - 0.0	50.7	11.7 - 151.2	1.00
3100201	81137	SARASOTA BAY	708.1	71.2	27 - 175	0.0	0.0 - 0.0	19.5	6.5 - 51.6	0.4	0.1 - 1.3	1.3	0.5 - 4.0	4.5	1.6 - 14.9	45.5	16.7 - 120.5	1.00
3100202	81138	MANATEE R	53.4	124.3	42 - 270	0.0	0.0 - 0.0	73.7	24.7 - 163.8	1.3	0.3 - 4.3	3.3	0.9 - 8.9	0.0	0.0 - 0.0	46.0	15.1 - 117.6	1.00
3100202	81139	MANATEE R	83.4	106.9	32 - 216	0.0	0.0 - 0.0	35.7	11.1 - 73.8	5.3	1.4 - 15.9	15.4	5.0 - 41.4	0.0	0.0 - 0.0	50.5	15.1 - 123.4	1.00
3100206	81140	TAMPA BAY	360.0	89.9	25 - 208	0.0	0.0 - 0.0	10.7	2.6 - 21.7	5.7	1.4 - 16.3	16.5	4.6 - 43.1	4.2	1.1 - 9.3	52.8	14.8 - 143.3	1.00
3100204	81141	HILLSBOROUGH B	3.4	2686.4	857 - 8812	0.0	0.0 - 0.0	3.4	1.1 - 9.0	0.0	0.0 - 0.0	0.0	0.0 - 0.0	2633.8	836.5 - 8638	49.2	16.6 - 149.4	1.00
3100206	81142	HILLSBOROUGH B	43.9	1238.0	437 - 2229	829.1	274.4 - 1586.9	15.0	5.1 - 30.1	1.3	0.4 - 4.0	0.7	0.3 - 1.7	346.4	122.7 - 867.	45.5	14.3 - 107.9	1.00
3100206	81143	HILLSBOROUGH B	52.3	4071.5	1146 - 9547	3715.6	1008.8 - 9174.	62.0	17.6 - 144.7	0.0	0.0 - 0.1	0.0	0.0 - 0.0	263.2	80.4 - 825.1	30.6	8.7 - 83.9	1.00
3100206	81144	OLD TAMPA BAY	16.7	40.8	12 - 112	0.0	0.0 - 0.0	11.1	3.2 - 26.4	0.0	0.0 - 0.0	0.0	0.0 - 0.0	0.0	0.0 - 0.0	29.7	8.9 - 91.1	1.00
3100206	81145	OLD TAMPA BAY	333.8	89.9	27 - 194	9.6	2.8 - 21.4	45.5	13.0 - 107.7	0.0	0.0 - 0.0	0.0	0.0 - 0.0	0.0	0.0 - 0.0	34.8	10.8 - 86.4	1.00
3100206	81146	TAMPA BAY	302.4	45.0	16 - 121	0.0	0.0 - 0.0	24.7	8.2 - 65.0	0.0	0.0 - 0.0	0.0	0.0 - 0.0	0.0	0.0 - 0.0	20.3	7.1 - 57.8	1.00
3100207	81147	GULF OF MEXICO	61.5	17.1	5 - 46	0.0	0.0 - 0.0	1.3	0.4 - 3.7	0.0	0.0 - 0.0	0.0	0.0 - 0.0	0.0	0.0 - 0.0	15.8	4.9 - 42.7	1.00
3100207	81149	GULF OF MEXICO	3.2	1.7	0 - 7	0.0	0.0 - 0.0	1.0	0.2 - 4.7	0.0	0.0 - 0.0	0.0	0.0 - 0.0	0.0	0.0 - 0.0	0.7	0.1 - 2.7	1.00
3100207	81151	GULF OF MEXICO	51.0	0.6	0 - 3	0.0	0.0 - 0.0	0.0	0.0 - 0.3	0.0	0.0 - 0.0	0.0	0.0 - 0.0	0.0	0.0 - 0.0	0.5	0.1 - 2.4	1.00
3100207	81152	GULF OF MEXICO	1639.2	12.3	4 - 28	0.0	0.0 - 0.0	7.1	2.1 - 18.5	0.5	0.1 - 1.4	1.0	0.3 - 3.1	0.0	0.0 - 0.0	3.7	1.3 - 9.3	1.00
3110101	81153	WACCASASSA BA	26.5	3.6	1 - 10	0.0	0.0 - 0.0	0.0	0.0 - 0.0	0.0	0.0 - 0.0	0.0	0.0 - 0.0	0.0	0.0 - 0.0	3.6	0.8 - 10.0	1.00
3110101	81155	WACCASASSA BA	586.6	5.1	1 - 13	0.0	0.0 - 0.0	0.1	0.0 - 0.2	0.0	0.0 - 0.0	0.0	0.0 - 0.0	0.0	0.0 - 0.0	5.0	1.3 - 12.4	1.00
3110102	81156	GULF OF MEXICO	375.1	26.0	9 - 86	0.0	0.0 - 0.0	0.4	0.1 - 1.4	0.1	0.0 - 0.3	0.1	0.0 - 0.2	0.0	0.0 - 0.0	25.4	9.1 - 89.4	1.00
3110102	81157	GULF OF MEXICO	41.6	45.7	13 - 108	0.0	0.0 - 0.0	0.8	0.2 - 1.7	0.1	0.0 - 0.3	0.2	0.1 - 0.5	0.0	0.0 - 0.0	44.6	13.4 - 108.9	1.00
3110102	81158	GULF OF MEXICO	549.0	14.8	5 - 37	0.0	0.0 - 0.0	2.8	0.9 - 6.4	0.3	0.1 - 0.9	0.9	0.3 - 2.3	0.0	0.0 - 0.0	10.8	3.6 - 26.4	1.00

8-digit HUC	ID	Name	Area	Catchment Yield		Point sources		Developed Land		Manure		Agricultural Land		Phosphate Mines		Soil parent rock		Frac
				kg/km^2	90% CI	kg/km^2	90% CI	kg/km^2	90% CI	kg/km^2	90% CI	kg/km^2	90% CI	kg/km^2	90% CI	kg/km^2	90% CI	
3110102	81159	GULF OF MEXICO	78.0	32.3	10 - 82	0.0	0.0 - 0.0	0.1	0.0 - 0.1	0.0	0.0 - 0.0	0.0	0.0 - 0.0	0.0	0.0 - 0.0	32.2	9.7 - 82.3	1.00
3110102	81160	GULF OF MEXICO	150.5	39.8	11 - 122	0.0	0.0 - 0.0	0.1	0.0 - 0.1	0.0	0.0 - 0.0	0.0	0.0 - 0.0	0.0	0.0 - 0.0	39.7	10.8 - 121.8	1.00
3110102	81161	GULF OF MEXICO	43.9	47.6	12 - 125	0.0	0.0 - 0.0	0.0	0.0 - 0.0	0.0	0.0 - 0.0	0.0	0.0 - 0.0	0.0	0.0 - 0.0	47.5	11.8 - 124.9	1.00
3110205	81162	SUWANNEE SOUN	70.2	0.1	0 - 1	0.0	0.0 - 0.0	0.0	0.0 - 0.0	0.0	0.0 - 0.0	0.0	0.0 - 0.0	0.0	0.0 - 0.0	0.1	0.0 - 1.0	1.00
3120001	81163	APPALACHEE BAY	240.5	40.6	11 - 117	0.0	0.0 - 0.0	0.4	0.1 - 0.8	0.0	0.0 - 0.1	0.0	0.0 - 0.0	0.0	0.0 - 0.0	40.2	11.3 - 116.9	1.00
3120003	81164	OCHLOCKNEE BA	1.7	60.5	17 - 214	0.0	0.0 - 0.0	0.0	0.0 - 0.0	0.0	0.0 - 0.0	0.0	0.0 - 0.0	0.0	0.0 - 0.0	60.5	17.2 - 214.1	1.00
3130011	81166	APALACHICOLA B	70.5	1.6	0 - 6	0.0	0.0 - 0.0	0.0	0.0 - 0.1	0.0	0.0 - 0.0	0.0	0.0 - 0.0	0.0	0.0 - 0.0	1.6	0.3 - 5.6	1.00
3130014	81167	APALACHICOLA B	270.1	20.6	7 - 37	1.1	0.4 - 2.0	4.7	1.4 - 9.1	0.0	0.0 - 0.0	0.0	0.0 - 0.1	0.0	0.0 - 0.0	14.7	5.0 - 30.7	1.00
3140101	81168	ST ANDREW BAY	39.9	30.9	11 - 66	0.0	0.0 - 0.0	2.3	0.7 - 4.3	0.1	0.0 - 0.3	0.4	0.1 - 0.9	0.0	0.0 - 0.0	28.2	9.7 - 65.0	1.00
3140101	81169	ST ANDREW BAY	154.3	35.4	13 - 79	0.0	0.0 - 0.0	7.3	2.6 - 15.9	0.1	0.0 - 0.4	0.3	0.1 - 0.7	0.0	0.0 - 0.0	27.6	9.9 - 77.2	1.00
3140101	81170	ST ANDREW BAY	219.6	115.2	41 - 301	25.4	8.3 - 63.4	69.8	22.5 - 185.6	0.0	0.0 - 0.1	0.5	0.2 - 1.3	0.0	0.0 - 0.0	19.5	7.2 - 57.6	1.00
3140101	81171	ST ANDREW BAY	8.9	40.0	11 - 80	0.0	0.0 - 0.0	22.5	6.4 - 47.9	0.1	0.0 - 0.2	2.5	0.8 - 6.3	0.0	0.0 - 0.0	14.9	4.4 - 34.0	1.00
3140101	81173	ST ANDREW BAY	152.1	23.5	8 - 47	0.0	0.0 - 0.0	6.8	2.2 - 14.3	0.1	0.0 - 0.2	2.6	0.7 - 6.4	0.0	0.0 - 0.0	14.0	4.3 - 31.7	1.00
3140101	81174	ST ANDREW BAY	22.6	13.4	4 - 30	0.0	0.0 - 0.0	1.0	0.3 - 2.2	0.0	0.0 - 0.0	0.2	0.0 - 0.6	0.0	0.0 - 0.0	12.3	3.2 - 28.2	1.00
3140101	81175	ST ANDREW BAY	12.4	12.9	4 - 30	0.0	0.0 - 0.0	0.5	0.1 - 1.2	0.0	0.0 - 0.0	0.0	0.0 - 0.1	0.0	0.0 - 0.0	12.3	3.5 - 29.4	1.00
3140101	81176	ST JOSEPH BAY	15.4	32.7	10 - 70	0.0	0.0 - 0.0	8.4	2.6 - 18.0	0.0	0.0 - 0.1	0.0	0.0 - 0.0	0.0	0.0 - 0.0	24.3	8.1 - 57.2	1.00
3140101	81178	ST JOSEPH BAY	109.6	31.2	10 - 55	0.0	0.0 - 0.0	8.5	3.0 - 16.3	0.0	0.0 - 0.0	0.0	0.0 - 0.0	0.0	0.0 - 0.0	22.7	7.6 - 43.3	1.00
3140101	81179	ST ANDREW BAY	154.3	138.2	51 - 330	107.5	38.2 - 303.8	12.3	4.0 - 26.0	0.0	0.0 - 0.1	0.4	0.1 - 1.0	0.0	0.0 - 0.0	18.0	7.0 - 40.5	1.00
3140102	81181	CHOCTAWHATCH	58.1	24.9	9 - 57	0.0	0.0 - 0.0	7.2	2.6 - 15.8	0.9	0.3 - 2.9	3.0	1.1 - 7.7	0.0	0.0 - 0.0	13.7	4.6 - 36.1	1.00
3140102	81182	CHOCTAWHATCH	34.5	24.5	8 - 56	0.0	0.0 - 0.0	4.3	1.5 - 8.7	0.8	0.2 - 2.0	1.3	0.4 - 3.8	0.0	0.0 - 0.0	18.1	5.7 - 41.8	1.00
3140102	81183	CHOCTAWHATCH	24.1	25.6	8 - 59	0.0	0.0 - 0.0	4.5	1.2 - 9.4	0.4	0.1 - 1.0	0.3	0.1 - 0.8	0.0	0.0 - 0.0	20.5	6.3 - 51.9	1.00
3140102	81184	CHOCTAWHATCH	157.1	35.5	12 - 85	0.0	0.0 - 0.0	22.2	6.4 - 53.1	0.3	0.1 - 1.1	0.4	0.1 - 1.3	0.0	0.0 - 0.0	12.6	4.4 - 36.5	1.00
3140102	81185	CHOCTAWHATCH	11.6	29.2	9 - 71	0.0	0.0 - 0.0	21.0	5.8 - 51.8	0.3	0.1 - 0.9	2.3	0.7 - 6.2	0.0	0.0 - 0.0	5.7	1.8 - 15.5	1.00
3140102	81186	CHOCTAWHATCH	18.8	101.4	37 - 229	0.0	0.0 - 0.0	92.1	32.7 - 208.9	0.1	0.0 - 0.2	0.0	0.0 - 0.0	0.0	0.0 - 0.0	9.2	3.4 - 24.6	1.00
3140102	81187	CHOCTAWHATCH	217.4	76.0	30 - 155	0.0	0.0 - 0.0	63.7	24.3 - 139.9	0.3	0.1 - 0.7	1.6	0.6 - 4.6	0.0	0.0 - 0.0	10.4	4.4 - 26.3	1.00
3140105	81188	SANTA ROSA SOU	419.3	41.5	16 - 97	2.2	0.8 - 4.9	23.1	7.8 - 49.2	0.0	0.0 - 0.1	0.2	0.1 - 0.5	0.0	0.0 - 0.0	16.0	5.8 - 37.8	1.00
3140105	81189	PENSACOLA BAY	136.5	42.7	15 - 82	0.0	0.0 - 0.0	29.1	9.2 - 61.8	0.0	0.0 - 0.1	0.3	0.1 - 0.7	0.0	0.0 - 0.0	13.3	4.9 - 29.3	1.00
3140105	81190	PENSACOLA BAY	144.5	14.3	4 - 35	0.0	0.0 - 0.0	6.3	1.5 - 14.3	0.1	0.0 - 0.4	0.3	0.1 - 0.9	0.0	0.0 - 0.0	7.6	2.4 - 19.9	1.00
3140103	81191	PENSACOLA BAY	49.8	39.8	13 - 81	0.0	0.0 - 0.0	22.0	6.7 - 46.0	0.7	0.2 - 1.7	8.6	3.0 - 22.3	0.0	0.0 - 0.0	8.5	2.6 - 18.2	1.00
3140105	81192	PENSACOLA BAY	282.2	24.7	9 - 59	7.4	2.6 - 17.6	8.9	2.9 - 22.1	0.2	0.0 - 0.5	1.2	0.4 - 3.1	0.0	0.0 - 0.0	7.1	2.5 - 18.3	1.00
3140105	81193	PENSACOLA BAY	75.4	59.9	18 - 134	0.0	0.0 - 0.0	51.2	15.5 - 114.3	0.0	0.0 - 0.1	0.2	0.0 - 0.4	0.0	0.0 - 0.0	8.5	2.6 - 18.9	1.00
3140105	81194	PENSACOLA BAY	149.5	134.5	48 - 281	53.4	19.2 - 121.8	70.4	23.9 - 170.0	0.1	0.0 - 0.3	0.4	0.1 - 1.0	0.0	0.0 - 0.1	10.2	3.7 - 28.1	1.00
3140107	81195	GULF OF MEXICO	274.0	23.1	8 - 53	0.0	0.0 - 0.0	6.6	2.0 - 15.6	0.0	0.0 - 0.0	0.0	0.0 - 0.0	0.0	0.0 - 0.0	16.5	5.5 - 38.0	1.00
3140107	81196	PERDIDO BAY	111.6	54.9	19 - 104	0.0	0.0 - 0.0	36.4	12.6 - 72.0	0.1	0.0 - 0.3	0.5	0.2 - 1.2	0.0	0.0 - 0.0	17.8	5.3 - 43.5	1.00
3140107	81197	PERDIDO BAY	12.8	42.0	15 - 78	0.0	0.0 - 0.0	23.3	7.5 - 52.1	0.1	0.0 - 0.4	1.1	0.4 - 2.8	0.0	0.0 - 0.0	17.5	6.6 - 41.6	1.00
3140107	81198	PERDIDO BAY	4.0	10.1	3 - 27	0.0	0.0 - 0.0	0.0	0.0 - 0.0	0.0	0.0 - 0.0	0.0	0.0 - 0.0	0.0	0.0 - 0.0	10.1	2.9 - 26.6	1.00
3140107	81200	PERDIDO BAY	219.7	71.8	23 - 162	0.0	0.0 - 0.0	15.1	4.4 - 35.1	4.2	1.2 - 13.7	32.4	9.5 - 87.3	0.0	0.0 - 0.0	20.0	6.1 - 48.0	1.00
3140107	81201	PERDIDO BAY	28.9	51.6	15 - 93	0.0	0.0 - 0.0	9.5	2.9 - 20.6	3.1	0.9 - 9.3	18.4	5.6 - 41.5	0.0	0.0 - 0.0	20.5	6.3 - 46.9	1.00
3140107	81202	GULF OF MEXICO	570.6	22.8	8 - 44	0.0	0.0 - 0.0	3.3	1.0 - 6.7	0.0	0.0 - 0.0	0.0	0.0 - 0.0	0.0	0.0 - 0.0	19.5	6.4 - 39.1	1.00

8-digit HUC	ID	Name	Area	Catchment Yield kg/km^2	Catchment Yield 90% CI	Point sources kg/km^2	Point sources 90% CI	Developed Land kg/km^2	Developed Land 90% CI	Manure kg/km^2	Manure 90% CI	Agricultural Land kg/km^2	Agricultural Land 90% CI	Phosphate Mines kg/km^2	Phosphate Mines 90% CI	Soil parent rock kg/km^2	Soil parent rock 90% CI	Frac
3160204	81203	MOBILE BAY	39.2	101.5	38 - 214	0.0	0.0 - 0.0	75.9	27.8 - 173.0	1.6	0.4 - 4.5	10.7	3.2 - 29.7	0.0	0.0 - 0.0	13.3	4.6 - 30.4	1.00
3160204	81205	MOBILE BAY	121.1	29.6	10 - 53	26.0	9.3 - 50.5	0.1	0.0 - 0.5	0.0	0.0 - 0.0	0.0	0.0 - 0.2	0.0	0.0 - 0.0	3.4	0.7 - 12.0	1.00
3160205	81206	MOBILE BAY	12.5	2443.6	836 - 5745	2440.5	836.6 - 5737.3	0.4	0.1 - 1.7	0.0	0.0 - 0.0	0.0	0.0 - 0.0	0.0	0.0 - 0.0	2.7	0.5 - 11.0	1.00
3160205	81207	MOBILE BAY	16.9	54.8	19 - 117	0.0	0.0 - 0.0	8.1	2.5 - 16.4	4.0	1.1 - 9.8	32.0	9.7 - 79.0	0.0	0.0 - 0.0	10.8	3.6 - 23.4	1.00
3160205	81208	MOBILE BAY	313.1	37.2	13 - 79	6.8	2.4 - 15.8	12.8	4.1 - 25.3	0.7	0.2 - 2.3	5.1	1.7 - 14.8	0.0	0.0 - 0.0	11.8	4.3 - 25.1	1.00
3160205	81209	MOBILE BAY	65.7	46.7	17 - 115	37.4	13.7 - 90.6	2.8	0.8 - 6.8	0.1	0.0 - 0.2	0.1	0.0 - 0.2	0.0	0.0 - 0.0	6.4	1.9 - 16.8	1.00
3160205	81210	MOBILE BAY	3.5	36.8	11 - 77	0.0	0.0 - 0.0	23.5	6.8 - 47.8	0.1	0.0 - 0.3	0.4	0.1 - 1.0	0.0	0.0 - 0.0	12.8	4.0 - 31.3	1.00
3160205	81211	MOBILE BAY	186.3	35.3	10 - 84	0.0	0.0 - 0.0	18.9	5.4 - 41.4	0.4	0.1 - 1.2	1.6	0.4 - 4.2	0.0	0.0 - 0.0	14.4	3.7 - 36.9	1.00
3160205	81212	MOBILE BAY	387.9	13.5	5 - 32	2.7	1.0 - 8.0	0.5	0.1 - 1.7	0.0	0.0 - 0.0	0.0	0.0 - 0.1	0.0	0.0 - 0.0	10.3	3.3 - 22.4	1.00
3170006	81213	PASCAGOULA BA	144.8	113.7	43 - 263	69.4	26.5 - 173.6	13.4	4.7 - 31.4	0.0	0.0 - 0.1	0.0	0.0 - 0.0	0.0	0.0 - 0.0	30.8	10.4 - 83.3	1.00
3170006	81214	PASCAGOULA R	9.6	304.6	56 - 900	0.0	0.0 - 0.0	6.1	1.9 - 12.7	0.1	0.0 - 0.4	0.0	0.0 - 0.0	0.0	0.0 - 0.0	298.3	54.8 - 910.9	1.00
3170006	81215	PASCAGOULA R	21.6	90.6	30 - 168	0.0	0.0 - 0.0	33.3	10.4 - 67.3	0.6	0.2 - 2.0	0.6	0.2 - 1.2	0.0	0.0 - 0.0	56.0	17.5 - 113.3	1.00
3170009	81216	PASCAGOULA BA	44.0	22.9	8 - 56	0.0	0.0 - 0.0	7.6	2.6 - 20.4	0.0	0.0 - 0.1	0.1	0.0 - 0.2	0.0	0.0 - 0.0	15.2	5.3 - 39.9	1.00
3170006	81217	PASCAGOULA R	2.9	309.4	76 - 1208	0.0	0.0 - 0.0	0.0	0.0 - 0.0	0.0	0.0 - 0.0	0.0	0.0 - 0.0	0.0	0.0 - 0.0	309.4	75.8 - 1208.[illegible]	1.00
3170006	81219	PASCAGOULA R	15.5	270.2	89 - 550	199.7	63.9 - 475.3	16.4	5.6 - 34.7	2.2	0.6 - 7.5	3.6	1.1 - 11.0	0.0	0.0 - 0.0	48.3	15.0 - 120.7	1.00
3170009	81220	MISSISSIPPI SOUN	773.2	28.9	8 - 60	1.4	0.4 - 2.9	1.8	0.5 - 4.0	0.9	0.2 - 2.1	4.7	1.2 - 12.1	0.0	0.0 - 0.0	20.0	5.7 - 46.6	1.00
3170009	81221	MISSISSIPPI SOUN	4.8	0.0	0 - 0	0.0	0.0 - 0.0	0.0	0.0 - 0.0	0.0	0.0 - 0.0	0.0	0.0 - 0.0	0.0	0.0 - 0.0	0.0	0.0 - 0.0	1.00
3170009	81222	MISSISSIPPI SOUN	36.5	1298.3	434 - 3329	1157.5	390.3 - 3106.1	52.4	17.5 - 123.1	0.9	0.3 - 2.6	1.0	0.3 - 2.7	58.8	17.1 - 158.6	27.7	8.6 - 67.6	1.00
3170009	81223	BILOXI BAY	44.4	130.5	50 - 287	28.9	10.8 - 59.7	76.9	29.2 - 185.0	0.5	0.2 - 1.2	1.4	0.5 - 3.8	0.0	0.0 - 0.0	22.8	9.2 - 51.2	1.00
3170009	81224	BILOXI BAY	11.4	143.1	44 - 339	3.0	1.0 - 7.9	53.2	15.8 - 126.3	2.2	0.6 - 8.6	13.6	4.3 - 38.6	0.0	0.0 - 0.0	71.1	22.6 - 218.6	1.00
3170009	81225	MISSISSIPPI SOUN	822.2	20.8	6 - 47	0.0	0.0 - 0.0	7.0	2.3 - 16.3	0.0	0.0 - 0.0	0.0	0.0 - 0.1	0.0	0.0 - 0.0	13.7	4.5 - 32.2	1.00
3170009	81226	ST LOUIS BAY	46.5	101.4	26 - 227	0.0	0.0 - 0.0	16.6	5.1 - 25.9	0.8	0.2 - 2.5	3.7	1.1 - 9.2	0.0	0.0 - 0.0	80.3	20.4 - 198.4	1.00
3170009	81227	BILOXI BAY	6.4	182.8	45 - 424	3.6	1.2 - 7.2	11.4	4.2 - 22.4	1.6	0.4 - 4.9	12.4	3.5 - 34.6	0.0	0.0 - 0.0	153.8	38.6 - 377.6	1.00
3170009	81228	BILOXI BAY	46.0	611.6	257 - 1091	553.6	230.9 - 1022.4	45.4	15.6 - 86.7	0.0	0.0 - 0.0	0.0	0.0 - 0.1	0.0	0.0 - 0.0	12.5	5.1 - 26.1	1.00
3170009	81229	BILOXI BAY	51.9	52.2	18 - 112	0.0	0.0 - 0.0	35.1	11.0 - 75.8	0.4	0.1 - 1.1	0.5	0.2 - 1.3	0.0	0.0 - 0.0	16.2	6.0 - 40.9	1.00
3170009	81230	ST LOUIS BAY	54.4	201.5	67 - 429	125.6	44.6 - 311.4	40.7	13.8 - 80.0	0.8	0.2 - 2.9	3.5	1.1 - 9.1	0.0	0.0 - 0.0	30.8	10.0 - 81.8	1.00
3170009	81231	ST LOUIS BAY	29.8	133.1	50 - 354	0.0	0.0 - 0.0	53.2	19.2 - 131.6	0.5	0.2 - 1.6	0.8	0.3 - 2.2	0.0	0.0 - 0.0	78.6	26.5 - 242.4	1.00
3170009	81232	MISSISSIPPI SOUN	163.5	36.6	11 - 88	0.0	0.0 - 0.0	9.5	2.8 - 21.6	0.2	0.1 - 0.6	0.1	0.0 - 0.4	0.0	0.0 - 0.0	26.8	7.9 - 68.4	1.00
3180004	81233	L BORGNE	34.8	1.5	0 - 4	0.0	0.0 - 0.0	1.5	0.5 - 3.9	0.0	0.0 - 0.0	0.0	0.0 - 0.0	0.0	0.0 - 0.0	0.0	0.0 - 0.0	1.00
3020105	81472	PAMLICO SOUND	67.9	0.0	0 - 0	0.0	0.0 - 0.0	0.0	0.0 - 0.0	0.0	0.0 - 0.0	0.0	0.0 - 0.0	0.0	0.0 - 0.0	0.0	0.0 - 0.0	1.00
3020104	81473	PAMLICO R	304.8	53.9	17 - 130	0.1	0.0 - 0.3	9.1	2.6 - 19.4	3.8	1.0 - 11.4	22.5	7.7 - 66.6	0.0	0.0 - 0.0	18.4	6.1 - 51.7	1.00
3020104	81474	PAMLICO R	60.8	586.3	204 - 1274	230.1	82.3 - 586.0	1.4	0.5 - 3.3	0.0	0.0 - 0.1	0.1	0.0 - 0.3	338.7	110.0 - 972.[illegible]	15.9	5.4 - 37.8	1.00
3030005	81475	LONG BAY	267.0	0.2	0 - 1	0.0	0.0 - 0.0	0.0	0.0 - 0.1	0.0	0.0 - 0.0	0.0	0.0 - 0.0	0.0	0.0 - 0.0	0.2	0.0 - 0.7	1.00
3040207	81476	WINYAH BAY	73.2	1.7	0 - 6	0.0	0.0 - 0.0	0.0	0.0 - 0.0	0.0	0.0 - 0.0	0.0	0.0 - 0.0	0.0	0.0 - 0.0	1.7	0.3 - 6.3	1.00
3070203	81477	TURTLE R	52.4	19.8	6 - 53	0.0	0.0 - 0.0	1.8	0.6 - 4.6	0.1	0.0 - 0.2	0.2	0.0 - 0.4	0.0	0.0 - 0.0	17.8	5.7 - 48.8	1.00
3070203	81478	TURTLE R	45.8	877.0	283 - 1749	862.0	280.3 - 1728.7	5.4	1.4 - 13.6	0.1	0.0 - 0.2	0.2	0.0 - 0.5	0.0	0.0 - 0.0	9.4	2.7 - 22.7	1.00
3080103	81479	ST JOHNS R	19.6	78.3	24 - 164	0.0	0.0 - 0.0	64.5	19.9 - 138.3	0.1	0.0 - 0.2	0.1	0.0 - 0.2	0.0	0.0 - 0.0	13.6	4.4 - 37.1	1.00
3010205	81498	PASQUOTUNK R	211.4	115.7	44 - 315	0.0	0.0 - 0.0	6.9	2.4 - 16.2	0.9	0.3 - 2.8	75.0	27.0 - 199.3	0.0	0.0 - 0.0	33.0	11.6 - 100.3	1.00
3010205	81499	ALLIGATOR R	875.4	0.1	0 - 1	0.0	0.0 - 0.0	0.0	0.0 - 0.0	0.0	0.0 - 0.0	0.0	0.0 - 0.3	0.0	0.0 - 0.0	0.1	0.0 - 0.5	1.00

8-digit HUC	ID	Name	Area	Catchment Yield		Point sources		Developed Land		Manure		Agricultural Land		Phosphate Mines		Soil parent rock		Frac
				kg/km^2	90% CI	kg/km^2	90% CI	kg/km^2	90% CI	kg/km^2	90% CI	kg/km^2	90% CI	kg/km^2	90% CI	kg/km^2	90% CI	
3010205	81500	ALLIGATOR R	82.5	0.0	0 - 0	0.0	0.0 - 0.0	0.0	0.0 - 0.0	0.0	0.0 - 0.0	0.0	0.0 - 0.1	0.0	0.0 - 0.0	0.0	0.0 - 0.2	1.00
3010205	81501	ALLIGATOR R	299.5	0.0	0 - 0	0.0	0.0 - 0.0	0.0	0.0 - 0.0	0.0	0.0 - 0.0	0.0	0.0 - 0.1	0.0	0.0 - 0.0	0.0	0.0 - 0.2	1.00
3010203	81502	CHOWAN R	113.6	42.1	15 - 92	0.0	0.0 - 0.0	0.7	0.2 - 1.6	7.9	2.5 - 24.4	20.4	7.2 - 52.9	0.0	0.0 - 0.0	13.1	5.1 - 33.8	1.00
3020104	81503	PUNGO R	60.6	5.7	2 - 12	4.1	1.4 - 9.2	0.1	0.0 - 0.4	0.1	0.0 - 0.4	0.5	0.1 - 2.4	0.0	0.0 - 0.0	0.9	0.2 - 3.0	1.00
3030001	81504	NEW R	114.5	27.4	9 - 58	13.5	4.3 - 32.5	5.4	1.7 - 12.8	1.2	0.3 - 3.3	1.4	0.5 - 3.9	0.0	0.0 - 0.0	6.0	1.9 - 13.2	1.00
3060204	81505	SAPELO R	42.6	10.4	2 - 30	0.0	0.0 - 0.0	0.1	0.0 - 0.4	0.0	0.0 - 0.0	0.1	0.0 - 0.3	0.0	0.0 - 0.0	10.2	2.0 - 29.9	1.00
3070106	81506	ALTAMAHA R	9.3	5.3	1 - 21	0.0	0.0 - 0.0	0.0	0.0 - 0.0	0.0	0.0 - 0.0	0.0	0.0 - 0.0	0.0	0.0 - 0.0	5.3	0.8 - 21.0	1.00
3070203	81507	TURTLE R	31.2	9.1	2 - 24	0.0	0.0 - 0.0	0.8	0.2 - 2.4	0.1	0.0 - 0.2	0.4	0.1 - 1.3	0.0	0.0 - 0.0	7.9	2.0 - 22.0	1.00
3070203	81508	TURTLE R	25.8	16.0	4 - 39	0.0	0.0 - 0.0	2.4	0.7 - 5.7	0.0	0.0 - 0.1	0.2	0.0 - 0.5	0.0	0.0 - 0.0	13.4	3.6 - 36.8	1.00
3080103	81512	ST JOHNS R	39.1	58.0	18 - 103	0.0	0.0 - 0.0	40.3	13.0 - 78.6	0.9	0.3 - 2.3	1.3	0.4 - 2.9	0.0	0.0 - 0.0	15.6	4.7 - 33.0	1.00
3080103	81513	ST JOHNS R	34.5	102.0	35 - 214	35.4	12.5 - 73.4	49.7	16.3 - 110.8	0.3	0.1 - 0.7	0.2	0.1 - 0.5	0.0	0.0 - 0.0	16.4	5.1 - 43.9	1.00
3080103	81514	ST JOHNS R	4.6	77.2	25 - 137	0.0	0.0 - 0.0	61.2	19.2 - 111.4	0.0	0.0 - 0.1	0.0	0.0 - 0.0	0.0	0.0 - 0.0	16.0	5.1 - 41.8	1.00
3080103	81515	ST JOHNS R	17.0	216.6	73 - 525	142.1	47.2 - 358.8	62.1	20.1 - 143.0	0.2	0.1 - 0.5	0.3	0.1 - 0.8	0.0	0.0 - 0.0	11.9	4.0 - 30.8	1.00
3080103	81516	ST JOHNS R	6.9	988.0	377 - 2237	943.4	355.7 - 2148.6	29.6	11.4 - 65.8	0.4	0.1 - 1.2	0.0	0.0 - 0.0	0.0	0.0 - 0.0	14.6	5.3 - 34.4	1.00
3080103	81517	ST JOHNS R	14.4	51.3	18 - 84	0.0	0.0 - 0.0	36.4	12.2 - 63.4	0.3	0.1 - 0.6	0.1	0.0 - 0.3	0.0	0.0 - 0.0	14.5	5.5 - 30.9	1.00
3080103	81518	ST JOHNS R	31.6	63.4	21 - 117	0.0	0.0 - 0.0	48.8	15.5 - 90.4	0.1	0.0 - 0.3	0.1	0.0 - 0.2	0.0	0.0 - 0.0	14.4	4.3 - 33.3	1.00
3080103	81519	ST JOHNS R	10.9	43.3	15 - 95	0.0	0.0 - 0.0	38.2	12.8 - 86.6	0.1	0.0 - 0.2	0.7	0.2 - 1.5	0.0	0.0 - 0.0	4.4	1.3 - 10.0	1.00
3080103	81521	ST JOHNS R	29.2	38.6	12 - 80	2.5	0.9 - 6.1	18.0	5.3 - 38.7	0.2	0.1 - 0.5	0.5	0.2 - 1.3	0.0	0.0 - 0.0	17.4	5.2 - 45.0	1.00
3080103	81522	ST JOHNS R	118.8	5.6	1 - 13	0.0	0.0 - 0.0	0.2	0.0 - 0.6	0.1	0.0 - 0.3	1.5	0.3 - 4.4	0.0	0.0 - 0.0	3.8	1.1 - 10.1	1.00
3080103	81524	0	5.2	1.3	0 - 5	0.0	0.0 - 0.0	0.3	0.0 - 1.6	0.0	0.0 - 0.1	0.0	0.0 - 0.2	0.0	0.0 - 0.0	1.0	0.2 - 3.3	1.00
3080103	81525	ST JOHNS R	16.5	58.3	20 - 128	0.0	0.0 - 0.0	43.1	15.3 - 97.5	0.1	0.0 - 0.3	0.1	0.0 - 0.2	0.0	0.0 - 0.0	15.0	5.0 - 42.3	1.00
3080203	81526	ATLANTIC OCEAN	246.9	22.0	7 - 57	0.0	0.0 - 0.0	5.5	1.7 - 12.2	0.1	0.0 - 0.5	0.7	0.2 - 2.1	0.0	0.0 - 0.0	15.7	5.2 - 45.8	1.00
3100102	81533	MYAKKA R	6.5	77.1	26 - 232	0.0	0.0 - 0.0	21.8	7.1 - 49.5	0.1	0.0 - 0.3	0.0	0.0 - 0.0	0.0	0.0 - 0.0	55.2	19.6 - 167.4	1.00
3140101	81534	ST JOSEPH BAY	758.6	27.2	10 - 65	2.9	1.0 - 6.8	4.4	1.5 - 10.2	0.0	0.0 - 0.0	0.0	0.0 - 0.0	0.0	0.0 - 0.0	19.9	7.0 - 50.5	1.00
3140203	81535	CHOCTAWHATCH	5.7	16.4	6 - 38	0.0	0.0 - 0.0	0.3	0.1 - 0.5	0.0	0.0 - 0.0	0.0	0.0 - 0.0	0.0	0.0 - 0.0	16.2	6.1 - 38.5	1.00
3160205	81536	MOBILE BAY	239.9	12.4	4 - 32	0.0	0.0 - 0.0	1.2	0.4 - 3.0	0.0	0.0 - 0.0	0.0	0.0 - 0.1	0.0	0.0 - 0.0	11.2	3.5 - 30.4	1.00
3160205	81537	MOBILE BAY	179.3	51.3	18 - 95	0.0	0.0 - 0.0	10.0	3.1 - 19.4	3.1	0.8 - 8.1	24.1	7.1 - 51.9	0.0	0.0 - 0.0	14.1	4.7 - 29.6	1.00
3170009	81538	MISSISSIPPI SOUN	304.0	296.6	104 - 887	0.7	0.2 - 1.6	11.3	4.1 - 25.5	2.8	0.8 - 9.9	6.1	1.9 - 16.7	0.0	0.0 - 0.0	275.7	97.6 - 827.6	1.00
3010205	81738	ATLANTIC OCEAN	542.8	15.5	6 - 32	0.0	0.0 - 0.0	1.2	0.5 - 2.5	0.0	0.0 - 0.1	4.7	1.7 - 11.6	0.0	0.0 - 0.0	9.6	3.4 - 23.0	1.00
3010205	81739	ATLANTIC OCEAN	546.9	2.1	1 - 6	0.0	0.0 - 0.0	0.3	0.1 - 1.0	0.0	0.0 - 0.0	0.1	0.0 - 0.4	0.0	0.0 - 0.0	1.6	0.5 - 4.6	1.00
3010205	81740	ALBEMARLE SOU	450.7	1.5	1 - 4	1.5	0.6 - 3.9	0.0	0.0 - 0.0	0.0	0.0 - 0.0	0.0	0.0 - 0.0	0.0	0.0 - 0.0	0.0	0.0 - 0.0	1.00
3010205	81741	CURRITUNK SOUN	128.5	0.2	0 - 1	0.0	0.0 - 0.0	0.0	0.0 - 0.0	0.0	0.0 - 0.0	0.0	0.0 - 0.3	0.0	0.0 - 0.0	0.1	0.0 - 0.5	1.00
3010205	81742	BACK BAY	217.8	0.3	0 - 1	0.0	0.0 - 0.0	0.0	0.0 - 0.0	0.0	0.0 - 0.0	0.1	0.0 - 0.7	0.0	0.0 - 0.0	0.1	0.0 - 0.6	1.00
3010205	81743	NORTH LANDING	73.8	0.5	0 - 2	0.0	0.0 - 0.0	0.0	0.0 - 0.0	0.0	0.0 - 0.0	0.3	0.0 - 1.5	0.0	0.0 - 0.0	0.2	0.0 - 0.8	1.00
3010205	81744	CURRITUNK SOUN	299.3	3.6	1 - 9	0.0	0.0 - 0.0	0.1	0.0 - 0.3	0.0	0.0 - 0.0	1.4	0.3 - 4.2	0.0	0.0 - 0.0	2.1	0.5 - 5.1	1.00
3010205	81745	NORTH R	115.2	0.0	0 - 0	0.0	0.0 - 0.0	0.0	0.0 - 0.0	0.0	0.0 - 0.0	0.0	0.0 - 0.0	0.0	0.0 - 0.0	0.0	0.0 - 0.0	1.00
3010205	81746	NORTH R	385.5	0.0	0 - 0	0.0	0.0 - 0.0	0.0	0.0 - 0.0	0.0	0.0 - 0.0	0.0	0.0 - 0.0	0.0	0.0 - 0.0	0.0	0.0 - 0.1	1.00
3010205	81747	PASQUOTUNK R	137.9	123.3	40 - 289	18.7	6.0 - 41.7	0.5	0.1 - 1.1	0.2	0.1 - 0.6	63.1	19.9 - 154.5	0.0	0.0 - 0.0	41.0	13.8 - 107.2	1.00
3010205	81748	ALBEMARLE SOU	208.8	81.9	25 - 158	0.0	0.0 - 0.0	0.3	0.1 - 0.7	6.5	1.9 - 19.4	46.9	13.8 - 112.0	0.0	0.0 - 0.0	28.1	9.4 - 66.9	1.00

8-digit HUC	ID	Name	Area	Catchment Yield		Point sources		Developed Land		Manure		Agricultural Land		Phosphate Mines		Soil parent rock		Frac
				kg/km^2	90% CI	kg/km^2	90% CI	kg/km^2	90% CI	kg/km^2	90% CI	kg/km^2	90% CI	kg/km^2	90% CI	kg/km^2	90% CI	
3010203	81749	CHOWAN R	151.0	32.2	10 - 57	0.0	0.0 - 0.0	0.4	0.1 - 0.7	3.0	0.9 - 7.1	17.5	5.7 - 38.8	0.0	0.0 - 0.0	11.3	3.9 - 21.1	1.00
3010205	81750	SCUPPERNONG R	22.0	0.5	0 - 1	0.5	0.2 - 1.1	0.0	0.0 - 0.0	0.0	0.0 - 0.0	0.0	0.0 - 0.0	0.0	0.0 - 0.0	0.0	0.0 - 0.0	1.00
3010205	81751	ALBEMARLE SOUI	232.4	0.0	0 - 0	0.0	0.0 - 0.0	0.0	0.0 - 0.0	0.0	0.0 - 0.0	0.0	0.0 - 0.0	0.0	0.0 - 0.0	0.0	0.0 - 0.0	1.00
3010205	81752	ALLIGATOR R	364.3	0.0	0 - 0	0.0	0.0 - 0.0	0.0	0.0 - 0.0	0.0	0.0 - 0.0	0.0	0.0 - 0.0	0.0	0.0 - 0.0	0.0	0.0 - 0.0	1.00
3020104	81753	PUNGO R	136.4	0.7	0 - 3	0.0	0.0 - 0.0	0.0	0.0 - 0.1	0.0	0.0 - 0.0	0.2	0.0 - 1.0	0.0	0.0 - 0.0	0.5	0.1 - 2.1	1.00
3020104	81754	PUNGO R	16.5	9.0	2 - 19	0.0	0.0 - 0.0	0.9	0.2 - 2.1	0.1	0.0 - 0.2	4.1	1.1 - 10.8	0.0	0.0 - 0.0	4.0	1.2 - 9.2	1.00
3020104	81755	PAMLICO R	177.4	10.9	3 - 29	0.0	0.0 - 0.0	0.3	0.1 - 1.0	0.7	0.2 - 2.5	4.2	1.2 - 13.8	0.0	0.0 - 0.0	5.7	1.7 - 16.3	1.00
3020104	81756	PAMLICO R	90.4	0.5	0 - 2	0.0	0.0 - 0.0	0.0	0.0 - 0.1	0.0	0.0 - 0.1	0.1	0.0 - 0.5	0.0	0.0 - 0.0	0.4	0.1 - 1.8	1.00
3020105	81757	PAMLICO SOUND	150.4	0.1	0 - 1	0.0	0.0 - 0.0	0.0	0.0 - 0.0	0.0	0.0 - 0.0	0.0	0.0 - 0.0	0.0	0.0 - 0.0	0.1	0.0 - 0.6	1.00
3020105	81758	PAMLICO SOUND	233.6	2.4	1 - 6	0.0	0.0 - 0.0	0.1	0.0 - 0.2	0.0	0.0 - 0.0	0.5	0.1 - 1.4	0.0	0.0 - 0.0	1.9	0.5 - 5.1	1.00
3020204	81759	NEUSE R	75.3	26.7	9 - 62	0.8	0.3 - 2.1	9.9	3.0 - 23.2	1.7	0.5 - 5.7	4.3	1.2 - 12.1	0.0	0.0 - 0.0	10.0	3.6 - 29.1	1.00
3020204	81760	NEUSE R	388.2	7.9	2 - 19	0.0	0.0 - 0.0	0.4	0.1 - 1.0	0.1	0.0 - 0.4	1.9	0.5 - 5.8	0.0	0.0 - 0.0	5.5	1.7 - 13.7	1.00
3020204	81761	NEUSE R	121.5	18.7	7 - 39	10.1	3.5 - 21.8	3.3	1.0 - 8.5	0.4	0.1 - 1.0	1.1	0.3 - 3.2	0.0	0.0 - 0.0	3.8	1.3 - 10.3	1.00
3020106	81762	WEST BAY	385.5	0.2	0 - 1	0.0	0.0 - 0.0	0.0	0.0 - 0.0	0.0	0.0 - 0.0	0.1	0.0 - 0.5	0.0	0.0 - 0.0	0.1	0.0 - 0.7	1.00
3020106	81763	CORE SOUND	1199.1	0.6	0 - 3	0.0	0.0 - 0.0	0.0	0.0 - 0.1	0.0	0.0 - 0.0	0.1	0.0 - 0.5	0.0	0.0 - 0.0	0.5	0.1 - 2.2	1.00
3020106	81764	NORTH R	294.1	13.3	4 - 36	2.3	0.8 - 5.9	1.3	0.4 - 4.0	0.0	0.0 - 0.0	5.0	1.3 - 16.9	0.0	0.0 - 0.0	4.7	1.5 - 12.2	1.00
3020106	81765	NEWPORT R	600.2	10.1	3 - 18	3.4	1.2 - 6.7	3.7	1.1 - 8.5	0.0	0.0 - 0.0	0.3	0.1 - 0.8	0.0	0.0 - 0.0	2.6	0.9 - 6.3	1.00
3020106	81766	WHITE OAK R	80.7	10.3	3 - 25	0.0	0.0 - 0.0	4.8	1.6 - 11.5	0.0	0.0 - 0.0	2.9	1.0 - 7.5	0.0	0.0 - 0.0	2.5	0.9 - 6.3	1.00
3020106	81767	WHITE OAK R	43.8	57.5	21 - 117	16.6	6.8 - 34.6	13.5	4.0 - 32.1	10.5	3.0 - 29.1	11.6	4.0 - 27.3	0.0	0.0 - 0.0	5.3	1.9 - 11.2	1.00
3020106	81768	BOGUE SOUND	158.0	4.0	1 - 7	0.0	0.0 - 0.0	1.9	0.6 - 3.5	0.3	0.1 - 0.6	0.3	0.1 - 0.8	0.0	0.0 - 0.0	1.5	0.5 - 3.0	1.00
3030001	81769	NEW R	160.6	27.2	9 - 51	0.0	0.0 - 0.0	17.2	5.1 - 36.1	1.0	0.3 - 2.6	1.0	0.4 - 2.4	0.0	0.0 - 0.0	8.0	2.6 - 17.2	1.00
3030001	81770	NEW R	19.3	90.9	33 - 193	16.7	6.1 - 35.2	53.1	18.5 - 125.8	3.5	1.1 - 8.2	3.9	1.4 - 9.0	0.0	0.0 - 0.0	13.8	5.1 - 33.8	1.00
3030005	81771	ONSLOW BAY	247.0	152.4	56 - 405	147.9	54.4 - 394.0	3.1	0.9 - 8.7	0.0	0.0 - 0.0	0.1	0.0 - 0.2	0.0	0.0 - 0.0	1.4	0.5 - 4.0	1.00
3030005	81772	CAPE FEAR R	351.0	9.2	3 - 18	6.2	2.3 - 14.0	1.4	0.4 - 4.3	0.2	0.1 - 0.6	0.4	0.1 - 1.3	0.0	0.0 - 0.0	0.9	0.3 - 2.4	1.00
3030005	81773	CAPE FEAR R	27.0	12.9	4 - 30	0.0	0.0 - 0.0	1.8	0.6 - 3.7	0.5	0.1 - 1.7	1.1	0.3 - 2.8	0.0	0.0 - 0.0	9.4	3.4 - 23.3	1.00
3040207	81774	WINYAH BAY	376.5	3.8	1 - 8	0.0	0.0 - 0.0	0.1	0.0 - 0.4	0.0	0.0 - 0.1	0.2	0.0 - 0.5	0.0	0.0 - 0.0	3.4	1.0 - 8.0	1.00
3050202	81775	ATLANTIC OCEAN	389.2	0.0	0 - 0	0.0	0.0 - 0.0	0.0	0.0 - 0.0	0.0	0.0 - 0.0	0.0	0.0 - 0.0	0.0	0.0 - 0.0	0.0	0.0 - 0.3	1.00
3050202	81776	ATLANTIC OCEAN	36.8	290.9	109 - 561	286.8	106.5 - 549.7	1.5	0.4 - 5.4	0.0	0.0 - 0.0	0.1	0.0 - 0.3	0.0	0.0 - 0.0	2.5	0.6 - 7.8	1.00
3050202	81777	ATLANTIC OCEAN	119.3	0.0	0 - 0	0.0	0.0 - 0.0	0.0	0.0 - 0.0	0.0	0.0 - 0.0	0.0	0.0 - 0.0	0.0	0.0 - 0.0	0.0	0.0 - 0.2	1.00
3050202	81778	ASHLEY R	31.0	1310.1	463 - 3326	1286.8	455.3 - 3279.7	13.7	3.8 - 33.1	0.0	0.0 - 0.0	0.3	0.1 - 0.7	0.0	0.0 - 0.0	9.3	2.7 - 25.2	1.00
3050201	81779	CHARLESTON HAI	25.6	1130.9	349 - 2764	1067.2	325.6 - 2628.7	44.8	16.0 - 93.8	0.1	0.0 - 0.2	0.6	0.2 - 1.5	0.0	0.0 - 0.0	18.3	5.7 - 53.5	1.00
3050202	81780	ASHLEY R	232.8	0.4	0 - 2	0.0	0.0 - 0.0	0.0	0.0 - 0.1	0.0	0.0 - 0.0	0.0	0.0 - 0.0	0.0	0.0 - 0.0	0.4	0.0 - 2.0	1.00
3050202	81781	STONO R	170.2	0.1	0 - 1	0.0	0.0 - 0.0	0.0	0.0 - 0.0	0.0	0.0 - 0.0	0.0	0.0 - 0.0	0.0	0.0 - 0.0	0.1	0.0 - 0.7	1.00
3050208	81782	ATLANTIC OCEAN	87.8	0.0	0 - 0	0.0	0.0 - 0.0	0.0	0.0 - 0.0	0.0	0.0 - 0.0	0.0	0.0 - 0.0	0.0	0.0 - 0.0	0.0	0.0 - 0.5	1.00
3050208	81783	ST HELENA SOUNI	259.0	4.8	1 - 11	4.0	1.1 - 10.2	0.0	0.0 - 0.1	0.0	0.0 - 0.1	0.2	0.0 - 0.9	0.0	0.0 - 0.0	0.6	0.1 - 2.4	1.00
3060204	81784	WILMINGTON R	270.3	33.8	10 - 71	17.3	6.1 - 37.7	5.2	1.3 - 13.5	0.0	0.0 - 0.1	0.2	0.1 - 0.5	0.0	0.0 - 0.0	11.1	3.1 - 30.7	1.00
3060204	81785	OSSABAW SOUND	558.2	7.1	2 - 21	0.0	0.0 - 0.0	0.0	0.0 - 0.1	0.0	0.0 - 0.0	0.0	0.0 - 0.0	0.0	0.0 - 0.0	7.1	1.8 - 21.5	1.00
3060204	81786	MEDWAY R	114.8	7.1	2 - 22	0.0	0.0 - 0.0	0.1	0.0 - 0.2	0.0	0.0 - 0.1	0.1	0.0 - 0.3	0.0	0.0 - 0.0	7.0	1.5 - 21.9	1.00
3060204	81787	ST CATHERINES S	193.8	9.1	2 - 26	0.0	0.0 - 0.0	0.1	0.0 - 0.3	0.0	0.0 - 0.0	0.0	0.0 - 0.1	0.0	0.0 - 0.0	8.9	2.0 - 25.9	1.00

8-digit HUC	ID	Name	Area	Catchment Yield		Point sources		Developed Land		Manure		Agricultural Land		Phosphate Mines		Soil parent rock		Frac
				kg/km^2	90% CI	kg/km^2	90% CI	kg/km^2	90% CI	kg/km^2	90% CI	kg/km^2	90% CI	kg/km^2	90% CI	kg/km^2	90% CI	
3060204	81788	SAPELO SOUND	248.6	7.7	2 - 25	0.0	0.0 - 0.0	0.1	0.0 - 0.3	0.0	0.0 - 0.0	0.0	0.0 - 0.2	0.0	0.0 - 0.0	7.5	1.9 - 24.3	1.00
3070106	81789	ALTAMAHA SOUN	10.1	13.9	4 - 42	0.0	0.0 - 0.0	0.0	0.0 - 0.0	0.0	0.0 - 0.0	0.0	0.0 - 0.2	0.0	0.0 - 0.0	13.9	4.2 - 41.8	1.00
3070203	81790	ALTAMAHA SOUN	46.0	8.7	3 - 27	0.0	0.0 - 0.0	0.4	0.1 - 1.2	0.0	0.0 - 0.0	0.0	0.0 - 0.0	0.0	0.0 - 0.0	8.4	2.5 - 27.1	1.00
3070203	81791	ST SIMONS SOUNI	70.7	25.5	9 - 62	14.1	4.2 - 39.4	0.6	0.2 - 2.0	0.0	0.0 - 0.0	0.1	0.0 - 0.5	0.0	0.0 - 0.0	10.6	2.8 - 33.4	1.00
3070201	81792	LITTLE SATILLA R	224.8	10.5	3 - 24	0.0	0.0 - 0.0	0.3	0.1 - 0.8	0.0	0.0 - 0.0	0.1	0.0 - 0.2	0.0	0.0 - 0.0	10.1	3.0 - 24.3	1.00
3070203	81793	CROOKED R	330.4	10.5	3 - 24	0.0	0.0 - 0.0	0.2	0.0 - 0.5	0.0	0.0 - 0.1	0.1	0.0 - 0.3	0.0	0.0 - 0.0	10.2	3.2 - 23.4	1.00
3070203	81794	CUMBERLAND R	102.9	8.7	2 - 22	0.2	0.1 - 0.5	0.9	0.2 - 2.4	0.0	0.0 - 0.0	0.1	0.0 - 0.4	0.0	0.0 - 0.0	7.4	1.9 - 19.0	1.00
3080103	81795	ST JOHNS R	90.5	0.5	0 - 3	0.0	0.0 - 0.0	0.1	0.0 - 0.7	0.0	0.0 - 0.0	0.0	0.0 - 0.0	0.0	0.0 - 0.0	0.4	0.1 - 1.8	1.00
3080103	81796	ST JOHNS R	85.3	345.6	128 - 649	321.1	120.4 - 625.2	19.3	6.0 - 39.3	0.0	0.0 - 0.1	0.0	0.0 - 0.1	0.0	0.0 - 0.0	5.1	1.9 - 11.7	1.00
3080103	81797	ST JOHNS R	147.4	239.5	95 - 536	146.4	57.4 - 336.4	78.5	29.6 - 182.1	0.2	0.1 - 0.7	0.3	0.1 - 0.7	0.0	0.0 - 0.0	14.1	5.1 - 37.0	1.00
3080103	81798	ST JOHNS R	19.4	84.0	28 - 226	0.0	0.0 - 0.0	69.8	22.1 - 188.5	0.1	0.0 - 0.3	0.1	0.0 - 0.3	0.0	0.0 - 0.0	14.0	5.3 - 36.0	1.00
3080103	81799	ST JOHNS R	58.9	68.7	24 - 170	21.2	8.0 - 56.7	30.8	10.3 - 80.8	0.1	0.0 - 0.3	0.1	0.0 - 0.4	0.0	0.0 - 0.0	16.4	5.7 - 49.9	1.00
3080103	81800	ST JOHNS R	65.6	84.9	33 - 207	27.4	10.5 - 79.1	43.4	16.7 - 112.2	0.2	0.1 - 0.7	0.7	0.3 - 1.8	0.0	0.0 - 0.0	13.2	4.9 - 33.5	1.00
3080103	81801	ST JOHNS R	203.9	33.8	11 - 62	7.1	2.5 - 14.9	11.9	3.7 - 24.2	1.1	0.3 - 3.4	3.4	1.0 - 8.0	0.0	0.0 - 0.0	10.3	3.2 - 22.5	1.00
3080103	81802	ST JOHNS R	114.3	25.9	10 - 49	0.0	0.0 - 0.0	5.4	2.0 - 11.3	0.2	0.1 - 0.5	1.1	0.4 - 2.5	0.0	0.0 - 0.0	19.2	7.7 - 41.7	1.00
3080103	81803	ST JOHNS R	32.6	39.1	13 - 86	0.0	0.0 - 0.0	2.9	0.9 - 5.7	0.7	0.2 - 1.7	7.7	2.3 - 21.4	0.0	0.0 - 0.0	27.8	8.8 - 74.7	1.00
3080103	81804	ST JOHNS R	84.6	87.9	37 - 208	45.5	18.5 - 112.9	21.1	8.6 - 47.1	0.1	0.0 - 0.2	0.3	0.1 - 0.8	0.0	0.0 - 0.0	21.0	8.5 - 57.1	1.00
3080103	81805	ST JOHNS R	96.0	45.0	13 - 106	0.1	0.0 - 0.2	3.6	1.2 - 8.2	2.3	0.5 - 6.4	16.1	4.6 - 47.4	0.0	0.0 - 0.0	22.9	7.4 - 56.3	1.00
3080103	81806	ST JOHNS R	50.8	147.6	44 - 374	75.8	21.1 - 198.8	56.4	16.0 - 129.2	0.2	0.0 - 0.5	0.2	0.1 - 0.5	0.0	0.0 - 0.0	15.0	4.8 - 39.0	1.00
3080201	81807	ATLANTIC OCEAN	579.8	23.6	8 - 57	14.8	4.6 - 38.5	2.3	0.6 - 7.1	0.0	0.0 - 0.1	0.0	0.0 - 0.1	0.0	0.0 - 0.0	6.5	2.1 - 19.7	1.00
3100101	81814	PEACE R	56.2	67.4	24 - 116	0.0	0.0 - 0.0	15.1	5.9 - 27.5	7.6	2.5 - 22.5	26.7	9.0 - 57.3	0.0	0.0 - 0.0	18.0	7.3 - 44.2	1.00
3100102	81815	CHARLOTTE HARI	58.7	38.7	15 - 82	0.0	0.0 - 0.0	6.2	2.0 - 11.1	0.0	0.0 - 0.1	0.0	0.0 - 0.0	0.0	0.0 - 0.0	32.5	12.9 - 64.0	1.00
3100201	81816	MYAKKA R	418.0	79.9	30 - 205	0.0	0.0 - 0.0	21.8	8.1 - 46.8	0.7	0.2 - 3.0	1.3	0.5 - 4.0	0.0	0.0 - 0.0	56.0	19.4 - 161.2	1.00
3100201	81817	LEMON BAY	387.7	59.8	22 - 188	0.0	0.0 - 0.0	15.9	5.1 - 47.8	0.3	0.1 - 1.2	0.7	0.2 - 2.1	0.0	0.0 - 0.0	42.9	14.0 - 136.7	1.00
3100201	81818	GULF OF MEXICO	206.9	66.1	20 - 139	0.0	0.0 - 0.0	24.5	7.7 - 59.6	0.8	0.2 - 2.3	1.6	0.5 - 3.7	0.0	0.0 - 0.0	39.2	11.7 - 90.9	1.00
3100202	81819	MANATEE R	56.5	87.4	24 - 192	0.0	0.0 - 0.0	22.3	6.8 - 45.1	6.2	1.5 - 21.6	19.2	6.3 - 54.0	0.0	0.0 - 0.0	39.7	11.0 - 112.7	1.00
3100206	81820	TAMPA BAY	112.6	82.9	29 - 160	0.7	0.2 - 1.4	14.7	4.1 - 28.4	6.2	1.7 - 14.0	13.7	4.1 - 34.1	0.0	0.0 - 0.0	47.6	15.5 - 116.1	1.00
3100206	81821	HILLSBOROUGH B	270.6	65.5	24 - 162	0.0	0.0 - 0.0	32.8	11.6 - 82.6	0.0	0.0 - 0.1	0.0	0.0 - 0.0	0.0	0.0 - 0.0	32.6	10.5 - 94.0	1.00
3100207	81822	GULF OF MEXICO	318.1	96.8	32 - 228	21.4	7.2 - 51.6	49.8	15.8 - 113.3	0.0	0.0 - 0.0	0.0	0.0 - 0.0	0.0	0.0 - 0.0	25.6	8.0 - 72.4	1.00
3100207	81823	GULF OF MEXICO	735.2	32.7	10 - 75	1.2	0.4 - 2.5	14.8	4.7 - 33.0	0.0	0.0 - 0.0	0.0	0.0 - 0.0	0.0	0.0 - 0.0	16.7	5.4 - 49.3	1.00
3100207	81824	GULF OF MEXICO	1065.8	19.3	6 - 37	0.0	0.0 - 0.0	11.1	3.5 - 23.4	0.2	0.1 - 0.5	0.7	0.2 - 1.9	0.0	0.0 - 0.0	7.3	2.4 - 16.1	1.00
3110101	81825	WITHLACOOCHEE	114.1	3.3	1 - 12	0.0	0.0 - 0.0	0.0	0.0 - 0.0	0.0	0.0 - 0.0	0.0	0.0 - 0.0	0.0	0.0 - 0.0	3.3	0.7 - 11.9	1.00
3110102	81826	SUWANNEE SOUN	97.1	7.1	2 - 17	0.0	0.0 - 0.0	0.1	0.0 - 0.4	0.0	0.0 - 0.0	0.0	0.0 - 0.0	0.0	0.0 - 0.0	7.0	1.8 - 16.8	1.00
3120001	81827	APPALACHEE BAY	298.3	24.5	8 - 55	0.0	0.0 - 0.0	4.7	1.3 - 10.7	0.1	0.0 - 0.3	0.4	0.1 - 0.9	0.0	0.0 - 0.0	19.3	6.1 - 44.2	1.00
3120003	81828	OCHLOCKNEE BA	605.2	14.2	5 - 29	0.0	0.0 - 0.0	1.5	0.5 - 3.4	0.0	0.0 - 0.0	0.1	0.0 - 0.2	0.0	0.0 - 0.0	12.6	4.3 - 27.9	1.00
3130013	81829	OCHLOCKNEE BA	90.4	24.4	7 - 44	0.0	0.0 - 0.0	9.6	2.7 - 16.1	0.0	0.0 - 0.0	0.3	0.1 - 0.6	0.0	0.0 - 0.0	14.5	4.1 - 29.9	1.00
3130013	81830	ST GEORGE SOUN	184.2	28.1	9 - 62	0.0	0.0 - 0.0	4.1	1.2 - 8.0	0.0	0.0 - 0.0	0.1	0.0 - 0.1	0.0	0.0 - 0.0	24.0	7.5 - 53.5	1.00
3130013	81831	ST GEORGE SOUN	254.6	16.2	5 - 38	0.0	0.0 - 0.0	2.0	0.6 - 4.1	0.0	0.0 - 0.0	0.0	0.0 - 0.1	0.0	0.0 - 0.0	14.1	4.4 - 35.3	1.00
3140101	81832	GULF OF MEXICO	414.5	57.3	18 - 140	32.9	10.8 - 80.3	7.6	2.2 - 18.6	0.0	0.0 - 0.0	0.0	0.0 - 0.1	0.0	0.0 - 0.0	16.8	4.9 - 48.4	1.00

8-digit HUC	ID	Name	Area	Catchment Yield		Point sources		Developed Land		Manure		Agricultural Land		Phosphate Mines		Soil parent rock		Frac
				kg/km^2	90% CI	kg/km^2	90% CI	kg/km^2	90% CI	kg/km^2	90% CI	kg/km^2	90% CI	kg/km^2	90% CI	kg/km^2	90% CI	
3140101	81833	GULF OF MEXICO	241.5	29.7	9 - 69	0.0	0.0 - 0.0	13.6	4.1 - 33.4	0.1	0.0 - 0.2	0.0	0.0 - 0.1	0.0	0.0 - 0.0	16.0	4.6 - 39.2	1.00
3140101	81834	GULF OF MEXICO	212.5	23.2	8 - 47	0.0	0.0 - 0.0	6.5	2.5 - 13.3	0.1	0.0 - 0.2	0.0	0.0 - 0.0	0.0	0.0 - 0.0	16.6	5.8 - 38.4	1.00
3140102	81835	CHOCTAWHATCHI	182.9	30.9	10 - 74	0.0	0.0 - 0.0	14.7	5.2 - 34.6	0.4	0.1 - 1.3	0.1	0.0 - 0.2	0.0	0.0 - 0.0	15.7	5.9 - 42.3	1.00
3140102	81836	GULF OF MEXICO	528.2	47.3	16 - 100	0.0	0.0 - 0.0	13.2	4.5 - 24.6	0.0	0.0 - 0.0	0.0	0.0 - 0.0	0.0	0.0 - 0.0	34.1	11.1 - 79.3	1.00
3180004	81895	L BORGNE	66.5	0.1	0 - 1	0.0	0.0 - 0.0	0.0	0.0 - 0.0	0.0	0.0 - 0.0	0.0	0.0 - 0.0	0.0	0.0 - 0.0	0.1	0.0 - 1.0	1.00
3180004	81896	L BORGNE	31.0	1.0	0 - 6	0.0	0.0 - 0.0	0.0	0.0 - 0.0	0.0	0.0 - 0.0	0.0	0.0 - 0.0	0.0	0.0 - 0.0	1.0	0.1 - 6.4	1.00
3020104	81897	PUNGO R	67.9	3.1	1 - 8	0.0	0.0 - 0.0	0.1	0.0 - 0.4	0.0	0.0 - 0.0	0.7	0.2 - 2.1	0.0	0.0 - 0.0	2.3	0.5 - 6.5	1.00
